# Supplementary material for: Manganese catalyzed oximation of hydrocarbons to oximes
Source: Nat Commun. 2026 Jan 9;17:294. doi: 10.1038/s41467-025-66576-0 (PMC12789487; doi:10.1038/s41467-025-66576-0)
Supplement: Supplementary file 1 — Supplementary Information [file 41467_2025_66576_MOESM1_ESM.pdf]

Supplementary Information for

Manganese Catalyzed Oximation of Hydrocarbons to Oximes

Menghui Song[1,4], Hong Li[1,4], Li Xie[2], Xiaoxin Zhang[2], Xiaotian Weilian[1], Rucao Wang[1], Alexander Steiner[3], Huaming Sun[1], Chao Wang[1], Jianliang Xiao[3]*, Chaoqun Li[1]*

[1] Key Laboratory of Applied Surface and Colloid Chemistry, Ministry of Education and School of Chemistry and Chemical Engineering, Shaanxi Normal University, Xi'an, China.

[2] State Key Laboratory of Petroleum Molecular & Process Engineering, Sinopec Research Institute of Petroleum Processing, Beijing, China.

[3] Department of Chemistry, University of Liverpool, Liverpool, United Kingdom.

[4] These authors contributed equally: Menghui Song, Hong Li.

Email: jxiao@liverpool.ac.uk (J.L.X.); lichaoqun@snnu.edu.cn (C.Q. L.)

Table of Contents

1. The importance of oximes......3

2. Reported examples of catalytic C-H oxidation......7

3. General considerations......8

4. Procedures for preparation of ligands and manganese catalysts......8

5. Optimization of reaction conditions for oximation......22

6. Mechanistic studies......35

7. Synthesis and characterization of substrates......73

8. Synthesis of oximes from ketones to aid in product analysis......96

9. General procedure for oximation of hydrocarbons......97

10. Gram-scale synthesis of cyclododecanone oxime......100

11. Oximation of propane to acetone oxime......100

12. Analytical data of products......102

13. X-ray photoelectron spectroscopy characterization of ***rac*-C1**......187

14. X-ray structures and crystallographic data......187

15. ^{1}H, ^{13}C and ^{19}F NMR spectra......197

16. HPLC traces......536

17. References......540

1. The importance of oximes

Figure S-1. Selected examples of oximes seen in drug and related bioactive molecules [1-18]

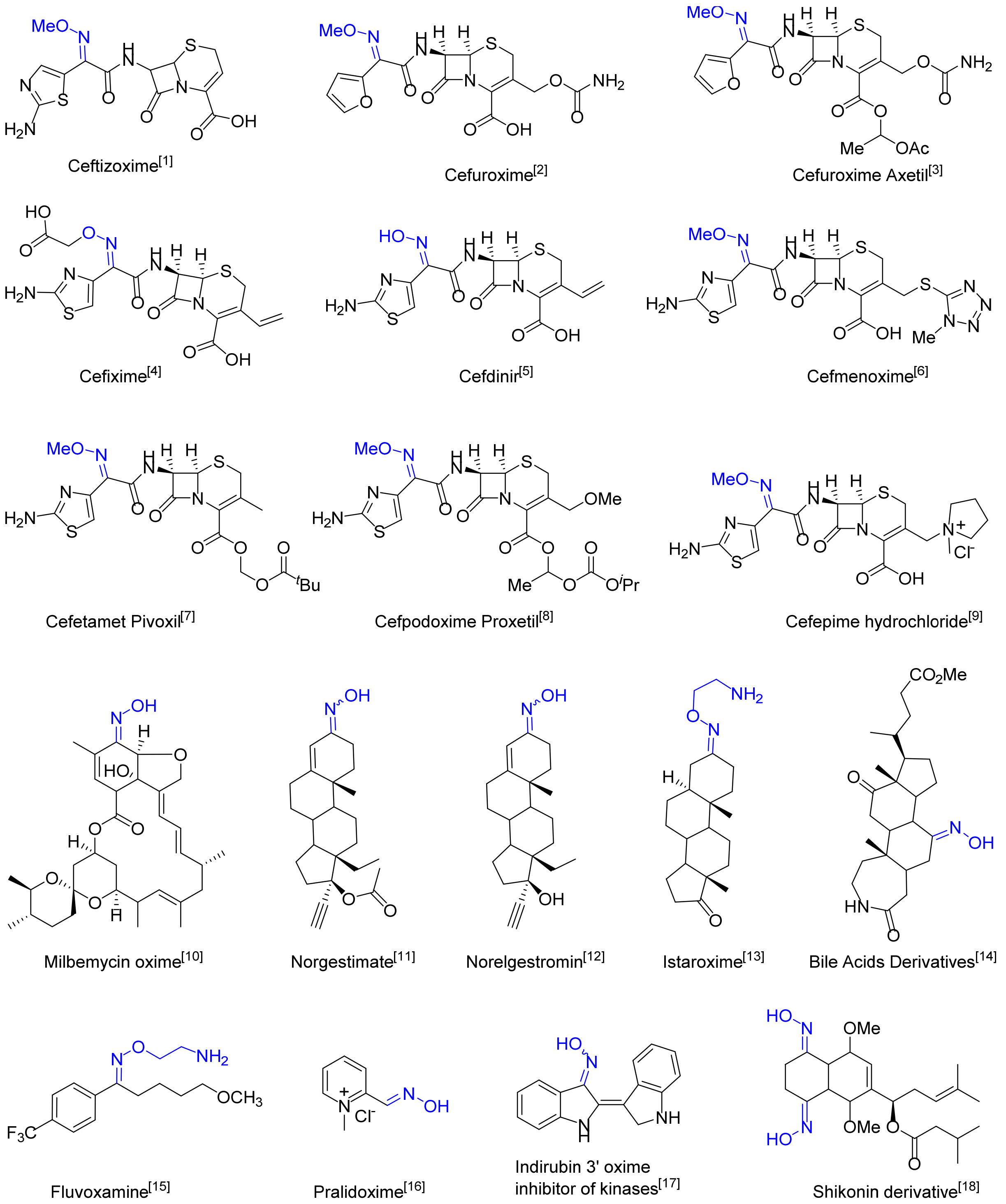

Figure S-2. Selected examples of oximes seen in natural products [19-29]

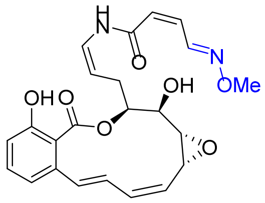

Oximidine I[19]

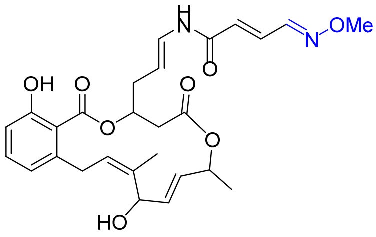

Labatamide C[20]

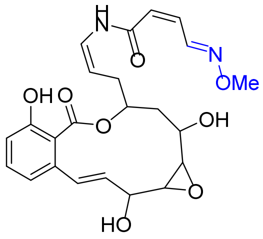

CJ-12950[21]

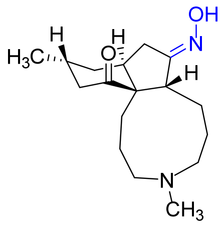

Lycoposerramine-B[22]

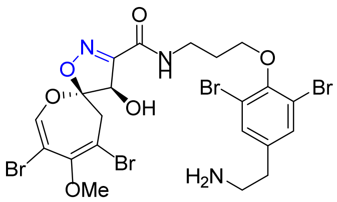

Psammaplysin A[23]

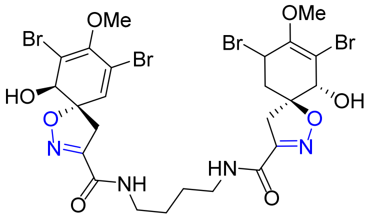

Aerothionin[24]

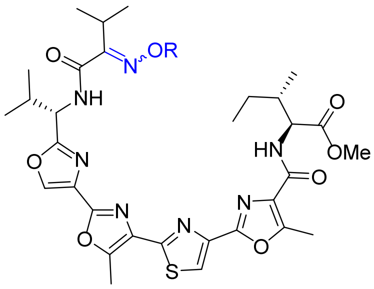

(*E*), R = H, Azolemycin A[25];
(*E*), R = Me, Azolemycin C[25];
(*Z*), R = H, Azolemycin B[25];
(*Z*), R = Me, Azolemycin D[25]

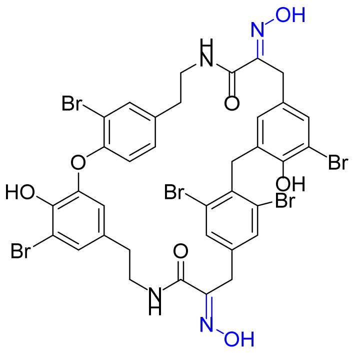

Bastadin[26]

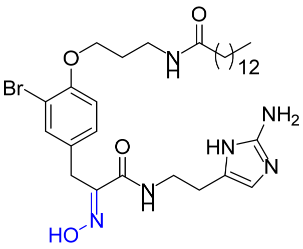

Lipopurealin A[27]

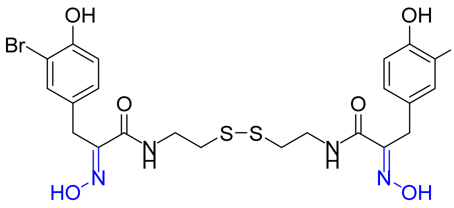

Psammaplin A[28]

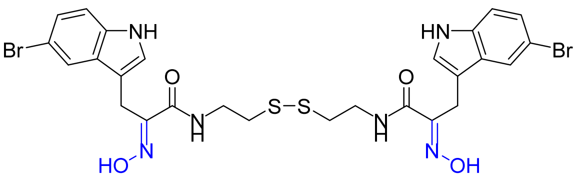

UVI5008[29]

Figure S-3. Current industrial processes for synthesis of nylon-6 [30-36]

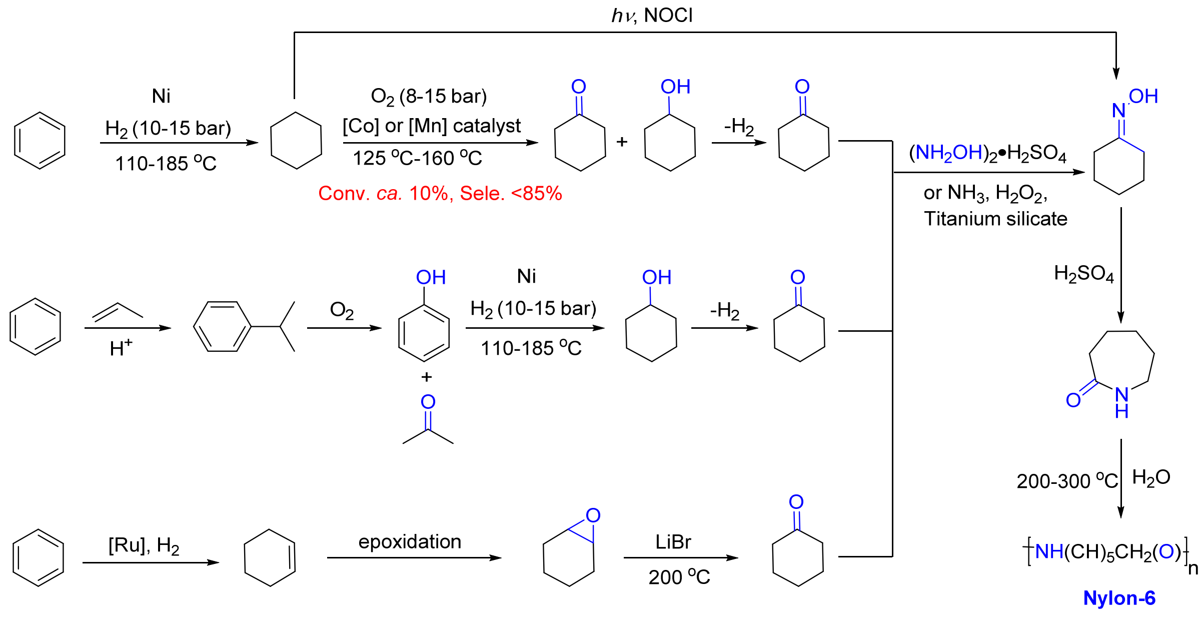

Figure S-4. Current industrial processes for synthesis of nylon-12 [34,37-40]

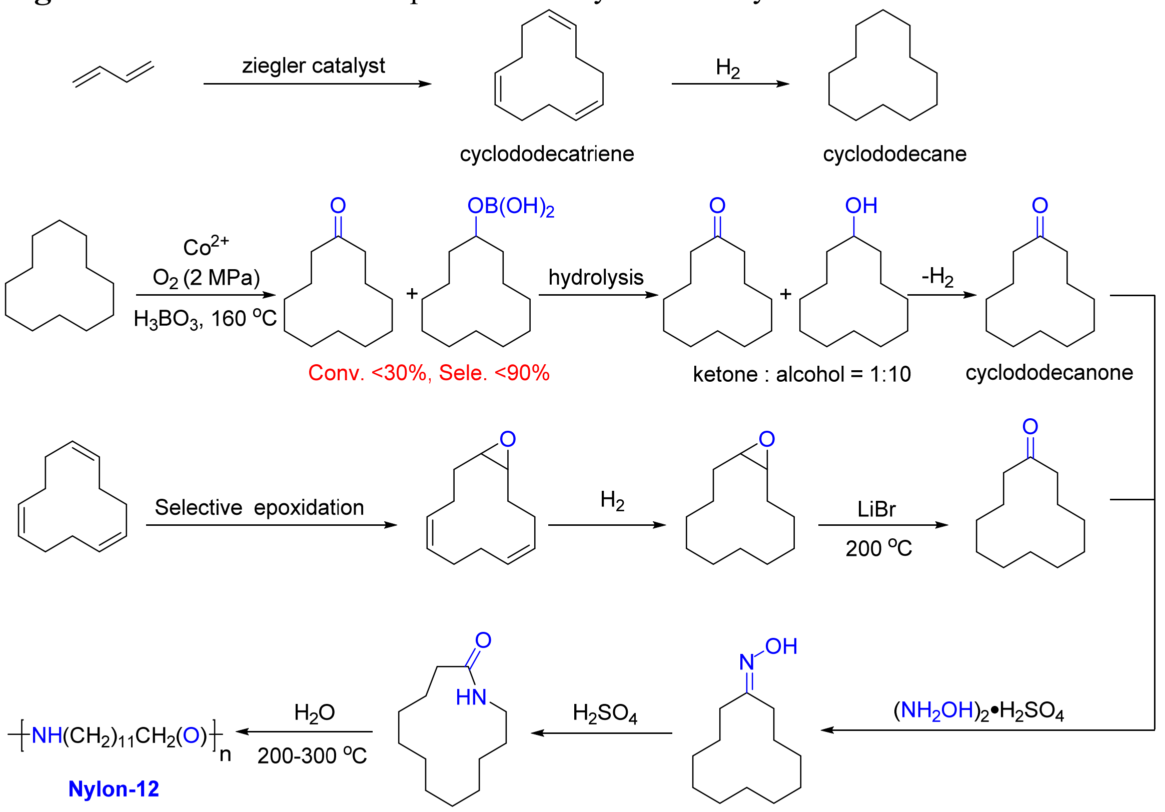

Figure S-5. Current industrial processes for synthesis of alkyl ketone oxime products [41]

a) Synthesis of acetone oxime

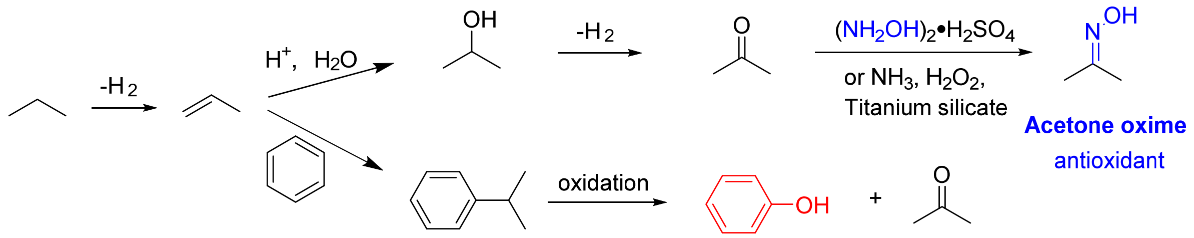

b) Synthesis of 2-butanone oxime

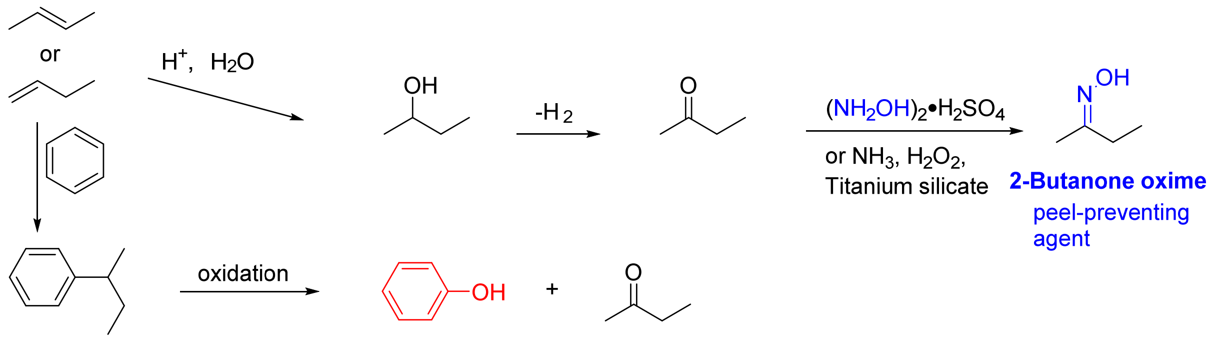

c) Synthesis of 2-pentanone oxime

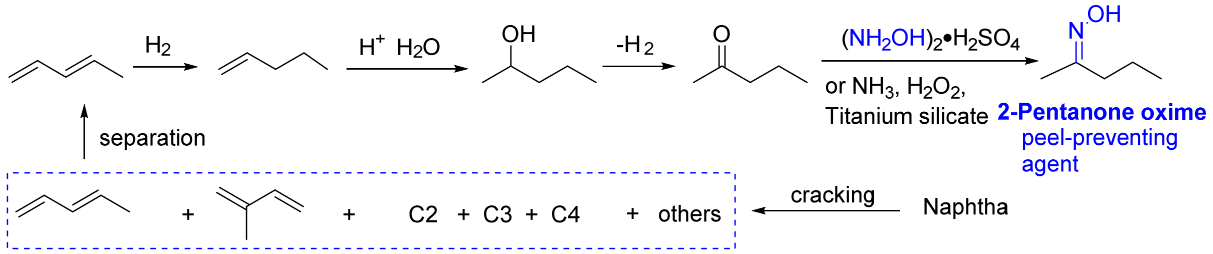

d) Synthesis of cyclopentanone oxime

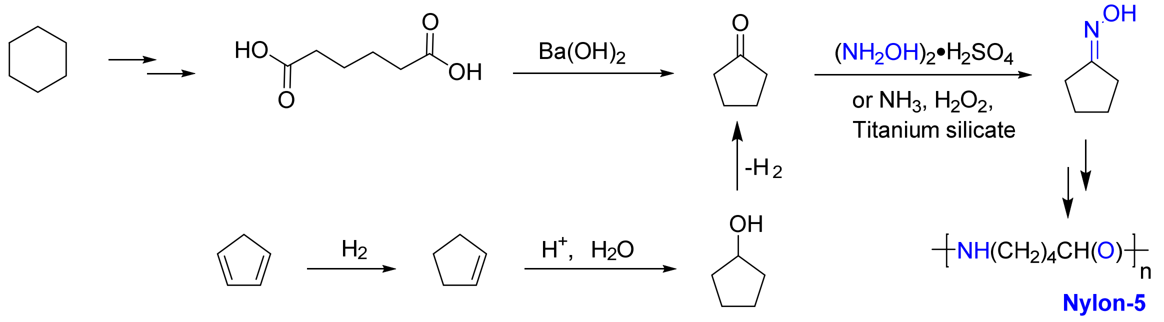

2. Reported examples of catalytic C-H oxidation

Figure S-6. Comparison of oximation (this work) with ketonization (literature) [38, 42-57]

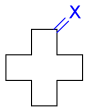

1a (X = NOH): 91%;
1a-one (X = O):
Electrochemical[42]: 21%
([Co]+NHPI)[43,38]: 34% + acid
[Fe][44]: 21% + A (A/K = 0.8)

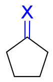

2a (X = NOH): 79%
2a-one (X = O):
([Co]+NHPI)[43,38]: 27%
[Mn][45]: 68%

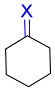

3a (X = NOH): 81%
3a-one (X = O):
[Mn][45]: 89%
[Fe][46]: 92%
[Mn][47]: 70%

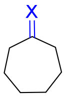

4a (X = NOH): 89%
4a-one (X = O):
Electrochemical[42]: 16%,
[Mn][45]: 72%
[Fe][44]: 29% + A (A/K = 0.5)

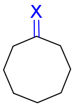

5a (X = NOH): 90%
5a-one (X = O):
Electrochemical[42]: 42%
([Co]+NHPI)[43,38]: 50% + others
[Fe][44]: 32% + A (A/K = 0.8)
[Mn][45]: 86%

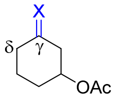

16a (X = NOH):
81%, γ:δ = 1.5:1
16a-one (X = O):
[Mn][48]: 90%, γ:δ = 1:21

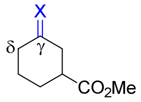

17a (X = NOH):
86%, γ:δ = 2.7:1
17a-one (X = O):
[Fe][49]: 64%, γ:δ = 2:1
[Mn][50]: 67%, γ:δ = 1.2:1

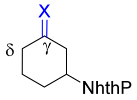

19a (X = NOH):
43%, γ:δ = 2.5:1
19a-one (X = O):
[Mn][50]: 37%, γ:δ = 0.8:1

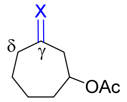

20a (X = NOH):
80%, γ:δ = 1:2
20a-one (X = O):
[Mn][48]: 85%, γ:δ = 1:5.5

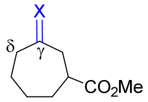

21a (X = NOH):
84%, γ:δ = 1:1.3
21a-one (X = O):
[Fe][49]: 73%, γ:δ = 1:1.6
[Mn][48]: 94%, γ:δ = 1:5.3

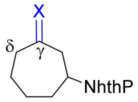

22a (X = NOH):
60%, γ:δ = 1:2.3
22a-one (X = O):
[Mn][48]: 95%, γ:δ = 1:4.5
Electrochemical[51]:
50%, γ:δ = 1:1.4

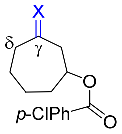

23a (X = NOH):
75%, γ:δ = 1:1.7
23a-one (X = O):
[TFDO][51]: 92%, γ:δ = 1:5.6
Electrochemical[51]:
78%, γ:δ = 1:2.3

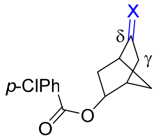

24a (X = NOH): 63%
24a-one(X = O):
[TFDO][51]: 95%, γ:δ > 1:20
Electrochemical[51]:
56%, γ:δ = 1:1.9

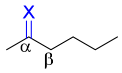

11a (X = NOH):
58%, β:α = 2:5
11a-one (X = O):
[Mn][45]: 36%, β:α = 1:1.5
[Fe][49]: 56%, β:α = 1:1.1
[Fe][52]: 57%, β:α = 1:1.1

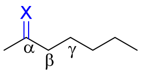

12a (X = NOH): 50%
γ:β:α = 1:3.3:8.3
12a-one (X = O):
[Fe][53]: 50%
γ:β:α = 1:2:2.9

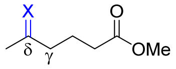

30a (X = NOH):
63%, γ:δ = 1:5
30a-one (X = O):
[Fe][49]: 72%, γ:δ = 1:2.3
[Fe][52]: 64%, γ:δ = 1:2.8

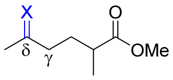

32a (X = NOH): 45%
32a-one (X = O):
[Fe][49]: 69%, γ:δ = 1:3.6

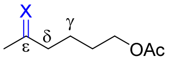

42a (X = NOH):
56%, δ:ε = 1:9
42a-one (X = O):
[Fe][53]: 26%, δ:ε = 1:3.2
[Mn][48]: 74%, γ:δ = 1:6.7

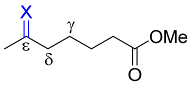

44a (X = NOH):
63%, γ:δ:ε = 1:6.7:16.7
44a-one (X = O):
[Fe][49]: 83%, γ:δ:ε = 1:1.3:3.6
[Mn][48]: 76%, γ:δ:ε = 1:6.5:41

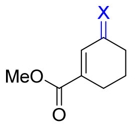

63a (X = NOH):
60%
63a-one (X = O):
[Rh][54]: 92%

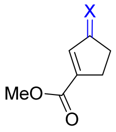

65a (X = NOH):
73%
65a-one (X = O):
[Rh][54]: 79%

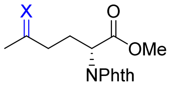

102a (X = NOH):
31%
102a-one (X = O):
[Mn][55]: 66%

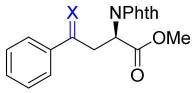

113a (X = NOH):
80%
113a-one (X = O):
[Mn][56]: 95%

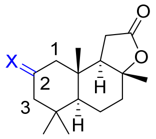

120-oxime (X = NOH):
67%, C1:C2:C3=1:14:1.8
120-one (X = O):
[Fe][49]:78%, C2:C3=1.4:1
[Fe][53]: 60%, C1:C2:C3=1:12:3.7
Electrochemical[51]: 51%, C2:C3=4.6:1

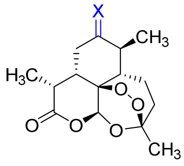

122a (X = NOH):
56%
122-one (X = O):
[Fe][57]: 52%

3. General considerations

The following commercially obtained reagents for the C-H oximation reactions were used as received: H_2O_2 (30 wt% in H_2O, Sinopharm), AcOH (Sinopharm), ^{t}BuOH (Sinopharm), and CH_3CN (Sinopharm). All oximation reactions were run under air with no precautions taken to exclude moisture. Commercial grade solvents used in the synthesis of components were used without further purification. Chemicals employed in the synthesis of ligands and substrates were purchased from commercial suppliers and used without further purification. ^{1}H NMR spectra were recorded on a Bruker Advance 400 (400 MHz) NMR spectrometer and reported in units of parts per million (ppm) relative to tetramethyl silane (δ 0 ppm) or $CDCl_3$ (δ 7.26 ppm) or DMSO-d_6 (δ 2.50 ppm) or CD_3OD (δ 3.31 ppm). Multiplicities are given as: brs (broad singlet), s (singlet), d (doublet), t (triplet), q (quartet), quint (quintet), sextet (sext), heptet (hept), dd (doublets of doublet), dt (doublets of triplet) or m (multiplet). ^{13}C NMR spectra were recorded on a Bruker Advance 400 (100 MHz) NMR spectrometer and reported in ppm relative to $CDCl_3$ (δ 77.16 ppm) or DMSO-d_6 (δ 40.0 ppm) or CD_3OD (δ 49.0 ppm). Coupling constants were reported as a *J* value in Hz. Gas chromatography was performed on a SHIMADZU GC-2014 with a WondaCap WAX column (df = 0.25 um, X = 30 m). For the products ***E*-22a-δ-oxime**, ***E*-65a**, **2*E*,4*E*-119a**, ***E*-120a**, **and *Z*-120b**, the *E/Z* isomerism was assigned based on their X-ray structures; for known products, the *E/Z* isomerism was assigned based on the literature reported; for unknown products, we assumed the major isomer to be the usually-thermodynamically more stable *E* isomer, although we understand that this is unlikely to be correct all the time, particularly when other structural factors come to play, e.g. hydrogen bonding. Regardless, we can only confirm the isomer ratio, not the absolute E/Z configuration, for these products. The enantiomeric excess (*ee*) of substrate **115** and product **115a** was determined by chiral phase HPLC analysis on a SHIMADZU LC-2010A HT HPLC. Mass spectra were obtained by electrospray ionization (ESI) at the Analytical Services of the Chemistry Department, Shannxi Normal University. UV-vis absorption spectra were collected on a PerkinElmer Lambda 365 UV-VIS spectrophotometer at room temperature (1 cm pathlength quartz cuvette). All reagents were prepared volumetrically and dispensed in stock solutions.

4. Procedures for preparation of ligands and manganese catalysts

4.1 Procedures for preparation of pyridine synthons

The pyridine synthons used for ligands synthesis were purchased from Bidepharm, Energy Chemical or synthesized following previously described procedures. [46, 50, 56, 58-62]

4.2 Procedures for preparation of ligands

A. Synthesis of L$^{1-2}$ [46, 56, 58]

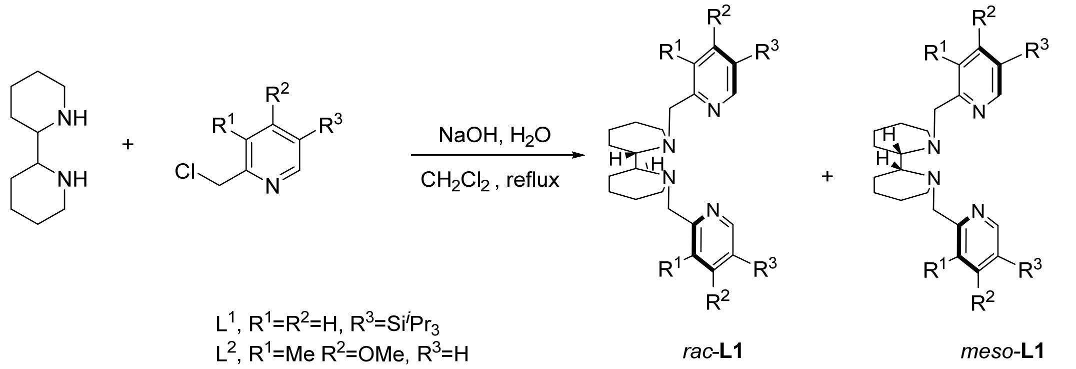

Synthesis of *rac*-L^1, *meso*-L^1, and *rac/meso*-L^1:

To a solution of 2,2'-bipiperidine (336.6 mg, 2.0 mmol, 1 equiv.) and 2-(chloromethyl)-5-(triisopropylsilyl)pyridine (1192.5 mg, 4.2 mmol, 2.1 equiv.) dissolved in DCM (20.0 mL), sodium hydroxide (400.0 mg, 10 mmol, 5 equiv.) dissolved in water (5.0 mL) was added dropwise for 5 minutes at 0 °C. The resulting biphasic mixture was stirred for 6 days under reflux. After the reaction was complete (monitored by TLC), the mixture was diluted with DCM (20 mL) and transferred to a separatory funnel. Afterwards, 20.0 mL H_2O was added and the mixture was extracted with DCM (3×20.0 mL). The combined organic layers were then dried over Na_2SO_4, and filtered. After concentration, the residue was purified by flash column chromatography on silica gel (petroleum ether/EtOAc/Et_3N = 100:5:1 to 100:20:1) to give the racemic (rac) ***rac*-L^1** (583.6 mg, 44% yield) and the meso analogue ***meso*-L^1** (172.5 mg, 13% yield), separately with both as a white solid. The mixture of unseparated ***rac*/*meso*-L^1** was prepared according to the same synthetic procedure; purification with flash column chromatography on silica gel (Petroleum ether/EtOAc/Et_3N = 100:20:1) gave a mixture of ***rac*/*meso*-L^1** (915.2 mg, 69% yield; *rac/meso* NMR ratio 35:9) as a white solid.

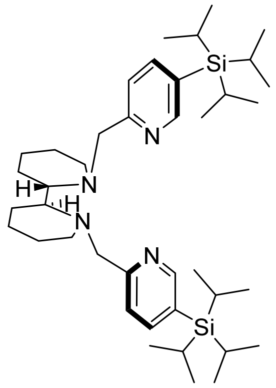

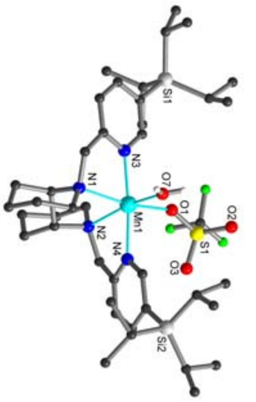

rac-**C1**

(*rac*)-1,1'-Bis((5-(triisopropylsilyl)pyridin-2-yl)methyl)-2,2'-bipiperidine (*rac*-L[1], the structure of ***rac*-L[1]** was assigned based on the X-ray structure of ***rac*-C1**; see Section 13): **^{1}H NMR** (400 MHz, $CDCl_3$) δ (ppm): 8.59 (s, 2H), 7.71 (dd, *J* = 7.6, 1.6 Hz, 2H), 7.43 (d, *J* = 8.0 Hz, 2H), 4.26 (d, *J* = 14.4 Hz, 2H), 3.20 (d, *J* = 14.4 Hz, 2H), 2.86 (d, *J* = 11.6 Hz, 2H), 2.70 (d, *J* = 10.4 Hz, 2H), 2.05–1.92 (m, 6H), 1.73 (d, *J* = 12.4 Hz, 2H), 1.59–1.45 (m, 6H), 1.44–1.35 (m, 6H), 1.07 (d, *J* = 7.6 Hz , 36H); **^{13}C NMR** (101 MHz, $CDCl_3$) δ (ppm): 160.4, 154.9, 143.4, 127.4, 122.2, 62.7, 60.0, 55.1, 25.8, 24.9(2C), 18.6, 10.8; **HRMS** (ESI) *m/z* $[M+H]^+$ calculated for $C_{40}H_{71}N_4Si_2^+$ 663.5212, found 663.5205.

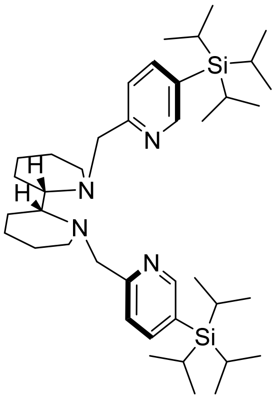

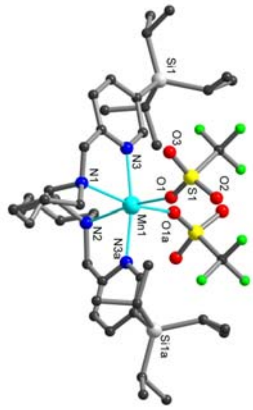

meso-**C1**

(*meso*)-1,1'-Bis((5-(triisopropylsilyl)pyridin-2-yl)methyl)-2,2'-bipiperidine (*meso*-L[1], the structure of ***meso*-L[1]** was assigned based on the X-ray structure of ***meso*-C1**; see Section 13): **^{1}H NMR** (400 MHz, $CDCl_3$) δ (ppm): 8.57 (s, 2H), 7.74 (d, *J* = 7.6 Hz, 2H), 7.50 (d, *J* = 8.0 Hz, 2H), 4.92 (d, *J* = 14.8 Hz, 2H), 3.28 (d, *J* = 14.8 Hz, 2H), 2.82 (d, *J* = 12.8 Hz, 2H), 2.59 (d, *J* = 8.4 Hz,

2H), 2.01 (t, J = 10.8 Hz, 2H), 1.76−1.63 (m, 6H), 1.46−1.26 (m, 12H), 1.08 (d, J = 7.6 Hz, 36H); **^{13}C NMR** (101 MHz, $CDCl_3$) δ (ppm): 161.2, 154.7, 143.6, 127.2, 122.0, 63.4, 60.4, 54.1, 27.3, 24.7(2C), 18.6, 10.8; **HRMS** (ESI) *m/z* $[M+H]^+$ calculated for $C_{40}H_{71}N_4Si_2^+$ 663.5212, found 663.5205.

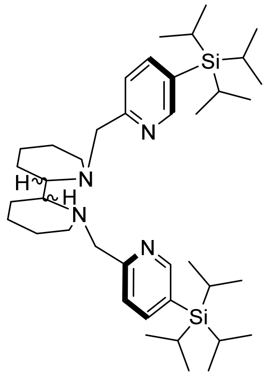

(*rac/meso*)-1,1'-Bis((5-(triisopropylsilyl)pyridin-2-yl)methyl)-2,2'-bipiperidine (*rac/meso*-L^1 *rac/meso* = 35:9**)**: **^{1}H NMR** (400 MHz, $CDCl_3$) δ (ppm): 8.60−8.55 (m, 2H), 7.76−7.68 (m, 2H), 7.52−7.40 (m, 2H), 4.92 (d, J = 14.8 Hz, 0.36 H), 4.26 (d, J = 14.0 Hz, 1.40 H), 3.31−3.16 (m, 2H), 2.92−2.78 (m, 2H), 2.76−2.54 (m, 2H), 2.08−1.88 (m, 4H), 1.78−1.65 (m, 3H), 1.58−1.31 (m, 13H), 1.08 (d, J = 7.2 Hz , 36H); **^{13}C NMR** (101 MHz, $CDCl_3$) δ (ppm): 161.1, 160.4, 154.9, 154.7, 143.6, 143.4, 127.4, 127.1, 122.2, 121.9, 62.7, 60.4, 59.9, 55.1, 54.2, 29.8, 27.3, 25.7, 24.9(2C), 24.7(2C), 18.6, 10.8; **HRMS** (ESI) *m/z* $[M+H]^+$ calculated for $C_{40}H_{71}N_4Si_2^+$ 663.5212, found 663.5205.

Synthesis of *rac*-L^2, *meso*-L^2 and *rac/meso*-L^2:

It was prepared in an analogous manner to ***rac*-L^1** and ***meso*-L^1** starting from 2,2'-bipiperidine (336.6 mg, 2.0 mmol, 1 equiv.) and 2-(chloromethyl)-4-methoxy-3-methylpyridine (720.8 mg, 4.2 mmol, 2.1 equiv.) to provide the racemic *rac*-**L^2** (342.1 mg, 39% yield) and the meso analogue *meso*-**L^2** (254.4 mg, 29% yield), as separated white solids.

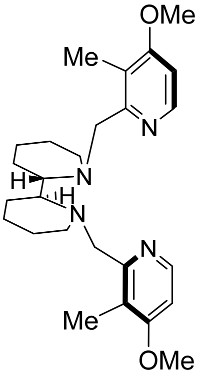

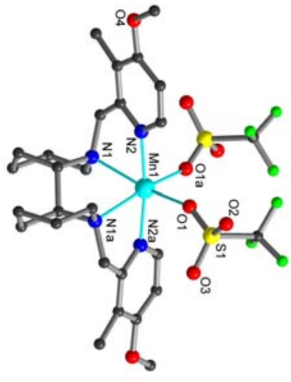

rac-**C2**

(*rac*)-1,1'-Bis((4-methoxy-3-methylpyridin-2-yl)methyl)-2,2'-bipiperidine (***rac*-L²**, the structure of ***rac*-L²** was assigned based on the X-ray structure of ***rac*-C2**; see Section 13): **^{1}H NMR** (400 MHz, $CDCl_3$) δ (ppm): 8.29 (d, *J* = 5.6 Hz, 2H), 6.62 (d, *J* = 5.6 Hz, 2H), 4.18 (d, *J* = 12.4 Hz, 2H), 3.82 (s, 6H), 3.27 (d, *J* = 12.0 Hz, 2H), 2.63 (d, *J* = 11.2 Hz, 4H), 2.24 (s, 6H), 2.05–1.95 (m, 4H), 1.67 (d, *J* = 12.8 Hz, 2H), 1.54–1.31 (m, 6H), 1.20–1.05 (m, 2H); **^{13}C NMR** (101 MHz, $CDCl_3$) δ (ppm): 163.8, 158.2, 147.7, 121.6, 104.3, 64.3, 59.6, 55.4, 54.4, 26.0, 24.9, 24.7, 10.6; **HRMS** (ESI) *m/z* $[M+H]^+$ calculated for $C_{26}H_{39}N_4O_2^+$ 439.3068, found 439.3070.

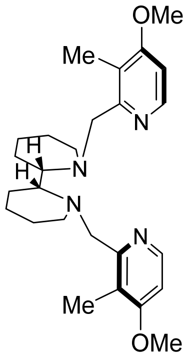

(*meso*)-1,1'-Bis((4-methoxy-3-methylpyridin-2-yl)methyl)-2,2'-bipiperidine (***meso*-L²**, the structure of ***meso*-L²** was assigned based on analysis of ^{1}H NMR of ***meso*-L²**, and comparison of ^{1}H NMR of ***meso*-L²** with ***rac*-L²**): **^{1}H NMR** (400 MHz, $CDCl_3$) δ (ppm): 8.28–8.21 (m, 2H), 6.66–6.61 (m, 2H), 4.16 (d, *J* = 12.4 Hz, 2H), 3.83 (s, 3H), 3.82 (s, 3H), 3.69 (d, *J* = 12.4 Hz, 2H), 2.82 (s, 2H), 2.67–2.57 (m, 2H), 2.32 (d, *J* = 12.0 Hz, 2H), 2.19 (s, 3H), 2.18 (s, 3H), 1.74–1.65 (m, 2H), 1.62–1.49 (m, 4H), 1.25–1.19 (m, 2H), 1.10–1.02 (m, 2H), 1.00–0.87 (m, 2H); **^{13}C NMR** (101 MHz, $CDCl_3$) δ (ppm): 163.9, 159.2, 147.1, 122.1, 104.5, 58.9, 56.1, 55.4, 48.8,

21.4, 21.1, 20.5, 10.5; **HRMS** (ESI) *m/z* $[M+H]^+$ calculated for $C_{26}H_{39}N_4O_2^+$ 439.3068, found 439.3070.

B. Synthesis of L^{3-12} [46, 50, 56, 58-62]

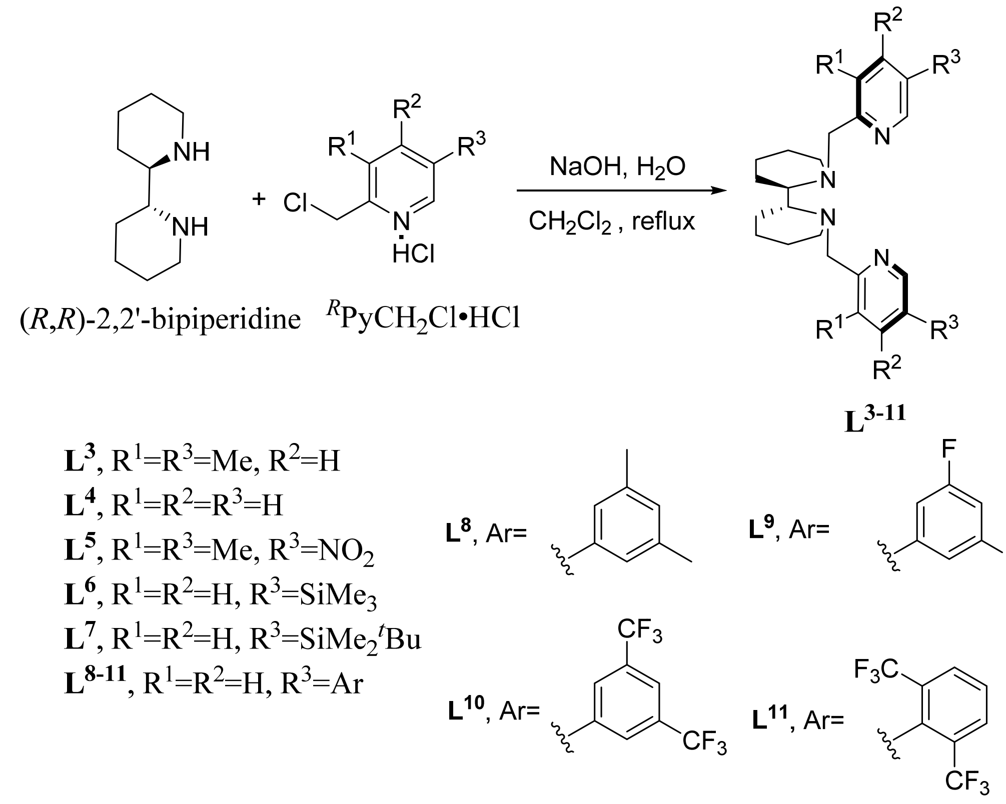

Synthesis of L^{3-5}:

L^3, L^4 and **L^5** were prepared following reported procedures [56]

Synthesis of L^6 [50, 56]**:**

A solution containing (*R,R*)-2,2'-bipiperidine (168.3 mg, 1.0 mmol, 1.0 equiv.) and NaOH (0.32 g, 8.0 mmol, 8.0 equiv.) in H_2O (5 mL) was added to a 50 mL round bottom flask charged with a stir bar and 2-(chloromethyl)-5-(trimethylsilyl)pyridine hydrochloride (496.0 mg, 2.1 mmol, 2.1 equiv.) dissolved in CH_2Cl_2 (20 mL). The combined mixture was stirred for 3 days. After the reaction was complete, the aqueous phase was extracted with CH_2Cl_2 (3 x 15 mL). The organic fractions were combined, and dried over $MgSO_4$. After concentration, the residue was purified by flash column chromatography on silica gel (petroleum ether/EtOAc/Et_3N = 100:5:1 to 100:30:1) to give **L^6** (356.3 mg, yield 72%) as a white solid.

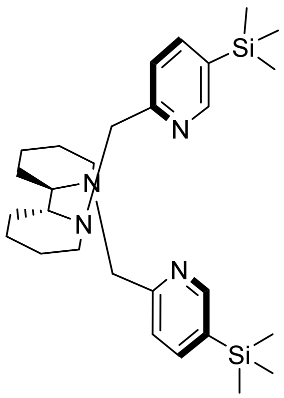

(2*R*,2'*R*)-1,1'-Bis((5-(trimethylsilyl)pyridin-2-yl)methyl)-2,2'-bipiperidine (L^6): ^{1}H NMR (400 MHz, $CDCl_3$) δ (ppm): 8.60 (s, 2H), 7.73 (dd, *J* = 7.6, 1.6 Hz, 2H), 7.43 (d, *J* = 7.6 Hz, 2H), 4.25 (d, *J* = 14.4 Hz, 2H), 3.21 (d, *J* = 14.4 Hz, 2H), 2.84 (d, *J* = 11.2 Hz, 2H), 2.73 (d, *J* = 10.4 Hz, 2H), 2.05−1.92 (m, 4H), 1.78−1.65 (m, 4H), 1.59−1.41 (m, 6H), 0.29 (s, 18H); **^{13}C NMR** (101 MHz, $CDCl_3$) δ (ppm): 160.8, 153.4, 141.6, 132.6, 122.2, 77.5, 62.4, 59.6, 55.0, 25.8, 24.9, -1.1. **HRMS** (ESI) *m/z* $[M+H]^+$ calculated for $C_{28}H_{47}N_4Si_2^+$ 495.3334, found 495.3333.

Synthesis of L^7 [50, 56]:

It was prepared in an analogous manner to **L^6** starting from (*R*,*R*)-2,2'-bipiperidine (168.3 mg, 1.0 mmol, 1 equiv.) and 5-(*tert*-butyldimethylsilyl)-2-(chloromethyl)pyridine hydrochloride (584.4 mg, 2.1 mmol, 2.1 equiv.) to provide 416.9 mg (72% yield) of a white solid.

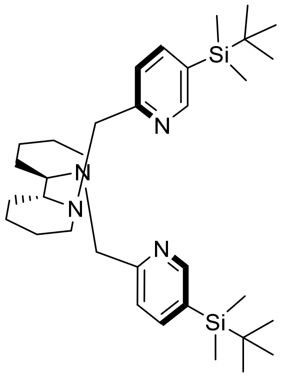

(2*R*,2'*R*)-1,1'-Bis((5-(*tert*-butyldimethylsilyl)pyridin-2-yl)methyl)-2,2'-bipiperidine (L^7): ^{1}H NMR (400 MHz, $CDCl_3$) δ (ppm): 8.60 (s, 2H), 7.72 (dd, *J* = 7.6, 2.0 Hz, 2H), 7.42 (d, *J* = 7.6 Hz, 2H), 4.26 (d, *J* = 14.0 Hz, 2H), 3.21 (d, *J* = 14.4 Hz, 2H), 2.84 (d, *J* = 10.8 Hz, 2H), 2.71 (d, *J* = 10.4 Hz, 2H), 2.05−1.91 (m, 4H), 1.73 (d, *J* = 10.4 Hz, 2H), 1.59−1.38 (m, 6H), 1.26−1.11 (m,

2H), 0.88 (s, 18H), 0.28 (s, 12H); **^{13}C NMR** (101 MHz, $CDCl_3$) δ (ppm): 160.7, 154.2, 142.6, 130.2, 122.1, 77.5, 62.6, 59.7, 55.0, 26.5, 25.8, 24.9, 17.0, -6.2;
HRMS (ESI) *m/z* $[M+H]^+$ calculated for $C_{34}H_{59}N_4Si_2^+$ 579.4273, found 579.4274.

Synthesis of L^8 [56, 59-60]:

It was prepared in an analogous manner to **L^6** starting from (*R,R*)-2,2'-bipiperidine (168.3 mg, 1.0 mmol, 1 equiv.) and 2-(chloromethyl)-5-(3,5-dimethylphenyl)pyridine hydrochloride (563.2 mg, 2.1 mmol, 2.1 equiv.) to provide 424.7 mg (76% yield) of a white solid.

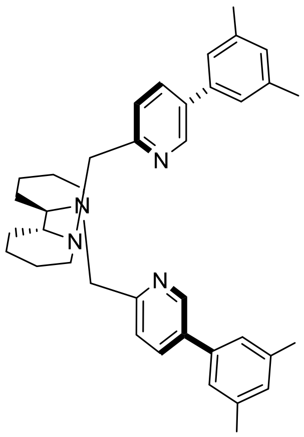

(2*R*,2'*R*)-1,1'-bis((5-(3,5-dimethylphenyl)pyridin-2-yl)methyl)-2,2'-bipiperidine (L^8): ^{1}H NMR (400 MHz, $CDCl_3$) δ (ppm): 8.74 (d, J = 2.4 Hz, 2H), 7.80 (dd, J = 8.0, 2.0 Hz, 2H), 7.50 (d, J = 8.0 Hz, 2H), 7.18 (s, 4H), 7.03 (s, 2H), 4.32 (d, J = 14.4 Hz, 2H), 3.31 (d, J = 14.0 Hz, 2H), 2.89 (d, J = 11.6 Hz, 2H), 2.79 (d, J = 10.0 Hz, 2H), 2.39 (s, 12H), 2.15–1.92 (m, 4H), 1.78 (d, J = 12.8 Hz, 2H), 1.57–1.46 (m, 4H), 1.35–1.12 (m, 4H). **^{13}C NMR** (101 MHz, $CDCl_3$) δ (ppm): 158.9, 147.5, 138.6, 137.9, 134.9, 134.8, 129.5, 125.0, 122.5, 62.4, 59.2, 54.8, 25.7, 25.1, 24.8, 21.4.
HRMS (ESI) *m/z* $[M+H]^+$ calculated for $C_{38}H_{47}N_4^+$ 559.3795, found 559.3806.

Synthesis of L^9 [56, 59-60]:

It was prepared in an analogous manner to **L^6** starting from (*R,R*)-2,2'-bipiperidine (168.3 mg, 1.0 mmol, 1 equiv.) and 2-(chloromethyl)-5-(3,5-difluorophenyl)pyridine hydrochloride (579.8 mg, 2.1 mmol, 2.1 equiv.) to provide 419.5 mg (73% yield) of a white solid.

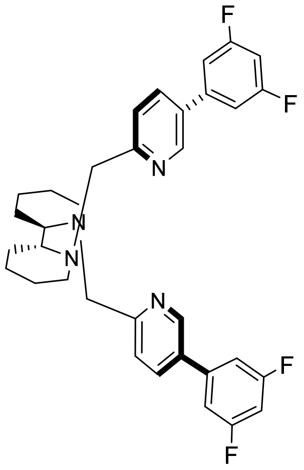

(2*R*,2'*R*)-1,1'-bis((5-(3,5-difluorophenyl)pyridin-2-yl)methyl)-2,2'-bipiperidine (L⁹): ^{1}**H NMR** (400 MHz, CDCl$_3$) δ (ppm): 8.73 (s, 2H), 7.77 (dd, *J* = 8.0, 1.2 Hz, 2H), 7.55 (d, *J* = 8.0 Hz, 2H), 7.08 (d, *J* = 6.4 Hz, 4H), 6.83 (t, *J* = 8.8 Hz, 2H), 4.33 (d, *J* = 14.8 Hz, 2H), 3.33 (d, *J* = 14.4 Hz, 2H), 2.87 (d, *J* = 11.6 Hz, 2H), 2.78 (d, *J* = 10.4 Hz, 2H), 2.12–2.01 (m, 2H), 1.96 (d, *J* = 13.6 Hz, 2H), 1.78 (d, *J* = 8.0 Hz, 2H), 1.64–1.46 (m, 6H), 1.28–1.19 (m, 2H). ^{13}**C NMR** (101 MHz, CDCl$_3$) δ (ppm): 163.6 (dd, *J* = 249.9, 13.1 Hz), 160.8, 147.3, 141.3 (t, *J* = 9.8 Hz), 134.8, 132.6, 122.8, 110.0 (dd, *J* = 18.8, 7.3 Hz), 103.3 (t, *J* = 25.6 Hz), 62.5, 59.2, 55.0, 25.7, 25.2, 24.9; ^{19}**F NMR** (376 MHz, CDCl$_3$) δ (ppm): -108.89;
HRMS (ESI) *m/z* $[M+H]^+$ calculated for $C_{34}H_{35}F_4N_4^+$ 575.2792, found 575.2799.

Synthesis of L^{10} [56, 59-60]:

It was prepared in an analogous manner to **L^6** starting from (*R*,*R*)-2,2'-bipiperidine (168.3 mg, 1.0 mmol, 1 equiv.) and 5-(3,5-bis(trifluoromethyl)phenyl)-2-(chloromethyl)pyridine hydrochloride (789.9 mg, 2.1 mmol, 2.1 equiv.) to provide 542.3 mg (70% yield) of a white solid.

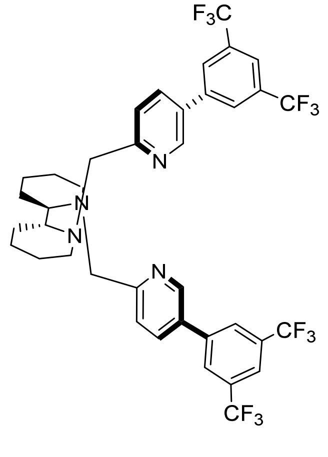

(2*R*,2'*R*)-1,1'-Bis((5-(3,5-bis(trifluoromethyl)phenyl)pyridin-2-yl)methyl)-2,2'-bipiperidine (L^{10}): ^{1}H NMR (400 MHz, $CDCl_3$) δ (ppm): 8.79 (s, 2H), 7.99 (s, 4H), 7.90 (s, 2H), 7.86 (dd, *J* = 8.0, 2.4 Hz, 2H), 7.63 (d, *J* = 8.0 Hz, 2H), 4.34 (d, *J* = 14.8 Hz, 2H), 3.38 (d, *J* = 10.8 Hz, 2H), 2.90–2.80 (m, 4H), 2.15–2.05 (m, 2H), 1.97 (d, *J* = 13.6 Hz, 2H), 1.79 (d, *J* = 14.8 Hz, 2H), 1.65–1.47 (m, 6H), 1.29–1.20 (m, 2H); **^{13}C NMR** (101 MHz, $CDCl_3$) δ (ppm): 161.4, 147.5, 140.3, 135.1, 132.5 (q, *J* = 34.3 Hz), 129.5 (q, *J* = 3.9 Hz), 127.3 , 123.3 (q, *J* = 273.8 Hz),123.0, 121.7 (q, *J* = 3.5 Hz), 62.3, 59.2, 55.0, 25.7, 25.1, 24.8. **^{19}F NMR** (376 MHz, $CDCl_3$) δ (ppm): -62.89; **HRMS** (ESI) *m/z* $[M+H]^+$ calculated for $C_{38}H_{35}F_{12}N_4^+$ 775.2665, found 775.2676.

Synthesis of L^{11} [56, 59-60]:

It was prepared in an analogous manner to **L^6** starting from (*R*,*R*)-2,2'-bipiperidine (168.3 mg, 1.0 mmol, 1 equiv.) and 5-(2,6-bis(trifluoromethyl)phenyl)-2-(chloromethyl)pyridine hydrochloride (789.9 mg, 2.1 mmol, 2.1 equiv.) to provide 511.3 mg (66% yield) of a white solid.

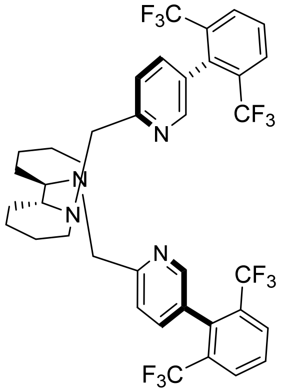

(2*R*,2'*R*)-1,1'-Bis((5-(2,6-bis(trifluoromethyl)phenyl)pyridin-2-yl)methyl)-2,2'-bipiperidine (L^{11}): ^{1}H NMR (400 MHz, $CDCl_3$) δ (ppm): 8.42 (s, 2H), 7.98 (d, *J* = 7.6 Hz, 4H), 7.66 (t, *J* = 8.0 Hz, 2H), 7.56–7.50 (m, 4H), 4.37 (d, *J* = 14.4 Hz, 2H), 3.34 (d, *J* = 14.4 Hz, 2H), 2.85 (d, *J* = 12.0 Hz, 2H), 2.77 (d, *J* = 10.4 Hz, 2H), 2.14–2.03 (m, 2H), 1.98 (d, *J* = 13.6 Hz, 2H), 1.79 (d, *J* = 12.4 Hz, 2H), 1.65–1.43 (m, 6H), 1.29–1.21 (m, 2H); **^{13}C NMR** (101 MHz, $CDCl_3$) δ (ppm): 160.7, 149.1, 137.7, 137.0, 131.9 (q, *J* = 29.8 Hz), 129.4 (d, *J* = 5.2 Hz), 128.7, 128.1, 123.2(q, *J* = 275.7 Hz), 121.2, 62.7, 59.8, 55.0, 25.7, 25.2, 24.8. **^{19}F NMR** (376 MHz, $CDCl_3$) δ (ppm): -57.40 **HRMS** (ESI) *m/z* $[M+H]^+$ calculated for $C_{38}H_{35}F_{12}N_4^+$ 775.2665, found 775.2669.

C. Synthesis of $L^{12\text{-}16}$

L[12] [62], L[13] [50], L[14] [59], L[15] [56] and L[16] [62] were prepared following reported procedures

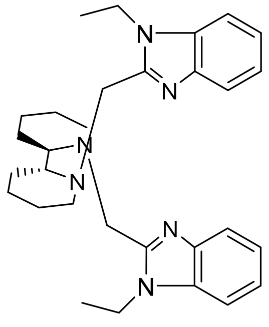

(2*R*,2'*R*)-1,1'-Bis((1-ethyl-1*H*-benzo[d]imidazol-2-yl)methyl)-2,2'-bipiperidine (L[12]): ^{1}H NMR (400 MHz, $CDCl_3$) δ (ppm): 7.78−7.72 (m, 2H), 7.35−7.28 (m, 2H), 7.26−7.20 (m, 4H), 4.53−4.40 (m, 2H), 4.35 (d, *J* = 13.6 Hz, 2H), 4.31−4.19 (m, 2H), 3.55 (d, *J* = 13.2 Hz, 2H), 2.74 (d, *J* = 10.0 Hz, 2H), 2.68 (d, *J* = 11.6 Hz, 2H), 2.19 (dt, *J* = 11.6, 2.8 Hz, 2H), 1.94−1.88 (m, 4H), 1.78−1.70 (m, 2H), 1.59−1.48 (m, 4H), 1.42 (t, *J* = 7.2 Hz, 6H), 1.31−1.18 (m, 2H). **^{13}C NMR** (101 MHz, $CDCl_3$) δ (ppm): 151.6, 142.6, 135.3, 122.5, 121.9, 119.7, 109.3, 63.8, 54.7, 52.3, 38.7, 25.5, 25.1, 24.4, 15.1.

HRMS (ESI) *m/z* $[M+H]^+$ calculated for $C_{30}H_{41}N_6^+$ 485.3387, found 485.3391.

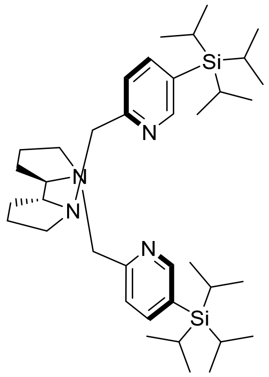

(2*R*,2'*R*)-1,1'-Bis((5-(triisopropylsilyl)pyridin-2-yl)methyl)-2,2'-bipyrrolidine (L[13]): ^{1}H NMR (400 MHz, $CDCl_3$) δ (ppm): 8.57 (s, 2H), 7.70 (dd, *J* = 7.6, 1.6 Hz, 2H), 7.37 (d, *J* = 7.6 Hz, 2H), 4.16 (d, *J* = 14.0 Hz, 2H), 3.48 (d, *J* = 14.4 Hz, 2H), 3.03 (quint, *J* = 4.4 Hz, 2H), 2.77 (t, *J* = 6.4 Hz, 2H), 2.23 (q, *J* = 8.8 Hz, 2H), 1.83−1.74 (m, 4H), 1.73−1.65 (m, 4H), 1.45−1.32 (m, 6H), 1.06 (d, *J* = 8.0 Hz, 36H); **^{13}C NMR** (101 MHz, $CDCl_3$) δ (ppm): 160.5, 154.8, 143.5, 127.5, 122.3, 65.5, 61.4, 55.7, 25.9, 23.6, 18.6, 10.8.

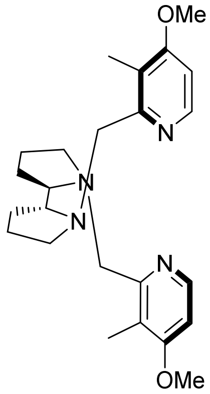

(2*R*,2'*R*)-1,1'-Bis((4-methoxy-3-methylpyridin-2-yl)methyl)-2,2'-bipyrrolidine (L[14]): ^{1}H NMR (400 MHz, $CDCl_3$) δ (ppm): 8.25 (d, *J* = 6.4 Hz, 2H), 6.66 (d, *J* = 6.4 Hz, 2H), 4.08 (d, *J* = 12.0 Hz, 2H), 3.84 (s, 6H), 3.38 (d, *J* = 11.6 Hz, 2H), 2.74 (t, *J* = 8.0 Hz, 2H), 2.61 (t, *J* = 6.8 Hz, 2H), 2.28–2.19 (m, 2H), 2.23 (s, 6H),1.76–1.46 (m, 8H). **^{13}C NMR** (101 MHz, $CDCl_3$) δ (ppm): 163.9, 158.4. 147.3, 121.8, 104.8, 65.4, 60.7, 55.6, 55.4, 26.1, 24.2, 10.4.

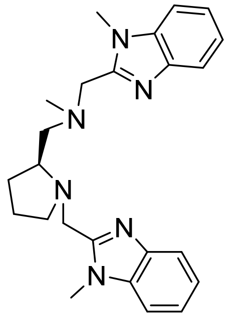

(*S*)-*N*-methyl-1-(1-methyl-1*H*-benzo[*d*]imidazol-2-yl)-*N*-((1-((1-methyl-1*H*-benzo[*d*]imidazol-2-yl)methyl)pyrrolidin-2-yl)methyl)methanamine (L[15]): ^{1}H NMR (400 MHz, $CDCl_3$) δ (ppm): 7.68–7.60 (m, 2H), 7.22–7.10 (m, 6H), 4.19 (d, *J* = 13.2 Hz, 1H), 3.72 (s, 3H), 3.70–3.58 (m, 3H), 3.57 (s, 3H), 2.72–2.60 (m, 2H), 2.46–2.40 (m, 1H), 2.36–2.21 (m, 2H), 2.18 (s, 3H), 1.93–1.82 (m, 1H), 1.59–1.38 (m, 3H); **^{13}C NMR** (101 MHz, $CDCl_3$) δ (ppm): 152.4, 151.8, 142.3, 136.3, 136.2, 122.6, 122.4, 122.0, 121.8, 119.7, 119.6, 109.2, 109.1, 62.7, 62.1, 56.1, 55.0, 52.5, 43.3, 30.4, 30.1, 29.9, 22.7.

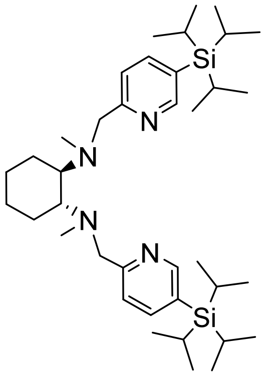

(1*R*,2*R*)-*N*¹,*N*²-dimethyl-*N*¹,*N*²-bis((5-(triisopropylsilyl)pyridin-2-yl)methyl)cyclohexane-1,2-diamine (L^{16}): **^{1}H NMR** (400 MHz, $CDCl_3$) δ (ppm): 8.51 (s, 2H), 7.65 (d, *J* = 8.0 Hz, 2H), 7.56 (d, *J* = 7.6 Hz, 2H), 3.88 (d, *J* = 14.8 Hz, 2H), 3.76 (d, *J* = 14.8 Hz, 2H), 2.63 (d, *J* = 9.2 Hz, 2H), 2.25 (s, 6H), 1.98–1.90 (m, 2H), 1.71 (d, *J* = 8.4 Hz, 2H), 1.39–1.30 (m, 6H), 1.29–1.09 (m, 4H), 1.02 (d, *J* = 8.0 Hz, 36H)**;** **^{13}C NMR** (101 MHz, $CDCl_3$) δ (ppm): 161.5, 154.4, 143.3, 127.1, 122.2, 64.8, 60.6, 36.8, 26.1, 25.9, 18.5, 10.7.

4.3 Procedures for preparation of manganese catalysts [46, 50, 56, 58-62]

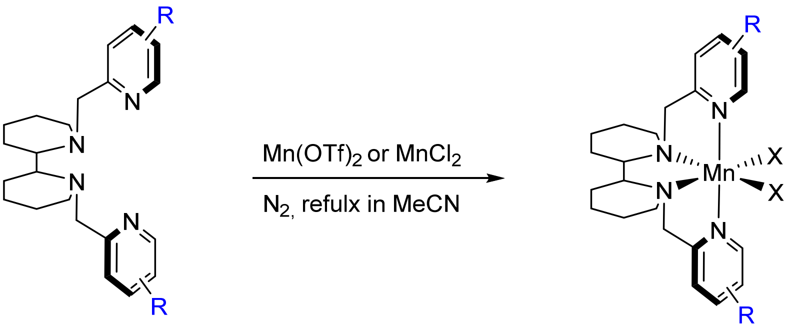

L^{1-12}

$MnL^{1-12}(OTf)_2$ **C1-12**

MnL^1Cl_2 **C17**

C3-5 and **C13-16** were prepared following the procedures reported by White [46], Costas [49, 59, 62] and Li group [56], *et. al.*

C1, **C2** and **C17** were prepared according to the following procedure:

L^1 or **L^2** (0.5 mmol, 1 equiv.) and $Mn(OTf)_2$ (0.5 mmol, 177 mg) or $MnCl_2$ (0.5 mmol, 63 mg) were placed in an oven-dried, resealable Schlenk tube. The tube was capped with a Teflon screwcap, evacuated, and backfilled with nitrogen. The screw cap was replaced with a rubber septum, and dry CH_3CN (4.0 mL) was added via a syringe. The tube was purged with nitrogen for

1-2 minutes, and then the septum was replaced with the Teflon screw cap. The tube was sealed, and the reaction mixture was heated at 80 °C overnight. Then the resulting mixture was cooled to room temperature, and the solvent was removed under reduced pressure to afford the desired manganese catalyst.

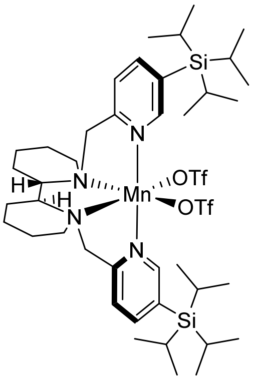

rac-**C1**

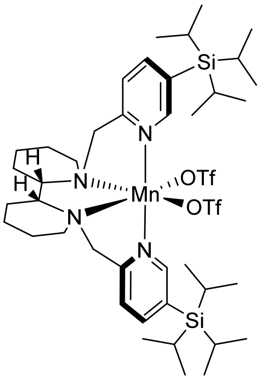

meso-**C1**

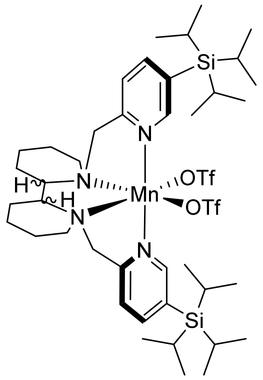

meso/rac-**C1**

Rac*-C1** was prepared according to the above procedure starting from (rac*-L**[1]) and $Mn(OTf)_2$ to obtain the product as a yellow solid. HRMS (ESI) *m/z* calcd for $C_{41}H_{70}F_3MnN_4O_3SSi_2$ $[M-OTf]^+$: 866.4040, found: 866.4026. The X-ray structure of ***rac*-C1** has been determined and is presented in Section **13**.

Meso*-C1** was prepared according to the above procedure starting from (meso*-L**[1]) and $Mn(OTf)_2$ to obtain the product as a yellow solid. HRMS (ESI) m/z calcd for $C_{41}H_{70}F_3MnN_4O_3SSi_2$ $[M-OTf]^+$: 866.4040, found: 866.4027. The X-ray structure of ***meso*-C1** has been determined and is presented in Section **13**.

Rac/meso*-C1** was prepared in analogous manner starting from a mixture of ***rac*-L**[1] and ***meso*-L**[1] (rac*/*meso*-L**[1], 35/9) and $Mn(OTf)_2$ to obtain the product as a yellow solid comprised of ***rac*-C1** and ***meso*-C1**.

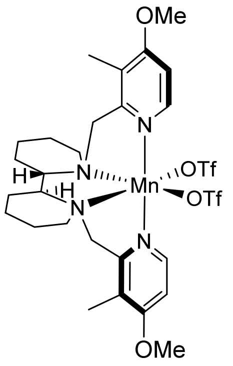

***rac*-C2**

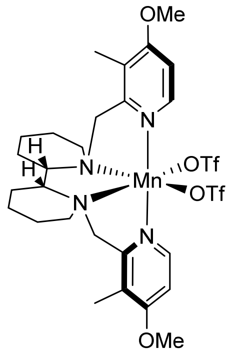

***meso*-C2**

Rac*-C2** was prepared according to the above procedure starting from (rac*-L²**) and $Mn(OTf)_2$ to obtain the product as a yellow solid. **HRMS** (ESI) *m/z* calcd for $C_{27}H_{38}F_3MnN_4O_5S$ $[M\text{-}OTf]^+$: 642.1896, found: 642.1890; The X-ray structure of ***rac*-C2** has been determined and is presented in Section **13**.

Meso*-C2** was prepared according to the above procedure starting from (meso*-L¹**) and $Mn(OTf)_2$ to obtain the product as a yellow solid. **HRMS** (ESI) *m/z* calcd for $C_{27}H_{38}F_3MnN_4O_5S$ $[M\text{-}OTf]^+$: 642.1896, found: 642.1890;

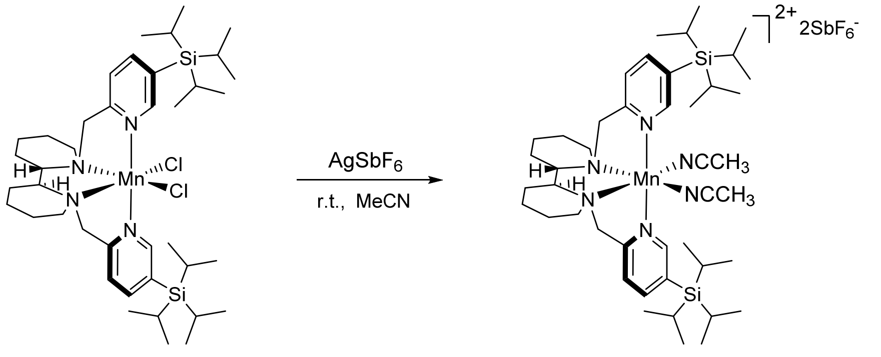

rac-**C17** *rac*-**C18**

Rac*-C17**) was prepared according to the above procedure starting from (rac*-L1**) and $MnCl_2$ to obtain the product as a yellow solid. HRMS (ESI) *m/z* calcd for $C_{40}H_{70}ClMnN_4Si_2^+$ $[M\text{-}Cl]^+$: 752.4203, found: 752.4193.

***Rac*-C18** were prepared according to the following procedure: ***Rac*-C17** (789.1 mg, 1 mmol, 1 equiv.) and silver hexafluoroantimonate ($AgSbF_6$, 687.3 mg, 2 mmol, 2 equiv.) were placed in an oven-dried, resealable Schlenk tube. The screw cap was replaced with a rubber septum, and dry CH_3CN (4.0 mL) was added via syringe. The tube was purged with nitrogen for 1-2 minutes, after which the septum was replaced with a Teflon screw cap. The tube was wrapped in aluminum foil to protect the silver salts from light. After stirring for 24 hours, the reaction mixture was filtered through Celite® and concentrated to dryness. The resulting solid was redissolved in CH_3CN, and the filtration/concentration process was repeated twice to ensure complete removal of residual silver salts. The final solution was dried under reduced pressure to afford the desired manganese catalyst ***rac*-C18** as a yellow powder. HRMS (ESI) *m/z* calcd for $C_{40}H_{70}F_6MnN_4SbSi_2^+$ $[M\text{-}SbF_6]^+$: 952.3456, found: 952.3451.

5. Optimization of reaction conditions for oximation

Table S-1. Screening manganese catalysts for oximation of cyclododecane[a]

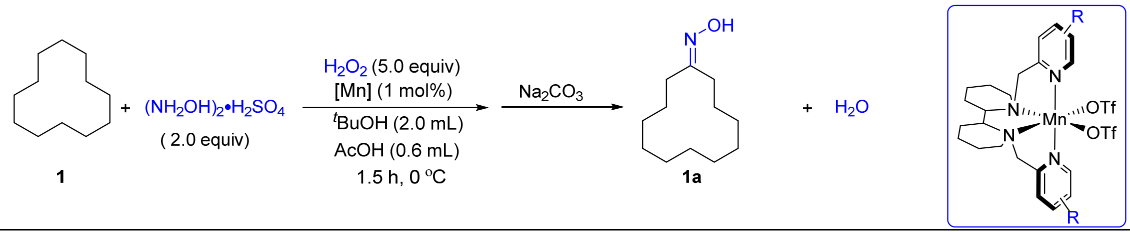

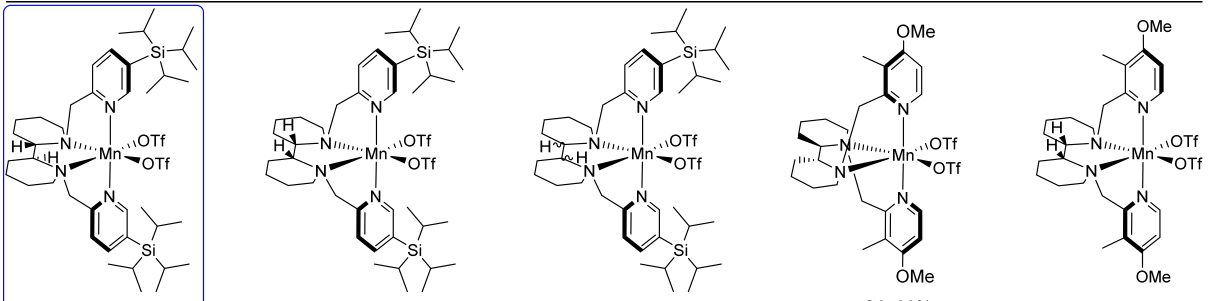

rac-**C1** 96% yield | *meso*-**C1** 93% yield | *meso/rac*-**C1** 94% yield | (2*R*,2*R*')-**C2** 88% yield | *meso*-**C2** 2% yield

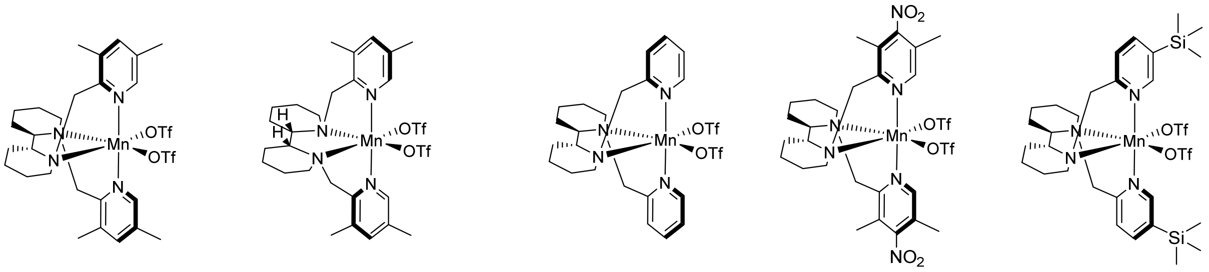

(2*R*,2*R*')-**C3** 85% yield | *meso*-**C3** 2% yield | (2*R*,2*R*')-**C4** 80% yield | (2*R*,2*R*')-**C5** 48% yield | (2*R*,2*R*')-**C6** 71% yield

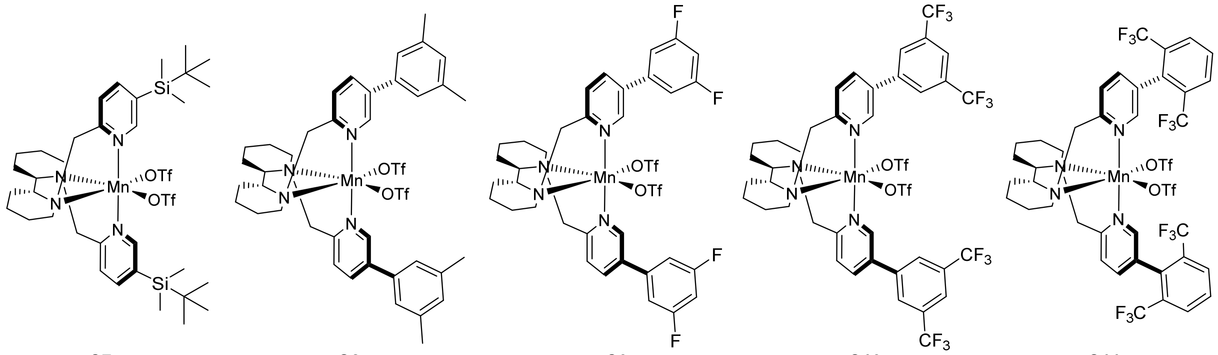

(2*R*,2*R*')-**C7** 74% yield | (2*R*,2*R*')-**C8** 58% yield | (2*R*,2*R*')-**C9** 54% yield | (2*R*,2*R*')-**C10** 48% yield | (2*R*,2*R*')-**C11** 45% yield

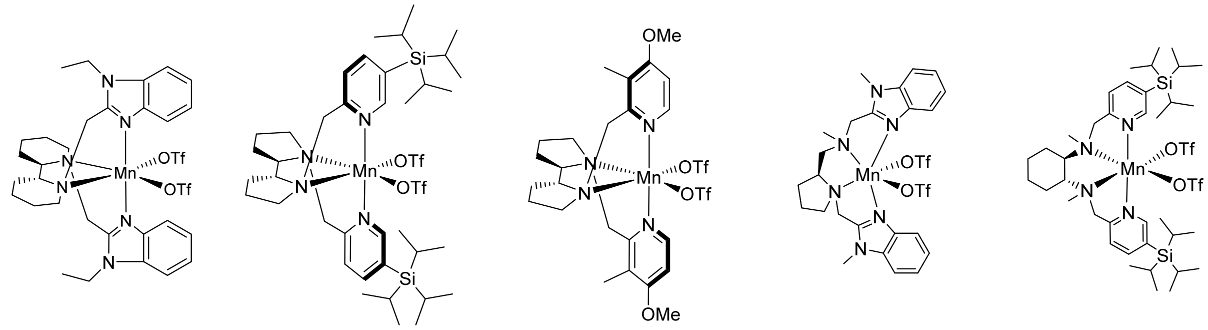

(2*R*,2*R*')-**C12** 66% yield | (2*R*,2*R*')-**C13** 48% yield | (2*R*,2*R*')-**C14** 65% yield | (*S*)- **C15** 19% yield | (2*R*,2*R*')-**C16** 85% yield

[a] General conditions: **1** (1 equiv., 0.5 mmol), $(NH_2OH)_2 \bullet H_2SO_4$ (2 equiv., 1.0 mmol), [Mn] (1 mol%), ^tBuOH (1 mL), AcOH (0.6 mL) and H_2O_2 (284 uL, 2.5 mmol, 5 equiv., 30% wt. in H_2O) in 1.0 mL ^tBuOH added dropwise via a syringe pump over 1 h under stirring at 0 °C. After stirring for an additional 0.5 h, the solution was quenched with Na_2SO_3 and then basified with Na_2CO_3 powder for 0.5 h at 0 °C; yield determined by ^1H NMR of crude reaction mixture.

Table S-2. Optimization conditions for oximation of cyclododecane[a]

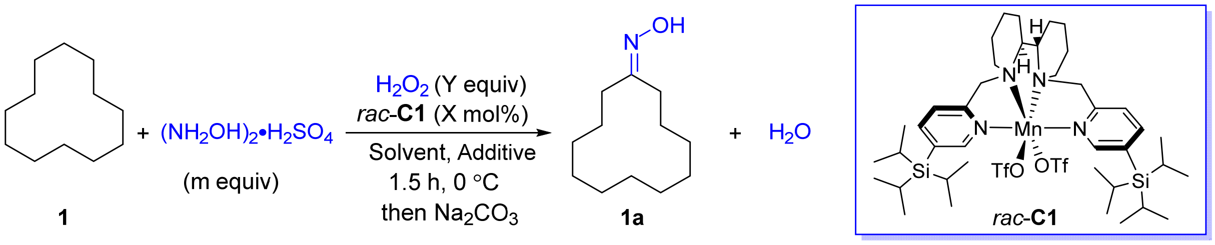

Entry	[Mn] (X mol%)	$(NH_2OH)_2$•H_2SO_4 (m equiv.)	H_2O_2 (Y equiv.)	Solvent (Z mL)	Additive (n mL)	Yield of[b] **1a** (%)
1	*rac*-**C1** (1.0)	2.0	5.0	^{t}BuOH (2.0)	AcOH (0.6)	96
2	*rac*-**L**[1](1.0)+Mn(OTf)$_2$ (1.0)	2.0	5.0	^{t}BuOH (2.0)	AcOH (0.6)	96
3	*rac*-**L**[1](1.0)+Mn(OAc)$_2$ (1.0)	2.0	5.0	^{t}BuOH (2.0)	AcOH (0.6)	95
4	*rac*-**L**[1](1.0)+Mn(OAc)$_3$ (1.0)	2.0	5.0	^{t}BuOH (2.0)	AcOH (0.6)	82
5	*rac*-**L**[1](1.0)+MnSO$_4$ (1.0)	2.0	5.0	^{t}BuOH (2.0)	AcOH (0.6)	54
6	*rac*-**L**[1](1.0)+Mn(NO$_3$)$_2$ (1.0)	2.0	5.0	^{t}BuOH (2.0)	AcOH (0.6)	93
7	*rac*-**C1** (0.5)	2.0	5.0	^{t}BuOH (2.0)	AcOH (0.6)	66
8	*rac*-**C1** (1.0)	2.0	5.0	^{t}BuOH (1.5)	AcOH (0.6)	84
9	*rac*-**C1** (1.0)	2.0	5.0	^{t}BuOH (2.5)	AcOH (0.6)	95
10	*rac*-**C1** (1.0)	2.0	5.0	^{t}BuOH (3.0)	AcOH (0.6)	95
11	*rac*-**C1** (1.0)	1.5	5.0	^{t}BuOH (2.0)	AcOH (0.6)	85
12	*rac*-**C1** (1.0)	2.5	5.0	^{t}BuOH (2.0)	AcOH (0.6)	91
13	*rac*-**C1** (1.0)	2.0	3.0	^{t}BuOH (2.0)	AcOH (0.6)	67
14	*rac*-**C1** (1.0)	2.0	4.0	^{t}BuOH (2.0)	AcOH (0.6)	85
15	*rac*-**C1** (1.0)	2.0	6.0	^{t}BuOH (2.0)	AcOH (0.6)	80
16	*rac*-**C1** (1.0)	2.0	5.0	^{t}BuOH (2.0)	AcOH (1.4)	80
17	*rac*-**C1** (1.0)	2.0	5.0	^{t}BuOH (2.0)	AcOH (1.2)	85
18	*rac*-**C1** (1.0)	2.0	5.0	^{t}BuOH (2.0)	AcOH (1.0)	89
19	*rac*-**C1** (1.0)	2.0	5.0	^{t}BuOH (2.0)	AcOH (0.8)	91
20	*rac*- **C1** (1.0)	2.0	5.0	^{t}BuOH (2.0)	AcOH (0.4)	86
21	*rac*-**C1** (1.0)	2.0	5.0	^{t}BuOH (2.0)	ClCH$_2$CO$_2$H (1.0[c])	76
22	*rac*-**C1** (1.0)	2.0	5.0	^{t}BuOH (2.0)	HCO$_2$H (1.0)	<5
23	*rac*-**C1** (1.0)	2.0	5.0	^{t}BuOH (2.0)	Pivalic acid (1.0)	77
24	*rac*-**C1** (1.0)	2.0	5.0	CH$_3$CN (2.0)	AcOH (1.0)	75
25	*rac*-**C1** (1.0)	2.0	5.0	DCM (2.0)	AcOH (1.0)	73
26	*rac*-**C1** (1.0)	2.0	5.0	EtOAc (2.0)	AcOH (1.0)	71
27	*rac*-**C1** (1.0)	2.0	5.0	MeOH (2.0)	AcOH (1.0)	<5
28	*rac*-**C1** (1.0)	2.0	5.0	CF$_3$CH$_2$OH (2.0)	AcOH (1.0)	67
29	*rac*-**C1** (1.0)	2.0	5.0	Et$_2$O (2.0)	AcOH (1.0)	23
30	*rac*-**C1** (1.0)	2.0	5.0	THF (2.0)	AcOH (1.0)	24
31	*rac*-**C1** (1.0)	2.0[d]	5.0	^{t}BuOH (2.0)	AcOH (0.6)	0
32[e]	*rac*-**C1** (1.0)	2.0	5.0	^{t}BuOH (8.0)	AcOH (2.4)	95
33[e]	*rac*-**C17** (1.0)	2.0	5.0	^{t}BuOH (8.0)	AcOH (2.4)	82
34[e]	*rac*-**C18** (1.0)	2.0	5.0	^{t}BuOH (8.0)	AcOH (2.4)	95
35[e]	*rac*-**C1** (0.2)	2.0	5.0	^{t}BuOH (8.0)	AcOH (2.4)	45
36[e]	*rac*-**C18** (0.2)	2.0	5.0	^{t}BuOH (8.0)	AcOH (2.4)	46

[a] General conditions: **1** (1 equiv., 0.5 mmol), $(NH_2OH)_2$•H_2SO_4 (m equiv.), *rac*-**C1** (X mol%), solvent (Z/2 mL), additive (n mL), and H_2O_2 (Y equiv., 30% wt. in H_2O) in Z/2 mL ^{t}BuOH added dropwise via a syringe pump over 1 h with under stirring at 0 °C;

after stirring for an additional 0.5 h, the solution was quenched with Na_2SO_3 and then basified with Na_2CO_3 powder for 0.5 h at 0 °C; [b] yield determined by ^{1}H NMR of crude reaction mixture; [c] 1.0 equiv.; [d] NH_2OH instead of $(NH_2OH)_2$•H_2SO_4. [e] **1** (2.0 mmol).

Table S-3. Effects of inorganic salts with different anions and cations on the manganese-catalyzed oxidation of cyclododecane[a]

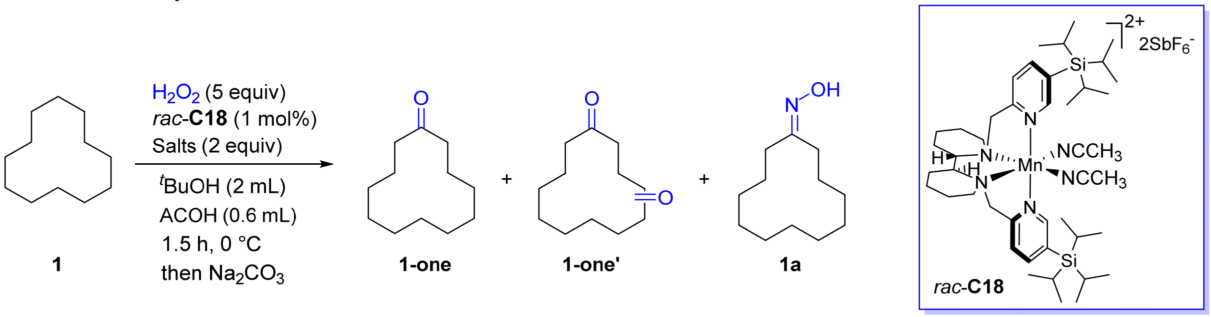

Entry	Inorganic salts	Remaining 1 (%)	1-one (%)	1-one' (%)	1a (%)
1	Na_2SO_4	6	78	16	/
2[b]	Na_2SO_4	38	52	10	/
3[c]	Na_2SO_4	0	55	45	/
4	$NaHSO_4$	24	66	10	/
5	$(NH_4)_2SO_4$	39	54	7	/
6	$(NMe_4)_2SO_4$	64	36	0	/
7	NaCl	83	17	0	/
8	NH_2OH•HCl	>99	0	0	0
9	Na_3PO_4	93	7	0	/
10	$(NH_2OH)_3$•H_3PO_4	>95	0	0	trace
11	NH_2OH•$HSbF_6$	8	0	0	92
12	$(NH_2OH)_2$•H_2SO_4	7	0	0	93
13	None	0	45	55	/

[a] General conditions: **1** (1 equiv., 0.5 mmol), inorganic salt (2 equiv. 1.0 mmol), ***rac*-18** (1 mol%), ^{t}BuOH (1 mL), AcOH (0.6 mL), and H_2O_2 (5 equiv., 2.5 mmol, 284 uL, 30% wt. in H_2O) in 1 mL of ^{t}BuOH introduced with a syringe pump over 1 h under stirring at 0 °C; after stirring for an additional 0.5 h, the solution was quenched with Na_2SO_3 and then basified with Na_2CO_3 powder for 0.5 h at 0 °C; NMR yield. [b] 0.5 mL H_2O added to increase the solubility of salt. [c] Stirred for an additional 1.5 h.

Table S-4. Effects of catalyst loading on the oximation of **1**[a].

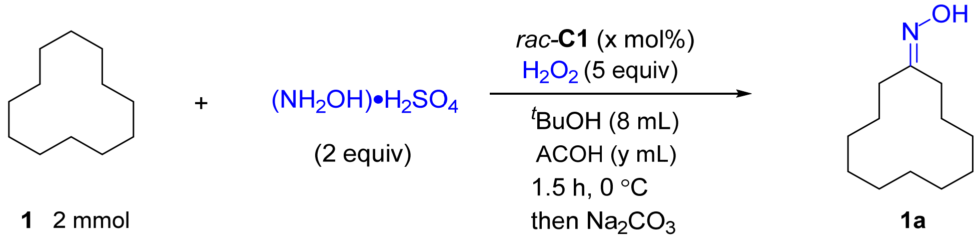

Entry	*rac*-C1 (x mol%)	AcOH (y mL)	Remaining 1 (%)	1a (%)
1	0.2	2.4	64	36
2	0.2	3.6	39	61
3	0.2	4.8	10	90 (84)[b]
4	0.1	4.8	52	48

5	0.1	6.0	28	72
6	0.1	7.2	60	40
7	0.1	8.4	74	26
8	0.1	9.6	75	25

[a] General conditions: **1** (1 equiv., 2 mmol), $(NH_2OH)_2•H_2SO_4$ (2 equiv. 4.0 mmol), ***rac*-1** (x mol%), ${}^tBuOH$ (4 mL), AcOH (y mL), and H_2O_2 (5 equiv., 10 mmol, 1136 uL, 30% wt. in H_2O) in 4 mL of ${}^tBuOH$ introduced with a syringe pump over 1 h under stirring at 0 °C; after stirring for an additional 0.5 h, the solution was quenched with Na_2SO_3 and then basified with Na_2CO_3 powder for 0.5 h at 0 °C; yield determined by ^1H NMR of crude reaction mixture. [b] Isolated yield.

Table S-5. Effect of the amount of AcOH on oximation of **17**[a].

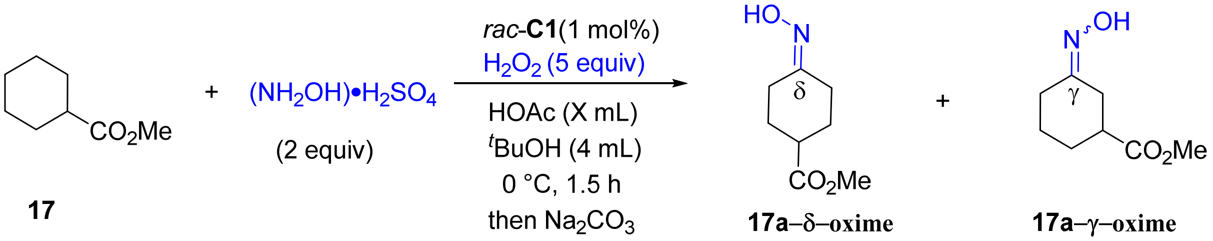

**Entry**	AcOH (X mL)	Isolate yield of **17a** (%)[b]	**17a-δ-oxime**/ **17a-γ-oxime**[b]	(*E*)–**17a-γ-oxime**/ (*Z*)–**17a-γ-oxime**[b]
**1**	1.2	54%	2.7:1	1:1
**2**	1.6	61%	2.7:1	1:1
**3**	2.0	70%	2.7:1	1:1
**4**	2.4	79%	2.7:1	1:1
**5**	2.8	86%	2.7:1	1:1
**6**	3.2	84%	2.7:1	1:1

[a] General conditions: **17** (1.0 mmol), *rac*-**C1** (1 mol%), $(NH_2OH)_2•H_2SO_4$ (2.0 mmol), and AcOH (X mL) were dissolved in ${}^tBuOH$ (2.0 mL), and then H_2O_2 (5.0 equiv., 5.0 mmol, 567 uL, 30% wt. in H_2O) in 2 mL of ${}^tBuOH$ introduced with a syringe pump over 1 h under stirring at 0 °C; after stirring for an additional 0.5 h, the solution was quenched with Na_2SO_3 and then basified with Na_2CO_3 powder for 0.5 h at 0 °C; [b] yield, site selectivity and the ratio of *E/Z* determined by ^1H NMR of crude reaction mixture.

Table S-6. Effect of the amount of AcOH on oximation of **30**[a].

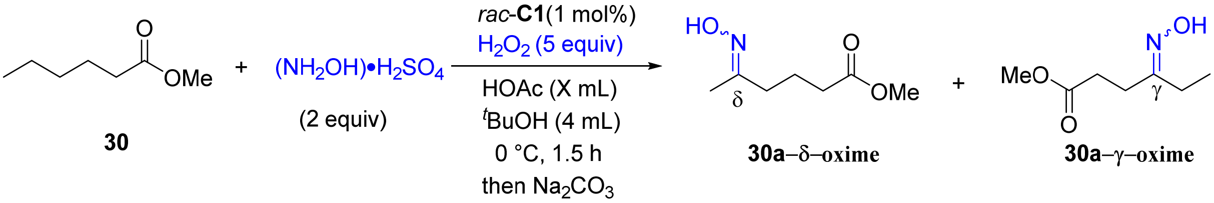

**Entry**	AcOH (X mL)	Isolate yield of **30a** (%)[b]	**30a-δ-oxime**/ **30a-γ-oxime**[b]	*E/Z* of **30a-δ-oxime**[b]	*E/Z* of **30a-γ-oxime**[b]
**1**	1.2	32%	5:1	3:1	1:1
**2**	1.6	39%	5:1	3:1	1:1
**3**	2.0	42%	5:1	3:1	1:1

**4**	2.4	53%	5:1	3:1	1:1
**5**	2.8	63%	5:1	3:1	1:1
**6**	3.2	64%	5:1	3:1	1:1

[a] General conditions: **30** (1.0 mmol), *rac*-**C1** (1 mol%), $(NH_2OH)_2$•H_2SO_4 (2.0 mmol), and AcOH (X mL) were dissolved in ^{t}BuOH (2.0 mL), and then H_2O_2 (5.0 equiv., 5.0 mmol, 567 uL, 30% wt. in H_2O) in 2 mL of ^{t}BuOH introduced with a syringe pump over 1 h under stirring at 0 ºC; after stirring for an additional 0.5 h, the solution was quenched with Na_2SO_3 and then basified with Na_2CO_3 powder for 0.5 h at 0 ºC; [b] yield, site selectivity and the ratio of *E/Z* determined by ^{1}H NMR of crude reaction mixture.

Table S-7. Screening manganese catalysts for oximation of (*E*)-hex-2-enoate (**50**)[a]

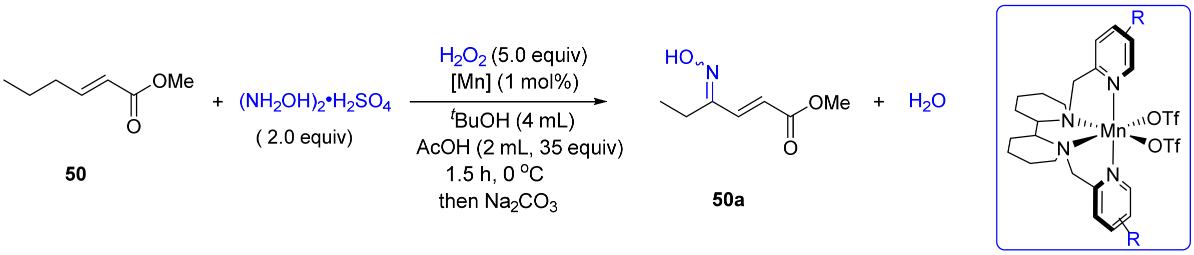

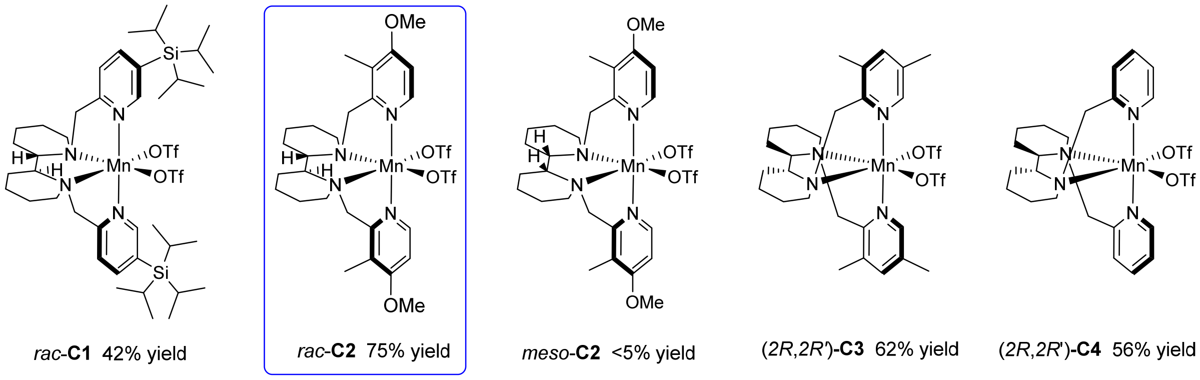

rac-**C1** 42% yield | *rac*-**C2** 75% yield | *meso*-**C2** <5% yield | (2*R*,2*R'*)-**C3** 62% yield | (2*R*,2*R'*)-**C4** 56% yield

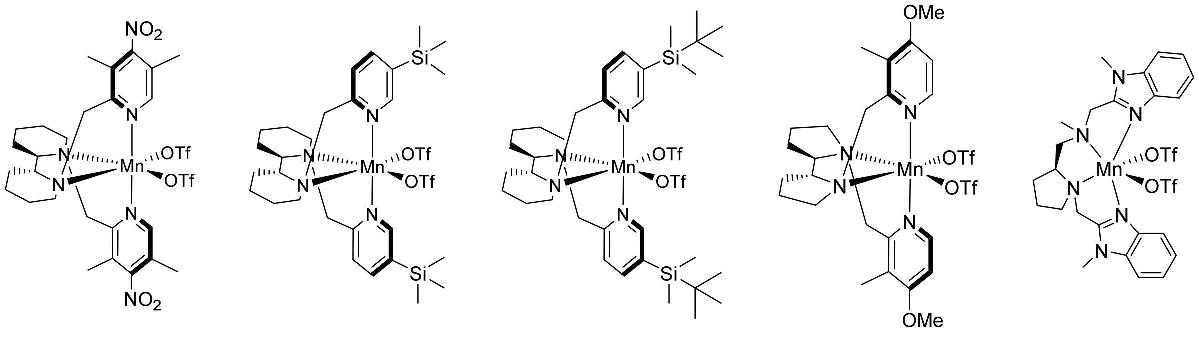

(2*R*,2*R'*)-**C5** 36% yield | (2*R*,2*R'*)-**C6** 64% yield | (2*R*,2*R'*)-**C7** 63% yield | (2*R*,2*R'*)-**C14** 35% yield | (*S*)- **C15** 10% yield

[a] General conditions: **50** (1 equiv., 1 mmol), $(NH_2OH)_2$•H_2SO_4 (2 equiv., 2 mmol), **[Mn]** (1 mol%), ^{t}BuOH (2 mL), AcOH (2 mL), and H_2O_2 (5 equiv., 5 mmol, 567 uL, 30% wt. in H_2O) in 2.0 mL of ^{t}BuOH introduced with a syringe pump over 1 h under stirring at 0 °C. After stirring for an additional 0.5 h, the solution was quenched with Na_2SO_3 and then basified with Na_2CO_3 powder for 0.5 h at 0 °C; isolated yields reported.

Table S-8. Optimization of conditions for oximation of (*E*)-hex-2-enoate (**50**)[a]

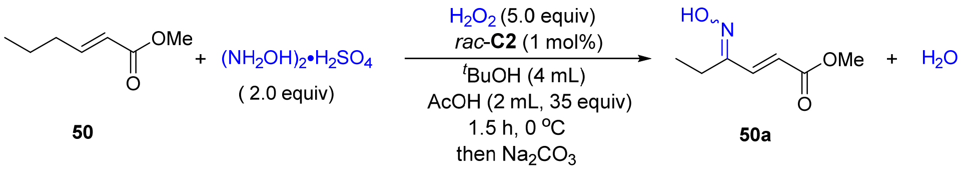

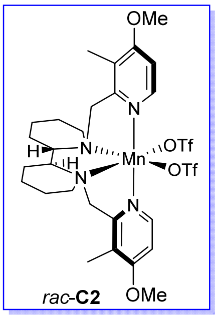

Entry	*rac*-**C2** (X mol %)	$(NH_2OH)_2$•H_2SO_4 (m equiv.)	H_2O_2 (Y equiv)	^{t}BuOH (Z ml)	AcOH (n mL)	Yield of[b] **50a** (%)
1	1.0	2.0	5.0	6	0	35
2	1.0	2.0	5.0	6	0.5	54
3	1.0	2.0	5.0	6	1.0	62
4	1.0	2.0	5.0	6	1.5	70
5	1.0	2.0	5.0	6	2.0	75
6	1.0	2.0	5.0	6	2.5	61
7	1.0	2.0	5.0	5	2.0	80
8	1.0	2.0	5.0	4	2.0	83
9	1.0	2.0	5.0	3	2.0	82
10	1.0	2.0	5.0	2	2.0	79
11	1.0	2.0	4.0	4	2.0	67
12	1.0	2.0	3.0	4	2.0	59
13	1.0	2.0	2.0	4	2.0	56
14	1.0	2.0	1.0	4	2.0	30
15	1.0	1.0	5.0	4	2.0	30
16	1.0	1.5	5.0	4	2.0	56
17	1.0	3.0	5.0	4	2.0	59
18	0.5	2.0	5.0	4	2.0	64
19	1.5	2.0	5.0	4	2.0	80
20	2	2.0	5.0	4	2.0	83

[a] General conditions: **50** (1 equiv., 1 mmol), $(NH_2OH)_2$•H_2SO_4 (2 equiv., 1 mmol), *rac*-**C2** (1 mol%), ^{t}BuOH ((Z-2) mL), AcOH (2 mL), and H_2O_2 (5 equiv., 5 mmol, 567 uL, 30% wt. in H_2O) in 2.0 mL of ^{t}BuOH introduced with a syringe pump over 1 h with under stirring at 0 °C; after stirring for an additional 0.5 h, the solution was quenched with Na_2SO_3 and then basified with Na_2CO_3 powder for 0.5 h at 0 °C; [b] isolated yields.

Table S-9. Formation of epoxides in *rac*-**C2** catalyzed allylic C–H oximation[a]

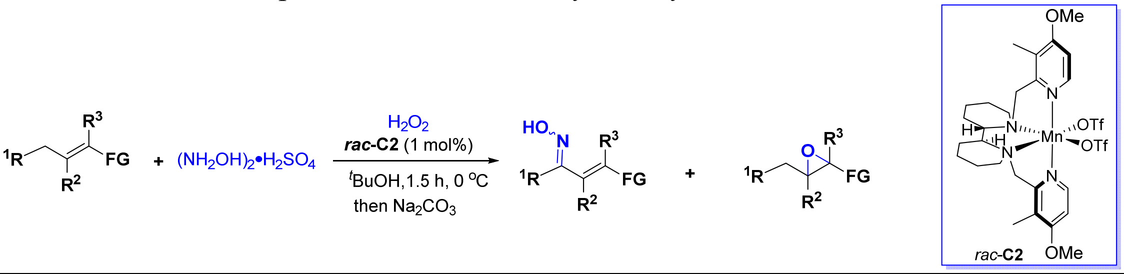

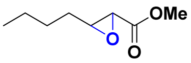

51b 8% yield

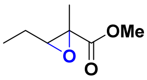

55b 20% yield

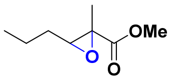

56b 29% yield

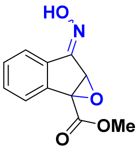

64a 28% yield

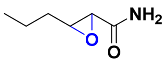

66b 34% yield

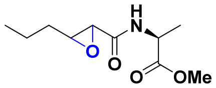

115b 34% yield
dr = 5:1 with (*R*,*R*)-**C2**

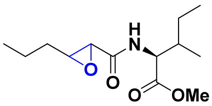

117b 28% yield
dr = 2:1 with (*R*,*R*)-**C2**

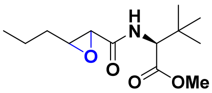

118b 34% yield, *dr* = 4:1 with (*R*, *R*)-**C2**
47% yield, *dr* > 99:1 with (*S*, *S*)-**C2**

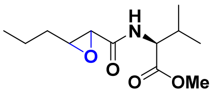

119b 8% yield
dr = 2:1 with (*R*,*R*)-**C2**

[a] General conditions: alkene (1 equiv., 1 mmol), $(NH_2OH)_2 \cdot H_2SO_4$ (2 equiv., 1 mmol), *rac*-**C2** (1 mol%), ^{t}BuOH (2 mL), AcOH (2 mL), H_2O_2 (5 equiv., 5 mmol, 567 uL, 30% wt. in H_2O) in 2.0 mL of ^{t}BuOH introduced with a syringe pump over 1 h under stirring at 0 °C. After stirring for an additional 0.5 h, the solution was quenched with Na_2SO_3 and then basified with Na_2CO_3 powder for 0.5 h at 0 °C; isolated yields. Only those showing yields >5% are reported.

Table S-10. Screening manganese catalysts for oximation of ethylbenzene[a]

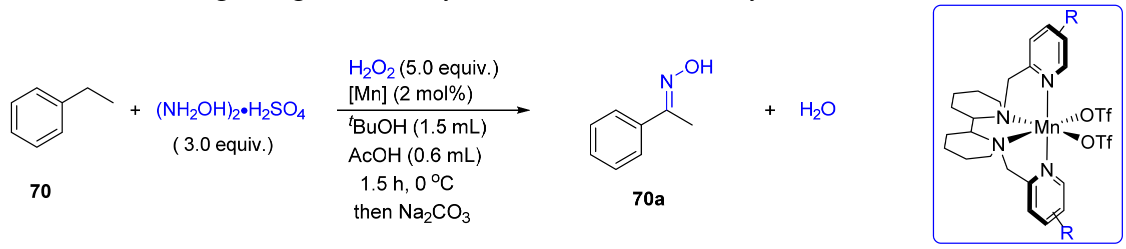

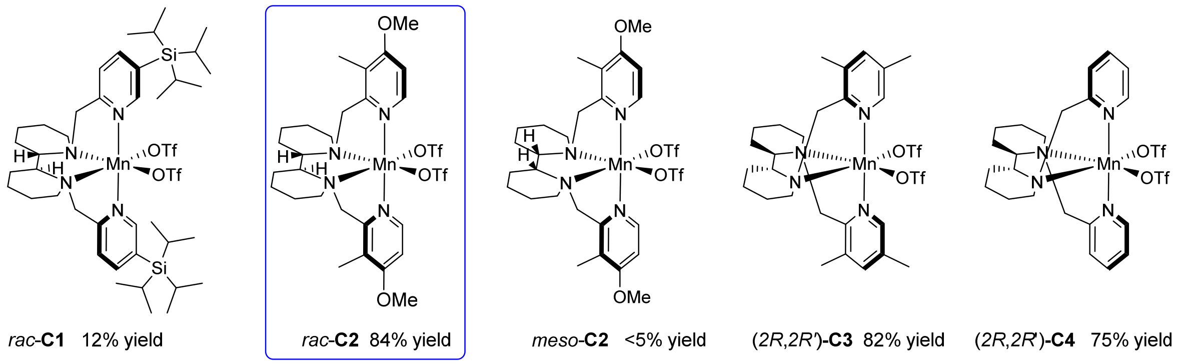

rac-**C1** 12% yield | *rac*-**C2** 84% yield | *meso*-**C2** <5% yield | (2*R*,2*R'*)-**C3** 82% yield | (2*R*,2*R'*)-**C4** 75% yield

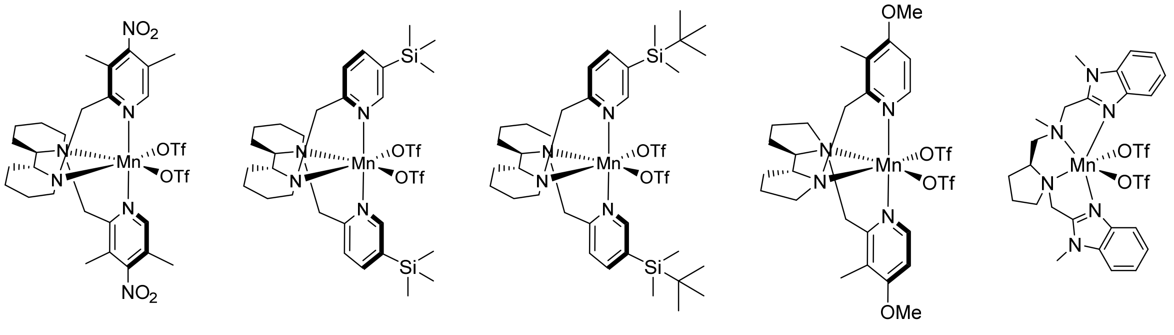

(2*R*,2*R'*)-**C5** 64% yield | (2*R*,2*R'*)-**C6** 44% yield | (2*R*,2*R'*)-**C6** 24% yield | (2*R*,2*R'*)-**C14** 65% yield | (*S*)-**C15** 8% yield

[a] General conditions: **70** (1 equiv., 0.5 mmol), $(NH_2OH)_2.H_2SO_4$ (3 equiv., 1 mmol), [Mn] (2 mol%), ^{t}BuOH (1.0 mL), AcOH (0.6 mL), and H_2O_2 (5 equiv., 2.5 mmol, 284 uL, 30% wt. in H_2O) in 0.5 mL of ^{t}BuOH introduced with a syringe pump over 1 h under stirring at 0 °C. After stirring for an additional 0.5 h, the solution was quenched with Na_2SO_3 and then basified with Na_2CO_3 powder for 0.5 h at 50 °C; yield determined by ^{1}H NMR of crude reaction mixture.

Table S-11. Optimization of conditions for oximation of ethylbenzene[a]

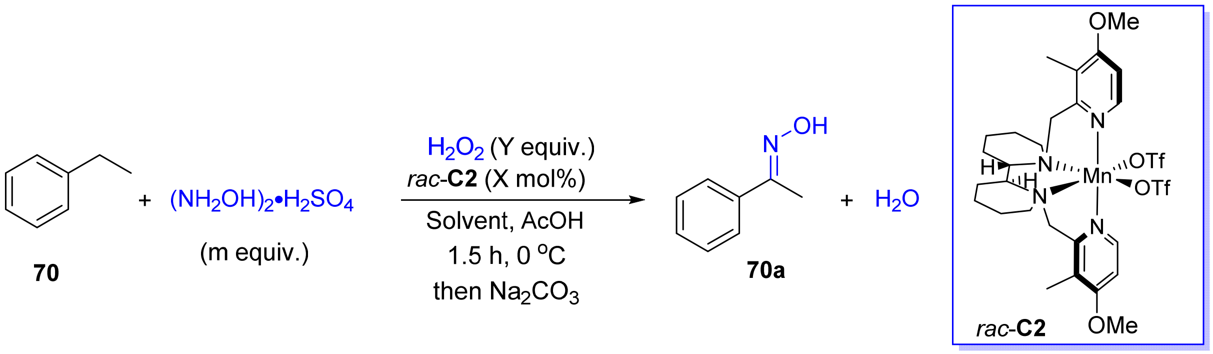

Entry	*rac*-**C2** (X mol %)	$(NH_2OH)_2$•H_2SO_4 (m equiv.)	H_2O_2 (Y equiv)	Solvent (Z ml)	AcOH (n mL)	Yield of **70a** (%)
1	2.0	3.0	5.0	^{t}BuOH(1.5)	0.2	63
2	2.0	3.0	5.0	^{t}BuOH(1.5)	0.4	67
3	2.0	3.0	5.0	^{t}BuOH(1.5)	0.6	84
4	2.0	3.0	5.0	^{t}BuOH(1.5)	0.5	81
5	2.0	3.0	5.0	^{t}BuOH(1.5)	1.0	82
6	2.0	3.0	5.0	^{t}BuOH(1.0)	0.5	51
7	2.0	3.0	5.0	^{t}BuOH(2.0)	0.5	77
8	2.0	3.0	5.0	^{t}BuOH(3.0)	0.5	67
9	2.0	3.0	5.0	MeOH(1.5)	0.5	/
10	2.0	3.0	5.0	CF_3CH_2OH(1.5)	0.5	/
11	2.0	3.0	4.0	CH_3CN(1.5)	0.5	65
12	2.0	3.0	6.0	^{t}BuOH(1.5)	0.5	78
13	2.0	1.0	5.0	^{t}BuOH(1.5)	0.5	25
14	2.0	2.0	5.0	^{t}BuOH(1.5)	0.5	69
15	2.0	4.0	5.0	^{t}BuOH(1.5)	0.5	79
16	2.0	5.0	5.0	^{t}BuOH(1.5)	0.5	26
17	1.0	3.0	5.0	^{t}BuOH(1.5)	0.5	76
18	4.0	3.0	5.0	^{t}BuOH(1.5)	0.5	69

[a] General conditions: **70** (1 equiv., 0.5 mmol), $(NH_2OH)_2$•H_2SO_4 (m equiv.), *rac*-**C2** (X mol%), solvent (1 mL), AcOH (n mL), and H_2O_2 (Y equiv., 30% wt. in H_2O) in 0.5 mL of ^{t}BuOH introduced with a syringe pump over 1 h under stirring at 0 °C; after stirring for an additional 0.5 h, the solution was quenched with Na_2SO_3 and then basified with Na_2CO_3 powder for 0.5 h at 50 °C; yield determined by ^{1}H NMR of crude reaction mixture.

Table S-12. Effect of electron-withdrawing group on the selectivity of oximation of alkyl esters [a]

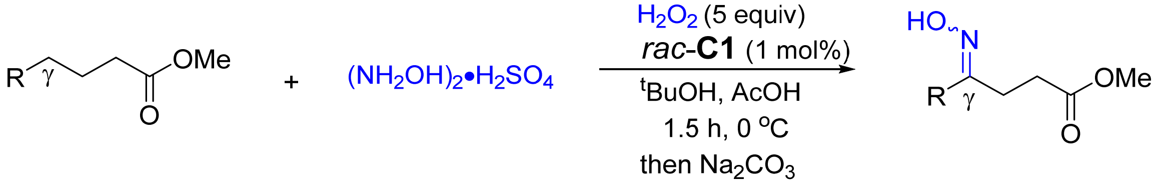

R=Me, single oxime product: γ;
R=Et, two oxime products: γ, δ
R=*n*-Pr, three oxime products: γ, δ, ε;
R=*n*-Bu, four oxime products: γ, δ, ε, ζ
R=*n*-Am, five oxime products: γ, δ, ε, ζ, η;
R=*n*-Hex, six oxime products: γ, δ, ε, ζ, η, θ

Entry	Substrate	Product	Yield of product
1	**27**	**27a-γ**	48%
2	**30**	**30a-(γ+δ)**	63% (γ/δ = 1:5)
3	**44**	**44a-(γ+δ+ε)**	63% (γ/δ/ε = 0.3:2:5)
4	**123**	**123a-(γ+δ+ε+ζ)**	ζ : 35%, (γ+δ+ε): 34%
5	**124**	**124a-(γ+δ+ε+ζ+η)**	η: 20%, (γ+δ+ε+ζ): 40%
6	**125**	**125a-(γ+δ+ε+ζ+η+θ)**	θ: 21%, (γ+δ+ε+ζ+η): 43%

[a] General conditions: alkyl ester (1 equiv., 1.0 mmol), $(NH_2OH)_2$•H_2SO_4 (2 equiv. 2.0 mmol), *rac*-**C1** (10.1 mg, 1 mol%), ^{t}BuOH (2 mL), AcOH (2.8 mL), and H_2O_2 (5 equiv., 5.0 mmol, 567 uL, 30% wt. in H_2O) in 2 mL of ^{t}BuOH introduced with a syringe pump over 1 h under stirring at 0 °C. After stirring for an additional 0.5 h, the solution was quenched with Na_2SO_3 and then basified with Na_2CO_3 powder for 0.5 h at 0 °C; isolated yields. Site selectivity was determined by integration of ^{1}H and ^{13}C NMR spectra of isolated mixture products. In all cases, the products refer to mono-oximated compounds.

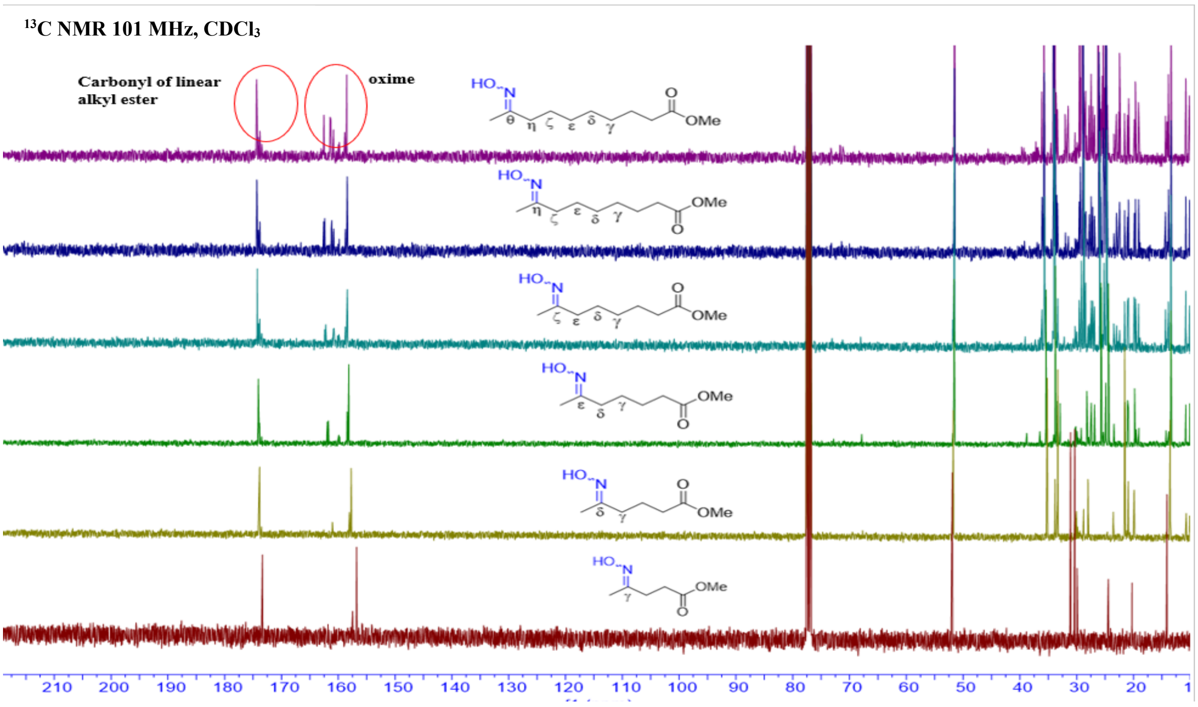

^{13}C NMR 101 MHz, $CDCl_3$

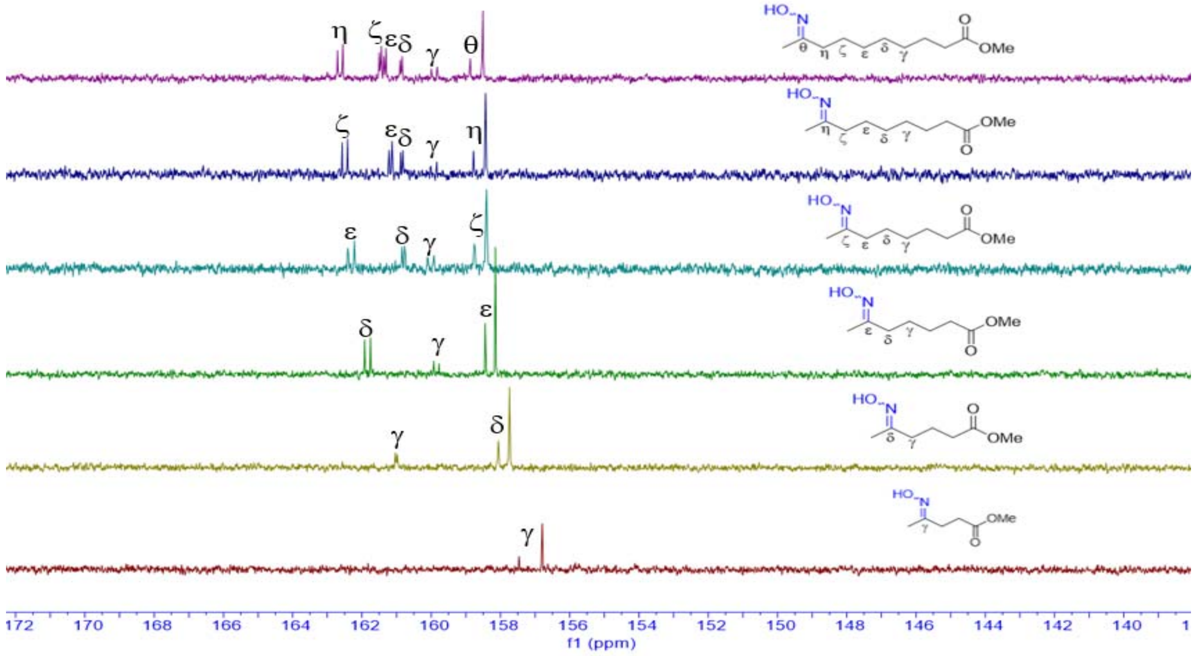

Figure S-7. ^{13}C NMR spectra of products from the oxidation of alkyl esters.

Table S-13. Catalyst-controlled site selectivity of the oximation of sclareolide[a]

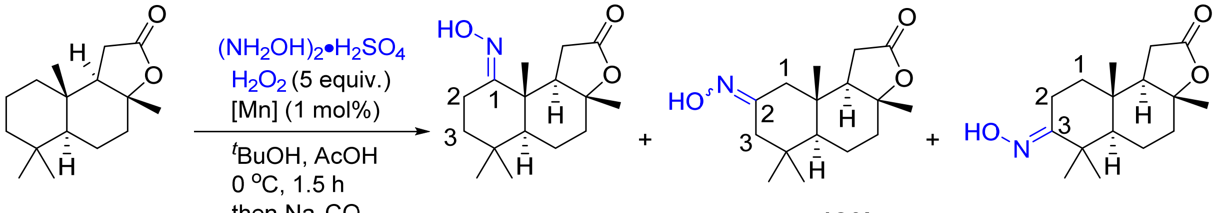

120 (***E***)**-120a** (***E/Z***)**-120b** (***E***)**-120c**

Entry	Catalyst	*E*-120a (%)	*E*-120b (%)	*Z*-120b (%)	*E*-120c (%)	120a/120b /120c	Remaining 120 (%)
1	*Rac*-**C1**	6	22	24	7	1:7.7:1.2	35
2	(*R*,*R*)-**C1**	6	18	24	6	1:7:1	42
3	(*S*,*S*)-**C1**	4	28	28	7	1:14:1.8	26
4	(*R*,*R*)-**C2**	7	22	19	7	1:5.9:1	39
5	(*S*,*S*)-**C2**	10	31	26	9	1:5.7:0.9	17

[a] General conditions: **120** (1 equiv., 1.0 mmol), $(NH_2OH)_2$•H_2SO_4 (2 equiv. 2.0 mmol), [Mn] (1 mol%), ^{t}BuOH (2 mL), AcOH (2.8 mL), and H_2O_2 (5 equiv., 5.0 mmol, 567 uL, 30% wt. in H_2O) in 2 mL of ^{t}BuOH introduced with a syringe pump over 1 h under stirring at 0 °C; after stirring for an additional 0.5 h, the solution was quenched with Na_2SO_3 and then basified with Na_2CO_3 powder for 0.5 h at 0 °C; isolated yield. Site selectivity was calculated based on the quantity of isolated yields. The structures of ***E*-120a** and ***Z*-120c** were assigned based on X-ray diffraction.

Table S-14. Optimization conditions for oximation of methyl dehydroabietate[a]

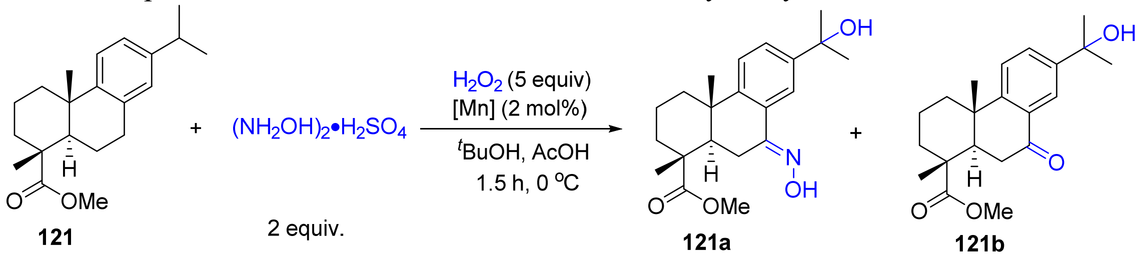

Entry	catalyst	Basification (time, h)	**121a** (%)	**121b** (%)	Remaining (%)
1	(*R*,*R*)-**C2**	0.5 h	19	26	29
2	(*R*,*R*)-**C2**	no basification, 20h	18	29	29
3	(*R*,*R*)-**C2**	20 h	42	9	30
4	(S,*S*)-**C2**	20 h	41	15	12

[a] General conditions: **121** (1 equiv., 0.5 mmol), $(NH_2OH)_2$•H_2SO_4 (3 equiv. 1.5 mmol), [Mn] (1 mol%), ᵗBuOH (2 mL), AcOH (0.6 mL), and H_2O_2 (5 equiv., 2.5 mmol, 284 uL, 30% wt. in H_2O) in 2 mL of ᵗBuOH introduced with a syringe pump over 1 h under stirring at 0 °C; after stirring for an additional 0.5 h, the solution was quenched with Na_2SO_3 and then basified with Na_2CO_3 powder for 0.5 h at 50 °C; isolated yield.

Table S-15. Catalyst-controlled site-selectivity of the oximation of (+)-artemisinin[a]

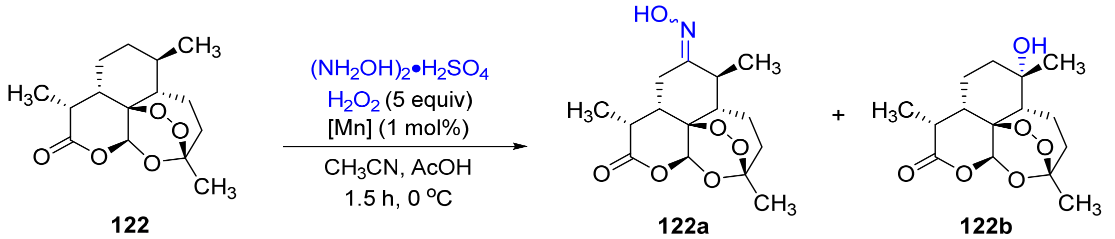

Entry	Catalyst	Solvent	No. of recycling **122**	Yield of **122a** (mg, %)	Yield of **122b**	Remaining **122** (mg, %)
1	(*R*,*R*)-**C1**	ᵗBuOH	0	17 mg, 11%	trace	121.6 mg, 86%
2	(*S*,*S*)-**C1**	ᵗBuOH	0	Mixture products		/
3	(*S*,*S*)-**C1**	CH_3CN	0	Mixture products		/
4	(*R*,*R*)-**C2**	ᵗBuOH	0	3 mg, 2%	trace	136.0 mg, 96%
5[b]	(*R*,*R*)-**C1**	CH_3CN	0	35.6 mg, 23%	trace	101.2 mg, 72%
			1	31.2 mg, 20%	trace	65.3 mg, 46%
			2	20.2 mg, 13%	trace	37.2 mg, 26%
			overall	87.0 mg, 56%	trace	37.2 mg, 26%

[a] General conditions: **122** (1 equiv., 0.5 mmol), $(NH_2OH)_2$•H_2SO_4 (2 equiv. 1.0 mmol), [Mn] (1 mol%), ᵗBuOH (1 mL), AcOH (1.4 mL), and H_2O_2 (5 equiv., 2.5 mmol, 284 uL, 30% wt. in H_2O) in 1 mL of ᵗBuOH introduced with a syringe pump over 1 h under stirring for at 0 °C; after stirring for an additional 0.5 h, the solution was quenched with Na_2SO_3 and then basified with Na_2CO_3 powder for 0.5 h at 0 °C; isolated yield. [b] Starting material was recycled twice.

6. Mechanistic studies

6.1 Time course of the *rac*-C1 catalyzed oximation of cyclododecane

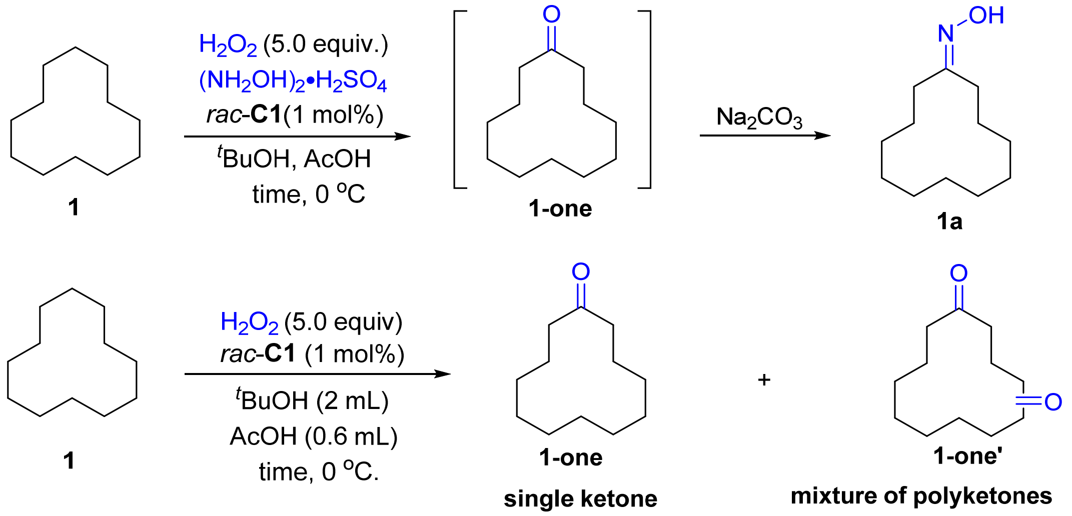

With $(NH_2OH)_2$•H_2SO_4: *rac*-C1 (5.1 mg, 1.0 mol%), hydroxylamine sulfate (164.2 mg, 1.0 mmol. 2.0 equiv.), cyclododecane (84.2 mg, 0.5 mmol, 1.0 equiv.), AcOH (0.6 mL) and ^{t}BuOH (1.0 mL) were added to a reaction tube. The mixture was cooled down to 0 °C in a cryogenic bath, and then H_2O_2 (2.5 mmol, 5.0 equiv., 284 uL, 30% wt. in H_2O, dropping rate: 0.02 mL/min) in 1 mL of ^{t}BuOH was added dropwise with a syringe pump over a certain period of time under stirring at 0 °C without nitrogen protection. After stirring for an additional 0.5 h, the reaction solution was quenched with Na_2SO_3 solid. And then the mixture was basified with Na_2CO_3 for 30 min at room temperature, filtered and washed with DCM (2.0 mL). The yield was determined by ^{1}H NMR of the crude reaction mixture. The same reaction was repeated but stopped after a different period of time (Yield variation with time is shown in Table **S-16**).

Without $(NH_2OH)_2$•H_2SO_4: The procedure was the same as the above expect without $(NH_2OH)_2$·H_2SO_4. Yield determined by ^{1}H and ^{13}C NMR analysis of the crude reaction mixture with 1,3,5-trimethoxybenzene as internal standard (Yield variation with is shown in Table **S-16**).

The data obatained are also graphically shown in Figure **S-8**.

Table S-16. Oxidative of cyclododecane **1** with or without $(NH_2OH)_2{\cdot}H_2SO_4$[a]

Entry	Amount of $(NH_3OH)_2{\bullet}SO_4$	Time (minus)	Conversion (%)	Yield of **1a** (%)	Yield of **1-one** (%)	Yield of **1-one'** (%)[b]
1	0 equiv	10	18	/	18	0
2	0 equiv.	20	33	/	33	0
3	0 equiv.	30	57	/	52	5
4	0 equiv.	40	>99	/	75	22
5	0 equiv.	50	>99	/	50	36
6	0 equiv.	60	>99	/	54	45
7	0 equiv.	70	>99	/	46	54
8	2 equiv.	10	5	5	/	/
9	2 equiv.	20	31	31	/	/
10	2 equiv.	30	45	45	/	/
11	2 equiv.	40	54	54	/	/
12	2 equiv.	50	65	65	/	/
13	2 equiv.	60	74	74	/	/
14	2 equiv.	70	87	87	/	/

[a] General conditions: **1** (1 equiv., 0.5 mmol), $(NH_2OH)_2{\bullet}H_2SO_4$ (shown in table), *rac*-**C1** (1 mol%), ^{t}BuOH (1 mL), AcOH (0.6 mL), and H_2O_2 (5 equiv., 2.5 mmol, 284 uL, 30% wt. in H_2O, dropping rate: 0.02 mL/min) in 1 mLof ^{t}BuOH introduced with a syringe pump over the time indicated under stirring at 0 °C; after stirring for an additional 0.5 h, the solution was quenched with Na_2SO_3 and then basified with Na_2CO_3 powder for 0.5 h at 0 °C; yield determined by ^{1}H NMR of crude reaction mixture. [b] **1-one'** refers to polyketones with yields calculated from the yield of **1-one** and remaining **1**.

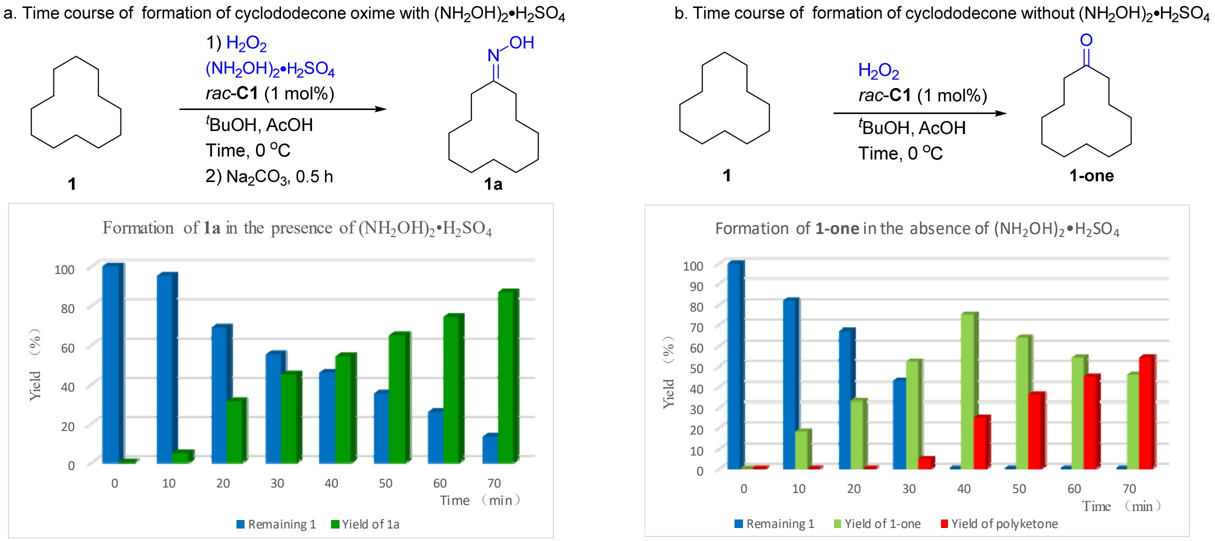

Figure S-8. Time course of oxidation of cyclododecane **1** with or without $(NH_2OH)_2{\bullet}H_2SO_4$

6.2 Examination of the possibility of formation of $(\mathbf{1a})_2{\bullet}H_2SO_4$ salt from cyclododecanone in the oximation of cyclododecane with $(NH_2OH)_2{\bullet}H_2SO_4$

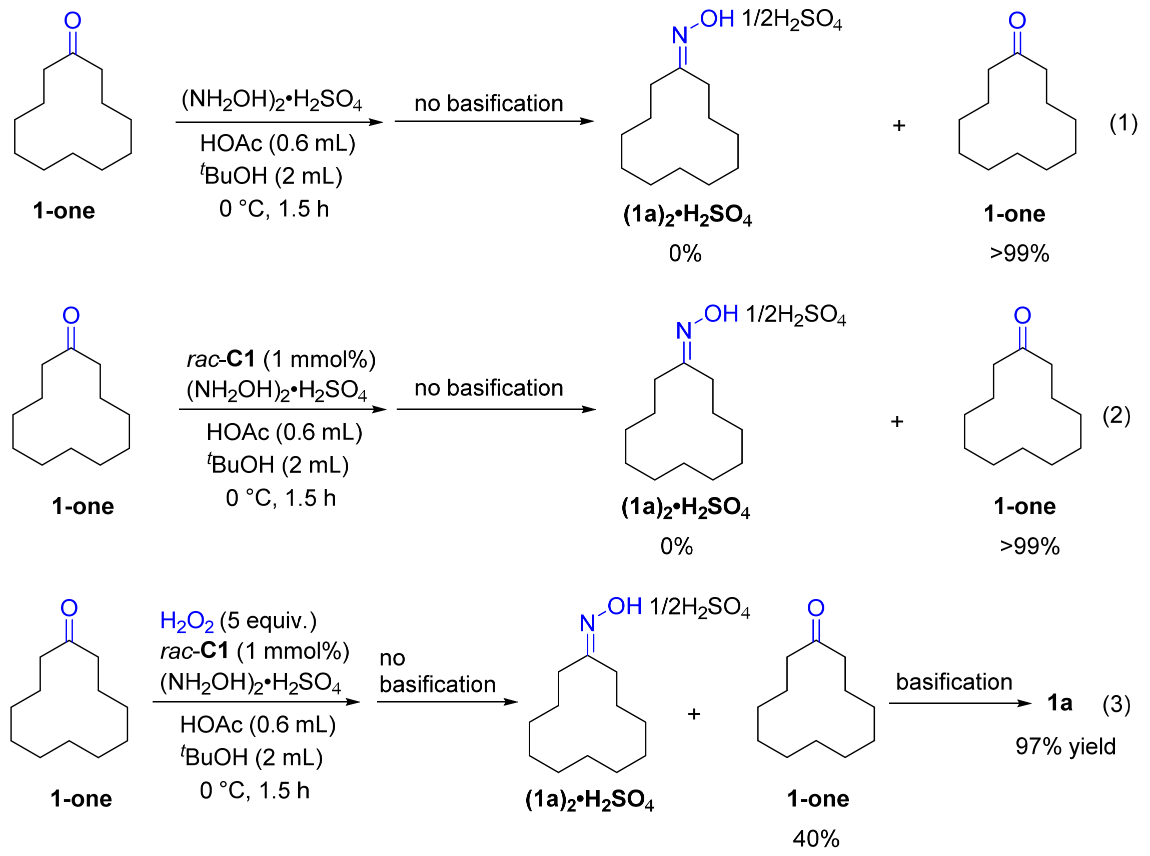

Eq. 1: In a reaction tube, cyclododecanone (91.2 mg, 0.5 mmol) and hydroxylamine sulfate (164.2 mg, 1.0 mmol. 2.0 equiv.) were added to a mixed solvent of AcOH (0.6 mL) and ^{t}BuOH (2 mL). After stirring for 1.5 hours, the reaction mixture was filtered and washed with DCM. The resulting solution was taken and the amount of **1-one** was analyzed by GC; however, **1a** and $(\mathbf{1a})_2{\bullet}H_2SO_4$ could not be analyzed by GC due to their high boiling points, which exceed the detection limit of the method. (**1-one**, 0.5 mmol, 99% GC yield; GC trace and calibration curve are shown **Fig S10-S11**; $y = 0.4807{*}x{+}0.0526$, $R^2{=}0.9992$, $x = A_{\mathbf{Cyclododecanone}} / A_{\mathbf{1,4\text{-}Dibromobenzene}}$; $y = n_{\mathbf{Cyclododecanone}} / n_{\mathbf{1,4\text{-}Dibromobenzene}}$). Then, the collected solution was concentrated under reduced pressure to produce the crude product. The crude mixture was measured by ^{1}H and ^{13}C NMR. No **1a** or $(\mathbf{1a})_2{\bullet}H_2SO_4$ salt was observed under this condition (**Fig S-9**).

Eq. 2: The reaction condition was the same as in **Eq. 1** but with *rac*-**C1** (5.1 mg, 1mol%) added. The amount of **1-one** was analyzed by GC (**1-one**, 0.5 mmol, 99% GC yield; GC trace and calibration curve are shown **Fig S12**; $y = 0.4807{*}x{+}0.0526$, $R^2{=}0.9992$, $x = A_{\mathbf{Cyclododecanone}} / A_{\mathbf{1,4\text{-}}}$

Dibromobenzene; y = $n_{Cyclododecanone}$ /$n_{1,4\text{-}Dibromobenzene}$). Likewise, no **1a** or **(1a)$_2$•H_2SO_4** salt was observed under this condition as analyzed by ^{1}H and ^{13}C NMR.

Eq. 3: The reaction condition was the same as in **Eq. 1** but with *rac*-**C1** (5.1 mg, 1mol%) and H_2O_2 (2.5 mmol, 5.0 equiv., 284 uL, 30% wt. in H_2O, dropping rate: 0.02 mL/min) added. The amount of **1-one** was analyzed by GC (**1-one**, 0.2 mmol, 40% GC yield; GC trace and calibration curve are shown **Fig S13**; $y = 0.4807*x+0.0526$, $R^2=0.9992$, x = $A_{cyclododecanone}$ / $A_{1,4\text{-}dibromobenzene}$; y = $n_{cyclododecanone}$ / $n_{1,4\text{-}dibromobenzene}$), and no **1a** or **(1a)$_2$•H_2SO_4** salt was observed under this condition analyzed by ^{1}H and ^{13}C NMR. Repeating the reaction again, after quenching with Na_2SO_3, basification with Na_2CO_3, and removal of the solvent, afforded the product cyclododecanone oxime as white solid (**96 mg, 97%** isolated yield).

*These results indicate that cyclododecanone (**1-one**) does not undergo conversion to **1a** or **(1a)$_2$•H_2SO_4** under the conditions of **Eq. 1** and **Eq. 2** (see Section 6.3 for comparison). However, under **Eq. 3** conditions, which are similar to those employed for the oxidative oximation of **1**, GC analysis indicates that only 40% of **1-one** remains, implying a conversion of ca 60% to oxime or its sulfate salt.* Considering that the boiling point of **1a** exceeds the detection limit of GC method, we turned attention to cyclohexanone **3-one**, which is of lower boiling point than **1-one**, to examine the possibility of oxime and its salt formation *via* GC (Section 6.3).

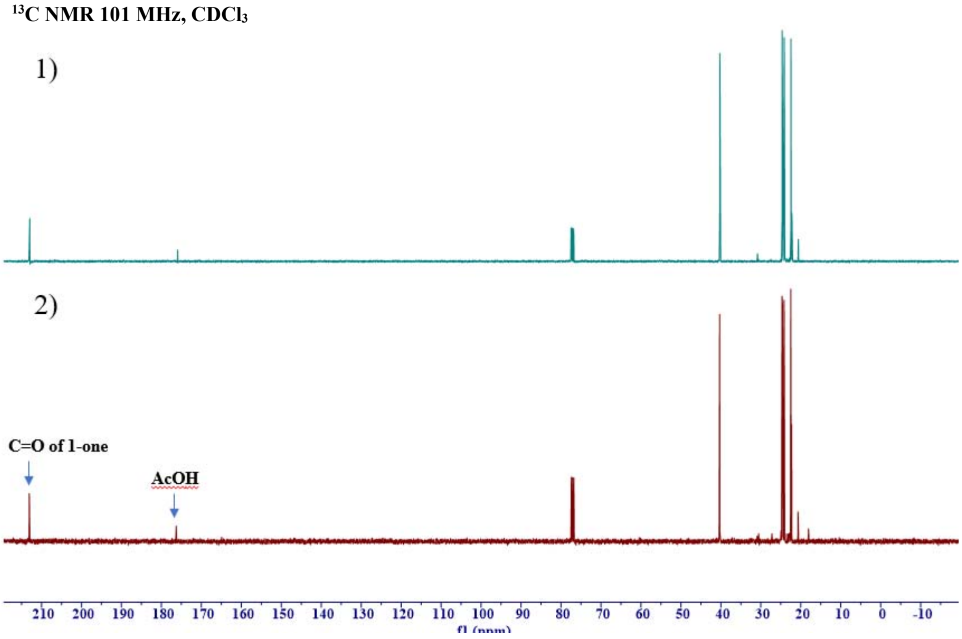

Figure S-9. ^{13}C NMR of the mixture of **1-one** and $(NH_2OH)_2$•H_2SO_4 (The numbers refer to the equations 1 and 2 above).

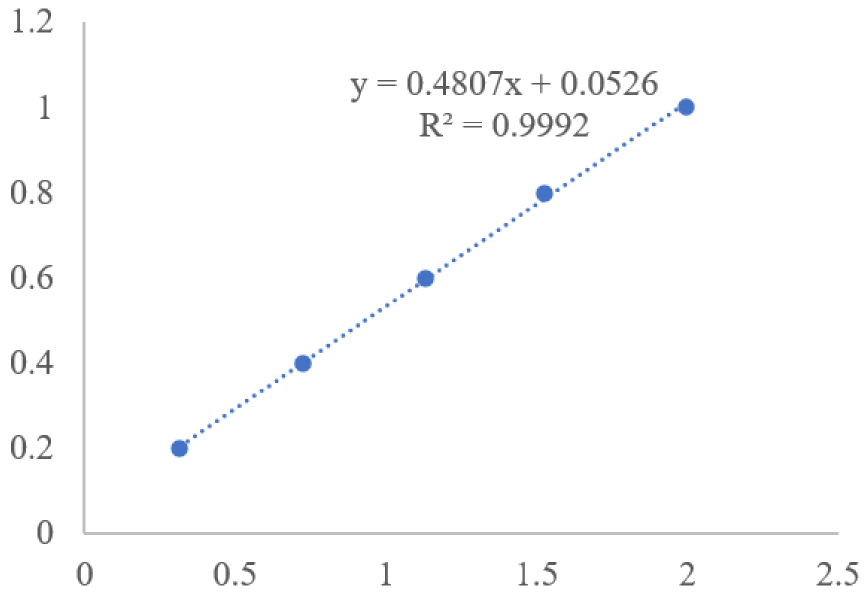

Figure S-10. GC calibration curve of **1-one** (commercial sample) and 1,4-dibromobenzene (internal standard).

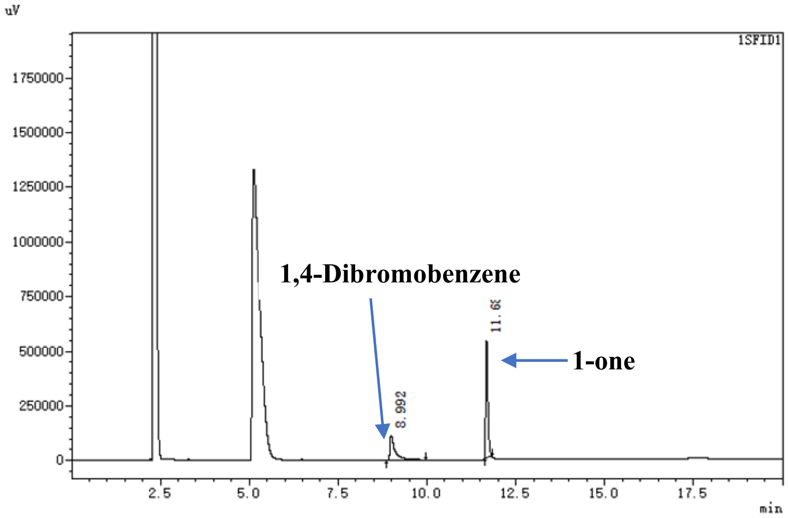

Peak#	Ret. Time	Area	Height	Concentration	Concentration Unit	Mark
1	8.992	1114531	112928	33.639		M
2	11.680	2198699	531212	66.361		M
Toal		3313230	437795	100.000		

Figure S-11. GC trace of the mixture resulting from **6.2 Eq. 1**; GC parameters: injection temperature = 250 °C, column temperature = 80 °C, detector temperature = 250 °C. The carrier gas was N_2.

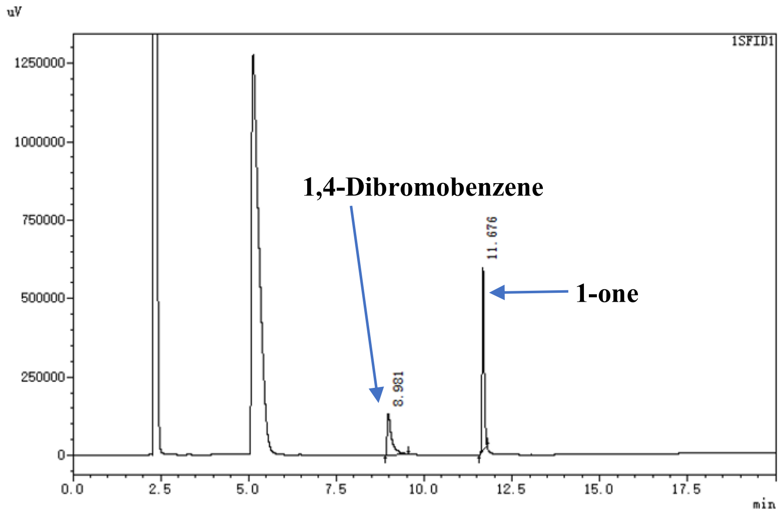

Peak#	Ret. Time	Area	Height	Concentration	Concentration Unit	Mark
1	8.981	1159241	130665	33.029		M
2	11.676	2350510	578735	66.971		M
Toal		3509751	709400	100.000		

Figure S-12. GC trace of the mixture resulting from **6.2 Eq. 2**; GC parameters: injection temperature = 250 ºC, column temperature = 80 ºC, detector temperature = 250 ºC. The carrier gas was N_2.

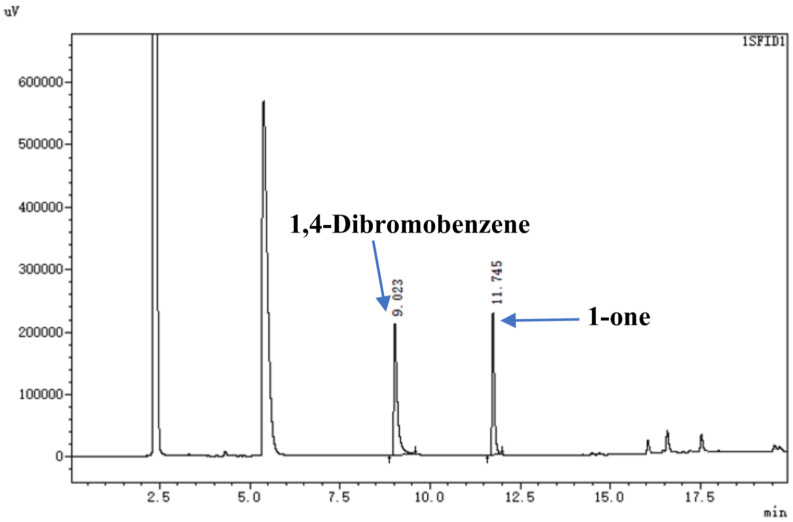

Peak#	Ret. Time	Area	Height	Concentration	Concentration Unit	Mark
1	9.023	1368866	210694	58.150		M
2	11.745	985154	227101	41.850		M
Toal		2354020	437795	100.000		

Figure S-13. GC trace of the mixture resulting from **6.2 Eq. 3**; GC parameters: injection temperature = 250 ºC, column temperature = 80 ºC, detector temperature = 250 ºC. The carrier gas was N_2.

6.3 Examination of the possibility of formation 3a or $(3a)_2$•H_2SO_4 salt in the oximation of cyclohexane with $(NH_2OH)_2$•H_2SO_4

Table S-17. Oximation of **3-one** with $(NH_2OH)_2$•H_2SO_4 under various conditions[a]

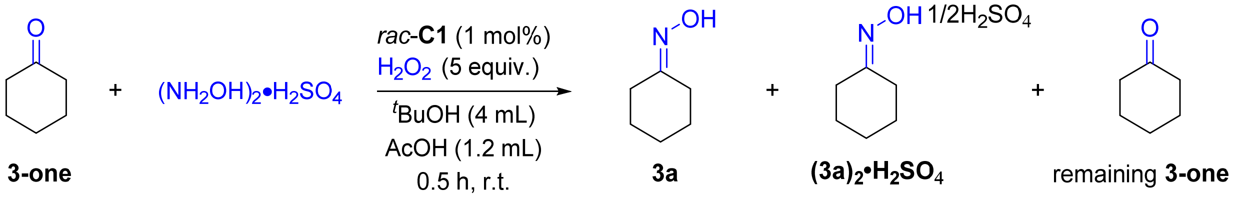

Entry	Deviation from general conditions	3a (%)	$(3a)_2$•H_2SO_4 (%)[b]	Remaining 3-one (%)
1	Without *rac*-**C1**, H_2O_2 and AcOH	7	0	93
2	Without *rac*-**C1** and H_2O_2	11	0	89
3	Without *rac*-**C1** and AcOH	21	2	77
4	Without H_2O_2 and AcOH	10	0	90
5	Without *rac*-**C1**	32	3	65
6	Without H_2O_2	14	0	86
7	Without AcOH	47	21	32
8	General conditions	46 (>99)[c]	32	22
9	General conditions[d]	27	0	73
10	General conditions[e]	72	4	24
12	General conditions[f]	70	17	13
13	General conditions[g]	61 (>99)[c]	26	13
14	General conditions[d,h]	34 (>99)[c]	25	41
15	H_2O (0.5 mL) instead of H_2O_2[d,h]	35	trace	64
16	General conditions[d,i]	8	trace	91
17	Without H_2O_2[d,i]	<1	trace	>99

[a] General conditions: **3-one** (1 equiv., 1 mmol), $(NH_2OH)_2$•H_2SO_4 (2 equiv. 2.0 mmol), ***rac*-1** (1 mol%), ^{t}BuOH (4 mL), AcOH (1.2 mL), and H_2O_2 (5 equiv., 10 mmol, 568 uL, 30% wt. in H_2O, added all at once, not in batches), r.t., 0.5 h; yield determined by GC of crude reaction mixture. [b] Calculated from the yields of **3a** and **remaining 3-one**, assuming the rest to be the sulfate salt. [c] Basified with Na_2CO_3 powder for 0.5 h, and then determined by GC of crude reaction mixture. [d] Determined by GC, 3 min after the reaction. [e] Determined by GC, 10 min after the reaction. [f] Determined by GC, 30 min after the reaction. [g] Determined by GC, 60 min after the reaction. [h] $Sc(OTf)_3$ (1 mol%) instead of *rac*-**C1** (1 mol%). [i] $Mn(OAc)_3$ (1 mol%) instead of *rac*-**C1** (1 mol%).

Entry 8: In a reaction tube, **3-one** (98.2 mg, 1 mmol), *rac*-**C1** (10.2 mg, 1 mol%) and hydroxylamine sulfate (328.4 mg, 2.0 mmol. 2.0 equiv.) were added to a mixed solvent of AcOH (1.2 mL) and ^{t}BuOH (4 mL). Then, H_2O_2 (5 equiv., 10 mmol, 568 uL, 30% wt. in H_2O) was added to this tube under room temperature. After stirring for 0.5 h, the resulting solution was taken and the amount of **3a** and **remaining 3-one** were analyzed by GC (**3a**, 46% GC yield, **remaining 3-**

one, 22% GC yield; calibration curve of **3a**: y = 1.0137*x+0.0362, R^2=0.9997, $x = A_{\mathbf{3a}} / A_{\mathbf{1,4\text{-}Dibromobenzene}}$; $y = n_{\mathbf{3a}} / n_{\mathbf{1,4\text{-}Dibromobenzene}}$; calibration curve of **3-one**: y = 1.1047*x+0.0058, R^2=0.9996, $x = A_{\mathbf{3\text{-}one}} / A_{\mathbf{1,4\text{-}Dibromobenzene}}$; $y = n_{\mathbf{3\text{-}one}} / n_{\mathbf{1,4\text{-}Dibromobenzene}}$). However, **$(3a)_2$•$H_2SO_4$** could not be analyzed by GC due to its high boiling point, which exceeds the detection limit of the method. Instead, the amount of **$(3a)_2$•H_2SO_4** was calculated from that of **3a** and **remaining 3-one**, assuming the rest to be the sulfate. The validity of the approach can be seen from the yields after basification (entries 8, 13 and 14). The GC trace is shown **Fig S14**. Afterwards, the reaction mixture was quenched with Na_2SO_3 and basified with Na_2CO_3 powder for 0.5 h, and the amount of **3a** was analyzed by GC (>99% yield); GC trace is shown **Fig S15**. For the other entries in **Table S-17**, similar procedures were followed, but under different conditions.

These results show that 1) oxime is almost instantly formed in the oximation of cyclohexanone with hydroxylammonium sulfate, 2) the oximation is catalyzed by the Lewis acid Sc(OTf)₃ and presumably by high valent manganese species generated from the oxidation of the catalyst with H_2O_2, and 3) the oxime salt is not detectable on GC. Note NMR cannot be reliably used to analyze the oxime salt due to decomposition resulting from sample preparation.

The lower reactivity of **1-one**, compared with that of **3-one**, is partly due to, unlike **3-one**, its being solid, requiring more forcing conditions to react (see Section 6.2).

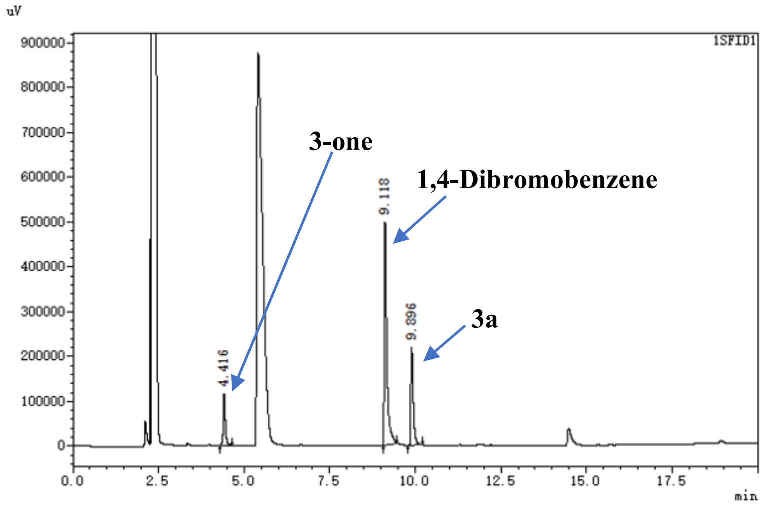

Peak#	Ret. Time	Area	Height	Concentration	Concentration Unit	Mark
1	4.416	496178	113725	12.080		M
2	9.118	2548253	497029	62042		M
3	9.896	1062842	217139	25.877		M
Toal		4107273	827893	100.000		

Figure S-14. GC trace of the mixture resulting from **Table S-17**, **entry 8** (no basification); GC parameters: injection temperature = 250 °C, column temperature = 80 °C, detector temperature = 250 °C. The carrier gas was N_2.

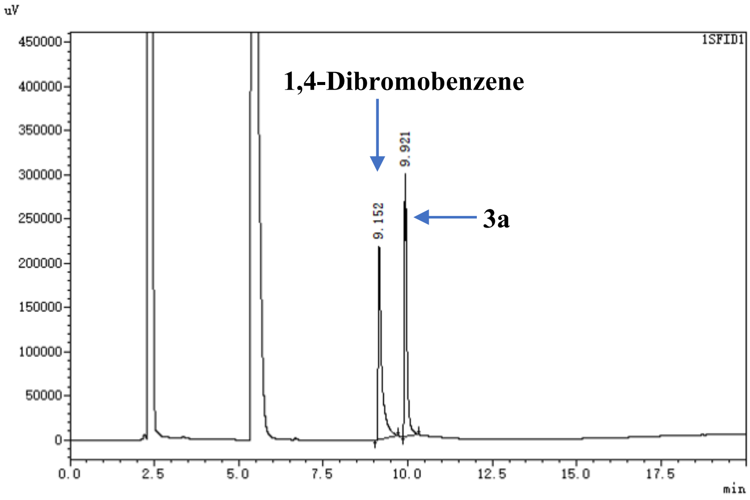

Peak#	Ret. Time	Area	Height	Concentration	Concentration Unit	Mark
1	9.152	1540582	218133	51.231		M
2	9.921	1466527	296115	48.769		M
Toal		3007109	514248	100.000		

Figure S-15. GC trace of the mixture resulting from **Table S-17**, **entry 8** (after basification with Na_2CO_3); GC parameters: injection temperature = 250 °C, column temperature = 80 °C, detector temperature = 250 °C. The carrier gas was N_2.

Table S-18. Investigation of the hydrolysis of **3a** under various conditions [a]

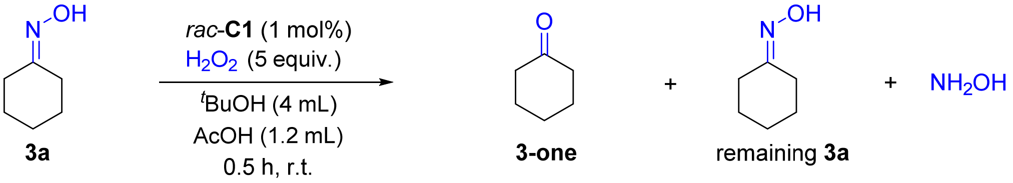

Entry	Deviation from general conditions	Remaining 3a (%)	3-one (%)
1	Without *rac*-**C1**, H_2O_2 and AcOH	100	0
2	Without *rac*-**C1** and H_2O_2,	100	0
3	Without H_2O_2 and AcOH	100	0
4	Without *rac*-**C1** and AcOH	99	1
5	Without H_2O_2,	100	0
6	Without *rac*-**C1**	97	3
7	Without AcOH	88	12
8	General conditions	52	46
9	General conditions [b]	58	39

10	General conditions [c]	57	40
12	General conditions [d]	98	2
13	General conditions [e]	55	45
14	General conditions [f]	50	49
15	General conditions [g]	54	45
16	General conditions [h]	56	44

[a] General conditions: **3a** (1 equiv., 1 mmol), ***rac*-1** (1 mol%), ^{t}BuOH (4 mL), AcOH (1.2 mL), and H_2O_2 (5 equiv., 10 mmol, 568 uL, 30% wt. in H_2O, added all at once, not in batches), r.t., 0.5 h; yield determined by GC of crude reaction mixture. [b] Continued stirring for another 1.5 h under general conditions; [c] Continued stirring for another 2.5 h under general conditions; [d] 0.5 mL H_2O instead of H_2O_2. [e] Determined by GC, 3 min after the reaction. [f] Determined by GC, 10 min after the reaction. [g] Determined by GC, 30 min after the reaction. [h] Determined by GC, 60 min after the reaction.

Entry 8: The reaction was carried out following the conditions described in **Table S17**, **entry 8,** but hydroxylamine sulfate was omitted and **3a** (113.2 mg, 1mmol) was used as the substrate instead of **3-one**. The conversion was monitored by GC analysis, tracking the formation of **3-one** and the consumption of **3a** (GC trace is shown Fig S15. **3-one**, 46%; **3a**, 52%). For the other entries, similar procedures were followed, but under the different conditions listed in the **Table S18**.

*The results indicate that 1) an equilibrium of the hydrolysis (water is omitted in the equation) is established almost immediately after subjecting the oxime **3a** to the reaction conditions, and 2) the hydrolysis is facilitated by both Bronsted and Lewis acid, with the later presumably generated by oxidation of the manganese to a higher oxidation state with H_2O_2.*

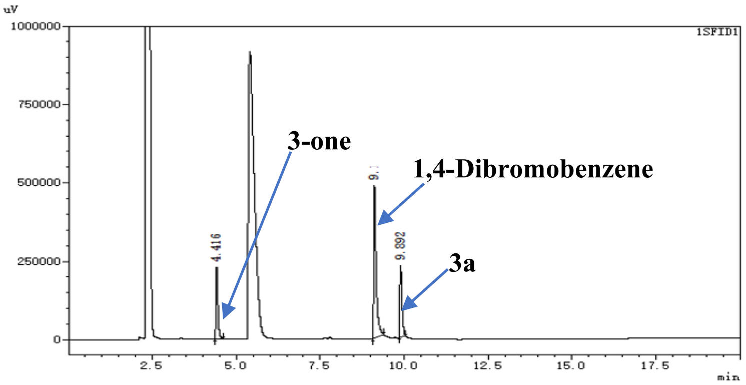

Peak#	Ret. Time	Area	Height	Concentration	Concentration Unit	Mark
1	4.416	895107	226692	20.556		M
2	9.117	2435168	485409	55.924		M
3	9.892	1024170	227983	23.520		M
Toal		4354444	940084	100.000		

Figure S-16. GC trace of the mixture resulting from **Table S-18**, **entry 8** (no basification); GC parameters: injection temperature = 250 °C, column temperature = 80 °C, detector temperature = 250 °C. The carrier gas was N_2.

Table S-19. Investigation of the hydrolysis of **3a** in the presence of cyclopentanone [a]

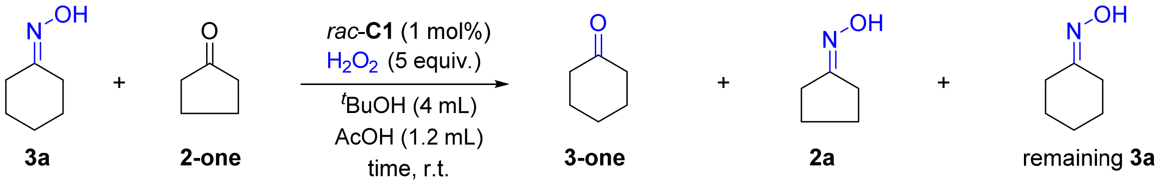

Entry	Conditions	Remaining 3a (%)	3-one (%)	2a (%)
1	3 mins later	57	40	3
2	30 mins later	50	43	6
3	60 mins later	46	47	7
4	**2-one** was added at 30 mins	53	41	6

[a] General conditions: **3a** (1 equiv., 1 mmol), **2-one** (1 equiv. 1 mmol), ***rac*-1** (1 mol%), ^{t}BuOH (4 mL), AcOH (1.2 mL), and H_2O_2 (5 equiv., 10 mmol, 568 uL, 30% wt. in H_2O, added all at once, not in batches), r.t., t minus; yield determined by GC of crude reaction mixture.

Entry 2: In a reaction tube, **3-one** (98.2 mg, 1 mmol), **2-one** (84.2 mg, 1 mmol), *rac*-**C1** (10.2 mg, 1 mol%) and hydroxylamine sulfate (328.4 mg, 2.0 mmol. 2.0 equiv.) were added to a mixed solvent of AcOH (1.2 mL) and ^{t}BuOH (4 mL). Then, H_2O_2 (5 equiv.,10 mmol, 568 uL, 30% wt. in H_2O) was added to the mixture under room temperature. After stirring for 0.5 hour, the resulting solution was taken and the amount of **remaining 3a**, **3-one** and **2a** was analyzed by GC (**remaining 3a**, 50% GC yield; **3-one**, 43% GC yield; **2a**, 6% GC yield; Calibration curve of **2a**: $y = 0.8013*x+0.0052$, $R^2=0.9996$, $x = A_{2a} / A_{1,4\text{-Dibromobenzene}}$; $y = n_{2a} / n_{1,4\text{-Dibromobenzene}}$); GC trace is shown in **Fig S17**. For the **entries 1** and **3**, similar procedures were followed, but under the different time listed in **Table S19**.

Entry 4: The reaction conditions were identical to those in **entry 2,** except that **2-one** was added 30 minutes later. After stirring for additional 0.5 h, the resulting solution was taken and the amount of **remaining 3a**, **3-one** and **2a** was analyzed by GC (**remaining 3a**, 53% GC yield; **3-one**, 41% GC yield; **2a**, 6% GC yield). GC trace is shown in **Fig S18**.

These results show that 1) under the reaction conditions, oxime undergoes fast hydrolysis, 2) the released hydroxylamine reacts with ketone almost instantly, and 3) the hydrolysis, involving reaction with cyclopentanone, reaches equilibrium within approximately half an hour.

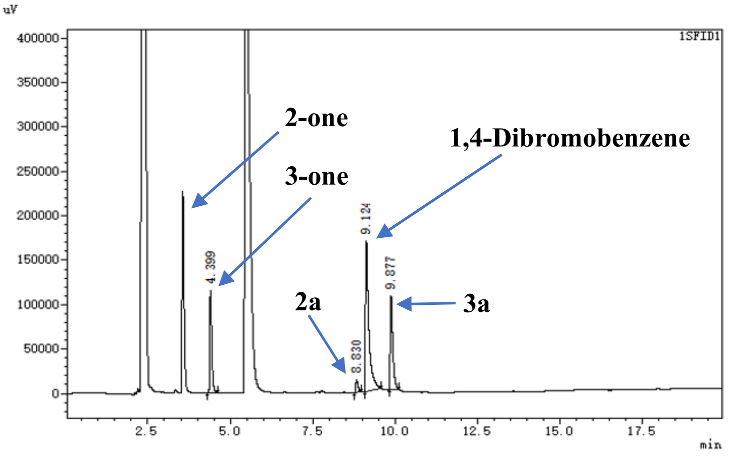

Peak#	Ret. Time	Area	Height	Concentration	Concentration Unit	Mark
1	4.399	476974	114527	20.429		M
2	8.830	65446	14261	2.803		M
3	9.124	1223182	169450	52.390		
4	9.877	569180	105803	24.378		
Toal		2334782	404042	100.000		

Figure S-17. GC trace of the mixture resulting from **entry 2**; GC parameters: injection temperature = 250 ºC, column temperature = 80 ºC, detector temperature = 250 ºC. The carrier gas was N_2.

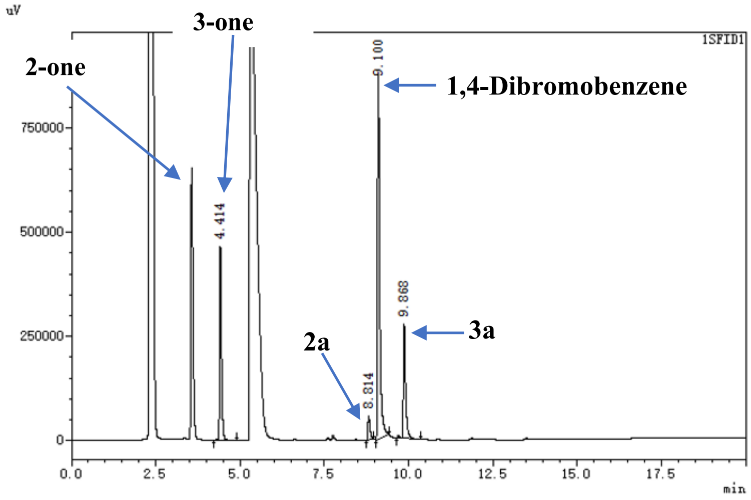

Peak#	Ret. Time	Area	Height	Concentration	Concentration Unit	Mark
1	4.414	1714831	463050	24.150		M
2	8.814	239268	56380	3.370		M
3	9.100	3844328	868603	54.139		
4	9.868	1302375	273909	18.341		
Toal		7100803	1661941	100.000		

Figure S-18. GC trace of the mixture resulting from **entry 4**; GC parameters: injection temperature = 250 ºC, column temperature = 80 ºC, detector temperature = 250 ºC. The carrier gas was N_2.

Table S-20. Examination of the possibility of formation **3a** or **(3a)$_2$•H$_2$SO$_4$** salt in the oximation of cyclohexane [a]

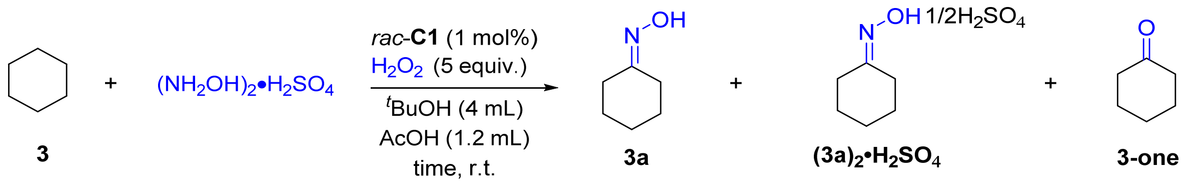

**Entry**	**Time** (x mins)	**3-one** (%)	**3a** (%)	**(3a)$_2$•H$_2$SO$_4$** (%)	**3a** (%) [b]
1	10	14.1	0.1	0.3	14.5
2	15	27.3	0.6	0.1	28.0
3	30	17.9	26.1	5.7	49.7
4	60	13.8	38.4	22.8	75.0

[a] General conditions: **3** (1 equiv., 1 mmol), $(NH_2OH)_2•H_2SO_4$ (2 equiv. 2 mmol), ***rac*-1** (1 mol%), ^{t}BuOH (2 mL), AcOH (1.2 mL), and H_2O_2 (5 equiv., 5 mmol, 568 uL, 30% wt. in H_2O) in 2 mL ^{t}BuOH added dropwise via a syringe pump over 1 h under stirring at 0 °C; yield determined by GC of crude reaction mixture. [b] Basified with Na_2CO_3 powder for 0.5 h at 0 °C.

Entry 1: In a reaction tube, **3** (84.2 mg, 1 mmol), *rac*-**C1** (10.2 mg, 1 mol%) and hydroxylamine sulfate (328.4 mg, 2.0 mmol. 2.0 equiv.) were added to a mixed solvent of AcOH (1.2 mL) and ^{t}BuOH (2 mL). Then, H_2O_2 (5 equiv., 10 mmol, 568 uL, 30% wt. in H_2O, dropping rate: 0.0428 mL/min) in 2 mL ^{t}BuOH added dropwise via a syringe pump over a certain period of time (x mins) under stirring at 0 °C without nitrogen protection. After the dropwise addition H_2O_2 over 10 minutes (for **entry 1**), the resulting solution was analyzed by GC to determine the amounts of **3-one** and **3a**. However, **(3a)$_2$•H$_2$SO$_4$** could not be detected by GC due to its high boiling point, which exceeds the GC detection limit. The corresponding GC trace is provided in **Fig S19** (left). Then, the collected solution was basified with Na_2CO_3 powder for 0.5 h, and the amount of **3a** was analyzed by GC (see **Fig S19**, right). The yield of **(3a)$_2$•H$_2$SO$_4$** salt was calculated to be approximately 0.3% based on the difference between the amount of **3a** measured after Na_2CO_3 basification and the combined amounts of **3a** and **3-one** present in the reaction mixture without basification. The basification step converts **3-one** and **(3a)$_2$•H$_2$SO$_4$** to **3a**. The same procedure was followed for the remaining entries, with variations in the H_2O_2 dropwise addition time as indicated in **Table S20**. The corresponding results are compiled in the same table.

These results show that the oxime is formed quickly in the oxidative oximation of cyclohexane and formed in a significant amount within half an hour. The initial slower formation of oxime is most likely due to the solubility of the hydroxylammonium sulfate, which is low initially but increases

with the gradual introduction of aqueous H_2O_2. Note as before, the oxime salt is not detectable on GC.

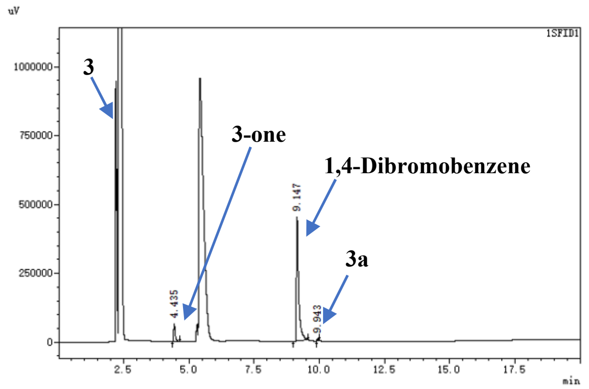

Peak#	Ret. Time	Area	Height	Concentration	Concentration Unit	Mark
1	4.435	250860	64136	8.416		M
2	9.147	2700887	448679	90.611		M
3	9.943	29015	7482	0.973		
Toal		2980762	520297	100.000		

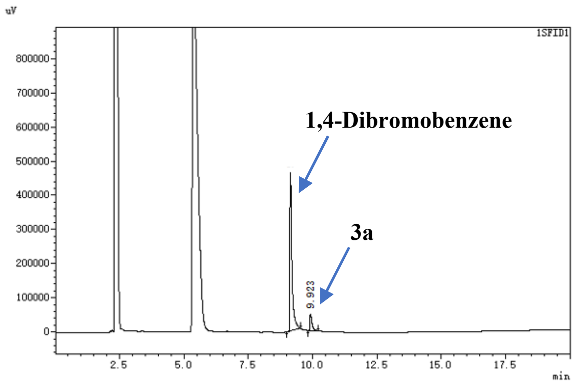

Peak#	Ret. Time	Area	Height	Concentration	Concentration Unit	Mark
1	9.137	2616010	463866	90.323		M
2	9.923	280274	48759	9.677		M
Toal		2896284	512625	100.000		

Figure S-19. GC trace of the mixture resulting from **Table S-20, entry 1 (left :** no basification; **right:** after basification with Na_2CO_3); GC parameters: injection temperature = 250 ºC, column temperature = 80 ºC, detector temperature = 250 ºC. The carrier gas was N_2.

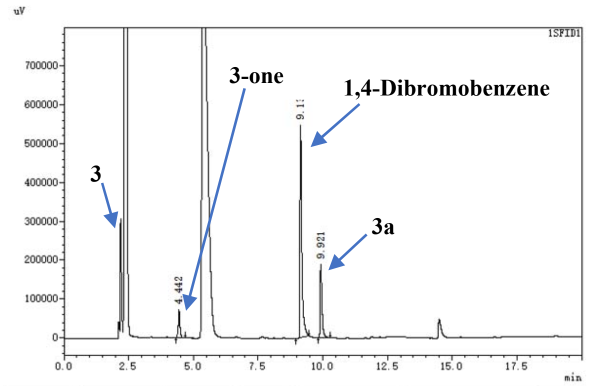

Peak#	Ret. Time	Area	Height	Concentration	Concentration Unit	Mark
1	4.442	318611	73386	8.168		M
2	9.139	2666908	543305	68.371		M
3	9.921	915149	188581	23.461		
Toal		3900669	520297	100.000		

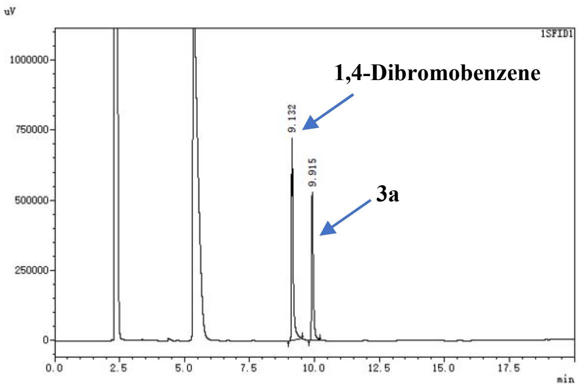

Peak#	Ret. Time	Area	Height	Concentration	Concentration Unit	Mark
1	9.132	3277659	721114	58.856		M
2	9.915	2291257	528918	41.144		M
Toal		5568916	1250032	100.000		

Figure S-20. GC trace of the mixture resulting from **Table S-20, entry 4 (left :** no basification; **right:** after basification with Na_2CO_3); GC parameters: injection temperature = 250 ºC, column temperature = 80 ºC, detector temperature = 250 ºC. The carrier gas was N_2.

6.4. Evaluation of the stability of **1a** and **1-one** in manganese catalyzed oxidation systems

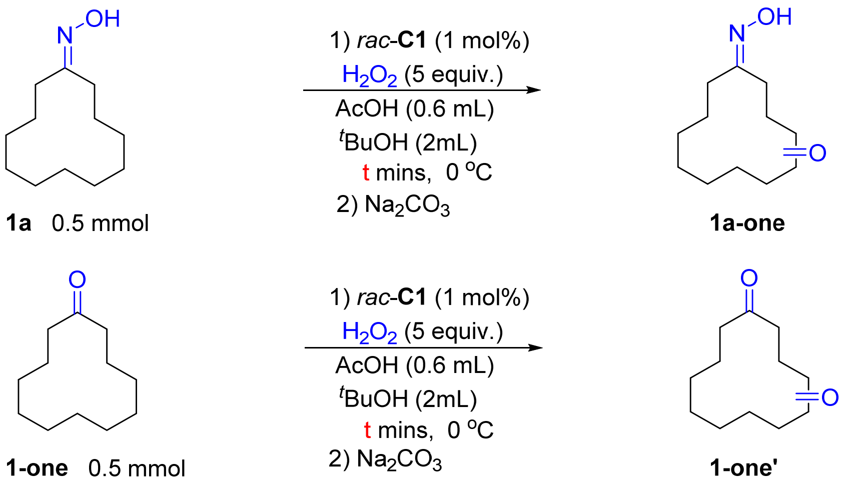

Table S-21. Comparison of the stability of **1a** and **1-one** under oxidation conditions

**Entry**	**Substrate**	**Time** (t mins)	**Remaining substrate** (%)	**Product** (%)
1	**1a**	20	96	4
2	**1a**	30	93	7
3	**1a**	40	87	13
4	**1-one**	20	71	29
5	**1-one**	30	63	37
6	**1-one**	40	49	51

[a] General conditions: **1a** or **1-one** (1 equiv., 0.5 mmol), *rac*-**C1** (1 mol%), ^{t}BuOH (1 mL), AcOH (0.6 mL), and H_2O_2 (5 equiv., 2.5 mmol, 284 uL, 30% wt. in H_2O, dropping rate: 0.0214 mL/min) in 1 mL of ^{t}BuOH introduced with a syringe pump over the time indicated under stirring at 0 °C; the solution was quenched with Na_2SO_3 and then basified with Na_2CO_3 powder for 0.5 h at 0 °C; yield determined by ^{1}H and ^{13}C NMR of crude reaction mixture.

***rac*-C1** (5.1 mg, 1.0 mol%), cyclododecanone (91.2 mg, 0.5 mmol, 1.0 equiv.) or cyclododecanone oxime (98.7 mg, 0.5 mmol, 1.0 equiv.), AcOH (0.6 mL) and ^{t}BuOH (1.0 mL) were added to a reaction tube. The mixture was cooled down to 0 °C in a cryogenic bath, and then H_2O_2 (2.5 mmol, 5.0 equiv., 284 uL, 30% wt. in H_2O, dropping rate: 0.0214 mL/min) in 1 mL of ^{t}BuOH was added dropwise with a syringe pump over a certain period of time under stirring at 0 °C without nitrogen protection. The reaction solution was quenched with Na_2SO_3 solid, and then the mixture was basified with Na_2CO_3 for 30 minutes at room temperature, filtered and washed with DCM (2.0 mL). The yield was determined by ^{1}H and ^{13}C NMR analysis of the crude reaction mixture with 1,3,5-trimethoxybenzene as internal standard (yield variation with time is shown in Table **S-21** and **Fig S21, S22**).

These results clearly show the deactivating effect of the oxime moiety on further C-H oxidation.

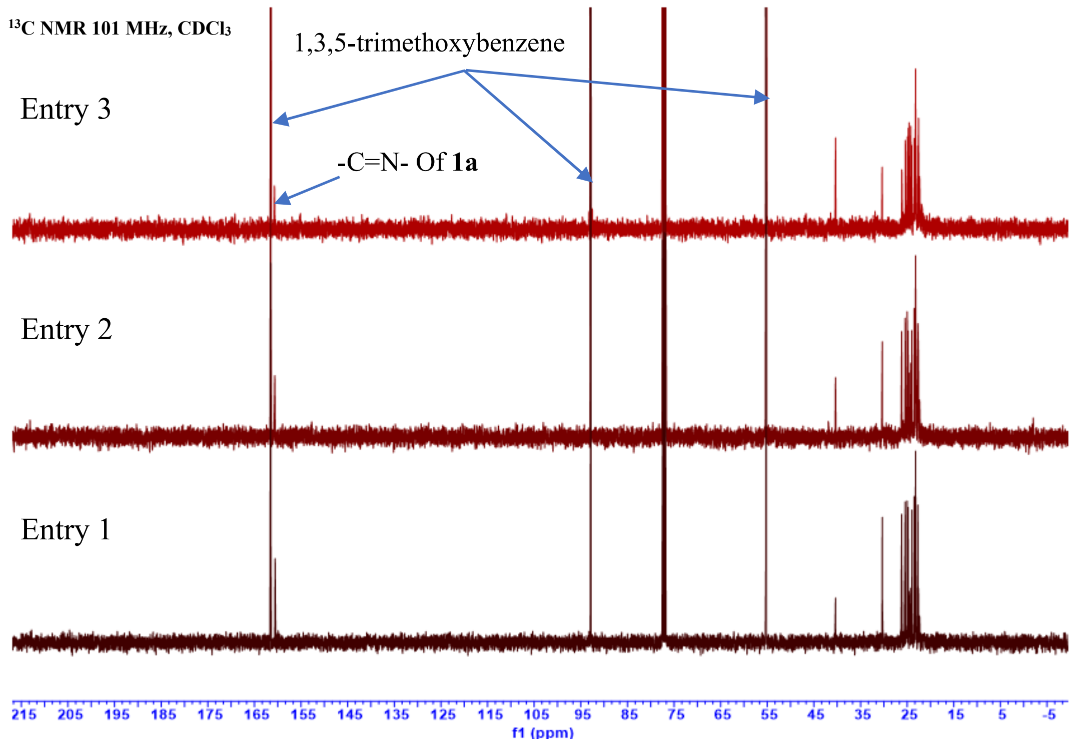

Figure S-21. ^{13}C NMR (101 MHz, $CDCl_3$) of **1a** in the manganese-catalyzed oxidation system

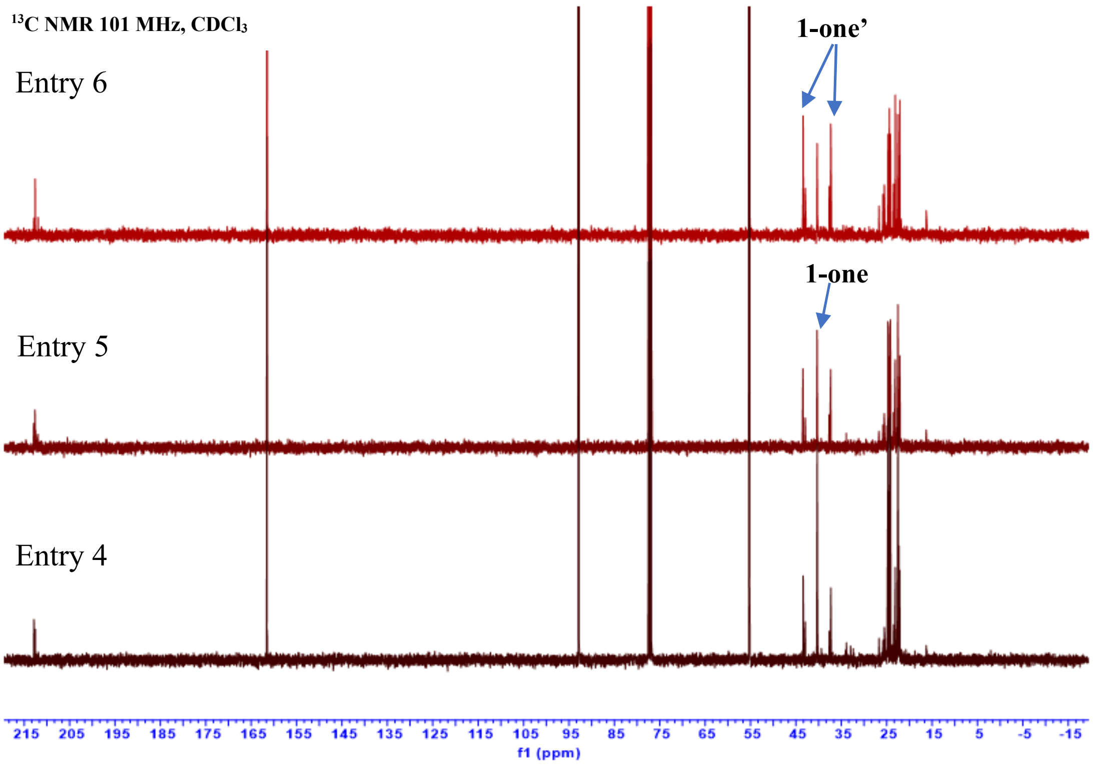

Figure S-22. ^{13}C NMR (101 MHz, $CDCl_3$) of **1-one** in the manganese catalyzed-oxidation system

6.5. Formation of cyclododecanone oxime by mixing cyclododecanone with hydroxylamine sulfate

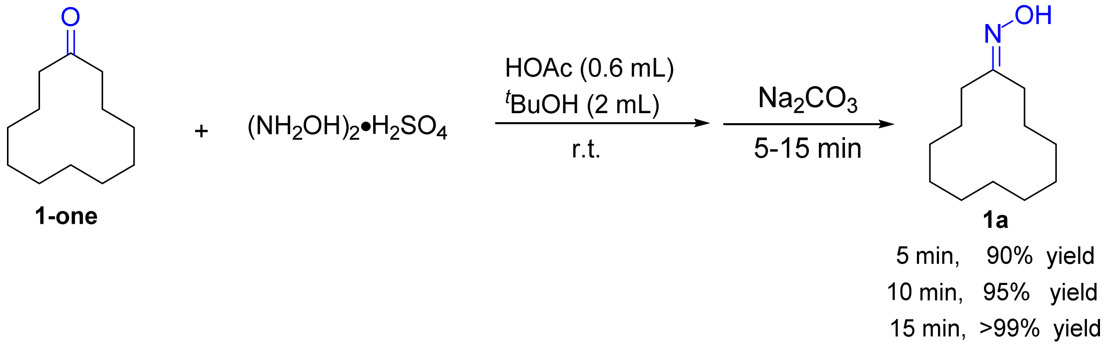

In a reaction tube, cyclododecanone (91.2 mg, 0.5 mmol) and hydroxylamine sulfate (164.2 mg, 1.0 mmol. 2.0 equiv.) were added to a mixed solvent of AcOH (0.6 mL) and ^{t}BuOH (2 mL). After briefly (<1 min) shaking the solution, the resulting mixture was basified with Na_2CO_3 for about 5 minutes at room temperature. Then, water (10.0 mL) was added, and the solution was extracted with DCM (3 × 15.0 mL). The combined organic phase was dried over anhydrous Na_2SO_4 and concentrated under reduced pressure to produce the crude product. The reaction was repeated under the same conditions except with a longer time of basification (10 and 15 min). Yields were determined by ^{1}H NMR of the crude reaction mixture.

6.6. Isolation of cyclododecanone in the oximation of cyclododecane

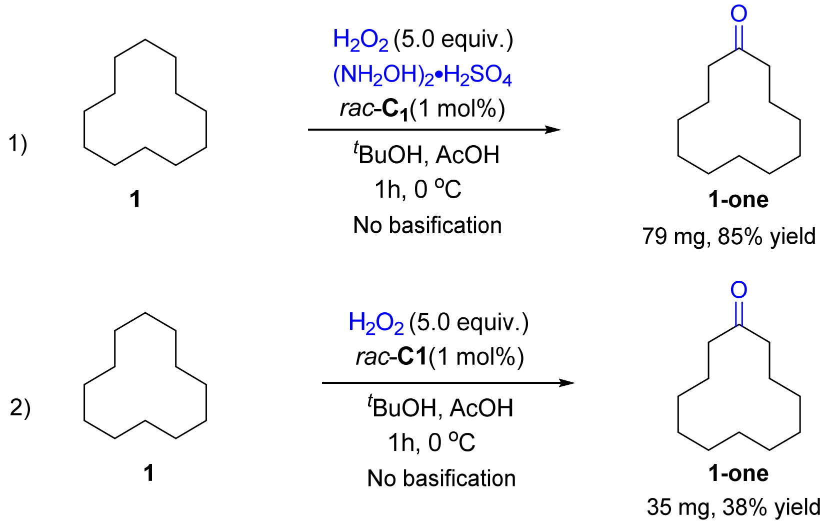

1) The reaction was carried out under the standard conditions (Section 9, procedure A), but without the basification step. The resulting mixture was evaporated to partially remove AcOH under reduced pressure to produce the crude product, and the residue was purified through flash

column chromatography (gradient elution: petroleum ether : EtOAc= 30:1 to 10:1, then 3:1) to give cyclododecanone (**1-one, 79 mg, 85% yield**).

2) The reaction was carried out under the standard conditions (Section 9, procedure A), but without $(NH_2OH)_2$•H_2SO_4. Cyclododecanone was isolated from the mixture of ketones (**1-one, 35 mg, 38% yield**).

6.7 Oximation of cyclododecane with NH_2OH in the presence/absence of sulfuric acid

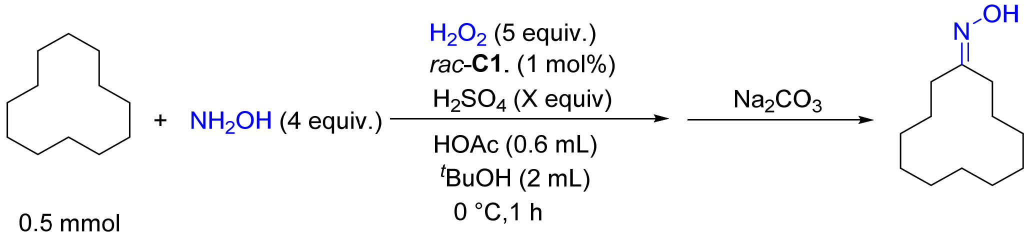

X	0	1	2	3	4
NMR yield	0	2%	38%	0%	0%

In a reaction tube, *rac*-**C1** (5.1 mg, 1mol%) and cyclododecane (84.2 mg, 0.5 mmol) were dissolved in the mixed solvent of AcOH (0.6 mL) and ^{t}BuOH (1 mL); then, NH_2OH (2 mmol, 4 equiv., 50 wt.% in H_2O) and H_2SO_4 (0–2 mmol**,** 0–4 equiv., 98 wt.%) were added to the mixed solvent. The mixture was cooled down to 0 ºC in a cryogenic bath, and then H_2O_2 (2.5 mmol, 5.0 equiv.) in 1 mL of ^{t}BuOH was added dropwise with a syringe pump over 1 h under stirring at 0 ºC without nitrogen protection. The reaction mixture was stirred for another 30 min, and then quenched with Na_2SO_3 solid. Next, the resulting mixture was basified with Na_2CO_3 for about 30 minutes at room temperature. After completion of the reaction, water (5.0 mL) was added, and the reaction solution was extracted with DCM (3 × 15.0 mL). The combined organic phase was dried over anhydrous Na_2SO_4 and concentrated under reduced pressure to produce the crude product. Yields determined by ^{1}H NMR of crude reaction mixture.

6.8. Effect of $(NH_2OH)_2$•H_2SO_4 on the regioselectivity of *rac*-C1 catalyzed oxidation of substrates 17 and 30

1)

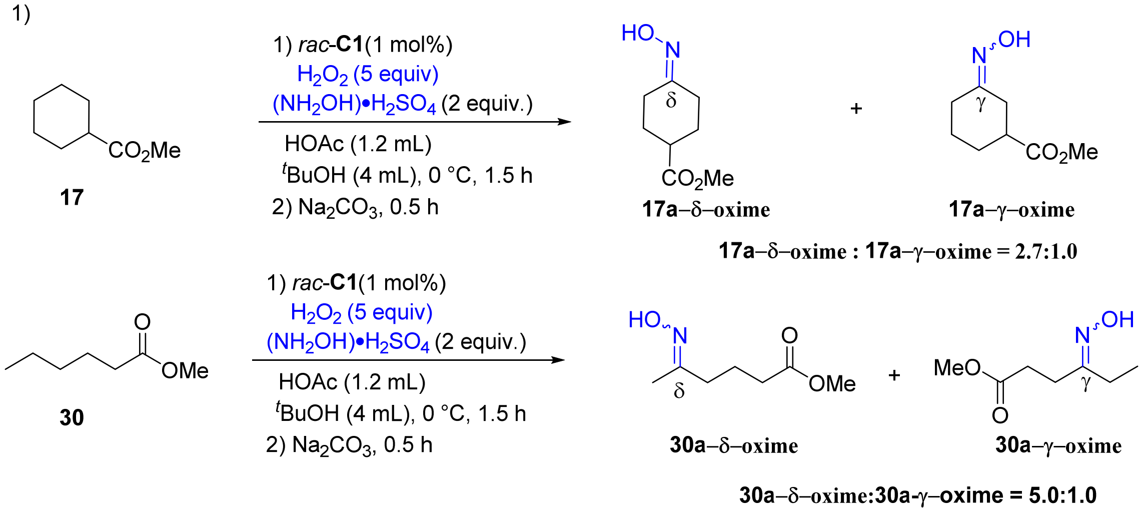

2)

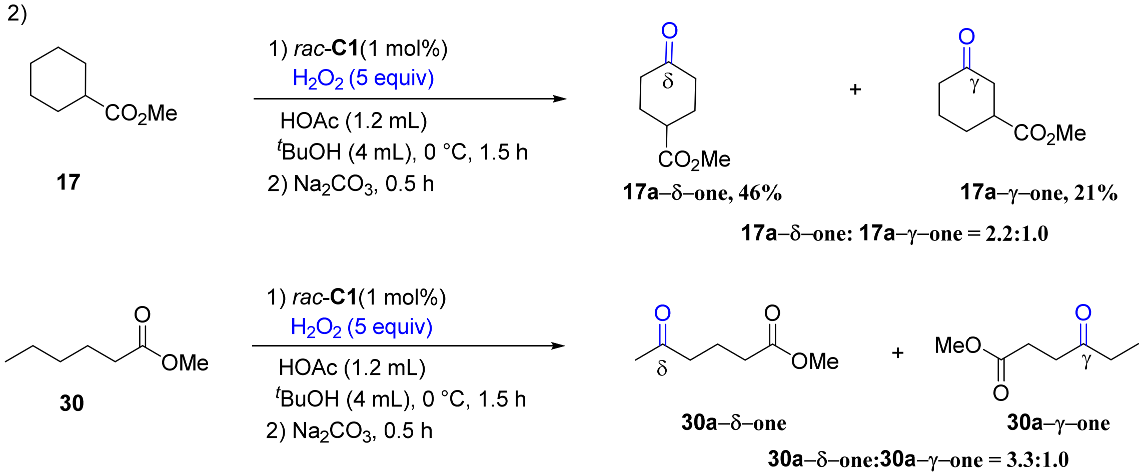

1) **With $(NH_2OH)_2$•H_2SO_4:** the reaction was performed according to Section 9, Procedure A: using either substrate **17** (142.2 mg, 1.0 mmol, 1.0 equiv.) or **30** (130.2 mg, 1.0 mmol, 1.0 equiv.) with H_2O_2 (5.0 equiv.).

For substrate **17**, the reaction yielded a mixture of two isomeric oxime products (**17a-δ-oxime** and **17a-γ-oxime**) in 86% combined yield. The regioselectivity was determined through analysis of the ^{1}H and ^{13}C NMR spectra of the product mixture (see Section 12 for full characterization data of product **17a**). Similarly, when using substrate **30**, we obtained a mixture of **30a-δ-oxime** and **30a-γ-oxime** isomers in 63% combined yield. The regiochemical assignment was also confirmed by NMR spectroscopic analysis (see **Section 12** for full characterization data of product **30a**).

2) **Without $(NH_2OH)_2$•H_2SO_4:** the reaction was performed according to Section 9, Procedure A: using either substrate **17** (142.2 mg, 1.0 mmol, 1.0 equiv.) or **30** (142.2 mg, 1.0 mmol, 1.0 equiv.) with H_2O_2 (5.0 equiv.), but without $(NH_2OH)_2$•H_2SO_4.

   For substrate **17**, the reaction yielded a mixture of two isomeric ketone products **17-δ-one**, and **17-γ-one**; regioselectivity was assigned based on analysis of the ^{13}C NMR of crude reaction mixture (see Figure S-23). For substrate **30**, a mixture of **30a-δ-one** and **30a-γ-one** was observed; regioselectivity was assigned based on analysis of the ^{1}H NMR of crude reaction mixture (see Figure S-24).

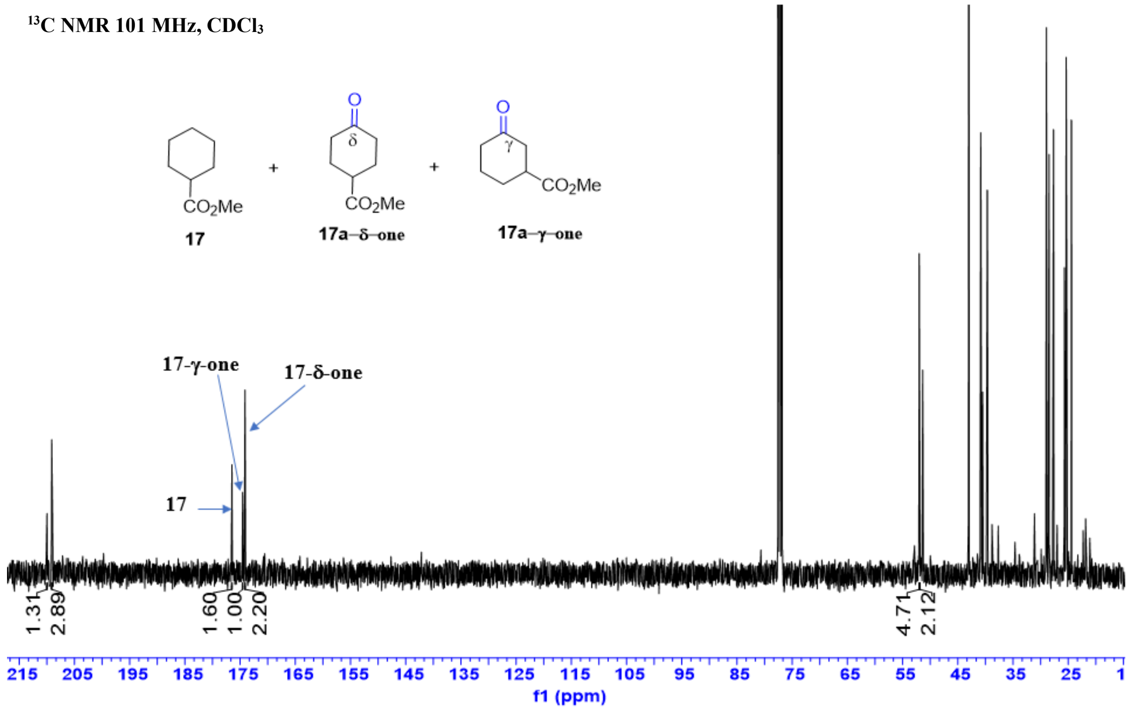

Fig S-23. Oxidation of **17** catalyzed by ***rac*-C1** without $(NH_2OH)_2$•H_2SO_4

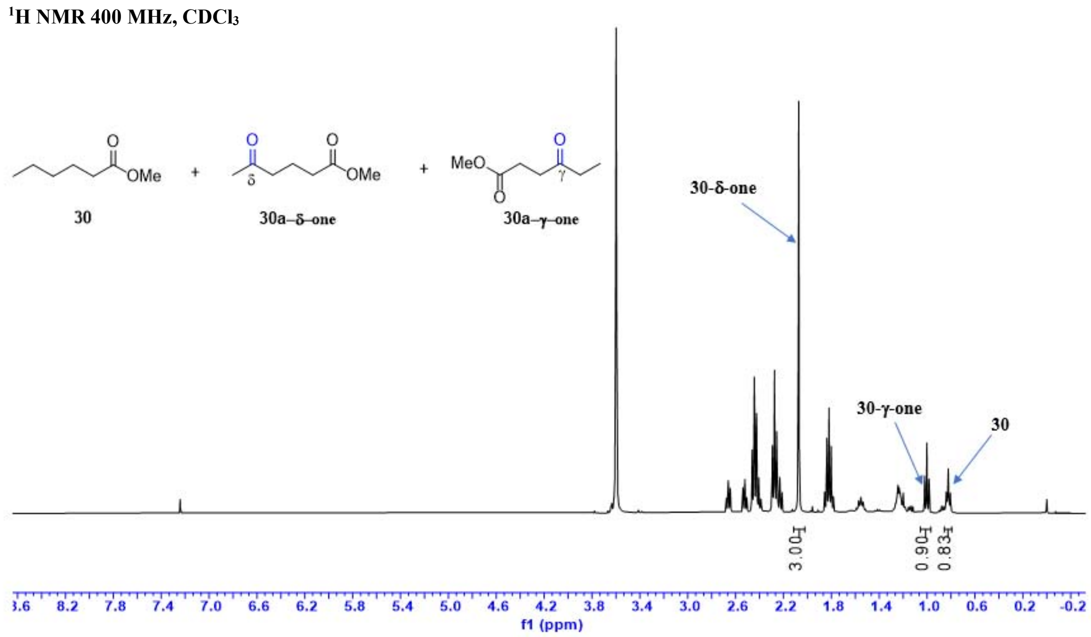

Fig S-24. Oxidation of **30** catalyzed by ***rac*-C1** without $(NH_2OH)_2{\bullet}H_2SO_4$

6.9. Effect of $(NH_2OH)_2{\bullet}H_2SO_4$ on the *rac*-C2 catalyzed oximation of (*E*)-hex-2-enoate

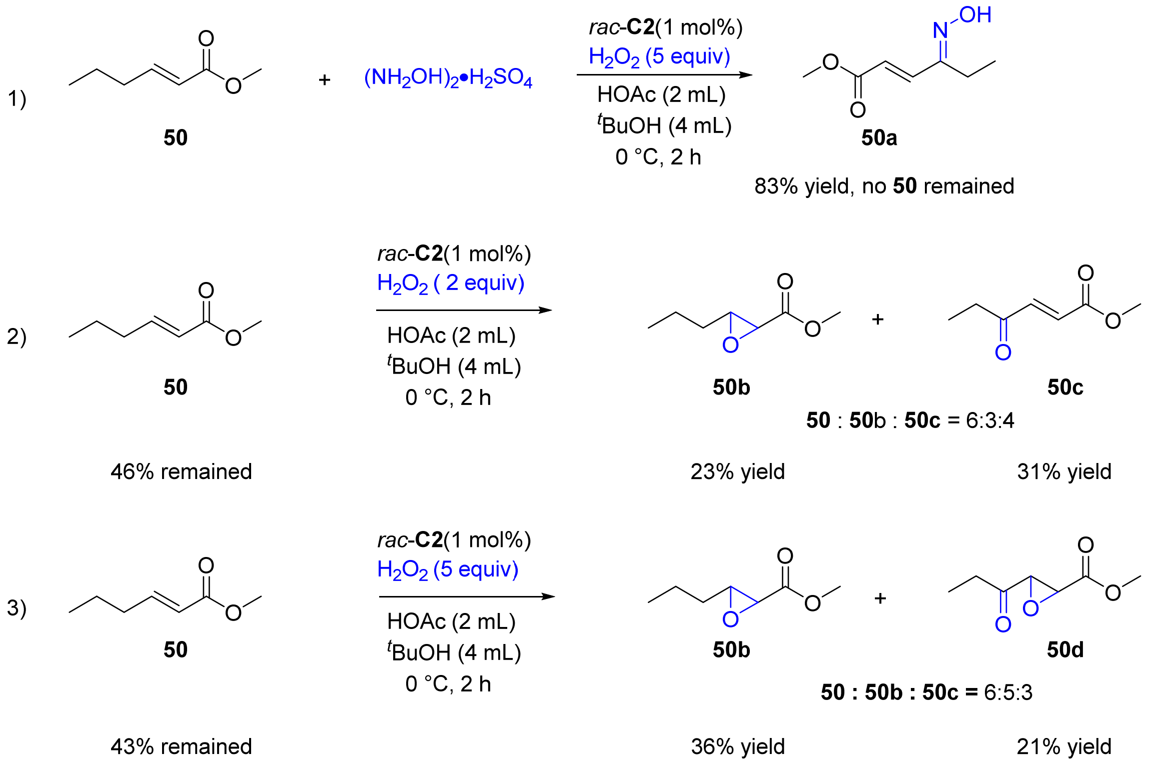

1) **With $(NH_2OH)_2{\bullet}H_2SO_4$:** the reaction was carried out according to Section 9, Procedure B, using (*E*)-hex-2-enoate (128.2 mg, 1.0 mmol, 1.0 equiv.) with H_2O_2 (5.0 equiv.). A mixture of

two isomers (*E*)-**50a** and (*Z*)-**50a** was obtained, in 83% isolated yield (see Figure S-25, 1). For the analytic data, see Section 12.

2) Without $(NH_2OH)_2$•H_2SO_4: the reaction was carried out according to Section 9, Procedure B using (*E*)-hex-2-enoate (128.2 mg, 1.0 mmol, 1.0 equiv.) with H_2O_2 (2.0 equiv.) but without $(NH_2OH)_2$•H_2SO_4. A mixture of **50** (**46%** remained), **50b** (**23%** yield), and **50c** (**31%** yield) was obtained; yield determined by ^{1}H NMR of crude reaction mixture (see Figure S-25, 2); **50b**[63], **HRMS (ESI):** *m/z* $[M+H]^+$ calcd for $C_7H_{13}O_3^+$:145.0859; found: 145.0873; **50c**[64], **HRMS (ESI):** *m/z* $[M+H]^+$ calcd for $C_7H_{11}O_3^+$:143.0703; found: 143.0707.

3) Without $(NH_2OH)_2$•H_2SO_4: the reaction was carried out according to Section 9, Procedure B using (*E*)-hex-2-enoate (128.2 mg, 1.0 mmol, 1.0 equiv.) with H_2O_2 (5.0 equiv.) but without $(NH_2OH)_2$•H_2SO_4. A mixture of **50** (**43%** remained), **50b** (**36%** yield), and **50d** (**21%** yield) was obtained; yield determined by ^{1}H NMR of crude reaction mixture (see Figure S-25, 3); **50d**, **HRMS (ESI):** *m/z* $[M+H]^+$ calcd for $C_7H_{11}O_4^+$:159.0652; found: 159.0666.

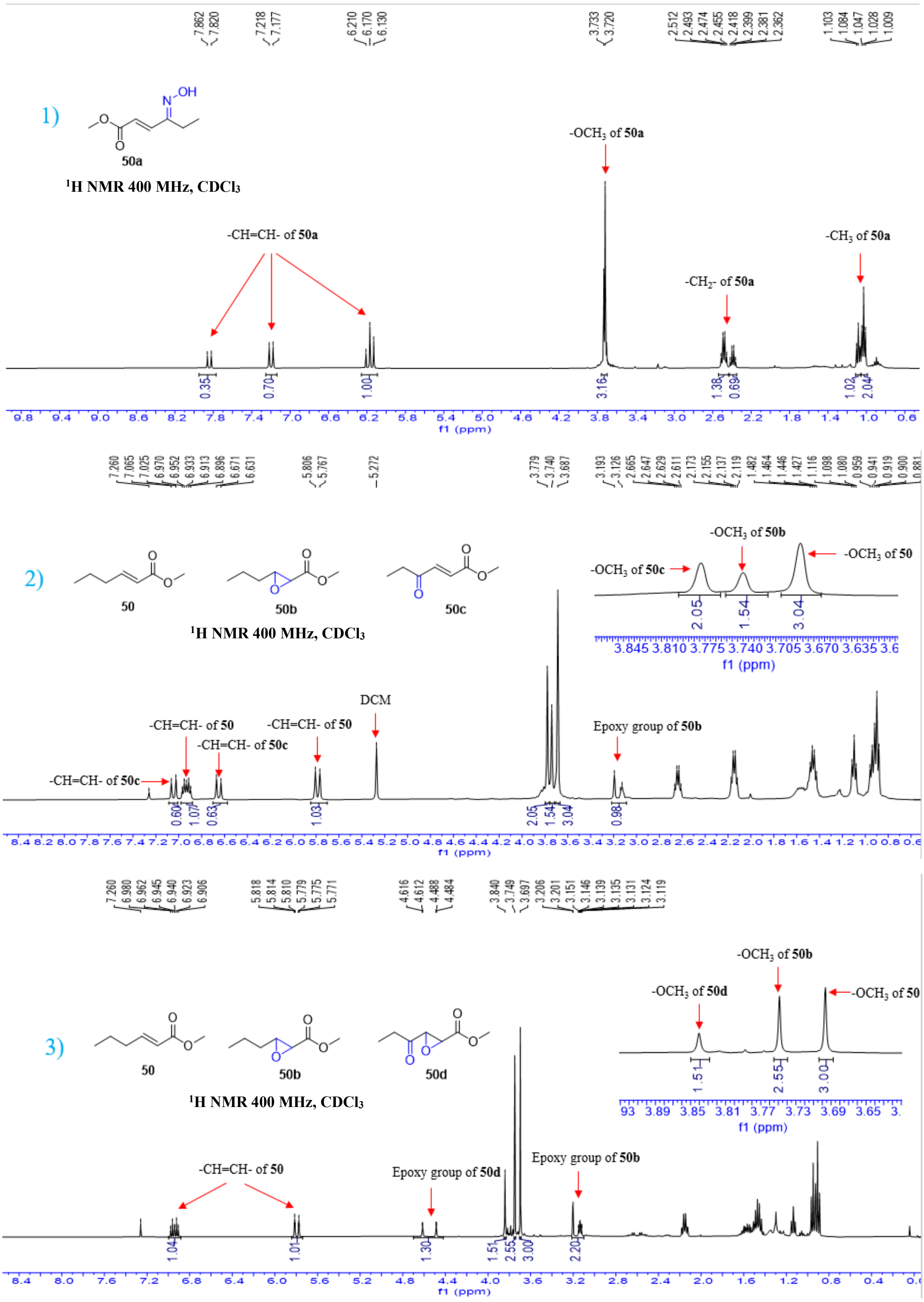

Fig S-25. Chemoselective oxidative of (*E*)-hex-2-enoate with or without $(NH_2OH)_2{\bullet}H_2SO_4$

6.10. Effect of $(NH_2OH)_2$•H_2SO_4 on the *rac*-C1 catalyzed oximation of sclareolide

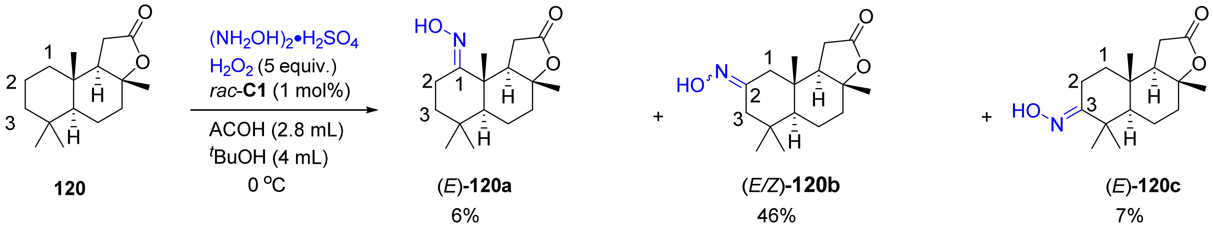

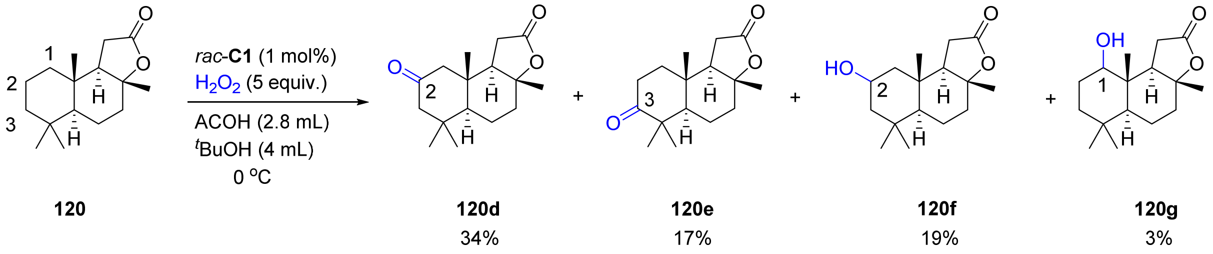

with $(NH_2OH)_2$•H_2SO_4 : $C_1/C_2/C_3$ = 1:7.7:1.2, no alcohol product observed
without $(NH_2OH)_2$•H_2SO_4 : $C_1/C_2/C_3$ = 1:18:6, alcohol : ketone = 1:2.3

1) **With $(NH_2OH)_2$·H_2SO_4:** the reaction was carried out according to Section 9, Procedure A using sclareolide (250.4 mg, 1.0 mmol, 1.0 equiv.) with *rac*-**C1** to give four separated products: ***E*-120a, *E*-120b, *Z*-120b** and ***E*-120c**; the analytic data for products are seen in the Section 12.

2) **Without $(NH_2OH)_2$·H_2SO_4:** the reaction was carried out according to Section 9, Procedure A using sclareolide (250.4 mg, 1.0 mmol, 1.0 equiv.) with *rac*-**C1** but without $(NH_2OH)_2$•H_2SO_4 to give a mixture of **120d** and **120e**, and separated products **120f** and **120g**; the analytic data for all products are seen in the Section 12.

6.11. Trapping of radical intermediates

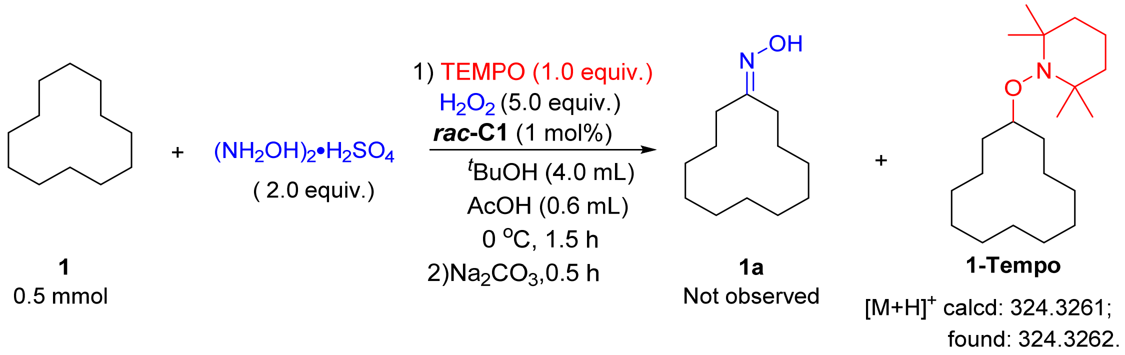

In a reaction tube, *rac*-**C1** (5.1 mg, 1mol%), hydroxylamine sulfate (164.2 mg, 1.0 mmol. 2.0 equiv.) and cyclododecane (84.2 mg, 0.5 mmol) were dissolved in a mixed solvent of AcOH (0.6

mL) and ^{t}BuOH (2 mL); then, TEMPO (78.2 mg, 0.5 mmo, 1 equiv.) was added to the mixed solvent. The mixture was cooled down to 0 ºC in a cryogenic bath, and then H_2O_2 (2.5 mmol, 5.0 equiv., 284 µL, 30% wt. in H_2O) in 2 mL of ^{t}BuOH was added dropwise with a syringe pump over 1 h under stirring at 0 ºC without nitrogen protection. The reaction mixture was stirred for another 30 min and then quenched with Na_2SO_3 solid. Next, the resulting mixture was basified with Na_2CO_3 for about 30 min at 0 ºC in a cryogenic bath. NMR monitoring showed that the reaction was completely inhibited by TEMPO, and HRMS confirmed the formation of the TEMPO-trapped radical intermediate. **1-TEMPO**, HRMS(APCl): calcd for $C_{21}H_{42}NO^+$ $[M+H]^+$ 324.3261; found 324.3262.

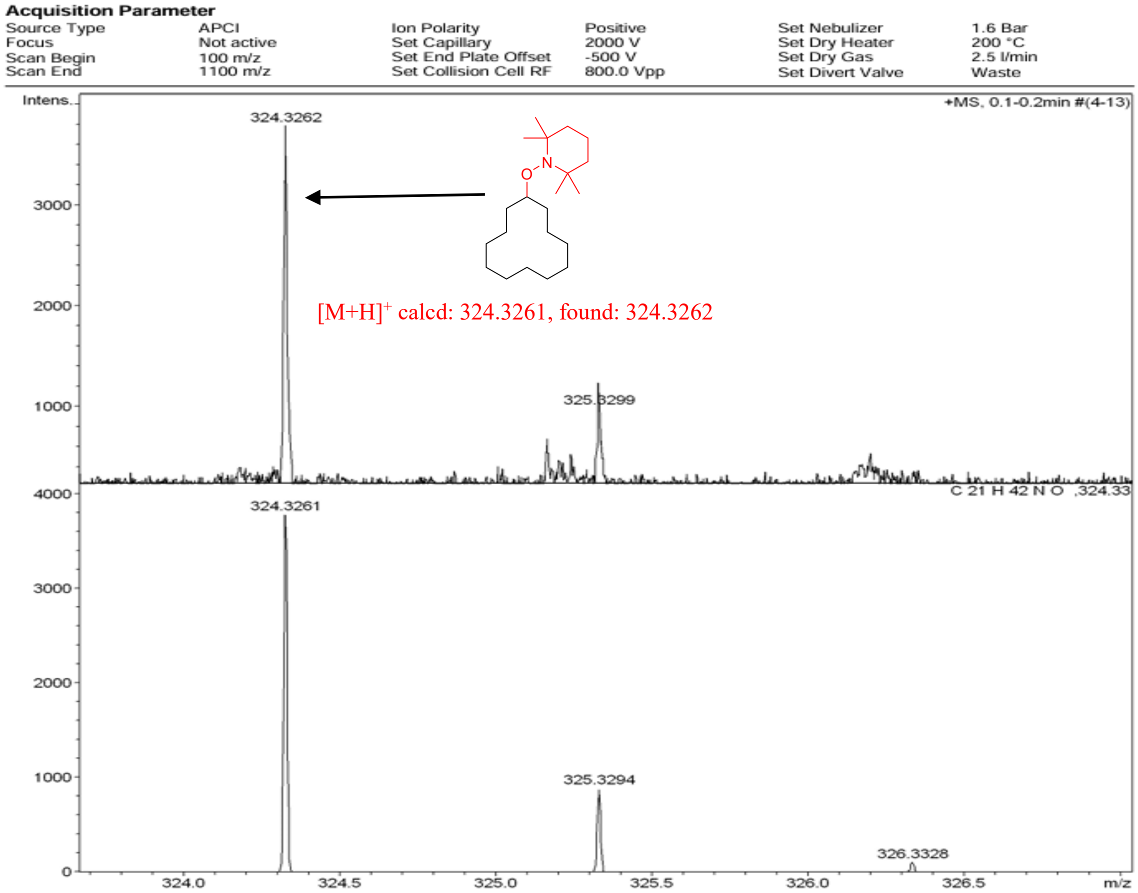

Figure S-26. TEMPO-trapped radical intermediate confirmed by HRMS

6.12. Kinetic isotope effect

1) KIE in the oximation of cyclohexane (*vs* cyclohexane-*d*$_{12}$) with $(NH_2OH)_2{\bullet}H_2SO_4$

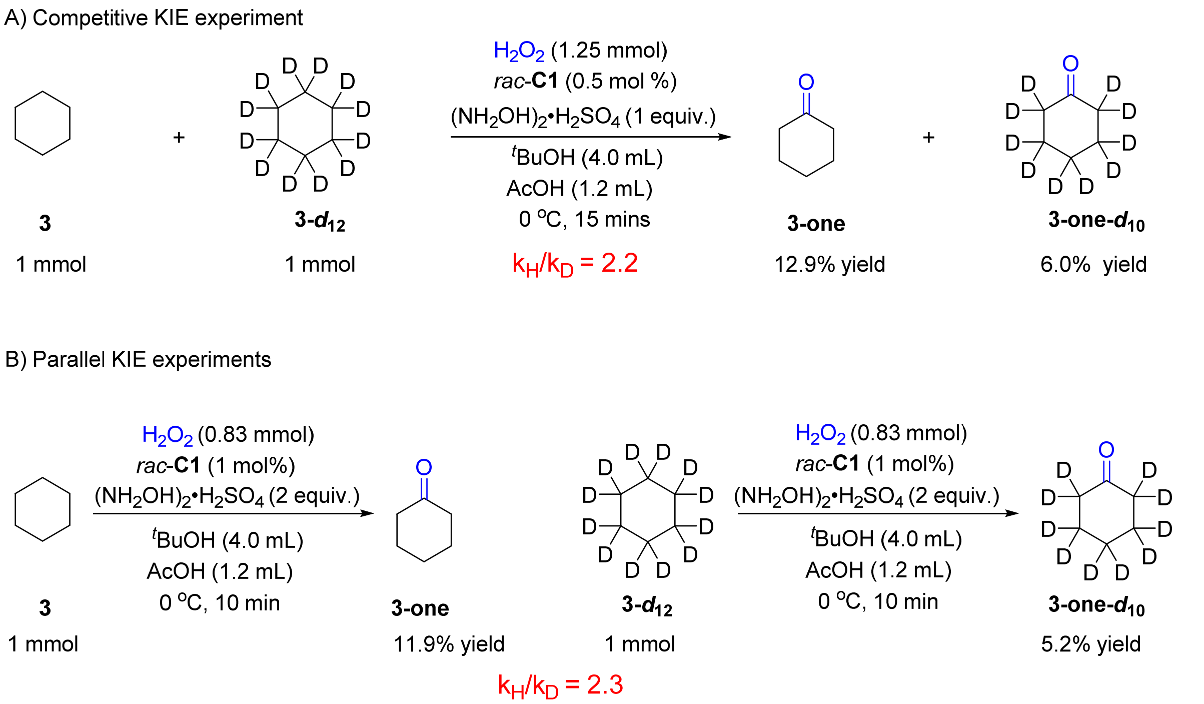

A) **Competitive KIE experiments:** In a reaction tube, ***rac*-C1** (10.2 mg, 0.5 mol%), hydroxylamine sulfate (328.4 mg, 2.0 mmol. 1.0 equiv.), cyclohexane **3** (84.2 mg, 1 mmol, 1.0 equiv.) and cyclohexane-d_{12} **3-*d*$_{12}$** (96.3 mg, 1 mmol, 1.0 equiv.) were dissolved in a mixed solvent of AcOH (1.2 mL) and ^{t}BuOH (2 mL). The mixture was cooled down to 0 ºC in a cryogenic bath, and then H_2O_2 (5 mmol, 2.5 equiv., 568 uL, 30% wt. in H_2O, dropping rate: 0.0428 mL/min) in 2 mL of ^{t}BuOH was added dropwise with a syringe pump under stirring at 0 ºC without nitrogen protection. After the addition of H_2O_2 dropwise for 15 min, the reaction solution was quenched with Na_2SO_3 solid. Next, nitrobenzene (123.1 mg, 1 mmol) as an internal standard was added to the solution. Then, the reaction solution was taken, and the yields of cyclohexanone **3-one** and cyclohexanone-d_{10} **3-one-*d*$_{10}$** were analyzed by GC (**3-one**, 12.9% GC yield; **3-one-*d*$_{10}$**, 6.0% GC yield. The KIE is estimated by using the low product yield obtained: Yield $_{\text{cyclohexanone}}$ / Yield $_{\text{cyclohexanone-}d10}$ = 2.2; GC trace and calibration curves are shown **Fig S27, S28;** calibration curve of cyclohexanone: $y = 1.0637*x+0.0334$, $R^2=0.9993$, $x = A_{\text{cyclohexanone}} / A_{\text{nitrobenzene}}$; $y = n_{\text{cyclohexanone}} / n_{\text{nitrobenzene}}$; calibration curve of cyclohexanone-d10: $y = 2.6638* x +0.0377$, $R^2=0.9993$, $x = A_{\text{cyclohexanone-d10}} / A_{\text{nitrobenzene}}$; $y = n_{\text{cyclohexanone-d10}} / n_{\text{nitrobenzene}}$).

Note that the formation of oxime is negligible under these conditions (see Table S-20). The same applies to the following KIE experiments.

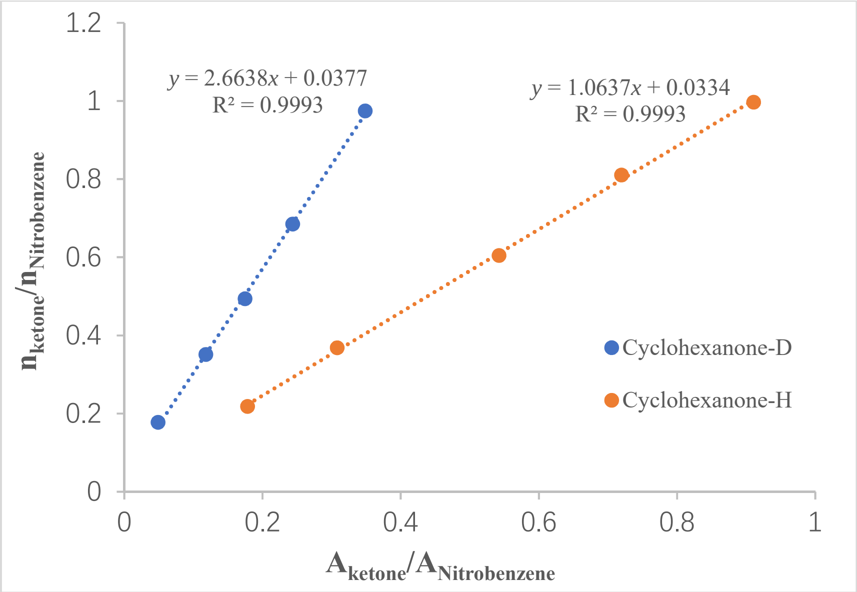

Figure S-27. GC calibration curve of **3-one** and **3-one-*d*$_{10}$** (commercial sample) with nitrobenzene (GC calibration curve: x = $A_{cyclohexanone}$ /$A_{nitrobenzene}$; y = $n_{cyclohexanone}$ /$n_{nitrobenzene}$; A: area; n: number of moles).

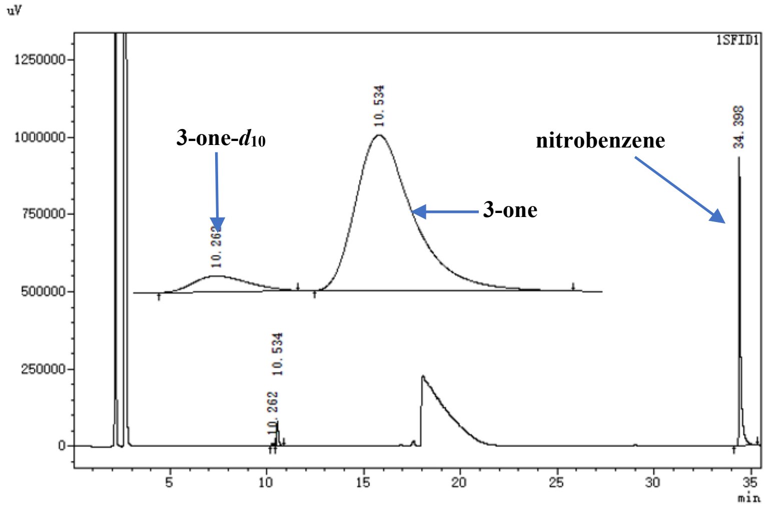

Peak#	Ret. Time	Area	Height	Concentration	Concentration Unit	Mark
1	10.262	49777	7784	0.775		M
2	10.534	524021	77194	8.156		M
3	34.398	5851188	928998	91.069		M
Toal		6424985	1013976	100.000		

Figure S-28. GC trace of the mixture resulting from oxidative oximation of **3-one** and **3-one-*d*$_{10}$**. GC parameters: injection temperature = 250 °C, column temperature = 70 °C, detector temperature = 250 °C. The carrier gas was N_2.

B) Parallel KIE experiments: ***rac*-C1** (10.2 mg, 1.0 mol%), hydroxylamine sulfate (328.4 mg, 2.0 mmol. 2.0 equiv.), AcOH (1.2 mL), ^{t}BuOH (2.0 mL) and cyclohexane **3** (84.2 mg, 1 mmol, 1.0 equiv.) or cyclohexane-d_{12} (96.3 mg, 1 mmol, 1.0 equiv.) were added to a reaction tube. The mixture was cooled down to 0 ºC in a cryogenic bath, and then H_2O_2 (5 mmol, 5.0 equiv., 568 uL, 30% wt. in H_2O, dropping rate: 0.0428 mL/min) in 2 mL of ^{t}BuOH was added dropwise with a syringe pump under stirring at 0 ºC without nitrogen protection. After the addition of H_2O_2 dropwise for 10 min, the reaction solution was quenched with Na_2SO_3 solid. Next, nitrobenzene (123.1 mg, 1 mmol) as an internal standard was added to the solution. Then, the reaction solution was taken, and the yield of cyclohexanone **3-one** or cyclohexanone-d_{10} **3-one-*d*$_{10}$** was analyzed by GC (**3-one**, 11.9% GC yield; **3-one-*d*$_{10}$**, 5.2% GC yield; KIE = Yield $_{\text{cyclohexanone}}$ / Yield $_{\text{cyclohexanone-}d10}$ = 2.3; GC trace and calibration curve are shown **Fig S29, S30**.)

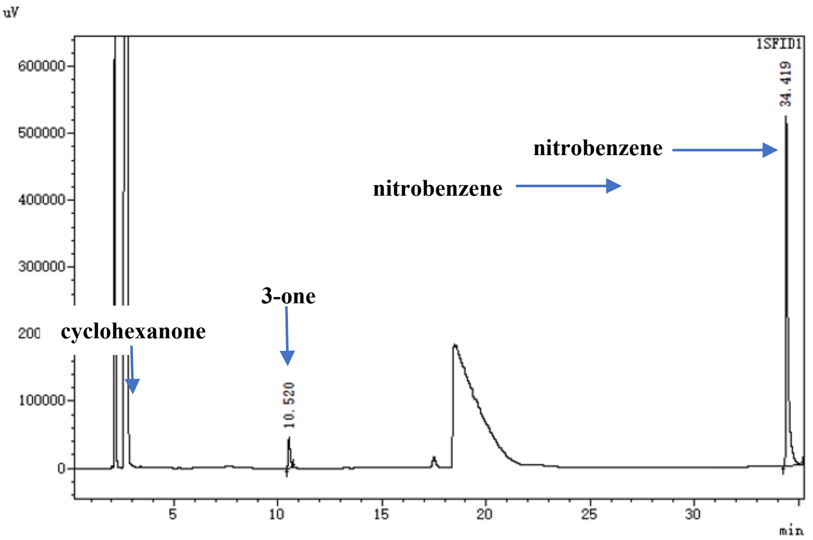

Peak#	Ret. Time	Area	Height	Concentration	Concentration Unit	Mark
1	10.520	313814	45462	7.517		M
2	34.419	3861088	521817	92.483		M
Toal		4174902	567280	100.000		

Figure S-29. GC trace of the mixture resulting from oxidative oximation of **3-one**. GC parameters: injection temperature = 250 ºC, column temperature = 70 ºC, detector temperature = 250 ºC. The carrier gas was N_2.

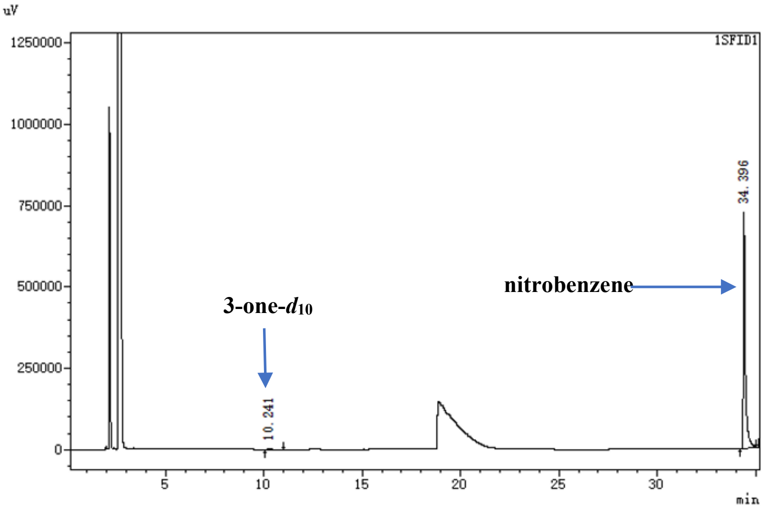

Peak#	Ret. Time	Area	Height	Concentration	Concentration Unit	Mark
1	10.241	25350	2477	0.518		M
2	34.396	4869749	725730	99.482		M
Toal		4895099	728206	100.000		

Figure S-30. GC trace of the mixture resulting from oxidative oximation of **3-one-*d*₁₀**. GC parameters: injection temperature = 250 °C, column temperature = 70 °C, detector temperature = 250 °C. The carrier gas was N_2.

2) KIE in the oximation of cyclohexane (vs cyclohexane-d_{12}) without $(NH_2OH)_2 \cdot H_2SO_4$

A) Competitive KIE experiment

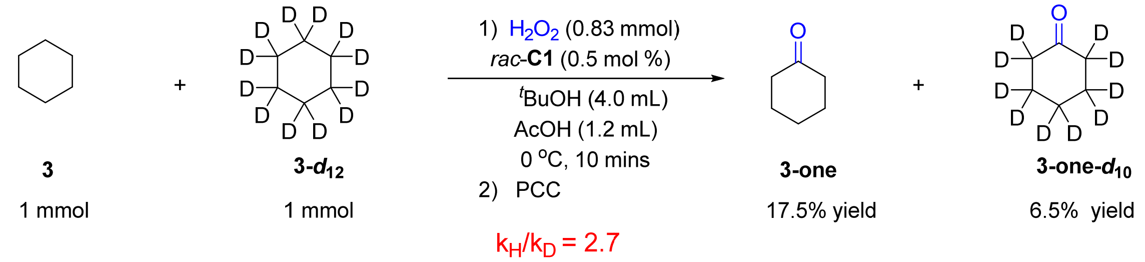

B) Parallel KIE experiments

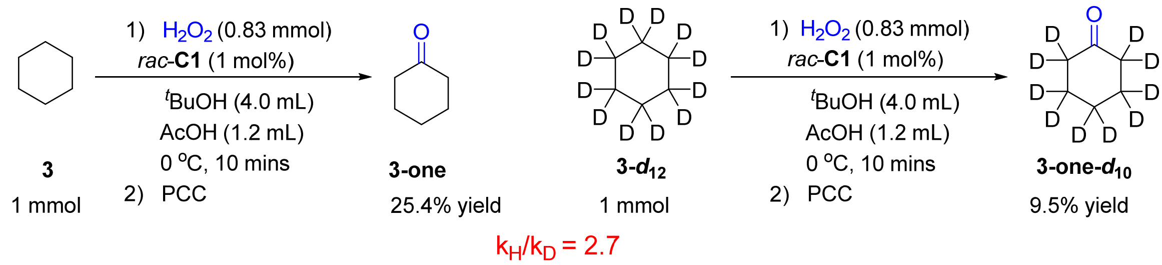

C) **Competitive KIE experiments:** In a reaction tube, ***rac*-C1** (10.2 mg, 0.5 mol%), cyclohexane (84.2 mg, 1 mmol, 1.0 equiv.) and cyclohexane-d_{12} (96.3 mg, 1 mmol, 1.0 equiv.) were

dissolved in a mixed solvent of AcOH (1.2 mL) and ^{t}BuOH (2 mL). The mixture was cooled down to 0 ºC in a cryogenic bath, and then H_2O_2 (5 mmol, 2.5 equiv., 568 uL, 30% wt. in H_2O, dropping rate: 0.0428 mL/min) in 2 mL of ^{t}BuOH was added dropwise with a syringe pump under stirring at 0 ºC without nitrogen protection. After the addition of H_2O_2 dropwise for 10 min, the reaction solution was quenched with Na_2SO_3 solid. Next, nitrobenzene (123.1 mg, 1 mmol) as an internal standard was added to the solution. Then, the reaction solution was taken and the yields of cyclohexanone **3-one** and cyclohexanone-d_{10} **3-one-d_{10}** were analyzed by GC (**3-one**, 17.5% GC yield; **3-one-d_{10}**, 6.5% GC yield; KIE = Yield $_{\text{cyclohexanone}}$ / Yield $_{\text{cyclohexanone-d10}}$ = 2.69; GC trace and calibration curve are shown **Fig S31**.)

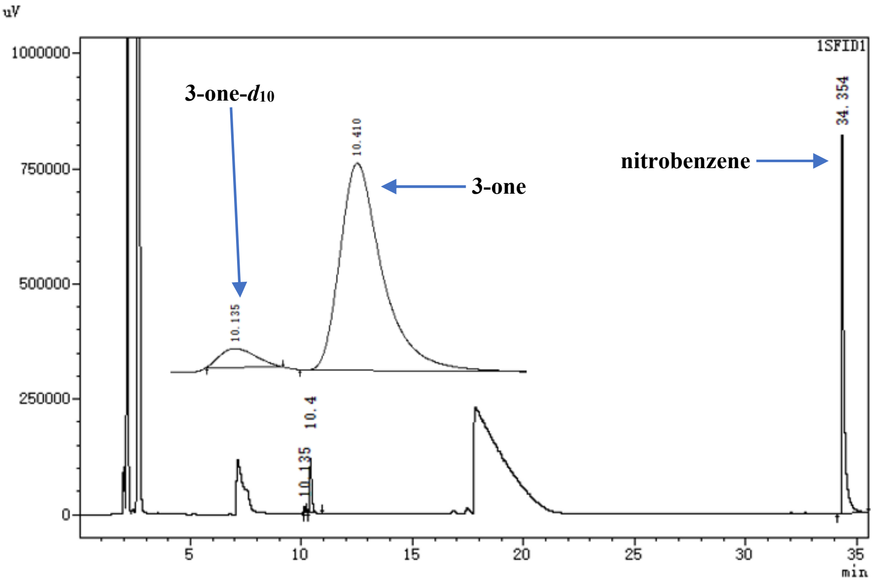

Peak#	Ret. Time	Area	Height	Concentration	Concentration Unit	Mark
1	10.135	62238	11089	0.908		M
2	10.410	795743	119862	11.615		M
3	34.354	5993277	819795	87.477		M
Toal		6851259	950746	100.000		

Figure S-31. GC trace of the mixture resulting from oxidation of **3-one** and **3-one-d_{10}**. GC parameters: injection temperature = 250 ºC, column temperature = 70 ºC, detector temperature = 250 ºC. The carrier gas was N_2.

D) **Parallel KIE experiments:** ***rac*-C1** (10.2 mg, 1.0 mol%), AcOH (1.2 mL), ^{t}BuOH (2.0 mL) and cyclohexane (84.2 mg, 1 mmol, 1.0 equiv.) or cyclohexane-d_{12} (96.3 mg, 1 mmol, 1.0 equiv.) were added to a reaction tube. The mixture was cooled down to 0 ºC in a cryogenic

bath, and then H_2O_2 (5 mmol, 5.0 equiv., 568 uL, 30% wt. in H_2O, dropping rate: 0.0428 mL/min) in 2 mL of ^{t}BuOH was added dropwise with a syringe pump under stirring at 0 ºC without nitrogen protection. After the addition of H_2O_2 dropwise for 10 min, the reaction solution was quenched with Na_2SO_3 solid. Next, nitrobenzene (123.1 mg, 1 mmol) as an internal standard was added to the solution. Then, the reaction solution was taken and the yield of cyclohexanone **3-one** or cyclohexanone-d_{10} **3-one-*d*10** was analyzed by GC (**3-one**, 25.4% GC yield; **3-one-*d*10**, 9.5% GC yield; KIE = Yield $_{\text{cyclohexanone}}$ / Yield $_{\text{cyclohexanone-}d10}$ = 2.67; GC trace and calibration curve are shown **S31, S32.**)

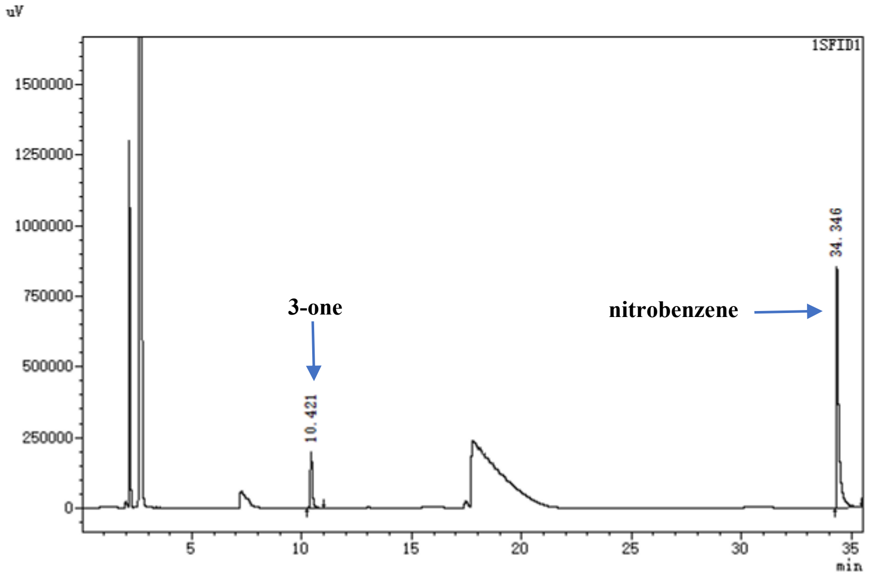

Peak#	Ret. Time	Area	Height	Concentration	Concentration Unit	Mark
1	10.421	1279558	197281	17.178		M
2	34.346	6169394	853697	82.822		M
Toal		7448952	1050979	100.000		

Figure S-32. GC trace of the mixture resulting from oxidation of **3-one**. GC parameters: injection temperature = 250 ºC, column temperature = 70 ºC, detector temperature = 250 ºC. The carrier gas was N_2.

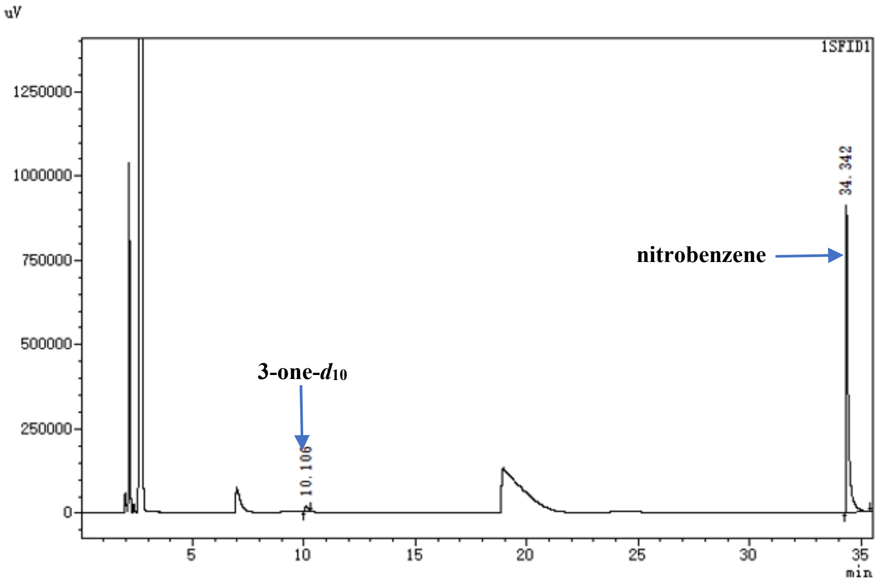

Peak#	Ret. Time	Area	Height	Concentration	Concentration Unit	Mark
1	10.106	135308	15072	2.124		M
2	34.342	6234813	908748	97.876		M
Toal		6370120	923820	100.000		

Figure S-33. GC trace of the mixture resulting from oxidation of **3-one-*d*₁₀**. GC parameters: injection temperature = 250 ºC, column temperature = 70 ºC, detector temperature = 250 ºC. The carrier gas was N_2.

6.13. UV-Vis spectra of *rac*-C1 and relevant reagents under various conditions

Where mixed reagents are concerned, all measurements (Figures S34-44) were undertaken immediately upon mixing the reagents in the solvent. All measurements were taken in cuvettes with a path length of 1 cm at 25 ºC.

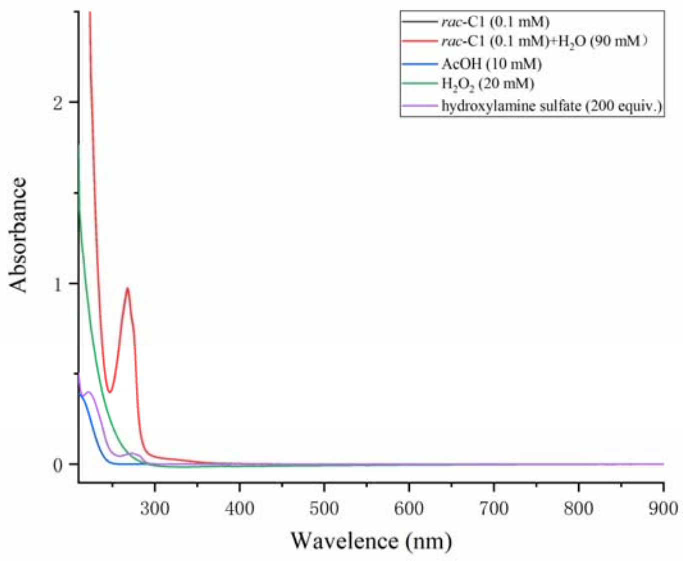

Figure S-34. UV-vis absorption spectrum of *rac*-**C1** (0.1 mM) in CH_3CN (black line), H_2O_2 (20 mM) in CH_3CN (red line), and hydroxylamine sulfate ($(NH_2OH)_2$•H_2SO_4 is only slightly soluble in CH_3CN) in CH_3CN (blue line). The added water has no effect on the absorption of *rac*-**C1**.

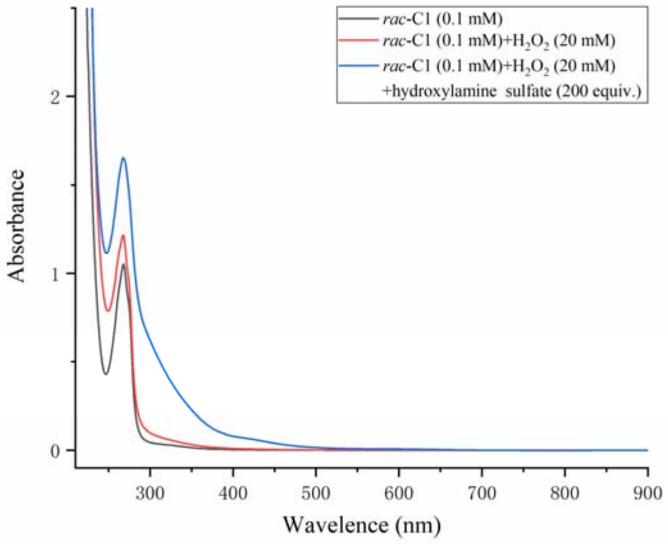

Figure S-35. UV-vis absorption spectra of *rac*-**C1** (0.1 mM) in CH_3CN (black line), *rac*-**C1** (0.1 mM) in the presence of H_2O_2 (20 mM) in CH_3CN (red line), and *rac*-**C1** (0.1 mM) in the presence of H_2O_2 (20 mM) and hydroxylamine sulfate in CH_3CN (blue line).

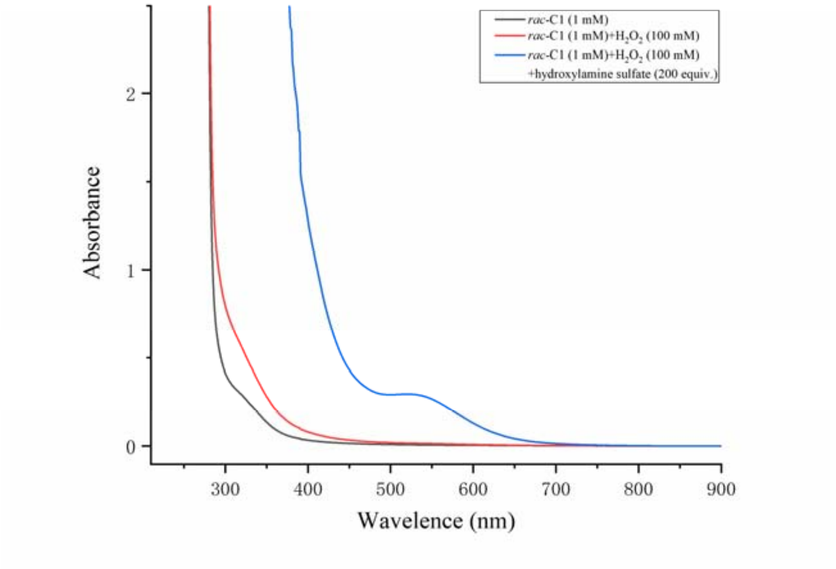

Figure S-36. UV-vis absorption spectra of *rac*-**C1** (1 mM) in CH_3CN (black line), *rac*-**C1** (1 mM) in the presence of H_2O_2 (100 mM) in CH_3CN (red line), and *rac*-**C1** (1 mM) in the presence of H_2O_2 (100 mM) and hydroxylamine sulfate in CH_3CN (blue line).

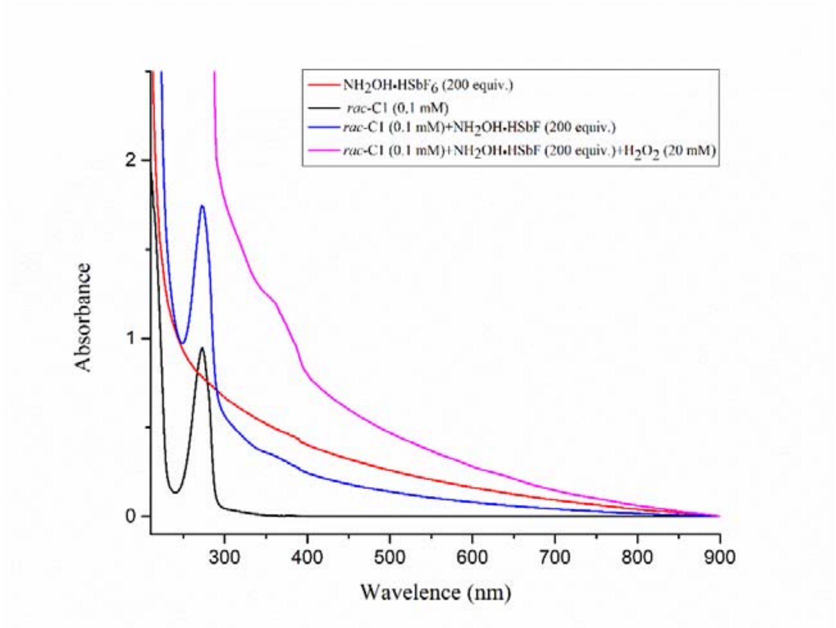

Figure S-37. UV-vis absorption spectra of NH_2OH•$HSbF_6$ in CH_3CN (partially soluble, cloudy solution; red line), *rac*-**C1** (0.1 mM) in CH_3CN (black line), *rac*-**C1** (0.1 mM) in the presence of NH_2OH•$HSbF_6$ in CH_3CN (blue line), and *rac*-**C1** (0.1 mM) in the presence of H_2O_2 (20 mM) and NH_2OH•$HSbF_6$ in CH_3CN (purple line).

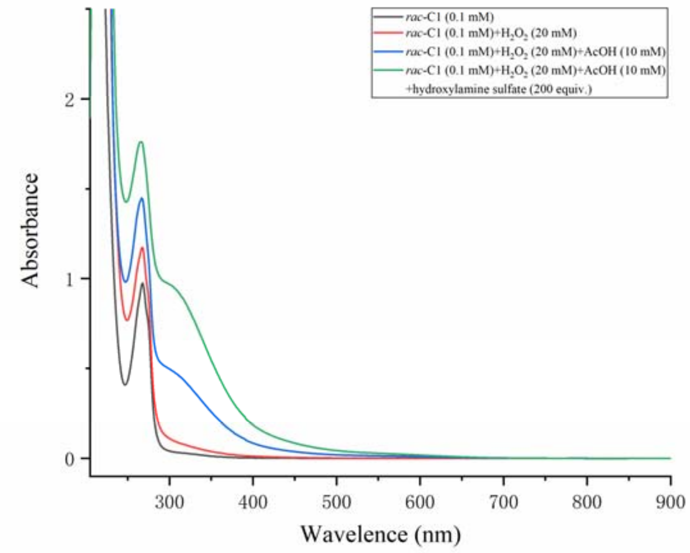

Figure S-38. UV-vis absorption spectra of *rac*-**C1** (0.1 mM) in CH_3CN (black line), *rac*-**C1** (0.1 mM) in the presence of H_2O_2 (20 mM) in CH_3CN (red line), *rac*-**C1** (0.1 mM) in the presence of H_2O_2 (20 mM) and AcOH (10 Mm) in CH_3CN (blue line), and *rac*-**C1** (0.1 mM) in the presence of H_2O_2 (20 mM), AcOH (10 Mm), and hydroxylamine sulfate in CH_3CN (green line).

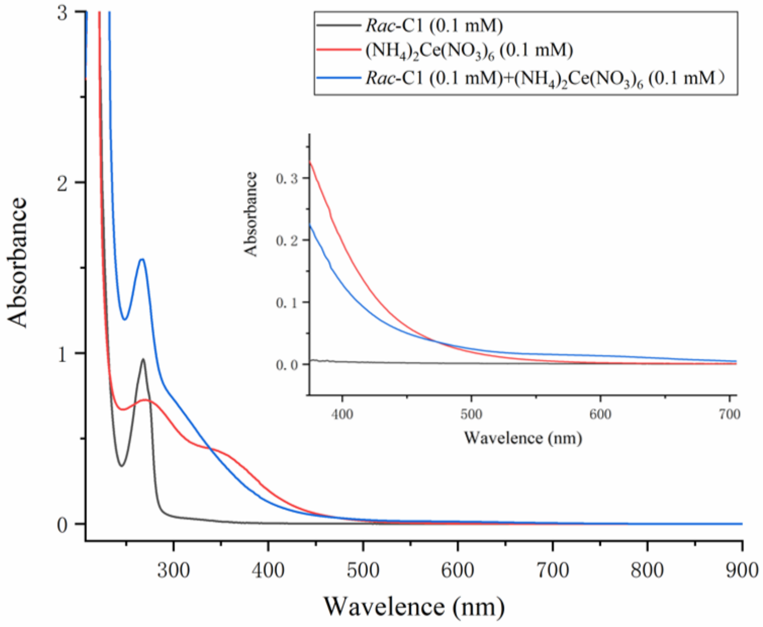

Figure S-39. UV-vis absorption spectra of *rac*-**C1** (0.1 mM) in CH_3CN (black line), $(NH_4)_2Ce(NO_3)_6$ (0.1 mM) in CH_3CN (red line), and *rac*-C1 (0.1 mM) in the presence of $(NH_4)_2Ce(NO_3)_6$ (0.1 mM) in CH_3CN (blue line).

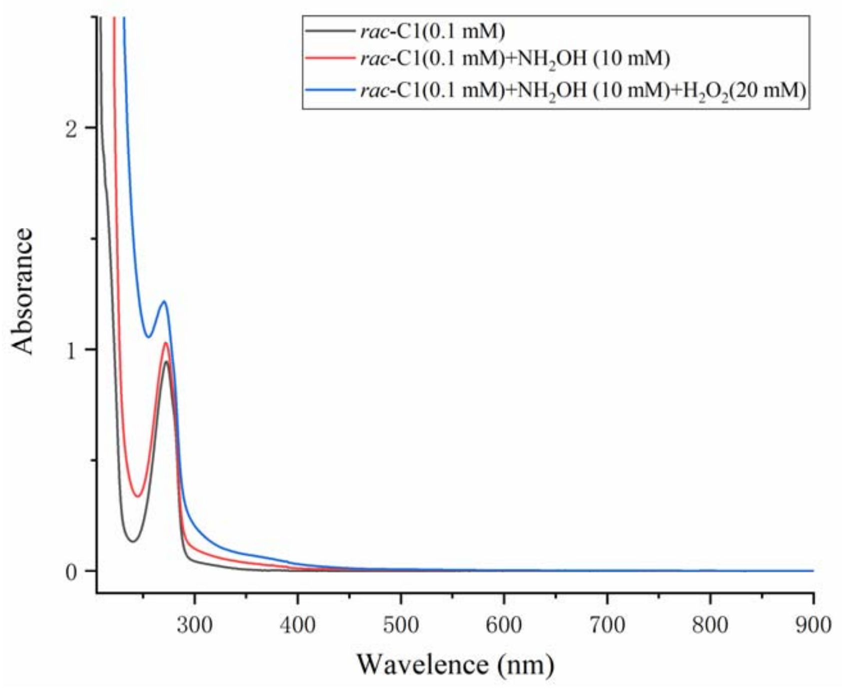

Figure S-40. UV-vis absorption spectra of *rac*-**C1** (0.1 mM) in CH_3CN (black line), *rac*-**C1** (0.1 mM) in the presence of NH_2OH (10 mM) in CH_3CN (red line), and *rac*-**C1** (0.1 mM) in the presence of H_2O_2 (20 mM) and NH_2OH (10 mM) in CH_3CN (blue line).

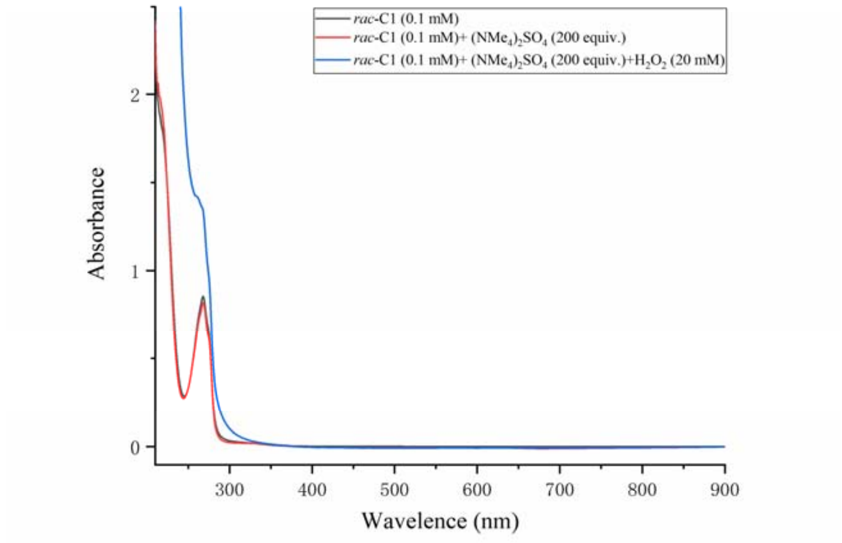

Figure S-41. UV-vis absorption spectra of *rac*-**C1** (0.1 mM) in CH_3CN (black line), *rac*-**C1** (0.1 mM) in the presence of $(NMe_4)_2SO_4$ in CH_3CN (red line), and *rac*-**C1** (1 mM) in the presence of H_2O_2 (20 mM) and $(NMe_4)_2SO_4$ in CH_3CN (blue line).

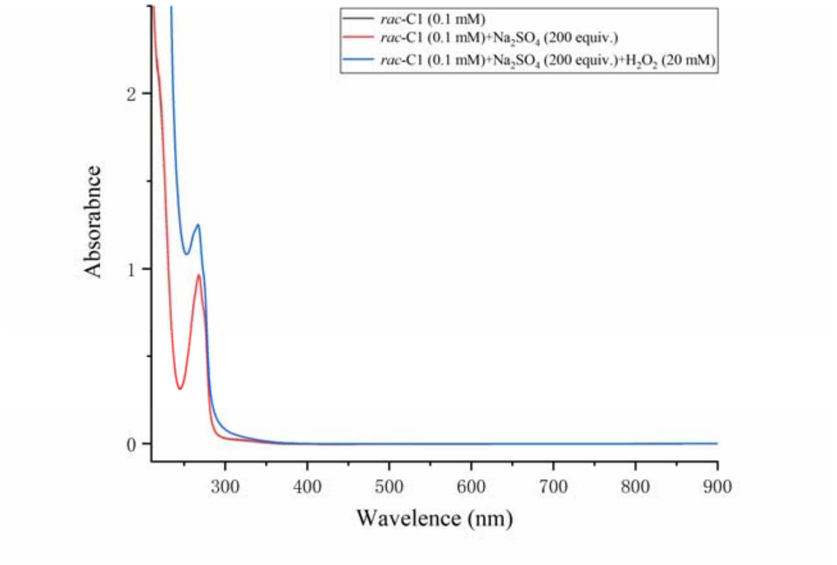

Figure S-42. UV-vis absorption spectra of *rac*-**C1** (0.1 mM) in CH_3CN (black line), *rac*-**C1** (0.1 mM) in the presence of Na_2SO_4 in CH_3CN (red line), and *rac*-**C1** (0.1 mM) in the presence of H_2O_2 (20 mM) and Na_2SO_4 in CH_3CN (blue line).

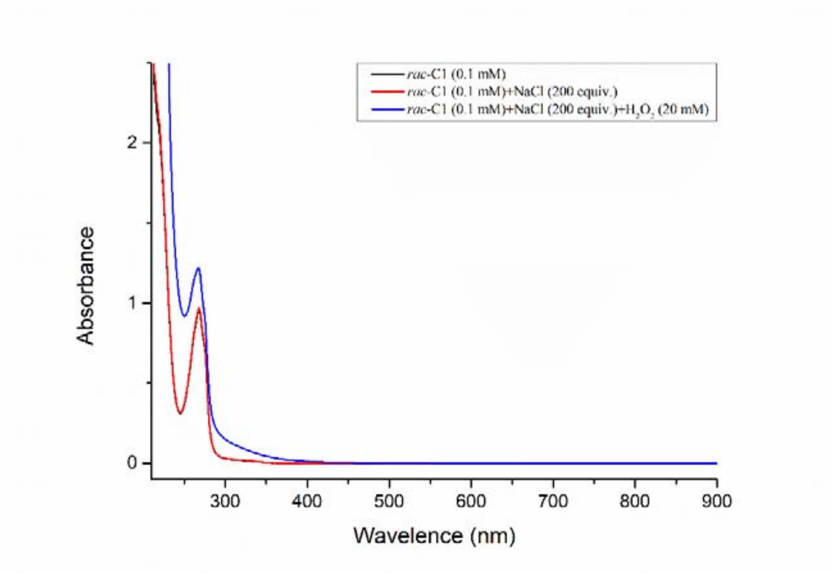

Figure S-43. UV-vis absorption spectra of *rac*-**C1** (0.1 mM) in CH_3CN (black line), *rac*-**C1** (0.1 mM) in the presence of NaCl in CH_3CN (red line), and *rac*-**C1** (0.1 mM) in the presence of H_2O_2 (20 mM) and NaCl in CH_3CN (blue line).

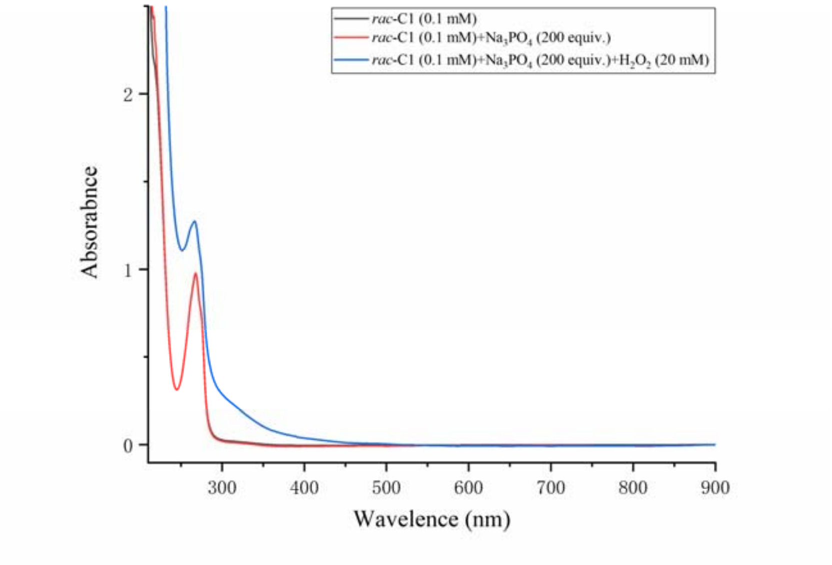

Figure S-44. UV-vis absorption spectra of *rac*-**C1** (0.1 mM) in CH_3CN (black line), *rac*-**C1** (0.1 mM) in the presence of Na_3PO_4 in CH_3CN (red line), and *rac*-**C1** (0.1 mM) in the presence of H_2O_2 (20 mM) and Na_3PO_4 in CH_3CN (blue line).

By comparing with the literature data and figures,[65-68] the UV-vis measurements above indicate that the oxidation of ***rac*-1** with H_2O_2 to higher oxidation states, e.g. (***rac*-L**^{1})Mn(III) and/or (***rac*-L**^{1})Mn(III)(μ-O)$_2$Mn(IV)((***rac*-L**^{1}), is accelerated by hydroxylamine sulfate, but inhibited by NH_2OH, Cl^-, SO_4^{2-} and PO_4^{3-}. However, the data are insufficient for us to determine the high oxidation states of manganese.

6.14. Proposed mechanism for manganese catalyzed oximation of hydrocarbons

Based on the mechanistic study above and the literature, both acetic acid and hydroxylammonium ion could be involved in the manganese-catalyzed oxidation, presumably independent of each other in catalytic turnover. The acetic acid-assisted mechanism (starting from species **A'** below) has been widely documented and will not be repeated here [56, 68-72]. Below we suggest a simplified mechanistic pathway for the oxidative oximation of a hydrocarbon involving hydroxylammonium. Note that the hydrogen bonding of hydroxylammonium with the hydroperoxyl intermediate is by

analogy with that involving a carboxylic acid (**A'**) or water, and although the hydrogen bonding with Mn(V)=O is highly speculative, there is ample evidence of high valent metal oxo complexes engaged in hydrogen bonding with hydrogen bond donors.[73]

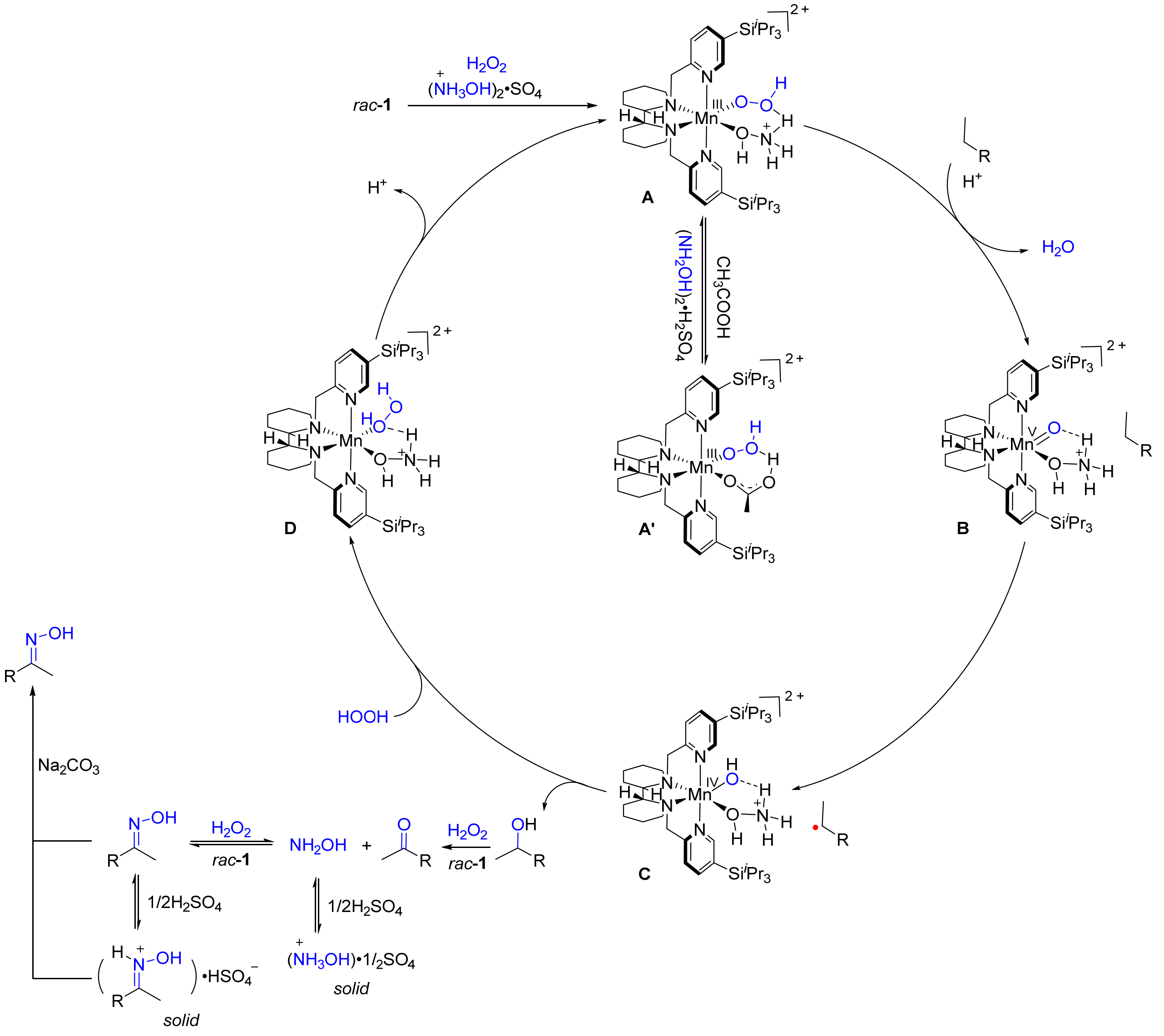

Figure S-45. Proposed mechanism for manganese catalyzed oximation of hydrocarbons

7. Synthesis and characterization of substrates

7.1 General procedure for synthesis of 13, 16, 20, 41, 47–48, 83 [51]

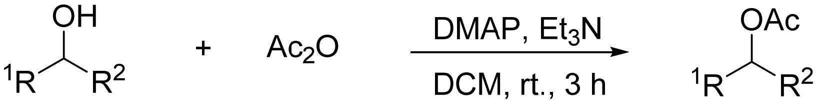

To a solution of an alcohols (10.0 mmol, 1.0 equiv.) and 4-dimethylpyridine (DMAP, 122.2 mg, 1.0 mmol, 10 mol %) in DCM (30.0 mL), acetic anhydride (12.0 mmol, 1.13 mL, 1.2 equiv.) was slowly added at room temperature with stirring. After 30 minutes, Et_3N (1.4 mL, 10.0 mmol, 1 equiv.) was added and then the solution stirred for 3 hours. After the reaction was complete, saturated sodium bicarbonate solution was added to remove the excess acetic anhydride. After addition of DCM (20.0 mL) and H_2O (20.0 mL), the reaction mixture was extracted with DCM (3×20.0 mL). The combined organic layers were washed with brine and dried over Na_2SO_4. After concentration, the residue was purified by flash column chromatography on silica gel (gradient elution: Petroleum ether /EtOAc = 100:1–20:1) to give **13, 16, 20, 41, 47–48, 83.**

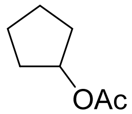

Cyclopentyl acetate (13) [74] was synthesized according to procedure **7.1** as a colorless liquid (1.21 g, 95% yield). **^{1}H NMR** (400 MHz, $CDCl_3$) δ (ppm): 5.18–5.11 (m, 1H), 2.01 (s, 3H), 1.90–1.81 (m, 2H), 1.75–1.62(m, 4H), 1.61–1.55 (m, 2H); **^{13}C NMR** (101 MHz, $CDCl_3$) δ (ppm): 171.1, 77.1, 32.7, 23.8, 21.5.

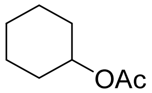

Cyclohexyl acetate (16) [75] was synthesized according to procedure **7.1** as a colorless liquid (1.34 g, 94% yield). **^{1}H NMR** (400 MHz, $CDCl_3$) δ (ppm): 4.78–4.68 (m, 1H), 2.03 (s, 3H), 1.88–1.80 (m, 2H), 1.75–1.68 (m, 2H), 1.58–1.51 (m, 1H), 1.45–1.29 (m, 4H), 1.29–1.19 (m, 1H); **^{13}C NMR** (101 MHz, $CDCl_3$) δ (ppm): 170.7, 72.8, 31.8, 25.5, 23.9, 21.6.

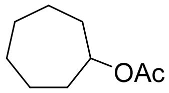

Cycloheptyl acetate (20) [75] was synthesized according to procedure **7.1** as a colorless liquid (1.50 g, 96% yield). **^{1}H NMR** (400 MHz, $CDCl_3$) δ (ppm): 4.98–4.83 (m, 1H), 2.02 (s, 3H), 1.94–1.84 (m, 2H), 1.69–1.59 (m, 4H), 1.58–1.53 (m, 4H), 1.50–1.38 (m, 2H); **^{13}C NMR** (101 MHz, $CDCl_3$) δ (ppm): 170.6, 75.3, 33.9, 28.4, 23.0, 21.6.

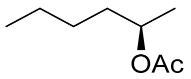

(*R*)-Hexan-2-yl acetate (41) [76] was synthesized according to procedure **7.1** as a colorless liquid (1.37 g, 95% yield). **^{1}H NMR** (400 MHz, $CDCl_3$) δ (ppm): 4.90–4.81 (m, 1H), 1.99 (s, 3H), 1.60−1.50 (m, 1H), 1.48−1.38 (m, 1H), 1.32−1.21 (m, 4H), 1.16 (d, *J* = 6.0 Hz, 3H), 0.86 (t, *J* = 6.8 Hz, 3H); **^{13}C NMR** (101 MHz, $CDCl_3$) δ (ppm): 170.9, 71.1, 35.7, 27.7, 22.6, 21.4, 20.0, 14.1.

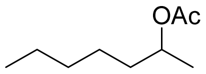

Heptan-2-yl acetate (47) [75] was synthesized according to procedure **7.1** as a colorless liquid (1.52 g, 96% yield). **^{1}H NMR** (400 MHz, $CDCl_3$) δ (ppm): 4.93–4.81 (m, 1H), 2.01 (s, 3H), 1.62−1.51 (m, 1H), 1.49−1.40 (m, 1H), 1.35−1.22 (m, 6H), 1.19 (d, *J* = 6.0 Hz, 3H), 0.87 (t, *J* = 6.8 Hz, 3H); **^{13}C NMR** (101 MHz, $CDCl_3$) δ (ppm): 170.9, 71.2, 36.0, 31.8, 25.2, 22.7, 21.5, 20.1, 14.1.

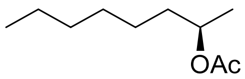

(*R*)-Octan-2-yl acetate (48) [77] was synthesized according to procedure **7.1** as a colorless liquid (1.63 g, 95% yield). **^{1}H NMR** (400 MHz, $CDCl_3$) δ (ppm): 4.92–4.81 (m, 1H), 2.00 (s, 3H), 1.61−1.51 (m, 1H), 1.49−1.39 (m, 1H), 1.30−1.21 (m, 8H), 1.18 (d, *J* = 6.4 Hz, 3H), 0.86 (t, *J* = 6.8 Hz, 3H); **^{13}C NMR** (101 MHz, $CDCl_3$) δ (ppm): 170.9, 71.2, 36.1, 31.9, 29.2, 25.5, 22.7, 21.5, 20.1, 14.2.

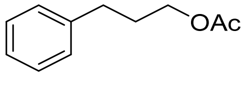

3-Phenylpropyl acetate (83) [78] was synthesized according to procedure **7.1** as a colorless liquid (1.73 g, 97% yield). **^{1}H NMR** (400 MHz, $CDCl_3$) δ (ppm): 7.30–7.24 (m, 2H), 7.21−7.15 (m, 3H), 4.08 (t, *J* = 6.4 Hz, 2H), 2.69 (t, *J* = 7.6 Hz, 2H), 2.05 (s, 3H), 2.00−1.87 (quint, *J* = 7.2 Hz, 2H); **^{13}C NMR** (101 MHz, $CDCl_3$) δ (ppm): 171.3, 141.3, 128.6, 128.5, 126.1, 64.0, 32.3, 30.3, 21.1.

7.2 Synthesis of 23–24 [51]

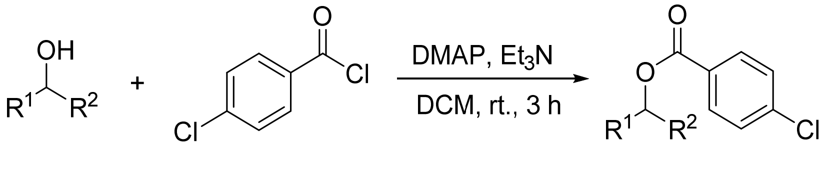

To a solution of alcohols (10 mmol, 1 equiv.) and 4-dimethylpyridine (DMAP, 122.2 mg, 1.0 mmol, 10 mol%) in DCM (30.0 mL), 4-chlorobenzoyl chloride (12.0 mmol, 2.1 g, 1.2 equiv.) was slowly added at room temperature with stirring. After 30 minutes, Et_3N (1.4 mL, 10 mmol, 1.0 equiv.) was added and then stirred for 3 hours. After the reaction was complete, sodium saturated bicarbonate solution was added to remove the excess 4-chlorobenzoyl chloride. After addition of DCM (20.0 mL) and H_2O (20.0 mL), the reaction mixture was extracted with DCM (3×20.0 mL). The combined organic layers were washed with brine and dried over Na_2SO_4. After concentration, the residue was purified by flash column chromatography on silica gel (gradient elution: Petroleum ether/EtOAc = 101:1–20:1) to give **23–24**.

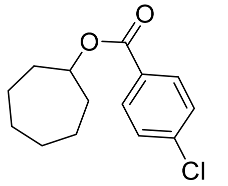

Cycloheptyl 4-chlorobenzoate (23) [51] was synthesized according to procedure **7.2** as a colorless liquid (2.30 g, 91% yield). **^{1}H NMR** (400 MHz, $CDCl_3$) δ (ppm): 7.97 (d, *J* = 8.4 Hz, 2H), 7.40 (d, *J* = 8.8 Hz, 2H), 5.23–5.14 (m, 1H), 2.06–1.96 (m, 2H), 1.86–1.78 (m, 2H), 1.76–1.67 (m, 2H), 1.64–1.59 (m, 4H), 1.58–1.47 (m, 2H); **^{13}C NMR** (101 MHz, $CDCl_3$) δ (ppm): 165.2, 139.2, 131.1, 129.7, 128.7, 76.2, 34.0, 28.5, 23.1.

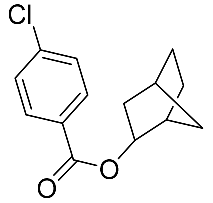

***endo/exo*-Bicyclo[2.2.1]heptan-2-yl 4-chlorobenzoate (24)** [79] was synthesized according to procedure **7.2** as a colorless liquid (2.26 g, 90% yield, *endo*/*exo* = 9:1). **^{1}H NMR** (400 MHz, $CDCl_3$) δ (ppm): 8.08–7.88 (m, 2H), 7.54–7.31 (m, 2H), 5.22–5.14 (m, 0.9H, *endo*), 4.84 (d, *J* = 7.2 Hz, 0.1H, *exo*), 2.63–2.57 (m, 0.9 H, *endo*), 2.43 (d, *J* = 4.8 Hz, 0.1H, *exo*), 2.36–2.32 (m, 0.1H, *exo*), 2.30–2.25 (m, 0.9H, *endo*), 2.16–2.06 (m, 1H), 1.91–1.79 (m, 1H), 1.68–1.60 (m, 1H), 1.58–1.42 (m, 2H), 1.42–1.32 (m, 2H), 1.17–1.09 (m, 1H); **^{13}C NMR** (101 MHz, $CDCl_3$) δ (ppm): 165.8 (*endo*), 165.4 (*exo*), 139.3 (*endo*), 139.2 (*exo*), 131.0 (*endo*), 129.8 (*exo*), 129.4 (*exo*), 129.3 (*endo*), 128.73 (*endo*), 128.67 (*exo*), 78.5 (*exo*), 76.6 (*endo*), 41.7 (*exo*), 40.6 (*endo*), 39.7 (*exo*), 37.5

(*endo*), 37.2 (*endo*), 36.7 (*endo*), 35.5 (*exo*), 35.2 (*exo*), 29.5(*endo*), 28.3 (*exo*), 24.4 (*exo*), 21.3 (*endo*).

7.3 Synthesis of 15, 21, 32–34, 45, 86–94 [80]

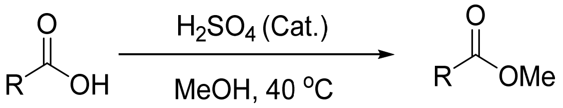

To a solution of a carboxylic acid (10.0 mmol) in a mixture of DCM (40.0 mL) and MeOH (5.0 mL) at 0 °C, H_2SO_4 (100.0 mg, 1.0 mmol, 98 wt %) was added dropwise under stirring over 60 s. Then, the reaction mixture was stirred for 4–18 hours at 40 °C. After addition of DCM (40.0 mL) and H_2O (20.0 mL), the reaction mixture was extracted with DCM (3×20.0 mL). The combined organic layers were then dried over Na_2SO_4, and filtered. After concentration, the residue was purified by flash column chromatography on silica gel (gradient elution: petroleum ether/EtOAc = 200:1-50:1) to give **15, 21, 32–34, 45, 86–94.**

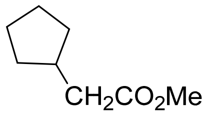

Methyl 2-cyclopentylacetate (15) was synthesized according to procedure **7.3** as a colorless liquid (1.31 g, 92% yield). **^{1}H NMR** (400 MHz, $CDCl_3$) δ (ppm): 3.66 (s, 3H), 2.31 (d, J = 7.2 Hz, 2H), 2.28–2.15 (m, 1H), 1.88–1.75 (m, 2H), 1.67–1.49 (m, 4H), 1.21–1.08 (m, 2H); **^{13}C NMR** (101 MHz, $CDCl_3$) δ (ppm): 174.0, 51.5, 40.3, 36.6, 32.6, 25.1.

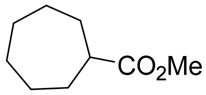

Methyl cycloheptanecarboxylate (21) [81] was synthesized according to procedure **7.3** as a colorless liquid (1.42 g, 91% yield). **^{1}H NMR** (400 MHz, $CDCl_3$) δ (ppm): 3.65 (s, 3H), 2.54–2.40 (m, 1H), 1.97–1.85 (m, 2H), 1.76–1.63 (m, 4H), 1.59–1.41 (m, 6H); **^{13}C NMR** (101 MHz, $CDCl_3$) δ (ppm): 177.7, 51.6, 45.1, 31.0, 28.4, 26.5.

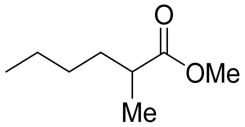

Methyl 2-methylhexanoate (32) [81] was synthesized according to procedure **7.3** as a colorless liquid (1.38 g, 96% yield). **^{1}H NMR** (400 MHz, $CDCl_3$) δ (ppm): 3.64 (s, 3H), 2.40 (sext, J = 7.2

Hz, 1H), 1.68–1.57 (m, 1H), 1.43–1.33 (m, 1H), 1.31–1.90 (m, 4H), 1.11 (d, J = 6.8 Hz, 3H), 0.86 (t, J = 6.8 Hz, 3H); **^{13}C NMR** (101 MHz, $CDCl_3$) δ (ppm): 177.5, 51.5, 39.5, 33.6, 29.5, 22.7, 17.2, 14.0.

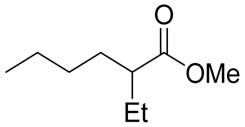

Methyl 2-ethylhexanoate (33) [82] was synthesized according to procedure **7.3** as a colorless liquid (1.49 g, 94% yield). **^{1}H NMR** (400 MHz, $CDCl_3$) δ (ppm): 3.65 (s, 3H), 2.30–2.21 (m, 1H), 1.66–1.38 (m, 4H), 1.32–1.17 (m, 4H), 0.86 (t, J = 7.6 Hz, 6H); **^{13}C NMR** (101 MHz, $CDCl_3$) δ (ppm): 177.0, 51.4, 47.4, 31.9, 29.8, 25.6, 22.8, 14.0, 12.0.

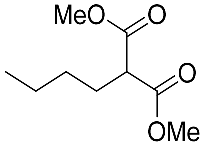

Dimethyl 2-butylmalonate (34) [83] was synthesized according to procedure **7.3** as a colorless liquid (1.79 g, 95% yield). **^{1}H NMR** (400 MHz, $CDCl_3$) δ (ppm): 3.69 (s, 6H), 3.31 (td, J = 7.6, 2.4 Hz, 1H), 1.90–1.81 (m, 2H), 1.33–1.19 (m, 4H), 0.87–0.82 (m, 3H); **^{13}C NMR** (101 MHz, $CDCl_3$) δ (ppm): 170.0, 52.4, 51.7, 29.5, 28.6, 22.3, 13.8.

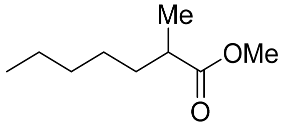

Methyl 2-methylheptanoate (45) [81] was synthesized according to procedure **7.3** as a colorless liquid (1.44 g, 91% yield). **^{1}H NMR** (400 MHz, $CDCl_3$) δ (ppm): 3.64 (s, 3H), 2.40 (sext, J = 7.2 Hz, 1H), 1.67–1.56 (m ,1H), 1.42–1.33 (m, 1H), 1.31–1.19 (m, 6H), 1.11 (d, J = 7.2 Hz, 3H), 0.85 (t, J = 6.8 Hz, 3H); **^{13}C NMR** (101 MHz, $CDCl_3$) δ (ppm): 177.5, 51.5, 39.6, 33.9, 31.8, 27.0, 22.6, 17.1, 14.1.

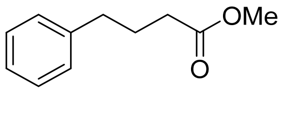

Methyl 4-phenylbutanoate (85) [84] was synthesized according to procedure **7.3** as a colorless liquid (1.64 g, 92% yield). **^{1}H NMR** (400 MHz, $CDCl_3$) δ (ppm): 7.33–7.27 (m, 2H), 7.23–7.17 (m, 3H), 3.67 (s, 3H), 2.66 (t, J = 7.6 Hz, 2H), 2.35 (t, J = 7.2 Hz, 2H), 1.97 (quint, J = 7.6 Hz,

2H); **^{13}C NMR** (101 MHz, $CDCl_3$) δ (ppm): 174.0, 141.5, 128.6, 128.5, 126.1, 51.6, 35.2, 33.5, 26.6.

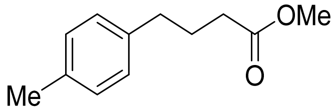

Methyl 4-(*p*-tolyl)butanoate (86) [84] was synthesized according to procedure **7.3** as a colorless liquid (1.73 g, 90% yield). **^{1}H NMR** (400 MHz, $CDCl_3$) δ (ppm): 7.14–7.07 (m, 4H), 3.68 (s, 3H), 2.63 (t, J = 7.6 Hz, 2H), 2.35 (t, J = 7.6 Hz, 2H), 2.34 (s, 3H),1.96 (quint, J = 7.6 Hz, 2H); **^{13}C NMR** (101 MHz, $CDCl_3$) δ (ppm): 174.1, 138.4, 135.5, 129.2, 128.5, 51.6, 34.8, 33.5, 26.7, 21.1.

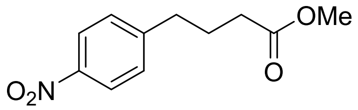

Methyl 4-(4-nitrophenyl)butanoate (87) [85] was synthesized according to procedure **7.3** as a colorless liquid (2.1 g, 94% yield). **^{1}H NMR** (400 MHz, $CDCl_3$) δ (ppm): 8.15 (d, J = 8.4 Hz, 2H), 7.34 (d, J = 8.8 Hz, 2H), 3.68 (s, 3H), 2.76 (t, J = 7.6 Hz, 2H), 2.35 (t, J = 7.6 Hz, 2H), 1.99 (quint, J = 7.6 Hz, 2H); **^{13}C NMR** (101 MHz, $CDCl_3$) δ (ppm): 173.5, 149.4, 146.7, 129.4, 123.8, 51.8, 35.1, 33.3, 26.1.

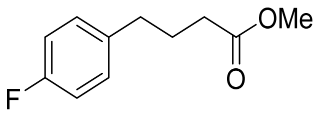

Methyl 4-(4-fluorophenyl)butanoate (88) [84] was synthesized according to procedure **7.3** as a colorless liquid (1.86 g, 95% yield). **^{1}H NMR** (400 MHz, $CDCl_3$) δ (ppm): 7.15–7.10 (m, 2H), 6.99–6.93 (m, 2H), 3.66 (s, 3H), 2.62 (t, J = 7.6 Hz, 2H), 2.32 (t, J = 7.2 Hz, 2H), 1.93 (quint, J = 7.6 Hz, 2H); **^{13}C NMR** (101 MHz, $CDCl_3$) δ (ppm): 174.0, 162.5 (d, $J_{C\text{-}F}$= 242.0 Hz), 137.1 (d, $J_{C\text{-}F}$= 3.0 Hz, 129.9 (d, $J_{C\text{-}F}$ = 8.0 Hz), 115.2 (d, $J_{C\text{-}F}$ = 21.0 Hz), 51.7, 34.4, 33.4, 26.7.

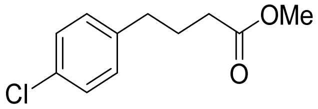

Methyl 4-(4-chlorophenyl)butanoate (89) [84] was synthesized according to procedure **7.3** as a colorless liquid (2.0 g, 94% yield). **^{1}H NMR** (400 MHz, $CDCl_3$) δ (ppm): 7.27–7.22 (m, 2H), 7.13–7.08 (m, 2H), 3.66 (s, 3H), 2.62 (t, J = 7.6 Hz, 2H), 2.32 (t, J = 7.6 Hz, 2H), 1.93 (quint, J =

7.6 Hz, 2H); **^{13}C NMR** (101 MHz, $CDCl_3$) δ (ppm): 173.9, 139.9, 131.9, 130.0, 128.6, 51.7, 34.6, 33.4, 26.5.

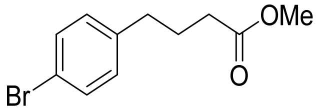

Methyl 4-(4-bromophenyl)butanoate (90) was synthesized according to procedure **7.3** as a colorless liquid (2.36 g, 92% yield). **^{1}H NMR** (400 MHz, $CDCl_3$) δ (ppm): 7.42−7.37 (m, 2H), 7.07−7.03 (m, 2H), 3.66 (s, 3H), 2.60 (t, *J* = 7.6 Hz, 2H), 2.31 (t, *J* = 7.6 Hz, 2H), 1.93 (quint, *J* = 7.6 Hz, 2H); **^{13}C NMR** (101 MHz, $CDCl_3$) δ (ppm): 173.8, 140.4, 131.6, 130.4, 119.9, 51.7, 34.6, 33.3, 26.4.

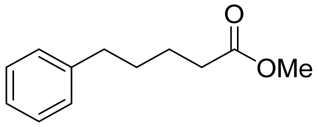

Methyl 5-phenylpentanoate (91) [86] was synthesized according to procedure **7.3** as a colorless liquid (1.79 g, 93% yield). **^{1}H NMR** (400 MHz, $CDCl_3$) δ (ppm): 7.31−7.26 (m, 2H), 7.22−7.16 (m, 3H), 3.67 (s, 3H), 2.64 (t, *J* = 7.2 Hz, 2H), 2.35 (t, *J* = 7.2 Hz, 2H), 1.74−1.60 (m, 4H); **^{13}C NMR** (101 MHz, $CDCl_3$) δ (ppm): 174.2, 142.2, 128.5, 128.4, 125.9, 51.6, 35.7, 34.1, 31.0, 24.7.

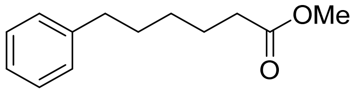

Methyl 6-phenylhexanoate (92) [87] was synthesized according to procedure **7.3** as a colorless liquid (1.88 g, 91% yield). **^{1}H NMR** (400 MHz, $CDCl_3$) δ (ppm): 7.32−7.27 (m, 2H), 7.21−7.17 (m, 3H), 3.68 (s, 3H), 2.63 (t, *J* = 7.6 Hz, 2H), 2.32 (t, *J* = 7.6 Hz, 2H), 1.73−1.61 (m, 4H), 1.43−1.33 (m, 2H); **^{13}C NMR** (101 MHz, $CDCl_3$) δ (ppm): 174.3, 142.6, 128.5, 128.4, 125.8, 51.6, 35.8, 34.1, 31.2, 28.9, 24.9.

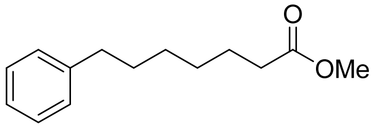

Methyl 7-phenylheptanoate (93) [88] was synthesized according to procedure **7.3** as a colorless liquid (1.98 g, 90% yield). **^{1}H NMR** (400 MHz, $CDCl_3$) δ (ppm): 7.29−7.23 (m, 2H), 7.19−7.14 (m, 3H), 3.65 (s, 3H), 2.59 (t, *J* = 7.6 Hz, 2H), 2.29 (t, *J* = 7.6 Hz, 2H), 1.66−1.57 (m, 4H),

1.39–1.29 (m, 4H); **^{13}C NMR** (101 MHz, $CDCl_3$) δ (ppm): 174.4, 142.8, 128.5, 128.4, 125.7, 51.6, 36.0, 34.2, 31.4, 29.1, 29.0, 25.0.

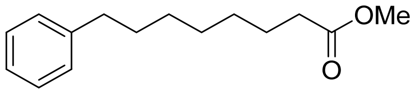

Methyl 8-phenyloctanoate (94) was synthesized according to procedure **7.3** as a colorless liquid (2.09 g, 89% yield). **^{1}H NMR** (400 MHz, $CDCl_3$) δ (ppm): 7.29–7.24 (m, 2H), 7.19–7.14 (m, 3H), 3.66 (s, 3H), 2.59 (t, J = 7.6 Hz, 2H), 2.29 (t, J = 7.6 Hz, 2H), 1.66–1.56 (m, 4H), 1.36–1.28 (m, 6H); **^{13}C NMR** (101 MHz, $CDCl_3$) δ (ppm): 174.4, 142.9, 128.5, 128.3, 125.7, 51.5, 36.0, 34.2, 31.5, 29.22, 29.20, 29.17, 25.0.

7.4 Synthesis of 19, 22, 37 [89]

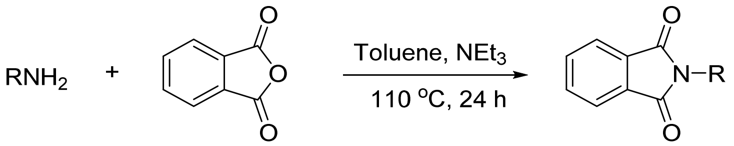

RNH_2 = Pentan-1-amine, Cyclohexanamine, Cycloheptanamine

A round flask was charged with phthalic anhydride (1.48 g, 10 mmol), primary amine (10 mmol), triethylamine (2.0 mL) and toluene (50.0 mL). The reaction mixture was heated to 110 °C and stirred for 24 hours. Then, the crude mixture was added water and extracted with DCM (2×20.0 mL) twice. The combined organic phase was then washed with water and brine, dried over anhydrous Na_2SO_4, filtered and concentrated. the residue was purified by flash column chromatography (petroleum ether /EtOAc=4:1) to give the desired product **19, 22, 37.**

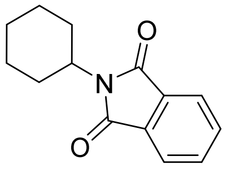

2-Cyclohexylisoindoline-1,3-dione (19) [90]**:** was synthesized according to procedure **7.4** as a white solid (1.88 g, 82% yield); **^{1}H NMR** (400 MHz, $CDCl_3$) δ (ppm): 7.81–7.75 (m, 2H), 7.70–7.63 (m, 2H), 4.13–4.03 (m, 1H), 2.24–2.12 (m, 2H), 1.88–1.80 (m, 2H), 1.74–1.63 (m, 3H), 1.42–1.20 (m, 3H); **^{13}C NMR** (101 MHz, $CDCl_3$) δ (ppm): 168.5, 133.8, 132.2, 123.1, 51.0, 30.0, 26.1, 25.2.

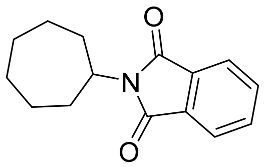

2-Cycloheptylisoindoline-1,3-dione (22) [91]**:** was synthesized according to procedure **7.4** as a white solid (2.12 g, 87% yield); **^{1}H NMR** (400 MHz, $CDCl_3$) δ (ppm): 7.83–7.78 (m, 2H), 7.70–7.65 (m, 2H), 4.30–4.21 (m, 1H), 2.31–2.20 (m, 2H), 1.86–1.75 (m, 4H), 1.69–1.57 (m, 4H), 1.57–1.45 (m, 2H); **^{13}C NMR** (101 MHz, $CDCl_3$) δ (ppm): 168.4, 133.9, 132.3, 123.1, 52.9, 32.8, 27.7, 25.6.

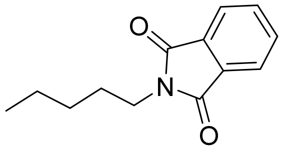

2-Pentylisoindoline-1,3-dione (37) [90]**:** was synthesized according to procedure **7.4** as a white solid (1.85 g, 85% yield); **^{1}H NMR** (400 MHz, $CDCl_3$) δ (ppm): 7.80–7.73 (m, 2H), 7.68–7.61 (m, 2H), 3.66–3.57 (m, 2H), 1.67–1.56 (m, 2H), 1.34–1.22 (m, 4H), 0.87–0.79 (m, 3H); **^{13}C NMR** (101 MHz, $CDCl_3$) δ (ppm): 168.4, 133.8, 132.2, 123.1, 38.0, 29.0, 28.3, 22.3, 13.9.

7.5 Synthesis of (*R*)-*N*-(1-(4-bromophenyl)ethyl)pentan-1-amine (38) [92]

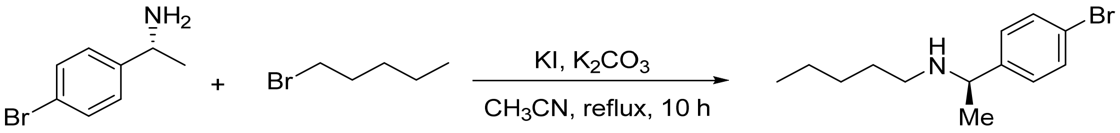

A solution of (*R*)-1-(4-bromophenyl)ethan-1-amine (2.4 g, 12.0 mmol, 1.2 equiv.), 1-bromopentane (1.51 g, 10.0 mmol, 1.0 equiv.), powdered K_2CO_3 (1.38 g, 10.0 mmol, 1.0 equiv.) and a catalytic amount of KI in acetonitrile (30.0 mL) was prepared. The reaction mixture was heated to reflux for 18 h, and then cooled to room temperature. K_2CO_3 was removed by filtration and the solvent was concentrated under vacuum, the residue was purified on silica gel chromatography (DCM/MeOH=101:1) to give the desired product **38** (2.48 g, 92% yield, colorless oil). **^{1}H NMR** (400 MHz, $CDCl_3$) δ (ppm): 7.45–7.41 (m, 2H), 7.21–7.17 (m, 2H), 3.71 (q, J = 6.8 Hz, 1H), 2.50–2.42 (m, 1H), 2.40–2.33 (m, 1H), 1.48–1.39 (m, 2H), 1.30 (d, J = 6.8 Hz, 3H), 1.29–1.21 (m, 4H), 0.87 (t, J = 7.2 Hz, 3H); **^{13}C NMR** (101 MHz, $CDCl_3$) δ (ppm): 145.2, 131.6, 128.5, 120.5, 58.0, 48.0, 30.1, 29.7, 24.6, 22.7, 14.2.

7.6 Synthesis of *N*-(4-bromobenzyl)-*N*-methylpentan-1-amine (39) [92]

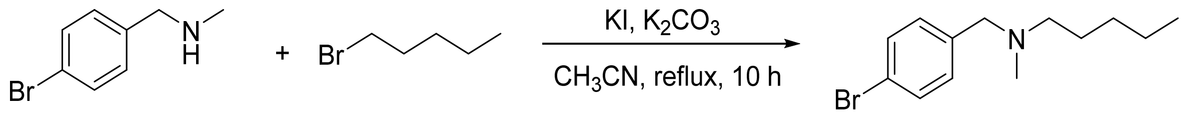

A solution of 1-(4-bromophenyl)-*N*-methylmethanamine (2.0 g, 10.0 mmol, 1.0 equiv.), 1-bromopentane (1.81 g, 12 mmol, 1.2 equiv.), powdered K_2CO_3 (1.38 g, 10.0 mmol, 1.0 equiv.) and a catalytic amount of KI in acetonitrile (30.0 mL) was prepared. The reaction mixture was heated to reflux for 1 h, and then cooled to room temperature. K_2CO_3 was removed by filtration and the solvent was concentrated under vacuum. The residue was purified on silica gel chromatography (DCM/MeOH=100:1) to give the desired product **39** [93] (2.50 g, 93% yield, colorless oil). **^{1}H NMR** (400 MHz, $CDCl_3$) δ (ppm): 7.45–7.41 (m, 2H), 7.21–7.17 (m, 2H), 3.41 (s, 2H), 2.33 (t, J = 7.2 Hz, 2H), 2.16 (s, 3H), 1.50 (quint, J = 7.6 Hz, 2H), 1.35–1.23 (m, 4H), 0.89 (t, J = 7.2 Hz, 3H); **^{13}C NMR** (101 MHz, $CDCl_3$) δ (ppm): 138.7, 131.4, 130.8, 120.7, 61.8, 57.7, 42.3, 29.8, 27.2. 22.8, 14.2.

7.7 Synthesis of *N*-(4-bromobenzyl)-*N*,2-dimethylbutan-1-amine (40) [94-95]

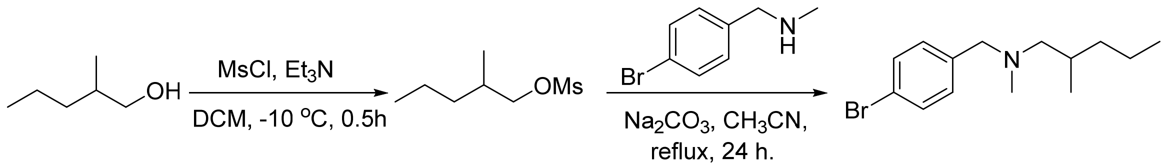

To a solution of 2-methylpentan-1-ol (817.0 mg, 8.0 mmol) and triethylamine (1.88 mL, 13.6 mmol) in dry DCM (20.0 mL) at -10 °C, methanesulfonyl chloride (0.74 mL, 9.6 mmol) was slowly added and the reaction mixture was stirred for 0.5 h. After the reaction was complete, saturated solution of $NaHCO_3$ (10.0 mL) and DCM (10.0 mL) were added to the reaction mixture. The organic layer was separated, and the aqueous layer was extracted with dichloromethane (3×15.0 mL) three times. The combined organic layers were washed with brine and dried over Na_2SO_4. After concentration, the crude 2-methylpentyl methanesulfonate was obtained without further purification.

Then, to a solution of 1-(4-bromophenyl)-*N*-methylmethanamine (1.20 g, 6 mmol) and powdered Na_2CO_3 (1.33 g, 12.5 mmol) in acetonitrile (20 mL) at room temperature, 2-methylpentyl methanesulfonate (0.90 g, 5.0 mmol) was added and the reaction mixture was heated to reflux for 24 h. After the reaction was complete, deionized water (10 mL) and DCM (10 mL)

were added to the reaction mixture. The organic layer was separated, and the aqueous layer was extracted with dichloromethane (3×15.0 mL) three times. The combined organic layers were washed with brine and dried over Na_2SO_4. After concentration, the residue was purified on silica gel chromatography (petroleum ether /EtOAc = 20:1) to give the desired product **40** (1.28 g, 90% yield). **^{1}H NMR** (400 MHz, $CDCl_3$) δ (ppm): 7.42 (d, J = 8.0 Hz, 2H), 7.20 (d, J = 8.0 Hz, 2H), 3.43 (q, J = 13.6 Hz, 1H), 3.35 (q, J = 13.6 Hz, 1H), 2.20–2.05 (m, 2H), 2.13 (s, 3H), 1.72−1.60 (m, 1H), 1.45−1.34 (m, 2H), 1.31−1.21 (m, 1H), 1.08–0.99 (m, 1H), 0.93–0.86 (m, 6H); **^{13}C NMR** (101 MHz, $CDCl_3$) δ (ppm): 139.1, 131.3, 130.7, 120.6, 64.9, 62.3, 42.8, 37.5, 31.0, 20.2, 18.3, 14.6.

7.8 Synthesis of heptan-2-one oxime (46) [96]

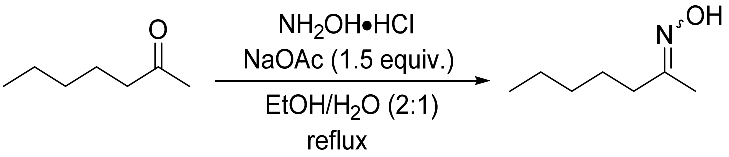

A solution of heptan-2-one (1.14 g, 10.0 mmol, 1.0 equiv.), sodium acetate (1.23 g, 15.0 mmol, 1.5 equiv.) and hydroxylamine hydrochloride (1.05 g, 15.0 mmol, 1.5 equiv.) in ethanol/water (20 mL/10mL) was prepared. The reaction mixture was heated to reflux for overnight, and then cooled to room temperature. The volatiles were removed by a rotary evaporator. To the crude mixture was added water and the mixture was extracted with ethyl acetate (2×20.0 mL) twice. The combined organic phase was then washed with water and brine, dried over anhydrous Na_2SO_4, filtered and concentrated. The oxime product was purified by flash column chromatography (petroleum ether /EtOAc = 10:1) to give the desired product **46** (1.18 g, 91% yield, *E/Z*= 2:1, colorless oil). **^{1}H NMR** (400 MHz, $CDCl_3$) δ (ppm): 9.68 (brs, 1H), 2.35 (t, J = 7.6 Hz, 0.67H, *Z*), 1.66 (t, J = 7.6 Hz, 1.37H, *E*), 1.86 (s, 2H, *E*), 1.85 (s, 1H, *Z*), 1.53−1.45 (m, 2H), 1.35−1.20 (m, 4H), 0.90–0.84 (m, 3H); **^{13}C NMR** (101 MHz, $CDCl_3$) δ (ppm): 159.1 (*Z*), 158.7 (*E*), 35.8, 32.0, 31.4, 28.7, 26.1, 25.2, 22.52, 22.47, 19.9, 14.0, 13.5.

7.9 Synthesis of methyl undec-2-enoate (54) [51]

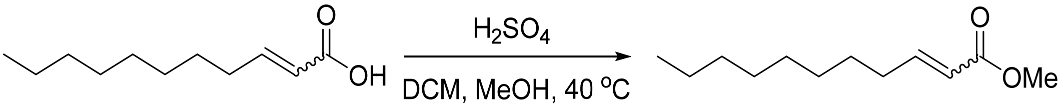

To a solution of (*Z*/*E*)-undec-2-enoic acid (921.4 mg, 5 mmol) in a mixture of DCM (20.0 mL) and methanol (3.0 mL) at 0 °C, H_2SO_4 (50 mg, 0.5 mmol, 98 wt%) was added dropwise over 60 s. Then, the reaction mixture was stirred for 4 hours at 40 °C. After addition of DCM (20.0 mL) and H_2O (10.0 mL), the reaction mixture was extracted with DCM (3×20.0 mL). The combined organic layers were then dried over Na_2SO_4, and filtered. After concentration, the residue was purified by flash column chromatography on silica gel (gradient elution: petroleum ether/ethyl acetate = 200:1-101:1) to give (*Z*/*E*)-**54** [97] (922.2 mg, 93% yield, *Z*/*E* = 1:3) as a colorless liquid. **^{1}H NMR** (400 MHz, $CDCl_3$) δ (ppm): 7.00–6.90 (m, 1H), 5.83–5.76 (m, 1H), 3.71 (s, 3H), 2.21–2.14 (m, 2H), 1.48–1.39 (m, 2H), 1.30–1.22 (m, 10H), 0.89–0.84 (m, 3H); **^{13}C NMR** (101 MHz, $CDCl_3$) δ (ppm): 167.3(*E*), 166.9(*Z*), 149.9(*E*), 149.6 (*Z*), 121.3(*Z*), 120.9(*E*), 60.2(*Z*), 51.5(*E*), 32.34, 32.0, 29.5, 29.31, 29.27, 28.1, 22.8, 14.4(*Z*), 14.2(*E*).

7.10 Synthesis of 58–60, 62 [51]

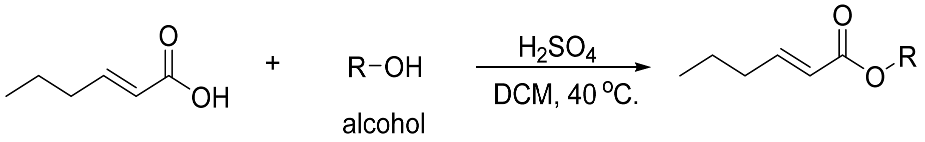

To a solution of (*E*)-hex-2-enoic acid (570.7 mg, 5 mmol) in a mixture of DCM (20.0 mL) and alcohol (3.0 mL) at 0 °C, H_2SO_4 (50.0 mg, 0.5 mmol, 98 wt%) was added dropwise under stirring over 60 s. Then, the reaction mixture was stirred for 4 hours at 40 °C. After addition of DCM (20.0 mL) and H_2O (10.0 mL), the reaction mixture was extracted with DCM (3×20.0 mL). The combined organic layers were then dried over Na_2SO_4, and filtered. After concentration, the residue was purified by flash column chromatography on silica gel (gradient elution: petroleum ether/EtOAc = 200:1-100:1) to give **58–60, 62**.

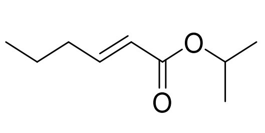

Isopropyl (*E*)-hex-2-enoate (58) [98] was synthesized according to procedure **7.10** as a colorless liquid (742.1 mg, 95% yield). **^{1}H NMR** (400 MHz, $CDCl_3$) δ (ppm): 6.93 (dt, *J* = 15.6, 6.8 Hz, 1H), 5.78 (dt, *J* = 16.0, 1.6 Hz, 1H), 5.04 (hept, *J* = 6.4 Hz, 1H), 2.19–2.12 (m, 2H), 1.47 (sext, *J* = 7.2 Hz, 2H), 1.25 (d, *J* = 6.4 Hz, 6H), 0.92 (t, *J* = 7.2 Hz, 3H); **^{13}C NMR** (101 MHz, $CDCl_3$) δ (ppm): 166.4, 149.0, 122.0, 67.5, 34.3, 22.0, 21.4, 13.8.

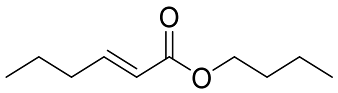

Butyl (*E*)-hex-2-enoate (59) was synthesized according to procedure **7.10** as a colorless liquid (672.9 mg, 79% yield). **^{1}H NMR** (400 MHz, $CDCl_3$) δ (ppm): 6.92 (dt, *J* = 15.6, 7.2 Hz, 1H), 5.78 (dt, *J* = 15.6, 1.6 Hz, 1H), 4.10 (t, *J* = 6.8 Hz, 2H), 2.18–2.10 (m, 2H), 1.65−1.56 (m, 2H), 1.46 (sext, *J* = 7.2 Hz, 2H), 1.41−1.31 (m, 2H), 0.94–0.88 (m, 6H); **^{13}C NMR** (101 MHz, $CDCl_3$) δ (ppm): 166.9, 149.1, 121.5, 64.1, 34.3, 30.9, 21.4, 19.3, 13.8, 13.7.

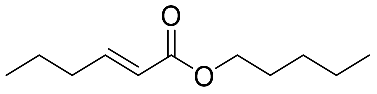

Pentyl (*E*)-hex-2-enoate (60) was synthesized according to procedure **7.10** as a colorless liquid (700.3 mg, 76% yield). **^{1}H NMR** (400 MHz, $CDCl_3$) δ (ppm): 6.93 (dt, *J* = 15.6, 7.2 Hz, 1H), 5.79 (dt, *J* = 15.6, 1.6 Hz, 1H), 4.09 (t, *J* = 6.8 Hz, 2H), 2.18–2.11 (m, 2H), 1.67−1.59 (m, 2H), 1.47 (sext, *J* = 7.2 Hz, 2H), 1.36−1.29 (m, 4H), 0.94–0.86 (m, 6H); **^{13}C NMR** (101 MHz, $CDCl_3$) δ (ppm): 166.9, 149.2, 121.5, 64.4, 34.3, 28.5, 28.2, 22.4, 21.4, 14.0, 13.7.

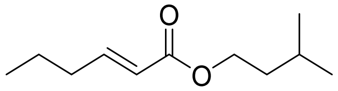

Isopentyl (*E*)-hex-2-enoate (62) was synthesized according to procedure **7.10** as a colorless liquid (737.1 mg, 80% yield). **^{1}H NMR** (400 MHz, $CDCl_3$) δ (ppm): 6.93 (dt, *J* = 15.6, 7.2 Hz, 1H), 5.79 (dt, *J* = 15.6, 1.6 Hz, 1H), 4.14 (t, *J* = 6.8 Hz, 2H), 2.19–2.12 (m, 2H), 1.69 (hept, *J* = 6.8 Hz, 1H), 1.56−1.43 (m, 4H), 0.94–0.88 (m, 9H); **^{13}C NMR** (101 MHz, $CDCl_3$) δ (ppm): 167.0, 149.2, 121.6, 63.0, 37.6, 34.3, 25.2, 22.6, 21.4, 13.8.

7.11 Synthesis of 67, 68, 115–119 [99]

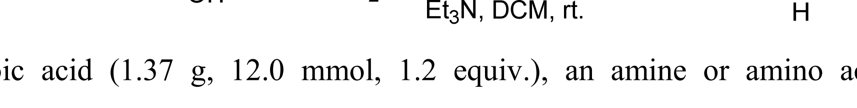

(*E*)-Hex-2-enoic acid (1.37 g, 12.0 mmol, 1.2 equiv.), an amine or amino acid derivative hydrochloride (10.0 mmol, 1.0 equiv.), 1-(3-Dimethylaminopropyl)-3-ethylcarbodiimide hydrochloride (EDCI, 2.76 g, 14.4 mmol, 1.44 equiv.), *N*,*N*-dimethylpyridin-4-amine (DMAP, 427.5 mg, 3.5 mmol, 0.35 equiv.), and DCM (30.0 mL) were added into a 100 mL round flask

equipped with a magnetic stirring bar. The reaction mixture was stirred rapidly and then NEt_3 (4.16 mL, 30 mmol, 3.0 equiv.) was added. After the reaction was complete, deionized water (20 mL) and DCM (20 mL) were added to the reaction mixture. The organic layer was separated, and the aqueous layer was extracted with dichloromethane (3×20.0 mL) three times. The combined organic layers were washed with brine and dried over Na_2SO_4. After concentration, the residue was purified through flash column chromatography (petroleum ether /EtOAc = 5:1 to 3:1) to give the desired product **67, 68, 115–119.**

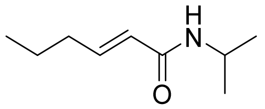

(*E*)-*N*-Isopropylhex-2-enamide (67) [99] was synthesized according to procedure **7.11** as a white solid (1.38 g, 89% yield). **^{1}H NMR** (400 MHz, $CDCl_3$) δ (ppm): 6.76 (dt, *J* = 15.6, 7.2 Hz, 1H), 5.74 (dt, *J* = 15.2, 1.6 Hz, 1H), 5.72 (brs, 1H), 4.17–4.04 (m, 1H), 2.13–2.06 (m, 2H), 1.42 (sext, *J* = 7.2 Hz, 2H), 1.13 (d, *J* = 6.4 Hz, 6H), 0.88 (t, *J* = 7.2 Hz, 3H); **^{13}C NMR** (101 MHz, $CDCl_3$) δ (ppm): 165.4, 144.1, 124.2, 41.3, 34.1, 22.8, 21.6, 13.8.

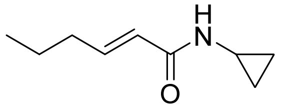

(*E*)-*N*-Cyclopropylhex-2-enamide (68) was synthesized according to procedure **7.11** as a white solid (1.39 g, 91% yield). **^{1}H NMR** (400 MHz, $CDCl_3$) δ (ppm): 6.83–6.75 (m, 1H), 6.12 (brs, 1H), 5.73 (d, *J* = 15.2 Hz, 1H), 2.77–2.72 (m, 1H), 2.13–2.07 (m, 2H), 1.47–1.39 (m, 2H), 0.93–0.86 (m, 3H), 0.78–0.71 (m, 2H), 0.53–0.48 (m, 2H); **^{13}C NMR** (101 MHz, $CDCl_3$) δ (ppm): 167.7, 144.4, 123.7, 34.1, 22.7, 21.6, 13.8, 6.6.

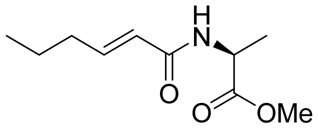

Methyl (*E*)-hex-2-enoyl-*L*-alaninate (115) was synthesized according to procedure **7.11** as a white solid (1.87 g, 94% yield). **^{1}H NMR** (400 MHz, $CDCl_3$) δ (ppm): 6.83 (dt, *J* = 15.6, 7.2 Hz, 1H), 6.19 (brs, 1H), 5.80 (dt, *J* = 15.6, 1.6 Hz, 1H), 4.65 (quint, *J* = 7.2 Hz, 1H), 3.73 (s, 3H), 2.17–2.09 (m, 2H), 1.45 (sext, *J* = 7.6 Hz 2H), 1.41 (d, *J* = 7.2 Hz 3H), 0.90 (t, *J* = 7.2 Hz, 3H); **^{13}C NMR** (101 MHz, $CDCl_3$) δ (ppm): 173.8, 165.5, 145.6, 123.3, 52.6, 48.0, 34.2, 21.5, 18.7,

13.8; Chiral HPLC trace (chiralpak AD-H column, *n*-hexane: isopropanol = 90:10; flow rate = 0.5 mL/min): t_{major} = 15.37 min., t_{minor} = 13.85 min.; >99% ee.

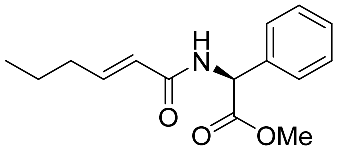

Methyl (*S,E*)-2-(hex-2-enamido)-2-phenylacetate (116) was synthesized according to procedure **7.11** as a white solid (2.35 g, 90% yield). **^{1}H NMR** (400 MHz, $CDCl_3$) δ (ppm): 7.39–7.28 (m, 5H), 6.86 (dt, *J* = 15.2, 6.8 Hz, 1H), 6.50 (brs, 1H), 5.84 (d, *J* = 15.2 Hz, 1H), 5.66 (d, *J* = 7.2 Hz, 1H), 3.73 (s, 3H), 2.18–2.11 (m, 2H), 1.46 (sext, *J* = 7.2 Hz, 2H), 0.91 (t, *J* = 7.2 Hz, 3H); **^{13}C NMR** (101 MHz, $CDCl_3$) δ (ppm): 171.7, 165.3, 146.1, 136.8, 129.1, 128.6, 127.4, 123.0, 56.5, 52.9, 34.2, 21.5, 13.8.

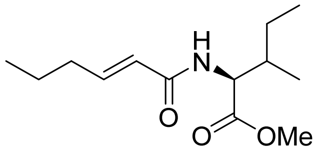

Methyl (2*S*)-2-((*E*)-hex-2-enamido)-3-methylpentanoate (117) was synthesized according to procedure **7.11** as a white solid (2.17 g, 90% yield). **^{1}H NMR** (400 MHz, $CDCl_3$) δ (ppm): 6.80 (dt, *J* = 15.2, 6.8 Hz, 1H), 6.23 (d, *J* = 8.8 Hz, 1H), 5.81 (dt, *J* = 15.2, 1.6 Hz, 1H), 4.63 (dd, *J* = 8.4, 5.2 Hz, 1H), 3.68 (s, 3H), 2.13–2.06 (m, 2H), 1.90–1.79 (m, 1H), 1.46–1.35 (m, 3H), 1.20–1.07 (m, 1H), 0.89–0.83 (m, 9H); **^{13}C NMR** (101 MHz, $CDCl_3$) δ (ppm): 172.8, 165.8, 145.4, 123.3, 56.3, 52.0, 38.1, 34.1, 25.3, 21.4, 15.4, 13.7, 11.5.

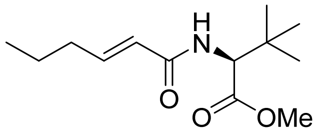

Methyl (*S, E*)-2-(hex-2-enamido)-3,3-dimethylbutanoate (118) was synthesized according to procedure **7.11** as a white solid (2.22 g, 92% yield). **^{1}H NMR** (400 MHz, $CDCl_3$) δ (ppm): 6.85 (dt, *J* = 15.2, 7.2 Hz, 1H), 5.99 (d, *J* = 8.4 Hz, 1H), 5.82 (d, *J* = 15.2 Hz, 1H), 4.56 (d, *J* = 9.6 Hz, 1H), 3.71 (s, 3H), 2.15 (q, *J* = 7.2 Hz, 2H), 1.47 (sext, *J* = 7.2 Hz, 2H), 0.97 (s, 9H), 0.92 (t, *J* = 7.2 Hz, 3H); **^{13}C NMR** (101 MHz, $CDCl_3$) δ (ppm): 172.5, 165.8, 145.8, 123.4, 59.9, 51.9, 35.0, 34.2, 26.7, 21.5, 13.8.

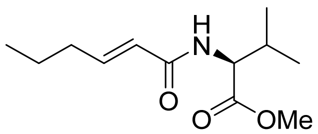

Methyl (*E*)-hex-2-enoyl-*L*-valinate (119) was synthesized according to procedure **7.11** as a white solid (2.07 g, 91% yield). **^{1}H NMR** (400 MHz, $CDCl_3$) δ (ppm): 6.86 (dt, *J* = 15.2, 7.2 Hz, 1H), 5.96 (d, *J* = 8.8 Hz, 1H), 5.83 (dt, *J* = 15.2, 1.6 Hz, 1H), 4.64 (dd, *J* = 8.4, 4.8 Hz,1H), 3.73 (s, 3H), 2.22–2.11 (m, 3H), 1.46 (sext, *J* = 7.2 Hz, 2H), 0.96–0.89 (m, 9H); **^{13}C NMR** (101 MHz, $CDCl_3$) δ (ppm): 172.9, 166.0, 145.7, 123.4, 57.1, 52.3, 34.2, 31.6, 21.6, 19.1, 13.8.

7.12 Synthesis of *tert*-butyl (*R*)-(4-phenylbutan-2-yl)carbamate (100) [100, 101]

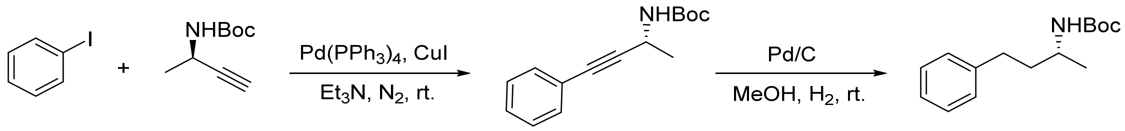

tert-Butyl (*R*)-but-3-yn-2-ylcarbamate (846 mg, 5.0 mmol, 1 equiv.), tetrakis(triphenylphosphine) palladium (173.3 mg, 0.15 mmol, 3 mol%) and cuprous iodide (29 mg, 0.15 mmol, 3 mol%) were placed in a 100 mL Schlenk reaction tube equipped with a magnetic stirring bar. Then, iodobenzene (0.73 mL, 6.5 mmol, 1.3 equiv.) and triethylamine (20.0 mL) were added under a nitrogen atmosphere, and the mixture was stirred overnight at room temperature. After the reaction was complete, deionized water (20.0 mL) and ethyl acetate (20.0 mL) were added to the reaction mixture. The organic layer was separated, and the aqueous layer was extracted with ethyl acetate (3×20.0 mL) three times. The combined organic layers were washed with brine and dried over Na_2SO_4. After concentration, the residue was purified on silica gel chromatography (petroleum ether/EtOAc=10:1) to give the desired product *tert*-butyl (*R*)-(4-phenylbut-3-yn-2-yl)carbamate (1.13 g, 92% yield).

***tert*-Butyl (*R*)-(4-phenylbutan-2-yl)carbamate** was prepared according to the following procedure: *tert*-butyl (*R*)-(4-phenylbut-3-yn-2-yl)carbamate (1.13 g) and palladium-carbon (159.6 mg, 15 mol%) were added to a reactor, methanol was then added (30 mL), and the reactor was degassed with hydrogen gas, filled with 40 bar hydrogen, and heated in a 30 °C water bath with stirring for 1 day. The residue was purified through flash column chromatography (petroleum ether /EtOAc=10:1 to 4:1) to give the desired product **100** (1.08 g, 87% yield). **^{1}H NMR** (400 MHz, $CDCl_3$) δ (ppm): 7.30–7.25 (m, 2H), 7.20–7.15 (m, 3H), 4.35 (brs, 1H), 3.74–3.69 (m, 1H),

2.70–2.60 (m, 2H), 1.76–1.68 (m, 2H), 1.45 (s, 9H), 1.16 (d, J = 6.4 Hz, 3H); **^{13}C NMR** (101 MHz, $CDCl_3$) δ (ppm): 154.8, 131.8, 128.37, 128.35, 122.9, 90.0, 82.2, 80.0, 39.2, 28.5, 23.1.

7.13 Synthesis of 101–103 [102-103]

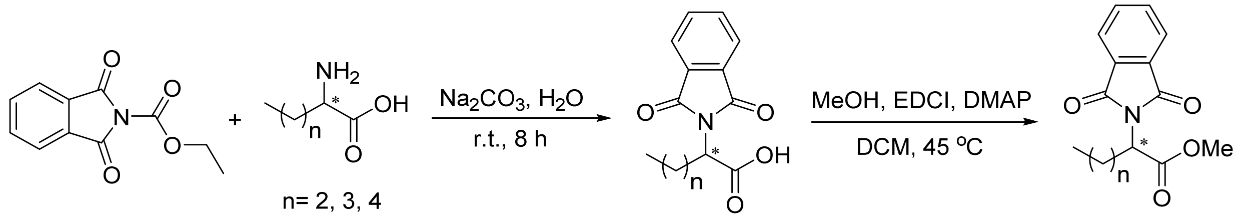

To a round flask equipped with a magnetic stirring bar was added the corresponding amino acid (10 mmol, 1.0 equiv.), *N*-ethoxycarbonylphthalimide (2.2 g, 10 mmol, 1equiv.), Na_2CO_3 (1.06 g, 10 mmol, 1.0 equiv.), and water (20.0 mL). The resulting mixture was stirred at room temperature for 8 h and acidified with aqueous HCl until precipitates were slowly generated. When no precipitate was generated further, the crude mixture was extracted with EtOAc (30.0 mL), and concentrated under reduced pressure. The residue was then purified by flash column chromatography (petroleum ether /EtOAc=10:1 to 4:1) to afford protected an amino acid**.**

Then, the protected amino acid (5.0 mmol, 1 equiv.), 1-(3-dimethylaminopropyl)-3-ethylcarbodiimide hydrochloride (EDCI, 1.25 g, 6.5 mmol, 1.3 equiv.), *N*, *N*-dimethylpyridin-4-amine (DMAP, 153 mg, 1.25 mmol, 0.25 equiv.), and DCM (20.0 mL) were added into a 100 mL round flask equipped with a magnetic stirring bar. The reaction mixture was stirred rapidly and then MeOH (3.0 mL) was added. After the reaction was complete, deionized water (10.0 mL) and DCM (10 mL) were added to the reaction mixture. The organic layer was separated, and the aqueous layer was extracted with dichloromethane (3×15.0 mL) three times. The combined organic layers were washed with brine and dried over Na_2SO_4. After concentration, the residue was purified through flash column chromatography (petroleum ether /EtOAc=5:1 to 3:1) to give the desired product **101–103.**

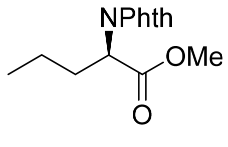

Methyl (*R*)-2-(1,3-dioxoisoindolin-2-yl)pentanoate (101) [104] was synthesized according to procedure **7.13** as a white solid (1.20 g, 92% yield); **^{1}H NMR** (400 MHz, $CDCl_3$) δ (ppm):

7.88–7.84 (m, 2H), 7.75–7.72 (m, 2H), 4.86 (dd, *J* = 10.8, 4.8 Hz, 1H), 3.72 (s, 3H), 2.33–2.21 (m, 1H), 2.20–2.11 (m, 1H), 1.37–1.25 (m, 2H), 0.92 (t, *J* = 7.2 Hz, 3H); **^{13}C NMR** (101 MHz, $CDCl_3$) δ (ppm): 170.1, 167.8, 134.3, 131.9, 123.6, 52.8, 52.0, 30.8, 19.7, 13.5.

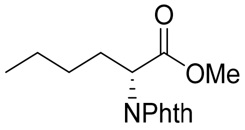

Methyl (*R*)-2-(1,3-dioxoisoindolin-2-yl)hexanoate (102) [105] was synthesized according to procedure **7.13** as a white solid (1.25 g, 91% yield); **H NMR** (400 MHz, $CDCl_3$) δ (ppm): 7.87–7.84 (m, 2H), 7.76–7.72 (m, 2H), 4.84 (dd, *J* = 10.4, 5.2 Hz, 1H), 3.72 (s, 3H), 2.31–2.15 (m, 2H), 1.41–1.21 (m, 4H), 0.86 (t, *J* = 7.2 Hz, 3H); **^{13}C NMR** (101 MHz, $CDCl_3$) δ (ppm): 170.1, 167.8, 134.3, 131.9, 123.7, 52.8, 52.3, 28.54, 28.49, 22.2, 14.0.

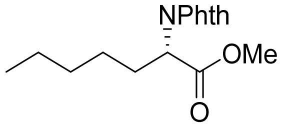

Methyl (*S*)-2-(1,3-dioxoisoindolin-2-yl)heptanoate (103) [106] was synthesized according to procedure **7.13** as a white solid (1.30 g, 90% yield); **^{1}H NMR** (400 MHz, $CDCl_3$) δ (ppm): 7.88–7.84 (m, 2H), 7.51–7.72 (m, 2H), 4.84 (dd, *J* = 10.4, 5.2 Hz, 1H), 3.72 (s, 3H), 2.27–2.16 (m, 2H), 1.33–1.23 (m, 6H), 0.83 (t, *J* = 7.2 Hz, 3H); **^{13}C NMR** (101 MHz, $CDCl_3$) δ (ppm): 170.1, 167.9, 134.3, 131.9, 123.7, 52.8, 52.3, 31.2, 28.7, 26.1, 22.5, 14.1.

7.14 Synthesis of methyl (*S*)-2-(4-chlorobenzamido)hexanoate (104) [107]

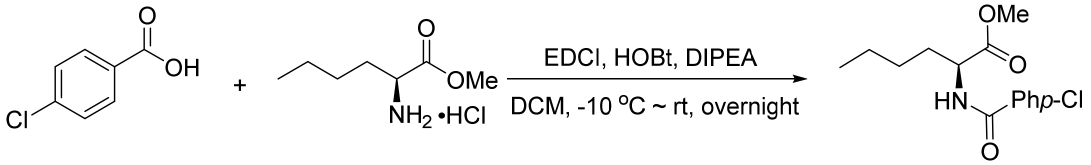

To a round flask equipped with a magnetic stirring bar was added 4-chlorobenzoic acid (1.72 g, 11.0 mmol, 1.1 equiv), methyl (*S*)-2-aminohexanoate hydrochloride (1.82 g, 10 mmol), EDCI (2.1 g, 11.0 mmol, 1.1 equiv), HOBt (1.49 g, 11.0 mmol, 1.1 equiv), and DIPEA (3.88 g, 30 mmol, 3.0 equiv) in DCM (40.0 mL). The resulting mixture was stirred at -10 °C for 2 hours, and then warmed to room temperature overnight. Upon reaction completion, water (20.0 mL) was added and the mixture was extracted with DCM (3×20.0 mL) three times. The combined organic layers were washed with brine and dried over Na_2SO_4. After concentration, the residue was purified on silica

gel chromatography (petroleum ether /EtOAc=50:1) to give the desired product **104** (2.55 g, 90% yield). ^{1}**H NMR** (400 MHz, $CDCl_3$) δ (ppm): 7.76–7.71 (m, 2H), 7.42–7.36 (m, 2H), 6.68 (brs, 1H), 4.78 (dd, *J* = 13.2, 7.2 Hz, 1H), 3.77 (s, 3H), 1.99–1.87 (m, 1H), 1.81–1.70 (m, 1H), 1.42–1.23 (m, 4H), 0.89 (t, *J* = 6.8 Hz, 3H); ^{13}**C NMR** (101 MHz, $CDCl_3$) δ (ppm): 173.4, 166.1, 138.1, 132.5, 129.0, 128.7, 52.8, 52.6, 32.4, 27.5, 22.5, 14.0.

7.15 Synthesis of 105-108, 110 [107]

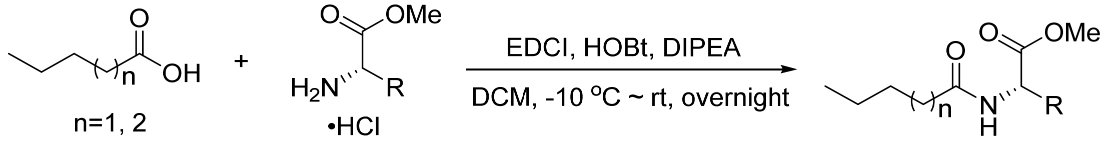

To a round flask equipped with a magnetic stirring bar was added valeric acid or hexanoic acid (11.0 mmol, 1.1 equiv), amino acid methyl ester hydrochloride (10 mmol), EDCI (2.1 g, 11.0 mmol, 1.1 equiv), HOBt (1.49 g, 11.0 mmol, 1.1 equiv), and DIPEA (3.88 g, 30 mmol, 3.0 equiv) in DCM (40.0 mL). The resulting mixture was stirred at −10 °C for 2 hours, and then warmed to room temperature overnight. Upon reaction completion, water (20.0 mL) was added and the mixture was extracted with DCM (3×20.0 mL) three times. The combined organic layers were washed with brine and dried over Na_2SO_4. After concentration, the residue was purified on silica gel chromatography (petroleum ether /EtOAc = 50:1) to give the desired product **105-108, 110**.

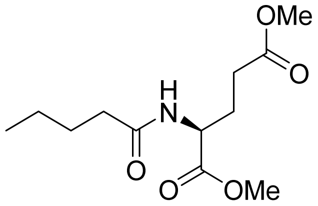

Dimethyl pentanoyl-*L*-glutamate (105) was synthesized according to procedure **7.15** as a white solid (2.31 g, 89% yield); ^{1}**H NMR** (400 MHz, $CDCl_3$) δ (ppm): 6.31 (brs, 1H), 4.57 (dd, *J* = 12.8, 7.6 Hz, 1H), 3.69 (s, 3H), 3.62 (s, 3H), 2.43–2.26 (m, 2H), 2.20–2.09 (m, 3H), 1.99–1.88 (m, 1H), 1.56 (quint, *J* = 7.6 Hz, 2H), 1.29 (sext, *J* = 7.6 Hz, 2H), 0.86 (t, *J* = 7.2 Hz, 3H); ^{13}**C NMR** (101 MHz, $CDCl_3$) δ (ppm): 173.3, 173.2, 172.5, 52.4, 51.8, 51.5, 36.2, 30.1, 27.7, 27.3, 22.3, 13.8.

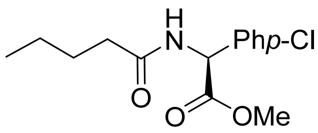

Methyl (S)-2-(4-chlorophenyl)-2-pentanamidoacetate (**106**) was synthesized according to procedure **7.15** as a white solid (2.50 g, 88% yield); **^{1}H NMR** (400 MHz, $CDCl_3$) δ (ppm): 7.35−7.26 (m, 4H), 6.53 (brs, 1H), 5.56 (d, *J* = 7.2 Hz, 2H), 3.73 (s, 3H), 2.24 (td, *J* = 7.6, 2.0 Hz, 2H), 1.61 (quint, *J* = 7.6 Hz, 2H), 1.33 (sext, *J* = 7.6 Hz, 2H), 0.90 (t, *J* = 7.2 Hz, 3H); **^{3}C NMR** (101 MHz, $CDCl_3$) δ (ppm): 172.6, 171.3, 135.5, 134.6, 129.2, 128.7, 55.8, 53.1, 36.2, 27.6, 22.4, 13.9.

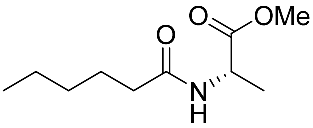

Methyl hexanoyl-*L*-alaninate (**107**) was synthesized according to procedure **7.15** as a white solid (1.87 g, 93% yield); **^{1}H NMR** (400 MHz, $CDCl_3$) δ (ppm): 6.03 (brs, 1H), 4.60 (quint, *J* = 7.2 Hz, 1H), 3.74 (s, 3H), 2.19 (t, *J* = 7.6 Hz, 2H), 1.63 (quint, *J* = 7.6 Hz, 2H), 1.39 (d, *J* = 7.2 Hz, 3H), 1.33−1.26 (m, 4H), 0.88 (t, *J* = 6.8 Hz, 3H); **^{13}C NMR** (101 MHz, $CDCl_3$) δ (ppm): 173.9, 172.8, 52.6, 48.0, 36.7, 31.5, 25.4, 22.5, 18.7, 14.1.

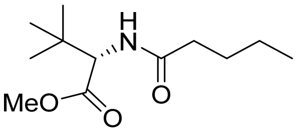

Methyl (*S*)-3,3-dimethyl-2-pentanamidobutanoate (**108**) was synthesized according to procedure **7.15** as a white solid (2.18 g, 95% yield); **^{1}H NMR** (400 MHz, $CDCl_3$) δ (ppm): 6.01 (brs, 1H), 4.45 (d, *J* = 9.2 Hz, 1H), 3.67 (s, 3H), 2.19 (t, *J* = 7.2 Hz, 2H), 1.58 (quint, *J* = 7.6 Hz, 2H), 1.31 (sext, *J* = 7.6 Hz, 2H), 0.92 (s, 9H), 0.87 (t, *J* = 7.2 Hz, 3H); **^{13}C NMR** (101 MHz, $CDCl_3$) δ (ppm): 172.9, 172.4, 59.8, 51.7, 36.5, 34.7, 27.8, 26.6, 22.4, 13.8.

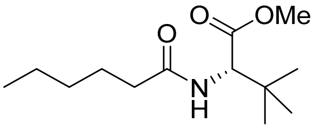

Methyl (*S*)-2-hexanamido-3,3-dimethylbutanoate (**110**) was synthesized according to procedure **7.15** as a white solid (2.21 g, 91% yield); **^{1}H NMR** (400 MHz, $CDCl_3$) δ (ppm): 5.95 (brs, 1H), 4.48 (d, *J* = 9.2 Hz, 1H), 3.71 (s, 3H), 2.21 (t, *J* = 7.6 Hz, 2H), 1.63 (quint, *J* = 7.2 Hz, 2H), 1.34−1.25 (m, 4H), 0.96 (s, 9H), 0.88 (t, *J* = 6.8 Hz, 3H); **^{13}C NMR** (101 MHz, $CDCl_3$) δ (ppm): 172.9, 172.5, 59.8, 51.9, 36.9, 34.8, 31.5, 26.7, 25.5, 22.5, 14.0.

7.16 Synthesis of methyl (*R*)-2-((*tert*-butoxycarbonyl)amino)-3-cyclopentylpropa-noate (109) [108]

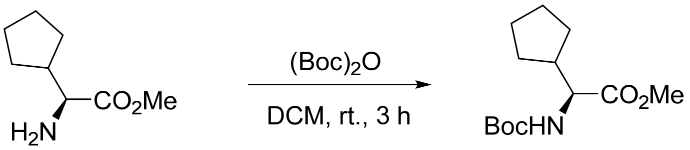

To a sealed tube equipped with a magnetic stir bar was added methyl (*R*)-2-amino-2-cyclopentylacetate (786.0 mg, 5 mmol, 1.0 equiv.), Boc anhydride (1.38 mL, 6.0 mmol, 1.2 equiv.) and DCM (20 mL). Then, the reaction mixture was stirred at room temperature for 3 hours. After addition of DCM (20.0 mL) and H_2O (10.0 mL), the reaction mixture was extracted with DCM (3×20.0 mL). The combined organic layers were then dried over Na_2SO_4. After concentration, the crude product **109** (1.22 g, 95% yield) was obtained without further purification. **^{1}H NMR** (400 MHz, $CDCl_3$) δ (ppm): 5.00 (brs, 1H), 4.20 (t, *J* = 3.2 Hz, 1H), 3.70 (s, 3H), 2.21–2.09 (m, 1H), 1.70–1.58 (m, 4H), 1.56–1.48 (m, 2H), 1.41 (s, 9H), 1.38–1.26 (m, 2H); **^{13}C NMR** (101 MHz, $CDCl_3$) δ (ppm): 173.5, 155.7, 79.9, 56.7, 52.1, 42.8, 29.0, 28.4, 25.4.

7.17 Synthesis of methyl 1-((*tert*-butoxycarbonyl)amino)cyclopentane-1-carboxylate (111)[109]

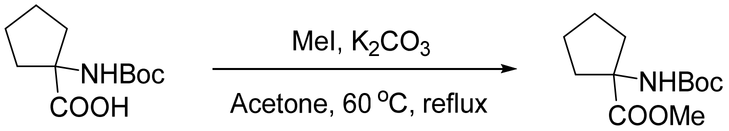

A solution of 1-((*tert*-butoxycarbonyl)amino)cyclopentane-1-carboxylic acid (1.146 g, 5 mmol, 1 equiv.) and potassium carbonate (1.728 g, 12.5 mmol, 2.5 equiv.) in 30 mL acetone was prepared. Methyl iodide (0.47 mL, 7.5 mmol, 1.5 equiv.) was slowly added and the reaction mixture was warmed up to 60 °C and stirred for 2 hours. After addition of 15.0 mL ethyl ether and 20.0 mL H_2O, the reaction mixture was extracted with ethyl ether (3x15 mL). The combined organic layers were then dried over Na_2SO_4, and filtered. After concentration, the crude product **111** (colorless liquid, 1.2 g, 98% yield) was obtained without further purification. **^{1}H NMR** (400 MHz, $CDCl_3$) δ (ppm): 4.85 (brs, 1H), 3.72 (s, 3H), 2.25–2.13 (m, 2H), 1.92–1.85 (m, 2H), 1.79–1.73 (m, 4H), 1.43 (s, 9H); **^{13}C NMR** (101 MHz, $CDCl_3$) δ (ppm): 175.4, 155.3, 66.2, 52.4, 38.4, 37.9, 28.4, 24.7.

7.18 Synthesis of methyl (*R*)-2-((*tert*-butoxycarbonyl)amino)-4-phenylbutanoate (113) [103]

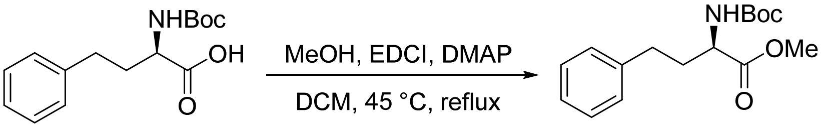

(*R*)-2-((*tert*-Butoxycarbonyl)amino)-4-phenylbutanoic acid (1.40 g, 5.0 mmol, 1.0 equiv.), 1-(3-dimethylaminopropyl)-3-ethylcarbodiimide hydrochloride (EDCI, 1.25 g, 6.5 mmol, 1.3 equiv.), *N*,*N*-dimethylpyridin-4-amine (DMAP, 153 mg, 1.25 mmol, 0.25 equiv.), and DCM (20.0 mL) were added into a 100 mL round flask equipped with a magnetic stirring bar. The reaction mixture was stirred rapidly and then MeOH (3.0 mL) was added. Upon reaction completion, water (20.0 mL) was added and the mixture was extracted with DCM (3×20.0 mL) three times. The combined organic layers were then dried over Na_2SO_4. After concentration, the residue was purified through flash column chromatography (petroleum ether /EtOAc=10:1 to 4:1) to give the desired product **113** [110] (colorless liquid, 1.39 g, 95% yield). **^{1}H NMR** (400 MHz, $CDCl_3$) δ (ppm): 7.31–7.25 (m, 2H), 7.22–7.15 (m, 3H), 5.04 (brs, 1H), 4.37–4.35 (m, 1H), 3.72 (s, 3H), 2.70–2.64 (m, 2H), 2.20–2.09 (m, 1H), 2.00–1.89 (m, 1H), 1.46 (s, 9H); **^{13}C NMR** (101 MHz, $CDCl_3$) δ (ppm): 173.2, 155.5, 140.9, 128.6, 128.5, 126.2, 80.0, 53.3, 52.3, 34.4, 31.7, 28.4.

7.19 Synthesis of methyl (*R*)-2-((*tert*-butoxycarbonyl)amino)-4-phenylbutanoate (114) [100, 101]

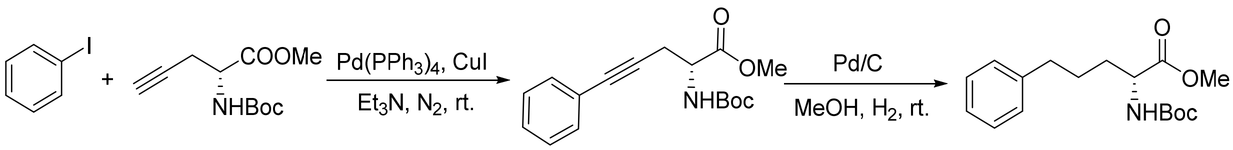

Methyl (*R*)-2-((*tert*-butoxycarbonyl)amino)pent-4-ynoate (1136 mg, 5.0 mmol, 1.0 equiv.), tetrakis(triphenylphosphine) palladium (173.0 mg, 0.15 mmol, 3 mol%) and cuprous iodide (29.0 mg, 0.15 mmol, 3 mol%) were placed in a 100 mL Schlenk reaction tube equipped with a magnetic stirring bar. Then, iodobenzene (0.73 mL, 6.5 mmol, 1.3 equiv.) and triethylamine (20.0 mL) were added under a nitrogen atmosphere, and the mixture was stirred overnight at room temperature. After the reaction was complete, deionized water (20.0 mL) and ethyl acetate (20.0 mL) were added to the reaction mixture. The organic layer was separated, and the aqueous layer was extracted with ethyl acetate (3×20.0 mL) three times. The combined organic layers were washed with brine and dried over Na_2SO_4. After concentration, the residue was purified on silica gel chromatography (petroleum ether /EtOAc=10:1) to give the desired product methyl (*R*)-2-((*tert*-butoxycarbonyl)amino)-5-phenylpent-4-ynoate (1.35 g, 89% yield).

The pure product methyl (*R*)-2-((*tert*-butoxycarbonyl)amino)-5-phenylpent-4-ynoate (1350.0 mg) and palladium-carbon (710 mg, 10 wt%, 15 mol%) were added to a reactor, methanol was then added (30 mL), and the reactor was degassed with hydrogen gas, filled with 40 bar hydrogen, and heated in a 30 °C water bath with stirring for one day. The residue was purified through flash column chromatography (petroleum ether /EtOAc=10:1 to 4:1) to give the desired product **114**[111] (1.20 g, 88%). **^{1}H NMR** (400 MHz, $CDCl_3$) δ (ppm): 7.28–7.23 (m, 2H), 7.19–7.12 (m, 3H), 5.12 (brs, 1H), 4.36–4.29 (m, 1H), 3.69 (s, 3H), 2.69–2.55 (m, 2H), 1.85–1.78 (m, 1H), 1.72–1.58 (m, 1H), 1.43 (s, 9H); **^{13}C NMR** (101 MHz, $CDCl_3$) δ (ppm): 173.3, 155.4, 141.7, 128.4, 128.3, 125.9, 79.8, 53.3, 52.2, 35.3, 32.2, 28.3, 27.1.

8. Synthesis of oximes from ketones to aid in product analysis

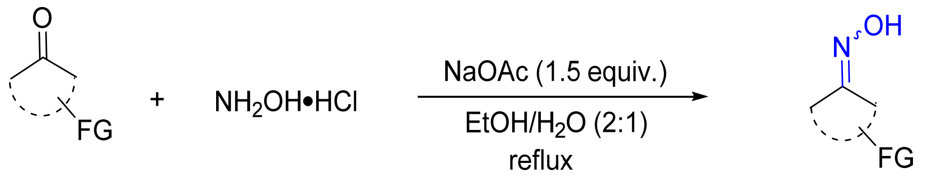

To a solution of ketone (1.0 equiv.) and hydroxylamine hydrochloride (1.5 equiv.) in ethanol/water (2:1) was added sodium acetate (1.5 equiv.) portion-wise. The reaction mixture was heated to reflux overnight, and then cooled to room temperature. Then, the reaction mixture was basified with Na_2CO_3 powder. The filtrate was concentrated under reduced pressure to produce the crude product, which was purified through flash column chromatography (gradient elution: petroleum ether : EtOAc= 30:1 to 20:1) to give desired ketone oxime.

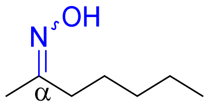

(*E/Z*)-Heptan-2-one oxime (12a-α-oxime) isomer mixtures (*E/Z* = 3:1):

Major (*E*)-12a-α-oxime: **^{1}H NMR** (400 MHz, $CDCl_3$) δ (ppm): 8.06 (brs, 1H), 2.17 (t, *J* = 7.8 Hz, 2H), 1.88 (s, 3H), 1.54–1.46 (m, 2H), 1.35–1.25 (m, 4H), 0.88 (t, *J* = 7.2 Hz, 3H); **^{13}C NMR** (101 MHz, $CDCl_3$) δ (ppm): 158.8, 35.9, 31.4, 26.0, 22.5, 14.1, 13.5.

Minor (*Z*)-12a-α-oxime: **^{1}H NMR** (400 MHz, $CDCl_3$) δ (ppm): 8.06 (brs, 1H), 2.36 (t, *J* = 8.0 Hz, 2H), 1.86 (s, 3H), 1.54–1.46 (m, 2H), 1.35–1.25 (m, 4H), 0.89 (t, *J* = 7.2 Hz, 3H); **^{13}C NMR** (101 MHz, $CDCl_3$) δ (ppm): 159.1, 32.0, 28.7, 25.3, 22.5, 21.0, 19.9.

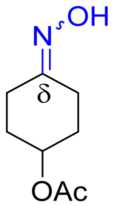

4-(Hydroxyimino)cyclohexyl acetate (**16a-δ-oxime**): **^{1}H NMR** (400 MHz, $CDCl_3$) δ (ppm): 9.13 (brs, 1H), 5.03–4.96 (m, 1H), 2.80–2.72 (m, 1H), 2.51–2.39 (m, 2H), 2.27–2.19 (m, 1H), 2.06 (s, 3H), 1.96–1.85 (m, 2H), 1.83–1.72 (m, 2H); **^{13}C NMR** (101 MHz, $CDCl_3$) δ (ppm): 170.7, 158.4, 70.4, 30.8, 29.5, 28.1, 21.4, 20.5.

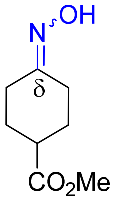

Methyl 4-(hydroxyimino)cyclohexane-1-carboxylate (**17a-δ-oxime**): **^{1}H NMR** (400 MHz, $CDCl_3$) δ (ppm): 7.93 (brs, 1H), 3.68 (s, 3H), 3.13 (d, *J* = 13.6 Hz, 1H), 2.56 (t, *J* = 10.4 Hz, 1H), 2.45–2.39 (m, 1H), 2.18–1.99 (m, 4H), 1.79–1.67 (m, 2H); **^{13}C NMR** (101 MHz, $CDCl_3$) δ (ppm): 175.2, 158.7, 51.9, 41.9, 30.3, 28.7, 27.4, 22.8.

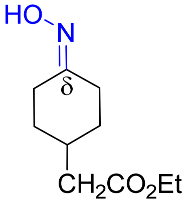

Ethyl 2-(4-(hydroxyimino)cyclohexyl)acetate (**18a-δ-oxime**): **^{1}H NMR** (400 MHz, $CDCl_3$) δ (ppm): 4.13 (q, *J* = 7.2 Hz, 2H), 3.27 (d, *J* = 14.0 Hz, 1H), 2.39 (d, *J* = 14.0 Hz, 1H), 2.24 (d, *J* = 7.2 Hz, 2H), 2.17–2.01 (m, 2H), 1.95–1.78 (m, 3H), 1.25 (t, *J* = 6.8 Hz, 3H),1.25–1.15 (m, 2H); **^{13}C NMR** (101 MHz, $CDCl_3$) δ (ppm): 172.7, 159.6, 60.4, 40.9, 34.0, 32.6, 31.5, 31.3, 23.4, 14.3.

9. General procedure for oximation of hydrocarbons

General Procedure A:

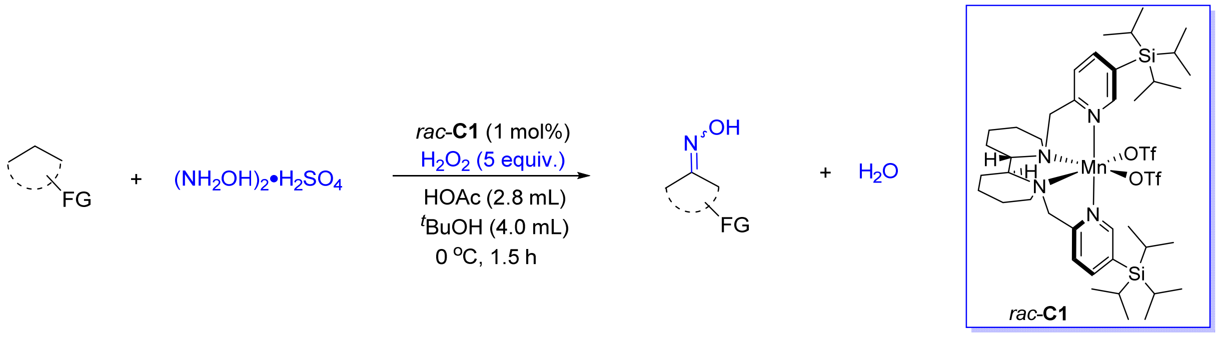

***Rac*-C1** (10.2 mg, 1.0 mol%), hydroxylamine sulfate (328.3 mg, 2.0 mmol, 2.0 equiv.), a hydrocarbon substrate (1.0 mmol, 1.0 equiv.), AcOH (2.8 mL for functionalized alkanes and nonfunctionalized acyclic alkane; 1.2 mL for nonfunctionalized cyclic alkanes) and ^{t}BuOH (2.0 mL) were added to a reaction tube. The mixture was cooled down to 0 °C in a cryogenic bath, and then H_2O_2 (5.0 equiv., 5.0 mmol, 567 μL, 30% wt. in H_2O) in 2 mL of ^{t}BuOH was added with a syringe pump over 1 h under stirring at 0 °C without nitrogen protection. After stirring for an additional 30 min, the reaction mixture was quenched with Na_2SO_3. Next, the resulting mixture was basified with Na_2CO_3 for 30 minutes at 0 °C in a cryogenic bath. After completion of the reaction, water (5.0 mL) was added, and the solution was extracted with DCM (3 × 15.0 mL). The combined organic phase was dried over anhydrous Na_2SO_4 and concentrated under reduced pressure to produce the crude product, which was purified by flash column chromatography to afford the desired product.

Because of the volatility of the substrates **2** and **10-12**, the amount of substrate was increased to 5 mmol, and that of hydroxylamine sulfate was reduced to 0.5 mmol, with the ratio of substrate to hydroxylamine sulfate being 10:1. For the gaseous substrates **8** and **9**, the amount of substrate was in much excess to the hydroxylamine sulfate (0.5 mmol). All the other reaction conditions remained unchanged. For reactions of these substrates (**2**, **8-12**), hydroxylamine sulfate is the limiting reagent.

General Procedure B:

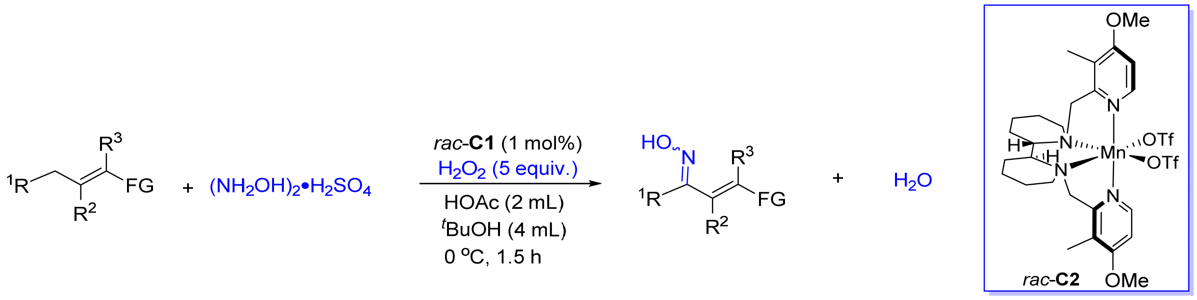

***Rac*-C2** (7.9 mg, 1.0 mol%), hydroxylamine sulfate (328.3 mg, 2.0 mmol. 2.0 equiv.), substrate (1.0 mmol, 1.0 equiv.), AcOH (2.0 mL), and ^{t}BuOH (2.0 mL) were added to a reaction tube. The mixture was cooled down to 0 ºC in a cryogenic bath, and then H_2O_2 (5.0 equiv., 5.0 mmol, 567 µL, 30% wt. in H_2O) in 2 mL of ^{t}BuOH was added with a syringe pump over 1 h under stirring at at 0 ºC without nitrogen protection. After stirring for another 30 min, the reaction mixture was quenched with Na_2SO_3. Next, the resulting mixture was basified with Na_2CO_3 for 30 minutes at 0 ºC in a cryogenic bath. After completion of the reaction, water (5 mL) was added, and the solution was extracted with DCM (3 × 15 mL). The combined organic phase was dried over anhydrous Na_2SO_4 and concentrated under reduced pressure to produce the crude product. The crude product was purified by flash column chromatography to afford the desired product.

General Procedure C:

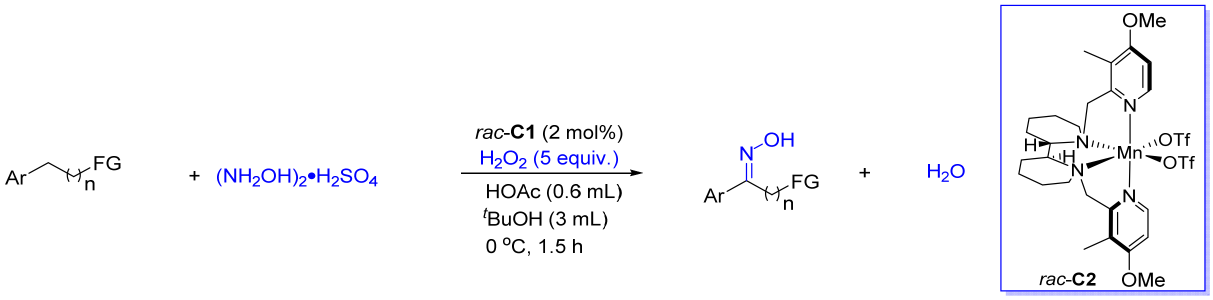

***Rac*-C2** (7.9 mg, 2.0 mol%), hydroxylamine sulfate (246.2 mg, 1.5 mmol. 3.0 equiv.), substrate (0.5 mmol, 1.0 equiv.), AcOH (0.6 mL), and ^{t}BuOH (1.0 mL) were added to a reaction tube. The mixture was cooled down to 0 ºC in a cryogenic bath and then H_2O_2 (2.5 mmol, 5 equiv., 284 µL, 30% wt. in H_2O) in 0.5 mL of ^{t}BuOH was added with a syringe pump over 1 h under stirring at 0 ºC without nitrogen protection. After stirring for another 30 min, the reaction mixture was quenched with Na_2SO_3 solid. Next, the resulting mixture was basified with Na_2CO_3 for 30 minutes at 50 ºC. After completion of the reaction, water (5.0 mL) was added, and the solution was extracted with DCM (3 × 15.0 mL). The combined organic phase was dried over anhydrous

Na_2SO_4 and concentrated under reduced pressure to produce the crude product. The crude product was purified by flash column chromatography to afford the desired product.

10. Gram-scale synthesis of cyclododecanone oxime

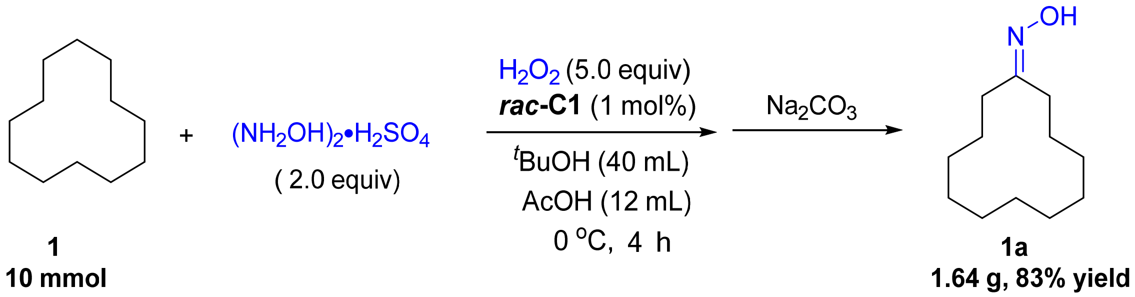

***Rac*-C1** (100.1 mg, 1.0 mol%), hydroxylamine sulfate (3.28 g, 20.0 mmol. 2.0 equiv.), cyclododecane (1.68 g, 10.0 mmol, 1.0 equiv.), AcOH (12.0 mL) and ^{t}BuOH (20.0 mL) were added to a reaction tube. The mixture was cooled down to 0 ºC in a cryogenic bath, and then H_2O_2 (5 equiv., 50 mmol, 5.67 mL, 30% wt. in H_2O) in 20 mL of ^{t}BuOH was added dropwise with a syringe pump over 3 h under stirring at 0 ºC without nitrogen protection. The reaction mixture was stirred for another 30 min, and then the mixture was quenched with Na_2SO_3 solid. Next, the resulting mixture was evaporated to partially remove AcOH under reduced pressure, and DCM (20 mL) was added to the remaining mixture. The resulting mixture was basified with Na_2CO_3 for about 30 minutes at room temperature. After completion, water (50.0 mL) was added, and the reaction solution was extracted with DCM (3 × 100.0 mL). The combined organic phase was dried over anhydrous Na_2SO_4 and concentrated under reduced pressure to produce the crude product, which was purified through flash column chromatography (petroleum ether /EtOAc = 100:1 to 5:1) to give pure cyclododecanone oxime (1.63 g, 83% yield).

11. Oximation of propane to acetone oxime

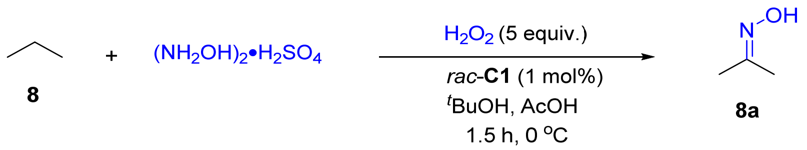

***Rac*-C1** (10.1 mg, 1.0 mol%), hydroxylamine sulfate (164.2 mg, 1.0 mmol. 1.0 equiv.), AcOH (2.8 mL) were added to a reaction tube in ^{t}BuOH (2.0 mL), and the tube was capped with a rubber stopper and cooled down to 0 ºC in an ice bath. Then, propane gas (flow rate 4 mL/min) was

steadily flowed through the reaction solution via a syringe needle for 1 h. Meanwhile, H_2O_2 (5.0 mmol, 5.0 equiv., 567 uL, 30% wt. in H_2O) in 2 mL of ^{t}BuOH was added with a syringe pump over 1 h under stirring at 0 °C. The reaction mixture was stirred for another 30 min, and then quenched with Na_2SO_3 solid. Next, 1,3,5-trimethoxybenzene (168.2 mg, 1 mmol) was added to the reaction tube and the resulting mixture was basified with Na_2CO_3 for about 30 minutes at 0 °C in an ice bath. After completion, the reaction solution was filtered and then washed with DCM (10.0 mL). The reaction mixture was analyzed by GC (GC trace and calibration curve are shown below: $y = 2.6407*x-0.0291$, $R^2=0.9991$, $x = A_{acetoxime} / A_{1,3,5\text{-Trimethoxybenzene}}$; $y = n_{acetoxime} / n_{1,3,5\text{-Trimethoxybenzene}}$; **8a**, 0.65 mmol, **65% yield**).

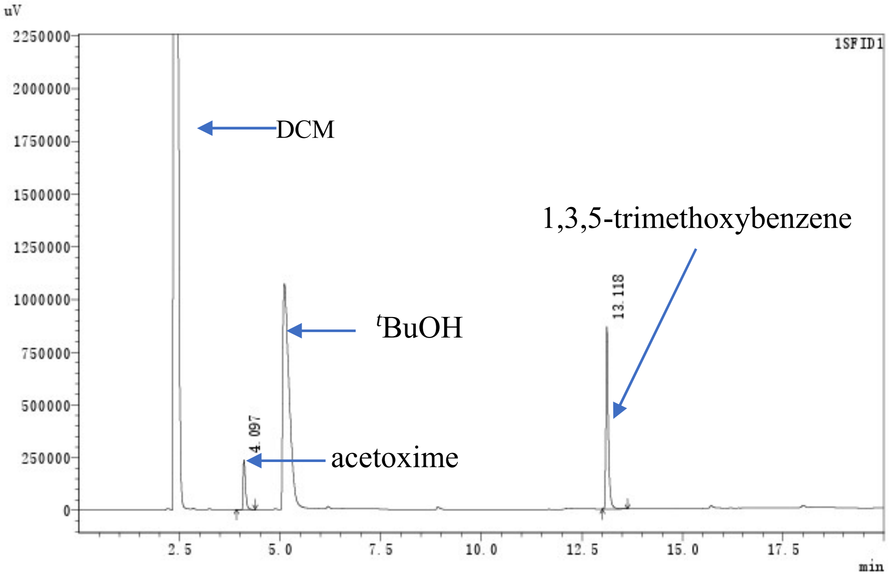

No.	Retention Time	Area	Height	Concentration	Concentration Unit	Mark
1	4.097	928935	234292	20.510		M
2	13.118	3600350	860456	79.490		M
Toal		4529285	1094747	100.000		

Figure S-46. GC trace of the mixture resulting from oxidative oximation of propane. GC parameters: injection temperature = 250 °C, column temperature = 90 °C, detector temperature = 250 °C. The carrier gas was N_2.

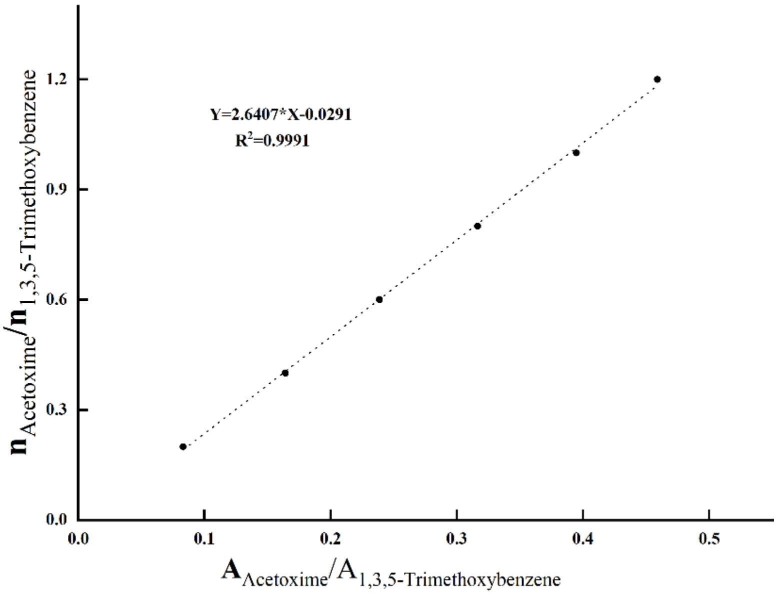

Figure S-47. GC calibration curve of acetoxime (commercial sample) and 1,3,5-trimethoxybenzene (GC calibration curve: $Y = 2.6407*X$-0.0291, R^2=0.9991, X = $A_{acetoxime}$ /$A_{1,3,5\text{-}Trimethoxybenzene}$; Y = $n_{acetoxime}$ /$n_{1,3,5\text{-}Trimethoxybenzene}$; A: area; n: number of moles).

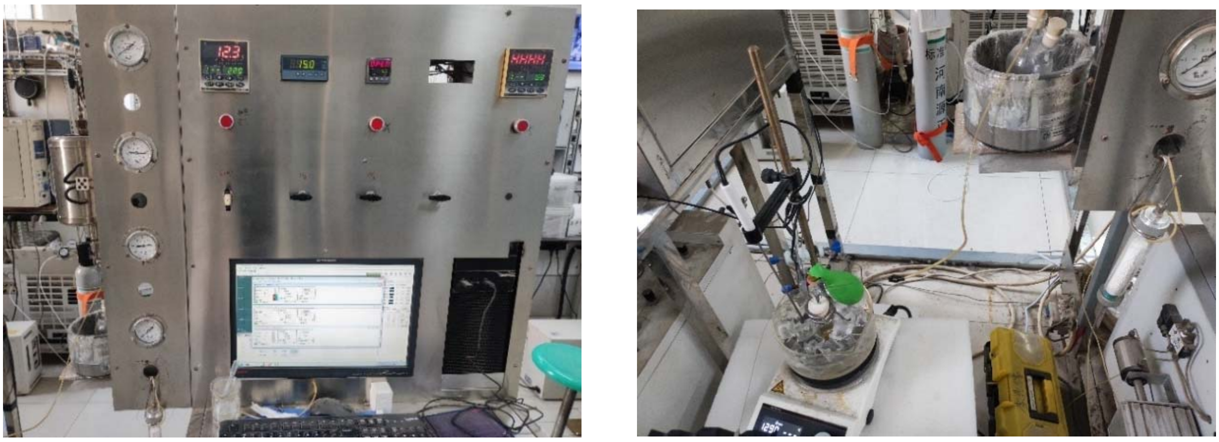

Figure S-48. The device used in oxidative oximation of propane gas.

12. Analytical data of products

The analysis of products was aided by the literature, commercial products and/or samples prepared in this study (Section 8).

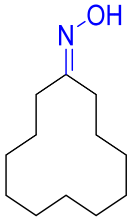

Cyclododecanone oxime (1a) [112] was synthesized by the general procedure **A** from cyclododecane (168.3 mg, 1 mmol) and purified by flash column chromatography on silica gel (gradient elution: Petroleum ether : EtOAc = 30:1 to 20:1) to afford the desired product cyclododecanone oxime as white solid (**179.6 mg, 91% yield**);

^{1}H NMR (400 MHz, $CDCl_3$) δ (ppm): 8.22 (brs, 1H), 2.42 (t, J = 6.4 Hz, 2H), 2.26 (t, J = 6.4 Hz, 2H), 1.68–1.53 (m, 4H), 1.45–1.27 (m, 14H);

^{13}C NMR (101 MHz, $CDCl_3$) δ (ppm): 160.7, 30.4, 26.3, 25.6, 25.2, 24.9, 24.1, 23.6, 23.4, 23.32, 23.26, 22.8;

HRMS (ESI) *m/z* $[M+Na]^+$ calculated for $C_{12}H_{23}NNaO^+$ 220.1672, found 220.1679.

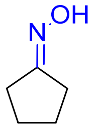

Cyclopentanone oxime (2a) [113] was synthesized by the general procedure **A** from hydroxylamine sulfate (164.2 mg, 1.0 mmol. 1.0 equiv.) and cyclopentane (351.0 mg, 5 mmol), and purified by flash column chromatography on silica gel (gradient elution: Petroleum ether : EtOAc = 30:1 to 20:1) to afford the desired product cyclopentanone oxime as white solid (**78.3 mg, 79% yield**);

^{1}H NMR (400 MHz, $CDCl_3$) δ (ppm): 8.43 (brs, 1H), 2.46 (t, J = 6.8 Hz, 2H), 2.36 (t, J = 6.8 Hz, 2H), 1.84–1.67 (m, 4H);

^{13}C NMR (101 MHz, $CDCl_3$) δ (ppm): 167.6, 31.0, 27.3, 25.3, 24.7;

HRMS (ESI) *m/z* $[M+Na]^+$ calculated for $C_5H_9NNaO^+$ 122.0576, found 122.0581.

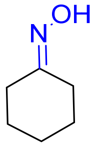

Cyclohexanone oxime (3a) [112] was synthesized by the general procedure **A** from cyclohexane (84.2 mg, 1 mmol) and purified by flash column chromatography on silica gel (gradient elution:

Petroleum ether : EtOAc = 30:1 to 20:1) to afford the desired product cyclohexanone oxime as white solid (**91.7 mg, 81% yield**);

^{1}H NMR (400 MHz, $CDCl_3$) δ (ppm): 7.37 (brs, 1H), 2.50 (t, J = 6.0 Hz, 2H), 2.21 (t, J = 6.0 Hz, 2H), 1.71–1.58 (m, 6H);

^{13}C NMR (101 MHz, $CDCl_3$) δ (ppm): 160.9, 32.3, 27.0, 25.9, 25.7, 24.6;

HRMS (ESI) m/z $[M+H]^+$ calculated for $C_6H_{12}NO^+$ 114.0913, found 114.0912.

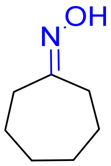

Cycloheptanone oxime (4a) [114] was synthesized by the general procedure **A** from cycloheptane (98.2 mg, 1 mmol) and purified by flash column chromatography on silica gel (gradient elution: Petroleum ether : EtOAc = 30:1 to 20:1) to afford the desired product cycloheptanone oxime as white solid (**113.2 mg, 89% yield**);

^{1}H NMR (400 MHz, $CDCl_3$) δ (ppm): 8.11 (brs, 1H), 2.58 (t, J = 6.0 Hz, 2H), 2.37 (t, J = 5.6 Hz, 2H), 1.70–1.51 (m, 8H);

^{13}C NMR (101 MHz, $CDCl_3$) δ (ppm): 164.4, 33.8, 30.5, 30.4, 28.6, 27.6, 24.6;

HRMS (ESI) m/z $[M+Na]^+$ calculated for $C_7H_{13}NNaO^+$: 150.0889, found 150.0885.

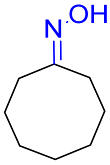

Cyclooctanone oxime (5a) [114] was synthesized by the general procedure **A** from cyclooctane (112.2 mg, 1 mmol) and purified by flash column chromatography on silica gel (gradient elution: Petroleum ether : EtOAc = 30:1 to 20:1) to afford the desired product cyclooctanone oxime as white solid (**127.1 mg, 90% yield**);

^{1}H NMR (400 MHz, $CDCl_3$) δ (ppm): 8.93 (brs, 1H), 2.45 (t, J = 6.4 Hz, 2H), 2.29 (t, J = 6.4 Hz, 2H), 1.82–1.69 (m, 4H), 1.52–1.46 (m, 6H);

^{13}C NMR (101 MHz, $CDCl_3$) δ (ppm): 164.3, 33.3, 27.4, 26.9, 26.7, 25.6, 24.8, 24.6;

HRMS (ESI) m/z $[M+Na]^+$ calculated for $C_8H_{15}NNaO^+$: 164.1046, found 164.1040.

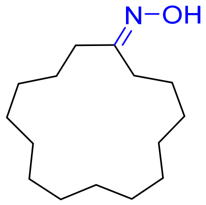

Cyclopentadecanone oxime (6a) was synthesized by the general procedure **A** from cyclopentadecane (210.4 mg, 1 mmol) and purified by flash column chromatography on silica gel (gradient elution: Petroleum ether : EtOAc = 30:1 to 20:1) to afford the desired product cyclopentadecanone oxime as white solid (**177.2 mg, 74% yield**);

^{1}H NMR (400 MHz, $CDCl_3$) δ (ppm): 8.09 (brs, 1H), 2.34 (t, J = 7.6 Hz, 2H), 2.19 (t, J = 7.6 Hz, 2H), 1.64–1.50 (m, 4H), 1.45–1.30 (m, 20H);

^{13}C NMR (101 MHz, $CDCl_3$) δ (ppm): 162.4, 34.5, 28.1, 27.6, 27.5, 26.8, 26.7, 26.53, 26.51, 26.44 (2C), 26.42, 25.3, 24.5;

HRMS (ESI) m/z $[M+H]^+$ calculated for $C_{15}H_{30}NO^+$: 240.2322, found 240.2319.

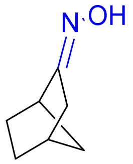

(*E/Z*)-Bicyclo[2.2.1]heptan-2-one oxime (7a) [115] was synthesized by the general procedure **A** from bicyclo[2.2.1]heptane (96.2 mg, 1 mmol) and purified by flash column chromatography on silica gel (gradient elution: Petroleum ether : EtOAc = 30:1 to 20:1) to afford the desired product (*E*/*Z*)-**7a** isomer mixture as white solid (**103.9 mg, 83% yield**, ***E/Z* = *7:1***);

The E/Z isomers were confirmed by ^{1}H and ^{13}C NMR and the *ratio of E/Z isomers was calculated by integration of ^{1}H and ^{13}C NMR spectra.*

Major (*E*)-7a: ^{1}H NMR (400 MHz, $CDCl_3$) δ (ppm): 9.17 (brs, 1H), 2.85 (s, 1H), 2.49 (s, 1H), 2.28 (d, J = 17.6 Hz, 1H), 2.09 (d, J = 17.6 Hz, 1H), 1.75–1.58 (m,2H), 1.52–1.42 (m, 2H), 1.40–1.34 (m, 1H), 1.34–1.24 (m, 1H); **^{13}C NMR** (101 MHz, $CDCl_3$) δ (ppm): 167.9, 42.4, 39.1, 35.5, 34.9, 27.8, 27.1.

Minor (*Z*)-7a: ^{1}H NMR (400 MHz, $CDCl_3$) δ (ppm): 9.17 (brs, 1H), 3.50 (s, 1H), 2.49 (s, 1H), 2.22 (d, J = 15.2 Hz, 1H), 1.97 (d, J = 15.2 Hz, 1H), 1.75–1.58 (m,2H), 1.52–1.42 (m, 2H), 1.40–1.34 (m, 1H), 1.34–1.24 (m, 1H); **^{13}C NMR** (101 MHz, $CDCl_3$) δ (ppm): 167.0, 38.6, 38.4, 37.6, 37.3, 27.4, 26.0.

HRMS (ESI) m/z $[M+Na]^+$ calculated for $C_7H_{11}NNaO^+$: 148.0733, found 148.0738.

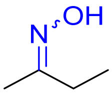

(*E/Z*)-Butan-2-one oxime (9a) [113] was synthesized according to the below procedure: ***rac*-C1** (10.1 mg, 1.0 mol%), hydroxylamine sulfate (164.2 mg, 1.0 mmol. 1.0 equiv.), and AcOH (2.8 mL) were added to a reaction tube containing ^{t}BuOH (2.0 mL), and the reaction tube was capped with a rubber stopper. Then, the solution was cooled down to 0 ºC in an ice bath, and *n*-butane (gas, stored in a balloon) was introduced into the reaction solution by a syringe needle for 1 h. Meanwhile, H_2O_2 (5.0 mmol, 5.0 equiv., 567 µL, 30% wt. in H_2O) in 2 mL of ^{t}BuOH was added dropwise with a syringe pump over 1 h under stirring at 0 ºC. The reaction mixture was stirred for another 30 min, and then quenched with Na_2SO_3 solid. Next, 1,3,5-trimethoxybenzene (168.2 mg, 1.0 mmol) was added to the reaction tube and the resulting mixture was basified with Na_2CO_3 for about 30 minutes at 0 ºC. After completion of the reaction, the solution was filtered and washed with DCM (2.0 mL). The reaction mixture was analyzed by GC (**75% yield**). Repeating the reaction again but without 1,3,5-trimethoxybenzene as an internal standard afforded, after extraction and removal of the solvent, a (*E*/*Z*)-**9a** isomer mixture as colorless oil (**40.1 mg, 46% yield**, ***E/Z* = *3:1***).

The E/Z isomers were confirmed by ^1H and ^{13}C NMR and the ratio of E/Z isomers was calculated by integration of ^1H and ^{13}C NMR spectra.

Major (*E*)-9a: ^{1}H NMR (400 MHz, $CDCl_3$) δ (ppm): 9.40 (brs, 1H), 2.20 (q, *J* = 7.6 Hz, 2H), 1.87 (s, 3H), 1.06 (t, *J* = 7.6 Hz, 3H); **^{13}C NMR** (101 MHz, $CDCl_3$) δ (ppm): 159.5, 29.2, 13.4, 10.8.

Minor (*Z*)-9a: ^{1}H NMR (400 MHz, $CDCl_3$) δ (ppm): 9.40 (brs, 1H), 2.38 (q, *J* = 7.6 Hz, 2H), 1.84 (s, 3H), 1.06 (t, *J* = 7.6 Hz, 3H); **^{13}C NMR** (101 MHz, $CDCl_3$) δ (ppm): 160.0, 22.0, 19.2, 9.9.

GC-MS (m/z): calculated for C_4H_9NO: 87.1, found 87.1.

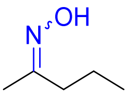

(*E/Z*)-Pentan-2-one oxime (10a) [116] was synthesized according to the below procedure: ***rac*-C1** (10.2 mg, 1.0 mol%), hydroxylamine sulfate (164.2 mg, 1.0 mmol. 1.0 equiv.), *n*-pentane (360.8

mg, 5 mmol), AcOH (2.8 mL) and ^{t}BuOH (2.0 mL) were added to a reaction tube. The mixture was then cooled down to 0 ºC in a cryogenic bath, and then H_2O_2 (5.0 mmol, 5.0 equiv., 567 µL, 30% wt. in H_2O) in 2 mL of ^{t}BuOH was added dropwise with a syringe pump over 1 h under stirring at 0 ºC without nitrogen protection. The reaction mixture was stirred for another 30 min, and then the reaction mixture was quenched with Na_2SO_3 solid. Next, 1,3,5-trimethoxybenzene (168.2 mg, 1.0 mmol) was added to the reaction tube and the resulting mixture was basified with Na_2CO_3 for about 30 minutes at 0 ºC in an ice bath. After completion of the reaction, the solution was filtered and washed with DCM (2.0 mL). The reaction mixture was analyzed by GC (**69% yield**). Repeating the reaction again but without 1,3,5-trimethoxybenzene as an internal standard afforded, after extraction and removal of the solvent, a (*E*/*Z*)-**10a** isomer mixture as colorless oil (**41.4 mg, 41% yield**, ***E/Z* = *3:1***).

The E/Z isomers were confirmed by ^{1}H and ^{13}C NMR and the *ratio of E/Z isomers was calculated by integration of ^{1}H and ^{13}C NMR spectra.*

Major (*E*)-10a: ^{1}H NMR (400 MHz, $CDCl_3$) δ (ppm): 8.51 (brs, 1H), 2.16 (t, *J* = 7.6 Hz, 2H), 1.87 (s, 3H), 1.58−1.49 (m, 2H), 0.92 (t, *J* = 7.6 Hz, 3H); **^{13}C NMR** (101 MHz, $CDCl_3$) δ (ppm): 158.5, 37.8, 19.7, 13.7, 13.5;

Minor (*Z*)-10a: ^{1}H NMR (400 MHz, $CDCl_3$) δ (ppm): 8.51 (brs, 1H), 2.35 (t, *J* = 8.0 Hz, 2H), 1.86 (s, 3H), 1.58−1.49 (m, 2H), 0.95 (t, *J* = 7.6 Hz, 3H); **^{13}C NMR** (101 MHz, $CDCl_3$) δ (ppm): 158.9, 30.7, 19.9, 19.0, 14.3.

GC-MS (m/z): calculated for $C_5H_{11}NO$: 101.1, found 101.1.

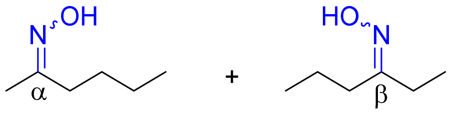

(*E/Z*)-Hexan-2-one oxime (11a-α-oxime) and **(*E/Z*)-hexan-3-one oxime (11a-β-oxime)** [117] was synthesized from *n*-hexane (430.9 mg, 5 mmol) and hydroxylamine sulfate (164.2 mg, 1.0 mmol. 1.0 equiv.), following a similar procedure to the synthesis of **(*E/Z*)-10a**. The product of **11a** was obtained as a mixture of two regioisomers with **58% GC yield**. Repeating the reaction again but without 1,3,5-trimethoxybenzene as an internal standard afforded, after extraction and removal of the solvent to afford a mixture of two regioisomers (*E*/*Z*)-**11a-α-oxime** and (*E*/*Z*)-**11a-β-oxime** as

colorless oil (**53.0 mg, 46% yield, β:α = 1:2.5**, ***α, E/Z = 3:1; β, E/Z =1:1***). The regioisomer (*E*/*Z*)-**11a-α-oxime** was isolated from the mixture in 30% yield (35 mg).

Site of oximation was assigned based on analysis of the ^{1}*H and* ^{13}*C NMR spectra of the mixture products with the isolated* (*E*/*Z*)-**11a-α-oxime**. *The E/Z isomers were confirmed by* ^{1}*H and* ^{13}*C NMR* and the *ratio of E/Z isomers was calculated by integration of* ^{1}*H and* ^{13}*C NMR spectra.*

^{1}H NMR (400 MHz, $CDCl_3$) δ (ppm): 9.56 (brs, 0.92H), 2.38–2.28 (m, 1.34H), 2.23–2.12 (m, 2.17 H), 1.85 (s, 1.99H), 1.84 (s, 0.66H), 1.56–1.42 (m, 2.88H), 1.36–1.22 (m, 3.03H), 1.06 (t, *J* = 7.2 Hz, 1.17 H), 0.95–0.86 (m, 4.55H);

^{13}C NMR (101 MHz, $CDCl_3$) δ (ppm): 162.7, 162.6, 159.0, 158.6, 35.8, 35.5, 29.6, 28.49, 28.46, 27.7, 27.5, 22.9, 22.3, 20.9, 19.8, 19.7, 19.2, 14.4, 13.9, 13.8, 13.4, 10.9, 10.1;

HRMS (ESI+): *m/z* $[M+H]^+$ calculated for $C_6H_{15}NO^+$: 116.1070, found 116.1071.

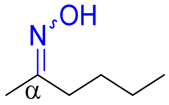

(*E/Z*)-Hexan-2-one oxime (**11a-α-oxime**) *isomer mixtures* (*E/Z* = 3:1):

Major (*E*)-11a-α-oxime: ^{1}H NMR (400 MHz, $CDCl_3$) δ (ppm): 8.79 (brs, 1H), 2.18 (t, *J* = 8.0 Hz, 2H), 1.87 (s, 3H), 1.53–1.43 (m, 2H), 1.39–1.27 (m, 2H), 0.91 (t, *J* = 7.2 Hz, 3H); **^{13}C NMR** (101 MHz, $CDCl_3$) δ (ppm): 158.7, 35.5, 28.5, 22.3, 13.8, 13.4.

Minor (*Z*)-11a-α-oxime: ^{1}H NMR (400 MHz, $CDCl_3$) δ (ppm): 8.79 (brs, 1H), 2.37 (t, *J* = 8.0 Hz, 2H), 1.86 (s, 3H), 1.53–1.43 (m, 2H), 1.39–1.27 (m, 2H), 0.92 (t, *J* = 7.2 Hz, 3H); **^{13}C NMR** (101 MHz, $CDCl_3$) δ (ppm): 159.1, 28.5, 27.7, 22.9, 19.8, 13.9.

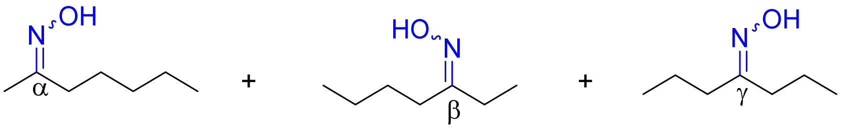

(*E/Z*)-Heptan-2-one oxime (12a-α-oxime), **(*E/Z*)-heptan-3-one oxime (12a-β-oxime)** and **heptan-4-one oxime (12a-γ-oxime) (12a)**[117] were synthesized from *n*-heptane (501.0 mg, 5 mmol) and hydroxylamine sulfate (164.2 mg, 1.0 mmol. 1.0 equiv.), following a similar procedure to the synthesis of **(*E/Z*)-10a.** The product **12a** was obtained as a mixture of three regioisomers with **50%**

GC yield. Repeating the reaction again but without 1,3,5-trimethoxybenzene as an internal standard afforded, after extraction and removal of the solvent to afford a mixture of three regioisomers (*E*/*Z*)-**12a-α-oxime**, (*E*/*Z*)-**12a-β-oxime** and **12a-γ-oxime** as colorless oil (**55.6 mg**, **43% yield**, **γ:β:α = 1:3.3:8.3**, ***α, E/Z = 3:1; β, E/Z = 1:1***), and the obtained product needs no further purification and was taken directly for NMR and HRMS analysis.

Site of oximation was assigned based on analysis of the ^{1}H and ^{13}C NMR of the mixture products with the synthesized (*E*/*Z*)-**12a-α-oxime**. The *E/Z isomers were confirmed by ^{1}H and ^{13}C NMR* and the *ratio of E/Z isomers was calculated by integration of ^{1}H and ^{13}C NMR spectra.*

^{1}H NMR (400 MHz, $CDCl_3$) δ (ppm): 2.38–2.28 (m, 1.67H), 2.23–2.12 (m, 2.57H), 1.86 (s, 2.10H), 1.84 (s, 0.71H), 1.55–1.42 (m, 3.71H), 1.37–1.22 (m, 6.29H), 1.07 (t, *J* = 7.6 Hz, 1.24H), 0.96–0.85 (m, 6.05H);

^{13}C NMR (101 MHz, $CDCl_3$) δ (ppm): 162.9, 162.8, 161.7, 159.0, 158.7, 36.2, 35.8, 33.5, 32.0, 31.4, 29.6, 28.7, 28.5, 27.9, 27.5, 27.4, 26.1, 25.2, 23.1, 22.51, 22.47, 20.9, 19.9, 19.7, 19.2, 14.0, 13.92, 13.89, 13.87, 13.4, 10.9, 10.2;

HRMS (ESI+): *m/z* $[M+Na]^+$ calculated for $C_7H_{15}NNaO^+$: 152.1046, found 152.1042.

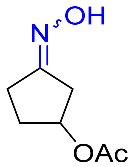

(*E/Z*)-3-(Hydroxyimino)cyclopentyl acetate (13a) [118] was synthesized by the general procedure **A** from cyclopentyl acetate (128.2 mg, 1 mmol) and purified by flash column chromatography on silica gel (gradient elution: Petroleum ether : EtOAc = 20:1 to 10:1) to afford (*E*/*Z*)-**13a** isomer mixture as white solid (**117.9 mg, 75% yield**);

The E/Z isomers were confirmed by ^{1}H and ^{13}C NMR and the *ratio of E/Z isomers* (***E/Z* = 1.6:1**) *was calculated by integration of ^{1}H and ^{13}C NMR spectra.*

^{1}H NMR (400 MHz, $CDCl_3$) δ (ppm): 8.07 (brs, 1H), 5.33–5.23 (m, 1H), 2.82–2.40 (m, 4H), 2.05–1.95 (m, 5H);

^{13}C NMR of major (*E*)-13a: (101 MHz, $CDCl_3$) δ (ppm): 170.8, 164.0, 74.0, 37.2, 30.5, 24.8, 21.3; **^{13}C NMR of minor (*Z*)-13a:** (101MHz, $CDCl_3$) δ (ppm): 170.8, 163.7, 74.0, 34.2, 31.0, 28.0, 21.3;

HRMS (ESI) *m/z* $[M+Na]^+$ calculated for $C_7H_{11}NNaO_3^+$: 180.0631, found 180.0639.

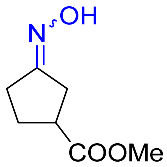

Methyl (*E/Z*)-3-(hydroxyimino)cyclopentane-1-carboxylate (14a) was synthesized by the general procedure **A** from methyl cyclopentanecarboxylate (128.2 mg, 1 mmol) and purified by flash column chromatography on silica gel (gradient elution: Petroleum ether : EtOAc = 30:1 to 10:1) to afford (*E*/Z)-**14a** isomer mixture as colorless oil (**127.3 mg, 81% yield**, ***E/Z =1:1***);

E/Z isomers were confirmed by ^{1}*H and* ^{13}*C NMR* and the *ratio of E/Z isomers was calculated by integration of* ^{1}*H and* ^{13}*C NMR spectra.*

^{1}H NMR (400 MHz, $CDCl_3$) δ (ppm): 9.58 (brs, 1H), 3.63 (s, 3H), 2.93–2.25 (m, 5H), 2.11–1.98 (m, 1H), 1.95–1.81 (m, 1H);

^{13}C NMR (101 MHz, $CDCl_3$) δ (ppm): 175.0, 174.8, 164.5, 164.3, 51.9, 42.5, 42.2, 33.8, 30.5, 29.8, 28.5, 28.0, 26.5;

HRMS (ESI) *m/z* $[M+Na]^+$ calculated for $C_7H_{11}NNaO_3^+$: 180.0631, found 180.0634.

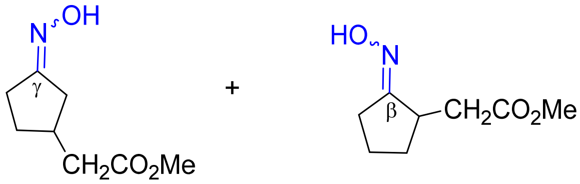

Methyl (*E/Z*)-2-(3-(hydroxyimino)cyclopentyl)acetate (15a-γ-oxime) and **methyl (*E/Z*)-2-(2-(hydroxyimino)cyclopentyl)acetate(15a-β-oxime) (15a)** was synthesized by the general procedure **A** from methyl 2-cyclopentylacetate (142.2 mg, 1 mmol) and purified by flash column chromatography on silica gel (gradient elution: Petroleum ether : EtOAc = 30:1 to 10:1) to afford a mixture of two regioisomers (*E*/*Z*)-**15a-γ-oxime** and (*E*/*Z*)-**15a-β-oxime** as colorless oil (**142.0 mg, 83% yield**, **β:γ = 1:4**, γ, ***E/Z* = 1:1, β, *E/Z* = 3:1**). The regioisomer (*E*/*Z*)-**15a-γ-oxime** was isolated from the mixture in 64% yield (110 mg).

Site of oximation was assigned based on analysis of the ^{1}*H and* ^{13}*C NMR spectra of the mixture products with the isolated* (*E*/*Z*)-**15a-γ-oxime**. *The E/Z isomers were confirmed by* ^{1}*H and* ^{13}*C NMR* and the *ratio of E/Z isomers was calculated by integration of* ^{1}*H and* ^{13}*C NMR spectra.*

^{1}H NMR (400 MHz, $CDCl_3$) δ (ppm): 9.00 (brs, 0.7H), 3.66 (s, 3H), 2.96–2.50 (m, 1.70H), 2.49–2.28 (m, 3.62H), 2.11−1.92(m, 1.78H), 1.92−1.49 (m, 0.92H), 1.44−1.17 (m, 1.33H);
^{13}C NMR (101 MHz, $CDCl_3$) δ (ppm): 173.0(2C), 172.98, 172.96, 167.4(2C), 165.8, 165.6, 51.7, 39.7, 39.4, 39.0, 37.0, 36.5, 35.3, 34.9, 33.5, 32.1, 31.2, 31.1, 30.5, 30.2, 27.2, 26.6, 22.5;
HRMS (ESI) *m/z* $[M+Na]^+$ calculated for $C_8H_{13}NNaO_3^+$: 194.0788, found 194.0788.

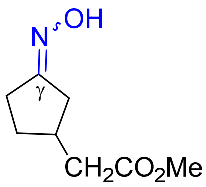

Methyl (*E/Z*)-2-(3-(hydroxyimino)cyclopentyl)acetate (15a-γ-oxime) isomer mixtures (*E/Z* = 1:1): **^{1}H NMR** (400 MHz, $CDCl_3$) δ (ppm): 9.40 (brs, 1H), 3.64 (s, 3H), 2.80 (dd, *J* = 18.4, 7.2 Hz, 0.55H), 2.65–2.56 (m, 1.2H), 2.47–2.30 (m, 4.25H), 2.06–1.97(m, 2H), 1.43–1.31(m, 1H); **^{13}C NMR** (101 MHz, $CDCl_3$) δ (ppm): 173.00, 172.99, 165.7, 165.6, 51.7, 39.3, 38.9, 36.9, 35.2, 34.8, 33.5, 31.1, 30.5, 30.1, 26.7.

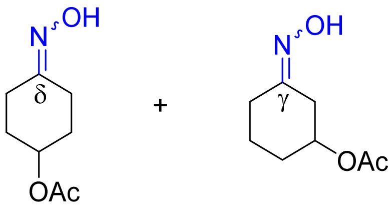

4-(Hydroxyimino)cyclohexyl acetate (16a-δ-oxime) and **(*E/Z*)-3-(hydroxyimino)cyclohexyl acetate (16a-γ-oxime) (16a)** was synthesized by the general procedure **A** from cyclohexyl acetate (142.2 mg, 1 mmol) and purified by flash column chromatography on silica gel (gradient elution: Petroleum ether : EtOAc = 30:1 to 10:1) to afford a mixture of two regioisomers **16a-δ-oxime** and (*E*/*Z*)-**16a-γ-oxime** as white solid (**138.7 mg, 81% yield**, **γ:δ = 1.5:1**, **γ, *E/Z* = 1:1**). *Site of oximation was assigned based on analysis of the ^{1}H and ^{13}C NMR spectra of the mixture products with the synthesized* **16a-δ-oxime**. *The E/Z isomers were confirmed by ^{1}H and ^{13}C NMR* and the *ratio of E/Z isomers was calculated by integration of ^{1}H and ^{13}C NMR spectra.*
^{1}H NMR (400 MHz, $CDCl_3$) δ (ppm): 9.13 (brs, 1H), 5.01–4.93 (m, 1H), 2.90–2.71 (m, 1H), 2.63–2.38 (m, 2H), 2.55–2.17 (m, 1H), 2.07–2.00 (m, 3H), 1.94–1.50 (m, 4H);

^{13}C NMR (101 MHz, $CDCl_3$) δ (ppm): 170.7, 170.5(2C), 158.4, 157.4, 156.9, 71.1, 70.5, 70.4, 36.9, 31.1, 30.8, 30.5, 30.4, 29.7, 29.5, 28.1, 23.7, 21.8, 21.4, 21.3, 20.7, 20.5;

HRMS (ESI) *m/z* $[M+Na]^+$ calculated for $C_8H_{13}NNaO_3^+$: 194.0788, found 194.0792.

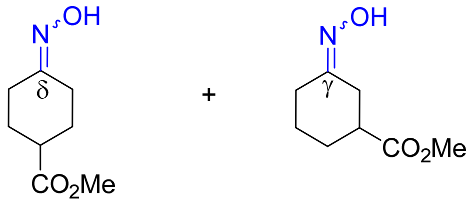

Methyl 4-(hydroxyimino)cyclohexane-1-carboxylate (17a-δ-oxime) and **methyl (*E/Z*)-3-(hydroxyimino)cyclohexane-1-carboxylate (17a-γ-oxime) (17a)** was synthesized by the general procedure **A** from methyl cyclohexanecarboxylate (142.2 mg, 1 mmol) and purified by flash column chromatography on silica gel (gradient elution: Petroleum ether : EtOAc = 20:1 to 5:1) to afford a mixture of two regioisomers **17a-δ-oxime** and (*E*/*Z*)-**17a-γ-oxime** as colorless oil (**147.2 mg, 86% yield**, γ:δ **= 2.7:1**, γ, ***E/Z*** **= 1:1**).

Site of oximation was assigned based on analysis of the ^{1}H and ^{13}C NMR spectra of the mixture products with the synthesized **17a-δ-oxime**. *The E/Z isomers were confirmed by ^{1}H and ^{13}C NMR* and the *ratio of E/Z isomers was calculated by integration of ^{1}H and ^{13}C NMR spectra.*

^{1}H NMR (400 MHz, $CDCl_3$) δ (ppm): 9.57 (brs, 1H), 3.65–3.62 (m, 3H), 3.37 (d, *J* = 14.4 Hz, 0.36H, *γ*-**17a**), 3.16–3.06 (m, 0.64H, (*γ*+*δ*)-**17a**), 2.58–2.20 (m, 2H), 2.16–1.30 (m, 6H);

^{13}C NMR (101 MHz, $CDCl_3$) δ (ppm) 175.2, 175.0, 174.9, 158.7, 158.4, 158.0, 51.9, 51.8, 43.1, 41.9, 41.8, 33.8, 31.2, 30.3, 28.7, 28.6, 28.5 27.4, 26.3, 25.1, 24.0, 23.8, 22.8;

HRMS (ESI) *m/z* $[M+Na]^+$ calculated for $C_8H_{13}NNaO_3^+$: 194.0788, found 194.0790.

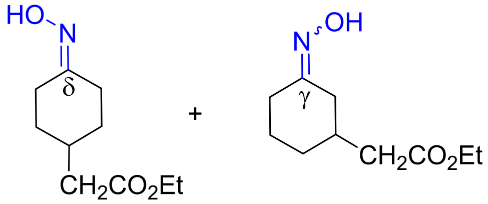

Ethyl 2-(4-(hydroxyimino)cyclohexyl)acetate (18a-δ-oxime) and **ethyl (*E/Z*)-2-(3-(hydroxyimino) cyclohexyl)acetate (18a-γ-oxime) (18a)** was synthesized by the general procedure **A** from ethyl 2-cyclohexylacetate (170.3 mg, 1 mmol) and purified by flash column

chromatography on silica gel (gradient elution: Petroleum ether : EtOAc = 20:1 to 5:1) to afford a mixture of two regioisomers **18a-δ-oxime** and (*E*/*Z*)-**18a-γ-oxime** as colorless oil (**173.3 mg, 87% yield**, **γ:δ = 2.5:1**, **γ, *E/Z* = 1:1**).

Site of oximation was assigned based on analysis of the ^{1}H and ^{13}C NMR spectra of the mixture products with the synthesized **18a-δ-oxime**. *The E/Z isomers were confirmed by ^{1}H and ^{13}C NMR* and the *ratio of E/Z isomers was calculated by integration of ^{1}H and ^{13}C NMR spectra.*

^{1}H NMR (400 MHz, $CDCl_3$) δ (ppm): 9.23 (brs, 1H), 4.13–4.00 (m, 2H), 3.26–3.05 (m, 1H), 2.44–2.16 (m, 3H), 2.16–1.94 (m, 2H), 1.91–1.73 (m, 3H), 1.65–1.37 (m, 1H), 1.24–1.70 (m, 4H); **^{13}C NMR** (101 MHz, $CDCl_3$) δ (ppm): 172.7, 172.32, 172.30, 159.6, 159.3, 159.1, 60.4, 41.1, 40.94, 40.85, 37.8, 35.1, 33.99, 33.95, 32.6, 31.7, 31.5, 31.3, 31.1, 30.2, 25.3, 24.0, 23.9, 14.27, 14.25;

HRMS (ESI) *m/z* $[M+Na]^+$ calculated for $C_{10}H_{17}NNaO_3^+$: 222.1101, found 222.1103.

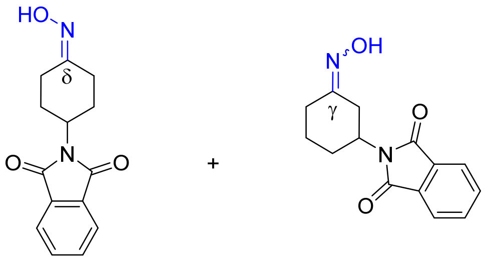

2-(4-(Hydroxyimino)cyclohexyl)isoindoline-1,3-dione (19a-δ-oxime) and **(*E/Z*)-2-(3-(hydroxyimino) cyclohexyl)isoindoline-1,3-dione (19a-γ-oxime) (19a)** was synthesized by the general procedure **A** from 2-cyclohexylisoindoline-1,3-dione (229.3 mg, 1 mmol) and purified by flash column chromatography on silica gel (gradient elution: Petroleum ether : EtOAc = 10:1 to 3:1) to afford a mixture of two regioisomers **19a-δ-oxime** and (*E*/*Z*)-**19a-γ-oxime** as white solid (**111.1 mg, 43% yield**, **γ:δ = 2.5:1**, **γ, *E/Z* = 1:1**), and remaining starting material (102.3 mg, 45% remaining);

Site of oximation was assigned based on analysis of the ^{13}C NMR spectra of the mixture products. The E/Z isomers were confirmed by ^{13}C NMR and the ratio of E/Z isomers was calculated by integration of ^{1}H and ^{13}C NMR spectra.

^{1}H NMR (400 MHz, $CDCl_3$) δ (ppm): 9.05 (brs, 1H), 7.85–7.77 (m, 2H), 7.72–7.65 (m, 2H), 4.42–4.16 (m, 1H), 3.52–3.43 [m, 0.7H, (*γ*+δ)-**19a**], 3.38 (d, *J* = 14.4 Hz, 0.25H, *γ*-**19a**), 3.09 (t,

J = 12.8 Hz, 0.25H, *γ*-**19a**), 2.73 (t, *J* = 12.8 Hz, 0.25H, *γ*-**19a**), 2.60–2.32 (m, 2.39 H, (*γ*+δ)-**19a**), 2.29–2.10 (m, 0.86H, (*γ*+δ)-**19a**), 2.04–1.70 [m, 2.94H, (*γ*+δ)-**19a**], 1.58–1.40 (m, 0.64H, (*γ*+δ)-**19a**);

^{13}C NMR (101 MHz, $CDCl_3$) δ (ppm): 168.3(δ-**19a**), 168.24(*γ*-**19a**), 168.22(*γ*-**19a**), 158.3 (δ-**19a**), 157.74(*γ*-**19a**), 157.67(*γ*-**19a**), 134.11, 134.07, 132.0, 131.9, 123.34, 123.31, 123.2, 49.9, 49.6, 48.8, 35.1, 30.9, 30.8, 29.4, 29.3, 29.2, 27.9, 27.7, 24.3, 23.4, 23.2, 23.1; **HRMS** (ESI+): *m/z* $[M+Na]^+$ calculated for $C_{14}H_{14}N_2NaO_3^+$: 281.0897, found 281.0892.

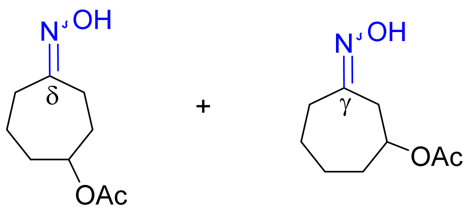

(*E/Z*)-4-(hydroxyimino)cycloheptyl acetate (20a-δ-oxime) and **(*E/Z*)-3-(hydroxyimino) cycloheptyl acetate (20a-γ-oxime) (20a)** was synthesized by the general procedure **A** from cycloheptyl acetate (156.2 mg, 1 mmol) and purified by flash column chromatography on silica gel (gradient elution: Petroleum ether : EtOAc = 30:1 to 10:1) to afford a mixture of two regioisomers (*E*/*Z*)-**20a-δ-oxime** and (*E*/*Z*)-**20a-γ-oxime** as colorless oil (**148.2 mg, 80% yield**, **γ:δ = 1:2**, ***δ*, *E/Z* = 1:1; *γ*, *E/Z* = 1:1**).

Site of oximation was assigned based on analysis of the ^{13}C NMR spectra of the mixture products. The E/Z isomers were confirmed by ^{13}C NMR and the ratio of E/Z isomers was calculated by integration of ^{13}C NMR spectrum.

^{1}H NMR (400 MHz, $CDCl_3$) δ (ppm): 9.08 (brs, 1H), 5.10–4.78 (m, 1H), 2.90–2.24 (m, 4H), 2.04–1.98 (m, 3H), 1.95–1.46 (m, 6H);

^{13}C NMR (101 MHz, $CDCl_3$) δ (ppm): 170.5(δ-**20a**), 170.44(δ-**20a**), 170.38(*γ*-**20a**), 170.3(*γ*-**20a**), 163.0(δ-**20a**), 162.5(δ-**20a**), 159.2(*γ*-**20a**), 158.9(*γ*-**20a**), 74.4, 73.7, 71.7, 71.2, 38.4, 35.7, 35.0, 34.8, 34.7, 33.9, 33.7, 33.1, 32.3, 29.5, 28.5, 28.3, 27.7, 27.0, 25.3, 24.4, 23.9, 22.7, 21.8, 21.44, 21.39, 21.3, 18.8;

HRMS (ESI) *m/z* $[M+Na]^+$ calculated for $C_9H_{15}NNaO_3^+$: 208.0944, found 208.0951.

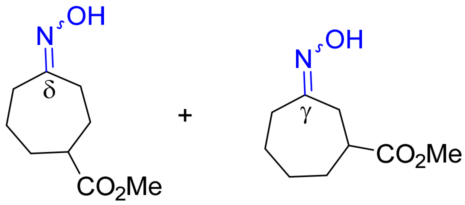

Methyl (*E/Z*)-4-(hydroxyimino)cycloheptane-1-carboxylate (21a-δ-oxime) and **methyl (*E/Z*)-3-(hydroxyimino)cycloheptane-1-carboxylate (21a-γ-oxime) (21a)** was synthesized by the general procedure **A** from methyl cycloheptanecarboxylate (156.2 mg, 1 mmol) and purified by flash column chromatography on silica gel (gradient elution: Petroleum ether : EtOAc = 20:1 to 5:1) to afford a mixture of two regioisomers (*E*/*Z*)-**21a-δ-oxime** and (*E*/*Z*)-**21a-γ-oxime** as colorless oil (**155.6 mg, 84% yield**, **γ:δ = 1:1.3**, ***δ, E/Z*** **= 1:1;** ***γ, E/Z*** **= 1:1**).

Site of oximation was assigned based on the analysis of the ^{13}C NMR spectrum of the mixture products. The E/Z isomers were confirmed by ^{13}C NMR and the ratio of E/Z isomers was calculated by integration of ^{13}C NMR spectrum.

^{1}H NMR (400 MHz, $CDCl_3$) δ (ppm): 9.51 (brs, 0.78H), 3.65–3.61 (m, 3H), 2.97 (d, *J* = 15.6 Hz, 0.30H), 2.78–2.40 (m, 4.09H), 2.35–2.20 (m, 1.16H), 2.05–1.33 (m, 6.31H);

^{13}C NMR (101 MHz, $CDCl_3$) δ (ppm): 176.2(δ-**21a**), 176.0((*δ*+*γ*)-**21a**), 175.7(*γ*-**21a**), 163.0(δ-**21a**), 162.8(δ-**21a**), 161.4(*γ*-**21a**), 160.9(*γ*-**21a**), 51.9(*γ*-**21a**), 51.8(*γ*-**21a**), 51.73(δ-**21a**), 51.71(δ-**21a**), 46.6, 46.4, 44.1, 41.5, 35.6, 33.5, 33.12, 33.09, 33.0, 32.6, 32.5, 31.5, 30.6, 29.6, 28.3, 28.24, 28.19, 28.0, 26.9, 26.8, 26.6, 25.6, 23.9, 22.6; **HRMS** (ESI) *m/z* $[M+Na]^+$ calculated for $C_9H_{15}NNaO_3^+$: 208.0944, found 208.0949.

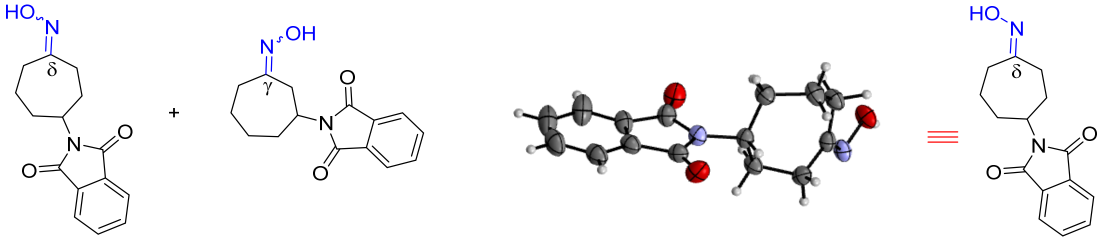

(*E/Z*)-2-(4-(hydroxyimino)cycloheptyl)isoindoline-1,3-dione (22a-δ-oxime) and **(*E/Z*)-2-(3-(hydroxyimino)cycloheptyl)isoindoline-1,3-dione (22a-γ-oxime) (22a)** was synthesized by the general procedure **A** from 2-cycloheptylisoindoline-1,3-dione (243.3 mg, 1 mmol) and purified by flash column chromatography on silica gel (gradient elution: Petroleum ether : EtOAc = 10:1 to

1:1) to afford two separated regioisomers (*E*/*Z*)-**22a-δ-oxime** (**114.4 mg, 42% yield**, ***E/Z*** **= 1:1**) and (*E*/*Z*)-**22a-γ-oxime** (**49.0 mg, 18% yield**, ***E/Z*** **= 1:1**) as white solid (**γ:δ = 1:2.3**), with remaining starting material <5%.

The E/Z isomers were confirmed by ^{1}H and ^{13}C NMR and the X-ray structure of the E isomer, and the ratio of E/Z isomers was determined by integration of ^{1}H and ^{13}C NMR spectra.

(*E*/*Z*)-**22a-δ-oxime:**

^{1}H NMR (400 MHz, $CDCl_3$) δ (ppm): 9.39 (brs, 1H), 7.79–7.75 (m, 2H), 7.68–7.64 (m, 2H), 4.24–4.16 (m, 0.5H), 4.11–4.03 (m, 0.5H), 2.90–2.75 (m, 1H), 2.63–2.15 (m, 5H), 2.05–1.78 (m, 3H), 1.67–1.38 (m, 1H); **^{13}C NMR** (101 MHz, $CDCl_3$) δ (ppm):168.1, 168.0, 162.7, 162.5, 133.9 (2C), 132.0, 131.9, 123.2 (2C), 53.7 (2C), 34.0, 33.7, 32.9, 31.1, 30.7, 29.4, 28.2, 25.9, 24.9, 22.0; **HRMS** (ESI+): *m/z* $[M+Na]^+$ calculated for $C_{15}H_{16}N_2NaO_3^+$: 295.1053, found 295.1053.

(*E*/*Z*)-**22a-γ-oxime:**

^{1}H NMR (400 MHz, $CDCl_3$) δ (ppm): 8.80 (brs, 1H), 7.83–7.77 (m, 2H), 7.71–7.65 (m, 2H), 4.49–4.39 (m, 0.5H), 4.26–4.17 (m, 0.5H), 3.42–3.30 (m, 1H), 2.90–2.63 (m, 1H), 2.61–2.20 (m, 3H), 2.04–1.80 (m, 3H), 1.57–1.21 (m, 2H); **^{13}C NMR** (101 MHz, $CDCl_3$) δ (ppm): 168.03, 167.96, 160.4, 159.5, 134.0 (2C), 132.05, 132.03, 123.30, 123.27, 51.5, 49.0, 38.5, 35.5, 34.6, 33.6, 32.3, 28.4, 27.5, 27.2, 26.5, 23.6; **HRMS** (ESI+): *m/z* $[M+Na]^+$ calculated for $C_{15}H_{16}N_2NaO_3^+$: 295.1053, found 295.1059.

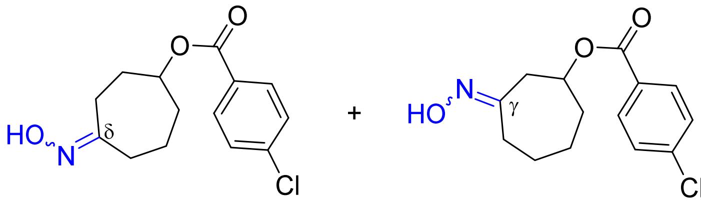

(*E*/*Z*)-4-(hydroxyimino)cycloheptyl 4-chlorobenzoate (23a-δ-oxime) and **(*E*/*Z*)-3-(hydroxyimino)cycloheptyl 4-chlorobenzoate (23a-γ-oxime) (23a)** was synthesized by the general procedure **A** from cycloheptyl 4-chlorobenzoate (252.7 mg, 1 mmol) and purified by flash column chromatography on silica gel (gradient elution: Petroleum ether : EtOAc = 40:1 to 20:1 to 10:1) to afford two separated regioisomers (*E*/*Z*)-**23a-δ-oxime** (**132.4 mg, 47% yield**, ***E/Z*** **= 1:1**) and (*E*/*Z*)-**23a-γ-oxime** (**78.9 mg, 28% yield**, ***E/Z*** **= 1:1**) as white solid (**γ:δ = 1:1.7**), and remaining starting material (27.8 mg, 11%);

The E/Z isomers *were* confirmed by *^{1}H and ^{13}C NMR and the ratio of E/Z* isomers *was determined by integration of ^{1}H and ^{13}C NMR spectra.*

(*E*/*Z*)-**23a-δ-oxime:**

^{1}H NMR (400 MHz, $CDCl_3$) δ (ppm): 9.44 (brs, 1H), 7.97–7.92 (m, 2H), 7.40–7.35 (m, 2H), 5.22–5.16 (m, 0.5H), 5.14–5.08 (m, 0.5H), 2.84–2.74 (m, 0.5H), 2.66–2.53 (m, 2H), 2.48–2.30 (m, 1.5H), 2.06–1.82 (m, 5H), 1.70–1.58 (m, 1H); **^{13}C NMR** (101 MHz, $CDCl_3$) δ (ppm): 164.89, 164.85, 162.9,162.5, 139.38, 139.36, 131.01, 131.00, 129.1, 128.74, 128.72, 75.0, 74.3, 34.7, 34.6, 33.1, 32.2, 29.4, 28.3, 27.7, 22.7, 21.8, 18.8; **HRMS** (ESI) *m/z* $[M+Na]^+$ calculated for $C_{14}H_{16}ClNNaO_3^+$: 304.0711, found 304.0708.

(*E*/*Z*)-**23a-γ-oxime:**

^{1}H NMR (400 MHz, $CDCl_3$) δ (ppm): 8.80 (brs, 1H), 7.98– 7.90 (m, 2H), 7.41–7.35 (m, 2H), 5.35–5.27 (m, 0.5H), 5.25–5.18 (m, 0.5H), 2.93 (d, *J* = 6.4 Hz, 0.80H), 2.84–2.78 (m, 0.6H), 2.73–2.67 (m, 0.6H), 2.63–2.48 (m, 1.5H), 2.40–2.57 (m, 0.50H), 2.04–1.57 (m, 6H); **^{13}C NMR** (101 MHz, $CDCl_3$) δ (ppm): 165.0, 164.8, 159.2, 158.8, 139.5, 139.4, 131.13, 131.11, 129.08, 129.05, 128.80, 128.75, 72.5, 72.0, 38.4, 35.6, 35.0, 34.1, 33.6, 28.6, 27.1, 25.1, 24.5, 24.0; **HRMS** (ESI) *m/z* $[M+Na]^+$ calculated for $C_{14}H_{16}ClNNaO_3^+$:304.0711, found 304.0708.

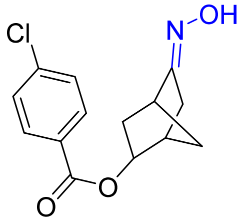

***endo*/*exo*-5-(Hydroxyimino)bicyclo[2.2.1]heptan-2-yl 4-chlorobenzoate (24a)** was synthesized by the general procedure **A** from *endo*/*exo*–bicyclo[2.2.1]heptan-2-yl 4-chlorobenzoate (250.7 mg, 1 mmol, *endo*/*exo* = 9:1) and purified by flash column chromatography on silica gel (gradient elution: Petroleum ether : EtOAc = 20:1 to 10:1) to afford a mixture of *endo*/*exo*-**24a** isomers as colorless oil (**176.2 mg, 63% yield, *endo*/*exo* = 9:1**) and remaining starting material (57.6 mg, 23%)*;*

*The endo/exo isomers were confirmed by ^{1}H and ^{13}C NMR and the ratio of endo/exo isomers is the same as that of **24** which was determined by integration of ^{1}H and ^{13}C NMR spectra.*

Major *endo*-24a: ^{1}H NMR (400 MHz, $CDCl_3$) δ (ppm): 8.61 (brs, 1H), 7.94 (d, *J* = 8.4 Hz, 2H), 7.40 (d, *J* = 8.8 Hz, 2H), 5.36–5.30 (m, 1H), 2.94–2.90 (m, 2H), 2.79–2.60 (m, 1H), 2.43–2.29 (m, 2H), 1.72–1.65 (m, 2H), 1.49 (d, *J* = 14.0 Hz, 1H); **^{13}C NMR** (101 MHz, $CDCl_3$) δ (ppm): 166.2, 165.7, 139.7, 131.1, 128.9, 128.6, 74.6, 42.5, 39.7, 37.8, 35.4, 27.4.

Minor *exo*-24a: ^{1}H NMR (400 MHz, $CDCl_3$) δ (ppm): 8.61 (brs, 1H), 7.94 (d, *J* = 8.4 Hz, 2H), 7.40 (d, *J* = 8.8 Hz, 2H), 5.01–4.95 (m, 1H), 2.94–2.90 (m, 2H), 2.79–2.60 (m, 1H), 2.22–2.10 (m, 2H), 1.86–1.81 (m, 2H), 1.57 (d, *J* = 14.0 Hz, 1H); **^{13}C NMR** (101 MHz, $CDCl_3$) δ (ppm): 165.4, 165.3, 139.7, 131.1, 128.9, 128.6, 74.6, 41.5, 41.1, 36.9, 36.2, 30.1.

HRMS (ESI) *m/z* $[M+Na]^+$ calculated for $C_{14}H_{14}ClNNaO_3^+$: 302.0554, found 302.0564;

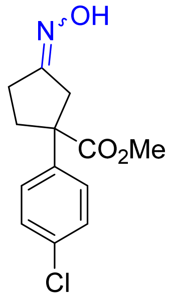

Methyl (*E/Z*)-1-(4-chlorophenyl)-3-(hydroxyimino)cyclopentane-1-carboxylate (25a) was synthesized by the general procedure **A** from methyl 1-(4-chlorophenyl)cyclopentane-1-carboxylate (238.7 mg, 1 mmol) and purified by flash column chromatography on silica gel (gradient elution: Petroleum ether : EtOAc = 20:1 to 5:1) to afford (*E*/Z)-**25a** isomer as colorless oil (**179.4 mg, 67% yield**, ***E/Z*** **= 1.2:1**)*;*

The E/Z isomers were confirmed by ^{13}C NMR and ratio of E/Z isomers was determined by integration of ^{13}C NMR spectrum.

^{1}H NMR (400 MHz, $CDCl_3$) δ (ppm): 9.17 (brs, 1H), 7.35–7.22 (m, 4H), 3.68–3.58 (m, 3.4H), 3.37 (d, *J* = 16.4 Hz, 0.6H), 2.88–2.72 (m, 2H), 2.67–2.44 (m, 2H), 2.15–1.99 (m, 1H); **^{13}C NMR of (*E*)-25a** (101 MHz, $CDCl_3$) δ (ppm):174.5, 163.5, 139.6, 133.5, 128.9, 128.1, 56.3, 53.0, 40.2, 34.1, 25.9; **^{13}C NMR of (*Z*)-25a** (101 MHz, $CDCl_3$) δ (ppm):174.7, 163.1, 140.1, 133.5, 128.8, 128.1, 56.2, 53.0, 37.4, 34.5, 28.9;

HRMS (ESI) *m/z* $[M+Na]^+$ calculated for $C_{13}H_{14}ClNNaO_3^+$: 290.0554, found 290.0563.

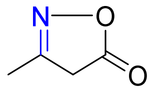

3-Methylisoxazol-5(4*H*)-one (26a') [119] was synthesized by the general procedure **A** from methyl butyrate (102.2 mg, 1 mmol) and purified by flash column chromatography on silica gel (gradient elution: Petroleum ether : EtOAc =50:1 to 5:1) to afford the product **26a'** as colorless oil (**23.8 mg, 24% yield**).

^{1}H NMR (400 MHz, $CDCl_3$) δ (ppm): 3.39 (s, 2H), 2.16 (s, 3H);

^{13}C NMR (101 MHz, $CDCl_3$) δ (ppm): 175.3 (C=O), 163.5 (C=N), 37.1, 14.9.

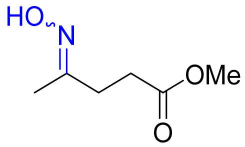

Methyl (*E/Z*)-4-(hydroxyimino)pentanoate (27a) was synthesized by the general Procedure **A** from methyl pentanoate (116.2 mg, 1 mmol). After completion of the reaction, the reaction solution was extracted with DCM, and the solvent DCM was removed to afford the product (*E/Z*)-**27a** isomer as colorless oil (**69.7 mg, 48% yield, *E/Z =3:1***); and the obtained product needs no further purification and was taken directly for NMR and HRMS analysis.

The E/Z isomers were confirmed by ^{1}H and ^{13}C NMR and the *ratio of E/Z isomers was calculated by integration of ^{1}H and ^{13}C NMR spectra.*

Major (*E*)-27a: ^{1}H NMR (400 MHz, $CDCl_3$) δ (ppm): 8.14 (brs, 1H), 3.68 (s, 3H), 2.55–2.51 (m, 4H), 1.89 (s, 3H); **^{13}C NMR** (101 MHz, $CDCl_3$) δ (ppm): 173.4, 156.8, 51.9, 31.1, 30.3, 14.1;

Minor (*Z*)-27a: ^{1}H NMR (400 MHz, $CDCl_3$) δ (ppm): 8.14 (brs, 1H), 3.69 (s, 3H), 2.68–2.56 (m, 4H), 1.89 (s, 3H); **^{13}C NMR** (101 MHz, $CDCl_3$) δ (ppm): 173.4, 157.5, 52.0, 29.9, 24.4, 20.3;

HRMS (ESI+): *m/z* $[M+Na]^+$ calculated for $C_6H_{11}NNaO_3^+$: 168.0631 found 168.0629.

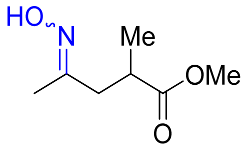

Methyl (*E/Z*)-4-(hydroxyimino)-2-methylpentanoate (28a) was synthesized by the general procedure **A** from methyl 2-methylpentanoate (130.2 mg, 1 mmol). After completion of the reaction, the reaction solution was extracted with DCM, and the solvent DCM was removed to afford the product of (*E/Z*)-**28a** isomer as colorless oil (**36.6 mg, 23% yield, *E/Z =3:1***). The obtained product needs no further purification and was taken directly for NMR and HRMS analysis.

The E/Z isomers were confirmed by ^{1}H and ^{13}C NMR and the *ratio of E/Z isomers was calculated by integration of ^{1}H and ^{13}C NMR spectra.*

Major (*E*)-28a : ^{1}H NMR (400 MHz, $CDCl_3$) δ (ppm): 8.22 (brs, 1H), 3.67 (s, 3H), 2.78 (dd, *J* = 14.0, 6.8 Hz, 1H), 2.62–2.55 (m, 1H), 2.27 (dd, *J* = 14.8, 6.8 Hz, 1H), 1.87 (s, 3H), 1.17 (d, *J* = 6.8 Hz, 3H); **^{13}C NMR** (101 MHz, $CDCl_3$) δ (ppm): 176.4, 156.3, 52.0, 39.6, 36.7, 17.2, 14.0;

Minor (*Z*)-28a: ^{1}H NMR (400 MHz, $CDCl_3$) δ (ppm): 8.22 (brs, 1H), 3.68 (s, 3H), 2.90 (dd, *J* = 14.4, 7.2 Hz, 1H), 2.62–2.55 (m, 1H), 2.27 (dd, *J* = 14.8, 6.8 Hz, 1H), 1.87 (s, 3H), 1.19 (d, *J* = 6.8 Hz, 3H); **^{13}C NMR** (101 MHz, $CDCl_3$) δ (ppm): 176.4, 156.7, 52.0, 36.4, 32.7, 20.7, 17.4;

HRMS (ESI+): *m/z* $[M+Na]^+$ calculated for $C_7H_{13}NNaO_3^+$: 182.0788 found 182.0790.

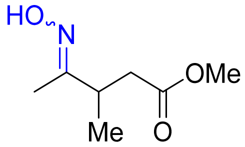

Methyl (*E/Z*)-4-(hydroxyimino)-3-methylpentanoate (29a) was synthesized by the general Procedure **A** from methyl 3-methylpentanoate (130.2 mg, 1 mmol). After completion of the reaction, the reaction solution was extracted with DCM, and the solvent DCM was removed to afford the product of (*E/Z*)**-29a** isomer as colorless oil (**51.2 mg, 32% yield**, ***E/Z* =*10:1***). The obtained product needs no further purification and was taken directly for NMR and HRMS analysis. *The E/Z isomers were confirmed by ^{1}H and ^{13}C NMR*, and the *ratio of E/Z isomers was calculated by integration of ^{1}H and ^{13}C NMR spectra.*

Major (*E*)-29a: ^{1}H NMR (400 MHz, $CDCl_3$) δ (ppm): 7.83 (brs, 1H), 3.67 (s, 3H), 2.91–2.79 (m, 1H), 2.62 (dd, *J* = 15.6, 7.2 Hz,1H), 2.33 (dd, *J* = 15.6, 7.6 Hz, 1H), 1.87 (s, 1H), 1.13 (d, *J* = 6.8 Hz, 3H); **^{13}C NMR** (101 MHz, $CDCl_3$) δ (ppm): 172.8, 160.5, 51.8, 38.4, 36.7, 18.1, 12.1;

Minor (*Z*)-29a: ^{1}H NMR (400 MHz, $CDCl_3$) δ (ppm): 7.83 (brs, 1H), 3.68 (s, 3H), 2.97–2.90 (m, 1H), 2.56 (dd, *J* = 15.6, 7.2 Hz, 1H), 2.39 (dd, *J* = 15.6, 7.6 Hz, 1H), 1.81 (s, 1H), 1.11 (d, *J* = 6.8 Hz, 3H);

HRMS (ESI+): *m/z* $[M+Na]^+$ calculated for $C_7H_{13}NNaO_3^+$: 182.0788 found 182.0789.

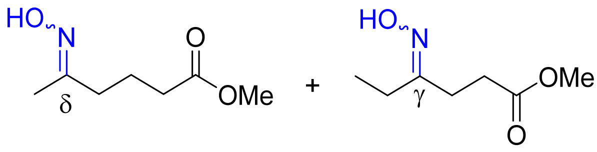

Methyl (*E/Z*)-5-(hydroxyimino)hexanoate (30a-δ-oxime) and **methyl (*E/Z*)-4-(hydroxyimino)hexanoate (30a-γ-oxime) (30a)** was synthesized by the general procedure **A** from methyl hexanoate (130.2 mg, 1 mmol) and purified by flash column chromatography on silica gel (gradient elution: Petroleum ether : EtOAc = 40:1 to 10:1) to afford a mixture of two regioisomers (*E*/*Z*)-**30a-δ-oxime** and (*E*/*Z*)-**30a-γ-oxime** as colorless oil (**100.3 mg, 63% yield**, **γ:δ = 1:5**, ***γ, E/Z=1:1; δ, E/Z=3:1***). The regioisomer (*E*/Z)-**30a-δ-oxime** was isolated from the mixture in 51% yield (81 mg).

Site of oximation was assigned based on analysis of the ^{1}H and ^{13}C NMR spectra of the mixture products with the isolated (*E*/*Z*)-**30a-δ-oxime**; *The E/Z isomers were confirmed by ^{1}H and ^{13}C NMR* and the *ratio of E/Z isomers was calculated by integration of ^{1}H and ^{13}C NMR spectra.*

^{1}H NMR (400 MHz, $CDCl_3$) δ (ppm): 8.92 (brs, 1H), 3.66 (s, 3H), 2.43–2.29 (m, 2.5H), 2.21 (t, *J* = 7.6 Hz, 1.5H), 1.89–1.77 (m, 5H);

^{13}C NMR (101 MHz, $CDCl_3$) δ (ppm): 173.8(4C), 161.0, 160.9, 158.0, 157.7, 51.85, 51.70, 51.67, 35.2, 33.8, 33.3, 30.3, 30.0, 29.8, 28.7, 28.0, 27.9, 23.5, 21.5, 20.9, 19.9, 13.5, 10.7, 10.1;

HRMS (ESI+): *m/z* calculated for $C_7H_{13}NNaO_3^+$: 182.0788 found 182.0791;

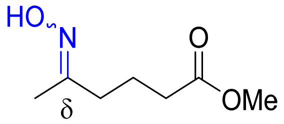

Methyl (*E/Z*)-5-(hydroxyimino)hexanoate (30a-δ-oxime): **^{1}H NMR** (400 MHz, $CDCl_3$) δ (ppm): 9.33 (brs, 1H), 3.62 (s, 3H), 2.40–2.16 (m, 4H), 1.85–1.77 (m, 5H); **^{13}C NMR of** (*E*)-**30a-δ-oxime:** (101 MHz, $CDCl_3$) δ (ppm): 173.7, 157.5, 51.6, 35.0, 33.2, 21.5, 13.4; **^{13}C NMR of** (Z)-**30a-δ-oxime** (101 MHz, $CDCl_3$) δ (ppm): 173.7, 157.8, 51.6, 33.7, 27.9, 20.8, 19.7.

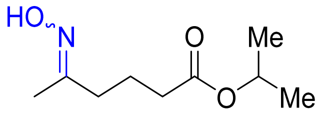

Isopropyl (*E/Z*)-5-(hydroxyimino)hexanoate (31a) was synthesized by the general Procedure **A** from isopropyl hexanoate (158.2 mg, 1 mmol) and purified by flash column chromatography on silica gel (gradient elution: Petroleum ether : EtOAc = 40:1 to 10:1) to afford the product (*E*/*Z*)-**31a** isomer as colorless oil (**86.1mg, 46% yield**, ***E/Z = 3:1***).

The E/Z isomers were confirmed by ^{1}H and ^{13}C NMR and the *ratio of E/Z isomers was calculated by integration of ^{1}H and ^{13}C NMR spectra.*

Major (*E*)-31a: ^{1}H NMR (400 MHz, $CDCl_3$) δ (ppm): 8.49 (brs, 1H), 5.05–4.95 (m, 1H), 2.33–2.26 (m, 2H), 2.21 (t, *J* = 7.6 Hz, 2H), 1.87 (s, 3H), 1.83 (t, *J* = 7.6 Hz, 2H), 1.21 (d, *J* = 6.4 Hz, 6H); **^{13}C NMR** (101 MHz, $CDCl_3$) δ (ppm): 172.9, 157.8, 67.8, 35.2, 34.0, 22.0, 21.7, 13.5.

Minor (*Z*)-31a: ^{1}H NMR (400 MHz, $CDCl_3$) δ (ppm): 8.49 (brs, 1H), 5.03–4.96 (m, 1H), 2.40 (t, *J* = 7.6 Hz, 2H), 2.33–2.26 (m, 2H), 1.86 (s, 3H), 1.81 (t, *J* = 7.6 Hz, 2H), 1.21 (d, *J* = 6.4Hz, 6H); **^{13}C NMR** (101 MHz, $CDCl_3$) δ (ppm): 172.9, 158.2, 67.8, 34.4, 28.0, 21.2, 21.0, 19.9;

HRMS (ESI+): *m/z* calculated for $C_9H_{17}NNaO_3^+$: 210.1101 found 210.1098.

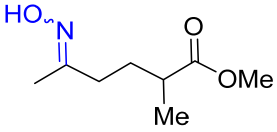

Methyl (*E/Z*)-5-(hydroxyimino)-2-methylhexanoate (32a) was synthesized by the general procedure **A** from methyl 2-methylhexanoate (144.2 mg, 1 mmol) and purified by flash column chromatography on silica gel (gradient elution: Petroleum ether : EtOAc = 40:1 to 10:1) to afford the product (*E*/*Z*)**-32a** isomer as colorless oil (**77.9 mg, 45% yield**, ***E/Z = 3:1***).

The E/Z isomers were confirmed by ^{1}H and ^{13}C NMR, and the *ratio of E/Z isomers was calculated by integration of ^{1}H and ^{13}C NMR spectra.*

Major (*E*)-32a: ^{1}H NMR (400 MHz, $CDCl_3$) δ (ppm): 8.72 (brs, 1H), 3.66 (s, 3H), 2.52–2.42 (m, 1H), 2.21–2.15 (m, 2H), 1.86 (s, 3H), 1.66–1.56 (m, 2H), 1.18 (d, *J* = 7.2 Hz, 3H); **^{13}C NMR** (101 MHz, $CDCl_3$) δ (ppm): 176.9, 157.9, 51.8, 39.0, 33.6, 30.1, 17.1, 13.6;

Minor (*Z*)-32a: ^{1}H NMR (400 MHz, $CDCl_3$) δ (ppm): 8.71 (brs, 1H), 3.67 (s, 3H),2.52–2.42(m, 1H), 2.42–2.39 (m, 2H), 1.94–1.86 (m, 2H), 1.85 (s, 3H), 1.16 (d, *J* = 7.2 Hz, 3H); **^{13}C NMR** (101 MHz, $CDCl_3$) δ (ppm): 176.9, 158.3, 50.8, 39.4, 29.3, 26.5, 19.9;

HRMS (ESI+): *m/z* $[M+Na]^+$ calculated for $C_8H_{15}NNaO_3^+$: 196.0944 found 196.0946.

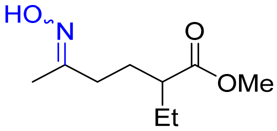

Methyl (*E/Z*)-2-ethyl-5-(hydroxyimino)hexanoate (33a) was synthesized by the general procedure **A** from methyl 2-ethylhexanoate (158.3 mg, 1 mmol) and purified by flash column

chromatography on silica gel (gradient elution: Petroleum ether : EtOAc = 40:1 to 10:1) to afford the product (*E/Z*)**-33a** isomer as colorless oil (**74.9 mg, 40% yield**, ***E/Z = 3:1***).

The E/Z isomers was confirmed by ^{1}H and ^{13}C NMR and the *ratio of E/Z isomers was calculated by integration of ^{1}H and ^{13}C NMR spectra.*

^{1}H NMR (400 MHz, $CDCl_3$) δ (ppm): 8.78 (brs, 1H), 3.66 (s, 3H), 2.33–2.23 (m, 1H), 2.15 (t, *J* = 7.6 Hz, 1H), 1.90–1.74 (m, 4H), 1.69–1.47 (m, 4H), 0.90–0.84 (m, 3H);

Major (*E*)-33a: ^{13}C NMR (101 MHz, $CDCl_3$) δ (ppm): 176.4, 157.8, 51.59, 46.6, 33.8, 28.3, 25.4, 13.6, 11.7; **Minor (*Z*)-33a: ^{13}C NMR** (101 MHz, $CDCl_3$) δ (ppm): 176.4, 158.2, 51.55, 47.0, 27.4, 26.7, 25.4, 19.9, 11.7;

HRMS (ESI+): *m/z* calculated for $C_9H_{17}NNaO_3^+$: 210.1101 found 210.1103.

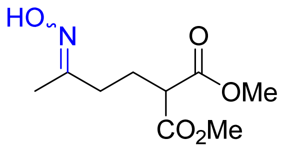

Dimethyl (*E/Z*)-2-(3-(hydroxyimino)butyl)malonate (34a) was synthesized by the general procedure **A** from dimethyl 2-butylmalonate (188.2 mg, 1 mmol) and purified by flash column chromatography on silica gel (gradient elution: Petroleum ether : EtOAc = 40:1 to 10:1) to afford the product (*E/Z*)**-34a** isomer as colorless oil (**99.9 mg, 46% yield**), and remaining starting material (75.3 mg, 40%).

The E/Z isomers was confirmed by ^{1}H and ^{13}C NMR and the *ratio of E/Z isomers* (***E/Z = 3:1***) *was calculated by integration of ^{1}H and ^{13}C NMR spectra.*

Major (*E*)**-34a: ^{1}H NMR** (400 MHz, $CDCl_3$) δ (ppm): 8.64 (brs, 1H), 3.71 (s, 6H), 3.42–3.33 (m, 1H), 2.24–2.19 (m, 2H), 2.14–2.07 (m, 2H), 1.86 (s, 3H); **^{13}C NMR** (101 MHz, $CDCl_3$) δ (ppm): 169.6, 157.0, 52.7, 50.9, 33.5, 25.3, 13.6.

Minor (*Z*)**-34a: ^{1}H NMR** (400 MHz, $CDCl_3$) δ (ppm): 8.64 (brs, 1H), 3.72 (s, 6H), 3.42–3.33 (m, 1H), 2.43–2.36 (m, 2H), 2.14–2.07 (m, 2H), 1.86 (s, 3H); **^{13}C NMR** (101 MHz, $CDCl_3$) δ (ppm): 169.6, 157.3, 52.7, 51.3, 26.4, 24.7, 19.8.

HRMS (ESI+): *m/z* $[M+Na]^+$ calculated for $C_9H_{15}NNaO_5^+$: 240.0842. found 240.0840.

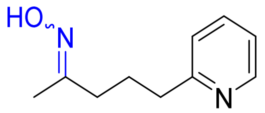

(*E/Z*)-5-(Pyridin-3-yl)pentan-2-one oxime (35a) was synthesized by the general procedure **A** from 3-pentylpyridine (149.3 mg, 1 mmol) and purified by flash column chromatography on silica gel (gradient elution: Petroleum ether : EtOAc = 10:1 to 2:1) to afford the product (*E*/*Z*)**-35a** isomer as colorless oil (**101.6 mg, 57% yield**, ***E/Z* = *3:1***) and remaining starting material (35.8 mg, 24%).

The E/Z isomers was confirmed by ^{1}H and ^{13}C NMR and the *ratio of E/Z isomers was calculated by integration of ^{1}H and ^{13}C NMR spectra.*

Major (*E*)**-35a:** **^{1}H NMR** (400 MHz, $CDCl_3$) δ (ppm): 9.88 (brs, 1H), 8.52–8.48 (m, 1H), 7.59–7.53 (m, 1H), 7.17–7.06 (m, 2H), 2.79 (t, *J* = 7.6 Hz, 2H), 2.23 (t, *J* = 7.6 Hz, 2H), 1.99–1.89 (m, 2H), 1.86 (s, 3H); **^{13}C NMR** (101 MHz, $CDCl_3$) δ (ppm): 161.6, 157.7, 149.2, 136.7, 123.1, 121.2, 37.6, 35.5, 26.5, 13.5.

Minor (*Z*)**-35a:** **^{1}H NMR** (400 MHz, $CDCl_3$) δ (ppm): 9.88 (brs, 1H), 8.52–8.48 (m, 1H), 7.59–7.53 (m, 1H), 7.17–7.06 (m, 2H), 2.83 (t, *J* = 7.6 Hz, 2H), 2.44 (t, *J* = 7.6 Hz, 2H), 1.98–1.90 (m, 2H), 1.85 (s, 3H); **^{13}C NMR** (101 MHz, $CDCl_3$) δ (ppm): 161.6, 158.1, 149.2, 136.7, 123.0, 121.3, 38.1, 28.4, 25.7, 19.9.

HRMS (ESI+): *m/z* $[M+H]^+$ calculated for $C_{10}H_{15}N_2O^+$: 179.1179, found 179.1176.

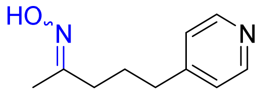

(*E/Z*)-5-(Pyridin-4-yl)pentan-2-one oxime (36a) was synthesized by the general procedure **A** from 4-pentylpyridine (149.3 mg, 1 mmol), and purified by flash column chromatography on silica gel (gradient elution: Petroleum ether : EtOAc = 10:1 to 4:1) to afford the product (*E*/*Z*)**-36a** isomer as white solid (**67.7 mg, 38% yield**, ***E/Z* = *3:1***), and remaining starting material (64.2 mg, 43%).

The E/Z isomers was confirmed by ^{1}H and ^{13}C NMR and the *ratio of E/Z isomers was calculated by integration of ^{1}H and ^{13}C NMR spectra.*

Major (*E*)**-36a:** **^{1}H NMR** (400 MHz, $CDCl_3$) δ (ppm): 9.76 (brs, 1H), 8.48–8.45 (m, 2H), 7.14–7.09 (m, 2H), 2.67–2.59 (m, 2H), 2.22 (t, *J* = 7.6 Hz, 2H), 1.88–1.82 (m, 5H); **^{13}C NMR** (101 MHz, $CDCl_3$) δ (ppm): 157.4, 151.2, 149.6, 124.1, 35.3, 34.6, 26.8, 13.6;

Minor (*Z*)**-36a: ^{1}H NMR** (400 MHz, $CDCl_3$) δ (ppm): 9.76 (brs, 1H), 8.48–8.45 (m, 2H), 7.14–7.09 (m, 2H), 2.67–2.59 (m, 2H), 2.41 (t, *J* = 7.6 Hz, 1H), 1.88–1.82 (m, 5H); **^{13}C NMR** (101 MHz, $CDCl_3$) δ (ppm): 157.4, 151.2, 149.6, 124.0, 35.2, 28.3, 26.1, 20.0;
HRMS (ESI+): *m/z* $[M+H]^+$ calculated for $C_{10}H_{15}N_2O^+$: 179.1179, found 179.1183.

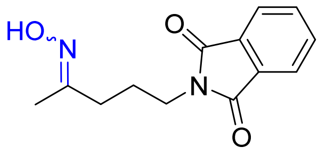

(*E/Z*)-2-(4-(Hydroxyimino)pentyl)isoindoline-1,3-dione (37a) was synthesized by the general procedure **A** from 2-pentylisoindoline-1,3-dione (217.3 mg, 1 mmol) and purified by flash column chromatography on silica gel (gradient elution: Petroleum ether : EtOAc = 10:1 to 1:1) to afford the product (*E*/*Z*)**-37a** isomer as white solid (**96.1 mg, 39% yield**, ***E/Z* = *3:1***), and remaining starting material (126.0 mg, 58%).
The E/Z isomers was confirmed by ^{1}H and ^{13}C NMR and the *ratio of E/Z isomers was calculated by integration of ^{1}H and ^{13}C NMR spectra.*
Major (*E*)**-37a: ^{1}H NMR** (400 MHz, $CDCl_3$) δ (ppm): 8.67 (brs, 1H), 7.84–7.79 (m, 2H), 7.71–7.65 (m, 2H), 3.72–3.65 (m, 2H), 2.23 (t, *J* = 7.6 Hz, 2H), 1.93–1.85 (m, 2H), 1.85 (s, 3H); **^{13}C NMR** (101 MHz, $CDCl_3$) δ (ppm):168.5, 157.3, 134.1, 132.2, 123.4, 37.6, 33.3, 25.1, 13.6;
Minor (*Z*)**-37a: ^{1}H NMR** (400 MHz, $CDCl_3$) δ (ppm): 8.67 (brs, 1H), 7.84–7.79 (m, 2H), 7.71–7.65 (m, 2H), 3.72–3.65 (m, 2H), 2.42 (t, *J* = 7.6 Hz, 2H), 1.93–1.85 (m, 2H), 1.85 (s, 3H); **^{13}C NMR** (101 MHz, $CDCl_3$) δ (ppm):168.5, 157.7, 134.1, 132.2, 123.4, 38.0, 26.1, 24.5, 19.8;
HRMS (ESI+): *m/z* $[M+Na]^+$ calculated for $C_{13}H_{14}N_2NaO_3^+$: 269.0897, found 269.0900.

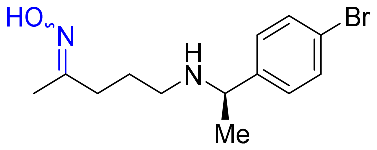

(*R*,*E*/*Z*)-5-((1-(4-Bromophenyl)ethyl)amino)pentan-2-one oxime (38a) was synthesized by the general procedure **A** from (*R*)-*N*-(1-(4-bromophenyl)ethyl)pentan-1-amine (270.2 mg, 1 mmol) and purified by flash column chromatography on silica gel (gradient elution: Petroleum ether :

EtOAc = 10:1 to 1:2) to afford the product **(*R*,*E*/*Z*)-38a** isomer as colorless oil (**131.7 mg, 44% yield**, ***E/Z* = 3:1**), and remaining starting material (86.4 mg, 32%).

The E/Z isomers was confirmed by ^{1}H and ^{13}C NMR and the *ratio of E/Z isomers was calculated by integration of ^{1}H and ^{13}C NMR spectra.*

Major (*R*,*E*)-38a : ^{1}H NMR (400 MHz, $CDCl_3$) δ (ppm): 7.43–7.40 (m, 2H), 7.19–7.15 (m, 2H), 5.49 (brs, 1H), 3.74–3.68 (m, 1H), 2.52–2.26 (m, 3H), 2.21–2.11 (m, 1H), 1.83 (s, 3H), 1.73–1.60 (m, 2H), 1.31 (d, *J* =6.8 Hz, 3H); **^{13}C NMR** (101 MHz, $CDCl_3$) δ (ppm): 157.5, 144.33, 131.6, 128.5, 120.6, 57.83, 46.95, 33.6, 26.5, 24.1, 13.6.

Minor (*R*,*Z*)-38a : ^{1}H NMR (400 MHz, $CDCl_3$) δ (ppm): 7.43–7.40 (m, 2H), 7.19–7.15 (m, 2H), 5.49 (brs, 1H), 3.74–3.68 (m, 1H), 2.52–2.26 (m, 3H), 2.21–2.11 (m, 1H), 1.81 (s, 3H), 1.73–1.60 (m, 2H), 1.31 (d, *J* =6.8 Hz, 3H); **^{13}C NMR** (101 MHz, $CDCl_3$) δ (ppm): 158.0, 144.37, 131.6, 128.5, 120.6, 57.75, 47.01, 26.2, 25.6, 21.2, 19.9.

HRMS (ESI+): *m/z* $[M+H]^+$ calculated for $C_{13}H_{20}BrN_2O^+$: 299.0754, found 299.0759.

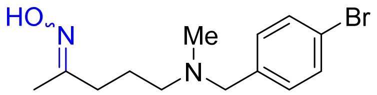

(*E*/*Z*)-5-((4-Bromobenzyl)(methyl)amino)pentan-2-one oxime (39a) was synthesized by the general procedure **A** from *N*-(4-bromobenzyl)-*N*-methylpentan-1-amine (270.2 mg, 1 mmol) and purified by flash column chromatography on silica gel (gradient elution: Petroleum ether : EtOAc = 10:1 to 1:3) to afford the product **(*E*/*Z*)-39a** isomer as colorless oil (**122.7 mg, 41% yield**, ***E/Z* = 3:1**), and remaining starting material (148.6 mg, 55%).

The E/Z isomers was confirmed by ^{1}H and ^{13}C NMR and the *ratio of E/Z isomers was calculated by integration of ^{1}H and ^{13}C NMR spectra.*

Major (*E*)-39a : ^{1}H NMR (400 MHz, $CDCl_3$) δ (ppm): 7.42–7.39 (m, 2H), 7.19–7.16 (m, 2H), 3.42 (s, 2H), 2.37 (t, *J* =7.6 Hz, 2H), 2.21–2.16 (m, 2H), 2.15 (s, 3H), 1.86 (s, 3H), 1.72 (quint, *J* = 7.6 Hz, 2H); **^{13}C NMR** (101 MHz, $CDCl_3$) δ (ppm): 157.8, 137.1, 131.3, 130.9, 120.8, 61.6, 56.7, 41.90, 33.7, 23.9, 13.6.

Minor (*Z*)-39a: ^{1}H NMR (400 MHz, $CDCl_3$) δ (ppm): 7.42–7.39 (m, 2H), 7.19–7.16 (m, 2H), 3.43 (s, 2H), 2.38 (t, *J* =7.6 Hz, 2H), 2.21–2.16 (m, 2H), 2.16 (s, 3H), 1.85 (s, 3H), 1.72 (quint, *J* = 7.6 Hz, 2H); **^{13}C NMR** (101 MHz, $CDCl_3$) δ (ppm): 158.3, 137.7, 131.3, 130.9, 120.8, 61.5, 57.0, 41.85, 26.6, 23.1, 20.0.

HRMS (ESI+): *m/z* [M+H]$^+$ calculated for $C_{13}H_{20}BrN_2O^+$: 299.0754, found 299.0751.

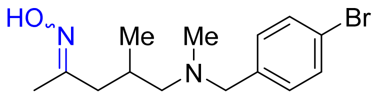

(*E/Z*)-5-((4-Bromobenzyl)(methyl)amino)-4-methylpentan-2-one oxime (40a) was synthesized by the general procedure **A** from *N*-(4-bromobenzyl)-*N*-methylpentan-1-amine (284.2 mg, 1 mmol) and purified by flash column chromatography on silica gel (gradient elution: Petroleum ether : EtOAc = 10:1 to 1:3) to afford the product **(*E*/*Z*)-40a** isomer as colorless oil (**103.4 mg, 33% yield**, ***E/Z* = *10:3***), and remaining starting material (179.1 mg, 63%).

The E/Z isomers was confirmed by ^{1}H and ^{13}C NMR and the *ratio of E/Z isomers was calculated by integration of ^{1}H and ^{13}C NMR spectra.*

Major (*E*)-40a: ^{1}H NMR (400 MHz, $CDCl_3$) δ (ppm): 9.32 (brs, 1H), 7.43–7.39 (m, 2H), 7.21–7.17 (m, 2H), 3.40 (s, 2H), 2.40 (dd, *J* = 13.6, 4.8 Hz, 1H), 2.21–2.10 (m, 6H), 2.02–1.91 (m, 1H), 1.86 (s, 3H), 0.87 (d, *J* = 6.8 Hz, 3H); **^{13}C NMR** (101 MHz, $CDCl_3$) δ (ppm): 157.9, 138.5, 131.4, 130.7, 120.7, 64.2, 62.2, 42.6, 41.3, 29.0, 18.2, 13.8.

Minor (*Z*)-40a: ^{1}H NMR (400 MHz, $CDCl_3$) δ (ppm): 9.32 (brs, 1H), 7.43–7.39 (m, 2H), 7.21–7.17 (m, 2H), 3.40 (s, 2H), 2.50 (dd, *J* = 13.2, 4.8 Hz, 1H), 2.21–1.90 (m, 7H), 1.87 (s, 3H), 0.91 (d, *J* = 6.0 Hz, 3H); **^{13}C NMR** (101 MHz, $CDCl_3$) δ (ppm): 158.3, 138.5, 131.4, 130.7, 120.7, 64.5, 62.1, 42.6, 34.3, 28.9, 20.9, 18.6.

HRMS (ESI+): *m/z* [M+H]$^+$ calculated for $C_{14}H_{22}BrN_2O^+$: 313.0910, found 313.0909.

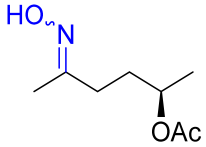

(*R,E/Z*)-5-(Hydroxyimino)hexan-2-yl acetate (41a) was synthesized by the general procedure **A** from (*R*)-hexan-2-yl acetate (144.2 mg, 1 mmol). After completion of the reaction, extraction with DCM, the solvent of DCM was removed to afford the product (*R,E/Z*)-**41a** isomer as colorless oil (**71.0 mg, 41% yield**, ***E/Z* = *3:1***), and the obtained product needs no further purification and taken directly for NMR and HRMS analysis.

The E/Z isomers was confirmed by ^{1}H and ^{13}C NMR and the *ratio of E/Z isomers was calculated by integration of ^{1}H and ^{13}C NMR spectra.*

Major (*R,E*)-41a: ^{1}H NMR (400 MHz, $CDCl_3$) δ (ppm): 8.61 (brs, 1H), 4.94–4.83 (m, 1H), 2.25–2.18 (m, 2H), 2.02 (s, 3H), 1.87 (s, 3H), 1.83–1.66 (m, 2H), 1.23 (t, J = 6.6 Hz, 3H); **^{13}C NMR** (101 MHz, $CDCl_3$) δ (ppm): 170.9, 157.8, 70.5, 32.3, 32.1, 21.4, 20.0, 13.7.

Minor (*R,Z*)-41a: ^{1}H NMR (400 MHz, $CDCl_3$) δ (ppm): 8.61 (brs, 1H), 4.92–4.84 (m, 1H), 2.44–2.32 (m, 2H), 2.03 (s, 3H), 1.86 (s, 3H), 1.83–1.66 (m, 2H), 1.26 (t, J = 6.6 Hz, 3H); **^{13}C NMR** (101 MHz, $CDCl_3$) δ (ppm): 171.0, 158.1, 70.8, 31.5, 24.9, 20.0, 19.9, 13.7.

HRMS (ESI+): *m/z* $[M+Na]^+$ calculated for $C_8H_{15}NNaO_3^+$: 196.0944, found 196.0943.

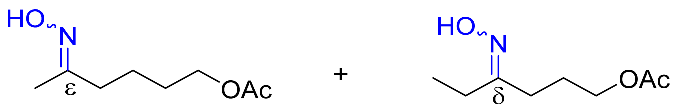

(*E/Z*)-5-(Hydroxyimino)hexyl acetate (42a-ε-oxime) and **(*E/Z*)-4-(hydroxyimino)hexyl acetate (42a-δ-oxime) (42a)** was synthesized by the general procedure **A** from hexyl acetate (144.2 mg, 1 mmol) and purified by flash column chromatography on silica gel (gradient elution: Petroleum ether : EtOAc = 40:1 to 10:1) to afford a mixture of two regioisomers (*E*/*Z*)-**42a-ε-oxime** and (*E*/*Z*)-**42a-δ-oxime** as colorless oil (**97.0 mg, 56% yield**, **δ:ε = 1:9**, **δ, *E/Z* = 1:1; ε, *E/Z* = 3:1**), and the product of **42a-ε-oxime** (**85.2 mg, 49% yield**) was isolated from two regioisomers mixture.

Site of oximation was assigned based on analysis of the ^{1}H and ^{13}C NMR spectra of the mixture products with the isolated (*E*/*Z*)-**42a-ε-oxime**. *The E/Z isomers were confirmed by ^{1}H and ^{13}C NMR and the ratio of E/Z isomers was calculated by integration of ^{13}C NMR spectrum.*

^{1}H NMR (400 MHz, $CDCl_3$) δ (ppm): 9.08 (brs, 1H), 4.28–4.22 (m, 0.20H), 4.10–4.01 (m, 1.82H), 2.66 (t, J = 6.8 Hz, 0.10H), 2.49 (t, J = 6.8 Hz, 0.10H), 2.42–3.30 (m, 0.86H), 2.27–2.17 (m, 1.51H), 2.02 (s, 3H), 1.85 and 1.84 (s, 1.75H), 1.69–1.51 (m, 2.85H), 1.06(t, J = 7.6 Hz, 0.73H), 0.96–0.89 (m, 0.52H);

^{13}C NMR (101 MHz, $CDCl_3$) δ (ppm):171.4(2C), 171.3(2C), 161.7, 161.6, 158.4, 158.1, 64.4, 64.22, 64.18, 63.9, 35.4, 30.3, 28.6, 28.1, 27.6, 25.2, 24.8, 24.4, 22.7, 22.0, 21.09, 21.03, 21.00, 20.97, 19.8, 19.5, 13.5, 10.8, 10.1;

HRMS (ESI+): *m/z* $[M+Na]^+$ calculated for $C_8H_{15}NNaO_3^+$: 196.0944, found 196.0946.

(*E*)-**42a-ε-oxime:**

^{1}H NMR (400 MHz, $CDCl_3$) δ (ppm): 8.97 (brs, 1H), 4.10–4.02 (m, 2H), 2.20 (t, *J* = 7.2 Hz, 2H), 2.02 (s, 3H), 1.86 (s, 3H), 1.68–1.51 (m, 4H); **^{13}C NMR** (101 MHz, $CDCl_3$) δ (ppm): 171.4, 158.2, 64.21, 35.4, 28.2, 22.7, 21.1, 13.5.

(*Z*)-**42a-ε-oxime:**

^{1}H NMR (400 MHz, $CDCl_3$) δ (ppm): 8.97 (brs, 1H), 4.10–4.02 (m, 2H), 2.38 (t, *J* = 7.6 Hz, 2H), 2.02 (s, 3H), 1.85 (s, 3H), 1.68–1.51 (m, 4H); **^{13}C NMR** (101 MHz, $CDCl_3$) δ (ppm): 171.4, 158.5, 64.24, 30.2, 28.6, 22.1, 21.0, 19.8.

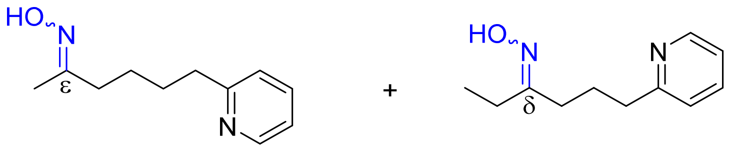

(*E/Z*)-6-(pyridin-2-yl)hexan-2-one oxime (43a-ε-oxime) and **(*E/Z*)-6-(pyridin-2-yl)hexan-3-one oxime (43a-δ-oxime) (43a)** was synthesized by the general procedure **A** from 2-hexylpyridine (163.3 mg, 1 mmol) and purified by flash column chromatography on silica gel (gradient elution: Petroleum ether : EtOAc = 10:1 to 2:1) to afford two separated regioisomers (*E*/*Z*)-**43a-ε-oxime** (**98.1 mg, 51% yield**, ***E/Z*=3:1**) and (*E*/*Z*)-**43a-δ-oxime** (**23.1 mg, 12% yield**, ***E/Z* = 1:1**) as white solid (**ε:δ = 4.3:1**) and remaining starting material (36.5 mg, 22%).

The E/Z isomers were confirmed by *^{1}H, and ^{13}C NMR spectra and the ratio of E/Z* isomers *was calculated by integration of ^{1}H and ^{13}C NMR spectra.*

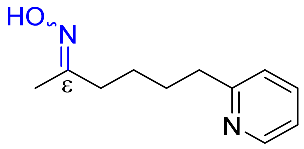

(*E*)-**43a-ε-oxime:**

^{1}H NMR (400 MHz, $CDCl_3$) δ (ppm): 8.49–8.46 (m, 1H), 7.55 (t, *J* = 7.6 Hz, 1H), 7.13–7.04 (m, 2H), 2.85–2.75 (m, 2H), 2.19 (t, *J* = 7.6 Hz, 2H), 1.82 (s, 3H), 1.77–1.66 (m, 2H), 1.58–1.49 (m, 2H); **^{13}C NMR** (101 MHz, $CDCl_3$) δ (ppm): 161.9, 157.9, 148.9, 136.7, 123.0, 121.1, 37.7, 35.6, 29.4, 26.0, 13.4.

(*Z*)-**43a-ε-oxime:**

^{1}H NMR (400 MHz, $CDCl_3$) δ (ppm): 8.49–8.46 (m, 1H), 7.55 (t, *J* = 7.6 Hz, 1H), 7.13–7.04 (m, 2H), 2.85–2.75 (m, 2H), 2.39 (t, *J* = 7.6 Hz, 2H), 1.80 (s, 3H), 1.77–1.66 (m, 2H), 1.58–1.49 (m, 2H); **^{13}C NMR** (101 MHz, $CDCl_3$) δ (ppm): 162.0, 158.3, 148.9, 136.7, 123.0, 121.1, 37.8, 29.9, 28.4, 25.2, 19.9.

HRMS (ESI+): *m/z* $[M+H]^+$ calculated for $C_{11}H_{17}N_2O^+$: 193.1335, found 193.1338.

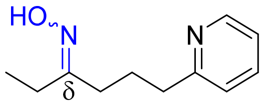

(*E*/*Z*)-**43a-δ-oxime:**

^{1}H NMR (400 MHz, $CDCl_3$) δ (ppm): 8.55–8.51 (m, 1H), 7.63–7.55 (m, 1H), 7.22–7.16 (m, 1H), 7.14–7.09 (m, 1H), 3.07–3.00 (m, 2H), 2.78–2.72 (m, 1H), 2.66–2.60 (m, 1H), 2.39–2.33 (m, 1H), 2.12 (t, *J* = 7.6Hz, 1H), 1.62–1.48 (m, 2H), 0.97–0.87 (m, 3H);**^{13}C NMR** (101 MHz, $CDCl_3$) δ (ppm): 161.2, 161.1, 160.9, 149.3, 136.74, 136.68, 123.1, 123.0, 121.5, 121.4, 36.6, 34.7, 34.1, 34.0, 30.0, 28.0, 19.7, 19.2, 14.5, 14.0;

HRMS (ESI+): *m/z* $[M+Na]^+$ calculated for $C_{11}H_{16}N_2NaO^+$: 215.1155, found 215.1158.

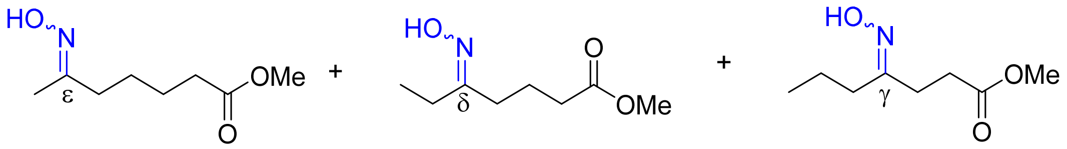

Methyl (*E/Z*)-6-(hydroxyimino)heptanoate (44a-ε-oxime), methyl (*E/Z*)-5-(hydroxyimino)heptanoate (44a-δ-oxime) and **methyl (*E/Z*)-4-(hydroxyimino)heptanoate (44a-γ-oxime) (44a)** was synthesized by the general procedure **A** from methyl heptanoate (144.2 mg, 1 mmol) and purified by flash column chromatography on silica gel (gradient elution: Petroleum ether : EtOAc = 40:1 to 10:1) to afford a mixture of three regioisomers (*E*/*Z*)-**44a-ε-oxime**, (*E*/*Z*)-**44a-δ-oxime** and (*E*/*Z*)-**44a-γ-oxime** as colorless oil (**109.2 mg, 63% yield**, γ:δ:ε = **1:6.7:16.7**, γ, ***E/Z* = 1:1; δ, *E/Z* = 1:1; ε, *E/Z* = 3:1**). The regioisomer (*E*/*Z*)-**44a-ε-oxime** was isolated from the mixture in 40% yield (70 mg).

*Site of oximation was assigned based on analysis of the ^{1}H and ^{13}C NMR spectra of the mixture products with the isolated (E/Z)-**44a-ε-oxime**. The E/Z isomers were confirmed by ^{1}H and ^{13}C NMR and the ratio of E/Z isomers were calculated by integration of ^{1}H and ^{13}C NMR spectra.*

^{1}H NMR (400 MHz, $CDCl_3$) δ (ppm): 9.12 (brs, 1H), 3.63 (s, 3H), 2.59–2.49 (m, 0.4H), 2.38–2.26 (m, 3.1H), 2.23–2.14 (m, 1.6H), 1.84 and 1.82 (s, 2.8H), 1.66–1.57 (m, 1.4H), 1.55–1.47 (m, 1.6H), 1.05 (t, *J* = 7.6Hz, 0.8H); **^{13}C NMR** (101 MHz, $CDCl_3$) δ (ppm): 174.12, 174.08, 173.87, 173.85, 173.49(2C), 161.9, 161.8, 159.9, 159.8, 158.4, 158.1, 51.79, 51.65, 51.63, 51.60, 35.4, 33.9, 33.8, 33.4, 32.9, 30.3, 30.2, 29.9, 29.2, 28.2, 27.4, 26.9, 25.7, 25.3, 24.99, 24.92, 24.88, 24.4, 23.5, 21.5, 21.1, 20.9, 19.8, 19.6, 19.1, 14.3, 13.8, 13.4, 10.8, 10.1; **HRMS** (ESI+): *m/z* calculated for $C_8H_{15}NNaO_3^+$: 196.0944. found 196.0946.

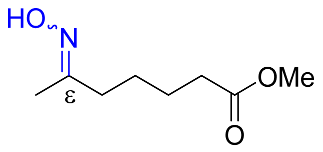

Methyl (*E/Z*)-6-(hydroxyimino)heptanoate (44a-ε-oxime): **^{1}H NMR** (400 MHz, $CDCl_3$) δ (ppm): 9.23 (brs, 1H), 3.64 (s, 3H), 2.40–2.13 (m, 4H), 1.84 (brs, 3H), 1.68–1.47 (m, 4H); **^{13}C NMR** of (*E*)-**44a-ε-oxime** (101 MHz, $CDCl_3$) δ (ppm): 174.1, 158.2, 51.6, 35.4, 33.8, 25.7, 24.4, 13.4; **^{13}C NMR** of (*Z*)-**44a-ε-oxime** (101 MHz, $CDCl_3$) δ (ppm): 174.1, 158.5, 51.6, 35.4, 33.8, 28.2, 25.0, 24.9, 19.8.

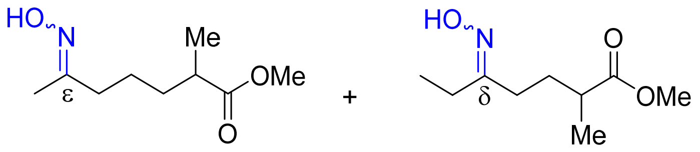

Methyl (*E/Z*)-6-(hydroxyimino)-2-methylheptanoate (45a-ε-oxime) and **methyl (*E/Z*)-5-(hydroxyimino)-2-methylheptanoate (45a-δ-oxime) (45a)** was synthesized by the general procedure **A** from methyl 2-methylheptanoate (158.2 mg, 1 mmol) and purified by flash column chromatography on silica gel (gradient elution: Petroleum ether : EtOAc = 40:1 to 10:1) to afford a mixture of two regioisomers (*E*/*Z*)-**45a-ε-oxime** and (*E*/*Z*)-**45a-δ-oxime** as colorless oil (**103.0 mg, 55% yield**, **δ:ε = 1:2**, **δ, *E/Z* = 1:1; ε, *E/Z* = 3:1**).

Site of oximation was assigned based on analysis of the ^{1}H and ^{13}C NMR spectra of the mixture products. The E/Z isomers were confirmed by ^{1}H and ^{13}C NMR and the ratio of E/Z isomers was calculated by integration of ^{13}C NMR spectrum.

^{1}H NMR (400 MHz, $CDCl_3$) δ (ppm): 8.92 (brs, 1H), 3.68–3.63 (m, 3H), 2.50–2.40 (m, 1H), 2.38–2.29 (m, 0.81H), 2.25–2.11 (m, 1.64H, ε-**45a**), 1.85 (s, 1.16H, ε-**45a**), 1.83 (s, 0.39H, ε-**45a**), 1.71–1.58 (m, 1.17H), 1.54–1.35 (m, 2.75H), 1.20–1.11 (m, 3.38H), 1.09–1.03 (m, 0.91H);
^{13}C NMR (101 MHz, $CDCl_3$) δ (ppm): 177.21 ((***Z***)**-45a-ε**), 177.17 ((***E***)**-45a-ε**), 176.9 (2C, **45a-*δ***), 162.1 (**45a-*δ***), 162.0 (**45a-*δ***), 158.5 ((***Z***)**-45a-ε**), 158.2 ((***E***)**-45a-ε**), 51.8, 51.72, 51.67, 39.6, 39.3, 39.0, 35.7, 33.7, 33.2, 31.4, 30.1, 29.4, 28.4, 27.5, 25.5, 24.0, 23.2, 21.0, 19.9, 17.17, 17.13, 13.4, 10.8, 10.2; **HRMS** (ESI+): *m/z* $[M+Na]^+$ calculated for $C_9H_{17}NNaO_3^+$: 210.1101. found 210.1113.

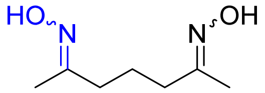

(*EE/ZE/ZZ*)-Heptane-2,6-dione dioxime (46a) was synthesized by the general procedure **A** from **(*E/Z*)-**heptan-2-one oxime (129.2 mg, 1 mmol, *E*:*Z* = 2:1) and purified by flash column chromatography on silica gel (gradient elution: Petroleum ether : EtOAc = 20:1 to 5:1) to afford the product **(*EE/ZE/ZZ*)-46a** isomer as colorless oil (**49.1 mg, 31% yield**, ***EE/ZE/ZZ* = *3.6:1.2:1***), and remaining starting material (85.4 mg, 66%).
The E/Z isomers were confirmed by ^{1}H and ^{13}C NMR, and the ratio of E/Z isomers was calculated by integration of ^{13}C NMR spectrum based on more product formed belong to thermodynamic stability of E-product.
^{1}H NMR (400 MHz, $CDCl_3$) δ (ppm): 9.12 (brs, 2H), 2.44–2.33 (m, 1.29H), 2.26–2.17 (m, 3.09H), 1.90–1.85 (m, 6.0H), 1.79–1.68 (m, 2.03H);
^{13}C NMR (101 MHz, $CDCl_3$) δ (ppm): 158.4, 158.13, 158.05, 35.8, 35.2, 29.8, 28.3, 22.8, 22.2, 20.0, 13.6;
HRMS (ESI+): *m/z* calculated for $C_7H_{14}N_2NaO_2^+$: 181.0947. found 181.0952.

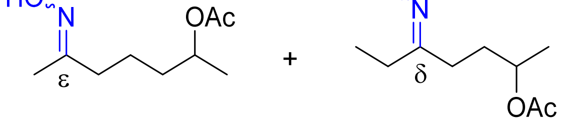

(*E/Z*)-6-(Hydroxyimino)heptan-2-yl acetate (47a-ε-oxime) and **(*E/Z*)-5-(hydroxyimino) heptan-2-yl acetate (47a-*δ*-oxime)** and **(47a)** was synthesized by the general procedure **A** from heptan-2-yl acetate (158.2 mg, 1 mmol) and purified by flash column chromatography on silica

gel (gradient elution: Petroleum ether : EtOAc = 40:1 to 10:1) to afford a mixture of two regioisomers (*E*/Z)-**47a-ε-oxime** and (*E*/Z)-**47a-δ-oxime** as colorless oil (**88.0 mg, 47% yield**, **δ:ε = 1:3**, **δ, *E/Z* = 1:1; ε, *E/Z* = 3:1**).

Site of oximation was assigned based on analysis of the ^{1}H and ^{13}C NMR spectra of the mixture products. The E/Z isomers were confirmed by ^{1}H and ^{13}C NMR spectra and the ratio of E/Z isomers was calculated by integration of ^{1}H and ^{13}C NMR spectra.

^{1}H NMR (400 MHz, $CDCl_3$) δ (ppm): 4.92–4.84 (m, 1H), 2.41–2.30 (m, 1H), 2.23–2.14 (m, 1.53H, **47a-ε**) 2.00 (s, 3.2H), 1.85 and 1.83 (s, 2.1H, **47a-ε**) 1.78–1.40 (m. 4.07H), 1.23–1.16 (m, 3.70H), 1.06 (t, *J* = 7.6 Hz, 0.71H, **47a-*δ***);

^{13}C NMR (101 MHz, $CDCl_3$) δ (ppm): 171.0(4C), 162.0 (**47a-*δ***), 161.8 (**47a-*δ***), 158.4 ((***Z***)-**47a-ε**), 158.1((***E***)-**47a-ε**), 70.9, 70.7, 70.6, 35.8, 35.6, 35.4, 32.3, 31.6, 29.7, 29.4, 28.4, 27.5, 25.0, 24.8, 23.8, 22.2, 21.5, 21.40, 21.37, 21.04, 20.01, 19.96, 19.85, 19.83, 13.5, 10.8, 10.1;

HRMS (ESI+): *m/z* $[M+Na]^+$ calculated for $C_9H_{17}NNaO_3^+$: 210.1101, found 210.1107.

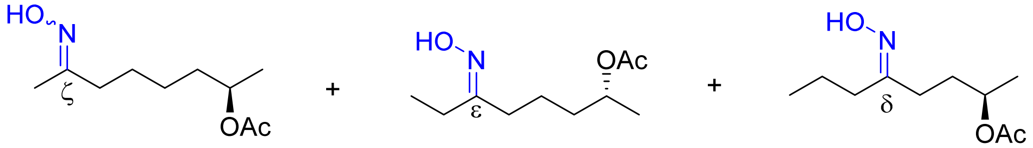

(*R,E*/*Z*)-7-(Hydroxyimino)octan-2-yl acetate (48a-ζ-oxime), **(*R,E*/*Z*)-6-(hydroxyimino)octan-2-yl acetate (48a-ε-oxime)** and **(*R,E*/*Z*)-5-(hydroxyimino)octan-2-yl acetate (48a-δ-oxime) (48a)** was synthesized by the general procedure **A** from (*R*)-octan-2-yl acetate (172.3 mg, 1 mmol) and purified by flash column chromatography on silica gel (gradient elution: Petroleum ether : EtOAc = 40:1 to 10:1) to afford a mixture of three regioisomers (*E*/*Z*)-**48a-ζ-oxime**, (*E*/*Z*)-**48a-ε-oxime**, and (*E*/Z)-**48a-δ-oxime** as colorless oil (**106.7 mg, 53% yield**, **δ:ε:ζ = 1:1.8:5**, **δ, *E/Z* = 1:1; ε, *E/Z* = 1:1; ζ, *E/Z* = 3:1**).

Site of oximation was assigned based on analysis of the ^{1}H and ^{13}C NMR spectra of the mixture products. The E/Z isomers were confirmed by ^{1}H and ^{13}C NMR and the ratio of E/Z isomers was calculated by integration of ^{1}H and ^{13}C NMR spectra.

^{1}H NMR (400 MHz, $CDCl_3$) δ (ppm): 9.43 (brs, 1H), 4.89–4.75 (m, 1H), 2.33–2.21 (m, 0.77H), 2.16–2.05 (m, 1.18H), 1.98–1.92 (m, 3H), 1.79 and 1.77 (s, 1.31H), 1.56–1.37 (m, 3.29H), 1.27–1.16 (m, 3.16H), 1.15–1.08 (m, 2.95H), 1.00 (t, *J* = 7.6 Hz, 0.52H), 0.90–0.80 (m, 0.66H),

0.80–0.76 (m, 0.47H); **^{13}C NMR** (101 MHz, $CDCl_3$) δ (ppm): 170.87, 170.84, 170.82, 162.1(**48a-**ε), 161.9(**48a-**ε), 160.5 (**48a-**δ), 160.4 (**48a-**δ), 158.4 ((***Z***)**-48a-**ζ), 158.0 ((***E***)**-48a-**ζ), 71.1, 70.83, 70.81, 70.64, 70.61, 70.48, 36.0, 35.9, 35.8, 35.53, 35.50, 35.4, 33.3, 32.2, 31.9, 31.7, 31.5, 30.0, 29.6, 29.1, 28.4, 27.3, 27.15, 27.13, 26.0, 25.4, 25.3, 25.2, 24.8, 23.6, 22.5, 22.1, 21.5, 21.30, 21.28, 21.2, 20.8, 19.89, 19.86, 19.7, 19.6, 19.0, 14.3, 14.0, 13.7, 13.3, 10.7, 10.0;
HRMS (ESI+): *m/z* $[M+Na]^+$ calculated for $C_{10}H_{19}NNaO_3^+$: 224.1257, found 224.1255.

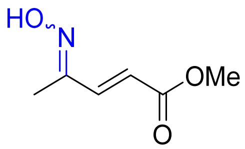

Methyl (2*E*,4*E*/4*Z*)-4-(hydroxyimino)pent-2-enoate (49a) was synthesized by the general procedure **B** from methyl (2*E*)-pent-2-enoate (114.1 mg, 1 mmol) and purified by flash column chromatography on silica gel (gradient elution: Petroleum ether : EtOAc = 20:1) to afford a mixture of *E/Z* isomers **(2*E*,4*E*/4*Z*)-49a** as a white solid (**101.2 mg, 71% yield**); and no epoxide was obtained from the reaction.
The E/Z isomerism is with respect to the C=N bond. Ratio of ***(2E,4E)/(2E,4Z) = 2:1****;*
Major (2*E*,4*E*)-49a:
^{1}H NMR (400 MHz, $CDCl_3$) δ (ppm): 9.96 (brs, 1H), 7.32 (d, *J* = 16.0 Hz, 1H), 6.18 (d, *J* = 16.0 Hz, 1H), 3.76 (s, 3H), 2.01 (s, 3H); **^{13}C NMR** (101 MHz, $CDCl_3$) δ (ppm): 167.0, 155.3, 141.8, 122.5, 52.1, 9.8.
Minor (2*E*,4*Z*)-49a:
^{1}H NMR (400 MHz, $CDCl_3$) δ (ppm): 9.96 (brs, 1H), 7.97 (d, *J* = 16.4 Hz, 1H), 6.20 (d, *J* = 16.4 Hz, 1H), 3.78 (s, 3H), 2.03 (s, 3H); **^{13}C NMR** (101 MHz, $CDCl_3$) δ (ppm): 167.0, 151.5, 131.3, 125.3, 52.2, 16.7.
HRMS (ESI): *m/z* $[M+Na]^+$ calcd for $C_6H_9NNaO_3^+$: 166.0475; found: 166.0474.

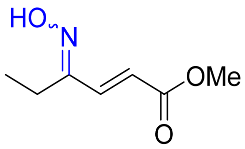

Methyl (2*E*,4*E*/4*Z*) 4-(hydroxyimino)hex-2-enoate (50a) was synthesized by the general procedure **B** from methyl (*E*)-hex-2-enoate (128.2 mg, 1 mmol). After completion of the reaction,

extraction with DCM and removal of the solvent DCM afforded a mixture of *E/Z* isomers (**2*E*,4*E*/4*Z*)-50a** as a white solid (**130.5 mg, 83% yield**); the product needs nofurther purification and was taken directly for NMR and HRMS analysis. No epoxide was obtained from the reaction.

The E/Z isomerism is with respect to C=N bond. Ratio of ***(2E,4E)/(2E,4Z) = 2:1;***

Major (2*E*,4*E*)-50a:

^{1}H NMR (400 MHz, $CDCl_3$) δ (ppm): 9.28 (brs, 1H), 7.20 (d, *J* = 16.4 Hz, 1H), 6.15 (d, *J* = 16.0 Hz, 1H), 3.72 (s, 3H), 2.48 (q, *J* = 7.6 Hz, 2H), 1.03 (t, *J* = 7.6 Hz, 3H); **^{13}C NMR** (101 MHz, $CDCl_3$) δ (ppm): 167.1, 159.9, 140.9, 122.1, 52.0, 17.7, 10.6.

Minor (2*E*,4*Z*)-50a:

^{1}H NMR (400 MHz, $CDCl_3$) δ (ppm): 9.28 (brs, 1H), 7.84 (d, *J* = 16.8 Hz, 1H), 6.19 (d, *J* = 16.0 Hz, 1H), 3.73 (s, 3H), 2.39 (q, *J* = 7.6 Hz, 2H), 1.08 (t, *J* = 7.6 Hz, 3H); **^{13}C NMR** (101 MHz, $CDCl_3$) δ (ppm): 167.1, 155.3, 130.8, 124.8, 52.2, 24.3, 11.5.

HRMS (ESI): *m/z* $[M+Na]^+$ calcd for $C_7H_{11}NNaO_3^+$: 180.0631; found: 180.0625.

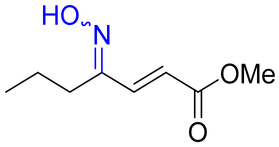

Methyl (2*E*,4*E*/4*Z*)-4-(hydroxyimino)hept-2-enoate (51a) was synthesized by the general procedure **B** from methyl (*E*)-hept-2-enoate (142.2 mg, 1 mmol) and purified by flash column chromatography on silica gel (gradient elution: Petroleum ether : EtOAc = 20:1) to afford the product (2*E*,4*E*/4*Z*)**-51a** as a mixture of *E/Z* isomers. Epoxide was also obtained from the reaction.

The E/Z isomerism is with respect to C=N bond. Ratio of ***(2E,4E)/(2E,4Z) = 2:1;***

51a: 109.6 mg, 64% yield, white solid; **rsm:** 11.4 mg, 8%; **epoxide:**12.6 mg, 8% yield;

Major (2*E*,4*E*)-isomer: ^{1}H NMR (400 MHz, $CDCl_3$) δ (ppm): 8.87 (brs, 1H), 7.25 (d, *J* = 16.4 Hz, 1H), 6.18 (d, *J* = 16.4 Hz, 1H), 3.76 (s, 3H), 2.49 (t, *J* = 7.6 Hz, 2H), 1.60–1.47 (m, 2H), 2.49 (t, *J* = 8.0 Hz, 3H); **^{13}C NMR** (101 MHz, $CDCl_3$) δ (ppm): 167.1, 158.7, 141.3, 122.2, 52.0, 26.2, 19.7, 14.3.

Minor (2*E*,4*Z*)-isomer: ^{1}H NMR (400 MHz, $CDCl_3$) δ (ppm): 8.87 (brs, 1H), 7.88 (d, *J* = 16.4 Hz, 1H), 6.23 (d, *J* = 16.8 Hz, 1H), 3.77 (s, 3H), 2.37 (t, *J* = 7.6 Hz, 2H), 1.60–1.47 (m, 2H), 2.49 (t, *J* = 8.0 Hz, 3H); **^{13}C NMR** (101 MHz, $CDCl_3$) δ (ppm): 167.1, 154.3, 130.9, 124.9, 52.2, 32.8, 20.6, 13.8.

HRMS (ESI): *m/z* [M+H]$^+$ calcd for $C_8H_{13}NNaO_3^+$: 194.0788; found: 194.0793.

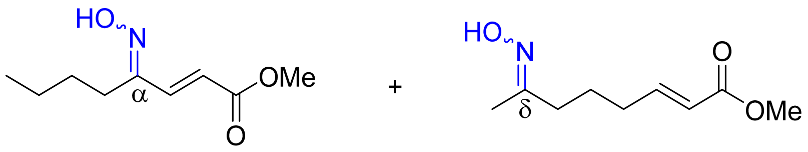

Methyl (2*E*,4*E*/4*Z*)-4-(hydroxyimino)oct-2-enoate (52a-α-oxime) and **methyl (2*E*,7*E*/7*Z*)-7-(hydroxyimino)oct-2-enoate (52a-δ-oxime) (52a)** were synthesized by the general Procedure **B** from methyl (*E*)-oct-2-enoate (156.2 mg, 1 mmol) and purified by flash column chromatography on silica gel (gradient elution: Petroleum ether : EtOAc = 20:1 to 5:1) to afford isolated (2*E*,4*E*/4*Z*)-**52a-α-oxime** as an *E/Z* mixture, and isolated (2*E*,7*E*/7*Z*)-**52a-δ-oxime** as an *E/Z* mixture with small amount of other regioisomers. No epoxide was obtained from the reaction.
The E/Z isomerism is with respect to the C=N bond. *Ratio of* ***(2E,4E)/(2E,4Z) = 2:1;***
(2*E*,4*E*/4*Z*)-**52a-α-oxime**, 103.7 mg, 56% yield, colorless oil; (2*E*,7*E*/7*Z*)-**52a-δ-oxim,** 10% yield; **rsm**: 6%;
(2*E*,4*E*/4*Z*)-**52a-α-oxime** isomer:

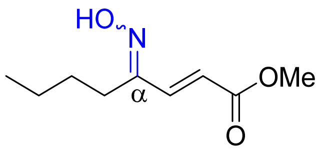

Major (2*E*,4*E*)-isomer: ^{1}H NMR (400 MHz, $CDCl_3$) δ (ppm): 9.80 (brs, 1H), 7.26 (d, *J* = 16.0 Hz, 1H), 6.20 (d, *J* = 16.4 Hz, 1H), 3.78 (s, 3H), 2.53 (t, *J* = 7.6 Hz, 2H), 1.56–1.43 (m, 2H), 1.41–1.29 (m, 2H), 0.91 (t, *J* = 7.2 Hz, 3H); **^{13}C NMR** (101 MHz, $CDCl_3$) δ (ppm): 167.0, 159.1, 141.3, 122.3, 52.1, 28.4, 24.1, 23.0, 13.9.
Minor (2*E*,4*Z*)-isomer: ^{1}H NMR (400 MHz, $CDCl_3$) δ (ppm): 9.80 (brs, 1H), 7.89 (d, *J* = 16.4 Hz, 1H), 6.25 (d, *J* = 16.8 Hz, 1H), 3.79 (s, 3H), 2.41 (t, *J* = 7.8 Hz, 2H), 1.56–1.43 (m, 2H), 1.41–1.29 (m, 2H), 0.90 (t, *J* = 7.2 Hz, 3H); **^{13}C NMR** (101 MHz, $CDCl_3$) δ (ppm): 167.0, 154.7, 130.9, 125.0, 52.2, 30.7, 29.4, 22.5, 13.8.
HRMS (ESI): *m/z* [M+Na]$^+$ calcd for $C_9H_{15}NNaO_3^+$: 208.0944; found: 208.0938.

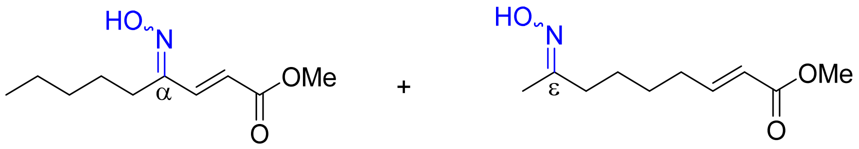

Methyl (2*E*,4*E*/4*Z*)-4-(hydroxyimino)non-2-enoate (53a-α-oxime) and **methyl (2*E*,8*E*/8*Z*)-8-(hydroxyimino)non-2-enoate (53a-ε-oxime) (53a)** was synthesized by the general Procedure **B** from methyl (*E*)-non-2-enoate (170.3 mg, 1 mmol) and purified by flash column chromatography on silica gel (gradient elution: Petroleum ether : EtOAc = 20:1 to 5:1) to afford isolated (2*E*,4*E*/4*Z*)-**53a-α-oxime** as an *E/Z* mixture, and (2*E*,8*E*/8*Z*)-**53a-ε-oxime** as an *E/Z* mixture. No epoxide was obtained from the reaction.

The E/Z isomerism is with respect to C=N bond. Ratio of ***(2E,4E)/(2E,4Z) = 2:1, (2E,8E)/(2E,8Z) = 8.3:1;***

(2*E*,4*E*/4*Z*)-**53a-α-oxime,** 111.6 mg, 56% yield, colorless oil; (2*E*,8*E*/8*Z*)-**53a-ε-oxime**, 35.9 mg, 18% yield, colorless oil;

(2*E*,4*E*/4*Z*)-**53a-α-oxime isomer:**

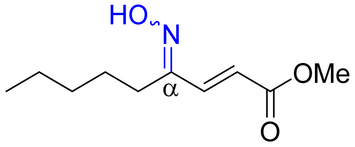

Major (2*E*,4*E*)-isomer: ^{1}**H NMR** (400 MHz, $CDCl_3$) δ (ppm): 9.43 (brs, 1H), 7.25 (d, *J* = 16.4 Hz, 1H), 6.18 (d, *J* = 16.4 Hz, 1H), 3.77 (s, 3H), 2.51 (t, *J* = 7.6 Hz, 2H), 1.55–1.46 (m, 2H), 1.33–1.28 (m, 4H), 0.86 (t, *J* = 6.6 Hz, 3H); ^{13}**C NMR** (101 MHz, $CDCl_3$) δ (ppm): 167.0, 159.0, 141.3, 122.2. 52.0, 32.0, 25.9, 24.3, 22.5, 14.0

Minor (2*E*,4*Z*)-isomer: ^{1}**H NMR** (400 MHz, $CDCl_3$) δ (ppm): 9.43 (brs, 1H), 7.88 (d, *J* = 16.8 Hz, 1H), 6.24 (d, *J* = 16.8 Hz, 1H), 3.78 (s, 3H), 2.39 (t, *J* = 7.6 Hz, 2H), 1.55–1.46 (m, 2H), 1.33–1.28 (m, 4H), 0.86 (t, *J* = 6.6 Hz, 3H); ^{13}**C NMR** (101 MHz, $CDCl_3$) δ (ppm): 167.0, 154.7, 130.9, 125.0, 52.2, 31.5, 30.9, 27.0, 22.4, 14.0.

HRMS (ESI): *m/z* $[M+Na]^+$ calcd for $C_{10}H_{17}NNaO_3^+$: 222.1101; found: 222.1097.

(2*E*,8*E*/8*Z*)-**53a-ε-oxime isomer:**

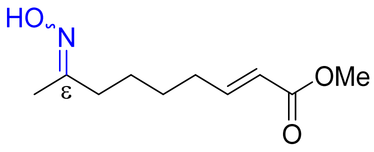

^{1}H NMR (400 MHz, $CDCl_3$) δ (ppm): 9.05 (brs, 1H), 7.95−7.88 (m, 0.16H, (2*E*,8*Z*)), 7.31−7.25 (m, 1.32H), 6.27−6.21 (m, 1.10H, (2*E*,8*E*)), 3.80 (s, 0.37H, (2*E*,8*Z*)), 3.79 (s, 3H, (2*E*,8*E*)), 2.60−2.54 (m, 1.92H), 2.47−2.42 (m, 0.83H), 2.29−2.25 (m, 1.81H), 1.90 (s, 2.68H, (2*E*,8*E*)), 1.89 (s, 0.32H, (2*E*,8*Z*)), 1.81−1.71 (m, 2.21H), 1.26−1.90 (m, 1.32H);

^{13}C NMR (101 MHz, $CDCl_3$) δ (ppm): 167.1, 158.2, 141.2, 122.3, 52.1, 35.6, 23.6, 22.7, 19.9, 13.7;

HRMS (ESI): *m/z* $[M+Na]^+$ calcd for $C_{10}H_{17}NNaO_3^+$: 222.1101; found: 222.1099.

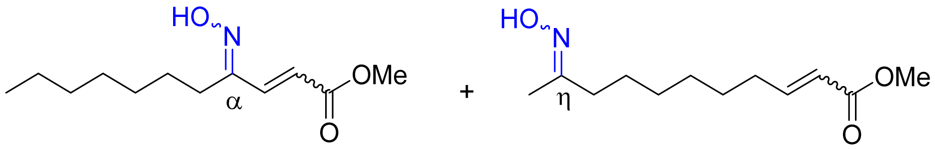

Mmethyl 4-(hydroxyimino)undec-2-enoate (54a-α-oxime) and **methyl-10-(hydroxyimino)undec-2-enoate (54a-η-oxime) (54a)** was synthesized by the general procedure **B** from methyl (2*E*/2*Z*)-undec-2-enoate (198.3 mg, 1 mmol, 2*E*/2Z = 3:1) and purified by flash column chromatography on silica gel (gradient elution: Petroleum ether : EtOAc = 20:1 to 5:1) to afford isolated (2*E*/2*Z*, 4*E*/4*Z*)-**54a-α-oxime** as an *E/Z* mixture, and isolated (2*E*/2*Z*,10*E*/10*Z*)-**54a-η-oxime** with small amount of other site regioisomers. No epoxide was obtained from the reaction.;

The 2E/2Z isomerism is with respect to C=C bond, ratio of ***2E/2Z=[(2E,4E) + (2E,4Z)]/[(2Z,4E) + (2Z,4Z)] = 3:1****; the 4E/4Z isomerismis with respect to C=N bond, ratio of* ***4E/4Z=[(2E,4E) + (2Z,4E)]/[(2E,4Z) + (2Z,4Z)] = 2:1;***

(2*E*/2*Z*, 4*E*/4*Z*)-**54a-α-oxime:** 111.4 mg, 49% yield, colorless oil; (2*E*/2*Z*,10*E*/10*Z*)-**54a-η-oxime:** 25% yield colorless oil (containing small amount of other site regioisomers);

(2*E*/2*Z*, 4*E*/4*Z*)-**54a-α-oxime** isomer:

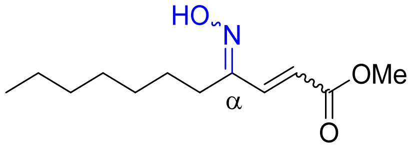

^{1}H NMR (400 MHz, $CDCl_3$) δ (ppm): 9.75 (brs, 0.78H), 7.91−7.86 (m, 0.3H, (2*E*,4Z)+(2Z,4Z)), 7.28−7.23 (m, 0.64H, (2*E*,4*E*)+(2Z,4*E*)), 6.27−6.16 (m, 1H), 3.79 (s, 0.64H, (2*E*,4Z)+(2Z,4Z))

3.78(s, 1.91H, (2*E*,4*E*)+(2*Z*,4*E*)), 2.52 (t, *J* = 8.0 Hz, 1.29H, (2*E*,4*E*)+(2*Z*,4*E*)), 2.40 (t, *J* = 7.6 Hz, 0.65H, (2*E*,4Z)+(2Z,4Z)), 1.57−1.45 (m, 2.16H), 1.37−1.24 (m, 8.30H), 0.86 (t, *J* = 6.8 Hz, 3.12H); **^{13}C NMR** (101 MHz, $CDCl_3$) δ (ppm): 167.0 ((2*E*,4*E*)+(2*E*,4*Z*)), 166.6 ((2*Z*,4*E*)+(2*Z*,4*Z*)), 159.14 (2*Z*, 4*E*), 159.08 (2*E*, 4*E*), 154.8 (2*Z*, 4*Z*), 154.7 (2*E*, 4*Z*), 141.3 (2*E*, 4*E*), 141.0 (2*Z*, 4*E*), 130.9 (2*E*,4*Z*), 130.6 (2*Z*,4*Z*), 125.5 (2*Z*, 4*Z*), 125.0 (2*E*, 4*Z*), 122.8 (2*Z*, 4*E*), 122.3 (2*E*, 4*E*), 61.2 (2*Z*, 4*Z*), 61.0 (2*Z*, 4*E*), 52.2 (2*E*, 4Z), 52.0 (2*E*, 4*E*), 31.8, 31.0, 29.9, 29.4, 29.11, 29.06, 27.3, 26.3, 24.4, 22.7, 14.3, 14.2.
HRMS (ESI): *m/z* $[M+Na]^+$ calcd for $C_{12}H_{21}NNaO_3^+$: 250.1414; found: 250.1408.

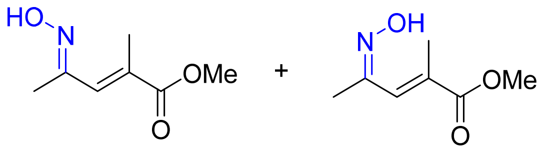

Methyl (2*E*,4*E*)-4-(hydroxyimino)-2-methylpent-2-enoate and **methyl (2*E*,4*Z*)-4-(hydroxyimino)-2-methylpent-2-enoate (55a)** was synthesized by the general procedure **B** from methyl (*E*)-pent-2-enoate (128.2 mg, 1 mmol) and purified by flash column chromatography on silica gel (gradient elution: Petroleum ether : EtOAc = 40:1) to afford (2*E*,4*E*)-**55a** and (2*E*,4*Z*)-**55a** as two separated single isomers. Epoxide was also obtained from the reaction.
(2*E*,4*E*)-**55a** isomer:

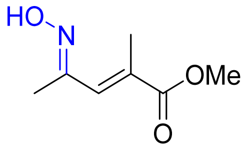

(2*E*,4*E*)-**55a:** 36.2 mg, 23%, white solid; **^{1}H NMR** (400 MHz, $CDCl_3$) δ (ppm): 9.15 (brs, 1H), 7.00 (s, 1H), 3.78 (s, 3H), 2.09 (s, 3H), 2.07 (s, 3H); **^{13}C NMR** (101 MHz, $CDCl_3$) δ (ppm): 168.7, 154.8, 134.2, 132.0, 52.4, 14.90, 14.86; **HRMS (ESI):** *m/z* $[M+Na]^+$ calcd for $C_7H_{11}NNaO_3^+$: 180.0631; found: 180.0628.
(2*E*,4*Z*)-**55a** isomer:

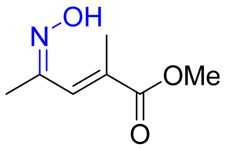

(2*E*,4*Z*)-**55a:** 20.5 mg, 13% yield, white solid; **^{1}H NMR** (400 MHz, $CDCl_3$) δ (ppm):8.57 (brs, 1H), 7.20 (s. 1H), 3.78 (s, 3H), 2.05 (s, 3H), 1.94 (s, 3H); **^{13}C NMR** (101 MHz, $CDCl_3$) δ (ppm): 168.0, 152.9, 132.8, 130.4, 52.4, 19.9, 15.3; **HRMS (ESI):** *m/z* $[M+Na]^+$ calcd for $C_7H_{11}NNaO_3^+$: 180.0631; found: 180.0629.

Methyl 3-ethyl-2-methyloxirane-2-carboxylate (55b):

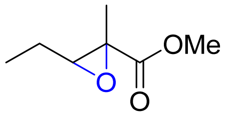

55b: 28.8mg, 20% yield, colorless oil; **^{1}H NMR** (400 MHz, $CDCl_3$) δ (ppm): 3.75 (s, 3H), 3.14 (t, *J* = 6.4 Hz, 1H), 1.66–1.58 (m, 2H), 1.52 (s, 3H), 1.06 (t, *J* = 7.6 Hz, 3H); **^{13}C NMR** (101 MHz, $CDCl_3$) δ (ppm): 174.7, 63.6, 57.8, 52.7, 21.6, 13.5, 10.4; **HRMS (ESI):** *m/z* $[M+Na]^+$ calcd for $C_7H_{12}NaO_3^+$: 167.0679; found: 167.0684.

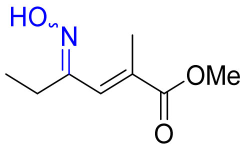

Methyl (2*E*,4*E*/4*Z*)-4-(hydroxyimino)-2-methylhex-2-enoate (56a) was synthesized by the general procedure **B** from methyl (*E*)-2-methylhex-2-enoate (142.2 mg, 1 mmol) and purified by flash column chromatography on silica gel (gradient elution: Petroleum ether : EtOAc = 40:1) to afford isolated product (2*E*,4*E*/4*Z*)-**56a** as an *E/Z* mixture. Epoxide was also obtained from the reaction.

*The E/Z isomerism is with respect to C=N bond. Ratio of **(2E,4E)/(2E,4Z) = 2:1;***

56a: 60.2 mg, 35% yield, white solid; **rsm**: <5%;

Major (2*E*,4*E*)-isomer: **^{1}H NMR** (400 MHz, $CDCl_3$) δ (ppm): 8.88 (brs, 1H), 6.97 (s, 1H), 3.78 (s, 3H), 2.51 (q, *J* = 7.6 Hz, 2H), 2.07 (s, 3H), 1.10 (t, *J* = 4.0 Hz, 3H); **^{13}C NMR** (101 MHz, $CDCl_3$) δ (ppm): 168.6, 159.3, 132.8, 130.5, 52.4, 21.9, 14.8, 10.3.

Minor (2*E*,4*Z*)-isomer: **^{1}H NMR** (400 MHz, $CDCl_3$) δ (ppm): 8.88 (brs, 1H), 7.03 (s, 1H), 3.78 (s, 3H), 2.36 (q, *J* = 7.6 Hz, 2H), 1.88 (s, 3H), 1.09 (t, *J* = 4.0 Hz, 3H); **^{13}C NMR** (101 MHz, $CDCl_3$) δ (ppm): 167.8, 157.1, 133.2, 132.7, 52.4, 27.4, 15.5, 11.1.

HRMS (ESI): *m/z* $[M+H]^+$ calcd for $C_8H_{13}NNaO_3^+$: 194.0788; found: 194.0789.

Methyl 2-methyl-3-propyloxirane-2-carboxylate (56b):

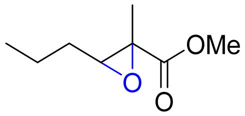

56b: 45.9 mg, 29% yield, colorless oil; **^{1}H NMR** (400 MHz, $CDCl_3$) δ (ppm): 3.71 (s, 3H), 3.14 (t, *J* = 6.0 Hz, 1H), 1.58–1.47 (m, 4H), 1.48 (s, 3H), 0.95 (t, *J* = 7.2 Hz, 3H);**^{13}C NMR** (101 MHz, $CDCl_3$) δ (ppm): 172.2, 62.4, 57.5, 52.6, 30.0, 19.7, 13.9, 13.6; **HRMS (ESI):** *m/z* $[M+Na]^+$ calcd for $C_8H_{14}NaO_3^+$: 181.0835; found: 181.0834.

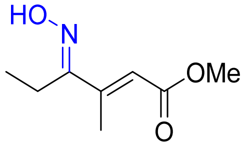

Methyl (2*E*,4*E*)-4-(hydroxyimino)-3-methylhex-2-enoate (57a) was synthesized by the general procedure **B** from methyl (*E*)-3-methylhex-2-enoate (142.2 mg, 1 mmol) and purified by flash column chromatography on silica gel (gradient elution: Petroleum ether : EtOAc = 40:1) to afford the product (2*E*,4*E*)-**57a** as a single *E*-isomer. No epoxide was obtained from the reaction.

57a: 82.2 mg, 48% yield, white solid; **rsm**: 21.1 mg, 15%;

^{1}H NMR (400 MHz, $CDCl_3$) δ (ppm): 9.19 (brs, 1H), 6.14 (s, 1H), 3.75 (s, 3H), 2.59 (q, *J* = 7.6 Hz, 2H), 2.33 (s, 3H), 1.09 (t, *J* = 7.6 Hz, 3H);

^{13}C NMR (101 MHz, $CDCl_3$) δ (ppm): 167.2, 162.1, 149.8, 119.2, 51.5, 18.0, 14.4, 11.1;

HRMS (ESI): *m/z* $[M+H]^+$ calcd for $C_8H_{14}NO_3^+$: 172.0968; found: 172.0960.

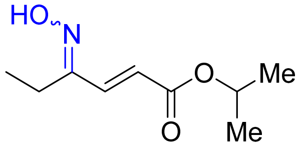

Isopropyl (2*E*,4*E*/4*Z*)-4-(hydroxyimino)hex-2-enoate (58a) was synthesized by the general procedure **B** from methyl isopropyl (*E*)-hex-2-enoate (156.2 mg, 1 mmol) and purified by flash column chromatography on silica gel (gradient elution: Petroleum ether : EtOAc = 20:1) to afford the product (2*E*,4*E*/4*Z*)**-58a** as a mixture of *E/Z* isomers. No epoxide was obtained from the reaction.

The E/Z isomerism is with respect to C=N bond. Ratio of ***(2E,4E)/(2E,4Z) = 7:3;***

58a: 124.1 mg, 67% yield, white solid; **rsm**: <5%;

Major (2*E*,4*E*)-isomer: **^{1}H NMR** (400 MHz, $CDCl_3$) δ (ppm): 9.97 (brs, 1H), 7.22 (d, *J* = 16.4 Hz, 1H), 6.18 (d, *J* = 16.0 Hz, 1H), 5.14–5.03 (m, 1H), 2.54 (q, *J* = 7.6 Hz, 2H), 1.26 (d, *J* = 6.4 Hz, 6H), 1.09 (t, *J* = 7.6 Hz, 3H); **^{13}C NMR** (101 MHz, $CDCl_3$) δ (ppm): 166.2, 160.0, 140.3, 123.2, 68.5, 21.9, 17.7, 10.7.

Minor (2*E*,4*Z*)-isomer: **^{1}H NMR** (400 MHz, $CDCl_3$) δ (ppm): 9.97 (brs, 1H), 7.86 (d, *J* = 16.4 Hz, 1H), 6.22 (d, *J* = 16.4 Hz, 1H), 5.14–5.03 (m, 1H), 2.44 (q, *J* = 7.6 Hz, 2H), 1.27 (d, *J* = 6.4 Hz, 6H), 1.14 (t, *J* = 7.6 Hz, 3H); **^{13}C NMR** (101 MHz, $CDCl_3$) δ (ppm): 166.2, 155.4, 130.2, 125.9, 68.7, 24.3, 21.9, 11.6.

HRMS (ESI): *m/z* $[M+Na]^+$ calcd for $C_9H_{15}NNaO_3^+$: 208.0944; found: 208.0940.

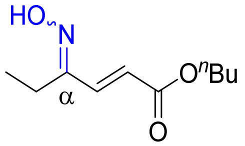

Butyl (2*E*,4*E*/4*Z*)-4-(hydroxyimino)hex-2-enoate (59a) was synthesized by the general procedure **B** from butyl (*E*)-hex-2-enoate (170.3 mg, 1 mmol) and purified by flash column chromatography on silica gel (gradient elution: Petroleum ether : EtOAc = 20:1) to afford the product (2*E*,4*E*/4*Z*)**-59a** as a mixture of *E/Z* isomers. No epoxide was obtained from the reaction. *The E/Z isomerism is with respect to C=N bond. Ratio of* ***(2E,4E)/(2E,4Z) =7:3****.*

59a: 133.5 mg, 67% yield, colorless oil; **rsm**: <5%;

Major (2*E*,4*E*)-isomer: **^{1}H NMR** (400 MHz, $CDCl_3$) δ (ppm): 9.89 (brs, 1H), 7.26 (d, *J* = 16.0 Hz, 1H), 6.23 (d, *J* = 16.0 Hz, 1H), 4.23–4.18 (m, 2H), 2.57 (q, *J* = 7.6 Hz, 2H), 1.72–1.64 (m, 2H), 1.47–1.37 (m, 2H), 1.12 (t, *J* = 7.6 Hz, 3H), 0.95 (t, *J* = 7.6 Hz, 3H); **^{13}C NMR** (101 MHz, $CDCl_3$) δ (ppm): 166.7, 160.1, 140.5, 122.7, 64.9, 30.8, 19.2, 13.8, 10.7.

Minor (2*E*,4*Z*)-isomer: **^{1}H NMR** (400 MHz, $CDCl_3$) δ (ppm): 9.89 (brs, 1H), 7.91 (d, *J* = 16.4 Hz, 1H), 6.27 (d, *J* = 16.0 Hz, 1H), 4.23–4.18 (m, 2H), 2.47 (q, *J* = 7.6 Hz, 2H), 1.72–1.64 (m, 2H), 1.47–1.37 (m, 2H), 1.18 (t, *J* = 7.6 Hz, 3H), 0.95 (t, *J* = 7.6 Hz, 3H); **^{13}C NMR** (101 MHz, $CDCl_3$) δ (ppm): 166.7, 155.4, 130.5, 125.4, 65.1, 30.7, 24.4, 17.7, 11.6.

HRMS (ESI): *m/z* $[M+Na]^+$ calcd for $C_{10}H_{17}NNaO_3^+$: 222.1101; found: 222.1092.

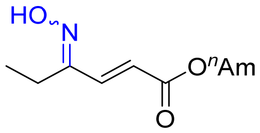

Pentyl (2*E*,4*E*)- and (2*E*,4*Z*)-4-(hydroxyimino)hex-2-enoate (60a) was synthesized by the general procedure **B** from pentyl (*E*)-hex-2-enoate (184.3 mg, 1 mmol) and purified by flash column chromatography on silica gel (gradient elution: Petroleum ether : EtOAc = 20:1) to afford the product (2*E*,4*E*/4*Z*)**-60a** as a mixture of *E/Z* isomers. No epoxide was obtained from the reaction.

*The E/Z isomerism is with respect to C=N bond. Ratio of **(2E,4E)/(2E,4Z) =7:3***;

60a: 117.3 mg, 55% yield, colorless oil; **rsm**: 38.7 mg, 21%;

Major (2*E*,4*E*)-isomer: ^{1}H NMR (400 MHz, $CDCl_3$) δ (ppm): 9.79 (brs, 1H), 7.19 (d, *J* = 16.0 Hz, 1H), 6.16 (d, *J* = 16.0 Hz, 1H), 4.15–4.09 (m, 2H), 2.49 (q, *J* = 7.6 Hz, 2H), 1.64–1.59 (m, 2H), 1.31−1.27 (m, 4H), 1.04 (t, *J* = 7.6 Hz, 3H), 0.84 (t, *J* = 6.8 Hz, 3H); **^{13}C NMR** (101 MHz, $CDCl_3$) δ (ppm): 166.7, 160.1, 140.5, 122.7, 65.2, 28.4, 28.2, 22.4, 14.0.

Minor (2*E*,4*Z*)-isomer: ^{1}H NMR (400 MHz, $CDCl_3$) δ (ppm): 9.79 (brs, 1H), 7.84 (d, *J* = 16.4 Hz, 1H), 6.20 (d, *J* = 16.0 Hz, 1H), 4.15–4.09 (m, 2H), 2.40 (q, *J* = 7.6 Hz, 2H), 1.64–1.59 (m, 2H), 1.31−1.27 (m, 4H), 1.10 (t, *J* = 7.6 Hz, 3H), 0.84 (t, *J* = 6.8 Hz, 3H); **^{13}C NMR** (101 MHz, $CDCl_3$) δ (ppm): 166.7, 155.4, 130.5, 125.4, 65.4, 28.4, 24.4, 17.7, 11.6.

HRMS (ESI): *m/z* $[M+Na]^+$ calcd for: $C_{11}H_{19}NNaO_3^+$: 236.1257; found: 236.1249.

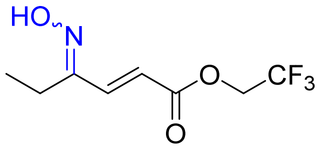

2,2,2-Trifluoroethyl (2*E*,4*E*/4*Z*)-4-(hydroxyimino)hex-2-enoate (61a) was synthesized by the general procedure **B** from 2,2,2-trifluoroethyl (*E*)-hex-2-enoate (196.2 mg, 1 mmol) and purified by flash column chromatography on silica gel (gradient elution: Petroleum ether : EtOAc = 10:1) to afford the product (2*E*,4*E*/4Z)**-61a** as a mixture of *E/Z* isomers. No epoxide was obtained from the reaction.

*The E/Z isomerism is with respect to C=N bond. Ratio of **(2E,4E)/(2E,4Z) =7:1;***

61a: 27.1 mg, 12% yield, colorless oil;

Major (2*E*,4*E*)-isomer: ^{1}H NMR (400 MHz, $CDCl_3$) δ (ppm): 9.04 (brs, 1H), 7.35 (d, *J* = 16.4 Hz, 1H), 6.27 (d, *J* = 16.4 Hz, 1H), 4.63–4.52 (m, 2H), 2.57 (q, *J* = 7.6 Hz, 2H), 1.12 (t, *J* = 7.6 Hz, 3H). **Minor (2*E*,4*Z*)-isomer: ^{1}H NMR** (400 MHz, $CDCl_3$) δ (ppm): 9.04 (brs, 1H), 8.01(d, *J*

= 16.4 Hz, 1H), 6.31 (d, J = 16.4 Hz, 1H), 4.63–4.52 (m, 2H), 2.48 (q, J = 7.6 Hz, 2H), 1.18 (t, J = 7.6 Hz, 3H).

^{13}C NMR (101 MHz, $CDCl_3$) δ (ppm): 164.8, 160.1, 155.2, 143.0, 132.6, 123.1(q, J = 275.4 Hz), 123.0, 120.4, 60.8 (q, J = 36.0 Hz), 24.4, 17.7, 11.4, 10.7.

HRMS (ESI): *m/z* $[M+Na]^+$ calcd for: $C_8H_{10}F_3NNaO_3^+$: 248.0505; found: 248.0503.

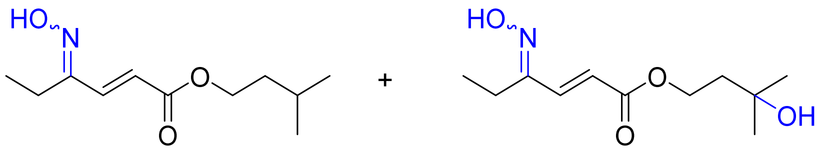

Isopentyl (2*E*,4*E*/4*Z*)-4-(hydroxyimino)hex-2-enoate (62a) and **3-hydroxy-3-methylbutyl (2*E*,4*E*/4*Z*)-4-(hydroxyimino)hex-2-enoate (62a-5'-OH)** was synthesized by the general procedure **B** from pentyl isopentyl (*E*)-hex-2-enoate (184.3 mg, 1 mmol) and purified by flash column chromatography on silica gel (gradient elution: Petroleum ether : EtOAc = 20:1) to afford separated (2*E*,4*E*/4Z)-**62a** and (2*E*,4*E*/4Z)-**62a-5'-OH** as mixture *E/Z* isomers. No epoxide was obtained from the reaction.

*The E/Z isomerism is with respect to C=N bond. Ratio of **(2E,4E)/(2E,4Z)-62a** = 7:3, **(2E,4E)/(2E,4Z)-62a-5'-OH** = 8:3*;

(2*E*,4*E*/4*Z*)-62a:

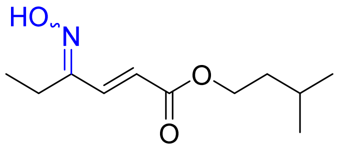

62a: 140.8 mg, 66% yield, colorless oil; **rsm**: 0%;

Major (2*E*,4*E*)-isomer: ^{1}H NMR (400 MHz, $CDCl_3$) δ (ppm): 9.80 (brs, 1H), 7.26 (d, J = 16.4 Hz, 1H), 6.23 (d, J = 16.4 Hz, 1H), 4.26–4.21 (m, 2H), 2.57 (q, J = 7.6 Hz, 2H), 1.79–1.68 (m, 1H), 1.62–1.55 (m, 2H), 1.12 (t, J =7.6 Hz, 3H), 0.94 (d, J = 6.8 Hz, 6H); **^{13}C NMR** (101 MHz, $CDCl_3$) δ (ppm): 166.7, 160.1, 140.5, 122.7, 63.7, 37.4, 25.2, 22.6, 17.7, 10.7.

Minor (2*E*,4*Z*)-isomer: ^{1}H NMR (400 MHz, $CDCl_3$) δ (ppm): 9.80 (brs, 1H), 7.91(d, J = 16.4 Hz, 1H), 6.26 (d, J = 16.4 Hz, 1H), 4.26–4.21 (m, 2H), 2.47 (q, J = 7.6 Hz, 2H), 1.79–1.68 (m, 1H), 1.62–1.55 (m, 2H), 1.17 (t, J =7.6 Hz, 3H), 0.94 (d, J = 6.8 Hz, 6H); **^{13}C NMR** (101 MHz, $CDCl_3$) δ (ppm): 166.7, 155.4, 130.5, 125.4, 63.9, 37.4, 24.4, 22.6, 17.7, 11.6.

HRMS (ESI): *m/z* $[M+Na]^+$ calcd for: $C_{11}H_{19}NNaO_3^+$: 236.1257; found: 236.1252.

(2*E*,4*E*/4*Z*)-62a-5'-OH:

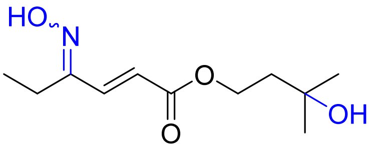

62a-5'-OH: 25.2 mg, 11% yield, colorless oil;

Major (2*E*,4*E*)-isomer: ^{1}H NMR (400 MHz, $CDCl_3$) δ (ppm): 9.07 (brs, 1H), 7.02 (d, *J* = 16.4 Hz, 1H), 5.96 (d, *J* = 16.4 Hz, 1H), 4.18–4.11 (m, 2H), 2.30 (q, *J* = 7.6 Hz, 2H), 1.71–1.65 (m, 2H), 1.05 (s, 6H), 0.86 (t, *J* = 7.6 Hz, 3H); **^{13}C NMR** (101 MHz, $CDCl_3$) δ (ppm): 166.6, 160.1, 141.0, 122.3, 70.4, 62.0, 41.7, 29.8, 17.7, 10.7.

Minor (2*E*,4*Z*)-isomer: ^{1}H NMR (400 MHz, $CDCl_3$) δ (ppm): 9.07 (brs, 1H), 7.66 (d, *J* = 16.4 Hz, 1H), 5.99 (d, *J* = 16.4 Hz, 1H), 4.18–4.11 (m, 2H), 2.21 (q, *J* = 7.6 Hz, 2H), 1.71–1.65 (m, 2H), 1.06 (s, 6H), 0.91 (t, *J* = 7.6 Hz, 3H); **^{13}C NMR** (101 MHz, $CDCl_3$) δ (ppm): 166.6, 155.4, 130.8, 125.0, 70.4, 62.1, 29.8, 28.1, 24.4, 11.7.

HRMS (ESI): *m/z* $[M+Na]^+$ calcd for: $C_{11}H_{19}NNaO_4^+$: 252.1206; found: 252.1207.

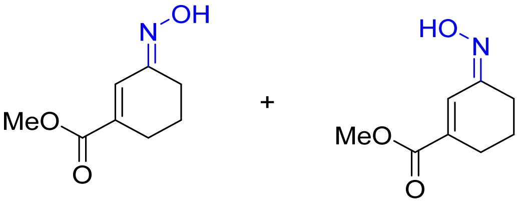

Methyl (*E/Z*)-3-(hydroxyimino)cyclohex-1-ene-1-carboxylate (63a) was synthesized by the general procedure **B** from methyl cyclohex-1-ene-1-carboxylate (140.2 mg, 1 mmol) and purified by flash column chromatography on silica gel (gradient elution: Petroleum ether : EtOAc = 40:1) to afford *E*-**63a** and *Z*-**63a** as two separated single isomers. No epoxide was obtained from the reaction;

***E*-63a isomer:**

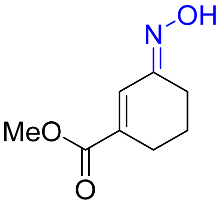

***E*-63a:** 47.4 mg, 28% yield, white solid; **^{1}H NMR** (400 MHz, $CDCl_3$) δ (ppm): 9.70 (brs, 1H), 7.13 (s, 1H), 3.78 (s, 3H), 2.61 (t, *J* = 6.6 Hz, 2H), 2.43 (t, *J* =6.0 Hz, 2H), 1.79 (quint, *J* = 6.4 Hz,

2H); **^{13}C** NMR (101 MHz, $CDCl_3$) δ (ppm): 167.4, 156.5, 136.5, 132.3, 52.2, 24.3, 22.0, 20.6; **HRMS (ESI):** *m/z* $[M+Na]^+$ calcd for $C_8H_{11}NNaO_3^+$: 192.0631; found: 192.0632.

***Z*-63a isomer:**

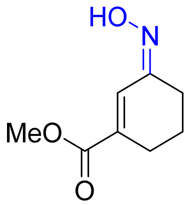

***Z*-63a:** 53.2 mg, 32% yield, white solid; **^{1}H NMR** (400 MHz, $CDCl_3$) δ (ppm): 9.50 (brs, 1H), 7.76 (s, 1H), 3.79 (s, 3H), 2.47 (t, *J* = 6.0 Hz, 2H), 2.41 (t, *J* =6.4 Hz, 2H), 1.86 (quint, *J* = 6.4 Hz, 2H); **^{13}C NMR** (101 MHz, $CDCl_3$) δ (ppm):167.7, 153.0, 139.0, 123.0, 52.3, 27.7, 25.5, 22.0; **HRMS (ESI):** *m/z* $[M+Na]^+$ calcd for $C_8H_{11}NNaO_3^+$: 192.0631; found: 192.0631.

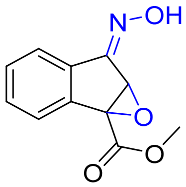

Methyl (*E*)-6-(hydroxyimino)-6,6a-dihydro-1a*H*-indeno[1,2-*b*]oxirene-1a-carboxylate (64a) was synthesized by the general procedure **B** from methyl 1*H*-indene-3-carboxylate (174.2 mg, 1 mmol) and purified by flash column chromatography on silica gel (gradient elution: Petroleum ether : EtOAc = 20:1) to afford the product *E*-**64a** as the major isomer.

64a: 61.4 mg, 28% yield, white solid; **rsm:** <5%;

^{1}H NMR (400 MHz, DMSO-*d6*) δ (ppm): 12.12 (s, 1H), 7.83–7.78 (m, 1H), 7.65–7.60 (m, 1H), 7.47–7.40 (m, 2H), 5.02 (s, 1H), 3.82 (s, 3H);**^{13}C NMR** (101 MHz, DMSO-*d6*) δ (ppm): 166.0, 151.9, 138.6, 136.2, 129.8, 129.6, 126.7, 121.7, 61.1, 55.3, 52.9;

HRMS (ESI): *m/z* $[M+Na]^+$ calcd for: $C_{11}H_9NNaO_4^+$: 242.0424; found: 242.0424.

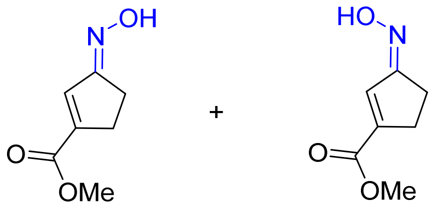

Methyl (*E*/*Z*)-3-(hydroxyimino)cyclopent-1-ene-1-carboxylate (65a) was synthesized by the general procedure **B** from methyl methyl cyclopent-1-ene-1-carboxylate (126.1 mg, 1 mmol) and

purified by flash column chromatography on silica gel (gradient elution: Petroleum ether : EtOAc = 40:1) to afford *E*-**65a** and *Z*-**65a** as two separated single isomers, and the structure of *E*-**65a** was assigned based on the X-ray structure (see Section 13). No epoxide was obtained from the reaction.

E-**65a** isomer**:**

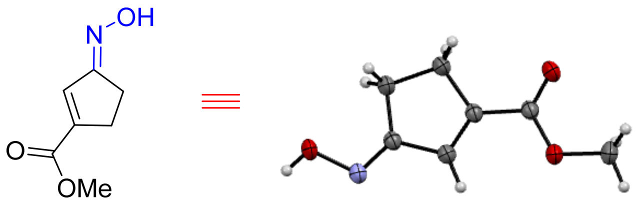

E-**65a:** 83.8 mg, 54% yield, white solid; **^{1}H NMR** (400 MHz, $CDCl_3$) δ (ppm): 9.51 (brs, 1H), 7.00 (s, 1H), 3.79 (s, 3H), 2.92–2.62 (m, 4H); **^{13}C NMR** (101 MHz, $CDCl_3$) δ (ppm): 167.4, 165.1, 147.8, 136.5, 52.1, 29.8, 25.3. The X-ray structure of ***E*-65a** has been determined.

Z-**65a** isomer**:**

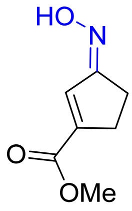

Z-**65a:** 29.6 mg, 19% yield; white solid; **^{1}H NMR** (400 MHz, $CDCl_3$) δ (ppm): 8.85 (brs, 1H), 7.42 (s, 1H), 3.81 (s, 3H), 2.74 (m, 4H); **^{13}C NMR** (101 MHz, $CDCl_3$) δ (ppm): 165.5, 164.1, 148.8, 129.7, 52.3, 29.0, 27.0.

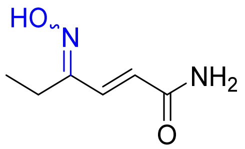

(*2E*,*4E*/*4Z*) 4-(hydroxyimino)hex-2-enamide (66a) was synthesized by the general procedure **B** from (*E*)-hex-2-enamide (113.2 mg, 1 mmol) and purified by flash column chromatography on silica gel (gradient elution: Petroleum ether : EtOAc = 3:1 to 1:1) to afford the product (2*E*,4*E*/4*Z*)-**66a** as a mixture of *E/Z* isomers. And epoxide was also obtained from the reaction.

*The E/Z isomerism is with respect to C=N bond., Ratio of **(2E,4E)/(2E,4Z) = 3:1;***

66a: 25.6 mg, 18% yield, white solid; **rsm**: <5%;

Major (2*E*,4*E*)-isomer: **^{1}H NMR** (400 MHz, DMSO-*d6*) δ (ppm): 11.58 (s, 1H), 7.56 (brs, 1H), 7.15 (brs, 1H), 6.89 (d, *J* =16.0 Hz, 1H), 6.33 (d, *J* =16.0 Hz, 1H), 2.42 (q, *J* =7.6 Hz, 2H), 1.00 (t, *J* =7.6 Hz, 3H); **^{13}C NMR (**101 MHz**,** DMSO-*d6***)** δ (ppm): 166.1, 157.8, 135,8, 124.7, 16.8, 10.3.

Minor (2*E*,4*Z*)-isomer: **^{1}H NMR** (400 MHz, DMSO-*d6*) δ (ppm): 11.24 (s, 1H), 7.65 (brs, 1H), 7.55 (d, *J* =16.4 Hz, 1H), 7.25 (brs, 1H), 6.36 (d, *J* =16.4 Hz, 1H), 2.36 (q, *J* =7.6 Hz, 2H), 1.07 (t, *J* =7.6 Hz, 3H); **^{13}C NMR (**101 MHz**,** DMSO-*d6*) δ (ppm): 166.2, 153.2, 127.2, 125.7, 23.5, 11.5.

HRMS (ESI): *m/z* $[M+Na]^+$ calcd for $C_6H_{10}N_2NaO_2^+$: 165.0634; found: 165.0631.

3-Propyloxirane-2-carboxamide (66b):

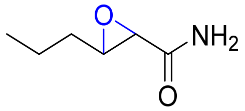

66b: 43.9 mg, 34% yield; **^{1}H NMR** (400 MHz, $CDCl_3$) δ (ppm): 6.59 (brs, 1H), 6.21 (brs, 1H), 3.15–3.13 (m, 1H), 3.00–2.95 (m, 1H), 1.63–1.40 (m, 4H), 0.92 (t, *J* = 7.2 Hz, 3H); **^{13}C NMR** (101 MHz, $CDCl_3$) δ (ppm): 172.4, 59.4, 55.0, 33.7, 19.00, 13.8; **HRMS (ESI):** *m/z* $[M+Na]^+$ calcd for $C_6H_{11}NNaO_2^+$: 152.0682; found: 152.0679.

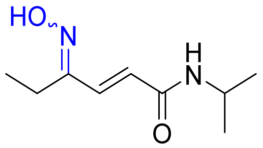

(2*E*,4*E*/4*Z*)-4-(hydroxyimino)-*N*-isopropylhex-2-enamide (67a) was synthesized by the general procedure **B** from (*E*)-*N*-isopropylhex-2-enamide (155.2 mg, 1 mmol) and purified by flash column chromatography on silica gel (gradient elution: Petroleum ether : EtOAc = 3:1) to afford one product (2*E*,4*E*/4*Z*)-**67a** as an *E/Z* mixture, and another product (2*E*,4*E*/4*Z*)-**66a** as an *E/Z* mixture. No epoxide was obtained from the reaction.

The of E/Z isomerism is with respect to C=N bond. Ratio of ***(2E,4E)/(2E,4Z) = 4:1;***

67a: 119.7 mg, 65% yield, white solid; and **66a**, 29.9 mg, 21% yield; **rsm**: <5%;

Major (2*E*,4*E*)-isomer: **^{1}H NMR** (400 MHz, DMSO-*d6*) δ (ppm): 11.55 (s, 1H), 8.01 (d, *J* =7.6 Hz, 1H), 6.87 (d, *J* =16.0 Hz, 1H), 6.32 (d, *J* =16.0 Hz, 1H), 3.97–3.84 (m, 1H), 2.41 (q, *J* =7.6 Hz, 2H), 1.08 (d, *J* =6.4 Hz, 6H), 0.99 (t, *J* =7.4 Hz, 3H); **^{13}C NMR (**101 MHz, DMSO-*d6***)** δ (ppm): 163.3, 157.8, 135.0, 125.0, 40.2, 22.1, 16.8, 10.3.

Minor (2*E*,4*Z*)-isomer: **^{1}H NMR** (400 MHz, DMSO-*d6*) δ (ppm): 11.21 (s, 1H), 8.12 (d, *J* =7.6 Hz, 1H), 7.54 (d, *J* =16.4 Hz, 1H), 6.36 (d, *J* =16.4 Hz, 1H), 3.97–3.84 (m, 1H), 2.35 (q, *J* =7.6

Hz, 2H), 1.08 (d, *J* =6.4 Hz, 6H), 0.99 (t, *J* =7.4 Hz, 3H); **^{13}C NMR (**101 MHz**,** DMSO-*d*6**)** δ (ppm):163.4, 153.3, 127.5, 124.9, 40.3, 23.6, 22.0, 11.5.

HRMS (ESI): *m/z* [M+Na]$^+$ calcd for $C_9H_{16}N_2NaO_2^+$: 207.1104; found: 207.1109.

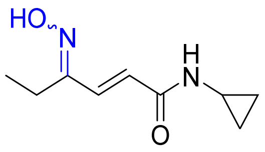

(2*E*,4*E*/4*Z*)-*N*-cyclopropyl-4-(hydroxyimino)hex-2-enamide (68a) was synthesized by the general procedure **B** from (*E*)-*N*-cyclopropylhex-2-enamide (153.2 mg, 1 mmol) and purified by flash column chromatography on silica gel (gradient elution: Petroleum ether : EtOAc = 3:1) to afford the product (2*E*,4*E*/4*Z*)-**68a** as a mixture of *E/Z* isomers. No epoxide was obtained from the reaction.

The E/Z isomerism is with respect to C=N bond. Ratio of ***(2E,4E)/(2E,4Z) = 4:1***;

68a: 93.0 mg, 51%, white solid; **rsm:** 23.0 mg, 15%;

Major (2*E*,4*E*)-isomer: ^{1}H NMR (400 MHz, DMSO-*d*6) δ (ppm): 11.56 (s, 1H), 8.19 (s, 1H), 6.89 (d, *J* =16.0 Hz, 1H), 6.25 (d, *J* =16.0 Hz, 1H), 2.78–2.68 (m, 1H), 2.40 (q, *J* =7.6 Hz, 2H), 0.98 (t, *J* =7.6 Hz, 3H), 0.68–0.63 (m, 2H), 0.46–0.39 (m, 2H); **^{13}C NMR (**101 MHz, DMSO-*d*6**)** δ (ppm): 165.4, 157.7, 135.0, 124.3, 22.2, 16.7, 10.3, 5.5.

Minor (2*E*,4*Z*)-isomer: ^{1}H NMR (400 MHz, DMSO-*d*6) δ (ppm): 11.23 (s, 1H), 8.30 (s, 1H), 7.55 (d, *J* =16.4 Hz, 1H), 6.29 (d, *J* =16.4 Hz, 1H), 2.78–2.68 (m, 1H), 2.34 (q, *J* =7.6 Hz, 2H), 1.06 (t, *J* =7.6 Hz, 3H), 0.68–0.63 (m, 2H), 0.46–0.39 (m, 2H); **^{13}C NMR (**101 MHz**,** DMSO-*d*6**)** δ (ppm):165.5, 153.2, 126.9, 124.9, 23.5, 22.2, 11.4, 5.5.

HRMS (ESI): *m/z* [M+Na]$^+$ calcd for $C_9H_{14}N_2NaO_2^+$: 205.0947; found: 205.0943.

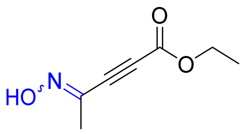

Ethyl (*E*/*Z*)-4-(hydroxyimino)pent-2-ynoate (69a) was synthesized by the general procedure **B** from ethyl pent-2-ynoate (126.2 mg, 1 mmol) and purified by flash column chromatography on silica gel (gradient elution: Petroleum ether : EtOAc = 20:1) to afford the product(*E/Z*)-**69a** as a mixture of *E/Z* isomers.

The E/Z isomerism is with respect to C=N bond. Ratio of E/Z = 1:1.

69a: 32.6 mg, 21% yield, colorless oil;

^{1}H NMR (400 MHz, $CDCl_3$) δ (ppm): 9.06 (brs, 1H), 4.32–4.23 (m, 2H), 2.10 (s, 1.5H), 2.07 (s, 1.5H), 1.34–1.30 (m, 3H);

^{13}C NMR (101 MHz, $CDCl_3$) δ (ppm): 153.4, 153.2, 141.6, 137.1, 89.3, 81.2, 80.3, 75.7, 62.8, 62.6, 19.6, 15.5, 14.1.

HRMS (ESI): *m/z* $[M+Na]^+$ calcd for $C_7H_9NNaO_3^+$: 178.0475; found 178.0475.

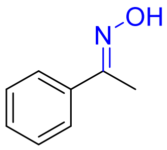

(*E*)-1-Phenylethan-1-one oxime (70a) [112] was synthesized by the general procedure **C** from ethylbenzene (53.1 mg, 0.5 mmol) and purified by flash column chromatography on silica gel (gradient elution: Petroleum ether : EtOAc = 10:1) to afford the product *E*-**70a** as a single isomer.

70a: 52.7 mg, 78% yield, white solid;

^{1}H NMR (400 MHz, $CDCl_3$) δ (ppm): 8.76 (brs, 1H), 7.70–7.56 (m, 2H), 7.45–7.33 (m, 3H), 2.31 (s, 3H); **^{13}C NMR** (101 MHz, $CDCl_3$) δ (ppm): 156.1, 136.6, 129.4, 128.7, 126.2, 12.5; **HRMS** (ESI) *m/z* $[M+H]^+$ calcd for $C_8H_{10}NO^+$:136.0757; found 136.0757.

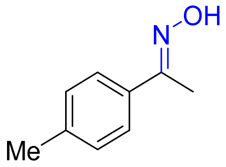

(*E*)-1-(*p*-Tolyl)ethan-1-one oxime (71a) [120] was synthesized by the general procedure **C** from 1-ethyl-4-methylbenzene (60.1 mg, 0.5 mmol) and purified by flash column chromatography on silica gel (gradient elution: Petroleum ether : EtOAc = 10:1) to afford the product *E*-**71a** as a single isomer.

71a: 64.5 mg, 86% yield, white solid;

^{1}H NMR (400 MHz, $CDCl_3$) δ (ppm): 8.99 (brs, 1H), 7.53 (d, *J* = 8.0 Hz, 2H), 7.20 (d, *J* = 8.0 Hz, 2H), 2.37 (s, 3H), 2.29 (s, 3H); **^{13}C NMR** (101 MHz, $CDCl_3$) δ (ppm): 156.1, 139.4, 133.9, 129.4, 126.1, 21.4, 12.3; **HRMS** (ESI) *m/z* $[M+H]^+$ calcd for $C_9H_{12}NO^+$:150.0913, found 150.0920.

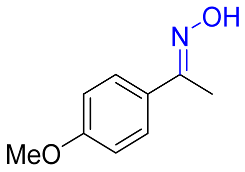

(*E*)-1-(4-Methoxyphenyl)ethan-1-one oxime (72a) [120] was synthesized by the general procedure **C** from 1-ethyl-4-methoxybenzene (68.1 mg, 0.5 mmol) and purified by flash column chromatography on silica gel (gradient elution: Petroleum ether : EtOAc = 10:1) to afford the product *E*-**72a** as a single isomer.

72a: 54.8 mg, 66% yield, white solid;

^{1}H NMR (400 MHz, $CDCl_3$) δ (ppm): 8.81 (brs, 1H), 7.58 (d, *J* = 8.4 Hz, 2H), 6.91 (d, *J* = 8.4 Hz, 2H), 3.83 (s, 3H), 2.28 (s, 3H); **^{13}C NMR** (101 MHz, $CDCl_3$) δ (ppm): 160.6, 155.7, 129.2, 127.5, 114.0, 55.5, 12.3; **HRMS** (ESI) *m/z* $[M+Na]^+$ calcd for $C_9H_{11}NNaO_2^+$ 188.0682, found 188.0679.

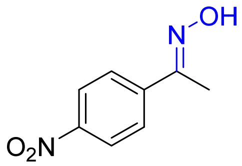

(*E*)-1-(4-Nitrophenyl)ethan-1-one oxime (73a) [120] was synthesized by the general procedure **C** from 1-ethyl-4-nitrobenzene (75.6 mg, 0.5 mmol) and purified by flash column chromatography on silica gel (gradient elution: Petroleum ether : EtOAc =10:1 to 6:1) to afford the product *E*-**73a** as a single isomer.

73a: 79.5 mg, 88% yield, white solid;

^{1}H NMR (400 MHz, $CDCl_3$ δ (ppm): 8.24 (d, *J* = 8.4 Hz, 2H), 8.07 (brs, 1H), 7.81 (d, *J* = 8.4 Hz, 2H), 2.32 (s, 3H); **^{13}C NMR** (101 MHz, CD_3OD) δ (ppm): 153.8, 149.1, 144.8, 127.8, 124.4, 11.5; **HRMS** (ESI) *m/z* $[M+Na]^+$ calcd for $C_8H_8N_2NaO_3^+$: 203.0427, found 203.0428.

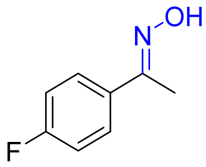

(*E*)-1-(4-Fluorophenyl)ethan-1-one oxime (74a) [120] was synthesized by the general procedure **C** from 1-ethyl-4-fluorobenzene (62.1 mg, 0.5 mmol) and purified by flash column chromatography on silica gel (gradient elution: Petroleum ether : EtOAc = 10:1) to afford the product *E*-**74a** as a single isomer.

74a: 69.7 mg, 91% yield, white solid;

^{1}H NMR (400 MHz, $CDCl_3$) δ (ppm): 8.42 (brs, 1H), 7.64–7.58 (m, 2H), 7.10–7.04 (m, 2H), 2.28 (s, 3H); **^{13}C NMR** (101 MHz, $CDCl_3$) δ (ppm): 163.6 (d, $J_{C\text{-}F}$ = 247.0 Hz), 155.3, 132.7 (d, $J_{C\text{-}F}$ = 4.0 Hz), 128.0 (d, $J_{C\text{-}F}$ = 8.0 Hz), 115.7 (d, $J_{C\text{-}F}$ = 21.0 Hz), 12.51; **^{19}F NMR** (376 MHz, $CDCl_3$) δ (ppm): -112.2; **HRMS** (ESI) *m/z* $[M+Na]^+$ calcd for $C_8H_8FNNaO^+$: 176.0482, found 176.0488.

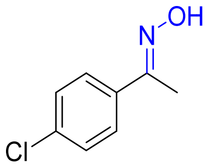

(*E*)-1-(4-Chlorophenyl)ethan-1-one oxime (75a) [120] was synthesized by the general procedure **C** from 1-chloro-4-ethylbenzene (70.3 mg, 0.5 mmol) and purified by flash column chromatography on silica gel (gradient elution: Petroleum ether : EtOAc = 10:1) to afford the product *E*-**75a** as a single isomer.

75a: 75.5 mg, 89% yield, white solid;

^{1}H NMR (400 MHz, $CDCl_3$) δ (ppm): 8.67 (brs, 1H), 7.56 (d, *J* = 8.4 Hz, 2H), 7.35 (d, *J* = 8.8 Hz, 2H), 2.28 (s, 3H); **^{13}C NMR** (101 MHz, $CDCl_3$) δ (ppm): 155.3, 135.4, 135.0, 128.9, 127.5, 12.3; **HRMS** (ESI) *m/z* $[M+H]^+$ calcd for $C_8H_9ClNO^+$: 170.0367, found 170.0372.

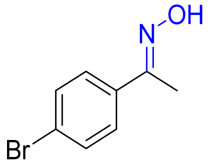

(*E*)-1-(4-Bromophenyl)ethan-1-one oxime (76a) [112] was synthesized by the general procedure **C** from 1-bromo-4-ethylbenzene (92.6 mg, 0.5 mmol) and purified by flash column chromatography on silica gel (gradient elution: Petroleum ether : EtOAc = 10:1) to afford the product *E*-**76a** as a single isomer.

76a: 92.5 mg, 86% yield, white solid;

^{1}H NMR (400 MHz, $CDCl_3$) δ (ppm): 7.96 (brs, 1H), 7.51 (s, 4H), 2.26 (s, 3H); **^{13}C NMR** (101 MHz, $CDCl_3$) δ (ppm): 155.3, 135.5, 131.8, 127.7, 123.7, 12.3; **HRMS** (ESI) m/z $[M+Na]^+$ calcd for $C_8H_8BrNaNO^+$: 235.9681, found 235.9686.

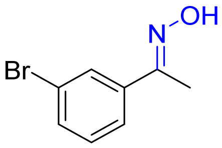

(*E*)-1-(3-Bromophenyl)ethan-1-one oxime (77a) [120] was synthesized by the general procedure **C** from 1-bromo-3-ethylbenzene (92.6 mg, 0.5 mmol) and purified by flash column chromatography on silica gel (gradient elution: Petroleum ether : EtOAc = 10:1) to afford the product *E*-**77a** as a single isomer.

77a: 85.6 mg, 80% yield, white solid;

^{1}H NMR (400 MHz, $CDCl_3$) δ (ppm): 8.52 (brs, 1H), 7.81–7.79 (m, 1H), 7.60–7.51 (m, 2H), 7.31–7.27 (m, 1H), 2.30 (s, 3H); **^{13}C NMR** (101 MHz, $CDCl_3$) δ (ppm): 155.1, 138.6, 132.3, 130.1, 129.3, 124.8, 122.8, 12.4; **HRMS** (ESI) m/z $[M+Na]^+$ calcd for $C_8H_8BrNaNO^+$: 235.9681, found 235.9690.

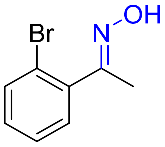

(*E*)-1-(2-Bromophenyl)ethan-1-one oxime (78a) was synthesized by the general procedure **C** from 1-bromo-2-ethylbenzene (92.6 mg, 0.5 mmol) and purified by flash column chromatography on silica gel (gradient elution: Petroleum ether : EtOAc = 10:1) to afford the product *E*-**78a** as a single isomer.

78a: 76.0 mg, 71% yield, white solid;

^{1}H NMR (400 MHz, $CDCl_3$) δ (ppm): 8.59 (brs, 1H), 7.61–7.58 (m, 1H), 7.36–7.27 (m, 2H), 7.26–7.20 (m, 1H), 2.26 (s, 3H); **^{13}C NMR** (101 MHz, $CDCl_3$) δ (ppm): 158.1, 138.9, 133.3, 130.3, 127.6, 121.9, 16.1; **HRMS** (ESI) *m/z* $[M+Na]^+$ calcd for $C_8H_8BrNaNO^+$: 235.9681, found 235.9688.

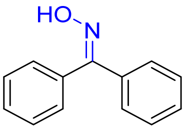

Diphenylmethanone oxime (79a) [121] was synthesized by the general procedure **C** from diphenylmethane (84.1 mg, 0.5 mmol) and purified by flash column chromatography on silica gel (gradient elution: Petroleum ether : EtOAc = 10:1) to afford the product of **79a.**

79a: 66.1 mg, 67% yield, white solid;

^{1}H NMR (400 MHz, $CDCl_3$) δ (ppm): 9.33 (brs, 1), 7.50–7.44 (m, 6H), 7.42–7.30 (m, 4H); **^{13}C NMR** (101 MHz, $CDCl_3$) δ (ppm): 158.0, 136.3, 132.8, 129.7, 129.4, 129.3, 128.5, 128.4, 128.0; **HRMS** (ESI) *m/z* $[M+Na]^+$ calcd for $C_{13}H_{11}NNaO^+$: 220.0733, found 220.0740.

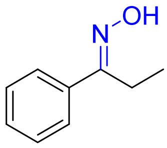

(*E*)-1-Phenylpropan-1-one oxime (80a) [120] was synthesized by the general procedure **C** from propylbenzene (60.1 mg, 0.5 mmol) and purified by flash column chromatography on silica gel (gradient elution: Petroleum ether : EtOAc = 10:1) to afford the product *E*-**80a** as a single isomer.

80a: 64.5 mg, 86% yield, white solid;

^{1}H NMR (400 MHz, $CDCl_3$) δ (ppm): 9.34 (brs, 1H), 7.65–7.61 (m, 2H), 7.42–7.37 (m, 3H), 2.84 (q, J = 7.6 Hz, 2H), 1.19 (t, J = 7.6 Hz, 3H); **^{13}C NMR** (101 MHz, $CDCl_3$) δ (ppm): 160.9, 135.7, 129.3, 128.7, 126.4, 19.9, 11.0; **HRMS** (ESI) *m/z* $[M+Na]^+$ calcd for $C_9H_{11}NNaO^+$: 172.0733, found 172.0733.

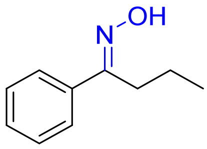

(*E*)-1-Phenylbutan-1-one oxime (81a) [120] was synthesized by the general procedure **C** from butylbenzene (67.1 mg, 0.5 mmol) and purified by flash column chromatography on silica gel (gradient elution: Petroleum ether : EtOAc = 10:1) to afford the product *E*-**81a** as a single isomer.

81a: 67.0 mg, 82% yield, white solid;

^{1}H NMR (400 MHz, $CDCl_3$) δ (ppm): 9.13 (brs, 1H), 7.63–7.59 (m, 2H), 7.43–7.36 (m, 3H), 2.81 (t, J = 7.6 Hz, 2H), 1.67–1.56 (m, 2H), 0.99 (t, J = 7.2 Hz, 3H); **^{13}C NMR** (101 MHz, $CDCl_3$) δ (ppm): 159.9, 136.0, 129.3, 128.7, 126.5, 28.2, 19.9, 14.4; **HRMS** (ESI) *m/z* $[M+Na]^+$ calcd for $C_{10}H_{13}NNaO^+$: 186.0889, found 186.0883.

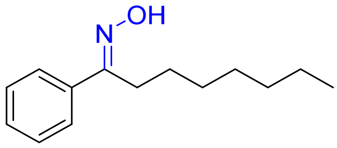

(*E*)-1-Phenyloctan-1-one oxime (82a) was synthesized by the general procedure **C** from octylbenzene (95.2 mg, 0.5 mmol) and purified by flash column chromatography on silica gel (gradient elution: Petroleum ether : EtOAc = 10:1) to afford the product *E*-**82a** as a single isomer.

82a: 87.7 mg, 80% yield, white solid;

^{1}H NMR (400 MHz, $CDCl_3$) δ (ppm): 9.20 (brs, 1H), 7.63–7.59 (m, 2H), 7.42–7.37 (m, 3H), 2.82 (t, *J* = 7.6 Hz, 2H), 1.62–1.53 (m, 2H), 1.42–1.23 (m, 8H), 0.88 (t, *J* = 6.4 Hz, 3H); **^{13}C NMR** (101 MHz, $CDCl_3$) δ (ppm): 160.1, 136.0, 129.3, 128.7, 126.5, 31.9, 30.0, 29.2, 26.5, 26.4, 22.8, 14.2; **HRMS** (ESI) *m/z* $[M+Na]^+$ calcd for $C_{14}H_{21}NNaO^+$: 242.1515, found 242.1513.

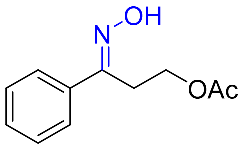

(*E*)-3-(Hydroxyimino)-3-phenylpropyl acetate (83a) was synthesized by the general procedure **C** from 3-phenylpropyl acetate (89.1 mg, 0.5 mmol) and purified by flash column chromatography on silica gel (gradient elution: Petroleum ether : EtOAc = 10:1) to afford the product *E*-**83a** as a single isomer.

83a: 86.0 mg, 83% yield, colorless oil;

^{1}H NMR (400 MHz, $CDCl_3$) δ (ppm): 9.46 (brs, 1H), 7.66–7.62 (m, 2H), 7.42–7.37 (m, 3H), 4.36 (t, *J* = 7.2 Hz, 2H), 3.18 (t, *J* = 7.2 Hz, 2H), 1.97 (s, 3H); **^{13}C NMR** (101 MHz, $CDCl_3$) δ (ppm): 171.2, 156.2, 135.6, 129.6, 128.8, 126.4, 60.9, 26.5, 21.0; **HRMS** (ESI) *m/z* $[M+Na]^+$ calcd for $C_{11}H_{13}NNaO_3^+$: 230.0788, found 230.0785.

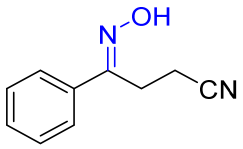

(*E*)-4-(Hydroxyimino)-4-phenylbutanenitrile (84a) was synthesized by the general procedure **C** from 4-phenylbutanenitrile (72.6 mg, 0.5 mmol) and purified by flash column chromatography on silica gel (gradient elution: Petroleum ether : EtOAc = 10:1) to to afford the product *E*-**84a** as a single isomer.

84a: 77.5 mg, 89% yield, white solid;

^{1}H NMR (400 MHz, $CDCl_3$) δ (ppm): 9.17 (brs, 1H), 7.63–7.59 (m, 2H), 7.46–7.41 (m, 3H), 3.17 (t, J = 7.6 Hz, 2H), 2.70 (t, J = 7.6 Hz, 2H); **^{13}C NMR** (101 MHz, $CDCl_3$) δ (ppm): 156.2, 134.3, 130.0, 129.0, 126.3, 118.9, 22.9, 14.0; **HRMS** (ESI) *m/z* $[M+Na]^+$ calcd for $C_{10}H_{10}N_2NaO^+$: 197.0685, found 197.0688.

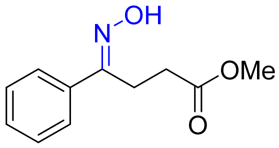

Methyl (E)-4-(hydroxyimino)-4-phenylbutanoate (85a) [121] was synthesized by the general procedure **C** from methyl 4-phenylbutanoate (89.1 mg, 0.5 mmol) and purified by flash column chromatography on silica gel (gradient elution: Petroleum ether : EtOAc = 10:1) to afford the product *E*-**85a** as a single isomer.

85a: 86.0 mg, 83% yield, colorless oil;

^{1}H NMR (400 MHz, $CDCl_3$) δ (ppm): 9.33 (brs, 1H), 7.63–7.59 (m, 2H), 7.41–7.37 (m, 3H), 3.66 (s, 3H), 3.14 (t, J = 8.0 Hz, 2H), 2.64 (t, J = 8.0 Hz, 2H); **^{13}C NMR** (101 MHz, $CDCl_3$) δ (ppm): 173.2, 158.2, 135.2, 129.6, 128.8, 126.4, 51.9, 30.6, 22.1; **HRMS** (ESI) *m/z* $[M+Na]^+$ calcd for $C_{11}H_{13}NNaO_3^+$: 230.0788, found 230.0785.

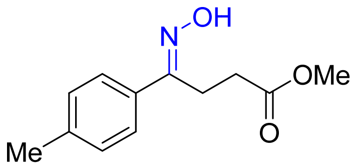

Methyl (*E*)-4-(hydroxyimino)-4-(*p*-tolyl)butanoate (86a) was synthesized by the general procedure **C** from methyl 4-(*p*-tolyl)butanoate (96.1 mg, 0.5 mmol) and purified by flash column chromatography on silica gel (gradient elution: Petroleum ether : EtOAc = 10:1) to afford the product *E*-**86a** as a single isomer.

86a: 90.7 mg, 82% yield, colorless oil;

^{1}H NMR (400 MHz, $CDCl_3$) δ (ppm): 8.60 (brs, 1H), 7.50 (d, J = 8.0 Hz, 2H), 7.19 (d, J = 8.0 Hz, 2H), 3.66 (s, 3H), 3.11 (t, J = 8.0 Hz, 2H), 2.61 (t, J = 8.8 Hz, 2H), 2.37 (s, 3H); **^{13}C NMR** (101 MHz, $CDCl_3$) δ (ppm): 173.3, 158.0, 139.7, 132.3, 129.5, 126.3, 51.9, 30.6, 22.0, 21.4; **HRMS** (ESI) *m/z* $[M+H]^+$ calcd for $C_{12}H_{16}NO_3^+$: 222.1125, found 222.1132.

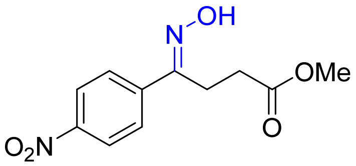

Methyl (*E*)-4-(hydroxyimino)-4-(4-nitrophenyl)butanoate (87a) was synthesized by the general procedure **C** from methyl 4-(4-nitrophenyl)butanoate (111.6 mg, 0.5 mmol) and purified by flash column chromatography on silica gel (gradient elution: Petroleum ether : EtOAc = 10:1 to 5:1) to afford the product *E*-**87a** as a single isomer.

87a: 107.0 mg, 85% yield, yellow oil;

^{1}H NMR (400 MHz, $CDCl_3$) δ (ppm): 8.94 (brs, 1H), 8.23 (d, J = 8.8 Hz, 2H), 7.80 (d, J = 8.8 Hz, 2H), 3.66 (s, 3H), 3.13 (t, J = 8.0 Hz, 2H), 2.66 (t, J = 8.0 Hz, 2H); **^{13}C NMR** (101 MHz, $CDCl_3$) δ (ppm): 173.0, 156.7, 148.4, 141.4, 127.3, 124.0, 52.1, 30.3, 21.9; **HRMS** (ESI) *m/z* $[M+Na]^+$ calcd for $C_{11}H_{12}N_2NaO_5^+$: 275.0638, found 275.0644.

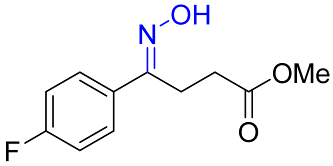

Methyl (*E*)-4-(4-fluorophenyl)-4-(hydroxyimino)butanoate (88a) was synthesized by the general procedure **C** from methyl 4-(4-fluorophenyl)butanoate (98.1 mg, 0.5 mmol) and purified by flash column chromatography on silica gel (gradient elution: Petroleum ether : EtOAc =10:1) to afford the product *E*-**88a** as a single isomer.

88a: 100.5 mg, 89% yield, colorless oil;

^{1}H NMR (400 MHz, $CDCl_3$) δ (ppm): 9.06 (brs, 1H), 7.62–7.57 (m, 2H), 7.10–7.04 (m, 2H), 3.66 (s, 3H), 3.10 (t, J = 8.0 Hz, 2H), 2.62 (t, J = 8.0 Hz, 2H); **^{13}C NMR** (101 MHz, $CDCl_3$) δ (ppm): 173.2, 163.7 (d, $J_{C\text{-}F}$= 248.0 Hz), 157.4, 131.3 (d, $J_{C\text{-}F}$= 4.0 Hz), 128.3 (d, $J_{C\text{-}F}$= 8.0 Hz), 115.8 (d, $J_{C\text{-}F}$= 22.0 Hz), 52.0, 30.5, 22.2; **^{19}F NMR** (376 MHz, $CDCl_3$) δ (ppm): -111.6; **HRMS** (ESI) *m/z* $[M+Na]^+$ calcd for $C_{11}H_{12}FNNaO_3^+$: 248.0693, found 248.0694.

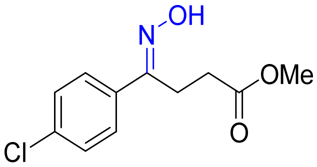

Methyl (*E*)-4-(4-chlorophenyl)-4-(hydroxyimino)butanoate (89a) was synthesized by the general procedure **C** from methyl 4-(4-chlorophenyl)butanoate (106.3 mg, 0.5 mmol) and purified by flash column chromatography on silica gel (gradient elution: Petroleum ether : EtOAc = 10:1) to afford the product *E*-**89a** as a single isomer.

89a: 103.9 mg, 86% yield, colorless oil;

^{1}H NMR (400 MHz, $CDCl_3$) δ (ppm): 9.38 (brs, 1H), 7.54 (d, J = 8.4 Hz, 2H), 7.35 (d, J = 8.4 Hz, 2H), 3.65 (s, 3H), 3.09 (t, J = 8.0 Hz, 2H), 2.62 (t, J = 8.0 Hz, 2H); **^{13}C NMR** (101 MHz, $CDCl_3$) δ (ppm): 173.2, 157.3, 135.7, 133.6, 129.0, 127.7, 52.0, 30.4, 22.0; **HRMS** (ESI) *m/z* $[M+Na]^+$ calcd for $C_{11}H_{12}ClNNaO_3^+$: 264.0398, found 264.0400.

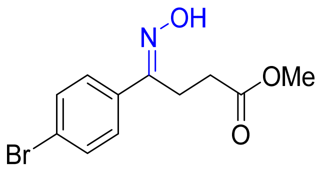

Methyl (*E*)-4-(4-bromophenyl)-4-(hydroxyimino)butanoate (90a) was synthesized by the general procedure **C** from methyl 4-(4-bromophenyl)butanoate (128.6 mg, 0.5 mmol) and purified by flash column chromatography on silica gel (gradient elution: Petroleum ether : EtOAc = 10:1) to afford the product *E*-**90a** as a single isomer.

90a: 125.9 mg, 88% yield, colorless oil;

^{1}H NMR (400 MHz, $CDCl_3$) δ (ppm): 8.82 (brs, 1H), 7.53−7.46 (m, 4H), 3.66 (s, 3H), 3.08 (t, J = 8.0 Hz, 2H), 2.61 (t, J = 8.0 Hz, 2H); **^{13}C NMR** (101 MHz, $CDCl_3$) δ (ppm): 173.1, 157.4, 134.1, 132.0, 128.0, 124.0, 52.0, 30.5, 21.9; **HRMS** (ESI) *m/z* $[M+Na]^+$ calcd for $C_{11}H_{12}BrNaO_3^+$: 307.9893, found 307.9892.

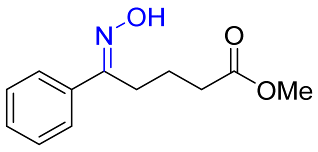

Methyl (*E*)-5-(hydroxyimino)-5-phenylpentanoate (91a) was synthesized by the general procedure **C** from methyl 5-phenylpentanoate (96.1 mg, 0.5 mmol) and purified by flash column chromatography on silica gel (gradient elution: Petroleum ether : EtOAc = 10:1) to afford the product *E*-**91a** as a single isomer.

91a: 92.9 mg, 84% yield, colorless oil;

^{1}H NMR (400 MHz, $CDCl_3$) δ (ppm): 8.45 (brs, 1H), 7.66–7.61(m, 2H), 7.42–7.36 (m, 3H), 3.67 (s, 3H), 2.87 (t, *J* = 7.6 Hz, 2H), 2.41 (t, *J* = 7.6 Hz, 2H), 1.92 (quint, *J* = 7.6 Hz, 2H); **^{13}C NMR** (101 MHz, $CDCl_3$) δ (ppm): 173.8, 159.1, 135.5, 129.5, 128.8, 126.4, 51.7, 33.7, 25.3, 21.7; **HRMS** (ESI) *m/z* $[M+Na]^+$ calcd for $C_{12}H_{15}NNaO_3^+$: 244.0944, found 244.0945.

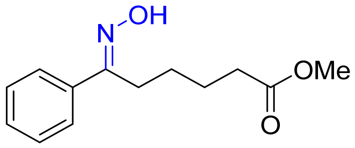

Methyl (*E*)-6-(hydroxyimino)-6-phenylhexanoate (92a) was synthesized by the general procedure **C** from methyl 6-phenylhexanoate (103.1 mg, 0.5 mmol) and purified by flash column chromatography on silica gel (gradient elution: Petroleum ether : EtOAc = 10:1) to afford the product *E*-**92a** as a single isomer.

92a: 95.3 mg, 81% yield, colorless oil;

^{1}H NMR (400 MHz, $CDCl_3$) δ (ppm): 8.91 (brs, 1H), 7.62–7.57 (m, 2H), 7.41–7.36 (m, 3H), 3.65 (s, 3H), 2.84 (t, *J* = 7.6 Hz, 2H), 2.34 (t, *J* = 7.6 Hz, 2H), 1.72 (quint, *J* = 7.6 Hz, 2H), 1.66–1.53 (m, 2H); **^{13}C NMR** (101 MHz, $CDCl_3$) δ (ppm): 174.1, 159.5, 135.7, 129.4, 128.7, 126.4, 51.7, 33.9, 25.9, 25.8, 25.1; **HRMS** (ESI) *m/z* $[M+Na]^+$ calcd for $C_{13}H_{17}NNaO_3^+$: 258.1101, found 258.1100.

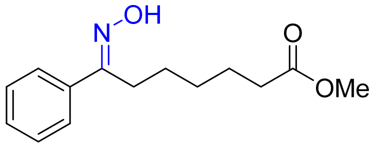

Methyl (*E*)-7-(hydroxyimino)-7-phenylheptanoate (93a) was synthesized by the general procedure **C** from methyl 7-phenylheptanoate (110.2 mg, 0.5 mmol) and purified by flash column chromatography on silica gel (gradient elution: Petroleum ether : EtOAc = 10:1) to afford the product *E*-**93a** as a single isomer.

93a: 103.5 mg, 83% yield, colorless oil;

^{1}H NMR (400 MHz, $CDCl_3$) δ (ppm): 9.27 (brs, 1H), 7.61–7.57 (m, 2H), 7.42–7.35 (m, 3H), 3.65 (s, 3H), 2.82 (t, *J* = 7.6 Hz, 2H), 2.30 (t, *J* = 7.6 Hz, 2H), 1.70–1.53 (m, 4H), 1.46–1.36 (m, 2H); **^{13}C NMR** (101 MHz, $CDCl_3$) δ (ppm): 174.3, 159.7, 135.8, 129.3, 128.7, 126.4, 51.6, 34.0, 29.3,

26.09, 26.05, 24.7; **HRMS** (ESI) *m/z* $[M+Na]^+$ calcd for $C_{14}H_{19}NNaO_3^+$: 272.1257, found 272.1256.

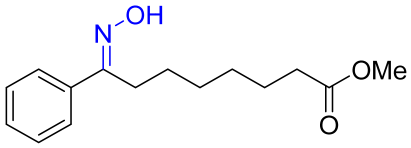

Methyl (*E*)-8-(hydroxyimino)-8-phenyloctanoate (94a) was synthesized by the general procedure **C** from methyl 8-phenyloctanoate (117.1 mg, 0.5 mmol) and purified by flash column chromatography on silica gel (gradient elution: Petroleum ether : EtOAc = 10:1) to afford the product *E*-**94a** as a single isomer.

94a: 105.3 mg, 80% yield, colorless oil;

^{1}**H NMR** (400 MHz, $CDCl_3$) δ (ppm): 8.98 (brs, 1H), 7.62–7.57 (m, 2H), 7.42–7.35 (m, 3H), 3.65 (s, 3H), 2.80 (t, J = 7.6 Hz, 2H), 2.29 (t, J = 7.6 Hz, 2H), 1.65–1.51 (m, 4H), 1.43–1.29 (m, 4H); ^{13}**C NMR** (101 MHz, $CDCl_3$) δ (ppm): 174.4, 159.8, 135.9, 129.3, 128.7, 126.4, 51.6, 34.1, 29.5, 28.9, 26.23, 26.20, 24.9; **HRMS** (ESI) *m/z* $[M+Na]^+$ calcd for $C_{15}H_{21}NNaO_3^+$: 286.1414, found 286.1414.

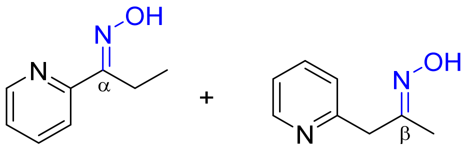

(*E*)-1-(Pyridin-2-yl)propan-1-one oxime (95a-α-oxime) and (*E*)-1-(Pyridin-2-yl)propan-2-one oxime (95a-β-oxime) (95a) was synthesized by the general procedure **A** or procedure **C** from 2-propylpyridine (121.2 mg, 1 mmol), and purified by flash column chromatography on silica gel (gradient elution: Petroleum ether : EtOAc = 10:1 to 3:1) to afford isolated product (*E*)**-95a-α-oxime** as a single isomer, and small amount of **95a-β-oxime**.

General procedure A, 95a: α, 24.1mg, 16% yield, white solid; β<5%,

General procedure C, 95a: α, 12.0 mg, 8% yield, white solid; β<5%;

95a-α-oxime:

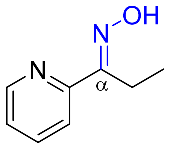

^{1}H NMR (400 MHz, $CDCl_3$) δ (ppm): 8. 94 (brs, 1H), 8.57 (d, *J* = 4.8 Hz, 1H), 7.74 (d, *J* = 8.0 Hz, 1H), 7.62 (td, *J* = 7.6, 1.6 Hz, 1H), 7.22–7.18 (m, 1H), 2.93 (q, *J* = 7.6 Hz, 2H), 1.12 (t, *J* = 7.6 Hz, 3H); **^{13}C NMR** (101 MHz, $CDCl_3$) δ (ppm): 161.7, 153.8 (C=N), 149.2, 136.5, 123.8, 121.1, 18.3, 10.9; **HRMS** (ESI+): *m/z* $[M+Na]^+$ calculated for $C_8H_{10}N_2NaO^+$: 173.0685, found 173.0689.

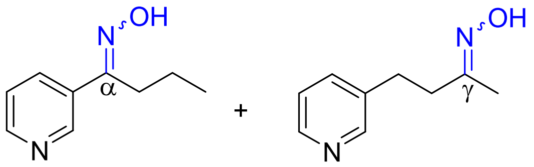

(*E/Z*)-(pyridin-3-yl)butan-1-one oxime (96-α-oxime) and **(*E*/*Z*)-4-(pyridin-3-yl)butan-2-one oxime (96a-γ-oxime) (96a)** was synthesized by the general procedure **A** or procedure **C** from 3-butylpyridine (135.2 mg, 1 mmol) and purified by flash column chromatography on silica gel (gradient elution: Petroleum ether : EtOAc = 10:1 to 3:1) to afford two separated regioisomers (*E*/*Z*)-**96-α-oxime** as an *E/Z* mixture and **(*E*/*Z*)-96a-γ-oxime** as an *E/Z* mixture.

The E/Z isomers were confirmed by *^{1}H and ^{13}C NMR, and the ratio of E/Z* was *calculated by integration of ^{1}H and ^{13}C NMR spectra*;

General procedure A, 96a: α, 13.1 mg, 8% yield, *E/Z* = 4:1, white solid; γ, 34.5 mg, 21% yield, *E/Z* = 2.4:1;

General procedure C, 96a: α, 50.9 mg, 31% yield, *E/Z* = 4:1, white solid; γ, 11.5 mg, 7% yield, *E/Z* = 2.4:1;

(*E*/*Z*)-**96-α-oxime**:

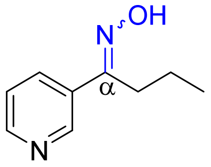

Major *E*-isomer: ^{1}H NMR (400 MHz, $CDCl_3$) δ (ppm): 10.17 (brs, 1H), 8.91 (d, *J* = 1.6 Hz, 1H), 8.62–8.57 (m, 1H), 7.96–7.92 (m, 1H), 7.34–7.29 (m, 1H), 2.80 (t, *J* = 7.6 Hz, 2H), 1.61 (sext, *J* = 7.6Hz, 2H), 0.99 (t, *J* = 7.6 Hz, 3H). **Minor *Z*-isomer: ^{1}H NMR** (400 MHz, $CDCl_3$) δ (ppm): 10.17 (brs, 1H), 8.74 (d, *J* = 1.6 Hz, 1H), 8.62–8.57 (m, 1H), 7.85–7.81 (m, 1H), 7.39–7.34 (m, 1H), 2.56 (t, *J* = 7.6 Hz, 2H), 1.49 (sext, *J* = 7.6 Hz, 2H), 0.93 (t, *J* = 7.6 Hz, 3H).

^{13}C NMR (101 MHz, $CDCl_3$) δ (ppm): 157.1, 149.7, 149.6, 148.9, 147.7, 136.1, 133.9, 132.2, 123.5, 123.3, 37.0, 29.8, 27.7, 19.8, 14.4, 13.7.

HRMS (ESI+): *m/z* $[M+H]^+$ calculated for $C_9H_{13}N_2O^+$: 165.1022, found 165.1026.

(*E*/*Z*)-96a-γ-oxime:

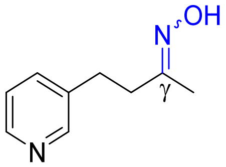

Major *E*-isomer: ^{1}H NMR (400 MHz, $CDCl_3$) δ (ppm): 9.64 (brs, 1H), 8.50–8.42 (m, 2H), 7.59–7.49 (m, 1H), 7.24–7.19 (m, 1H), 2.87–2.82 (m, 2H), 2.53–2.48 (m, 2H), 1.91 (s, 3H); **^{13}C NMR** (101 MHz, $CDCl_3$) δ (ppm): 156.7, 149.8, 147.4, 136.8, 136.2, 123.6, 37.3, 29.7, 13.9.

Minor *Z*-isomer: ^{1}H NMR (400 MHz, $CDCl_3$) δ (ppm): 9.64 (brs, 1H), 8.50–8.42 (m, 2H), 7.59–7.49 (m, 1H), 7.24–7.19 (m, 1H), 2.87–2.82 (m, 2H), 2.69–2.64 (m, 2H), 1.82 (s, 3H); **^{13}C NMR** (101 MHz, $CDCl_3$) δ (ppm): 157.1, 149.7, 147.5, 136.8, 136.2, 123.6, 30.3, 28.8, 20.4.

HRMS (ESI+): *m/z* $[M+H]^+$ calculated for $C_9H_{13}N_2O^+$: 165.1022, found 165.1025.

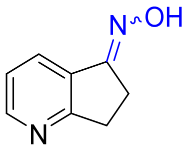

(*E*/*Z*)-6,7-dihydro-5*H*-cyclopenta[*b*]pyridin-5-one oxime (97a) was synthesized by the general procedure **A** from 6,7-dihydro-5*H*-cyclopenta[*b*]pyridine (119.2 mg, 1 mmol) and purified by flash column chromatography on silica gel (gradient elution: Petroleum ether : EtOAc = 10:1 to 2:1) to afford the product (*E/Z*)-**97a** as a mixture of *E/Z* isomers.

The E/Z isomers were confirmed by ^{1}H, ^{13}C NMR, and the *ratio of E/Z was calculated by integration of ^{1}H, ^{13}C NMR spectra.*

97a: 88.9 mg, 60% yield, *E/Z* = 7:1, white solid; **rsm**: 22.6 mg, 19%;

Major *E*-isomer: ^{1}H NMR (400 MHz, DMSO-*d6*) δ (ppm): 11.15 (brs, 1H), 8.50–8.47 (m, 2H), 7.89–7.84 (m, 1H), 7.27–7.21 (m, 1H), 3.10–3.01 (m, 2H), 2.83–2.74 (m, 2H); **^{13}C NMR** (101 MHz, DMSO-*d6*) δ (ppm): 167.3, 158.5, 150.6, 129.9, 128.2, 121.9, 29.8, 23.9.

Minor *Z*-isomer: **^{1}H NMR** (400 MHz, DMSO-*d6*) δ (ppm): 11.22 (brs, 1H), 8.55−8.51 (m, 2H), 7.89−7.84 (m, 1H), 7.27−7.21 (m, 1H), 3.10−3.01 (m, 2H), 2.83−2.74 (m, 2H); **^{13}C NMR** (101 MHz, DMSO-*d6*) δ (ppm): 168.0, 155.4, 150.8, 135.7, 127.0, 121.6, 30.2, 25.8.
HRMS (ESI+): *m/z* $[M+Na]^+$ calculated for $C_8H_8N_2NaO^+$: 171.0529, found 171.0531.

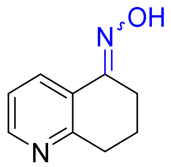

(*E*/Z)-7,8-dihydroquinolin-5(6*H*)-one oxime (98a) was synthesized by the general procedure **A** from 5,6,7,8-tetrahydroquinoline (133.2 mg, 1 mmol) and purified by flash column chromatography on silica gel (gradient elution: Petroleum ether : EtOAc = 10:1 to 2:1) to afford the product (*E/Z*)-**98a** as a mixture of *E/Z* isomers.
The E/Z isomers were confirmed by ^{1}H, ^{13}C NMR, and the *ratio of E/Z was calculated by integration of ^{1}H, ^{13}C NMR spectra.*
98a: 110.3 mg, 68% yield, *E/Z = 14:1*, white solid; **rsm**: 21.3 mg, 16% yield;
Major *E*-isomer: **^{1}H NMR** (400 MHz, DMSO-*d6*) δ (ppm): 11.37 (s, 1H), 8.41 (d, *J* = 4.0 Hz, 1H), 8.14 (d, *J* = 8.0 Hz, 1H), 7.21 (dd, *J* = 8.0, 4.4 Hz, 1H), 2.84 (t, *J* = 6.2 Hz, 2H), 2.67 (t, *J* = 6.4 Hz, 2H), 1.81 (quint, *J* = 6.4 Hz, 2H); **^{13}C NMR** (101 MHz, DMSO-*d6*) δ (ppm): 157.6, 151.4, 148.8, 130.6, 126.5, 121.6, 31.8, 22.6, 20.1; **HRMS** (ESI+): *m/z* $[M+H]^+$ calculated for $C_9H_{11}N_2O^+$: 163.0866, found 163.0868.

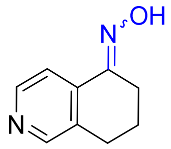

(*E*/*Z*)-7,8-dihydroisoquinolin-5(6*H*)-one oxime (99a) was synthesized by the general procedure **A** from 5,6,7,8-tetrahydroisoquinoline (133.2 mg, 1 mmol) and purified by flash column chromatography on silica gel (gradient elution: Petroleum ether : EtOAc = 10:1 to 2:1) to afford the product (*E/Z*)-**99a** as a mixture of *E/Z* isomers.
The E/Z isomers were confirmed by ^{1}H, ^{13}C NMR, and the *ratio of E/Z was calculated by integration of ^{1}H, ^{13}C NMR spectra.*
99a: 64.9 mg, 40% yield, *E/Z = 7:3*, white solid; **rsm**: 38.6 mg, 29%;

Major *E*-isomer: ^{1}H NMR (400 MHz, DMSO-*d6*) δ (ppm): 11.39 (s, 1H), 8.96 (s, 1H), 8.33 (d, *J* =5.2 Hz, 1H), 7.16 (d, *J* =4.8 Hz, 1H), 2.71–2.63 (m, 4H), 1.78–1.69 (m, 2H); **^{13}C NMR** (101 MHz, DMSO-*d6*) δ (ppm): 150.7, 148.1, 146.8, 144.5, 127.1, 123.2, 28.0, 22.8, 20.0.

Minor *Z*-isomer: ^{1}H NMR (400 MHz, DMSO-*d6*) δ (ppm): 11.67 (s, 1H), 8.41 (s, 1H), 8.33 (d, *J* =5.2 Hz, 1H), 7.67 (d, *J* =5.2 Hz, 1H), 2.71–2.63 (m, 4H), 1.78–1.69 (m, 2H); **^{13}C NMR** (101 MHz, DMSO-*d6*) δ (ppm): 150.9, 149.9, 146.8, 138.0, 133.3, 116.5, 25.5, 22.8, 20.3.

HRMS (ESI+): *m/z* $[M+Na]^+$ calculated for $C_9H_{10}N_2NaO^+$: 185.0685, found 185.0689.

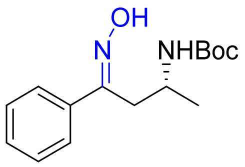

***tert*-Butyl (*R*,*E*)-(4-(hydroxyimino)-4-phenylbutan-2-yl)carbamate (100a)** was synthesized by the general procedure **C** from *tert*-butyl (*R*)-(4-phenylbutan-2-yl)carbamate (124.7 mg, 0.5 mmol) and purified by flash column chromatography on silica gel (gradient elution: Petroleum ether : EtOAc = 10:1 to 4:1) to afford the product (*R*,*E)*-**100a** as a single isomer.

100a: 116.9 mg, 84% yield, white solid;

^{1}H NMR (400 MHz, $CDCl_3$) δ (ppm): 9.61 (brs, 1H), 7.65–7.61 (m, 2H), 7.40–7.35 (m, 3H), 4.87 (brs, 1H), 4.02–3.95 (m, 1H), 3.20–3.10 (m, 1H), 2.96–2.85 (m, 1H), 1.39 (s, 9H), 1.16 (d, *J* = 6.4 Hz, 3H); **^{13}C NMR** (101 MHz, $CDCl_3$) δ (ppm): 157.1, 155.4, 135.7, 129.4, 128.7, 126.5, 79.2, 45.3, 32.8, 28.5, 21.3; **HRMS** (ESI) *m/z* $[M+Na]^+$ calcd for $C_{15}H_{22}N_2NaO_3^+$: 301.1523, found 301.1521.

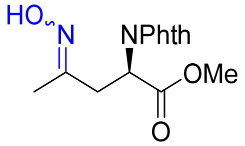

Methyl (*R*,*E/Z*)-2-(1,3-dioxoisoindolin-2-yl)-4-(hydroxyimino)pentanoate (101a) was synthesized by the general procedure **A** from methyl (*R*)-2-(1,3-dioxoisoindolin-2-yl)pentanoate (261.3 mg, 1 mmol) and purified by flash column chromatography on silica gel (gradient elution: Petroleum ether : EtOAc = 10:1 to 3:1) to afford the product (*R*,*E*/*Z*)-**101a** as a mixture of *E/Z* isomers.

The E/Z isomers were confirmed by ^{1}H and ^{13}C NMR and the *ratio of E/Z was calculated by integration of ^{1}H and ^{13}C NMR spectra.*

101a: 31.9 mg, 11% yield, *E/Z = 11:1*, white solid; **rsm**: 230.0 mg, 88%;

Major *E*-isomer: ^{1}H NMR (400 MHz, $CDCl_3$) δ (ppm): 7.89–7.81 (m, 2H), 7.74–7.70 (m, 2H), 5.21 (dd, *J* =10.8, 4.4 Hz, 1H), 3.74 (s, 3H), 3.20–2.98 (s, 2H), 1.87 (s, 3H); **^{13}C NMR** (101 MHz, $CDCl_3$) δ (ppm): 169.3, 167.6, 154.4, 134.3, 131.9, 123.8, 53.2, 49.1, 35.0, 13.8.

Minor *Z*-isomer: ^{1}H NMR (400 MHz, $CDCl_3$) δ (ppm): 7.89–7.81 (m, 2H), 7.74–7.70 (m, 2H), 5.32 (dd, *J* =10.8, 4.8 Hz, 1H), 3.75 (s, 3H), 3.20–2.98 (s, 2H), 1.87 (s, 3H).

HRMS (ESI+): *m/z* $[M+Na]^+$ calculated for $C_{14}H_{14}N_2NaO_5^+$: 313.0795, found 313.0800.

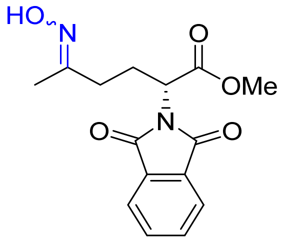

Methyl (*R,E/Z*)-2-(1,3-dioxoisoindolin-2-yl)-5-(hydroxyimino)hexanoate (102a) was synthesized by the general procedure **A** from methyl (*R*)-2-(1,3-dioxoisoindolin-2-yl)hexanoate (275.3 mg, 1 mmol) and purified by flash column chromatography on silica gel (gradient elution: Petroleum ether : EtOAc = 10:1 to 3:1) to afford the products (*R*,*E/Z*)-**102a** as a mixture of *E/Z* isomers.

The E/Z isomers were confirmed by ^{1}H and ^{13}C NMR and the *ratio of E/Z was calculated by integration of ^{1}H and ^{13}C NMR spectra.*

102a: 94.3 mg, 31% yield, *E/Z = 3:1*, white solid; **rsm**: 187.2 mg, 68%;

Major *E*-isomer: ^{1}H NMR (400 MHz, $CDCl_3$) δ (ppm): 8.61 (brs, 1H), 7.86–7.80 (m, 2H), 7.74–7.69 (m, 2H), 4.85 (dd, *J* =10.0, 5.2 Hz, 1H), 3.71 (s, 3H), 2.48–2.38 (m, 2H), 2.27–2.13 (m, 2H), 1.79 (s, 3H); **^{13}C NMR** (101 MHz, $CDCl_3$) δ (ppm): 169.6, 167.8, 156.6, 134.3, 131.8, 123.7, 52.9, 51.6, 32.6, 25.4, 13.6.

Minor *Z*-isomer: ^{1}H NMR (400 MHz, $CDCl_3$) δ (ppm): 8.61 (brs, 1H), 7.86–7.80 (m, 2H), 7.74–7.69 (m, 2H), 4.85 (dd, *J* =10.0, 5.2 Hz, 1H), 3.71 (s, 3H), 2.48–2.38 (m, 2H), 2.27–2.13 (m, 2H), 1.80 (s, 3H); **^{13}C NMR** (101 MHz, $CDCl_3$) δ (ppm): 169.6, 167.8, 156.6, 134.3, 131.8, 123.7, 52.9, 52.1, 25.5, 24.7, 19.8.

HRMS (ESI+): *m/z* $[M+Na]^+$ calculated for $C_{15}H_{16}N_2NaO_5^+$: 327.0951, found 327.0951.

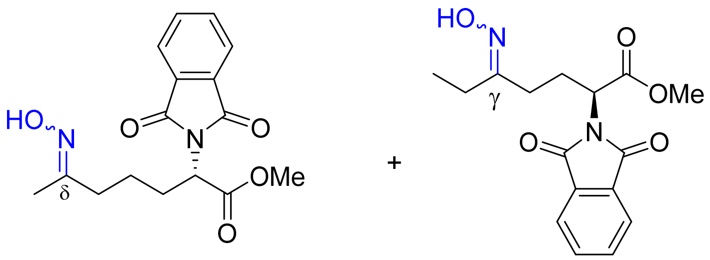

Methyl (*S,E*/*Z*)-2-(1,3-dioxoisoindolin-2-yl)-6-(hydroxyimino)heptanoate (103-δ-oxime) and **methyl (*S,E*/*Z*)-2-(1,3-dioxoisoindolin-2-yl)-5-(hydroxyimino)heptanoate (103-γ-oxime) (103a)** was synthesized by the general procedure **A** from methyl (*S*)-2-(1,3-dioxoisoindolin-2-yl)heptanoate (289.3 mg, 1 mmol) and purified by flash column chromatography on silica gel (gradient elution: Petroleum ether : EtOAc = 10:1 to 3:1) to afford a mixture of two regioisomers (*S*,*E*/*Z*)-**103a-δ-oxime** and (*S*,*E*/*Z*)**-103a-γ-oxime** as white solid**.**

Site of oximation was assigned based on analysis of the ^1H and ^{13}C NMR spectra of the mixture products. The E/Z isomers were confirmed by ^1H and ^{13}C NMR and the *ratio of E/Z isomers was calculated by integration of ^1H and ^{13}C NMR spectra.*

103a: 127.3 mg, 40% yield, γ:δ = 3:7, γ, *E/Z = 1:1;* δ, *E/Z = 10:3*, white solid; **rsm**: 167.2 mg, 58%.

^{1}H NMR (400 MHz, $CDCl_3$) δ (ppm): 8.86 (brs, 1H), 7.86–7.80(m, 2H), 7.74–7.68 (m, 2H), 4.91–4.79 (m, 1H), 3.69 (s, 3H), 2.46–2.12 (m, 4H), 1.77 and 1.76 (s, 1.83H, **103a-δ**, *E/Z*=10:3), 1.55–1.42 (m, 1.66H), 1.26–1.16 (m, 0.49H), 1.02–0.95 (0.79H).

^{13}C NMR (101 MHz, $CDCl_3$) δ (ppm): 169.8, 169.7, 169.6, 169.5, 167.74, 167.72, 160.81 (**103a-γ**), 160.79 (**103a-γ**), 157.9 (**103a-δ**, *Z*-isomer), 157.6 (**103a-δ**, *E*-isomer), 134.30, 134.28, 131.82, 131.78, 123.65, 123.62, 52.85, 52.83, 52.79, 52.17, 51.82, 51.79, 51.65, 35.0, 30.4, 28.6, 28.3, 27.8, 27.4, 25.3, 24.9, 24.5, 23.0, 22.3, 20.9, 19.8, 13.4, 10.7, 10.1.

HRMS (ESI+): *m/z* $[M+Na]^+$ calculated for $C_{16}H_{18}N_2NaO_5^+$: 341.1108, found 341.1111.

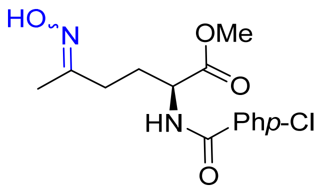

Methyl (*S,E*/*Z*)-2-(4-chlorobenzamido)-5-(hydroxyimino)hexanoate (104a) was synthesized by the general procedure **A** from methyl (*S*)-2-(4-chlorobenzamido) hexanoate (283.8 mg, 1 mmol) and purified by flash column chromatography on silica gel (gradient elution: Petroleum ether : EtOAc = 8:1 to 3:1) to afford the product (*S,E/Z*)-**104a** as a mixture of *E/Z* isomers.

The E/Z isomers were confirmed by ^{1}H and ^{13}C NMR and the *ratio of E/Z was calculated by integration of ^{1}H and ^{13}C NMR spectra.*

104a: 122.0 mg, 39% yield, *E/Z = 3:1*, white solid; **rsm:**133.4 mg, 47%;

Major *E*-isomer: ^{1}H NMR (400 MHz, $CDCl_3$) δ (ppm): 9.38 (brs, 1H), 7.72–7.68 (m, 2H), 7.48 (brs, 1H), 7.34–7.27 (m, 2H), 4.77–4.69 (m, 1H), 3.71 (s, 3H), 2.48–2.24 (m, 2H), 2.18–1.97 (m, 2H), 1.81 (s, 3H); **^{13}C NMR** (101 MHz, $CDCl_3$) δ (ppm): 172.9, 166.6, 157.5, 138.0, 132.0, 128.79, 128.75, 52.7, 52.6, 32.0, 28.1, 14.0.

Minor *Z*-isomer: ^{1}H NMR (400 MHz, $CDCl_3$) δ (ppm): 9.38 (brs, 1H), 7.72–7.68 (m, 2H), 7.34–7.27 (m, 2H), 6.66 (brs, 1H), 4.82–4.77 (m, 1H), 3.71 (s, 3H), 2.48–2.24 (m, 2H), 2.18–1.97 (m, 2H), 1.80 (s, 3H); **^{13}C NMR** (101 MHz, $CDCl_3$) δ (ppm):172.6, 169.2, 157.8, 138.3, 132.2, 131.6, 129.0, 52.7, 52.4, 27.4, 24.7, 19.8.

HRMS (ESI+): *m/z* $[M+Na]^+$ calculated for $C_{14}H_{17}ClN_2NaO_4^+$: 335.0769, found 335.0765.

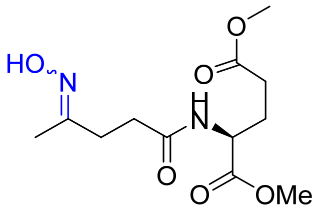

Dimethyl (*E*/*Z*)-(4-(hydroxyimino)pentanoyl)-*L*-aspartate (105a) was synthesized by the general procedure **A** from dimethyl pentanoyl-*L*-aspartate (259.3 mg, 1mmol) and purified by flash column chromatography on silica gel (gradient elution: Petroleum ether : EtOAc = 4:1 to 2:1) to afford the product (*E/Z*)-**105a** as a mixture of *E/Z* isomers.

The E/Z isomers were confirmed by ^{1}H and ^{13}C NMR and the *ratio of E/Z was calculated by integration of ^{1}H and ^{13}C NMR spectra.*

105a: 92.3 mg, 32% yield, *E/Z = 7:3*, colorless oil; **rsm**:155.6 mg, 60%

^{1}H NMR (400 MHz, $CDCl_3$) δ (ppm): 8.94 (brs, 1H), 6.86 (brs, 1H), 4.64–4.55 (m, 1H), 3.71–3.68 (m, 3H), 3.65–3.62 (m, 3H), 2.65–2.33 (m, 6H), 2.22–2.09 (m, 1H), 1.99–1.89 (m, 1H), 1.86–1.82 (m, 3H);

^{13}C NMR (101 MHz, $CDCl_3$) δ (ppm): 173.7, 173.5, 172.7, 172.6, 172.5, 172.0, 157.8, 157.0, 52.6, 52.0, 51.7, 51.5, 32.2, 31.9, 31.4, 30.1, 27.4, 27.2, 24.7, 20.1, 14.2.

HRMS (ESI+): *m/z* $[M+H]^+$ calculated for $C_{12}H_{21}N_2O_6^+$: 289.1394, found 289.1405.

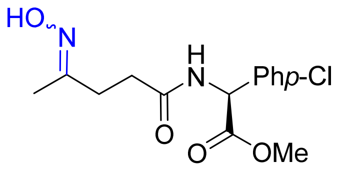

Methyl (*S*,*E*/*Z*)-2-(4-chlorophenyl)-2-(4-(hydroxyimino)pentanamido)acetate (106a) was synthesized by the general procedure **A** from methyl (*S*)-2-(4-chlorophenyl)-2-pentanamidoacetate (283.8 mg, 1 mmol) and purified by flash column chromatography on silica gel (gradient elution: Petroleum ether : EtOAc = 8:1 to 3:1) to afford the product (*S*,*E*/*Z*)-**106a** as a mixture of *E*/*Z* isomers.

The E/Z isomers were confirmed by ^{1}H and ^{13}C NMR and the *ratio of E/Z was calculated by integration of ^{1}H and ^{13}C NMR spectra.*

106a: 131.4 mg, 42% yield, *E/Z = 11:3*, white solid; **rsm**:141.9 mg, 50%;

Major *E*-isomer: ^{1}H NMR (400 MHz, $CDCl_3$) δ (ppm): 7.25–7.21 (m, 4H), 5.52 (d, *J* =7.2 Hz, 1H), 3.65 (s, 3H), 2.48–2.39 (m, 4H), 1.80 (s, 3H). **^{13}C NMR** (101 MHz, $CDCl_3$) δ (ppm): 171.9, 171.4, 157.1, 135.1, 134.4, 129.1, 128.8, 55.8, 53.0, 32.0, 31.3, 14.2.

Minor *Z*-isomer: ^{1}H NMR (400 MHz, $CDCl_3$) δ (ppm): 7.29–7.25 (m, 4H), 5.52 (d, *J* =7.2 Hz, 1H), 3.65 (s, 3H), 2.64–2.39 (m, 4H), 1.76 (s, 3H). **^{13}C NMR** (101 MHz, $CDCl_3$) δ (ppm): 171.5, 171.3, 157.7, 135.0, 134.5, 129.1, 128.8, 55.9, 53.0, 31.7, 24.6, 20.1.

HRMS (ESI+): *m/z* $[M+Na]^+$ calculated for $C_{14}H_{17}ClN_2NaO_4^+$: 335.0769, found 335.0769.

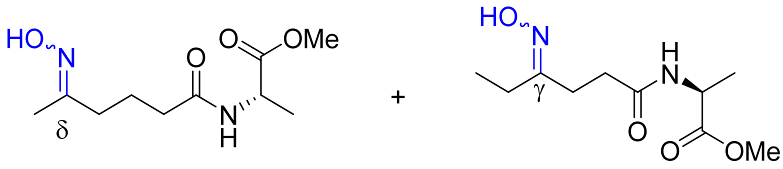

Methyl (*E*/*Z*)-(5-(hydroxyimino)hexanoyl)-*L*-alaninate (107a-δ-oxime) and **methyl (*E*/*Z*)-(4-(hydroxyimino)hexanoyl)-*L*-alaninate (107a-γ-oxime) (107a)** was synthesized by the general procedure **A** from methyl hexanoyl-*L*-alaninate (201.3 mg, 1 mmol) and purified by flash column chromatography on silica gel (gradient elution: Petroleum ether : EtOAc = 8:1 to 3:1) to afford a mixture of two regioisomers (*E*/*Z*)-**107a-δ-oxime** and **(*E*/*Z*)-107a-γ-oxime** as colorless oil**.**

Site of oximation was assigned based on analysis of the ^{1}H and ^{13}C NMR ofthe mixture products. The E/Z isomers were confirmed by ^{1}H and ^{13}C NMR and the *ratio of E/Z was calculated by integration of ^{1}H and ^{13}C NMR spectra.*

107a: 122.1 mg, 53% yield, γ:δ = 1:2, *γ, E/Z = 1:1; δ, E/Z = 3:1*, colorless oil; **rsm**: 85.6 mg, 43%;

^{1}H NMR (400 MHz, $CDCl_3$) δ (ppm): 9.14 (brs, 1H), 6.75–6.56 (m, 0.9H), 6.20–5.78 (m, 0.3H), 4.67–4.45 (m, 1H), 3.78–3.67 (m, 3H), 2.67–2.28 (m, 2.57H), 2.25–2.15 (m, 2.5H), 1.86–1.76 (m, 2.57H), 1.65–1.55 (m, 0.47H), 1.38–1.32 (m, 2.96H), 1.30–1.21 (m, 2.15H), 1.08–1.01 (m, 1.08H), 0.90–0.78 (m, 0.99H).

^{13}C NMR (101 MHz, $CDCl_3$) δ (ppm): 174.0, 173.9, 173.8, 172.5, 172.4, 172.21, 172.19, 171.82, 161.4, 161.3, 158.1, 157.7, 52.5, 48.1, 48.0, 35.9, 35.7, 35.3, 35.0, 32.2, 32.1, 31.48, 31.45, 30.3, 30.2, 29.7, 29.2, 27.9, 27.8, 25.3, 23.7, 22.4, 22.0, 21.54, 21.52, 19.7, 18.3, 14.0, 13.5, 10.8, 10.1.

HRMS (ESI+): *m/z* $[M+Na]^+$ calculated for $C_{10}H_{18}NaN_2O_4^+$: 253.1159, found 253.1166.

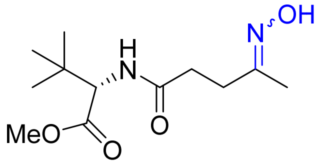

Methyl (*S,E/Z*)-2-(4-(hydroxyimino)pentanamido)-3,3-dimethylbutanoate (108a) was synthesized by the general procedure **A** from methyl (*S*)-3,3-dimethyl-2-pentanamidobutanoate (229.3 mg, 1mmol) and purified by flash column chromatography on silica gel (gradient elution: Petroleum ether : EtOAc = 15:1 to 3:1) to afford the product (*S*,*E*/*Z*)-**108a** as a mixture of *E/Z* isomers.

The E/Z isomers were confirmed by ^{1}H and ^{13}C NMR and the *ratio of E/Z was calculated by integration of ^{1}H and ^{13}C NMR spectra.*

108a: 77.5 mg, 30% yield, *E/Z = 3:1,* white solid; **rsm**:144.5 mg, 63%;

Major *E*-isomer: ^{1}H NMR (400 MHz, $CDCl_3$) δ (ppm): 9.07 (brs, 1H), 6.77 (d, *J* =5.2 Hz, 1H), 4.46 (d, *J* =5.6 Hz, 1H), 3.68 (s, 3H), 2.66–2.42 (m, 4H), 1.85 (s, 3H), 0.92 (s, 9H); **^{13}C NMR** (101 MHz, $CDCl_3$) δ (ppm): 172.8, 172.3, 157.0, 60.0, 51.94, 34.7, 32.5, 31.8, 26.6, 14.2.

Minor *Z*-isomer: ^{1}H NMR (400 MHz, $CDCl_3$) δ (ppm): 9.07 (brs, 1H), 6.53 (d, *J* =4.4 Hz, 1H), 4.46 (d, *J* =5.6 Hz, 1H), 3.68 (s, 3H), 2.66–2.42 (m, 4H), 1.85 (s, 3H), 0.92 (s, 9H); **^{13}C NMR** (101 MHz, $CDCl_3$) δ (ppm): 172.5, 171.8, 157.6, 60.1, 51.89, 34.4, 32.1, 26.6, 24.7, 20.1.

HRMS (ESI+): *m/z* $[M+H]^+$ calculated for $C_{12}H_{23}N_2O_4^+$: 259.1652, found 259.1652.

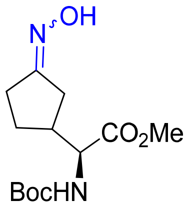

Methyl (2*S*)-2-((*tert*-butoxycarbonyl)amino)-2-(*E*/*Z*)-3-(hydroxyimino)cyclopentyl) acetate (109a) was synthesized by the general procedure **A** from methyl (*S*)-2-((*tert*-butoxycarbonyl)amino)-2-cyclopentylacetate (257.3 mg, 1 mmol) and purified by flash column chromatography on silica gel (gradient elution: Petroleum ether : EtOAc = 20:1 to 5:1) to afford the product (*S*, *E/Z*)-**109a** as a mixture of *E/Z* isomers.

The E/Z isomers were confirmed by ^{1}H and ^{13}C NMR and the *ratio of E/Z was calculated by integration of ^{1}H and ^{13}C NMR spectra.*

109a: 180.4 mg, 63% yield; colorless oil, *E/Z = 1.6:1*;

^{1}H NMR (400 MHz, $CDCl_3$) δ (ppm): 8.99 (brs, 1H), 5.33–5.20 (m, 1H), 4.40–4.30 (m, 1H), 3.72 (s, 3H), 2.73–2.55 (m, 1H), 2.52–2.15 (m, 4H), 1.94–1.80 (m, 1H), 1.59–1.47 (m, 1H), 1.41 (s, 9H).

^{13}C NMR (101 MHz, $CDCl_3$) δ (ppm): 172.7, 164.9, 164.7, 164.5, 155.7, 155.6, 80.3, 55.8, 55.6, 52.5, 41.8, 41.3, 33.8, 33.0, 30.2, 30.0, 29.8, 29.3, 28.4, 27.9, 27.3, 27.2, 26.8, 26.5.

HRMS (ESI) *m/z* $[M+Na]^+$ calculated for $C_{13}H_{22}N_2NaO_5^+$: 309.1421, found 309.1416;

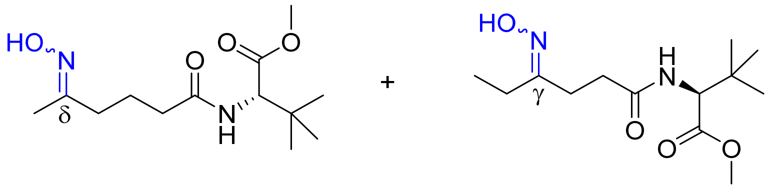

Methyl (*S*,*E*/*Z*)-2-(5-(hydroxyimino)hexanamido)-3,3-dimethylbutanoate (110a-δ-oxime) and methyl (*S*,*E*/*Z*)-2-(4-(hydroxyimino)hexanamido)-3,3-dimethylbutanoate (110a-γ-oxime) (110a) was synthesized by the general procedure **A** from methyl (*S*)-2-hexanamido-3,3-dimethylbutanoate (243.3 mg, 1 mmol) and purified by flash column chromatography on silica gel (gradient elution: Petroleum ether : EtOAc = 8:1 to 3:1) to afford a mixture of two regioisomers (*E*/*Z*)-**110a-δ-oxime** and **(*E*/*Z*)-110a-γ-oxime** as white solid**.**

Site of oximation was assigned based on analysis of the ^{1}H and ^{13}C NMR of the mixture products. The E/Z isomers were confirmed by ^{1}H and ^{13}C NMR and the *ratio of E/Z was calculated by integration of ^{1}H and ^{13}C NMR spectra.*

110a: 138.9 mg, 51% yield, γ:δ = 1:3, *γ, E/Z* = 1:1*; δ, E/Z* = 10:3, white solid; **rsm**:111.9 mg, 46%;

^{1}H NMR (400 MHz, $CDCl_3$) δ (ppm): 9.07 (brs, 0.6H), 6.75–6.45 (m, 0.87H), 4.46 (d, *J* = 9.6 Hz, 1H), 3.68 (s, 3.06H), 2.62–2.28 (m, 2.03H), 2.26–2.17 (m, 2.34H), 1.89–1.78 (m, 3.13H), 1.34–1.14 (m, 1.31H), 1.03 (t, *J* =7.6 Hz, 0.92H), 0.93 (s. 9.15H).

^{13}C NMR (101 MHz, $CDCl_3$) δ (ppm): 172.71, 172.67, 172.61, 172.56, 172.50, 172.45, 161.4 (**110a-γ**), 161.3 (**110a-γ**), 158.0 ((*Z*)-**110a-δ**), 157.7 ((*E*)-**110a-δ**), 60.06 (**110a-γ**), 60.04 (**110a-γ**), 60.00 ((*Z*)-**110a-δ**), 59.97 ((*E*)-**110a-δ**), 51.89, 51.87, 35.9, 35.6, 35.1, 34.7, 32.3, 29.4, 27.9, 27.8, 26.64, 26.61, 23.7, 22.1, 21.62, 21.57, 19.7, 13.5, 10.8, 10.0.

HRMS (ESI+): *m/z* $[M+H]^+$ calculated for $C_{13}H_{25}N_2O_4^+$: 273.1809, found 273.1810.

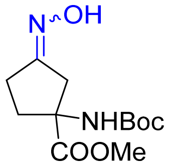

Methyl(*E*/*Z*)-1-((*tert*-butoxycarbonyl)amino)-3-(hydroxyimino)cyclo-pentane-1-carboxylate (111a) was synthesized by the general procedure **A** from methyl 1-((*tert*-butoxycarbonyl)amino) cyclopentane-1-carboxylate (243.3 mg, 1 mmol) and purified by flash column chromatography on silica gel (gradient elution: Petroleum ether : EtOAc = 20:1 to 5:1) to afford the product (*E/Z*)-**111a** as a mixture of *E/Z* isomers.

The E/Z isomers were confirmed by ^{1}H and ^{13}C NMR and the *ratio of E/Z was calculated by integration of ^{1}H and ^{13}C NMR spectra.*

111a: 191.0 mg, 70% yield, *E/Z* = 3:1, colorless oil;

^{1}H NMR (400 MHz, $CDCl_3$) δ (ppm): 8.85 (brs, 1H), 5.22 (brs, 1H), 3.73 (s, 3H), 3.14–2.97 (m, 1H), 2.93–2.54 (m, 3H), 2.31–2.14 (m, 2H), 1.41 (s, 9H).

^{13}C NMR (101 MHz, $CDCl_3$) δ (ppm): 173.8 (*Z*), 173.5 (*E*), 162.9 (*E*), 162.4 (*Z*), 155.4 (2C), 80.6, 64.3 (*Z*), 64.2 (*E*), 52.8, 41.4, 34.0, 29.8, 28.41, 28.36, 28.3, 25.1, 24.6.

HRMS (ESI) *m/z* $[M+Na]^+$ calculated for $C_{12}H_{20}N_2NaO_5^+$: 295.1264, found 295.1265.

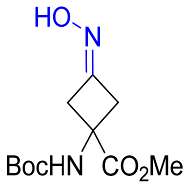

Methyl 1-((*tert*-butoxycarbonyl)amino)-3-(hydroxyimino)cyclobutane-1-carboxylate (112a) was synthesized by the general procedure **A** from methyl 1-((*tert*-butoxycarbonyl)amino) cyclobutane-1-carboxylate (229.3 mg, 1 mmol) and purified by flash column chromatography on silica gel (gradient elution: Petroleum ether : EtOAc = 20:1 to 4:1) to afford the product **112a**, and remaining staring material **112.** The collected **112** was reused for oxidative oximation twice, with the amount of other reagents required based on the amount of **112** used.

112a: 83.7 mg, 32% yield, white solid; **rsm**: 130.7 mg, 57%;

^{1}H NMR (400 MHz, $CDCl_3$) δ (ppm): 8.32 (brs, 1H), 5.50 (brs, 1H), 3.78 (s, 3H), 2.53–2.44 (m, 2H), 3.34–2.94 (m, 2H), 1.43 (s, 9H).

^{13}C NMR (101 MHz, $CDCl_3$) δ (ppm): 173.3, 155.1, 152.1, 80.8, 53.3, 53.1, 42.6, 41.8, 28.4.

HRMS (ESI) *m/z* $[M+Na]^+$ calculated for $C_{11}H_{18}N_2NaO_5^+$: 281.1108, found 281.1110;

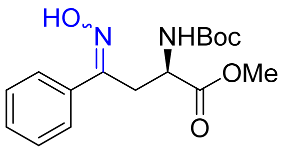

Methyl (*R*,*E*/*Z*)-2-((*tert*-butoxycarbonyl)amino)-4-(hydroxyimino)-4-phenylbutanoate (113a) was synthesized by the general procedure **C** from methyl (*R*)-2-((*tert*-butoxycarbonyl)amino)-4-phenylbutanoate (146.7 mg, 0.5 mmol) and purified by flash column chromatography on silica gel (gradient elution: Petroleum ether : EtOAc = 10:1 to 4:1) to afford the product (*R*, *E/Z*)-**113a** as a mixture of *E/Z* isomers.

113a: 128.9 mg, 80% yield, *E/Z* = 11:1; white solid;

^{1}H NMR (400 MHz, $CDCl_3$) δ (ppm): 9.71 (brs, 1H), 7.63–7.59 (m, 2H), 7.38–7.34 (m, 3H), 5.85–5.40 (m, 1H), 4.63–4.39 (m, 1H), 3.60 (s, 3H), 3.40–3.30 (m, 1H), 3.28–3.07 (m, 1H), 1.46 (s, 0.80H) 1.37 (s, 8.20H).

^{13}C NMR (101 MHz, $CDCl_3$) δ (ppm): 172.5, 155.4, 155.3, 135.2, 129.6, 128.7, 126.5, 80.1, 52.5, 51.7, 28.7, 28.4.

HRMS (ESI) *m/z* $[M+Na]^+$ calcd for $C_{16}H_{22}N_2NaO_5^+$: 345.1421, found 345.1423.

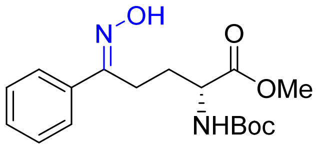

Methyl (*R*,*E*)-2-((*tert*-butoxycarbonyl)amino)-5-(hydroxyimino)-5-phenylpentanoate (114a) was synthesized by the general procedure **C** from methyl (*R*)-2-((*tert*-butoxycarbonyl)amino)-5-phenylpentanoate (153.7 mg, 0.5 mmol) and purified by flash column chromatography on silica gel (gradient elution: Petroleum ether : EtOAc = 10:1 to 4:1) to afford the product (*R,E)*-**114a** as a single isomer.

114a: 136.2 mg, 81% yield, white solid;

^{1}H NMR (400 MHz, $CDCl_3$) δ (ppm): 8.91 (brs, 1H), 7.60–7.54 (m, 2H), 7.39–7.34 (m, 3H), 5.29 (d, *J* = 8.4 Hz, 1H), 4.40 (dd, *J* = 14.0, 7.2 Hz, 1H), 3.72 (s, 3H), 2.90–2.80 (m, 2H), 2.21–2.06 (m, 1H), 2.00–1.85 (m, 1H), 1.45 (s, 9H).

^{13}C NMR (101 MHz, $CDCl_3$) δ (ppm): 173.1, 158.2, 155.6, 135.3, 129.4, 128.7, 126.2, 80.1, 53.6, 52.4, 28.9, 28.4, 22.4.

HRMS (ESI) *m/z* $[M+Na]^+$ calcd for $C_{17}H_{24}N_2NaO_5^+$: 359.1577, found 359.1576.

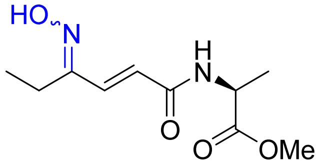

Methyl ((2*E*,4*E*/4*Z*)-4-(hydroxyimino)hex-2-enoyl)-*L*-alaninate (115a) was synthesized by the general procedure **B** from methyl (*E*)-hex-2-enoyl-*L*-alaninate (199.3 mg, 1 mmol) and purified by flash column chromatography on silica gel (gradient elution: Petroleum ether : EtOAc =10:1 to 3:1) to afford the product (2*E*,4*E*/4*Z*)**-115a** as a mixture of *E/Z* isomers, and epoxide was also obtained from the reaction.

The E/Z isomerism is with respect to the C=N bond, and ratio of ***(2E,4E)/(2E,4Z) = 3:1;***

115a: 103.0 mg, 45% yield, ((2*R*, 2*R'*)-**C2**) as catalyst, white solid;

Major (2*E*,4*E*)-isomer: ^{1}H NMR (400 MHz, $CDCl_3$) δ (ppm): 9.97 (brs, 1H), 7.19 (d, *J* =16.0 Hz, 1H), 6.89 (brs, 1H), 6.25 (d, *J* =16.0 Hz, 1H), 4.75–4.67 (m, 1H), 3.74 (s, 3H), 2.48 (q, *J* =7.6 Hz, 2H), 1.42 (d, *J* =7.2 Hz, 3H), 1.04 (t, *J* =7.6 Hz, 3H); **^{13}C NMR (**101 MHz, $CDCl_3$**)** δ (ppm): 174.1, 165.5, 159.6, 137.8, 124.1, 52.7, 48.3, 18.3, 17.8, 10.7; Chiral HPLC (chiralpak AD-H

column, *n*-hexane: isopropanol = 90:10; flow rate = 0.5 mL/min): t_{major} = 32.70 min., t_{minor} = 35.28 min.; >99% ee.

Minor (2*E*,4*Z*)-isomer: ^{1}H NMR (400 MHz, $CDCl_3$) δ (ppm): 9.97 (brs, 1H), 7.71 (d, *J* =16.4 Hz, 1H), 7.02 (brs, 1H), 6.36 (d, *J* =16.0 Hz, 1H), 4.75–4.67 (m, 1H), 3.74 (s, 3H), 2.38 (q, *J* =7.6 Hz, 2H), 1.42 (d, *J* =7.2 Hz, 3H), 1.09 (t, *J* =7.6 Hz, 3H); **^{13}C NMR (**101 MHz, $CDCl_3$**)** δ (ppm): 174.0, 165.5, 155.1, 127.8, 127.4, 52.7, 48.4, 24.6, 18.3, 11.7; Chiral HPLC (chiralpak AD-H column, *n*-hexane: isopropanol = 90:10; flow rate = 0.5 mL/min): t_{major} = 37.89 min., t_{minor} = 41.08 min.; >99% ee.

HRMS (ESI): *m/z* $[M+Na]^+$ calcd for $C_{10}H_{16}N_2NaO_4^+$: 251.1002; found: 251.1000.

Methyl (3-propyloxirane-2-carbonyl)-L-alaninate (115b)

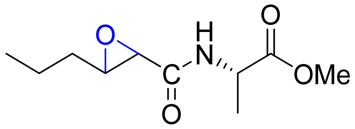

115b: 34% yield, *dr* = 5:1; **^{1}H NMR** (400 MHz, $CDCl_3$) δ (ppm): 6.71 –6.56 (m, 1H), 4.58–4.48 (m, 0.97H), 3.81–3.79 (m, 0.61H), 3.75–3.69 (m, 3.03H), 3.20 (s, 0.89H), 2.95–2.91 (m, 0.83H), 2.08–2.05 (m, 0.83H), 1.68–1.56 (m, 1.03H), 1.54–1.42 (m, 3.06H), 1.38–1.31 (m, 2.35H). 0.97–0.91 (m, 3.17H); **^{13}C NMR (**101 MHz, $CDCl_3$**)** δ (ppm): 172.9, 168.7, 59.6, 55.2, 52.6, 47.5, 33.7, 19.0, 18.3, 13.8; **HRMS (ESI):** *m/z* $[M+Na]^+$ calcd for $C_{10}H_{17}NNaO_4^+$: 238.1050; found: 238.1043

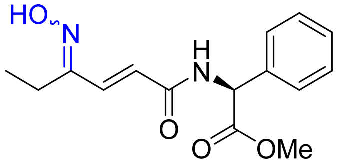

Methyl (*S*)-2-((2*E*,4*E/4Z*)-4-(hydroxyimino)hex-2-enamido)-2-phenylacetate (116a) was synthesized by the general procedure **B** from methyl (*S*,*E*)-2-(hex-2-enamido)-2-phenylacetate (261.1 mg, 1 mmol) and purified by flash column chromatography on silica gel (gradient elution: Petroleum ether : EtOAc = 3:1) to afford the products of (2*E*,4*E*/4*Z*)**-116a** as a mixture of *E/Z* isomers, and no epoxide was obtained from the reaction.

The E/Z isomerism is with respect to C=N bond, and ratio of ***(2E,4E)/(2E,4Z) = 3:1;***

116a: 130.6 mg, 45% yield, white solid;

Major (2*E*,4*E*)-isomer: ^{1}H NMR (400 MHz, $CDCl_3$) δ (ppm): 9.99 (brs, 1H), 7.38–7.28 (m, 5H), 7.21 (d, *J* = 16.0 Hz, 1H), 6.29 (d, *J* = 16.0 Hz, 1H), 5.72 (d, *J* = 7.2 Hz, 1H), 3.71 (s, 3H), 2.47 (q, *J* = 7.6 Hz, 2H), 1.03 (t, *J* = 7.6 Hz, 3H); **^{13}C NMR** (101 MHz, $CDCl_3$) δ (ppm): 171.8, 165.1, 159.6, 138.2, 136.2, 129.1, 128.7, 127.4, 123.8, 56.6, 53.0, 17.9, 10.7.

Minor (2*E*,4*Z*)-isomer: ^{1}H NMR (400 MHz, $CDCl_3$) δ (ppm): 9.99 (brs, 1H), 7.74 (d, *J* = 16.0 Hz, 1H), 7.38–7.28 (m, 5H), 6.39 (d, *J* = 16.0 Hz, 1H), 5.73 (d, *J* = 7.6 Hz, 1H), 3.71 (s, 3H), 2.35 (q, *J* = 7.6 Hz, 2H), 1.07 (t, *J* = 7.6 Hz, 3H); **^{13}C NMR** (101 MHz, $CDCl_3$) δ (ppm): 171.7, 165.1, 155.1, 138.2, 136.1, 128.1, 127.5, 127.1, 123.8, 56.7, 53.0, 24.6, 11.7.

HRMS (ESI): *m/z* $[M+Na]^+$ calcd for $C_{15}H_{18}N_2NaO_4^+$: 313.1159; found: 313.1162.

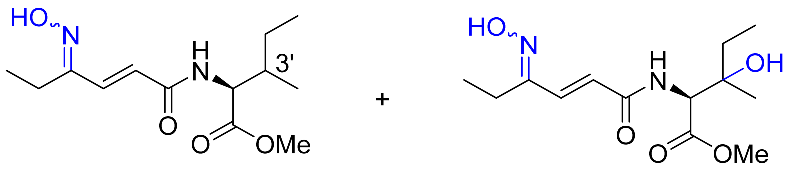

Methyl (2*S*)-2-((2*E*,4*E*/4*Z*)-4-(hydroxyimino)hex-2-enamido)-3-methylpentanoate (117a) and **methyl (2*S*)-3-hydroxy-2-((2*E*,4*E*/4Z)-4-(hydroxyimino)hex-2-enamido)-3-methylpentanoate (117a-3'-OH)** was synthesized by the general procedure **B** from methyl (2*S*)-2-((*E*)-hex-2-enamido)-3-methylpentanoate (241.3 mg, 1 mmol) and purified by flash column chromatography on silica gel (gradient elution: Petroleum ether : EtOAc = 3:1) to afford isolated product (2*E*,4*E*/4*Z*)-**117a** as an *E/Z* mixture, and trace amount of (2*E*,4*E*/4*Z*)-**117a-3'-OH** was also observed from the reaction. Epoxide was also obtained from the reaction.

The E/Z isomers is with respect to C=N bond, and ratio of (**2*E*,4*E***)/(**2*E*,4*Z***) = 2.4:1.

Methyl (2*S*)-2-((2*E*,4*E*/4*Z*)-4-(hydroxyimino)hex-2-enamido)-3-methylpentanoate:

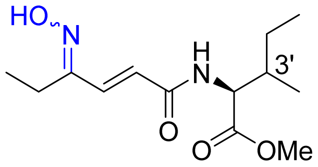

117a: (2*R*, 2*R'*)-C2 as catalyst, 116.2 mg, 43% yield, white solid; epoxide: 28% yield, *dr* = 2:1; white solid;

^{1}H NMR of major (2*E*,4*E*)-isomer: (400 MHz, $CDCl_3$) δ (ppm): 10.13 (brs, 1H), 7.19 (d, *J* = 16.0 Hz, 1H), 6.79–6.68 (m, 1H), 6.27 (d, *J* = 16.0 Hz, 1H). 4.75–4.68 (m, 1H), 3.72 (s, 3H), 2.48

(q, J = 7.6 Hz, 2H), 1.95–1.85 (m, 1H), 1.48–1.38 (m, 1H), 1.23–1.12 (m, 1H), 1.04 (t, J = 7.6 Hz, 3H), 0.91–0.85 (m, 6H).

^{1}H NMR of minor (2*E*,4*Z*)-isomer: (400 MHz, $CDCl_3$) δ (ppm): 10.13 (brs, 1H), 7.71 (d, J = 16.0 Hz, 1H), 6.89–6.75 (m, 1H), 6.38 (d, J = 16.0 Hz, 1H). 4.75–4.68 (m, 1H), 3.72 (s, 3H), 2.38 (q, J = 7.6 Hz, 2H), 1.95–1.85 (m, 1H), 1.48–1.38 (m, 1H), 1.23–1.12 (m, 1H), 1.09 (t, J = 7.6 Hz, 3H), 0.91–0.85 (m, 6H).

^{13}C NMR (101 MHz, $CDCl_3$) δ (ppm): 173.3, 173.1, 165.7, 159.5, 155.1, 137.8, 127.9, 127.5, 124.2, 56.8, 56.7, 52.4, 38.1, 25.4, 24.7, 17.9, 15.5, 11.7, 11.6, 10.7.

HRMS (ESI): *m/z* $[M+Na]^+$ calcd for $C_{13}H_{22}N_2NaO_4^+$: 293.1472; found: 293.1470.

Methyl (2*S*)-3-methyl-2-(3-propyloxirane-2-carboxamido)pentanoate

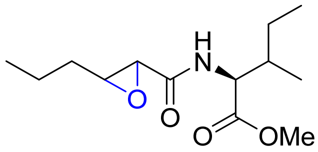

117b: 28% yield, *dr* = 2:1; **^{1}H NMR** (400 MHz, $CDCl_3$) δ (ppm): 6.61–6.49 (m, 1H), 4.53–4.47 (m, 1H), 3.70–3.67 (m, 3H), 3.21–3.19 (m, 1H), 3.00–2.86 (m, 1H), 1.90–1.78 (m ,1H), 1.65–1.60 (m ,1H), 1.55–1.42 (m, 3H), 1.39–1.29 (m, 1H), 1.16–1.03 (m, 1H), 0.95–0.91(m, 3H), 0.88–0.81 (m, 6H); **^{13}C NMR** (101 MHz, $CDCl_3$) δ (ppm): 172.1, 171.8, 168.70, 168.65, 59.8, 59.5, 55.8, 55.7, 55.3, 55.2, 52.20, 52.16, 37.9, 37.7, 33.73, 33.69, 25.12, 25.05, 19.0, 15.6, 15.5, 13.8, 11.6, 11.5; **HRMS (ESI):** *m/z* $[M+Na]^+$ calcd for $C_{13}H_{23}NNaO_4^+$: 280.1519; found: 280.1519.

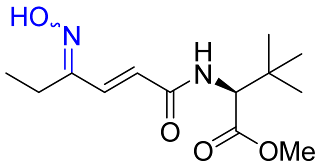

Methyl (*S*)-2-((2*E*,4*E*/4*Z*)-4-(hydroxyimino)hex-2-enamido)-3,3-dimethylbutanoate (118a) was synthesized by the general procedure **B** from methyl (*S*,*E*)-2-(hex-2-enamido)-3,3-dimethylbutanoate (241.3 mg, 1 mmol) and purified by flash column chromatography on silica gel (gradient elution: Petroleum ether : EtOAc =10:1 to 3:1) to afford the product (2*E*,4*E*/4*Z*)**-118a** as a mixture of *E*/*Z* isomers, and epoxide **118b** was also obtained from the reaction.

*The E/Z isomerism is with respect to C=N bond, and ratio of **(2E,4E)/(2E,4Z) = 3:1;***

(2*R*,2*R'*)-C2 as catalyst, **118a:**145.9 mg, 54% yield, white solid;

118b: 87.0 mg, 34% yield, *d.r.* = 4:1;

(2*S*,2*S'*)-C2 as catalyst, **118a:** 86.5 mg, 32% yield, white solid;

118b: 121.0 mg, 47% yield, *d.r.* > 99:1;

Major (2*E*,4*E*)-isomer: ^{1}**H NMR** (400 MHz, $CDCl_3$) δ (ppm): 10.35 (brs, 1H), 7.18 (d, *J* = 16.0 Hz, 1H), 6.85 (d, *J* = 9.6 Hz, 1H), 6.28 (d, *J* = 15.6 Hz, 1H), 4.58 (d, *J* = 9.6 Hz, 1H), 3.67 (s, 3H), 2.47–2.41 (m, 2H), 1.07–0.98 (m, 3H), 0.93 (s, 9H); ^{13}**C NMR** (101 MHz, $CDCl_3$) δ (ppm):172.7, 165.71, 165.69, 159.3, 137.9, 124.0, 60.2, 52.0, 34.9, 26.6, 17.8, 10.6.

Minor (2*E*,4*Z*)-isomer: ^{1}**H NMR** (400 MHz, $CDCl_3$) δ (ppm): 10.35 (brs, 1H), 7.79 (d, *J* = 16.0 Hz, 1H), 6.95 (d, *J* = 9.6 Hz, 1H), 6.40 (d, *J* = 16.0 Hz, 1H), 4.59 (d, *J* = 9.6 Hz, 1H), 3.67 (s, 3H), 2.37–2.31 (m, 2H), 1.07–0.98 (m, 3H), 0.93 (s, 9H); ^{13}**C NMR** (101 MHz, $CDCl_3$) δ (ppm): 172.5, 165.78, 165.75, 154.9, 127.9, 127.3, 60.3, 51.9, 34.9, 26.6, 24.6, 11.7.

HRMS (ESI): *m/z* $[M+Na]^+$ calcd for $C_{13}H_{22}N_2NaO_4^+$: 293.1472; found: 293.1473.

Methyl (2*S*)-3,3-dimethyl-2-(3-propyloxirane-2-carboxamido)butanoate (118b):

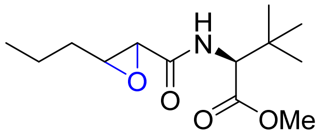

118b: 47% yield, *dr* > 99:1; ^{1}**H NMR** (400 MHz, $CDCl_3$) δ (ppm): 6.57 (d, *J* = 9.6 Hz, 1H), 4.35 (m, d, *J* = 10.0 Hz, 1H), 3.67 (s, 3H), 3.21 (d, *J* = 2.0 Hz,1H), 2.96–2.89 (m, 1H), 1.65–1.55 (m, 1H), 1.53–1.37 (m, 3H), 0.95–0.88 (m, 12H); ^{13}**C NMR (**101 MHz, $CDCl_3$**)** δ (ppm): 171.7, 168.5, 59.40, 59.37, 55.1, 51.8, 34.6, 33.7, 26.5, 19.0, 13.9;

HRMS (ESI): *m/z* $[M+Na]^+$ calcd for $C_{13}H_{23}NNaO_4^+$: 280.1519; found: 280.1519.

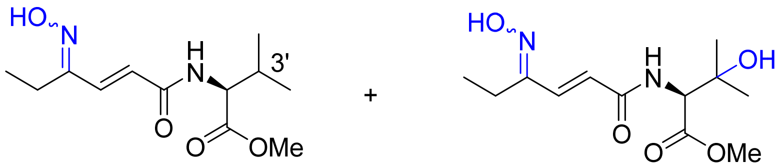

Methyl ((2*E*,4*E*/4*Z*)-4-(hydroxyimino)hex-2-enoyl)-*L*-valinate (119a) and **Methyl (*S*)-3-hydroxy-2-((2*E*,4*E*/4*Z*)-4-(hydroxyimino)hex-2-enamido)-3-methylbutanoate (119a-3'-OH)** was synthesized by the general Procedure **B** from methyl (*E*)-hex-2-enoyl-*L*-valinate (227.3 mg, 1 mmol) and purified by flash column chromatography on silica gel (gradient elution: Petroleum ether : EtOAc = 10:1 to 3:1) to afford isolated products (2*E*,4*E*/4*Z*)-**119a** as an *E/Z* mixture and

(2*E*,4*E*/4*Z*)-**119a-3’-OH** as an *E/Z* mixture; and a small amount of epoxide was obtained from the reaction.

The E/Z isomerism is with respect to C=N bond, and ratio of ***(2E,4E)/(2E,4Z)-119a = 7:2, (2E,4E)/(2E,4Z)-119a-OH = 4:1;***

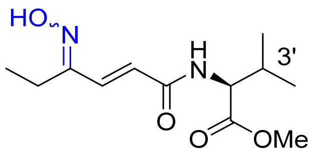

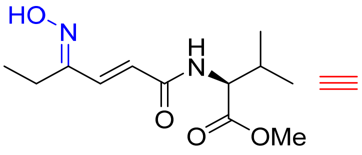

(2*E*,4*E*)-**119a**

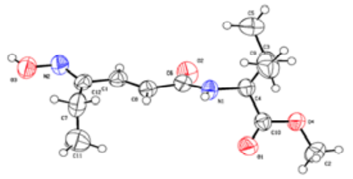

X-ray structure of (2*E*,4*E*)-**119a**

(2*E*,4*E/*4*Z*)-119a: 164.0 mg, 64% yield, white solid; **epoxide**: 8% yield;

Major (2*E*,4*E*)-isomer: ^{1}H NMR (400 MHz, $CDCl_3$) δ (ppm): 10.00 (brs, 1H), 7.21 (d, *J* = 16.0 Hz, 1H), 6.72 (d, *J* = 8.8 Hz, 1H), 6.28 (d, *J* = 16.0 Hz, 1H), 4.73–4.67 (m, 1H), 3.74 (s, 3H), 2.50 (q, *J* = 7.6 Hz, 2H), 2.14–2.12 (m, 1H), 1.06 (t, *J* = 7.6 Hz, 2H), 0.97–0.89 (m, 6H); **^{13}C NMR** (101 MHz, $CDCl_3$) δ (ppm): 173.4, 165.9, 159.6, 137.8, 124.2, 57.4, 52.5, 31.5, 19.1, 18.0, 10.7. The X-ray structure of **(2*E*,4*E*)-119a** has been determined.

Minor (2*E*,4*Z*)-isomer: ^{1}H NMR (400 MHz, $CDCl_3$) δ (ppm): 10.00 (brs, 1H), 7.72 (d, *J* = 16.4 Hz, 1H), 6.77 (d, *J* = 8.8 Hz, 1H), 6.40 (d, *J* = 16.4 Hz, 1H), 4.73–4.67 (m, 1H), 3.74 (s, 3H), 2.40 (q, *J* = 7.6 Hz, 2H), 2.14–2.12 (m, 1H), 1.11 (t, *J* = 7.6 Hz, 2H), 0.97–0.89 (m, 6H); **^{13}C NMR** (101 MHz, $CDCl_3$) δ (ppm): 173.2, 165.8, 155.1, 127.9, 127.6, 57.5, 52.5, 24.8, 18.1, 17.9, 11.7.

HRMS (ESI): *m/z* $[M+Na]^+$ calcd for $C_{12}H_{20}N_2NaO_4^+$: 279.1315; found: 279.1311.

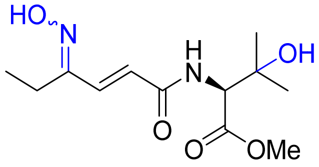

(2*E*,4*E*/4*Z*)-**119a-3’-OH:** 24.5 mg, 9% yield, white solid;

Major (2*E*,4*E*)-isomer: ^{1}H NMR (400 MHz, $CDCl_3$) δ (ppm): 7.16 (d, *J* = 16.0 Hz, 1H), 7.08–6.96 (m, 1H), 6.27 (d, *J* = 16.0 Hz, 1H), 4.64–4.58 (m, 1H), 3.70 (s, 3H), 2.44 (q, *J* = 7.6 Hz, 2H), 1.26–1.18 (m, 6H), 1.08–0.96 (m, 3H).

Minor (2*E*,4*Z*)-isomer: ^{1}H NMR (400 MHz, $CDCl_3$) δ (ppm): 7.69 (d, *J* = 16.0 Hz, 1H), 7.08–6.96 (m, 1H), 6.38 (d, *J* = 16.0 Hz, 1H), 4.64–4.58 (m, 1H), 3.68 (s, 3H), 2.34 (q, *J* = 7.6 Hz, 2H), 1.26–1.18 (m, 6H), 1.08–0.96 (m, 3H);

^{13}C NMR (101 MHz, $CDCl_3$) δ (ppm): 172.1, 166.2, 159.7 (2*E*,4*E*), 155.3 (2*E*,4*Z*), 138.2 (2*E*,4*E*), 128.2 (2*E*,4*Z*), 127.2 (2*E*,4*Z*), 123.9 (2*E*,4*E*), 72.2, 60.2, 52.6, 27.0, 26.8, 24.7, 17.9, 11.9, 10.7;
HRMS (ESI): *m/z* $[M+Na]^+$ calcd for $C_{12}H_{20}N_2NaO_5^+$: 295.1264; found: 295.1269.

(3a*R*,5a*S*,9a*S*,9b*R*,*E*)-9-(Hydroxyimino)-3a,6,6,9a-tetramethyldecahydronaphtho[2,1-*b*]furan-2(1*H*)-one (*E*-120a), (3a*R*,5a*S*,9aS,9b*R*,*E*)-8-(Hydroxyimino)-3a,6,6,9a-tetramethyldecahydronaphtho[2,1-*b*]furan-2(1*H*)-one (*E*-120b), (3a*R*,5a*S*,9a*S*,9b*R*,*Z*)-8-(Hydroxyimino)-3a,6,6,9a-tetramethyldecahydronaphtho[2,1-*b*]furan-2(1*H*)-one (*Z*-120b) and ***(3aR*,*5aR*,*9aS*,*9bR*,*E*)-7-(Hydroxyimino)-3a,6,6,9a-tetramethyldecahydronaphtho[2,1-*b*]furan-2(1*H*)-one (*E*-120c)** were synthesized by the general procedure **A** from sclareolide (250.4 mg, 1.0 mmol, 1.0 equiv.). Five different manganese catalysts, *rac*-**C1**, (*R*,*R*)-**C1**, (*S*,*S*)-**C1**, (*R*,*R*)-**C2**, and (*S*,*S*)-**C2**, were individually examined due to concerns over chirality match. (*S*,*S*)-**C1** was shown to be most efficient. The product was purified by flash column chromatography (gradient elution: petroleum ether : EtOAc=10:1 to 4:1, and then 3:1) to give four separated products: ***E*-120a, *E*-120b, *Z*-120b** and ***E*-120c**. The yields are shown in Table **S-11.**

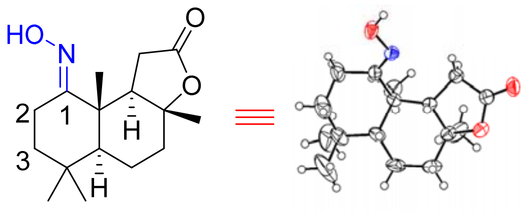

(3a*R*,5a*S*,9a*S*,9b*R*,*E*)-9-(Hydroxyimino)-3a,6,6,9a-tetramethyldecahydronaphtho[2,1-*b*]furan-2(1*H*)-one (*E*-120a):
^{1}H NMR (400 MHz, $CDCl_3$) δ (ppm): 7.16 (brs, 1H), 3.00 (dd, *J* = 16.8, 6.4 Hz, 1H), 2.87 (dt, 15.6, 6.0 Hz, 1H), 2.53 (t, *J* = 15.6 Hz, 1H), 2.40–2.26 (m, 2H), 2.10–2.02 (m, 1H), 1.87 (dd, *J* = 14.4, 3.2 Hz, 1H), 1.73–1.59 (m, 3H), 1.56–1.42 (m, 2H), 1.36 (s, 3H), 1.15 (s, 3H), 0.96 (s, 3H), 0.94 (s, 3H);
^{13}C NMR (101 MHz, $CDCl_3$) δ (ppm): 177.9, 166.0, 86.3, 55.2, 53.7, 43.1, 38.8, 38.0, 33.0, 32.0, 31.8, 23.0, 22.0, 21.0, 17.9, 16.3;
HRMS (ESI): *m/z* $[M+Na]^+$ calcd for $C_{16}H_{25}NNaO_3^+$: 302.1727; found: 302.1730.
The structure of ***E*-120a** has been determined by X-ray diffraction.

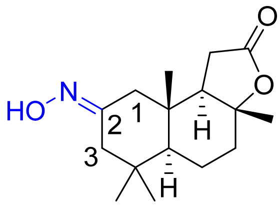

(3a*R*,5a*S*,9aS,9b*R*,*E*)-8-(Hydroxyimino)-3a,6,6,9a-tetramethyldecahydronaphtho[2,1-*b*]furan-2(1*H*)-one (*E*-120b):

^{1}H NMR (400 MHz, $CDCl_3$) δ (ppm): 8.48 (brs, 1H), 3.21 (d, *J* = 14.0 Hz, 1H), 2.44 (t, *J* = 15.6 Hz, 1H), 2.25 (dd, *J* = 16.0, 6.4 Hz, 1H), 2.15–2.03 (m, 3H), 1.97–1.84 (m, 2H), 1.79–1.68 (m, 1H), 1.58 (d, *J* = 14.0 Hz, 1H), 1.45–1.36 (m, 2H), 1.33 (s, 3H), 1.05 (s, 3H), 0.89 (s, 3H), 0.85 (s, 3H);

^{13}C NMR (101 MHz, $CDCl_3$) δ (ppm):176.3, 157.2, 86.1, 58.3, 56.6, 45.9, 39.7, 38.7, 38.4, 36.5, 32.8, 28.8, 22.3, 21.4, 20.7, 15.4;

HRMS (ESI): *m/z* $[M+Na]^+$ calcd for $C_{16}H_{25}NNaO_3^+$: 302.1727; found: 302.1731.

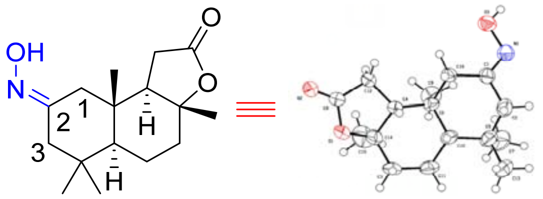

(3a*R*,5a*S*,9a*S*,9b*R*,Z)-8-(Hydroxyimino)-3a,6,6,9a-tetramethyldecahydronaphtho[2,1-*b*]furan-2(1*H*)-one (*Z*-120b):

^{1}H NMR (400 MHz, $CDCl_3$) δ (ppm): 8.67 (brs, 1H), 3.13 (d, *J* = 13.6 Hz, 1H), 2.49 (t, *J* = 15.6 Hz, 1H), 2.31 (dd, *J* = 16.0, 6.4 Hz, 1H), 2.19–2.07 (m, 3H), 2.02 (d, *J* = 13.6 Hz, 1H), 1.96–1.86 (m, 1H), 1.78–1.66 (m, 1H), 1.51–1.36 (m, 3H), 1.32 (s, 3H), 1.01 (s, 3H), 0.89 (s, 3H), 0.86 (s, 3H);

^{13}C NMR (101 MHz, $CDCl_3$) δ (ppm): 176.3, 157.2, 86.1, 58.2, 56.5, 47.2, 38.7, 38.4, 38.3, 36.5, 32.7, 28.9, 21.7, 21.3, 20.7, 15.9;

HRMS (ESI): *m/z* $[M+Na]^+$ calcd for $C_{16}H_{25}NNaO_3^+$: 302.1727; found: 302.1731.

The structure of ***Z*-120b** has been determined by X-ray diffraction.

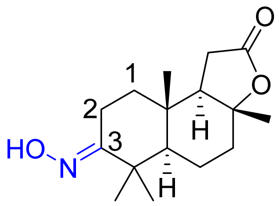

(*3aR*,*5aR*,*9aS*,*9bR*,*E*)-7-(Hydroxyimino)-3a,6,6,9a-tetramethyldecahydronaphtho[2,1-*b*]furan-2(1*H*)-one (*E*-120c):

^{1}H NMR (400 MHz, $CDCl_3$) δ (ppm): 3.09 (d, J = 14.0 Hz, 1H), 2.44 (t, J = 15.2 Hz, 1H), 2.36–2.20 (m, 2H), 2.10 (d, J = 12.0 Hz, 1H), 1.96–1.86 (m, 2H), 1.74–1.63 (m, 1H), 1.62–1.54 (m, 1H), 1.51–1.42 (m, 1H), 1.35 (s, 3H), 1.33–1.25 (m, 2H), 1.18 (s, 3H), 1.07 (s, 3H), 1.00 (s, 3H);

^{13}C NMR (101 MHz, $CDCl_3$) δ (ppm): 176.4, 165.3, 85.9, 58.5, 55.3, 40.3, 38.2, 37.3, 35.9, 28.7, 27.6, 22.8, 21.4, 21.0, 16.7, 14.6;

HRMS (ESI): *m/z* $[M+Na]^+$ calcd for $C_{16}H_{25}NNaO_3^+$: 302.1727; found: 302.1730.

The structures of compounds ***E-120a*** *and* ***Z-120b*** *were assigned based on their X-ray structures; The structures of compound* ***E-120b*** *and* ***E-120c*** *were determined by hydrolysis to the corresponding ketone according to the literature-reported protocol below; spectra data matched with values reported in the literature.* [122]

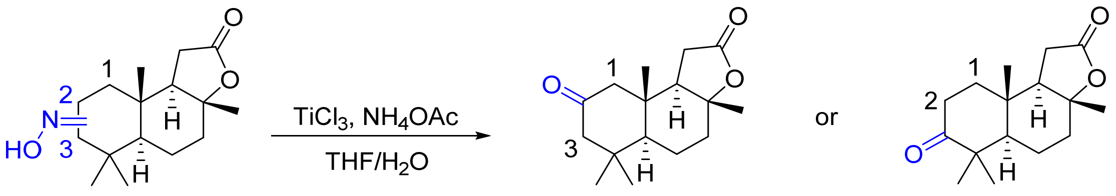

***E*-120b or *E*-120c** **120d**, 92%, isolated yield **120e**, 95%, isolated yield

To a solution of ***E-120b*** *or* ***E-120c*** (27.9 mg, 0.10 mmol) in THF/H_2O (4 mL/4 mL) were added NH_4OAc (100.2 mg, 1.3 mmol) and a buffered solution of $TiCl_3$ (0.22 mL of aqueous HCl solution containing 15% $TiCl_3$, 0.25 mmol). The mixture was stirred at rt for 10 h and then extracted with Et_2O. The extract was washed with saturated aqueous $NaHCO_3$ solution and brine, dried over Na_2SO_4, filtered, and evaporated in vacuo to give a solid. The solid was purified by silica gel flash column chromatography (petroleum ether/EtOAc: 4/1) to give **120d** as white solid (24.3 mg, 92%) or **120e** as white solid (25.1 mg, 95%).

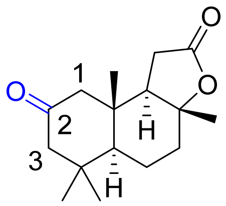

(3a*R*,5a*S*,9a*S*,9b*R*)-3a,6,6,9a-tetramethyldecahydronaphtho[2,1-*b*]furan-2,8-dione (**120d**) [119]: **^{1}H NMR** (400 MHz, $CDCl_3$) δ (ppm): 2.42 (t, *J* = 16.4 Hz, 1H), 2.30–2.10 (m, 7H), 1.99 (d, *J* = 13.6 Hz, 1H), 1.77 (td, *J* = 12.4, 4.0 Hz, 1H), 1.67 (td, *J* = 12.8 Hz, 1H), 1.46 (q, *J* = 9.6 Hz, 1H), 1.32 (s, 3H), 1.06 (s, 3H), 0.90 (s, 3H); **^{13}C NMR** (101 MHz, $CDCl_3$) δ (ppm): 209.4, 175.7, 85.7, 58.2, 56.6, 55.7, 55.0, 40.4, 38.7, 38.2, 33.3, 28.6, 22.7, 21.2, 20.8, 16.2.

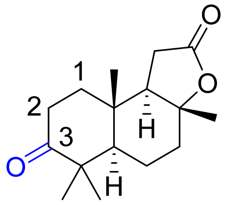

(3a*R*,5a*R*,9a*S*,9b*R*)-3a,6,6,9a-tetramethyloctahydronaphtho[2,1-*b*]furan-2,7(1*H*,3a*H*)-dione (**120e**) [123]: **^{1}H NMR** (400 MHz, $CDCl_3$) δ (ppm): 2.61–2.51 (m, 1H), 2.50–2.41 (m, 2H), 2.26 (dd, *J* = 16.0, 6.4 Hz, 1H), 2.10 (dt, *J* = 12.0, 3.2 Hz, 1H), 1.98 (dd, *J* = 15.2, 6.4 Hz, 1H), 1.86–1.79 (m, 1H), 1.76–1.66 (m, 2H), 1.63–1.46 (m, 3H), 1.36 (s, 3H), 1.10 (s, 3H), 1.04 (s, 3H), 1.01 (s, 3H); **^{13}C NMR** (101 MHz, $CDCl_3$) δ (ppm): 215.6, 176.0, 85.7, 58.2, 54.4, 47.4, 37.8, 37.7, 35.6, 33.5, 28.7, 26.7, 21.5, 21.2, 20.8, 14.6.

(3a*R*,5a*S*,9a*S*,9b*R*)-3a,6,6,9a-tetramethyldecahydronaphtho[2,1-*b*]furan-2,8-dione (**120d**), **(3a*R*,5a*R*,9a*S*,9b*R*)-3a,6,6,9a-tetramethyloctahydronaphtho[2,1-*b*]furan-2,7(1*H*,3a*H*)-dione** (**120e**), **(3a*R*,5a*S*,9a*S*,9b*R*)-8-hydroxy-3a,6,6,9a-tetramethyldecahydronaphtho[2,1-*b*]furan-2(1*H*)-one** (**120f**) **and** **(3a*R*,5a*S*,9a*S*,9b*R*)-9-hydroxy-3a,6,6,9a-tetramethyldecahydronaphtho[2,1-*b*]furan-2(1*H*)-one** (**120g**) were synthesized by the general procedure **A** from sclareolide (250.4 mg, 1.0 mmol, 1.0 equiv.) with the catalyst *rac*-**C1** (7.9 mg, 1.0 mol%) but **without $(NH_2OH)_2\cdot H_2SO_4$**. The residue was purified through flash column chromatography (gradient elution: Petroleum ether : EtOAc=10:1 to 4:1 to 3:1) to give the mixture of ketones **120d** and **120e** (135.0 mg, 51% yield, **120d: 120e** = 2:1), and the alcohols **120f** (51.0 mg, 19% yield) and **120g** (8.0 mg, 3% yield).

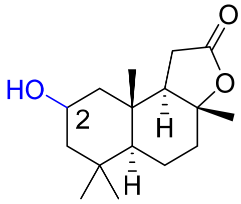

(3a*R*,5a*S*,9a*S*,9b*R*)-8-hydroxy-3a,6,6,9a-tetramethyldecahydronaphtho[2,1-*b*]furan-2(1*H*)-one (120f) [57]: **^{1}H NMR** (400 MHz, $CDCl_3$) δ (ppm): 4.01–3.90 (m, 1H), 2.41 (t, *J* = 15.2 Hz, 1H), 2.24 (dd, *J* = 16.4, 6.4 Hz, 1H), 2.06 (dt, *J* = 12.0, 3.6 Hz, 1H), 1.98 (dd, *J* = 14.8, 6.4 Hz, 1H), 1.92–1.84 (m, 1H), 1.83–1.75 (m, 2H), 1.66 (td, *J* = 12.4, 4.0 Hz, 1H), 1.40–1.32 (m, 1H), 1.30 (s, 3H), 1.17–1.09 (m, 1H), 1.09–1.02 (m, 2H), 0.92 (s, 6H), 0.85 (s, 3H); **^{13}C NMR** (101 MHz, $CDCl_3$) δ (ppm): 176.7, 86.3, 64.3, 58.9, 56.2, 51.4, 48.3, 38.5, 37.4, 34.8, 33.3, 28.8, 21.8, 21.7, 20.3, 16.2.

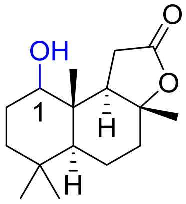

(3a*R*,5a*S*,9a*S*,9b*R*)-9-hydroxy-3a,6,6,9a-tetramethyldecahydronaphtho[2,1-*b*]furan-2(1*H*)-one (120g): **^{1}H NMR** (400 MHz, $CDCl_3$) δ (ppm): 3.21 (dd, *J* = 6.0, 4.0 Hz, 1H), 2.83 (d, *J* = 3.6 Hz, 1H), 2.42 (t, *J* = 15.6 Hz, 1H), 2.23 (dd, *J* = 16.0, 6.4 Hz, 1H), 2.06 (dt, *J* = 12.0, 3.2 Hz, 1H), 1.88 (dd, *J* = 14.8, 6.4 Hz, 1H), 1.84–1.75 (m, 2H), 1.66 (td, *J* = 12.4, 4.0 Hz, 1H), 1.59 (s, 1H), 1.48 (d, *J* = 14.4 Hz, 1H), 1.43–1.32 (m, 2H), 1.31 (s, 3H), 1.26–1.22 (m, 1H), 1.13 (s, 3H), 1.03 (s, 3H), 0.94 (s, 3H); **^{13}C NMR** (101 MHz, $CDCl_3$) δ (ppm): 176.4, 85.9, 61.7, 57.4, 52.0, 46.9, 39.7, 37.8, 35.0, 32.8, 28.5, 28.1, 21.5, 20.70, 20.65, 17.6.

Methyl (1*R*,4a*S*,10a*R*,*E*)-9-(hydroxyimino)-7-(2-hydroxypropan-2-yl)-1,4a-dimethyl-1,2,3,4,4a,9,10,10a-octahydrophenanthrene-1-carboxylate and **methyl (1*R*,4a*S*,10a*R*)-7-(2-hydroxypropan-2-yl)-1,4a-dimethyl-9-oxo-1,2,3,4,4a,9,10,10a-octahydrophenanthrene-1-carboxylate** were synthesized by the general Procedure **B** from methyl dehydroabietate (0.5 mmol, 125.2 mg, 1.0 equiv.) with the catalyst (*R*,*R*)-**C2** (7.9 mg, 2.0 mol%) or (*S*,*S*)-**C2** (7.9 mg, 2.0 mol%). The resulting reaction mixture was basified with Na_2CO_3 for 0.5 h or 20 h at room temperature, and the crude product was purified by flash column chromatography (gradient elution:

petroleum ether : EtOAc = 5:1 to 3:1) to afford **121a** and **121b**. The yields are shown in Table **S-12.**

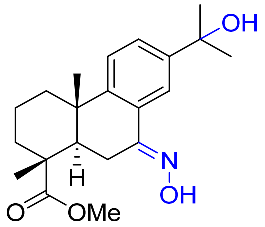

Methyl (1*R*,4a*S*,10a*R*,*E*)-9-(hydroxyimino)-7-(2-hydroxypropan-2-yl)-1,4a-dimethyl-1,2,3,4,4a,9,10,10a-octahydrophenanthrene-1-carboxylate (121a): **^{1}H NMR** (400 MHz, $CDCl_3$) δ (ppm): 7.95 (s, 1H), 7.45 (d, *J* = 8.0 Hz, 1H), 7.24 (d, *J* = 8.0 Hz, 1H), 3.64 (s, 3H), 2.69–2.62 (m, 2H), 2.34–2.25 (m, 2H), 1.78–1.69 (m, 4H), 1.56 (s, 6H), 1.36 (s, 3H), 1.22–1.33 (m, 2H), 1.10 (s, 3H); **^{13}C NMR** (101 MHz, $CDCl_3$) δ (ppm): 178.4, 155.7, 149.6, 147.1, 128.9, 126.2, 123.0, 120.4, 72.6, 52.3, 46.7, 41.7, 37.3, 37.2, 36.7, 31.7, 31.6, 24.0, 23.0, 18.2, 16.7; **HRMS (ESI):** *m/z* $[M+Na]^+$ calcd for $C_{21}H_{29}NNaO_4^+$: 382.1989; found: 382.1997.

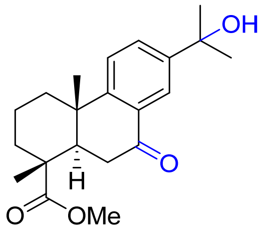

Methyl (1*R*,4a*S*,10a*R*)-7-(2-hydroxypropan-2-yl)-1,4a-dimethyl-9-oxo-1,2,3,4,4a,9,10,10a-octahydrophenanthrene-1-carboxylate (121b): **^{1}H NMR** (400 MHz, $CDCl_3$) δ (ppm): 8.04 (s, 1H), 7.73 (d, *J* = 8.4 Hz, 1H), 7.35 (d, *J* = 8.4 Hz, 1H), 3.64 (s, 3H), 2.77–2.65 (m, 2H), 2.40–2.31 (m, 2H), 1.84–1.61 (m, 6H), 1.57 (s, 6H), 1.33 (s, 3H), 1.25 (s, 3H); **^{13}C NMR** (101 MHz, $CDCl_3$) δ (ppm): 198.6, 177.9, 153.9, 147.5, 130.7, 130.6, 123.7, 123.3, 72.4, 52.4, 46.8, 43.8, 37.9, 37.5, 37.2, 36.6, 31.8, 31.7, 23.8, 18.2, 16.5; **HRMS (ESI):** *m/z* $[M+Na]^+$ calcd for $C_{21}H_{28}NaO_4^+$: 367.1880; found: 367.1875.

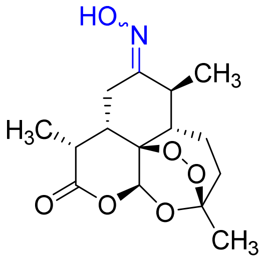

(3*R*,5a*S*,6*S*,8a*S*,9*R*,12*S*,12a*R*)-7-(Hydroxyimino)-3,6,9-trimethyloctahydro-12*H*-3,12-epoxy [1,2]dioxepino[4,3-*i*]isochromen-10(3*H*)-one (122a) was synthesized by the general procedure **A** (the solvent was changed to CH_3CN) from (+)-artemisinin (141.2 mg, 0.50 mmol, 1.0 equiv.), hydroxylamine sulfate (164.2 mg, 1.0 mmol. 2.0 equiv.) and AcOH (1.4 mL) with the manganese catalyst (*R*,*R*)-**C1**(5.1 mg, 1.0 mol%), and purified (gradient elution: petroleum ether/EtOAc= 4:1 to 2:1) to give **122a** and remaining **122.** The collected starting material **122** was reused for oxidative oximation twice, with the amount of other reagents required based on the amount of **122** used each time. The amount/yield of **122a** and that of recovered **122** are shown in Table **S-13.** When (*S*,*S*)-**C1** (5.1 mg, 1.0 mol%) or (*R*,*R*)-**C2** (4.0 mg, 1.0 mol%) was used as catalyst, the procedure for the oxidative oximation of **122** was similar with the exception that the remaining **122** was not recycled. **^{1}H NMR** (400 MHz, DMSO-*d6*) δ (ppm): 10.87 (s, 1H), 6.47 (s, 1H), 3.48 (dd, *J* = 14.0, 4.8 Hz, 1H), 3.24 (quint, *J* = 6.8 Hz, 1H), 2.50–2.42 (m, 1H), 2.36 (td, *J* = 12.8, 3.2 Hz, 1H), 2.20–2.12 (m, 1H), 2.09–2.01 (m, 1H), 1.92 (dt, *J* = 14.8, 4.8 Hz, 1H), 1.66–1.56 (m, 2H), 1.45 (s, 3H), 1.37–1.27 (m, 1H), 1.16 (d, *J* = 7.2 Hz, 3H), 1.13 (d, *J* = 7.2 Hz, 3H); **^{13}C NMR** (101 MHz, DMSO-*d6*) δ (ppm): 170.9, 155.1, 104.6, 92.6, 78.7, 49.2, 41.8, 39.7, 35.1, 32.4, 24.7, 24.6, 20.1, 12.9, 12.1; **HRMS (ESI):** *m/z* $[M+Na]^+$ calcd for $C_{15}H_{21}NNaO_6^+$: 334.1261; found: 334.1269.

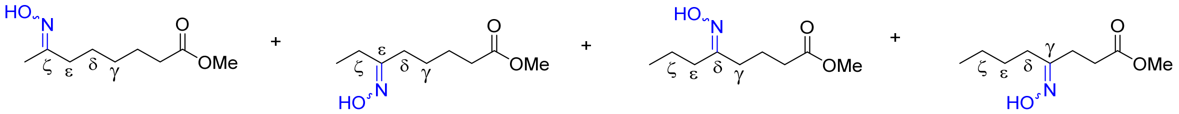

Methyl 7-(hydroxyimino)octanoate (123a-ζ-oxime), methyl 6-(hydroxyimino)octanoate (123a-ε-oxime), methyl 5-(hydroxyimino)octanoate (123a-δ-oxime) and **methyl 4-(hydroxyimino)octanoate (123a-γ-oxime) (123a)** were synthesized by the general procedure **A** from methyl octanoate (158.2 mg, 1 mmol) and purified by flash column chromatography on silica

gel (gradient elution: petroleum ether : EtOAc = 10:1 to 4:1) to afford the product **123a-ζ-oxime**, **123a-ε-oxime**, **123a-δ-oxime** and **123a-γ-oxime** (129.2 mg, 69% yield) as a mixture of four regioisomers with *E/Z* isomerism (Table S-10 and Fig. S-6).

Yield, 129.2 mg, 69%; ζ, 35%; γ+δ+ε=34%; colorless oil;

HRMS (ESI+): *m/z* $[M+Na]^+$ calculated for $C_9H_{17}NNaO_3^+$: 210.1101. found 210.1106.

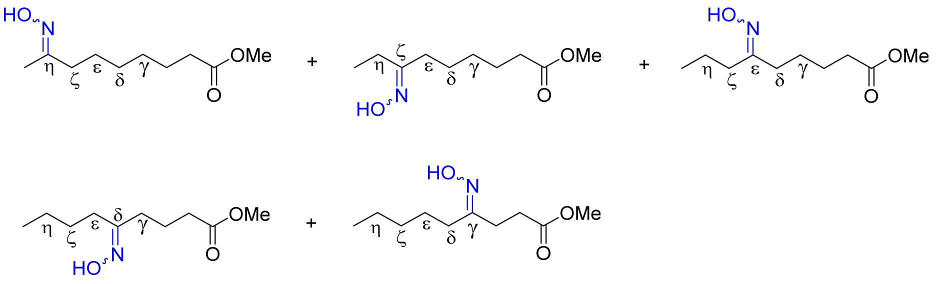

Methyl 8-(hydroxyimino)nonanoate (124a-η-oxime), methyl 7-(hydroxyimino)nonanoate (124a-ζ-oxime), methyl 6-(hydroxyimino)nonanoate (124a-ε-oxime), methyl 5-(hydroxyimino)nonanoate (124a-δ-oxime), and **methyl 4-(hydroxyimino)nonanoate (124a-γ-oxime) (124a)** were synthesized by the general **Procedure A** from methyl nonanoate (172.3 mg, 1 mmol) and purified by flash column chromatography on silica gel (gradient elution: petroleum ether : EtOAc = 10:1 to 4:1) to afford the product **124a-η-oxim**, **124a-ζ-oxime**, **124a-ε-oxime**, **124a-δ-oxime** and **124a-γ-oxime** (120.8 mg, 60% yield) as a mixture of five regioisomers with *E/Z* isomerism (Table S-10 and Fig. S-6).

Yield, 120.8 mg, 60%; η, 20%; γ+δ+ε+ζ=40%; colorless oil;

HRMS (ESI+): *m/z* $[M+Na]^+$ calculated for $C_{10}H_{19}NNaO_3^+$: 224.1257. found 224.1256.

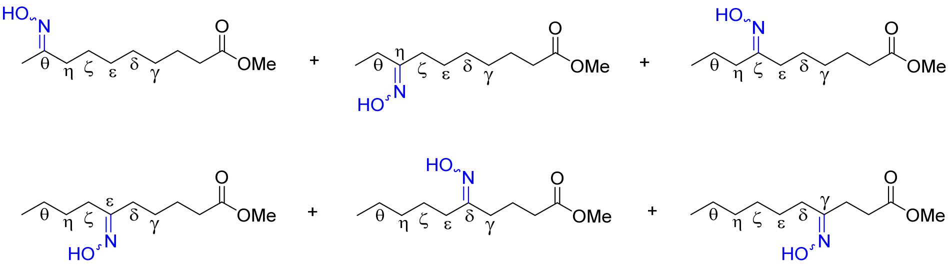

Methyl 9-(hydroxyimino)decanoate (125a-θ-oxime), methyl 8-(hydroxyimino)decanoate (125a-η-oxime), methyl 9-(hydroxyimino)decanoate (125a-ζ-oxime), methyl 9-(hydroxyimino)decanoate (125a-ε-oxime), methyl 9-(hydroxyimino)decanoate (125a-δ-oxime), and **methyl 9-(hydroxyimino)decanoate (125a-γ-oxime) (125a)** were synthesized by the general Procedure **A** from methyl decanoate (186.3 mg, 1 mmol) and purified by flash column

chromatography on silica gel (gradient elution: petroleum ether : EtOAc = 10:1 to 4:1) to afford the product **125a-θ-oxime**, **125a-η-oxim**, **125-ζ-oxime**, **125a-ε-oxime**, **125a-δ-oxime** and **125a-γ-oxime** (137.8 mg, 64%yield) as a mixture of six regioisomers with *E/Z* isomerism (Table S-10 and Fig. S-6).

Yield, 137.8 mg, 64%; θ, 21%; γ+δ+ε+ζ+η=43%; colorless oil;

HRMS (ESI+): *m/z* $[M+Na]^+$ calculated for $C_{11}H_{21}NNaO_3^+$: 238.1414. found 238.141

13. X-ray photoelectron spectroscopy characterization of *rac*-C1

The *XPS* spectrum of ***rac*-C1** shows Mn *2p* signals at 639.7 eV ($2p_{3/2}$) and 650.7 eV ($2p_{1/2}$), along with a Mn *3s* multiplet splitting of 5.9 eV. These values agree with the reported data for Mn^{II} complexes[124-126], supporting the +2 oxidation state of manganese in ***rac*-C1**.

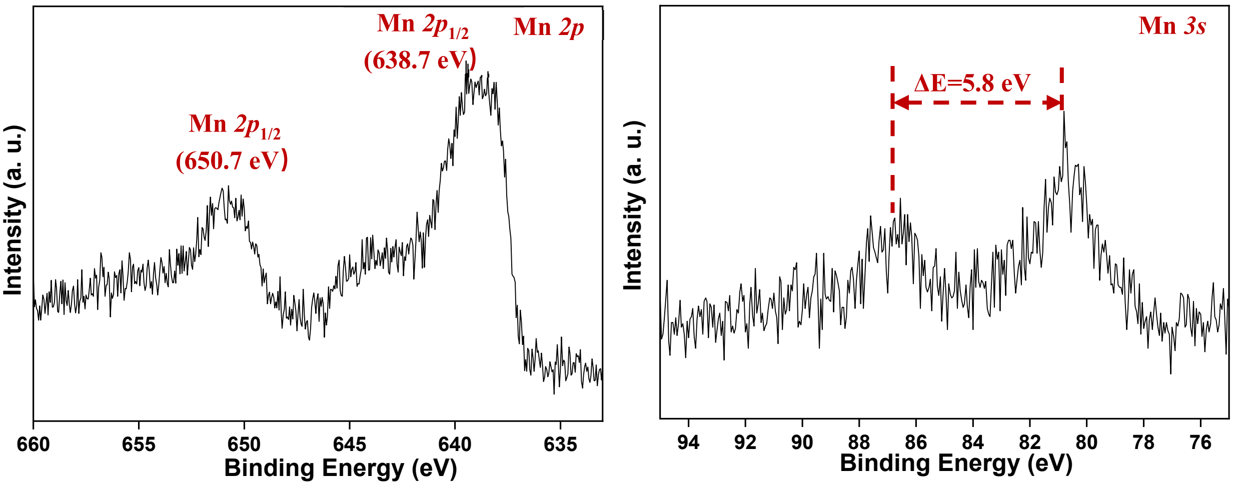

Fig. S-49. The XPS spectra of Mn *2p* and Mn *3s*

14. X-ray structures and crystallographic data

Crystals of ***rac*-C1**, ***meso*-C1** and ***rac*-C2** suitable for single crystal X-ray structure analysis were obtained from dry CH_3CN/CH_2Cl_2 under an N_2 atmosphere. Crystals of ***E*-22a-δ-oxime**, ***E*-65a**, **2*E*, 4*E*-119a**, ***E*-120a**, ***Z*-120b** were obtained from dry $CHCl_3/Et_2O$.

Reflections were collected on a Bruker Smart Apex II diffractometer using monochromated MoK_α (λ = 0.71073 Å) or CuK_α (λ = 1.54178 Å) radiation. All structures were refined against F^2 with full-matrix least-squares using the SHELX program[127]. Non-hydrogen atoms were refined anisotropically except those of some disordered groups. H-atoms were fixed in geometrical

positions. Disordered atom positions were split on two positions and refined with restraints. Crystallographic data are listed in Table Table S-22; structure diagrams with ellipsoids are shown in Figures S50 – S57.

Crystallographic data can be obtained free of charge from The Cambridge Crystallographic Data Centre (www.ccdc.cam.ac.uk/structures); deposition numbers are as follows: ***rac*-C1** (CCDC 2372363); ***meso*-C1** (CCDC 2372362); ***rac*-C2** (CCDC 2372364); ***E*-22a-δ-oxime** (CCDC 2372355); ***E*-65a** (CCDC 2372353); **2*E*,4*E*-119a** (CCDC 2372356); ***E*-120a** (CCDC 2372360); ***Z*-120b** (CCDC 2372361)

Table S-22. Crystallographic data.

	***rac*-C1**	***meso*-C1**	***rac*-C2**
Empirical formula	$C_{42}H_{74}F_6MnN_4O_8S_2Si_2$	$C_{43}H_{68}Cl_2F_6MnN_4O_6S_2Si_2$	$C_{28}H_{38}F_6MnN_4O_8S_2$
Formula weight	1052.29	1097.15	791.68
Temperature/K	183	254	200
Crystal system	monoclinic	orthorhombic	trigonal
Space group	$P2_1$	Fdd2	$P3_121$
a/Å	15.293(2)	16.9400(15)	12.7597(5)
b/Å	12.0168(17)	20.3528(17)	12.7597(5)
c/Å	16.775(2)	32.126(3)	22.1630(10)
α/°	90	90	90
β/°	93.547(5)	90	90
γ/°	90	90	120
Volume/Å^3	3076.8(7)	11076.3(16)	3124.9(3)
Z	2	8	3
ρ_{calc}g/cm^3	1.136	1.316	1.262
μ/mm^{-1}	0.382	0.519	0.487
F(000)	1114	4600	1227
Radiation	MoK_α	MoK_α	MoK_α
$2\Theta_{max}$/°	52.91	52.92	52.72
R_{int}	0.0816	0.0870	0.0493
R_{sigma}	0.0538	0.0421	0.0292
Data/restraints/parameters	12590/984/685	5714/1328/421	4248/184/261
Goodness-of-fit on F^2	1.051	1.125	1.059
R_1 [I>=2σ (I)]	0.0452	0.0809	0.0447
wR_2 [all data]	0.119	0.229	0.132
Largest diff. peak/hole / e Å^{-3}	0.52/-0.48	0.33/-0.29	0.25/-0.32
Flack parameter	0.04(2)	0.11(6)	0.02(3)

Table S-22 (continued)

	***E*-65a**	***E*-22a-δ-oxime**
Empirical formula	$C_7H_9NO_3$	$C_{16}H_{17}Cl_3N_2O_3$
Formula weight	155.15	391.66
Temperature/K	200	200
Crystal system	monoclinic	triclinic
Space group	$P2_1/n$	P-1
a/Å	5.1473(2)	5.3661(2)
b/Å	23.0631(7)	12.4165(6)
c/Å	6.4170(2)	15.0110(7)
α/°	90	110.102(2)
β/°	108.3150(10)	99.083(2)
γ/°	90	98.164(2)
Volume/Å^3	723.19(4)	906.44(7)
Z	4	2
ρ_{calc}g/cm^3	1.425	1.435
μ/mm^{-1}	0.951	0.522
F(000)	328	404
Radiation	CuK_α	MoK_α
$2\Theta_{max}$/°	136.47	52.83
R_{int}	0.0373	0.0618
R_{sigma}	0.0192	0.0346
Data/restraints/parameters	1321/0/118	3710/156/255
Goodness-of-fit on F^2	1.060	1.042
R_1 [I>=2σ (I)]	0.0336	0.0786
wR_2 [all data]	0.0911	0.2393
Largest diff. peak/hole / e Å^{-3}	0.23/-0.15	0.78/-0.67

Table S-22 (continued)

	**2*E*,4*E*-119a**	***E*-120a**	***Z*-120b**
Empirical formula	$C_{12}H_{20}N_2O_4$	$C_{16}H_{25}NO_3$	$C_{16}H_{25}NO_3$
Formula weight	256.30	279.37	279.37
Temperature/K	250	200	200
Crystal system	orthorhombic	monoclinic	orthorhombic
Space group	$P2_12_12$	$P2_1$	$P2_12_12_1$
a/Å	10.8793(5)	7.1240(4)	8.2569(3)
b/Å	13.4197(6)	20.9383(12)	13.3839(4)
c/Å	10.0807(4)	15.5395(8)	14.1988(4)
α/°	90	90	90
β/°	90	99.396(2)	90
γ/°	90	90	90
Volume/Å^3	1471.75(11)	2286.8(2)	1569.10(9)
Z	4	6	4
ρ_{calc}g/cm^3	1.157	1.217	1.183
μ/mm^{-1}	0.721	0.083	0.081
F(000)	552	912	608
Radiation	CuK_α	MoK_α	MoK_α
$2\Theta_{max}$/°	136.54	52.98	52.75
R_{int}	0.0593	0.1128	0.0820
R_{sigma}	0.0339	0.0931	0.0313
Data/restraints/parameters	2656/336/172	9367/49/556	3209/0/186
Goodness-of-fit on F^2	1.061	1.012	1.072
R_1 [I>=2σ (I)]	0.0551	0.0874	0.0388
wR_2 [all data]	0.1540	0.2603	0.1066
Largest diff. peak/hole / e Å^{-3}	0.44/-0.19	0.84/-0.44	0.17/-0.22
Flack parameter	0.09(10)	0.3(10)	-1.4(5)

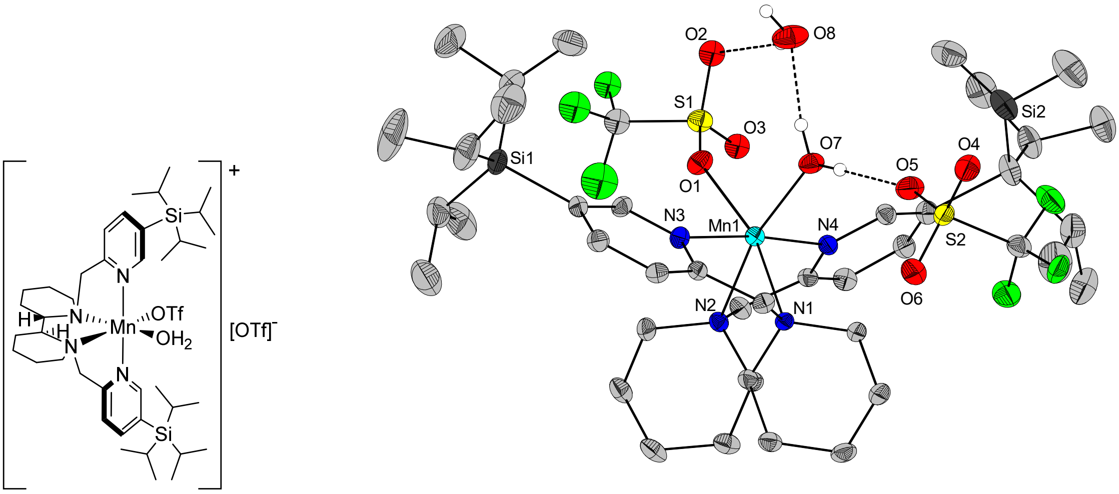

Figure S-50. Crystal structure of ***rac*-C1**. One triflate (S1) and one $(iPr)_3Si$ group (Si2) are disordered and were split over two positions; only one moiety is shown. Hydrogen atoms have been omitted for clarity (except those involved in hydrogen bonding). Thermal ellipsoids at 30% probability.

Table S-23. Selected bond lengths and angles of ***rac*-C1**:

Mn1	O1	2.171(3)
Mn1	O7	2.172(3)
Mn1	N3	2.214(3)
Mn1	N4	2.218(3)
Mn1	N1	2.274(3)
Mn1	N2	2.299(3)

O1	Mn1	O7	91.36(13)
O1	Mn1	N3	90.11(12)
O7	Mn1	N3	91.98(14)
O1	Mn1	N4	92.80(12)
O7	Mn1	N4	92.57(13)
N3	Mn1	N4	174.53(13)
O1	Mn1	N1	164.37(12)
O7	Mn1	N1	96.54(12)
N3	Mn1	N1	76.20(13)
N4	Mn1	N1	100.26(13)
O1	Mn1	N2	95.75(13)
O7	Mn1	N2	166.31(12)
N3	Mn1	N2	99.66(13)
N4	Mn1	N2	75.45(12)
N1	Mn1	N2	79.46(12)

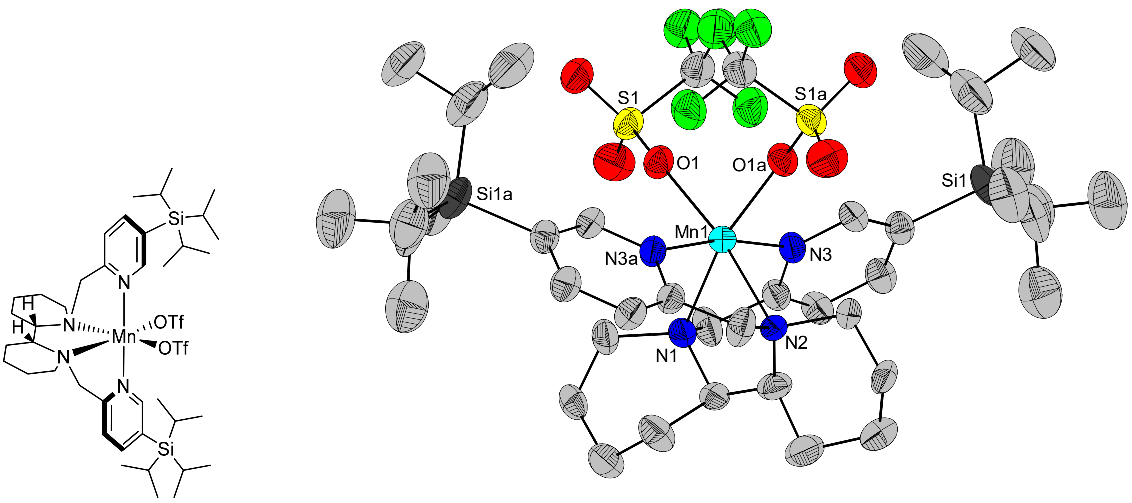

Figure S-51. Crystal structure of ***meso*-C1**. The triflate and the (iPr)$_3$Si group are disordered. The bipiperidine unit of the ligand is disordered by symmetry of the crystallographic 2-fold axis. All disordered atoms were split over two positions; only one moiety is shown. Hydrogen atoms and a disordered dichloromethane molecule have been omitted for clarity. Thermal ellipsoids at 30% probability.

Table S-24. Selected bond lengths and angles of ***meso*-C1** (these must be treated with caution due to the disorder of the bipiperidine section of the ligand):

Mn1	N1	2.37(3)
Mn1	N2	2.29(3)
Mn1	N3	2.213(7)
Mn1	O1	2.131(12)

N2	Mn1	N1	80.0(4)
N3a	Mn1	N1	98.4(7)
N3	Mn1	N1	73.7(7)
N3a	Mn1	N2	79.0(6)
N3	Mn1	N2	93.2(6)
N3	Mn1	N3a	169.9(4)
O1a	Mn1	N1	164.4(9)
O1	Mn1	N1	98.4(8)
O1a	Mn1	N2	97.0(7)
O1	Mn1	N2	169.8(8)
O1a	Mn1	N3	91.3(6)
O1	Mn1	N3	96.0(6)
O1	Mn1	O1a	87.2(11)

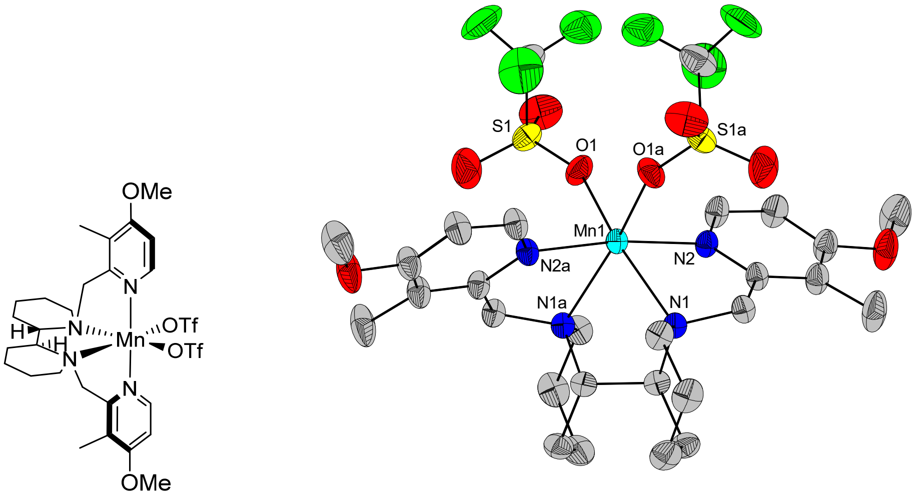

Figure S-52. Crystal structure of ***rac*-C1**. The CF_3 group of the triflate is disordered and was split over two positions; only one moiety is shown. Hydrogen atoms have been omitted for clarity Thermal ellipsoids at 30% probability.

Table S-25. Selected bond lengths and angles of ***rac*-C2**:

Mn1	N1	2.294(3)
Mn1	N2	2.212(3)
Mn1	O1	2.162(3)

N1	Mn1	N1a	79.68(16)
N2a	Mn1	N1	99.67(12)
N2	Mn1	N1	74.60(11)
N2a	Mn1	N2	172.71(19)
O1	Mn1	N1	161.56(12)
O1a	Mn1	N1	95.04(13)
O1a	Mn1	N2	95.86(13)
O1	Mn1	N2	89.07(12)
O1	Mn1	O1a	95.1(2)

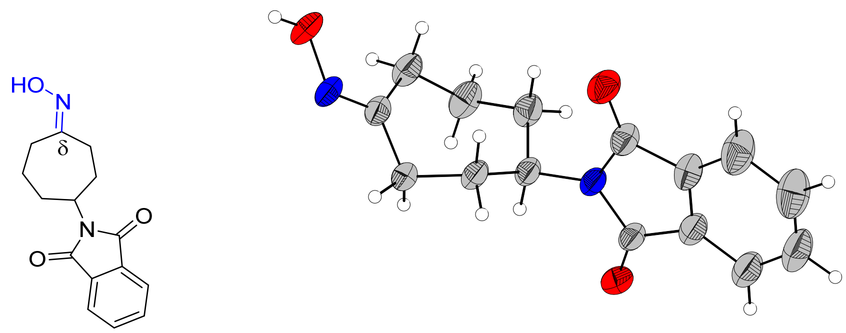

Figure S-53. Crystal structure of ***E*-22a-δ-oxime**. Thermal ellipsoids at 50% probability. There is also one chloroform molecule per formula unit which is disordered (not shown here).

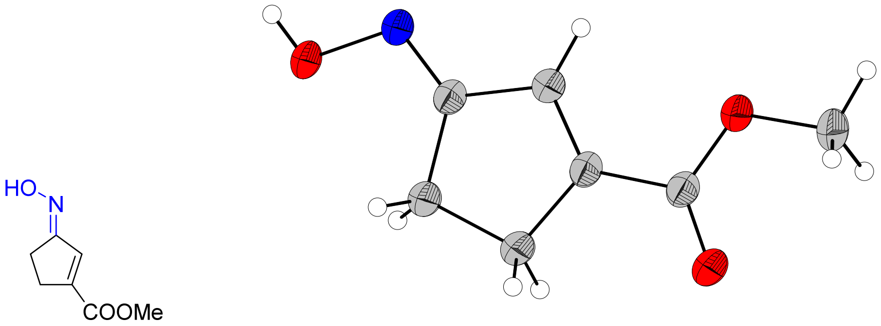

Figure S-54. Crystal structure of ***E*-65a**. Thermal ellipsoids at 50% probability.

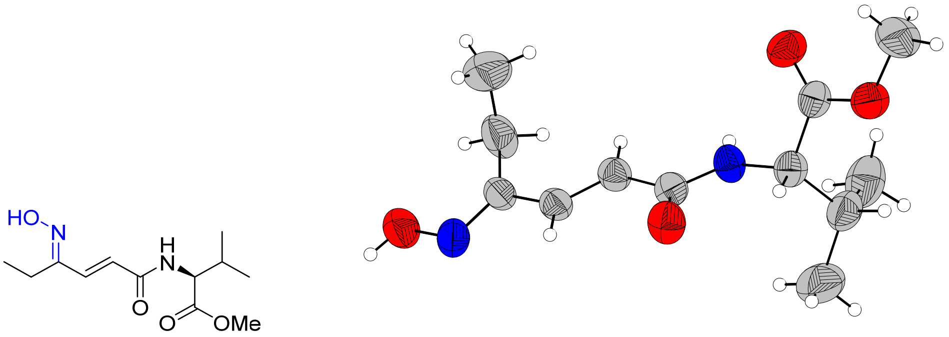

Figure S-55. Crystal structure of **2*E*,4*E*-119a**. Thermal ellipsoids at 50% probability.

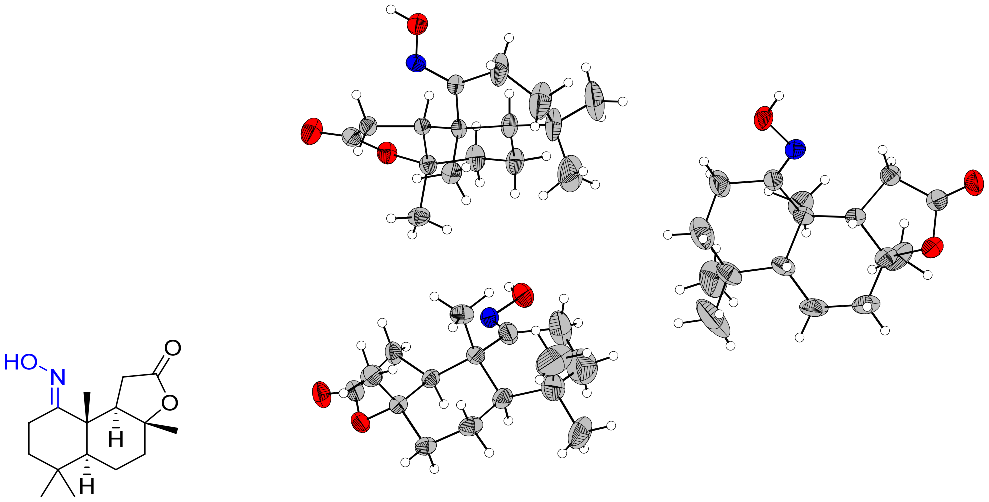

Figure S-56. Crystal structure of ***E*-120a** showing the three crystallographically independent molecules. Thermal ellipsoids at 50% probability.

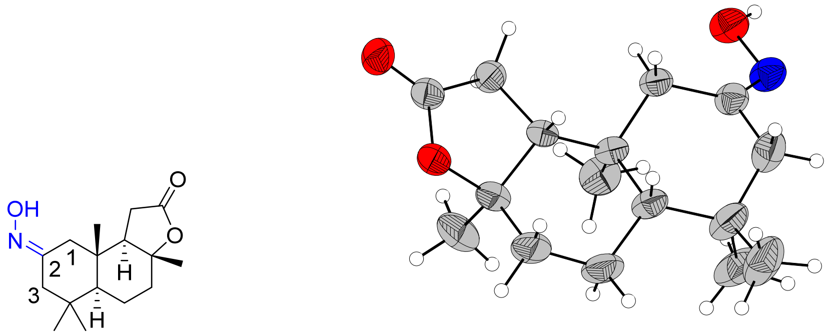

Figure S-57. Crystal structure of ***Z*-120b**. Thermal ellipsoids at 50% probability.

15. ^{1}H, ^{13}C and ^{19}F NMR spectra

^{1}H NMR, 400 MHz, $CDCl_3$

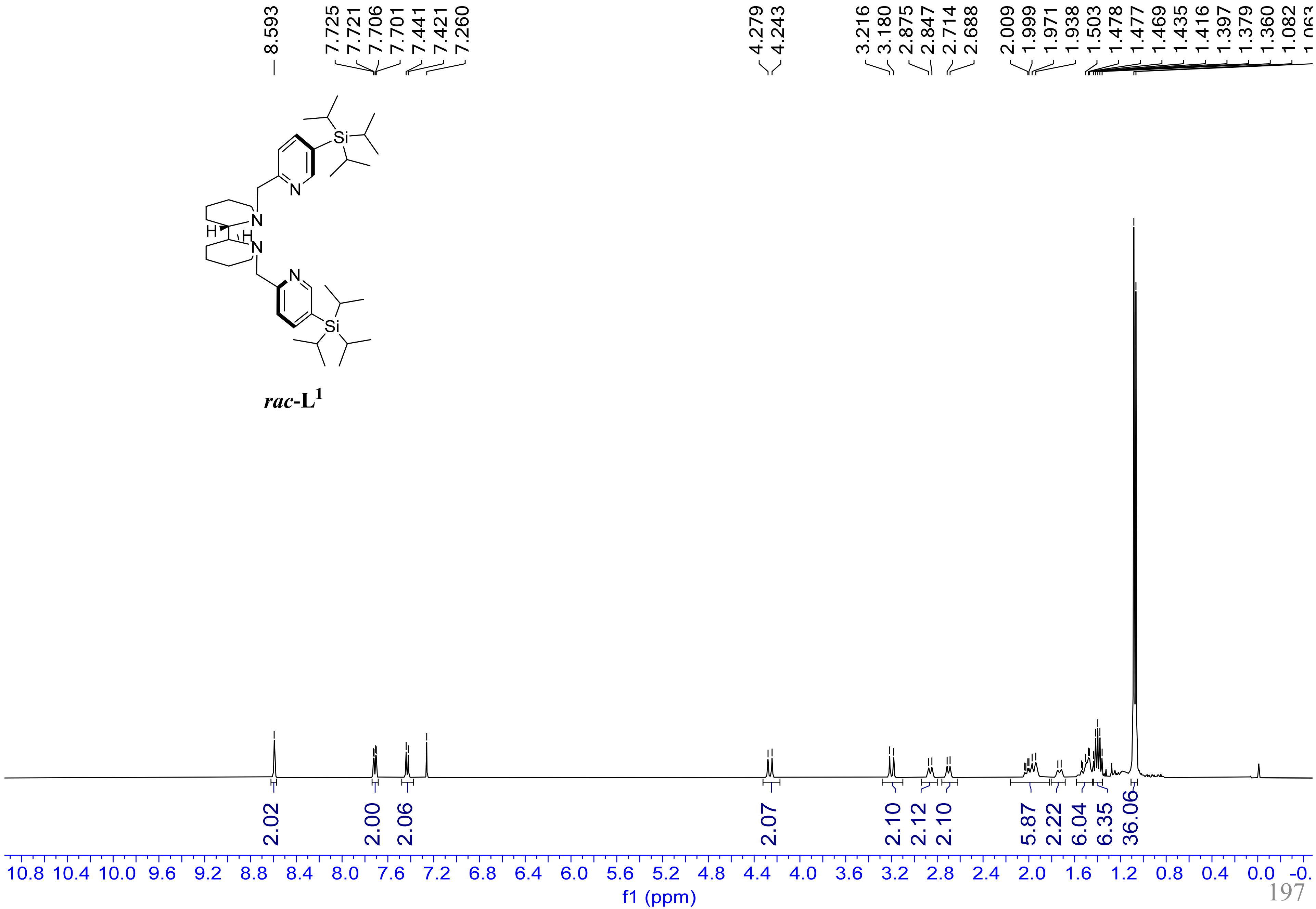

^{13}C NMR 101 MHz, $CDCl_3$

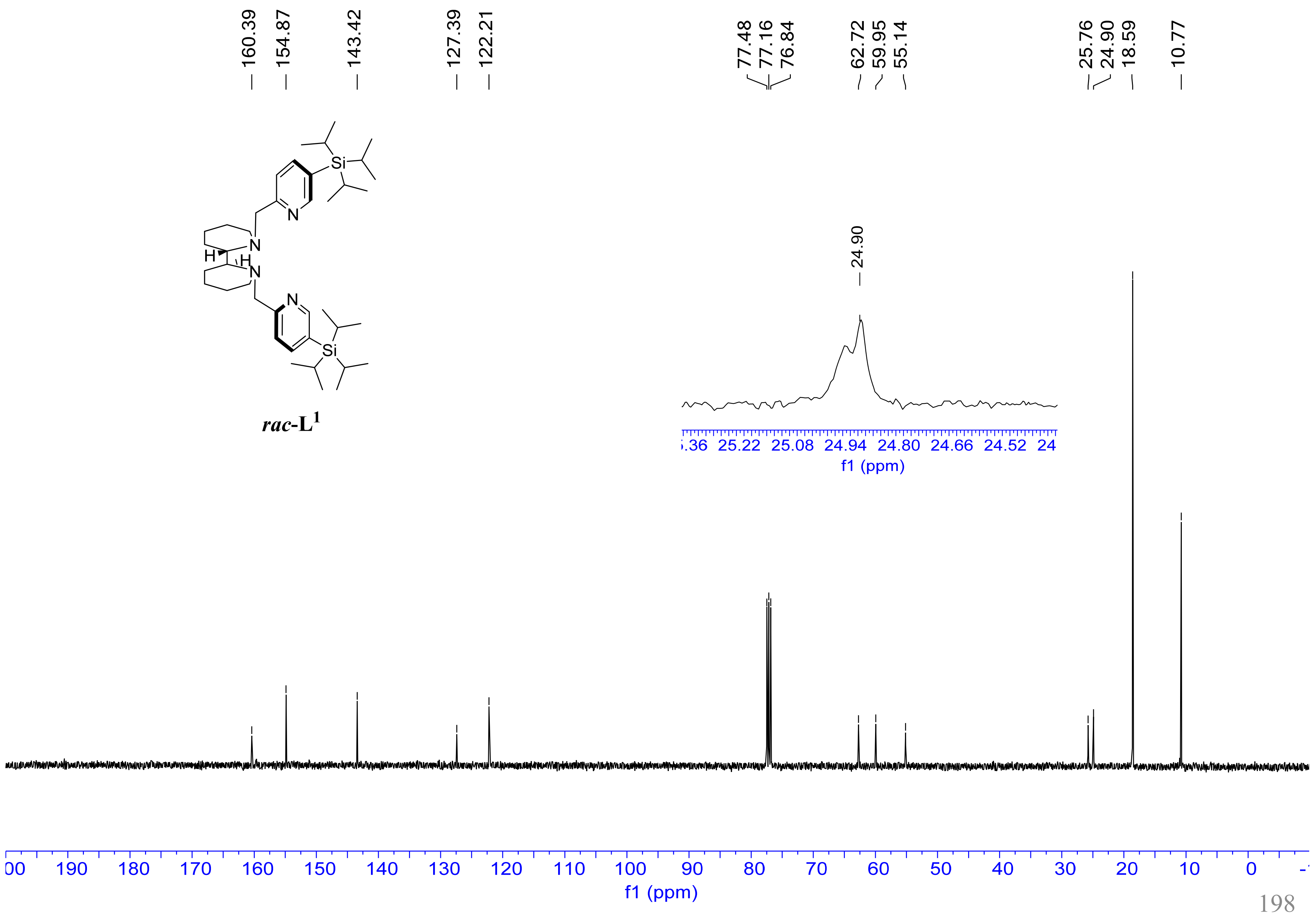

***rac*-L[1]**

^{1}H NMR, 400 MHz, $CDCl_3$

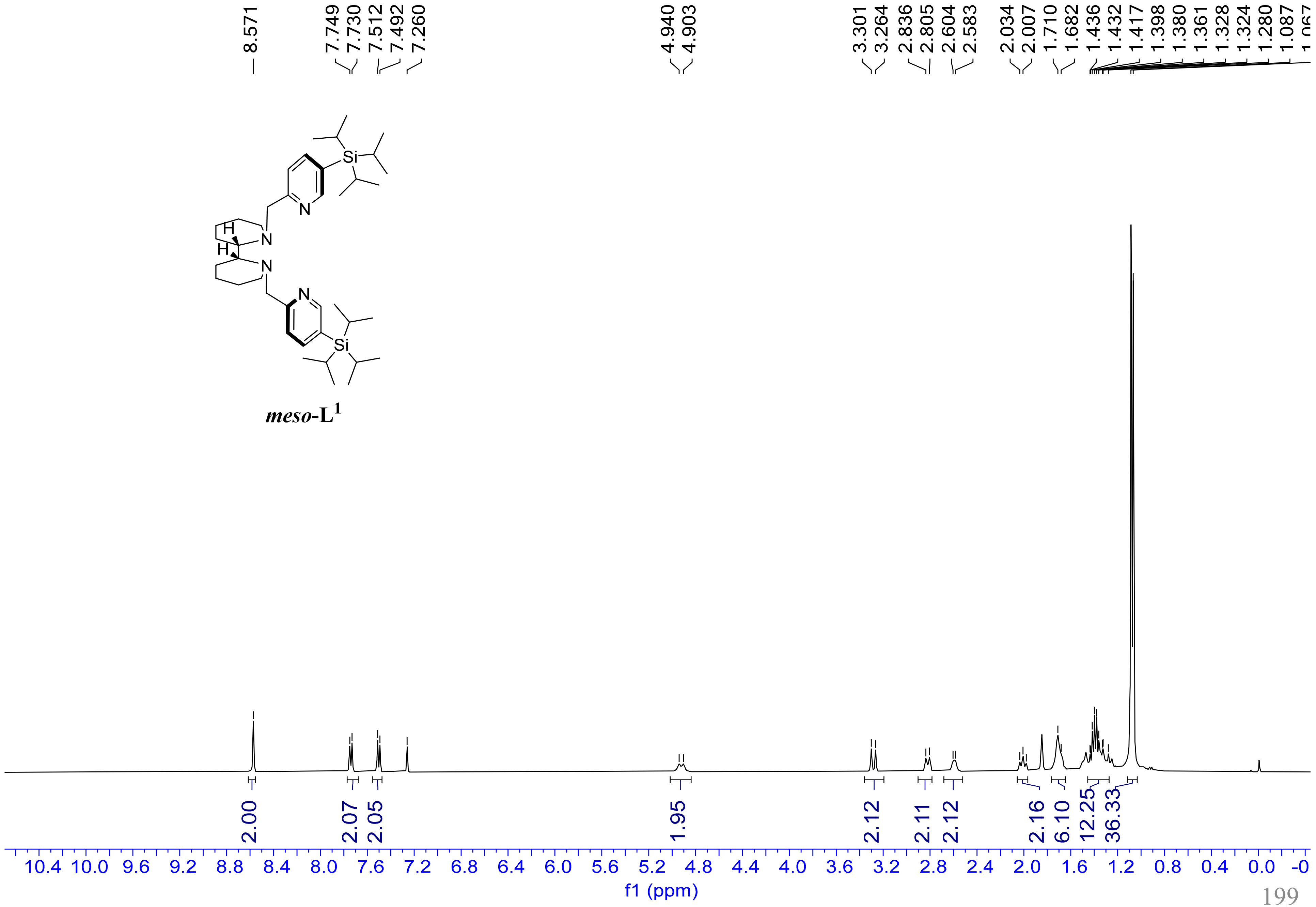

^{13}C NMR 101 MHz, $CDCl_3$

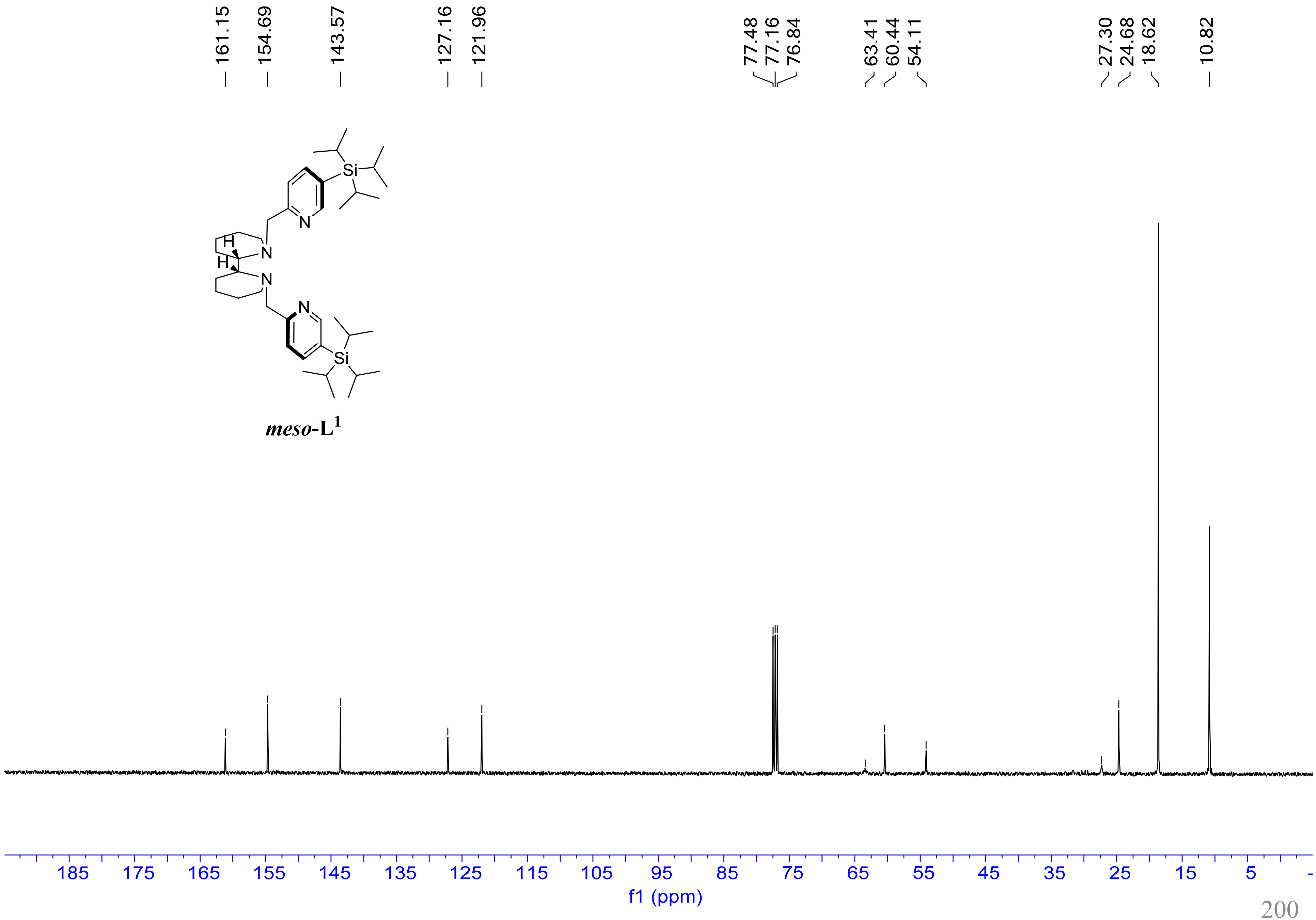

meso-L[1]

^{1}H NMR, 400 MHz, $CDCl_3$

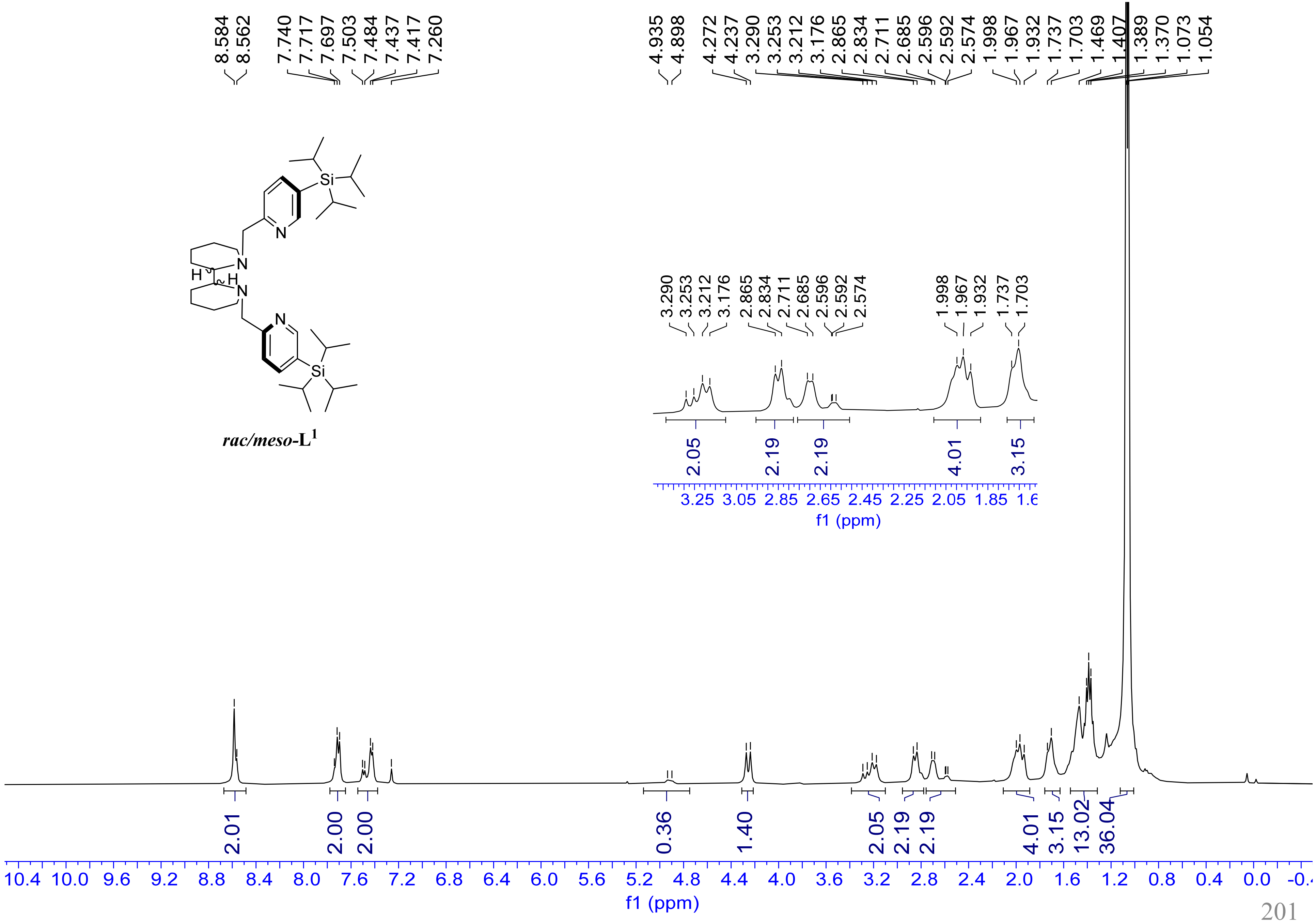

rac/meso-L[1]

^{13}C NMR 101 MHz, CDCl$_3$

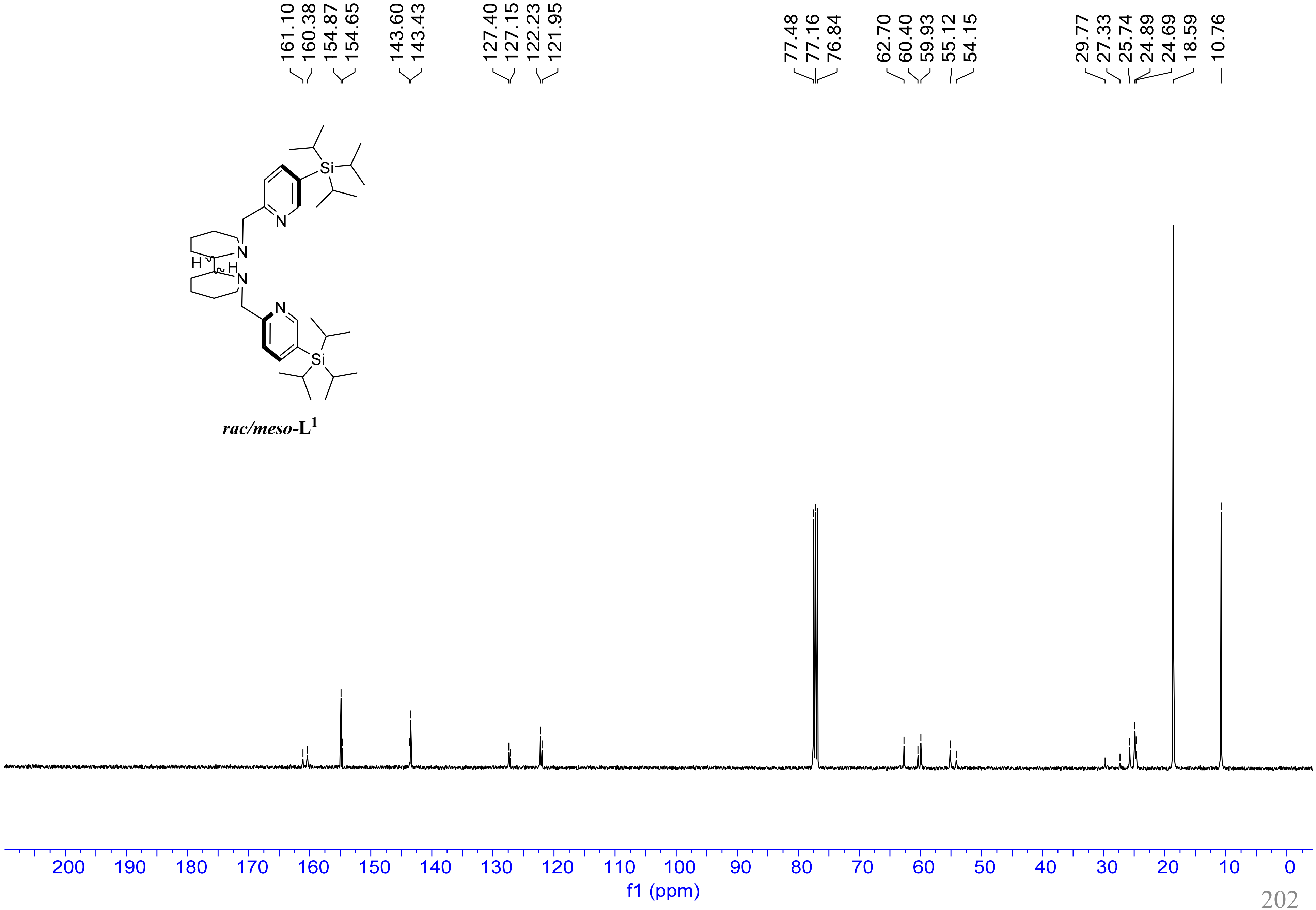

rac/meso-L[1]

^{1}H NMR, 400 MHz, $CDCl_3$

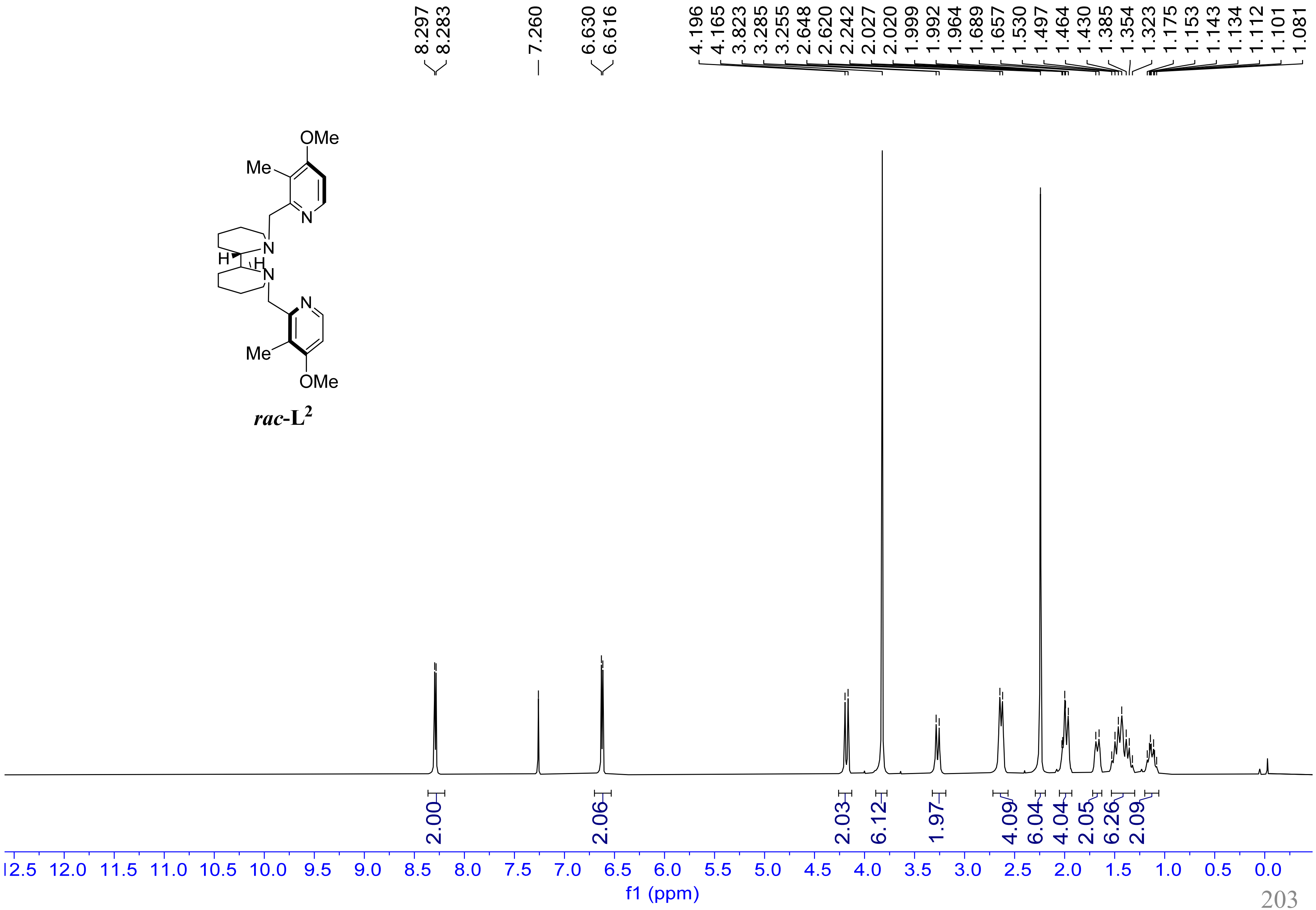

***rac*-L²**

^{13}C NMR 101 MHz, $CDCl_3$

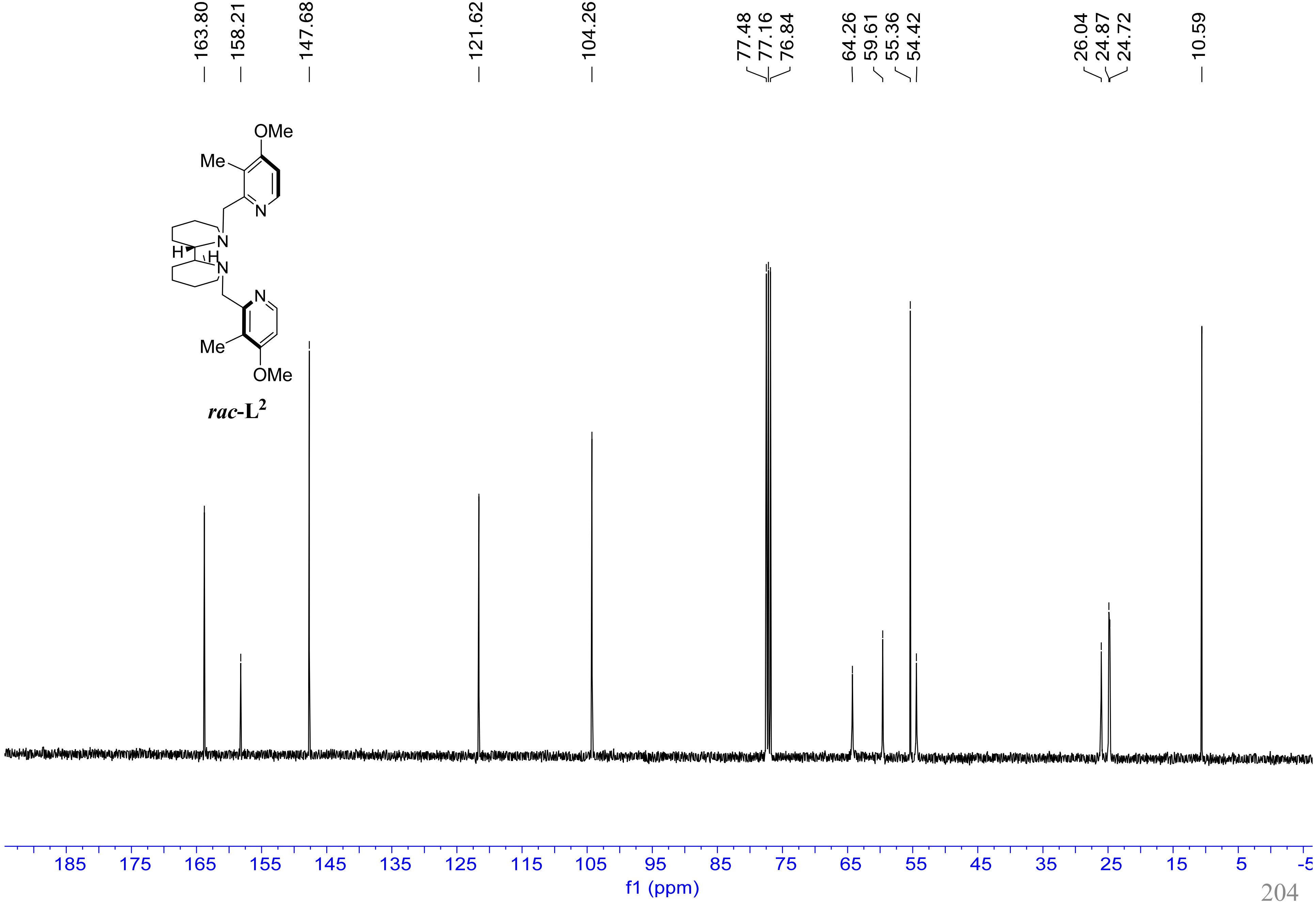

^{1}H NMR, 400 MHz, $CDCl_3$

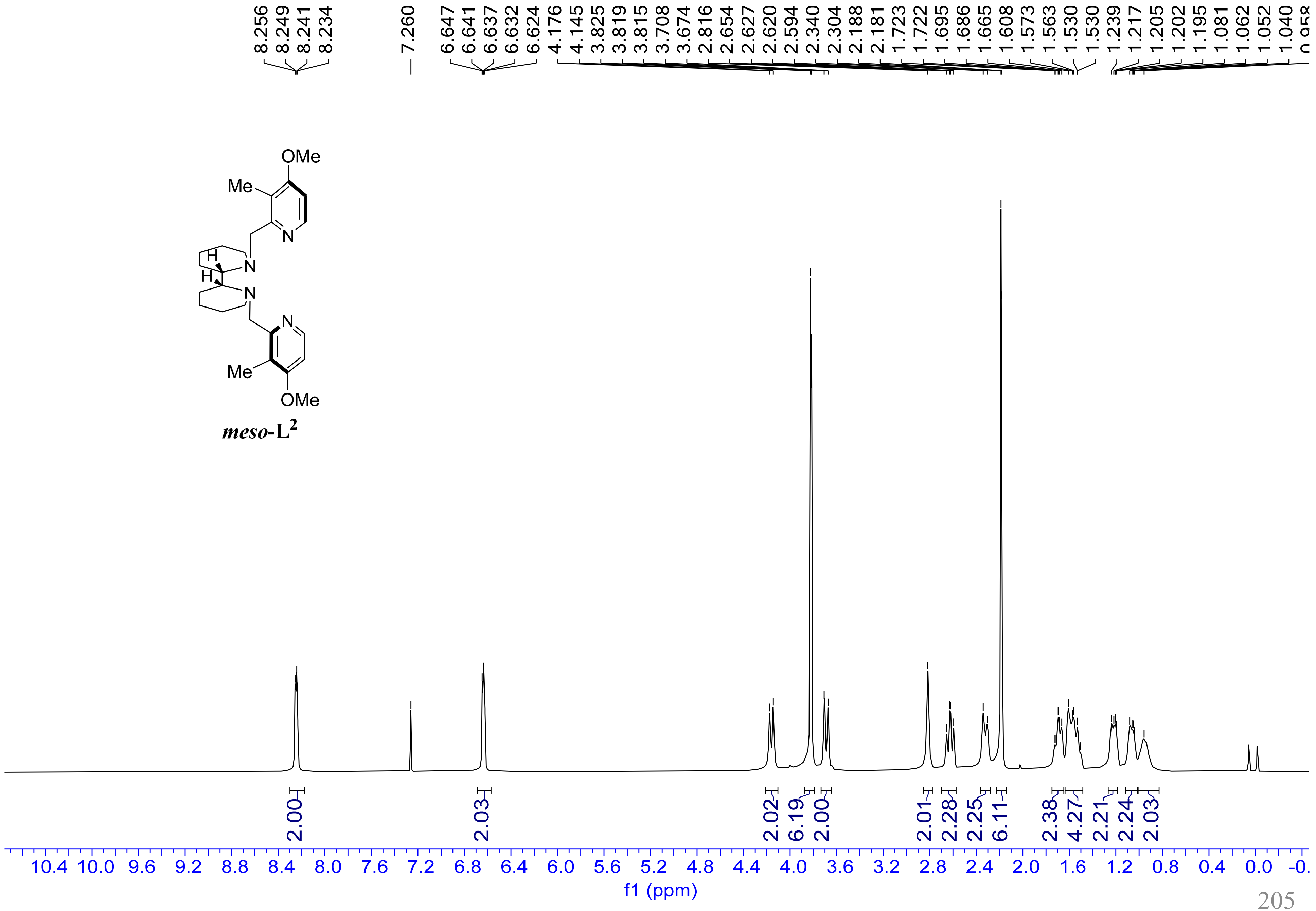

***meso*-L^2**

^{13}C NMR 101 MHz, $CDCl_3$

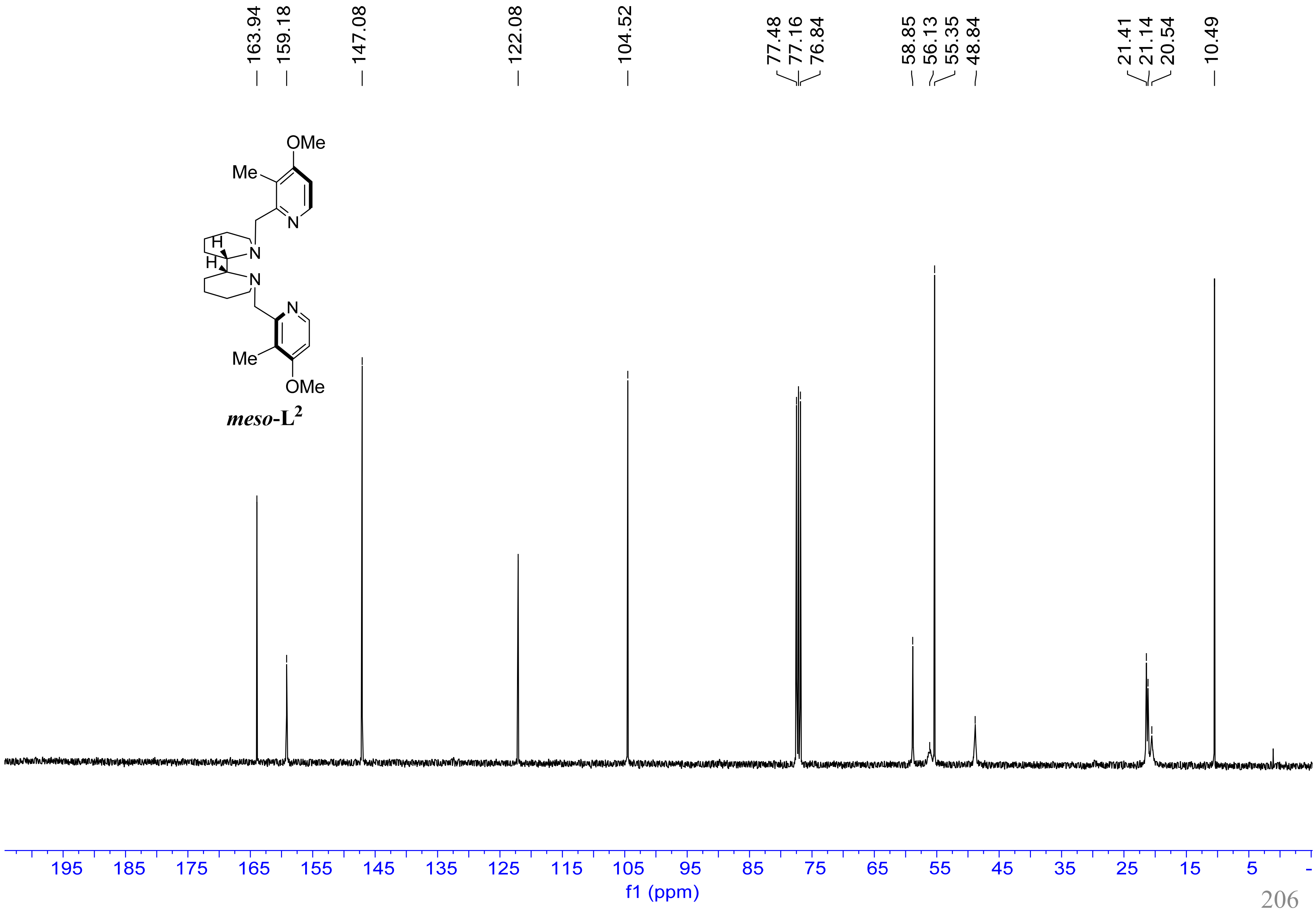

meso-L[2]

^{1}H NMR, 400 MHz, $CDCl_3$

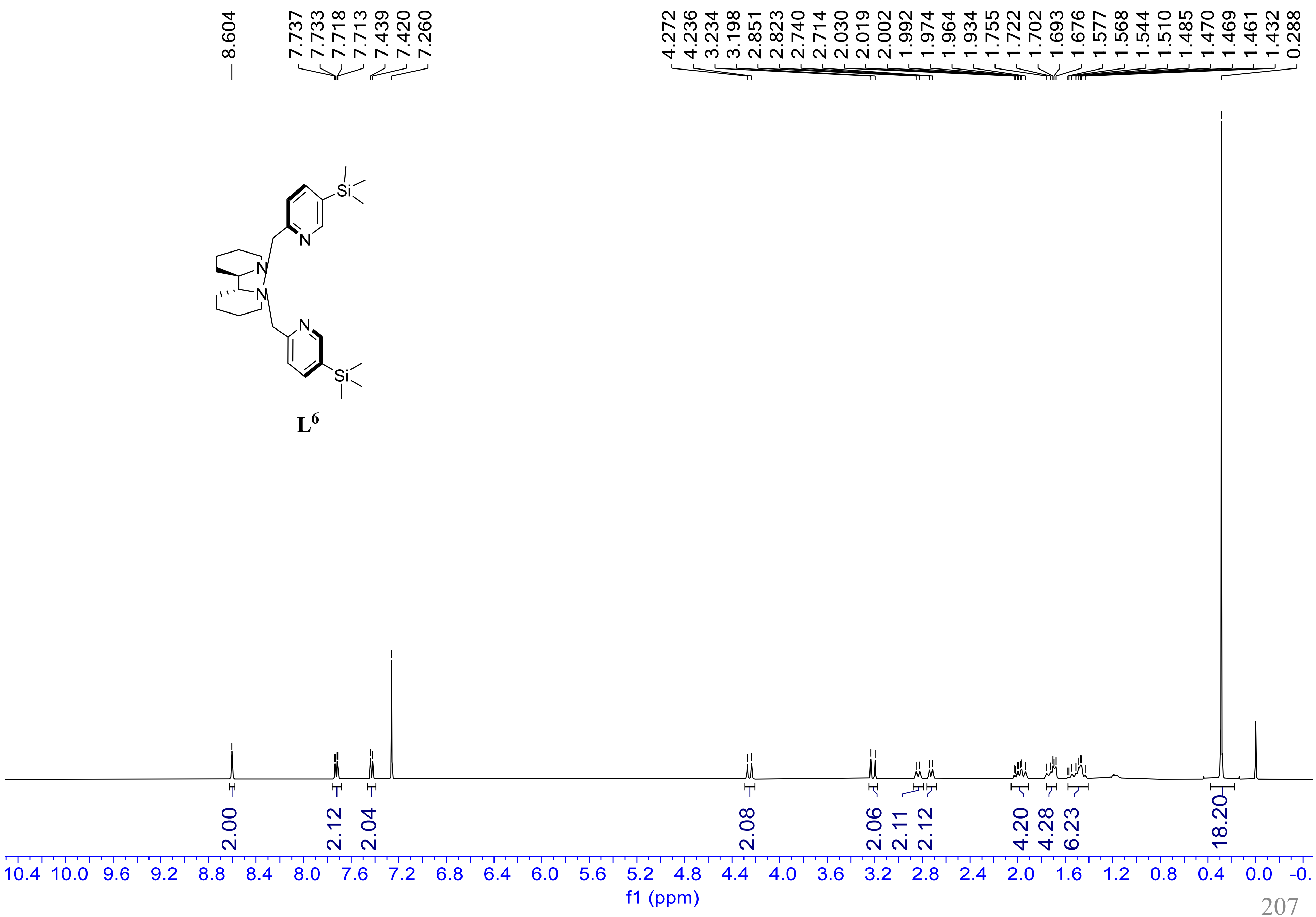

L[6]

^{13}C NMR 101 MHz, $CDCl_3$

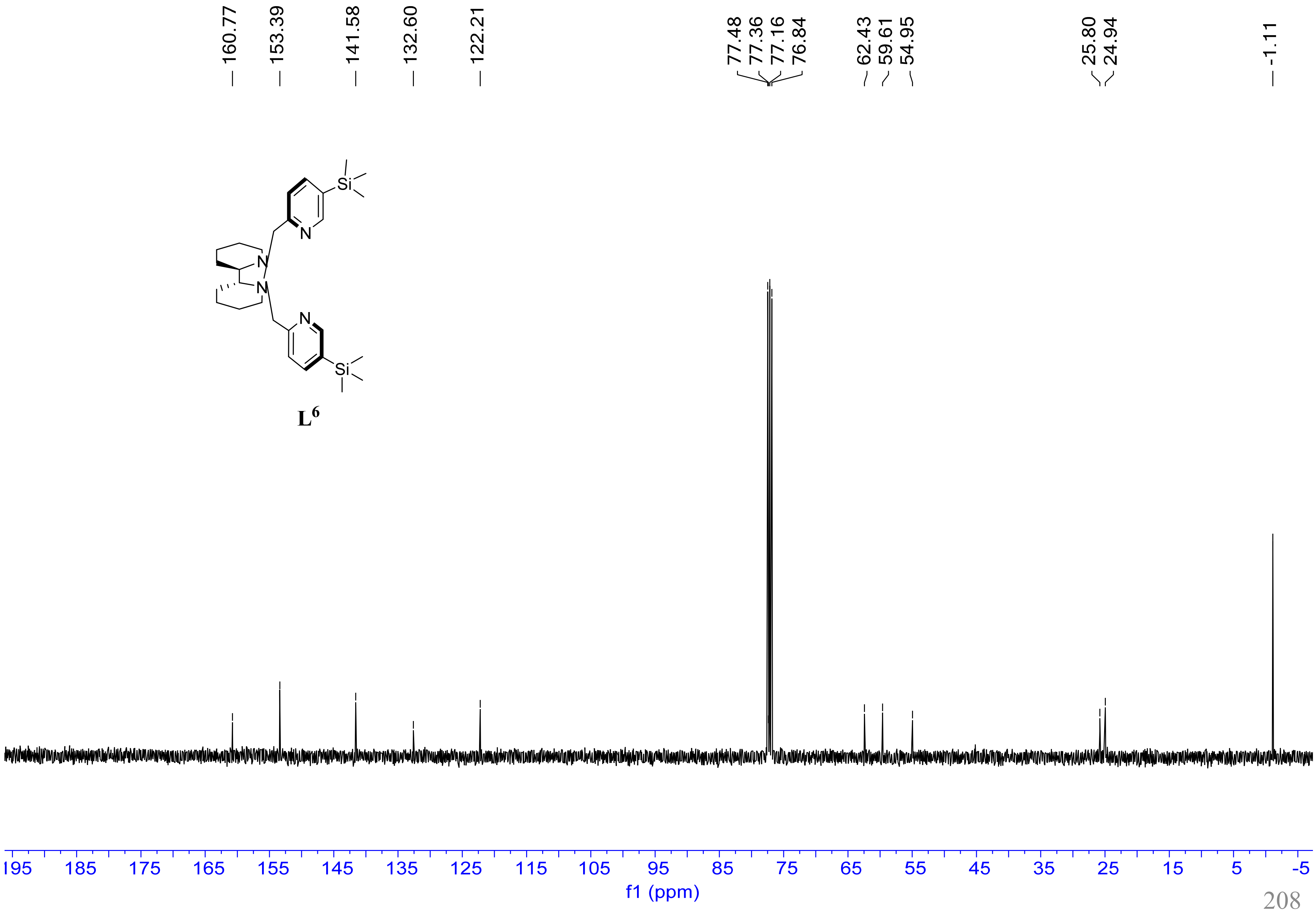

^{1}H NMR, 400 MHz, $CDCl_3$

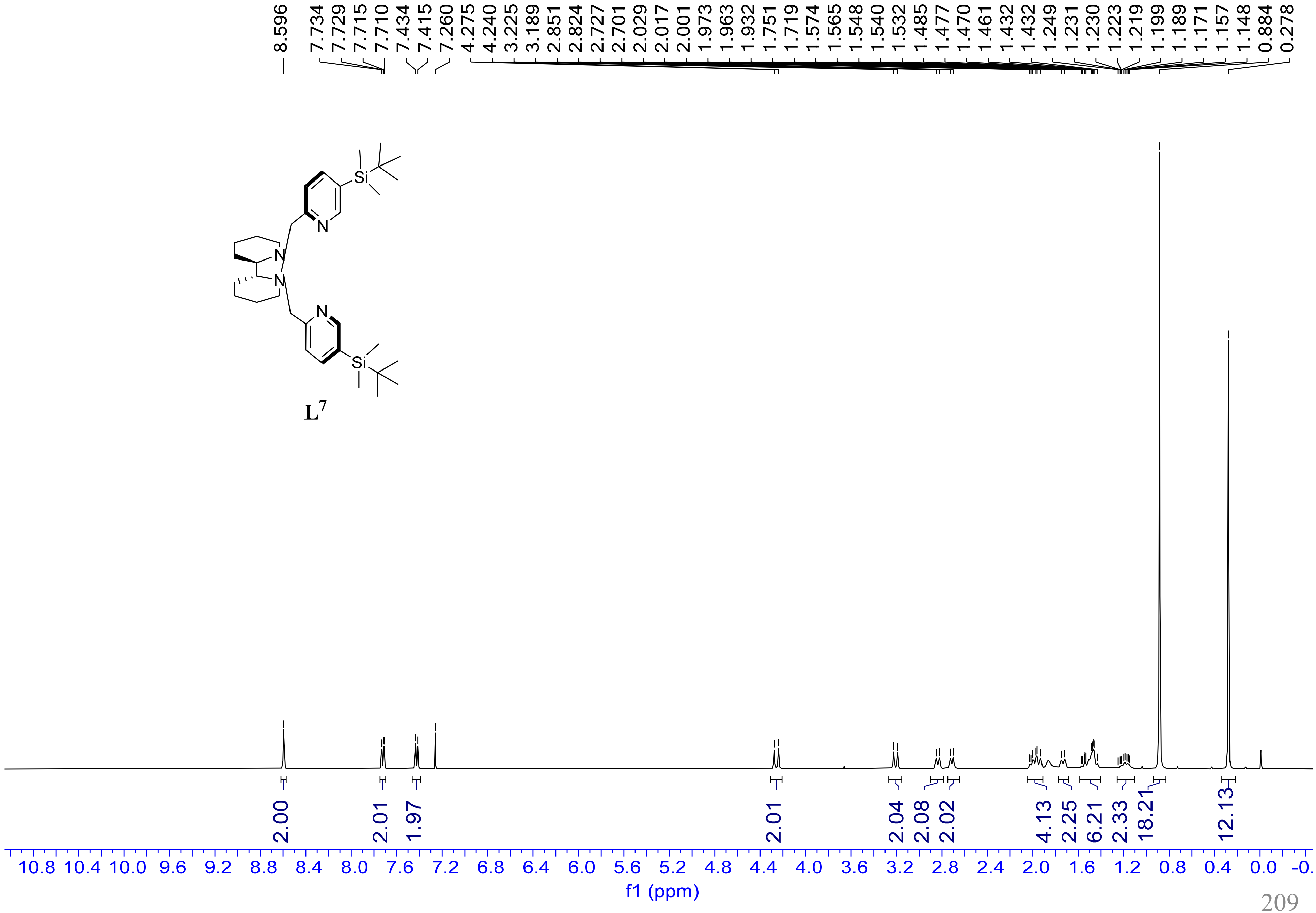

^{13}C NMR 101 MHz, $CDCl_3$

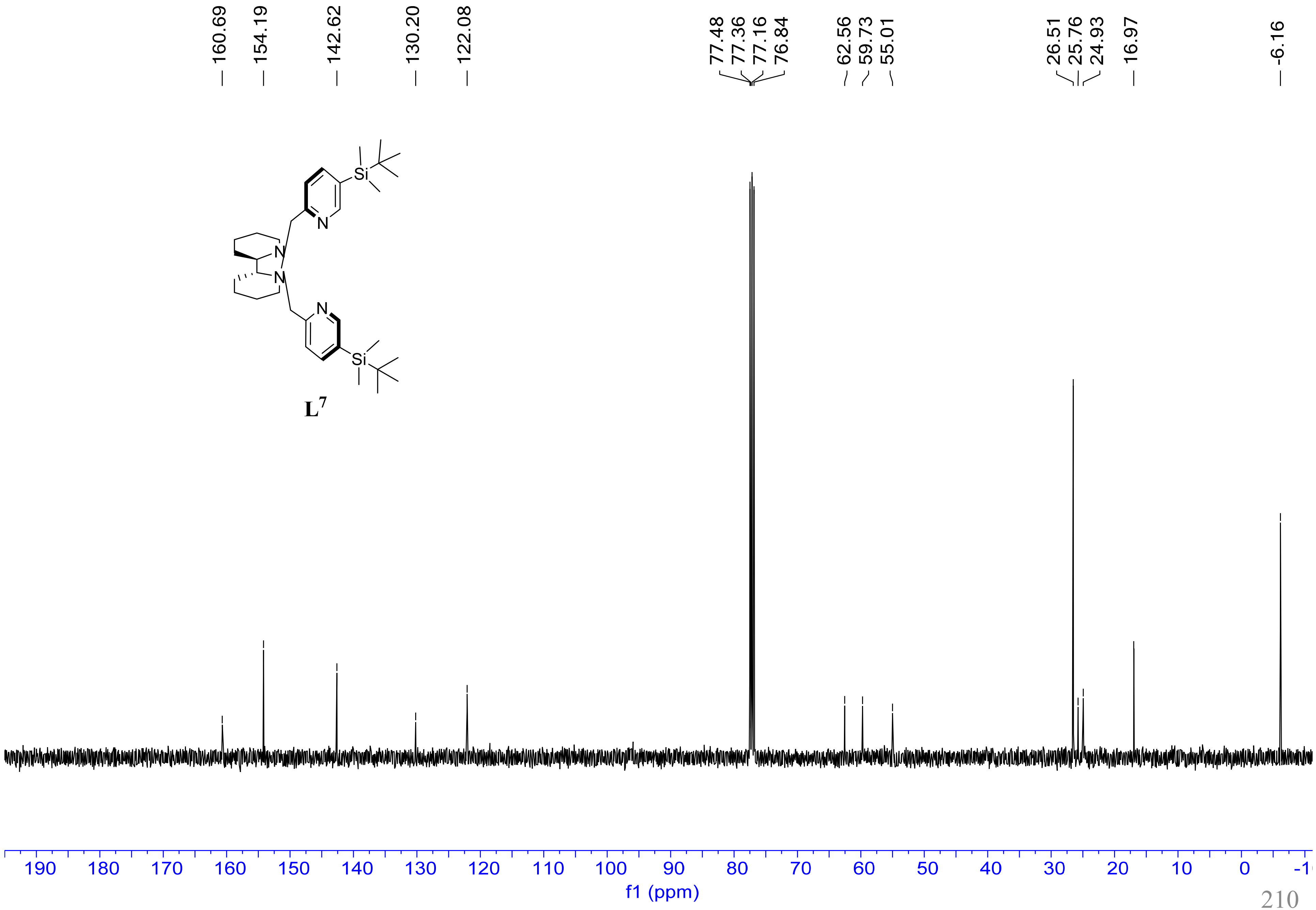

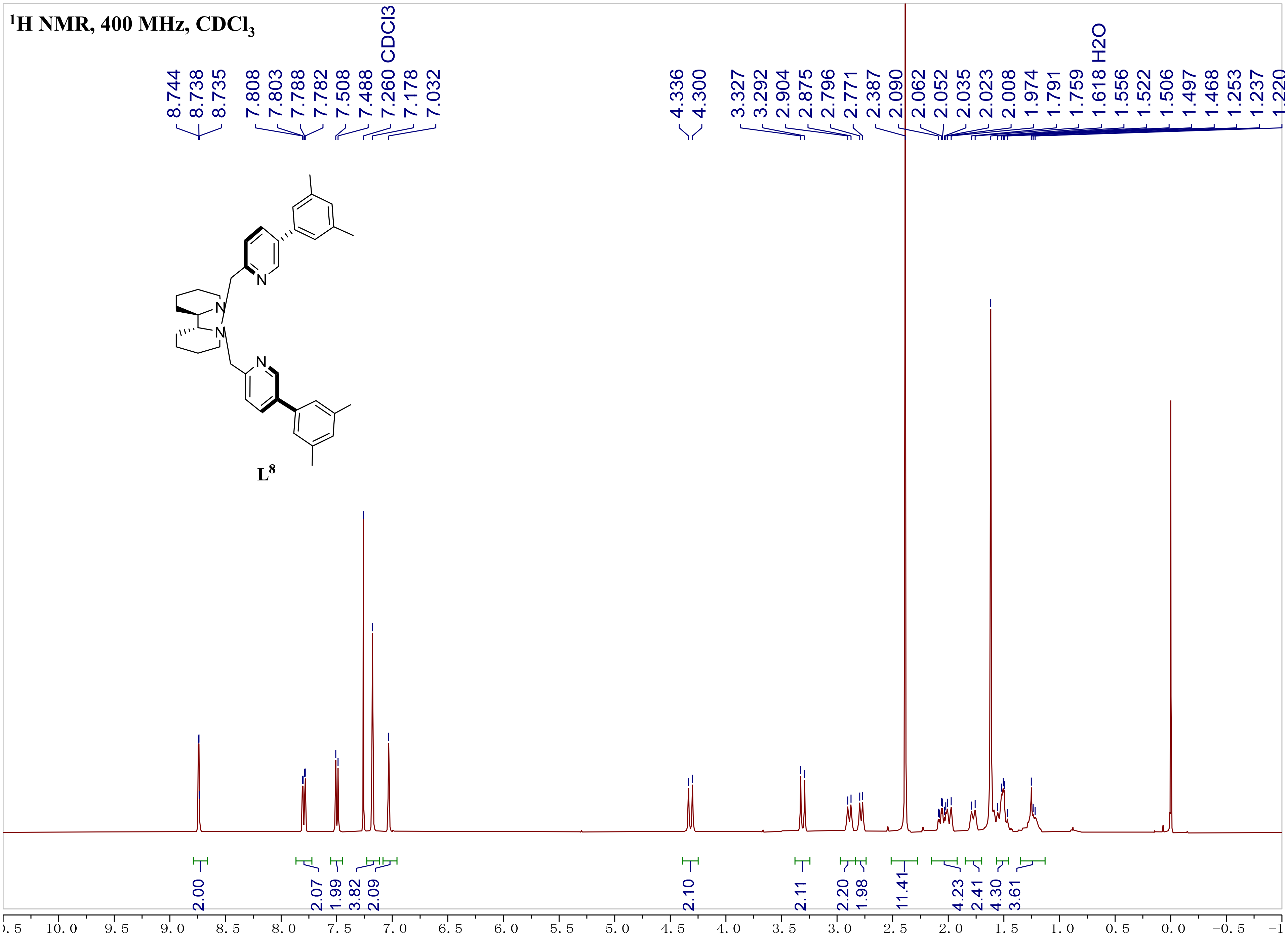

¹H NMR, 400 MHz, CDCl3
8.744 8.738 8.735 7.808 7.803 7.788 7.782 7.508 7.488 7.260 CDCl3 7.178 7.032
4.336 4.300 3.327 3.292 2.904 2.875 2.796 2.771 2.387 2.090 2.062 2.052 2.035 2.023 2.008 1.974 1.791 1.759 1.618 H2O 1.556 1.522 1.506 1.497 1.468 1.253 1.237 1.220
L8
2.00 2.07 1.99 3.82 2.09 2.10 2.11 2.20 1.98 11.41 4.23 2.41 4.30 3.61
10.0 9.5 9.0 8.5 8.0 7.5 7.0 6.5 6.0 5.5 5.0 4.5 4.0 3.5 3.0 2.5 2.0 1.5 1.0 0.5 0.0 −0.5
f1 (ppm)

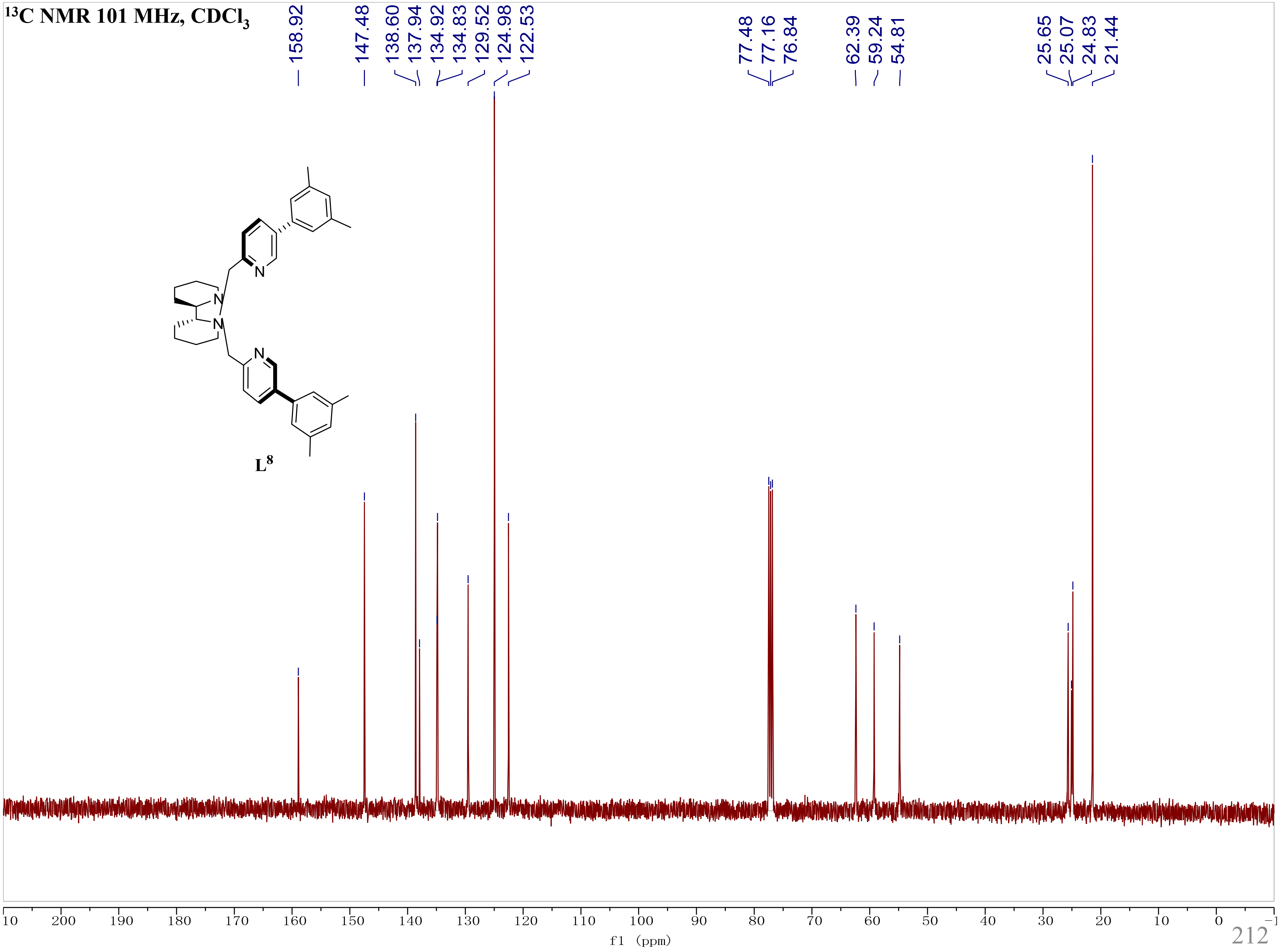

13C NMR 101 MHz, CDCl3
158.92
147.48
138.60
137.94
134.92
134.83
129.52
124.98
122.53
77.48
77.16
76.84
62.39
59.24
54.81
25.65
25.07
24.83
21.44
N
N
N
N
L8
10 200 190 180 170 160 150 140 130 120 110 100 90 80 70 60 50 40 30 20 10 0 -1
f1 (ppm)

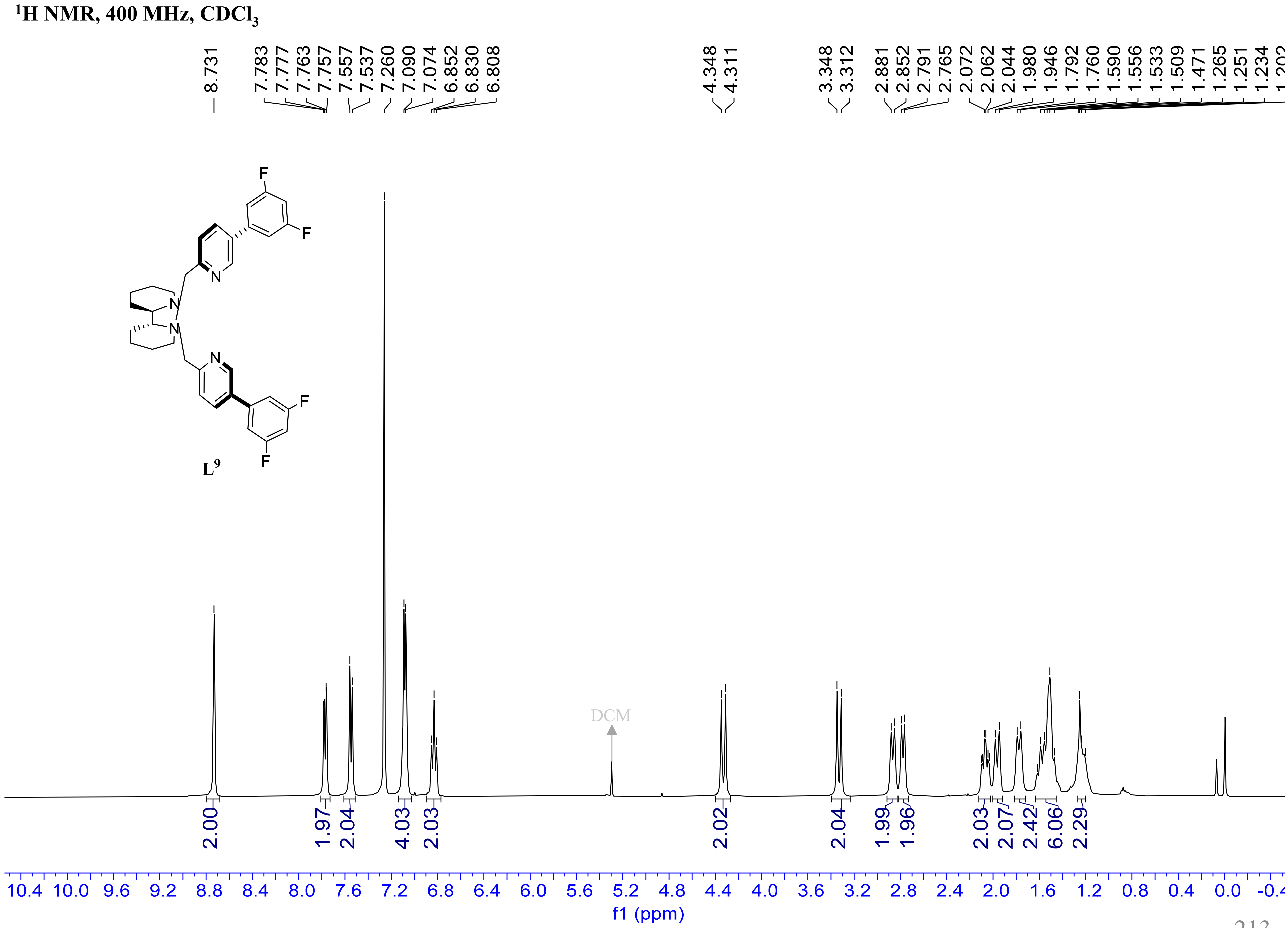

1H NMR, 400 MHz, CDCl3
8.731
7.783
7.777
7.763
7.757
7.557
7.537
7.260
7.090
7.074
6.852
6.830
6.808
4.348
4.311
3.348
3.312
2.881
2.852
2.791
2.765
2.072
2.062
2.044
1.980
1.946
1.792
1.760
1.590
1.556
1.533
1.509
1.471
1.265
1.251
1.234
F
N
L9
DCM
2.00
1.97
2.04
4.03
2.03
2.02
2.04
1.99
1.96
2.03
2.07
2.42
6.06
2.29
f1 (ppm)

^{13}C NMR 101 MHz, $CDCl_3$

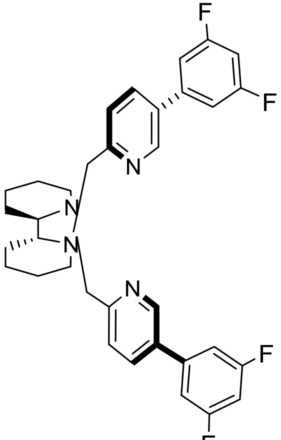

L[9]

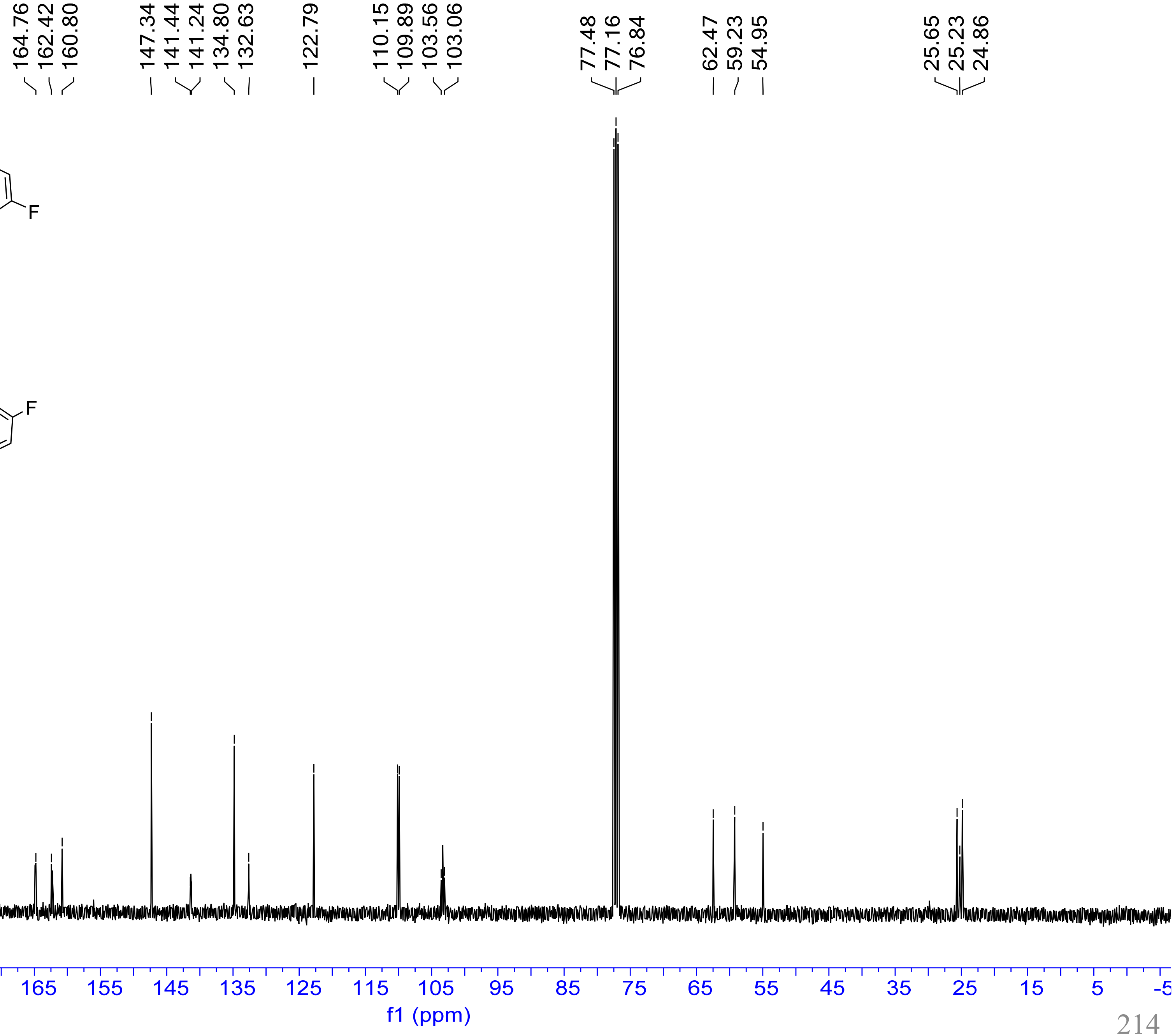

^{13}F NMR 376 MHz, $CDCl_3$

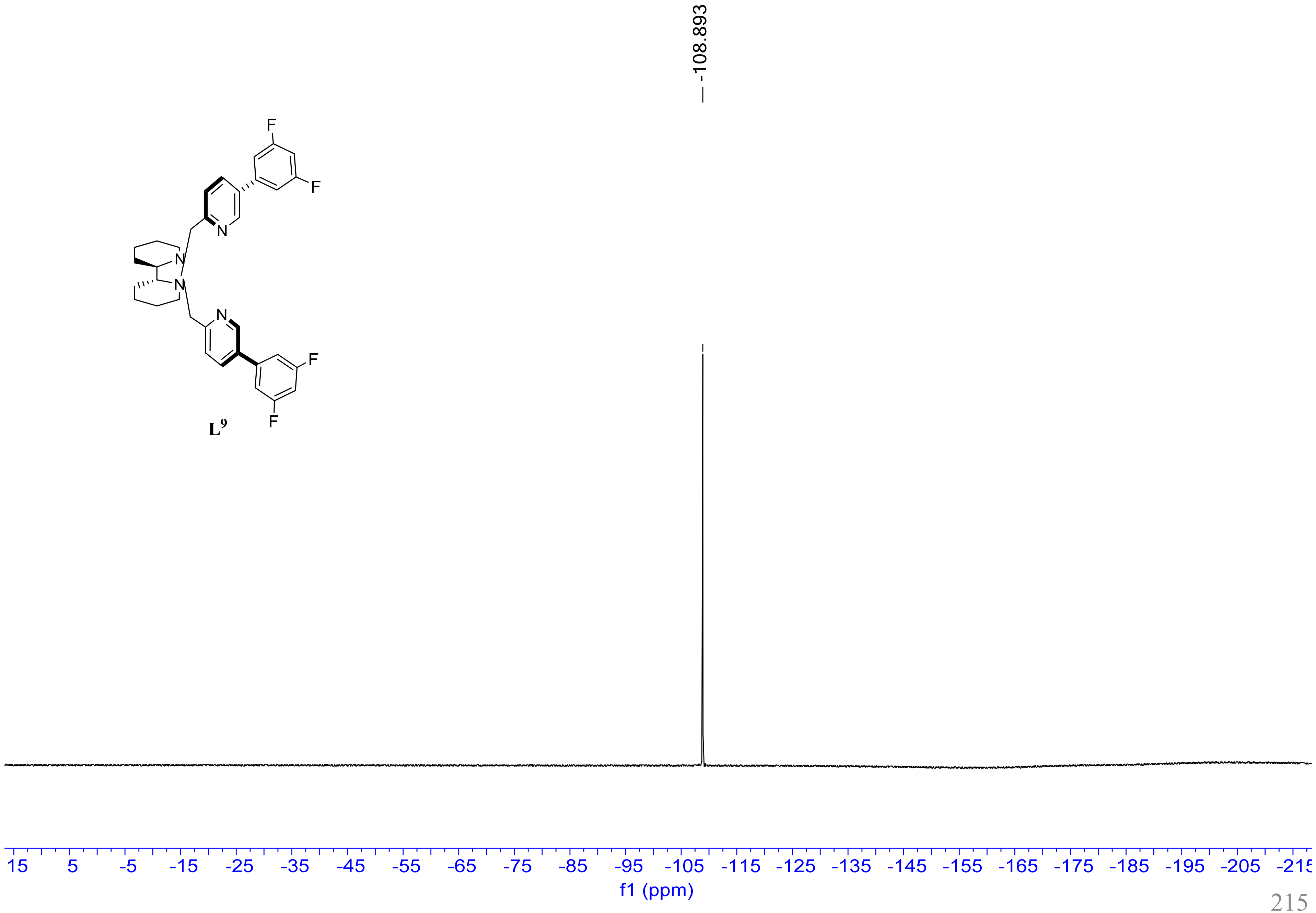

L^{9}

^{1}H NMR, 400 MHz, $CDCl_3$

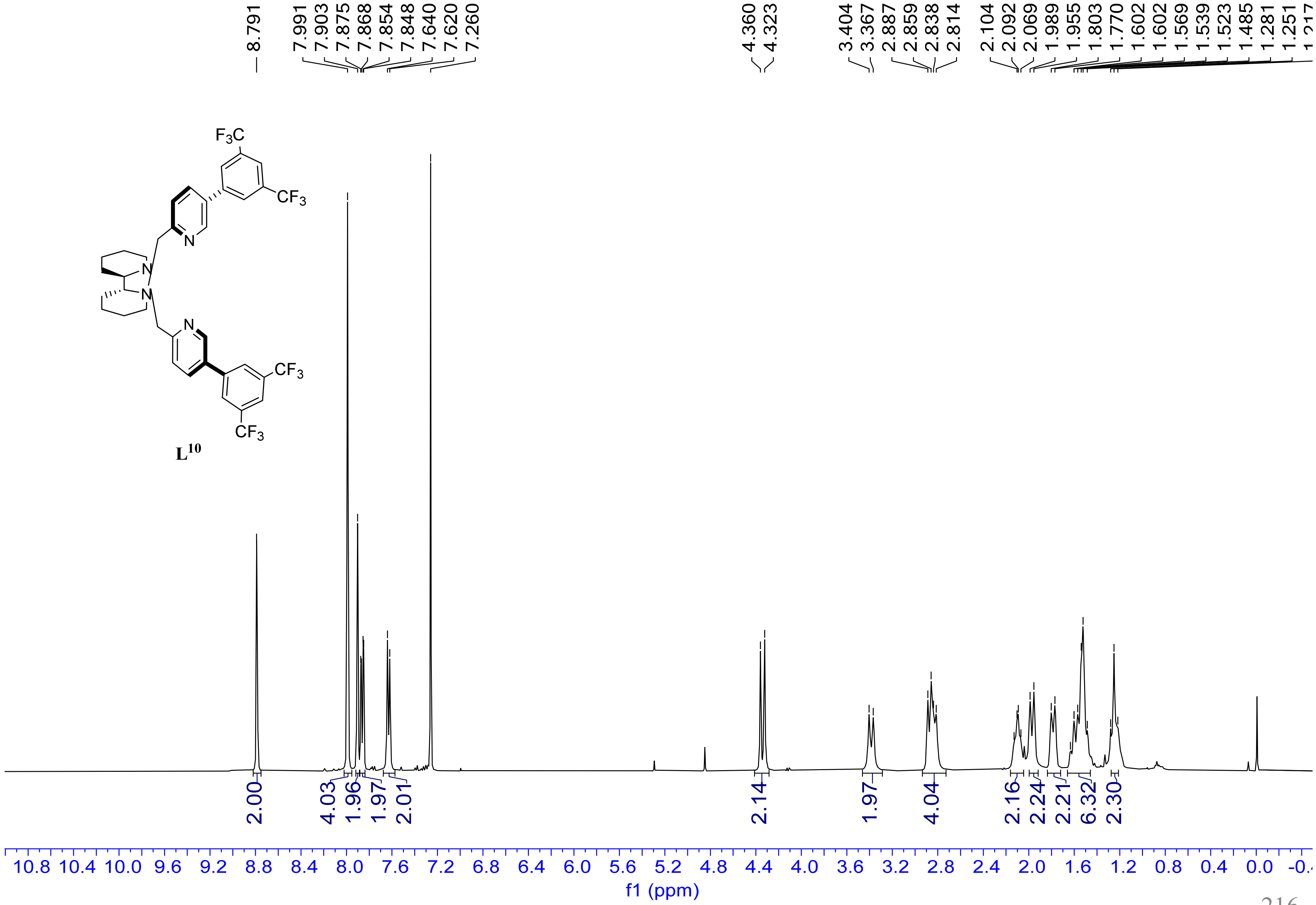

^{13}C NMR 101 MHz, $CDCl_3$

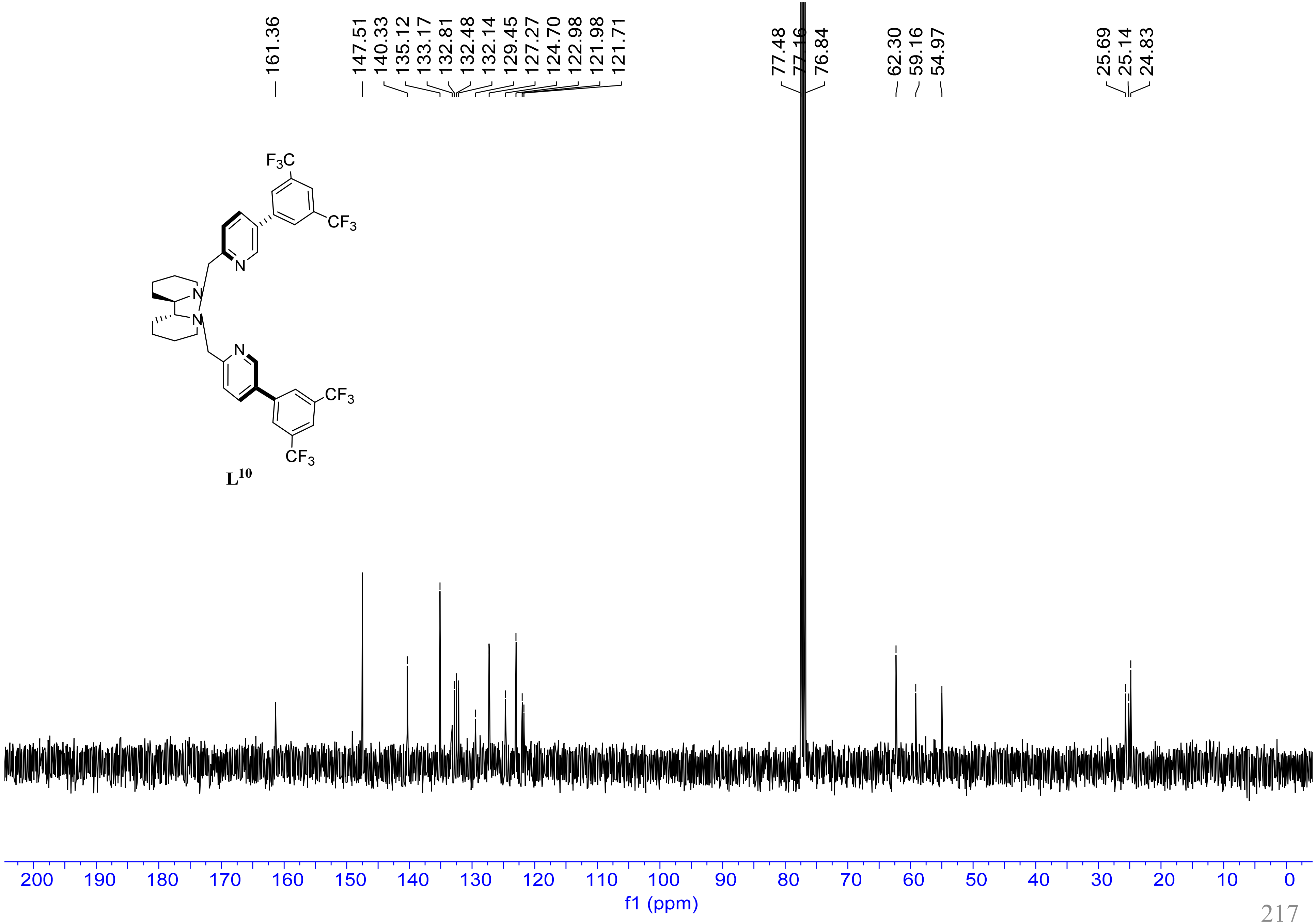

^{13}F NMR 376 MHz, $CDCl_3$

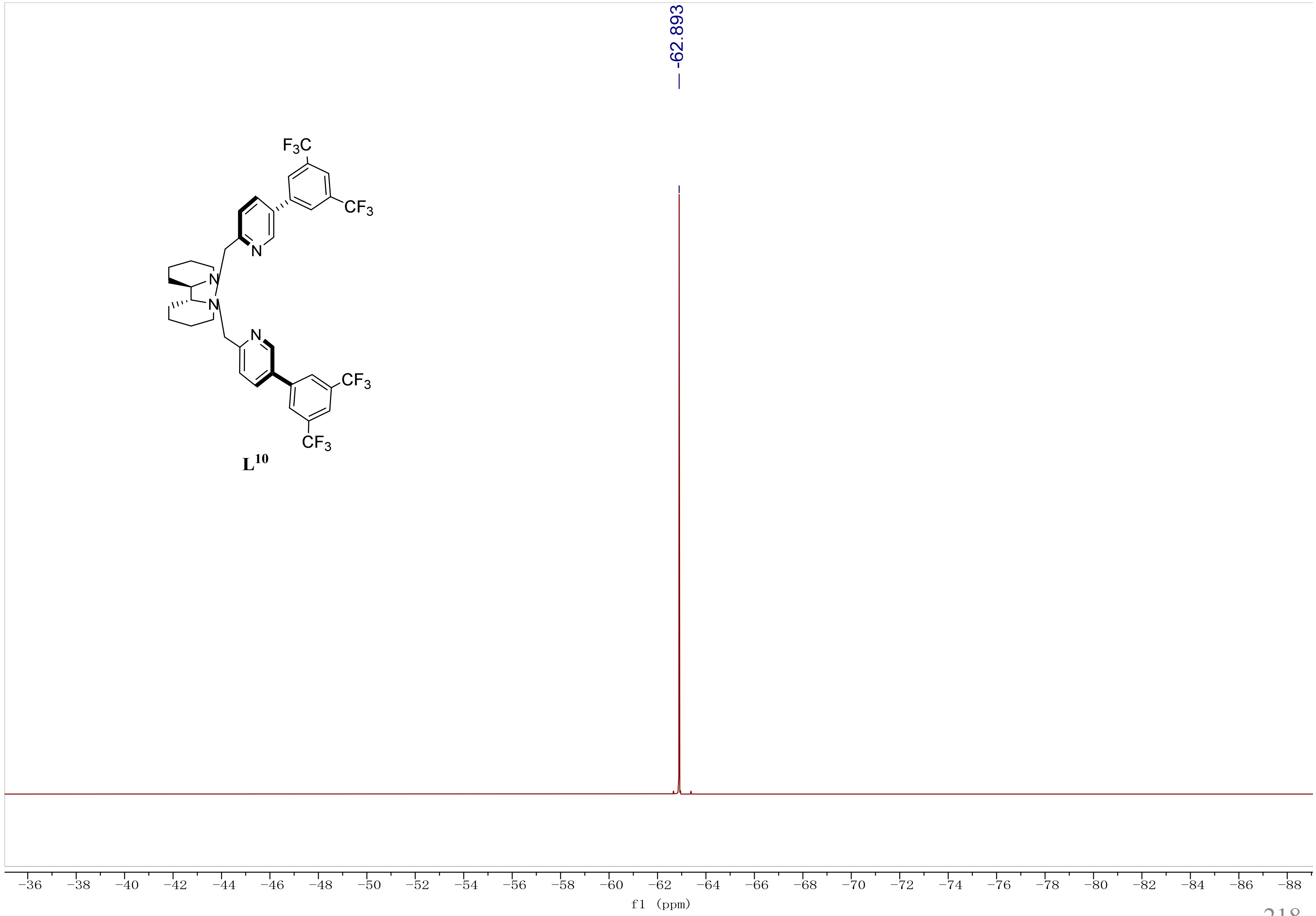

^{1}H NMR, 400 MHz, $CDCl_3$

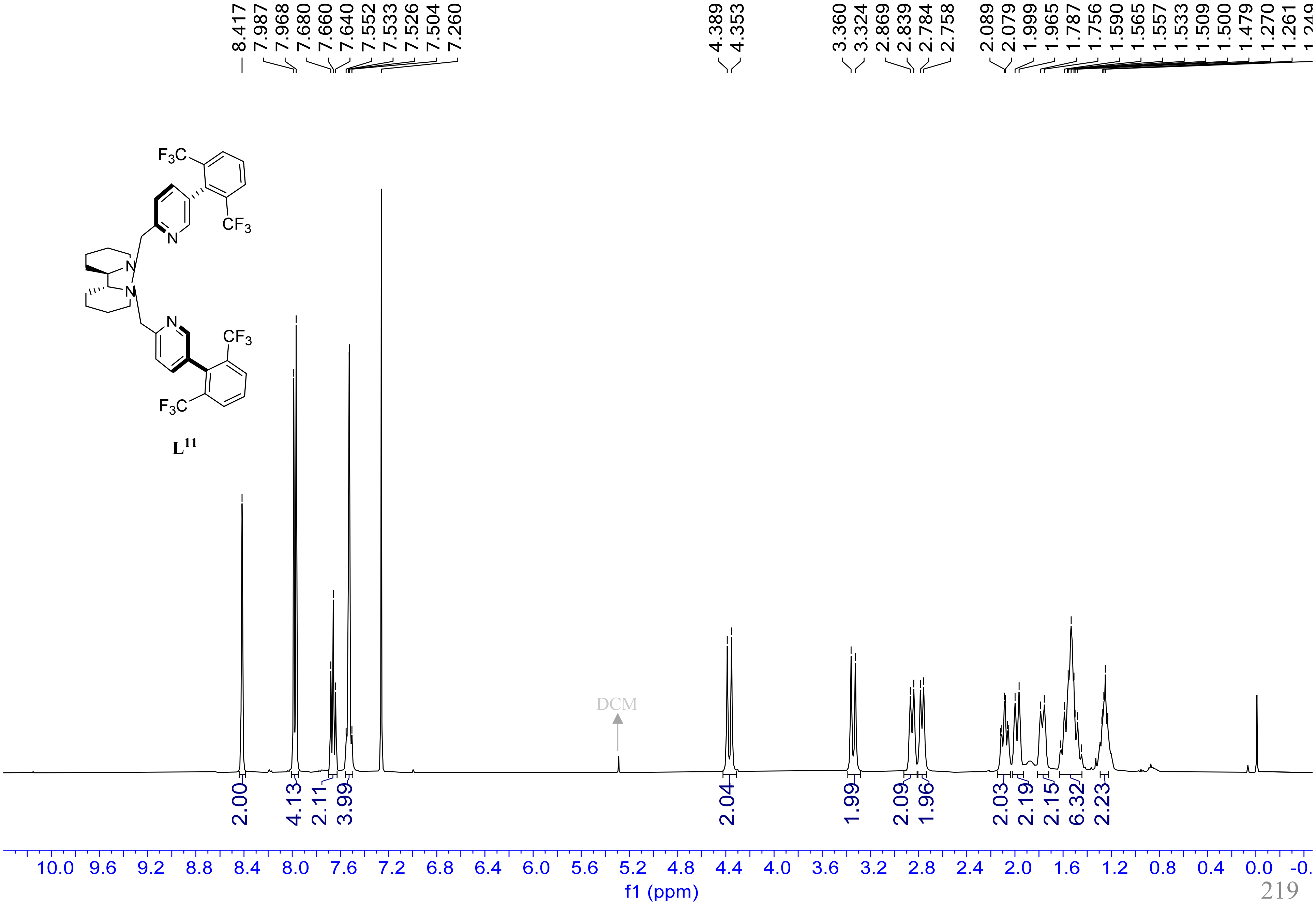

^{13}C NMR 101 MHz, $CDCl_3$

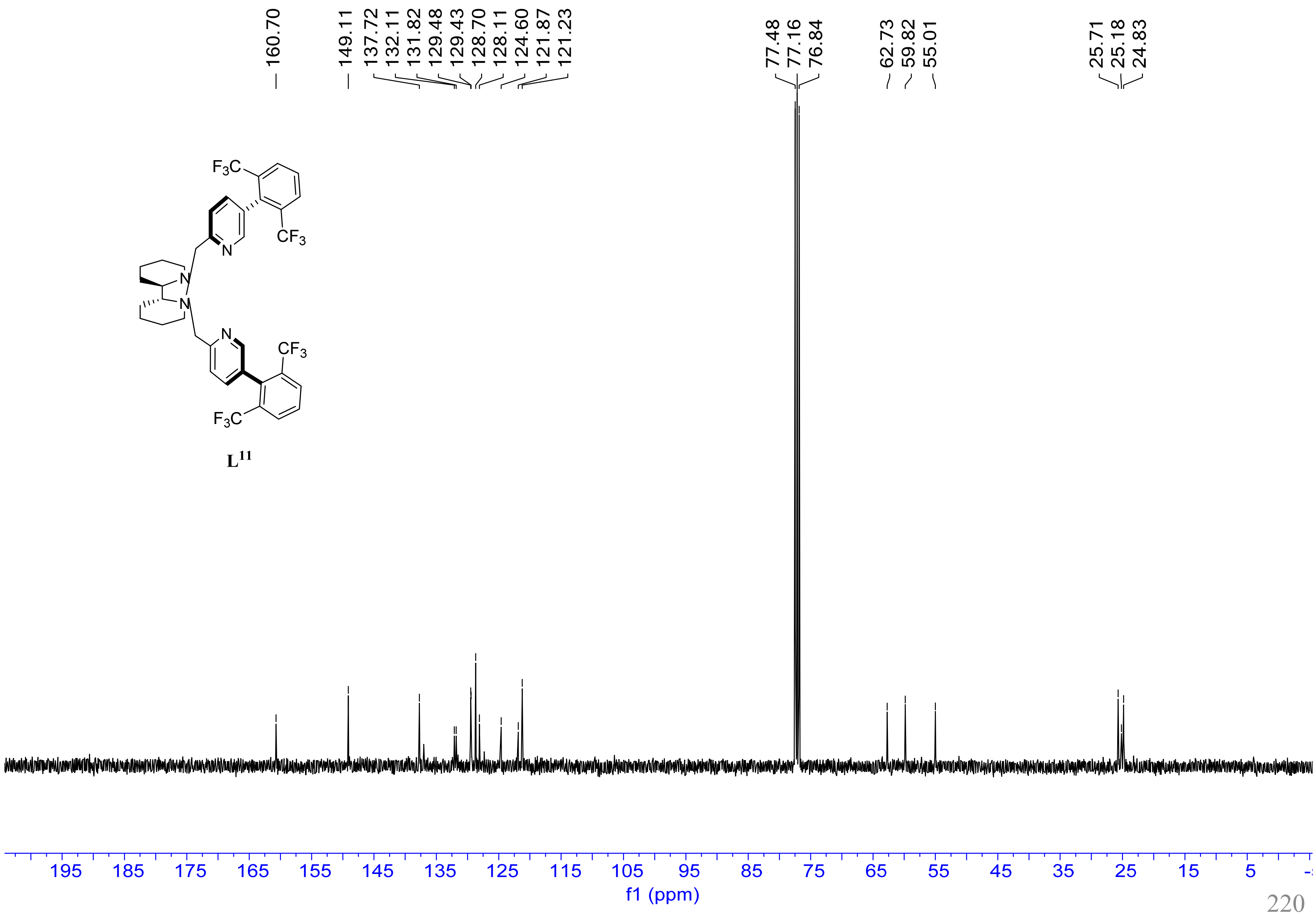

L[11]

^{13}F NMR 376 MHz, $CDCl_3$

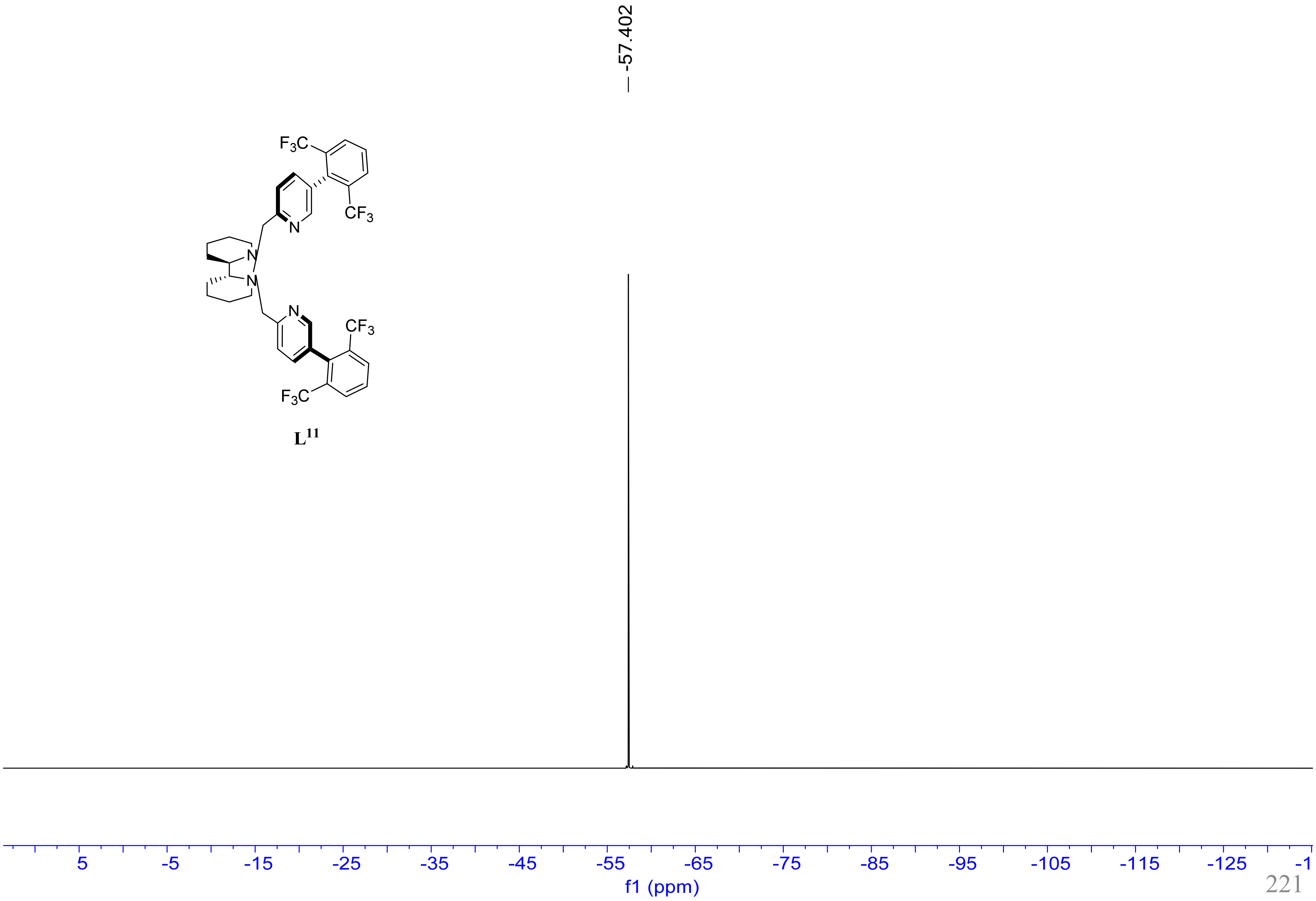

^1H NMR, 400 MHz, $CDCl_3$

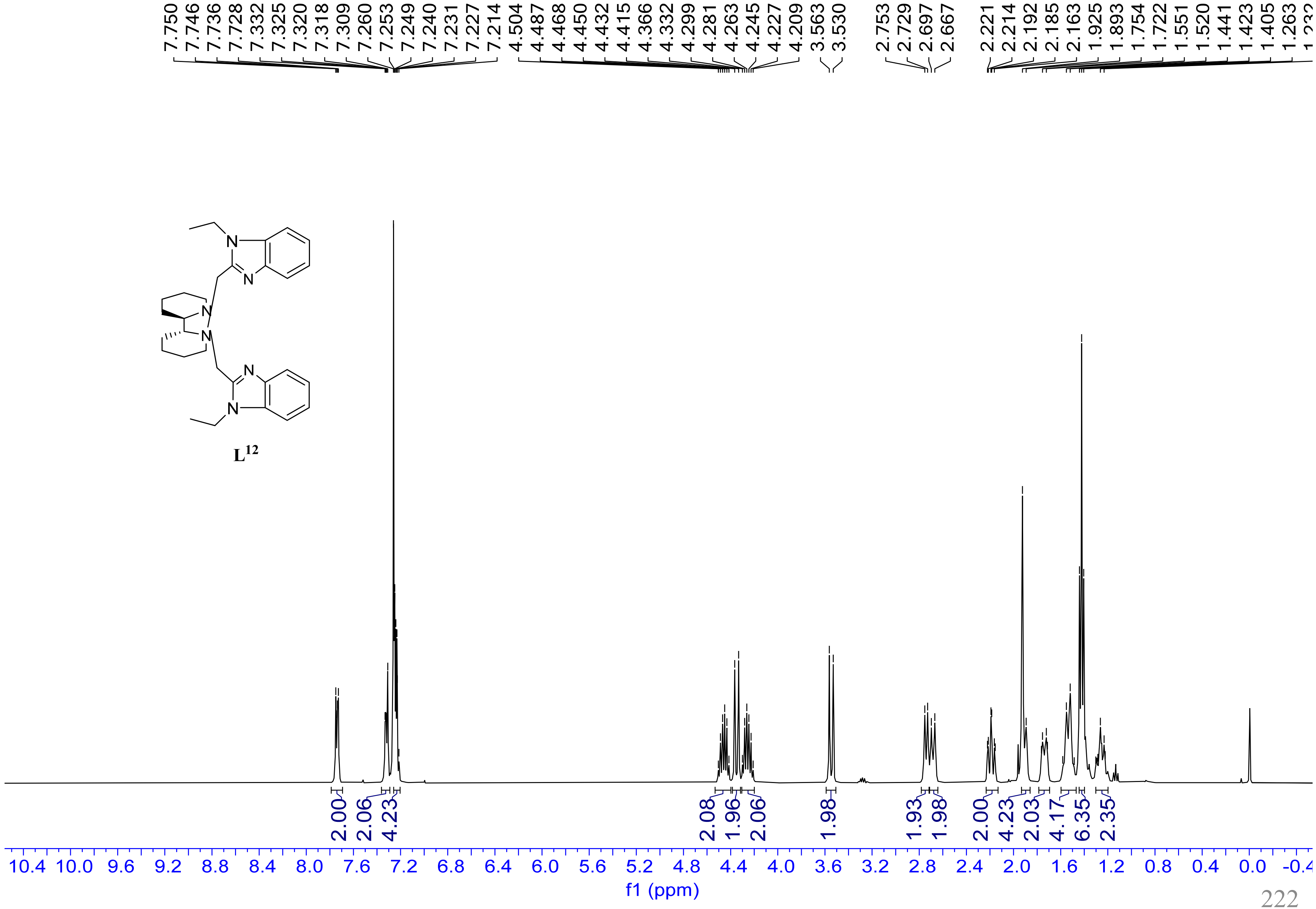

^{13}C NMR 101 MHz, $CDCl_3$

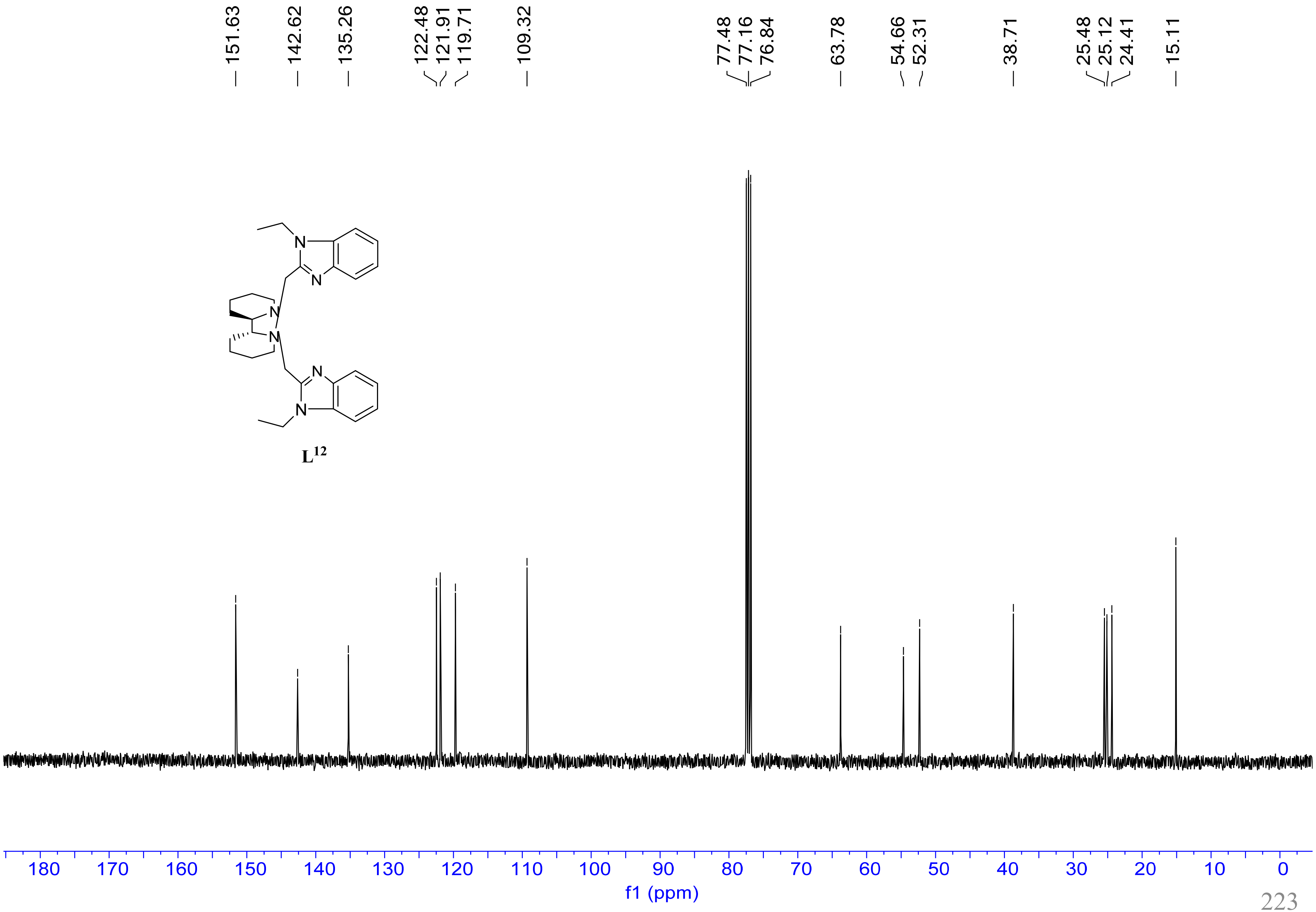

^{1}H NMR, 400 MHz, $CDCl_3$

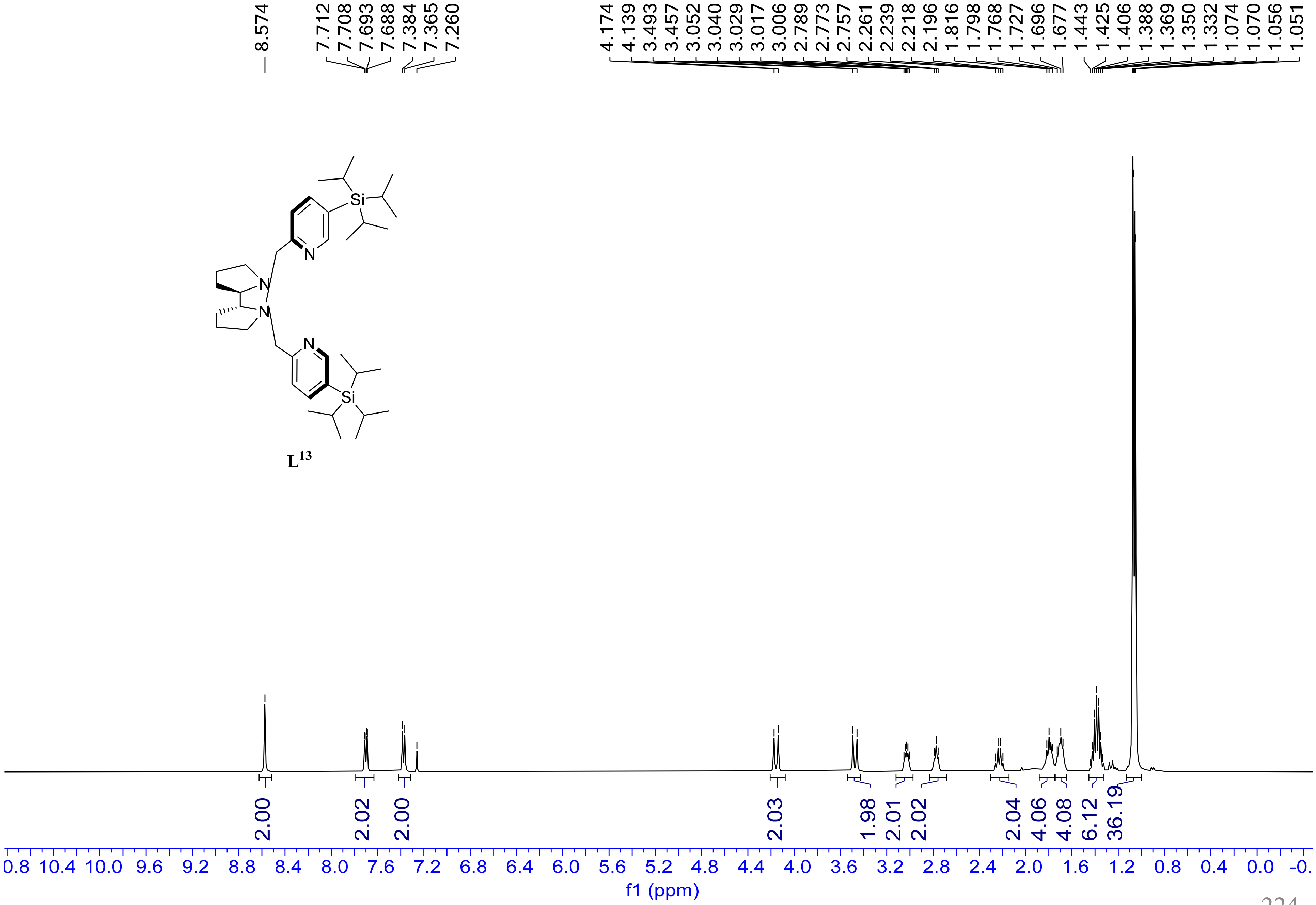

^{13}C NMR 101 MHz, $CDCl_3$

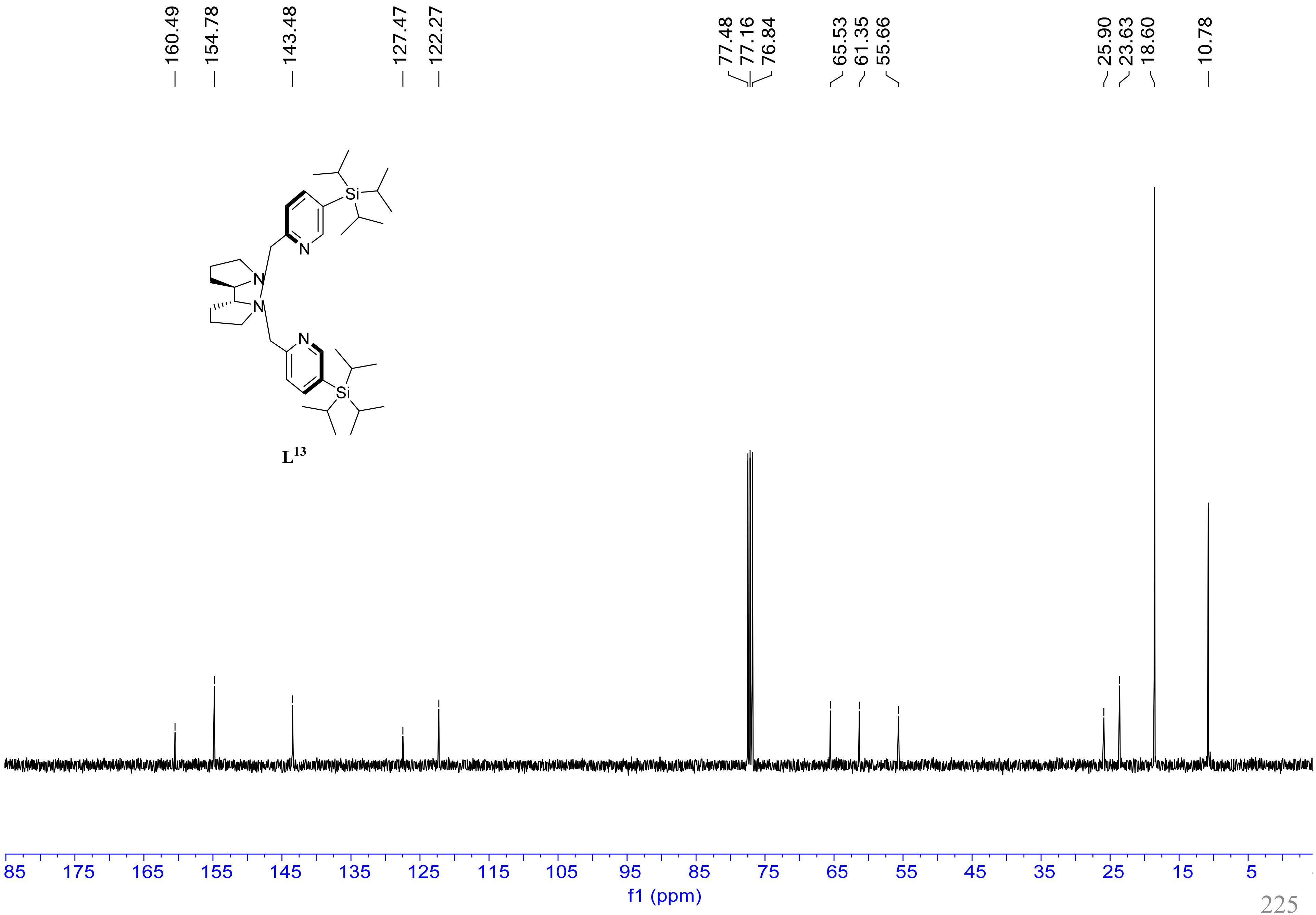

^{1}H NMR, 400 MHz, $CDCl_3$

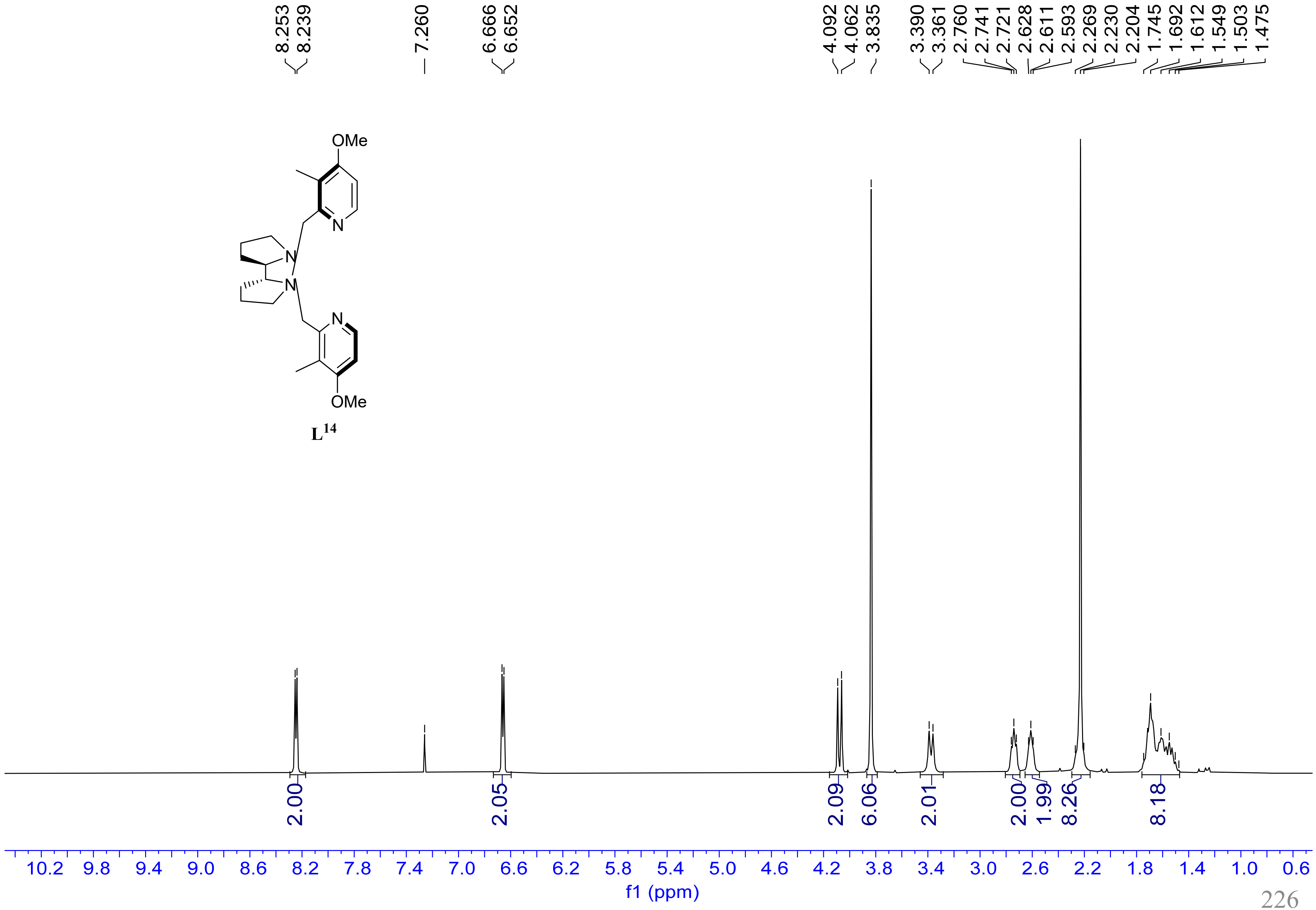

^{13}C NMR 101 MHz, $CDCl_3$

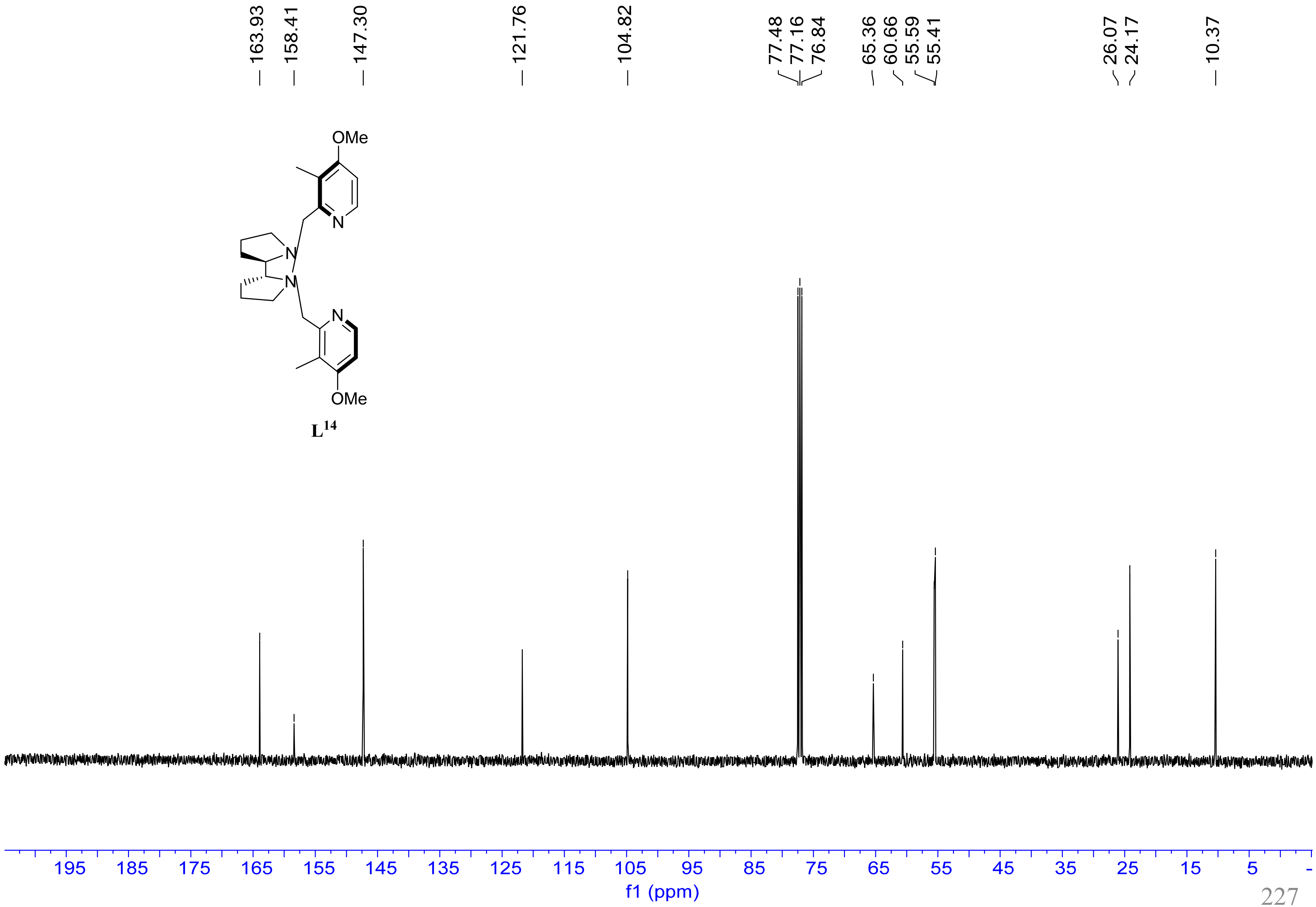

L[14]

^{1}H NMR, 400 MHz, $CDCl_3$

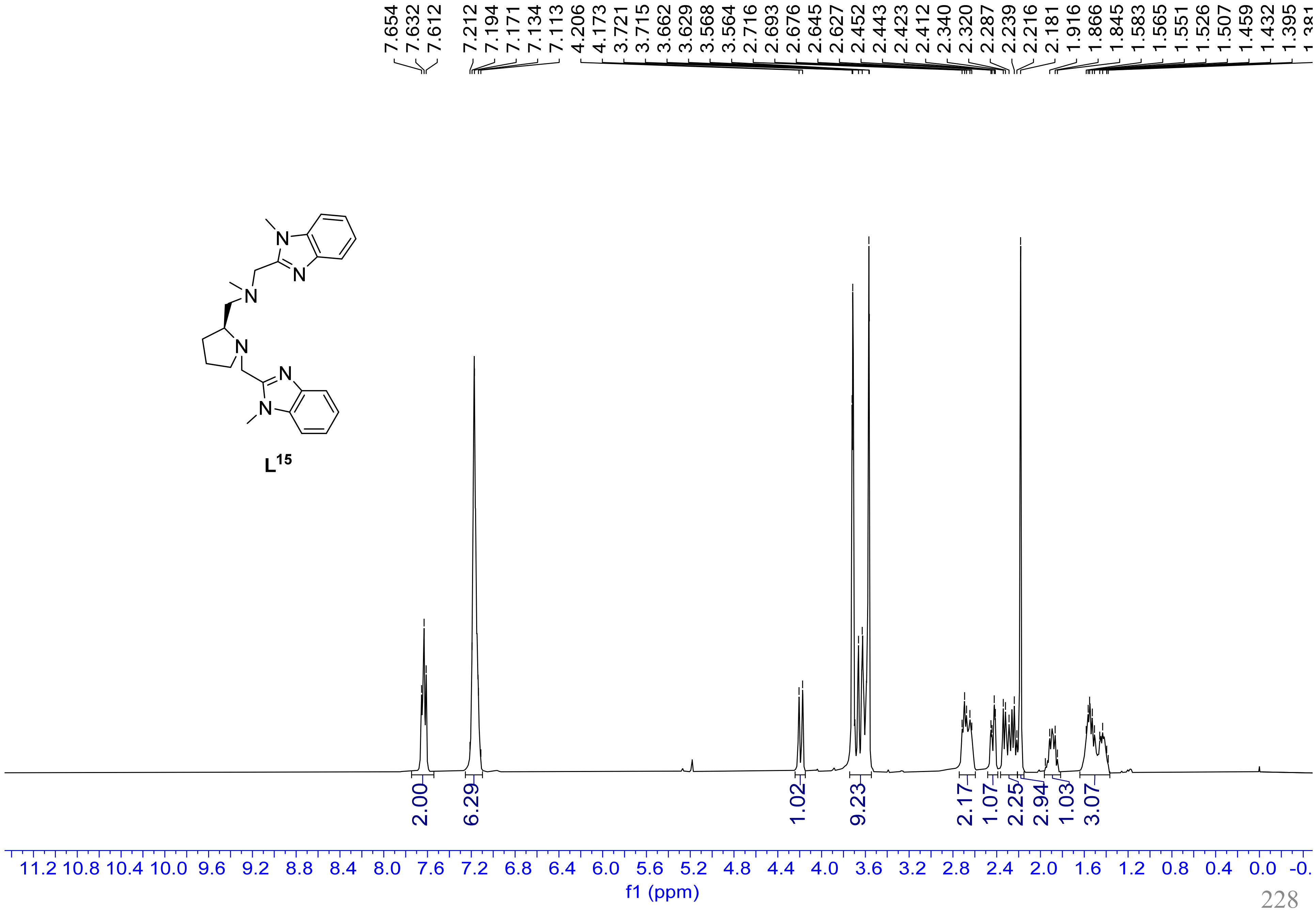

^{13}C NMR 101 MHz, $CDCl_3$

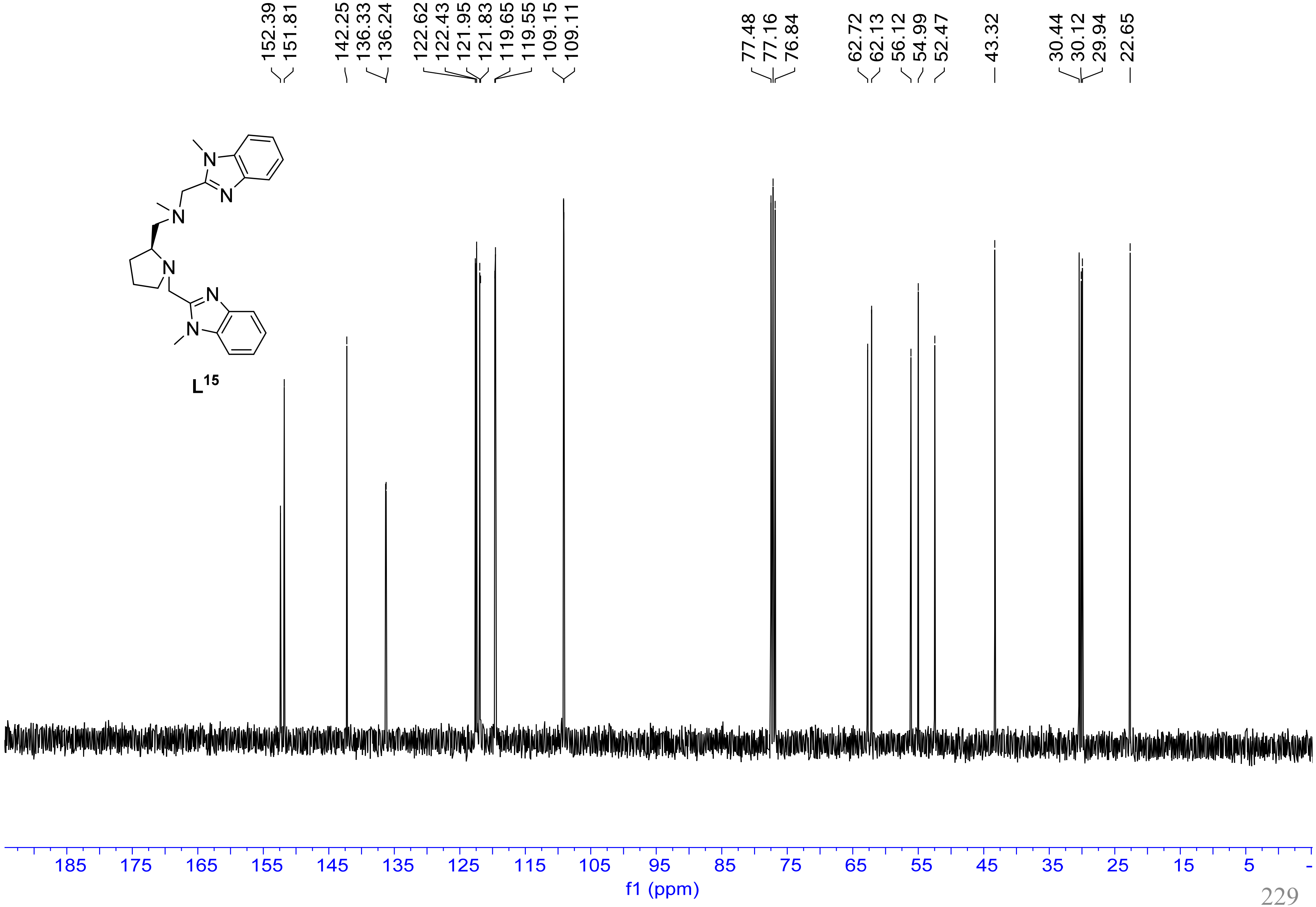

^{1}H NMR, 400 MHz, $CDCl_3$

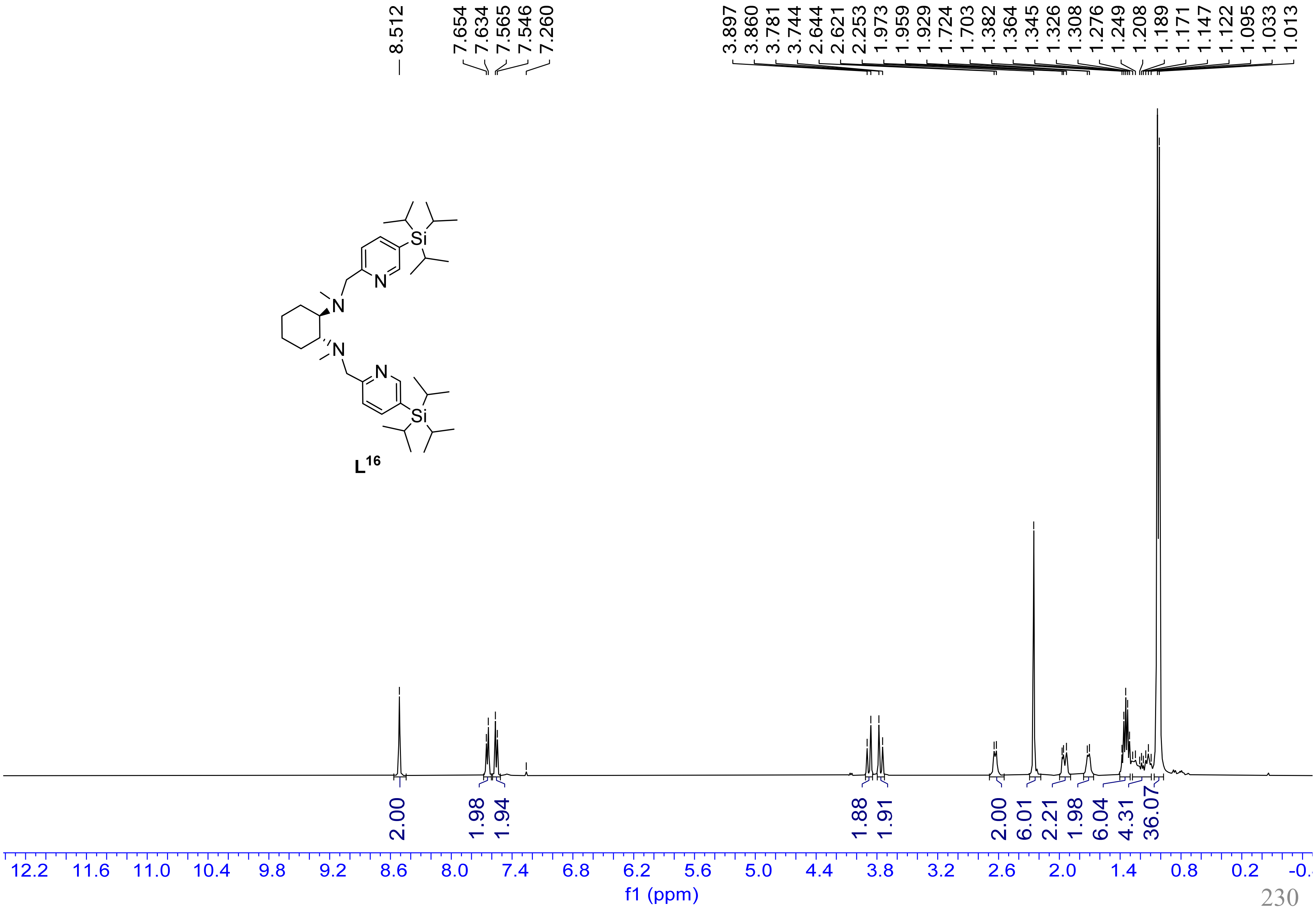

^{13}C NMR 101 MHz, $CDCl_3$

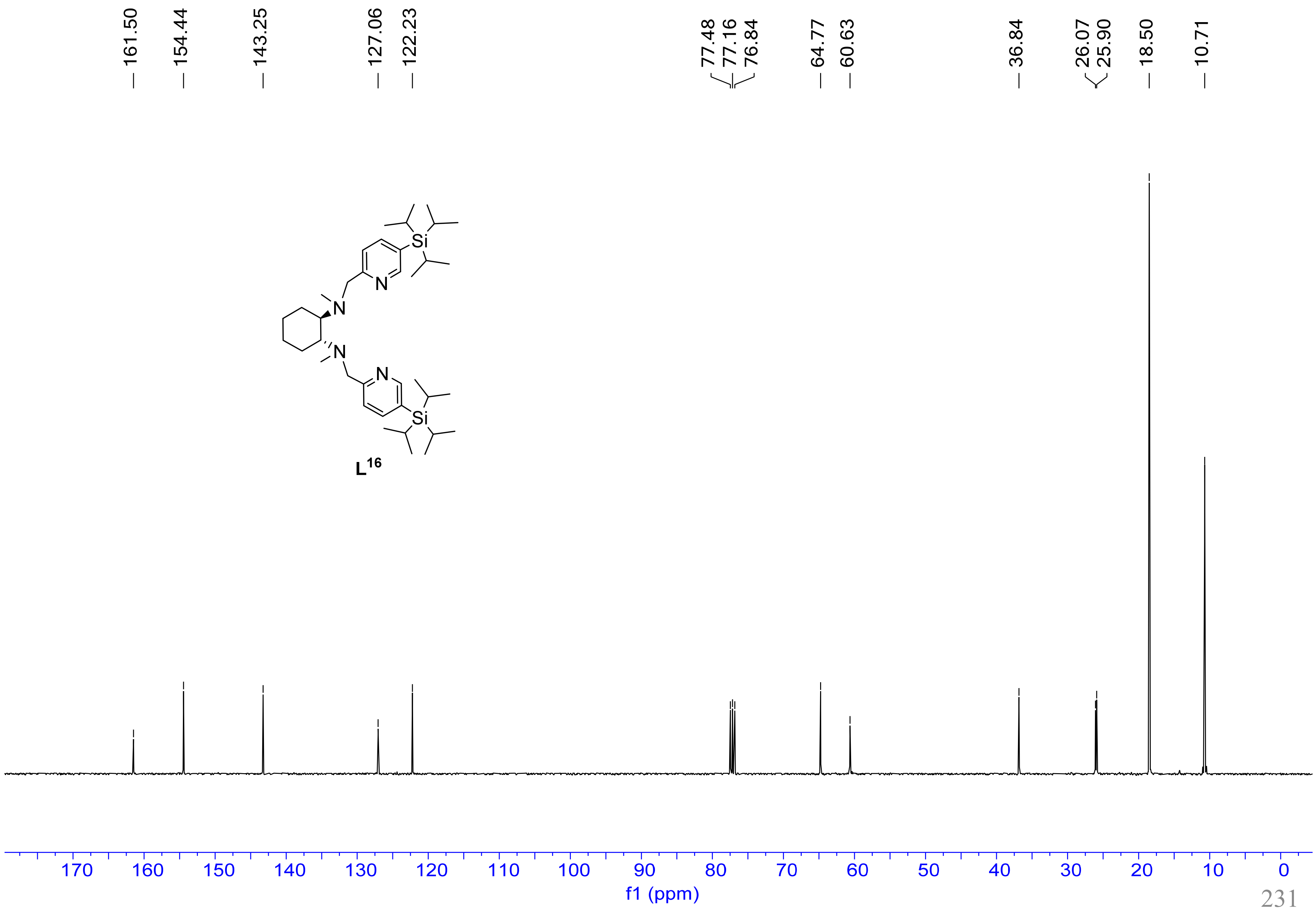

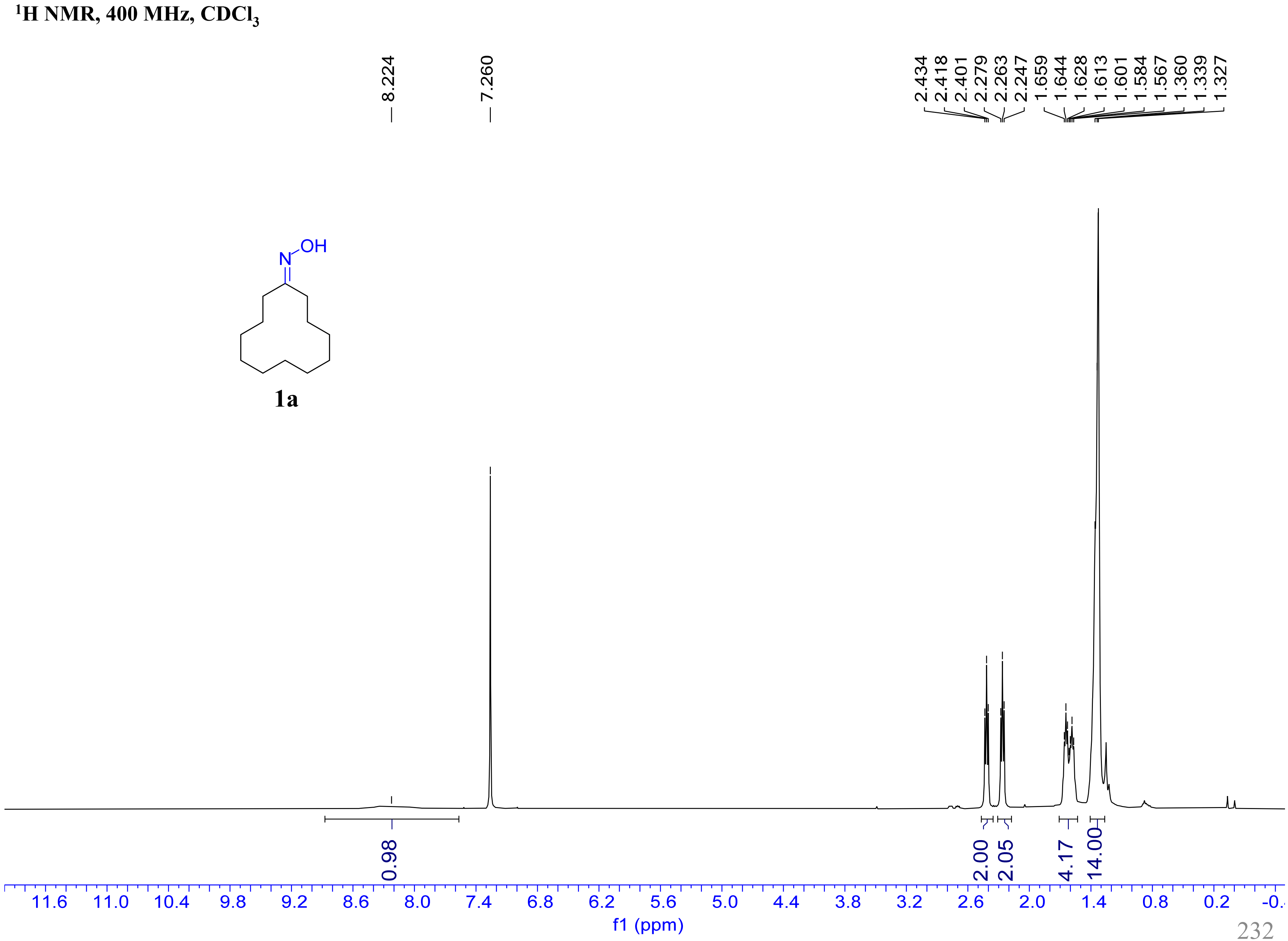
¹H NMR, 400 MHz, CDCl3
8.224
7.260
2.434
2.418
2.401
2.279
2.263
2.247
1.659
1.644
1.628
1.613
1.601
1.584
1.567
1.360
1.339
1.327
N
OH
1a
0.98
2.00
2.05
4.17
14.00
11.6
11.0
10.4
9.8
9.2
8.6
8.0
7.4
6.8
6.2
5.6
5.0
4.4
3.8
3.2
2.6
2.0
1.4
0.8
0.2
f1 (ppm)

^{13}C NMR 101 MHz, CDCl$_3$

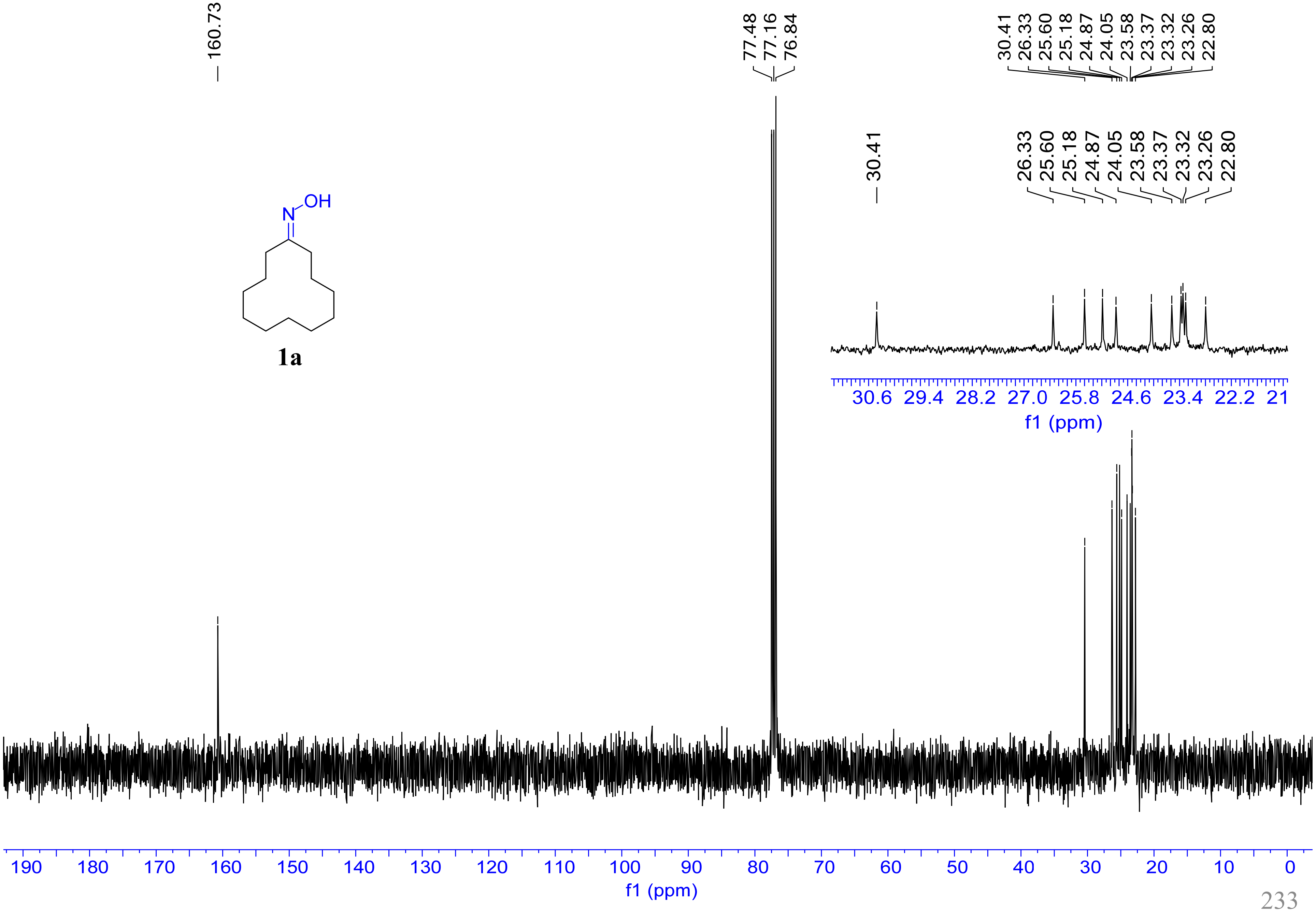

^{1}H NMR, 400 MHz, $CDCl_3$

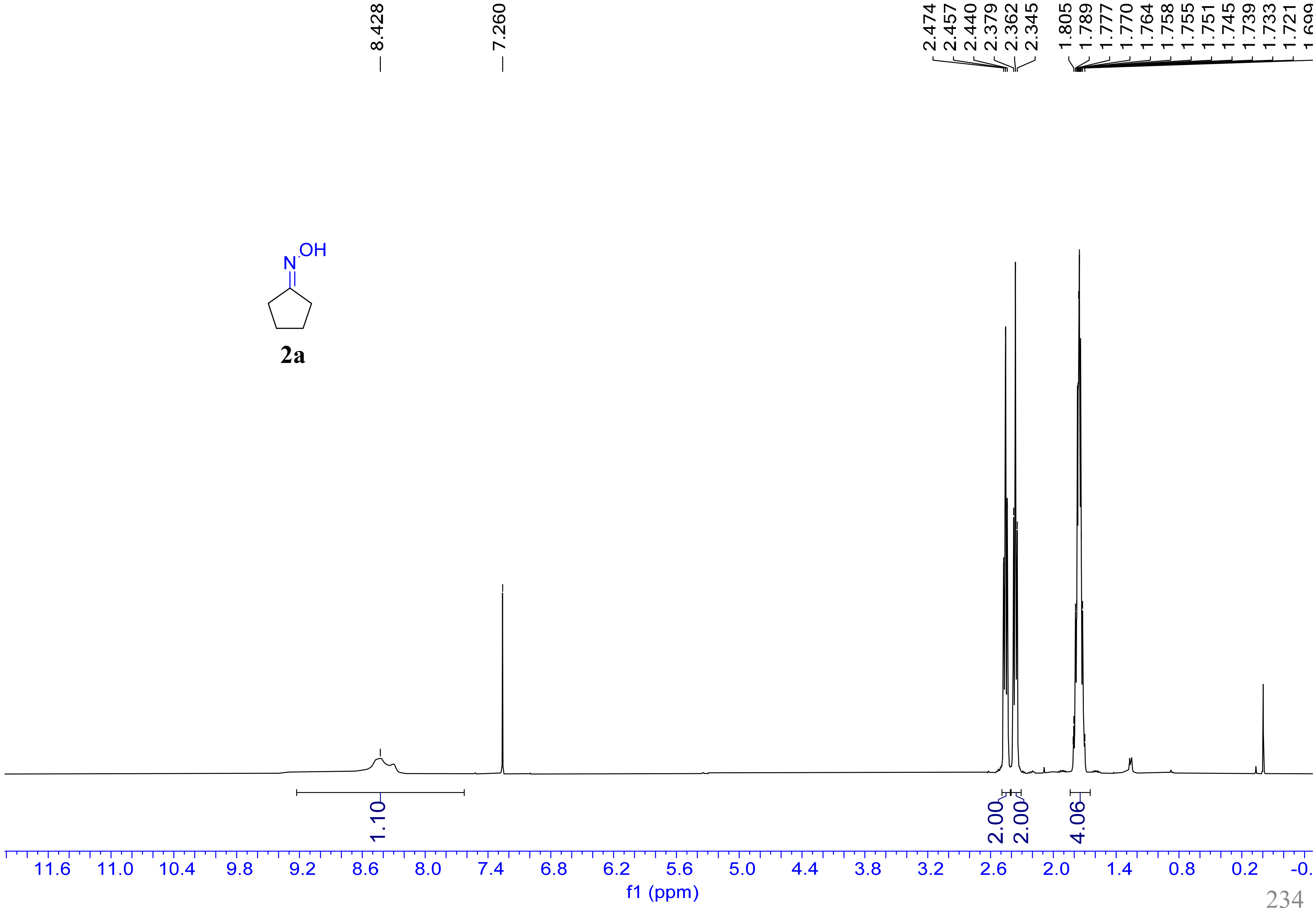

^{13}C NMR 101 MHz, $CDCl_3$

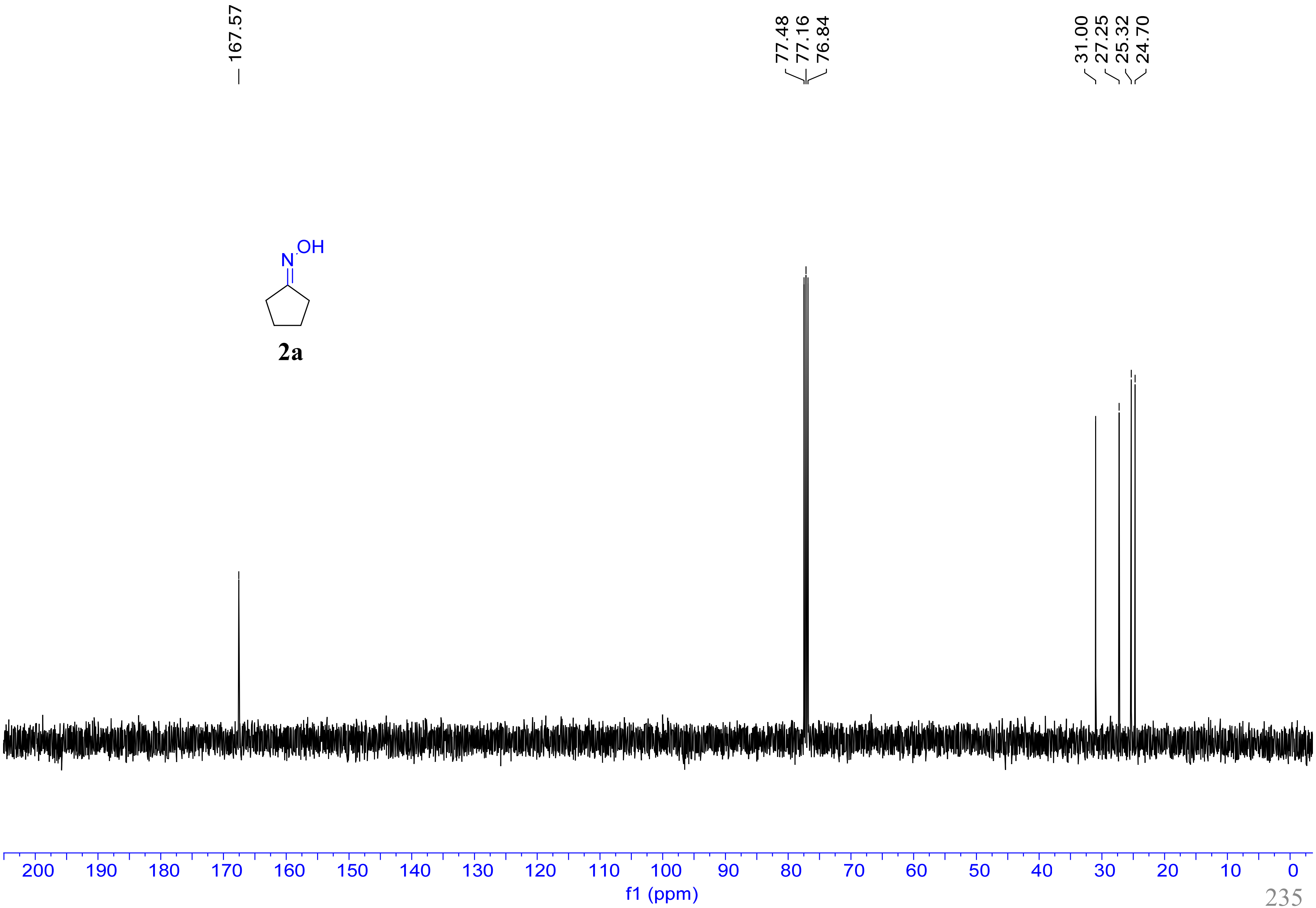

^{1}H NMR, 400 MHz, $CDCl_3$

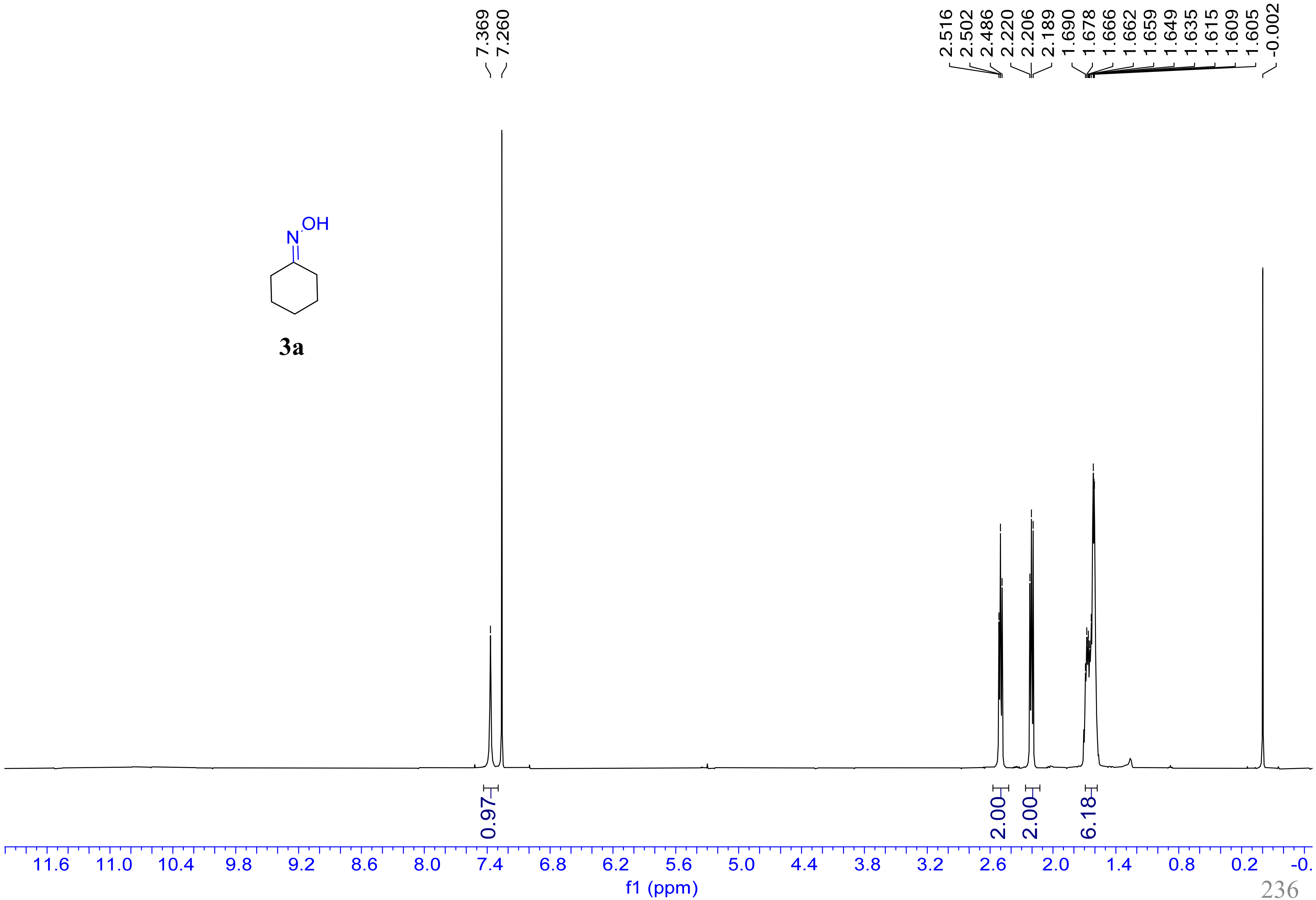

^{13}C NMR 101 MHz, $CDCl_3$

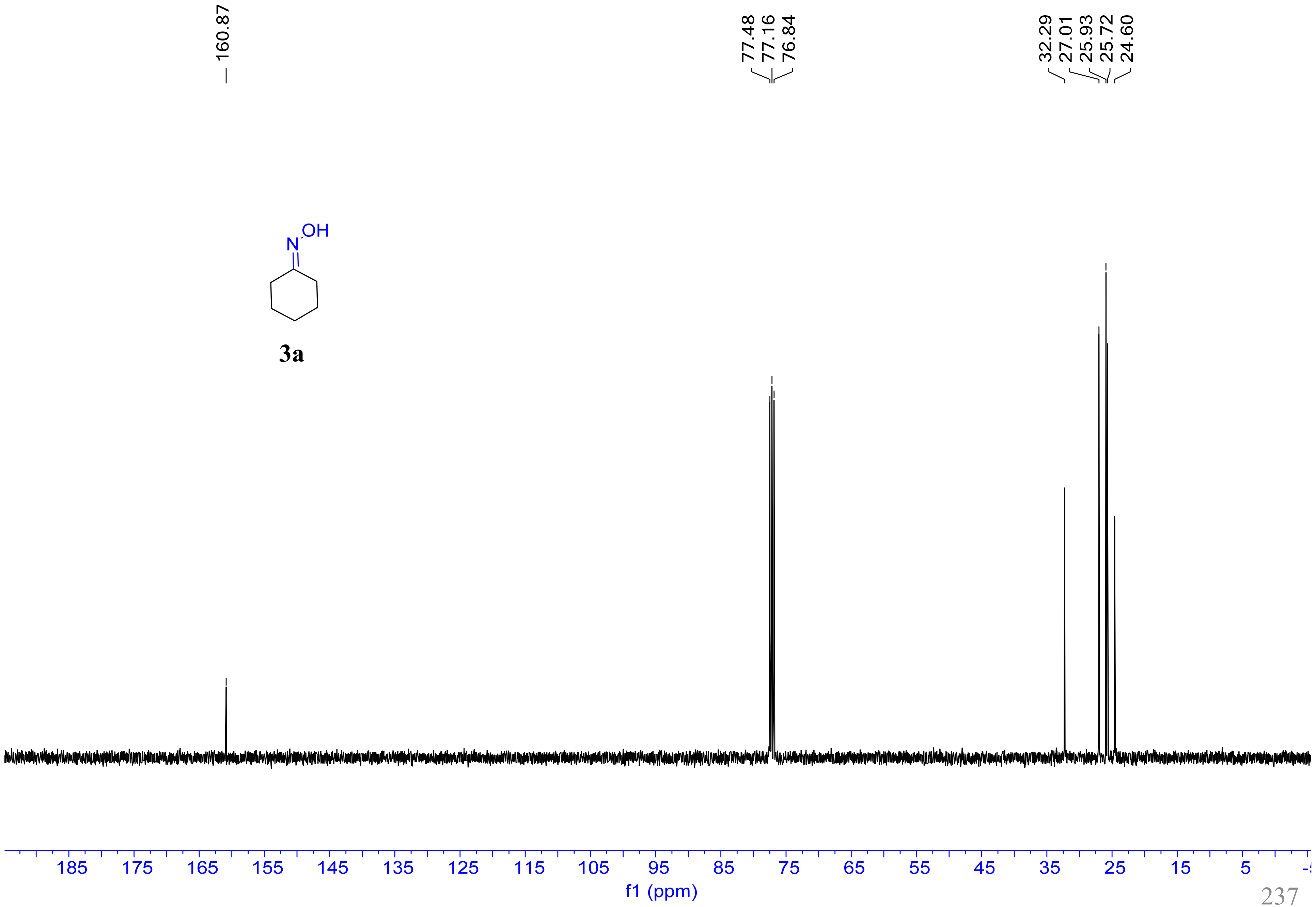

^{1}H NMR, 400 MHz, $CDCl_3$

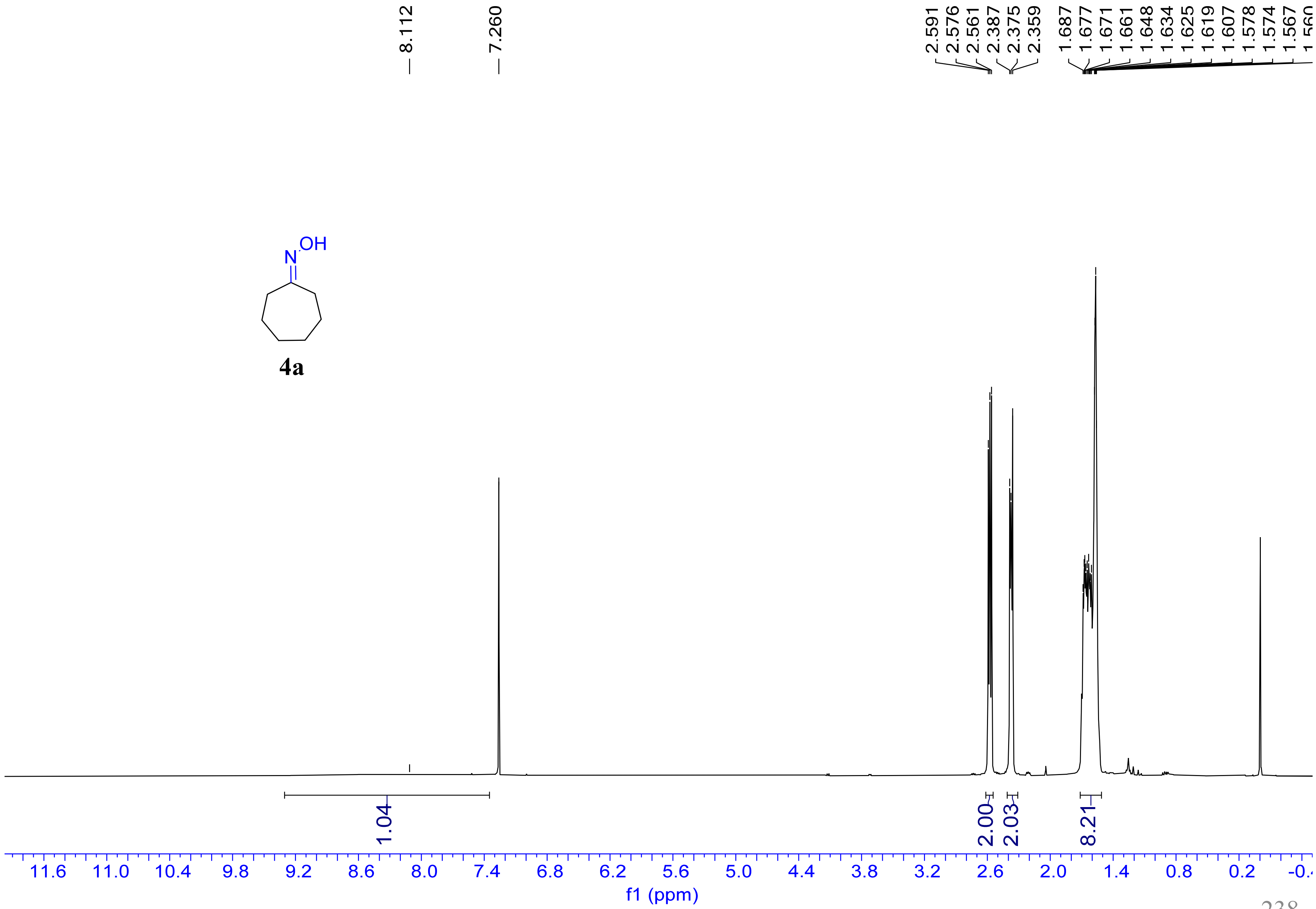

4a

^{13}C NMR 101 MHz, $CDCl_3$

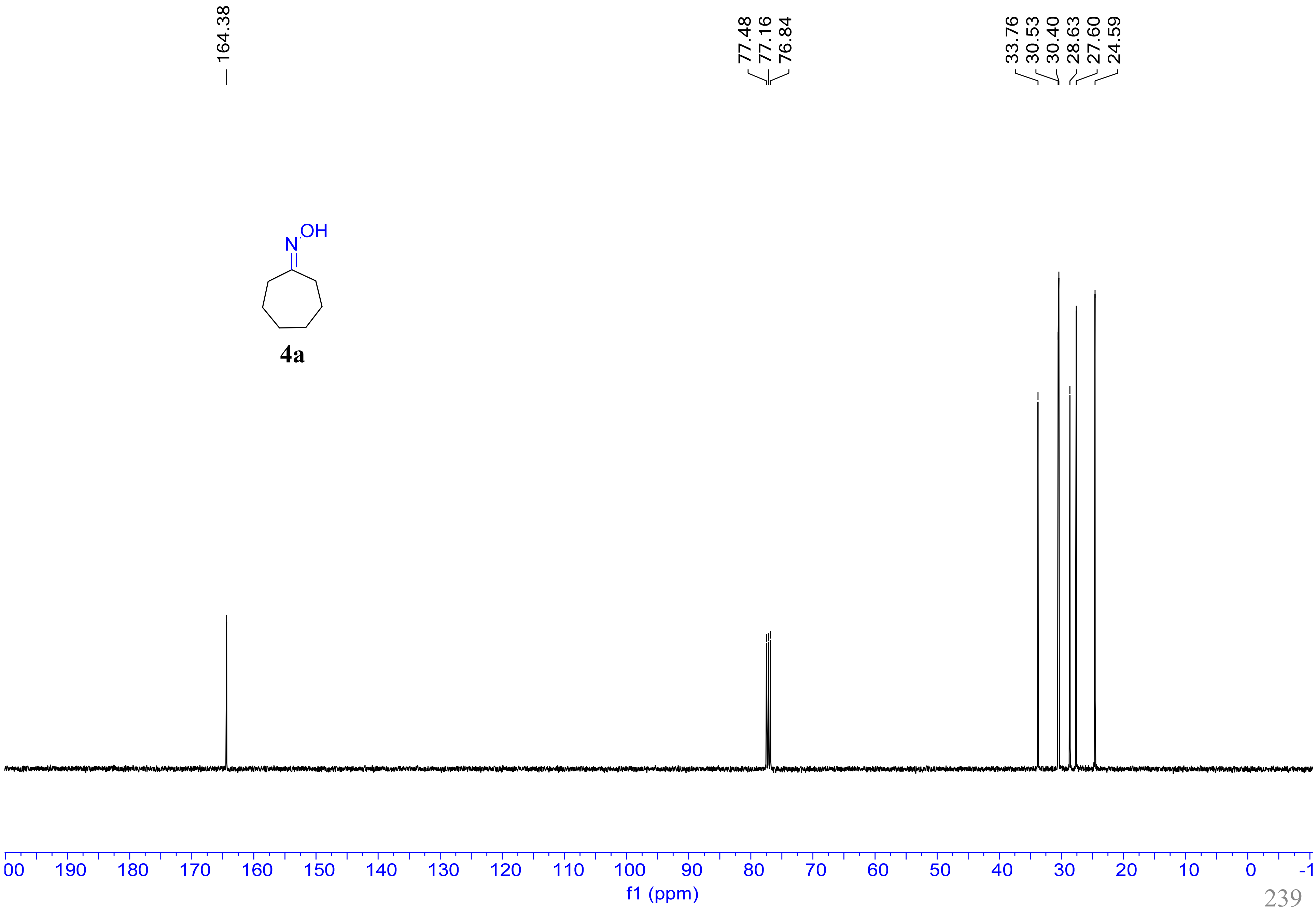

^{1}H NMR, 400 MHz, $CDCl_3$

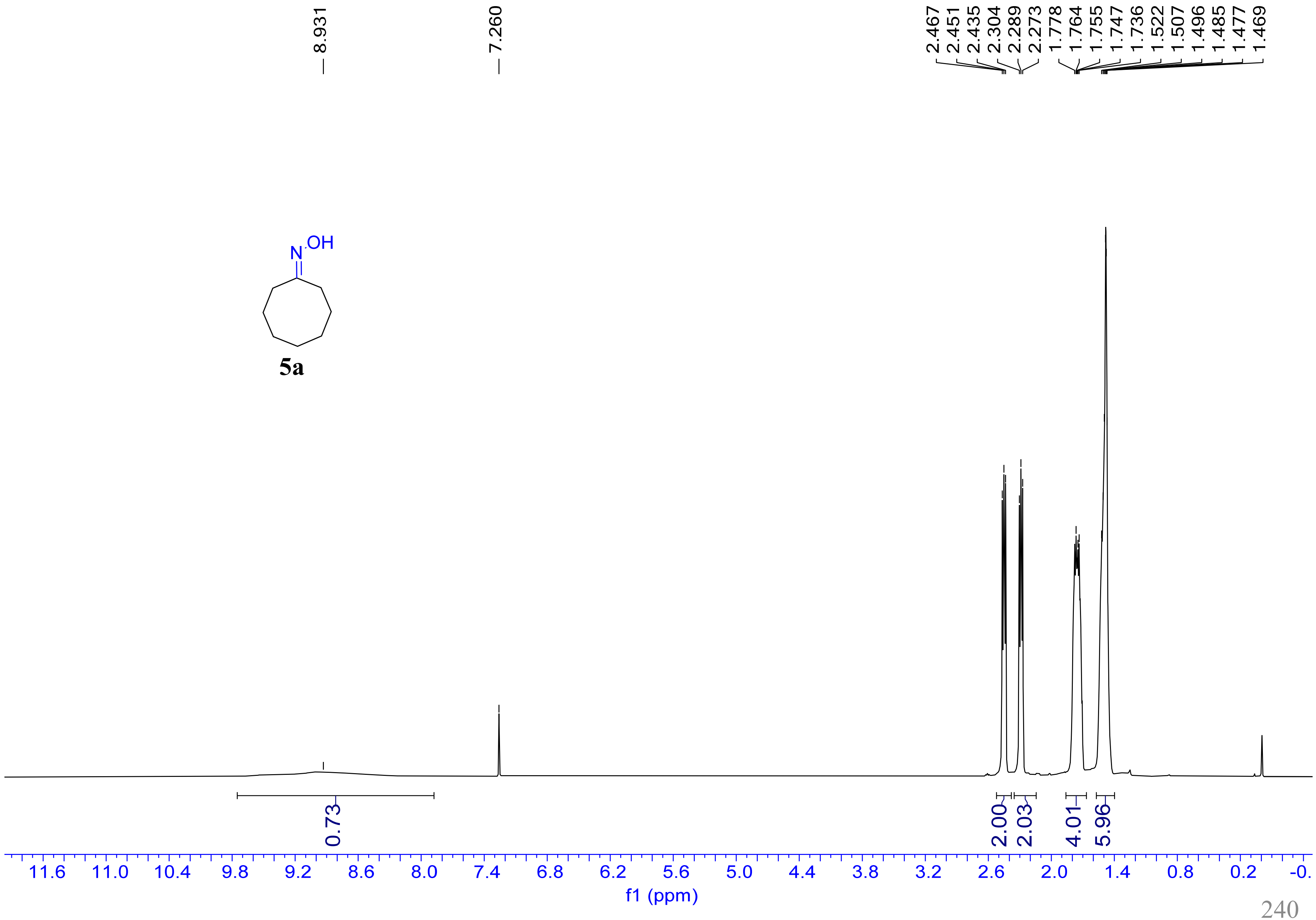

5a

^{13}C NMR 101 MHz, $CDCl_3$

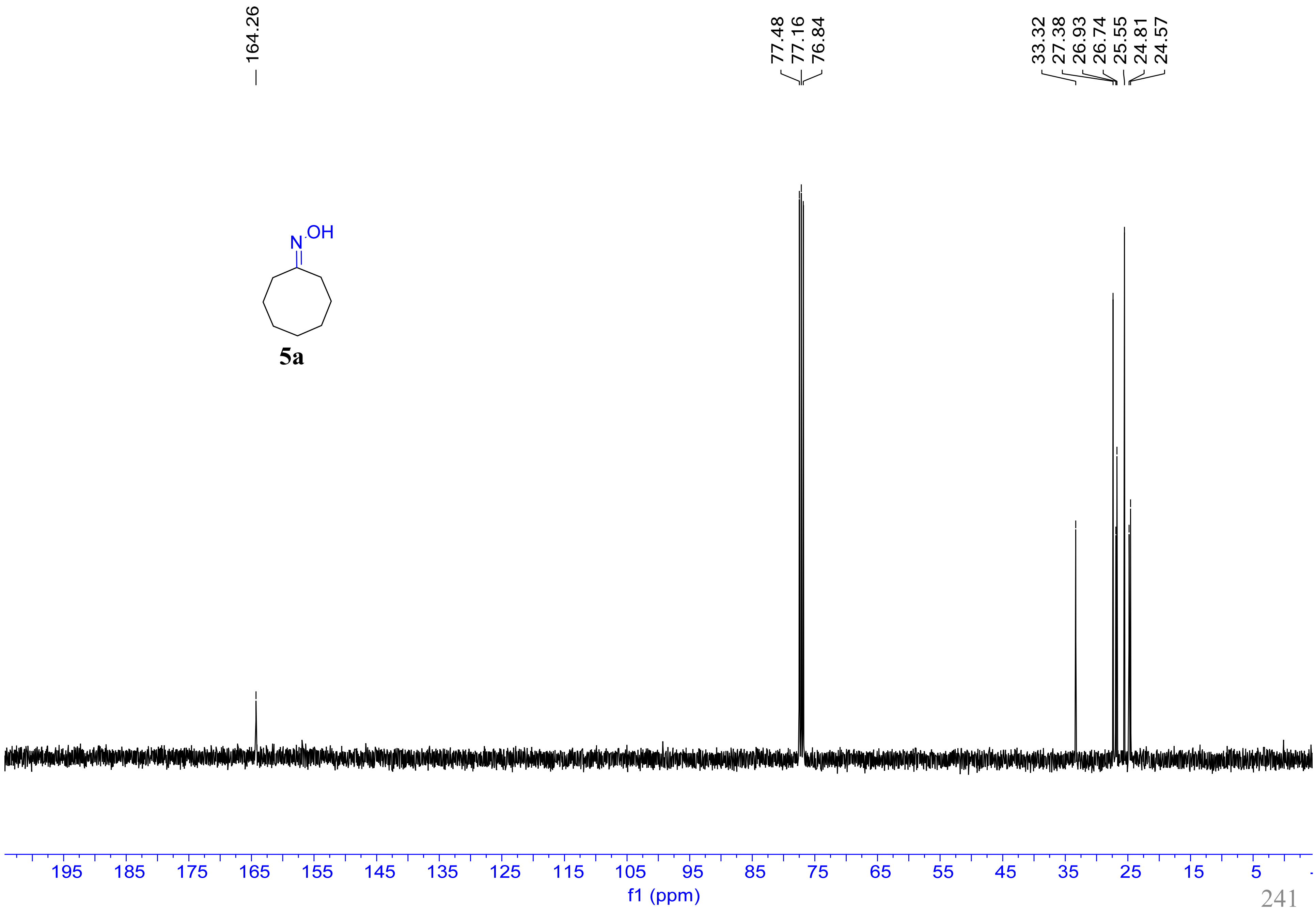

^{1}H NMR, 400 MHz, $CDCl_3$

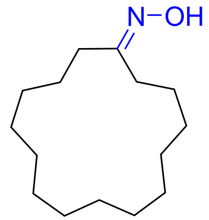

6a

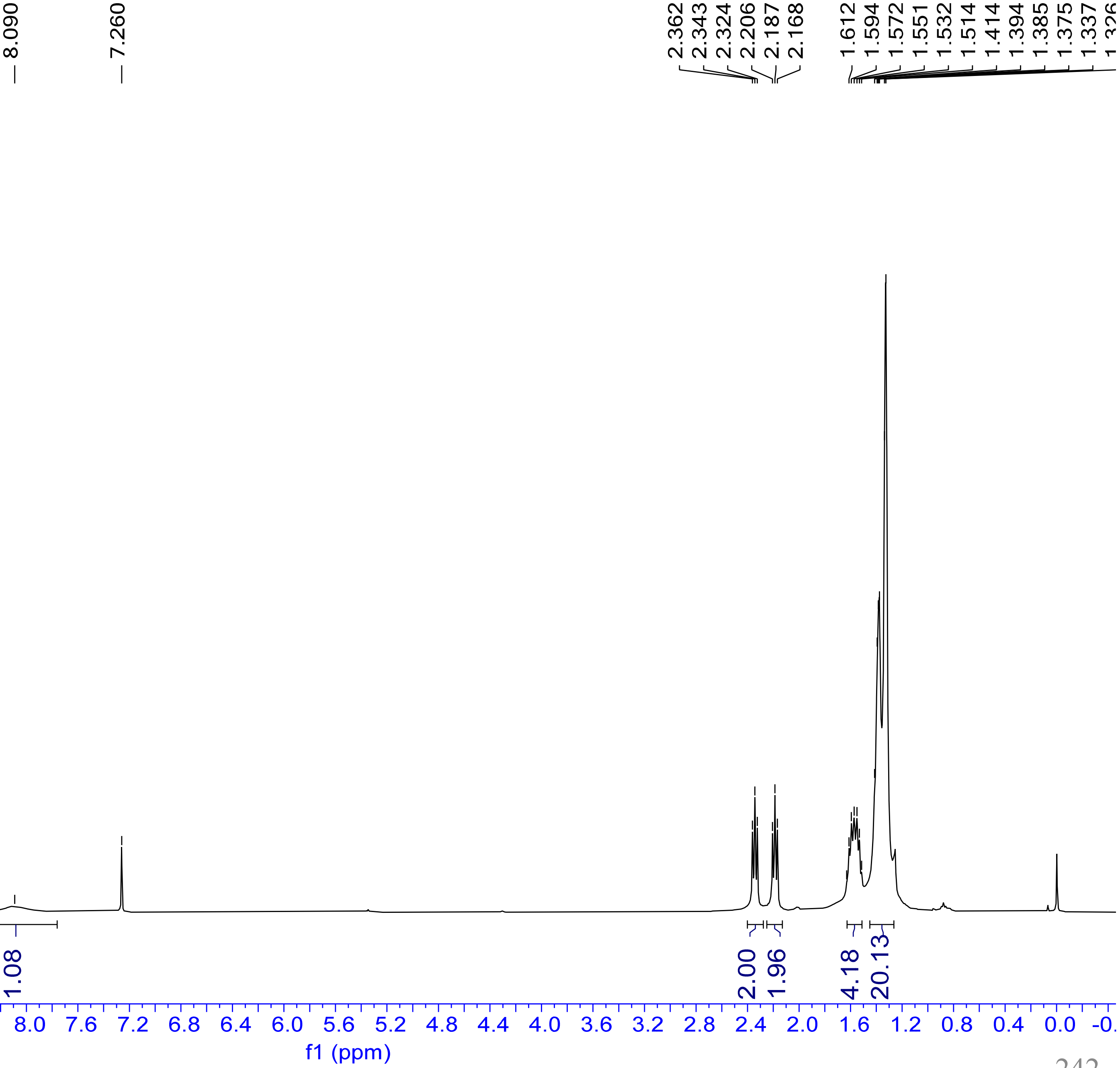

^{13}C NMR 101 MHz, $CDCl_3$

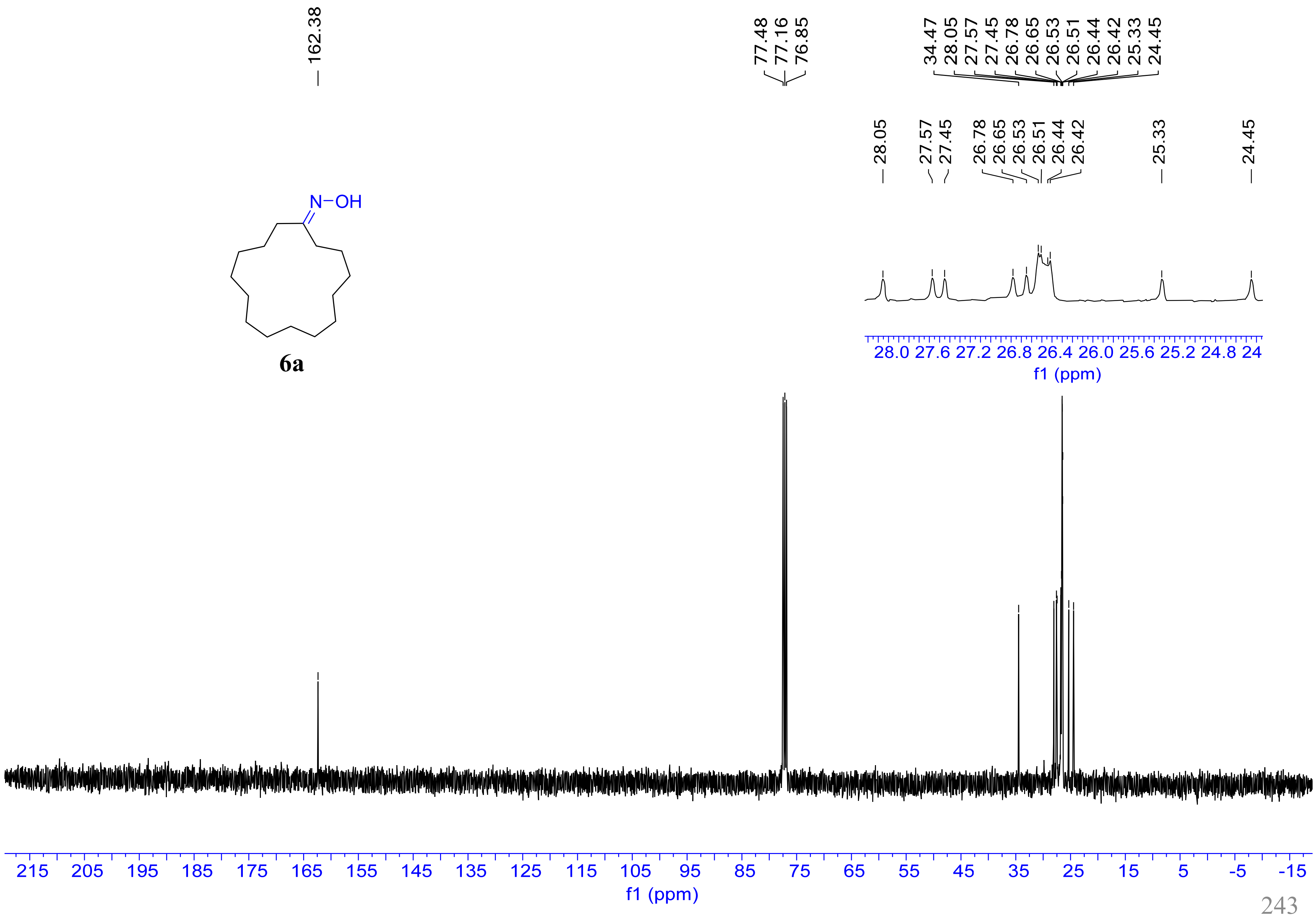

6a

^{1}H NMR, 400 MHz, CDCl$_3$

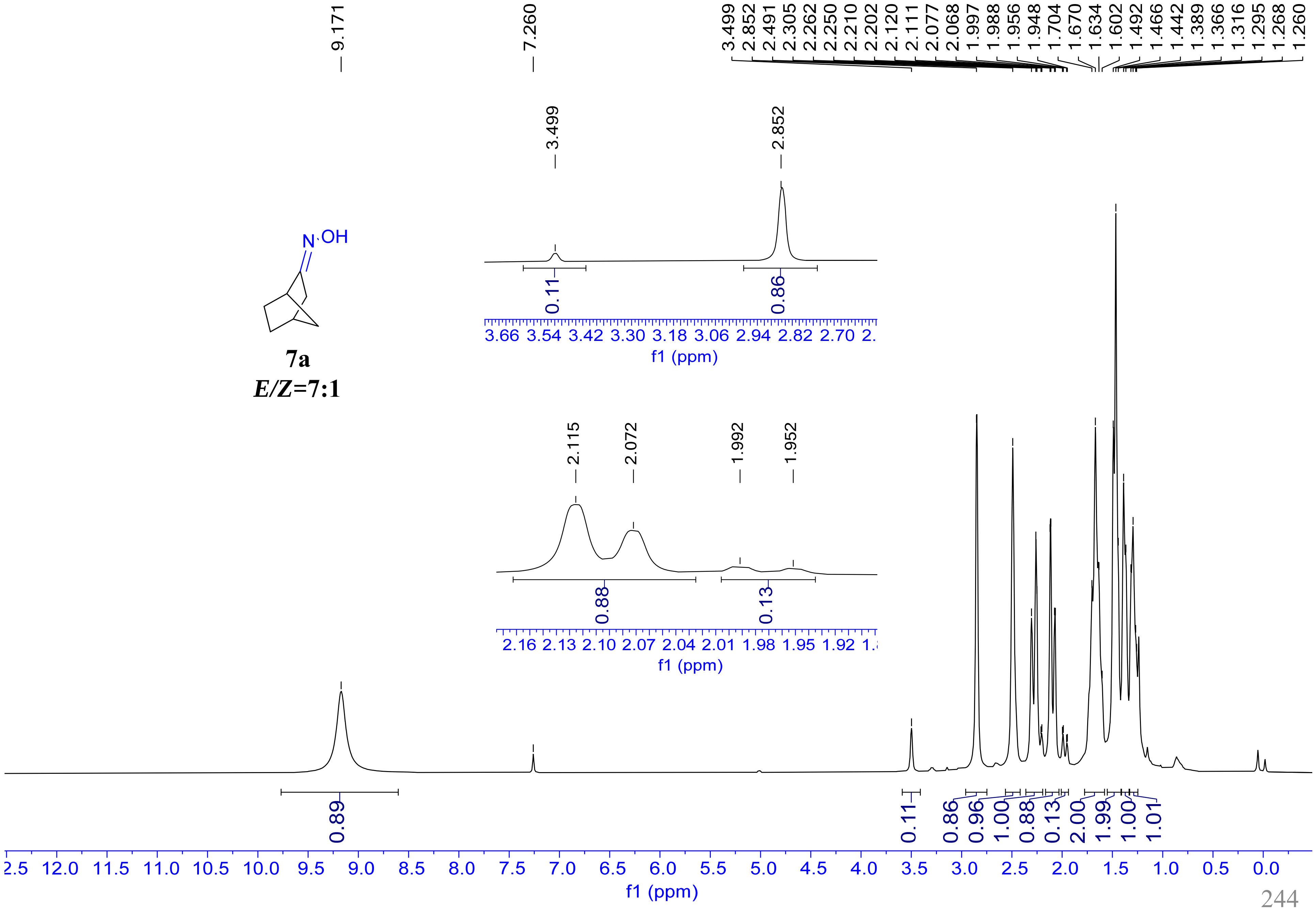

7a

***E/Z*=7:1**

^{13}C NMR 101 MHz, $CDCl_3$

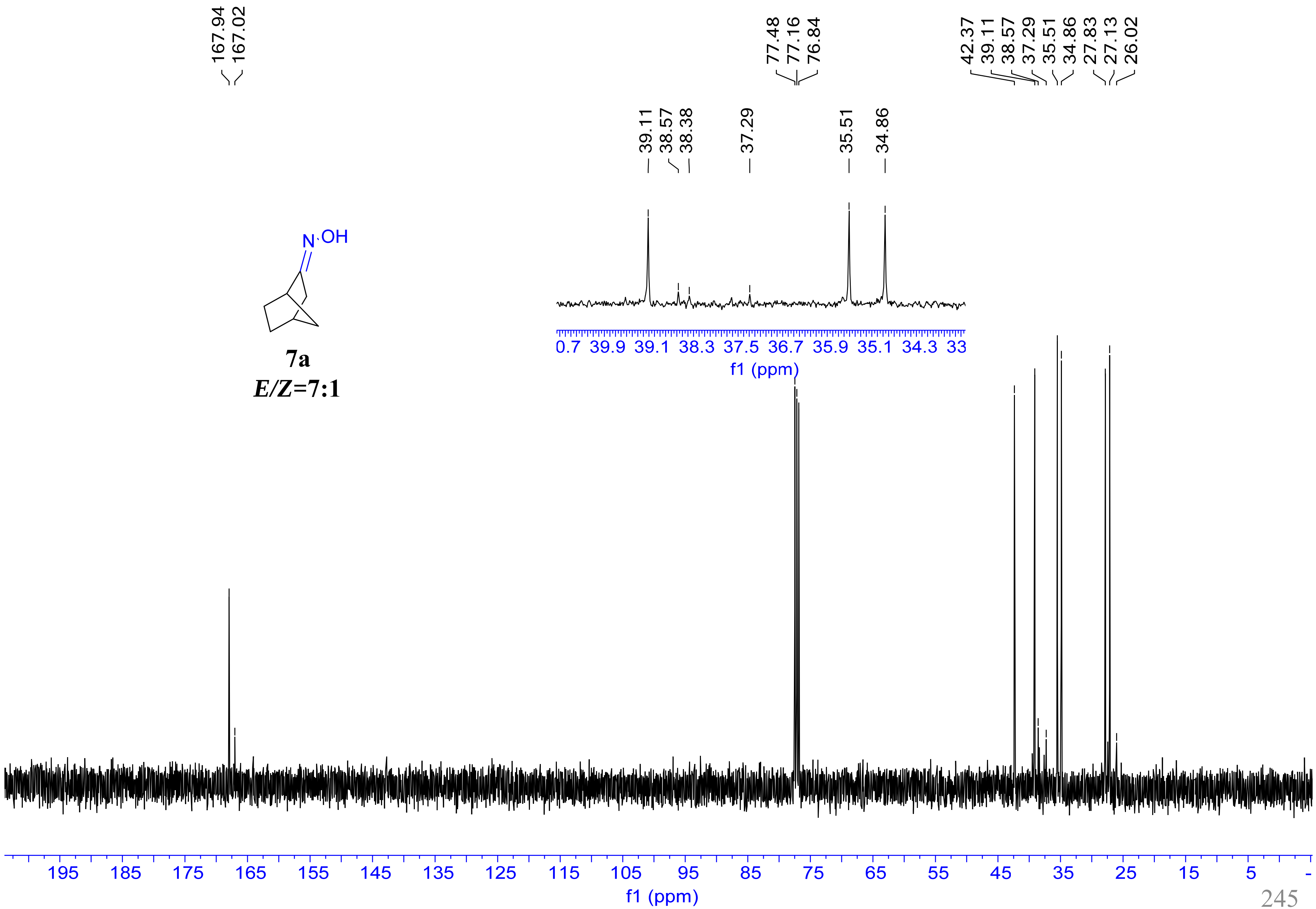

7a

***E/Z*=7:1**

^{1}H NMR, 400 MHz, $CDCl_3$

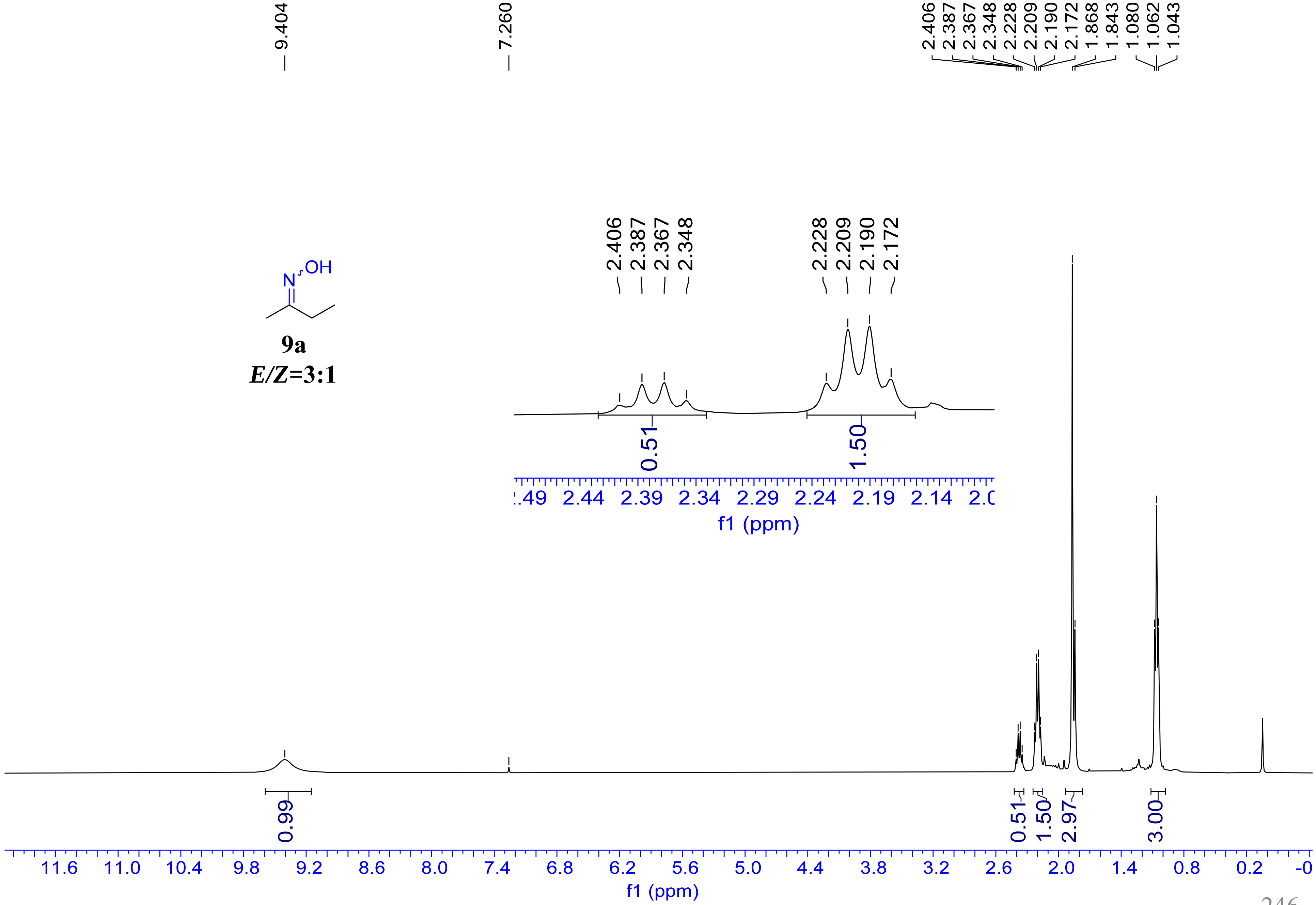

^{13}C NMR 101 MHz, $CDCl_3$

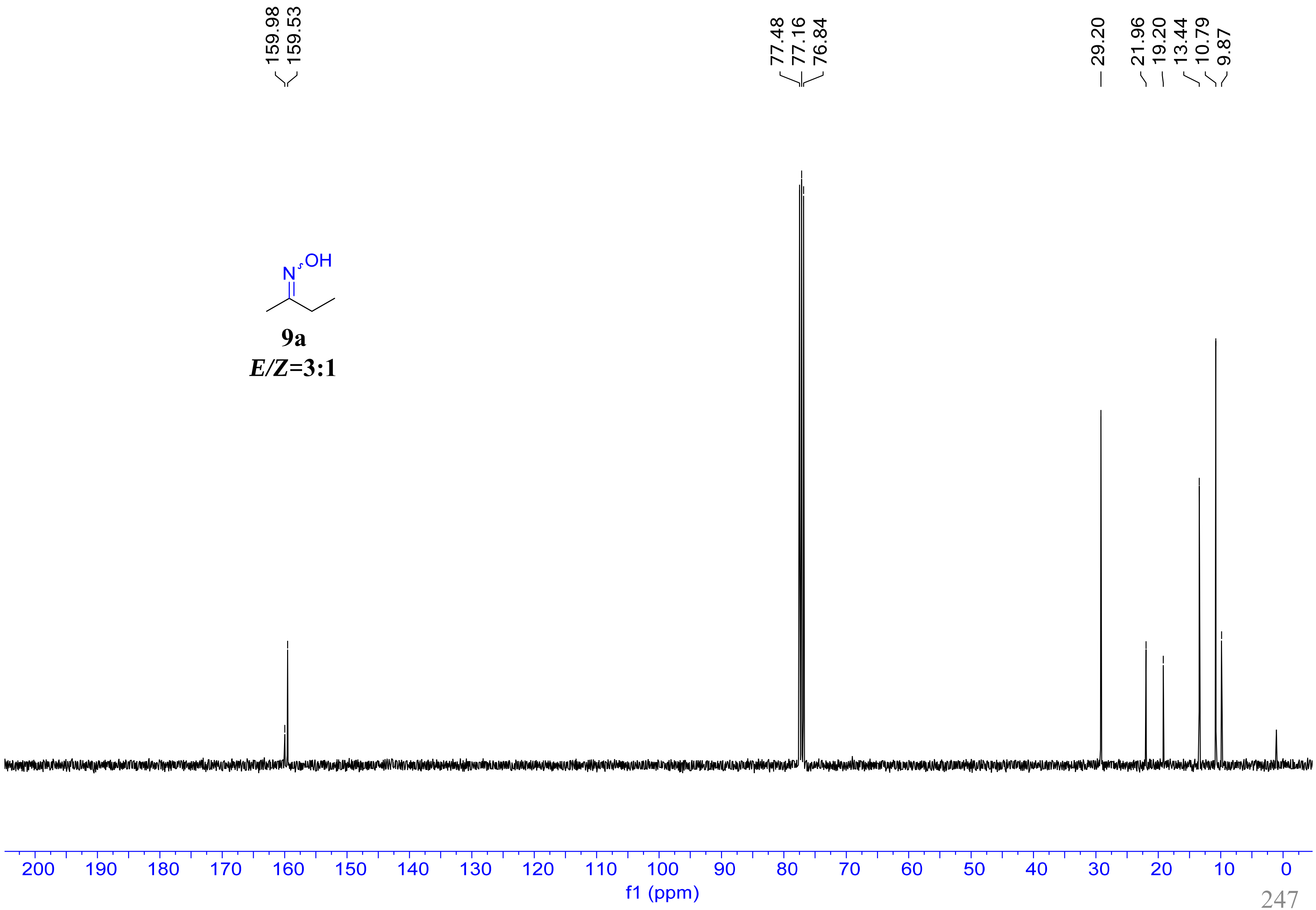

9a

***E*/*Z*=3:1**

^{1}H NMR, 400 MHz, $CDCl_3$

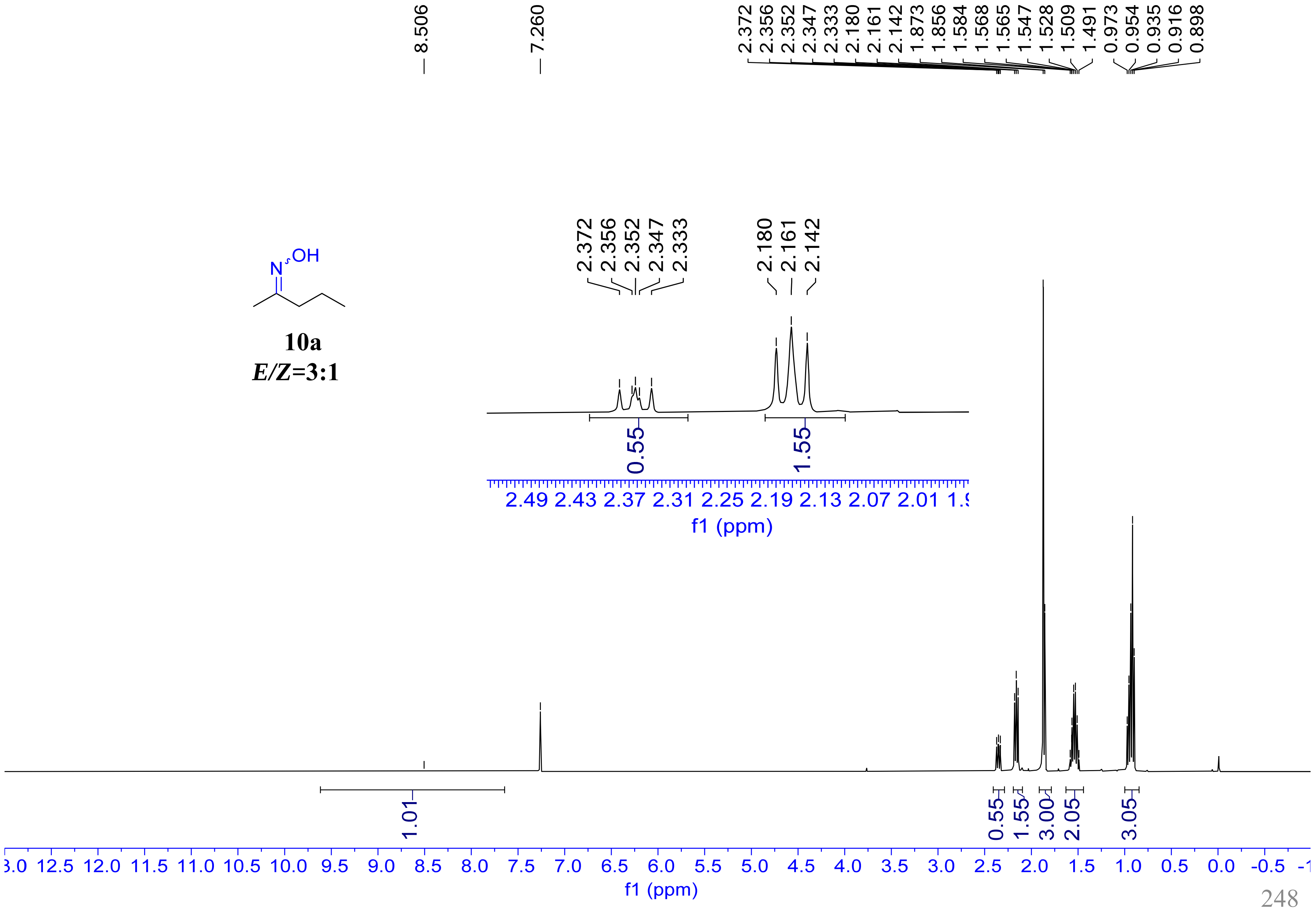

^{13}C NMR 101 MHz, $CDCl_3$

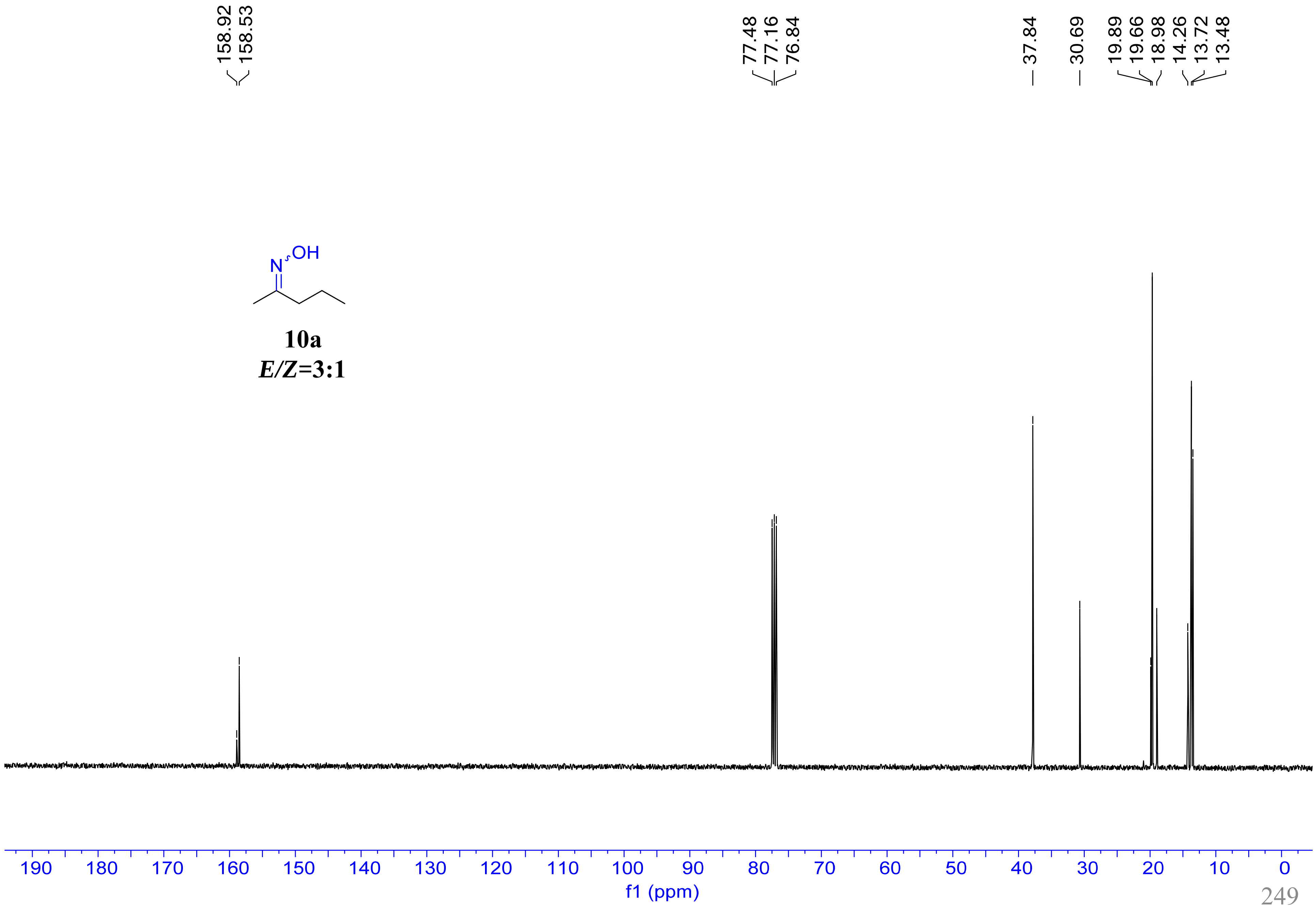

10a
***E/Z*=3:1**

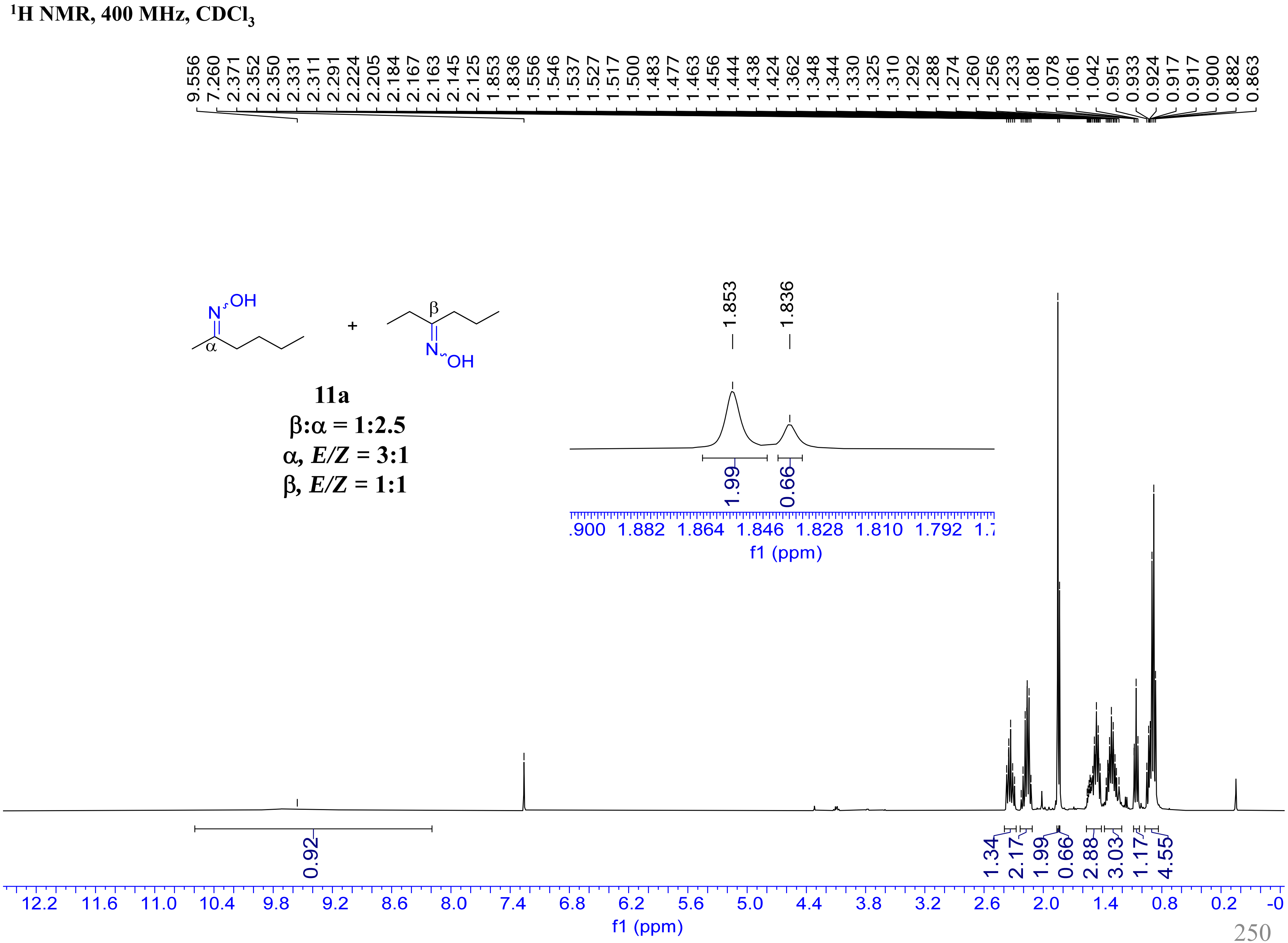

¹H NMR, 400 MHz, CDCl3
9.556 7.260 2.371 2.352 2.350 2.331 2.311 2.291 2.224 2.205 2.184 2.167 2.163 2.145 2.125 1.853 1.836 1.556 1.546 1.537 1.527 1.517 1.500 1.483 1.477 1.463 1.456 1.444 1.438 1.424 1.362 1.348 1.344 1.330 1.325 1.310 1.292 1.288 1.274 1.260 1.256 1.233 1.081 1.078 1.061 1.042 0.951 0.933 0.924 0.917 0.917 0.900 0.882 0.863
OH
N
α
+
β
N
OH
11a
β:α = 1:2.5
α, E/Z = 3:1
β, E/Z = 1:1
1.853
1.836
1.99
0.66
f1 (ppm)
0.92
1.34
2.17
1.99
0.66
2.88
3.03
1.17
4.55
f1 (ppm)

^{13}C NMR 101 MHz, $CDCl_3$

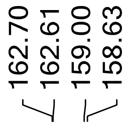

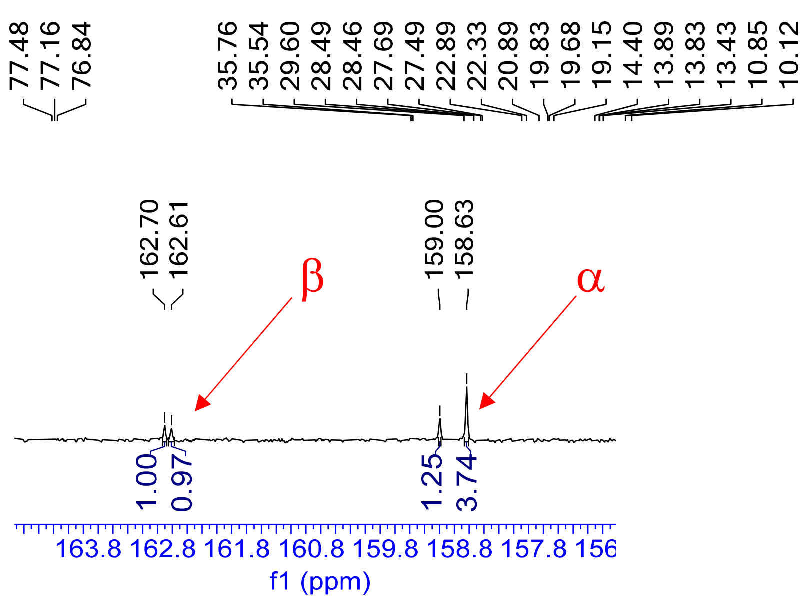

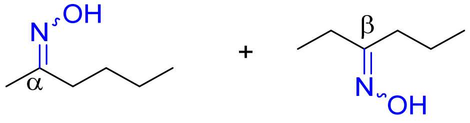

11a

β:α =1:2.5

α, *E/Z*=3:1

β, *E/Z*=1:1

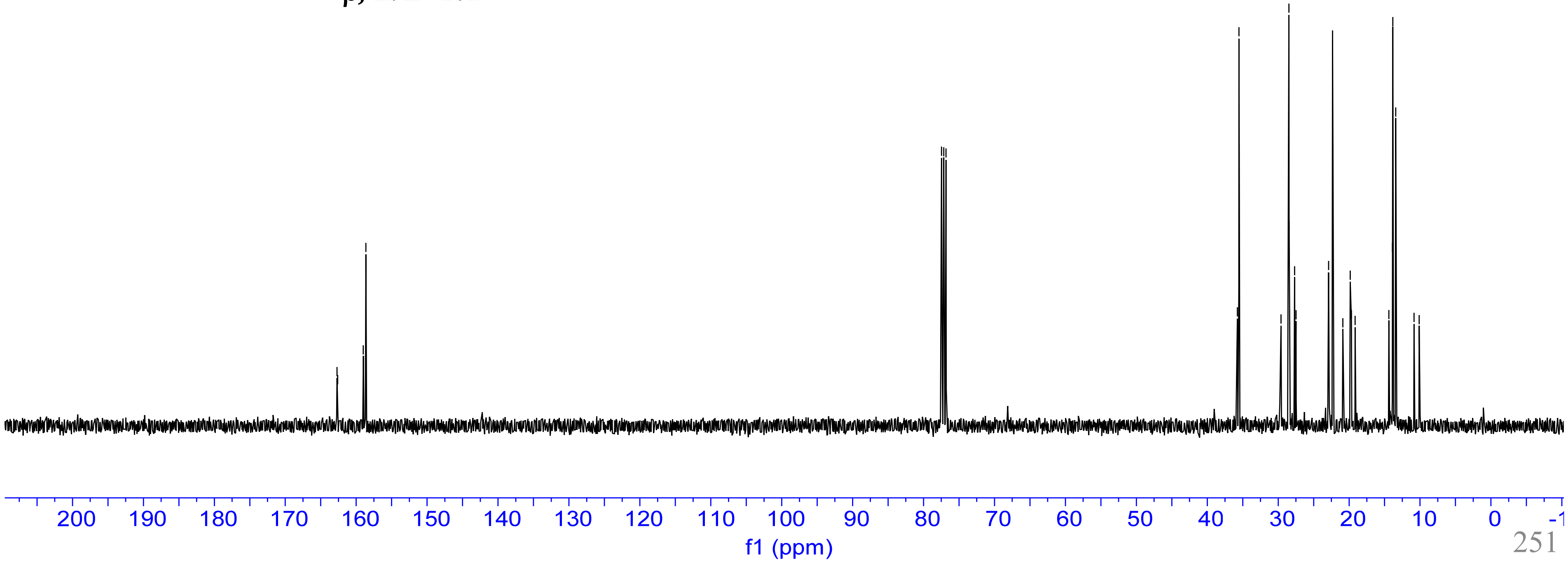

^{1}H NMR, 400 MHz, $CDCl_3$

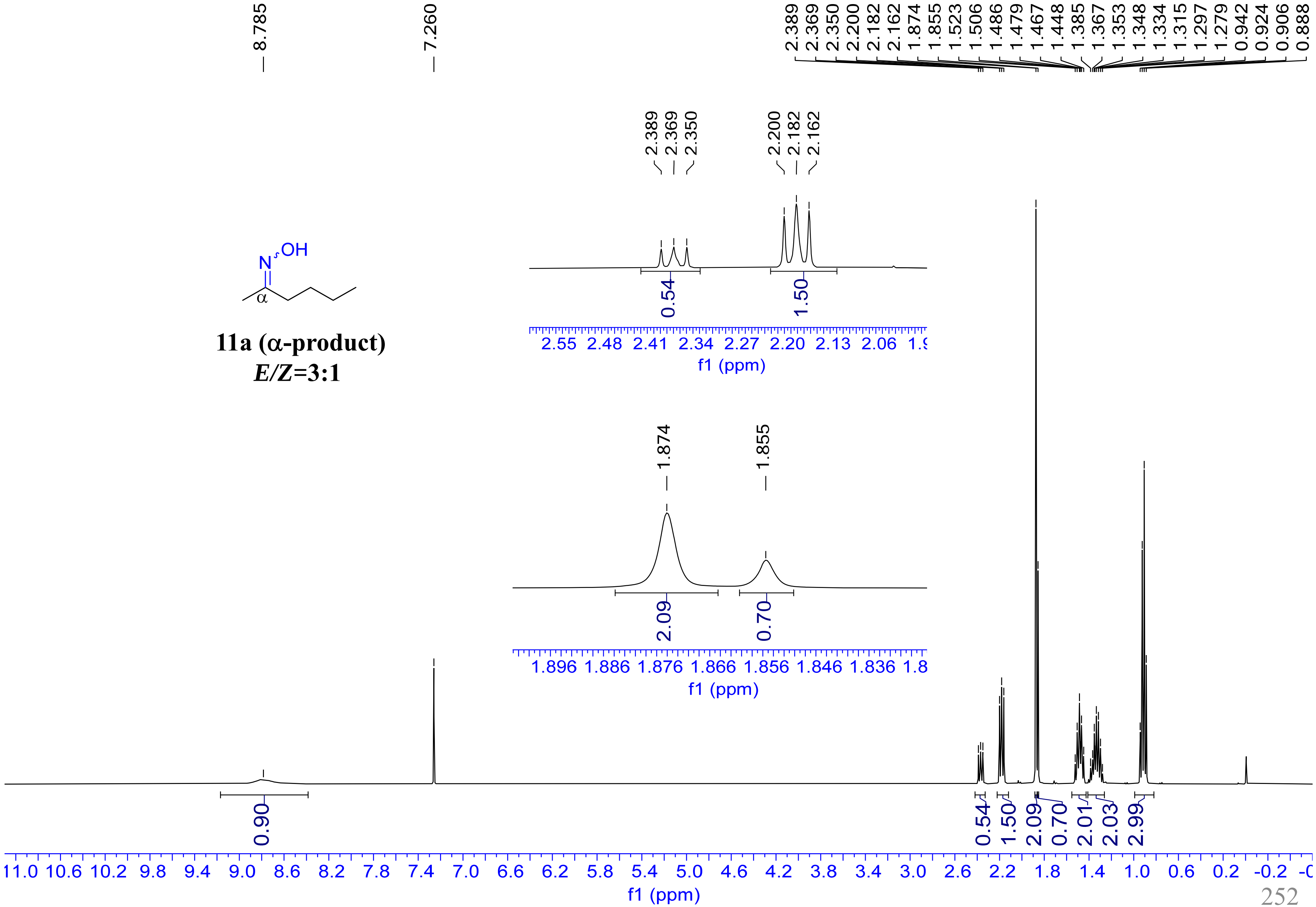

^{13}C NMR 101 MHz, $CDCl_3$

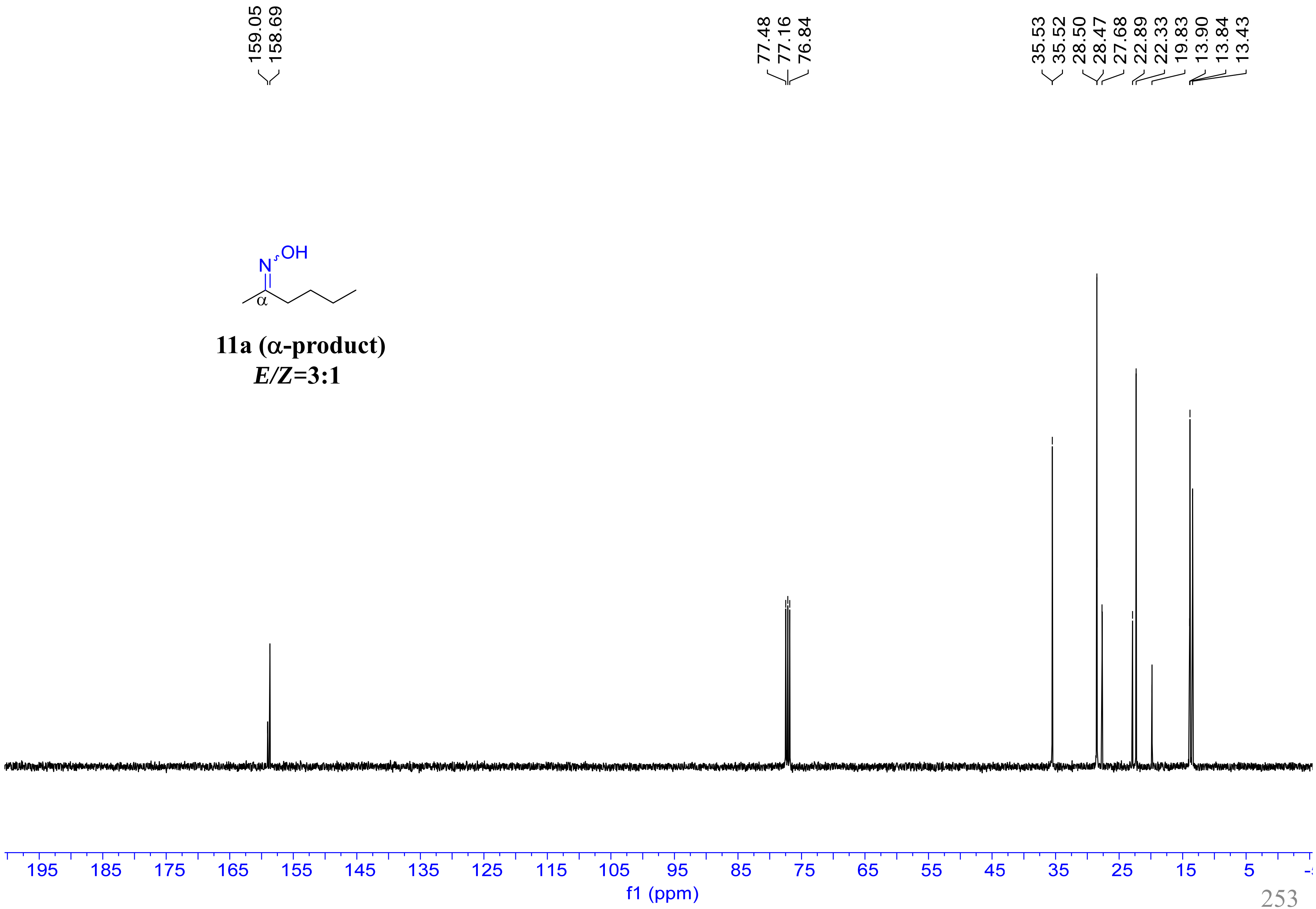

11a (α-product)

***E*/*Z*=3:1**

^{1}H NMR, 400 MHz, $CDCl_3$

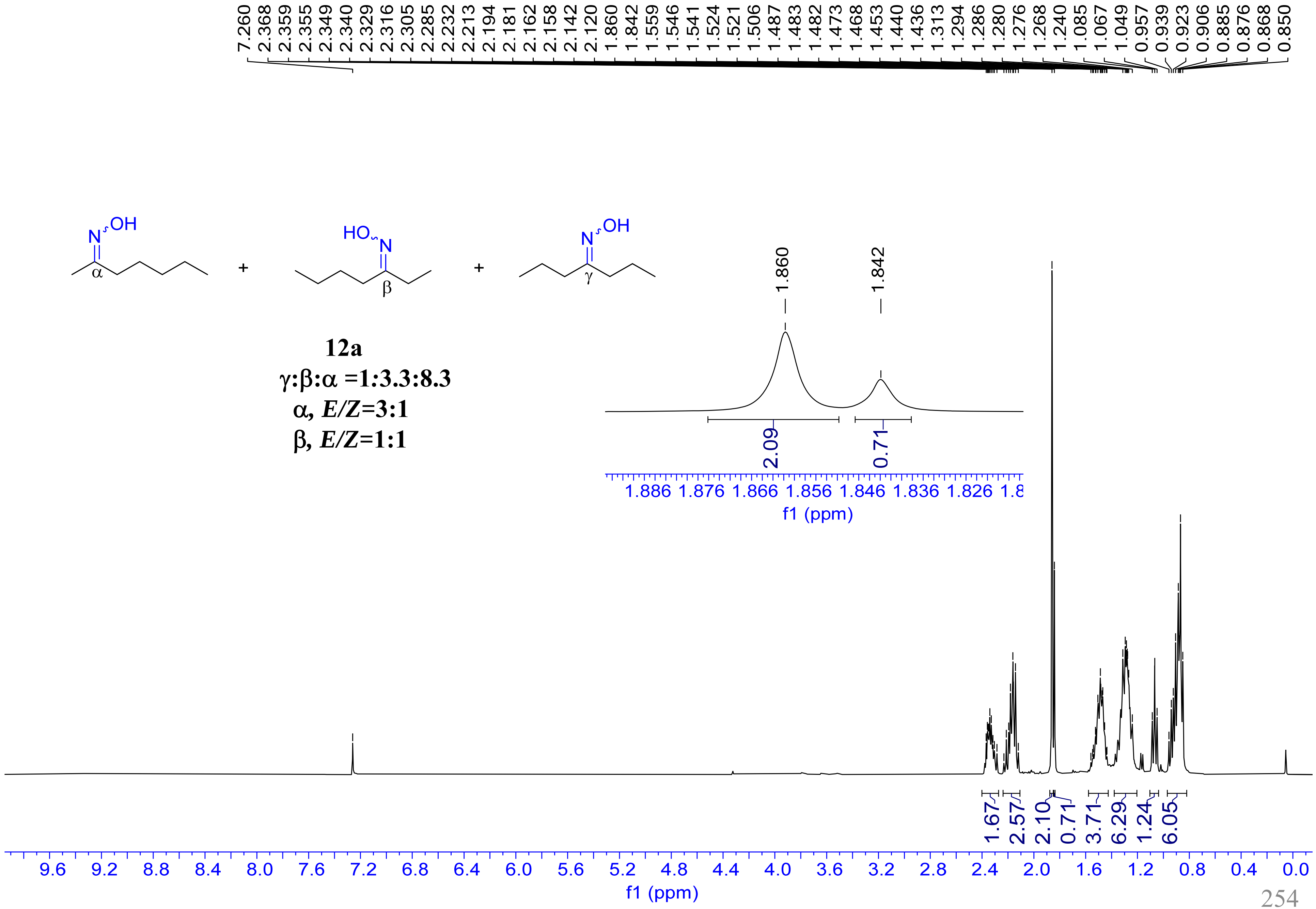

^{13}C NMR 101 MHz, $CDCl_3$

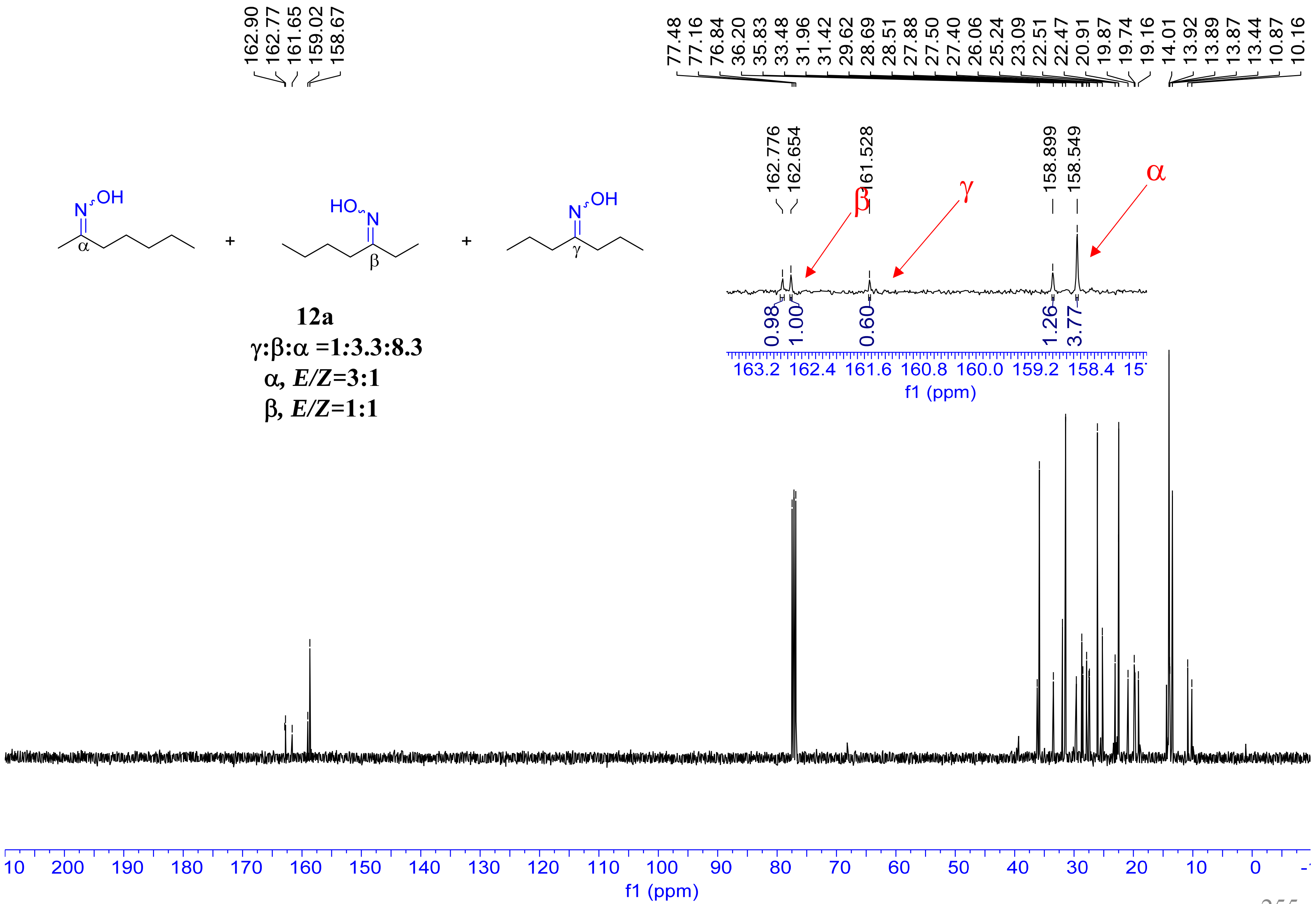

^{1}H NMR, 400 MHz, $CDCl_3$

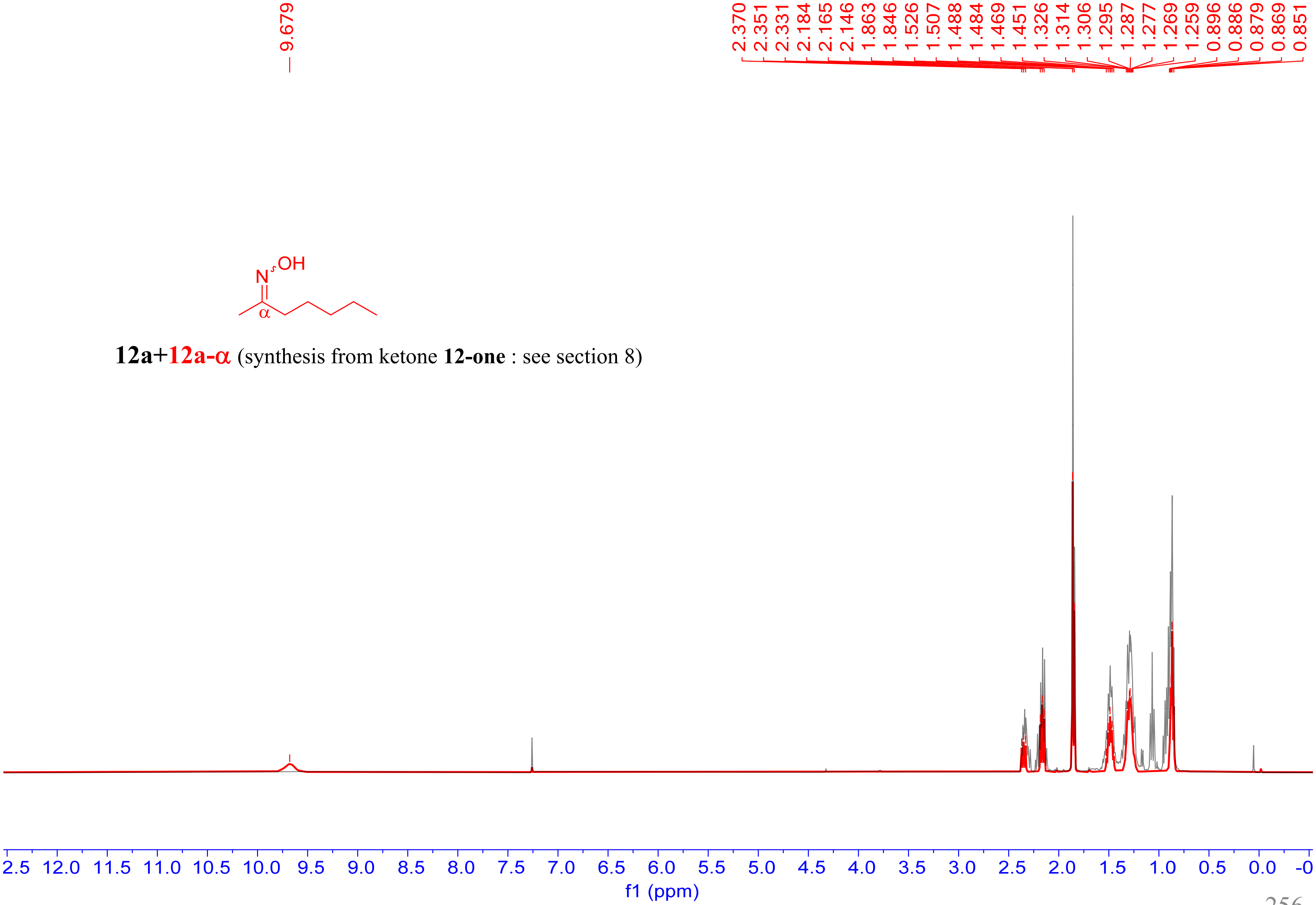

12a+**12a-α** (synthesis from ketone **12-one** : see section 8)

^{13}C NMR 101 MHz, $CDCl_3$

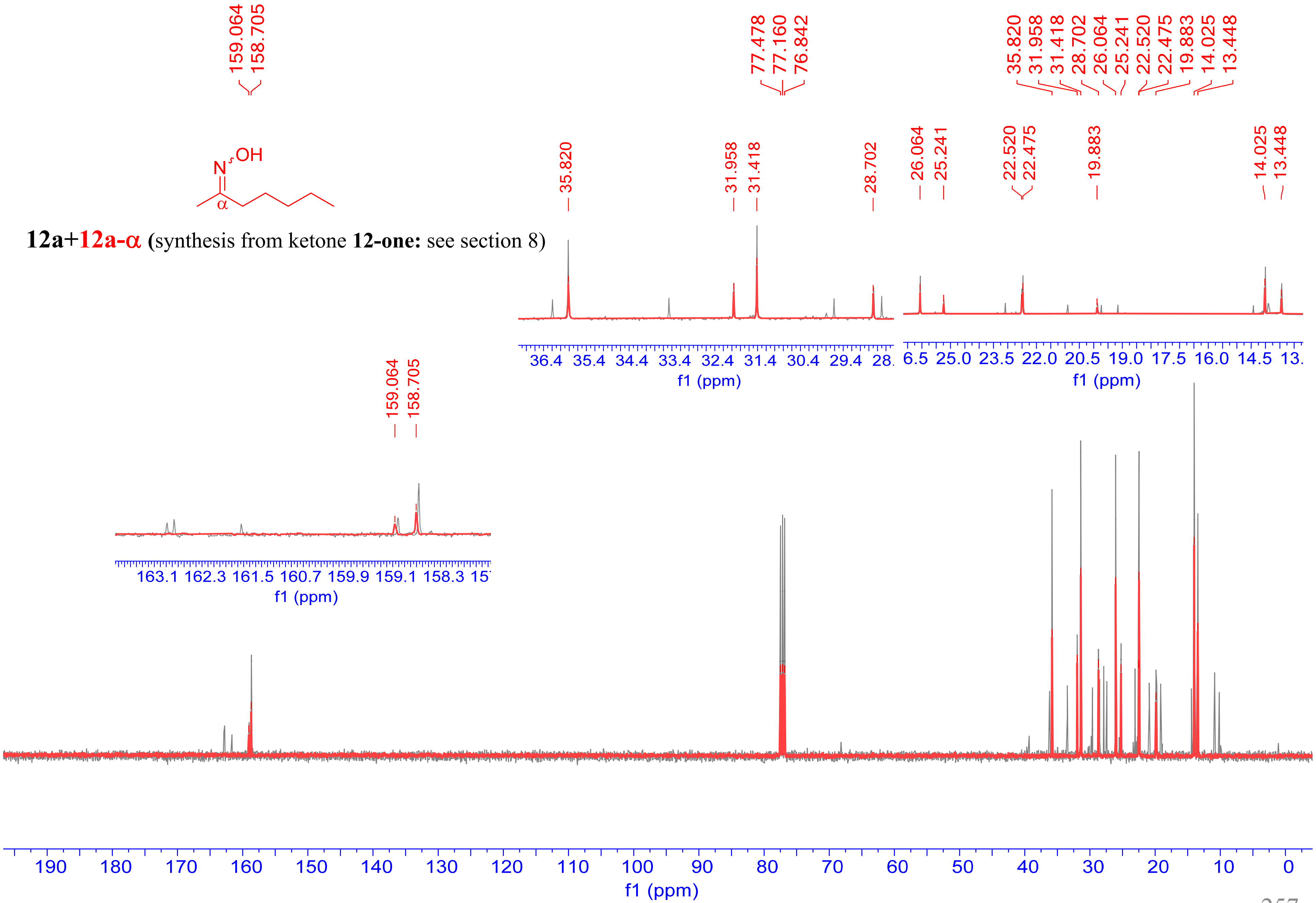

12a+12a-α (synthesis from ketone **12-one:** see section 8)

^{1}H NMR, 400 MHz, $CDCl_3$

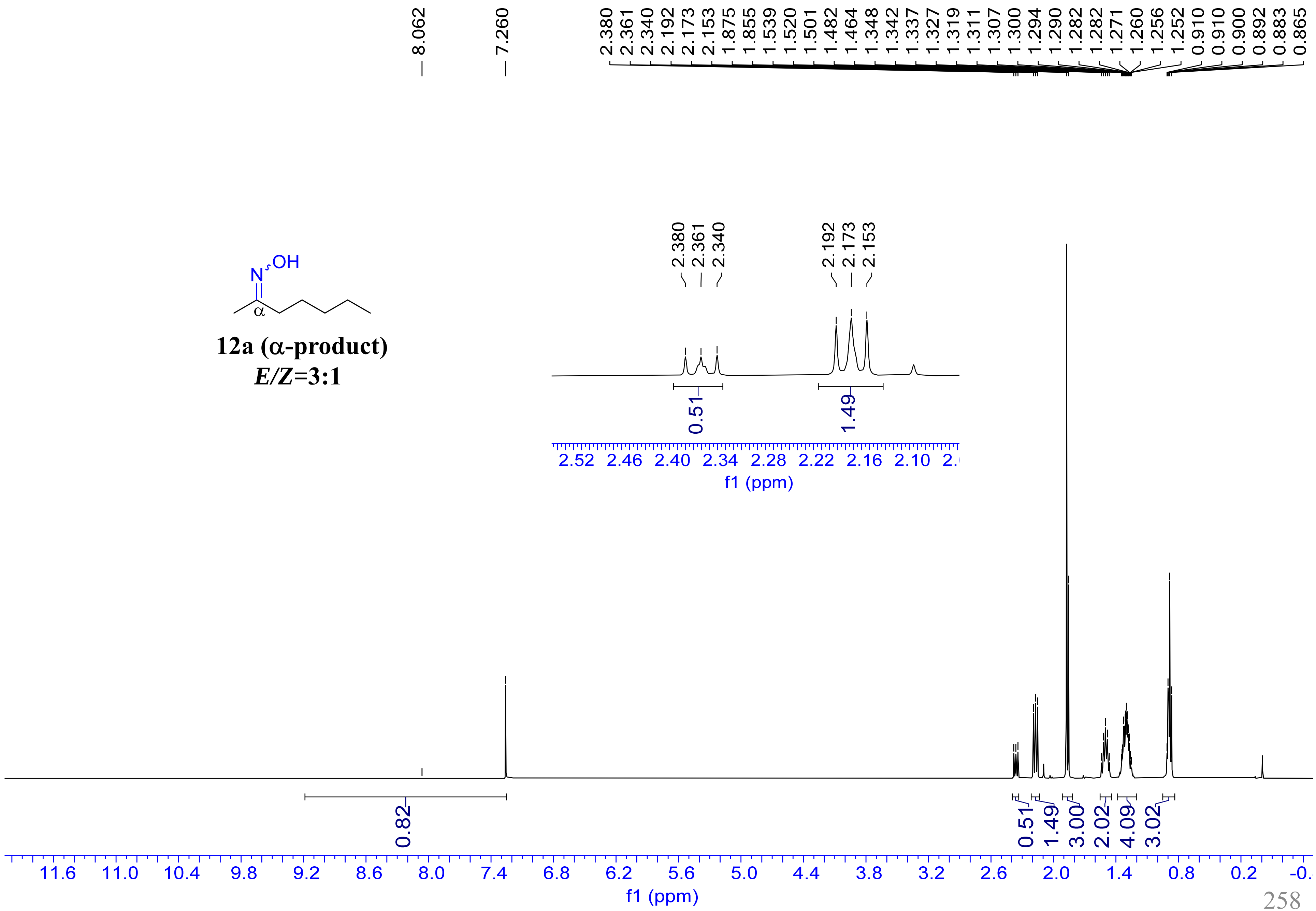

^{13}C NMR 101 MHz, $CDCl_3$

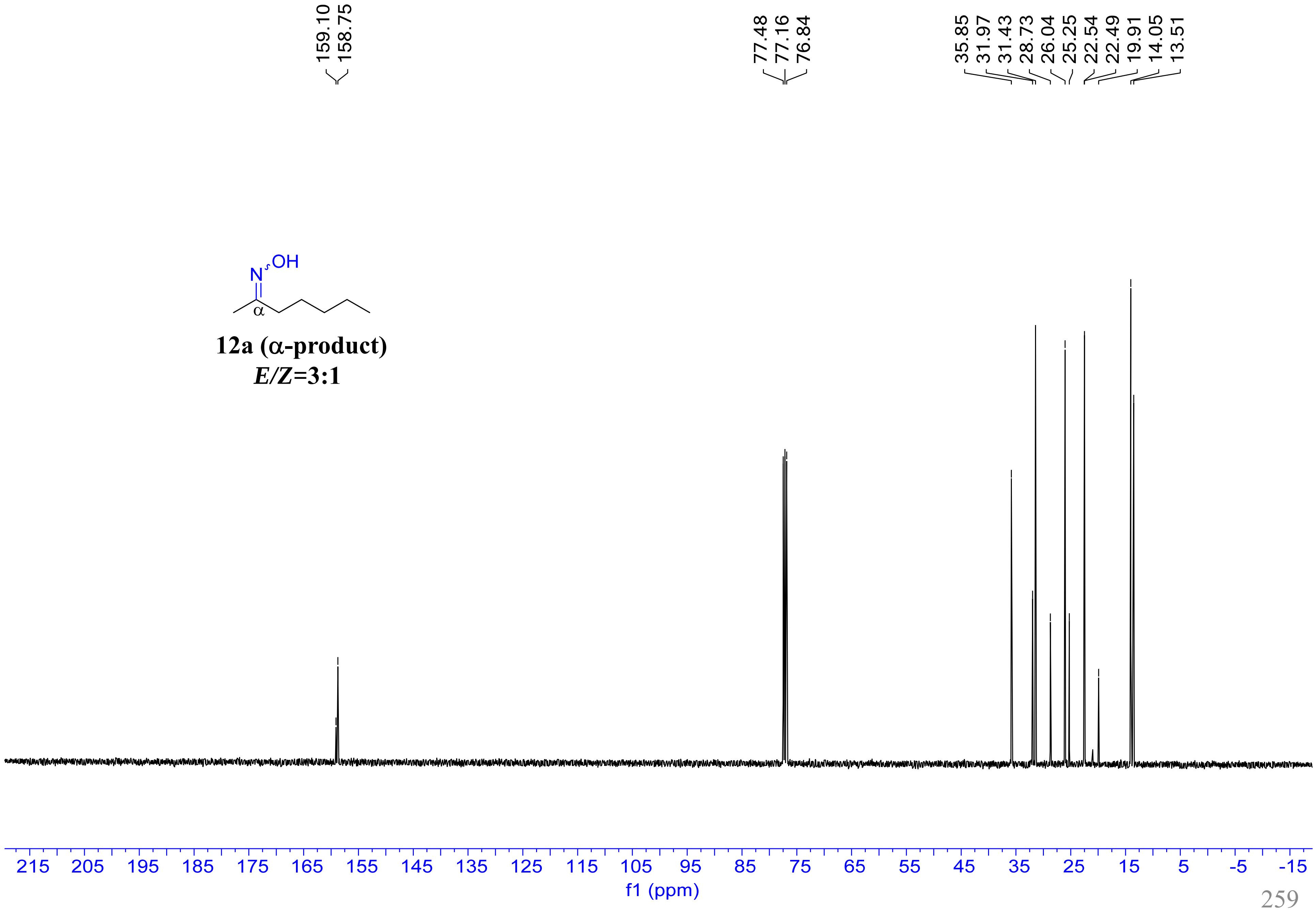

^{1}H NMR, 400 MHz, $CDCl_3$

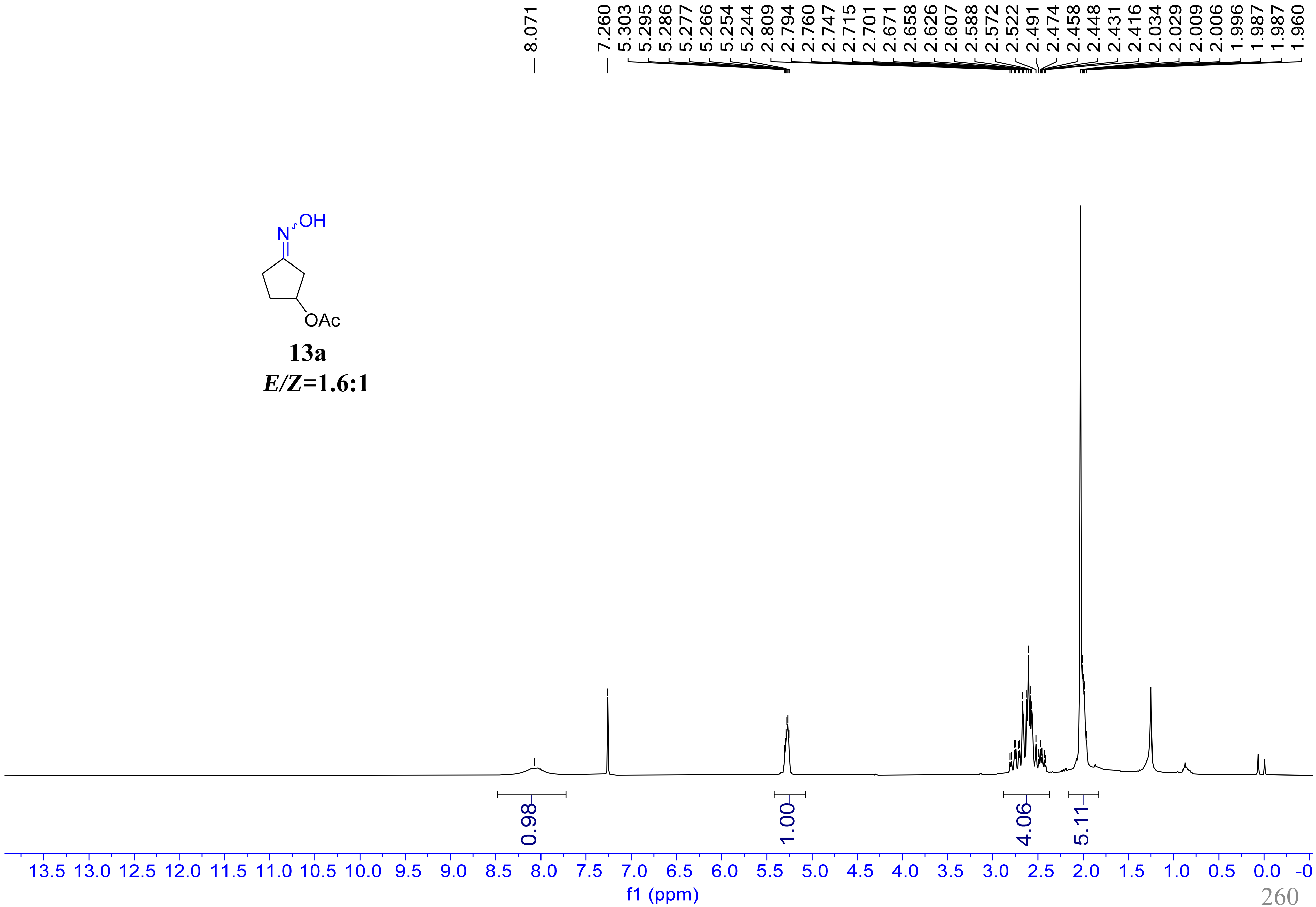

13a

***E/Z*=1.6:1**

^{13}C NMR 101 MHz, $CDCl_3$

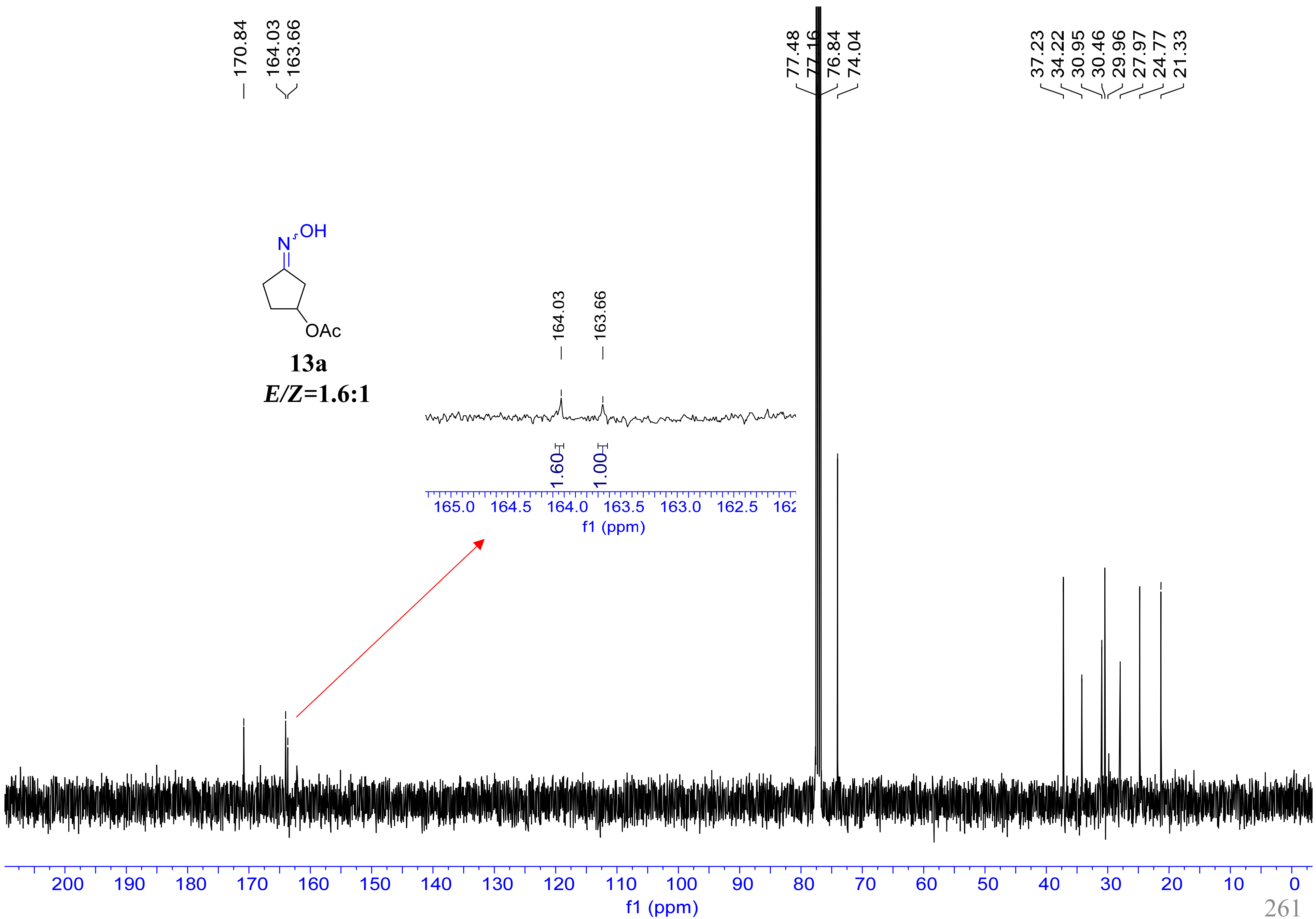

^1H NMR, 400 MHz, $CDCl_3$

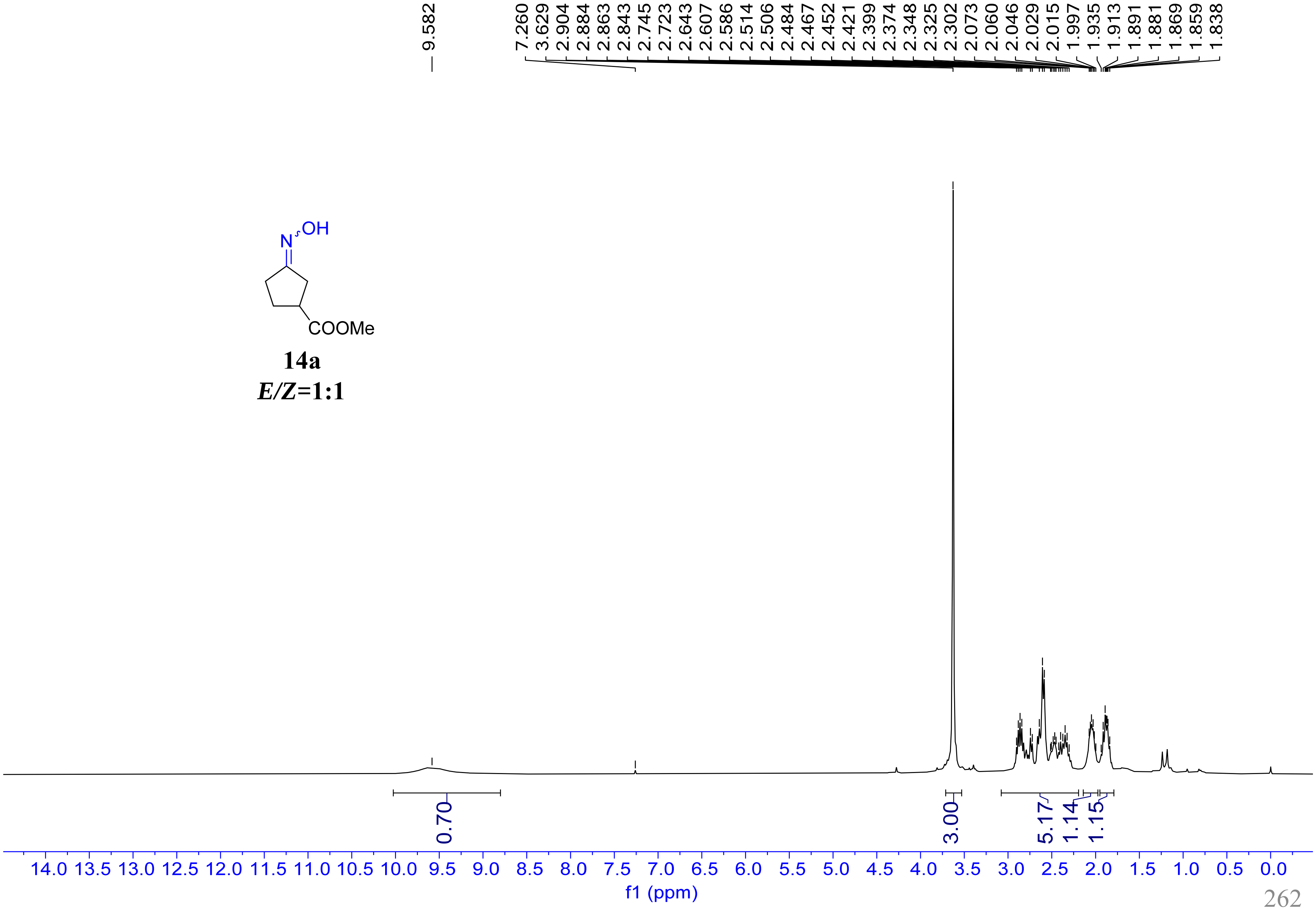

14a
***E/Z*=1:1**

^{13}C NMR 101 MHz, $CDCl_3$

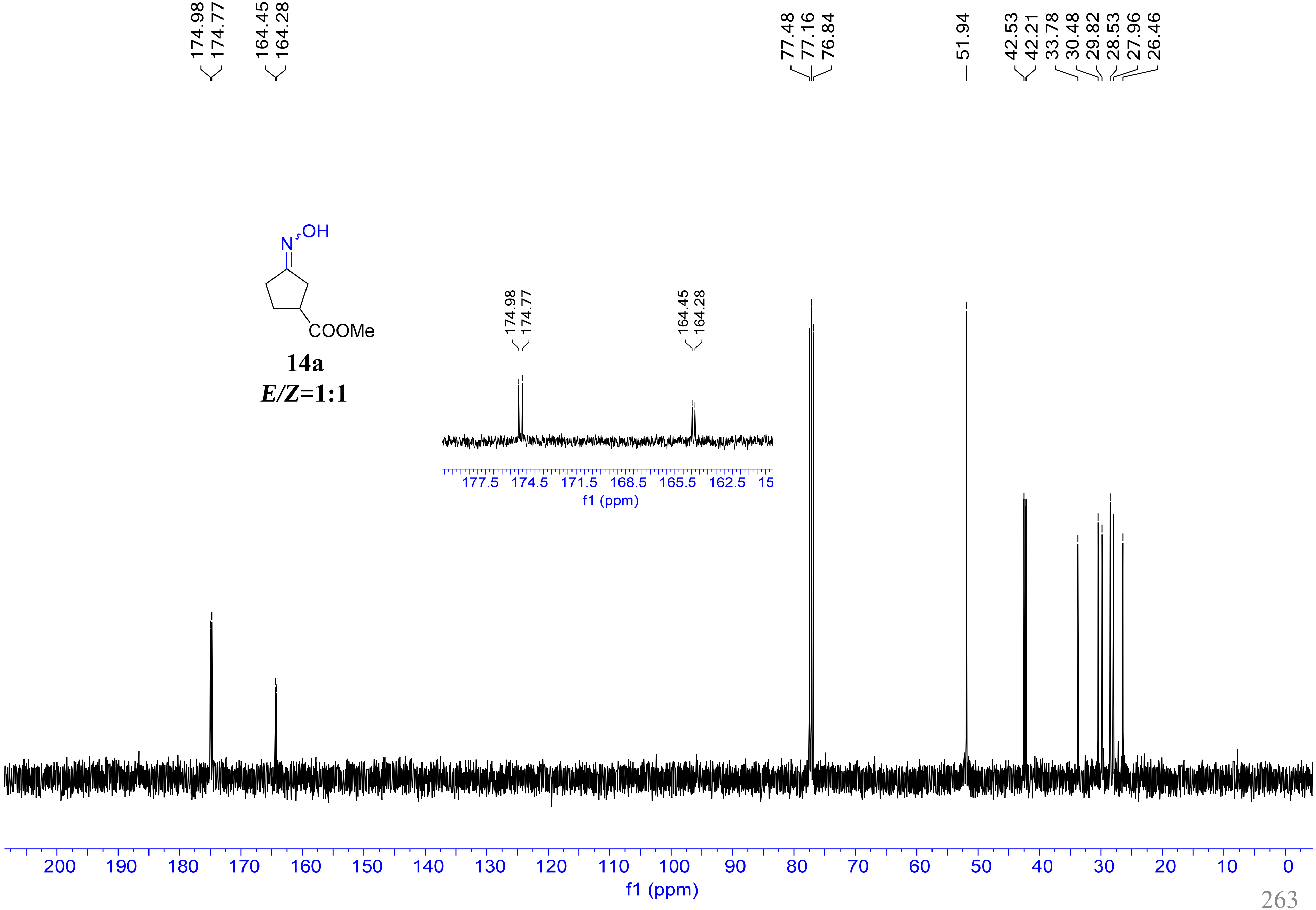

14a

***E*/*Z*=1:1**

^{1}H NMR, 400 MHz, $CDCl_3$

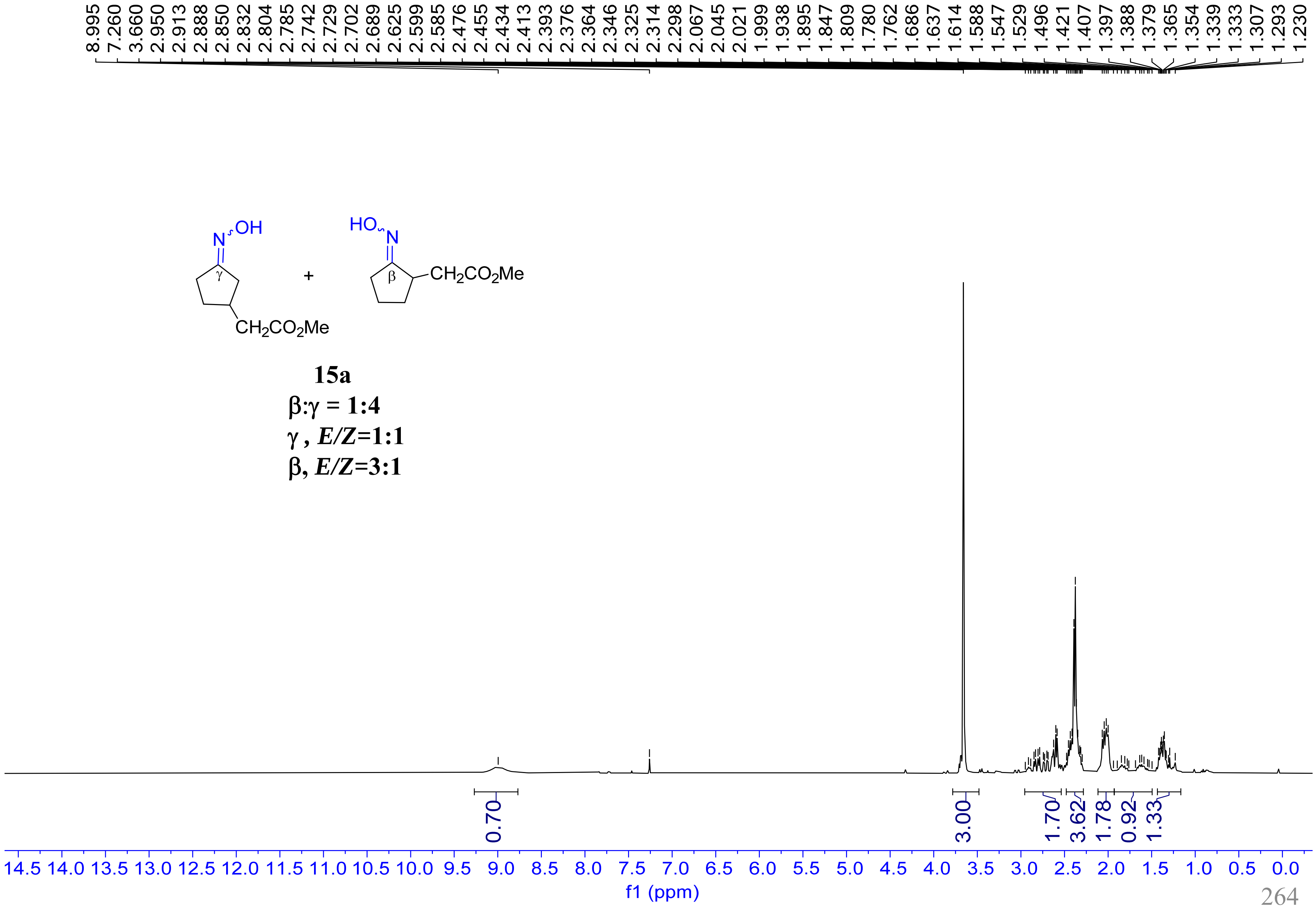

15a

β:γ = 1:4

γ , *E*/*Z*=1:1

β, *E*/*Z*=3:1

^{13}C NMR 101 MHz, $CDCl_3$

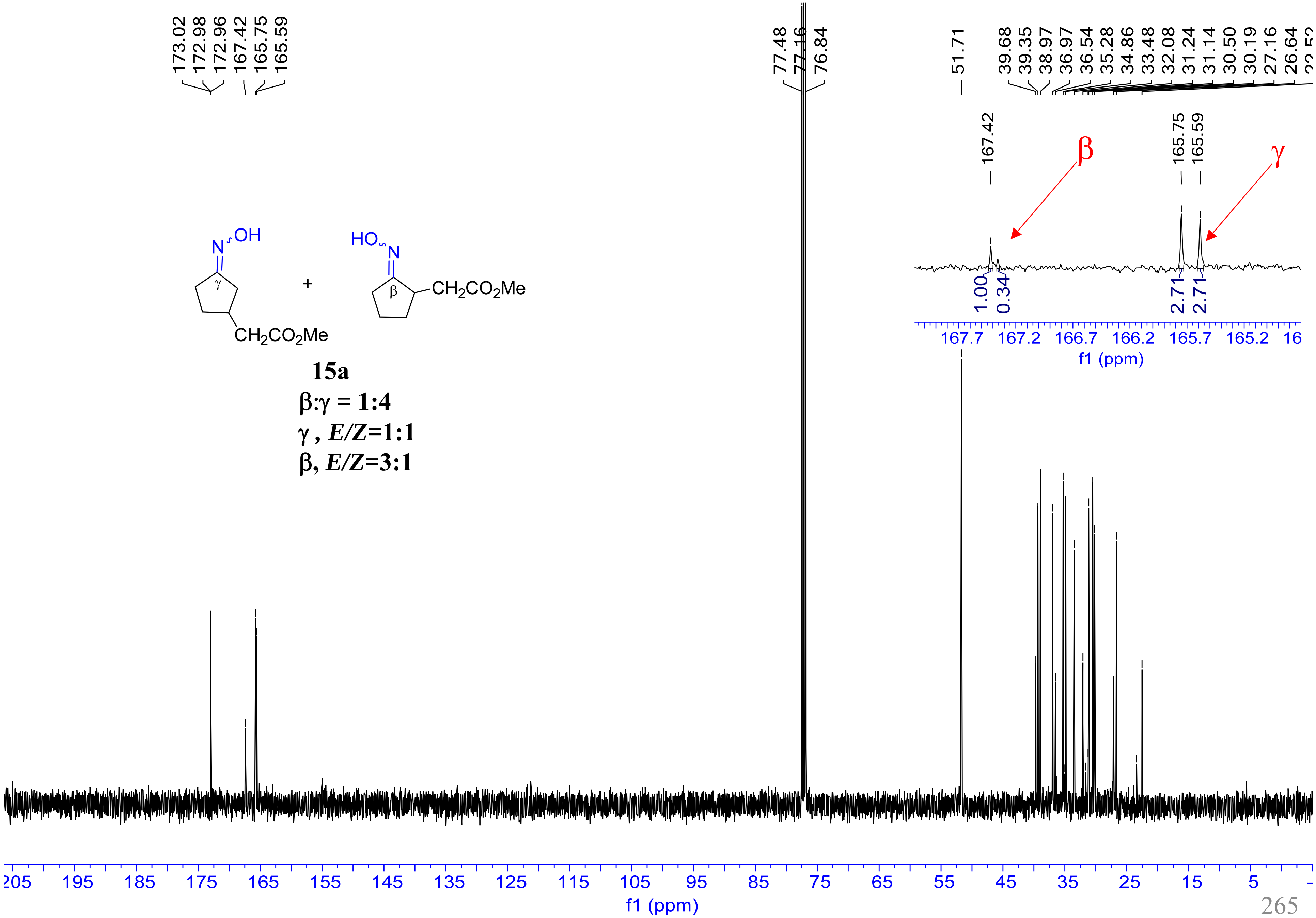

^{1}H NMR, 400 MHz, $CDCl_3$

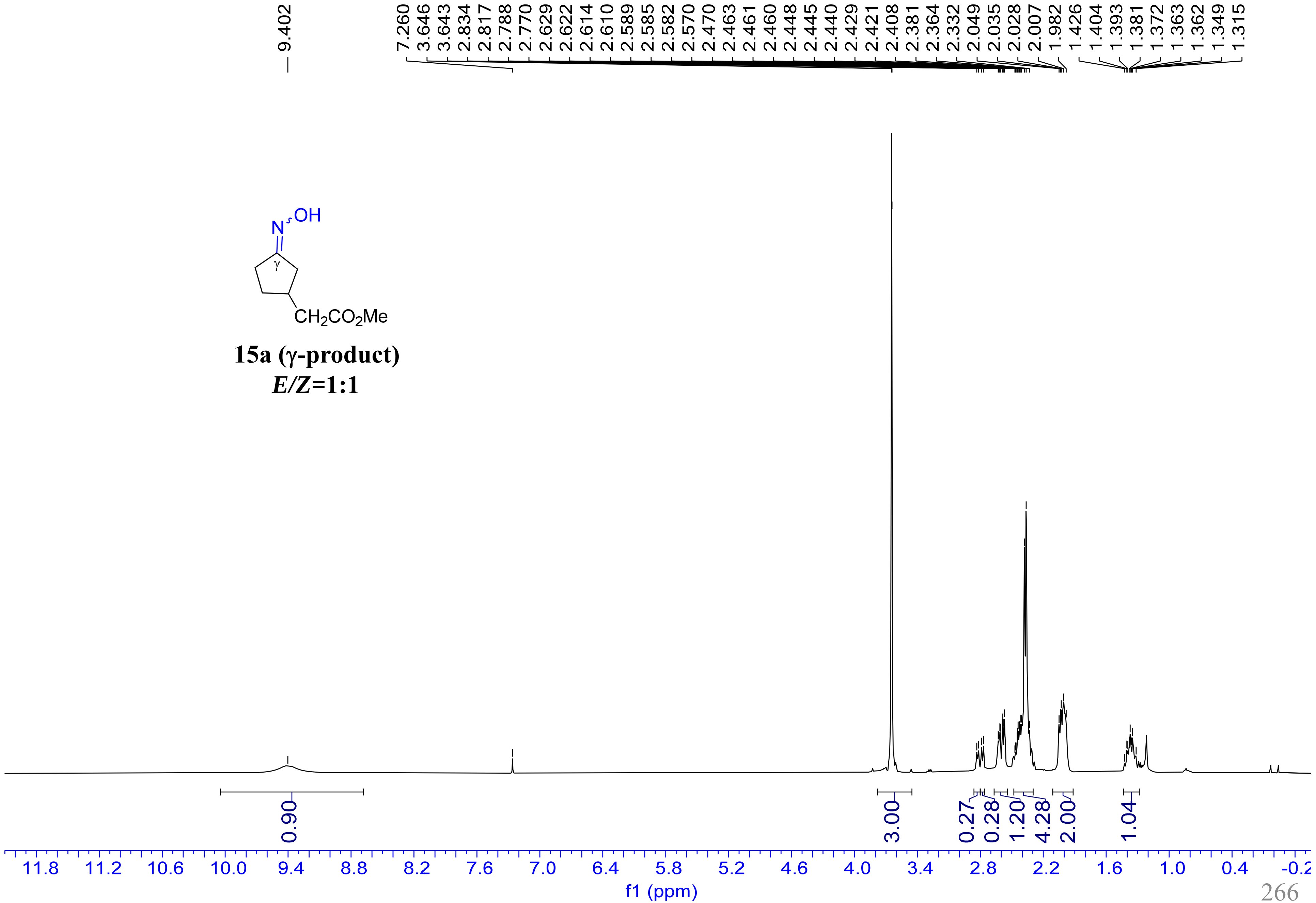

15a (γ-product)
***E*/*Z*=1:1**

^{13}C NMR 101 MHz, $CDCl_3$

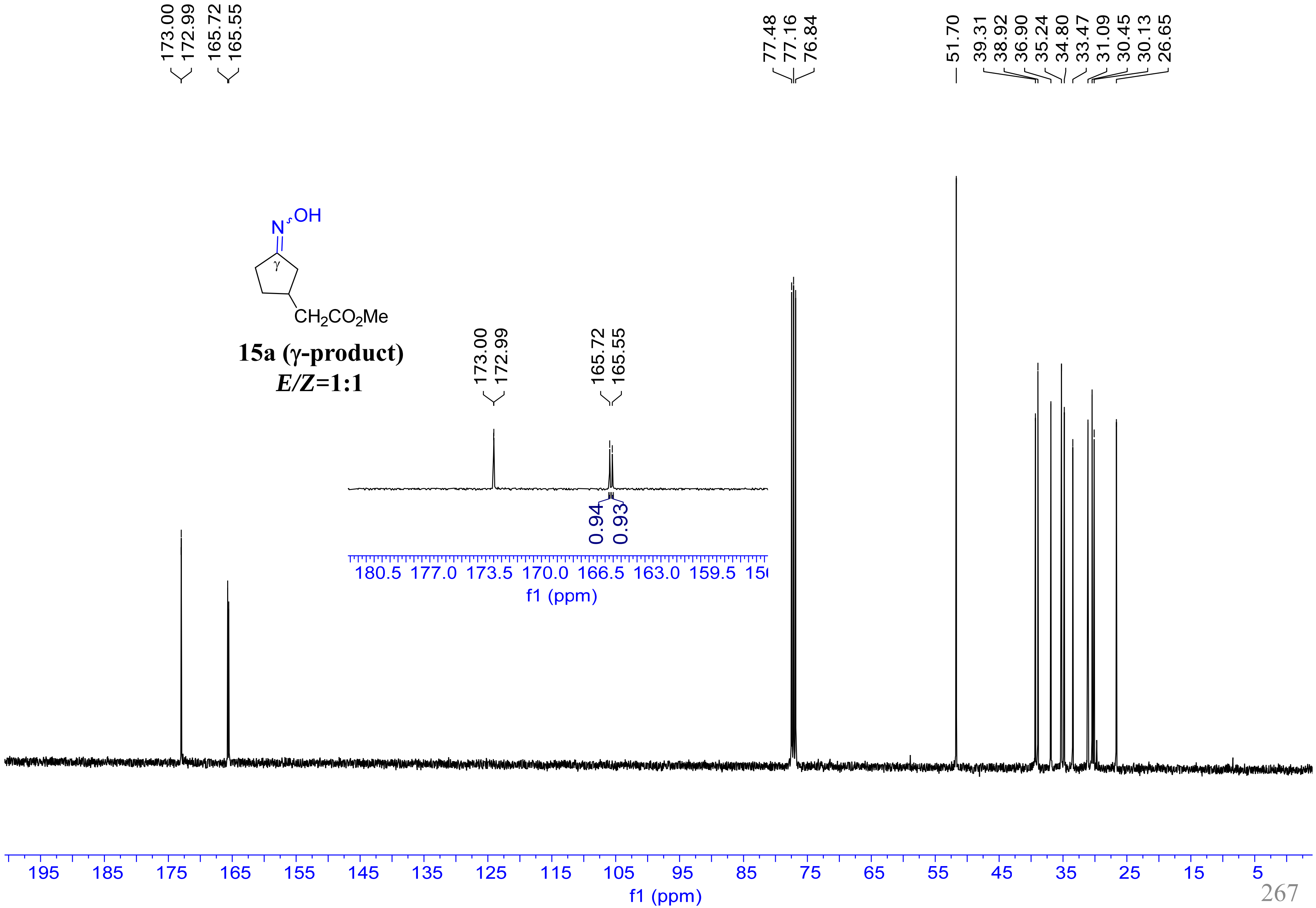

^{1}H NMR, 400 MHz, $CDCl_3$

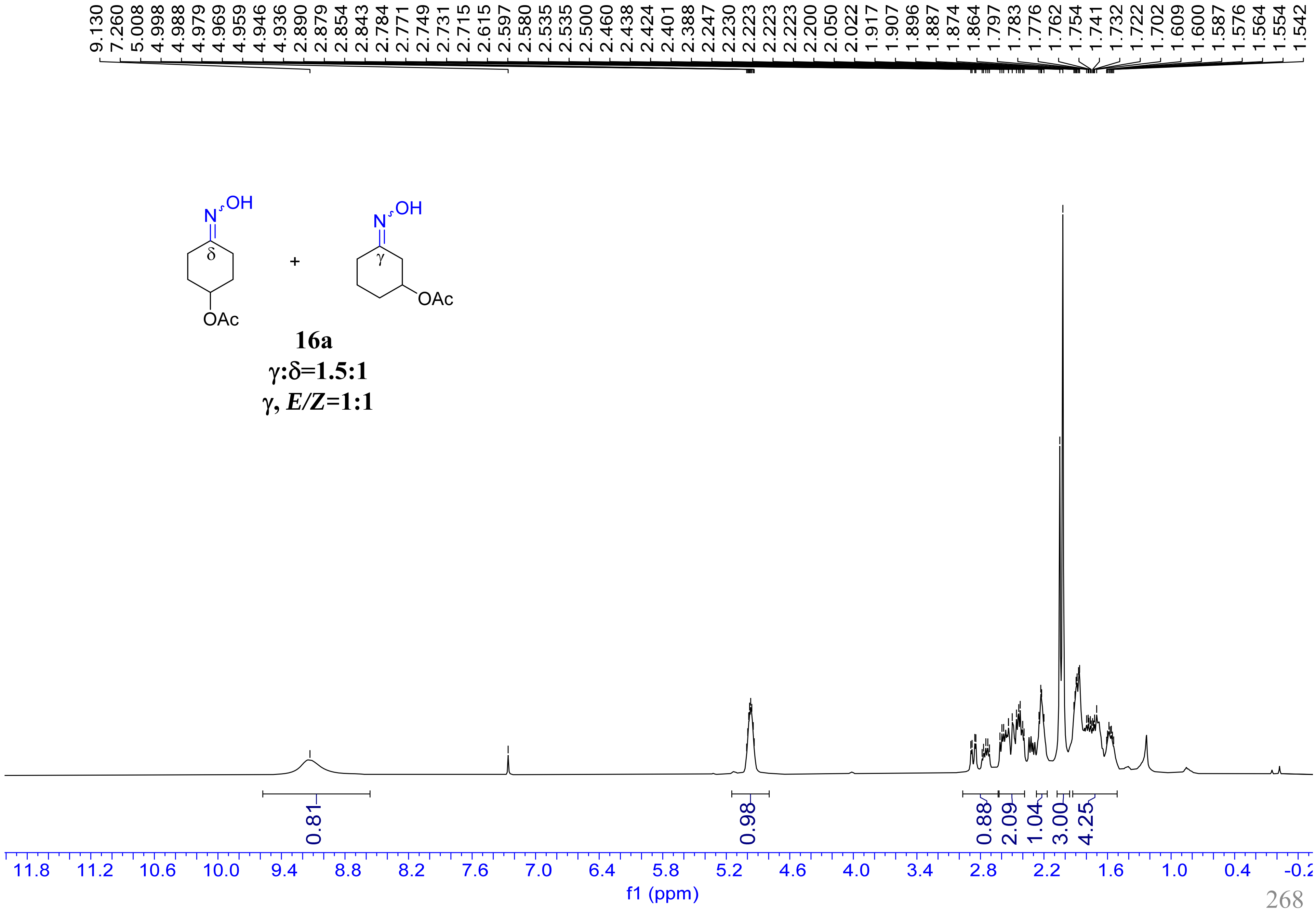

^{13}C NMR 101 MHz, CDCl$_3$

16a

γ:δ=1.5:1

γ, *E*/*Z*=1:1

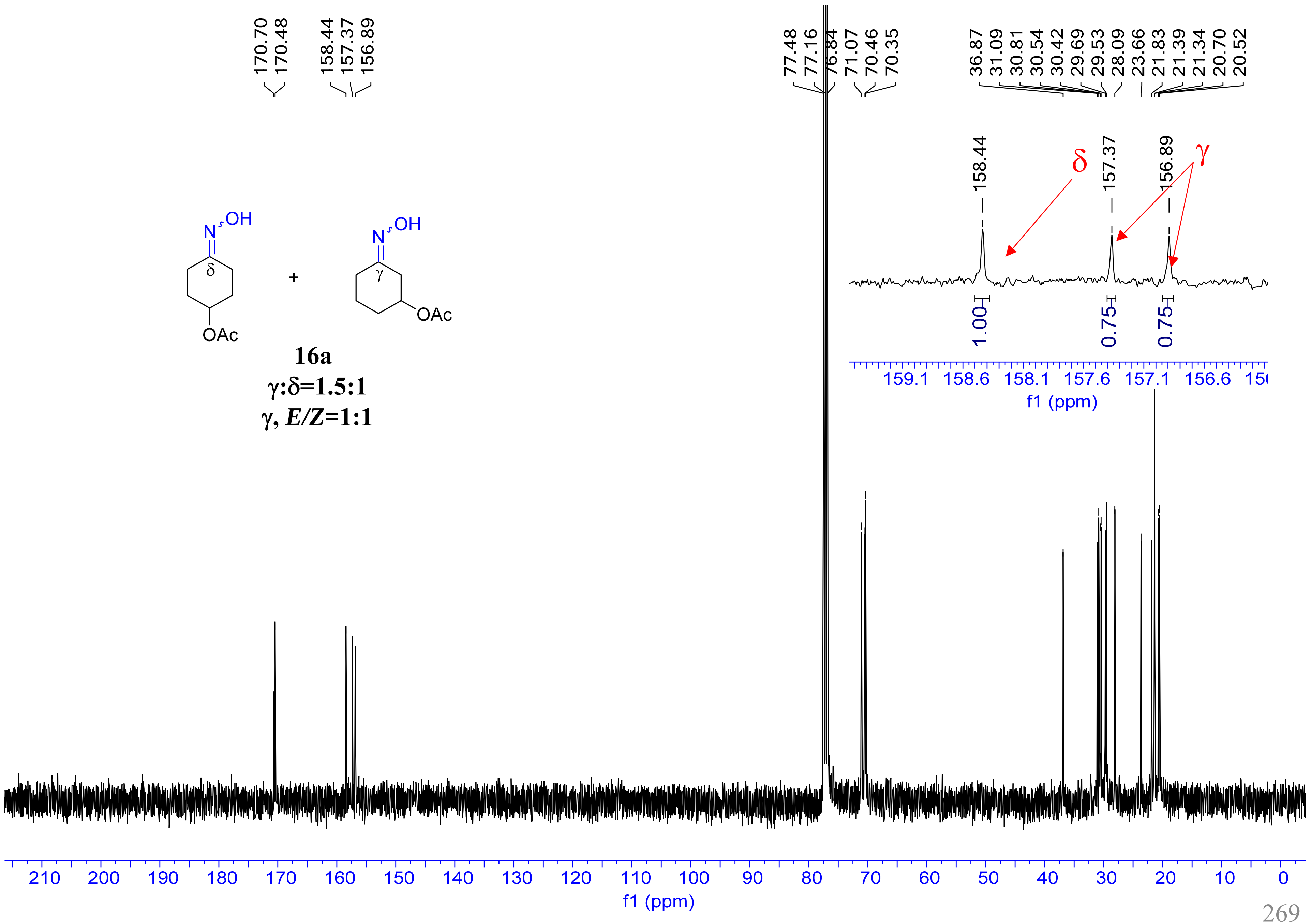

^1H NMR, 400 MHz, $CDCl_3$

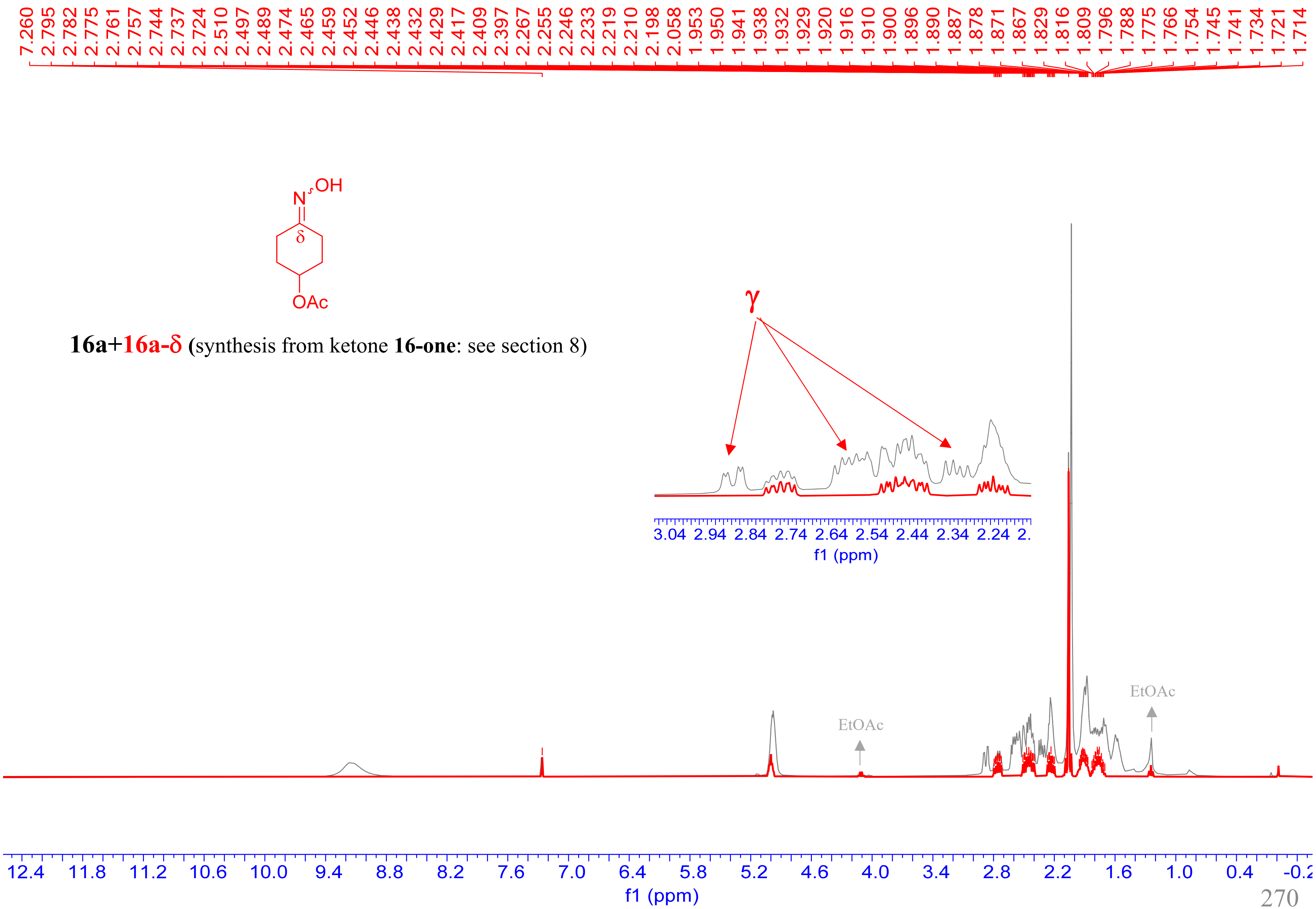

16a+**16a-δ** (synthesis from ketone **16-one**: see section 8)

^{13}C NMR 101 MHz, $CDCl_3$

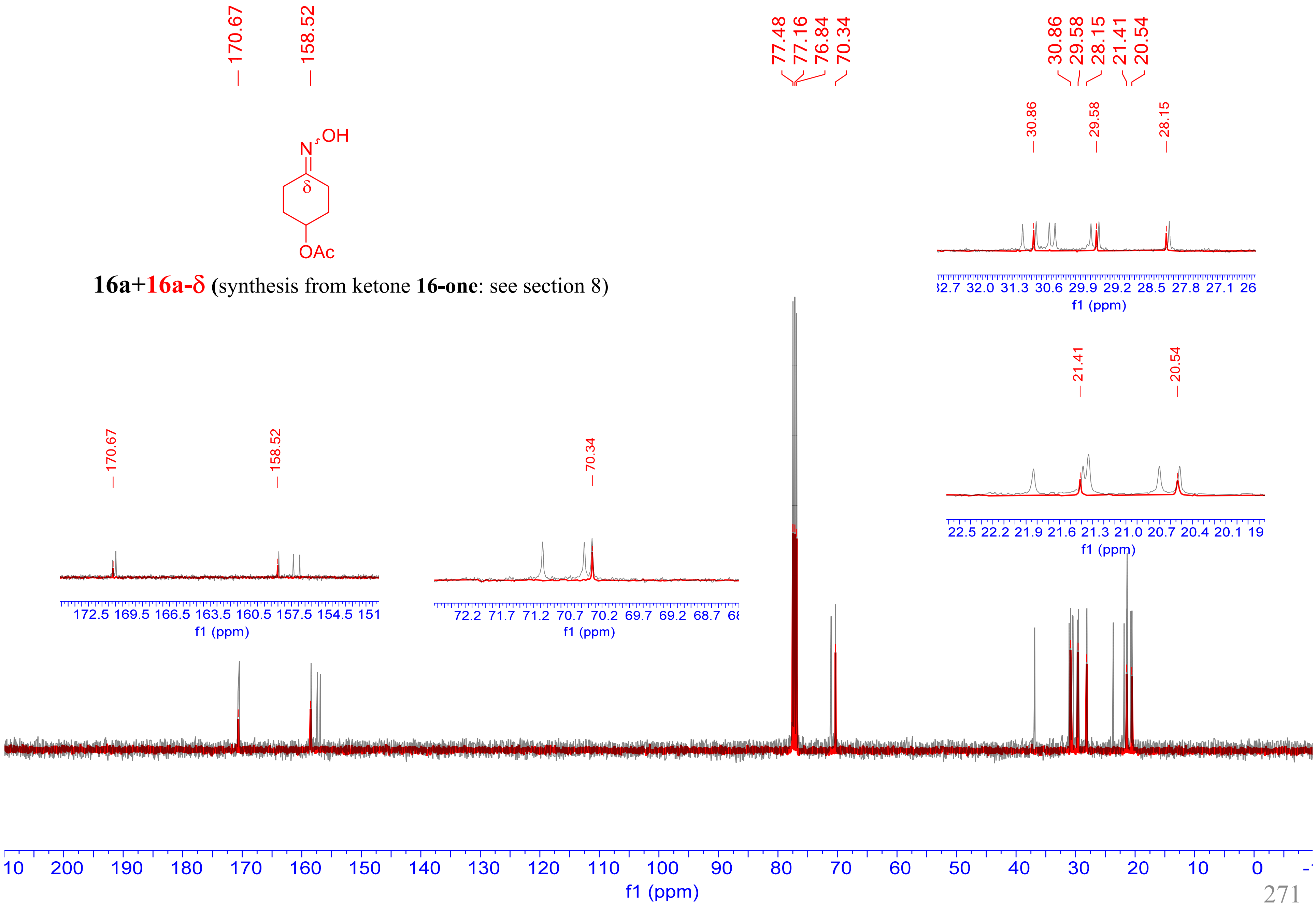

16a+**16a-δ** (synthesis from ketone **16-one**: see section 8)

^{1}H NMR, 400 MHz, $CDCl_3$

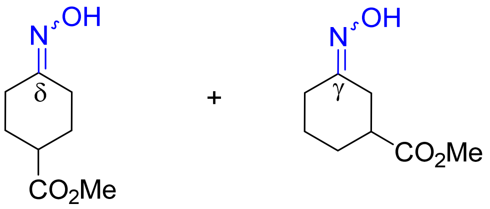

17a

γ:δ=2.7:1

γ, *E/Z*=1:1

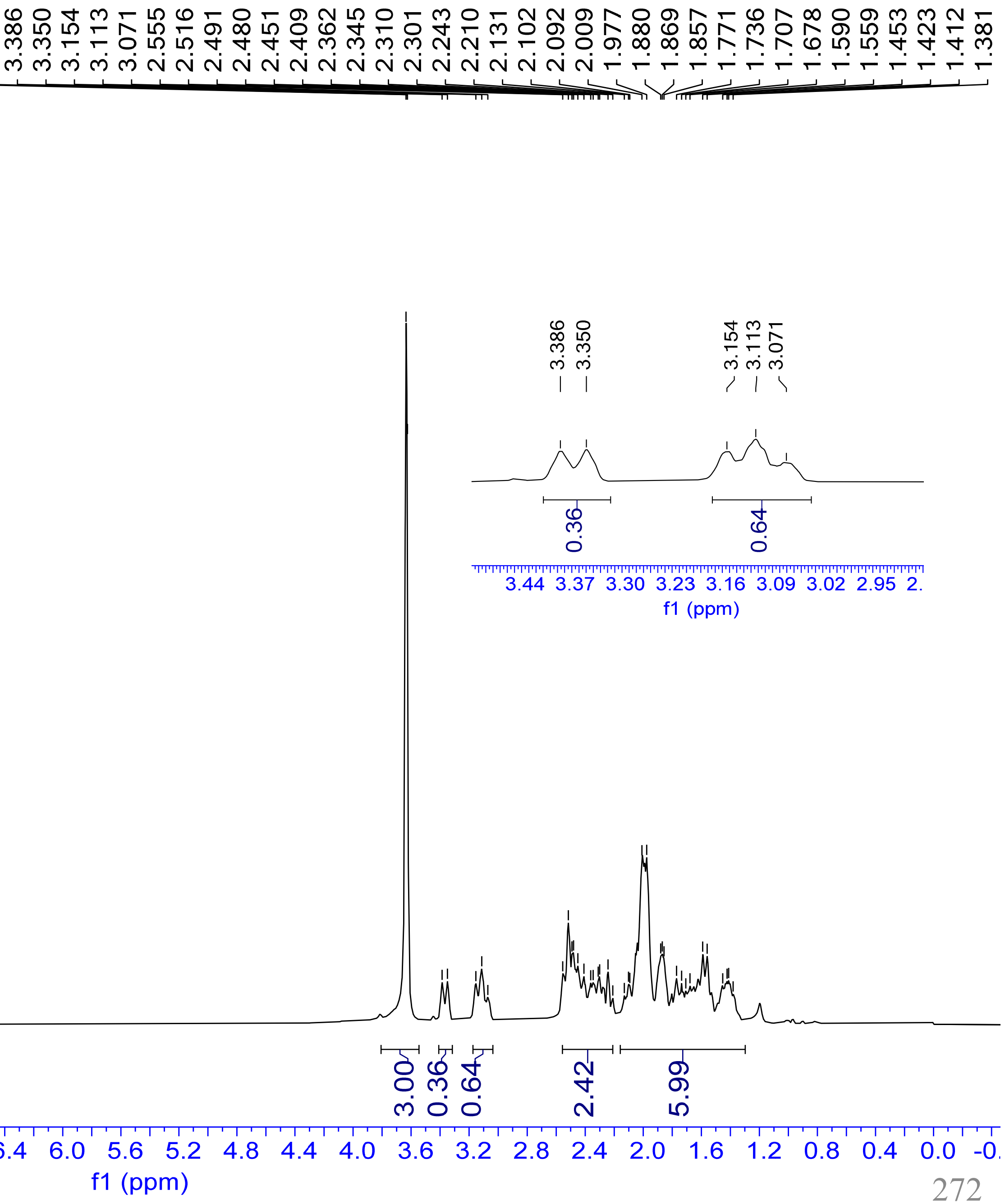

^{13}C NMR 101 MHz, $CDCl_3$

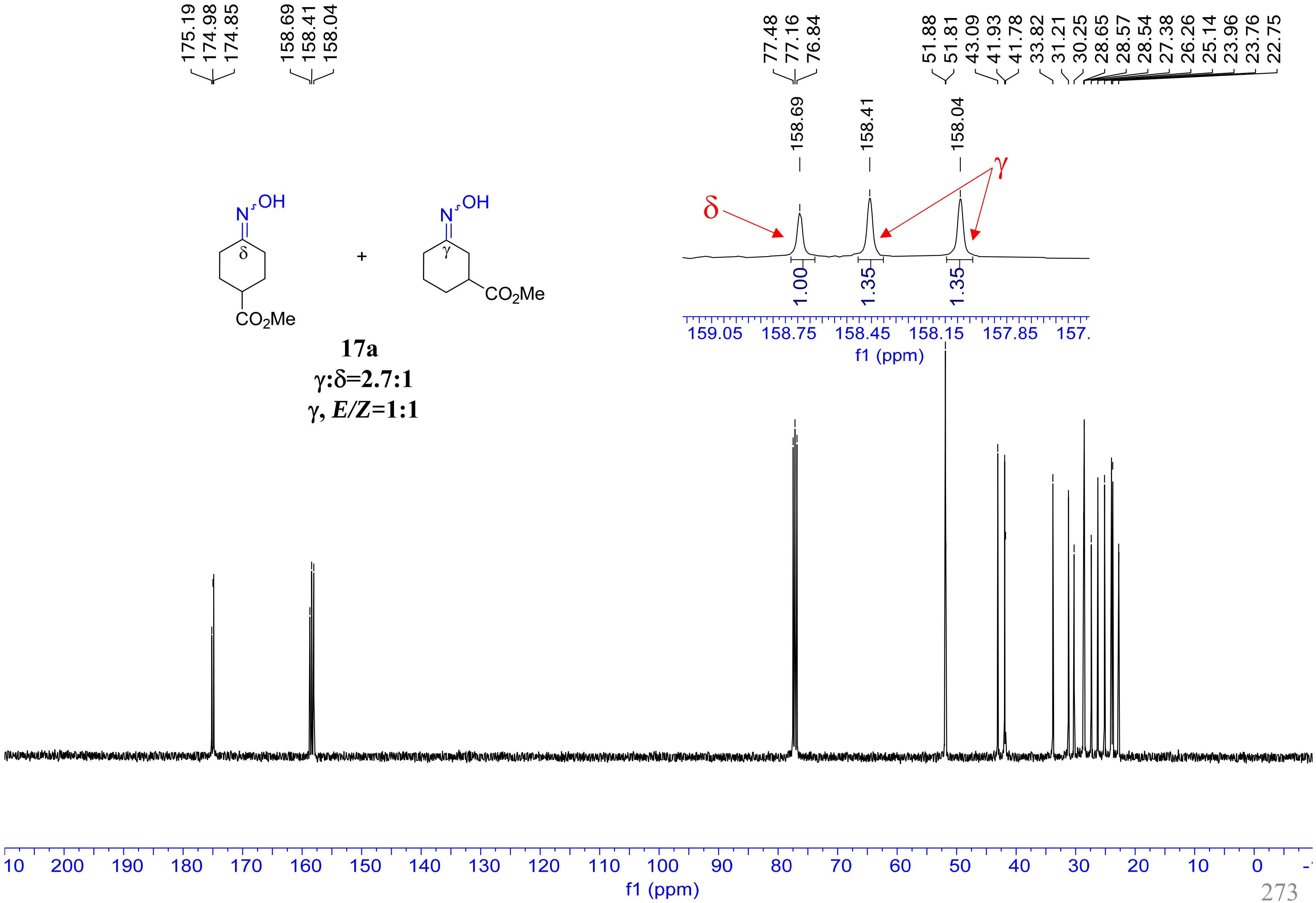

17a

γ:δ=2.7:1

γ, *E/Z*=1:1

^{1}H NMR, 400 MHz, $CDCl_3$

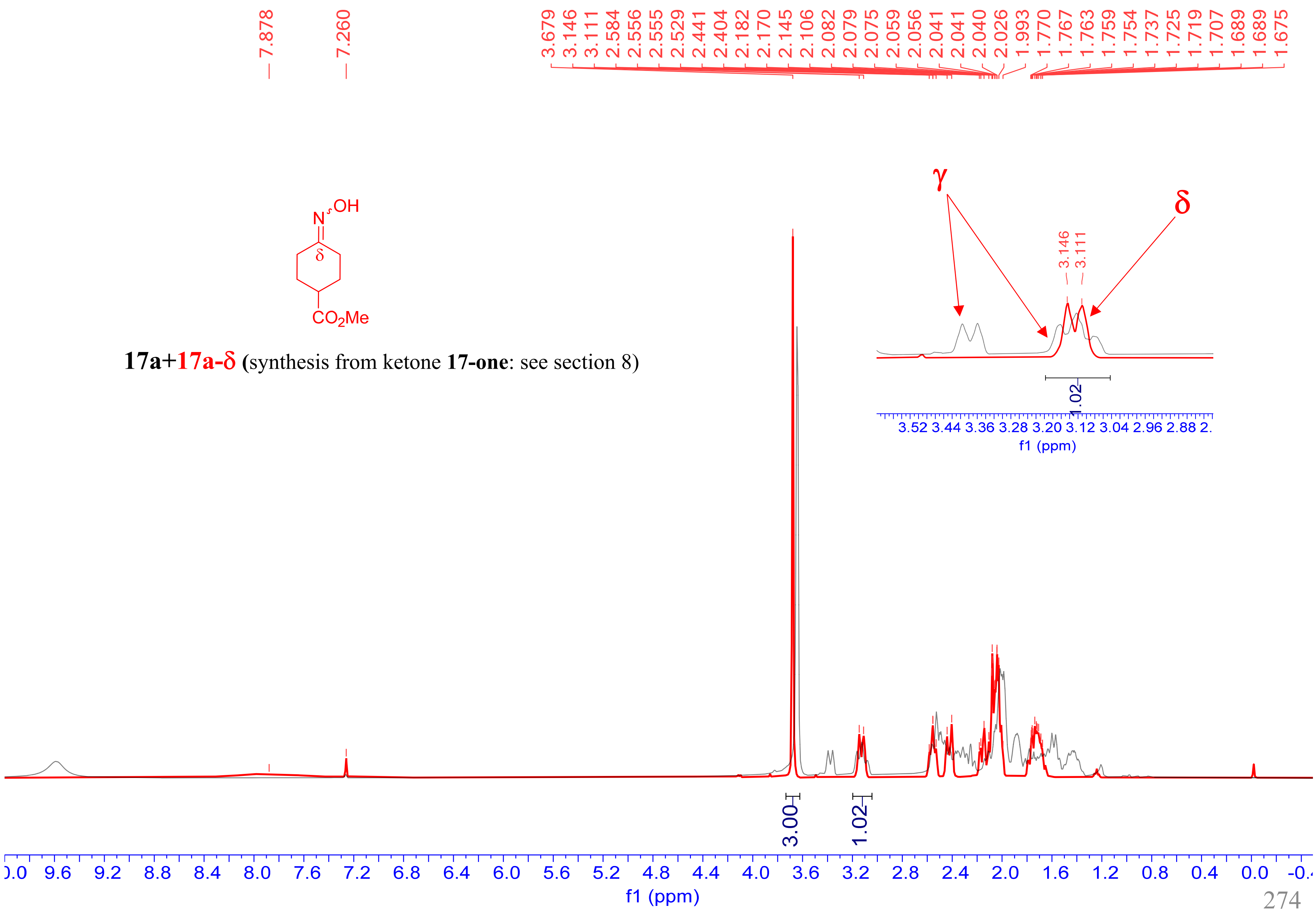

17a+**17a-δ** (synthesis from ketone **17-one**: see section 8)

^{13}C NMR 101 MHz, $CDCl_3$

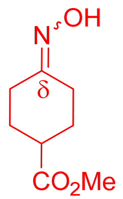

17a+**17a-δ** (synthesis from ketone **17-one**: see section 8)

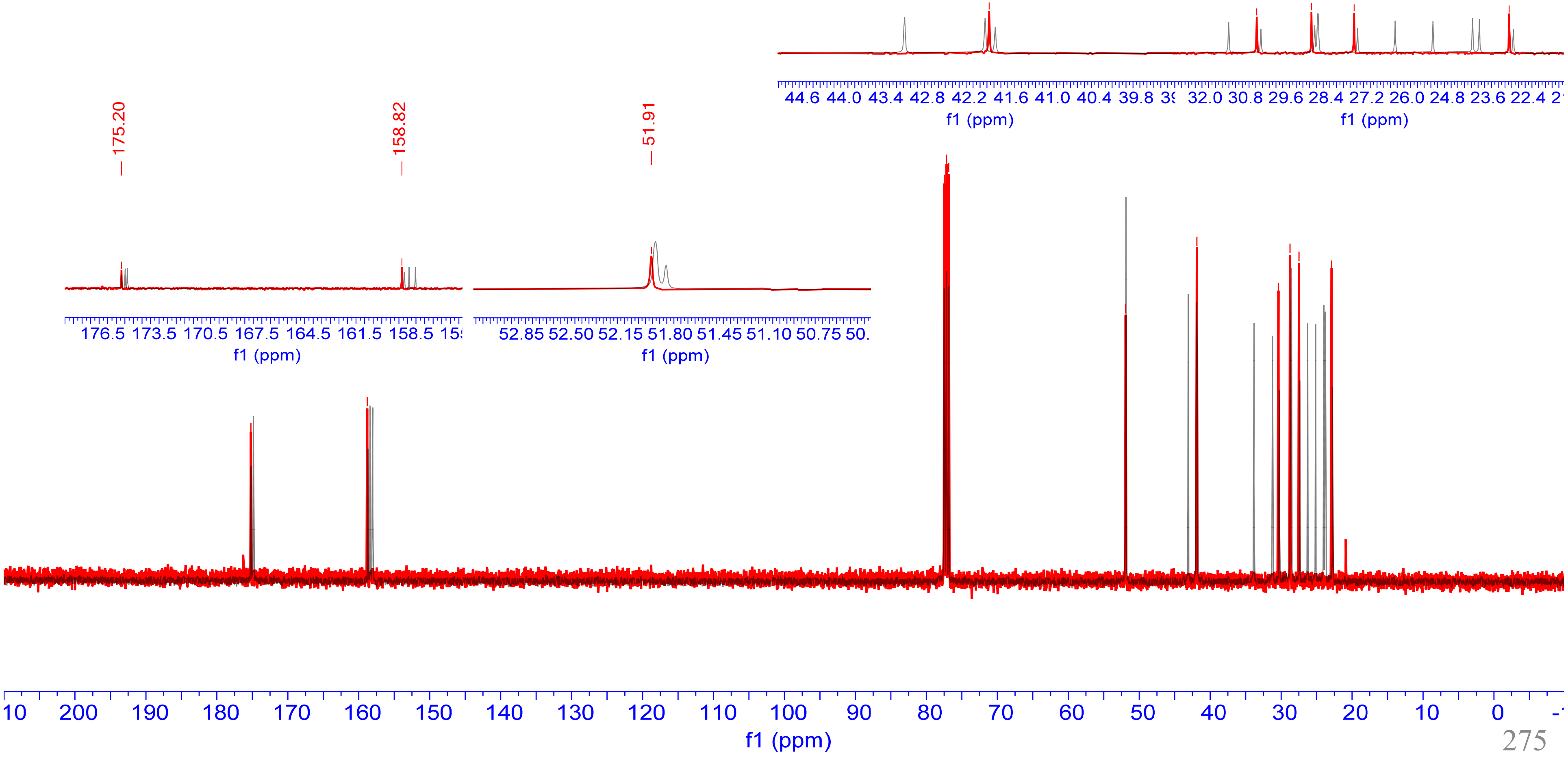

^{1}H NMR, 400 MHz, $CDCl_3$

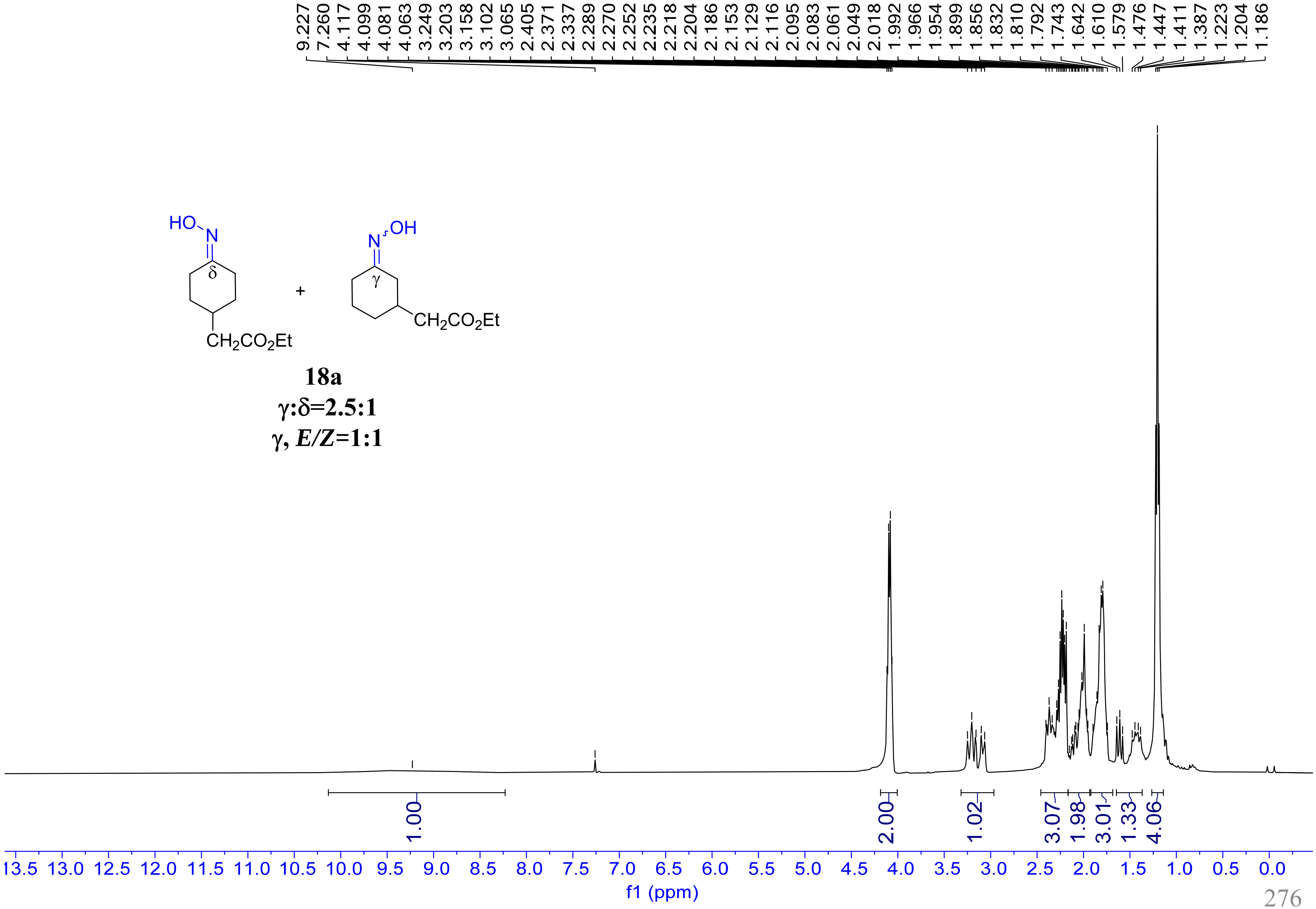

^{13}C NMR 101 MHz, $CDCl_3$

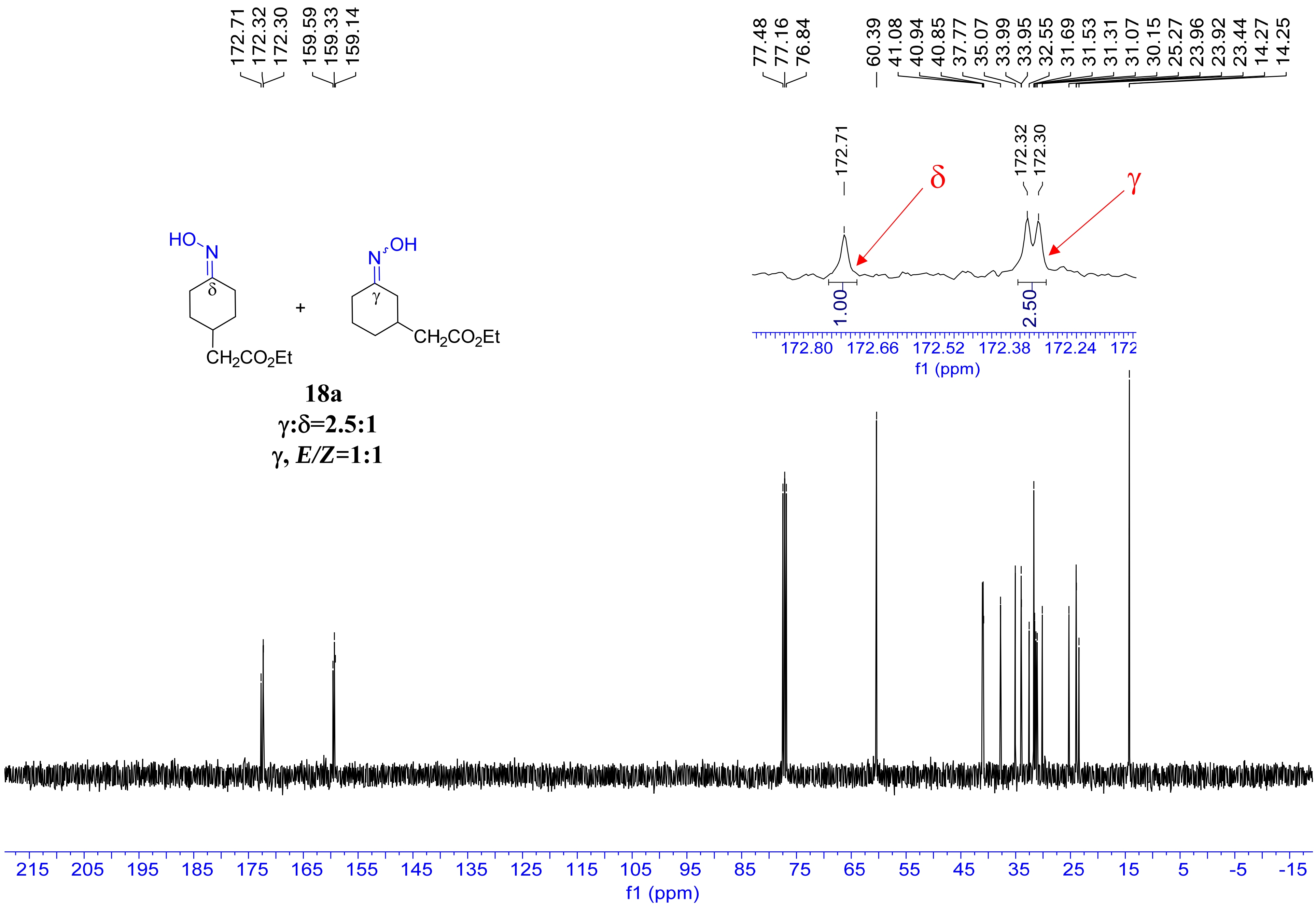

^{1}H NMR, 400 MHz, $CDCl_3$

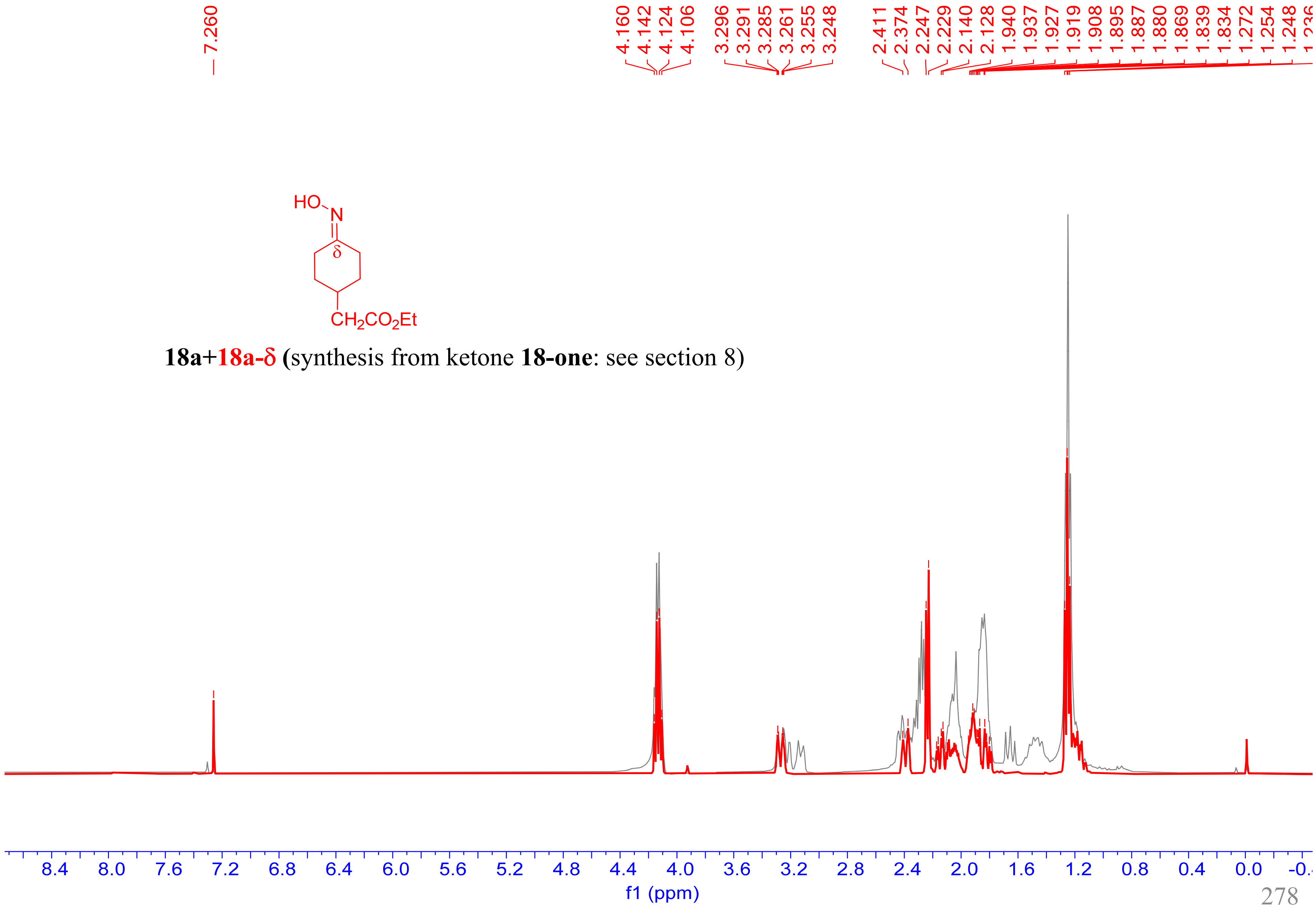

18a+**18a-δ** (synthesis from ketone **18-one**: see section 8)

^{13}C NMR 101 MHz, $CDCl_3$

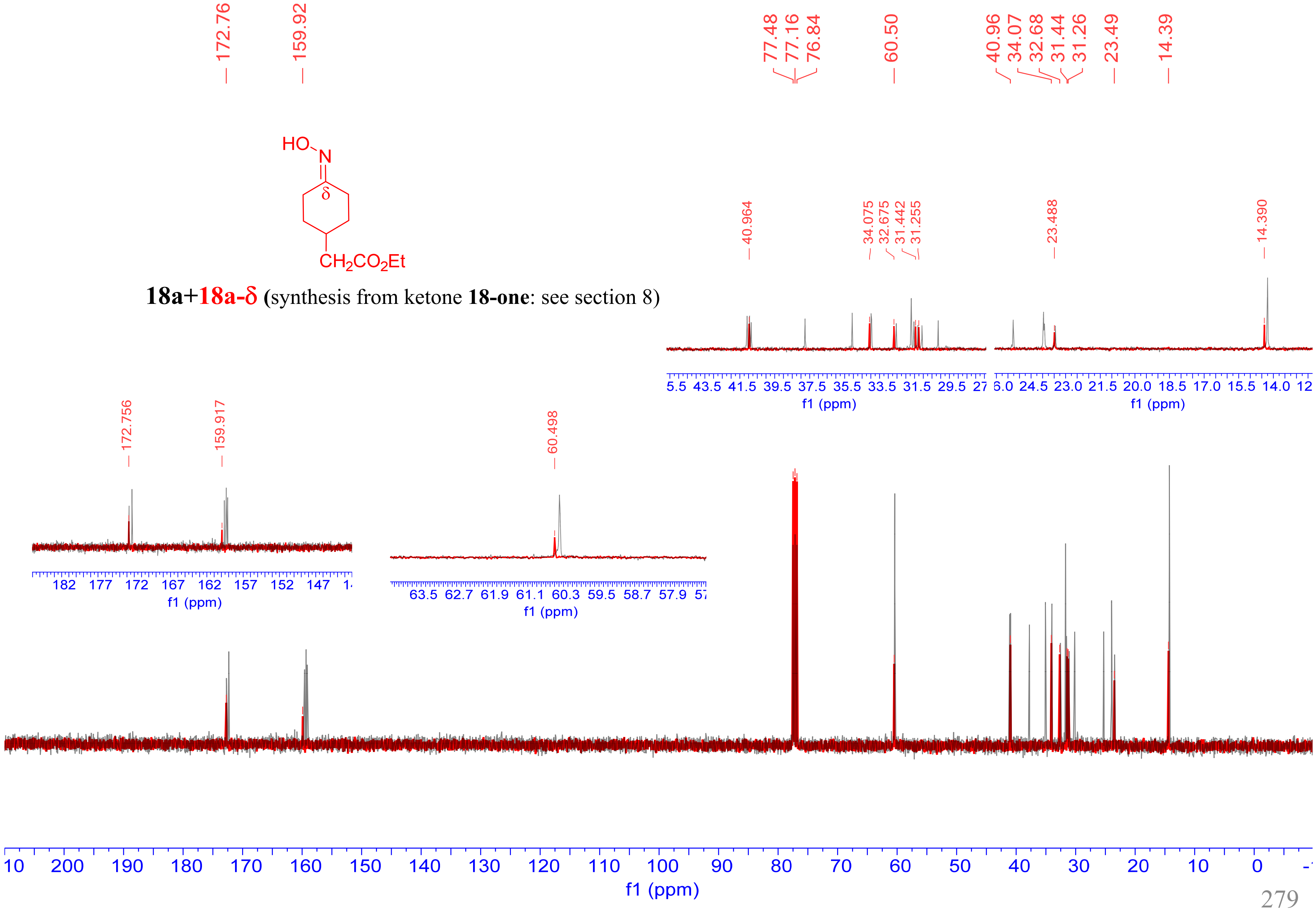

18a+**18a-δ** (synthesis from ketone **18-one**: see section 8)

^1H NMR, 400 MHz, $CDCl_3$

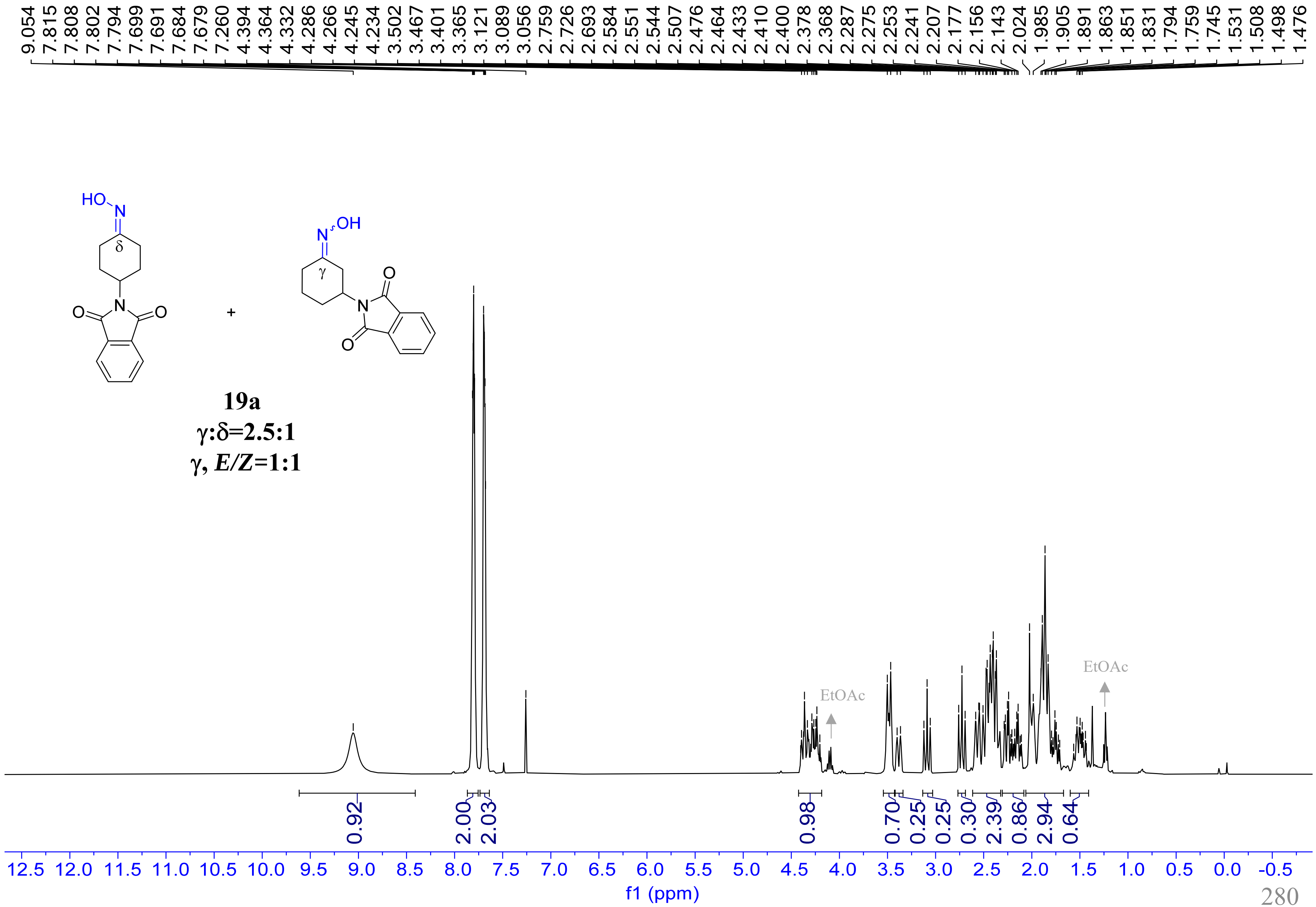

^{13}C NMR 101 MHz, $CDCl_3$

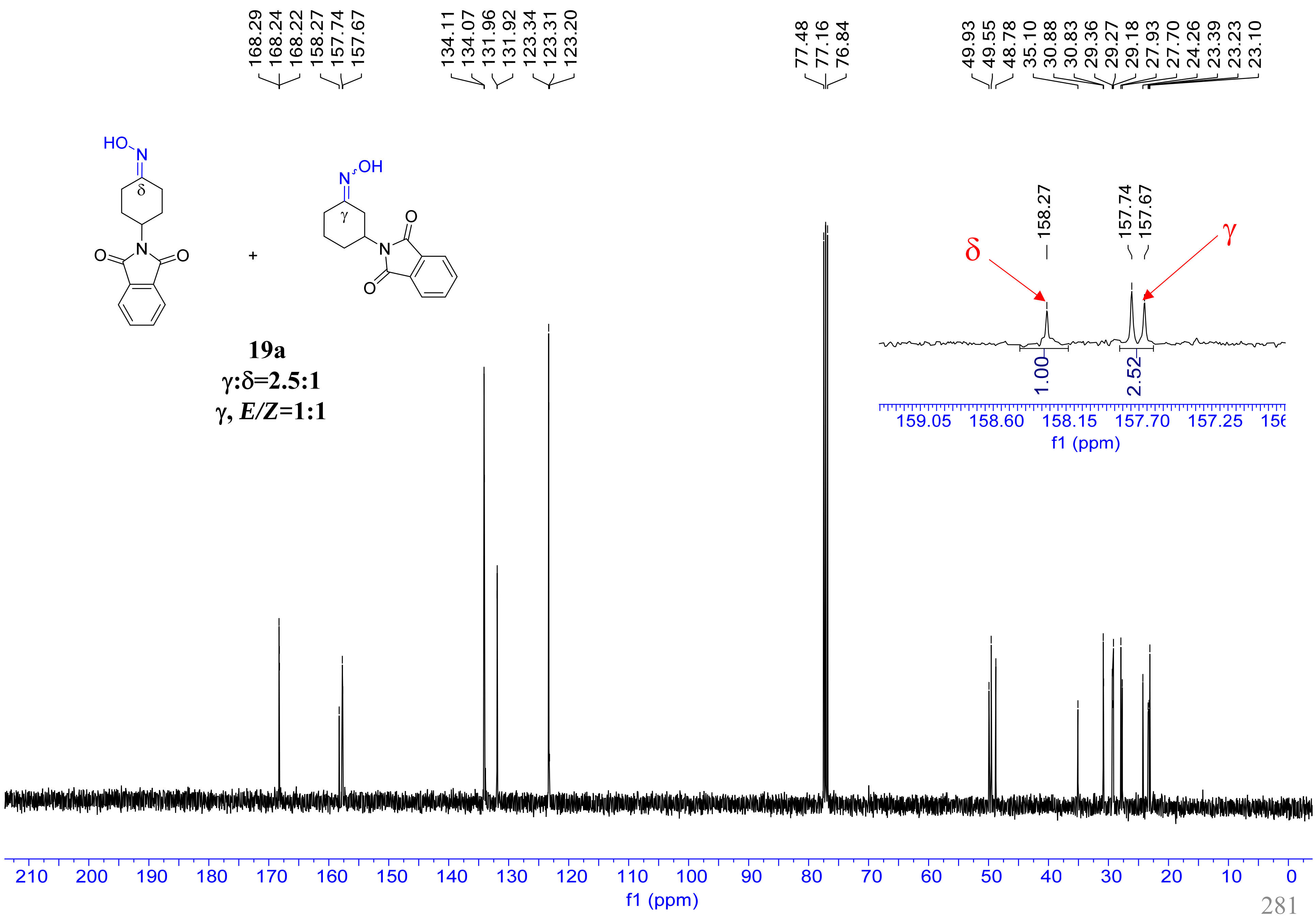

^1H NMR, 400 MHz, $CDCl_3$

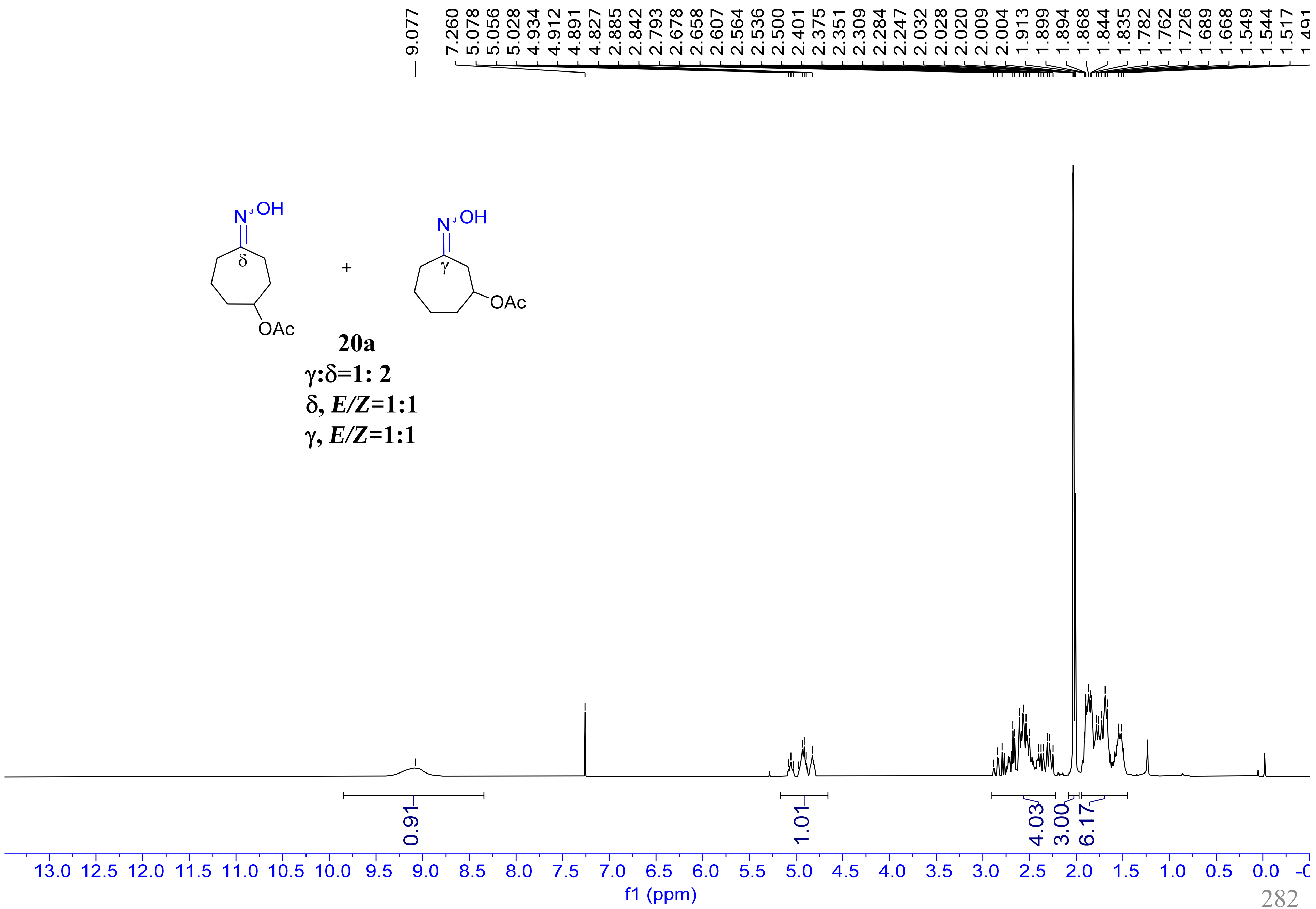

^{13}C NMR 101 MHz, $CDCl_3$

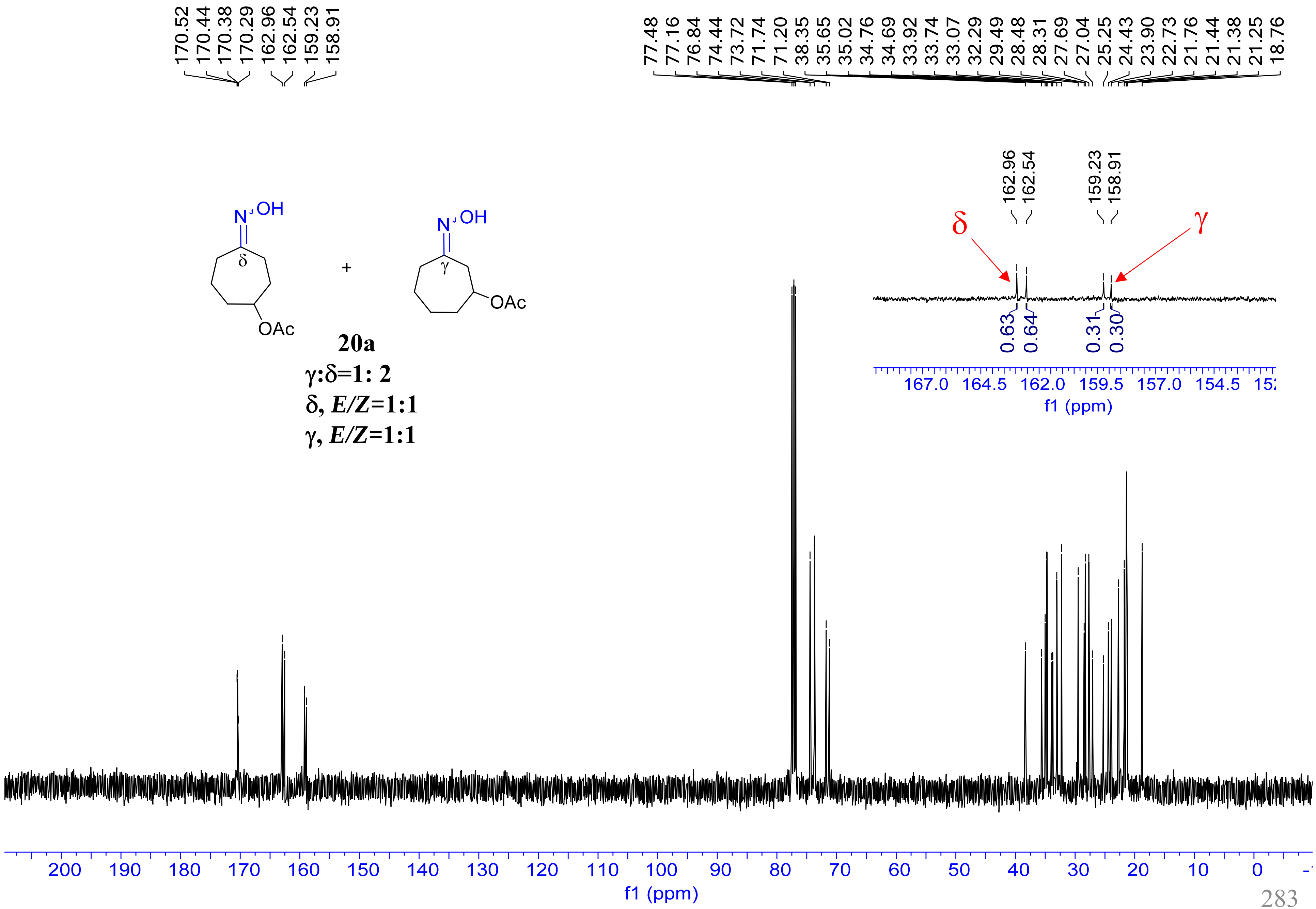

20a

γ:δ=1: 2

δ, *E/Z*=1:1

γ, *E/Z*=1:1

^{1}H NMR, 400 MHz, $CDCl_3$

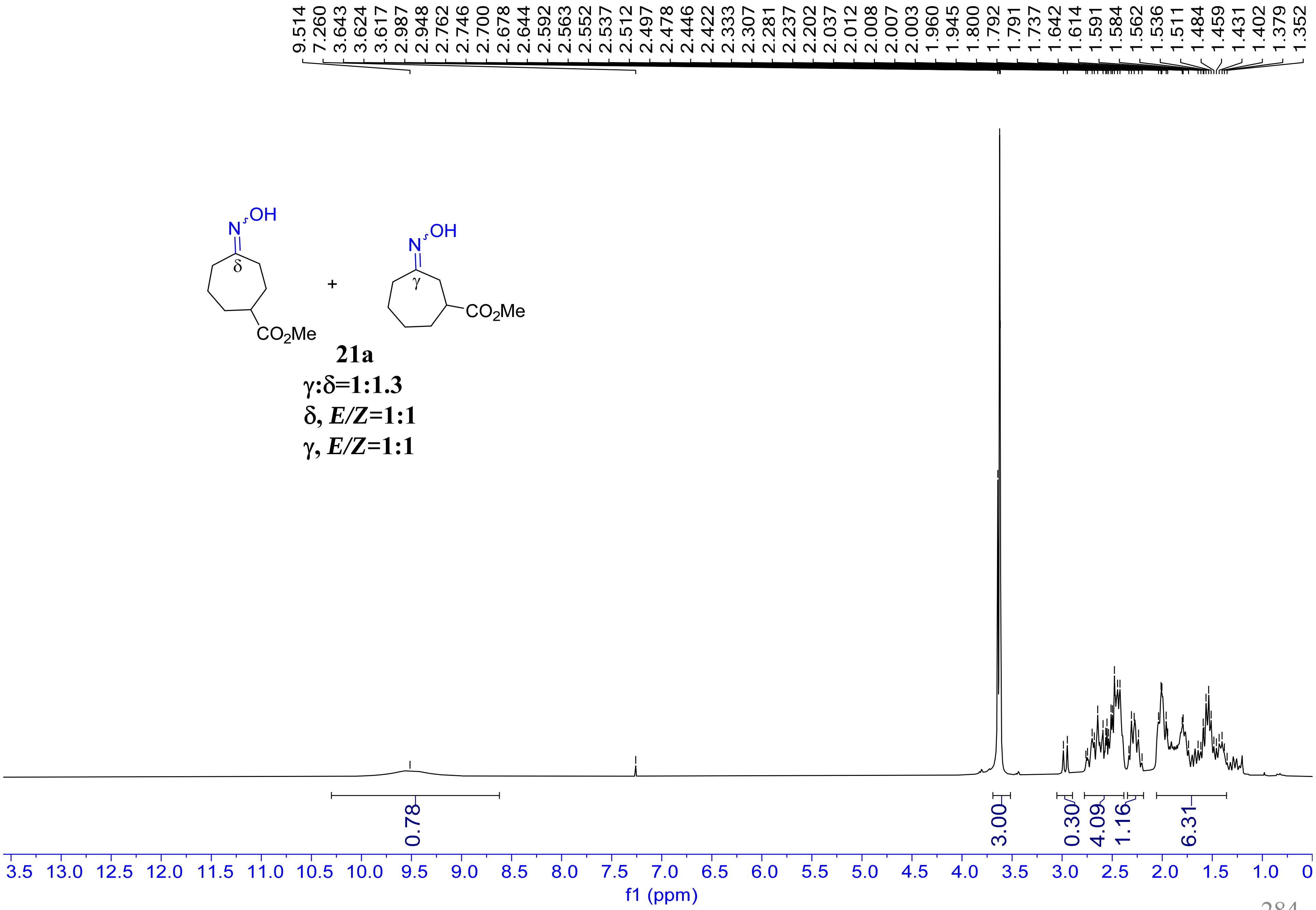

^{13}C NMR 101 MHz, $CDCl_3$

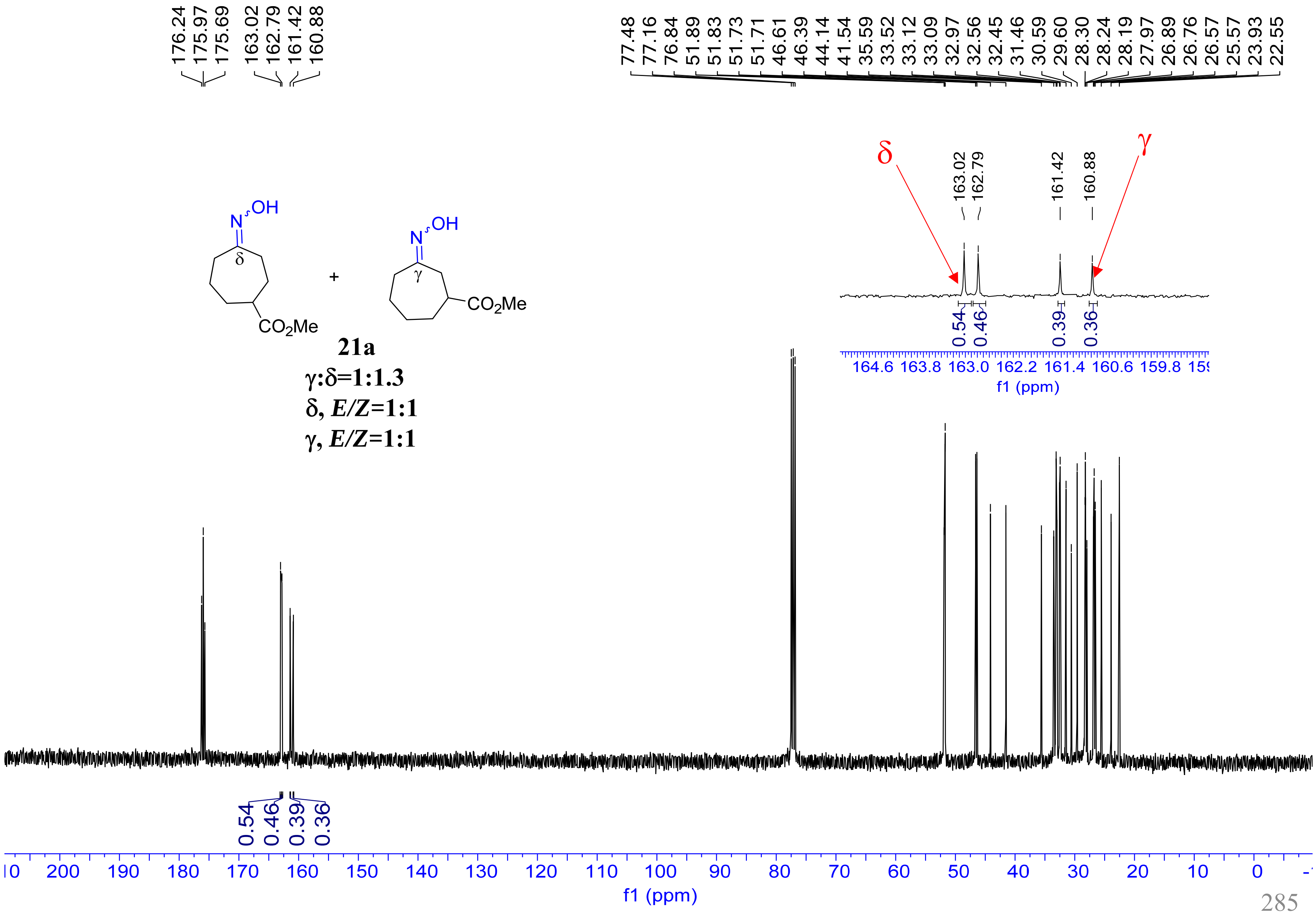

^1H NMR, 400 MHz, $CDCl_3$

22a (δ-product)
***E/Z*=1:1**

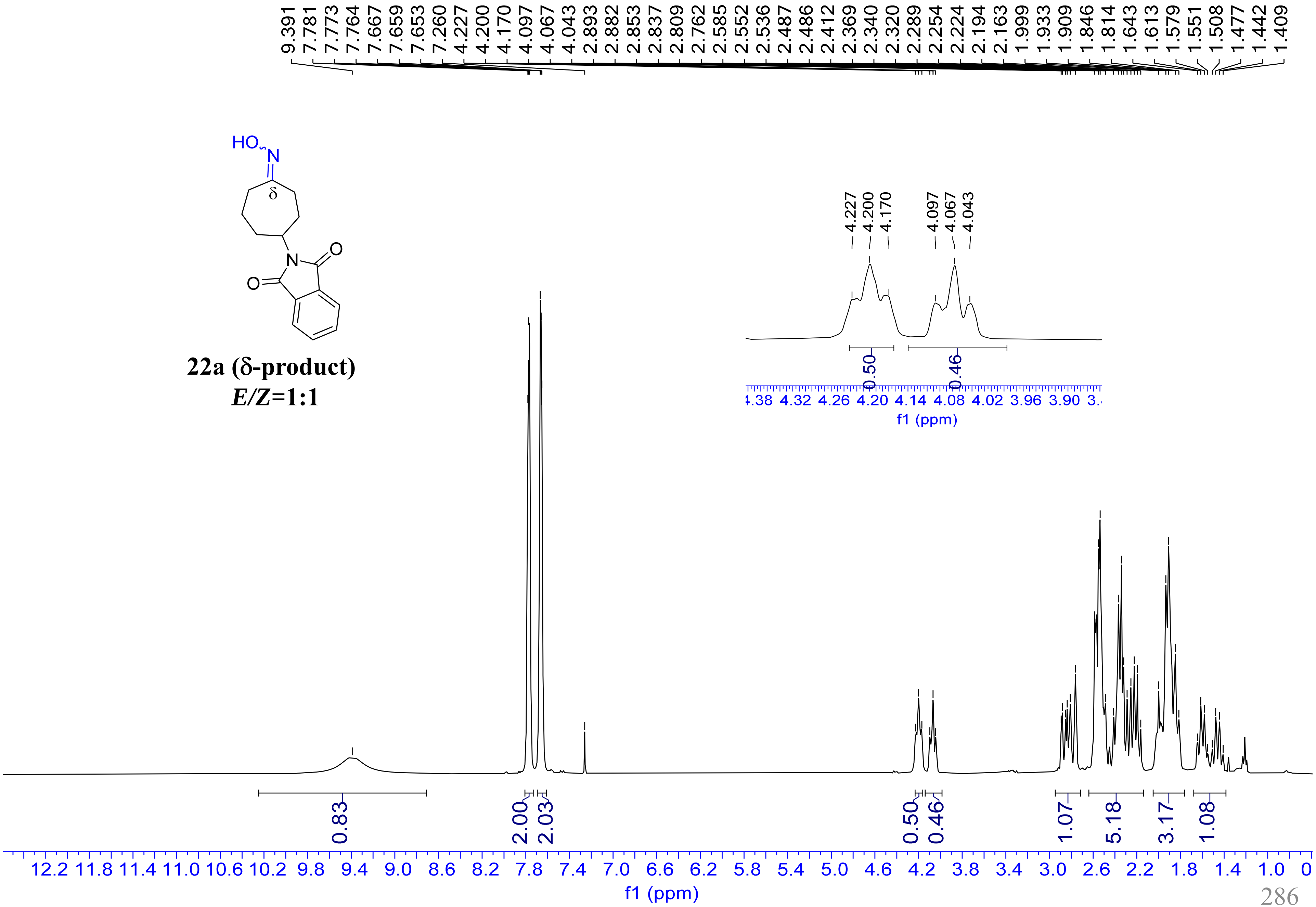

^{13}C NMR 101 MHz, CDCl$_3$

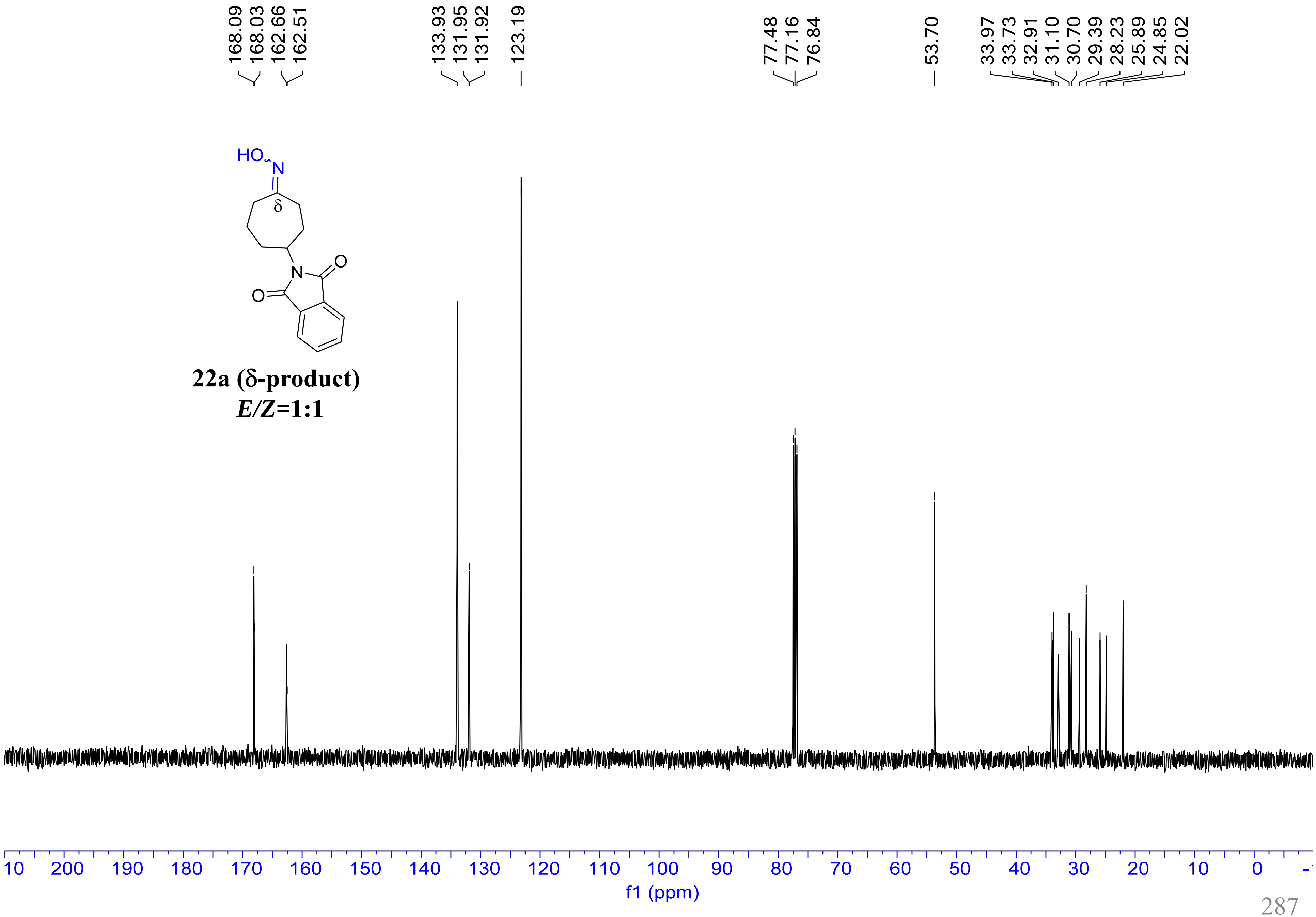

22a (δ-product)
***E*/*Z*=1:1**

^{1}H NMR, 400 MHz, $CDCl_3$

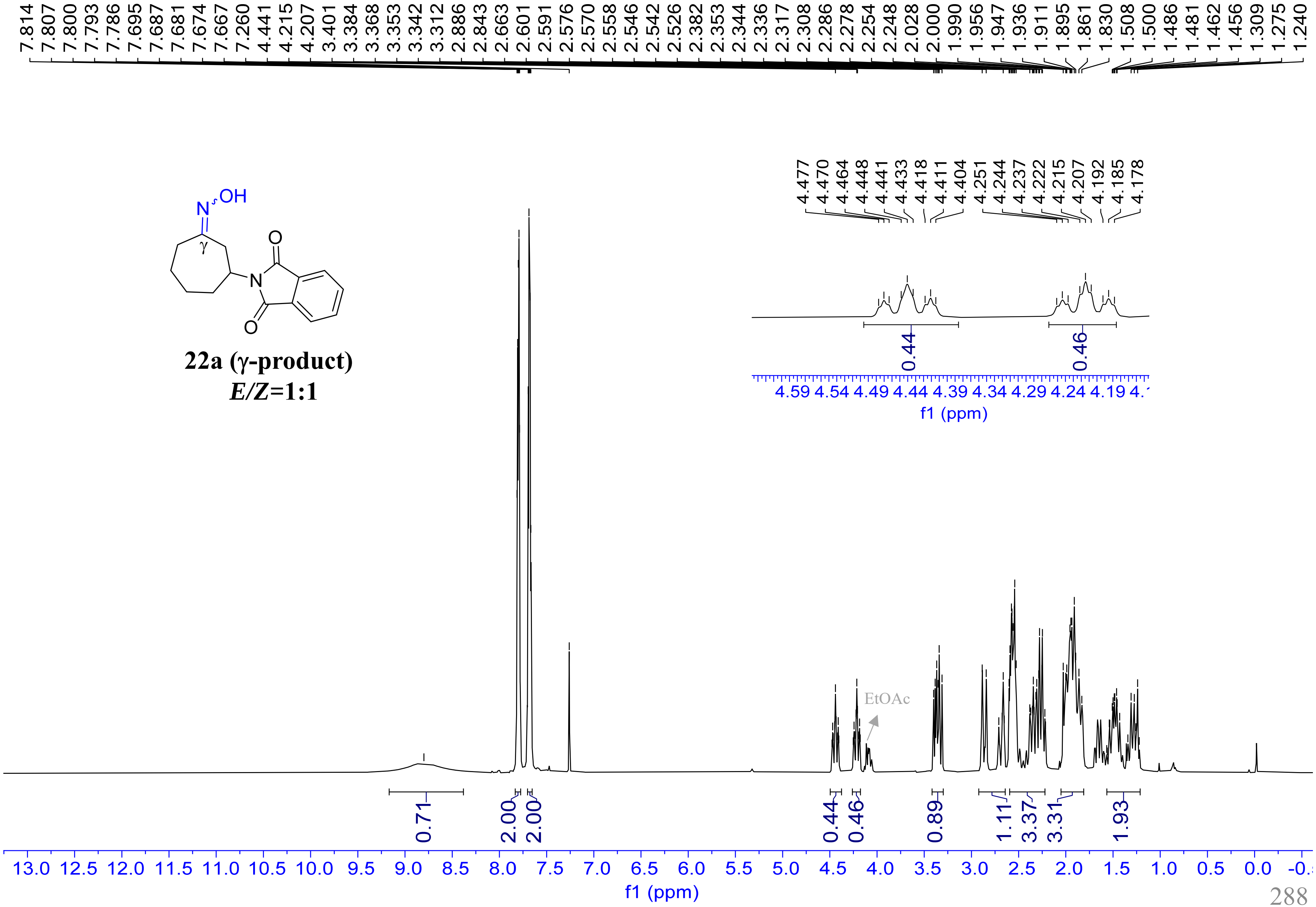

22a (γ-product)
***E/Z*=1:1**

^{13}C NMR 101 MHz, $CDCl_3$

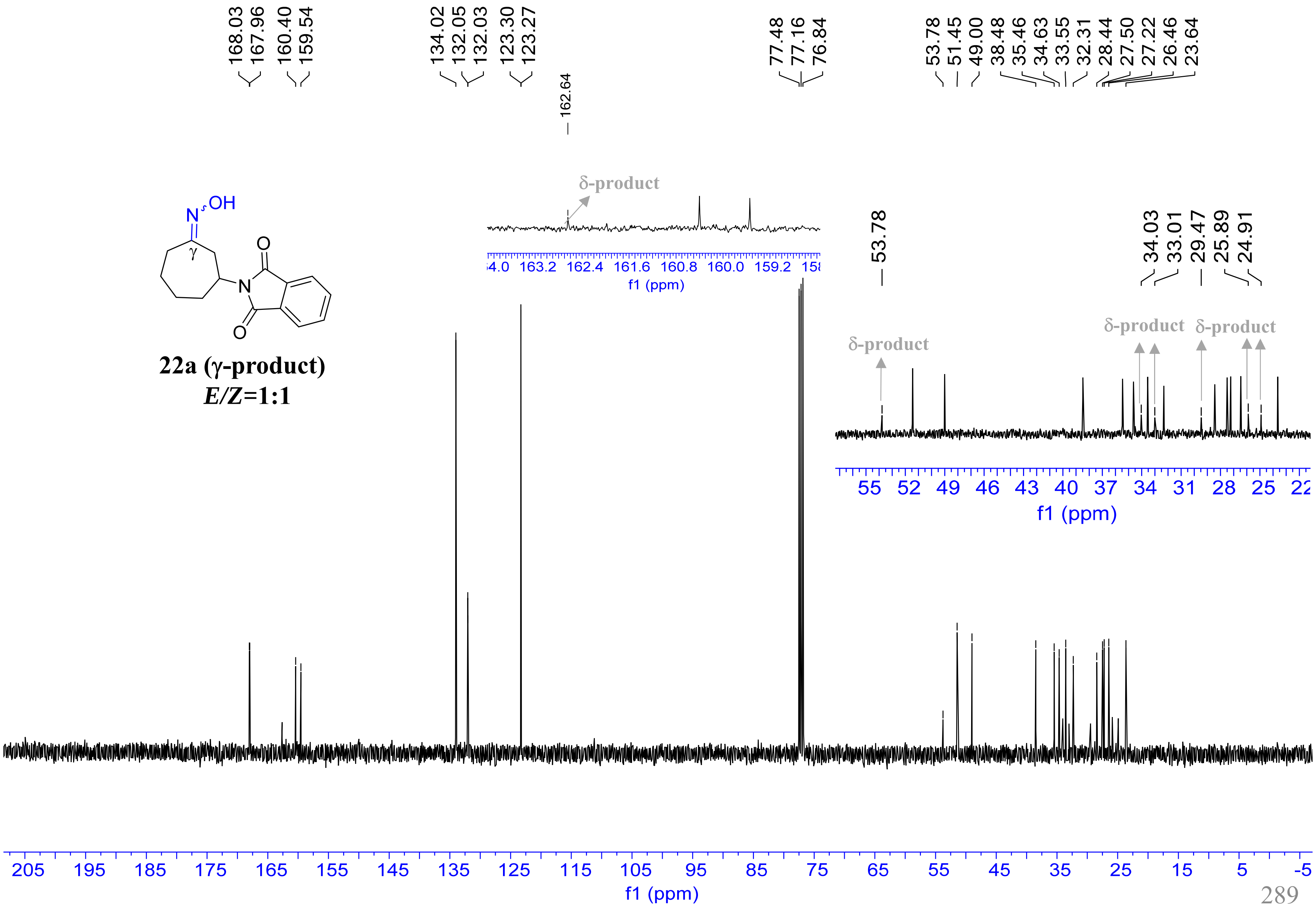

^{1}H NMR, 400 MHz, $CDCl_3$

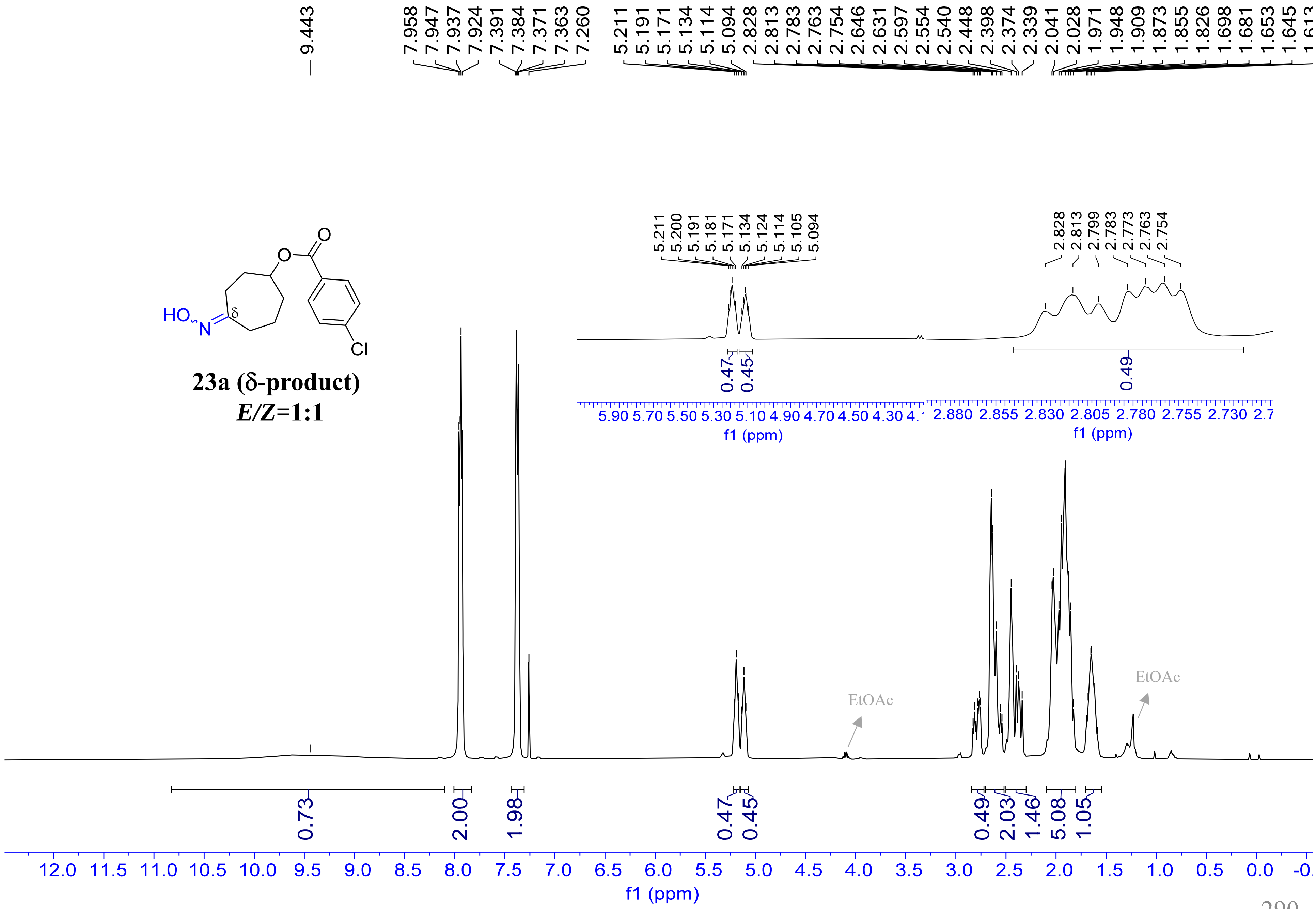

23a (δ-product)
***E/Z*=1:1**

^{13}C NMR 101 MHz, $CDCl_3$

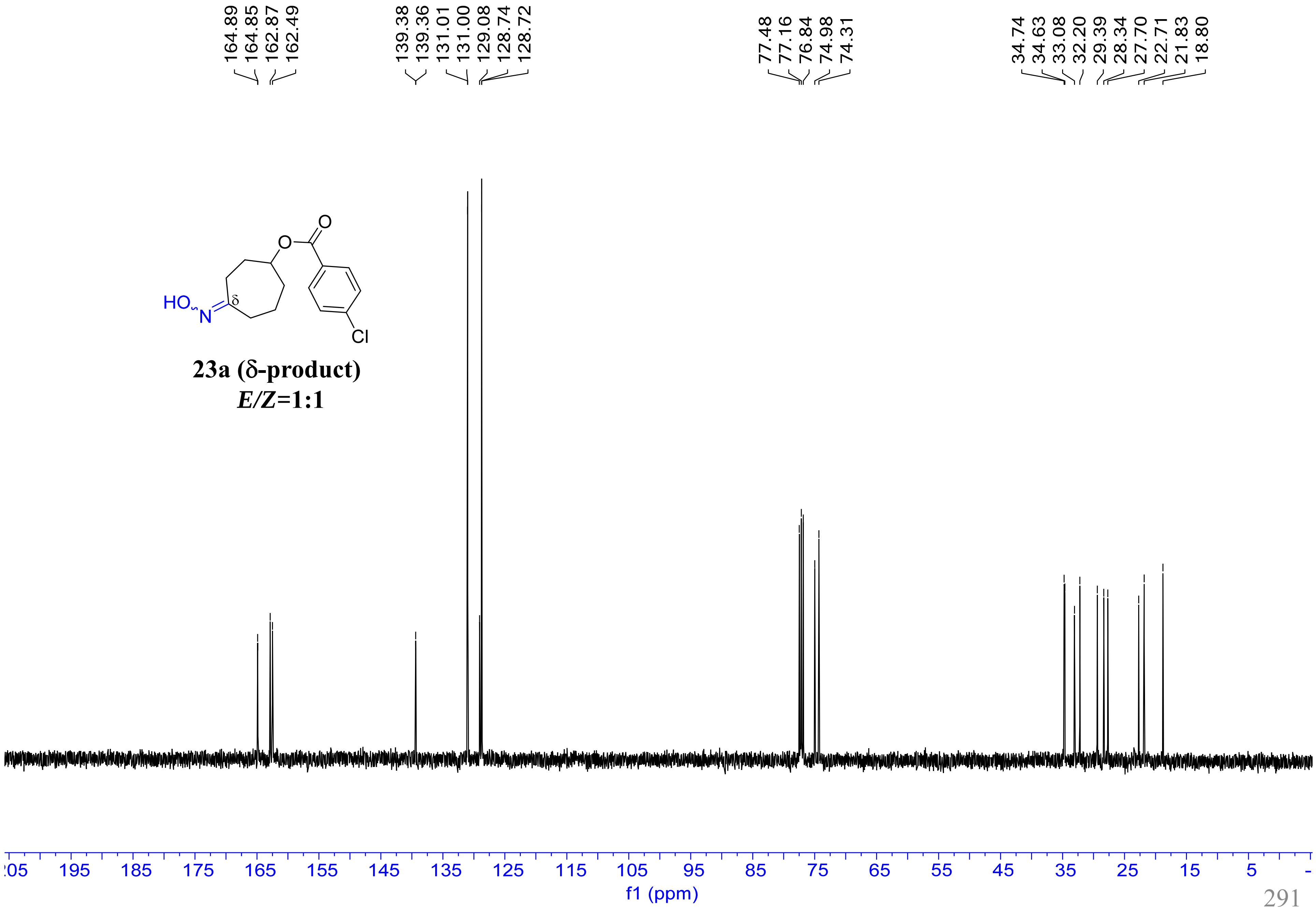

^{1}H NMR, 400 MHz, $CDCl_3$

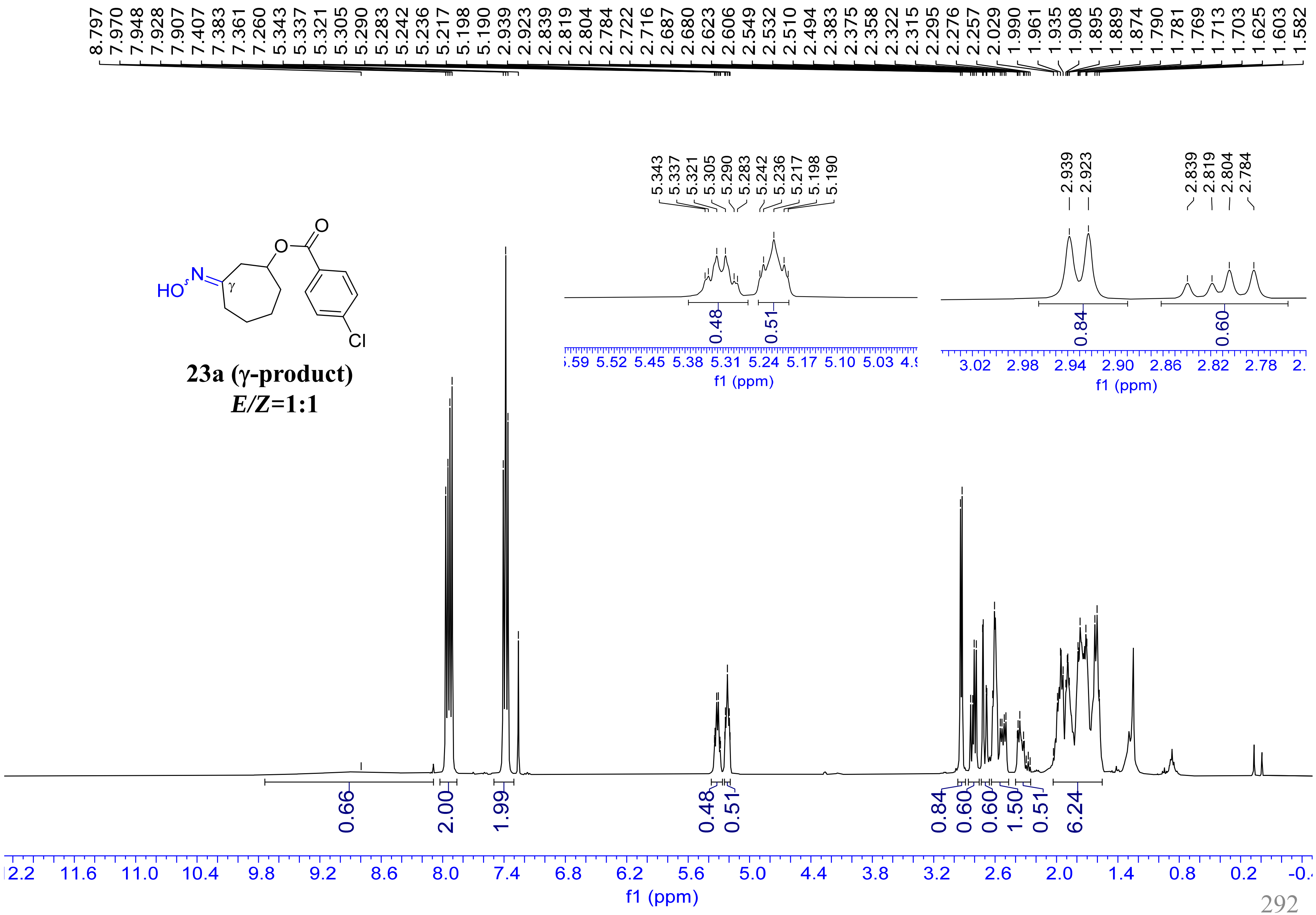

^{13}C NMR 101 MHz, $CDCl_3$

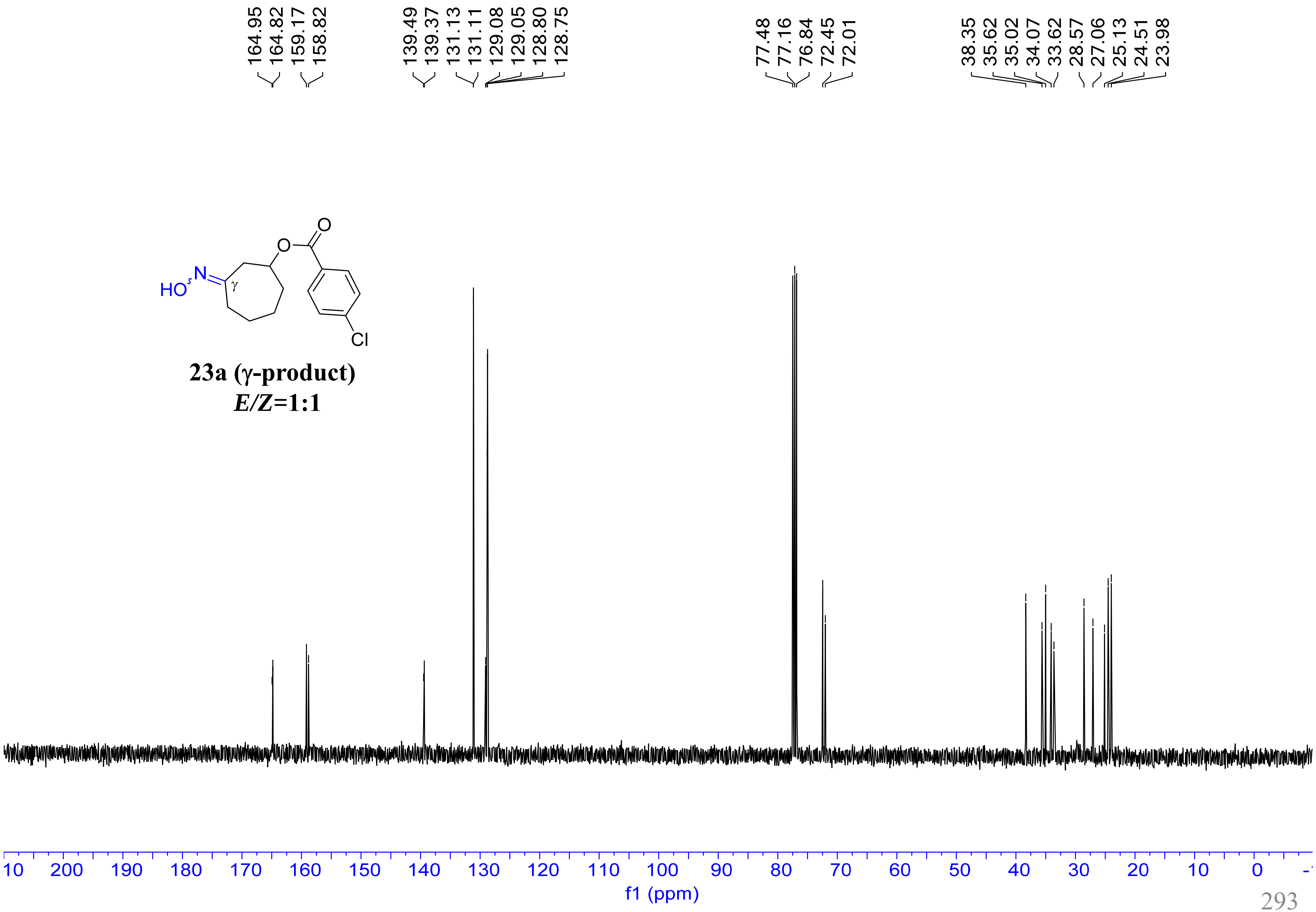

^{1}H NMR, 400 MHz, $CDCl_3$

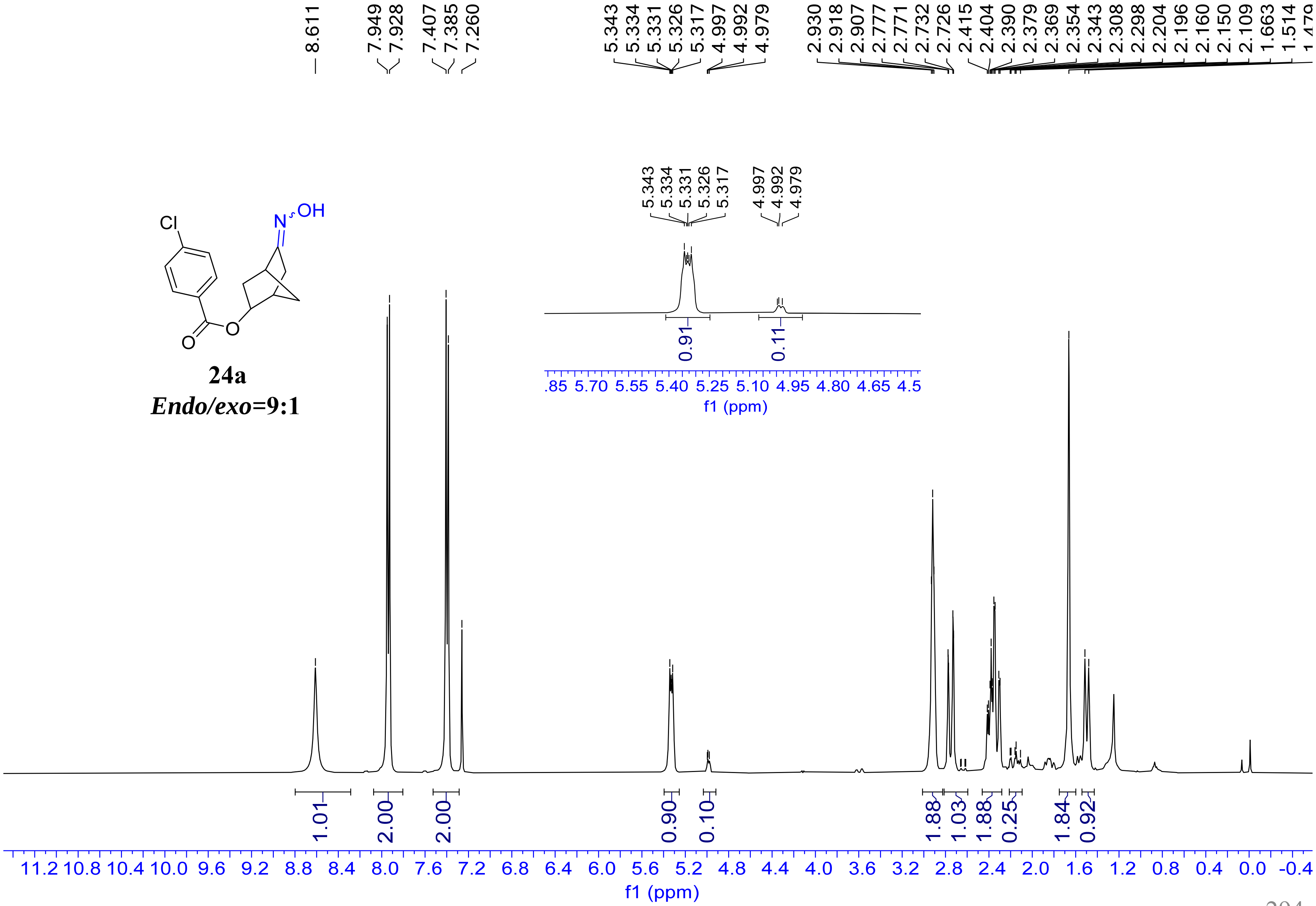

^{13}C NMR 101 MHz, $CDCl_3$

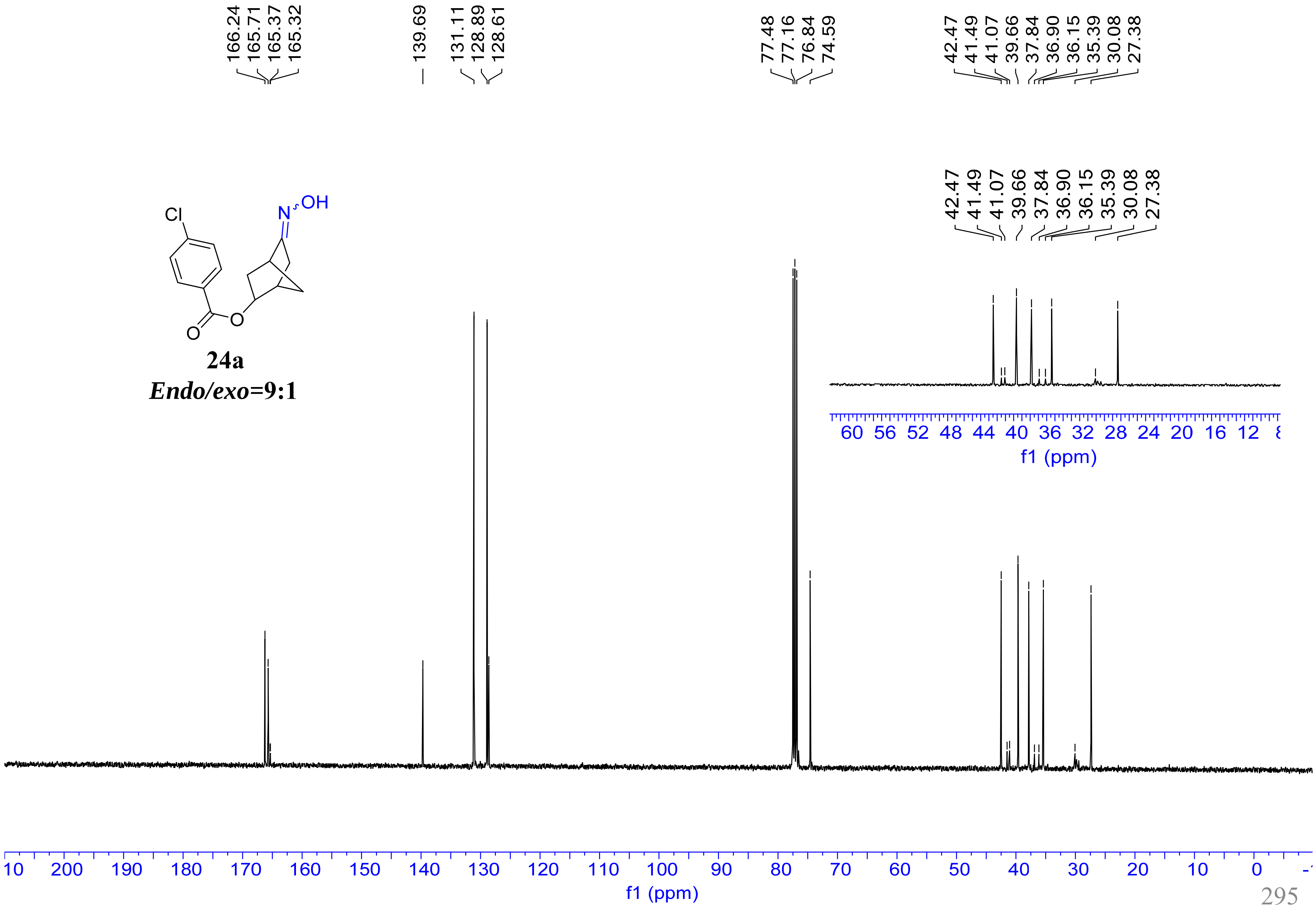

24a
Endo/exo=9:1

^1H NMR, 400 MHz, $CDCl_3$

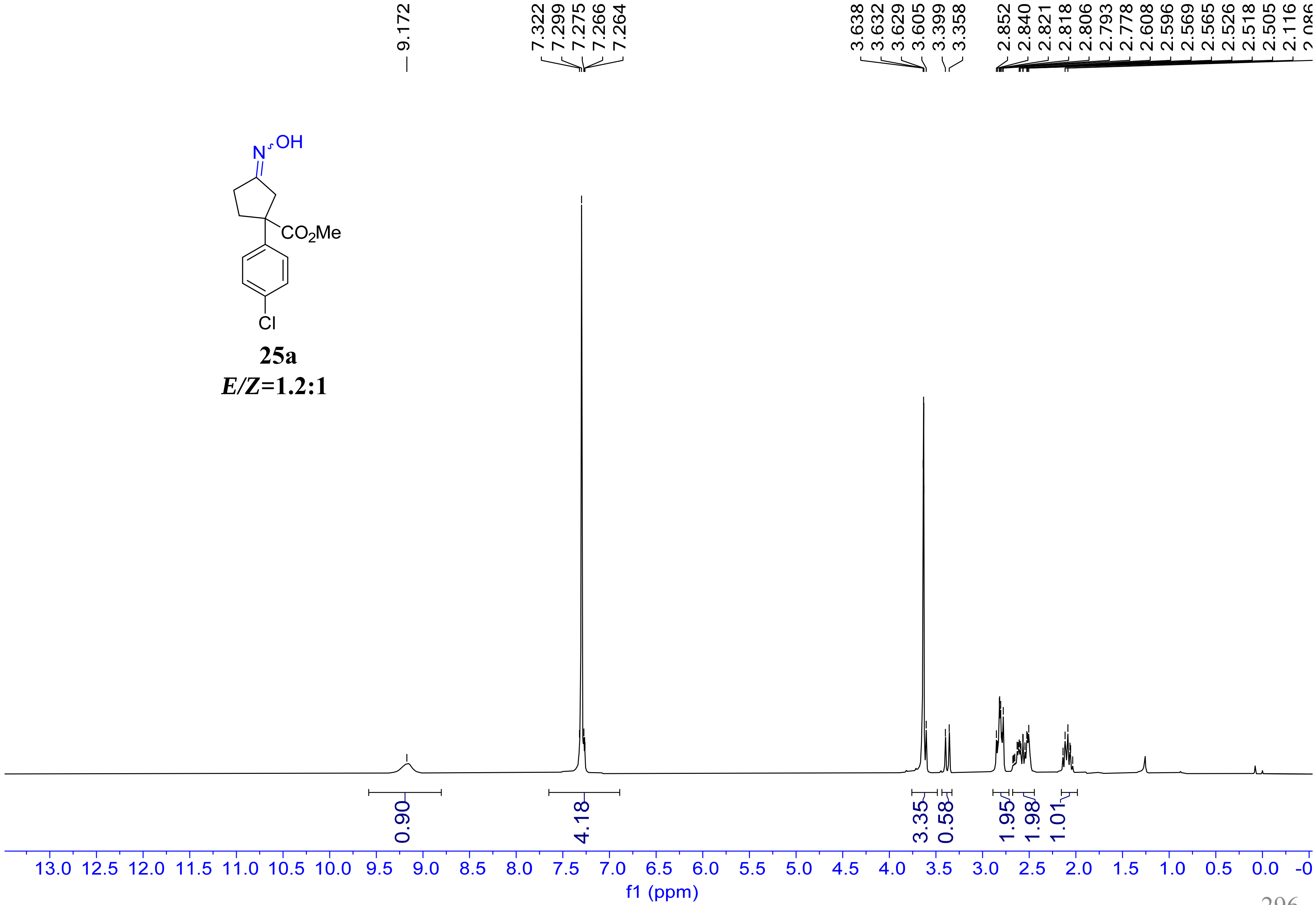

^{13}C NMR 101 MHz, $CDCl_3$

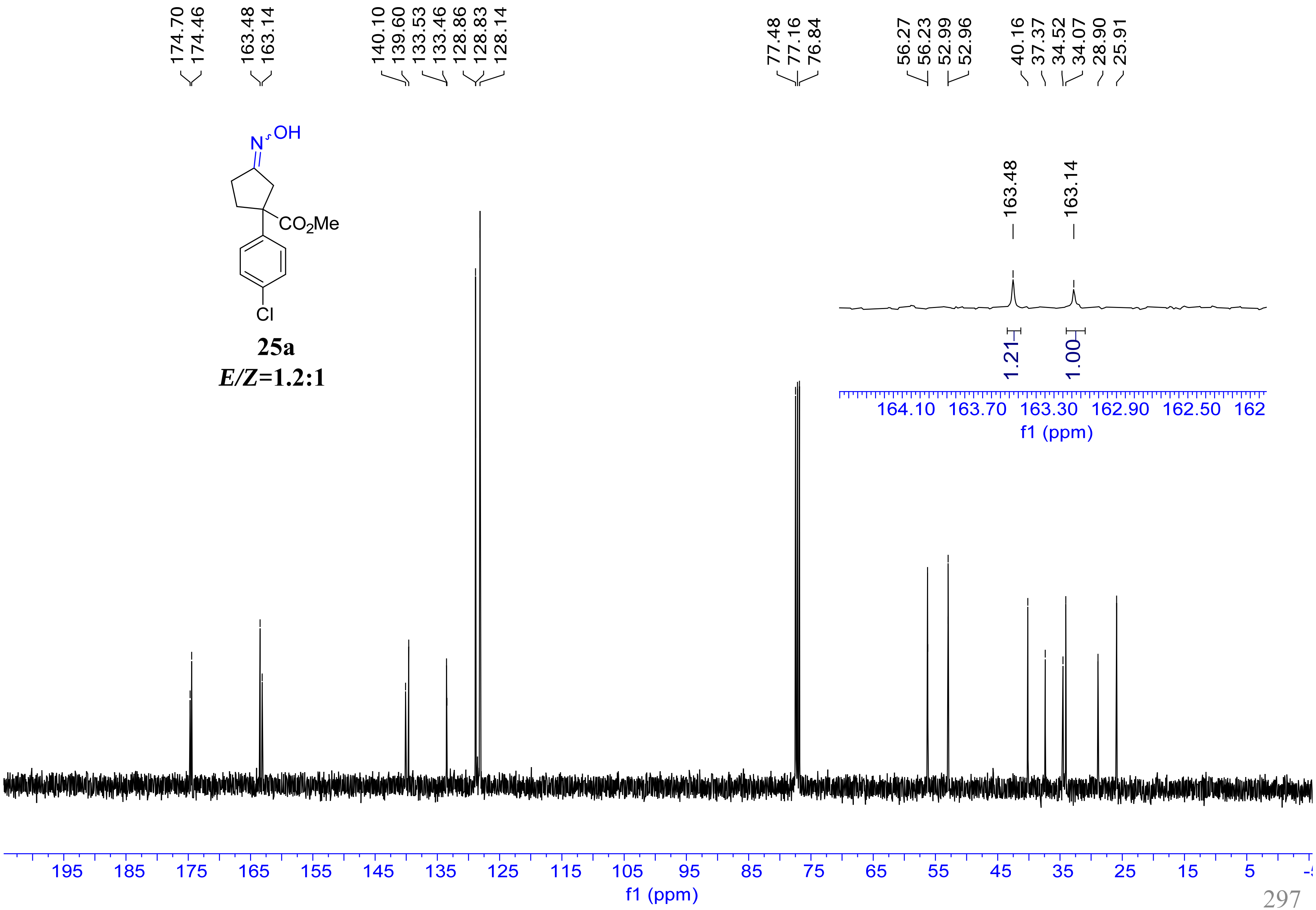

^{1}H NMR, 400 MHz, $CDCl_3$

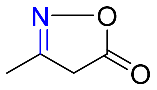

26a'

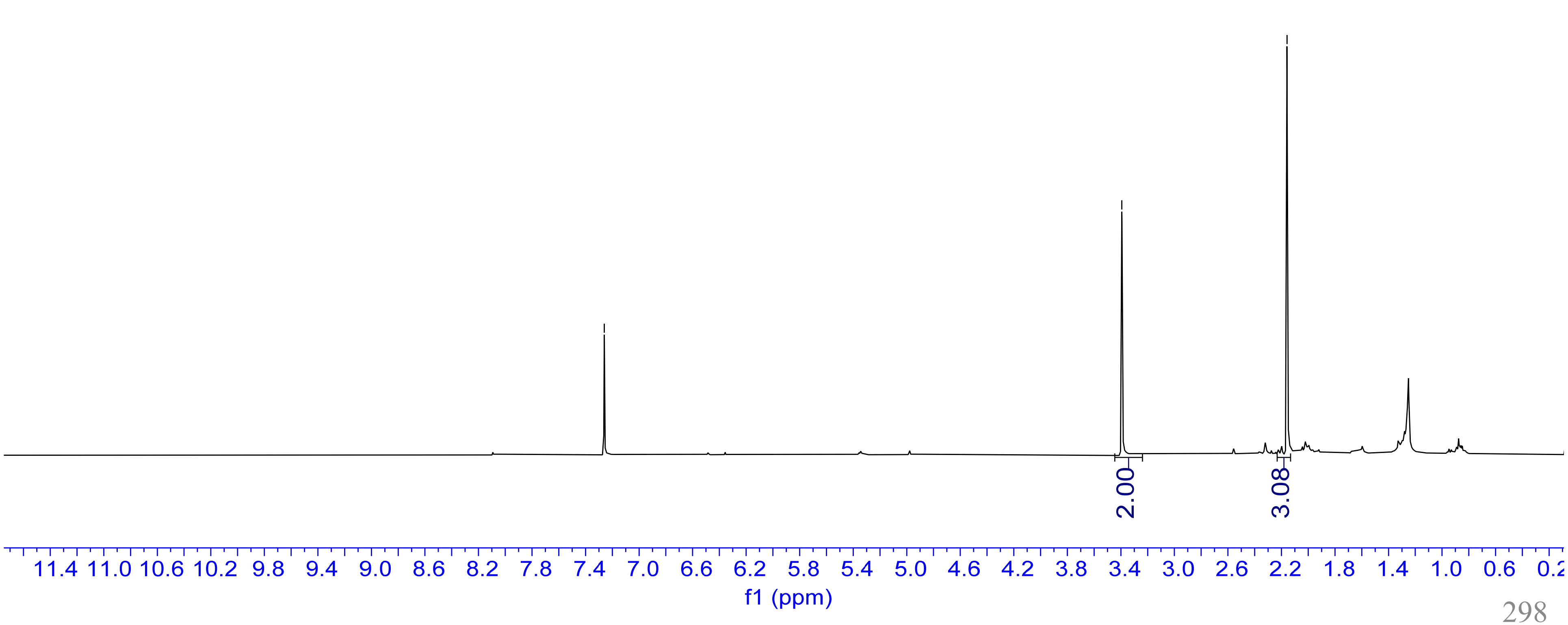

^{13}C NMR 101 MHz, $CDCl_3$

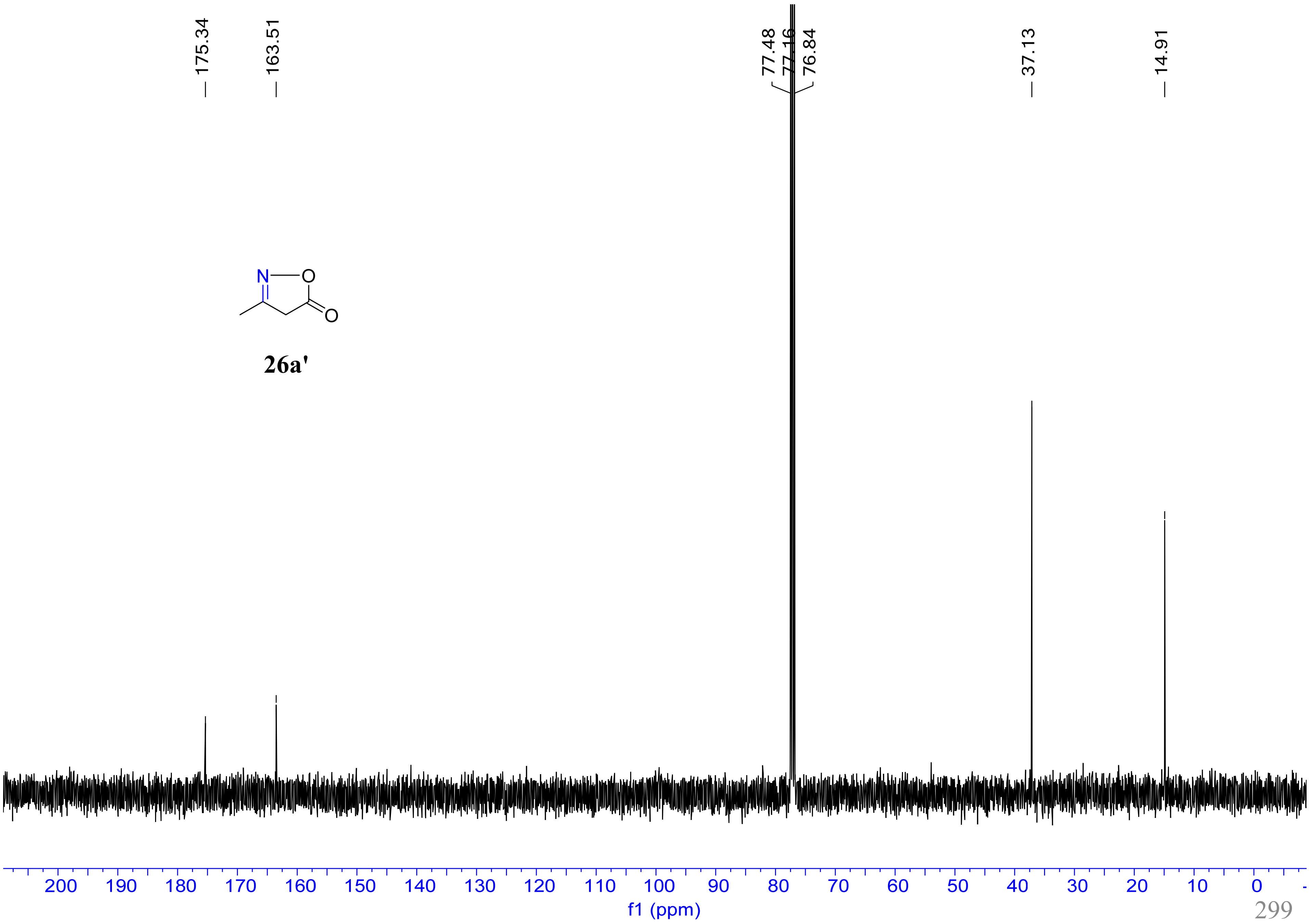

^1H NMR, 400 MHz, $CDCl_3$

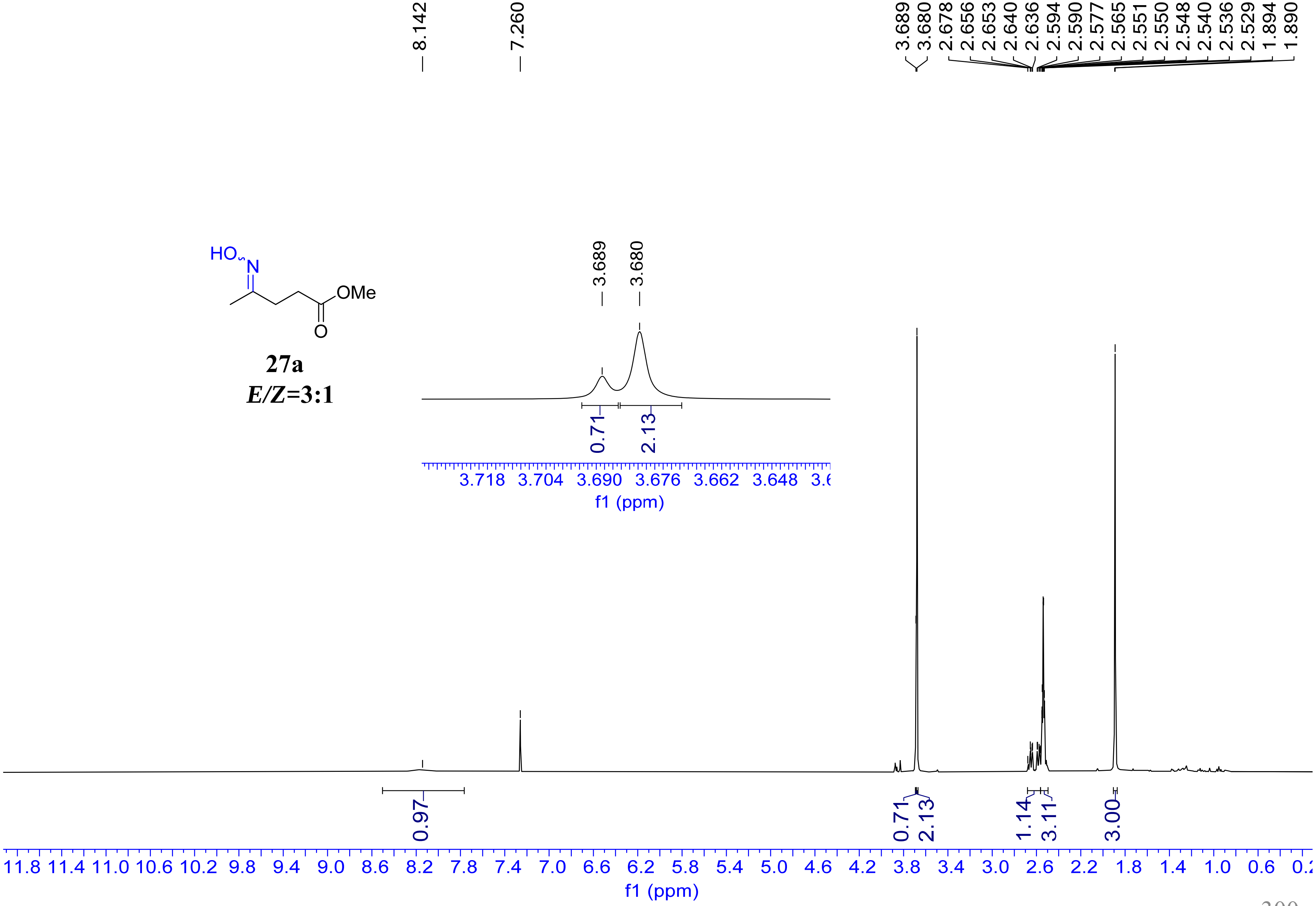

27a

***E/Z*=3:1**

^{13}C NMR 101 MHz, $CDCl_3$

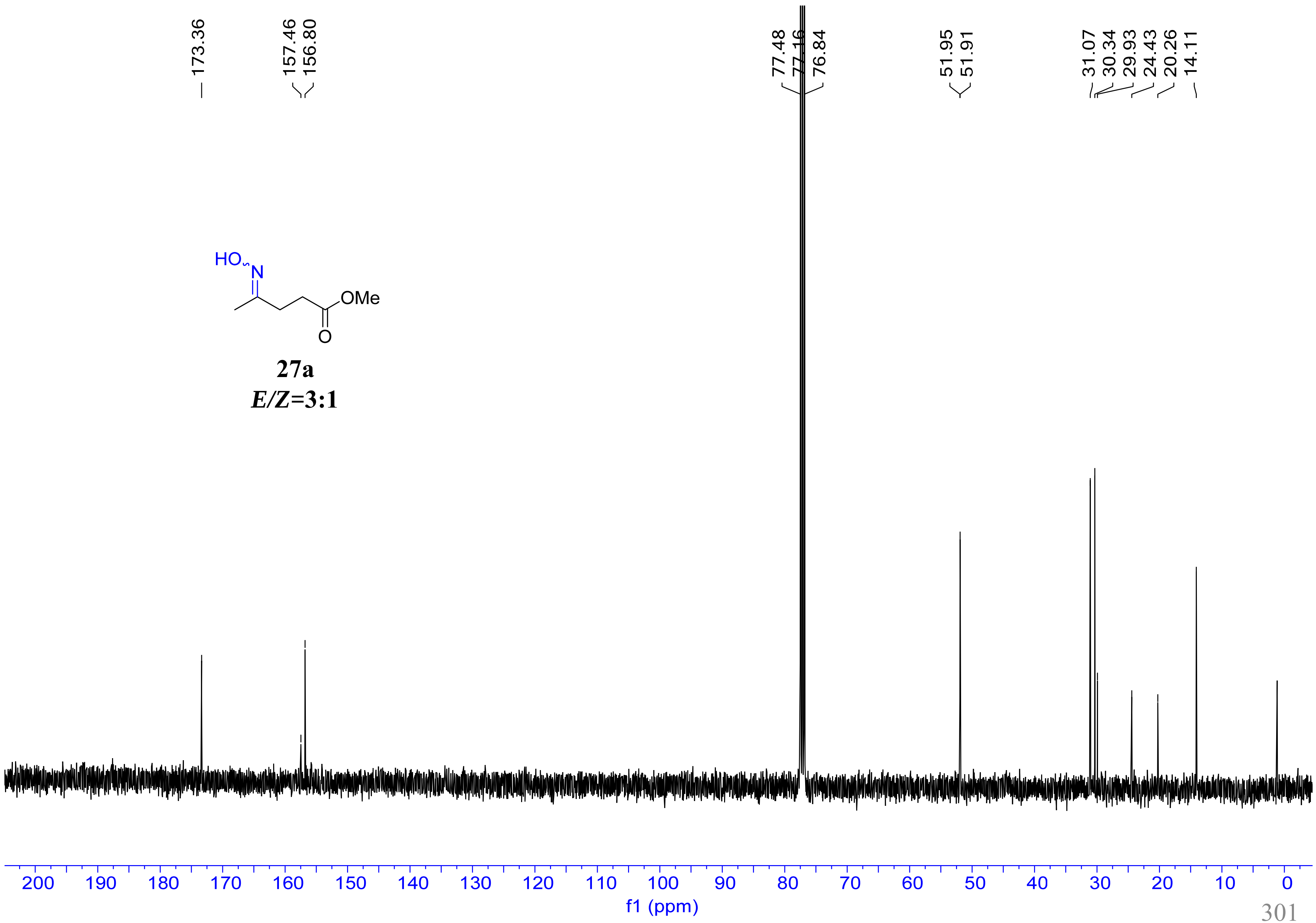

^{1}H NMR, 400 MHz, $CDCl_3$

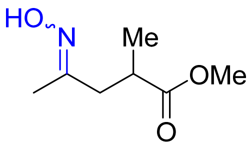

28a

***E/Z*=3:1**

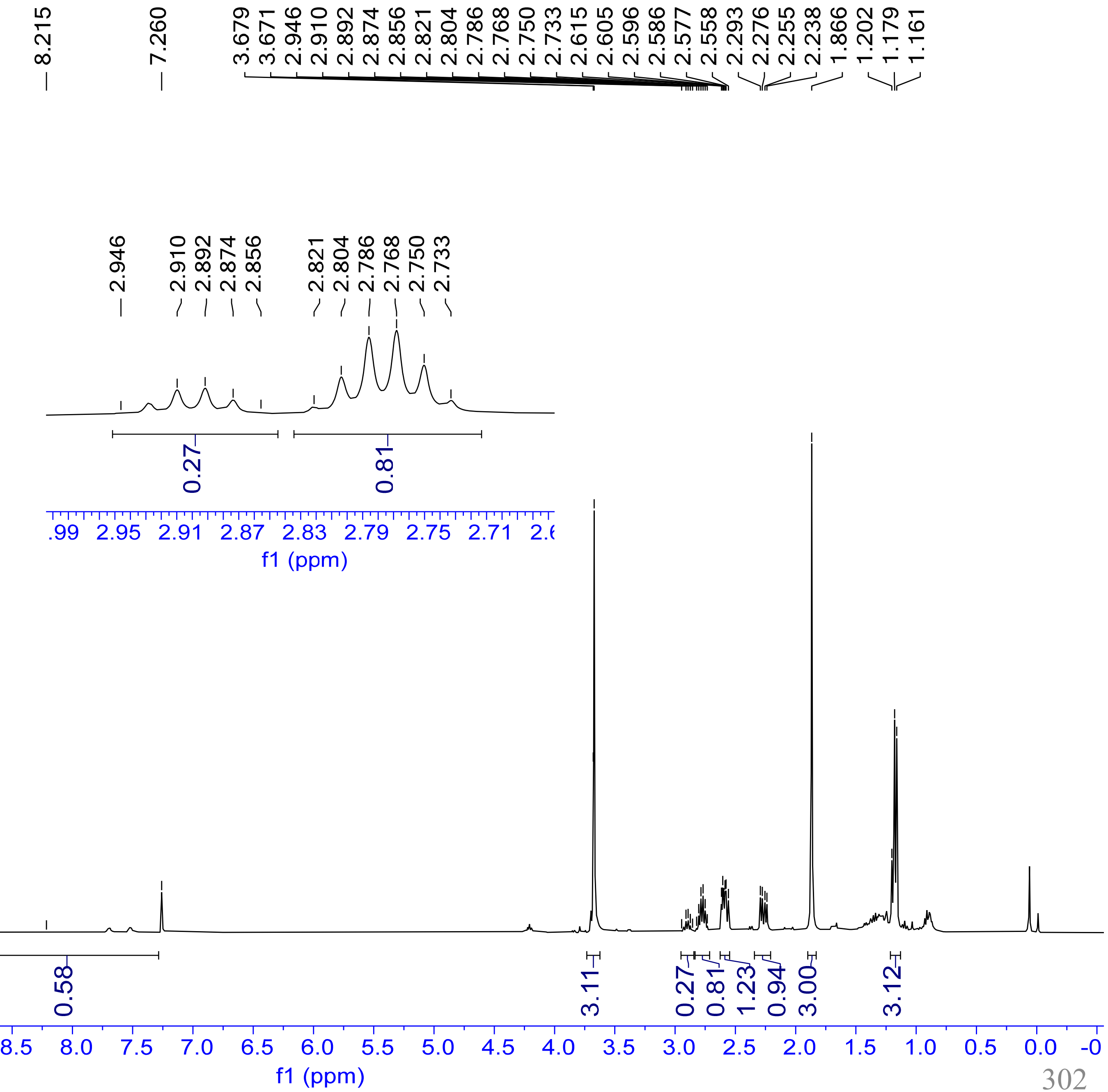

^{13}C NMR 101 MHz, CDCl$_3$

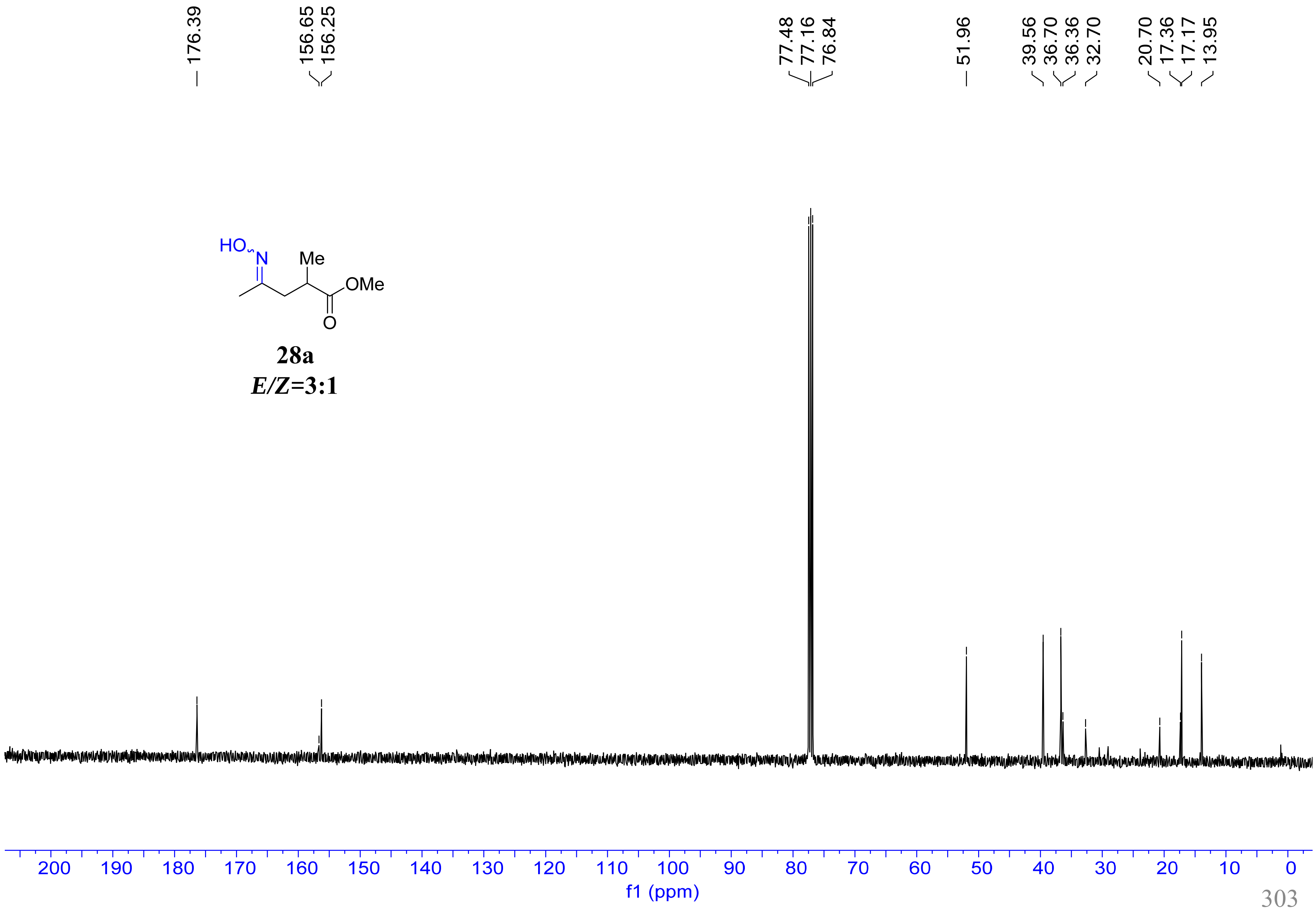

^1H NMR, 400 MHz, $CDCl_3$

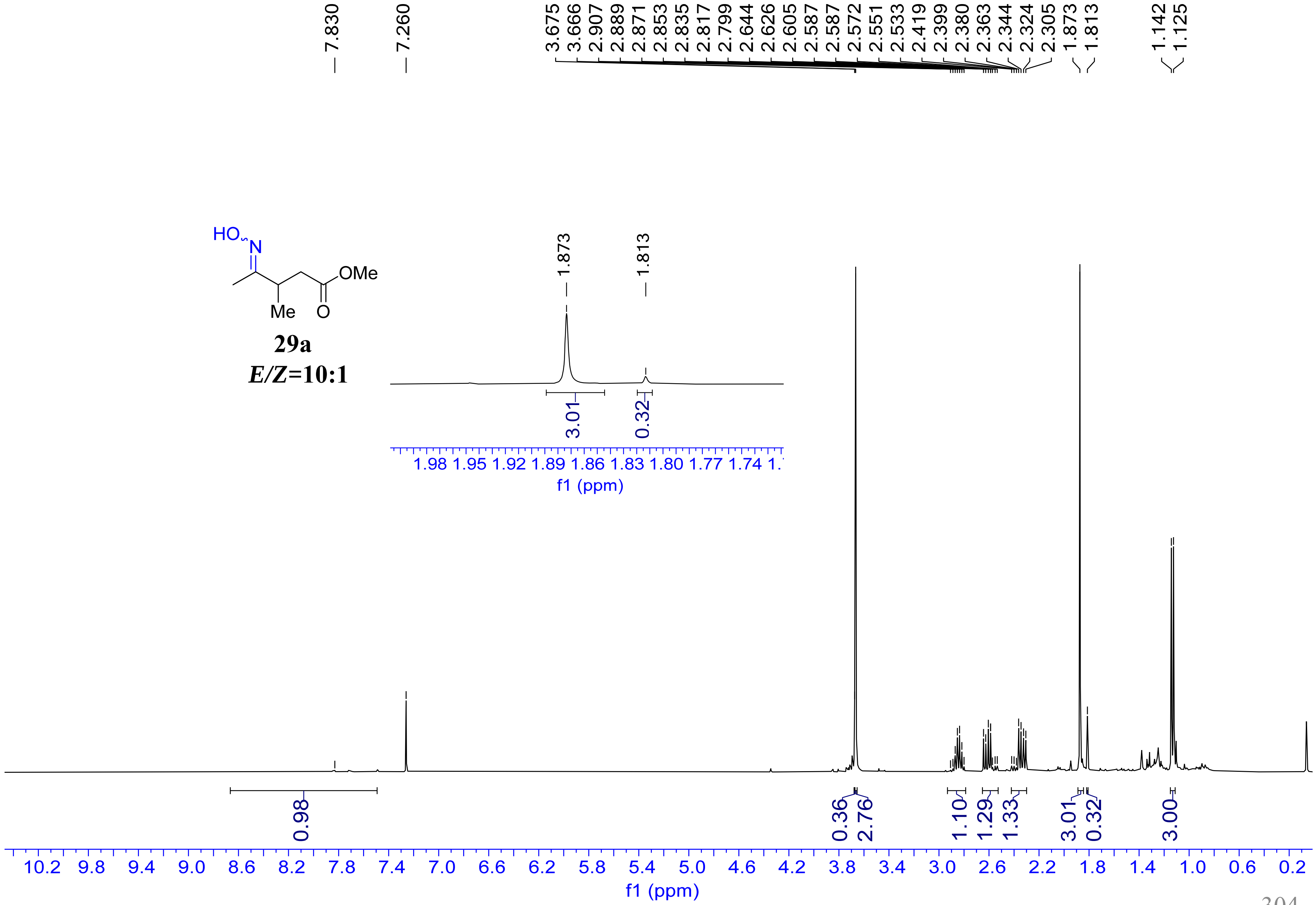

29a

***E*/*Z*=10:1**

^{13}C NMR 101 MHz, $CDCl_3$

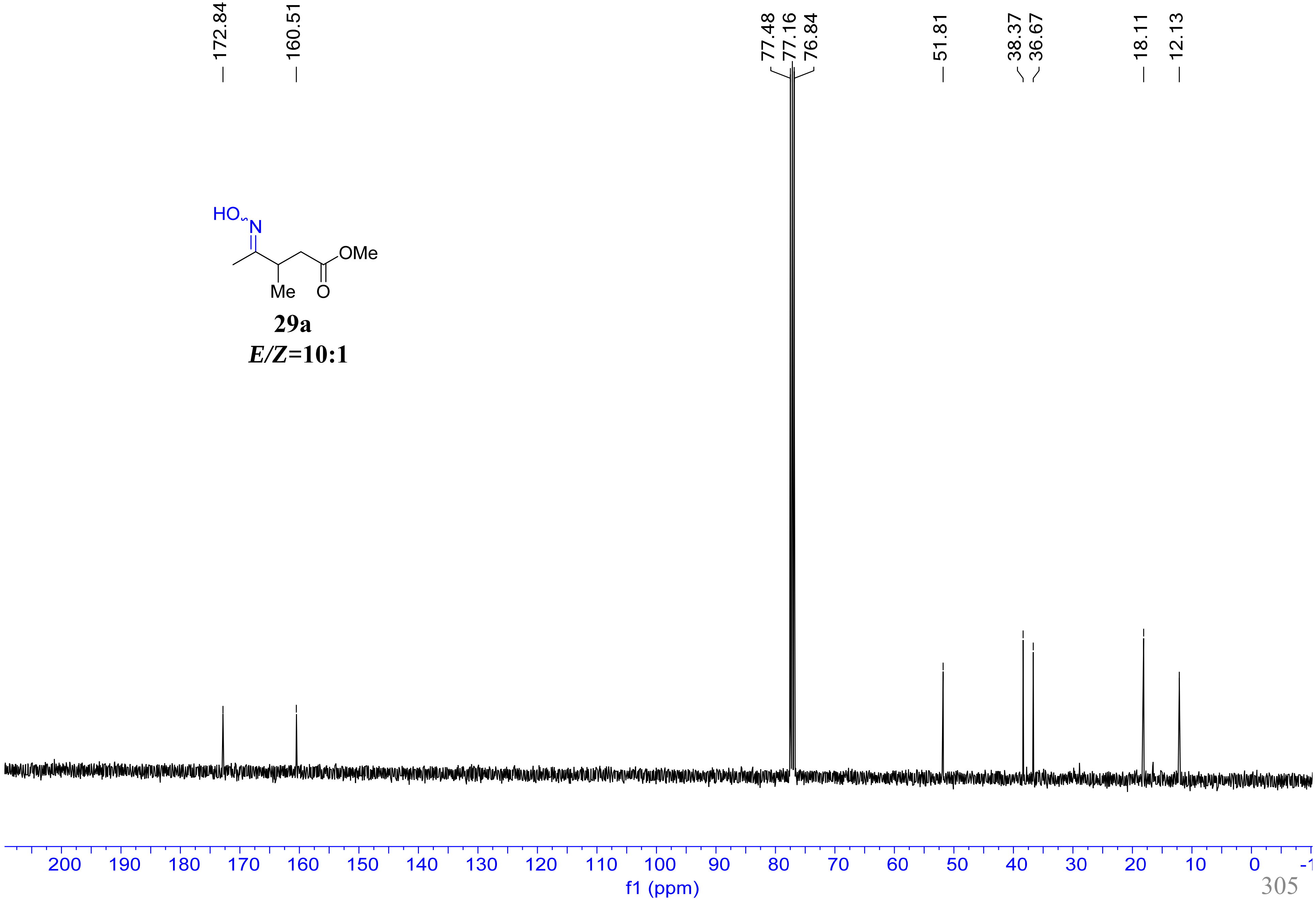

29a

***E/Z*=10:1**

^1H NMR, 400 MHz, $CDCl_3$

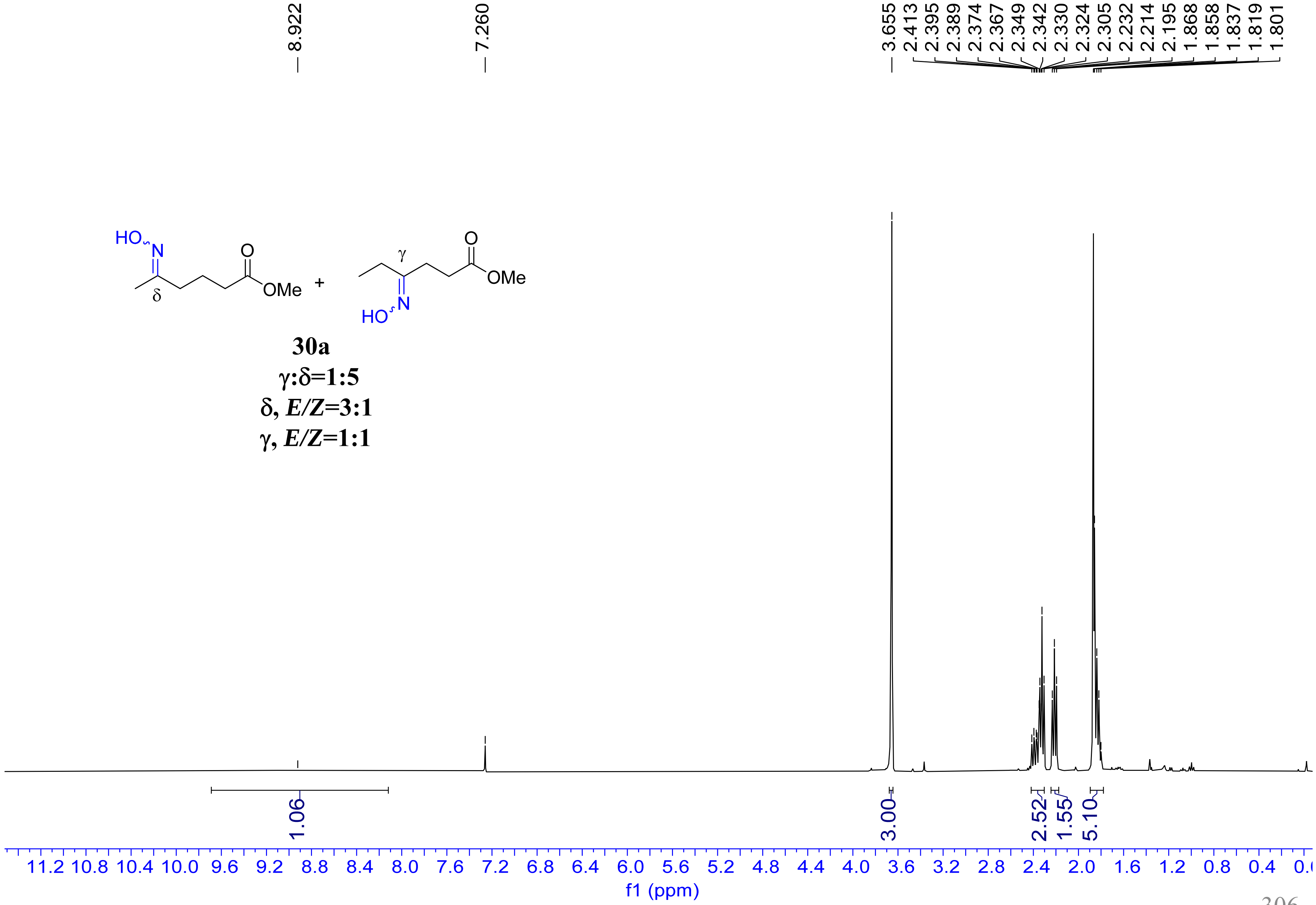

^{13}C NMR 101 MHz, $CDCl_3$

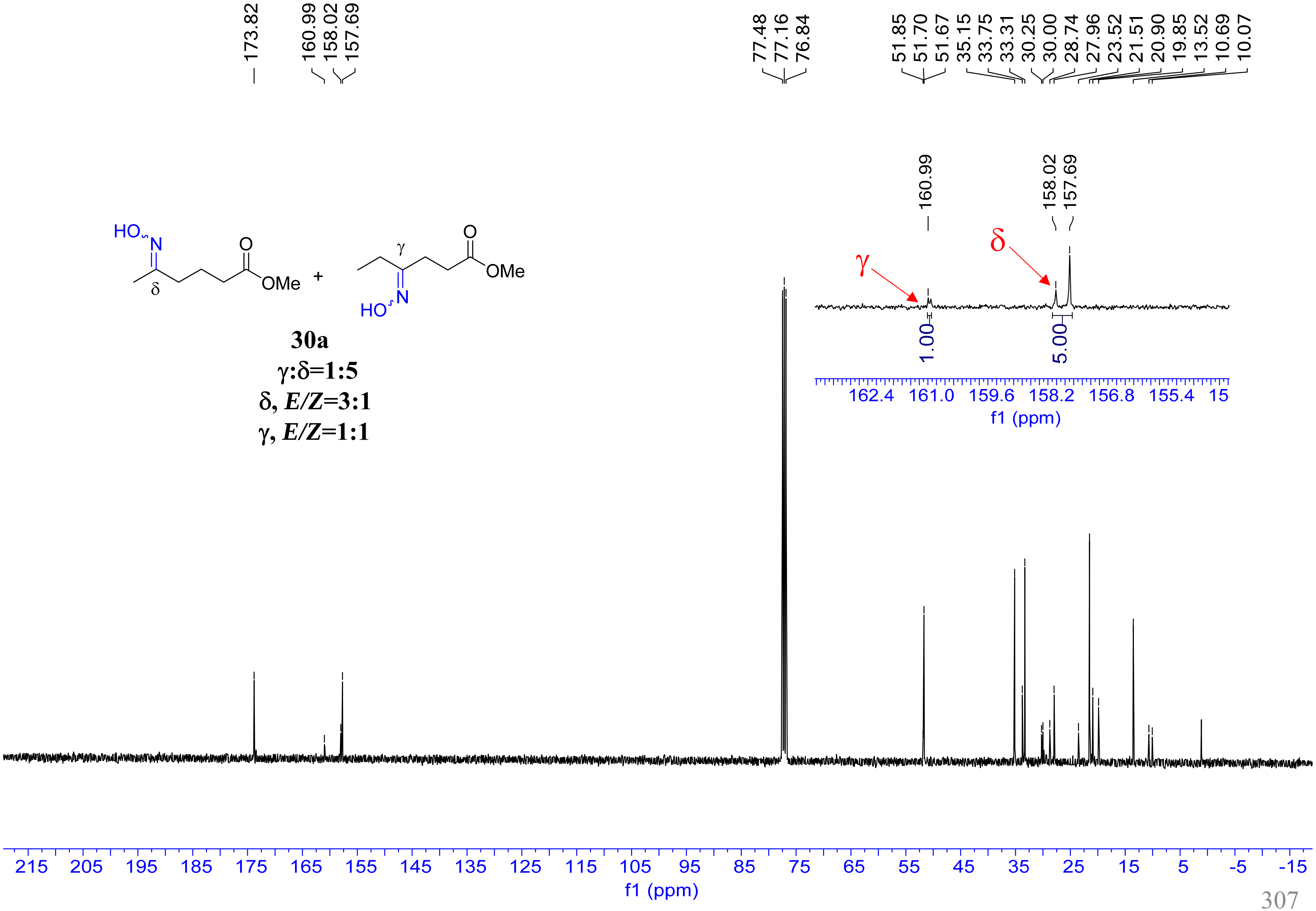

30a

γ:δ=1:5

δ, *E/Z*=3:1

γ, *E/Z*=1:1

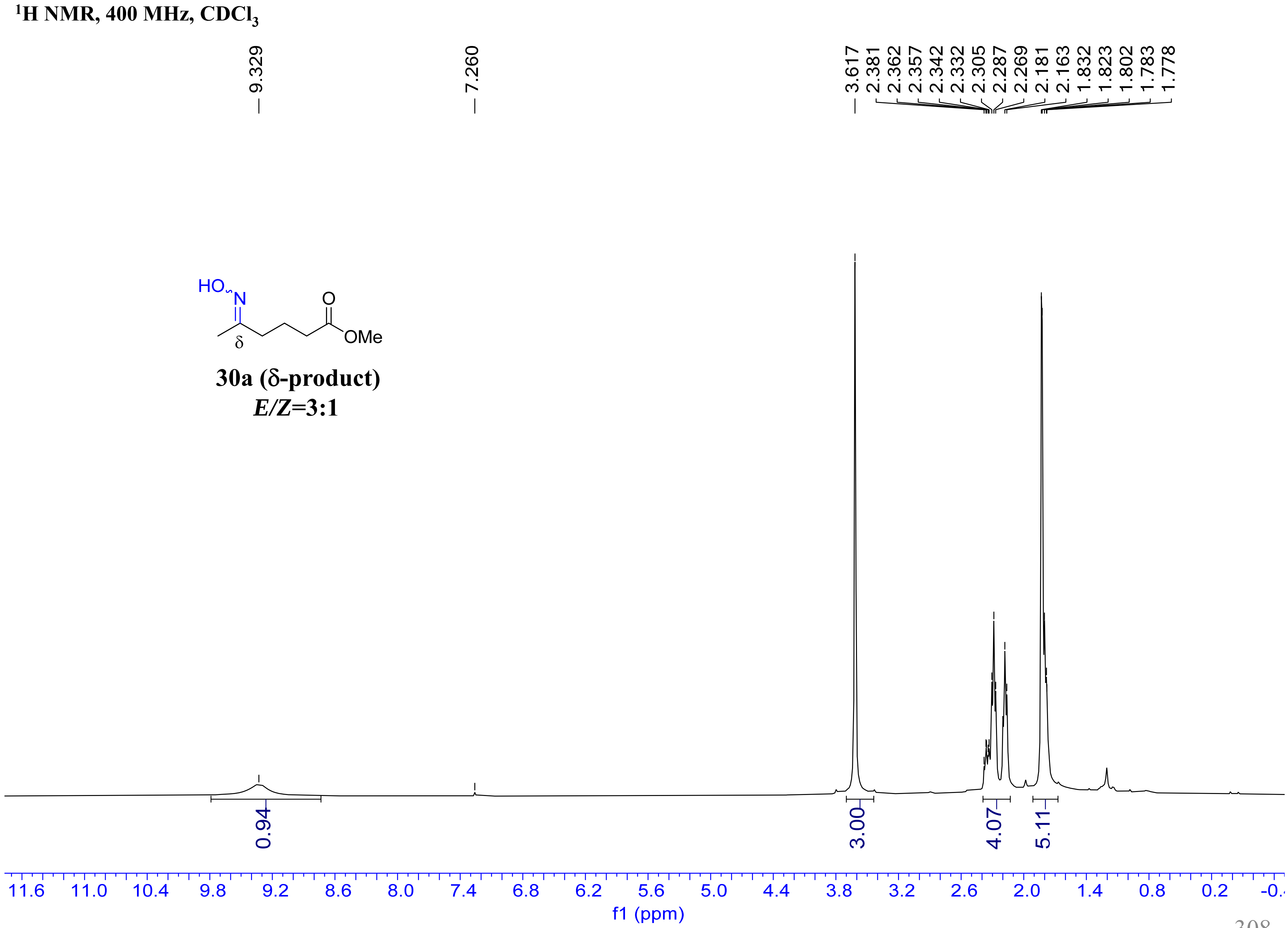

1H NMR, 400 MHz, CDCl3
9.329
7.260
3.617
2.381
2.362
2.357
2.342
2.332
2.305
2.287
2.269
2.181
2.163
1.832
1.823
1.802
1.783
1.778
HO
N
O
OMe
δ
30a (δ-product)
E/Z=3:1
0.94
3.00
4.07
5.11
11.6
11.0
10.4
9.8
9.2
8.6
8.0
7.4
6.8
6.2
5.6
5.0
4.4
3.8
3.2
2.6
2.0
1.4
0.8
0.2
f1 (ppm)

^{13}C NMR 101 MHz, $CDCl_3$

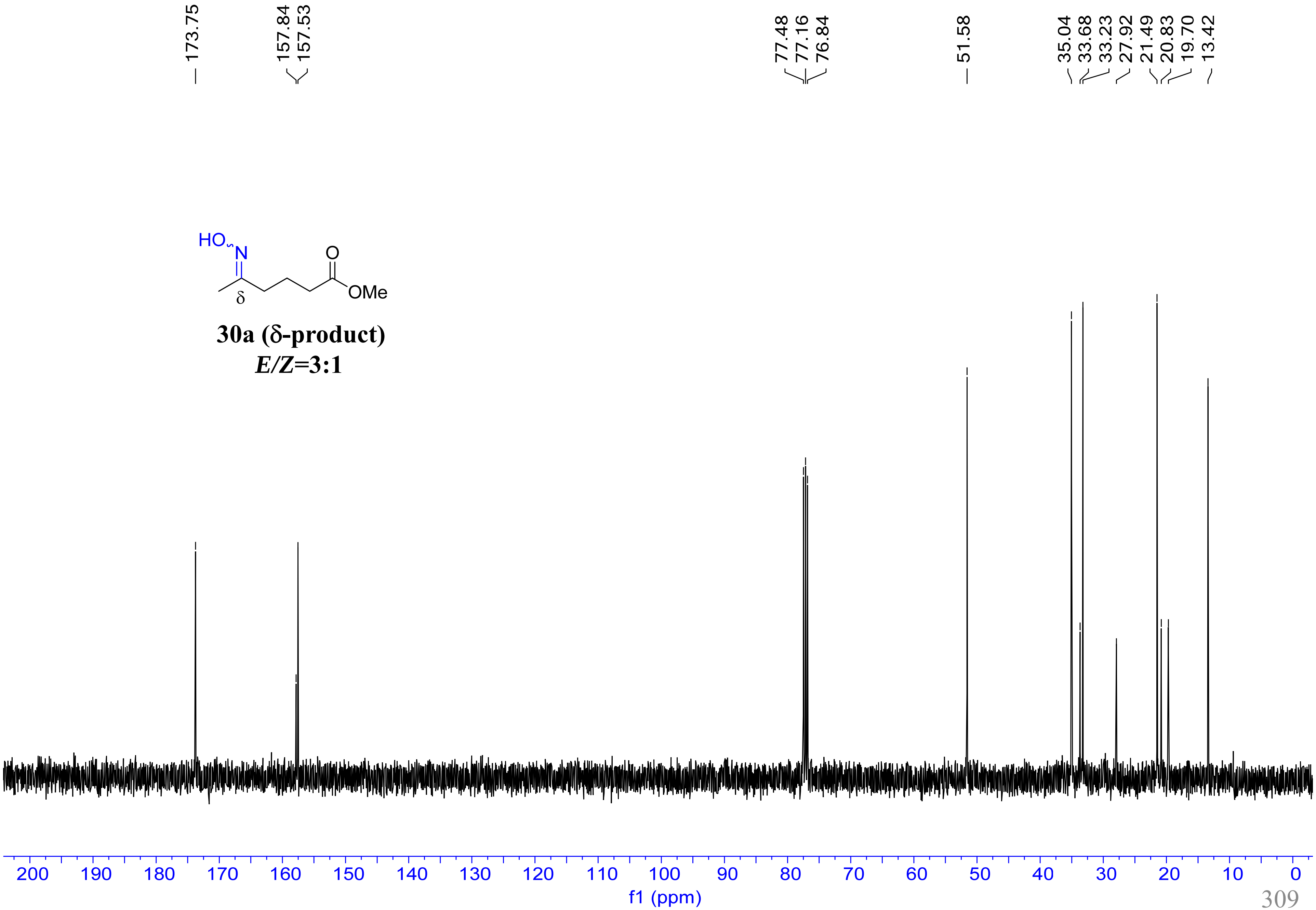

30a (δ-product)
***E*/*Z*=3:1**

^{1}H NMR, 400 MHz, $CDCl_3$

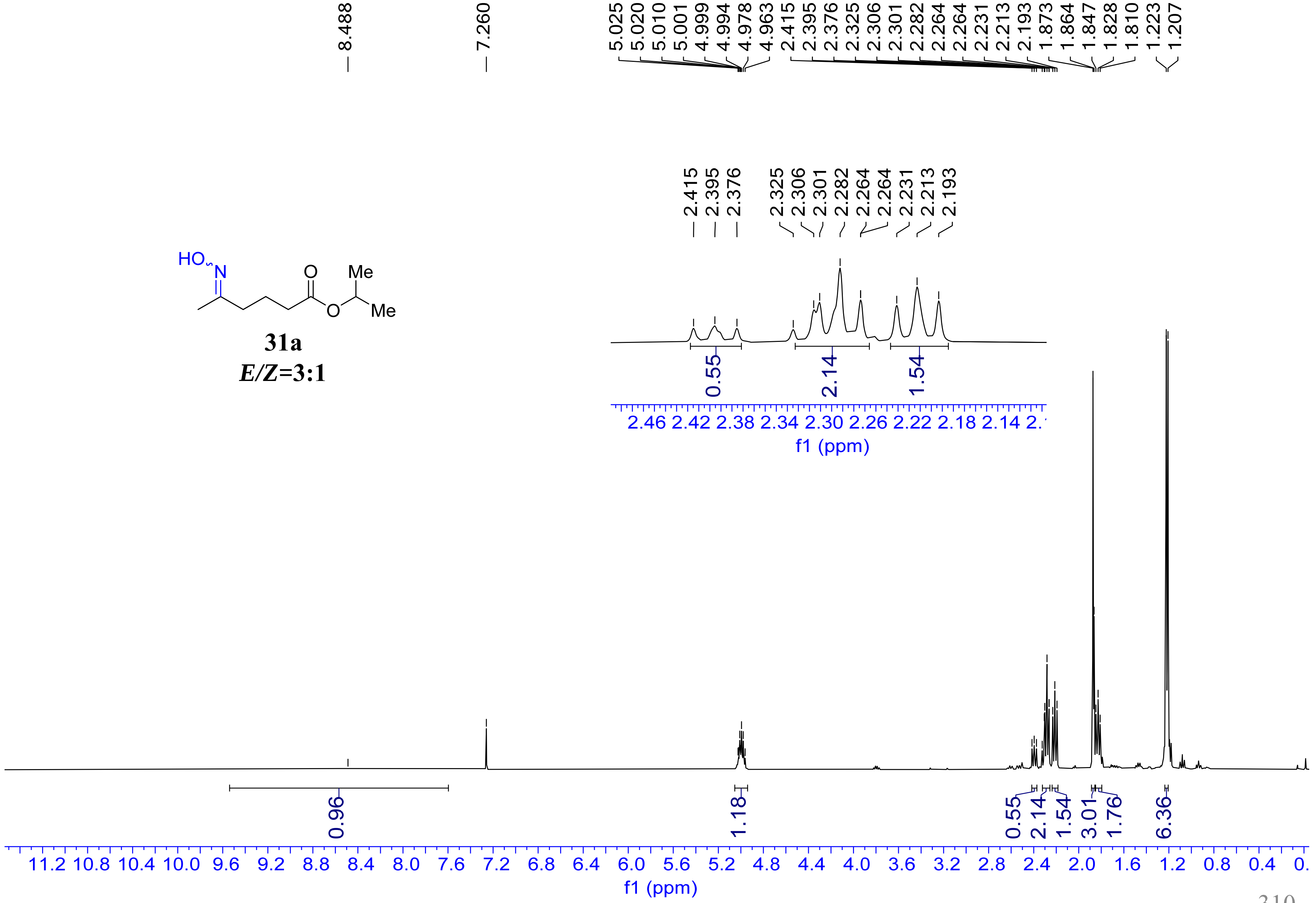

31a

***E/Z*=3:1**

^{13}C NMR 101 MHz, $CDCl_3$

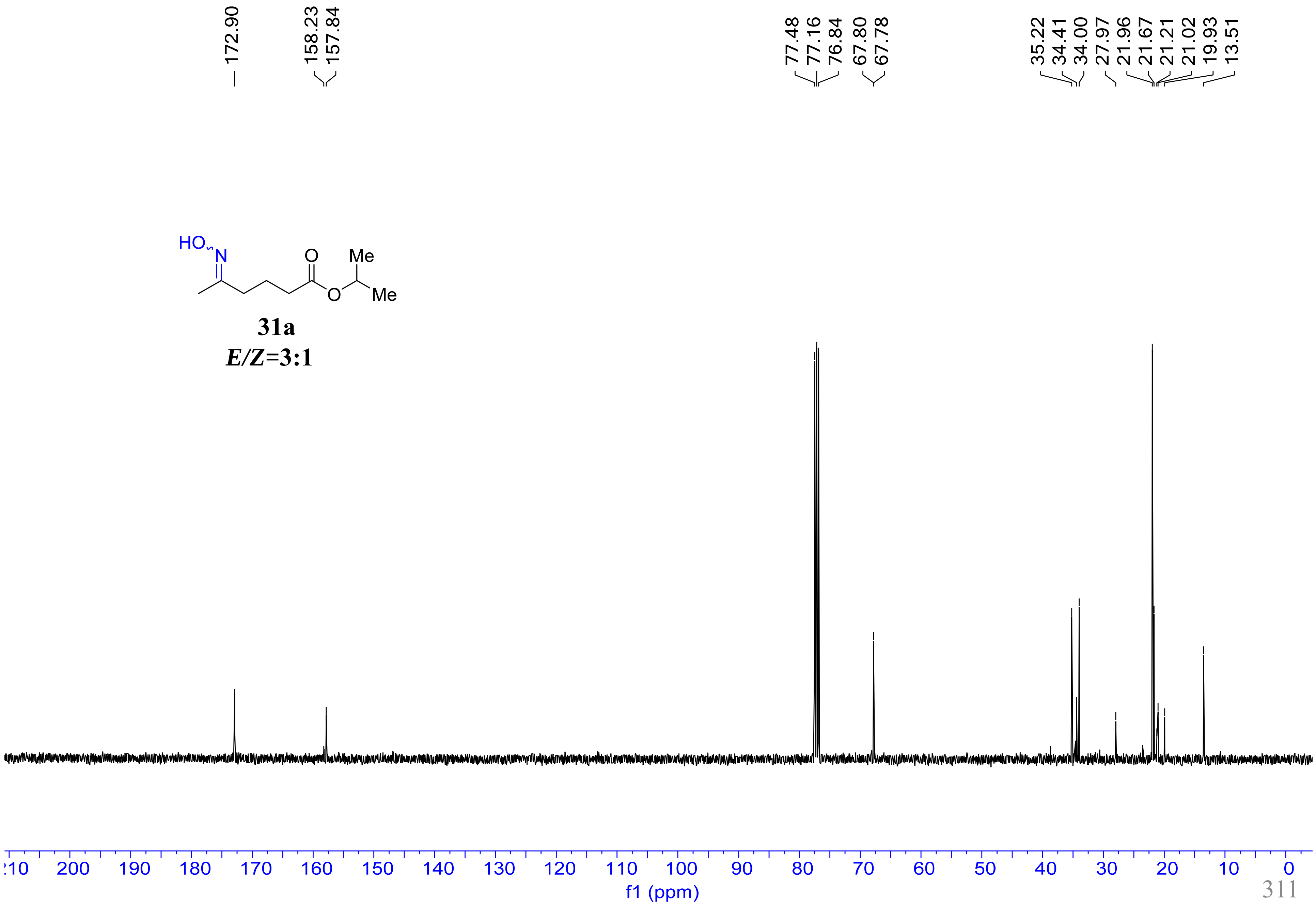

31a

***E*/*Z*=3:1**

^{1}H NMR, 400 MHz, $CDCl_3$

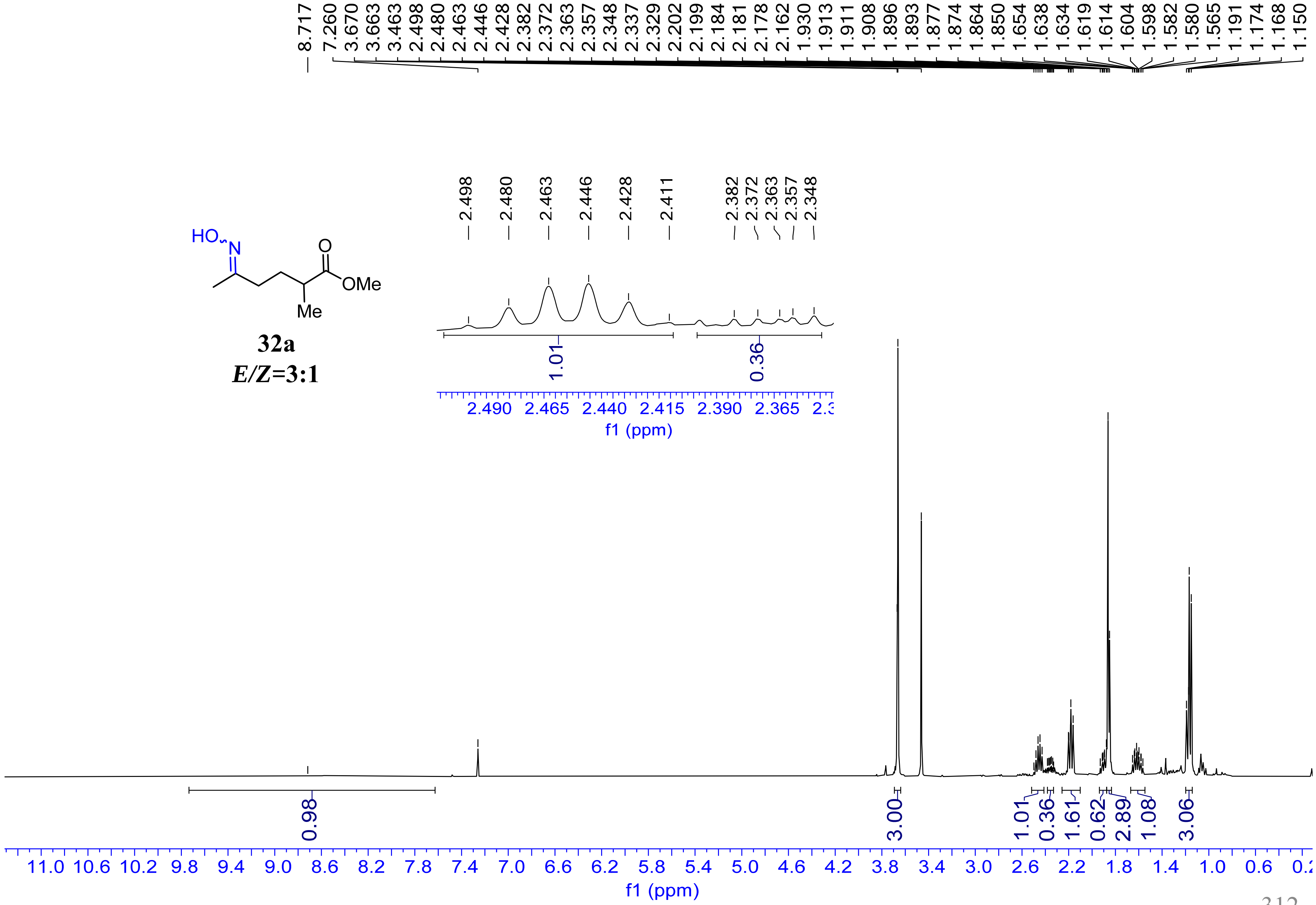

32a

***E/Z*=3:1**

^{13}C NMR 101 MHz, $CDCl_3$

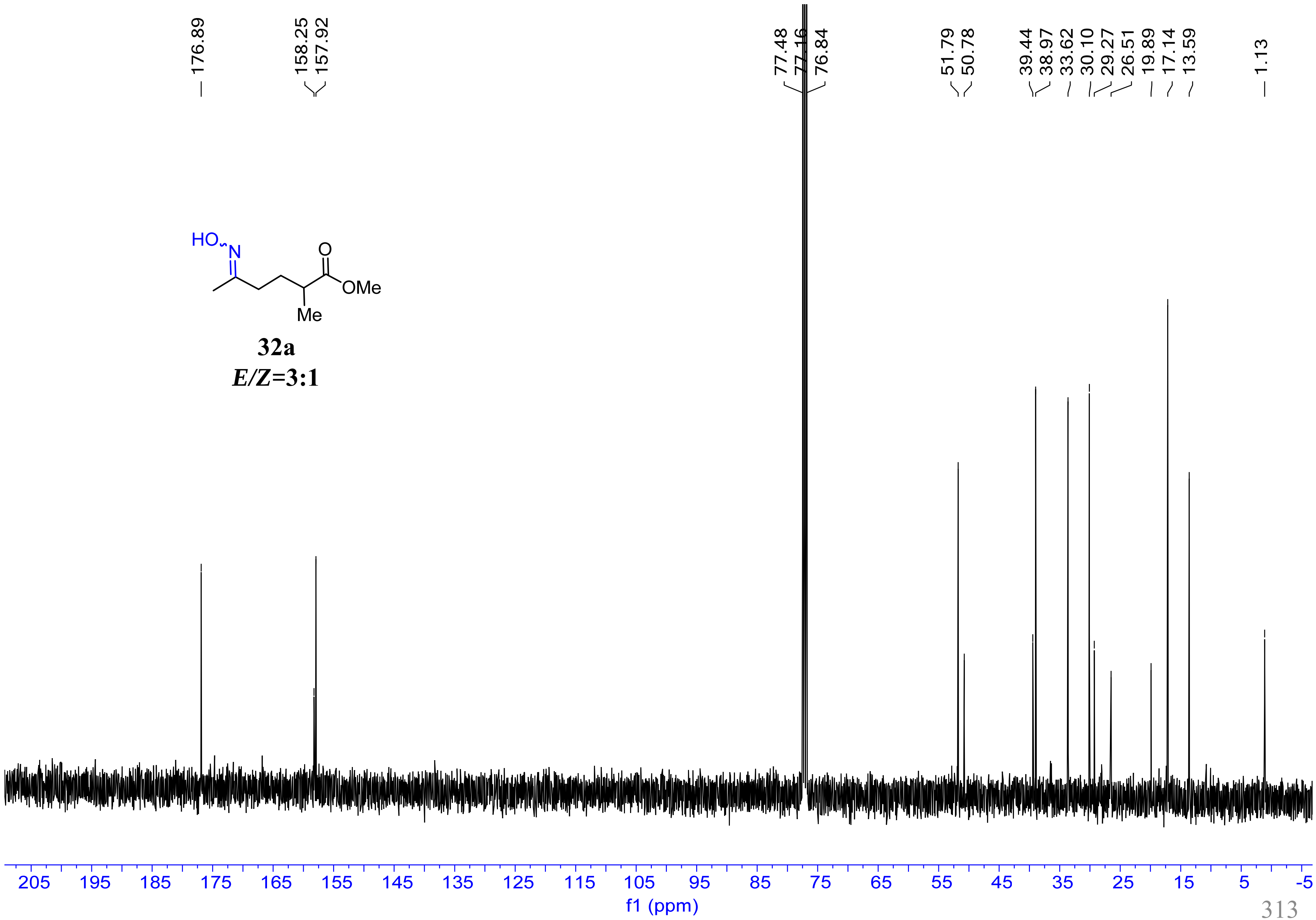

32a
***E/Z*=3:1**

^{1}H NMR, 400 MHz, $CDCl_3$

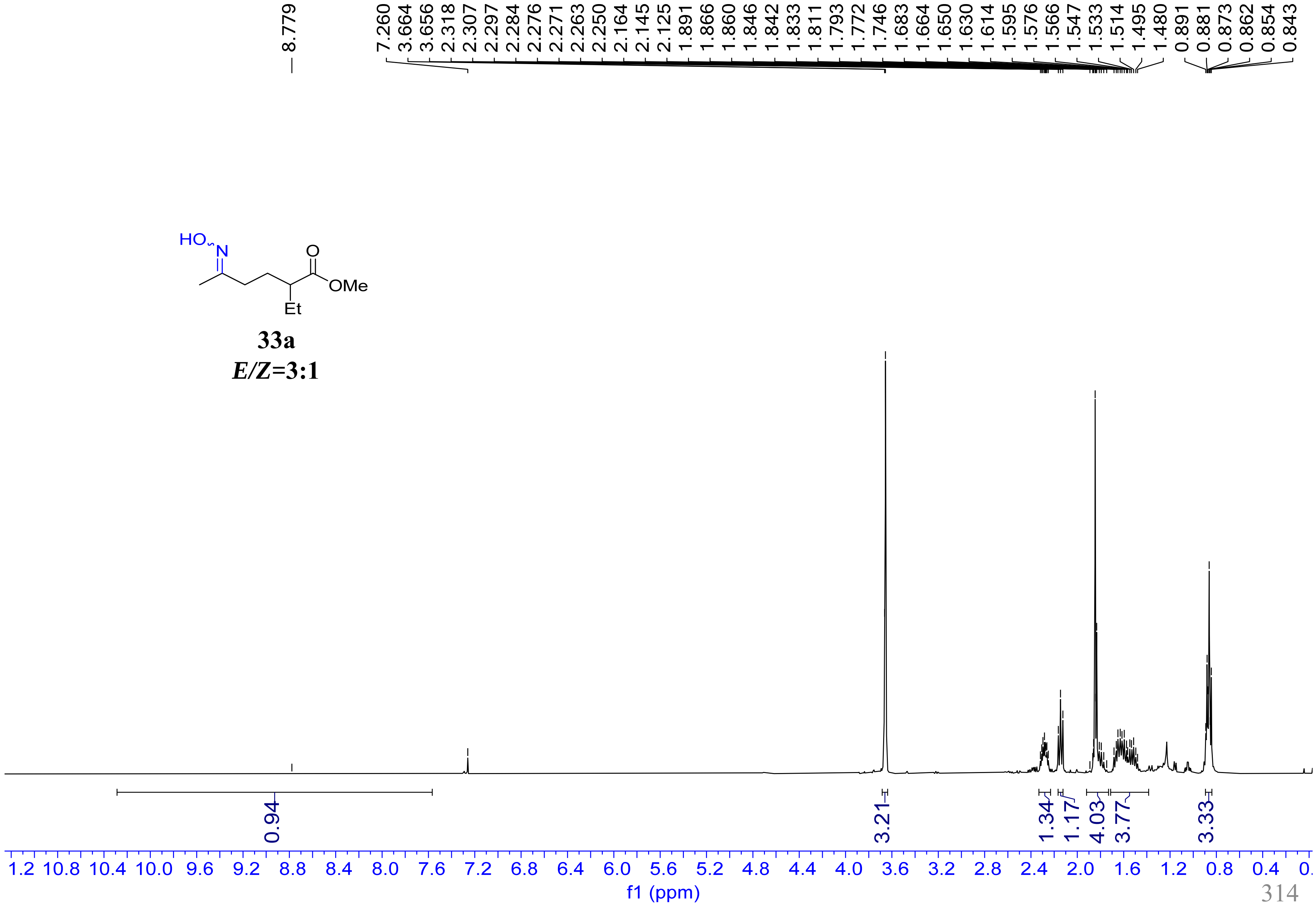

33a

***E*/*Z*=3:1**

^{13}C NMR 101 MHz, $CDCl_3$

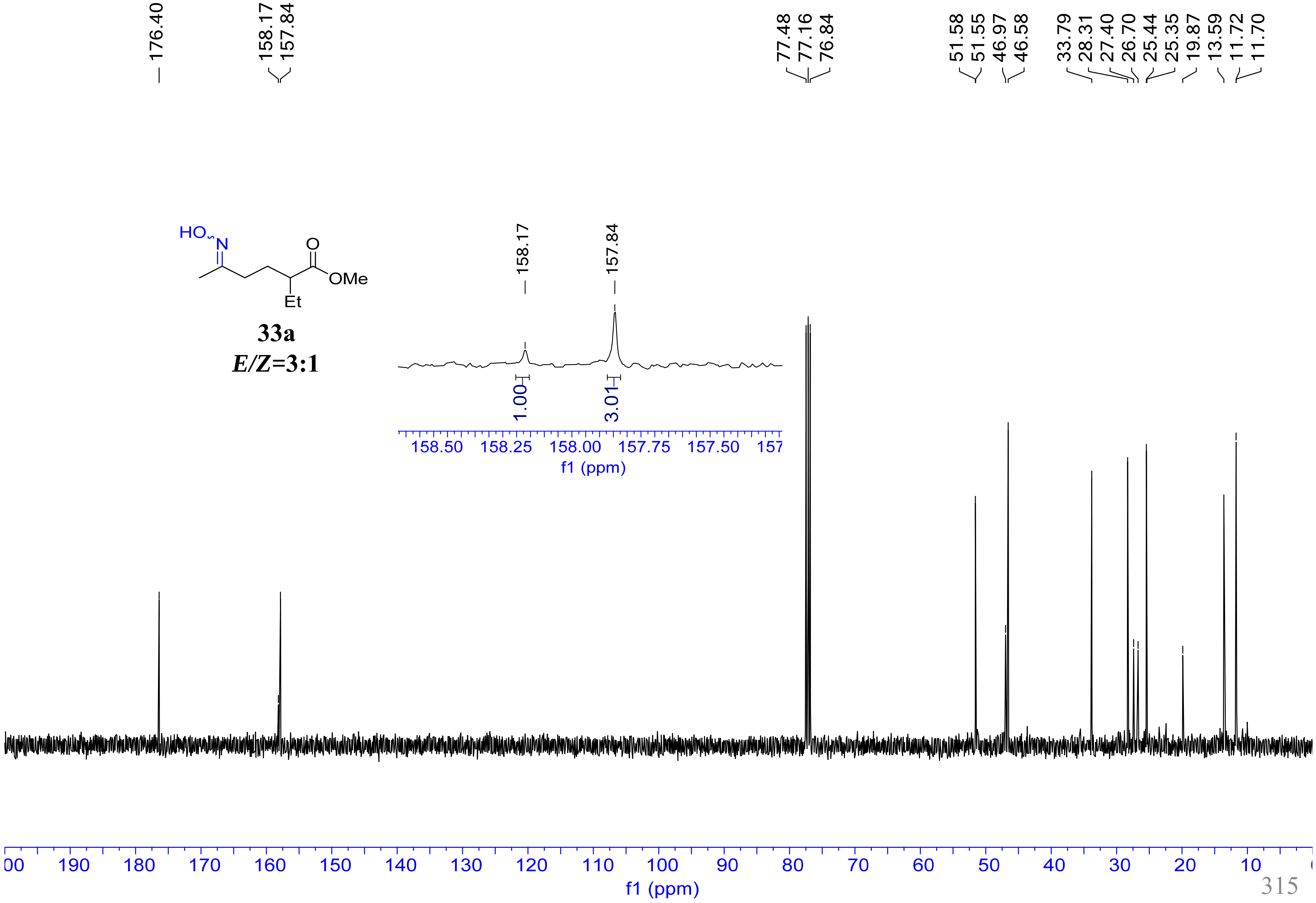

¹H NMR, 400 MHz, $CDCl_3$

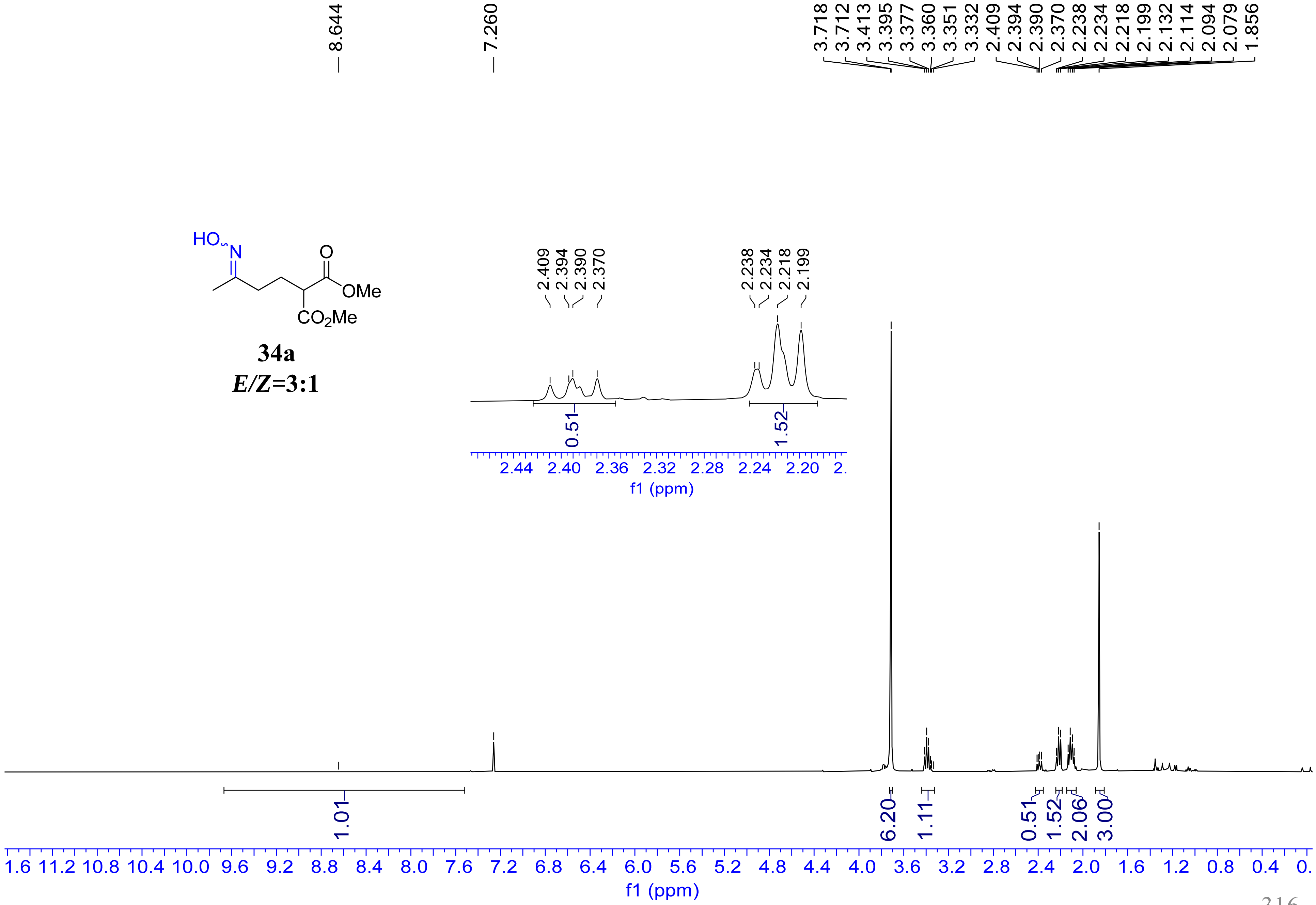

^{13}C NMR 101 MHz, $CDCl_3$

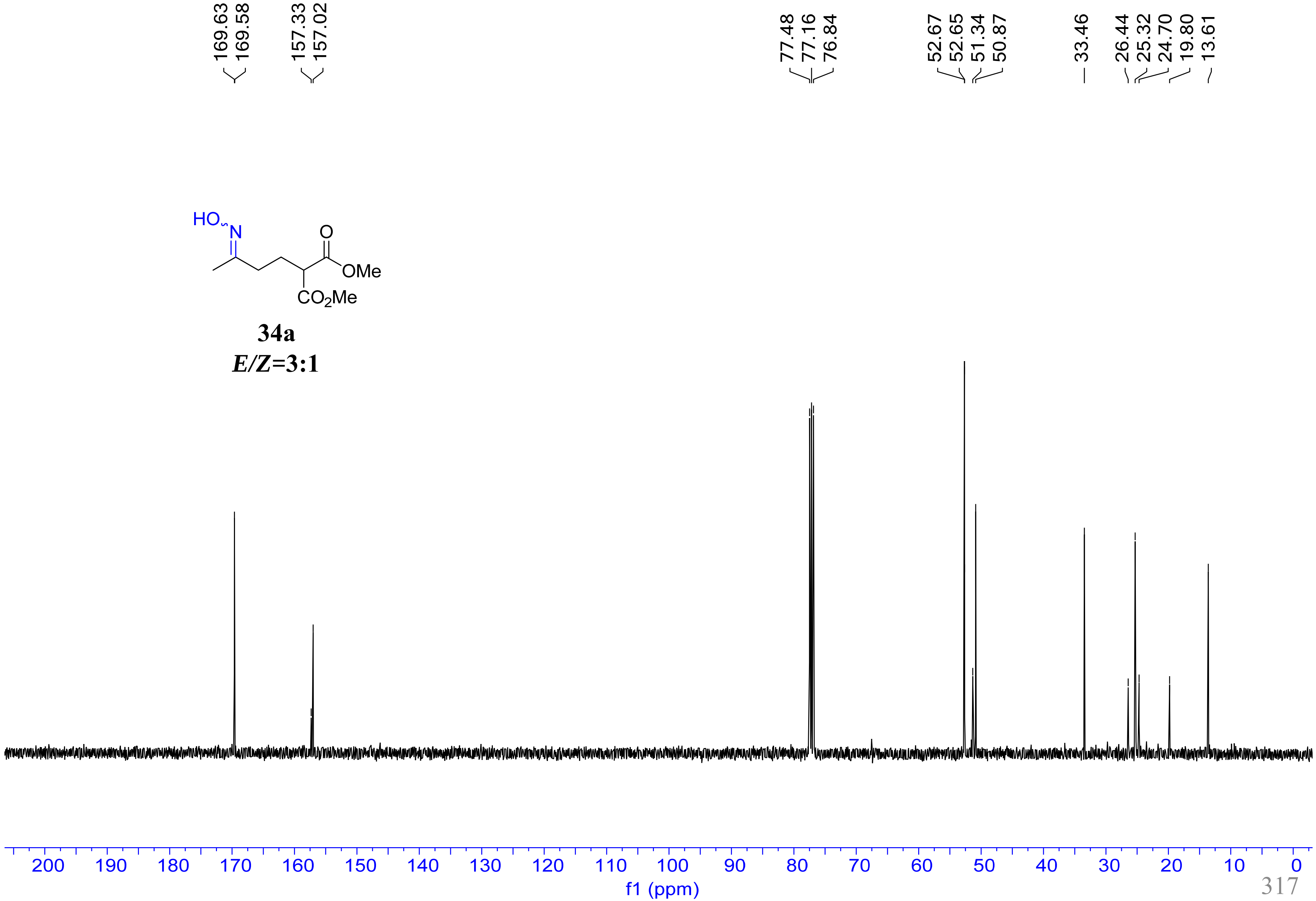

^{1}H NMR, 400 MHz, $CDCl_3$

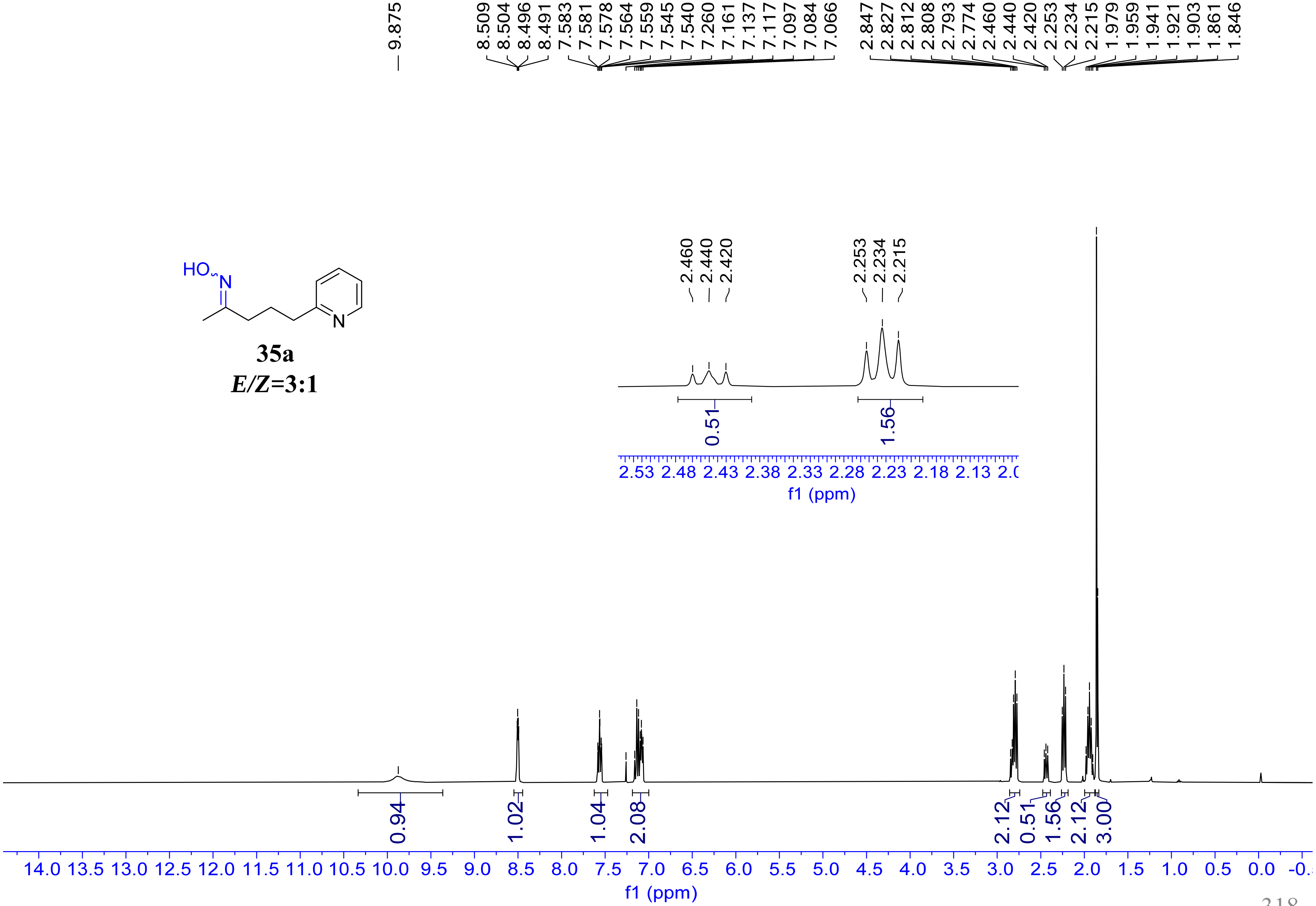

35a

***E/Z*=3:1**

^{13}C NMR 101 MHz, $CDCl_3$

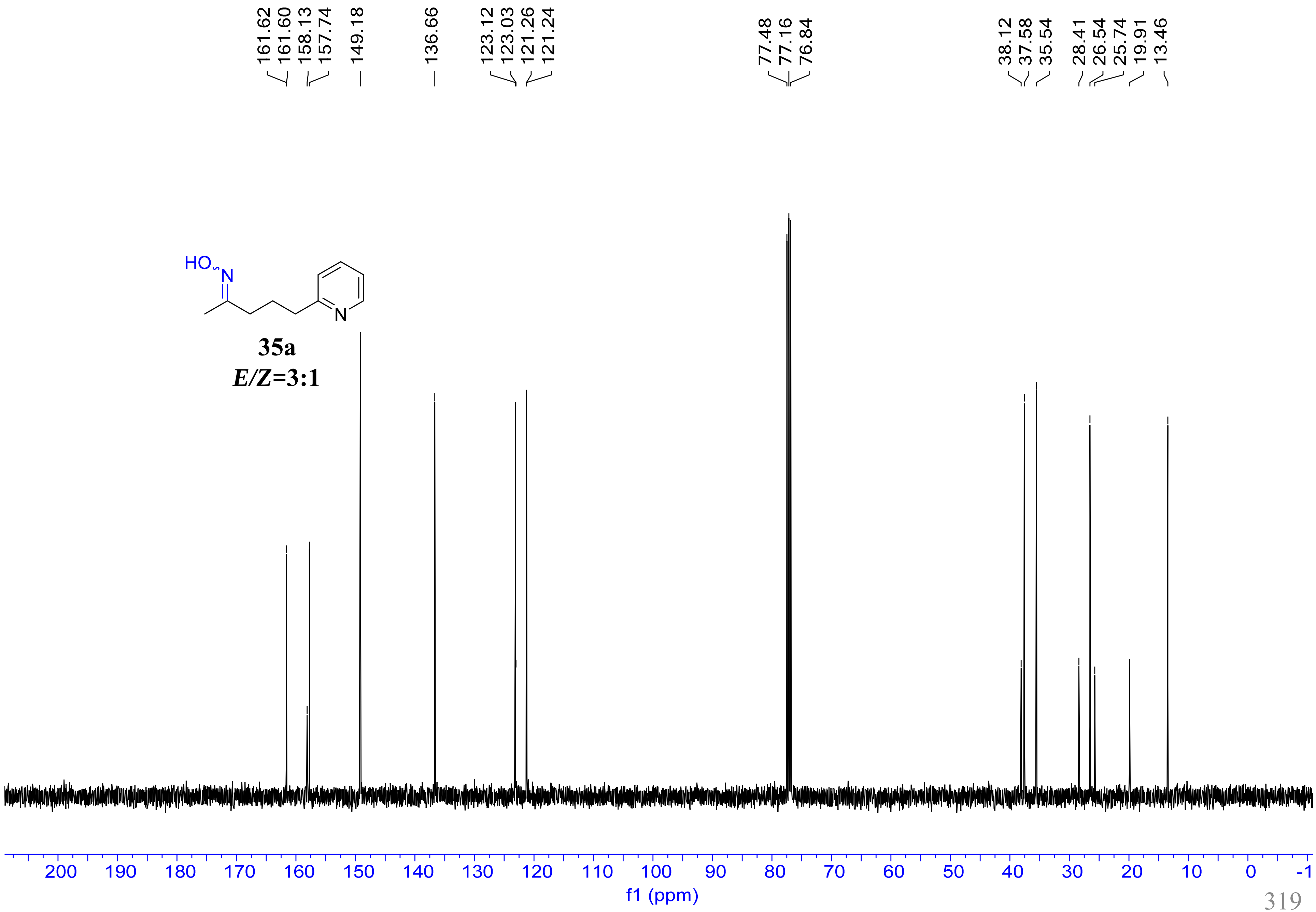

35a
***E/Z*=3:1**

^1H NMR, 400 MHz, $CDCl_3$

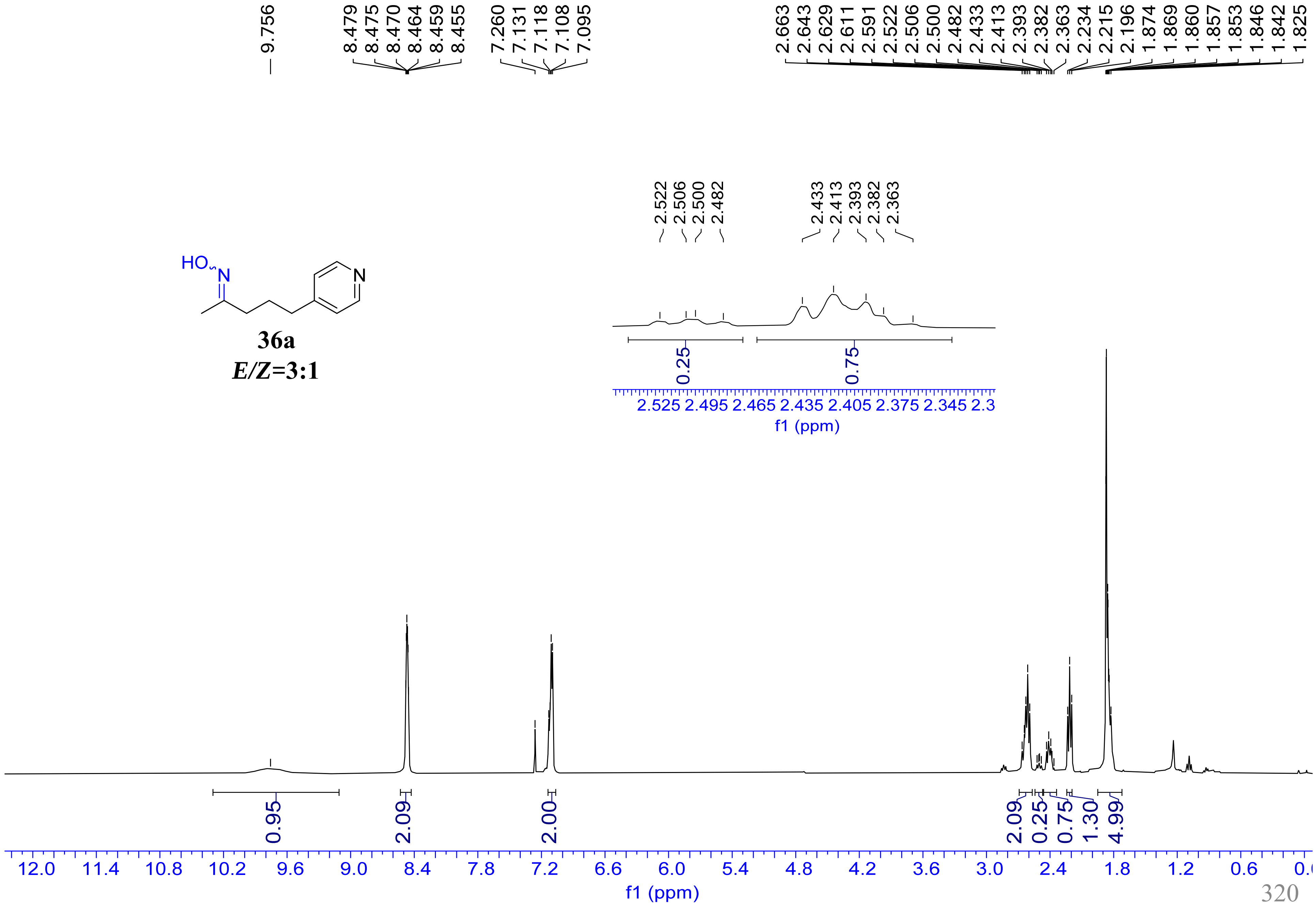

^{13}C NMR 101 MHz, $CDCl_3$

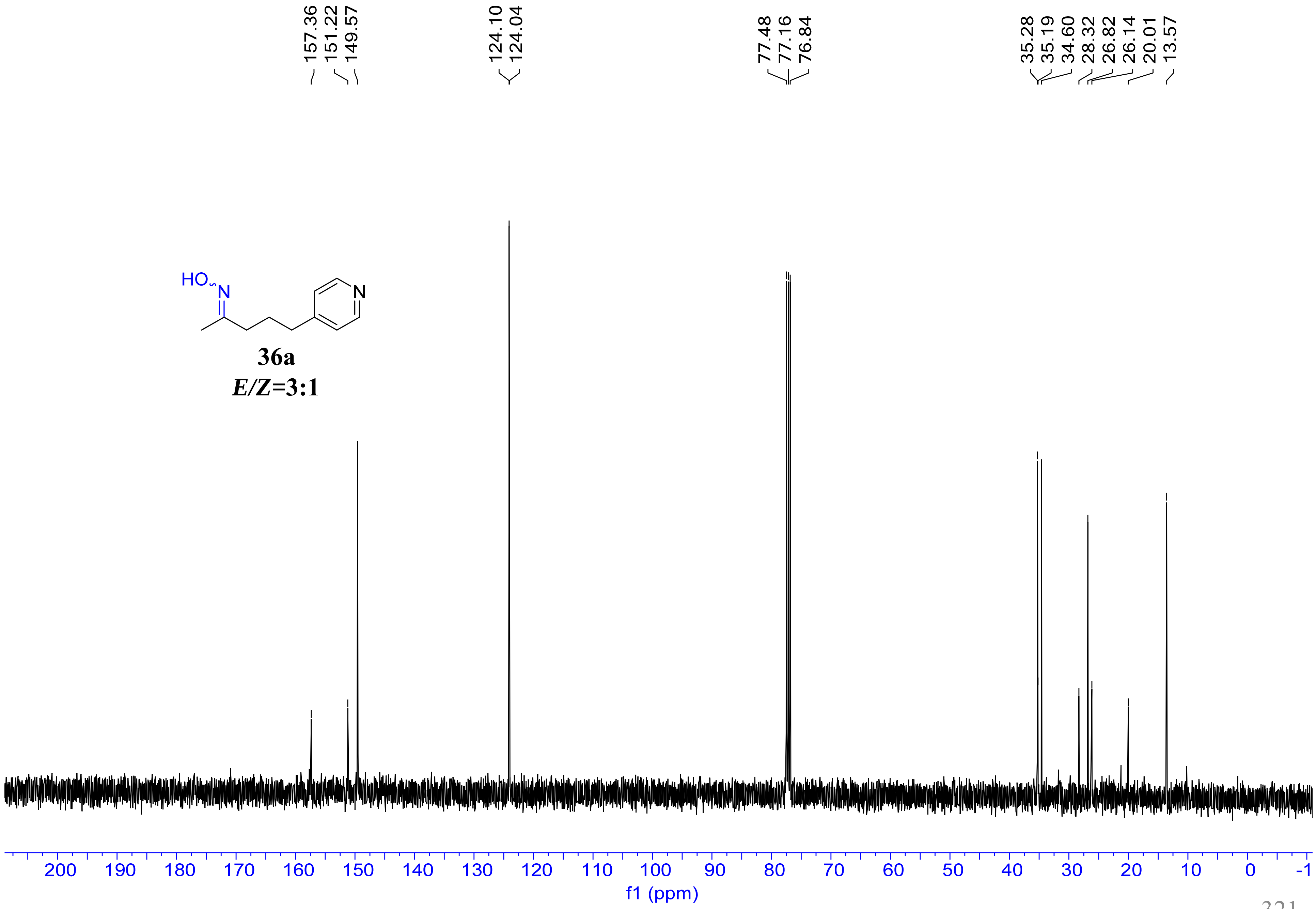

36a

***E/Z*=3:1**

^{1}H NMR, 400 MHz, $CDCl_3$

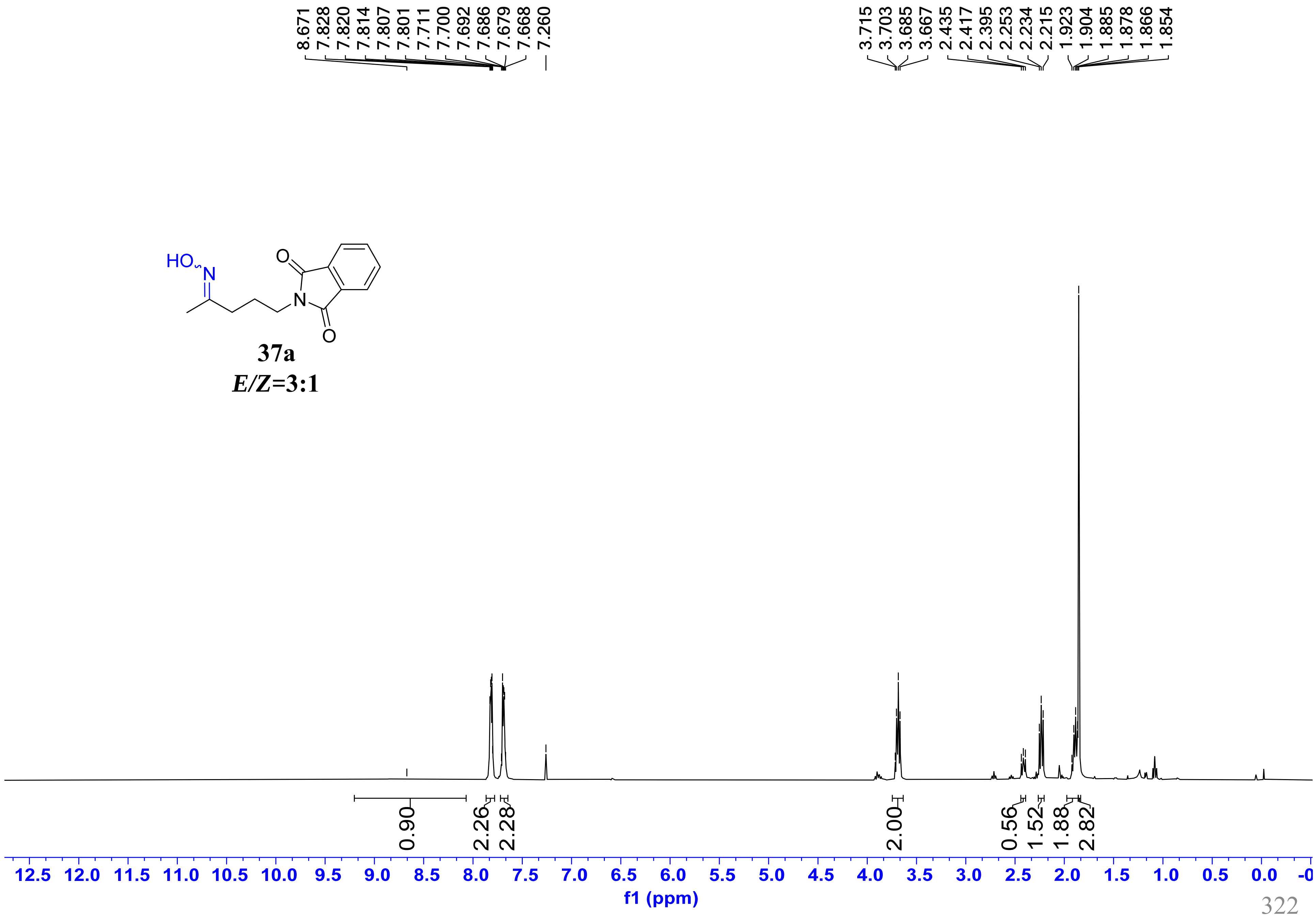

^{13}C NMR 101 MHz, $CDCl_3$

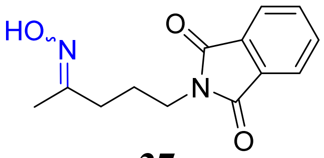

37a

***E/Z*=3:1**

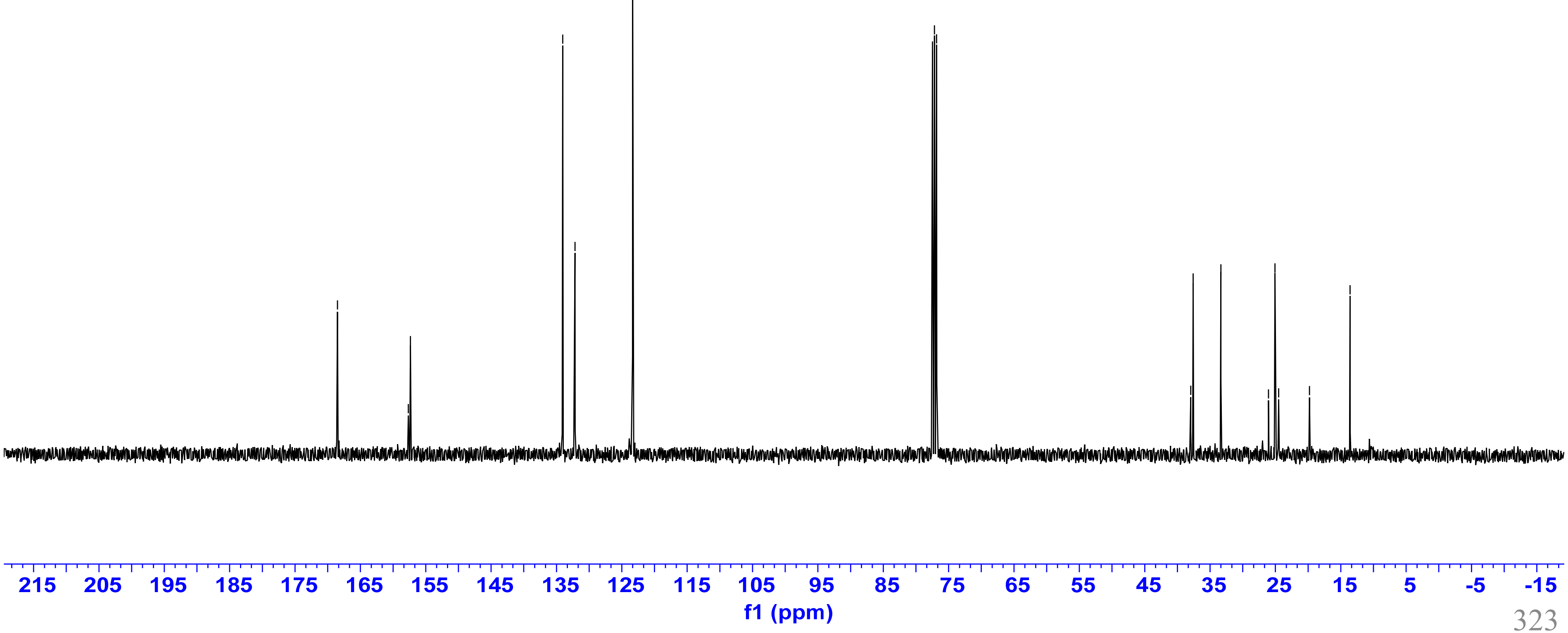

^{1}H NMR, 400 MHz, CDCl$_3$

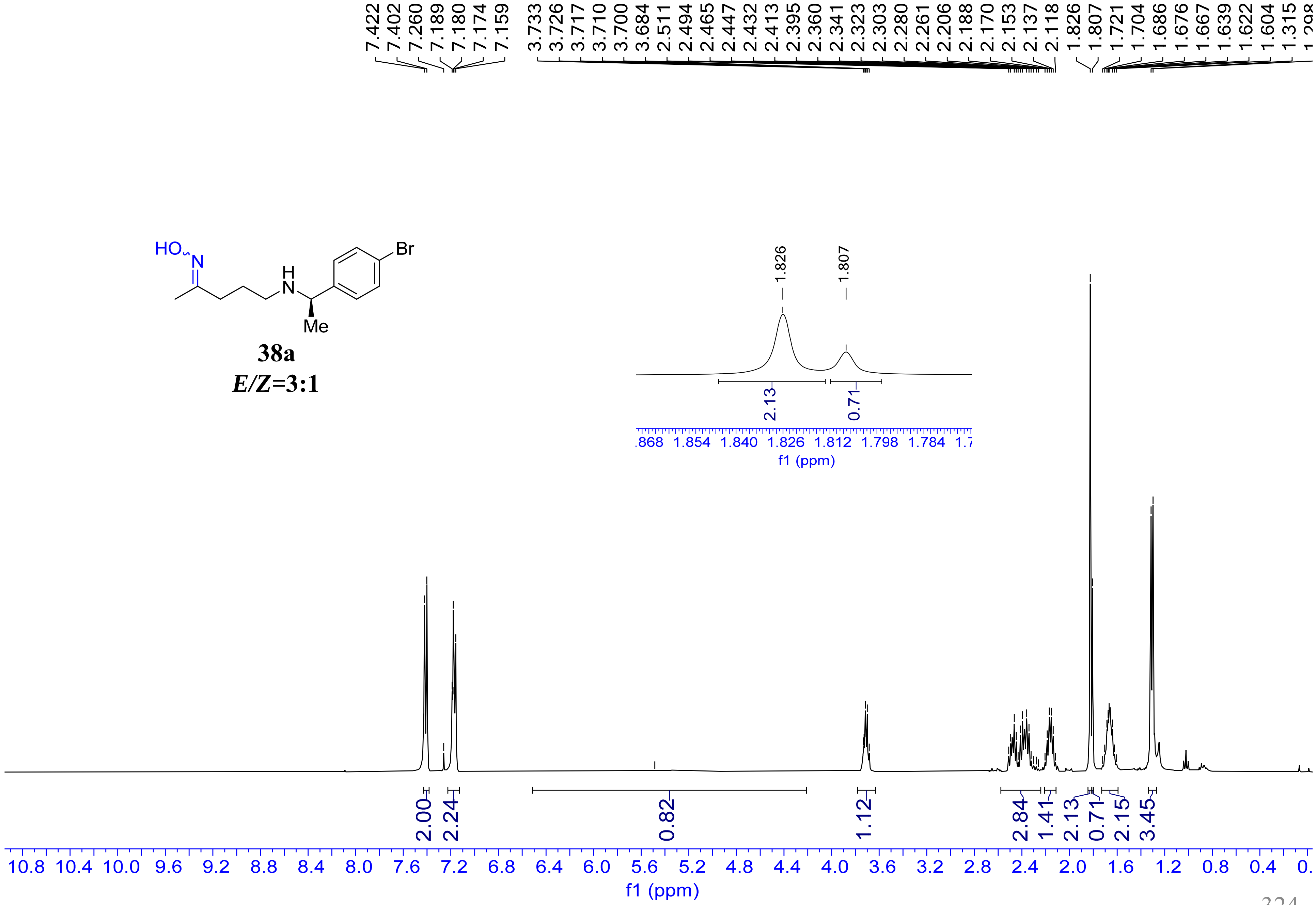

^{13}C NMR 101 MHz, $CDCl_3$

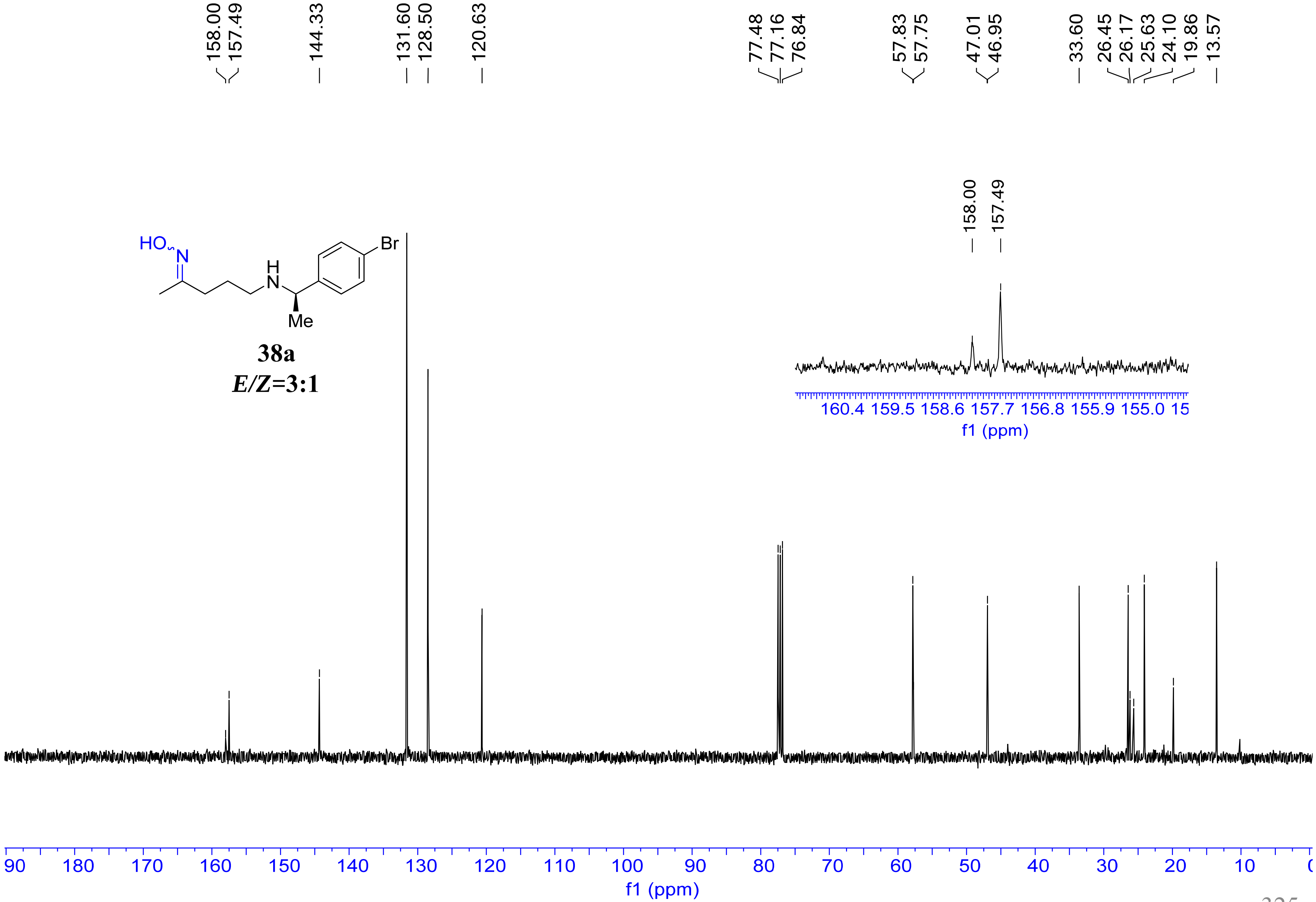

^{1}H NMR, 400 MHz, $CDCl_3$

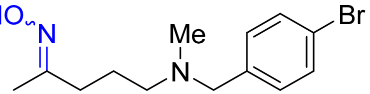

39a

***E/Z*=3:1**

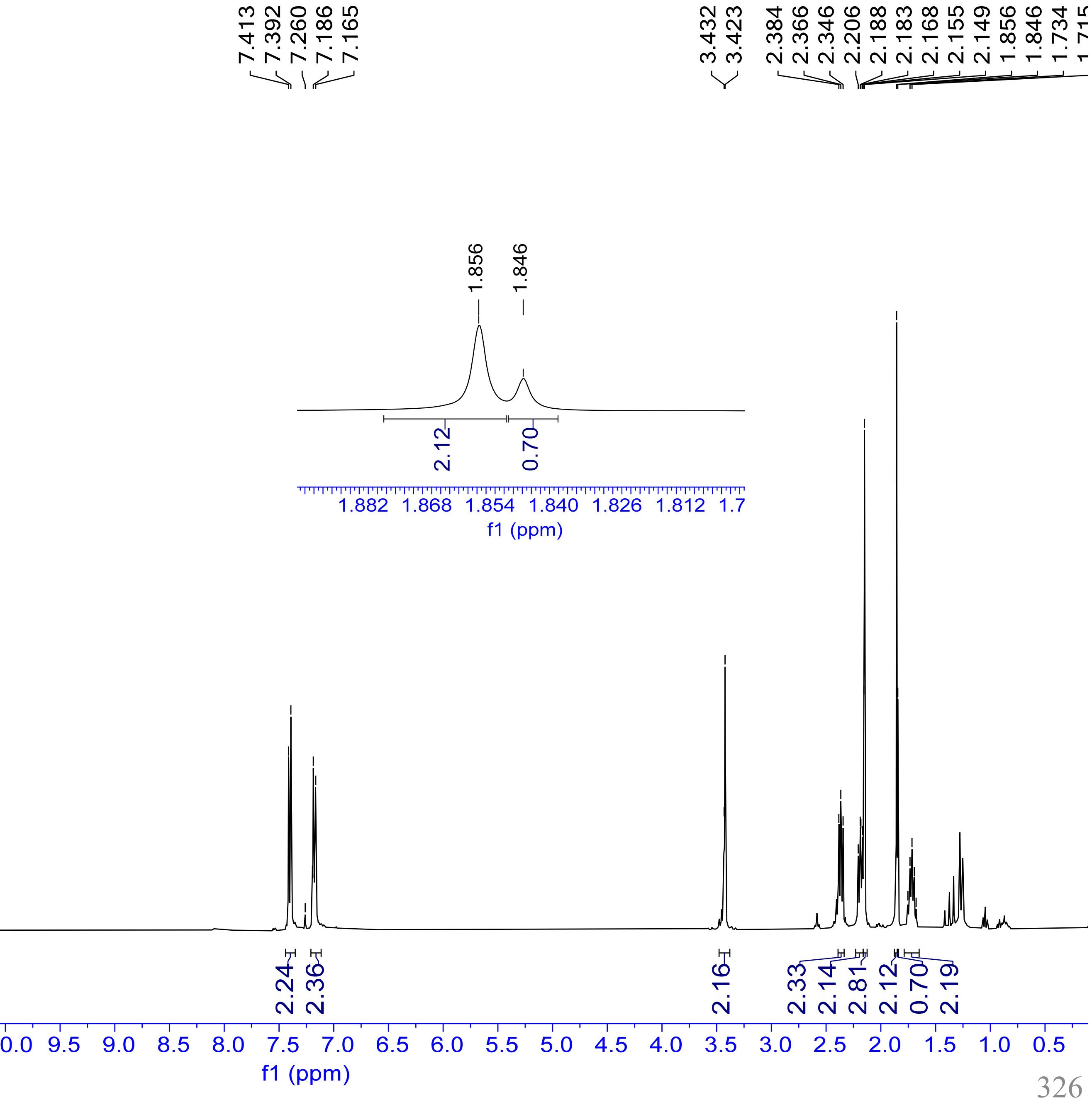

^{13}C NMR 101 MHz, $CDCl_3$

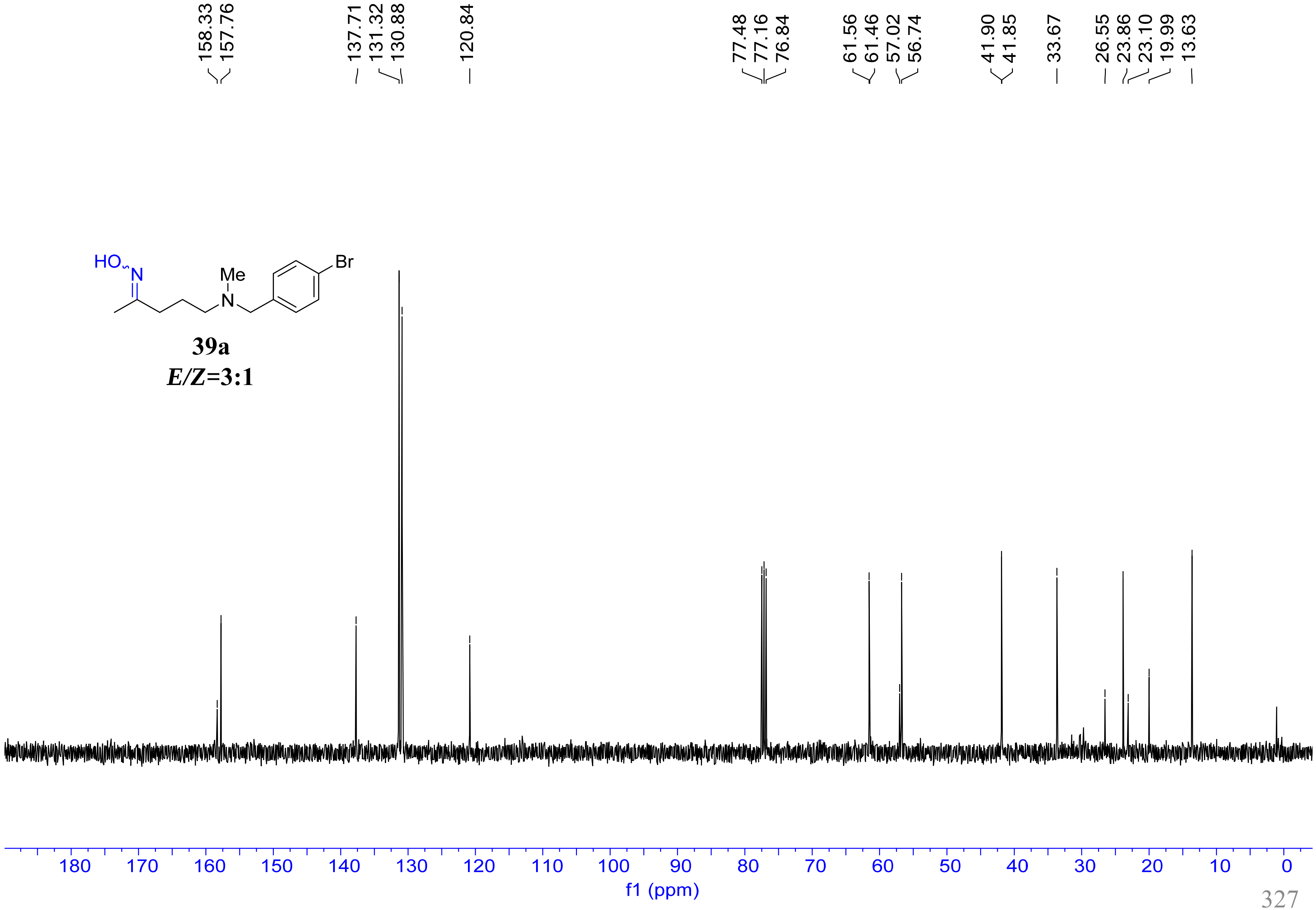

^{1}H NMR, 400 MHz, $CDCl_3$

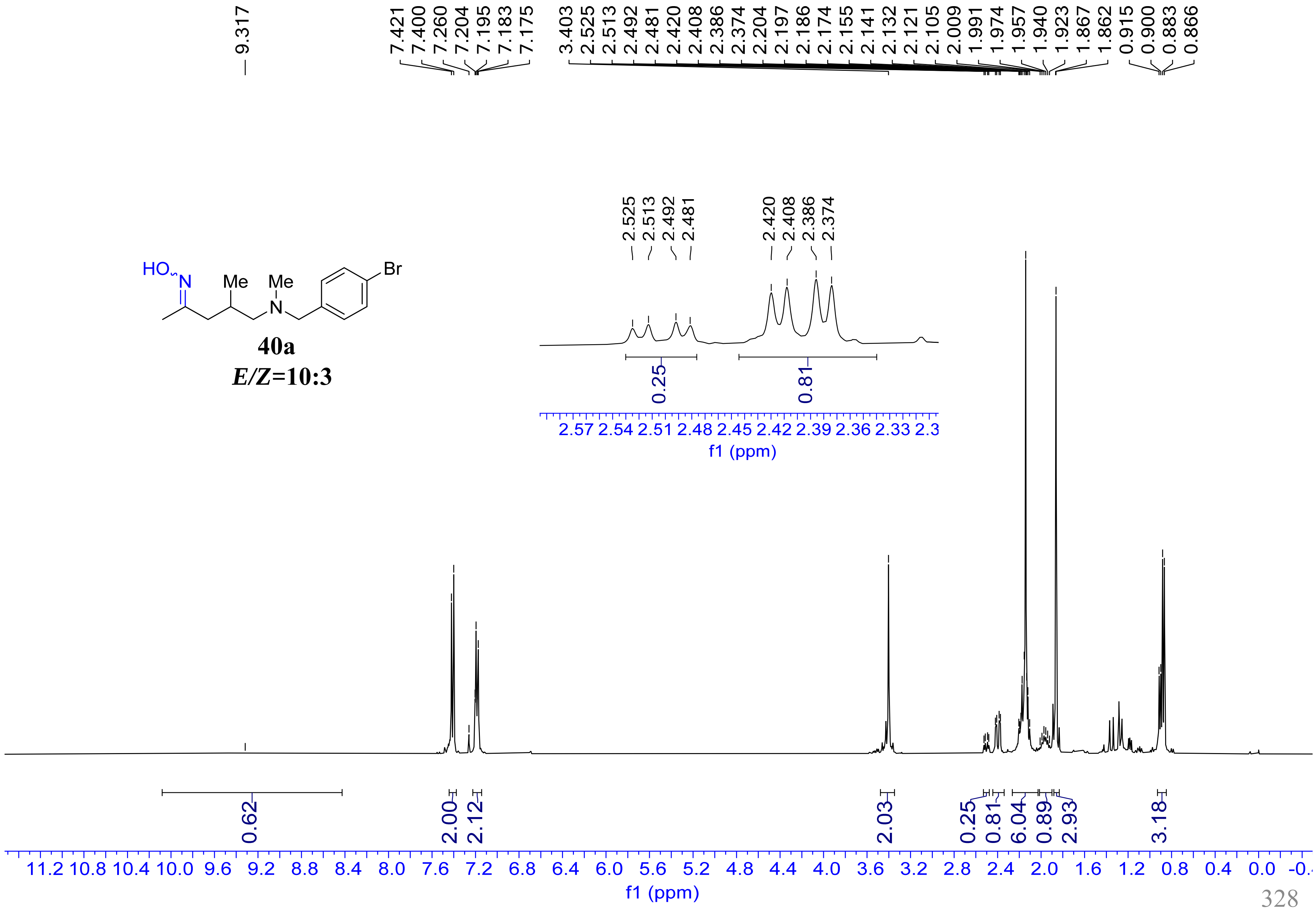

40a

***E/Z*=10:3**

^{13}C NMR 101 MHz, $CDCl_3$

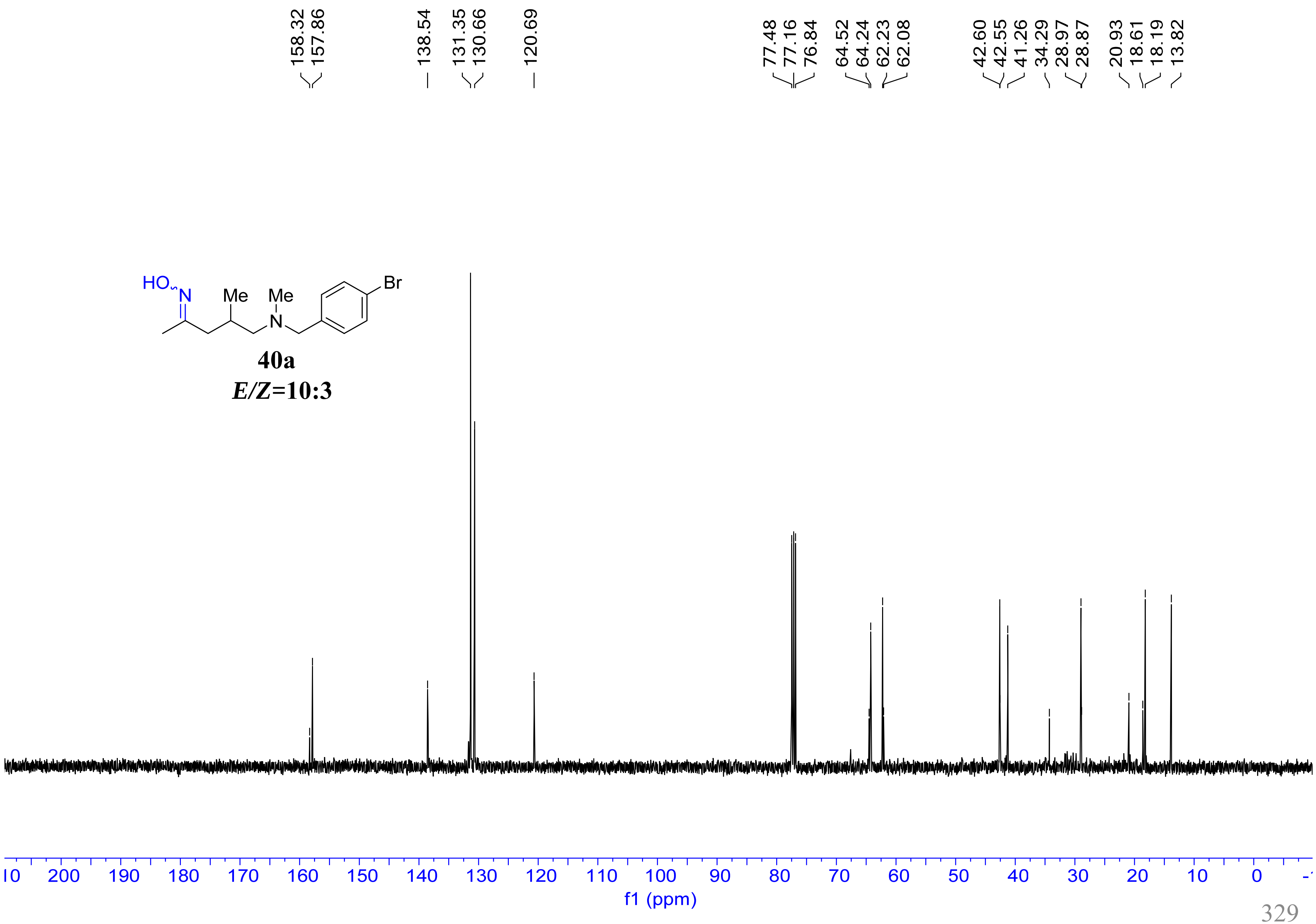

40a

***E/Z*=10:3**

^{1}H NMR, 400 MHz, $CDCl_3$

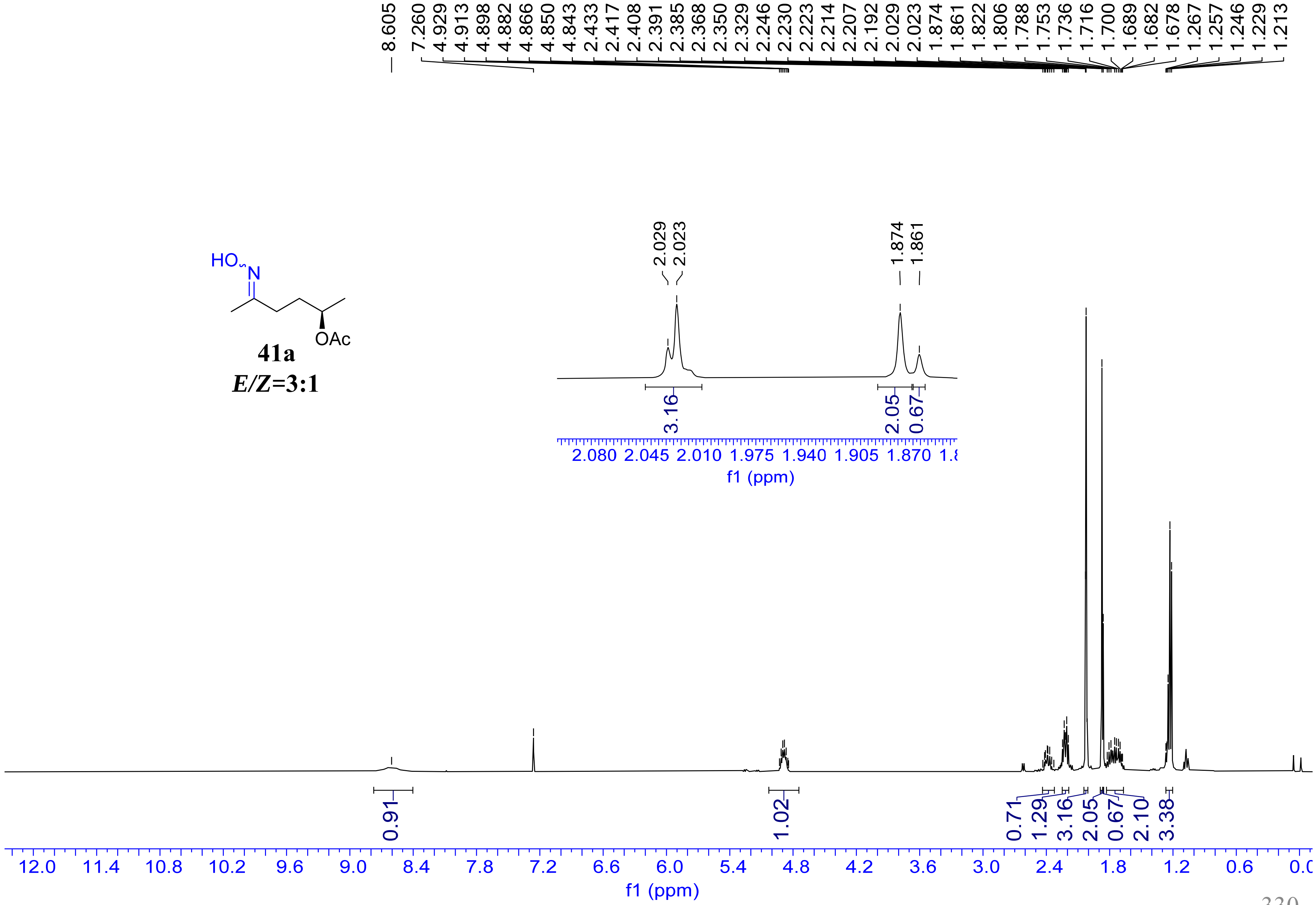

^{13}C NMR 101 MHz, CDCl$_3$

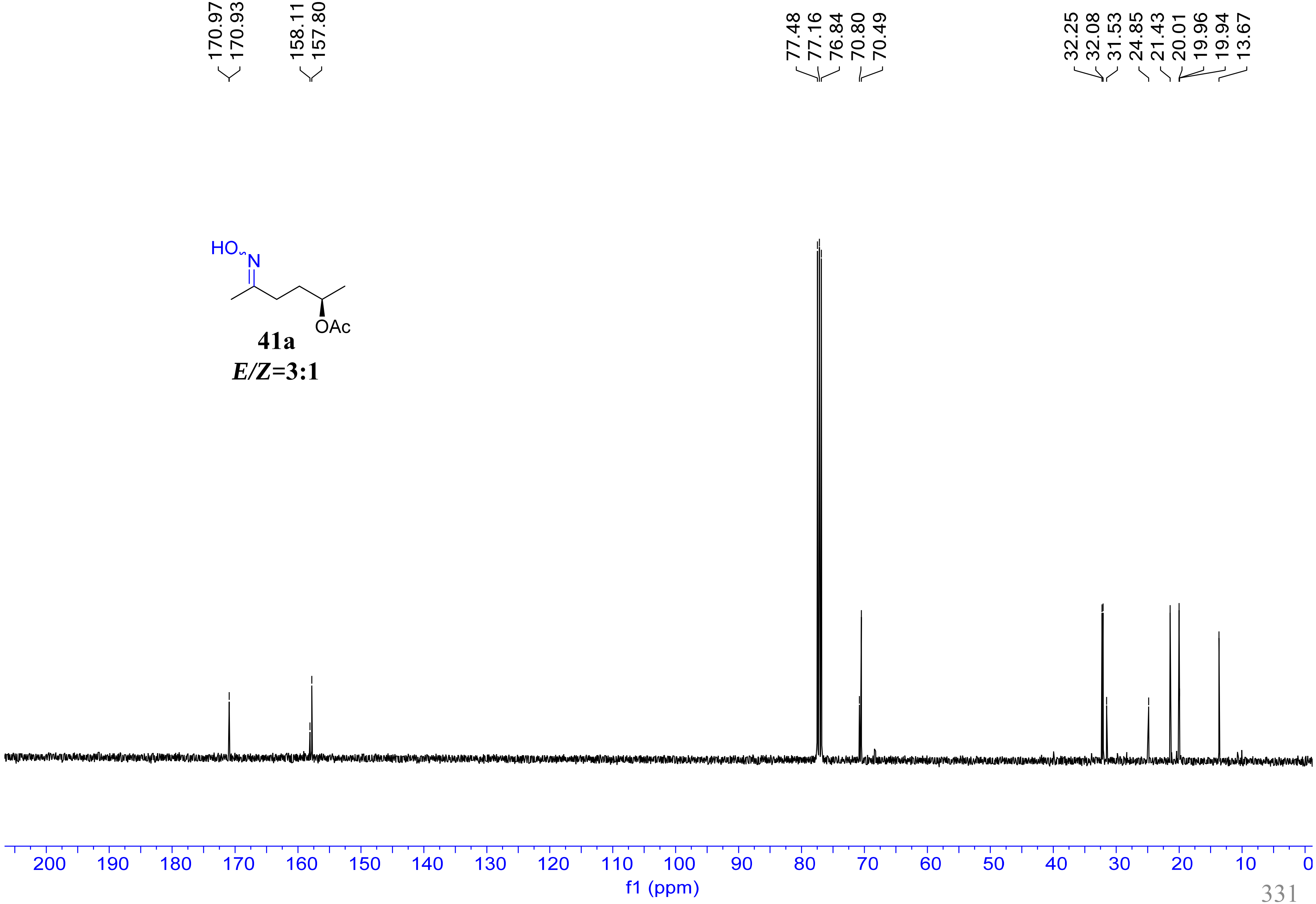

^{1}H NMR, 400 MHz, $CDCl_3$

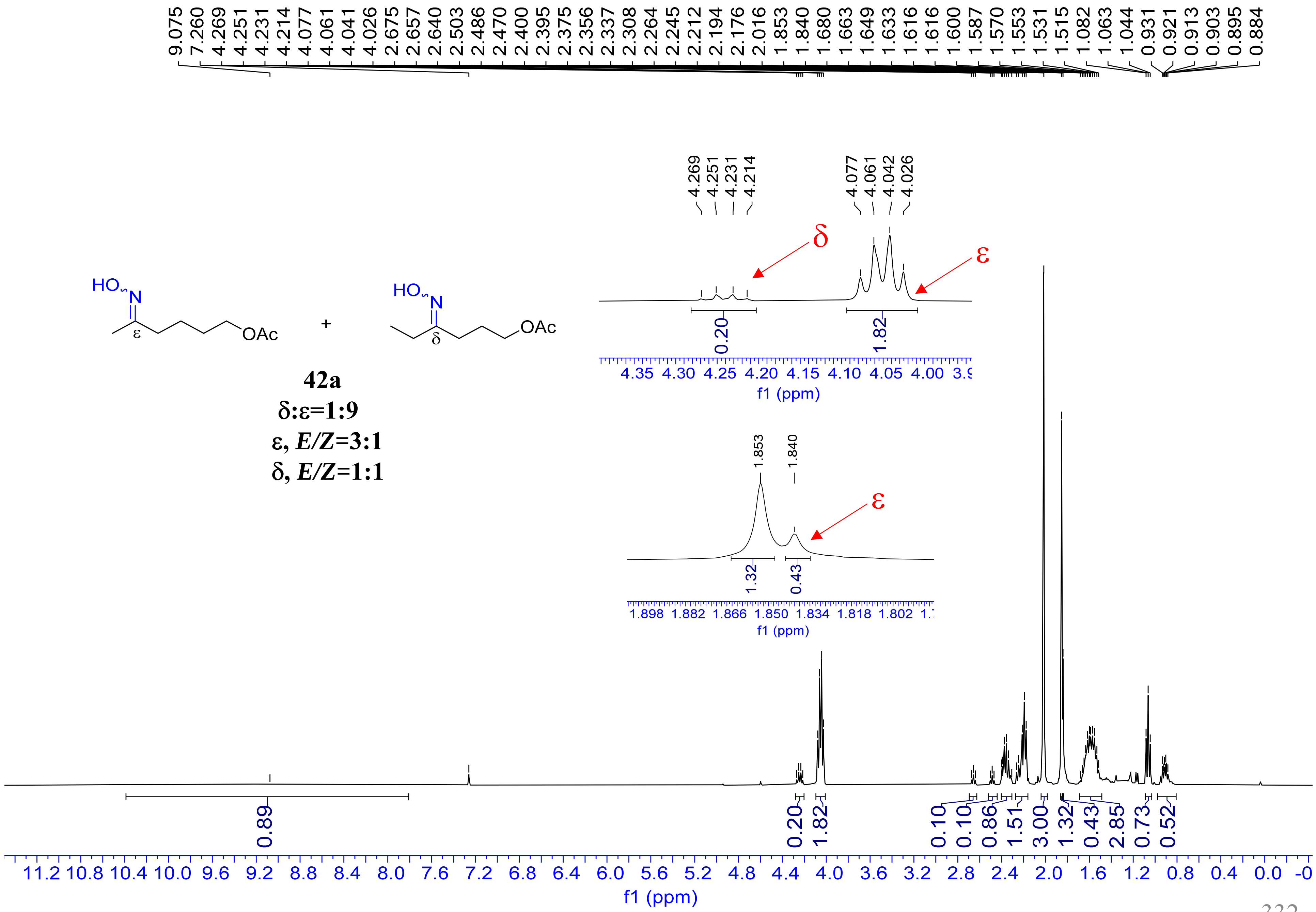

^{13}C NMR 101 MHz, $CDCl_3$

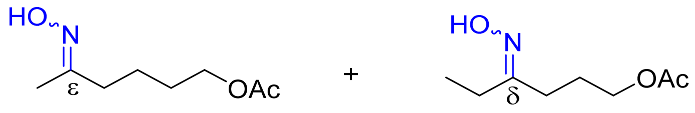

42a

δ:ε=1:9

ε, *E/Z*=3:1

δ, *E/Z*=1:1

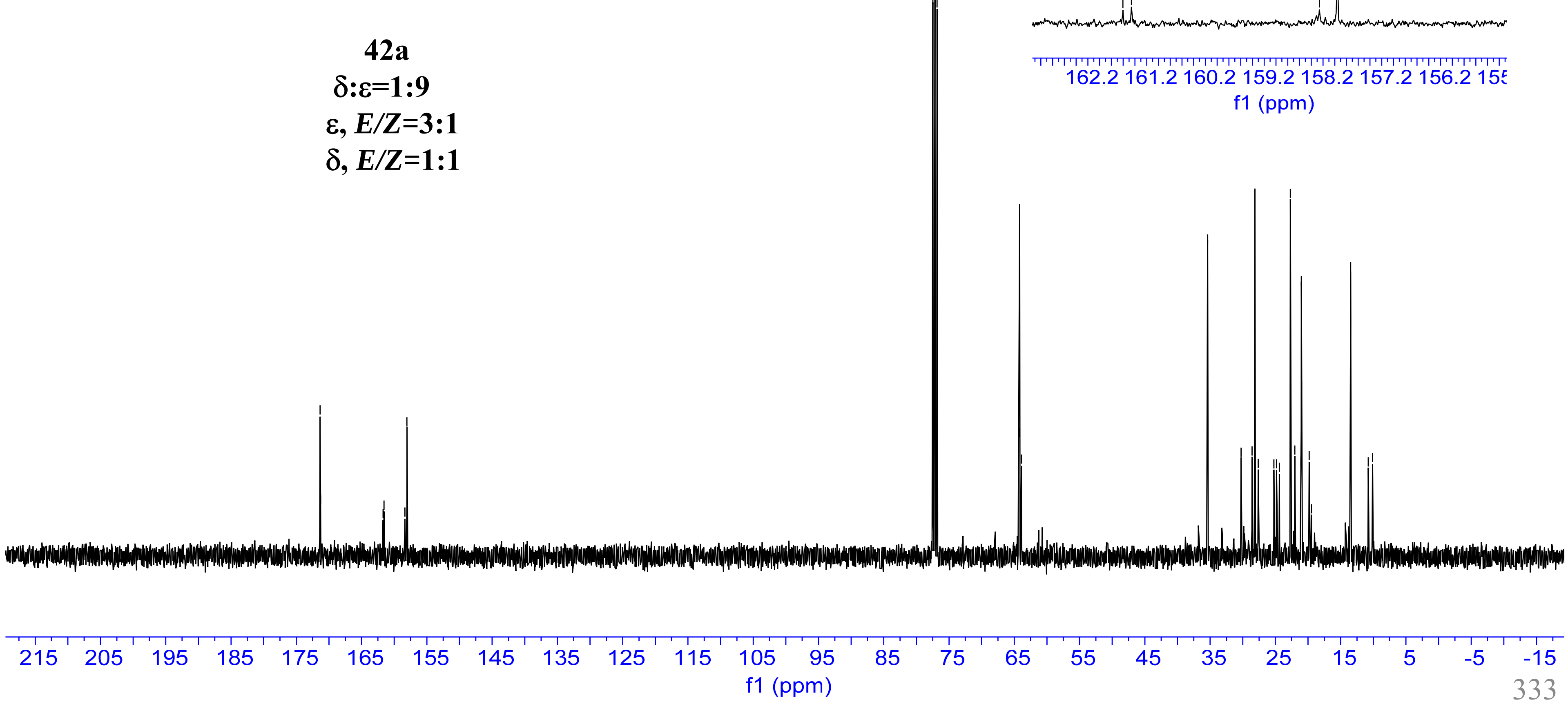

¹H NMR, 400 MHz, $CDCl_3$

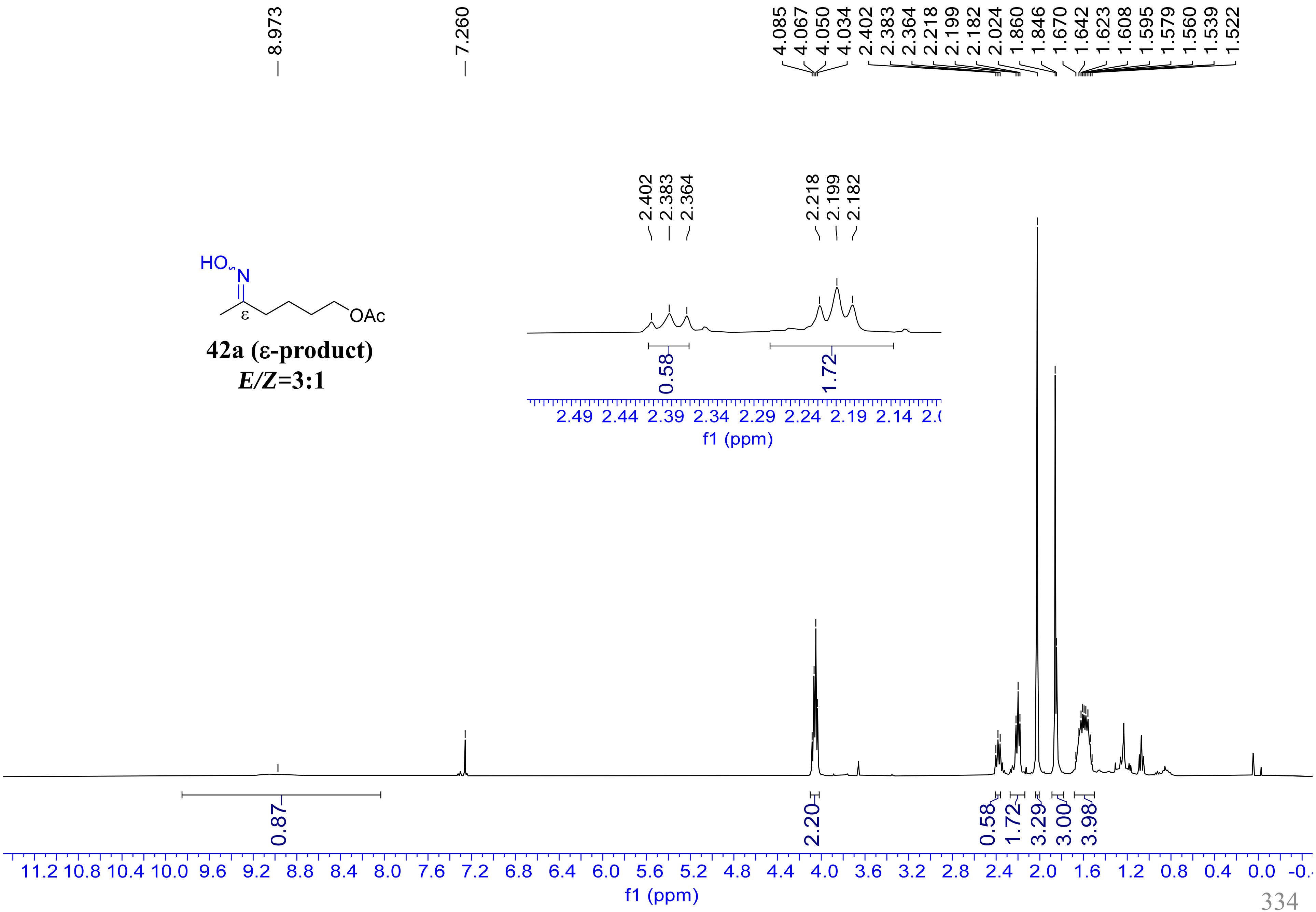

^{13}C NMR 101 MHz, $CDCl_3$

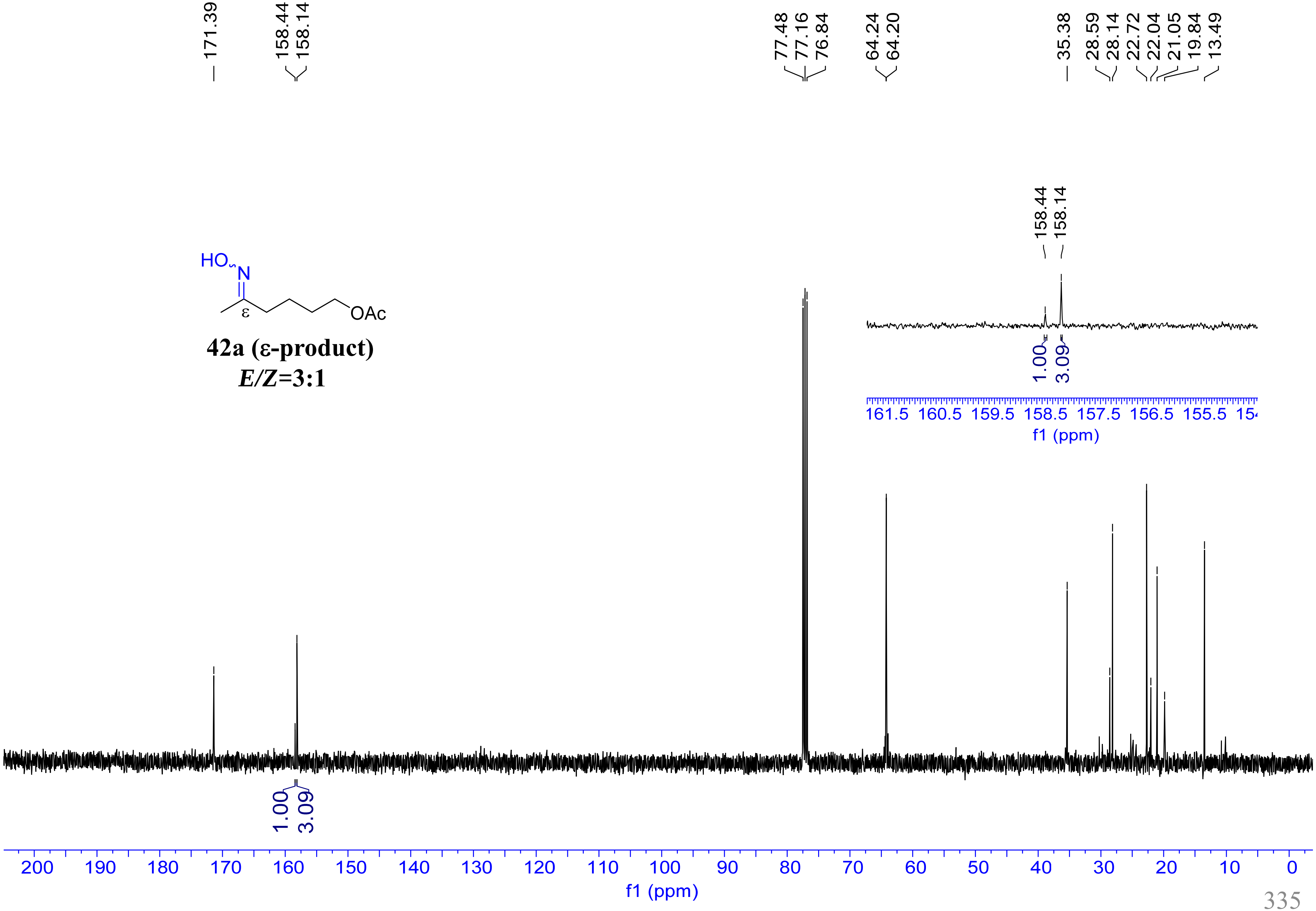

^{1}H NMR, 400 MHz, $CDCl_3$

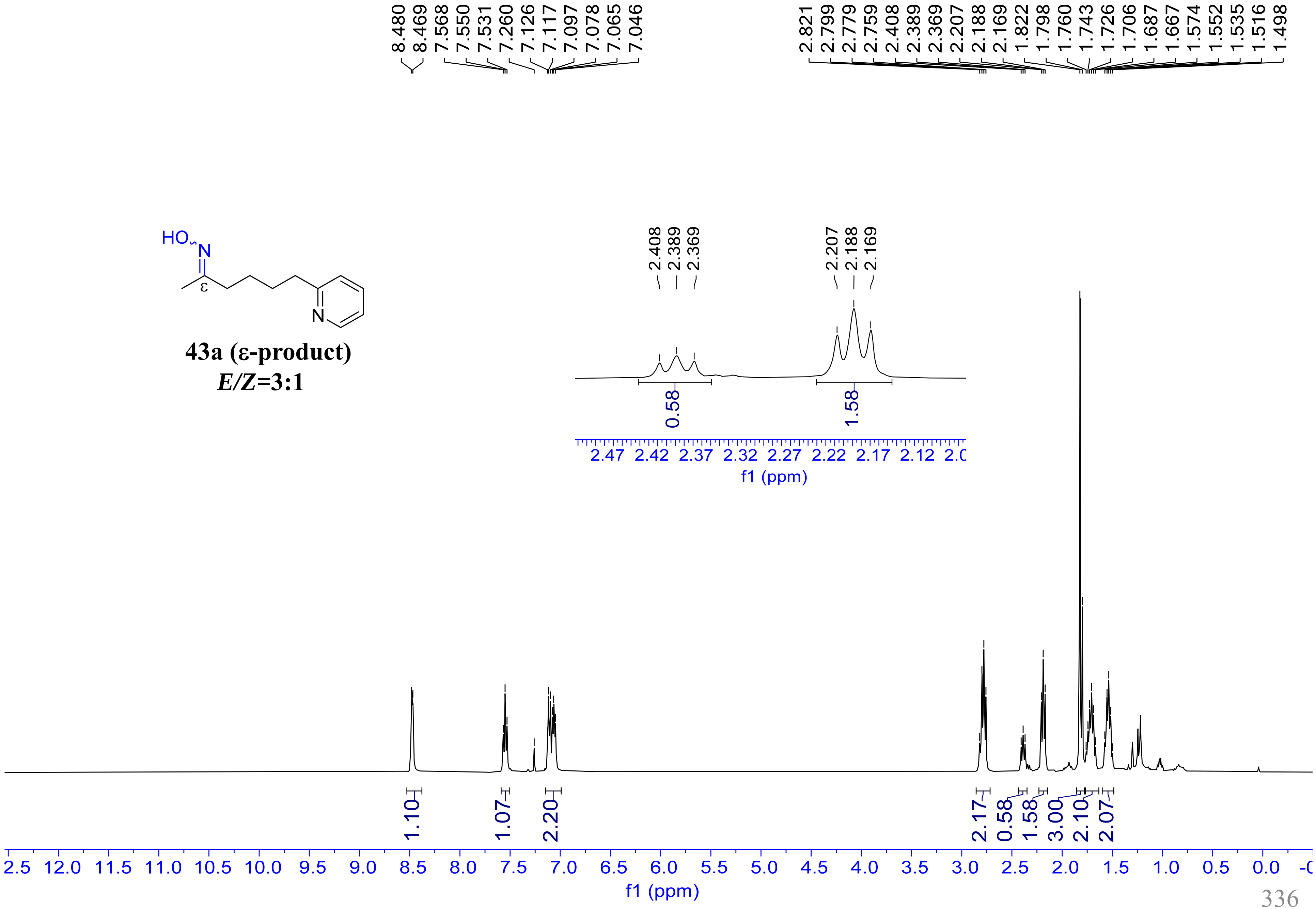

^{13}C NMR 101 MHz, $CDCl_3$

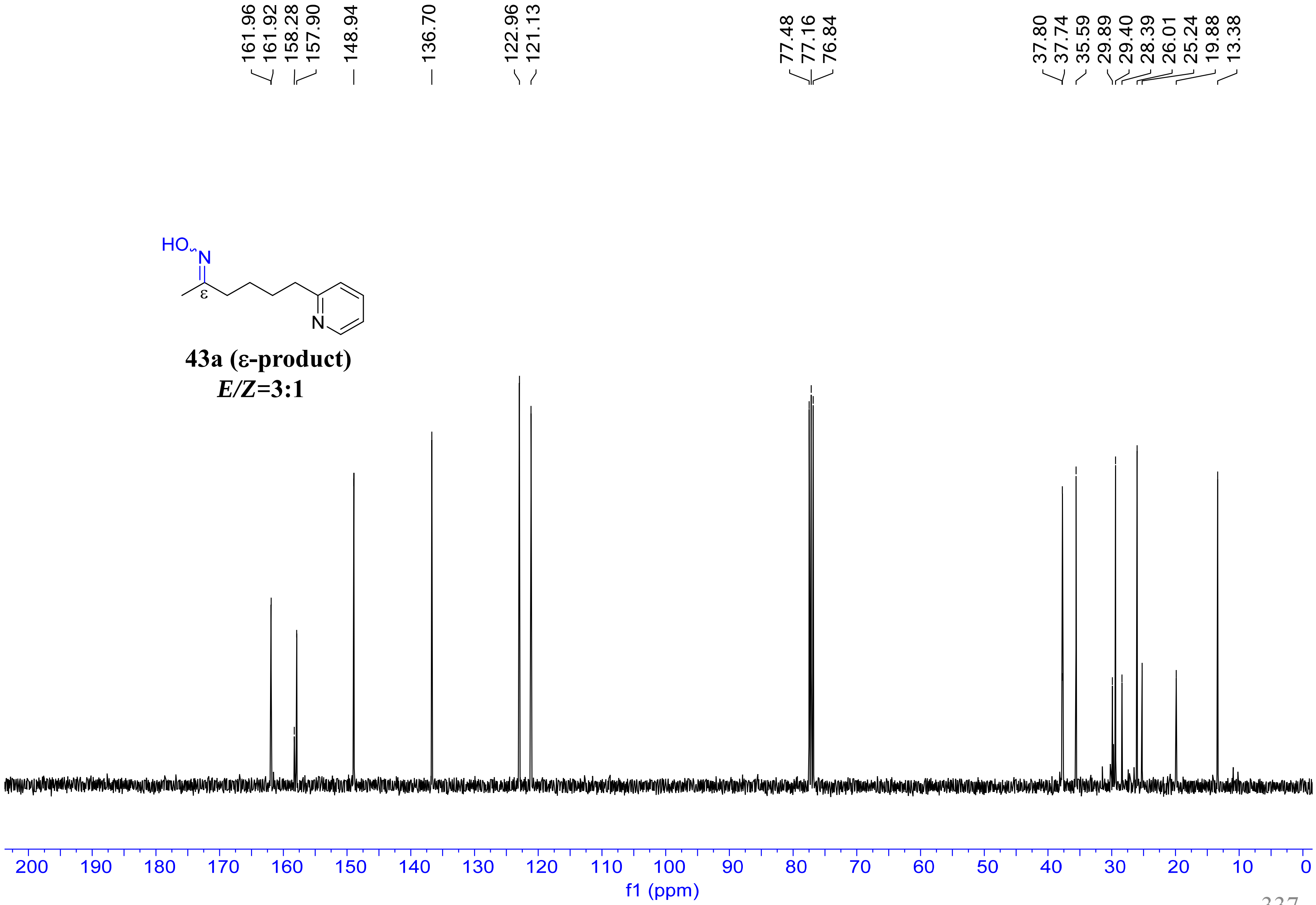

43a (ε-product)

***E/Z*=3:1**

^1H NMR, 400 MHz, $CDCl_3$

43a (δ-product)
***E/Z*=1:1**

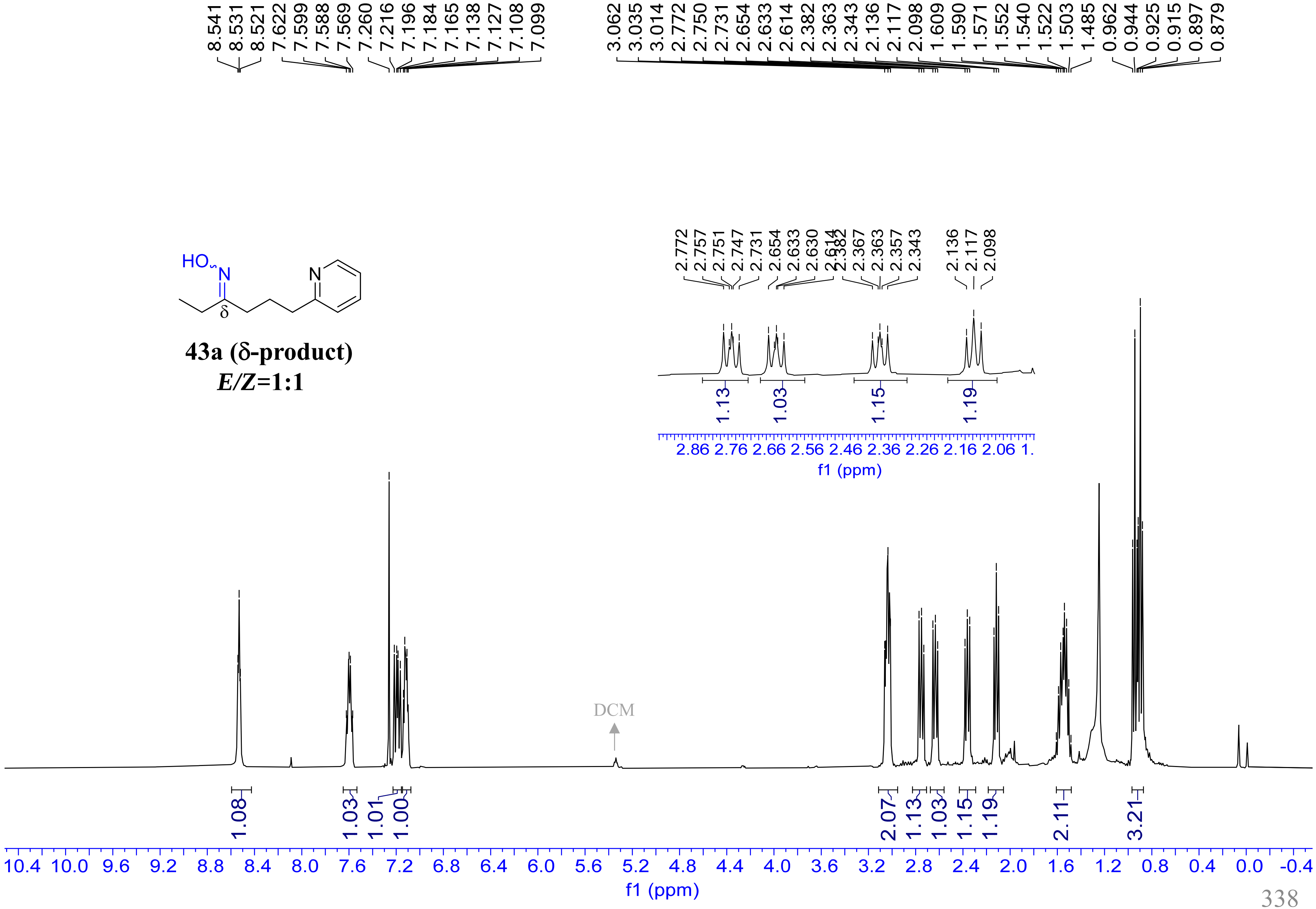

^{13}C NMR 101 MHz, $CDCl_3$

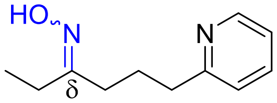

43a (δ-product)

***E/Z*=1:1**

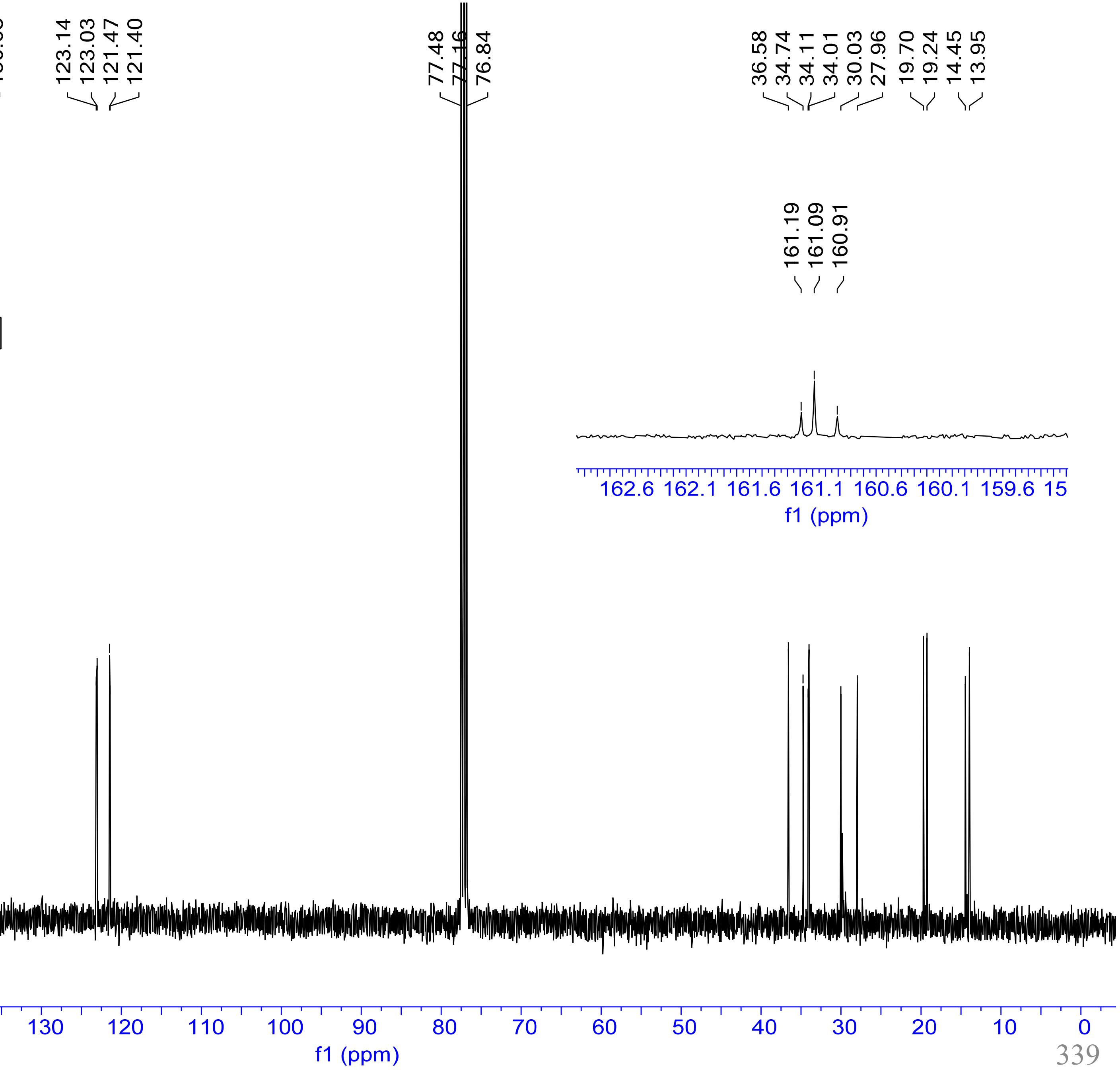

^{1}H NMR, 400 MHz, $CDCl_3$

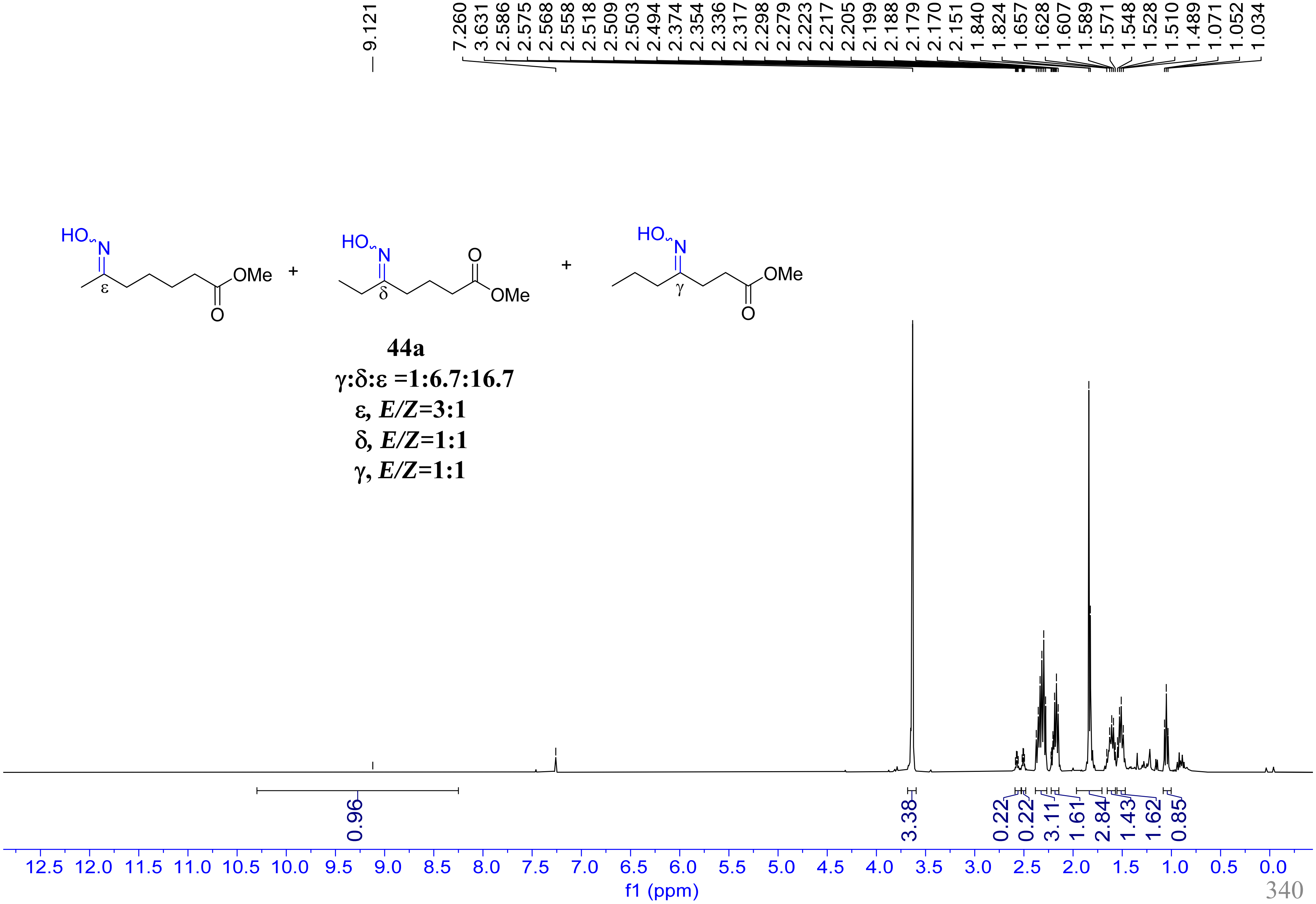

44a

γ:δ:ε =1:6.7:16.7

ε, *E*/*Z*=3:1

δ, *E*/*Z*=1:1

γ, *E*/*Z*=1:1

^{13}C NMR 101 MHz, $CDCl_3$

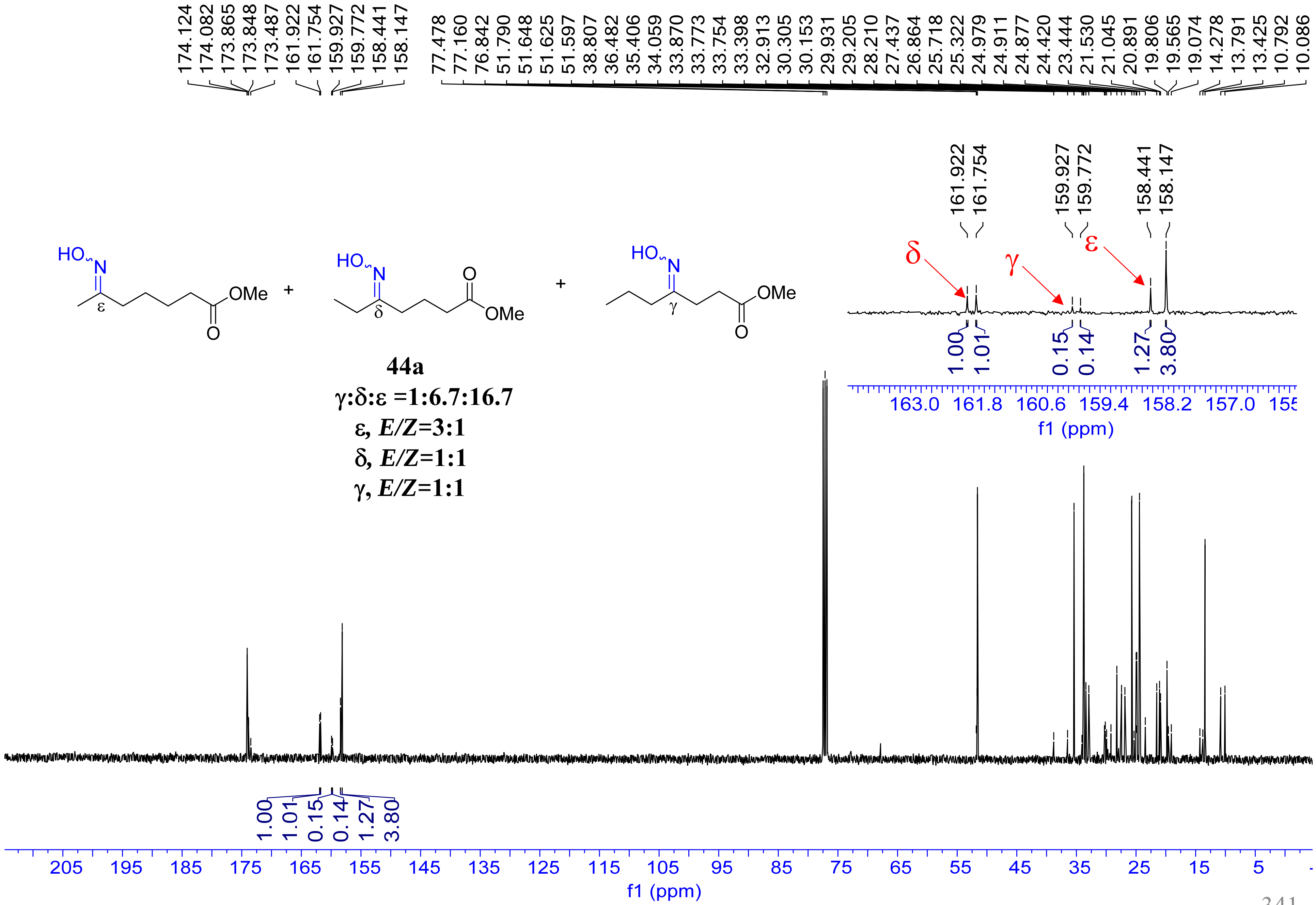

^{1}H NMR, 400 MHz, $CDCl_3$

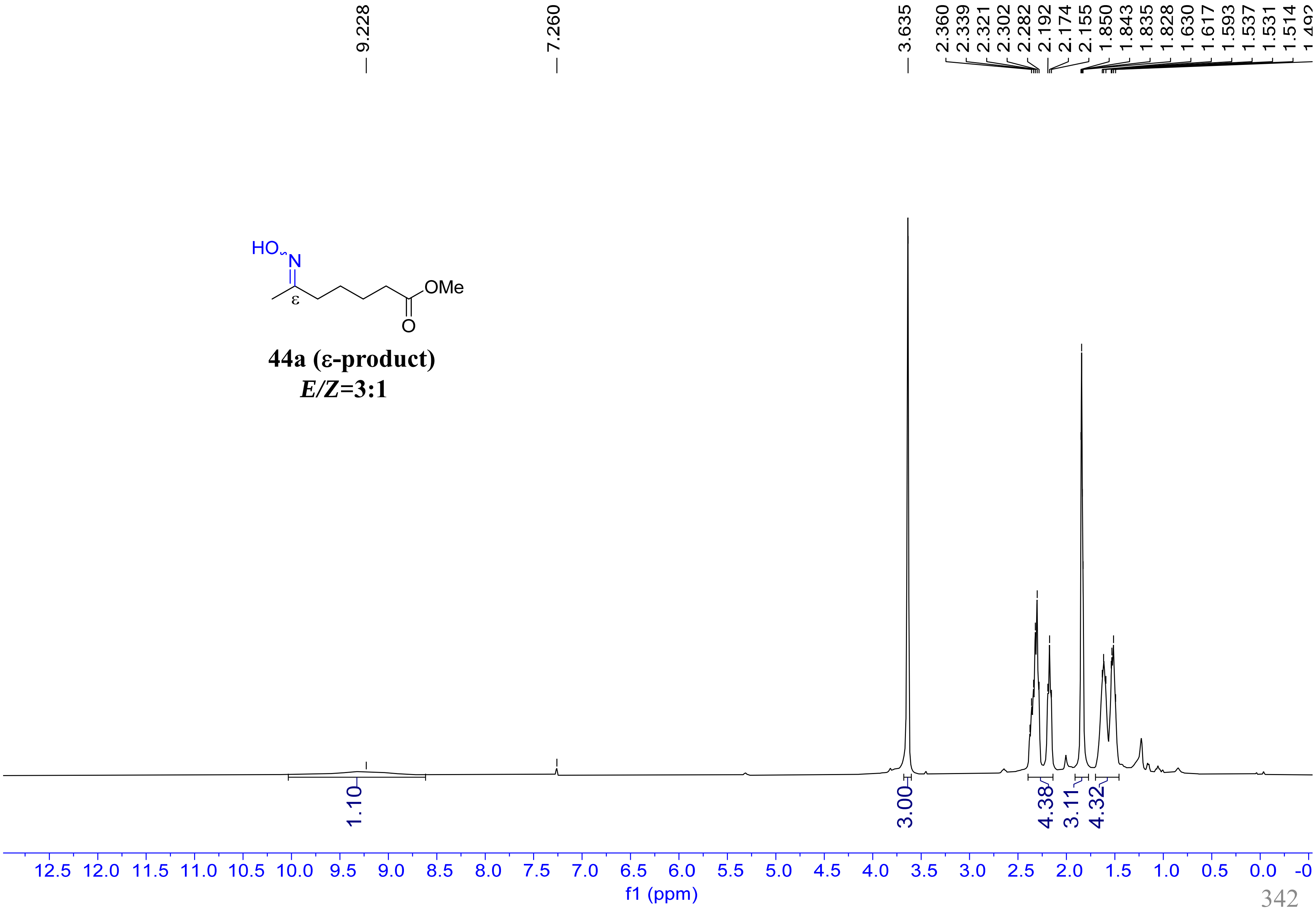

^{13}C NMR 101 MHz, $CDCl_3$

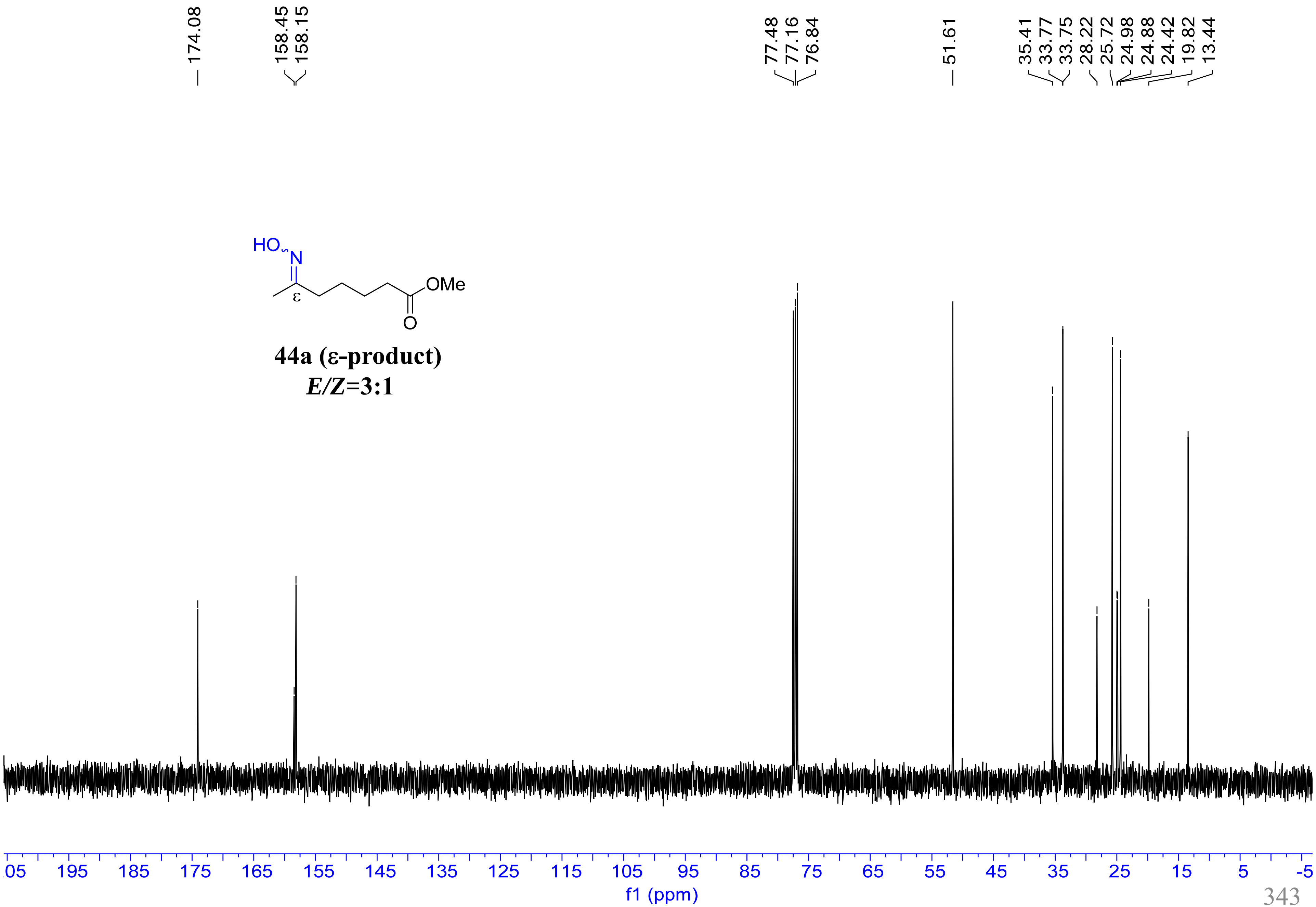

^{1}H NMR, 400 MHz, $CDCl_3$

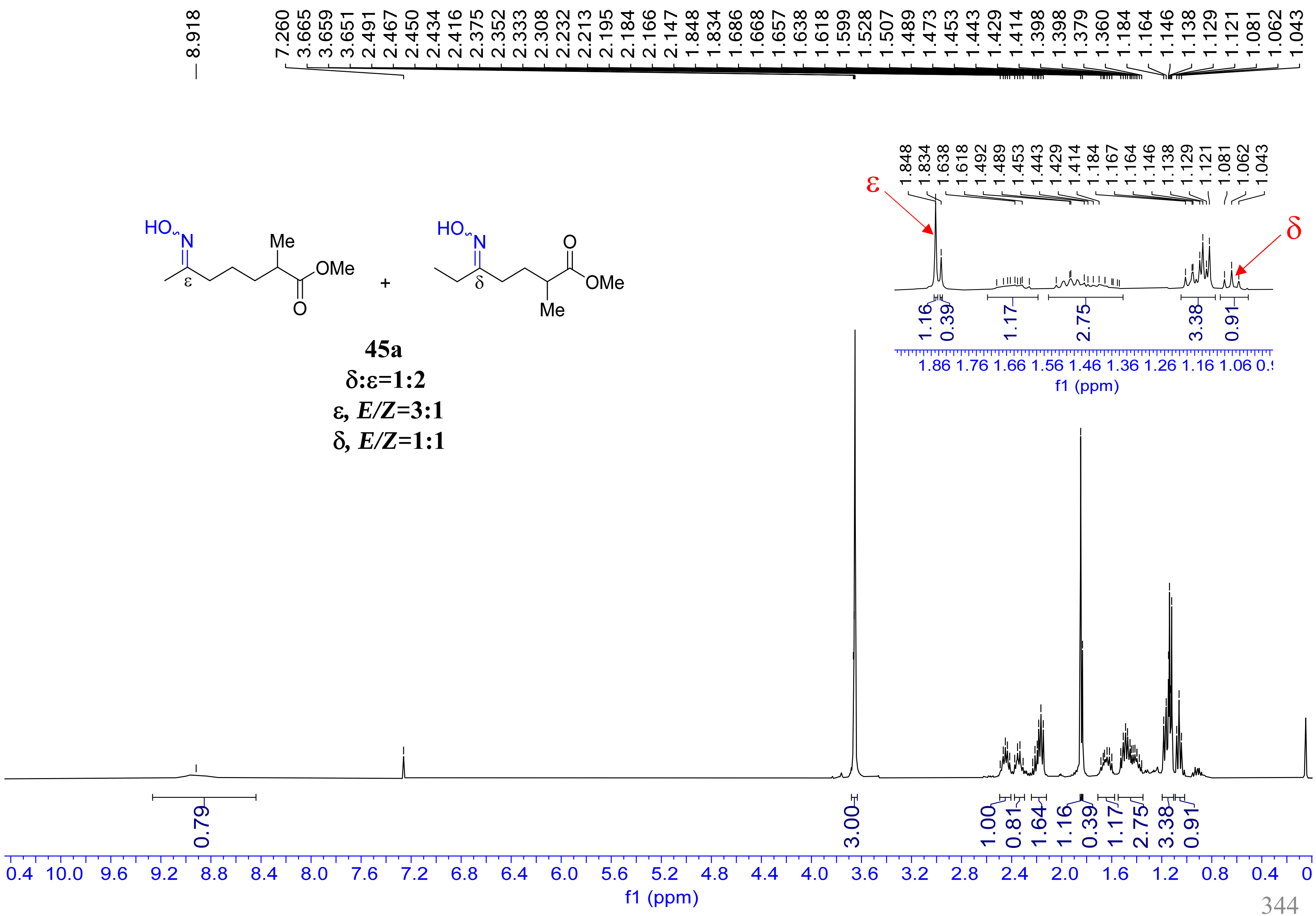

^{13}C NMR 101 MHz, $CDCl_3$

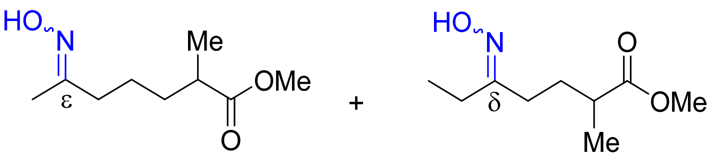

45a

δ:ε=1:2

ε, *E/Z*=3:1

δ, *E/Z*=1:1

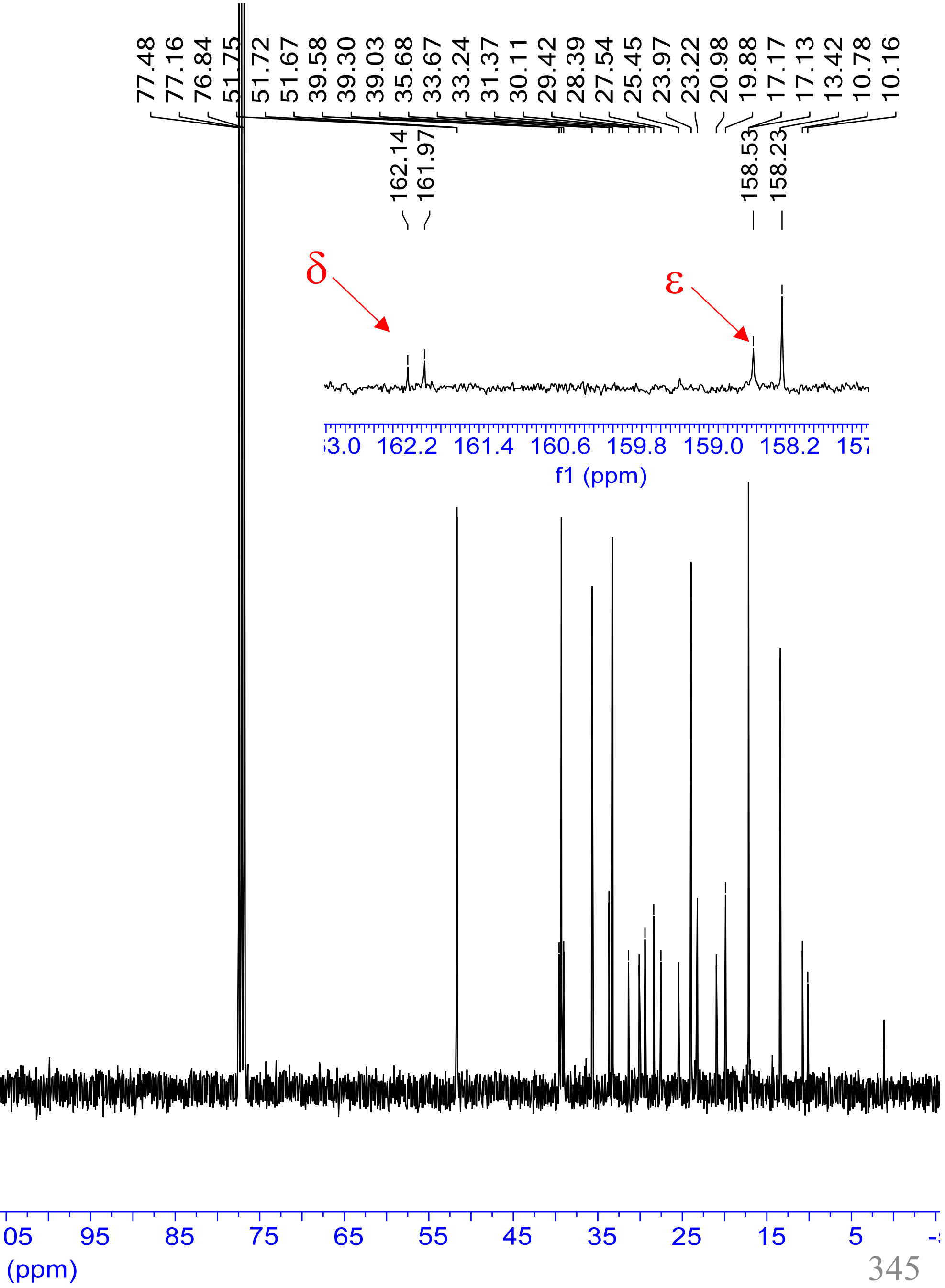

¹H NMR, 400 MHz, CDCl$_3$

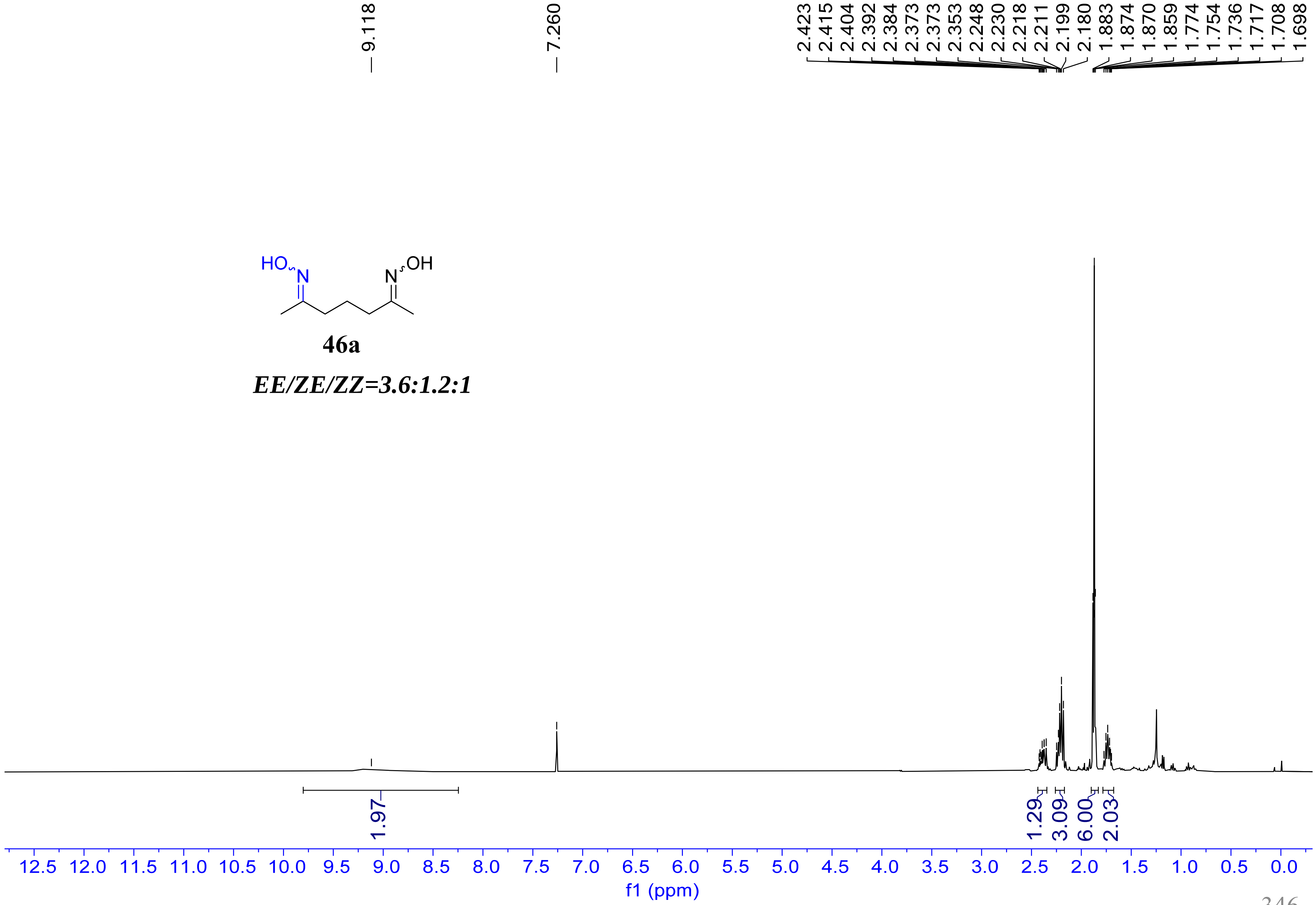

^{13}C NMR 101 MHz, $CDCl_3$

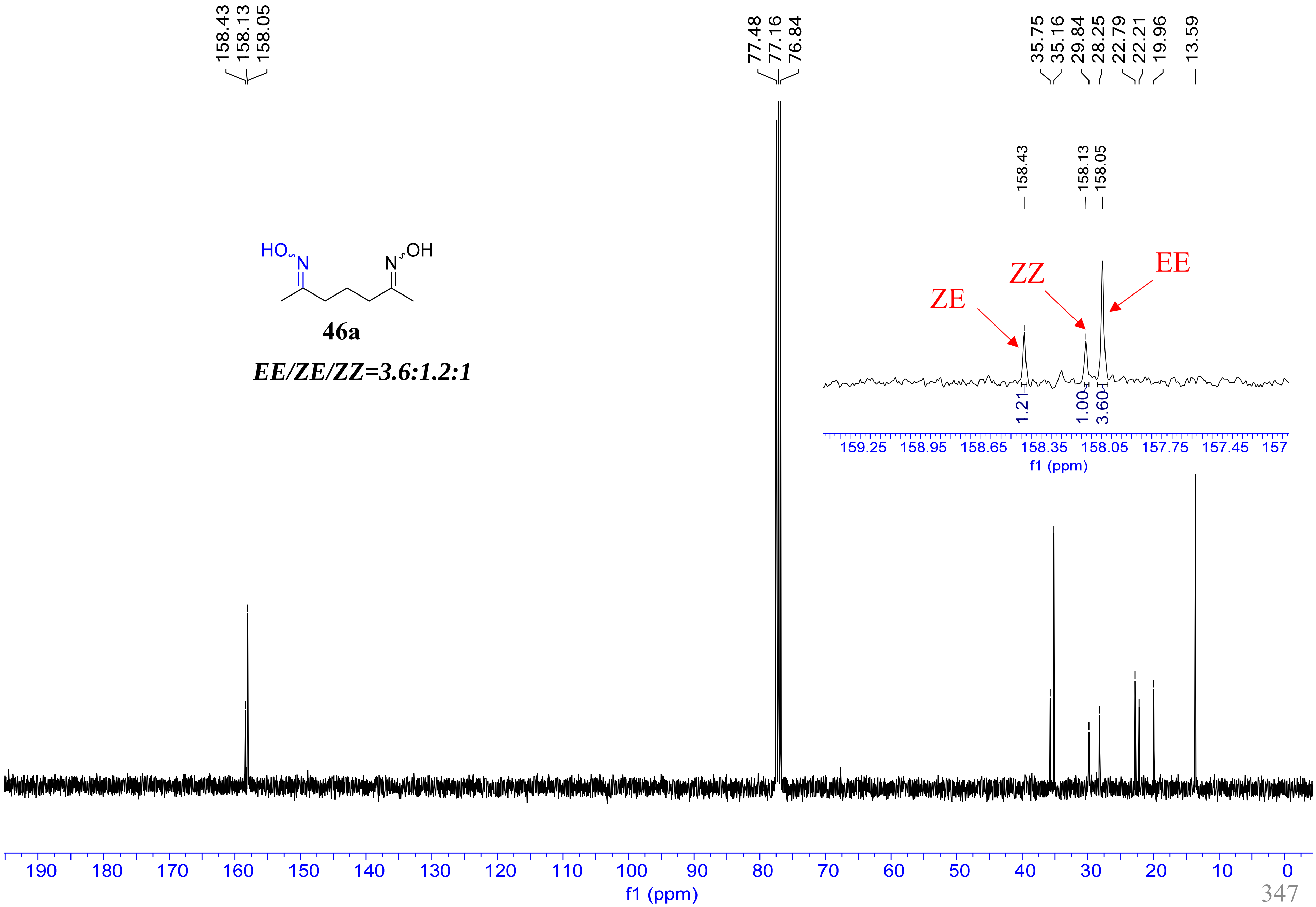

^{1}H NMR, 400 MHz, $CDCl_3$

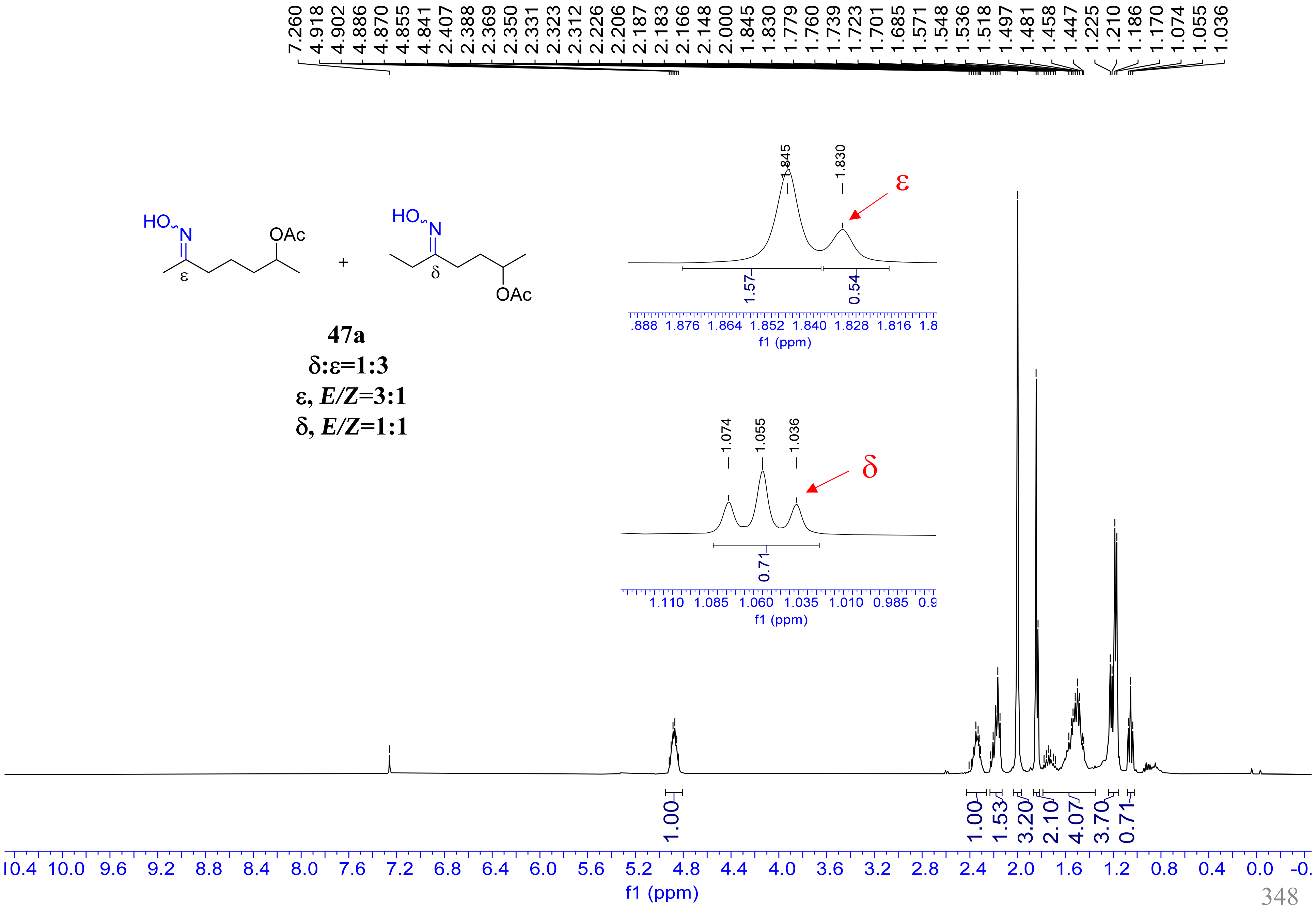

47a

δ:ε=1:3

ε, *E/Z*=3:1

δ, *E/Z*=1:1

^{13}C NMR 101 MHz, CDCl$_3$

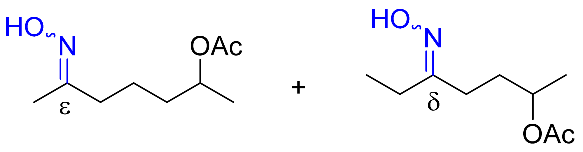

47a

δ:ε=1:3

ε, *E/Z*=3:1

δ, *E/Z*=1:1

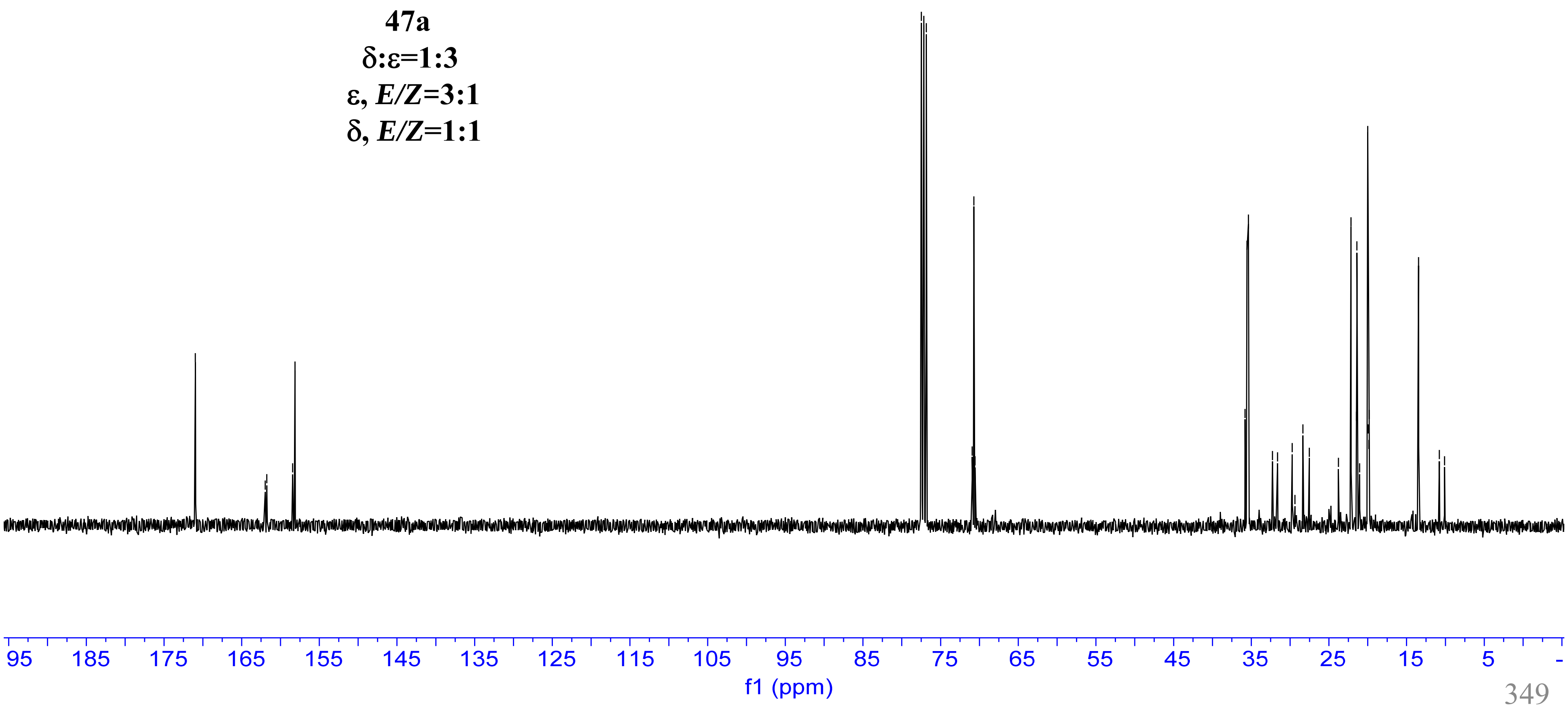

^{1}H NMR, 400 MHz, $CDCl_3$

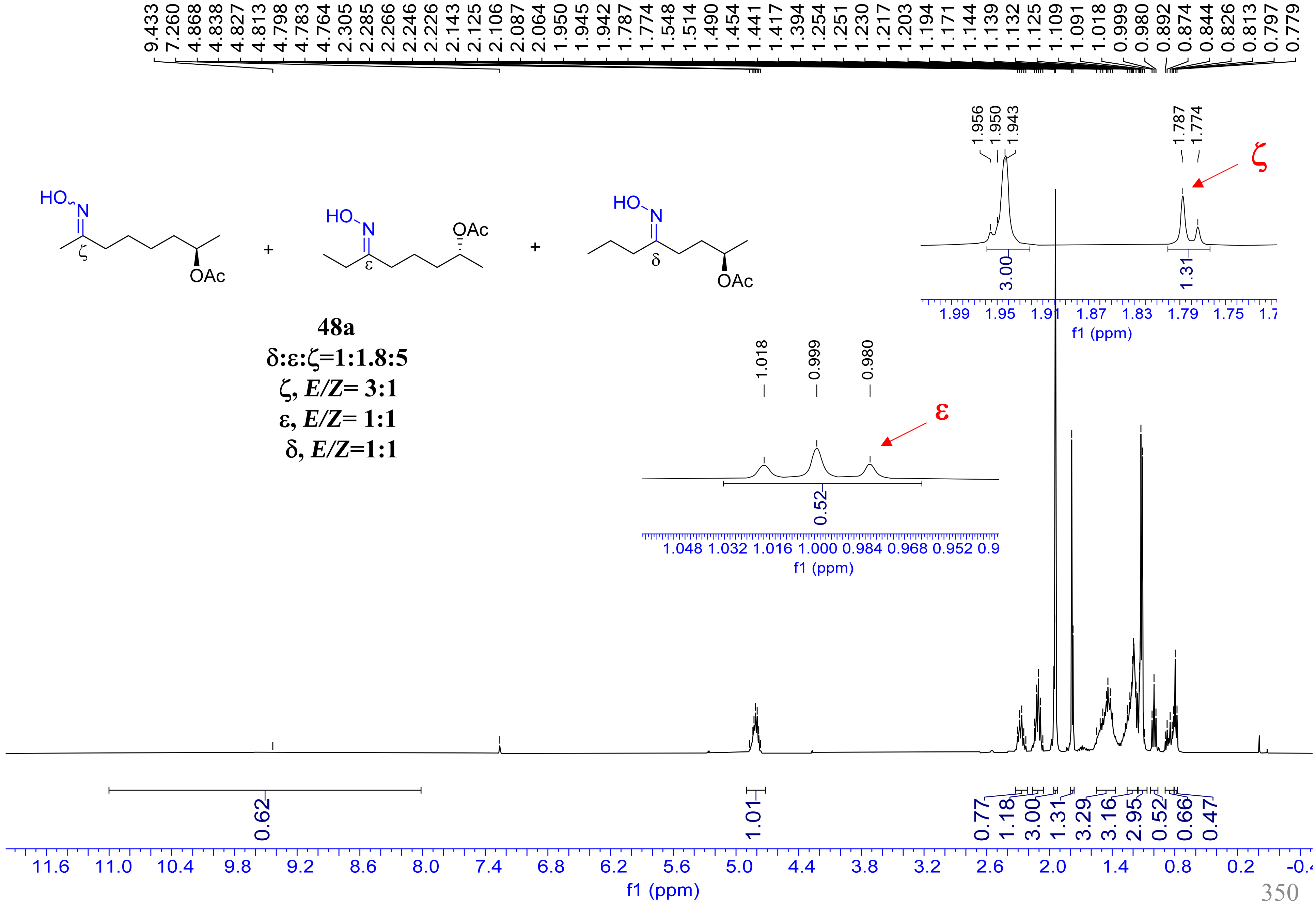

48a

δ:ε:ζ=1:1.8:5

ζ, *E/Z*= 3:1

ε, *E/Z*= 1:1

δ, *E/Z*=1:1

^{13}C NMR 101 MHz, $CDCl_3$

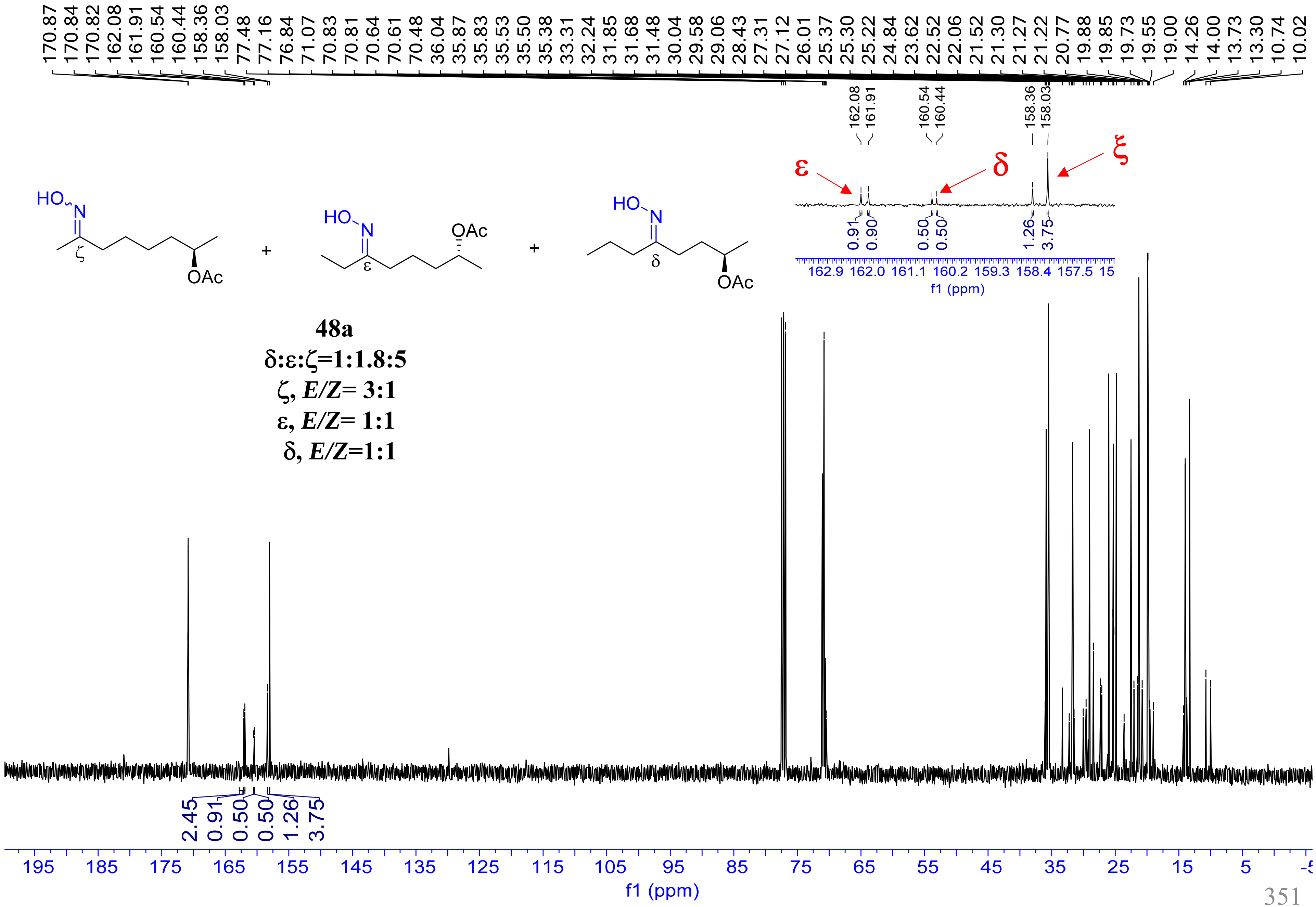

48a

δ:ε:ζ=1:1.8:5

ζ, *E/Z*= 3:1

ε, *E/Z*= 1:1

δ, *E/Z*=1:1

^1H NMR, 400 MHz, $CDCl_3$

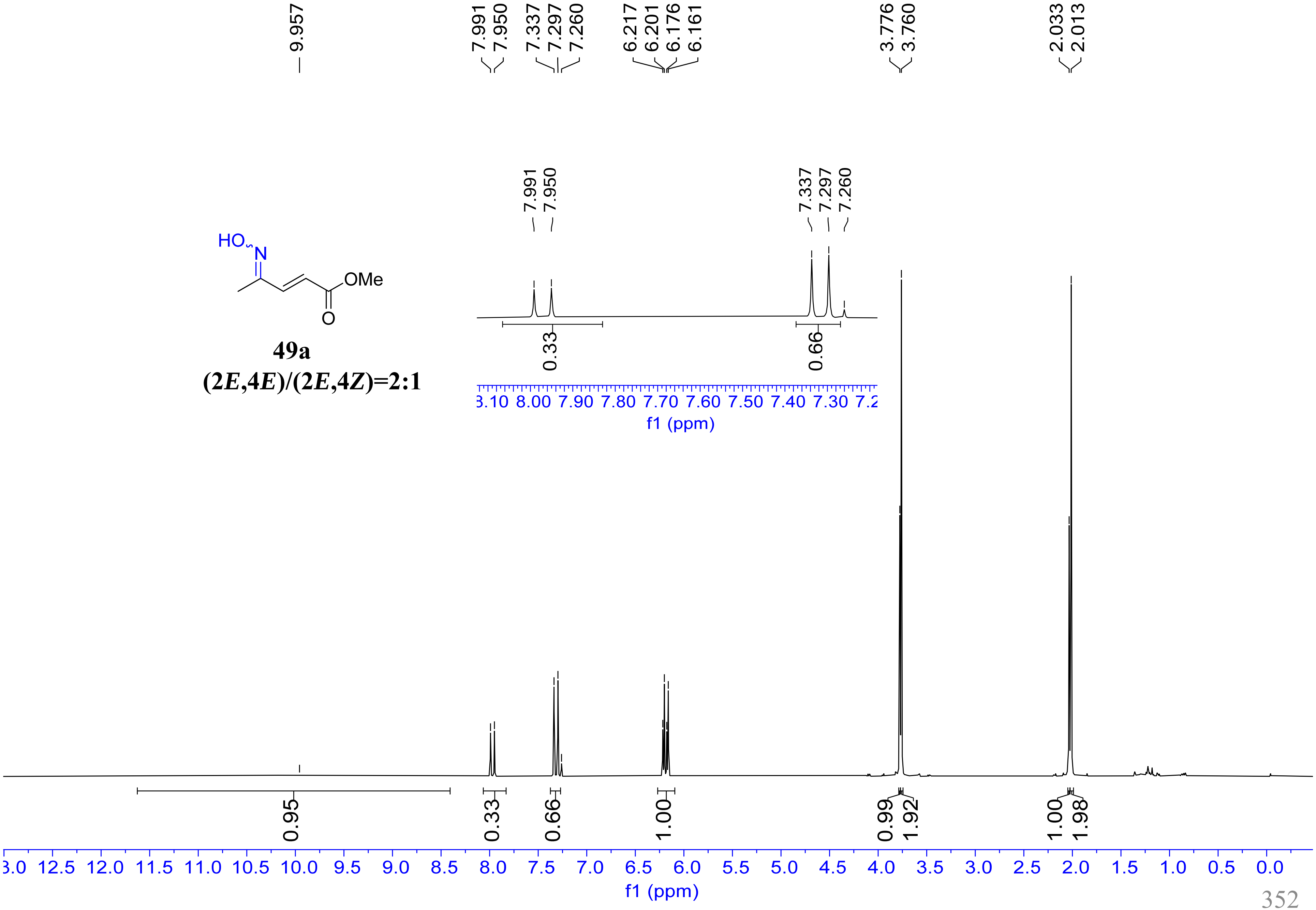

49a

(2*E*,4*E*)/(2*E*,4*Z*)=2:1

^{13}C NMR 101 MHz, $CDCl_3$

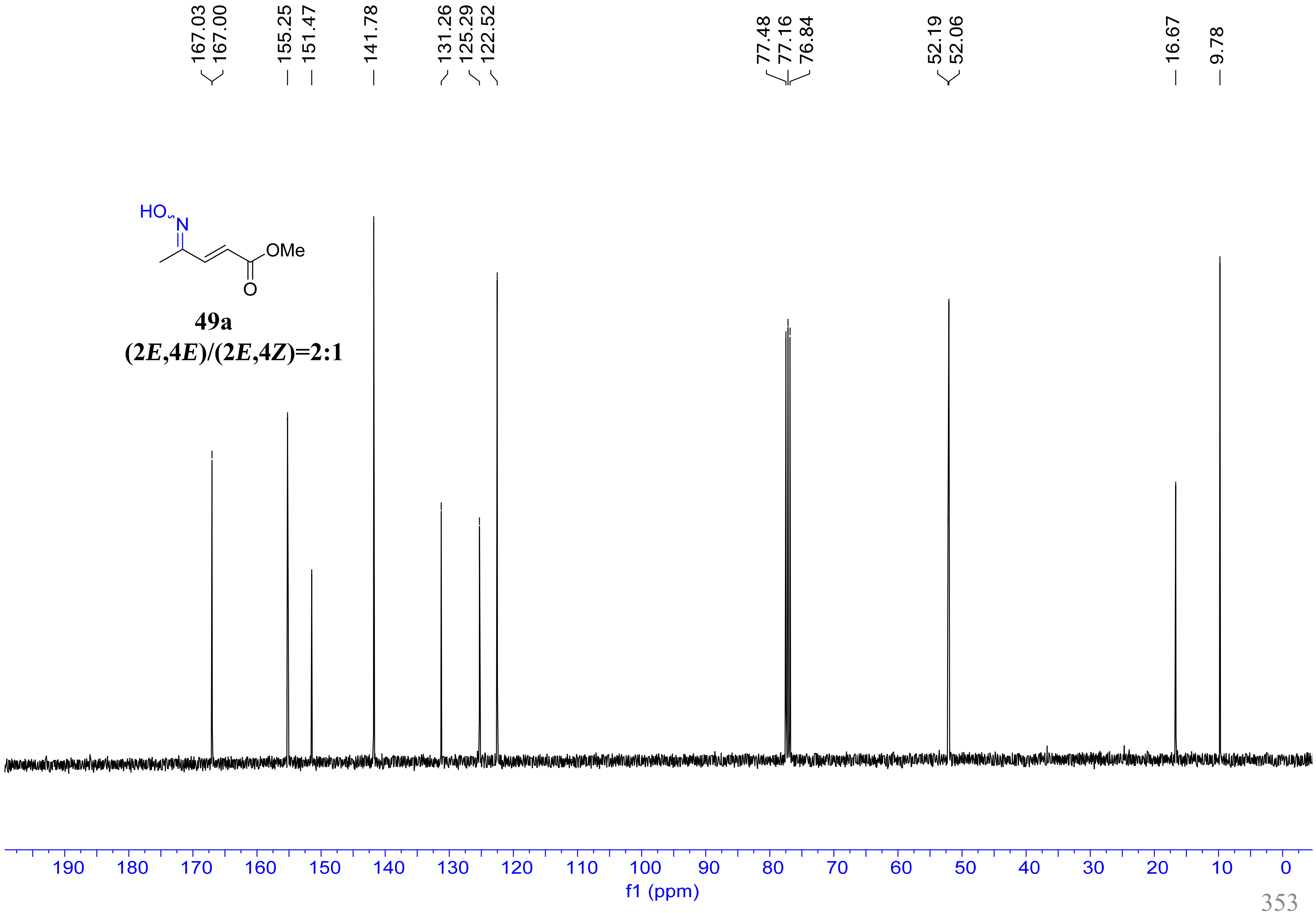

^{1}H NMR, 400 MHz, $CDCl_3$

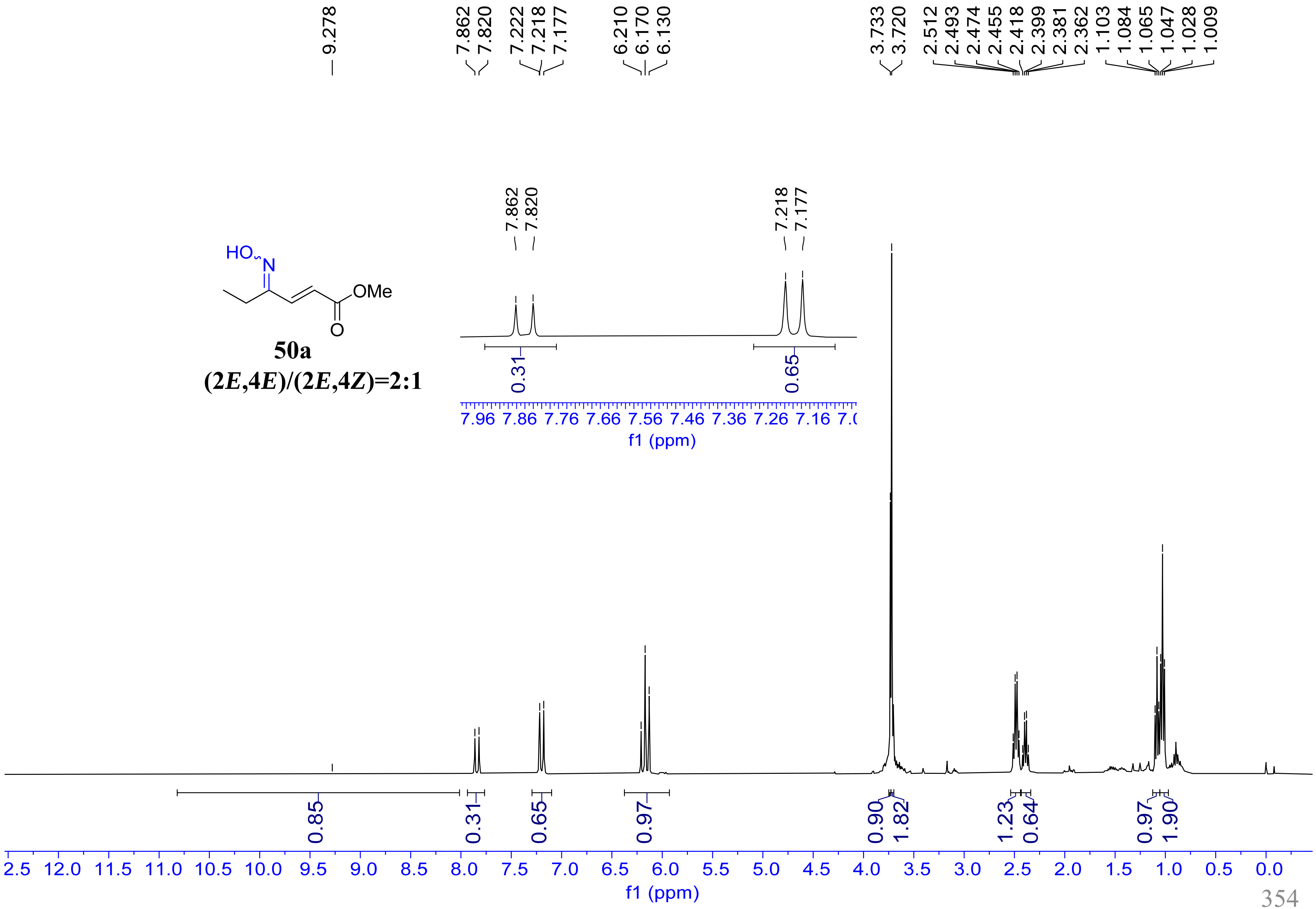

50a

(2*E*,4*E*)/(2*E*,4*Z*)=2:1

^{13}C NMR 101 MHz, $CDCl_3$

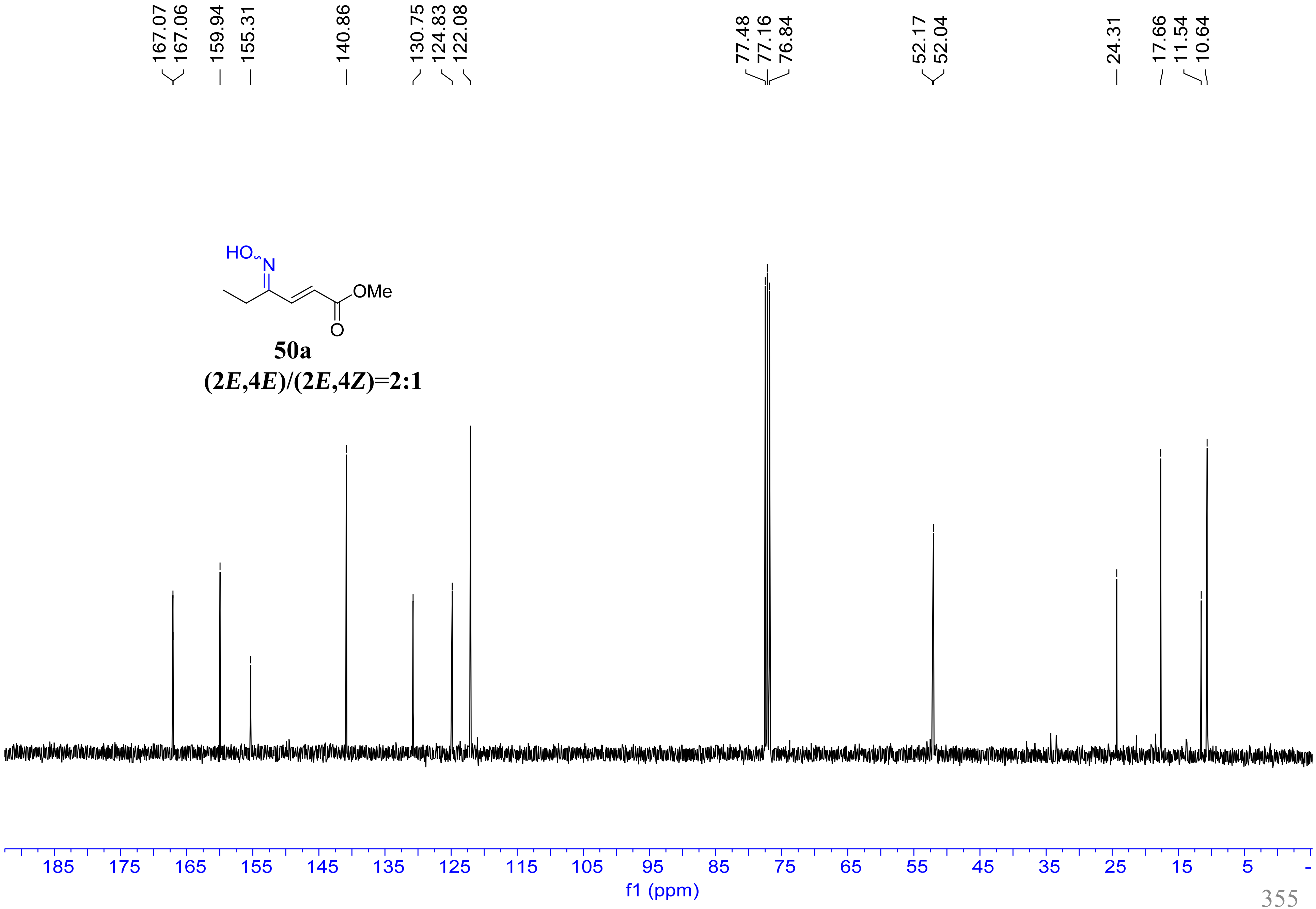

50a
(2*E*,4*E*)/(2*E*,4*Z*)=2:1

^{1}H NMR, 400 MHz, $CDCl_3$

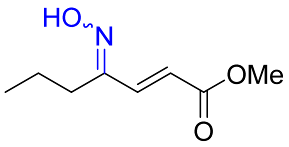

51a

(2*E*,4*E*)/(2*E*,4*Z*)=2:1

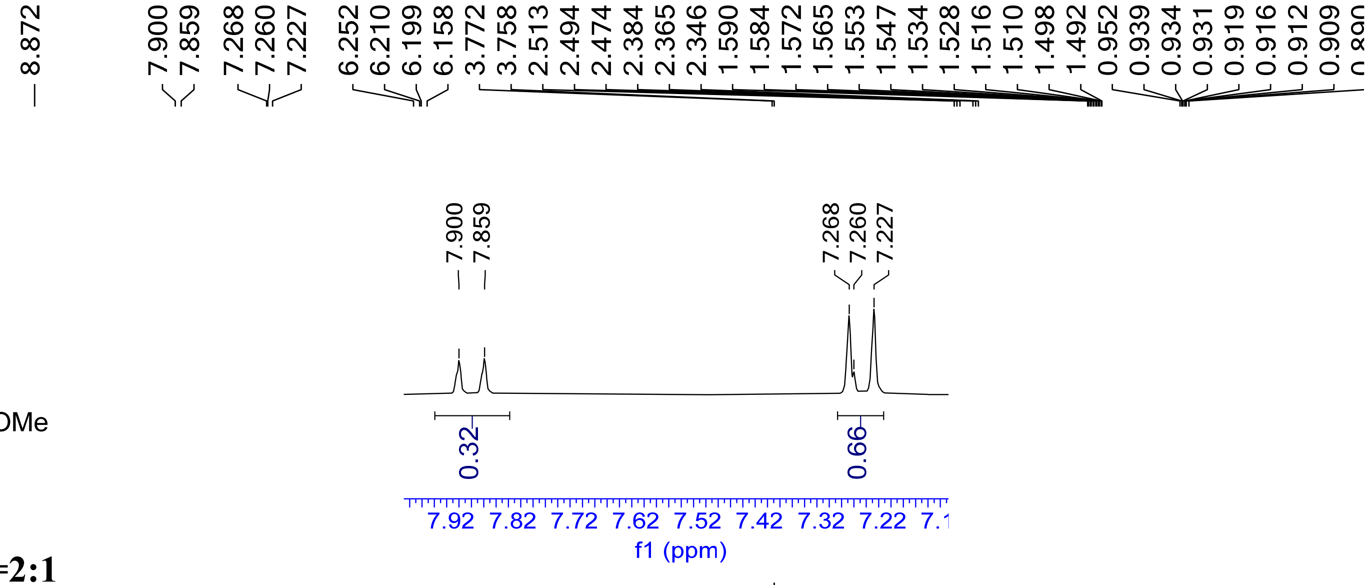

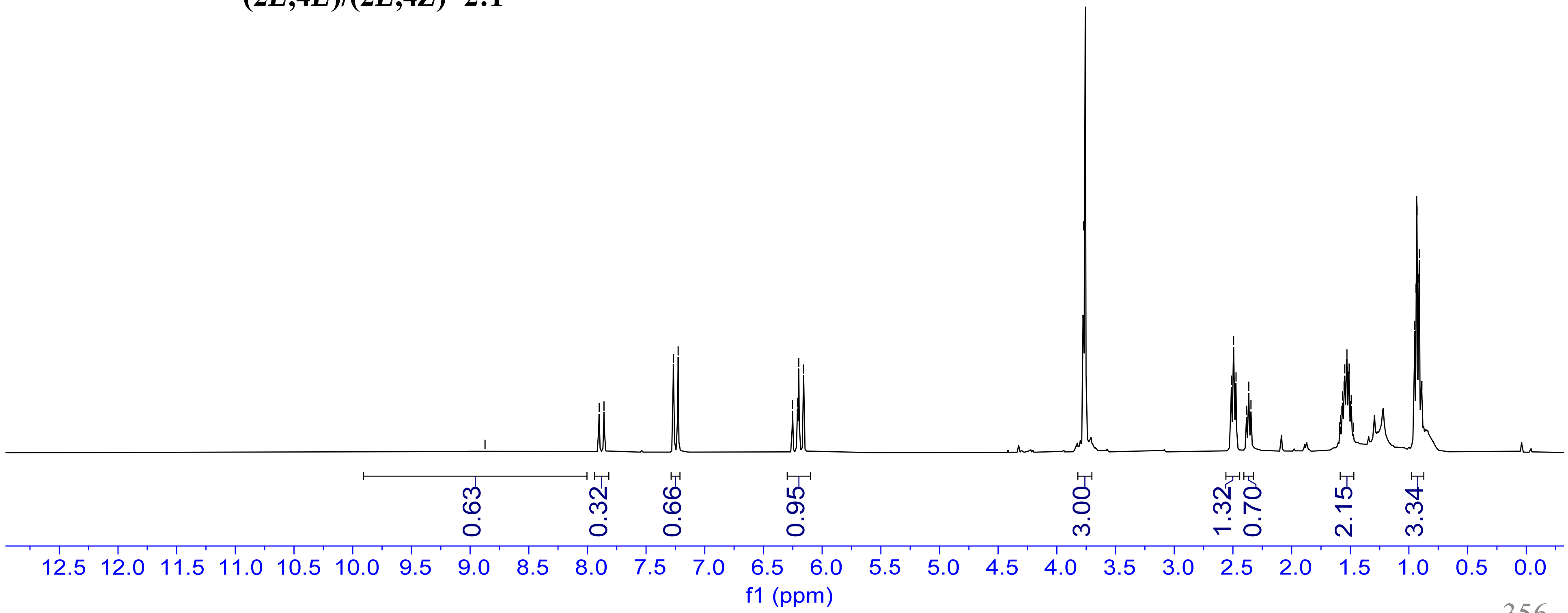

^{13}C NMR 101 MHz, $CDCl_3$

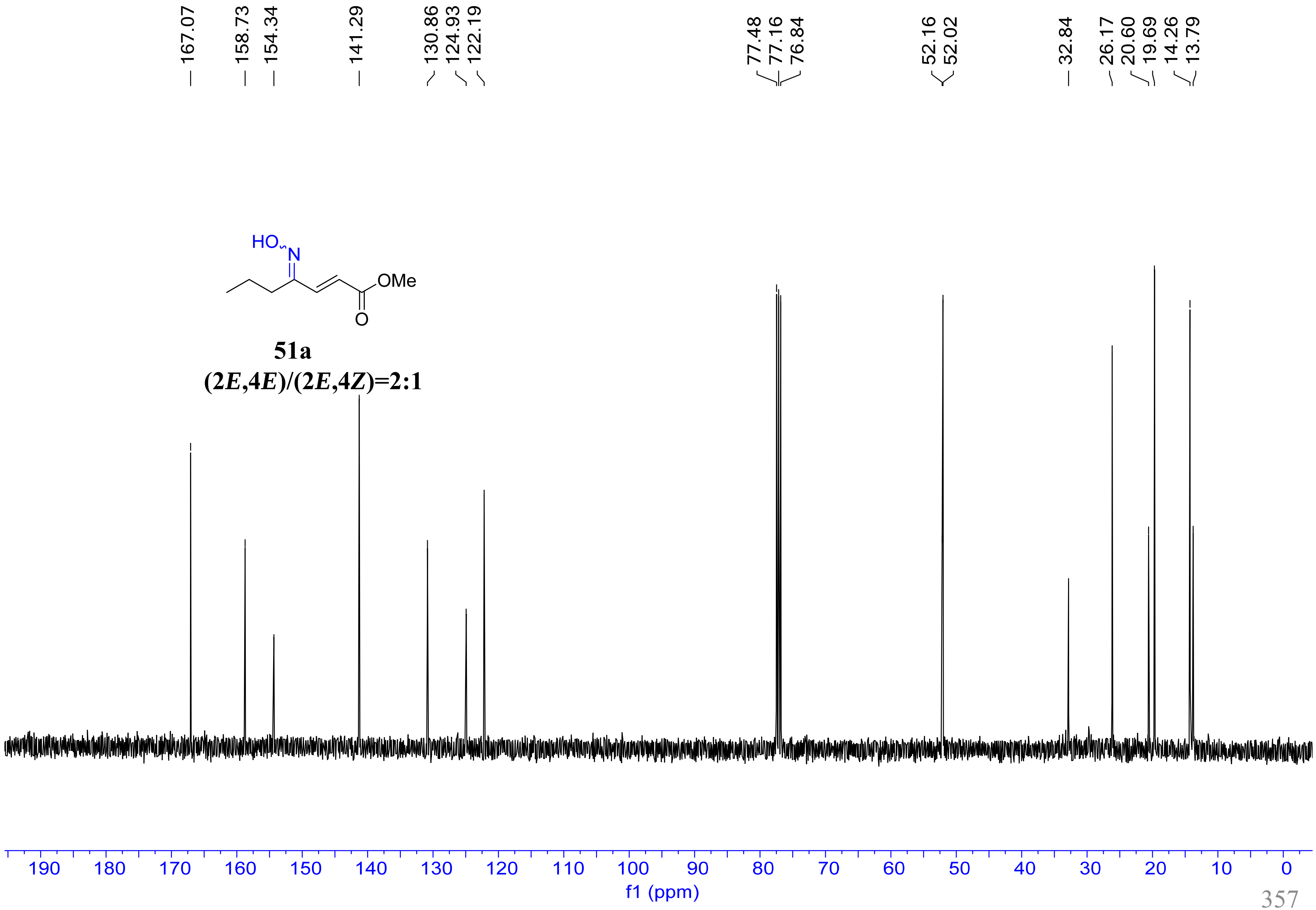

51a

(2*E*,4*E*)/(2*E*,4*Z*)=2:1

^1H NMR, 400 MHz, $CDCl_3$

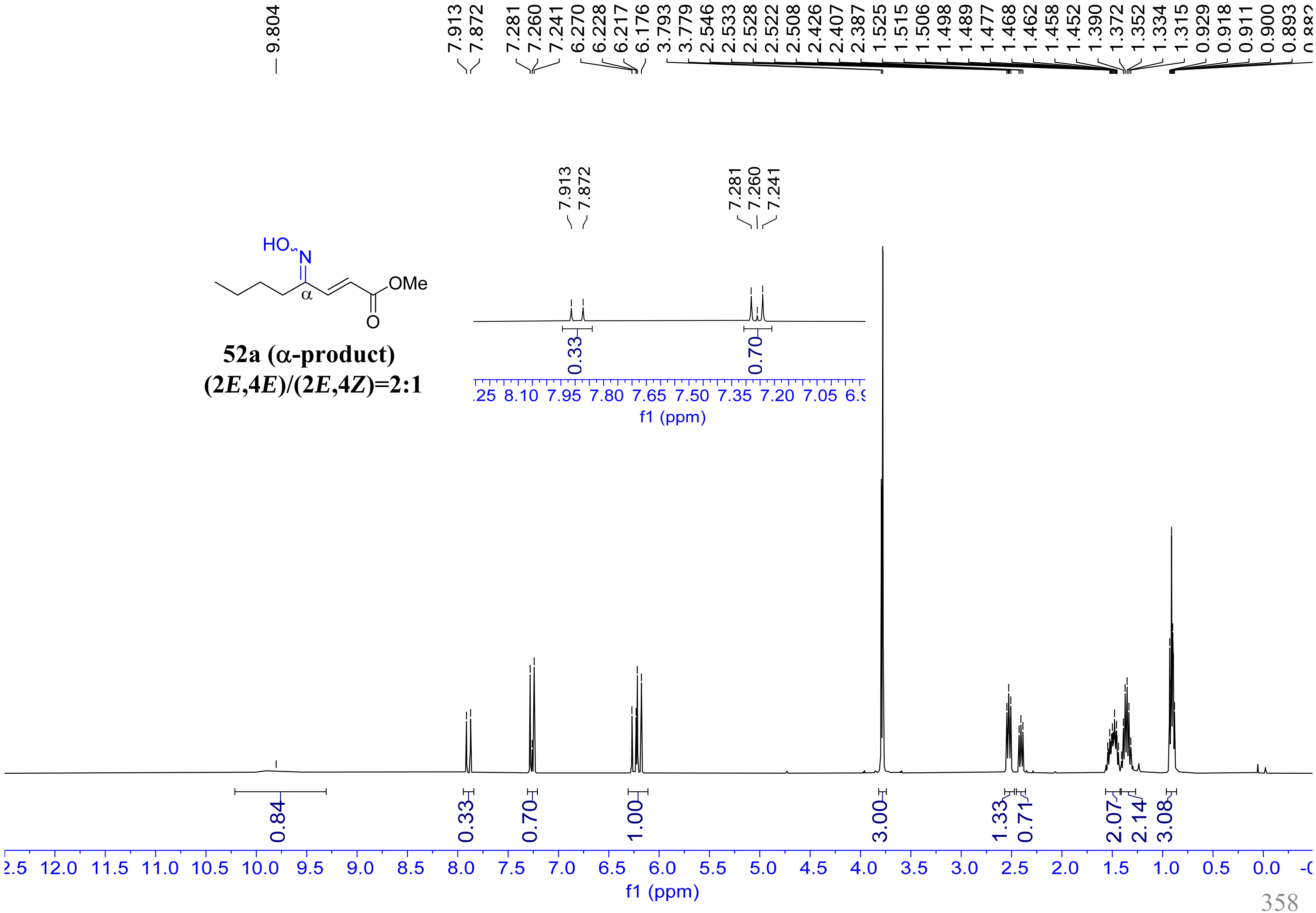

52a (α-product)
(2*E*,4*E*)/(2*E*,4*Z*)=2:1

^{13}C NMR 101 MHz, $CDCl_3$

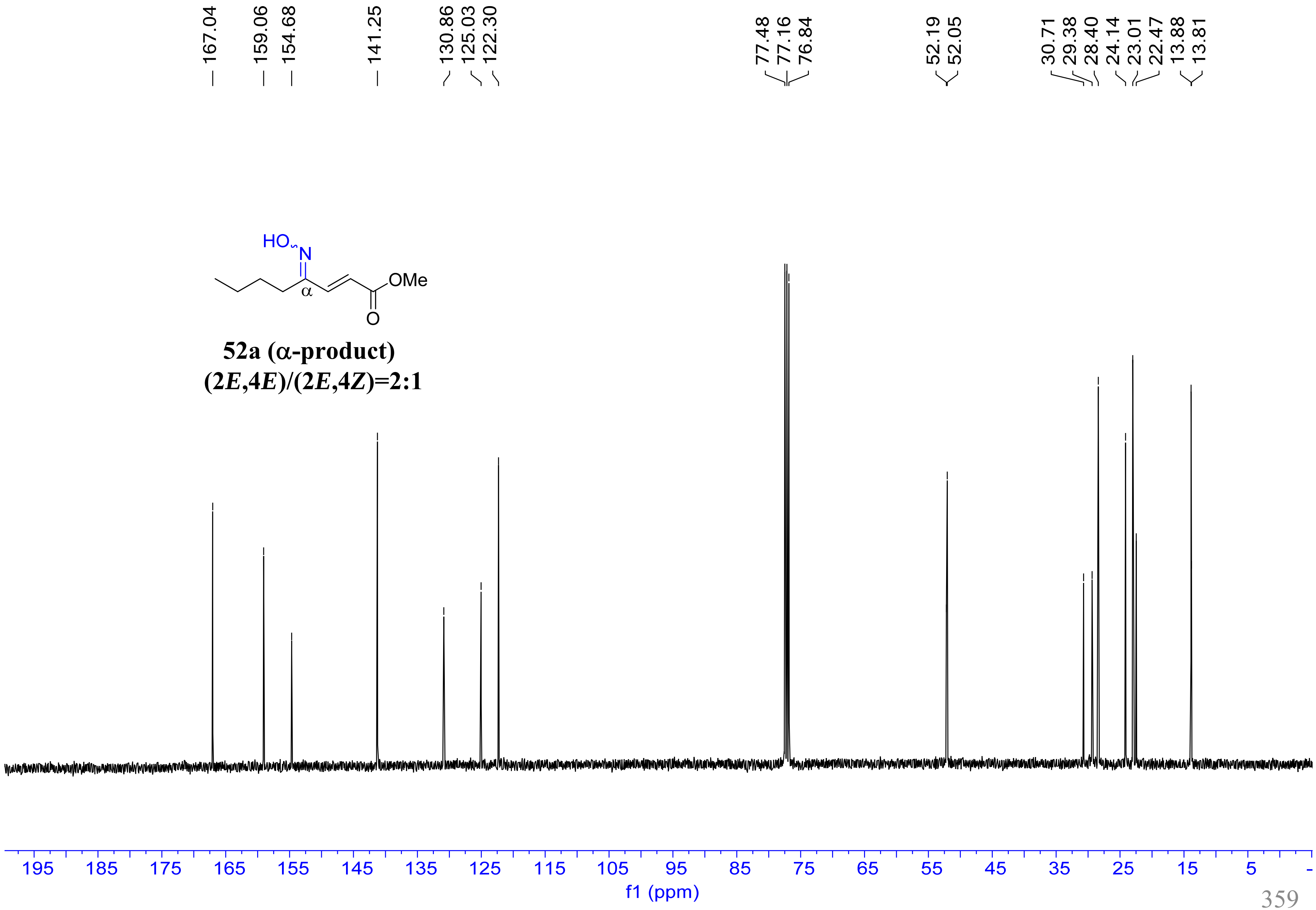

52a (α-product)
(2*E*,4*E*)/(2*E*,4*Z*)=2:1

^{1}H NMR, 400 MHz, CDCl$_3$

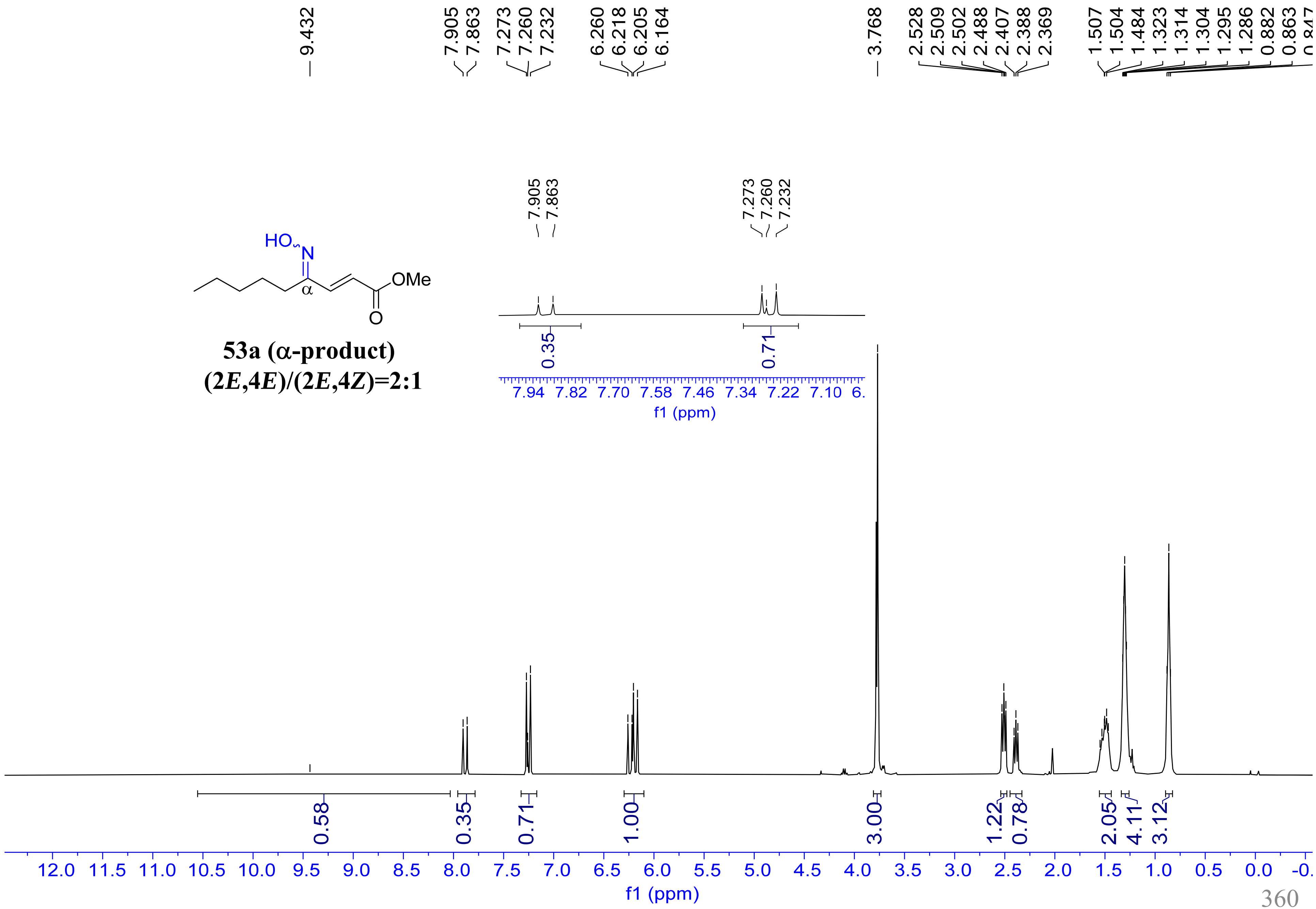

^{13}C NMR 101 MHz, $CDCl_3$

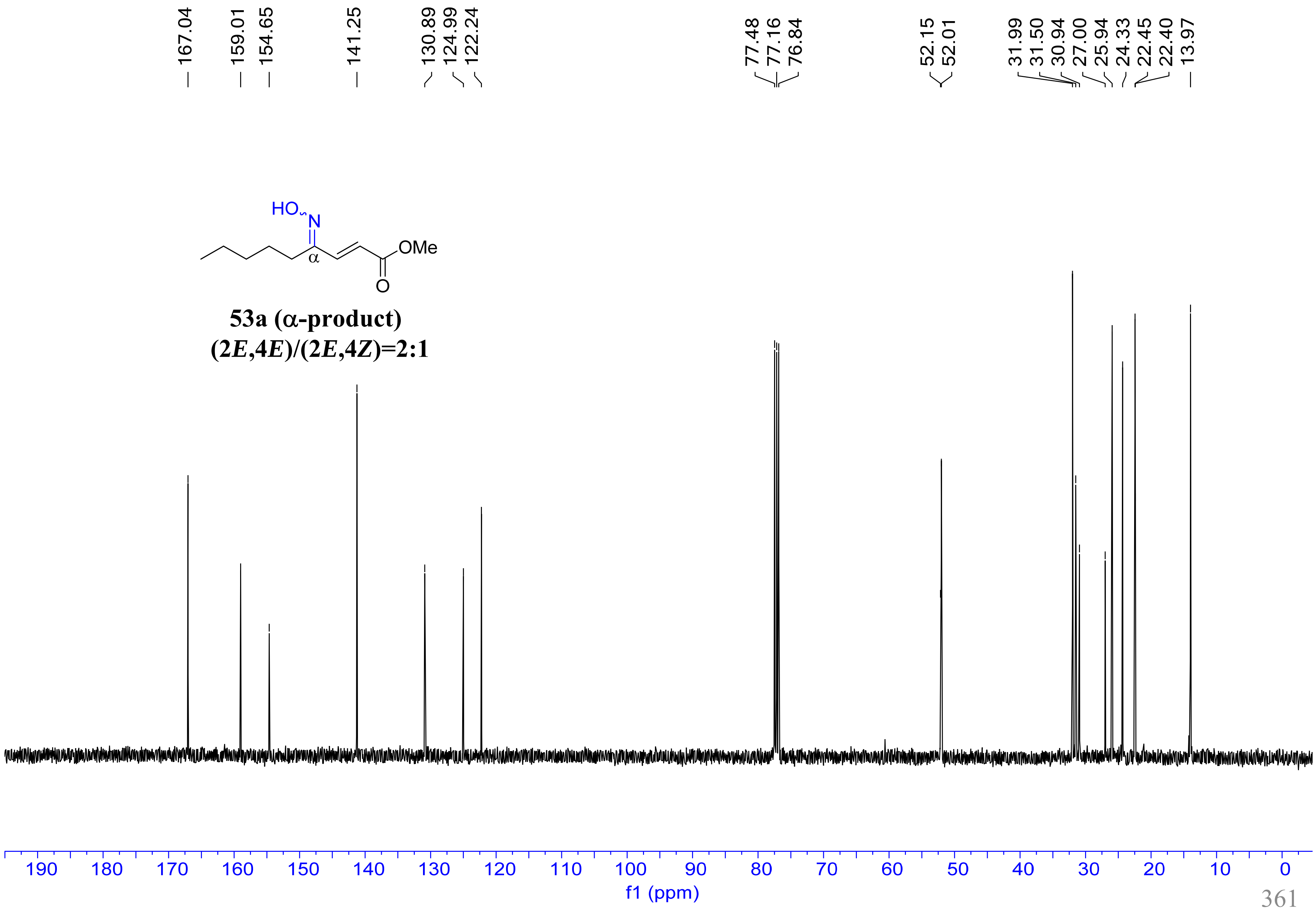

53a (α-product)

(2*E*,4*E*)/(2*E*,4*Z*)=2:1

^1H NMR, 400 MHz, $CDCl_3$

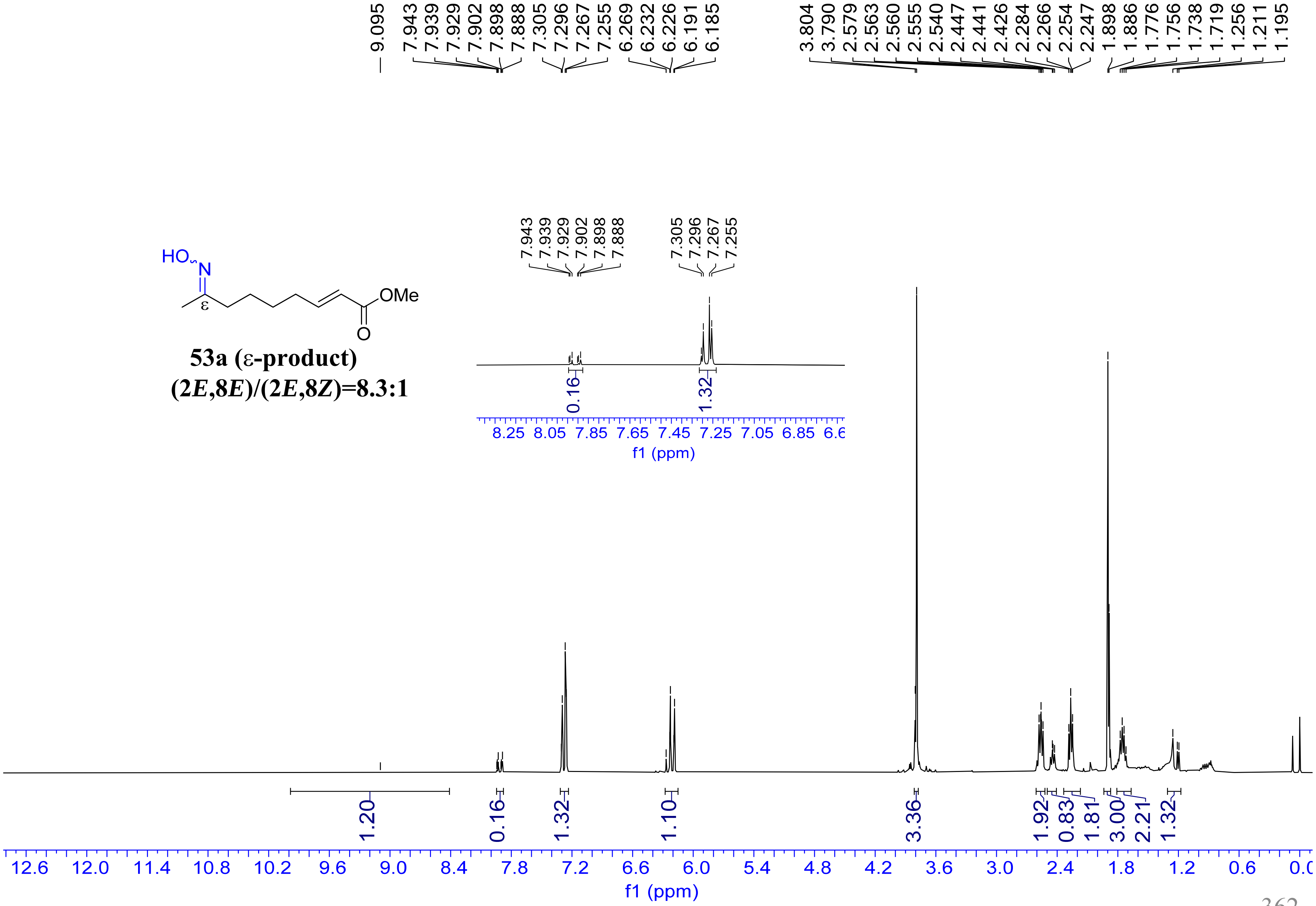

^{13}C NMR 101 MHz, $CDCl_3$

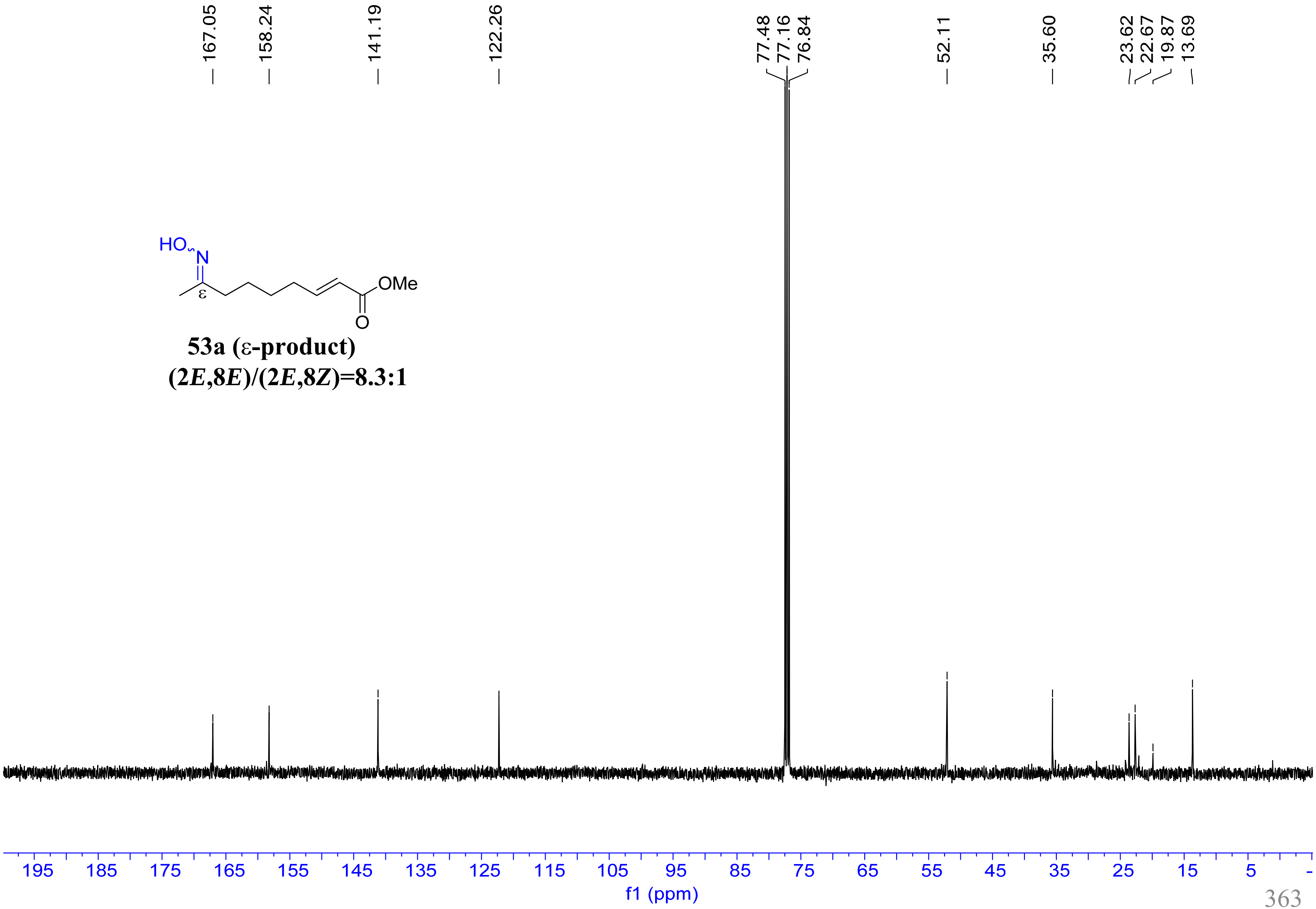

53a (ε-product)
(2*E*,8*E*)/(2*E*,8*Z*)=8.3:1

^{1}H NMR, 400 MHz, $CDCl_3$

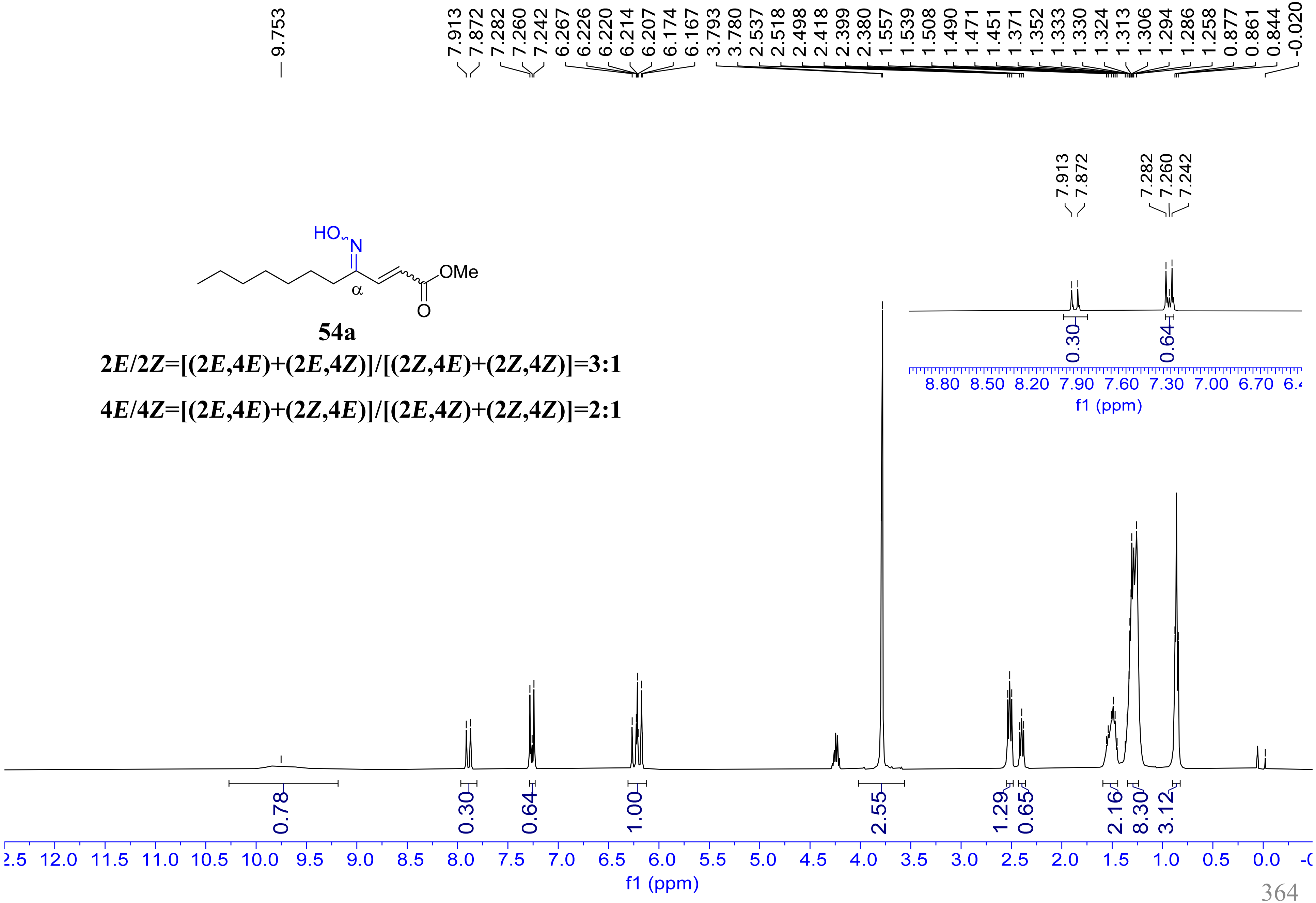

54a

2*E*/2*Z*=[(2*E*,4*E*)+(2*E*,4*Z*)]/[(2*Z*,4*E*)+(2*Z*,4*Z*)]=3:1

4*E*/4*Z*=[(2*E*,4*E*)+(2*Z*,4*E*)]/[(2*E*,4*Z*)+(2*Z*,4*Z*)]=2:1

^{13}C NMR 101 MHz, $CDCl_3$

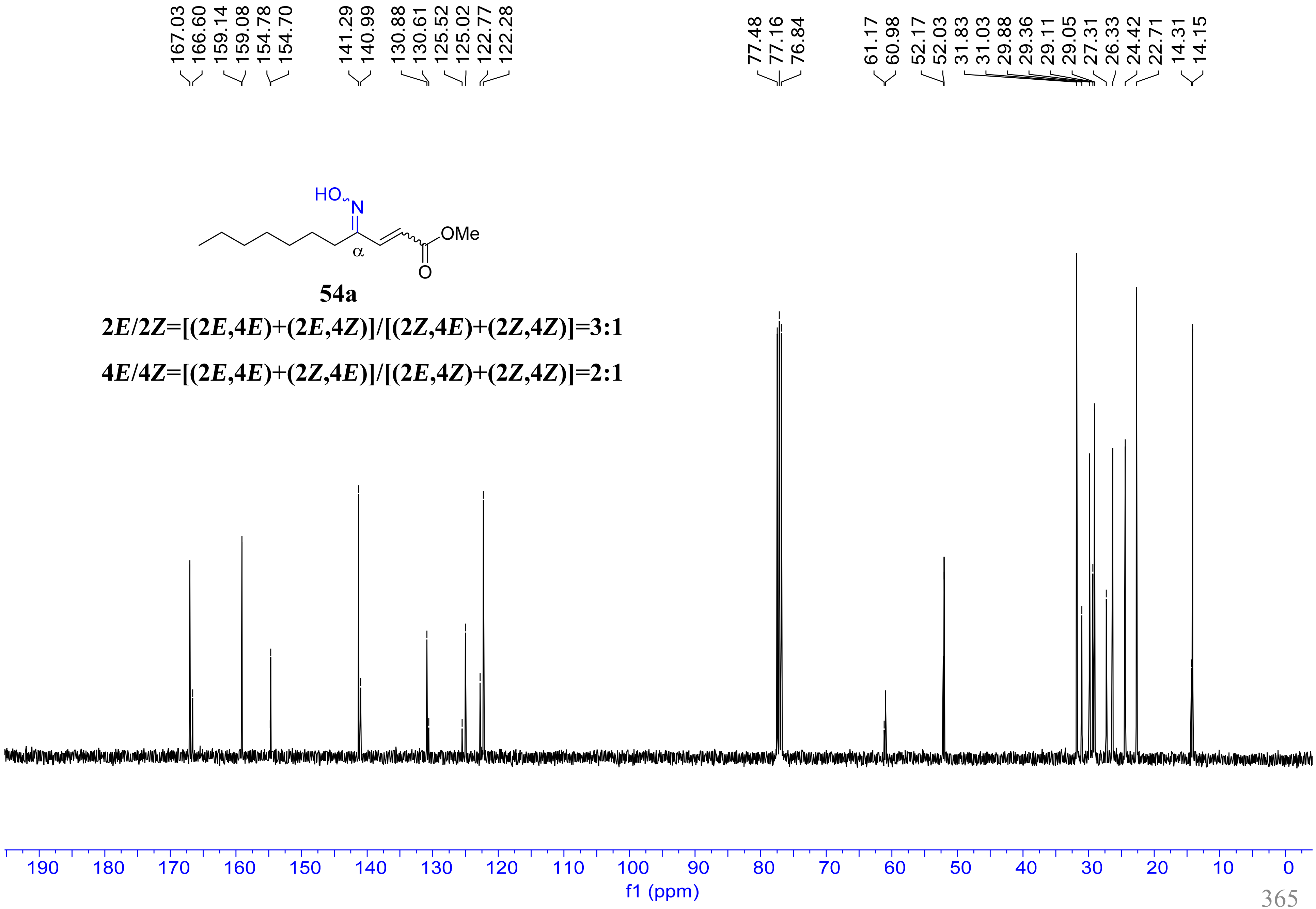

54a

2*E*/2*Z*=[(2*E*,4*E*)+(2*E*,4*Z*)]/[(2*Z*,4*E*)+(2*Z*,4*Z*)]=3:1

4*E*/4*Z*=[(2*E*,4*E*)+(2*Z*,4*E*)]/[(2*E*,4*Z*)+(2*Z*,4*Z*)]=2:1

^{1}H NMR, 400 MHz, $CDCl_3$

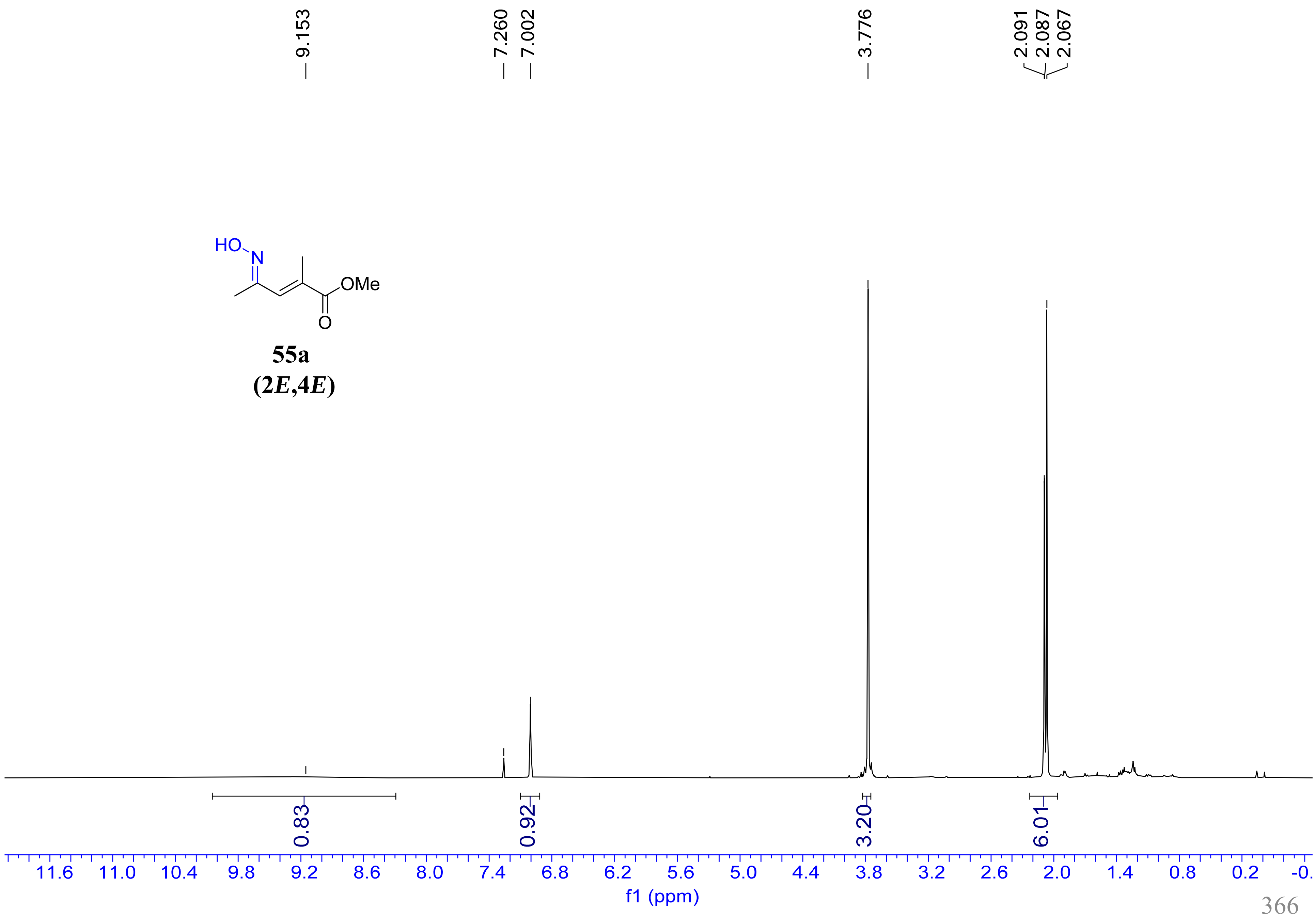

55a
(2*E*,4*E*)

^{13}C NMR 101 MHz, CDCl$_3$

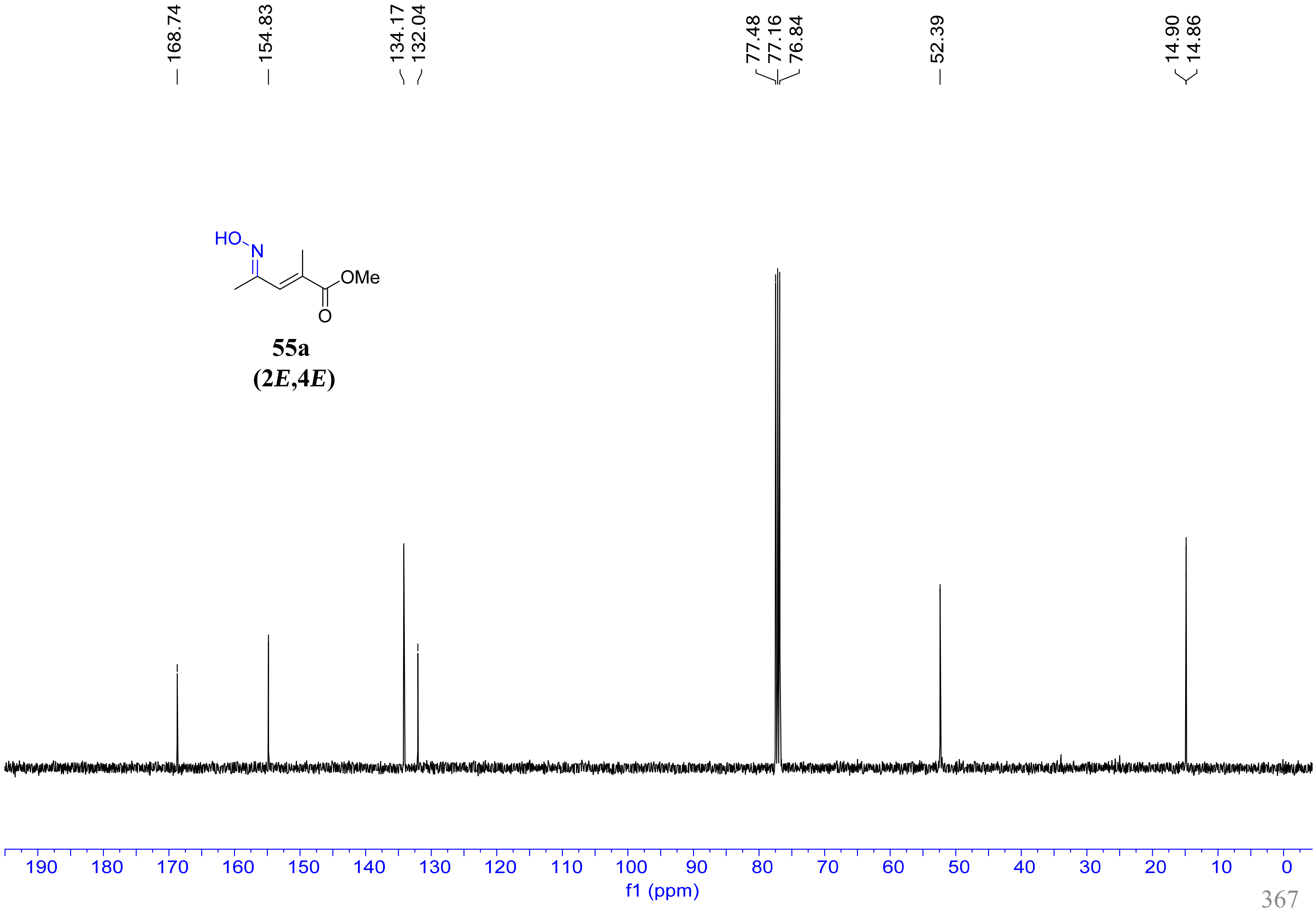

55a
(2*E*,4*E*)

^{1}H NMR, 400 MHz, $CDCl_3$

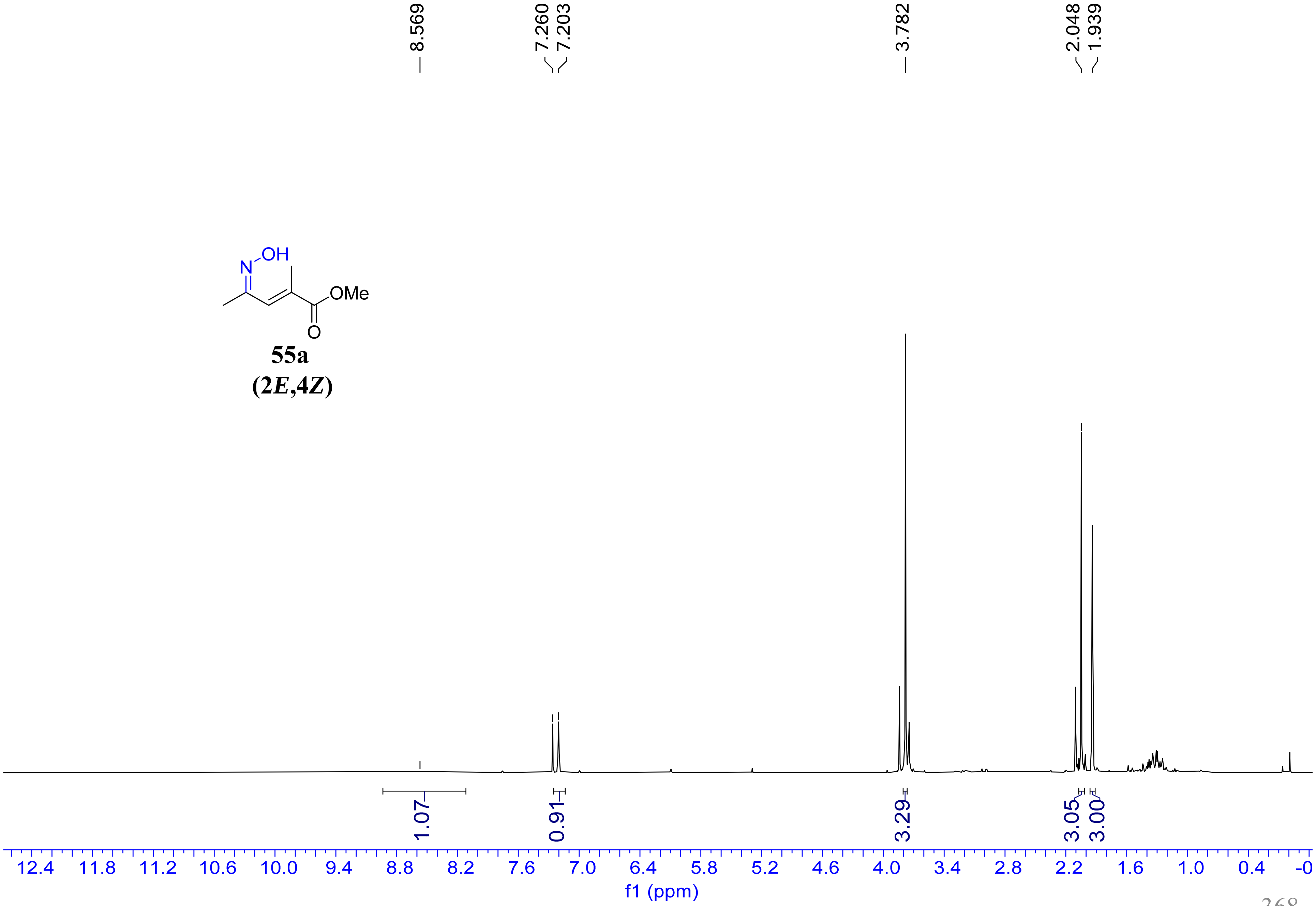

^{13}C NMR 101 MHz, $CDCl_3$

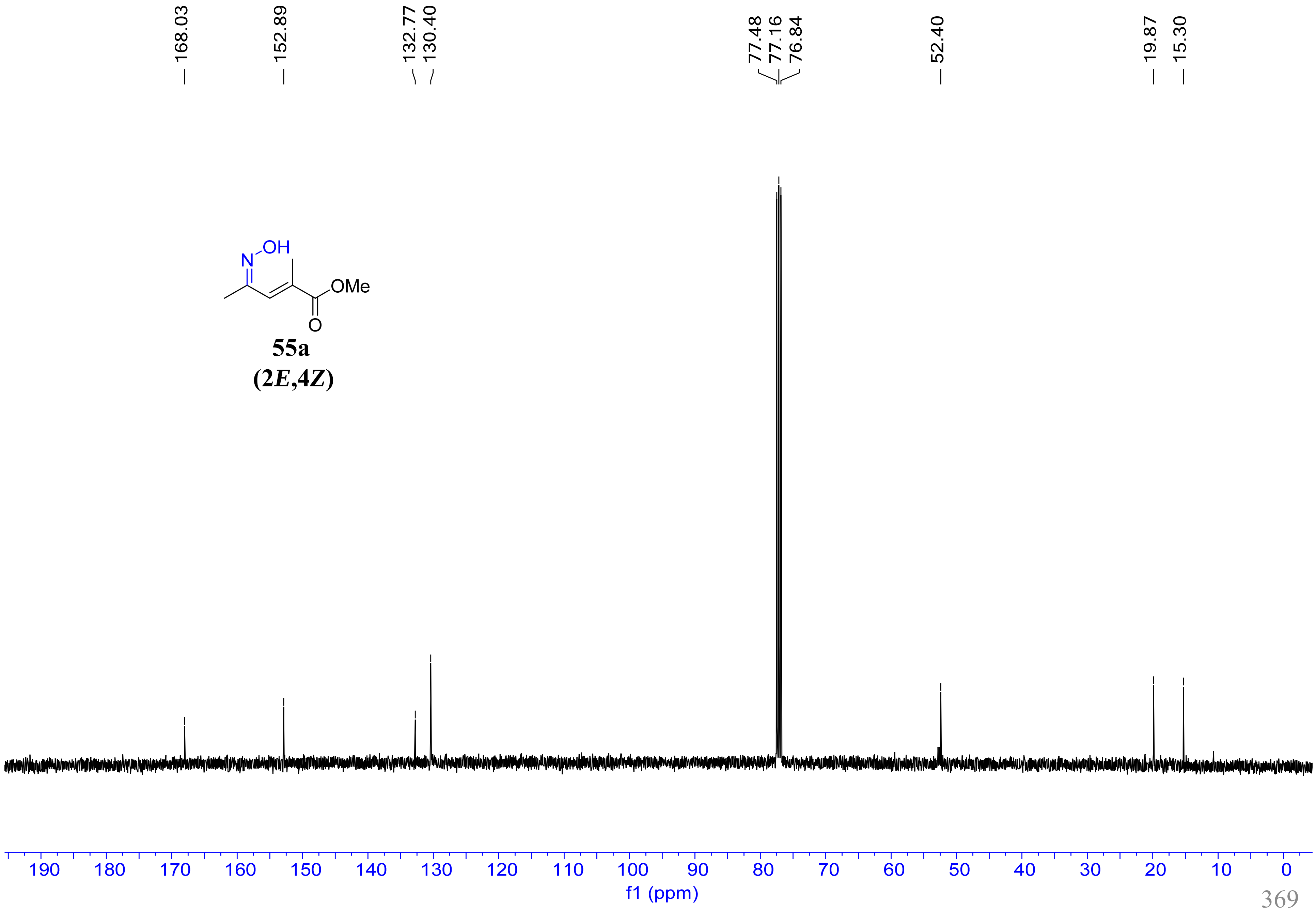

^{1}H NMR, 400 MHz, $CDCl_3$

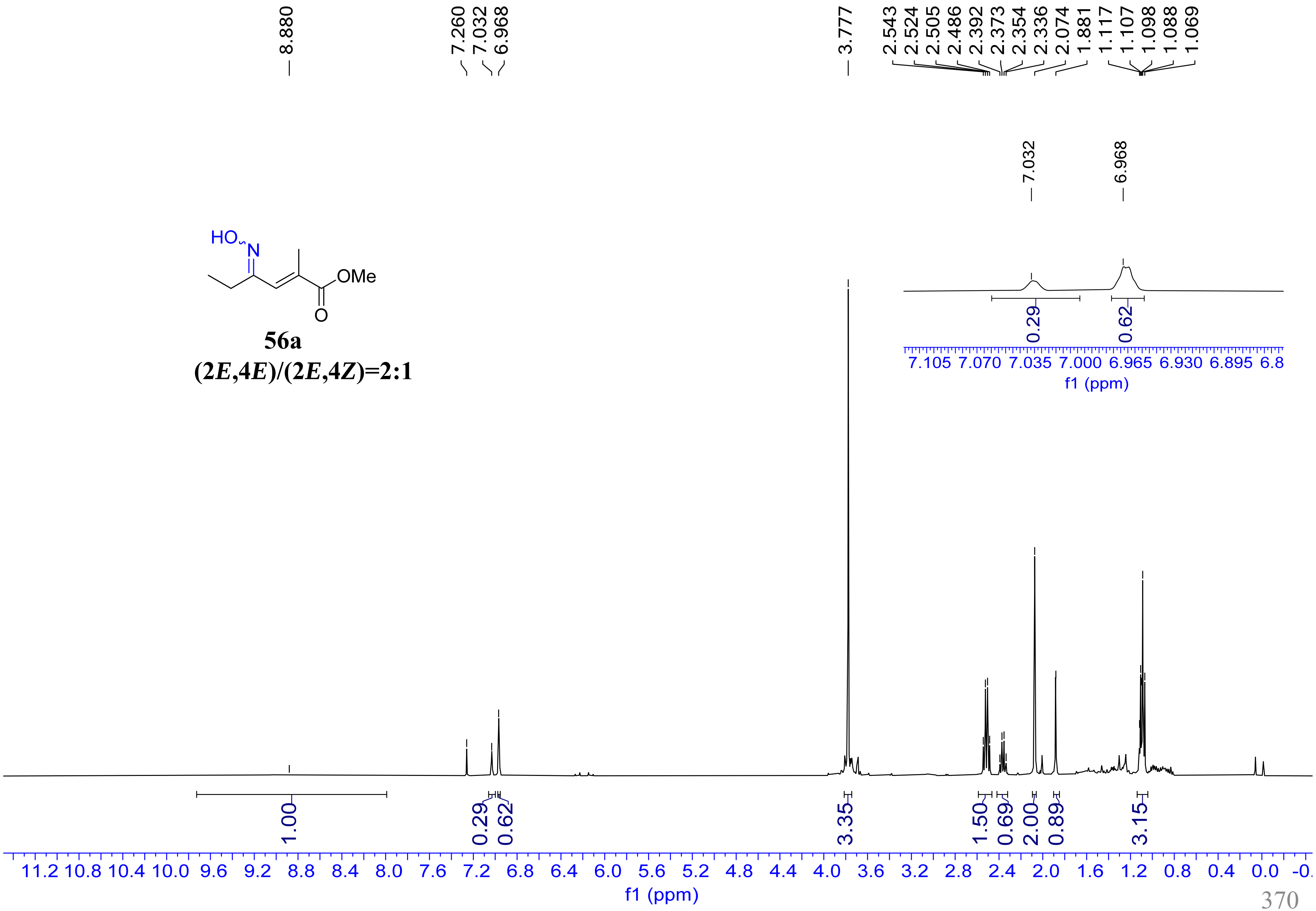

56a

(2*E*,4*E*)/(2*E*,4*Z*)=2:1

^{13}C NMR 101 MHz, $CDCl_3$

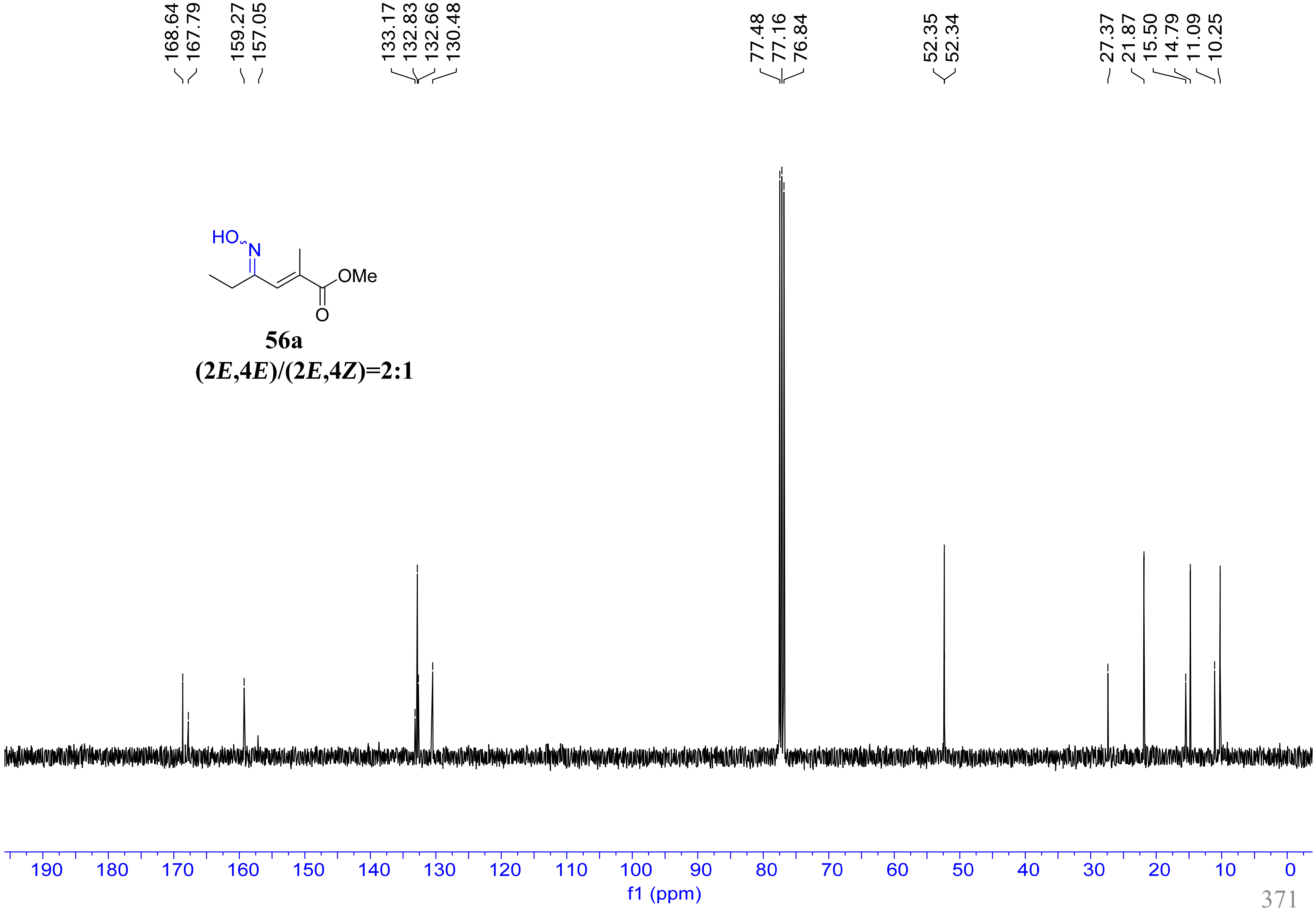

56a

(2*E*,4*E*)/(2*E*,4*Z*)=2:1

^{1}H NMR, 400 MHz, CDCl$_3$

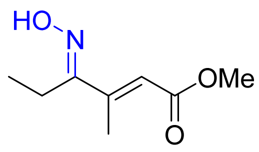

57a
(2*E*,4*E*)

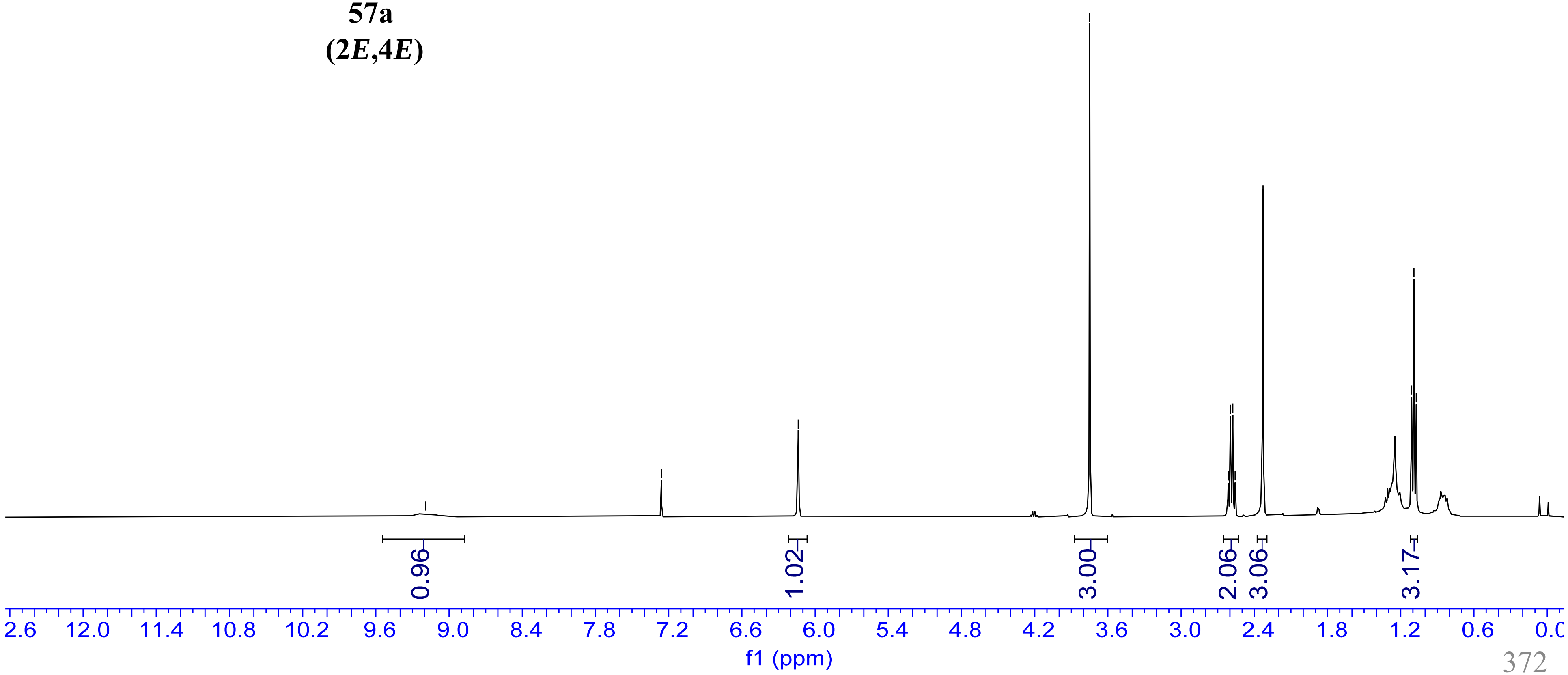

^{13}C NMR 101 MHz, $CDCl_3$

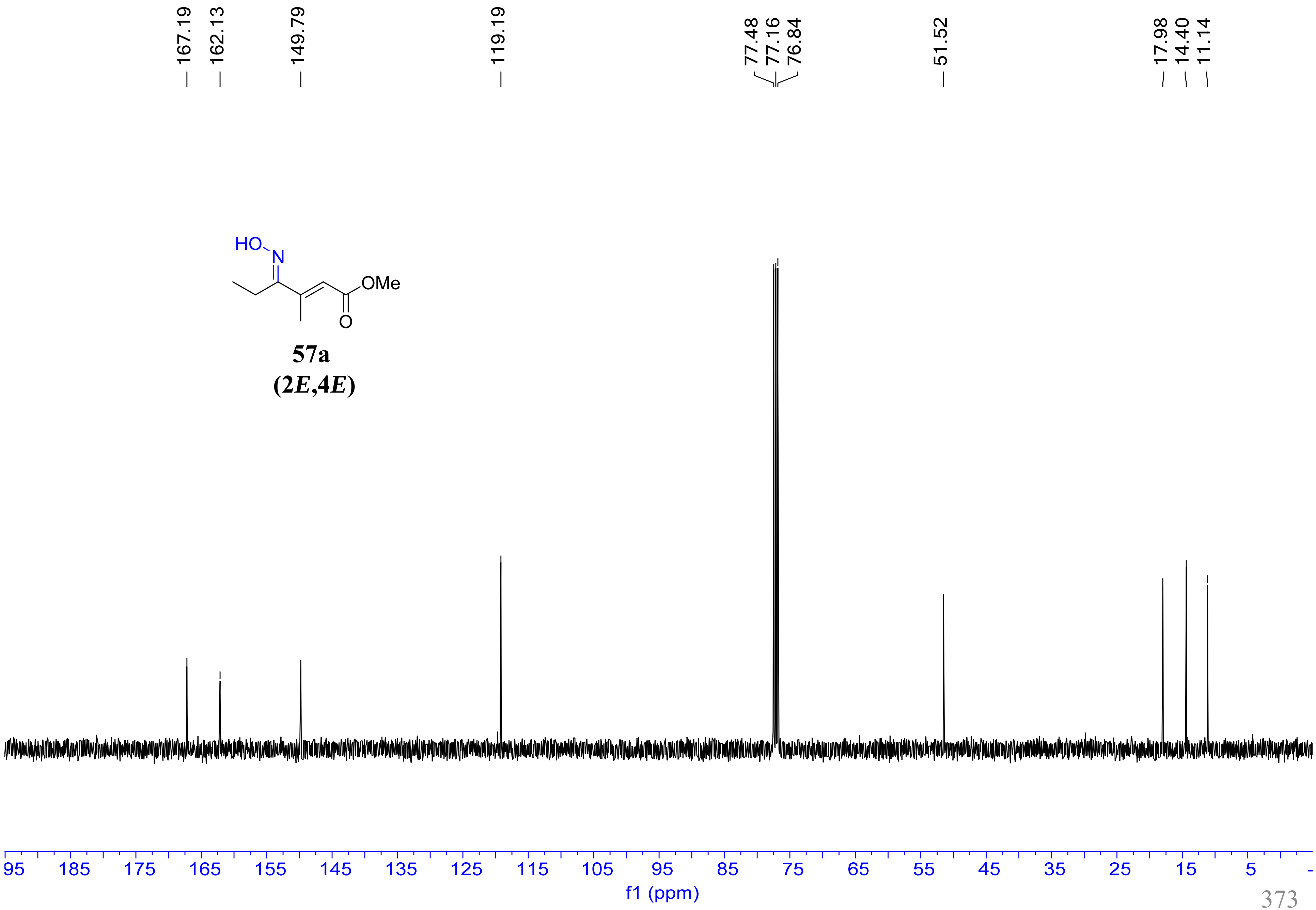

^1H NMR, 400 MHz, $CDCl_3$

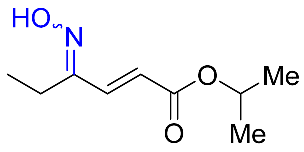

58a

(2*E*,4*E*)/(2*E*,4*Z*)=7:3

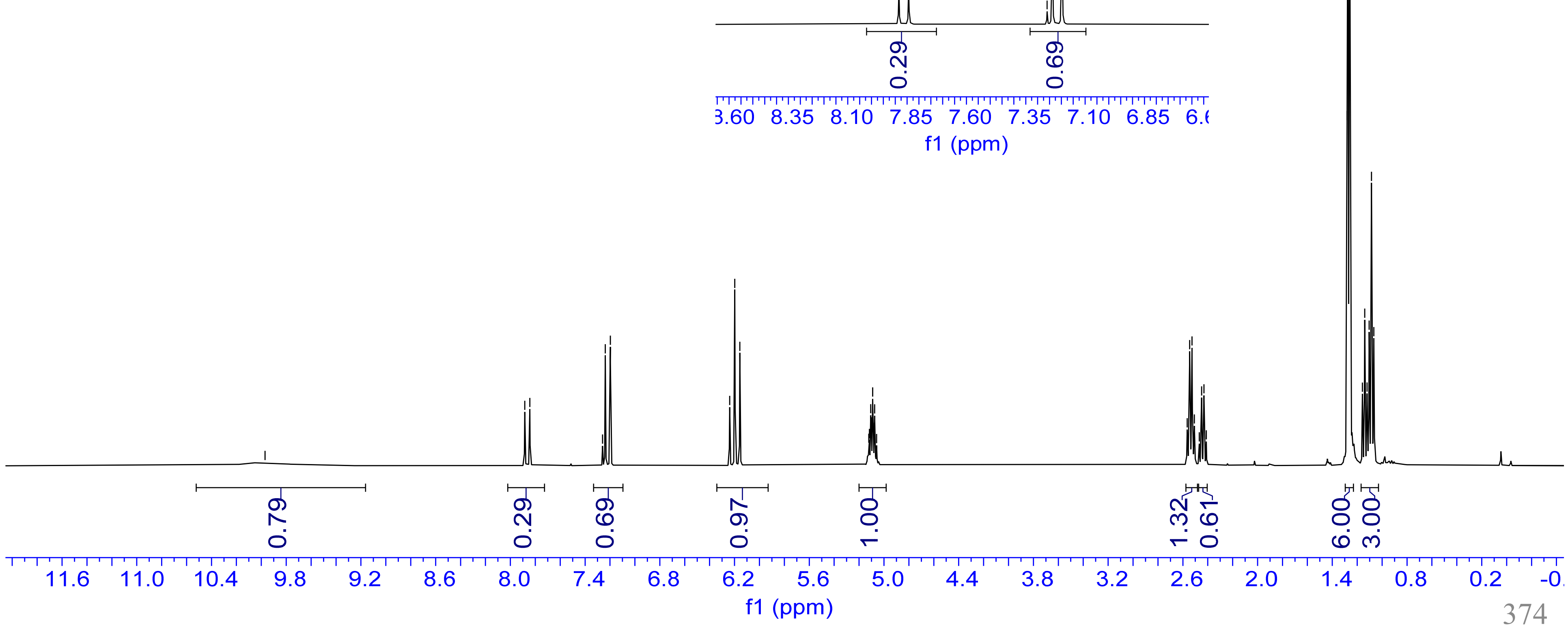

^{13}C NMR 101 MHz, $CDCl_3$

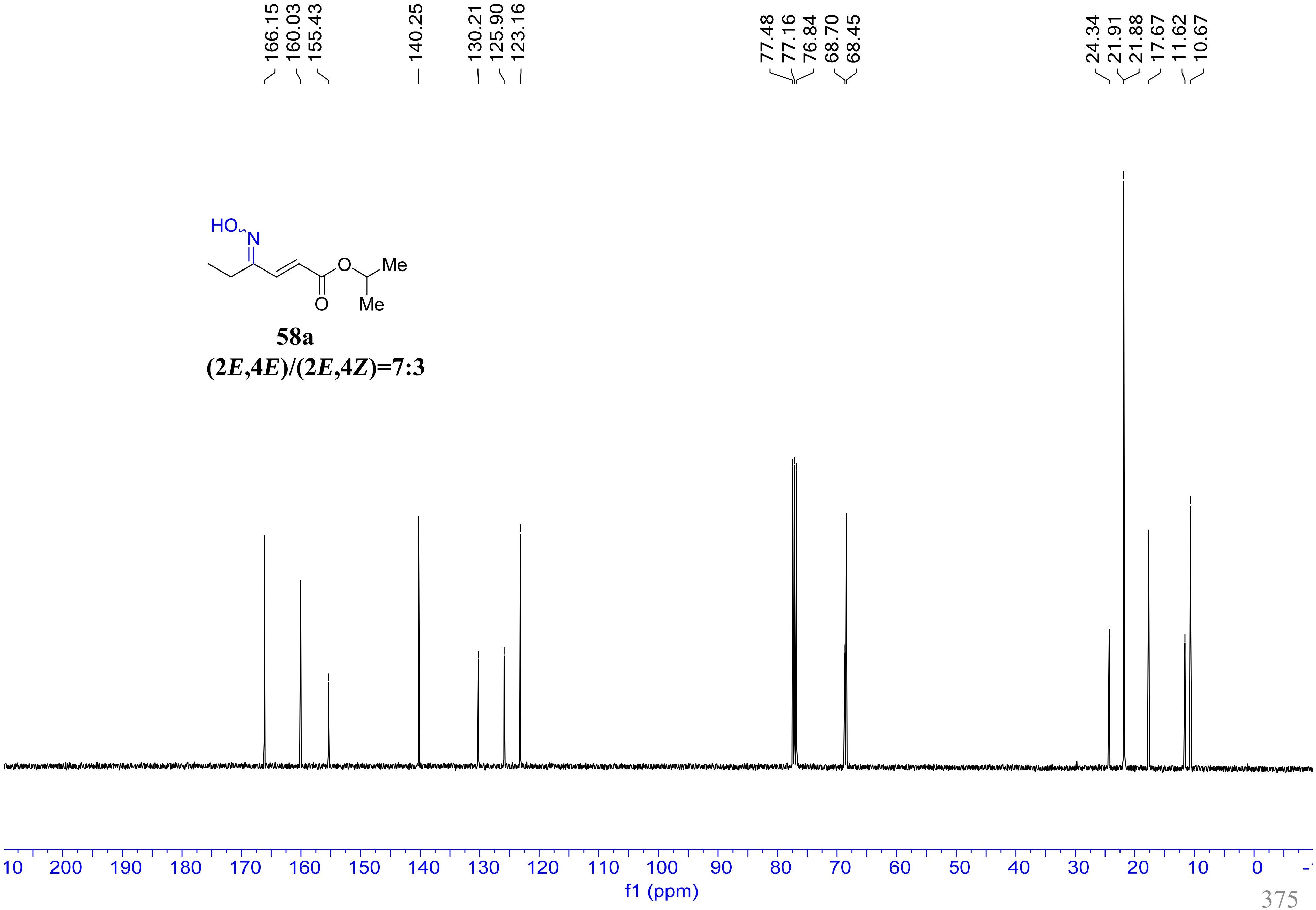

^1H NMR, 400 MHz, $CDCl_3$

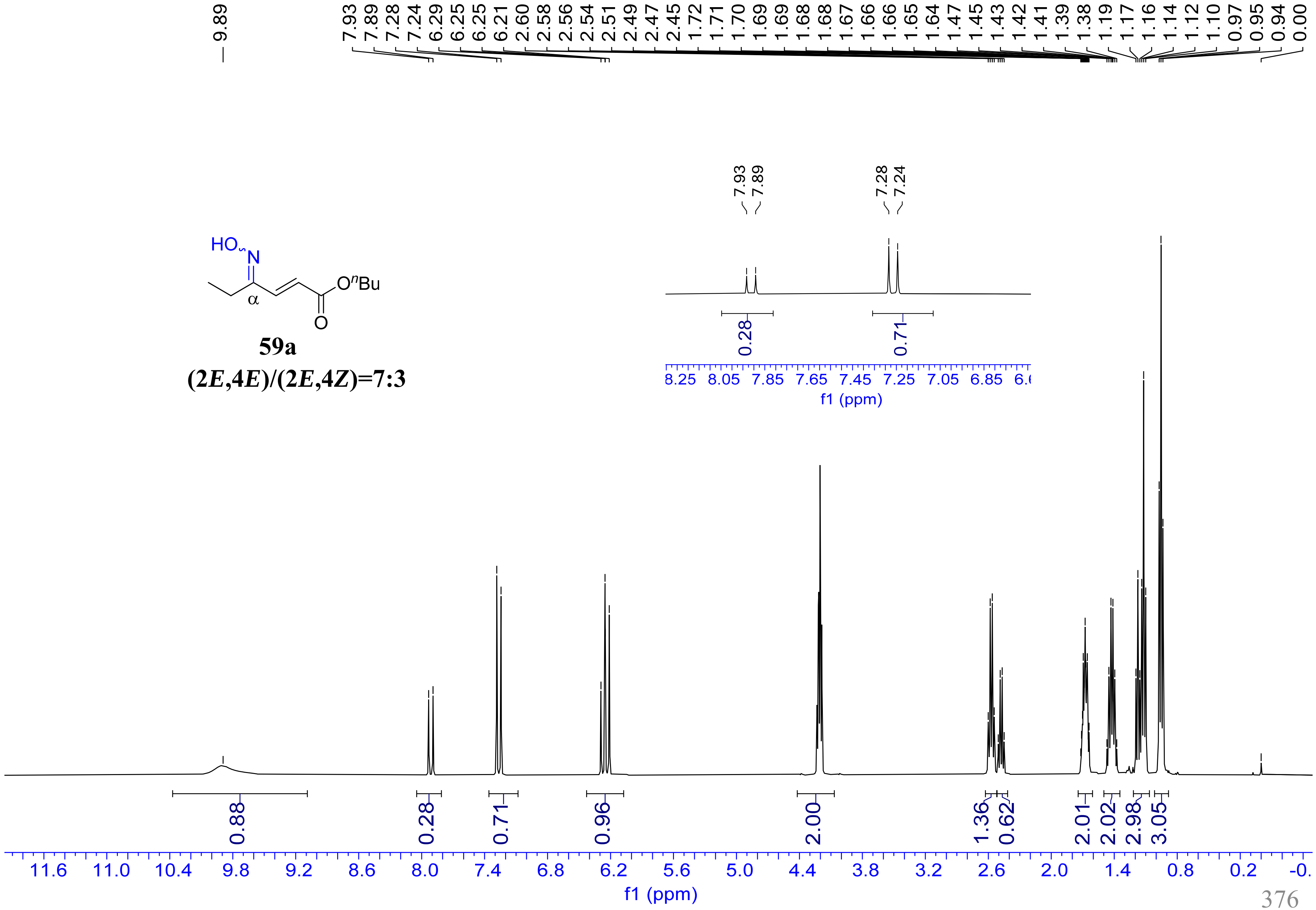

59a

(2*E*,4*E*)/(2*E*,4*Z*)=7:3

^{13}C NMR 101 MHz, $CDCl_3$

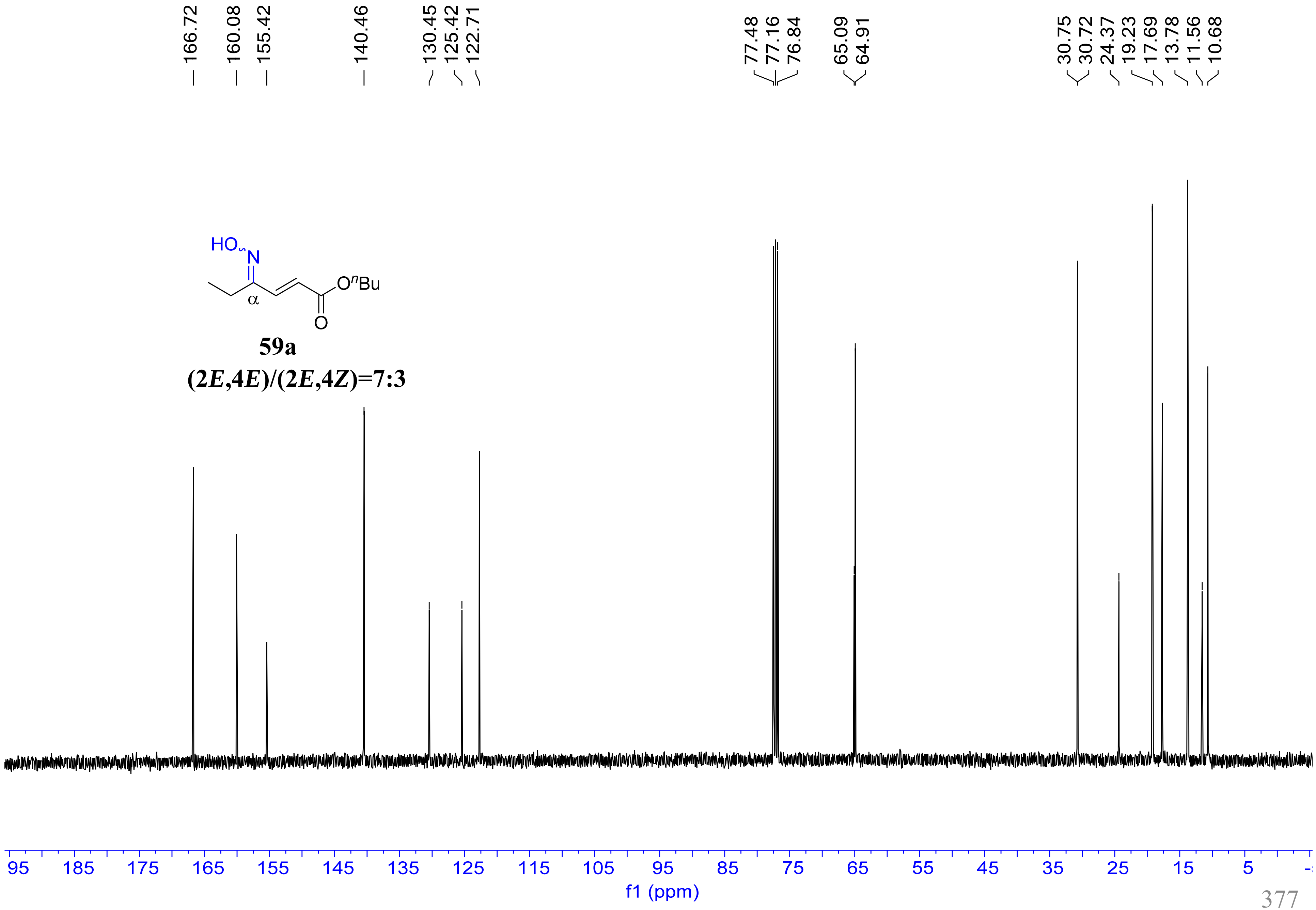

^1H NMR, 400 MHz, $CDCl_3$

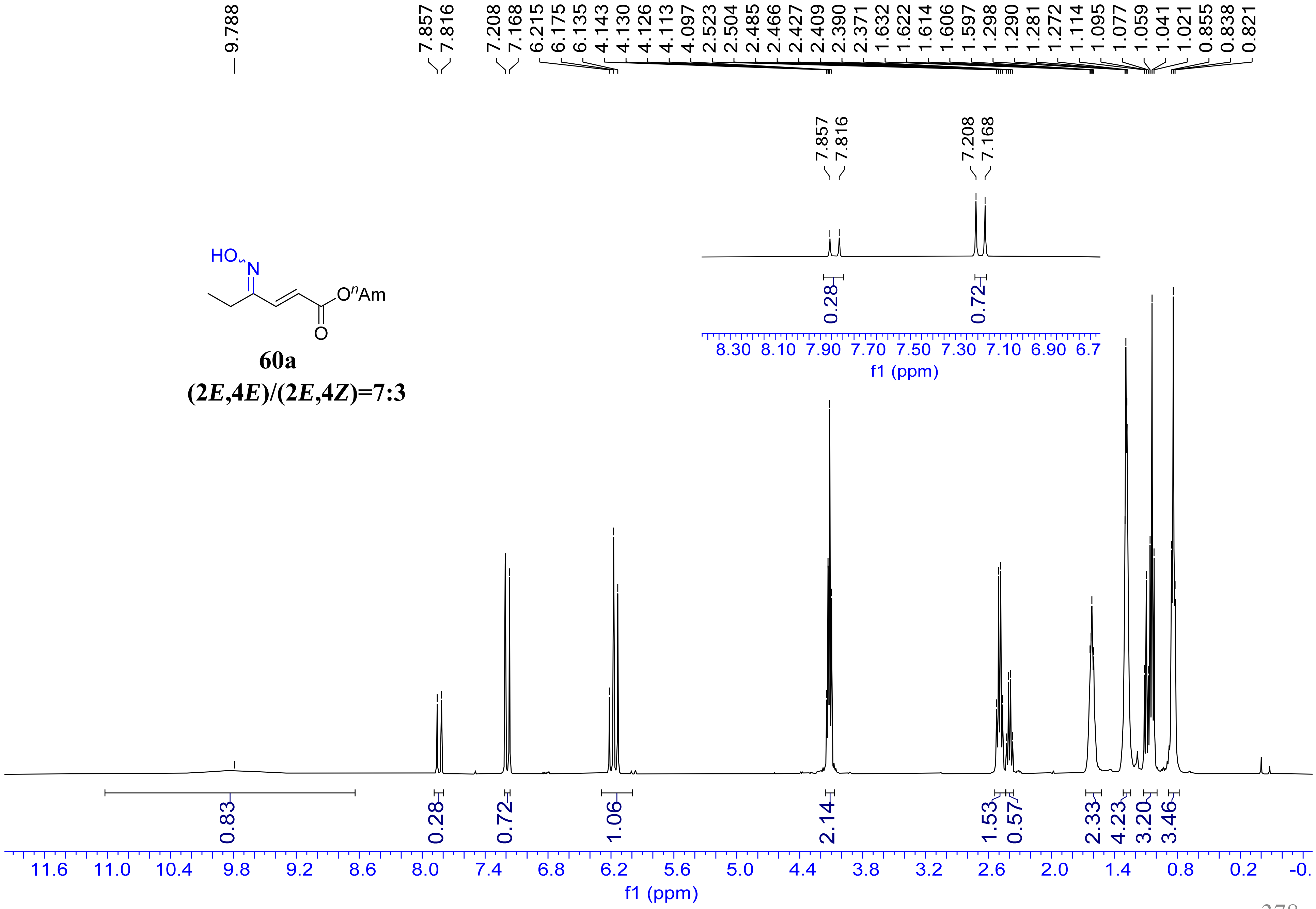

^{13}C NMR 101 MHz, CDCl$_3$

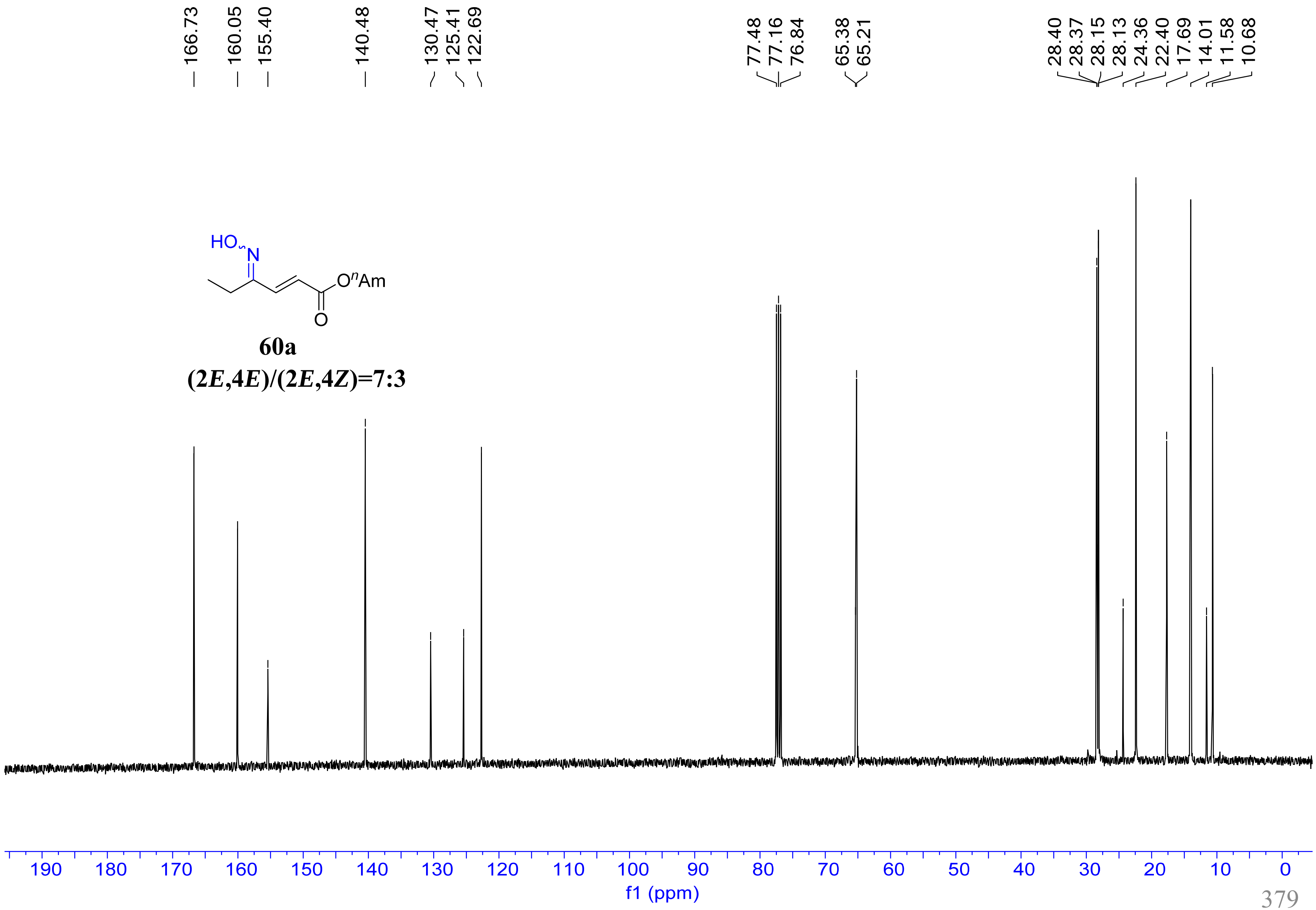

60a

(2*E*,4*E*)/(2*E*,4*Z*)=7:3

^{1}H NMR, 400 MHz, $CDCl_3$

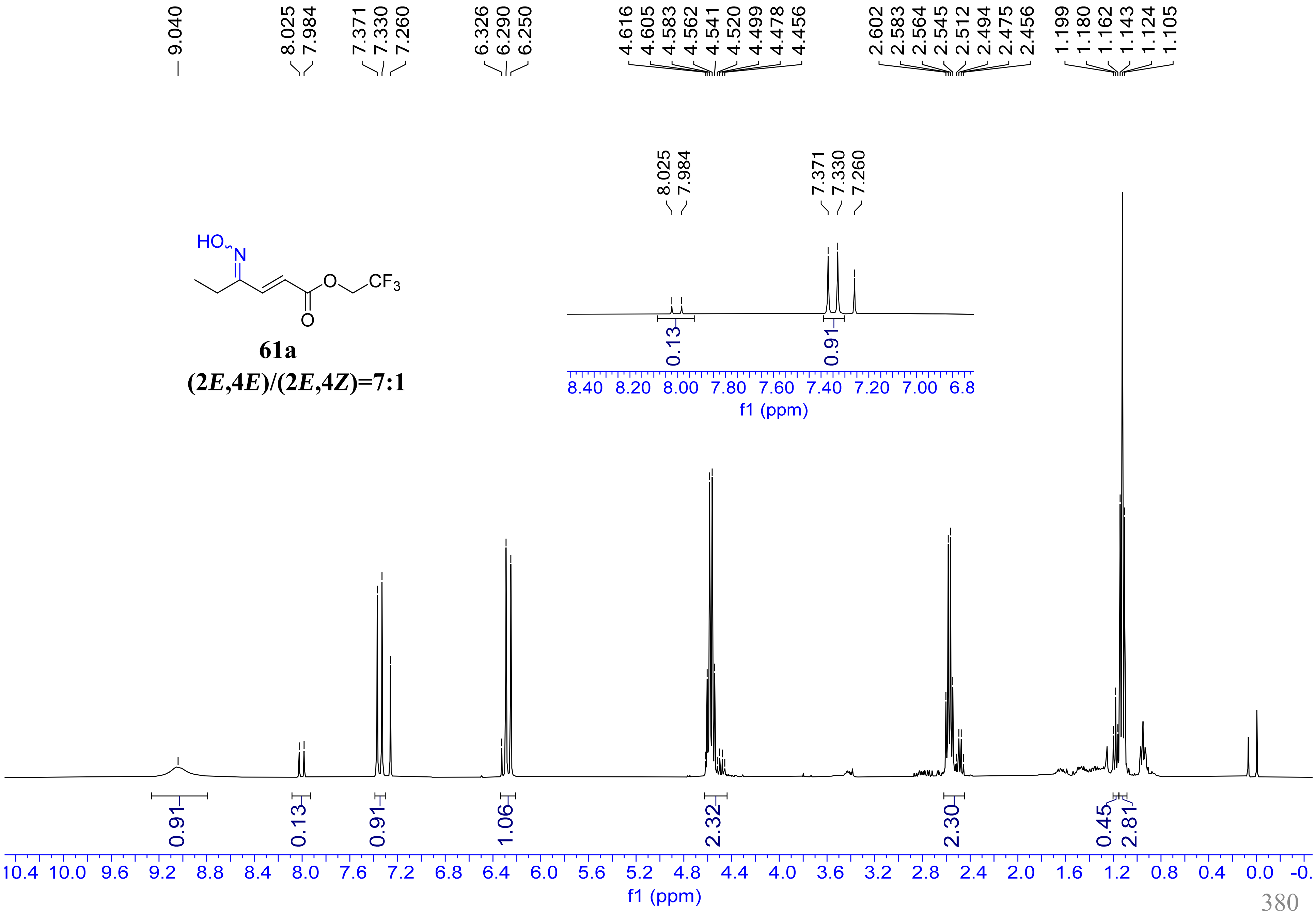

61a

(2*E*,4*E*)/(2*E*,4*Z*)=7:1

^{13}C NMR 101 MHz, $CDCl_3$

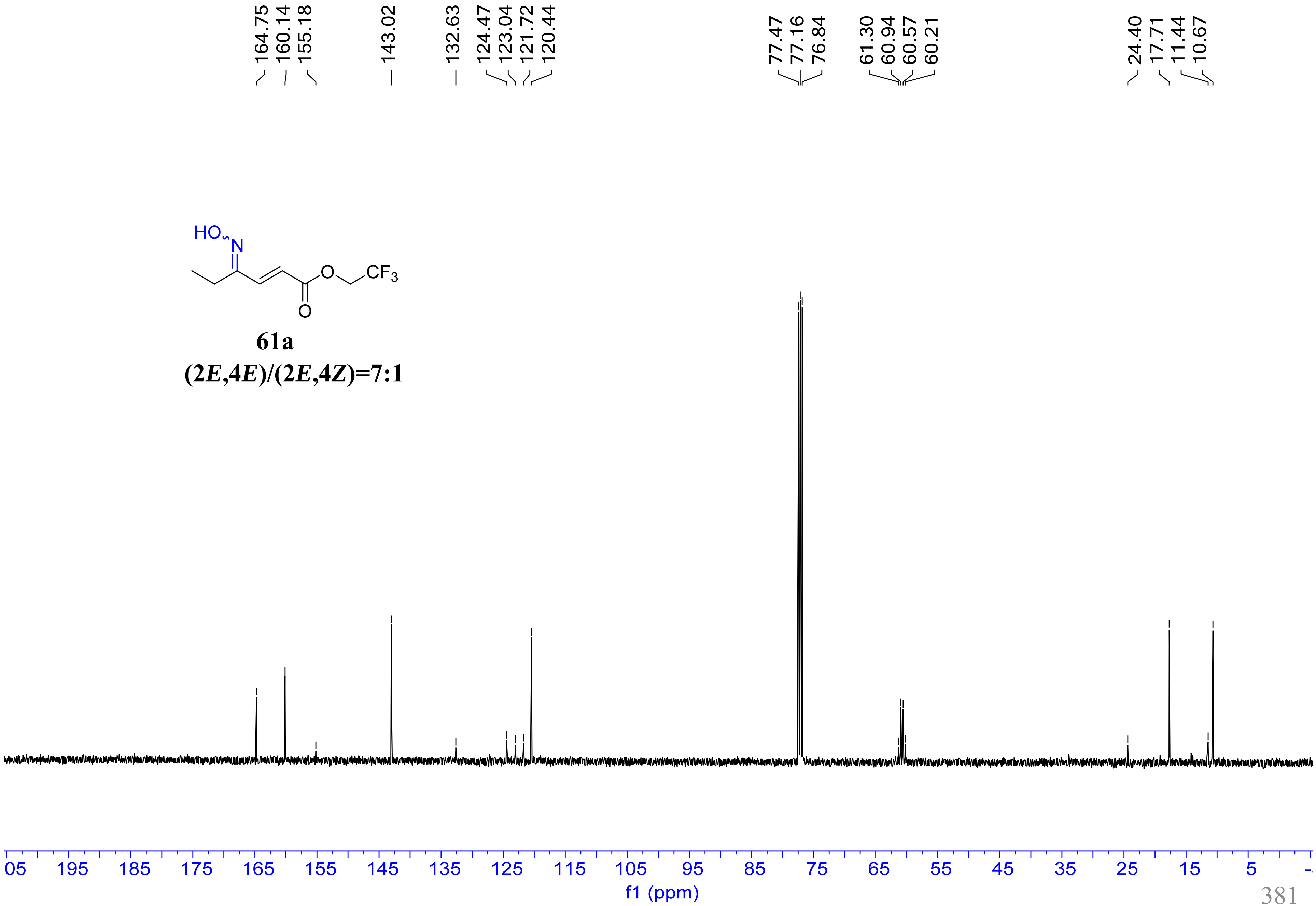

61a

(2*E*,4*E*)/(2*E*,4*Z*)=7:1

^{1}H NMR, 400 MHz, $CDCl_3$

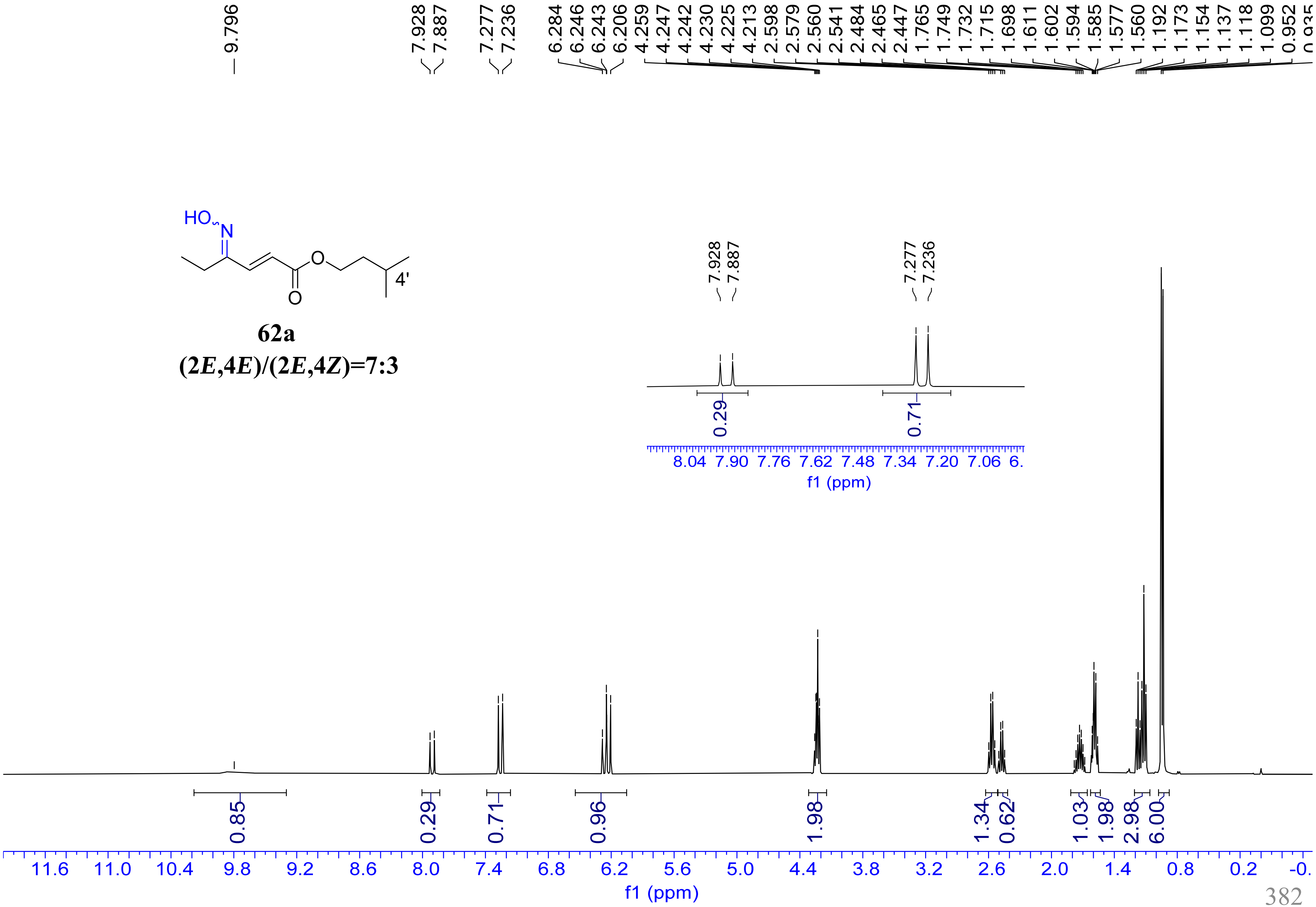

62a

(2*E*,4*E*)/(2*E*,4*Z*)=7:3

^{13}C NMR 101 MHz, $CDCl_3$

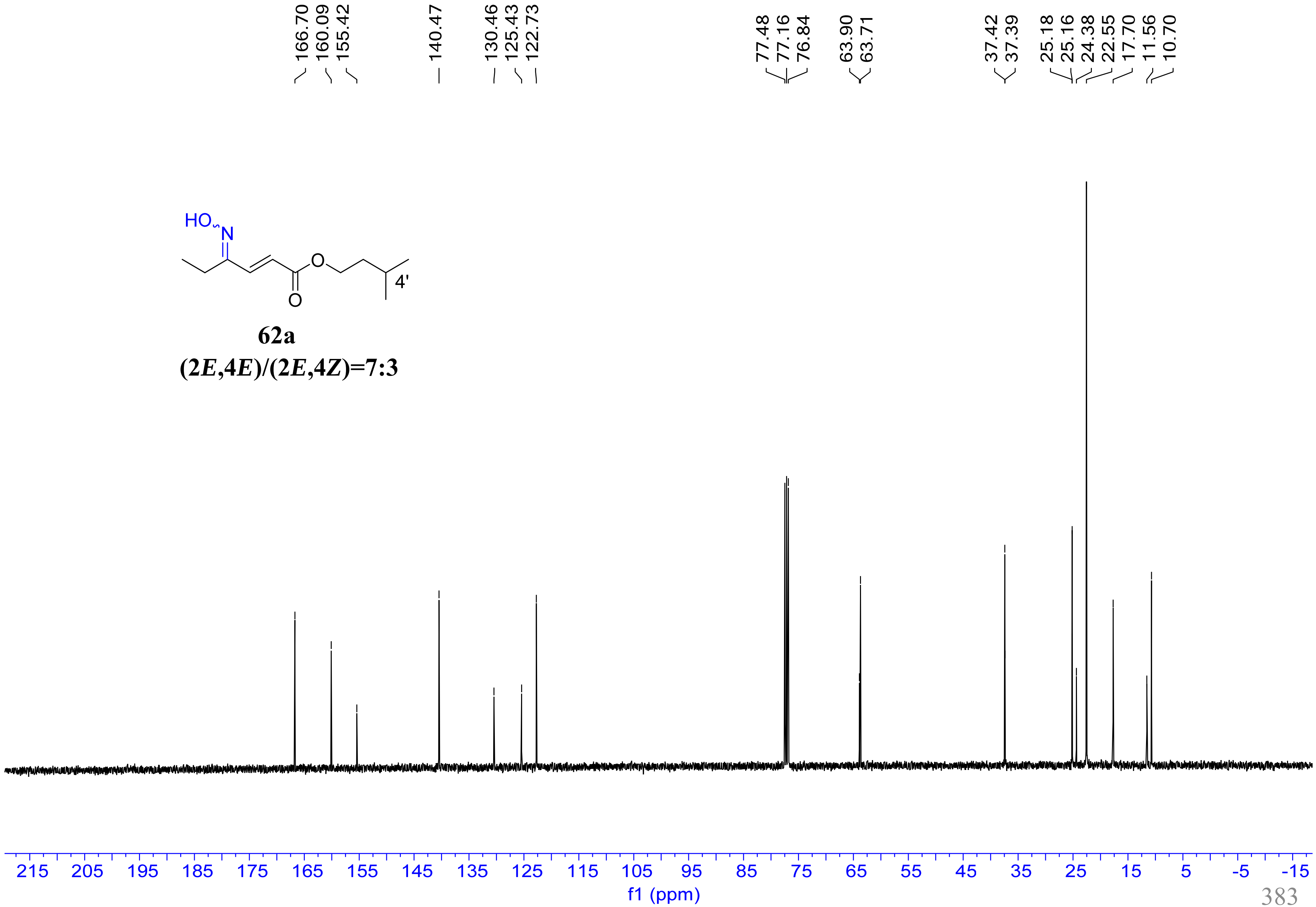

62a

(2*E*,4*E*)/(2*E*,4*Z*)=7:3

^{1}H NMR, 400 MHz, $CDCl_3$

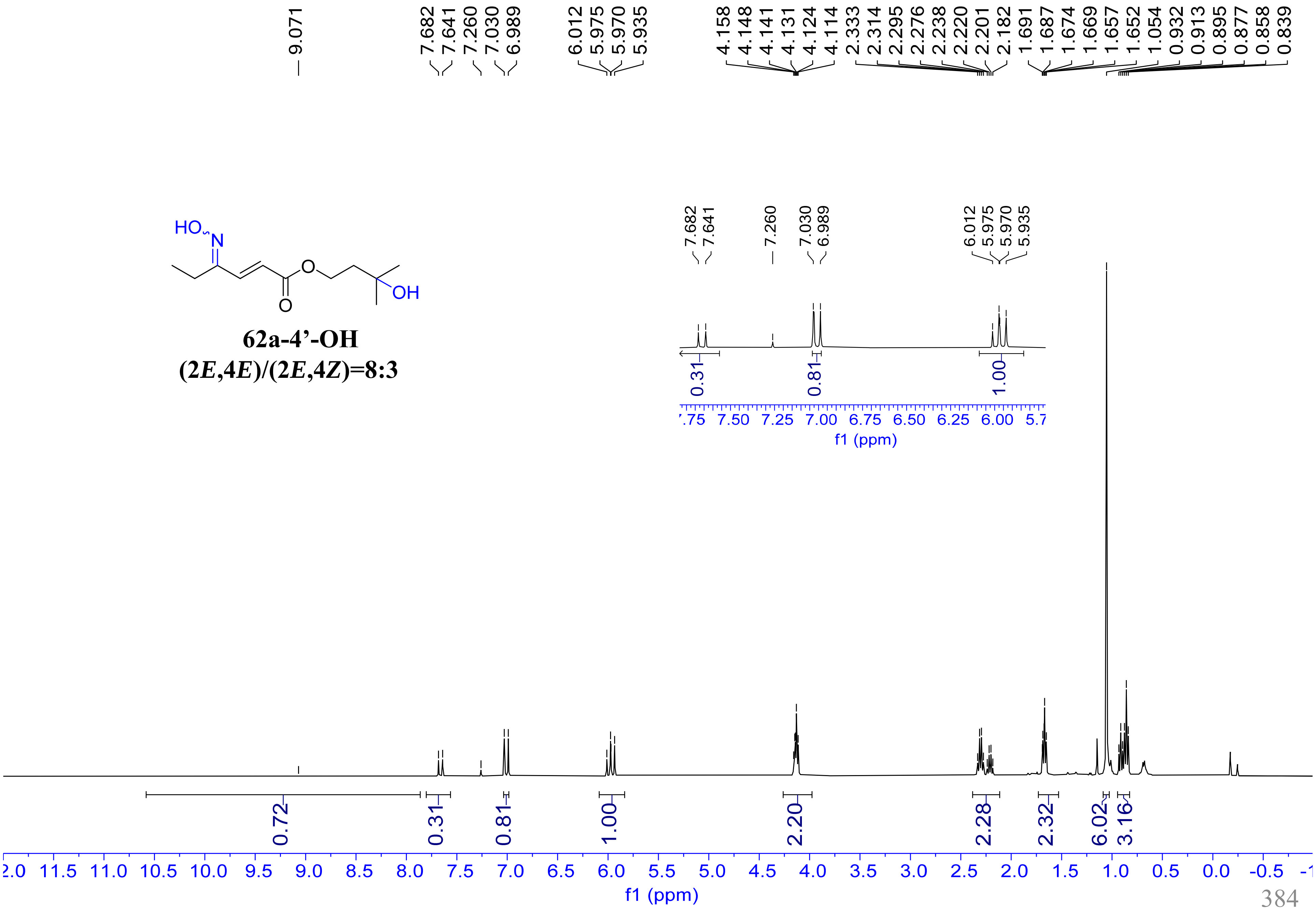

^{13}C NMR 101 MHz, $CDCl_3$

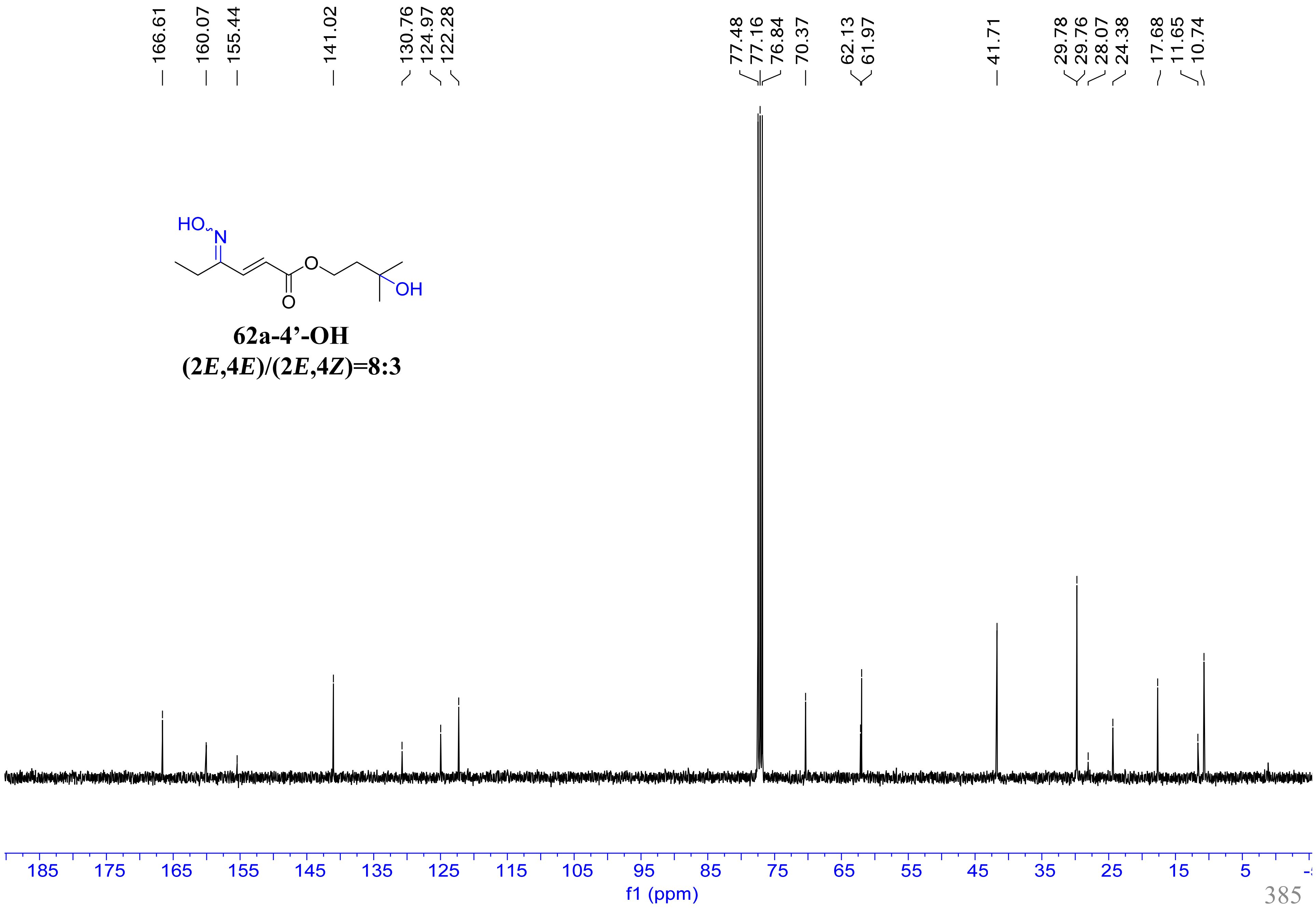

62a-4'-OH

(2*E*,4*E*)/(2*E*,4*Z*)=8:3

^{1}H NMR, 400 MHz, $CDCl_3$

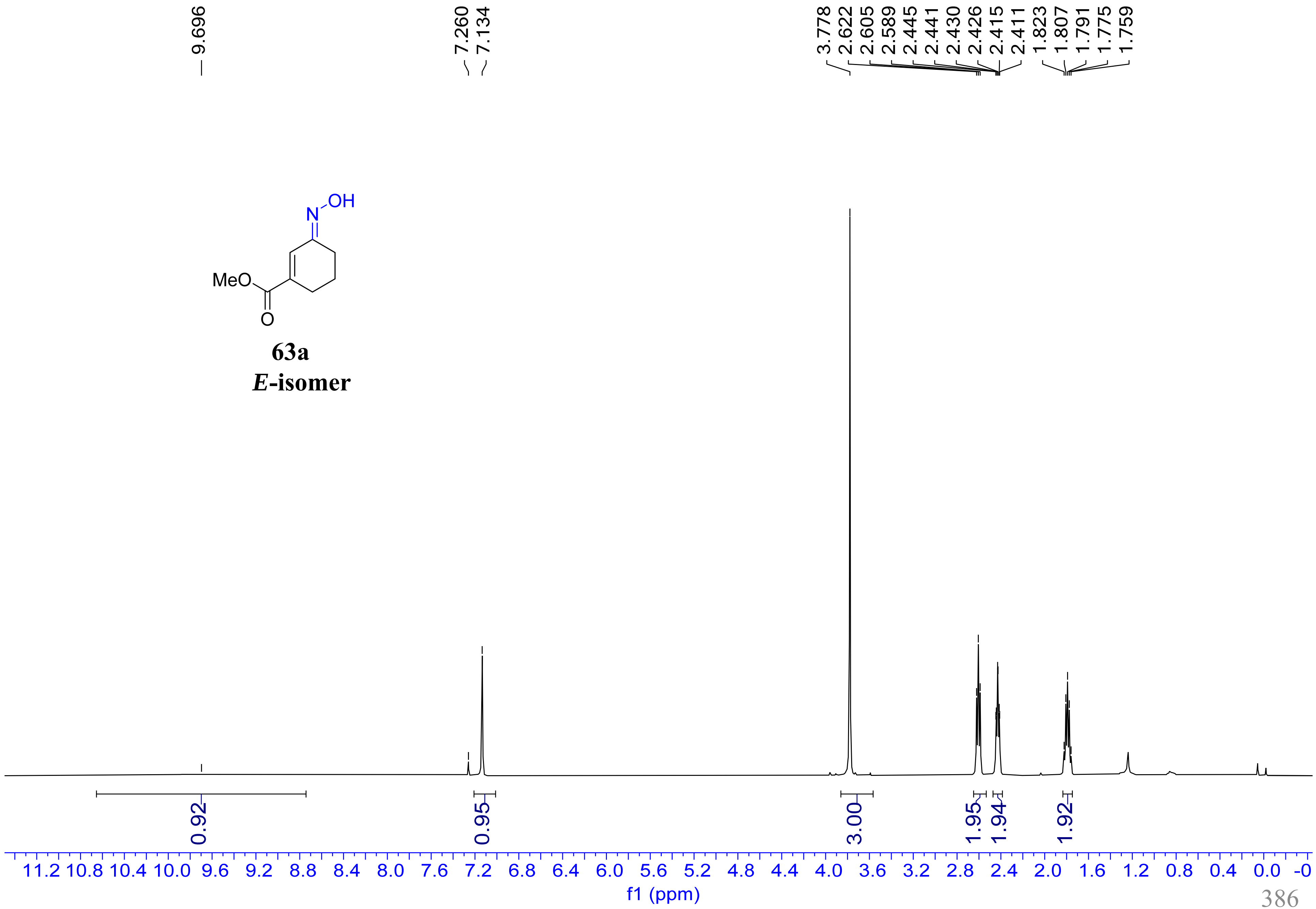

63a
***E*-isomer**

^{13}C NMR 101 MHz, $CDCl_3$

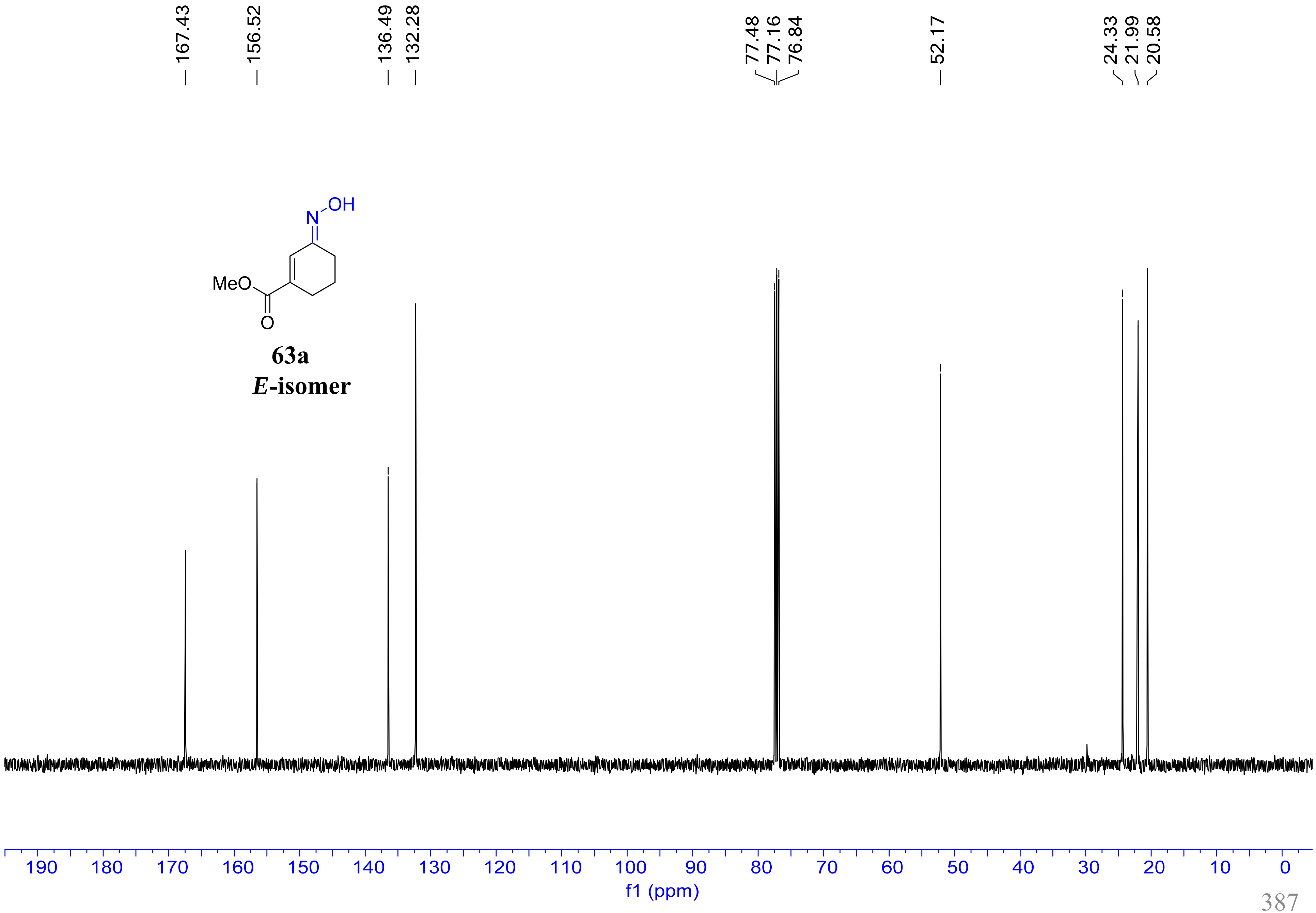

^{1}H NMR, 400 MHz, $CDCl_3$

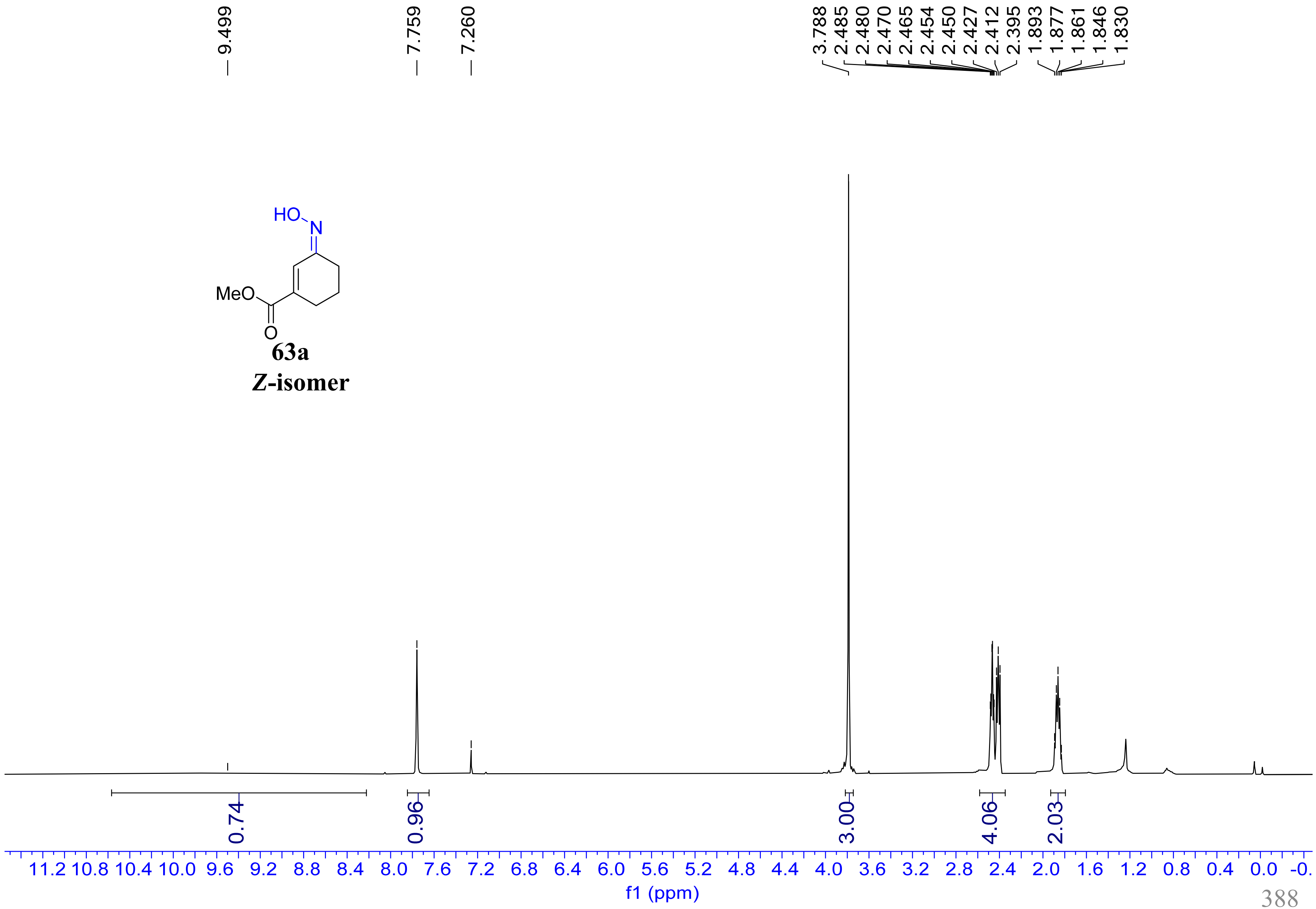

^{13}C NMR 101 MHz, $CDCl_3$

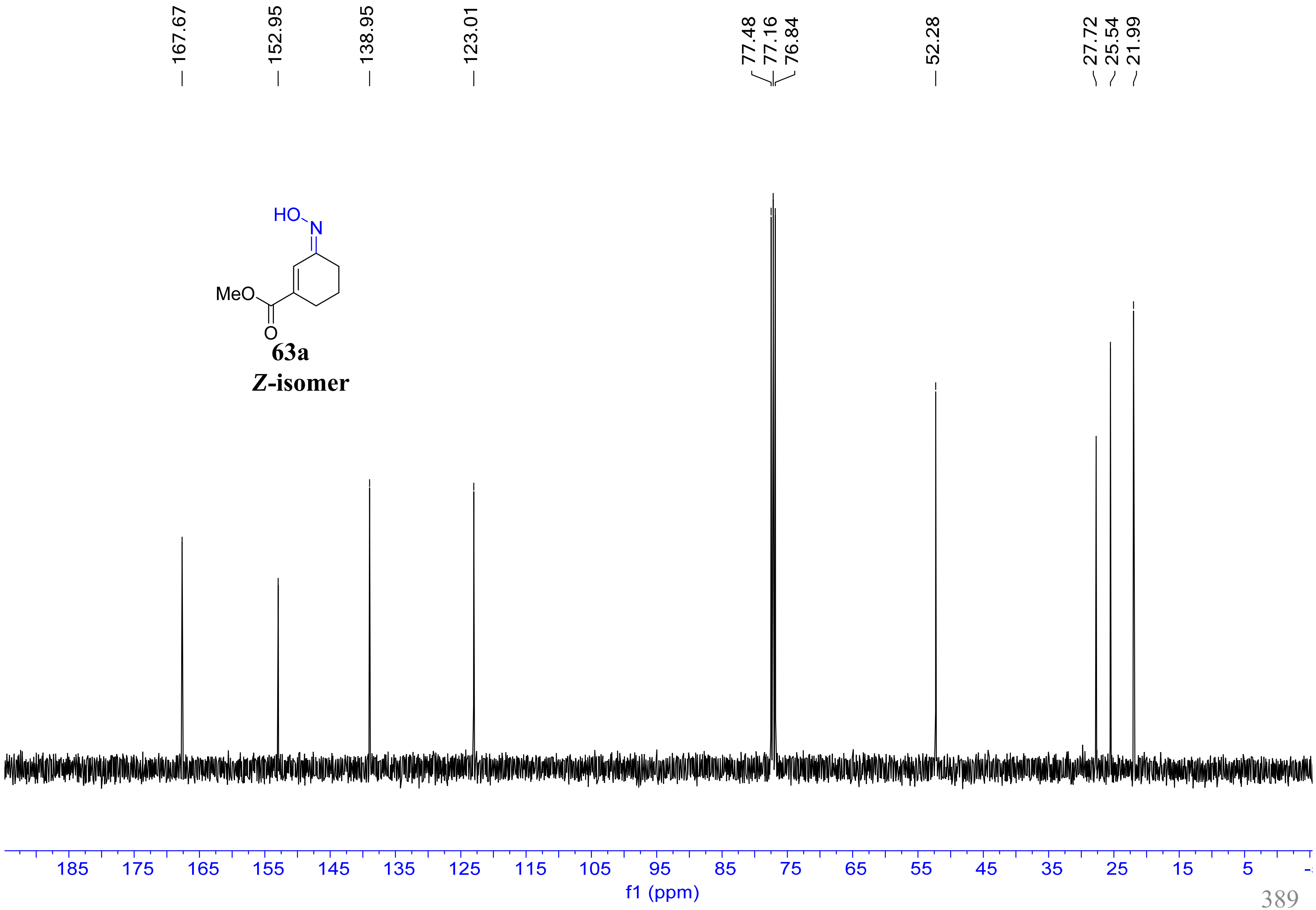

63a

***Z*-isomer**

^{1}H NMR, 400 MHz, DMSO-d6

64a

E/Z=29:1

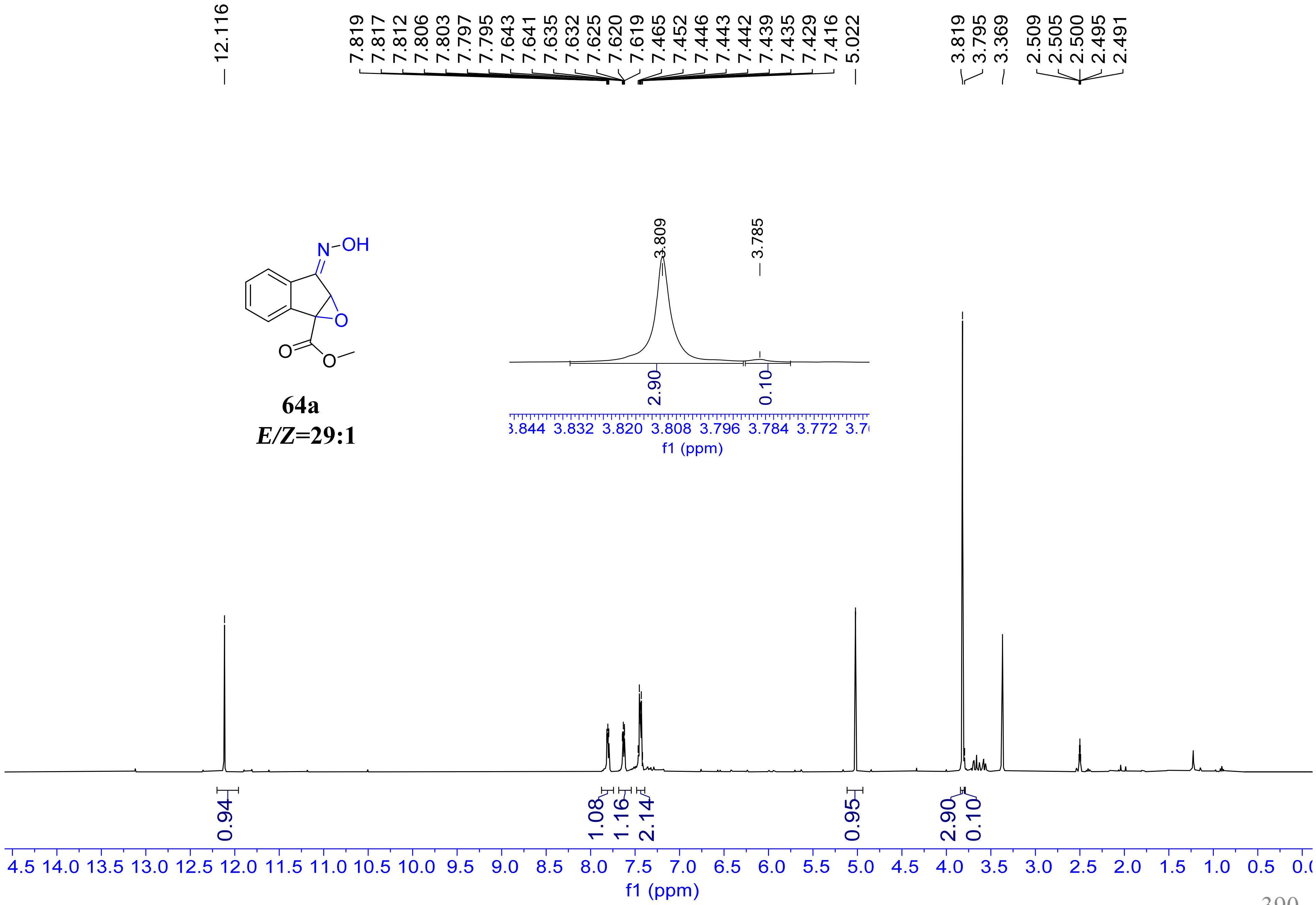

^{13}C NMR 101 MHz, DMSO-d6

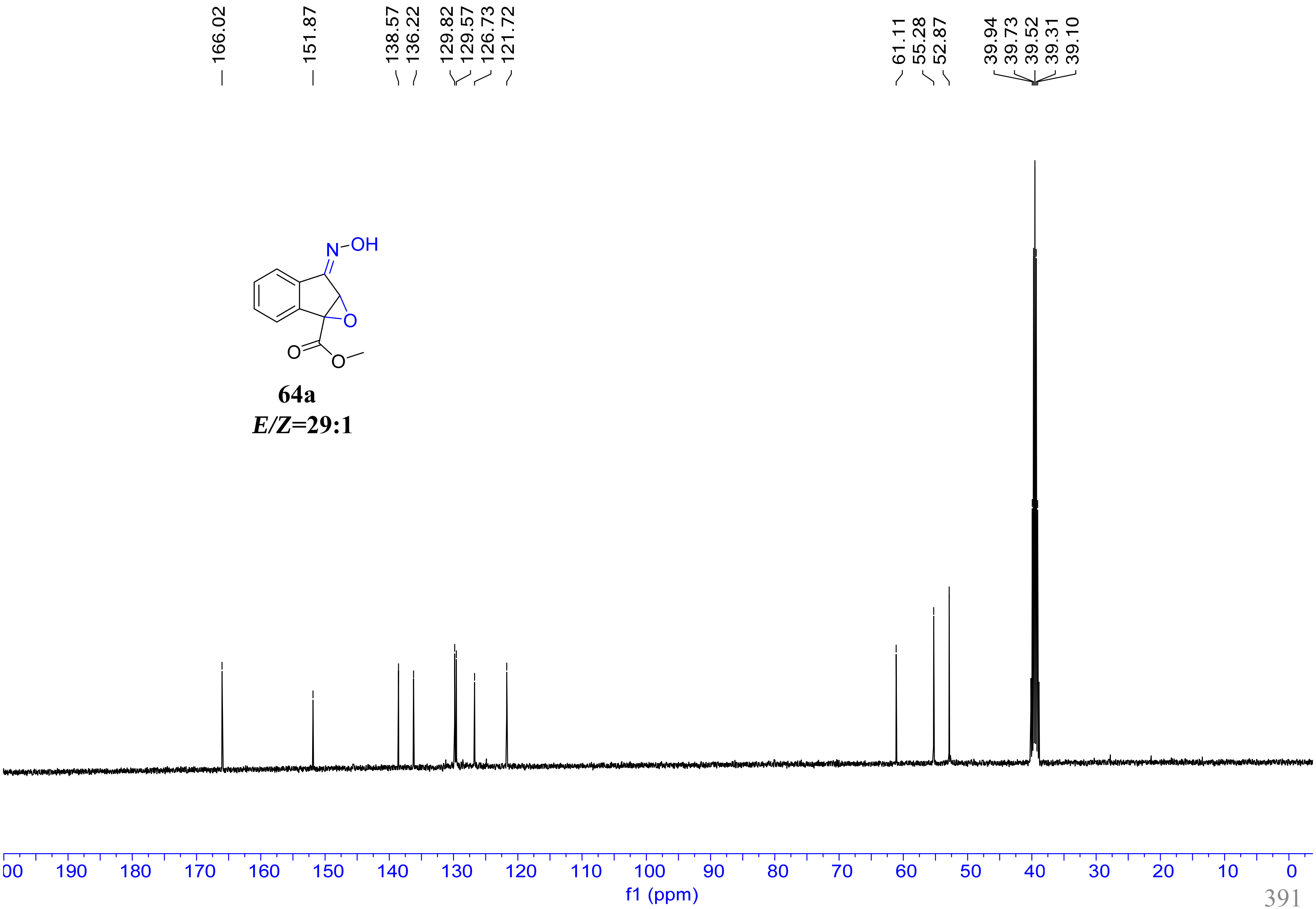

^{1}H NMR, 400 MHz, $CDCl_3$

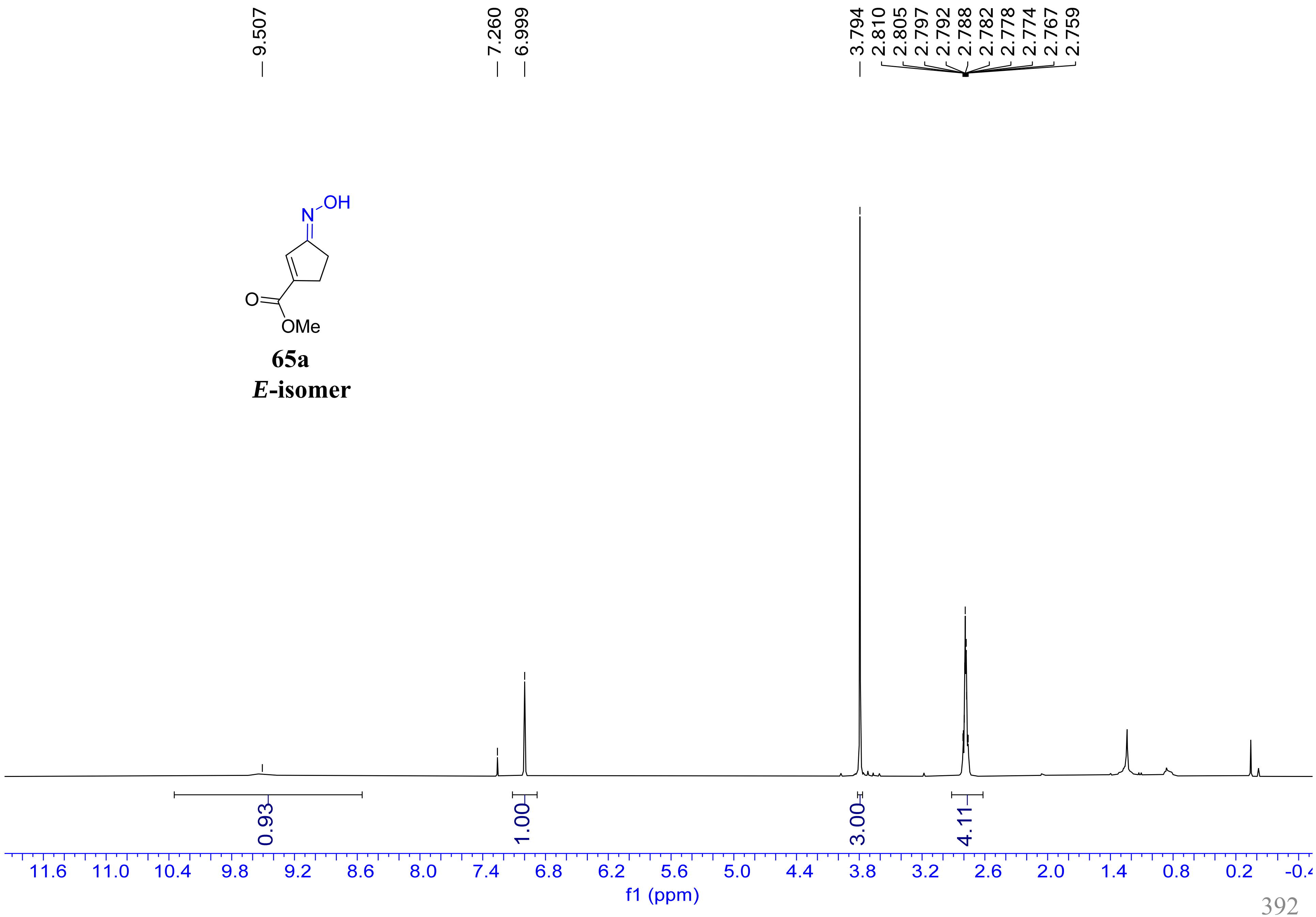

^{13}C NMR 101 MHz, $CDCl_3$

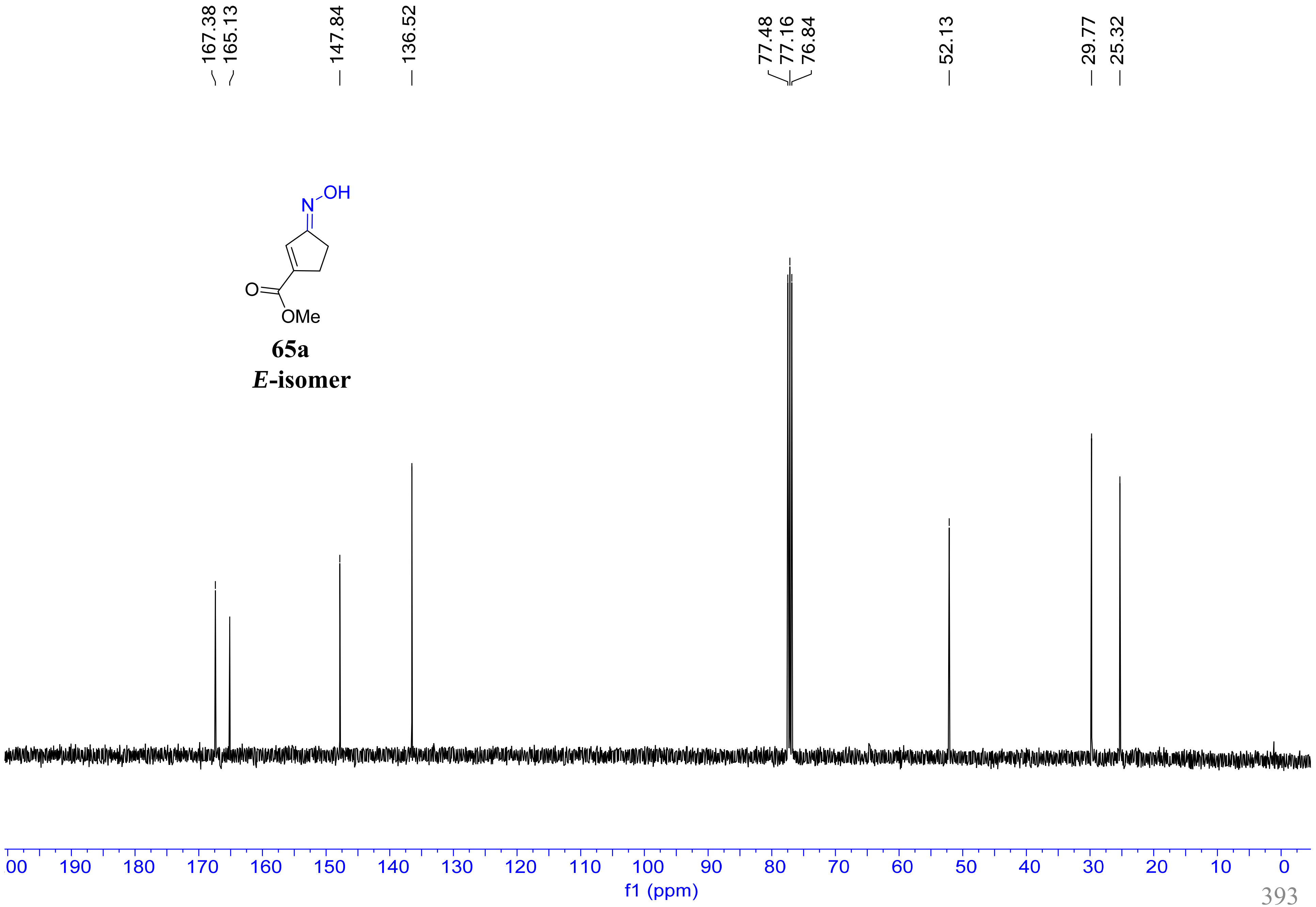

65a

E-isomer

^{1}H NMR, 400 MHz, $CDCl_3$

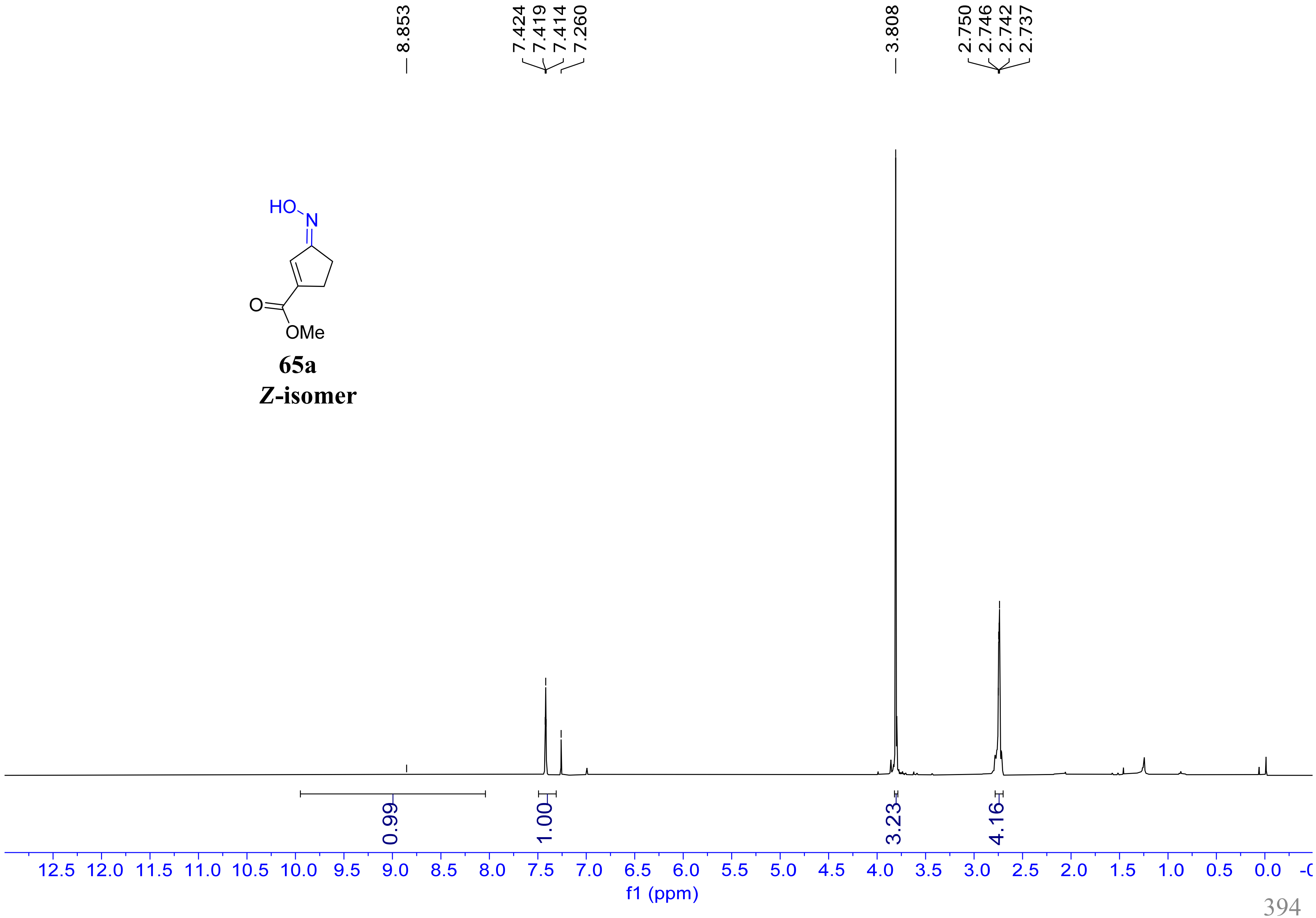

65a

***Z*-isomer**

^{13}C NMR 101 MHz, $CDCl_3$

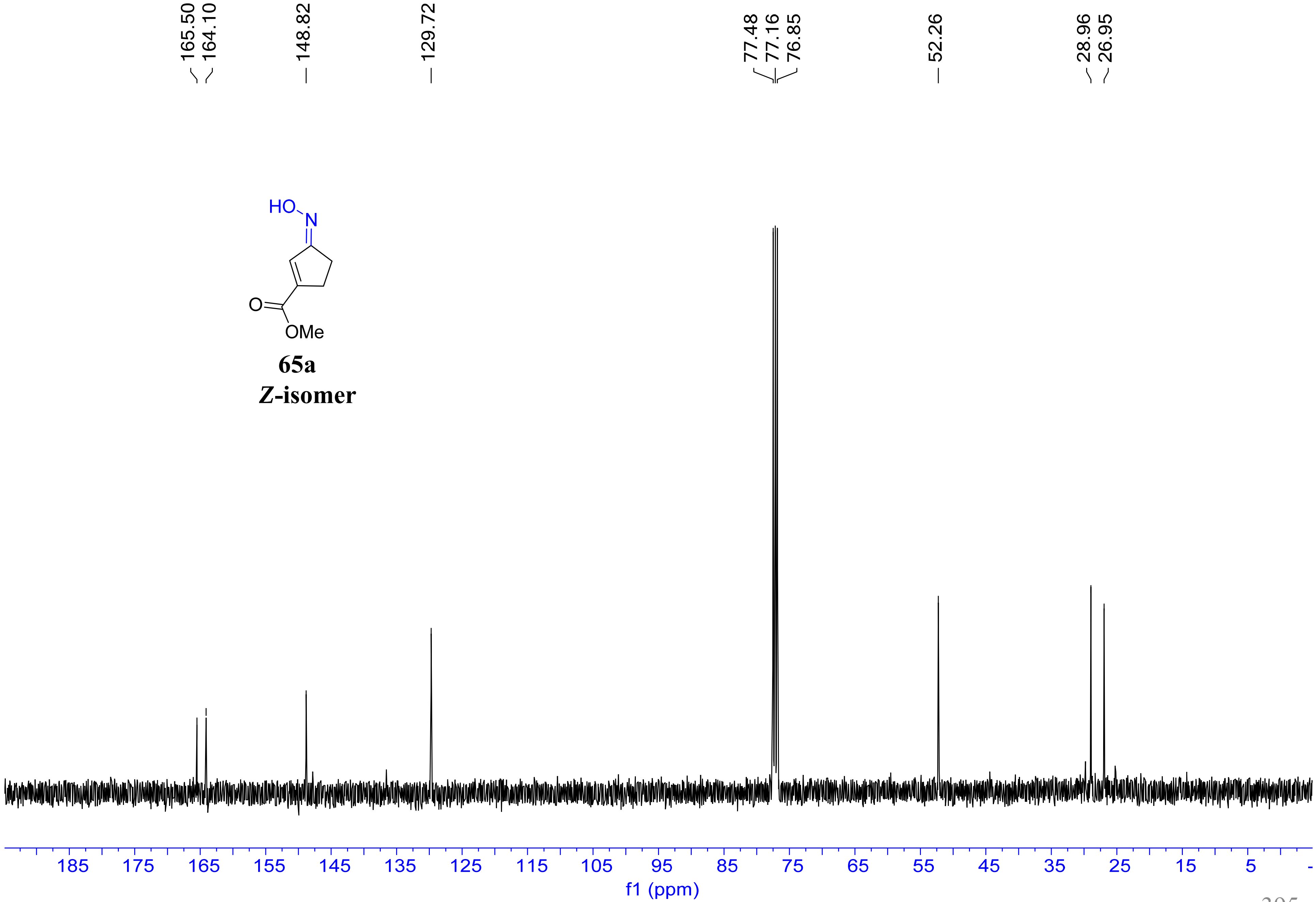

^{1}H NMR, 400 MHz, DMSO-d6

66a

(2*E*,4*E*)/(2*E*,4*Z*)=3:1

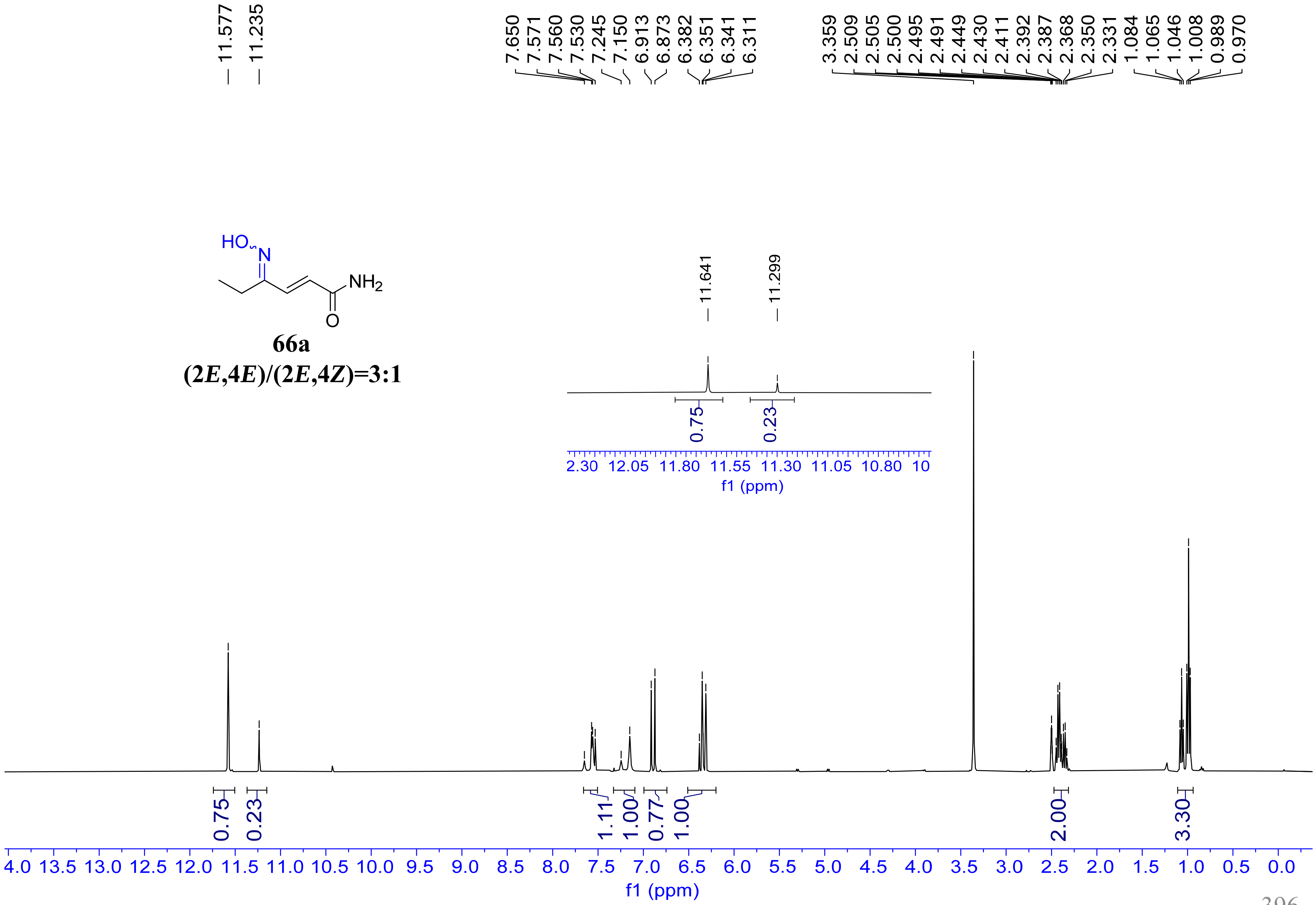

^{13}C NMR 101 MHz, DMSO-d6

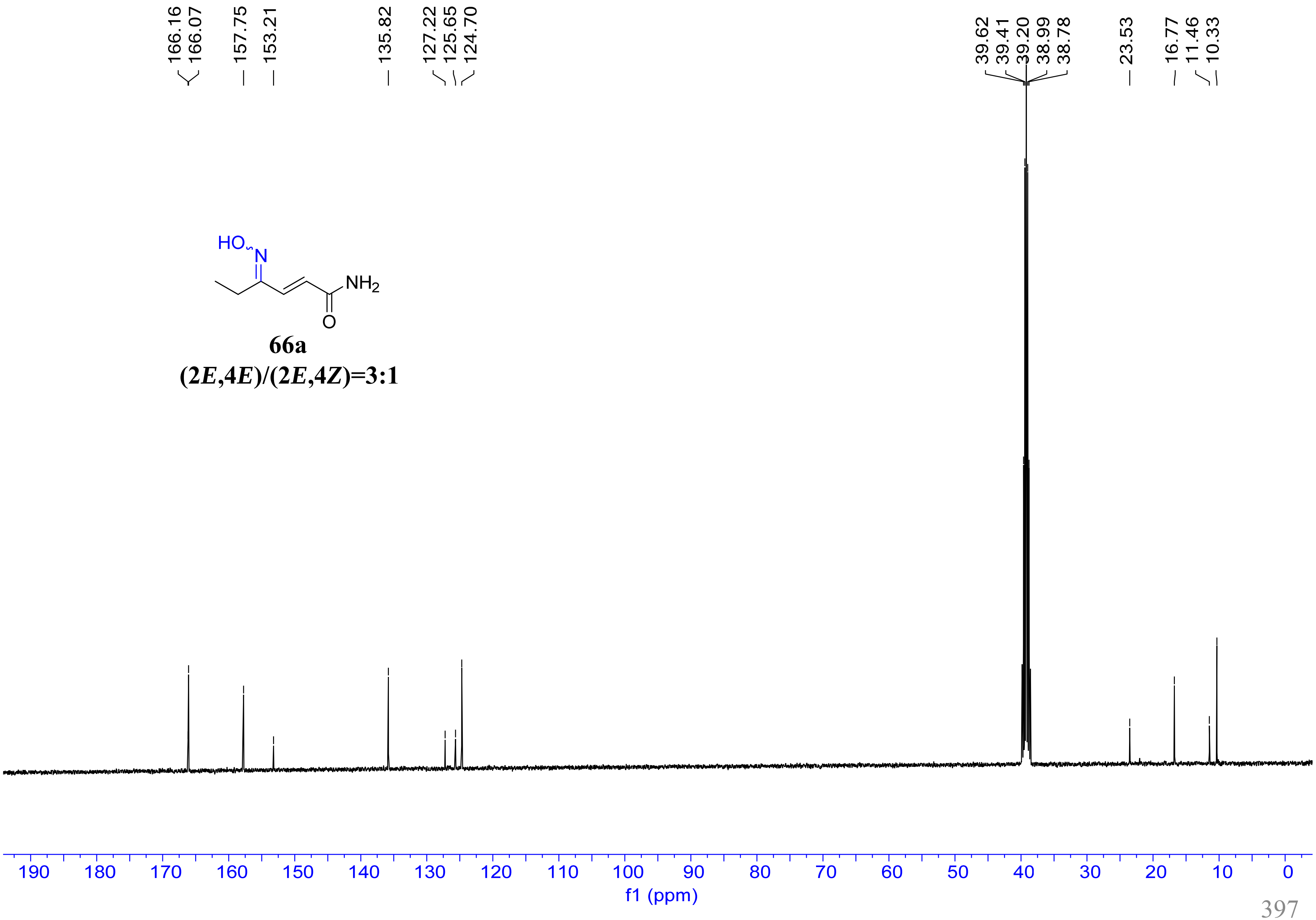

66a

(2*E*,4*E*)/(2*E*,4*Z*)=3:1

¹H NMR, 400 MHz, DMSO-d6

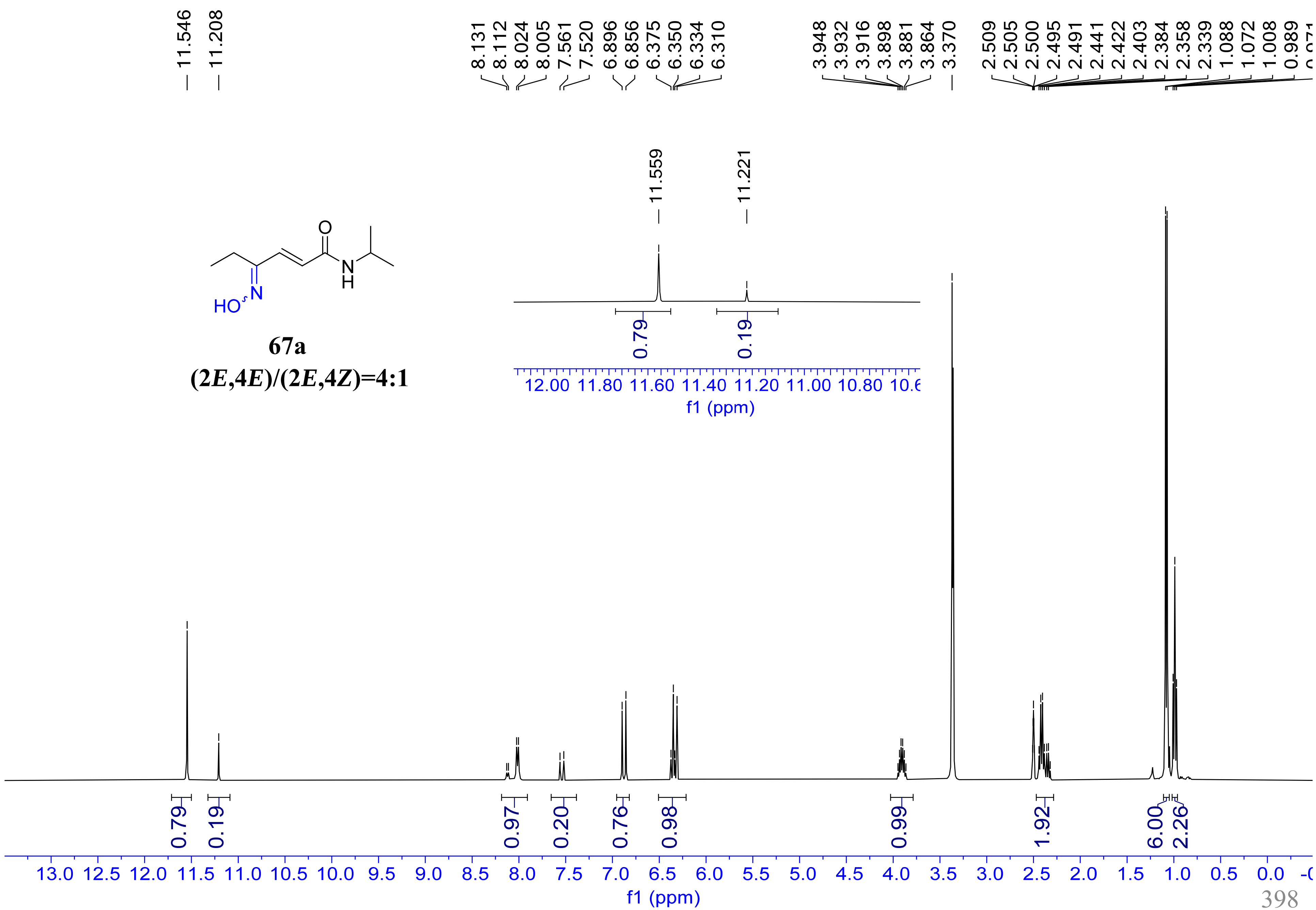

67a

(2*E*,4*E*)/(2*E*,4*Z*)=4:1

^{13}C NMR 101 MHz, DMS0-d6

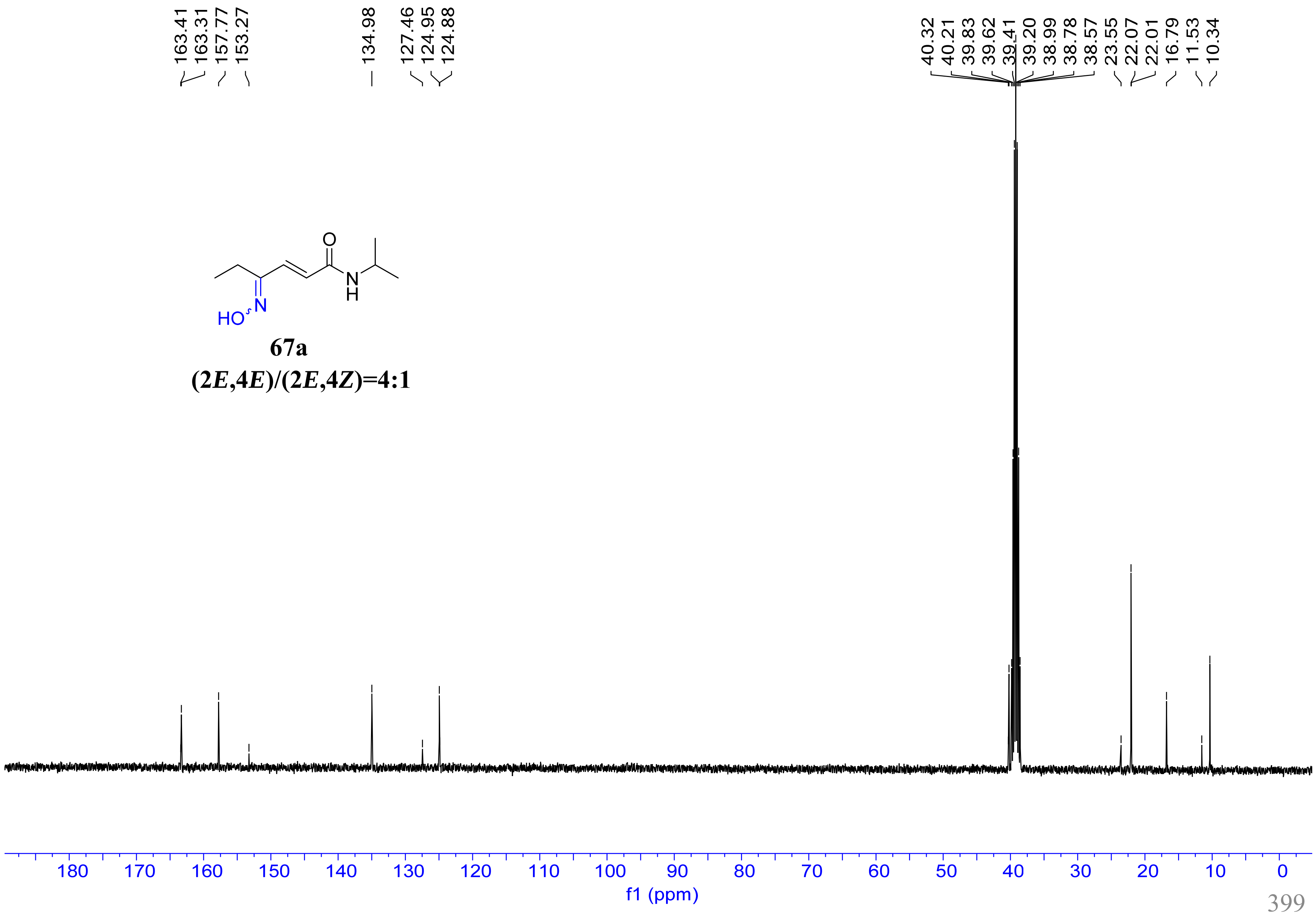

^{1}H NMR, 400 MHz, DMSO-d6

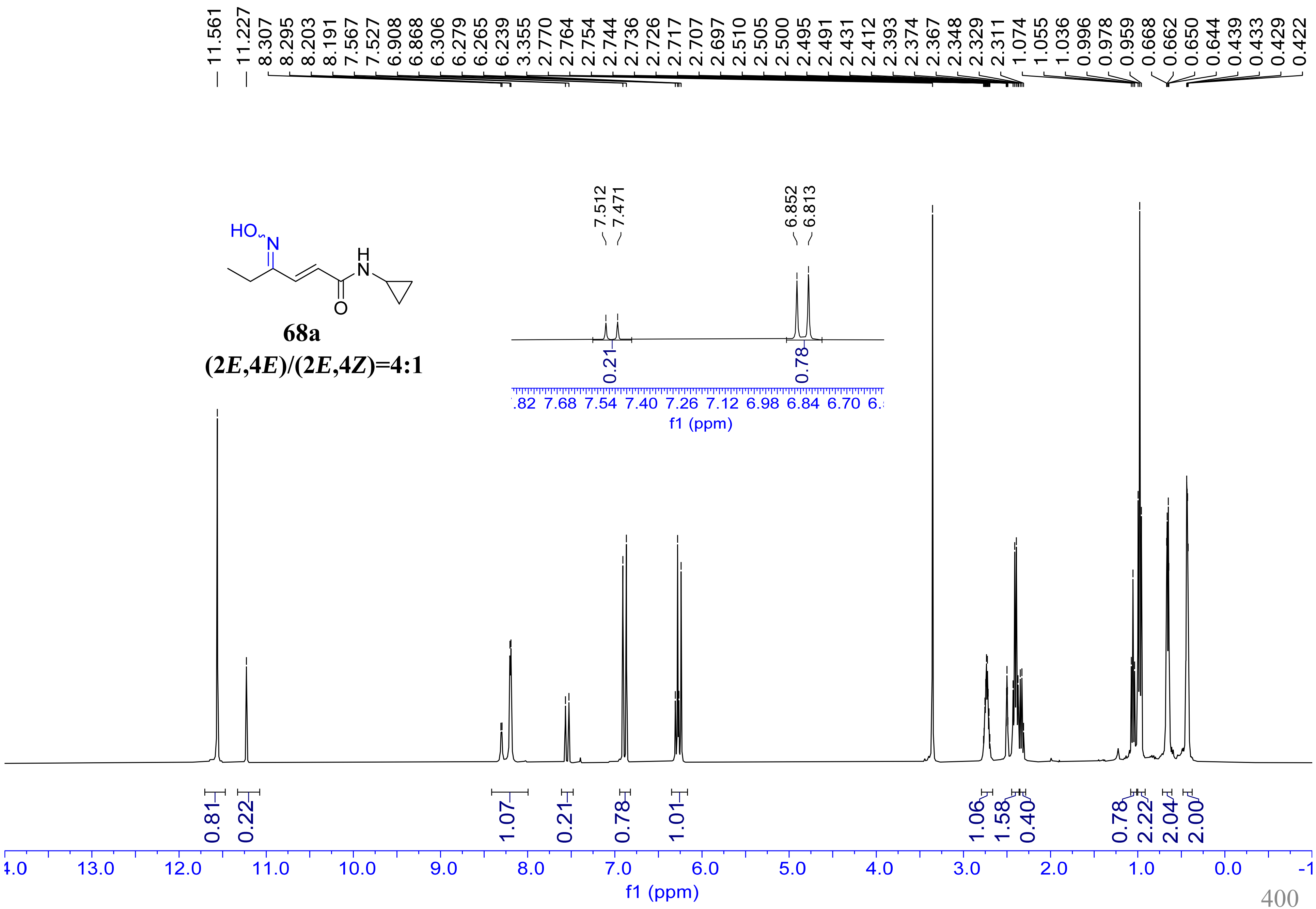

^{13}C NMR 101 MHz, DMSO-d6

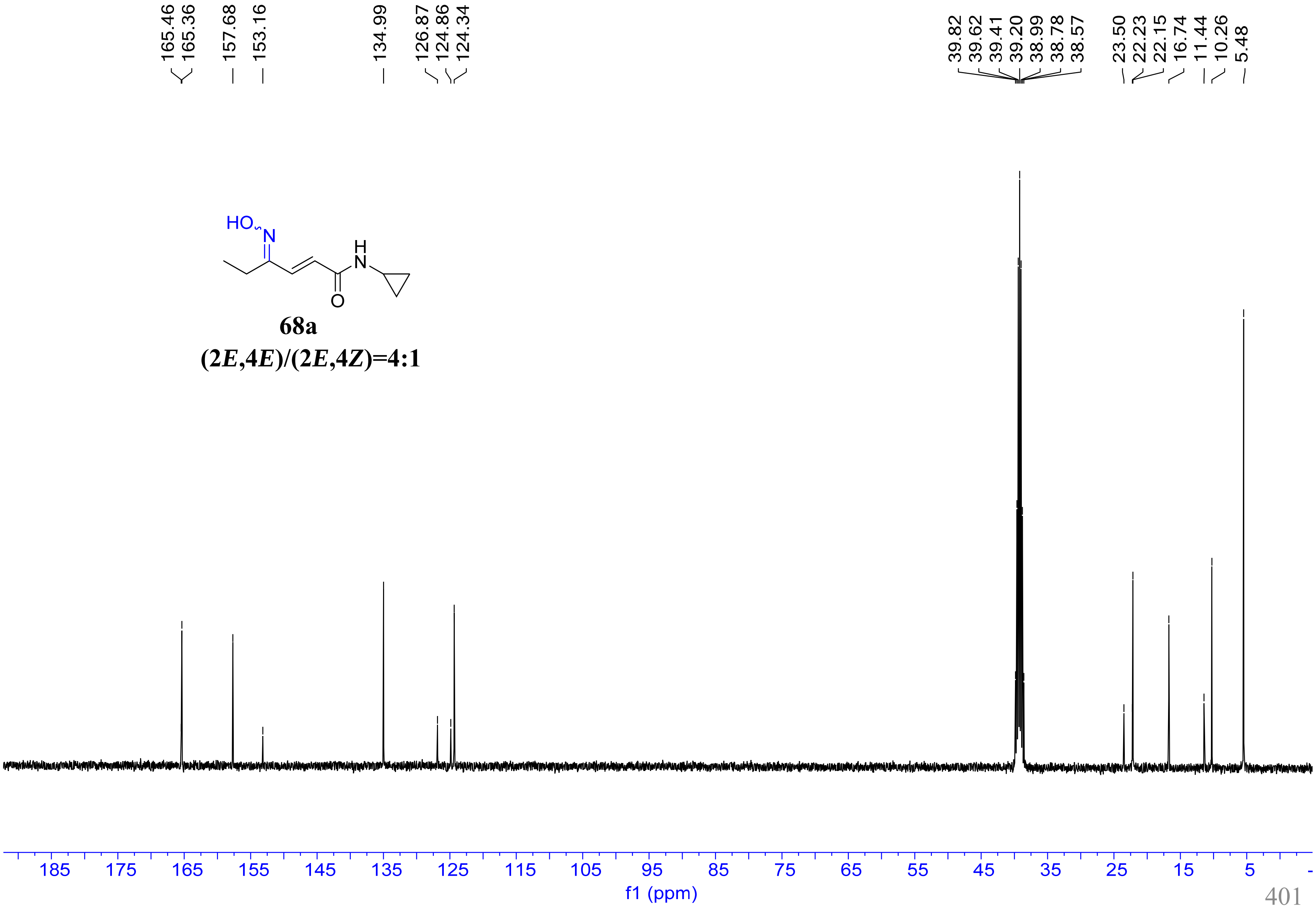

68a

(2*E*,4*E*)/(2*E*,4*Z*)=4:1

¹H NMR, 400 MHz, $CDCl_3$

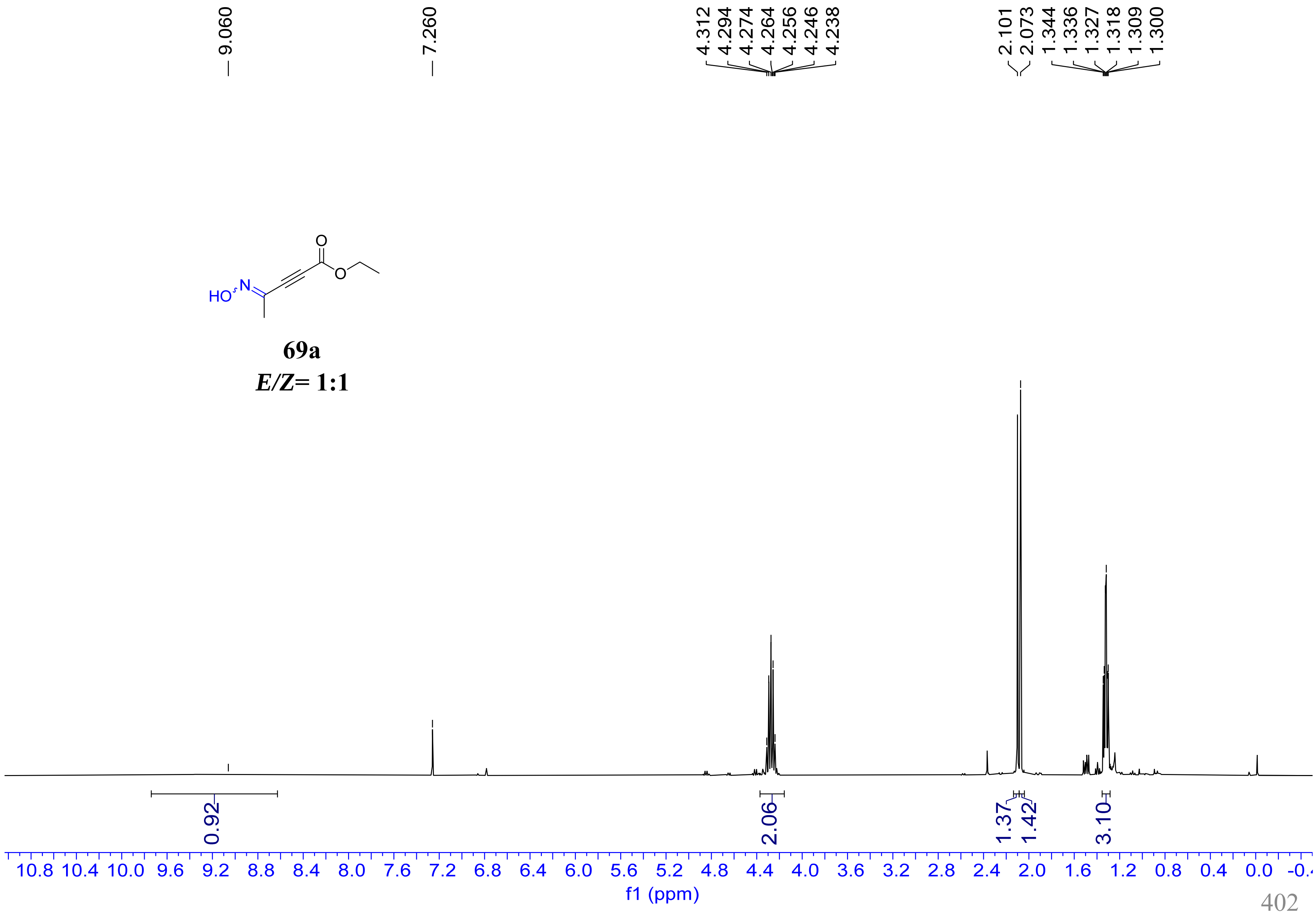

^{13}C NMR 101 MHz, $CDCl_3$

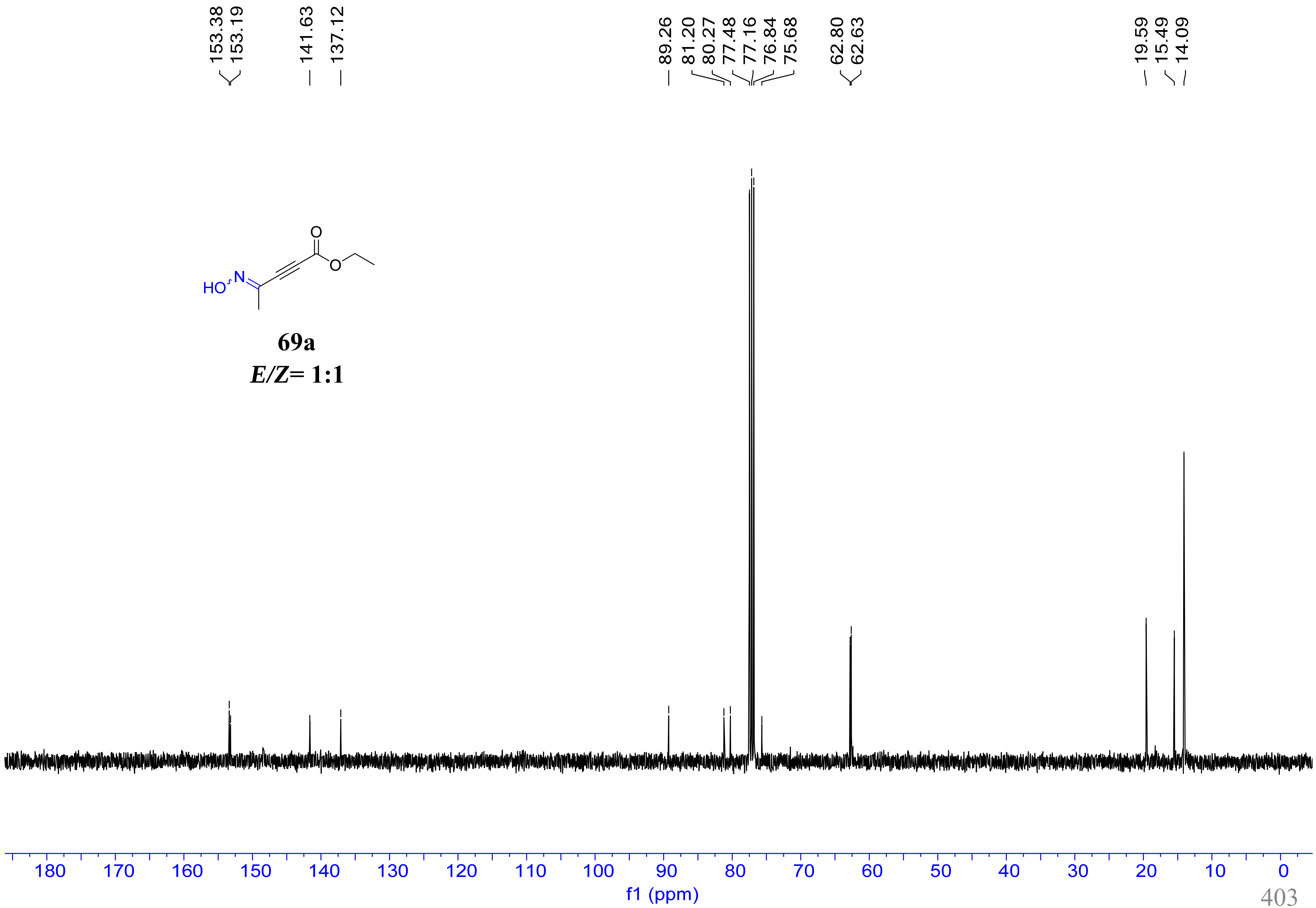

^{1}H NMR, 400 MHz, $CDCl_3$

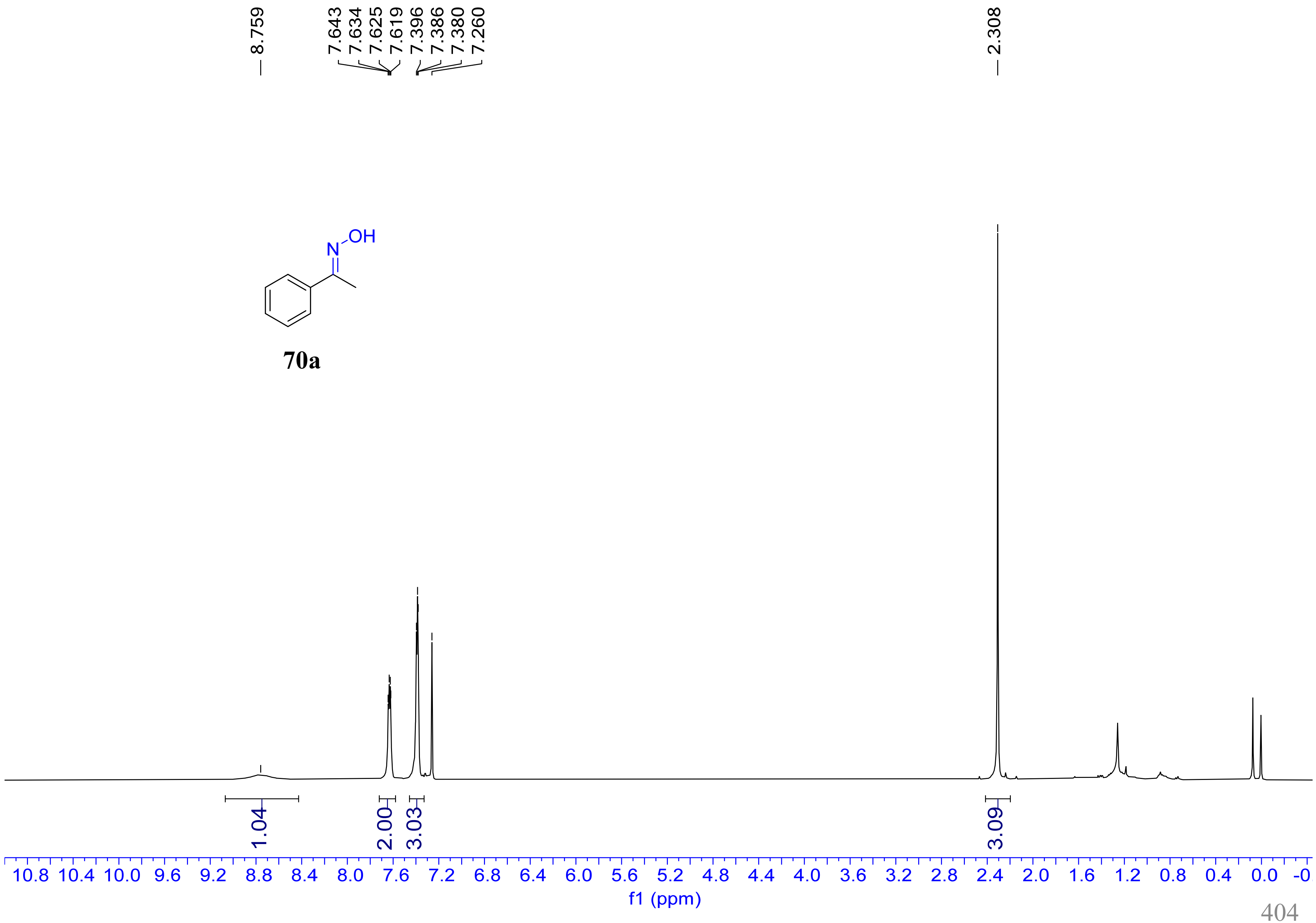

^{13}C NMR 101 MHz, $CDCl_3$

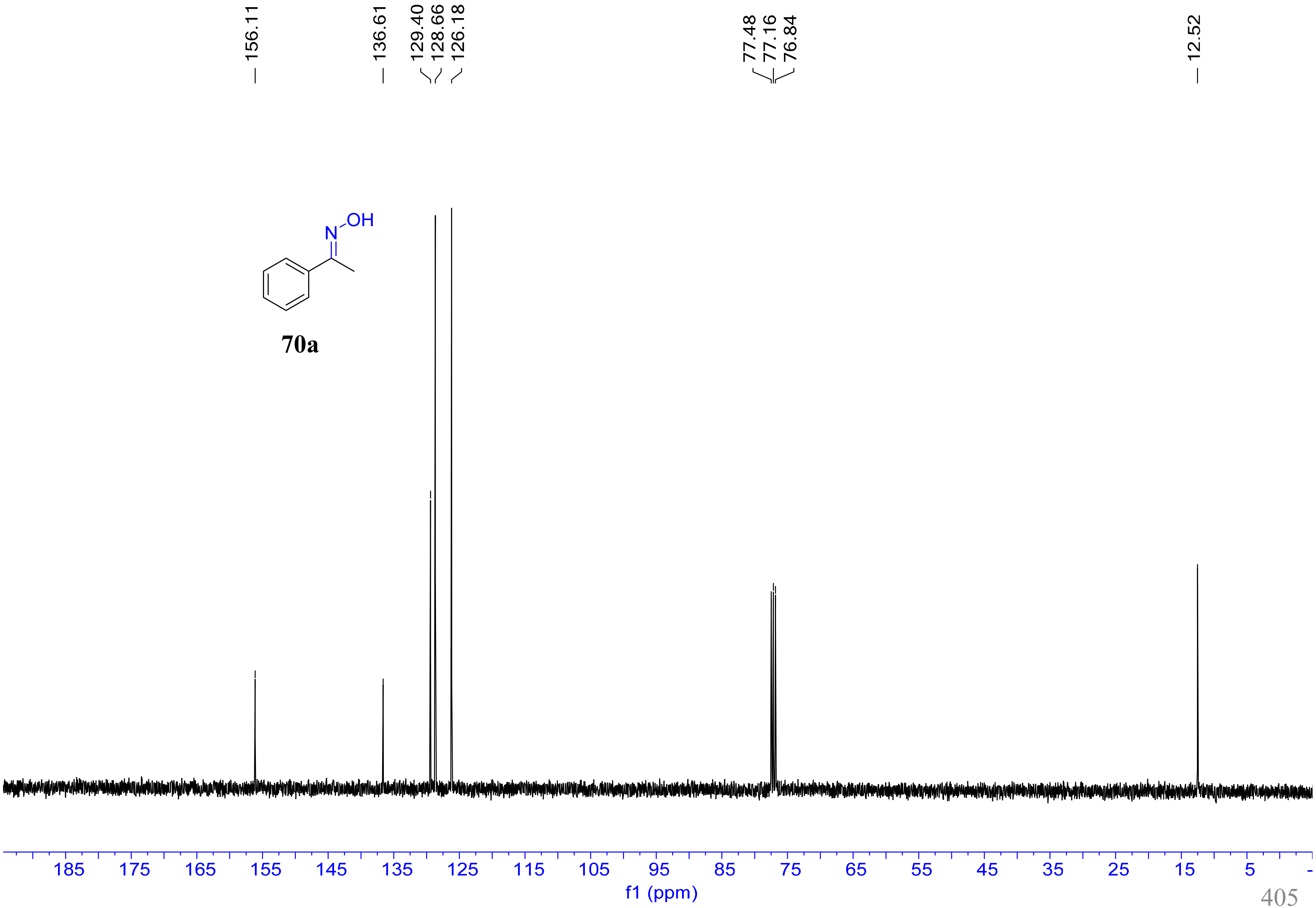

^{1}H NMR, 400 MHz, $CDCl_3$

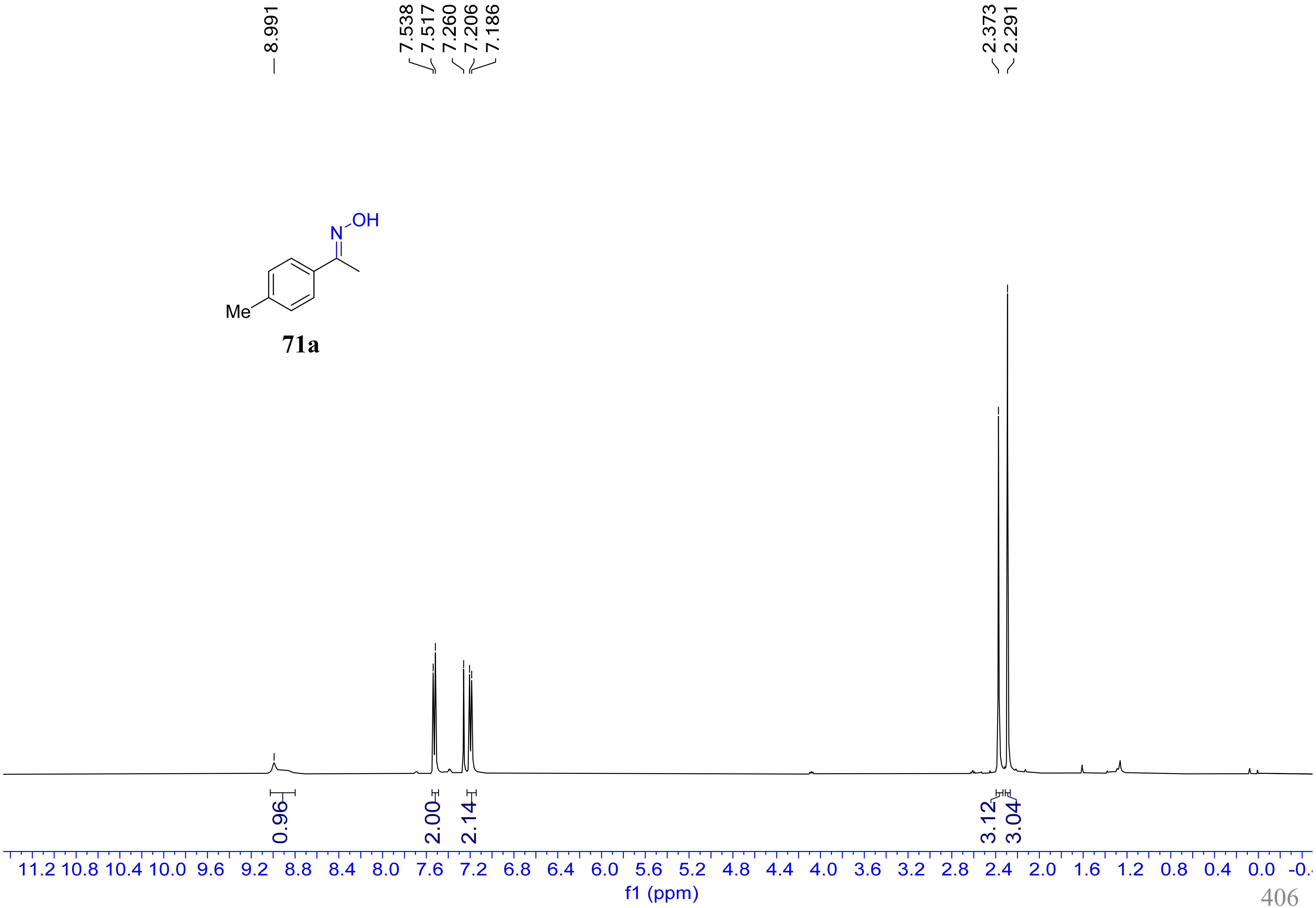

^{13}C NMR 101 MHz, $CDCl_3$

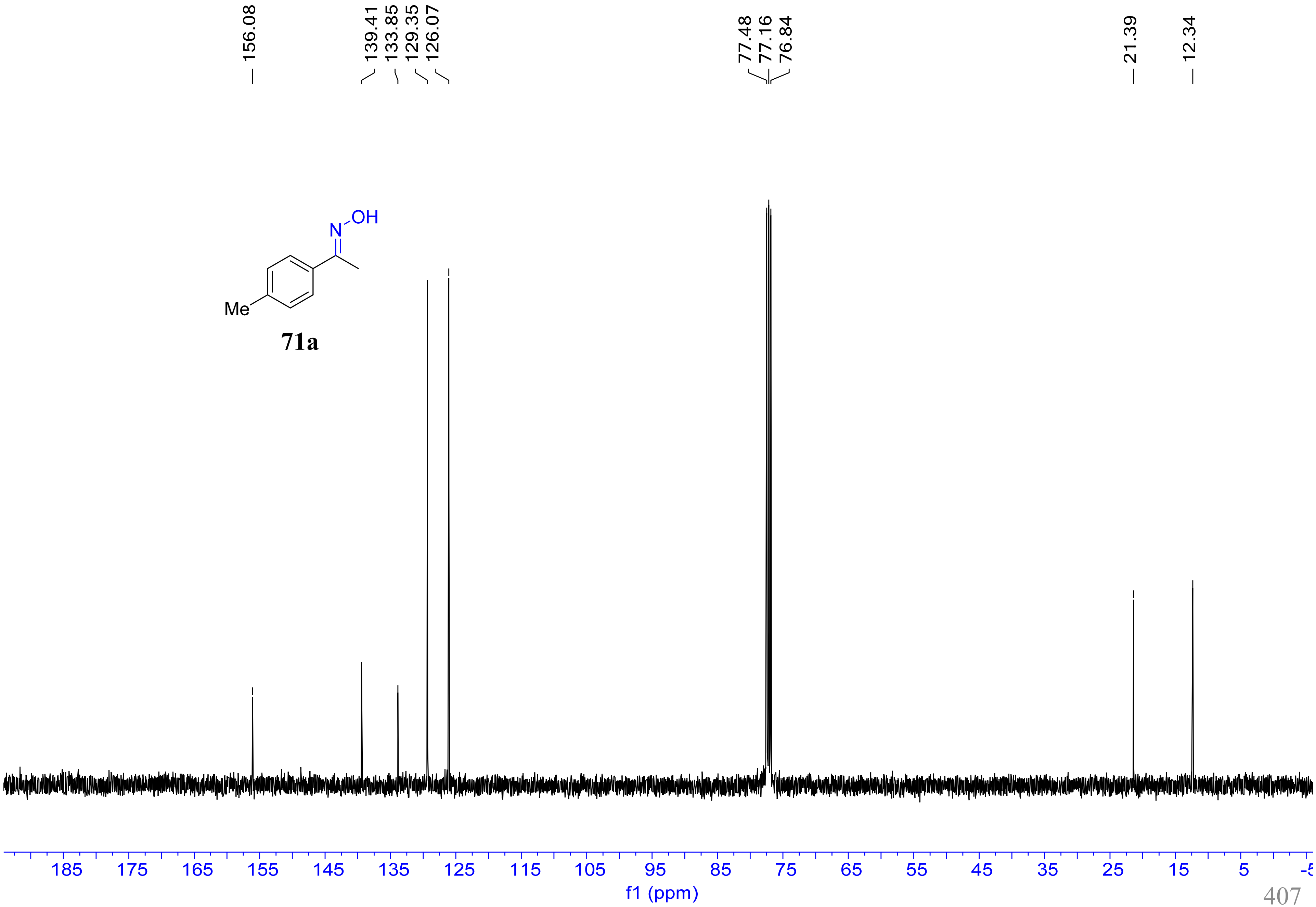

71a

^{1}H NMR, 400 MHz, CDCl$_3$

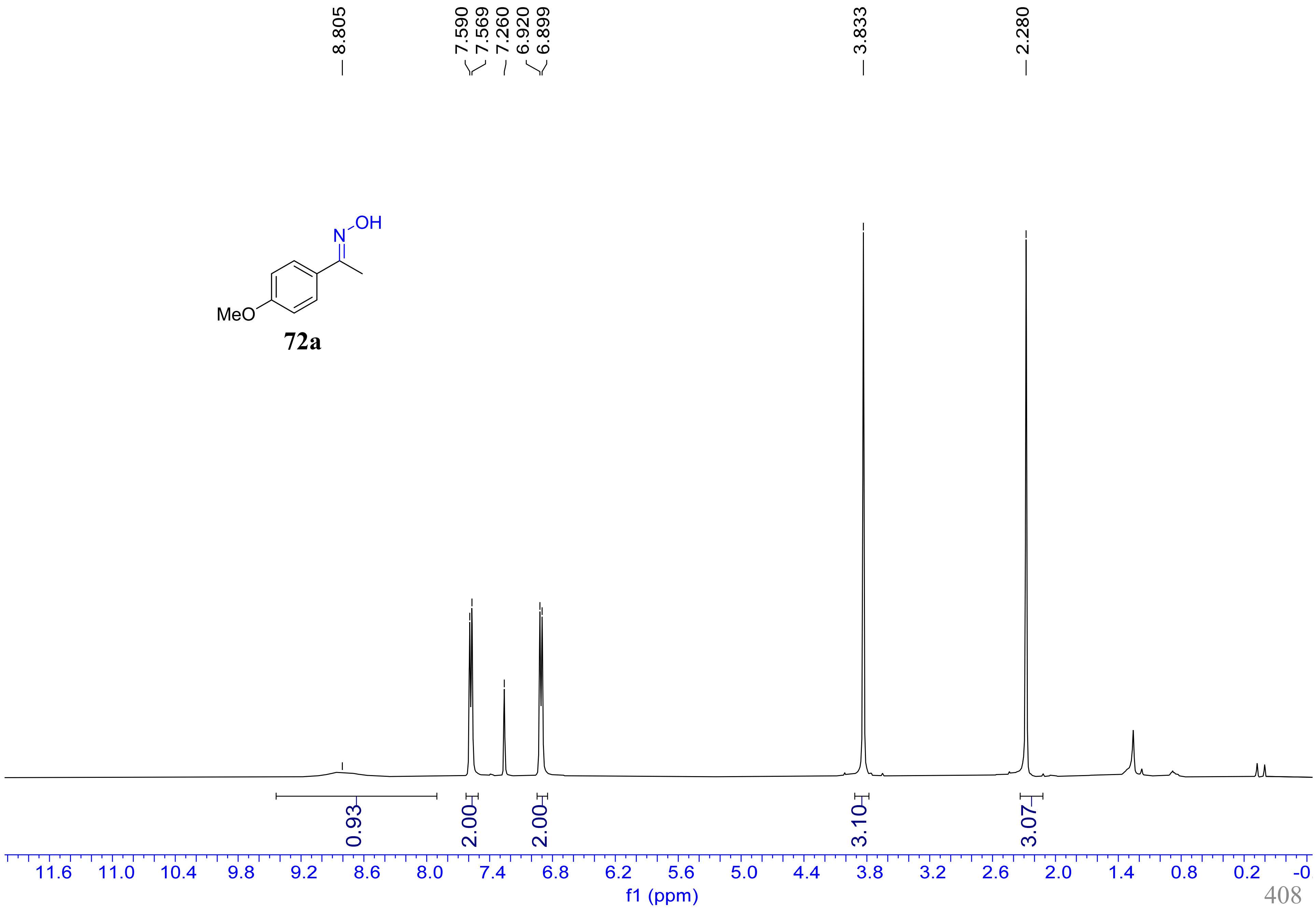

^{13}C NMR 101 MHz, $CDCl_3$

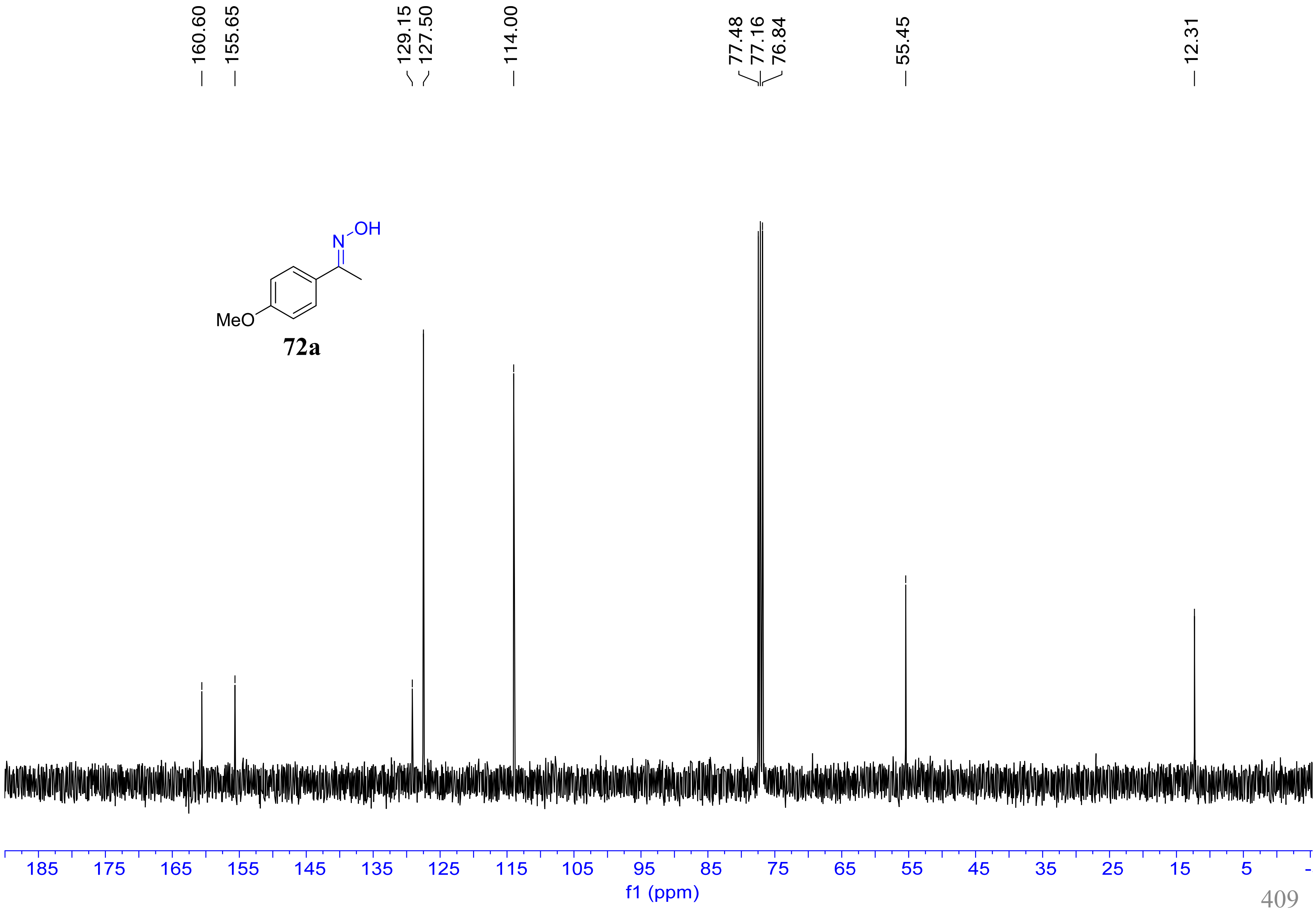

^{1}H NMR, 400 MHz, $CDCl_3$

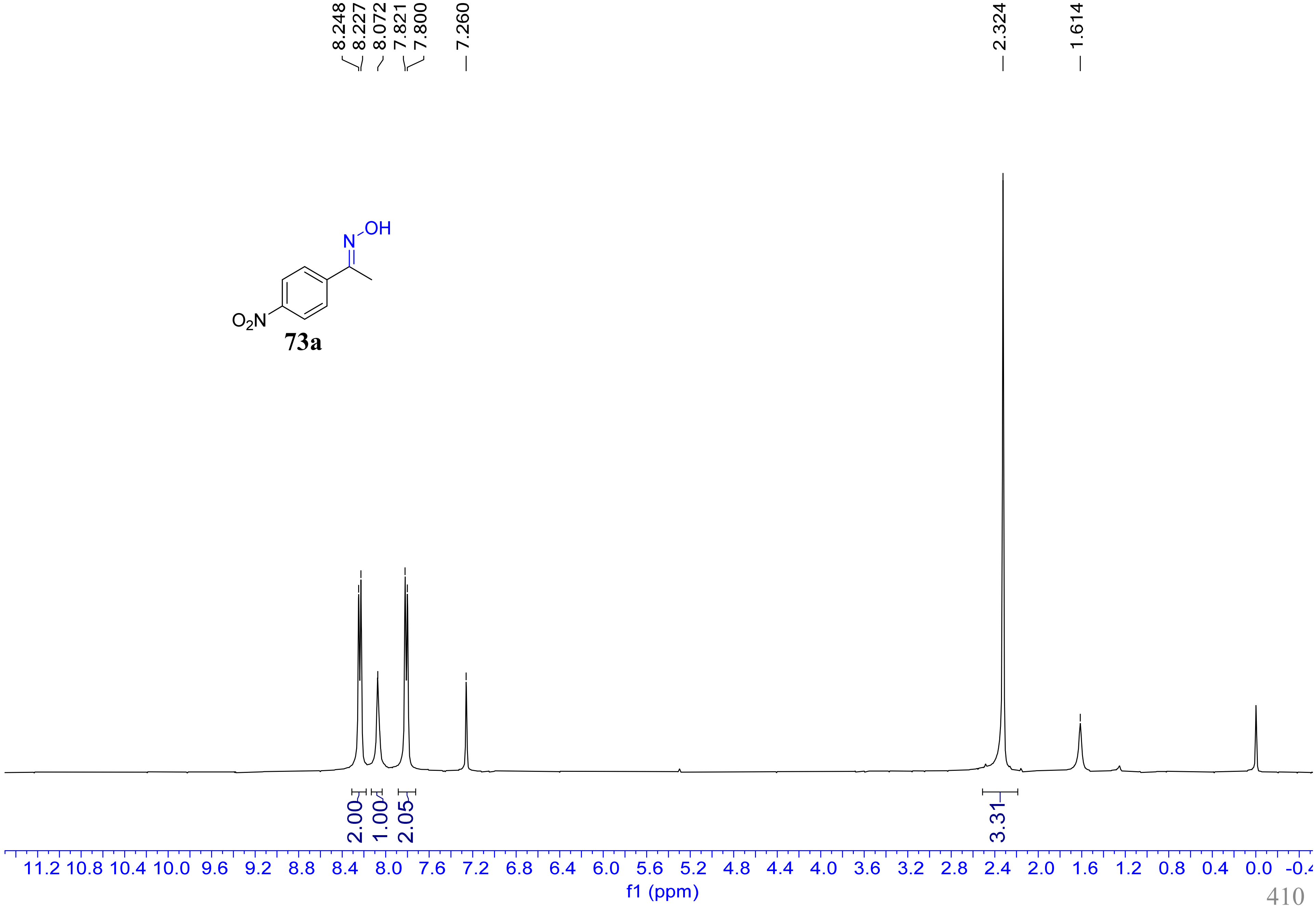

^{13}C NMR 101 MHz, CD_3OD

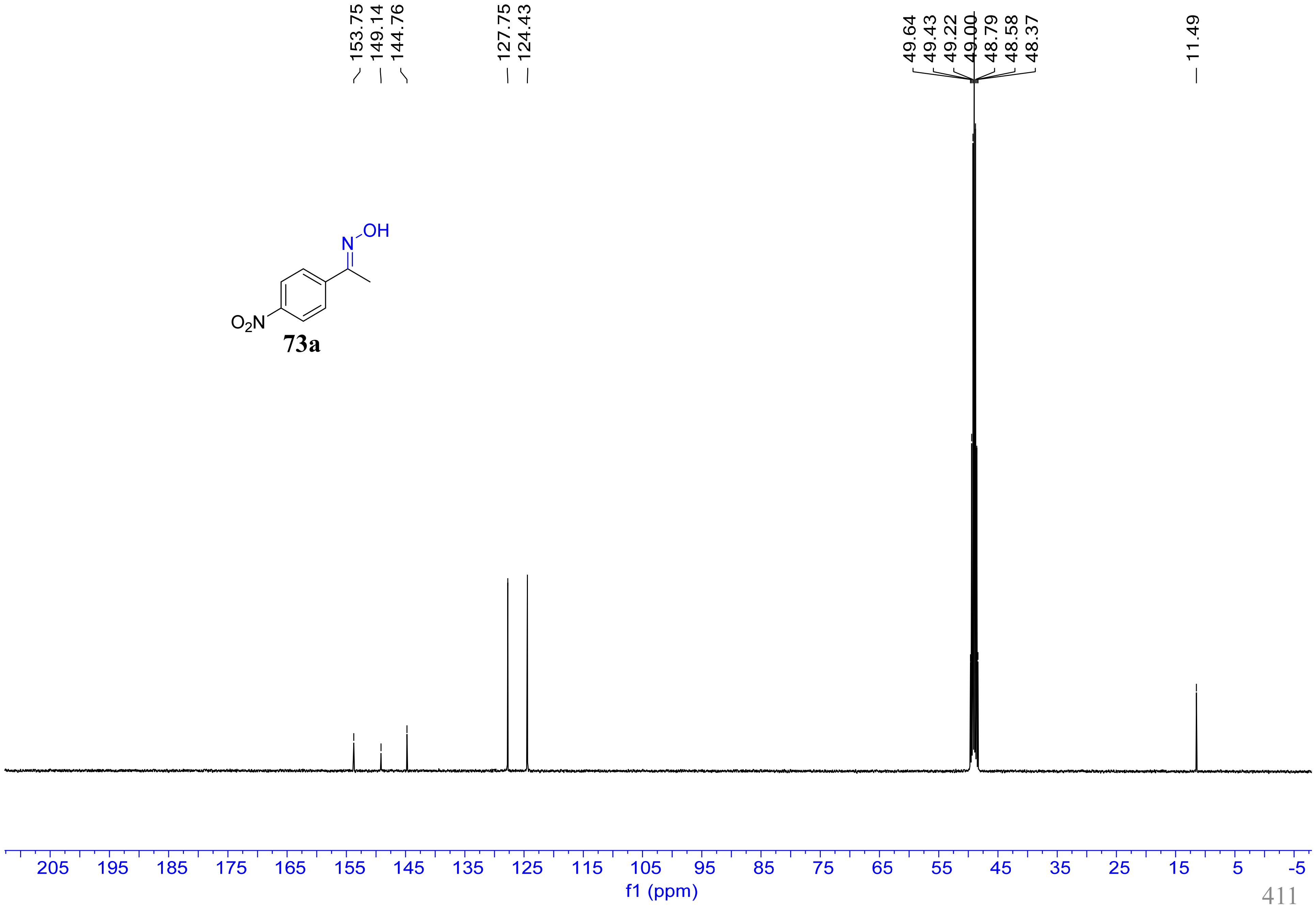

^{1}H NMR, 400 MHz, $CDCl_3$

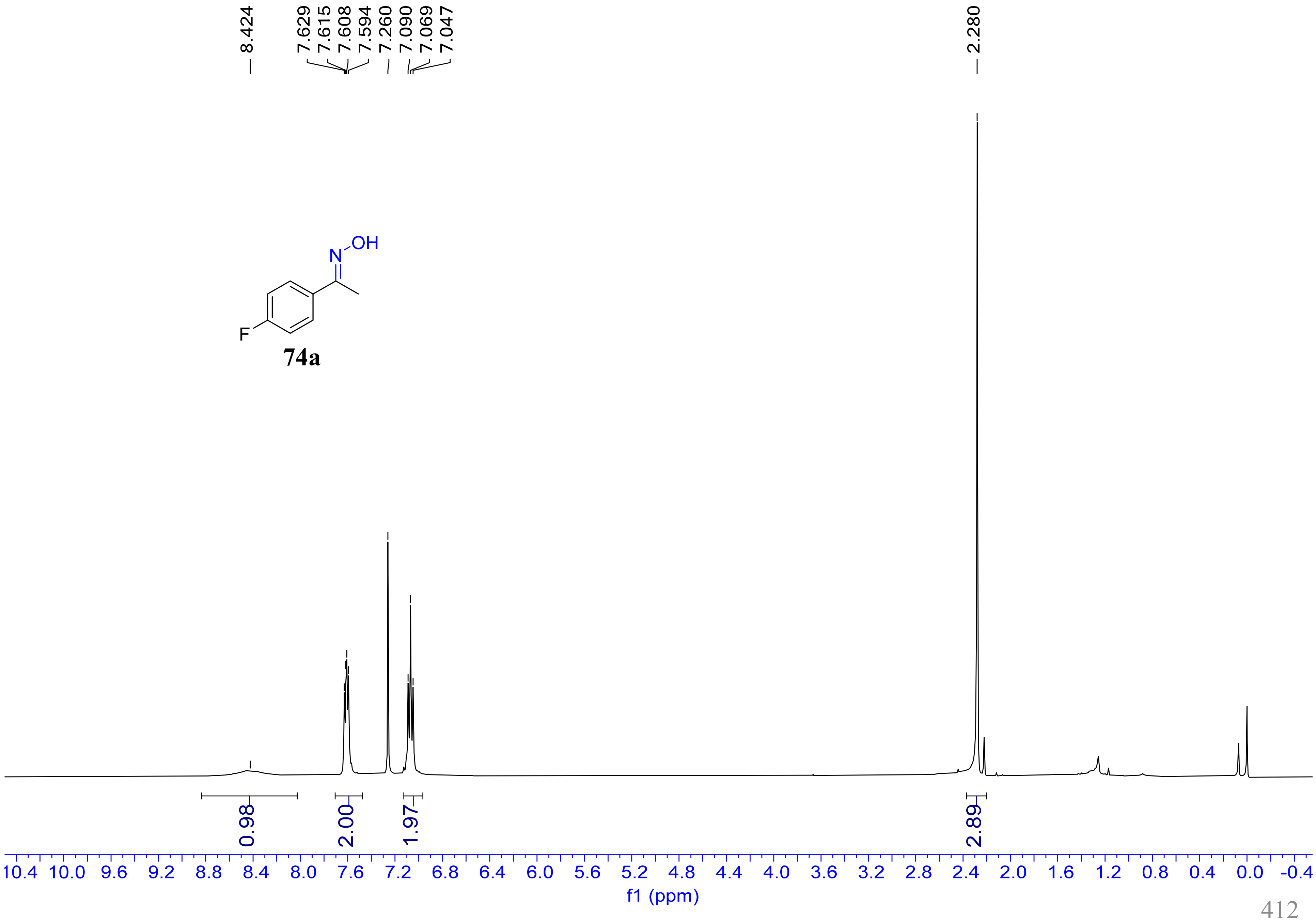

^{13}C NMR 101 MHz, $CDCl_3$

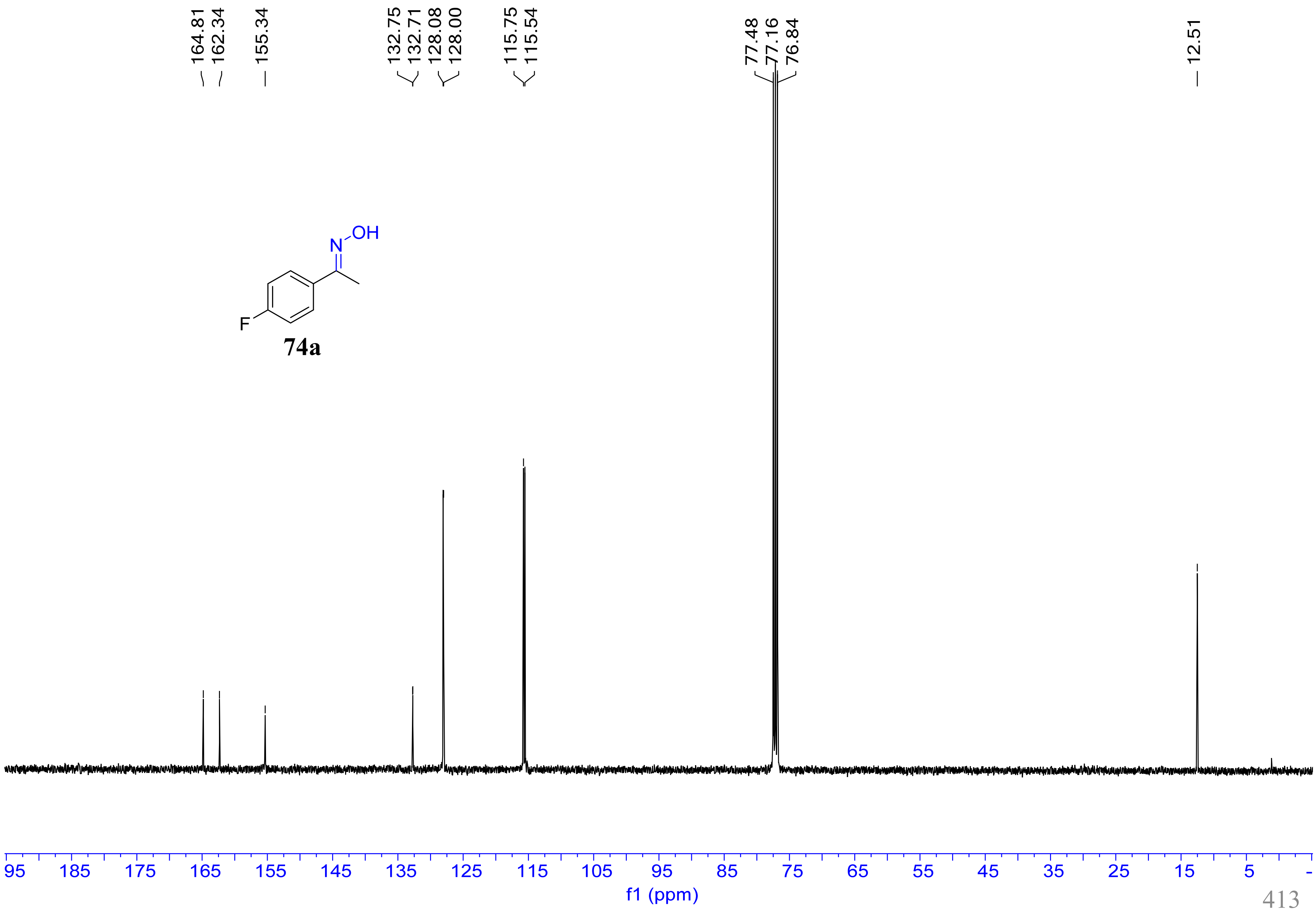

^{19}F NMR 376 MHz, $CDCl_3$

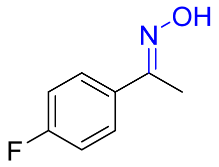

74a

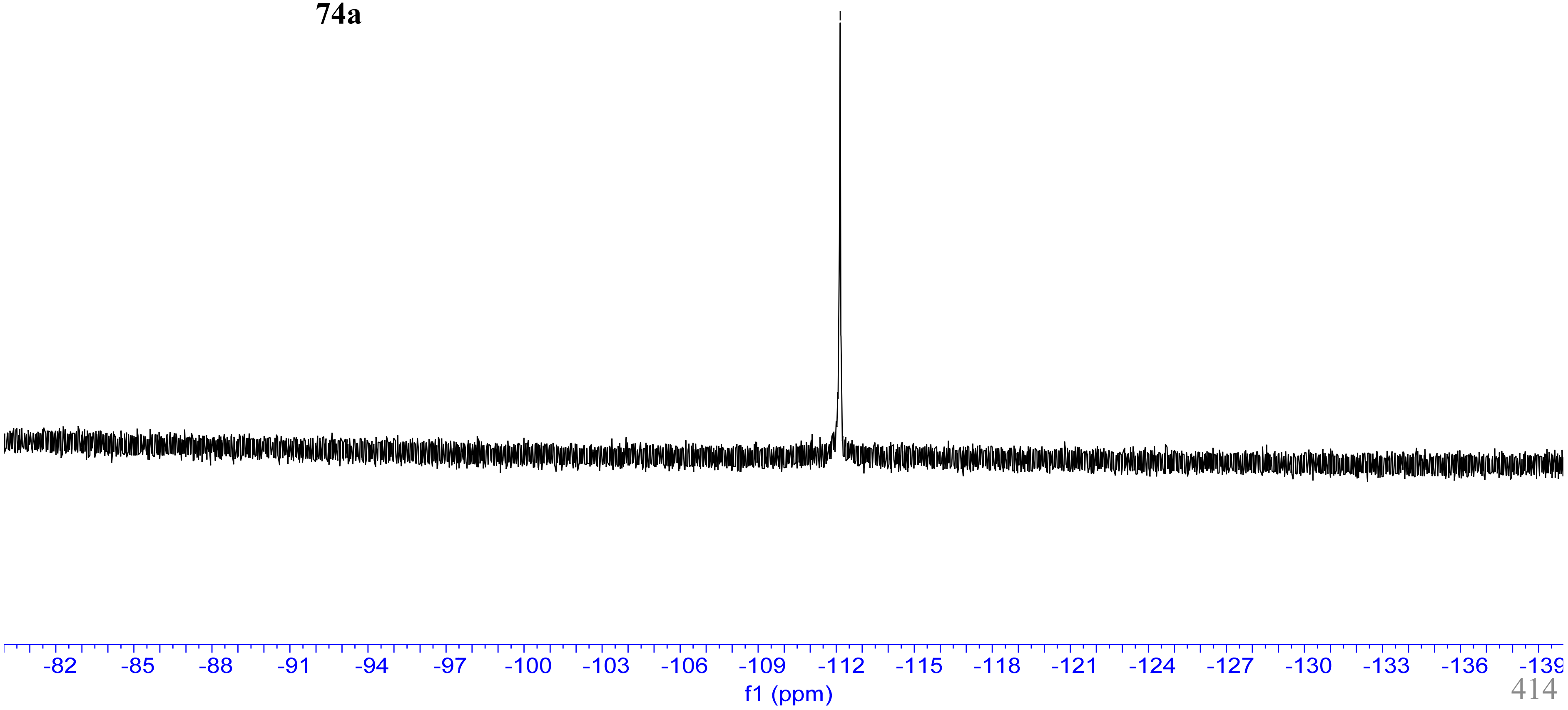

^{1}H NMR, 400 MHz, $CDCl_3$

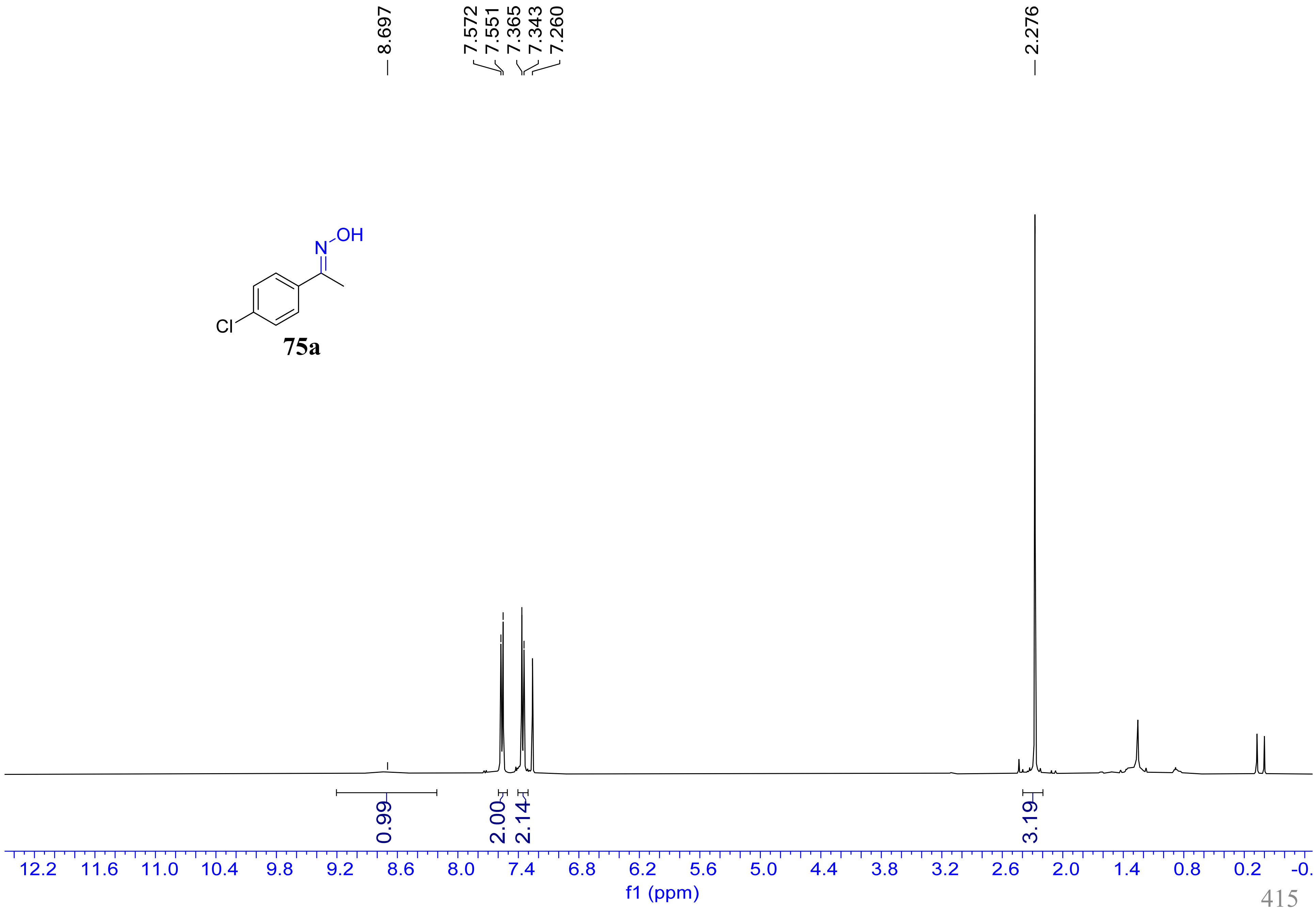

^{13}C NMR 101 MHz, $CDCl_3$

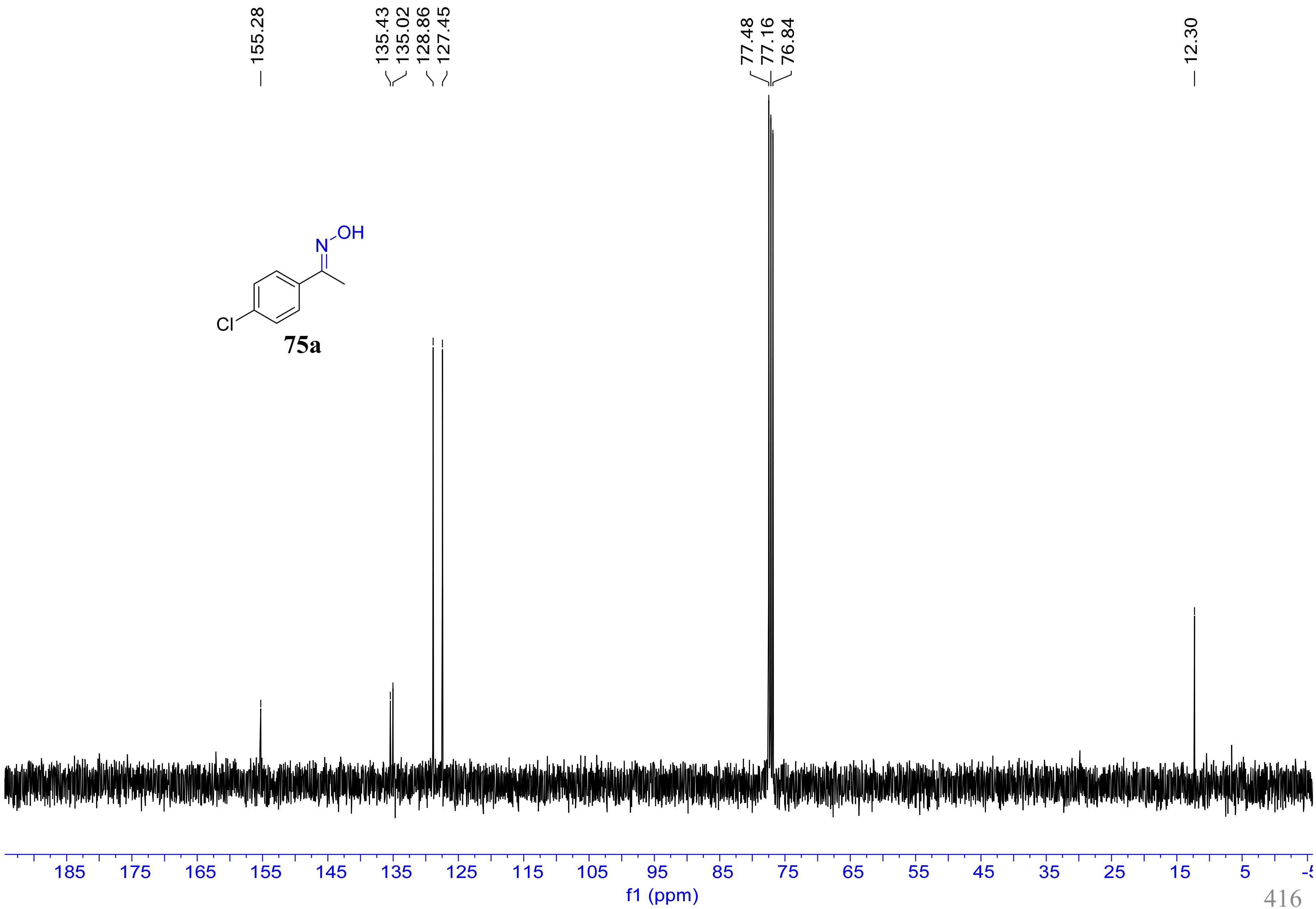

^{1}H NMR, 400 MHz, $CDCl_3$

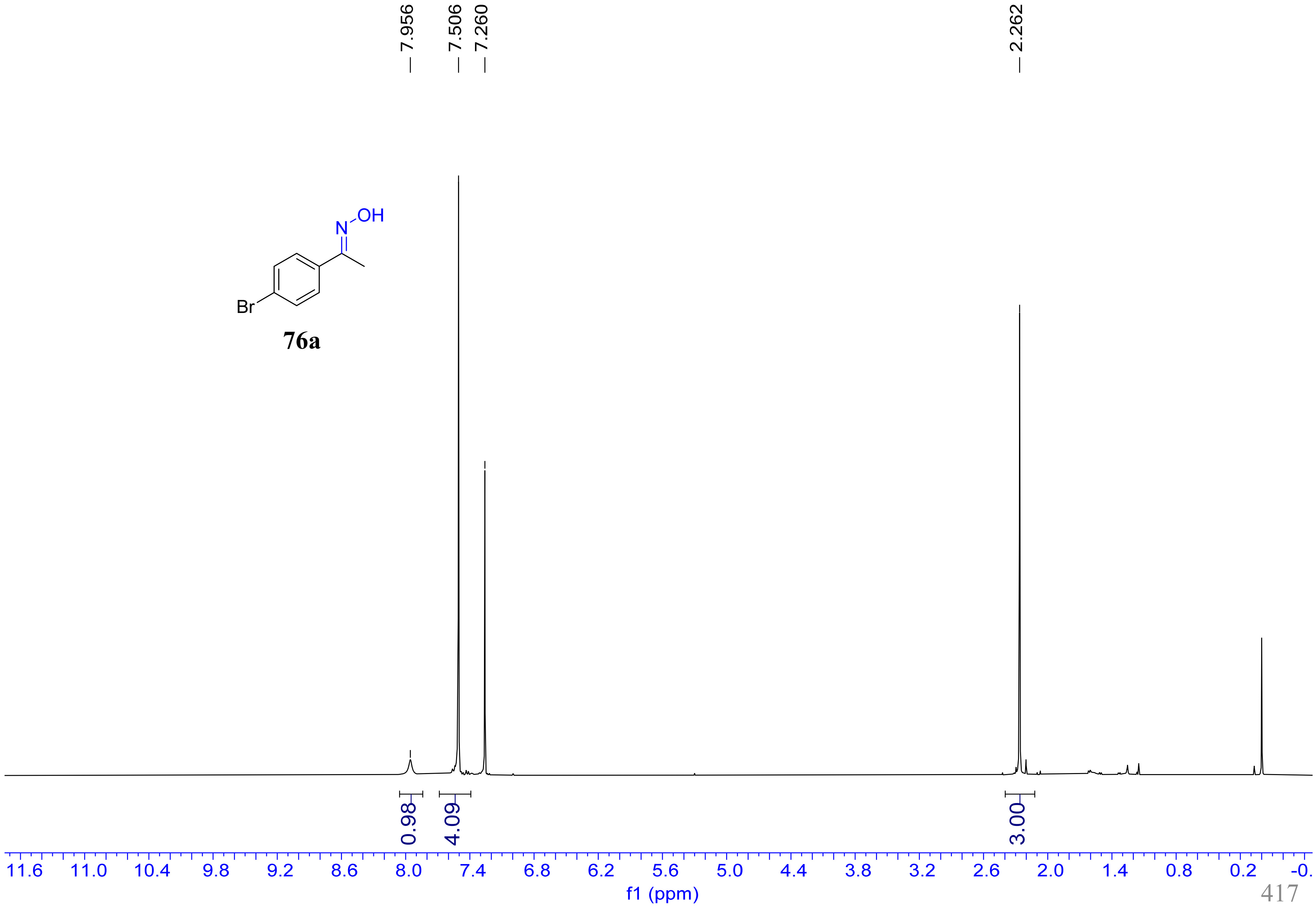

76a

^{13}C NMR 101 MHz, $CDCl_3$

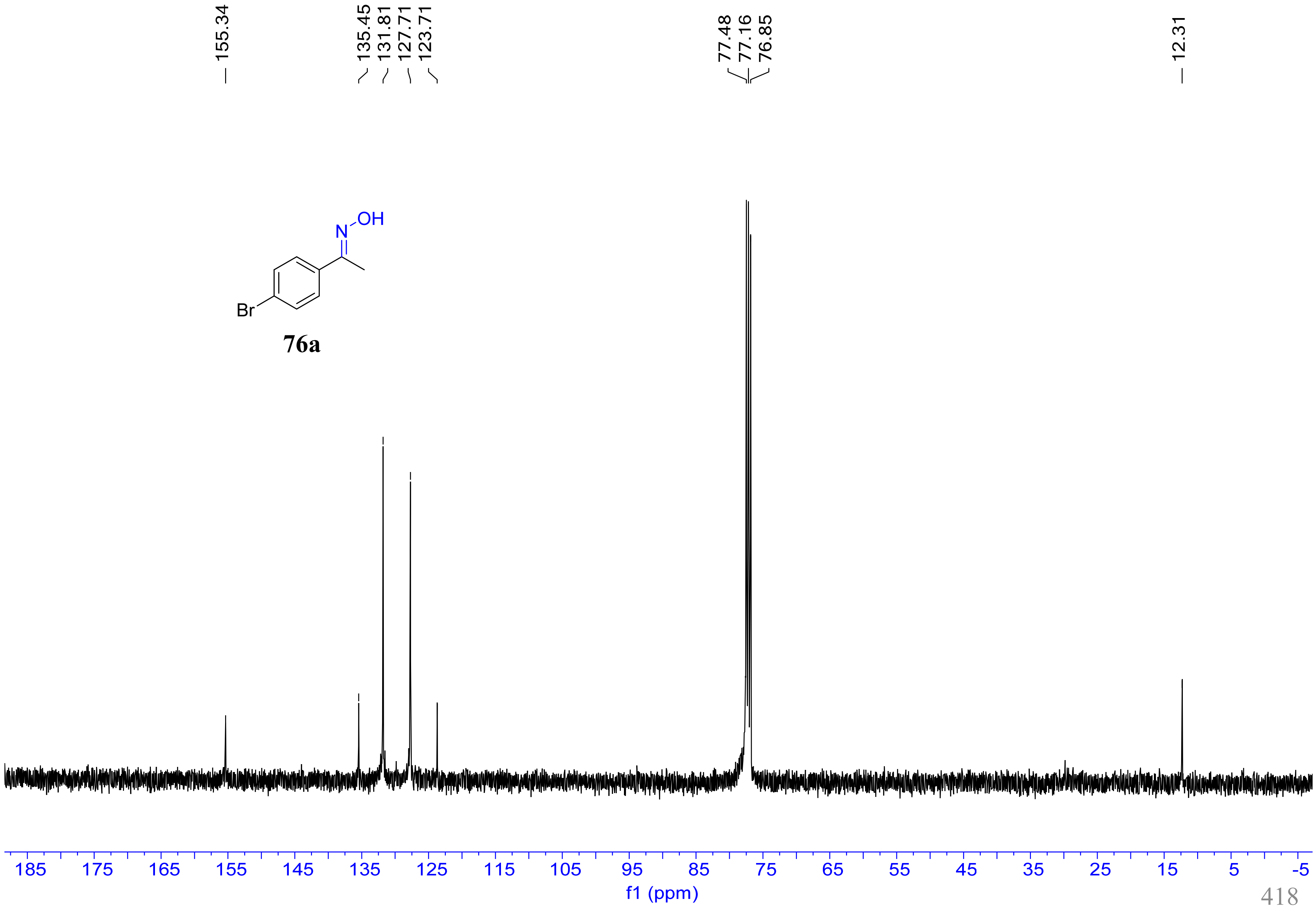

76a

^{1}H NMR, 400 MHz, $CDCl_3$

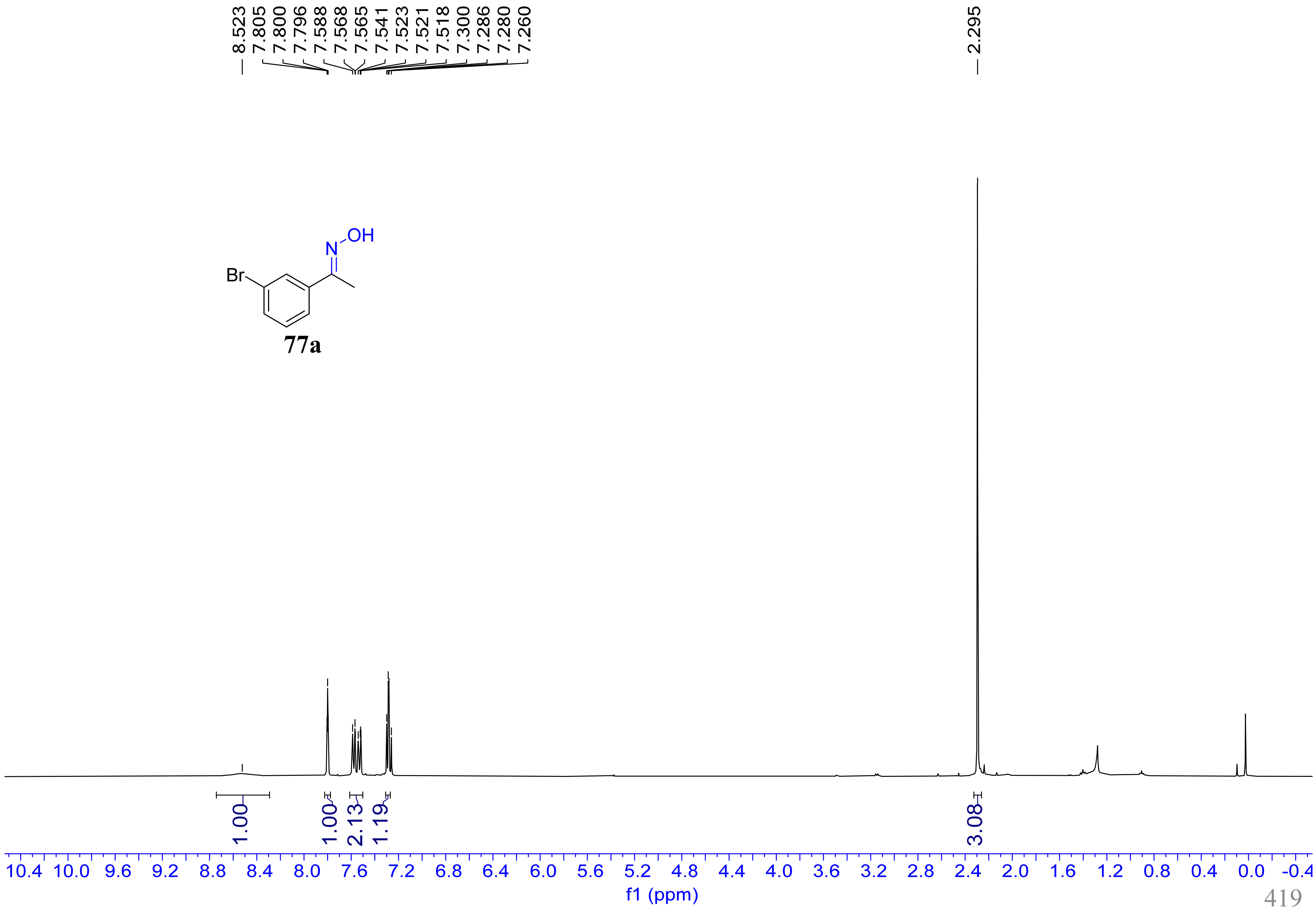

^{13}C NMR 101 MHz, $CDCl_3$

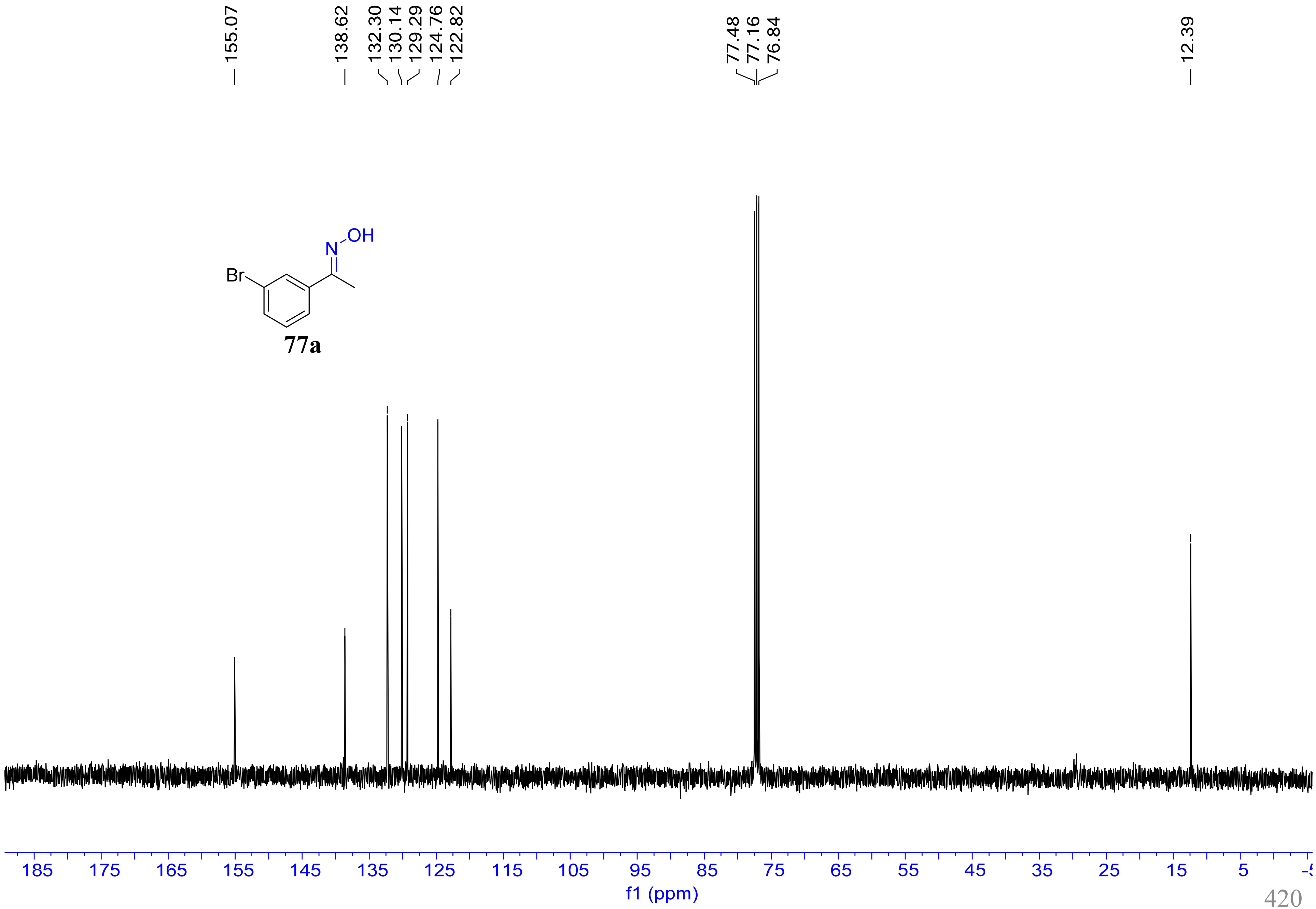

^{1}H NMR, 400 MHz, CDCl$_3$

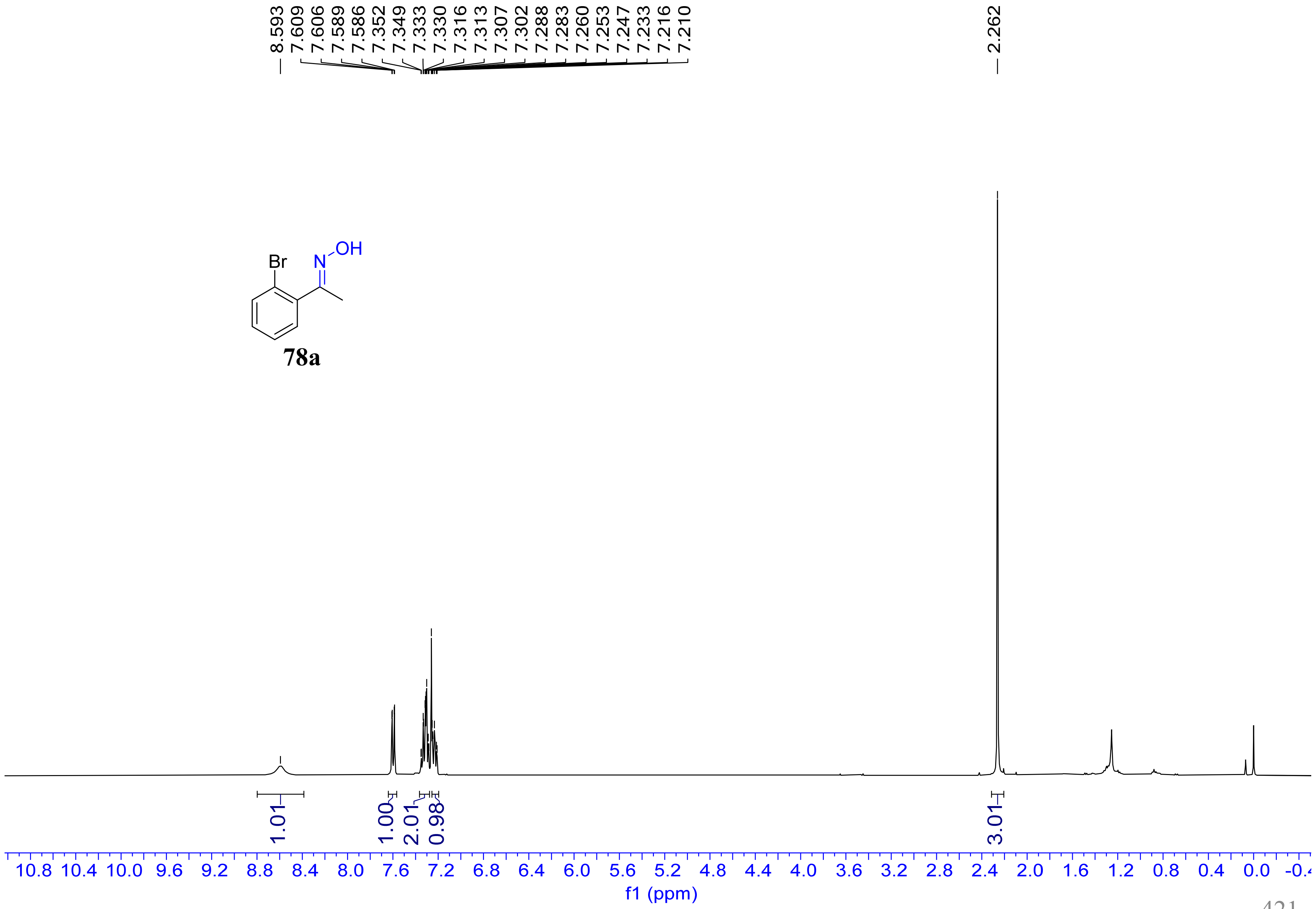

78a

^{13}C NMR 101 MHz, $CDCl_3$

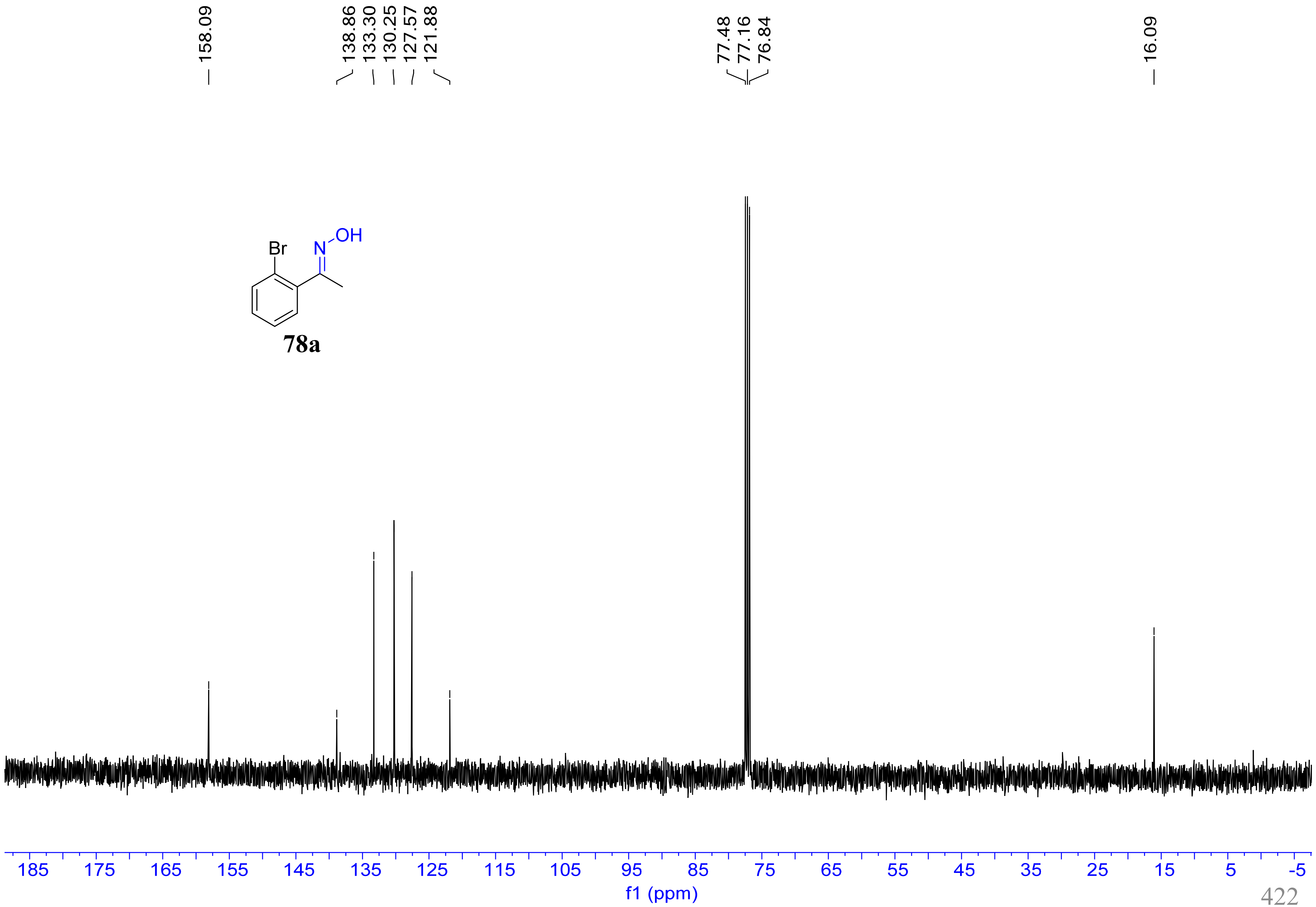

78a

^{1}H NMR, 400 MHz, $CDCl_3$

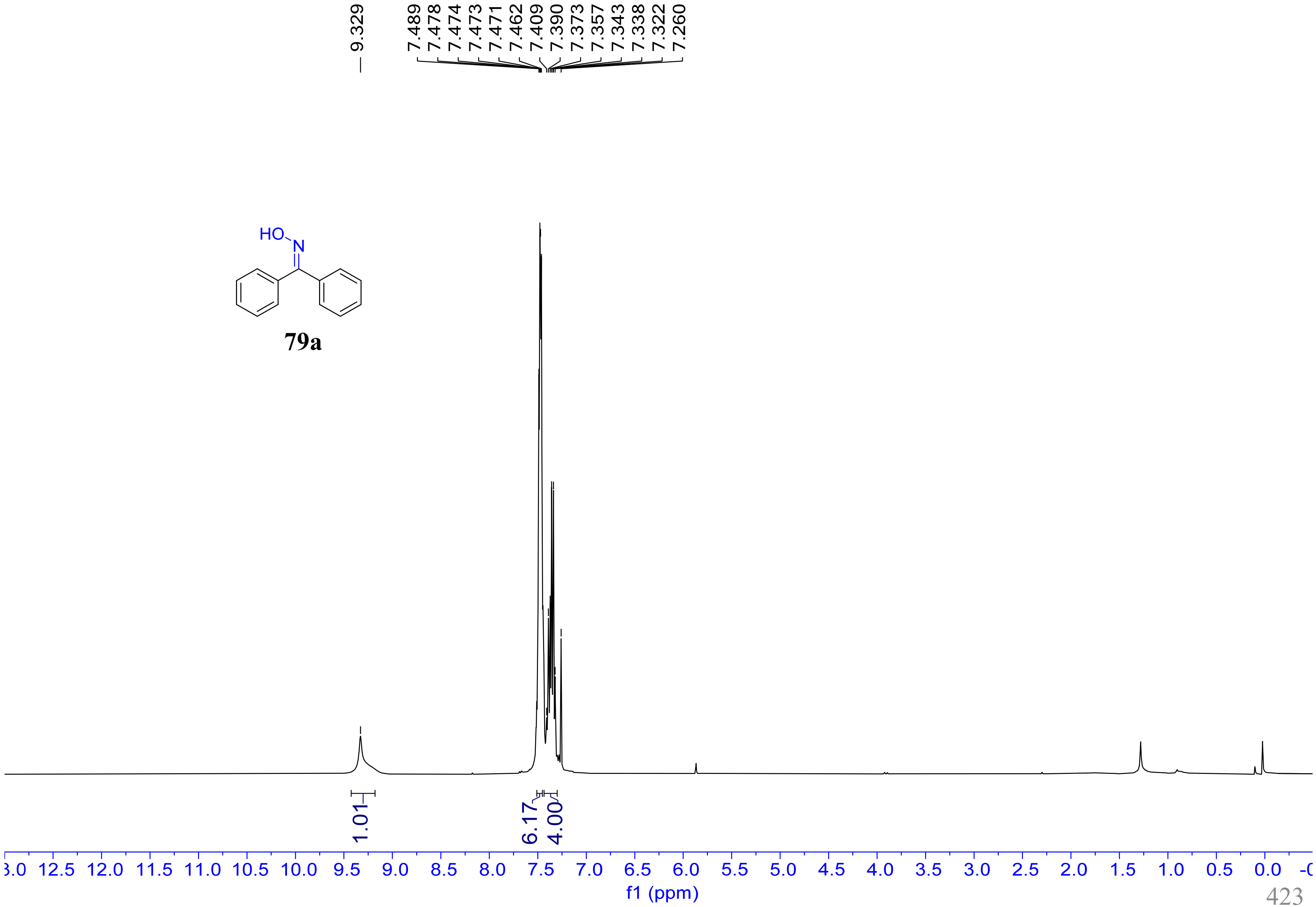

^{13}C NMR 101 MHz, $CDCl_3$

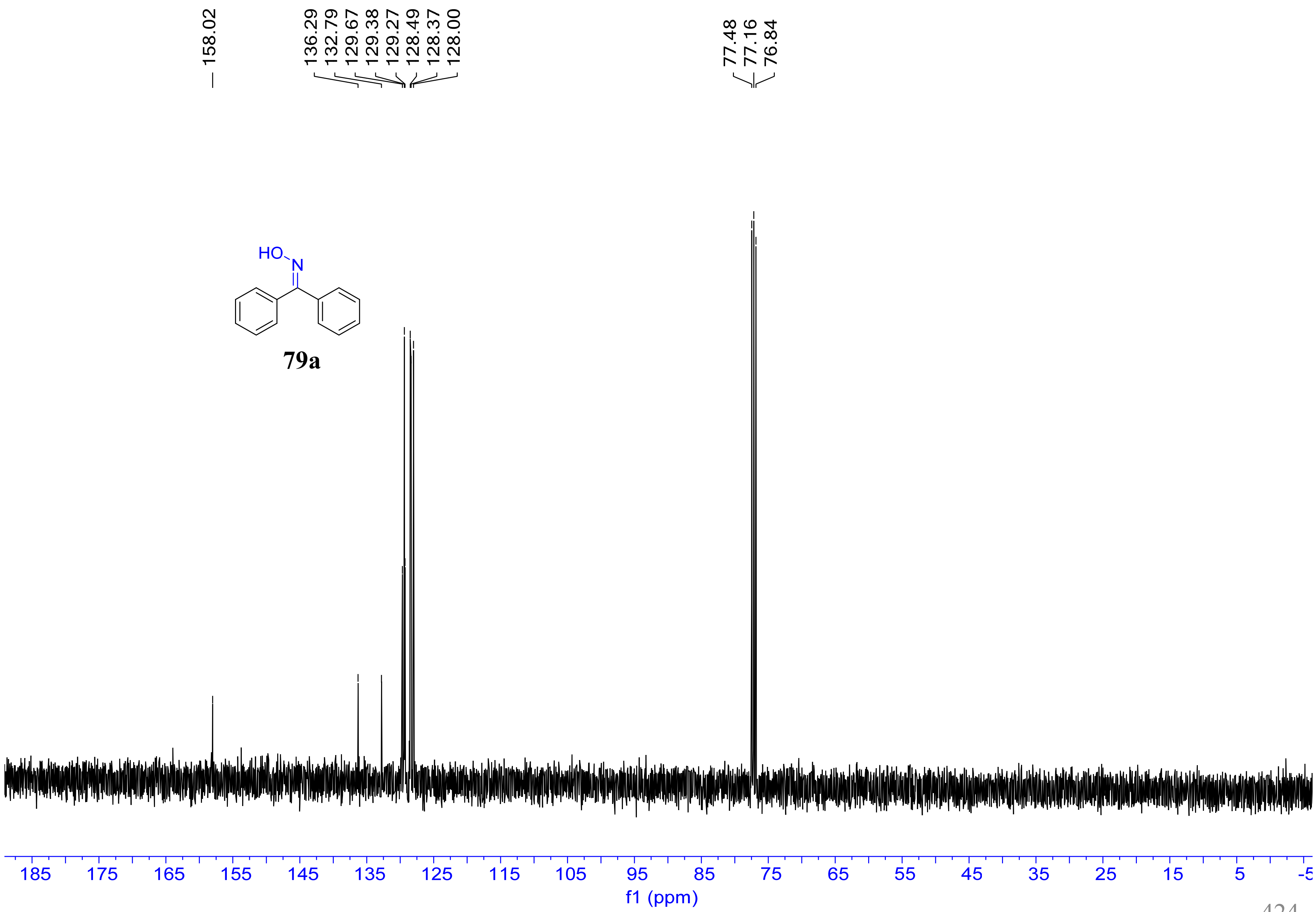

^{1}H NMR, 400 MHz, $CDCl_3$

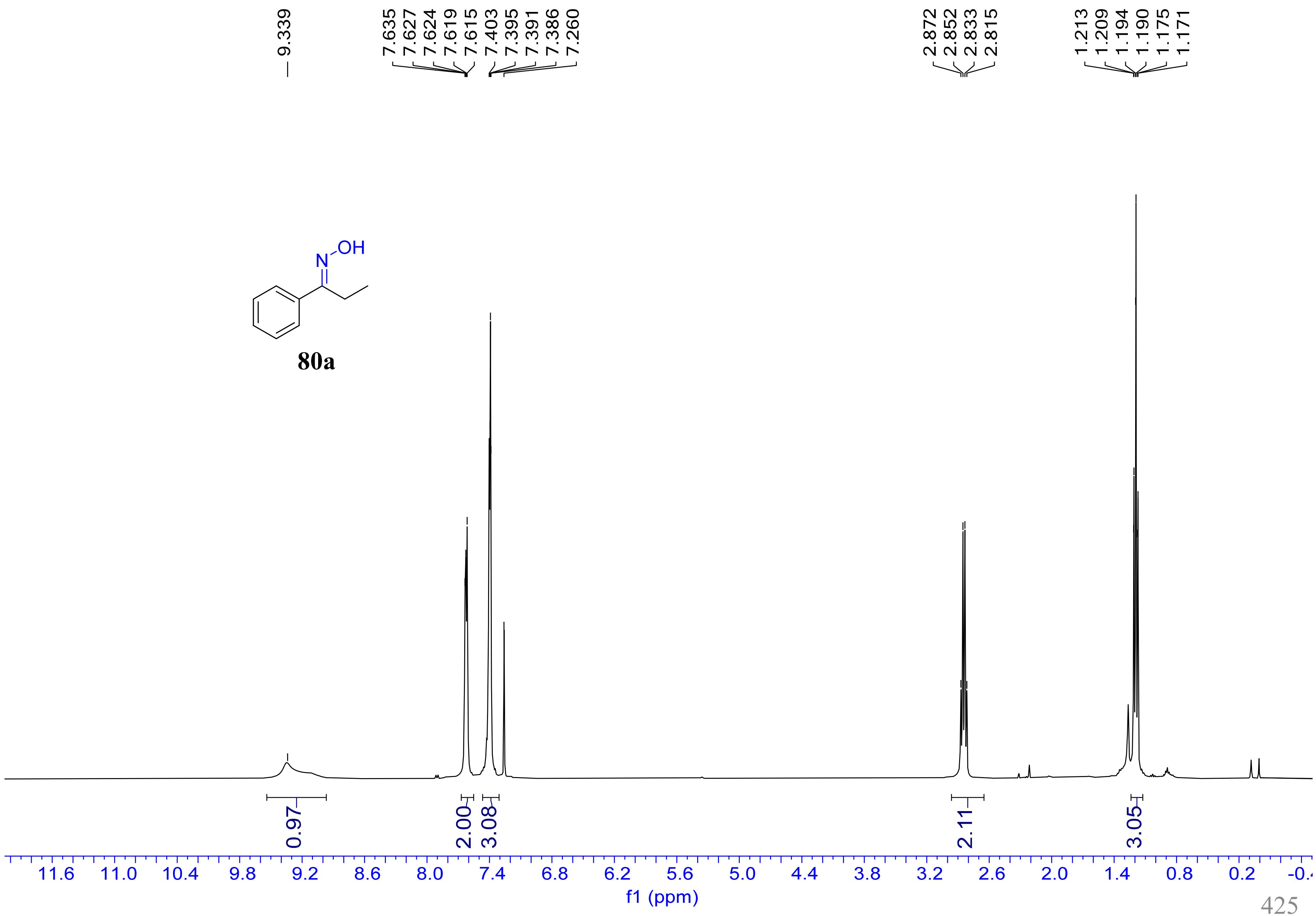

^{13}C NMR 101 MHz, $CDCl_3$

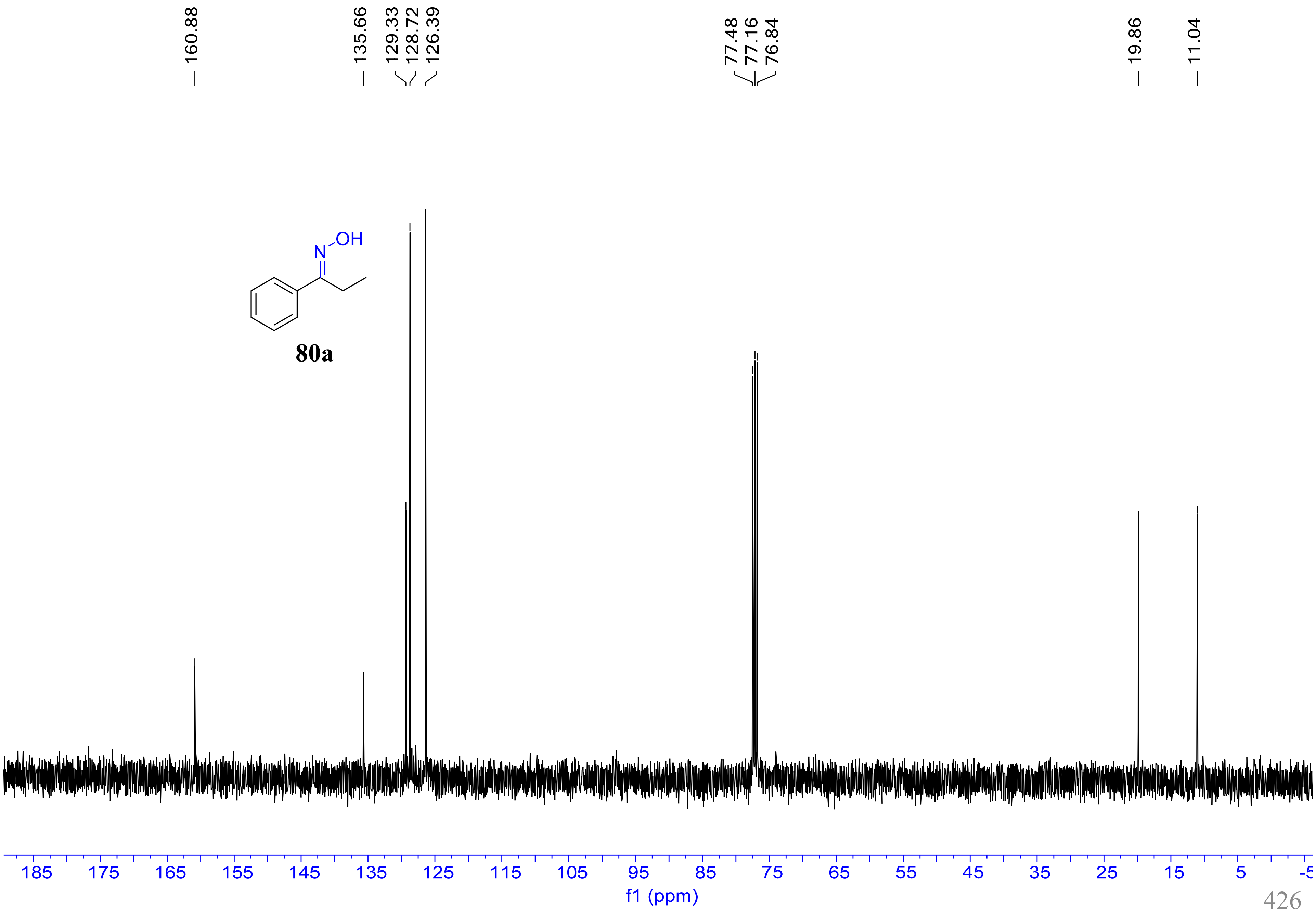

80a

^{1}H NMR, 400 MHz, $CDCl_3$

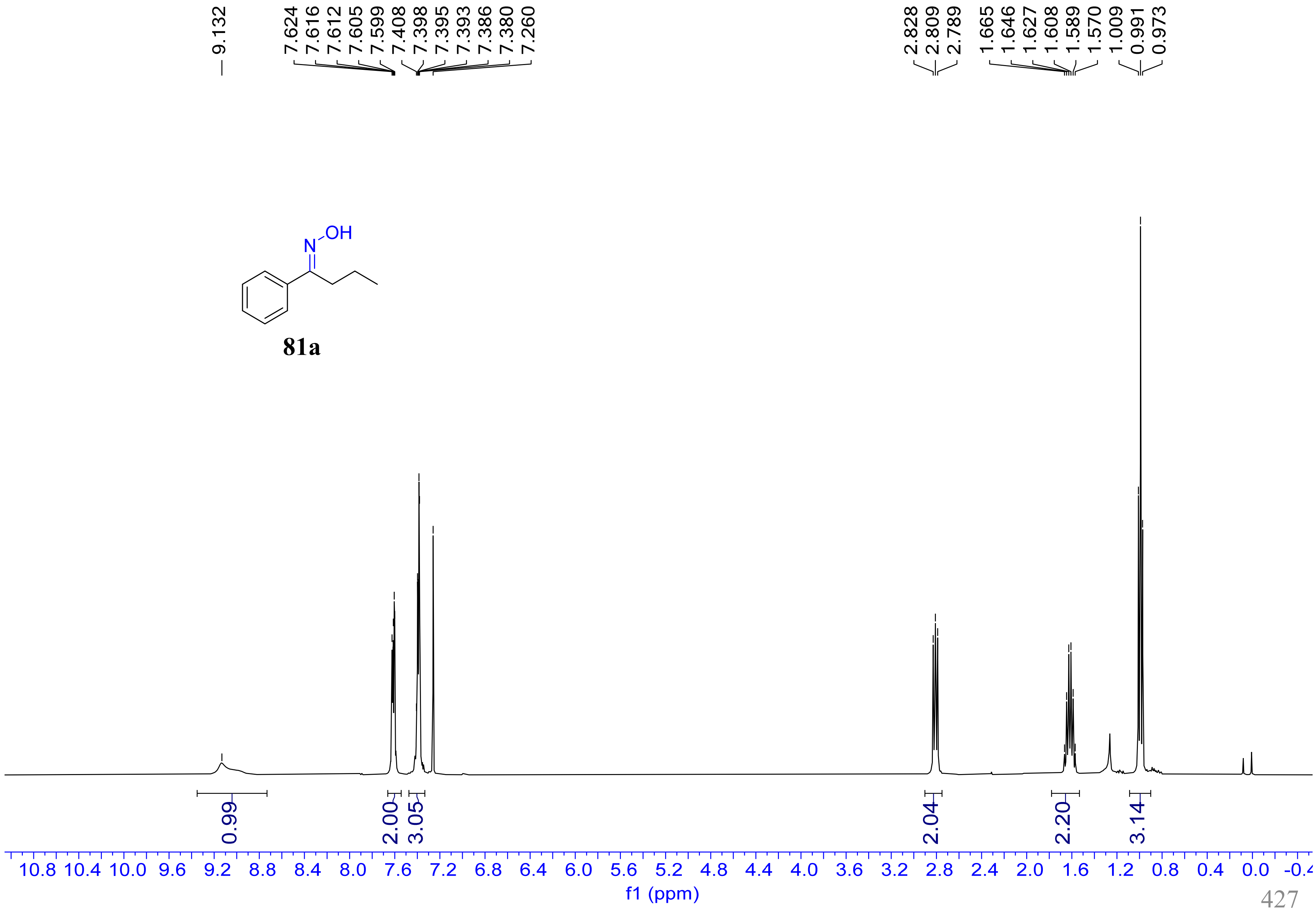

81a

^{13}C NMR 101 MHz, $CDCl_3$

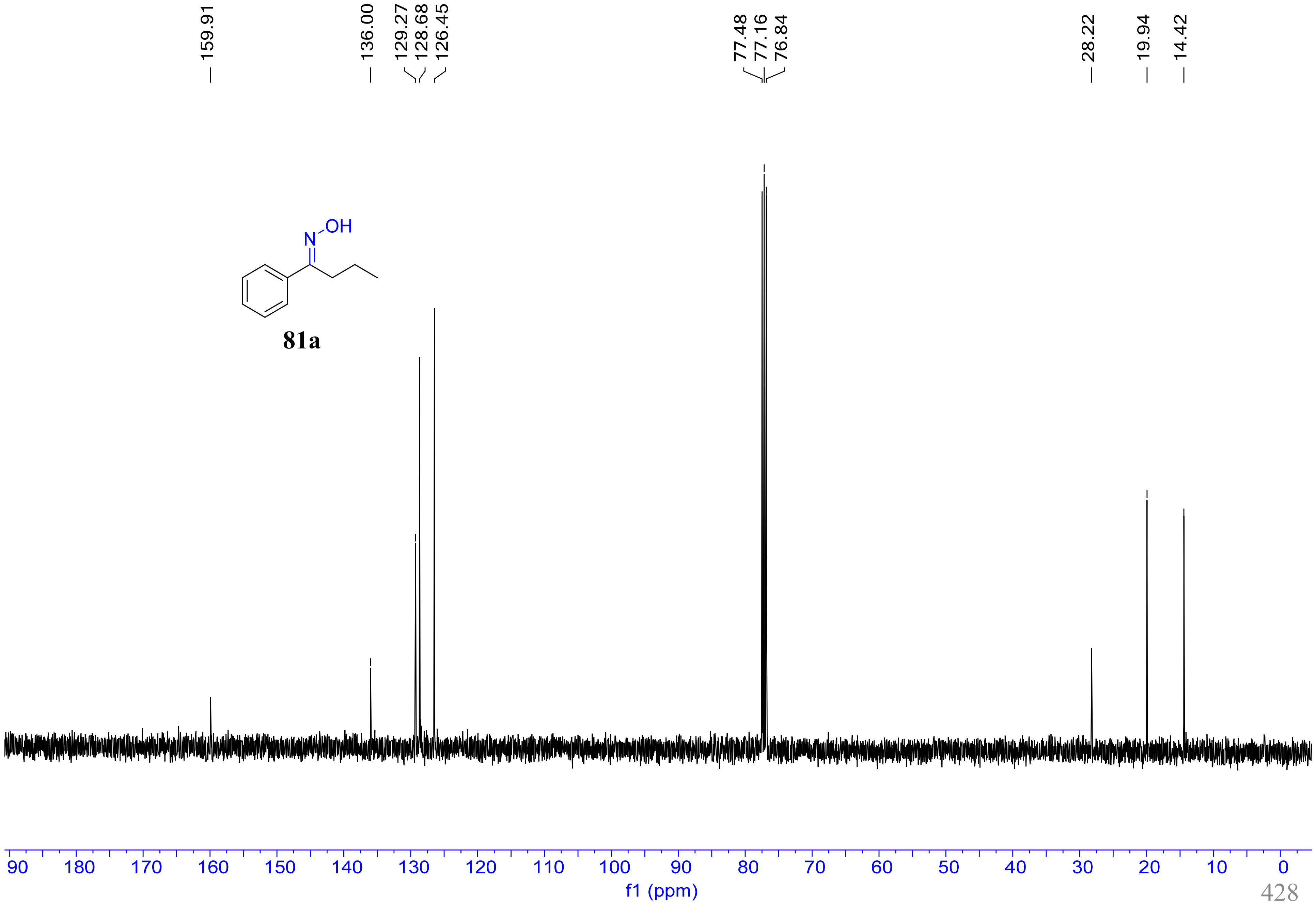

^1H NMR, 400 MHz, $CDCl_3$

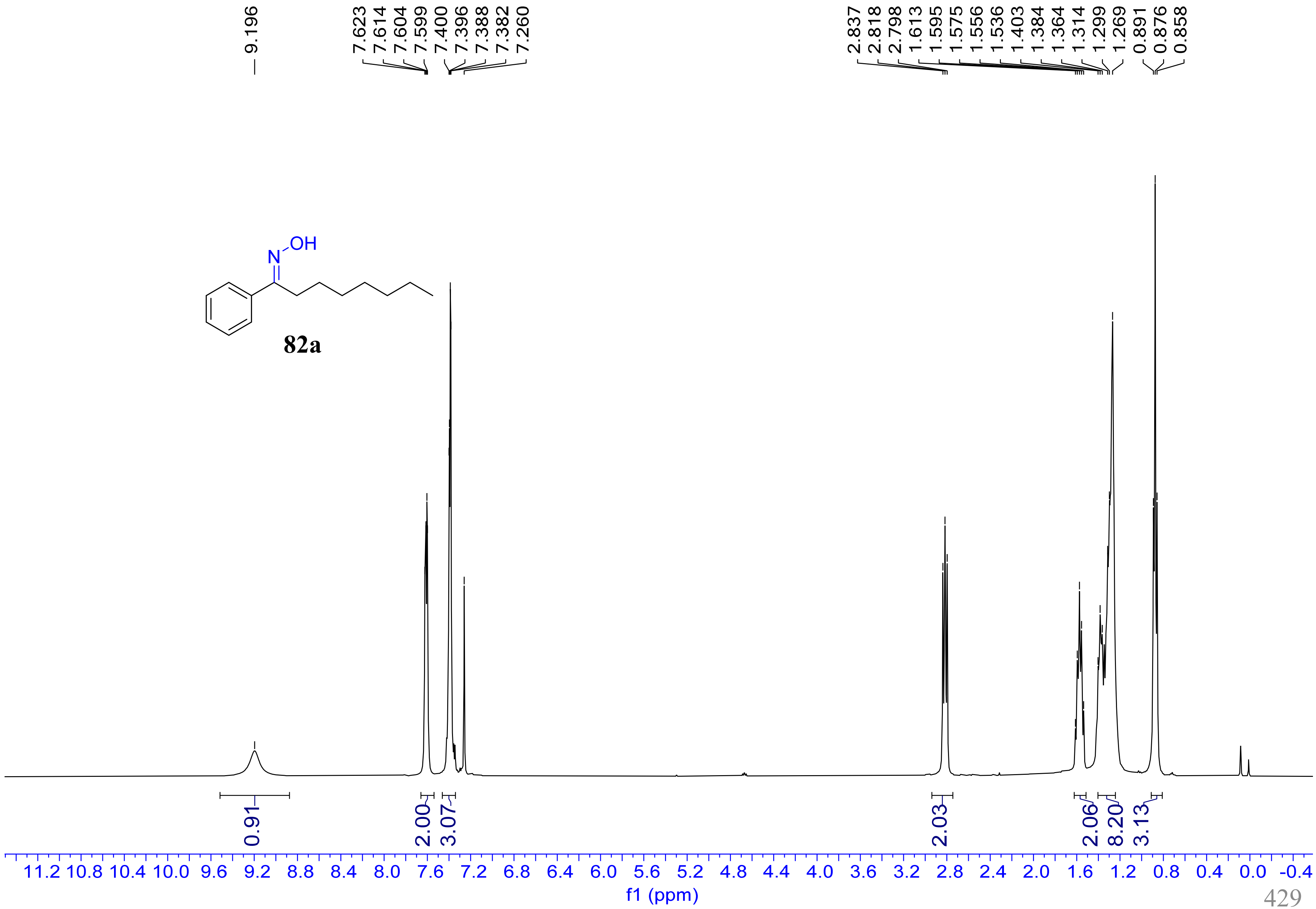

^{13}C NMR 101 MHz, $CDCl_3$

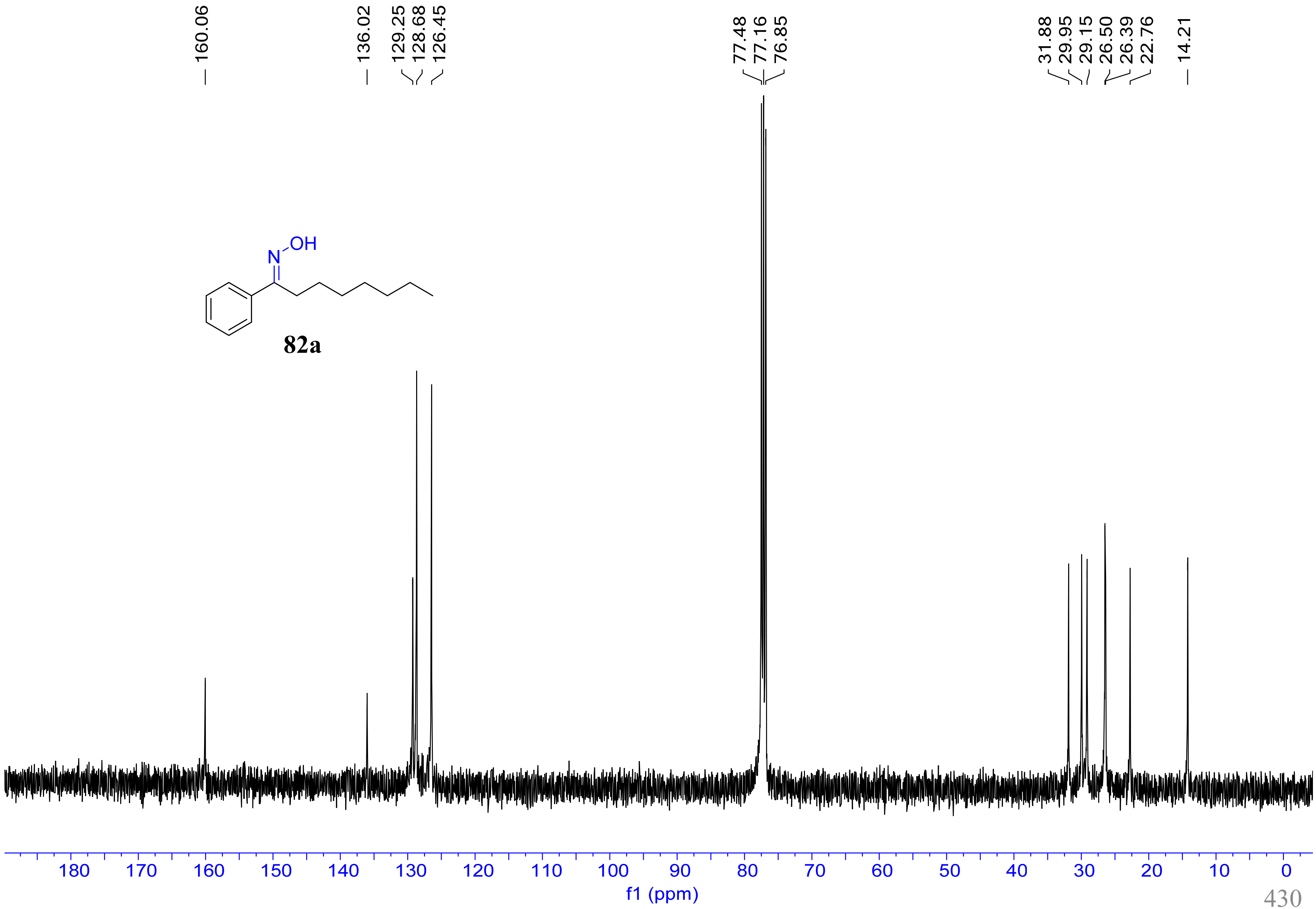

^{1}H NMR, 400 MHz, $CDCl_3$

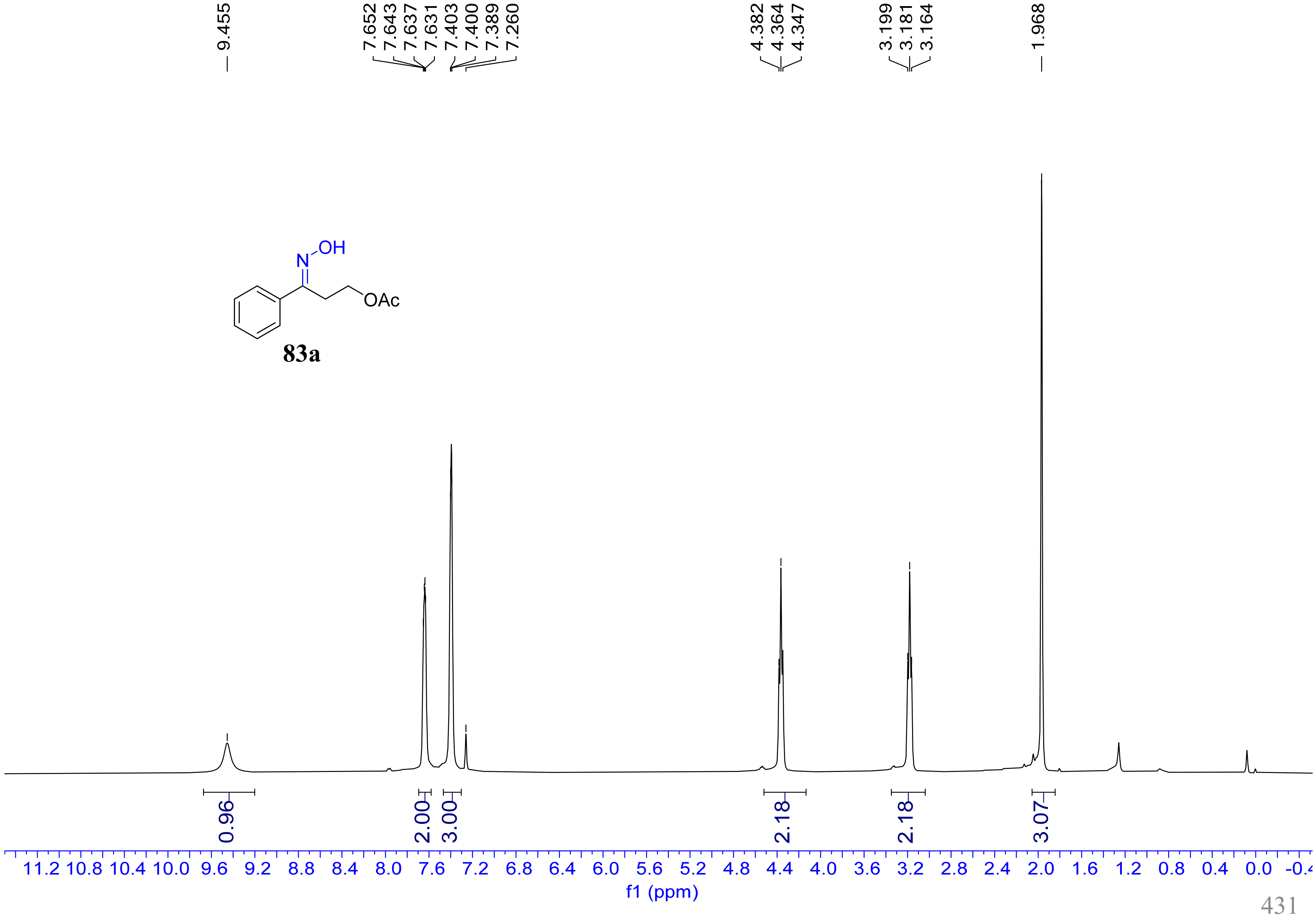

^{13}C NMR 101 MHz, $CDCl_3$

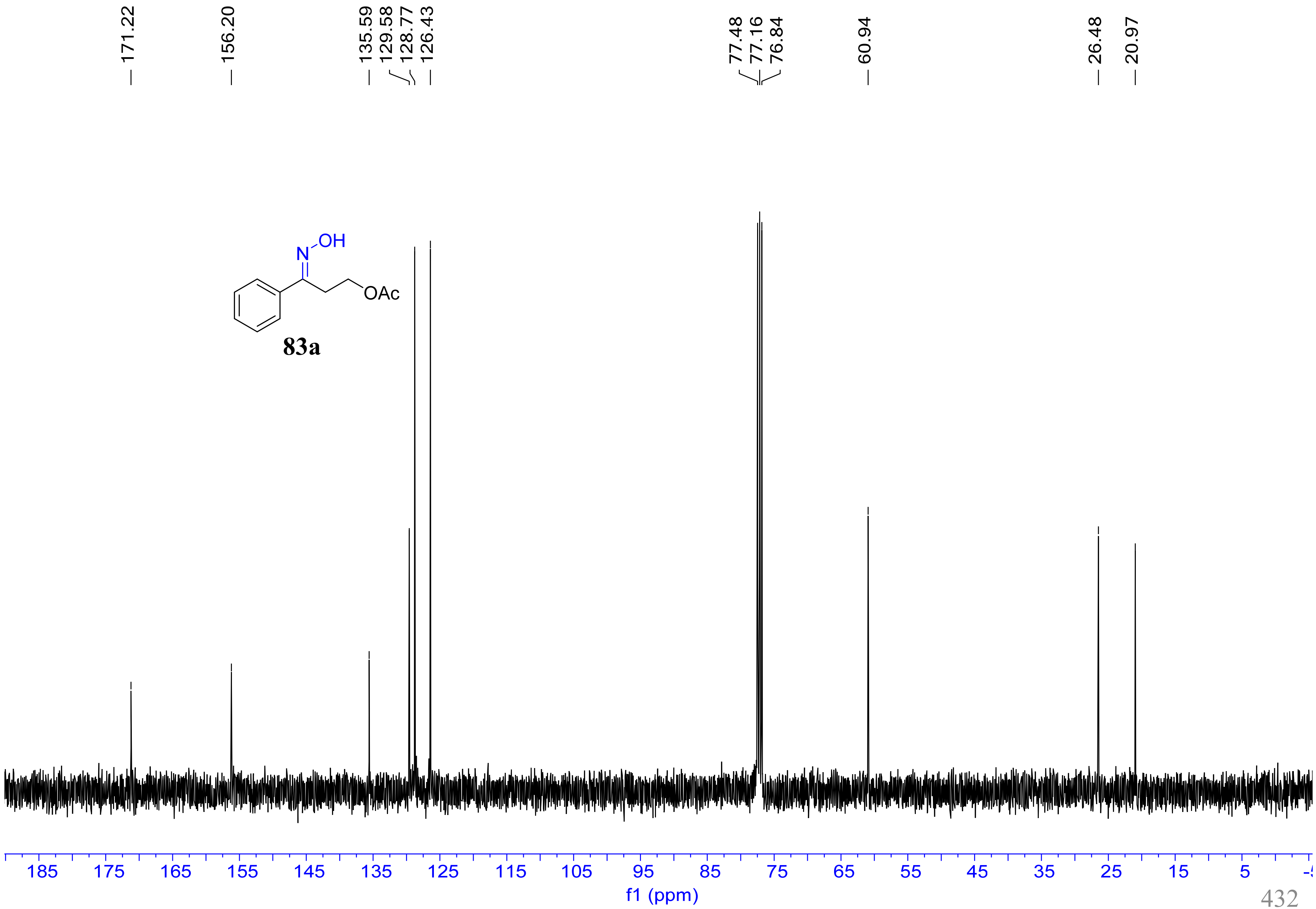

83a

^{1}H NMR, 400 MHz, $CDCl_3$

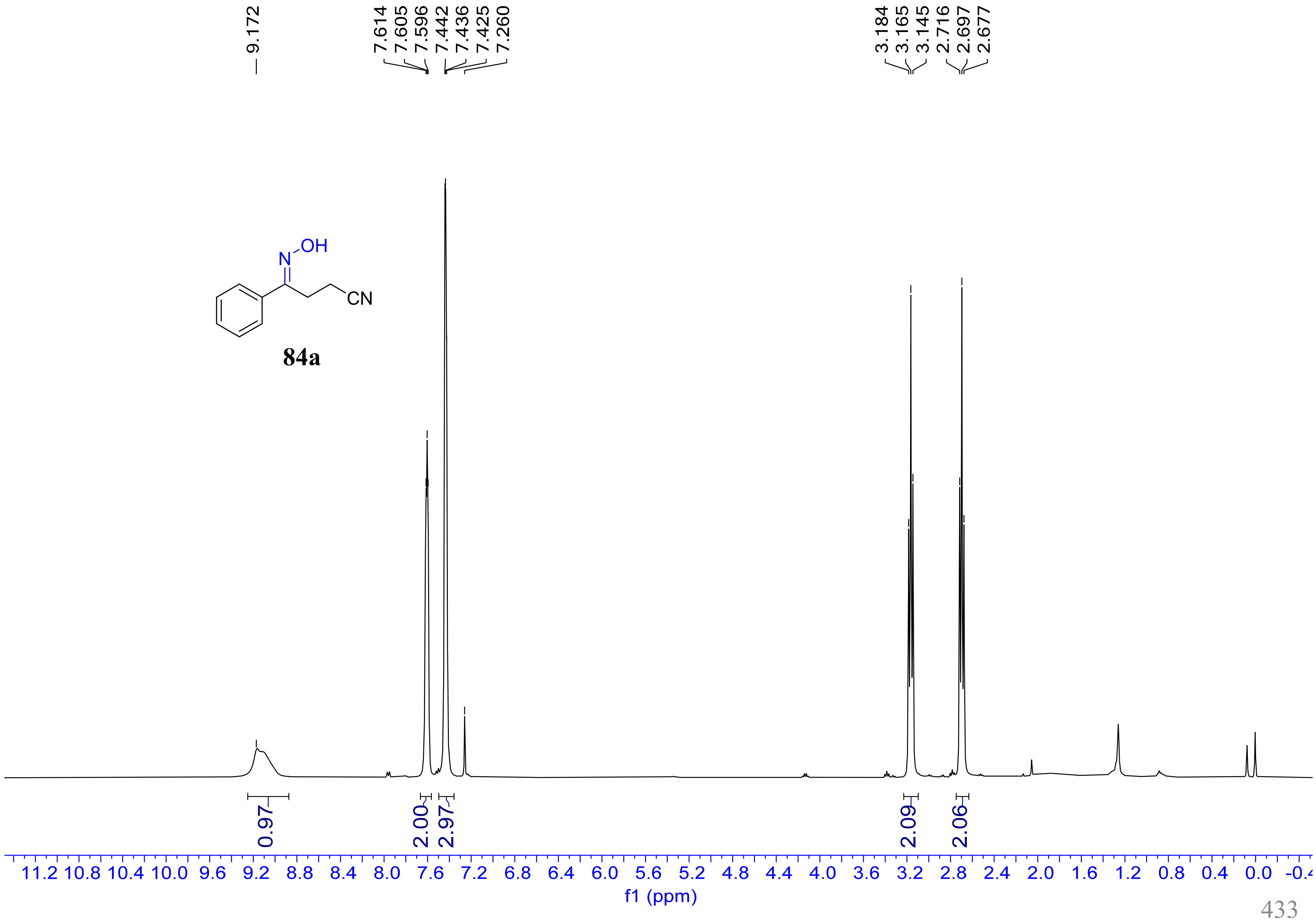

^{13}C NMR 101 MHz, $CDCl_3$

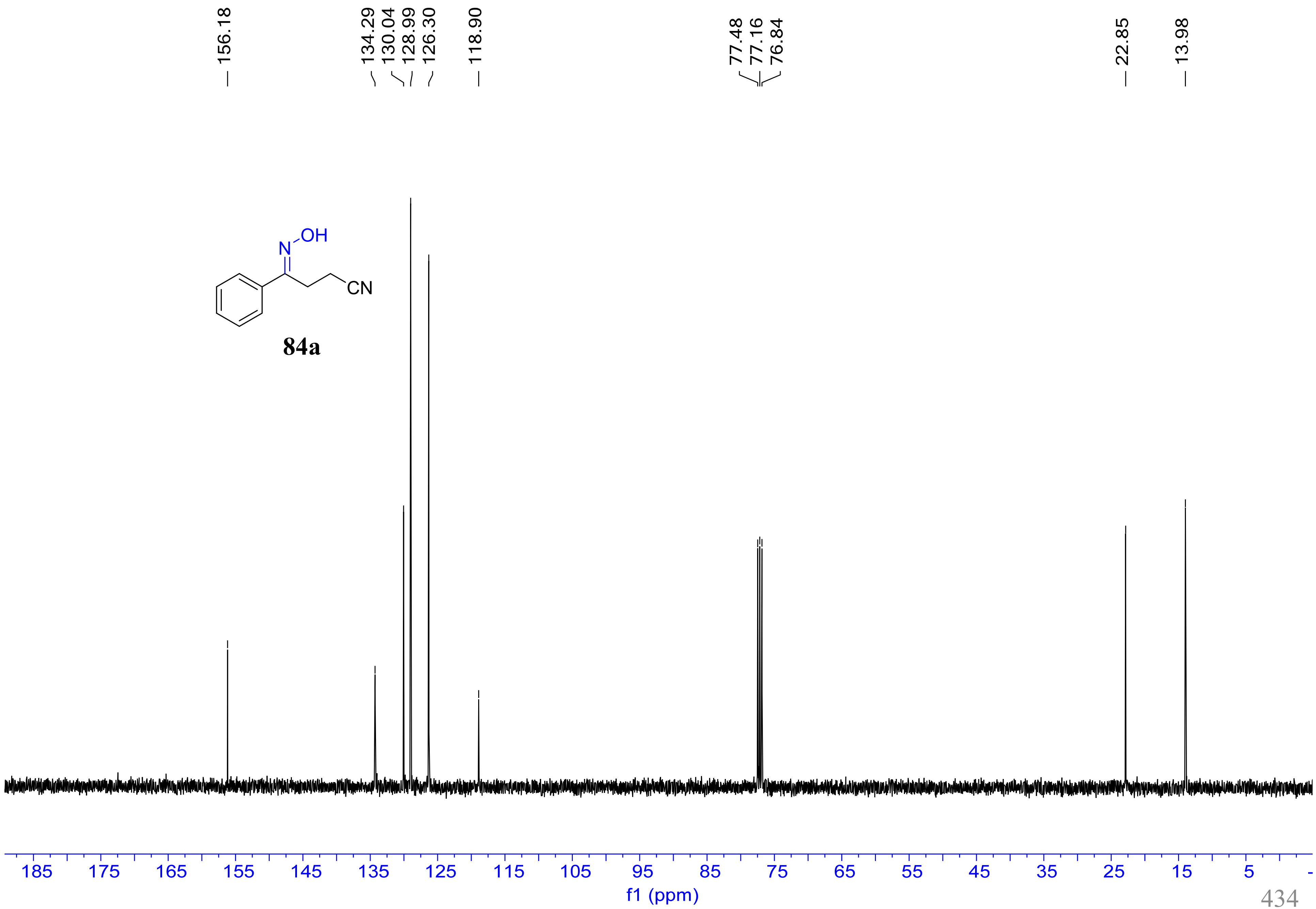

^{1}H NMR, 400 MHz, $CDCl_3$

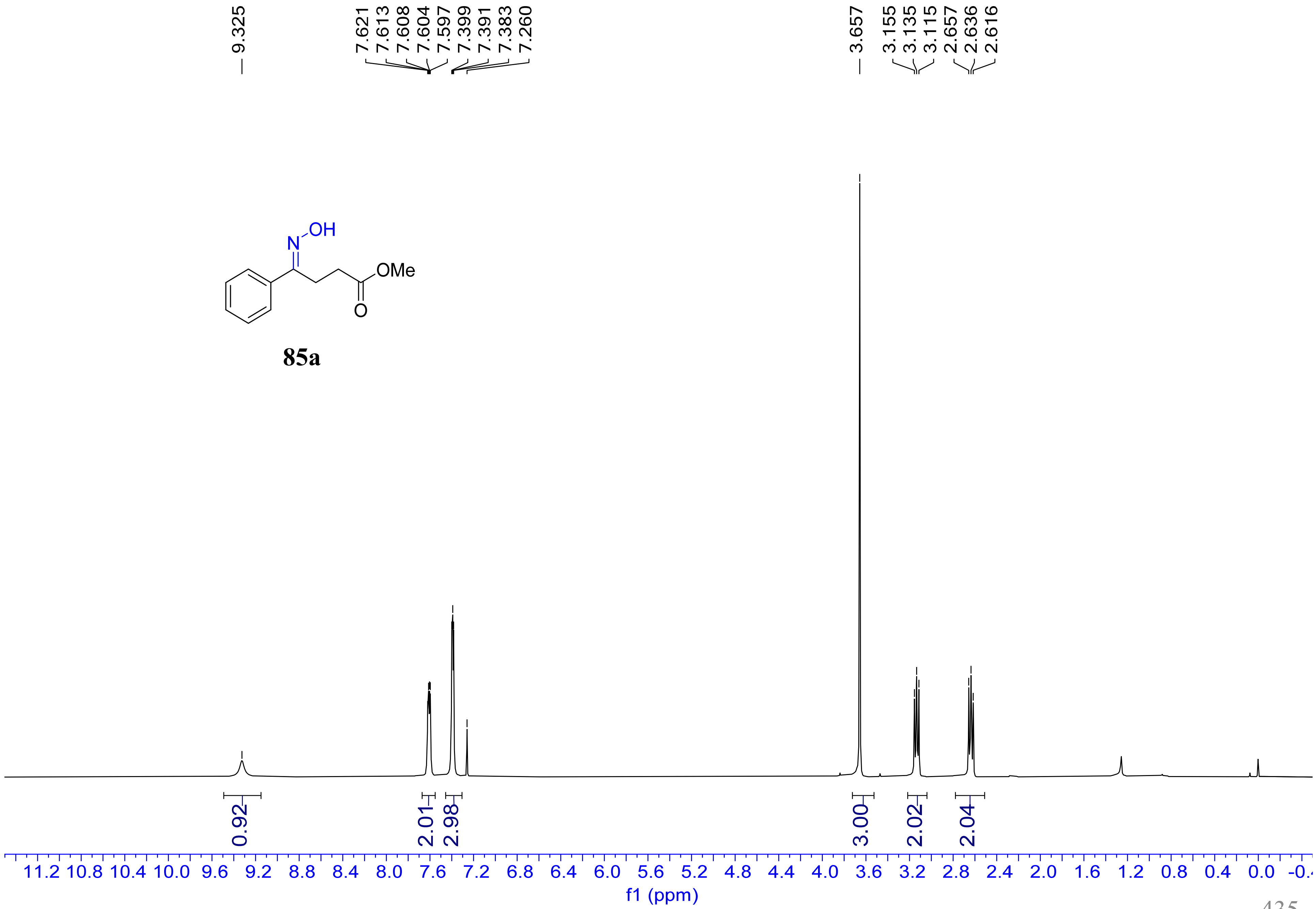

^{13}C NMR 101 MHz, $CDCl_3$

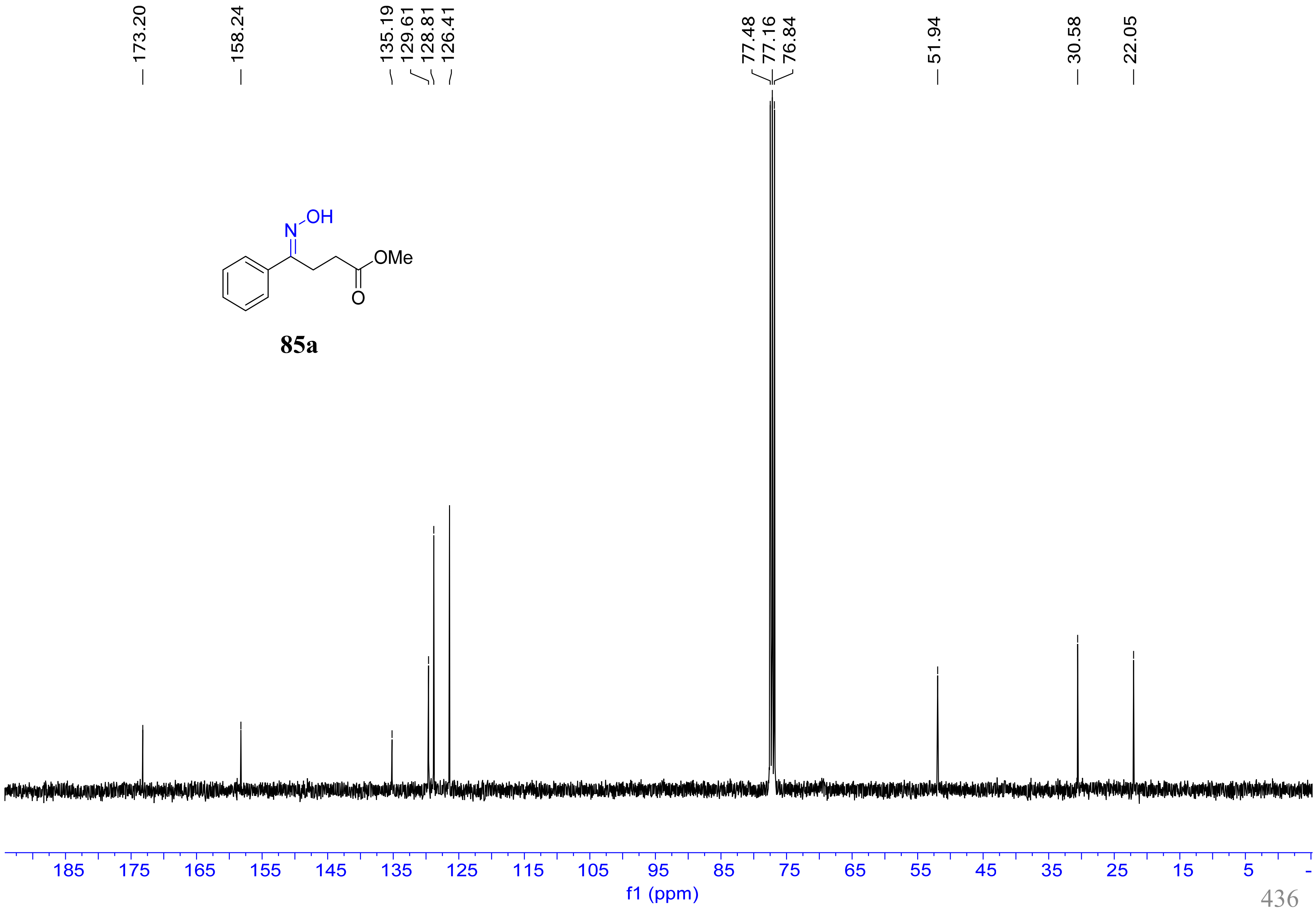

85a

^{1}H NMR, 400 MHz, $CDCl_3$

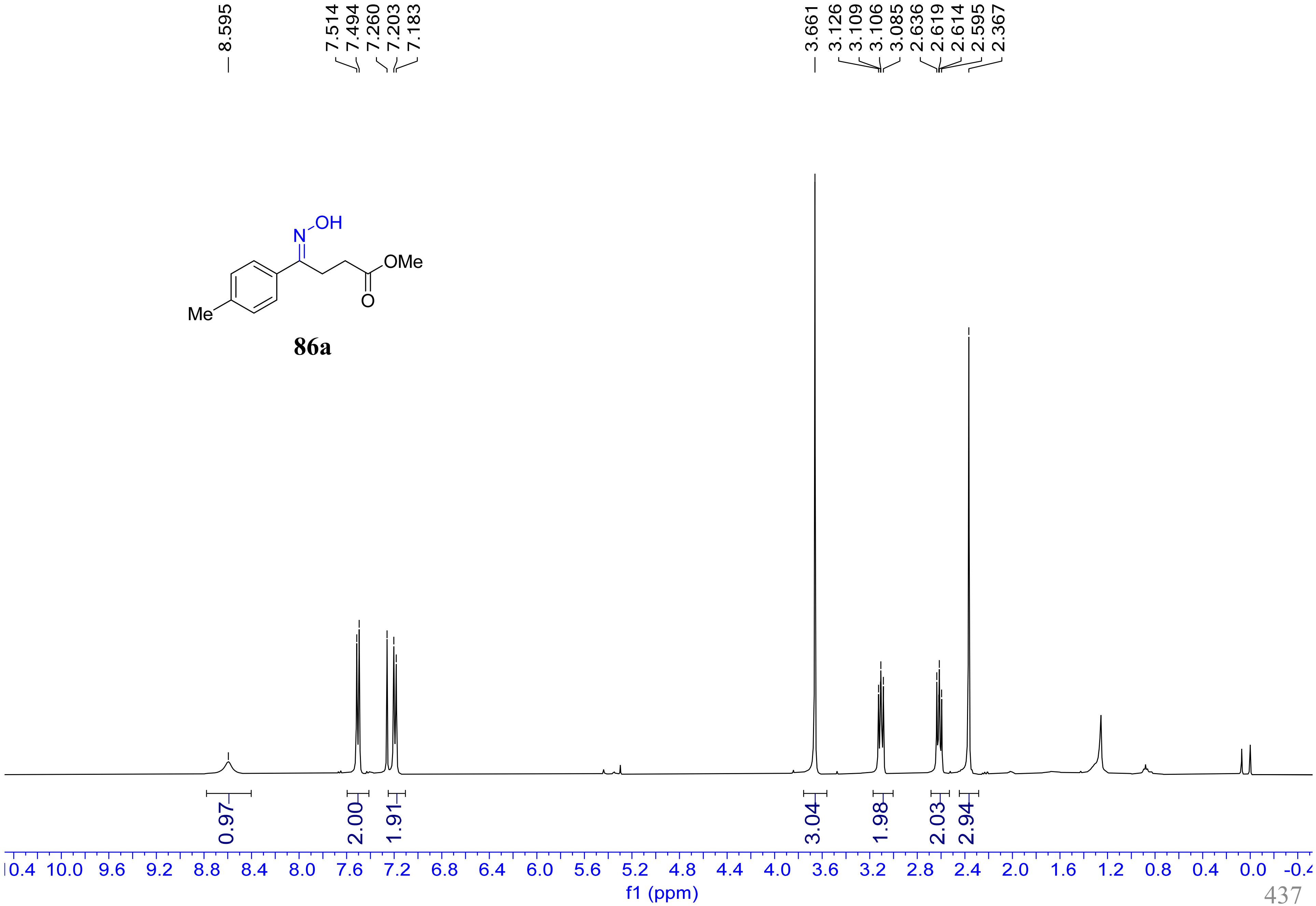

^{13}C NMR 101 MHz, $CDCl_3$

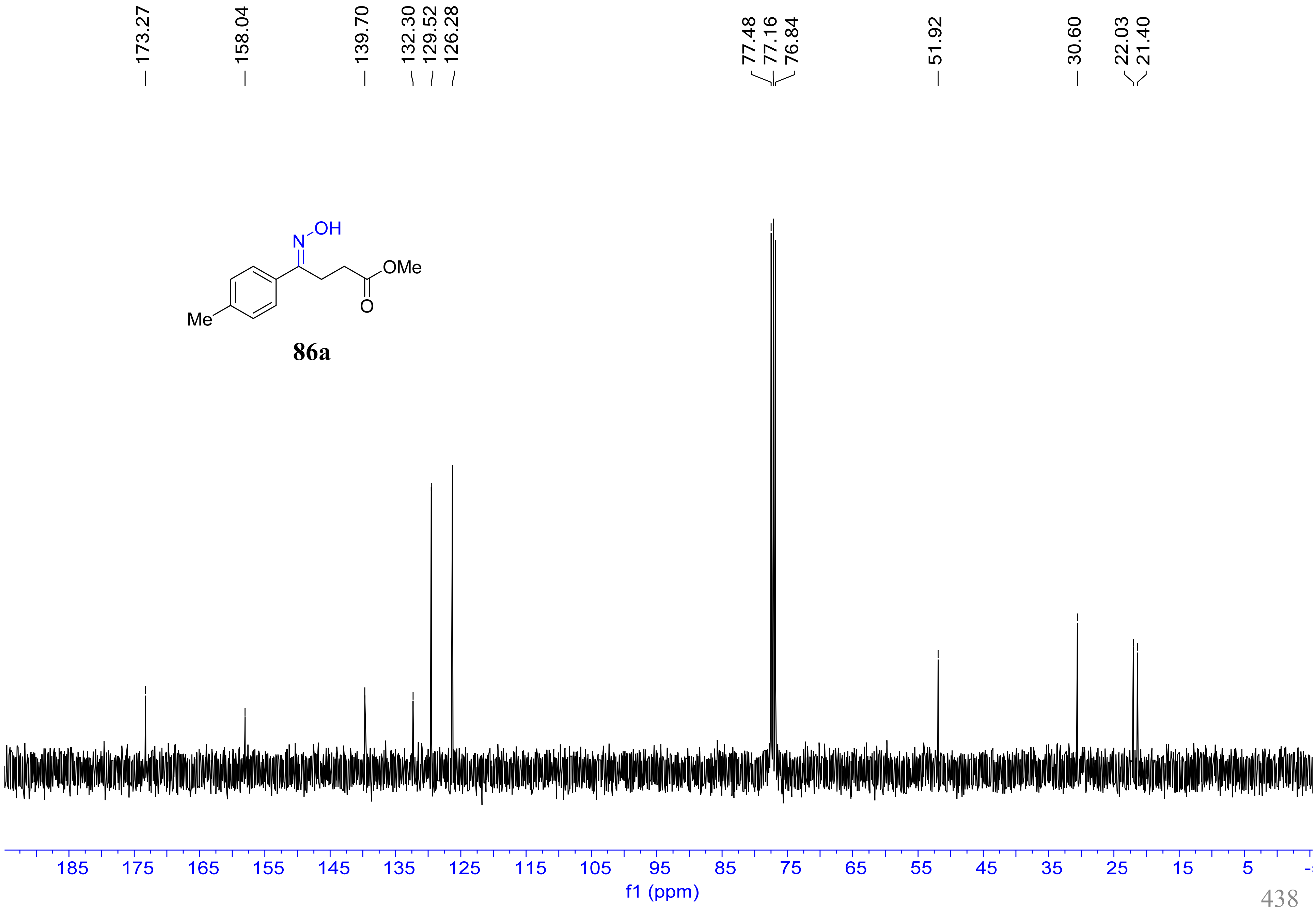

86a

^{1}H NMR, 400 MHz, $CDCl_3$

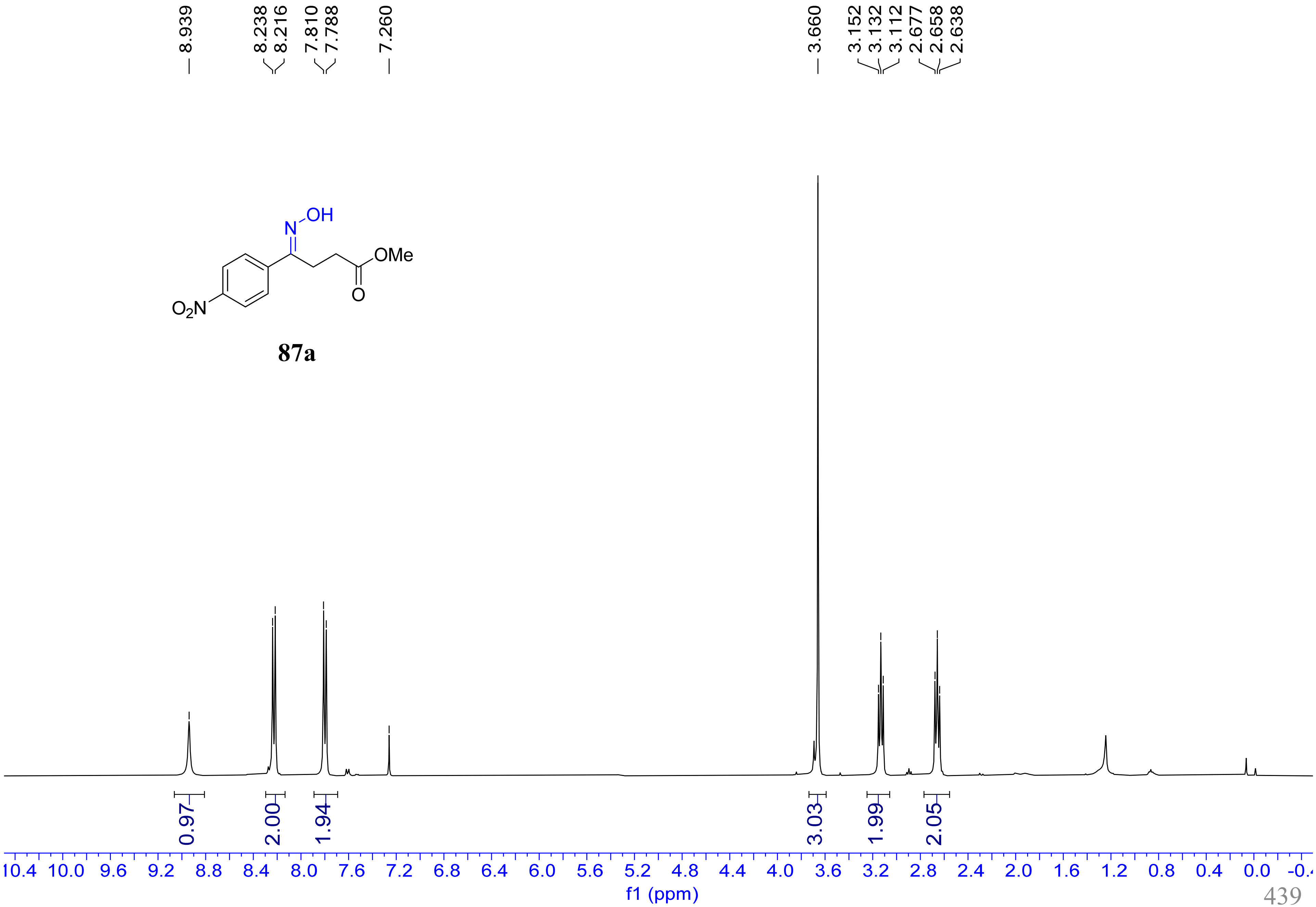

^{13}C NMR 101 MHz, $CDCl_3$

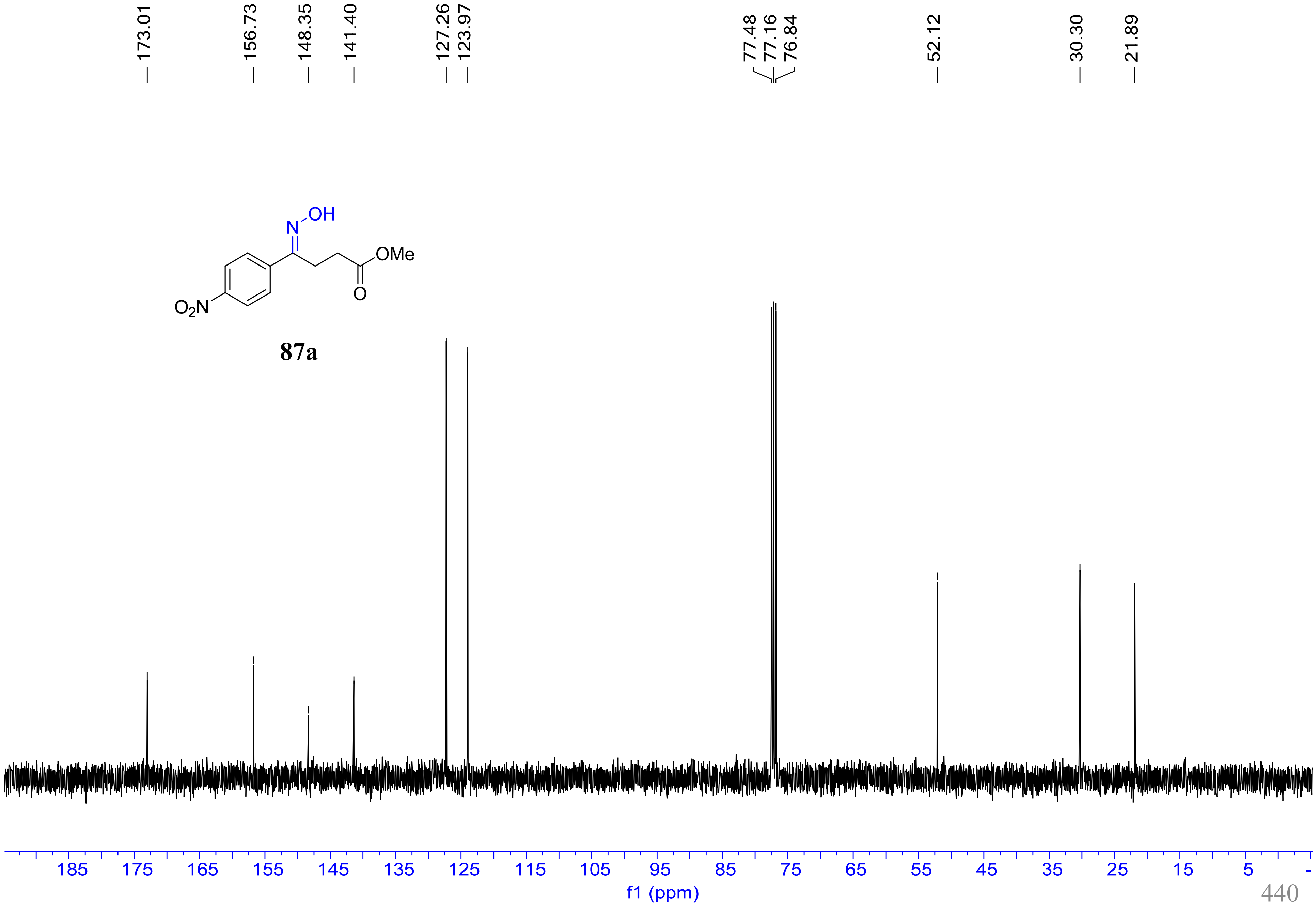

^{1}H NMR, 400 MHz, $CDCl_3$

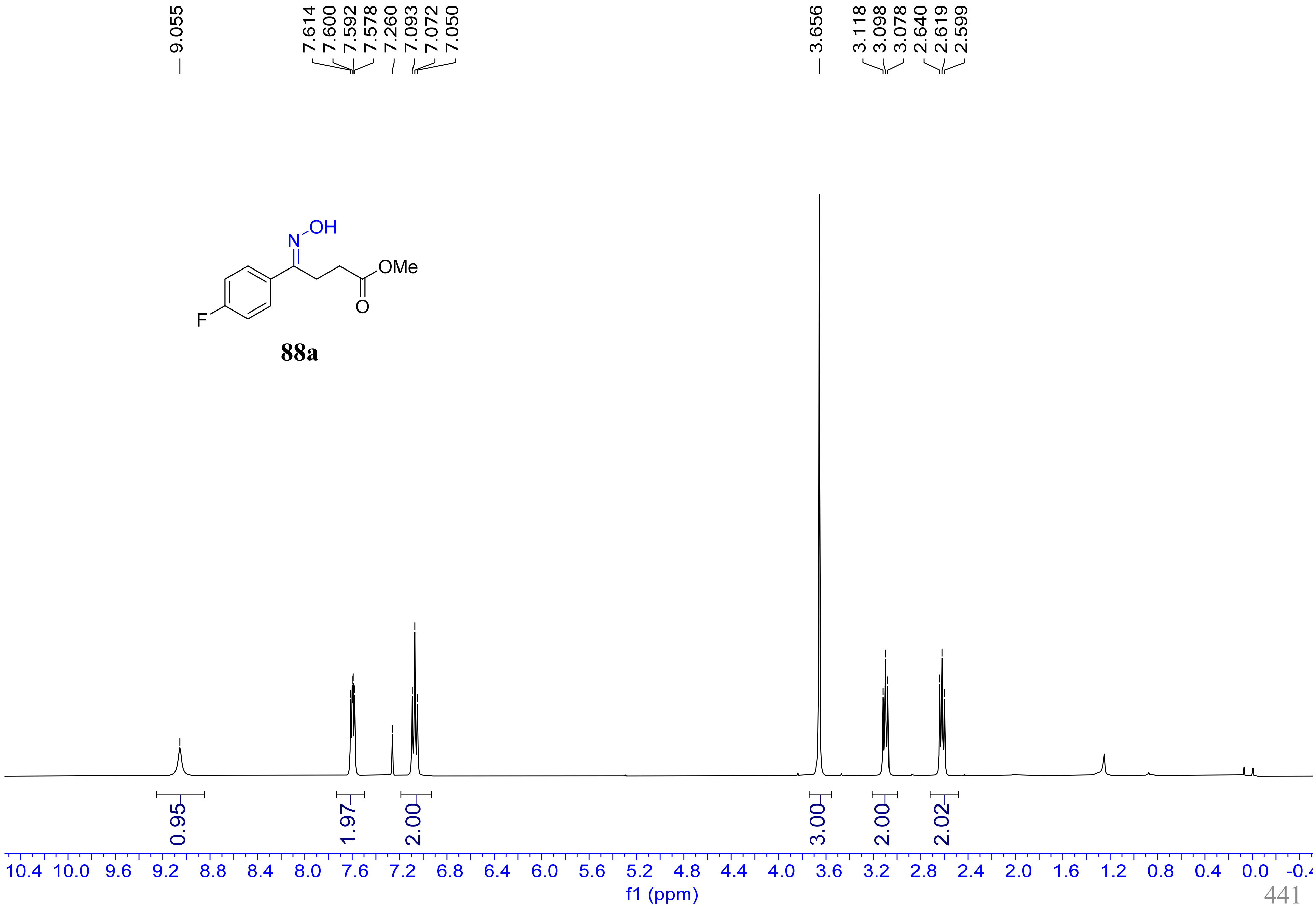

^{13}C NMR 101 MHz, $CDCl_3$

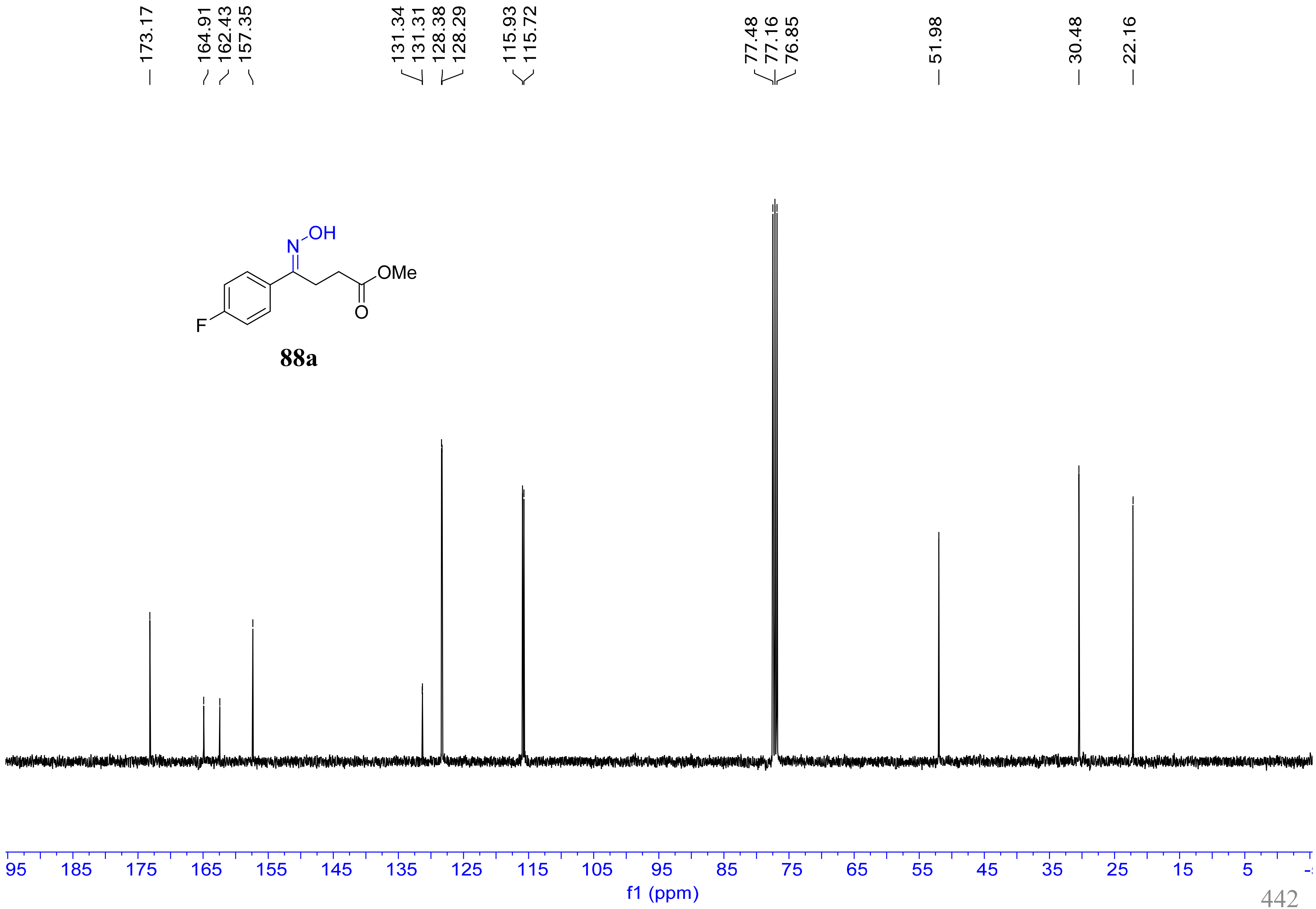

^{19}F NMR 376 MHz, $CDCl_3$

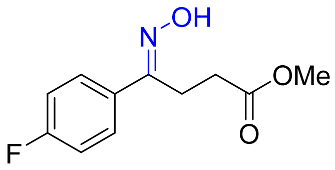

88a

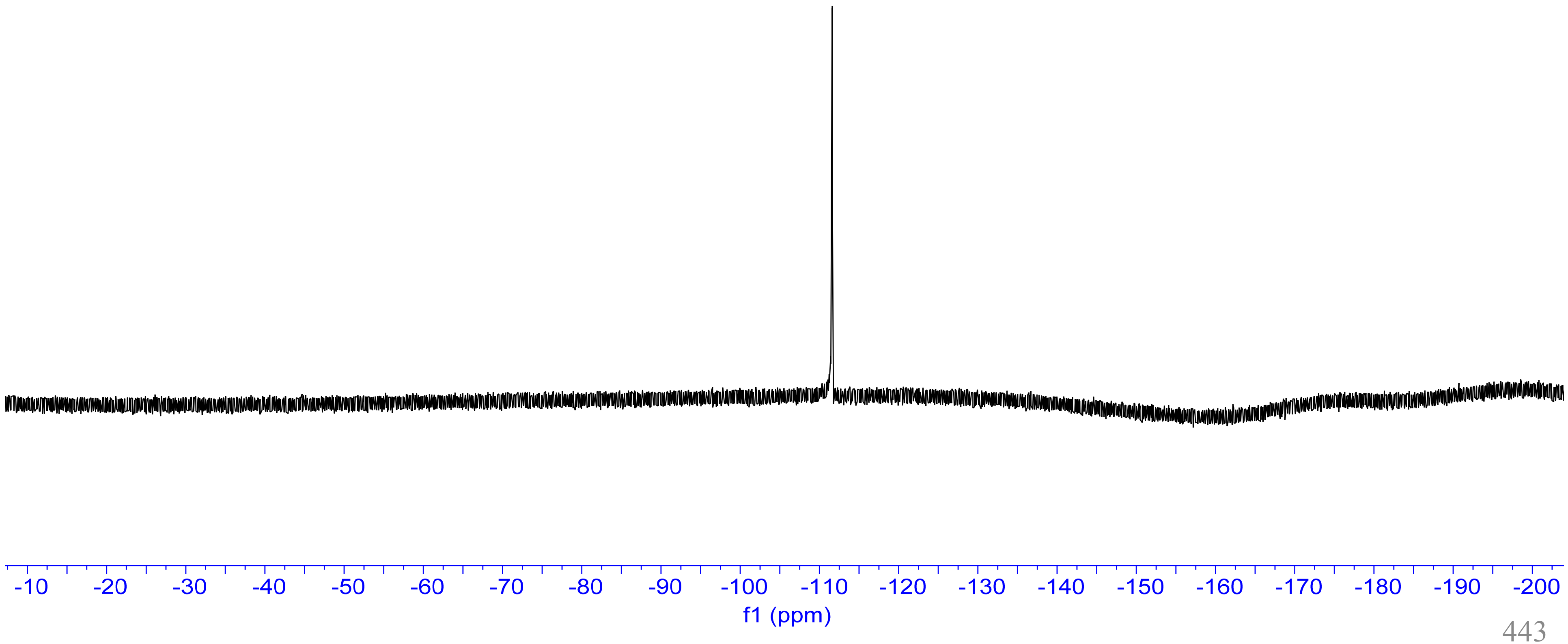

^{1}H NMR, 400 MHz, CDCl$_3$

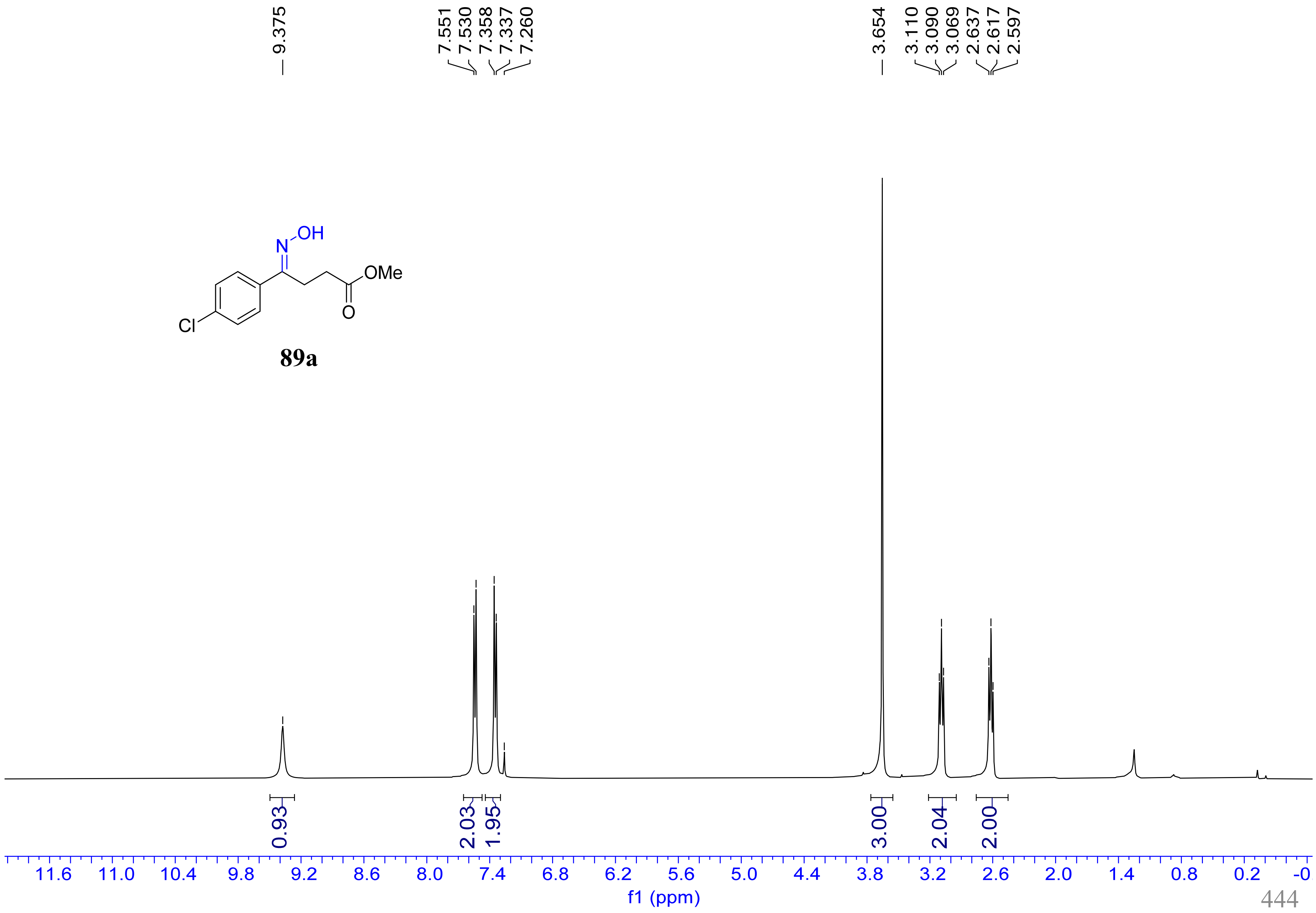

^{13}C NMR 101 MHz, $CDCl_3$

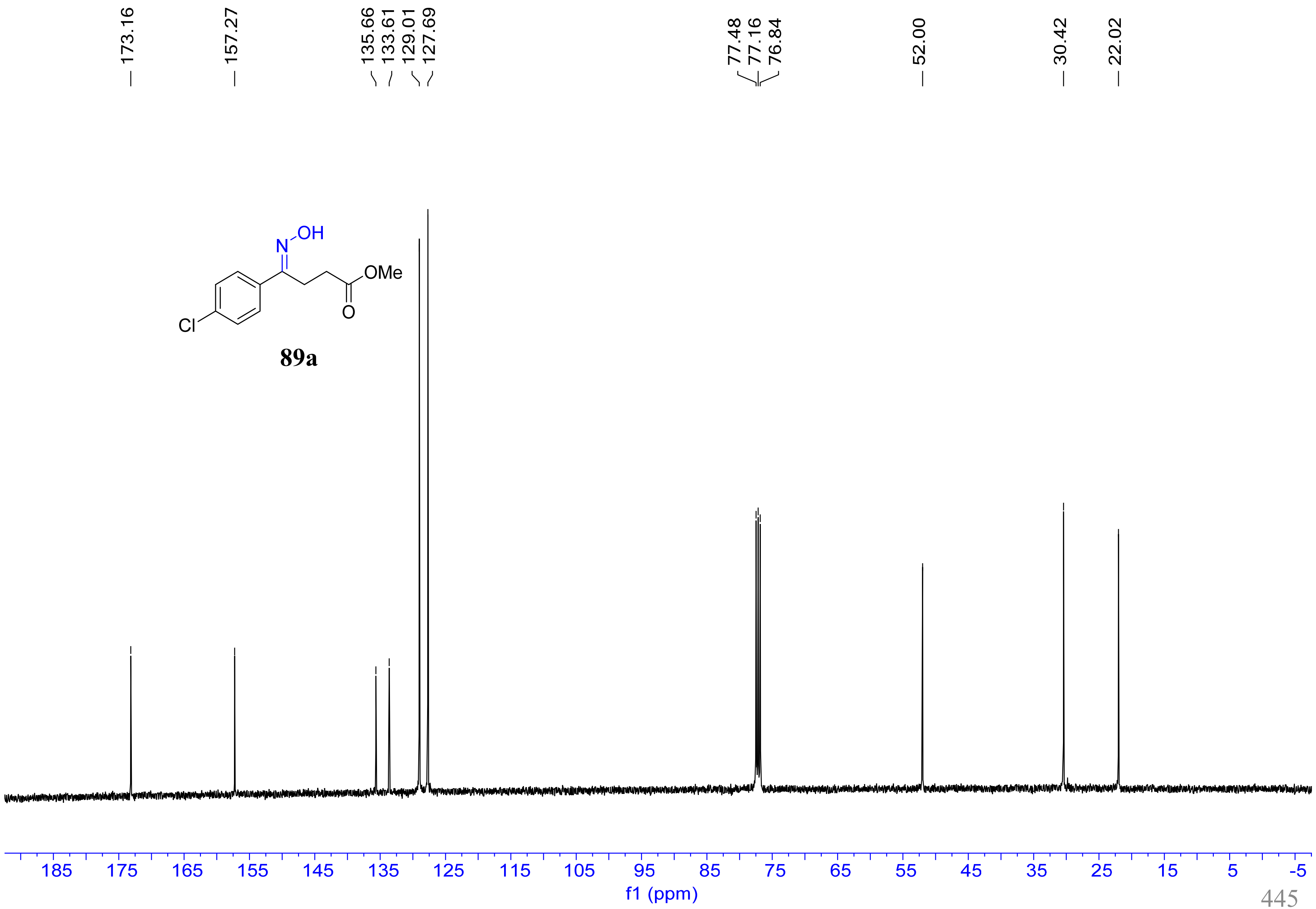

^{1}H NMR, 400 MHz, CDCl$_3$

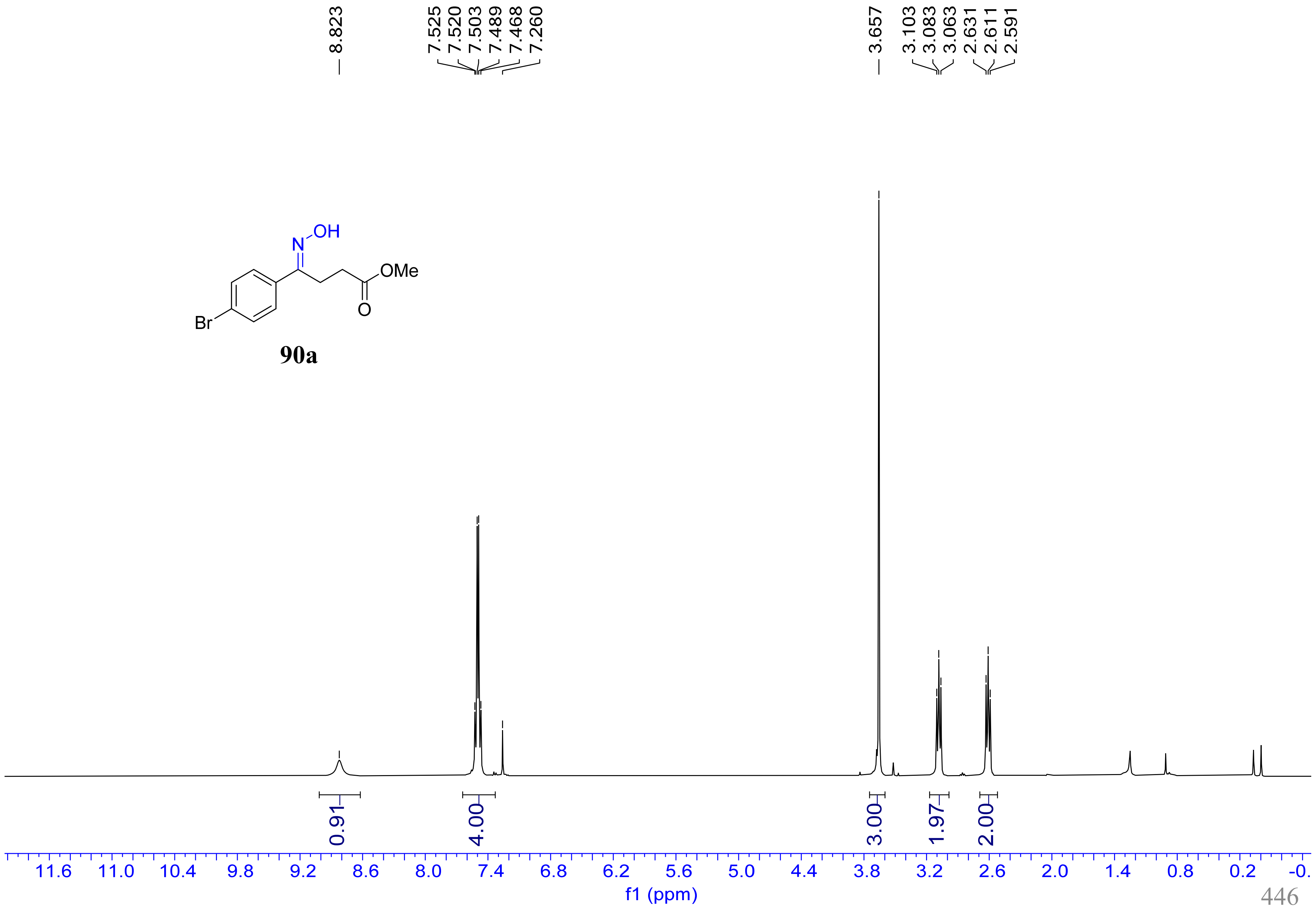

^{13}C NMR 101 MHz, $CDCl_3$

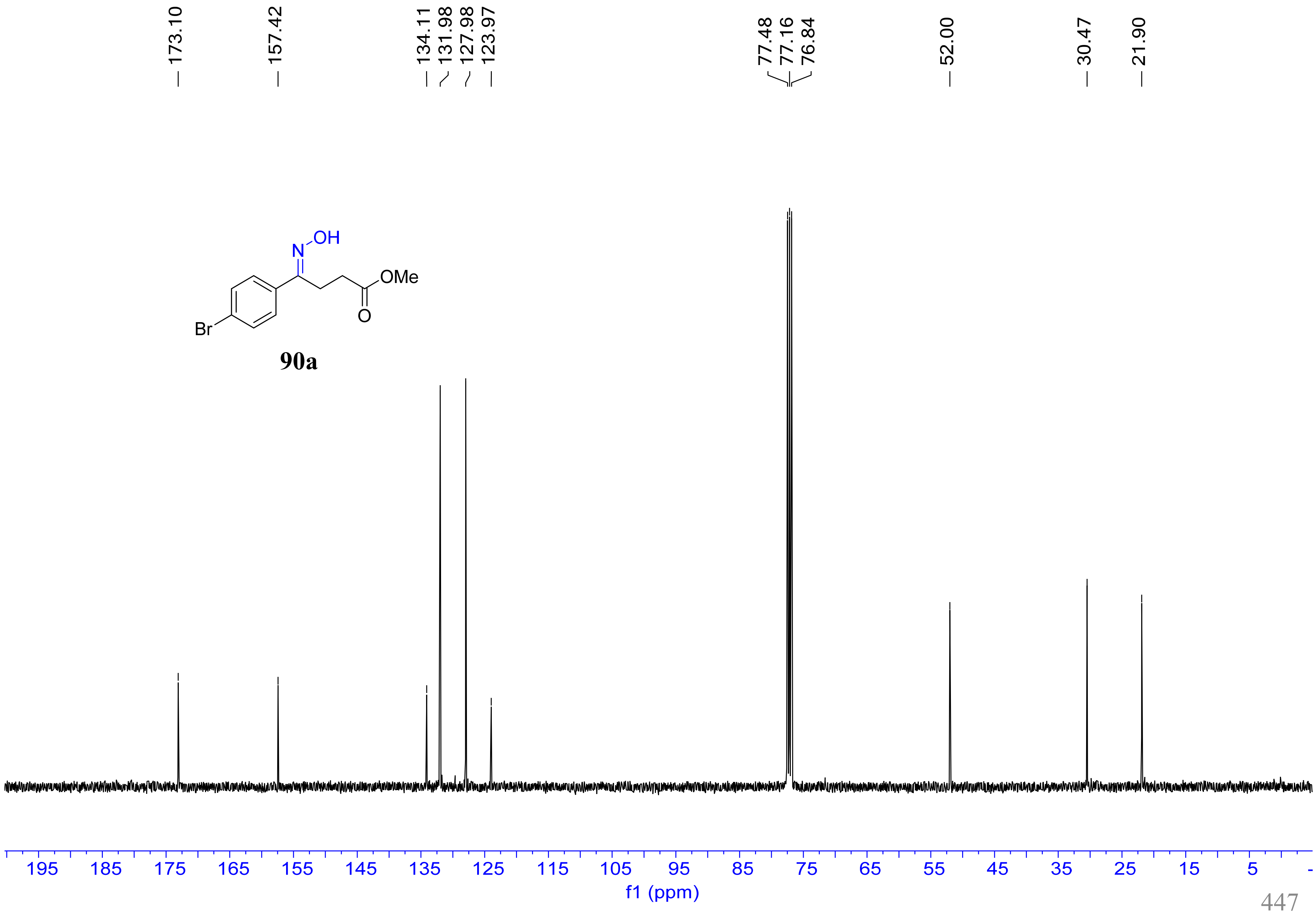

90a

^{1}H NMR, 400 MHz, $CDCl_3$

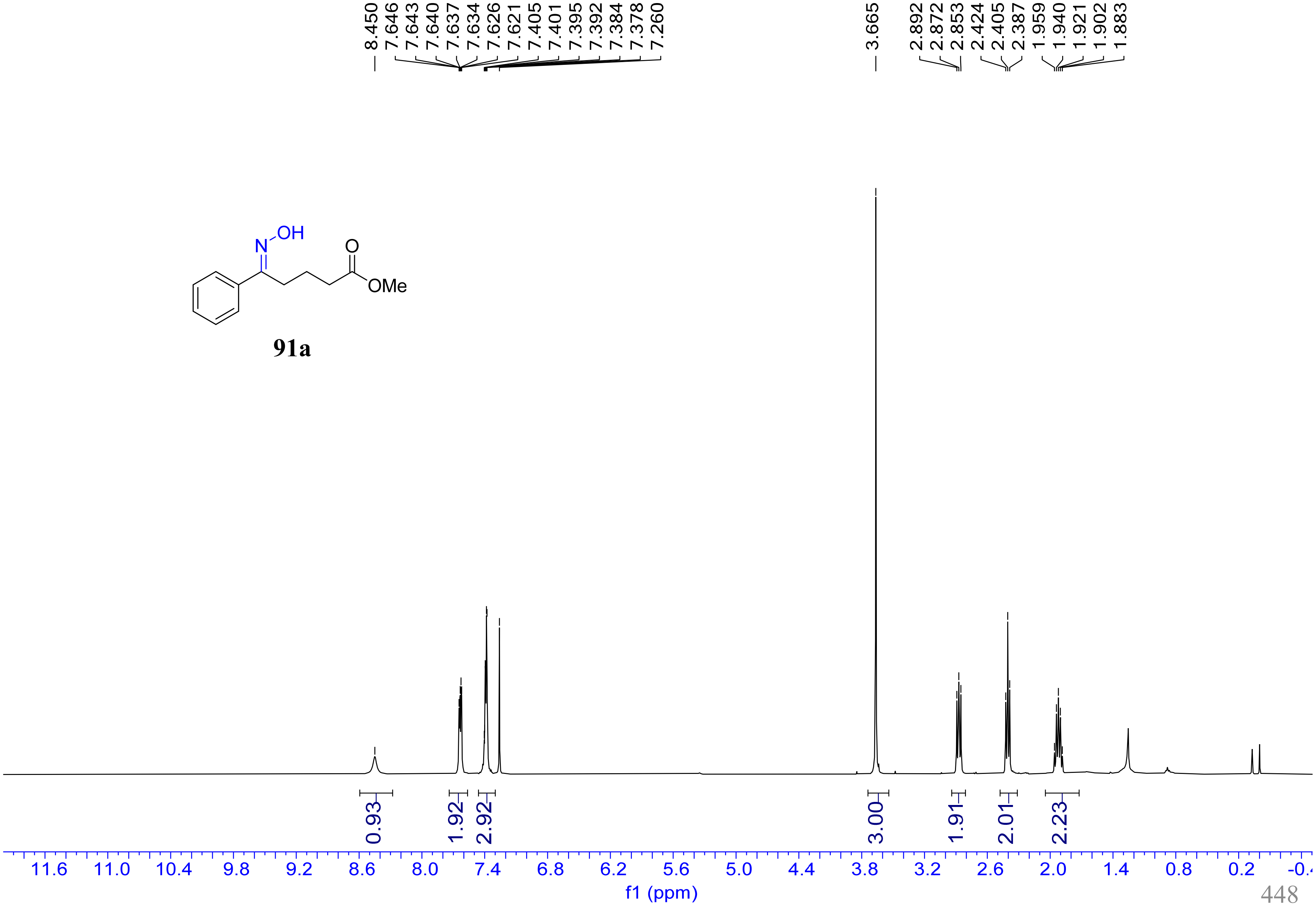

91a

^{13}C NMR 101 MHz, $CDCl_3$

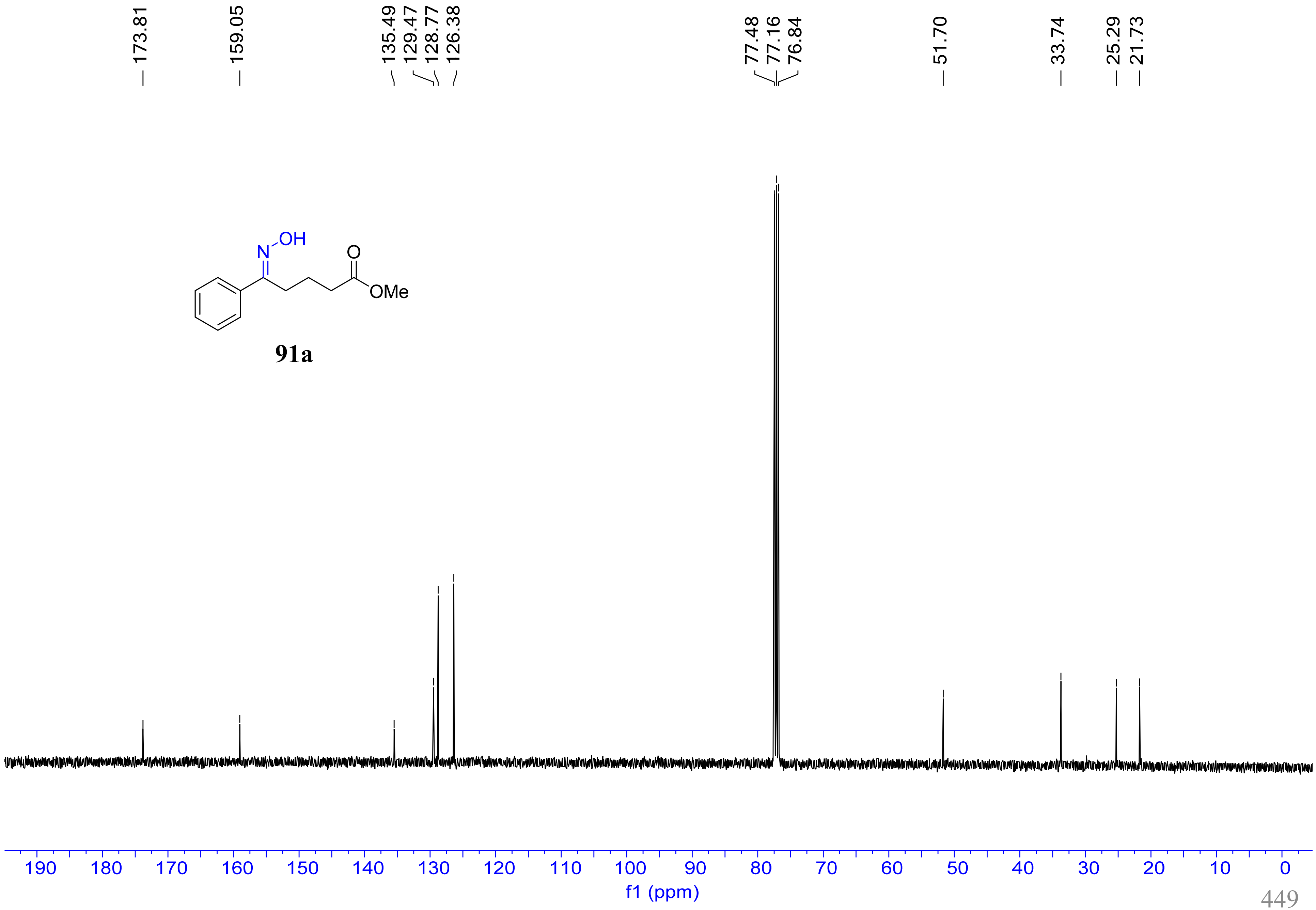

^{1}H NMR, 400 MHz, $CDCl_3$

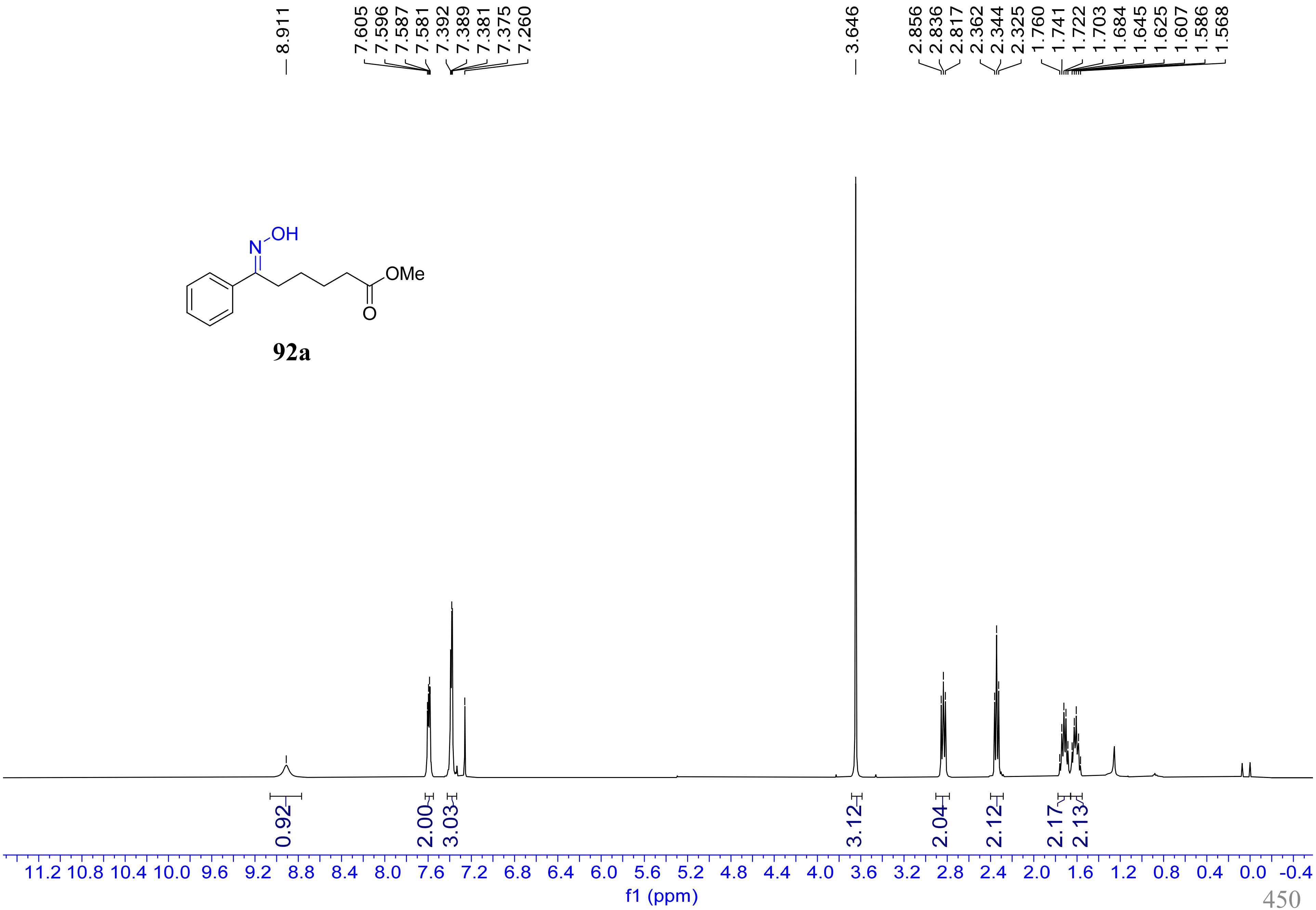

^{13}C NMR 101 MHz, $CDCl_3$

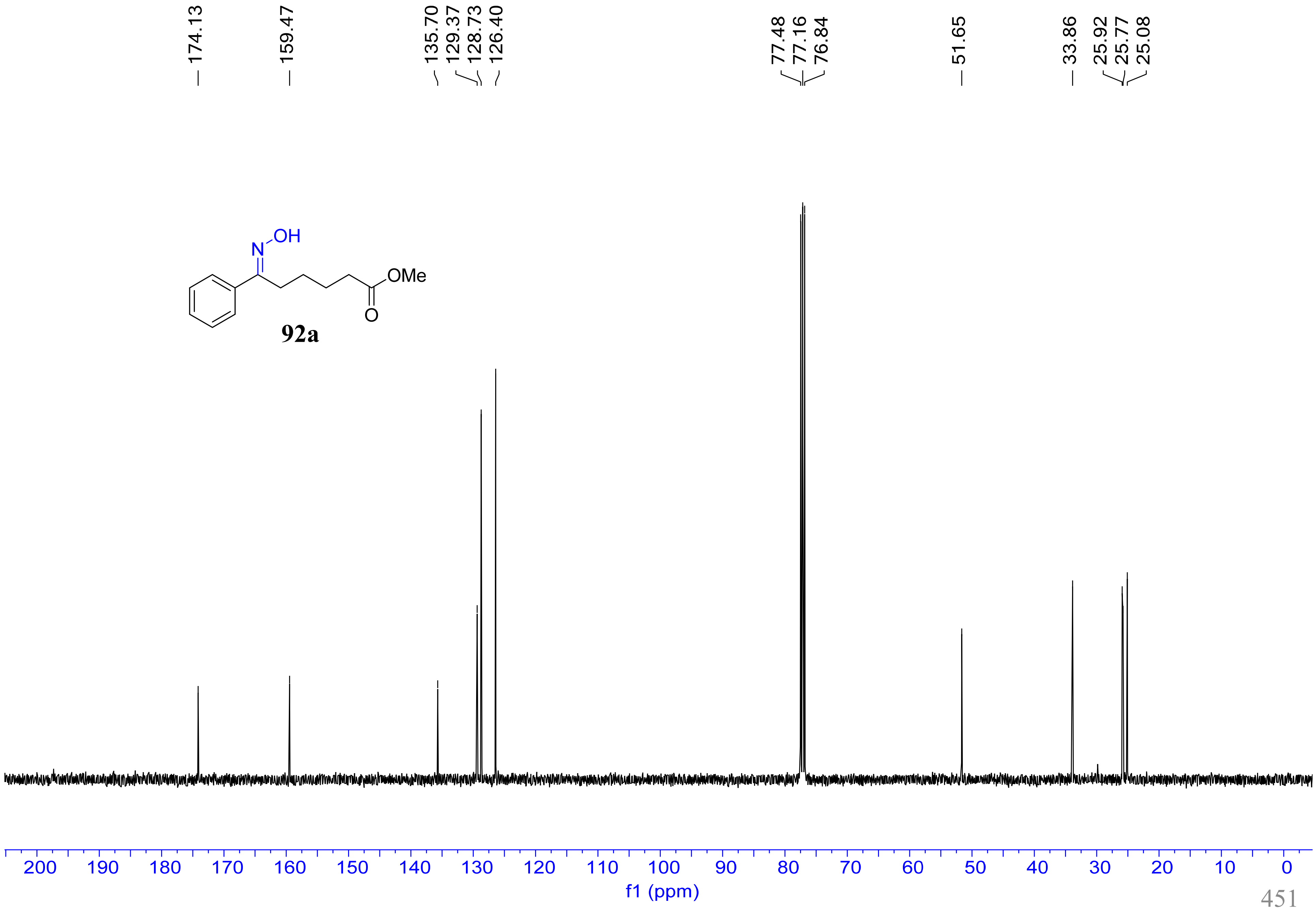

^{1}H NMR, 400 MHz, $CDCl_3$

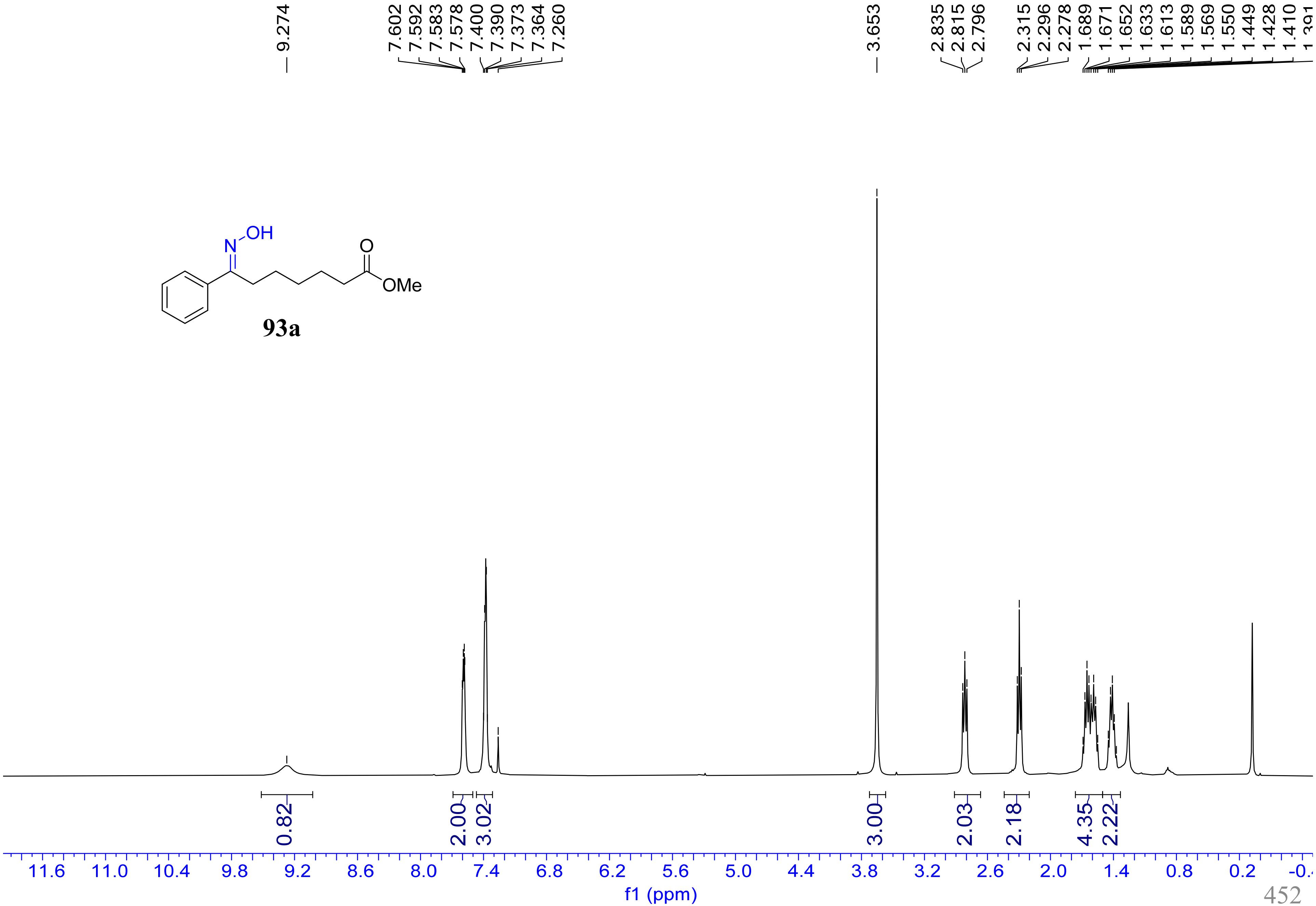

93a

^{13}C NMR 101 MHz, $CDCl_3$

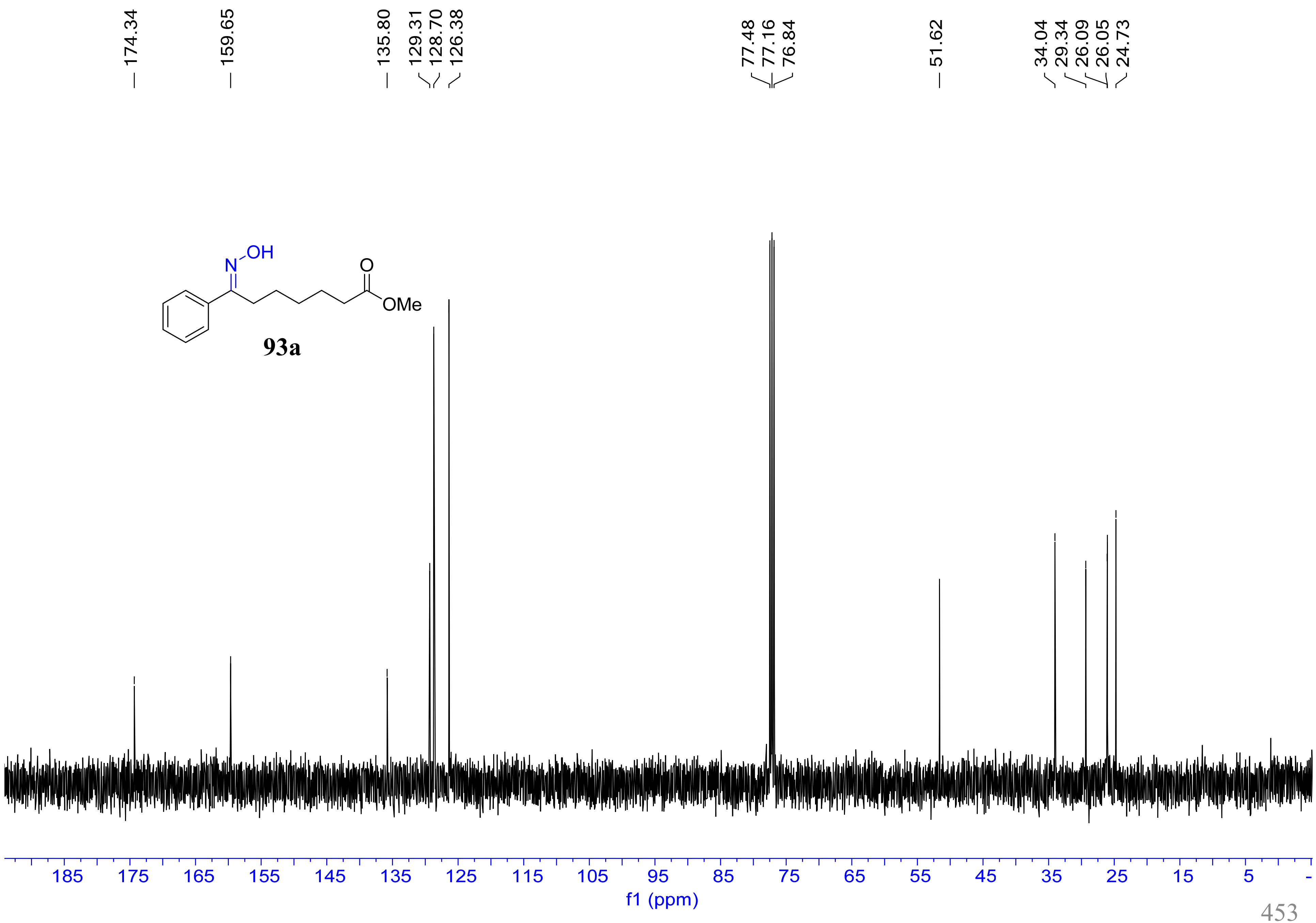

^{1}H NMR, 400 MHz, CDCl$_3$

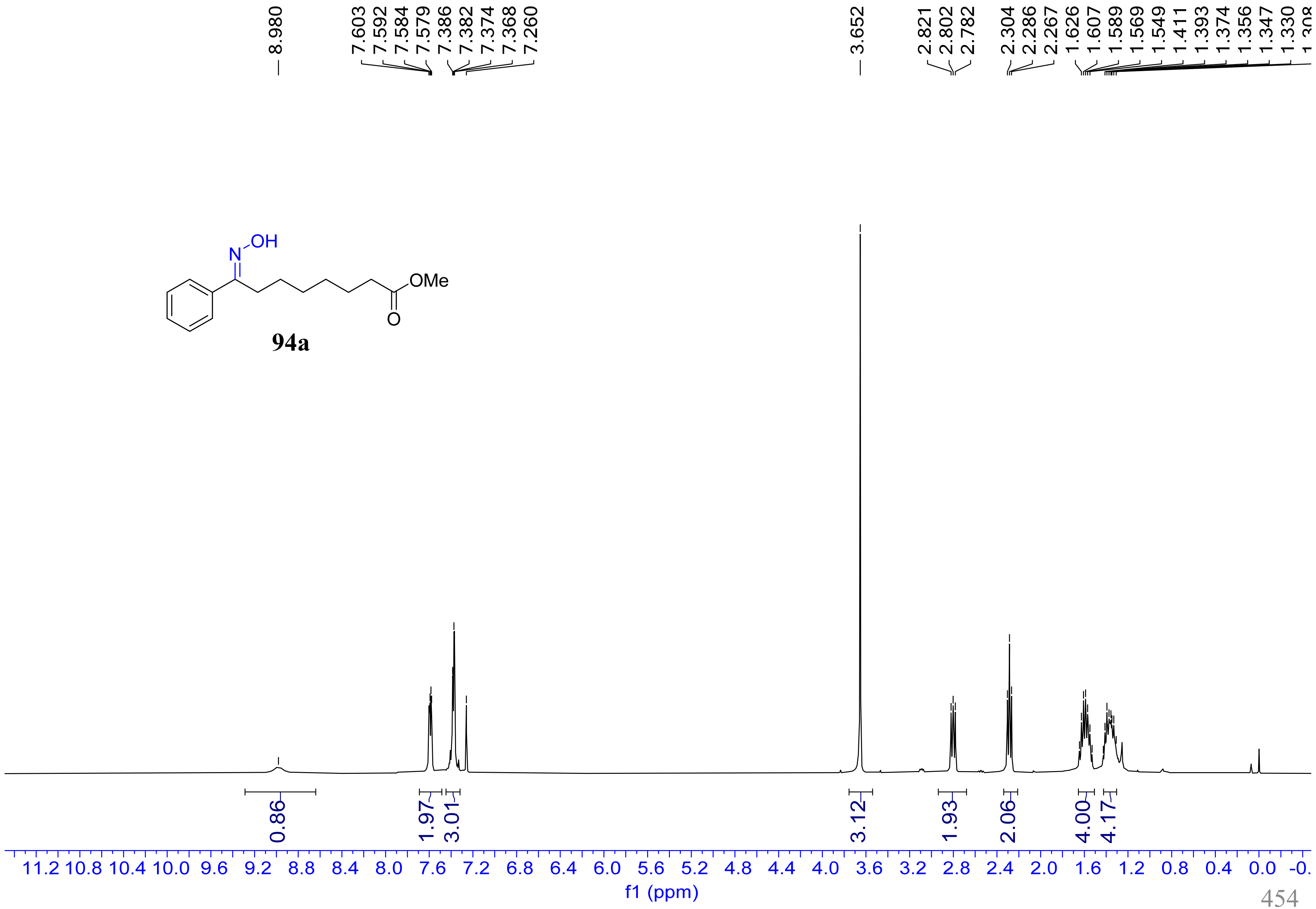

94a

^{13}C NMR 101 MHz, CDCl$_3$

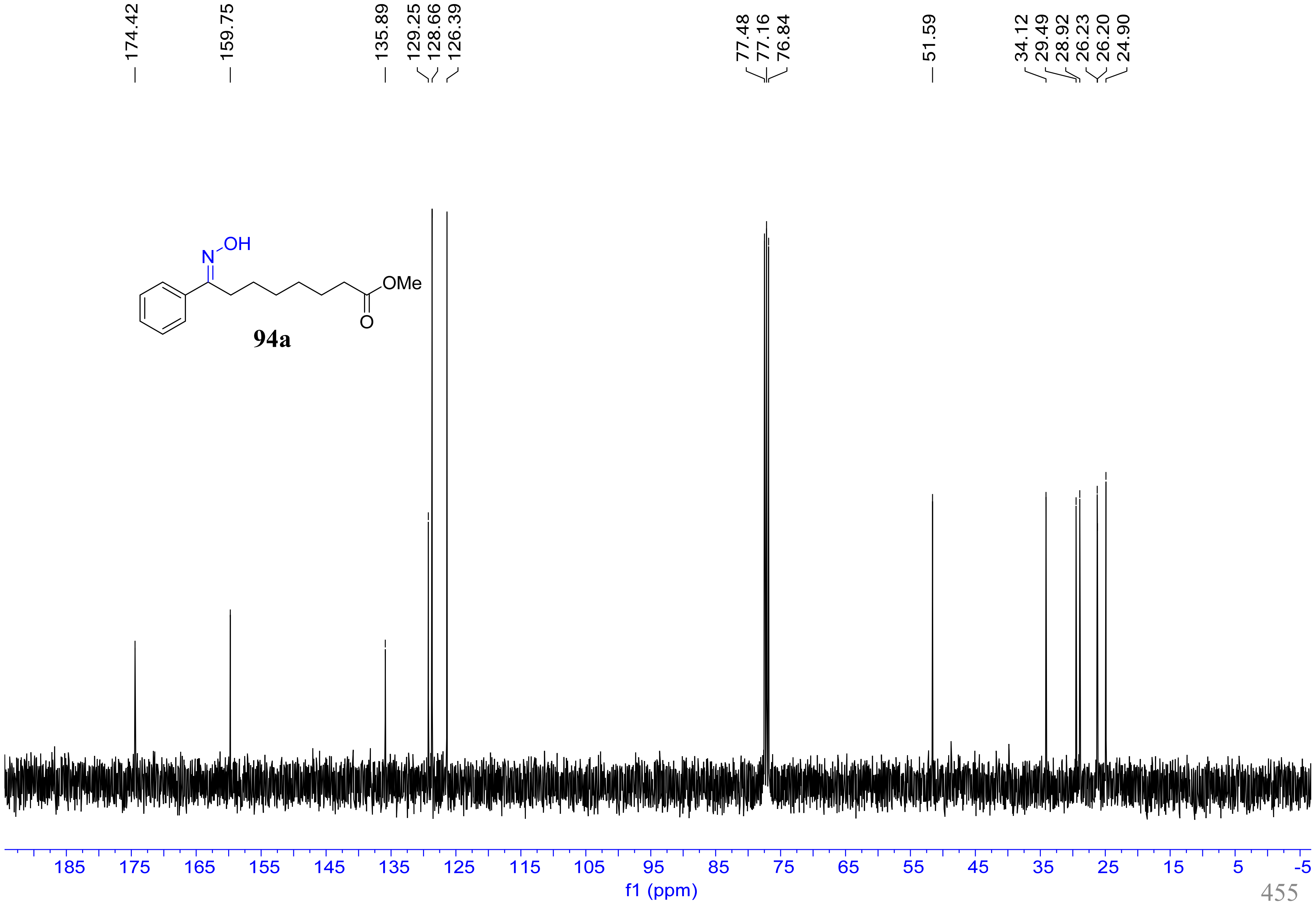

94a

^{1}H NMR, 400 MHz, $CDCl_3$

95a (α-product)

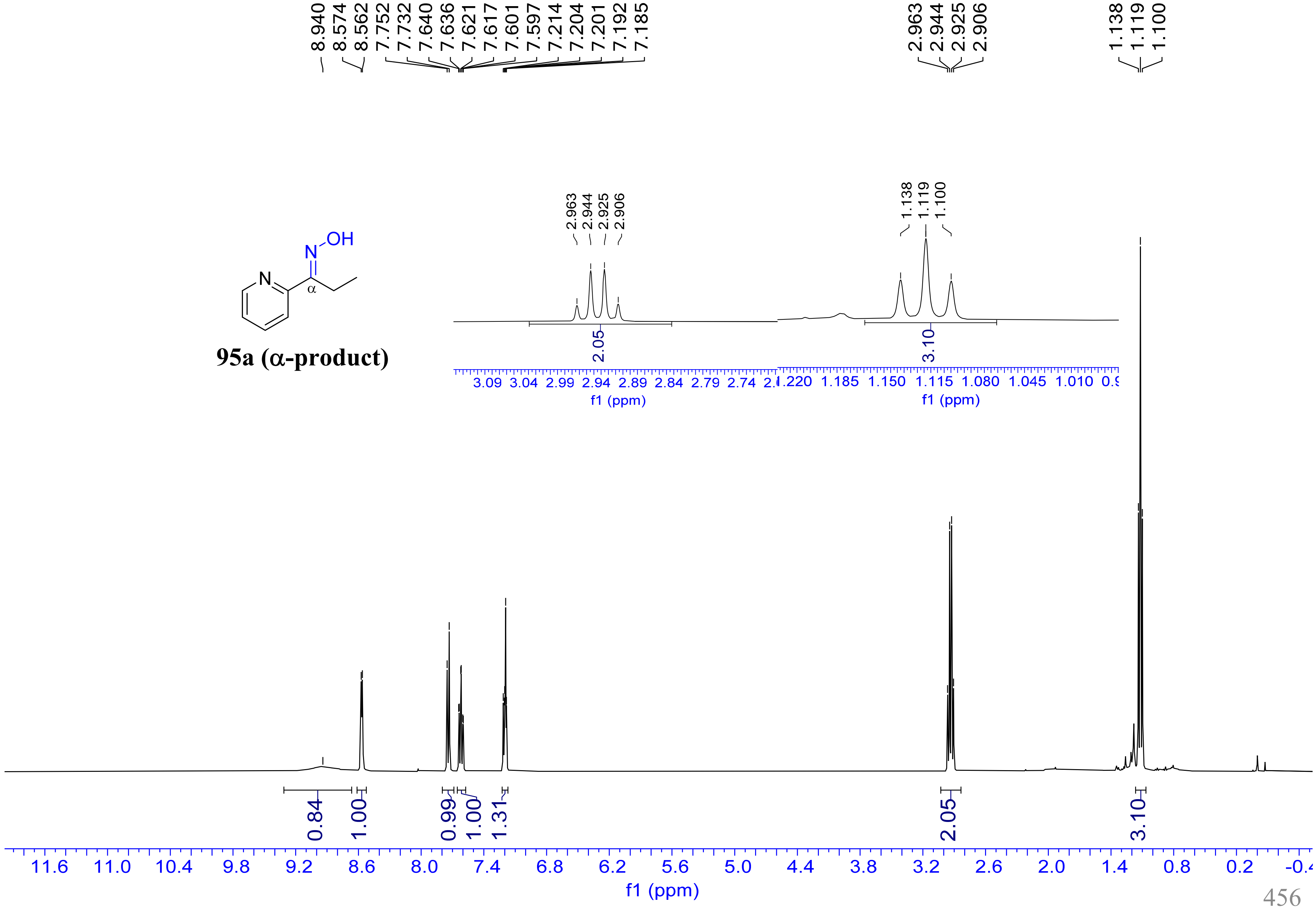

^{13}C NMR 101 MHz, $CDCl_3$

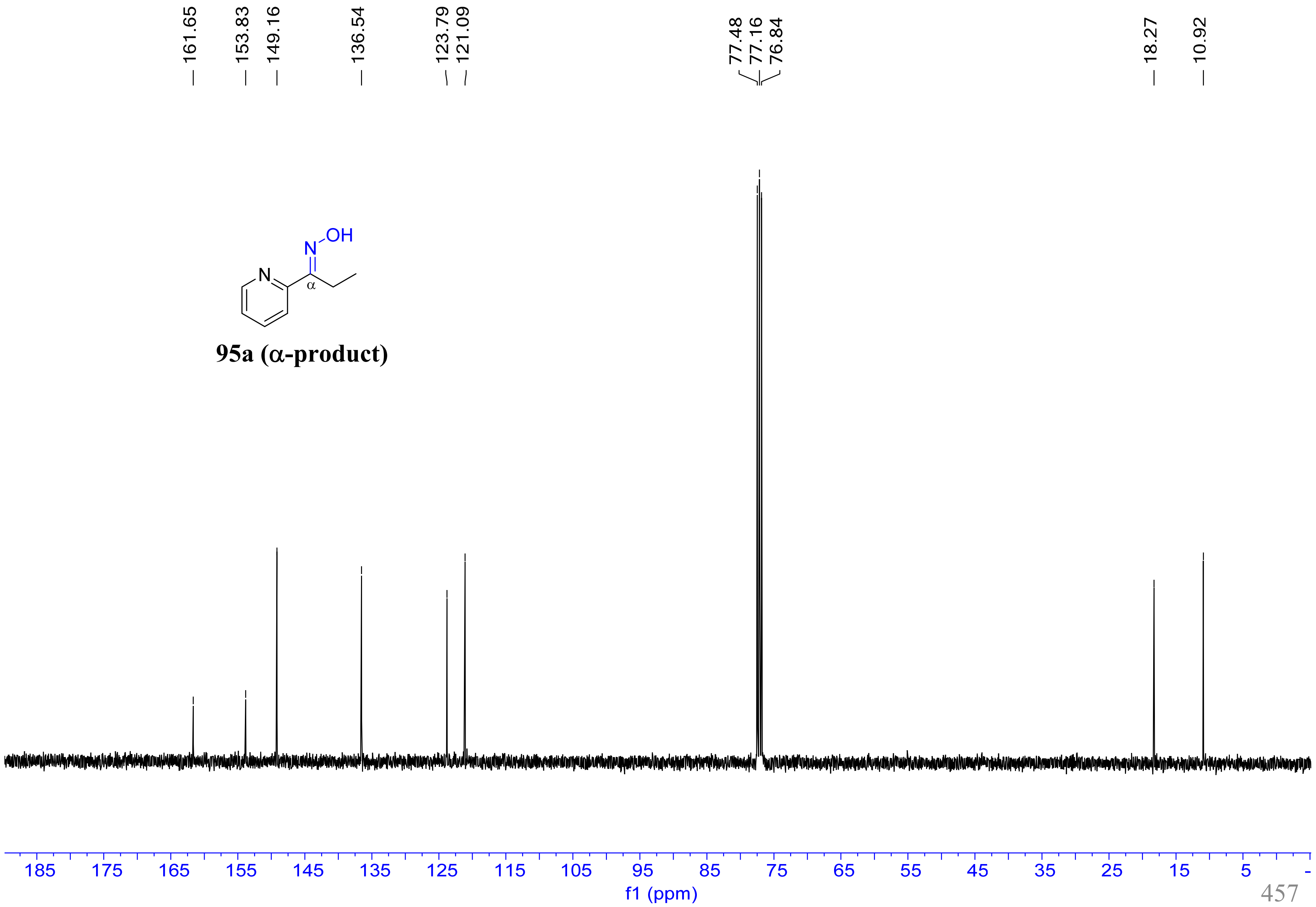

^{1}H NMR, 400 MHz, $CDCl_3$

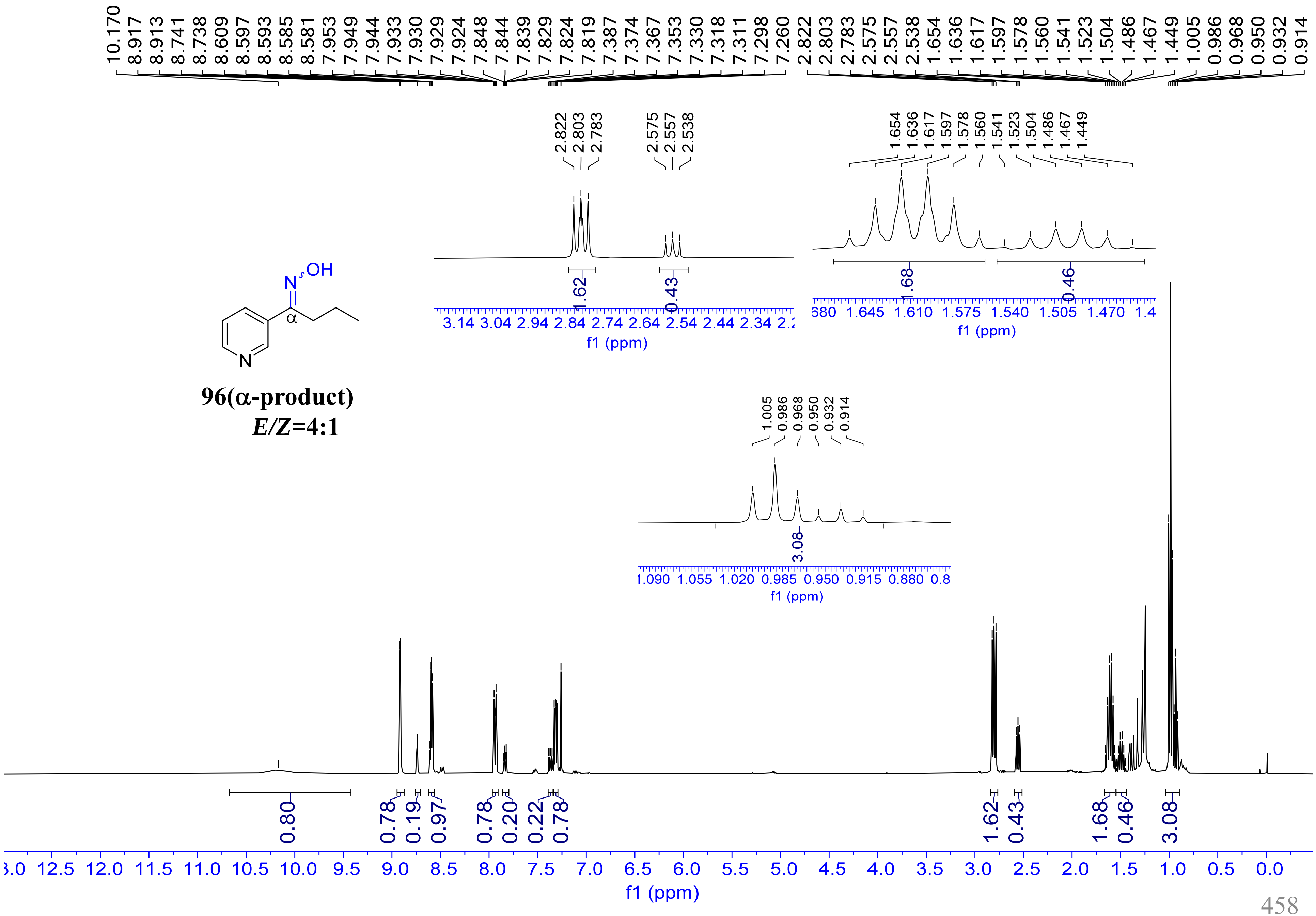

^{13}C NMR 101 MHz, $CDCl_3$

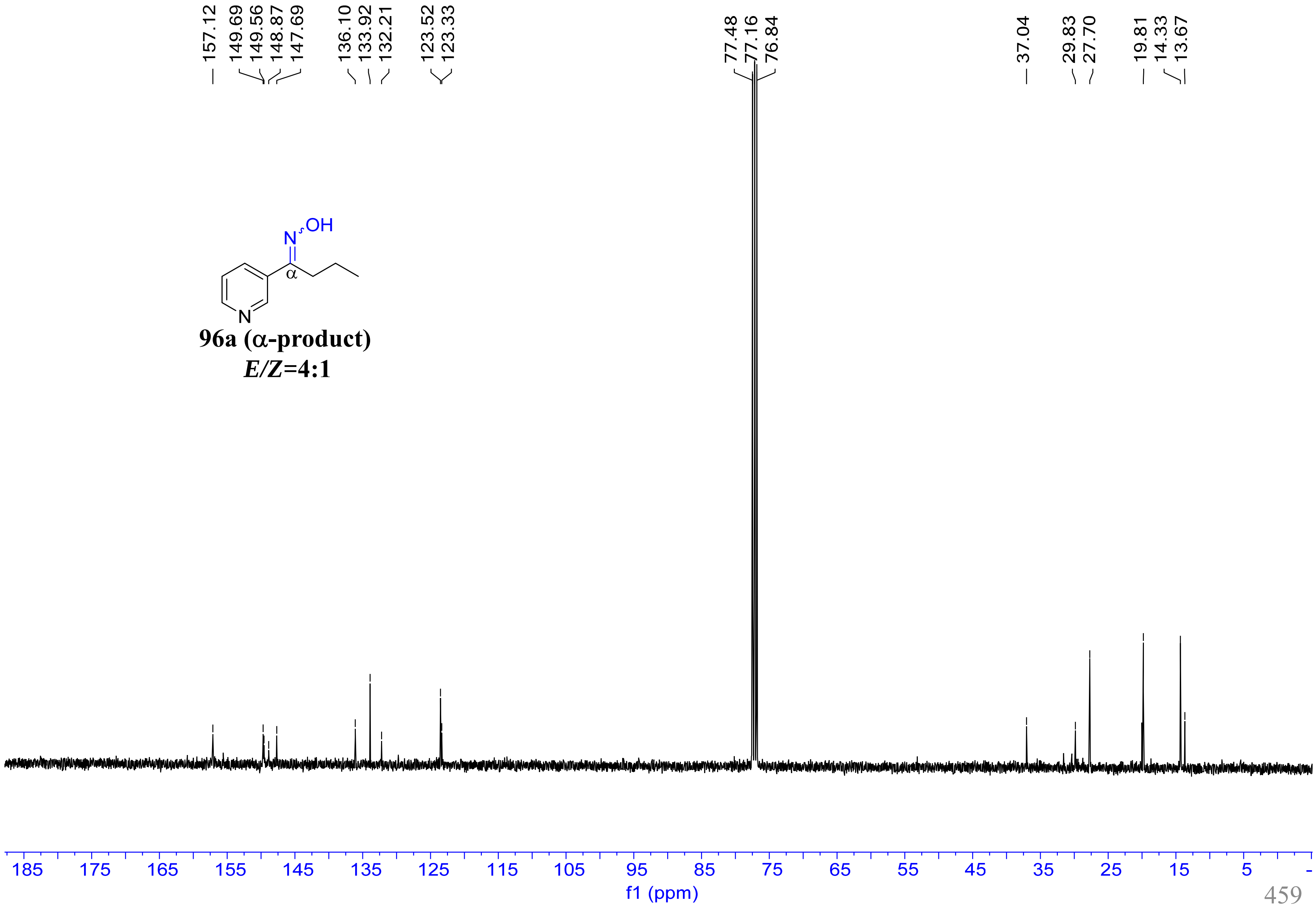

96a (α-product)
***E/Z*=4:1**

^{1}H NMR, 400 MHz, $CDCl_3$

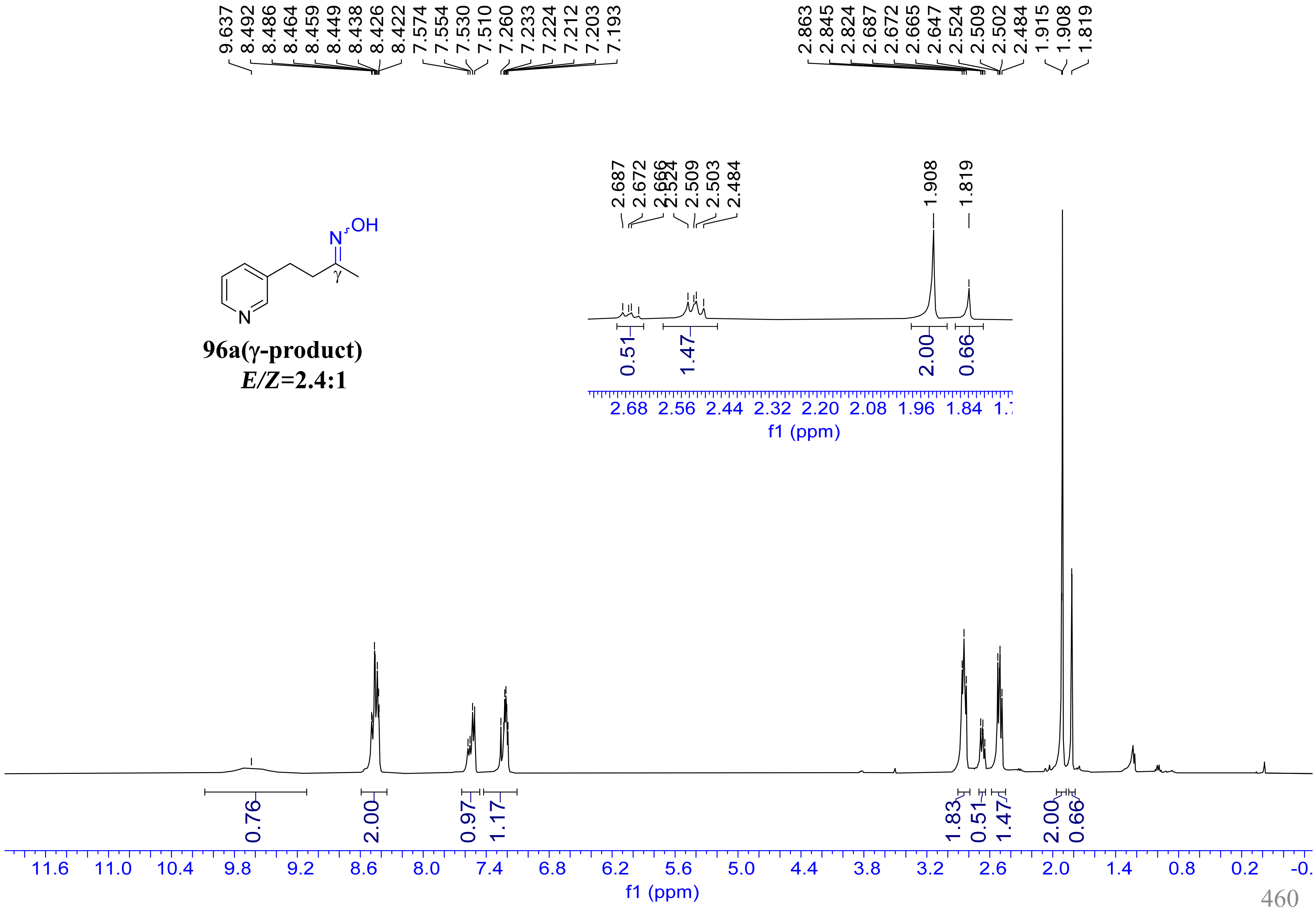

^{13}C NMR 101 MHz, CDCl$_3$

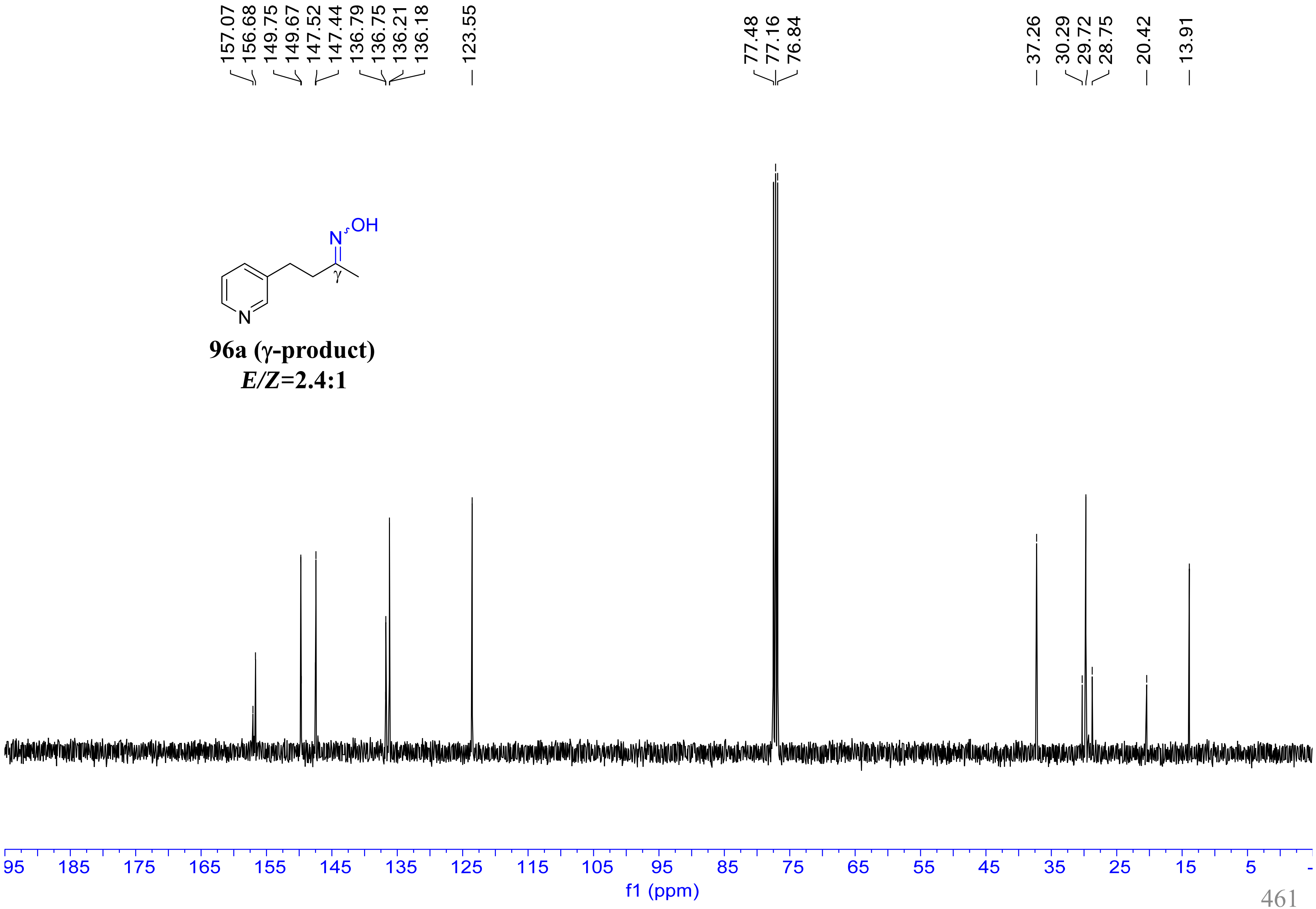

96a (γ-product)
***E/Z*=2.4:1**

^{1}H NMR, 400 MHz, DMSO-d6

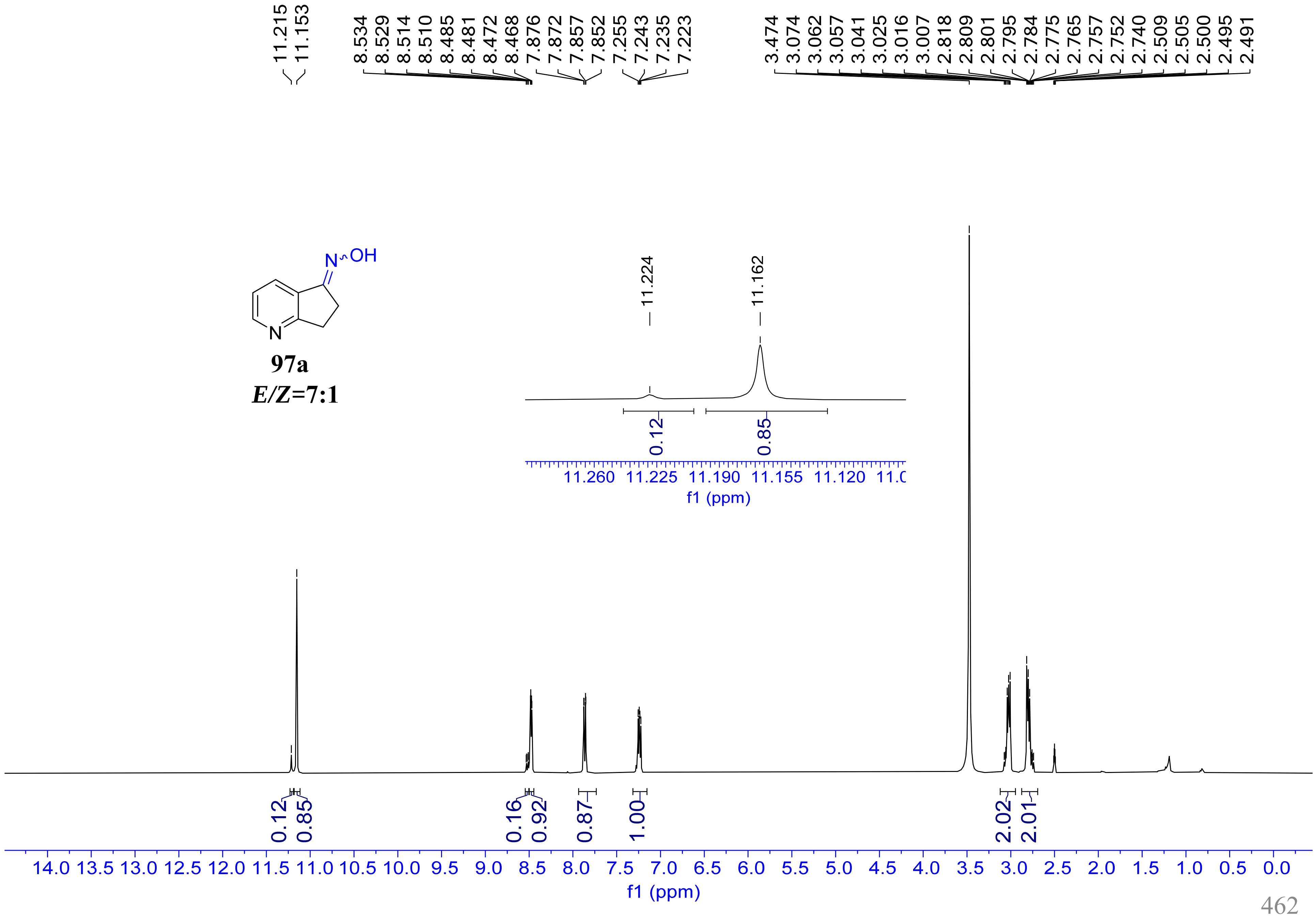

^{13}C NMR 101 MHz, DMSO-d6

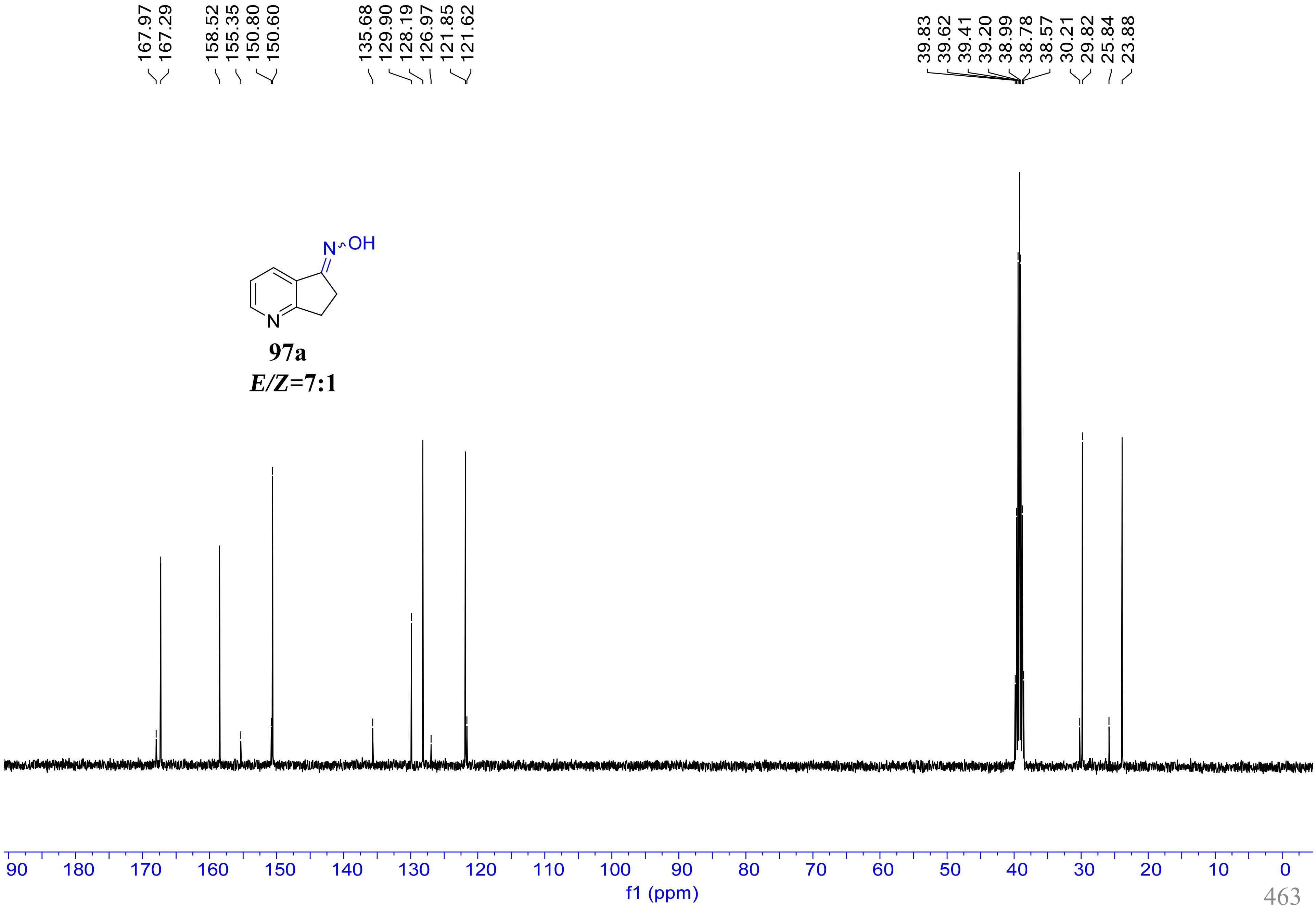

97a

***E/Z*=7:1**

^{1}H NMR, 400 MHz, DMSO-d6

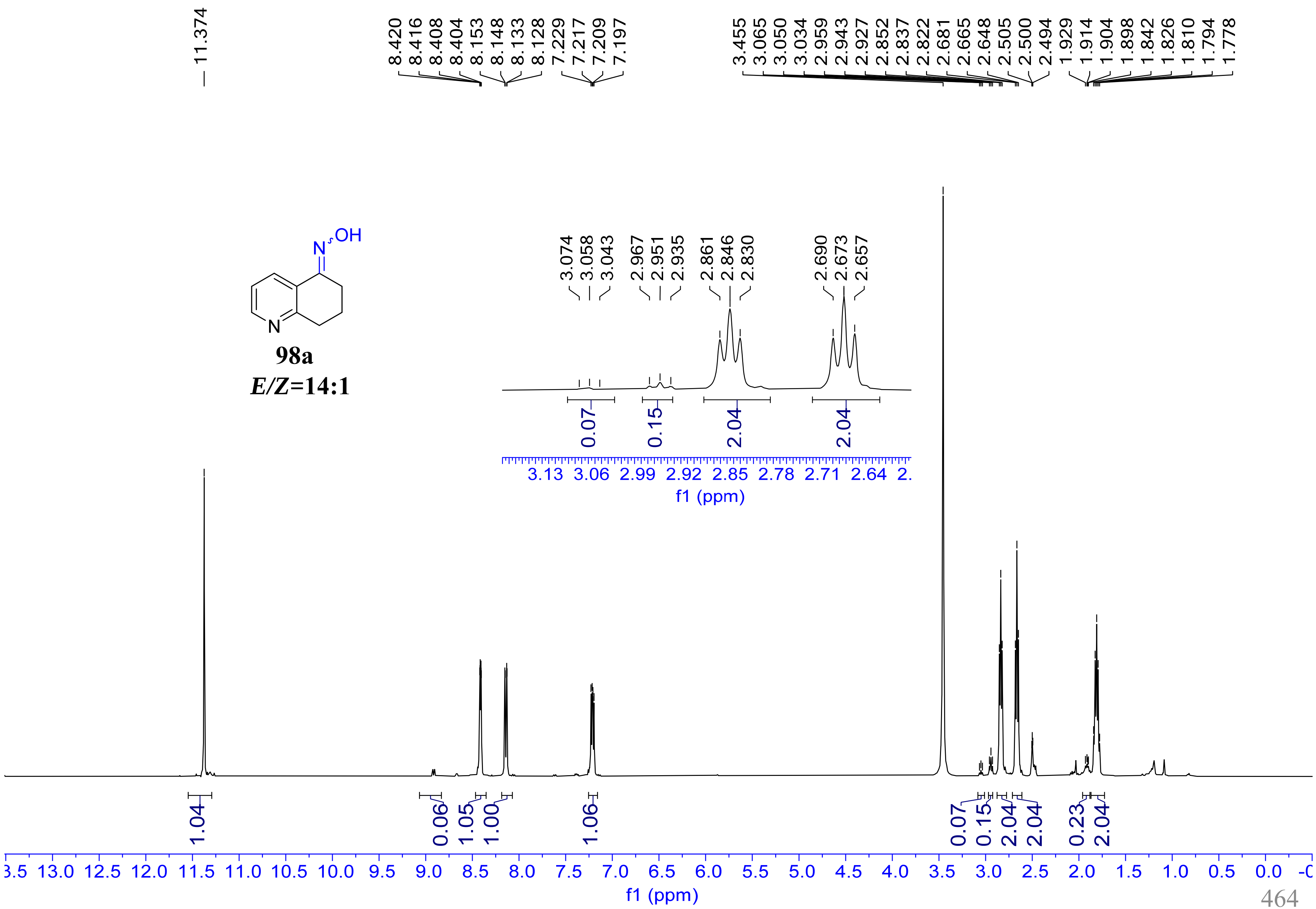

^{13}C NMR 101 MHz, DMSO-d6

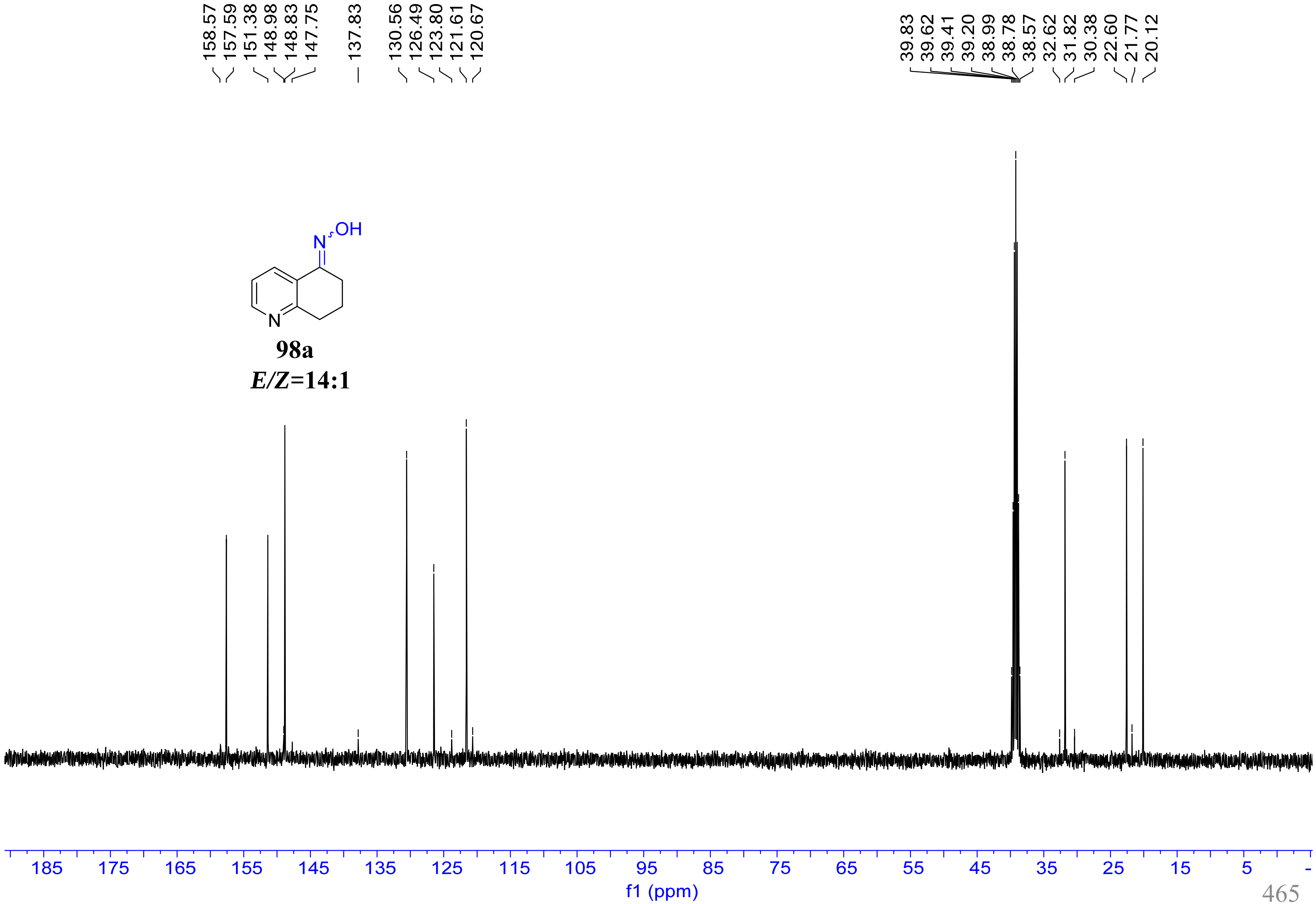

^{1}H NMR, 400 MHz, DMSO-d6

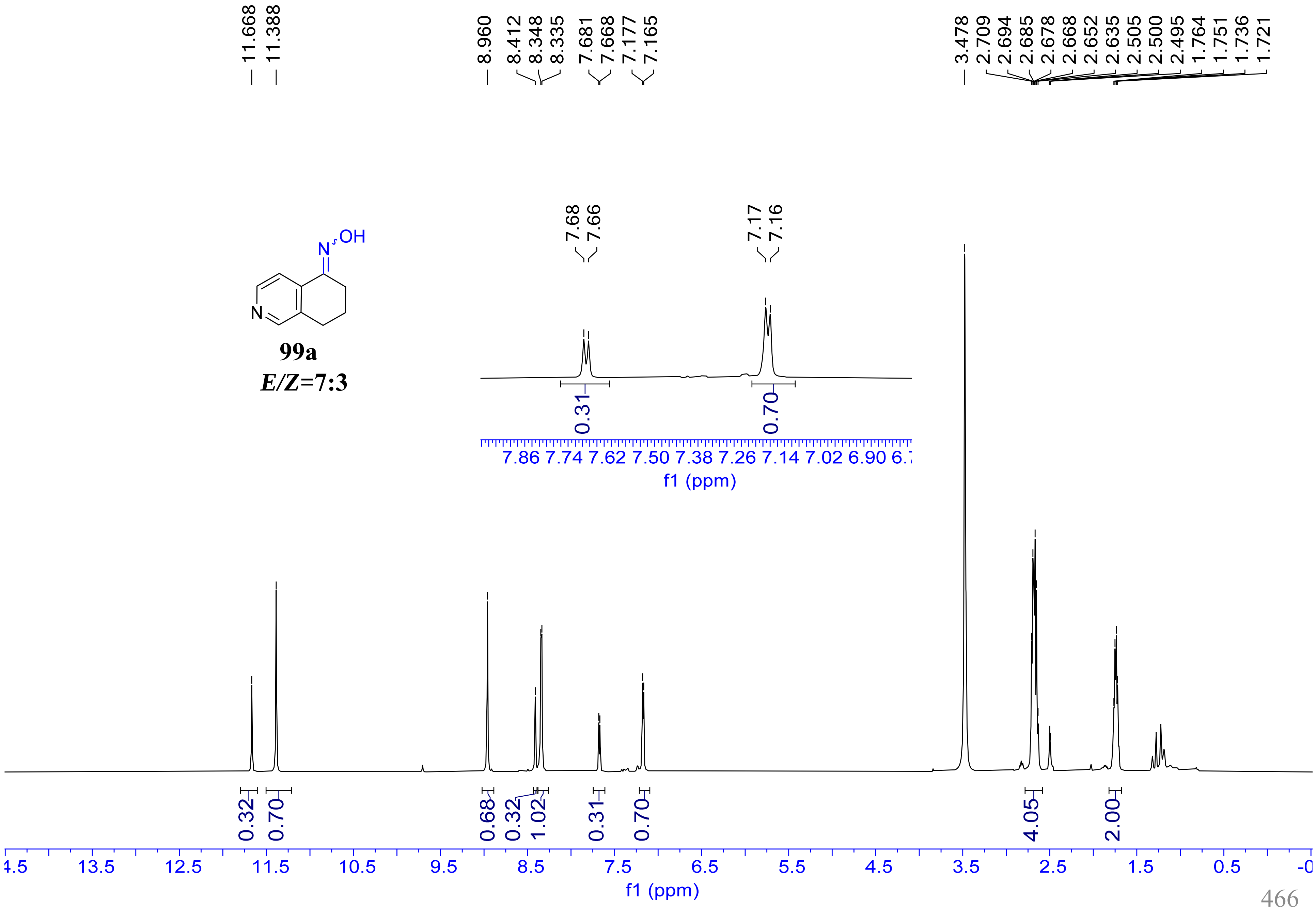

^{13}C NMR 101 MHz, DMSO-d6

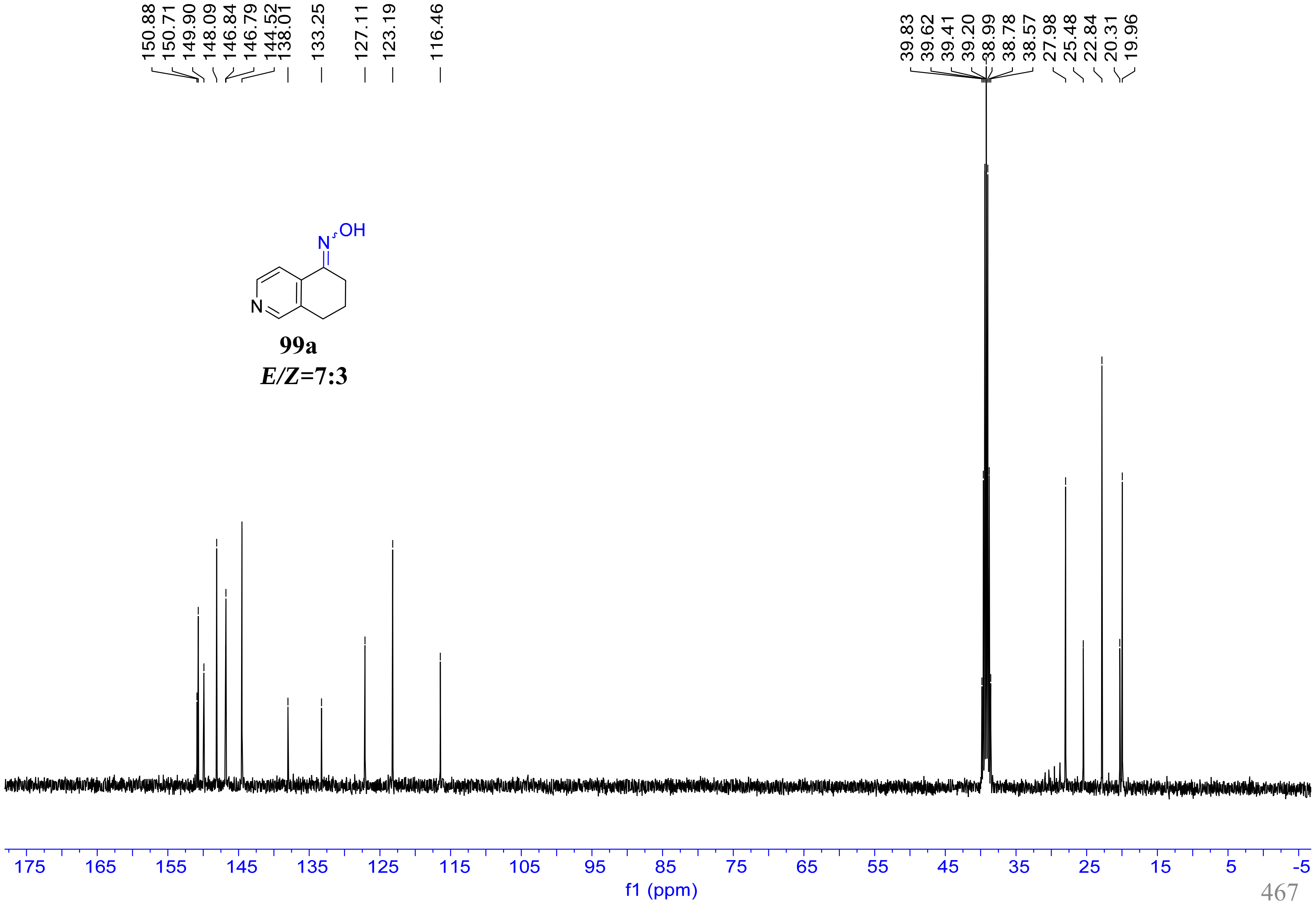

99a

***E/Z*=7:3**

^{1}H NMR, 400 MHz, $CDCl_3$

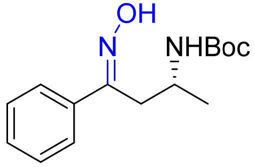

100a

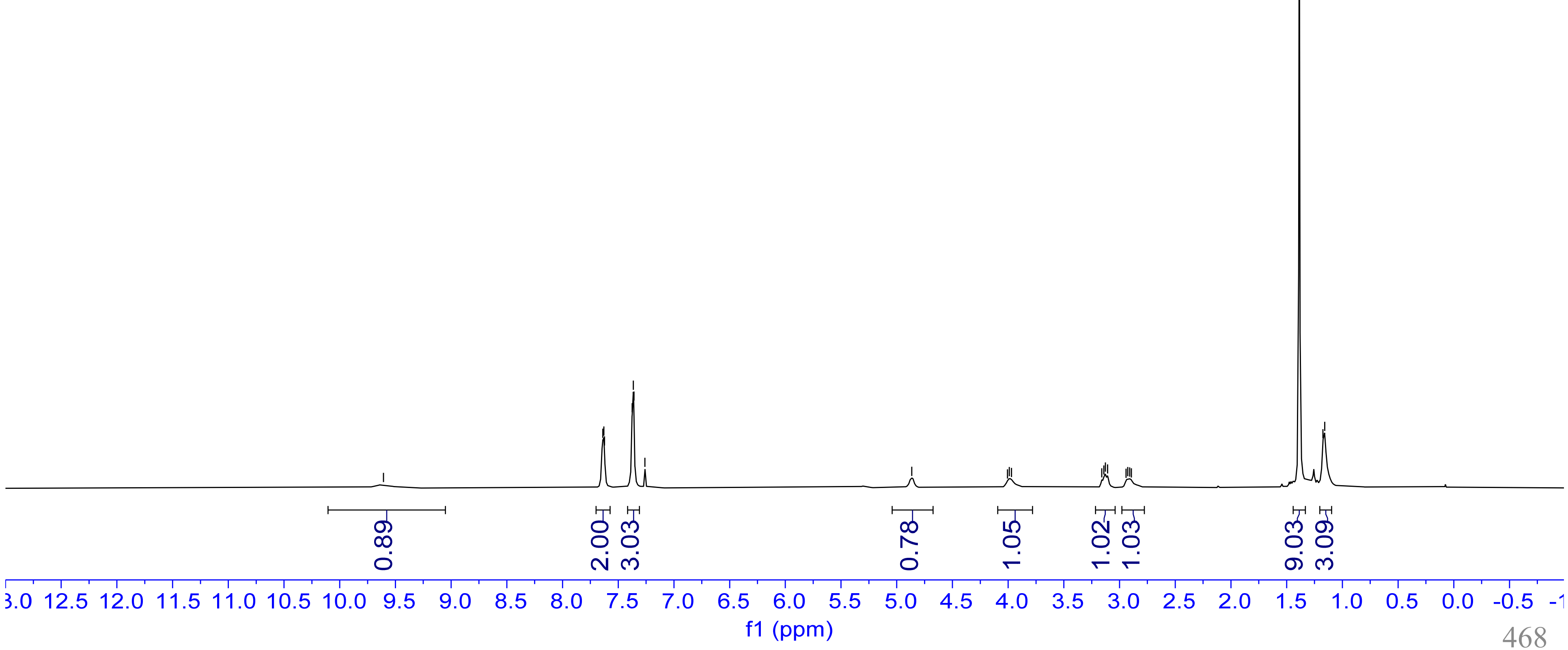

^{13}C NMR 101 MHz, $CDCl_3$

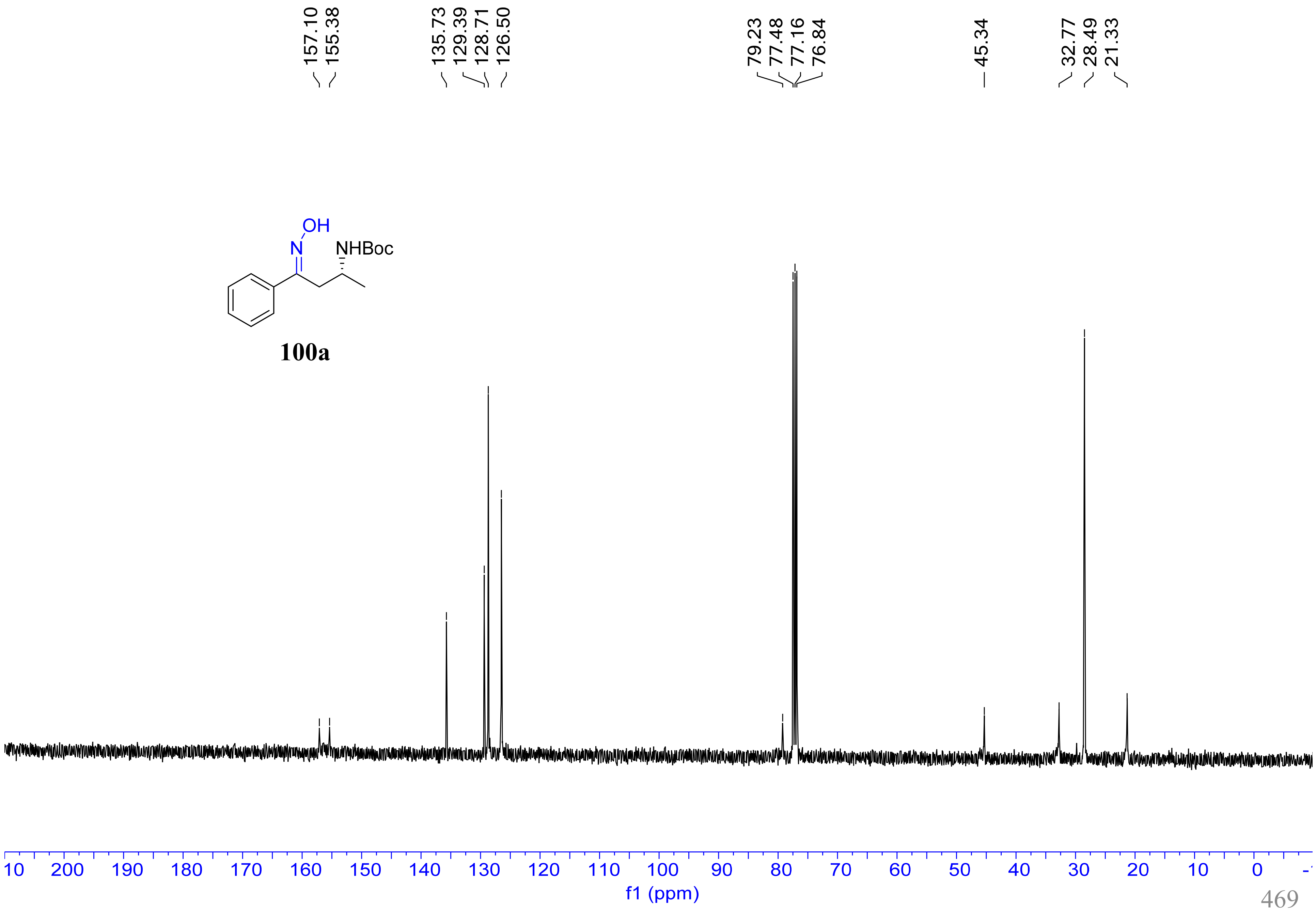

100a

^{1}H NMR, 400 MHz, $CDCl_3$

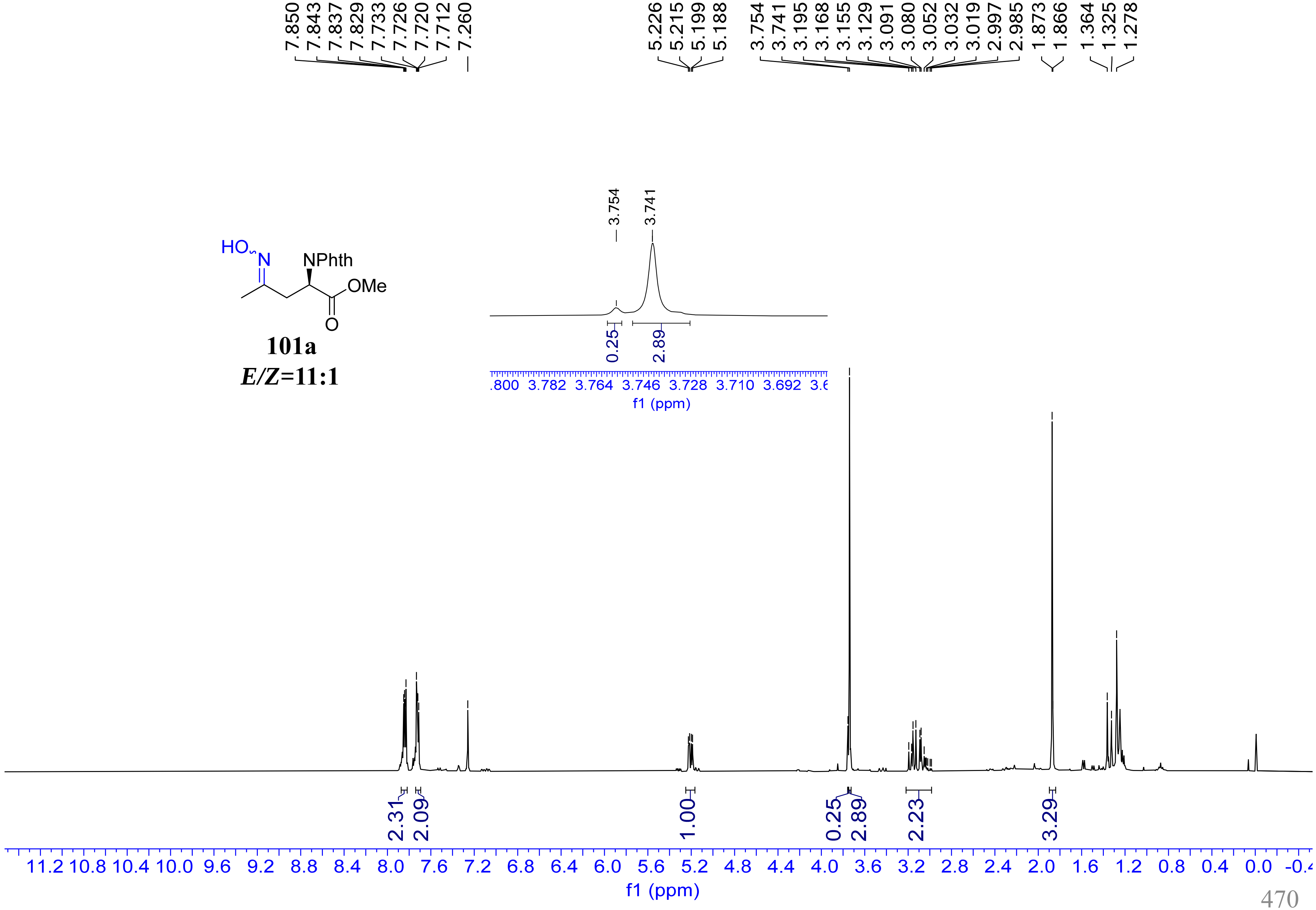

101a

***E/Z*=11:1**

^{13}C NMR 101 MHz, $CDCl_3$

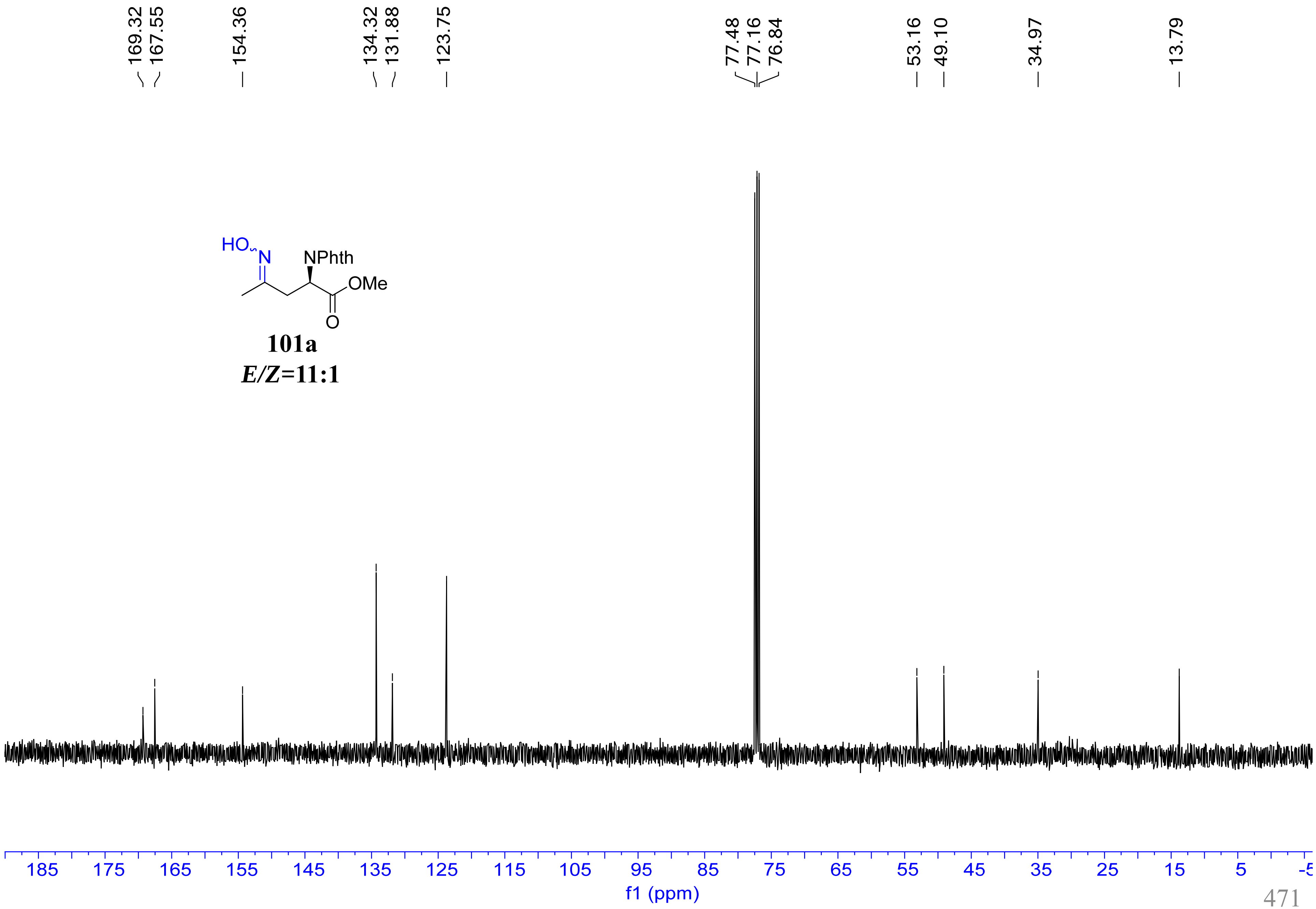

101a

***E*/*Z*=11:1**

^{1}H NMR, 400 MHz, $CDCl_3$

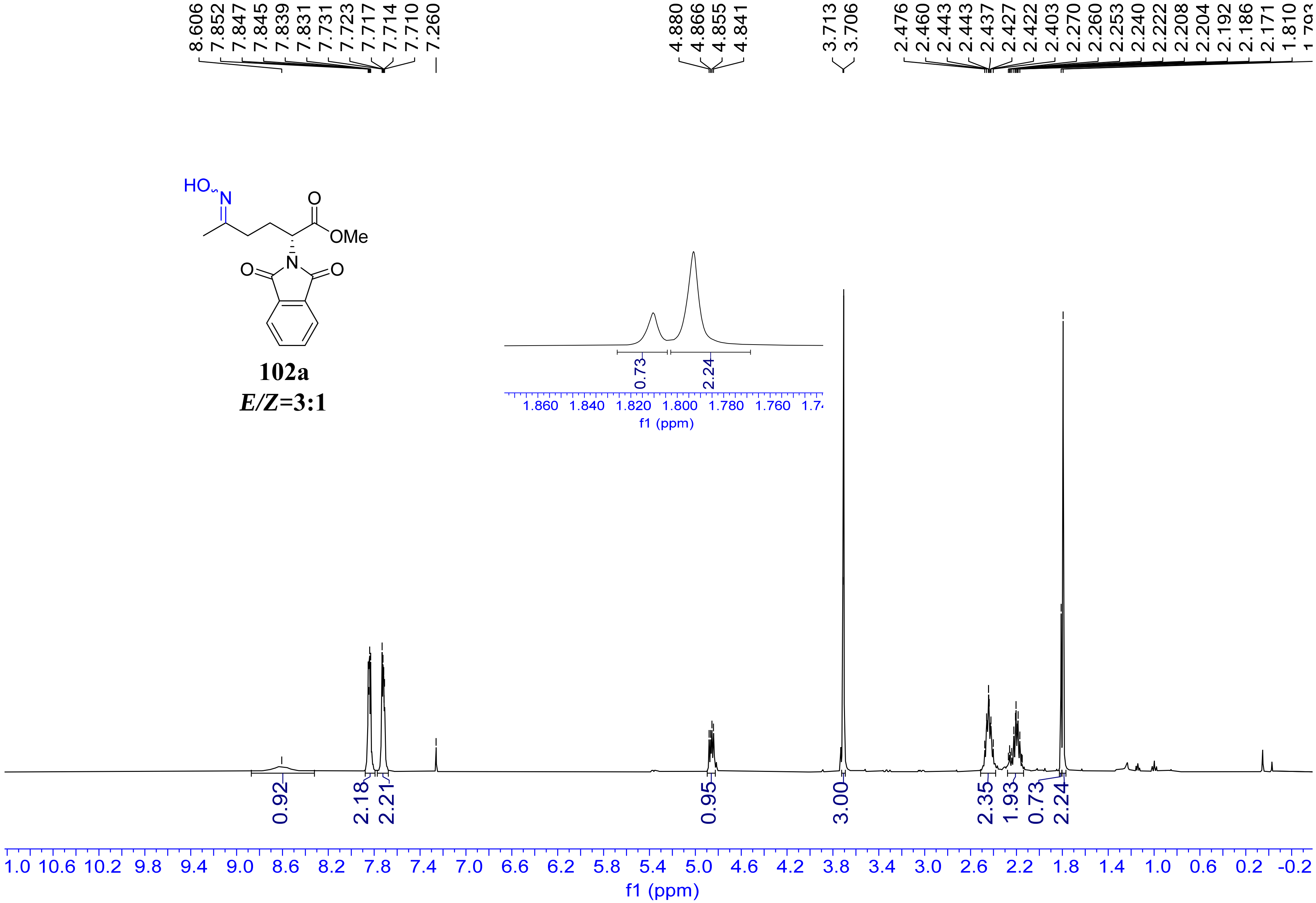

102a

E/*Z*=3:1

^{13}C NMR 101 MHz, $CDCl_3$

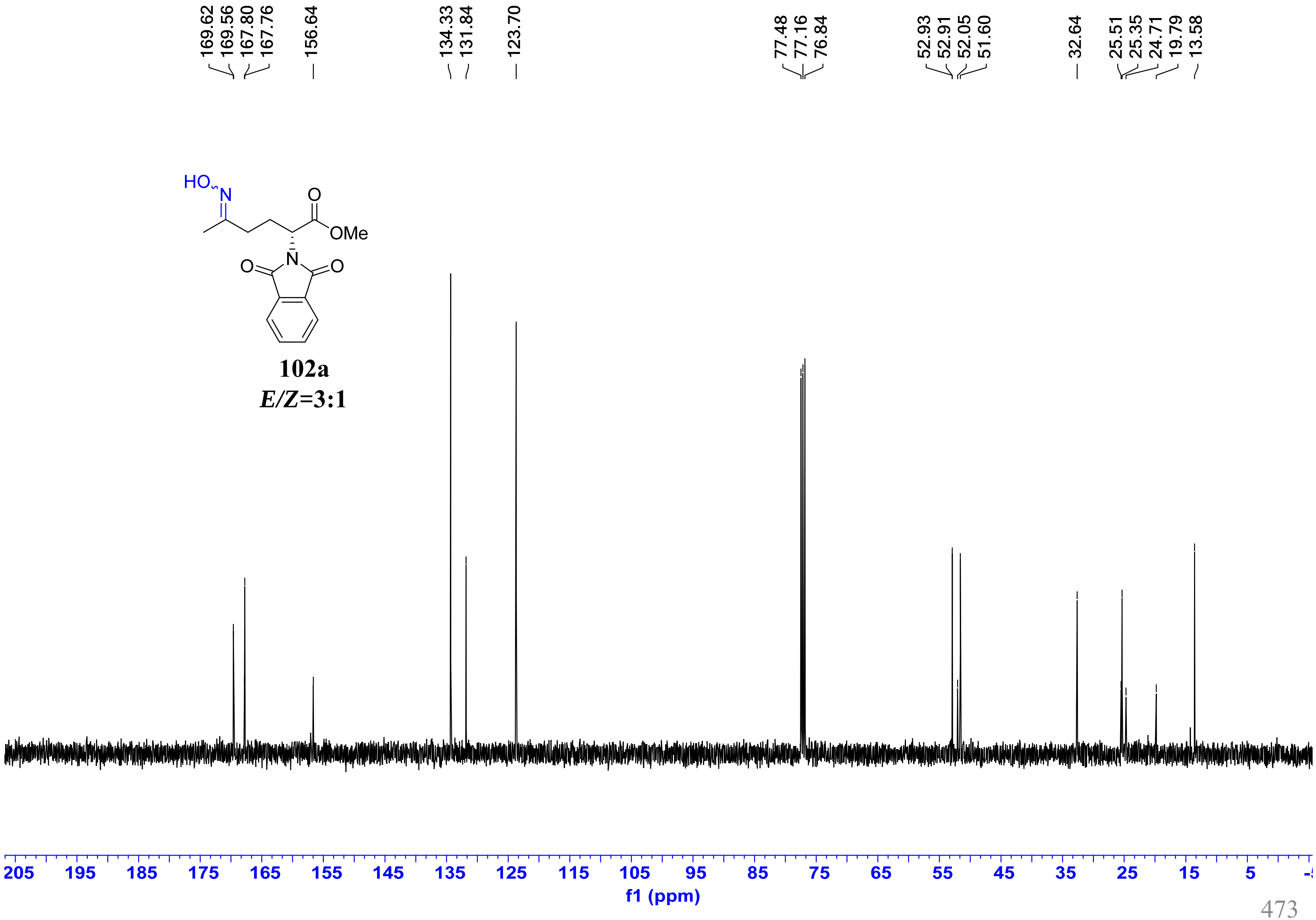

102a

***E*/*Z*=3:1**

^1H NMR, 400 MHz, $CDCl_3$

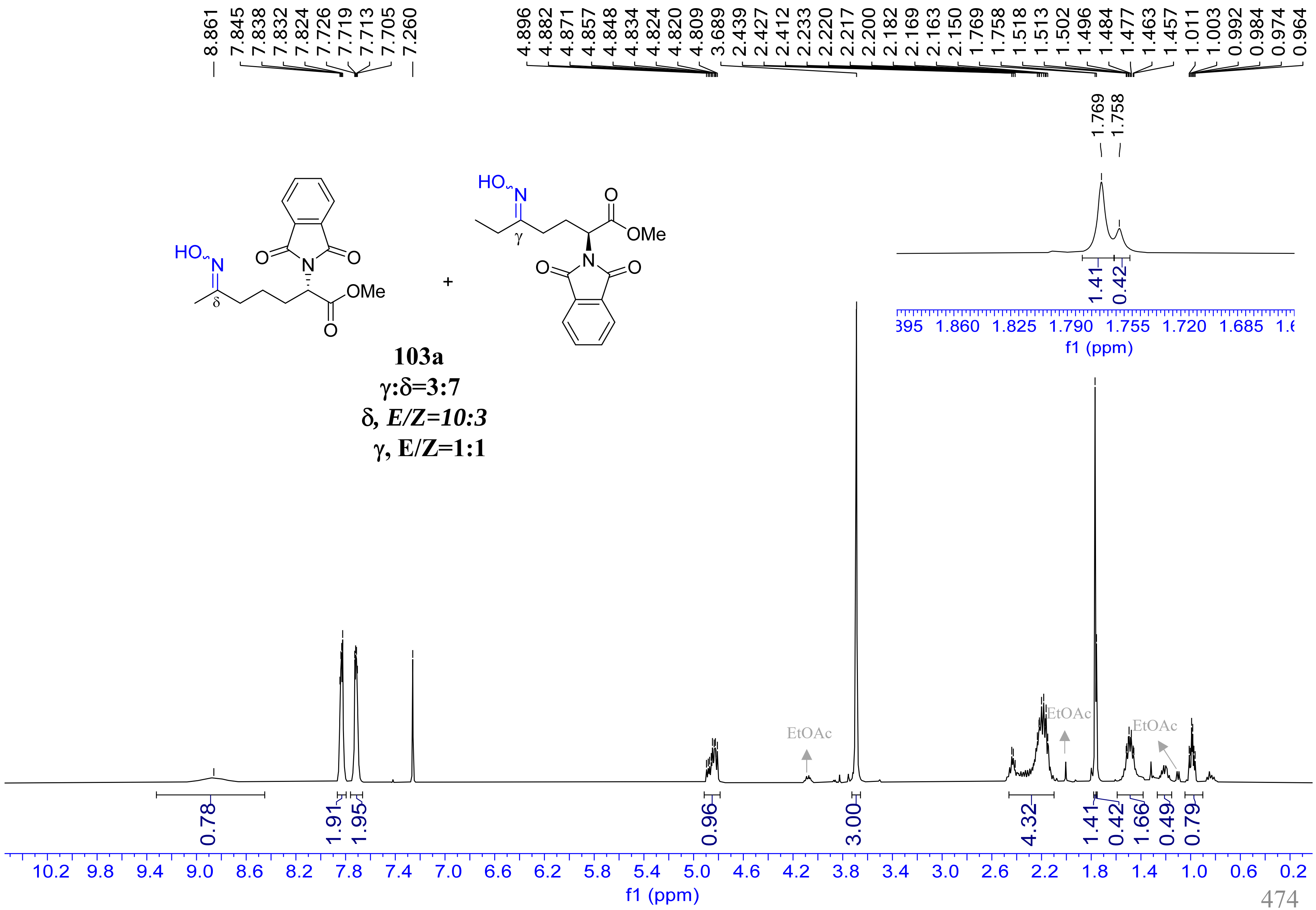

^{13}C NMR 101 MHz, $CDCl_3$

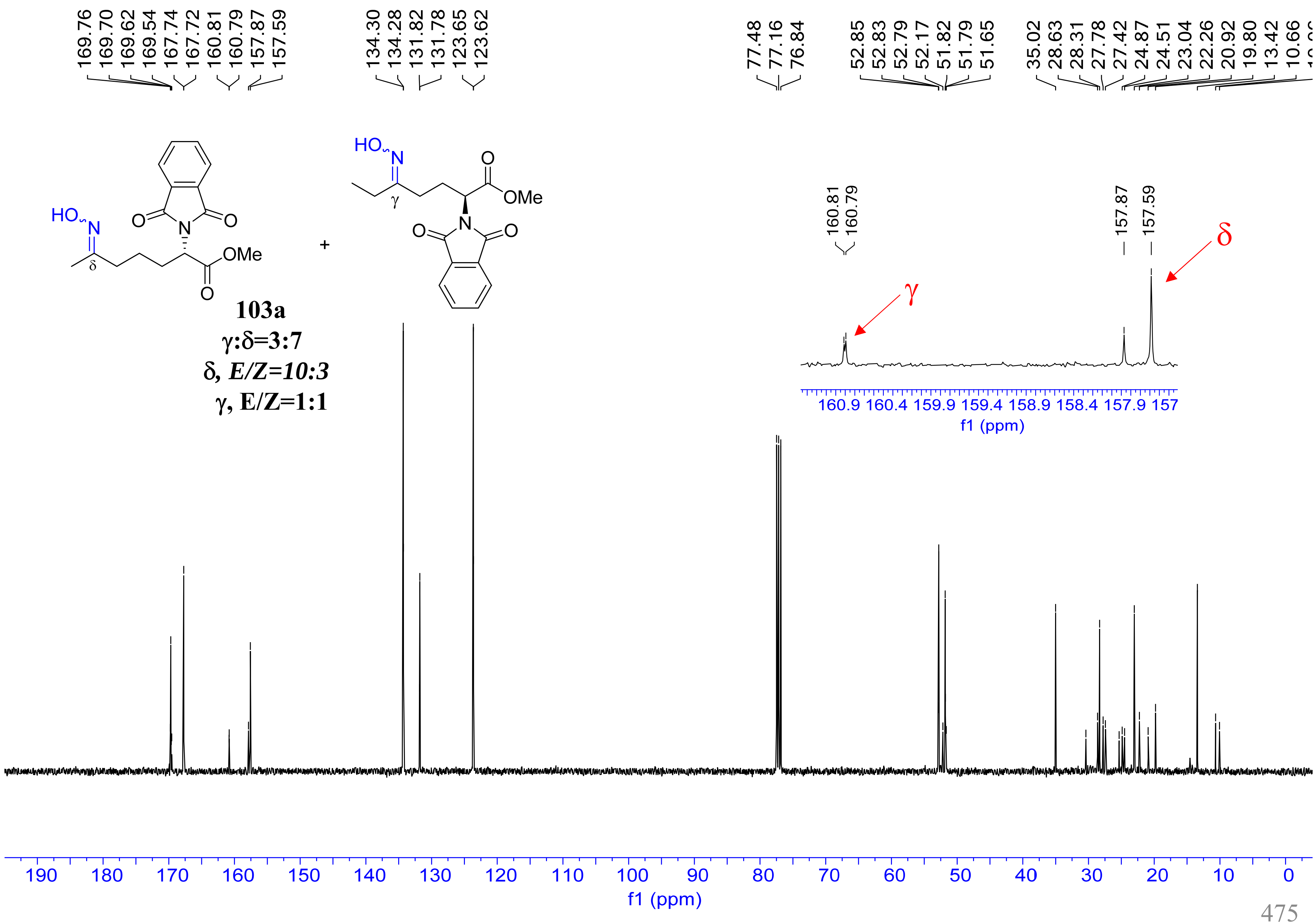

^{1}H NMR, 400 MHz, $CDCl_3$

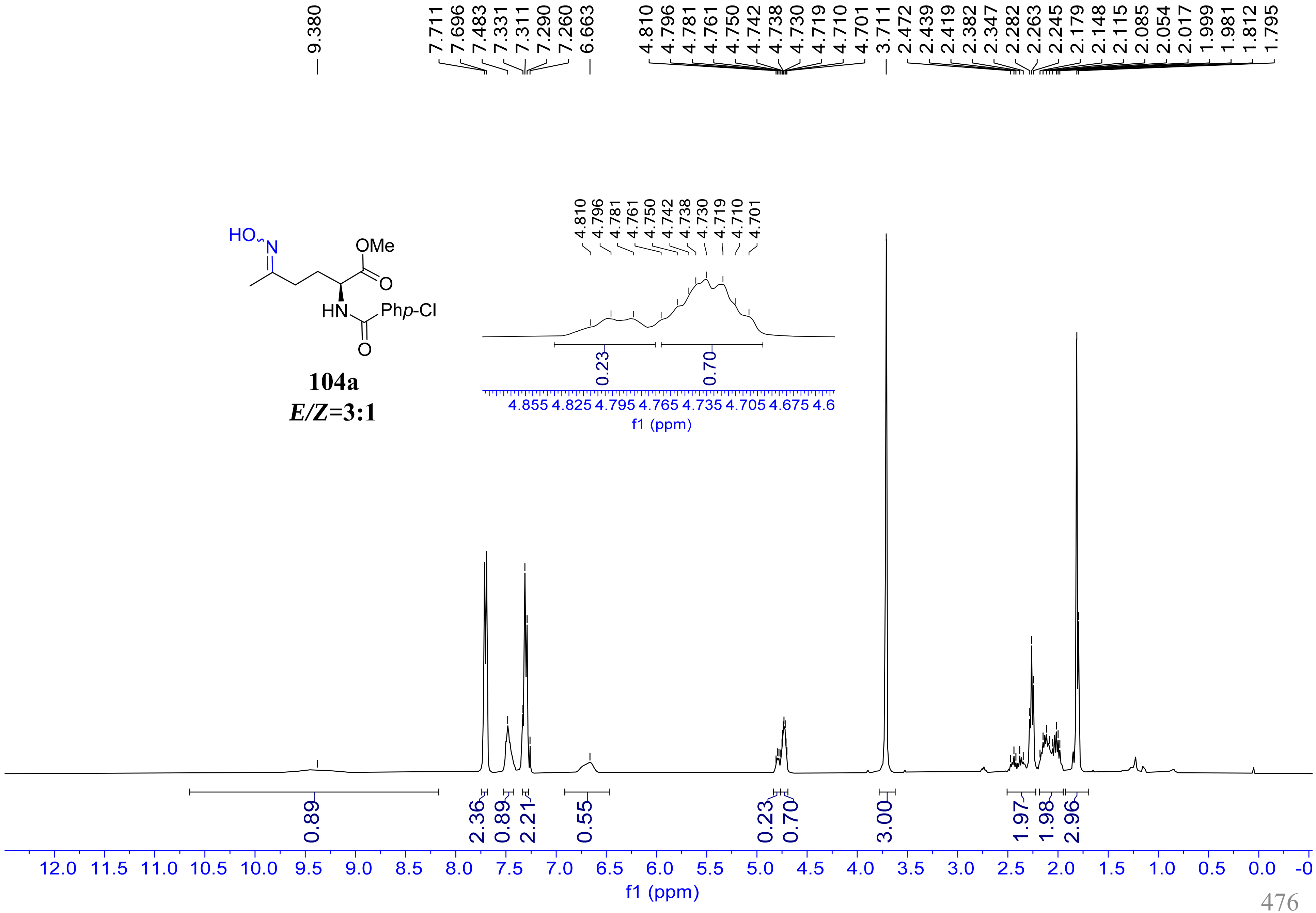

^{13}C NMR 101 MHz, CDCl$_3$

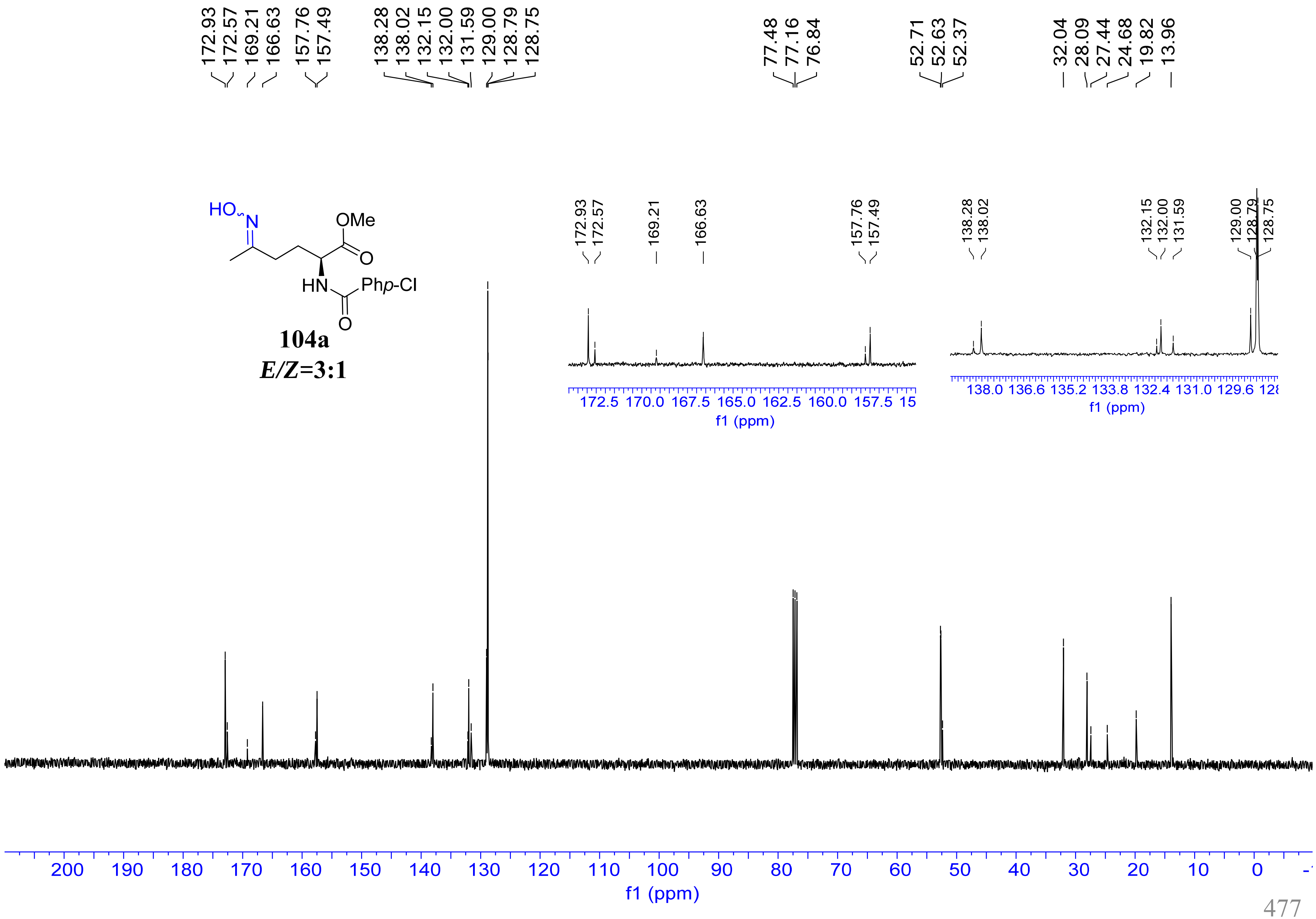

104a

***E/Z*=3:1**

^{1}H NMR, 400 MHz, $CDCl_3$

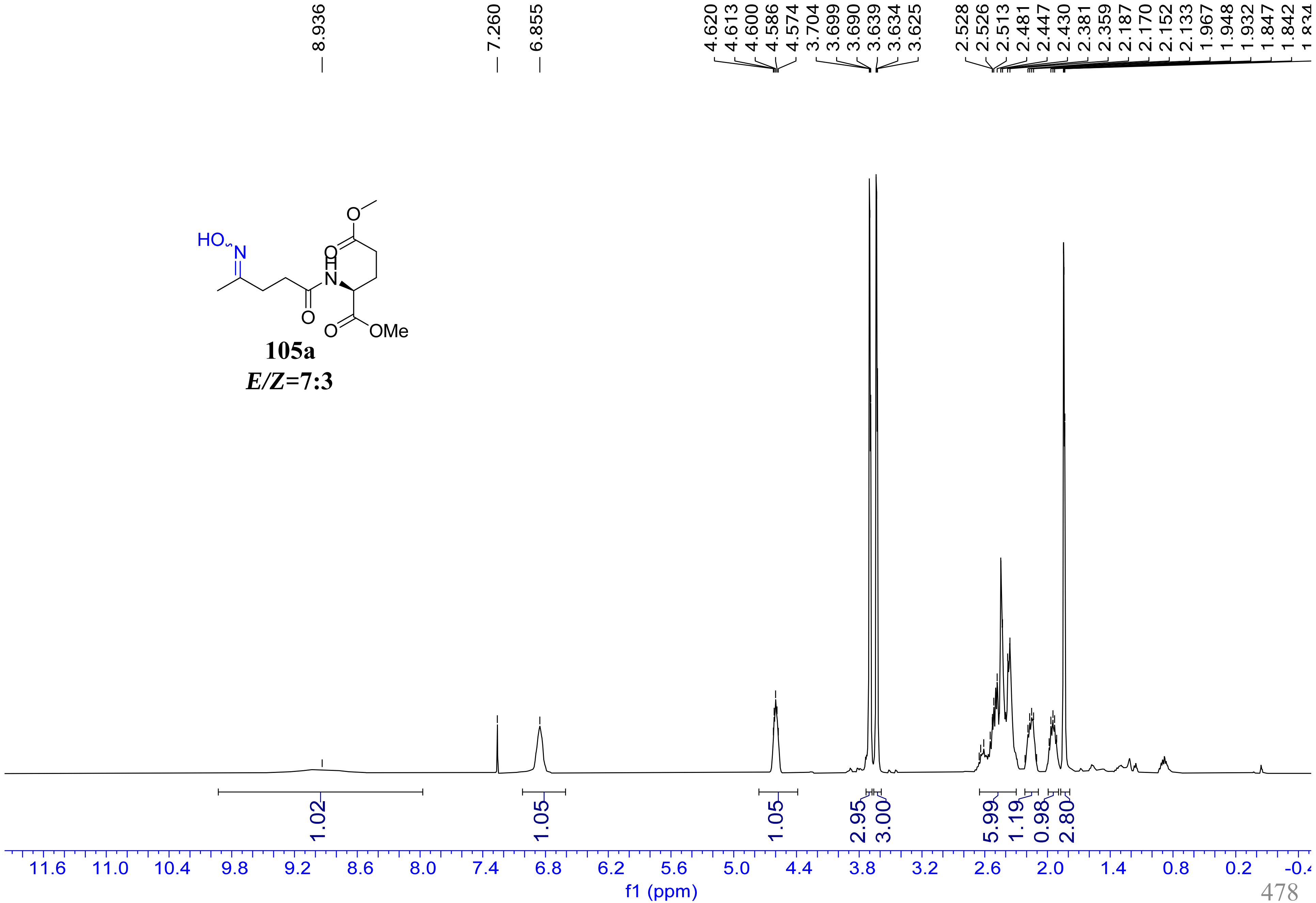

105a

***E/Z*=7:3**

^{13}C NMR 101 MHz, CDCl$_3$

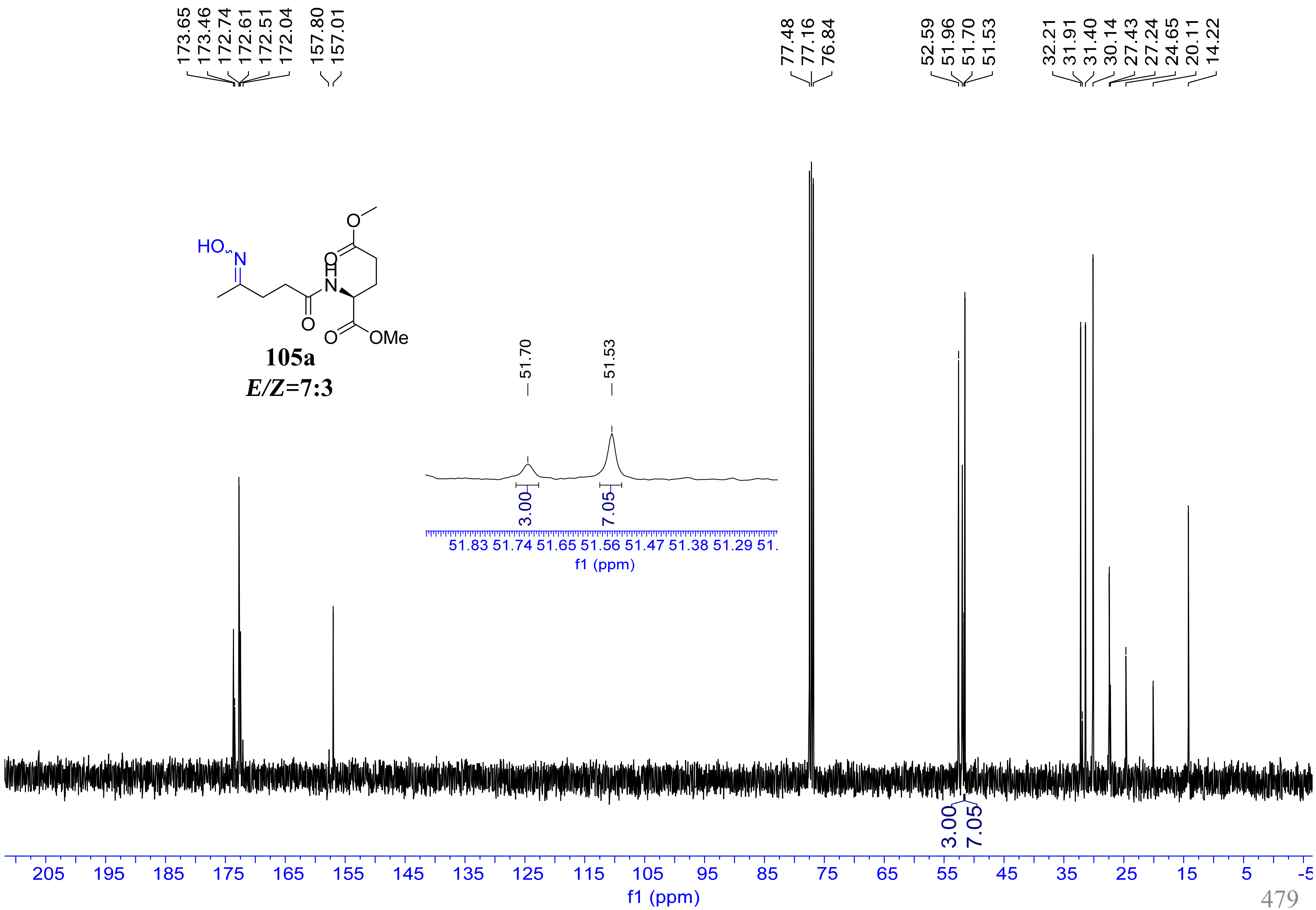

^{1}H NMR, 400 MHz, $CDCl_3$

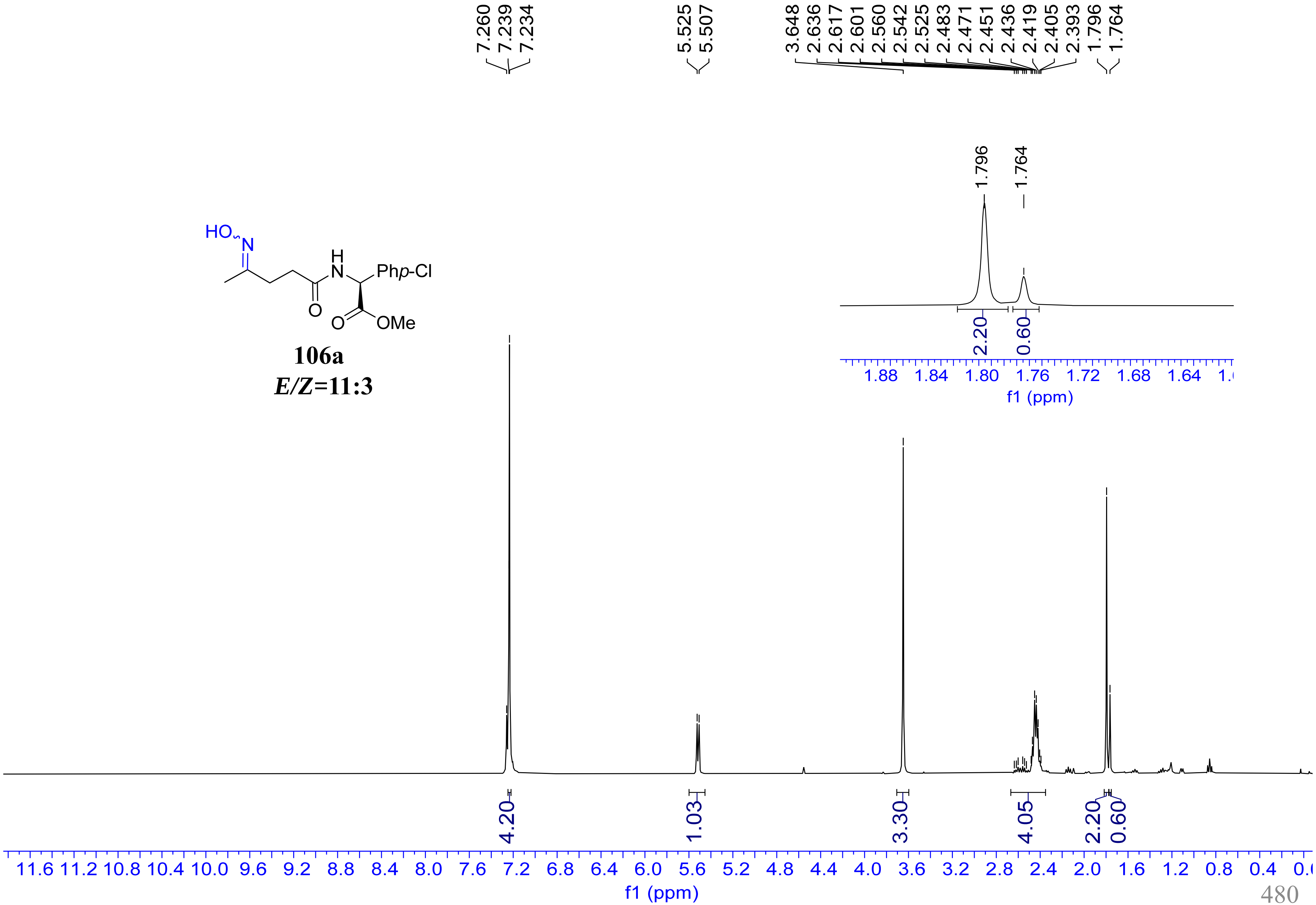

^{13}C NMR 101 MHz, $CDCl_3$

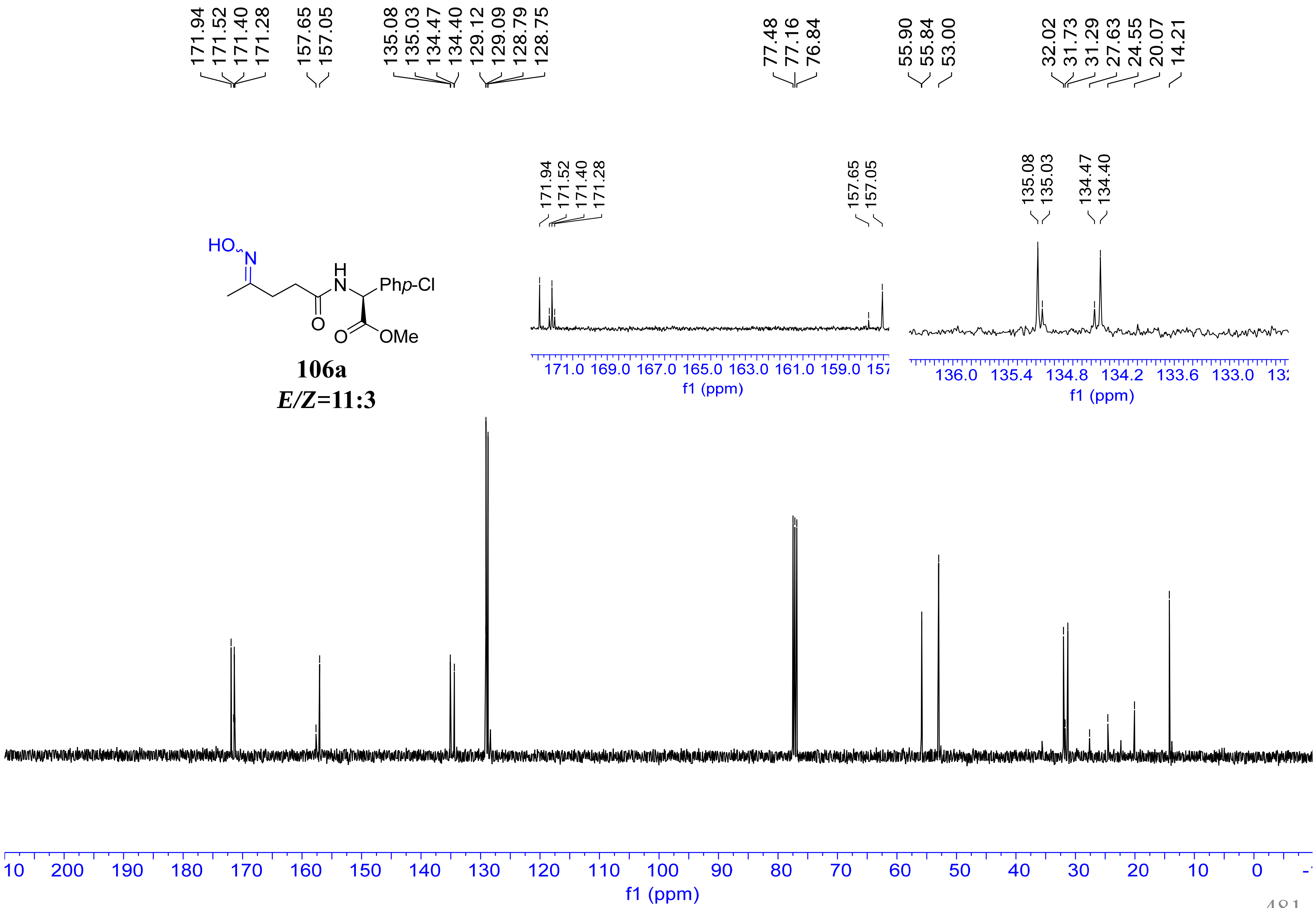

106a

***E*/*Z*=11:3**

¹H NMR, 400 MHz, $CDCl_3$

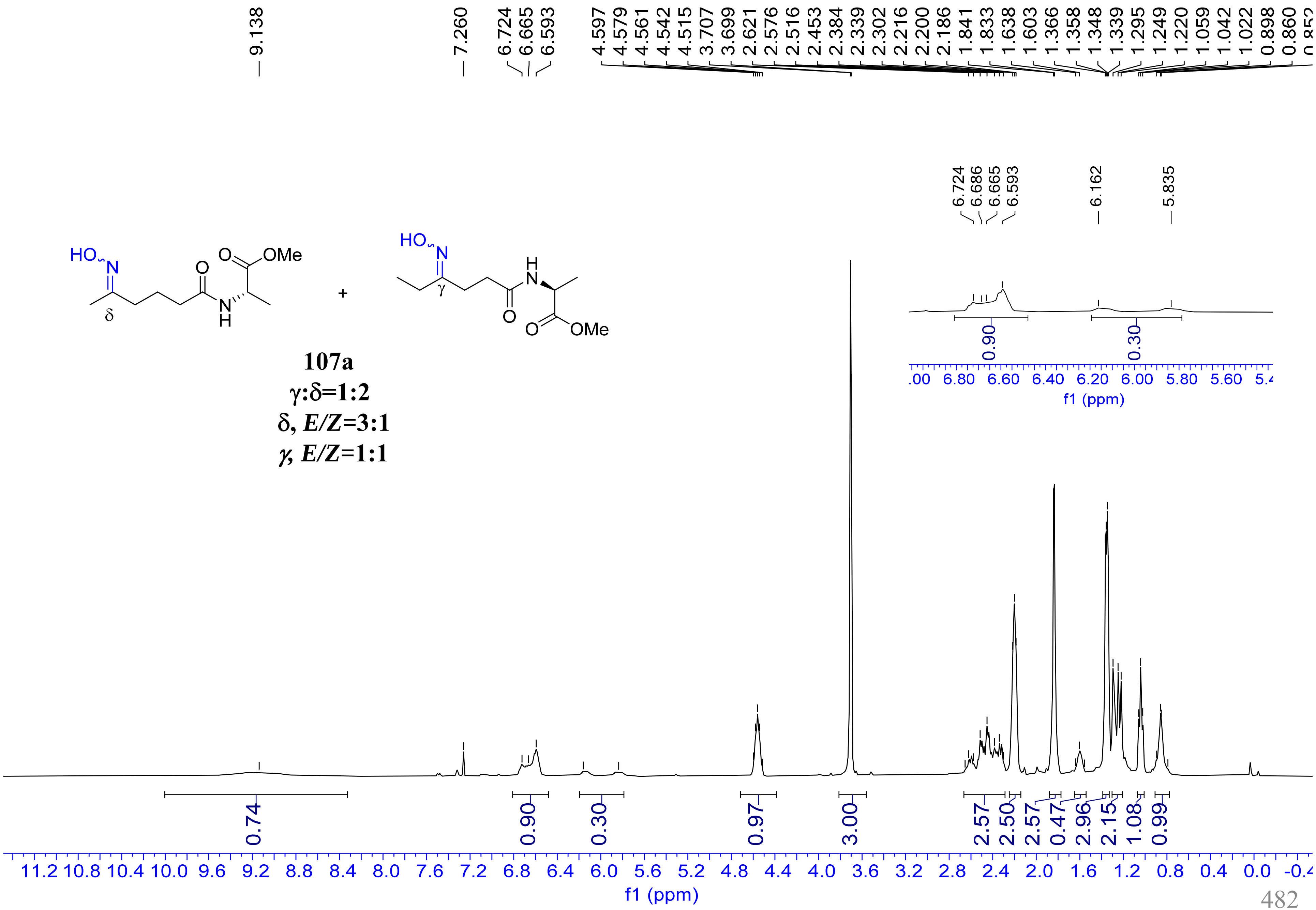

107a

γ:δ=1:2

δ, *E/Z*=3:1

γ, *E/Z*=1:1

^{13}C NMR 101 MHz, $CDCl_3$

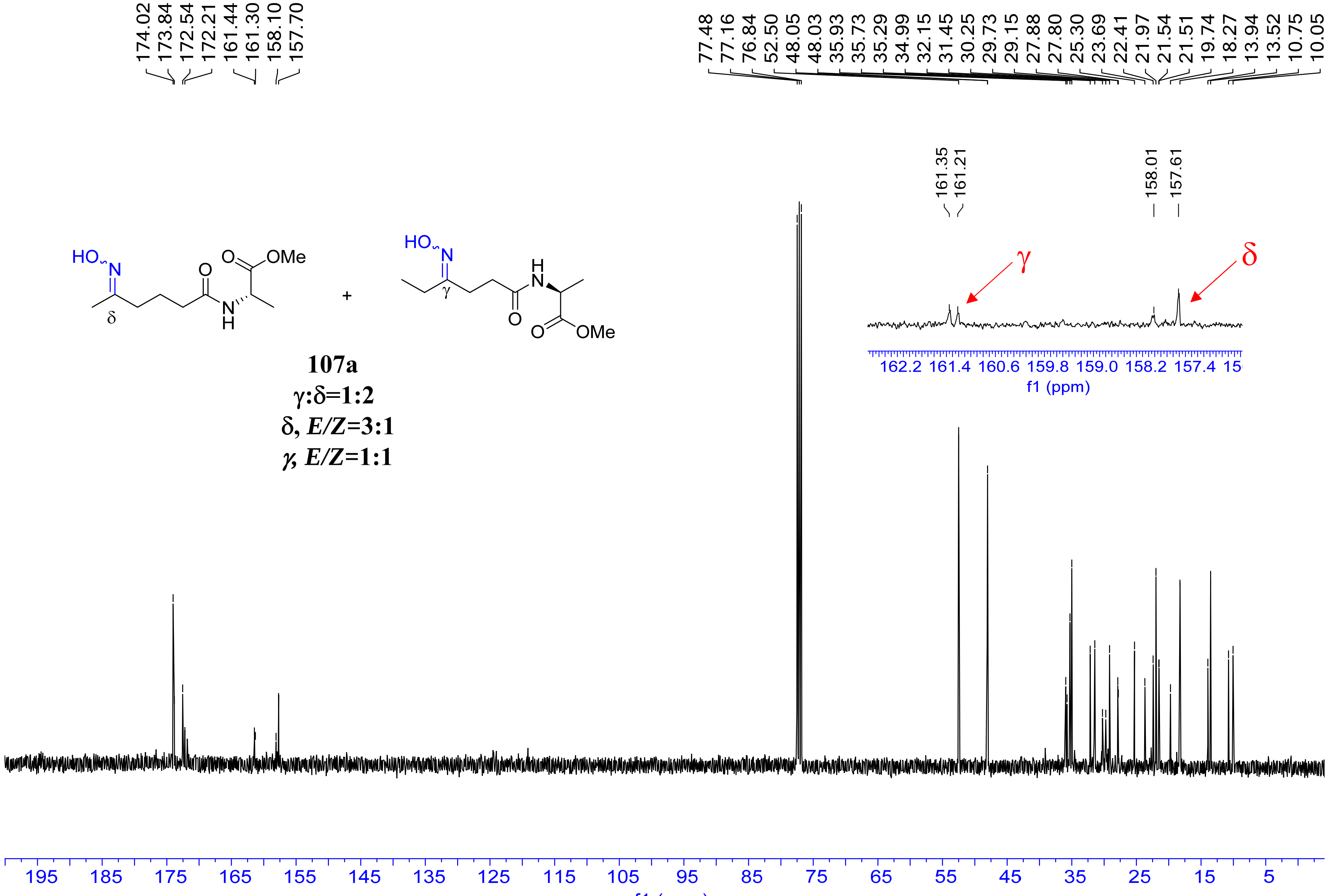

^{1}H NMR, 400 MHz, $CDCl_3$

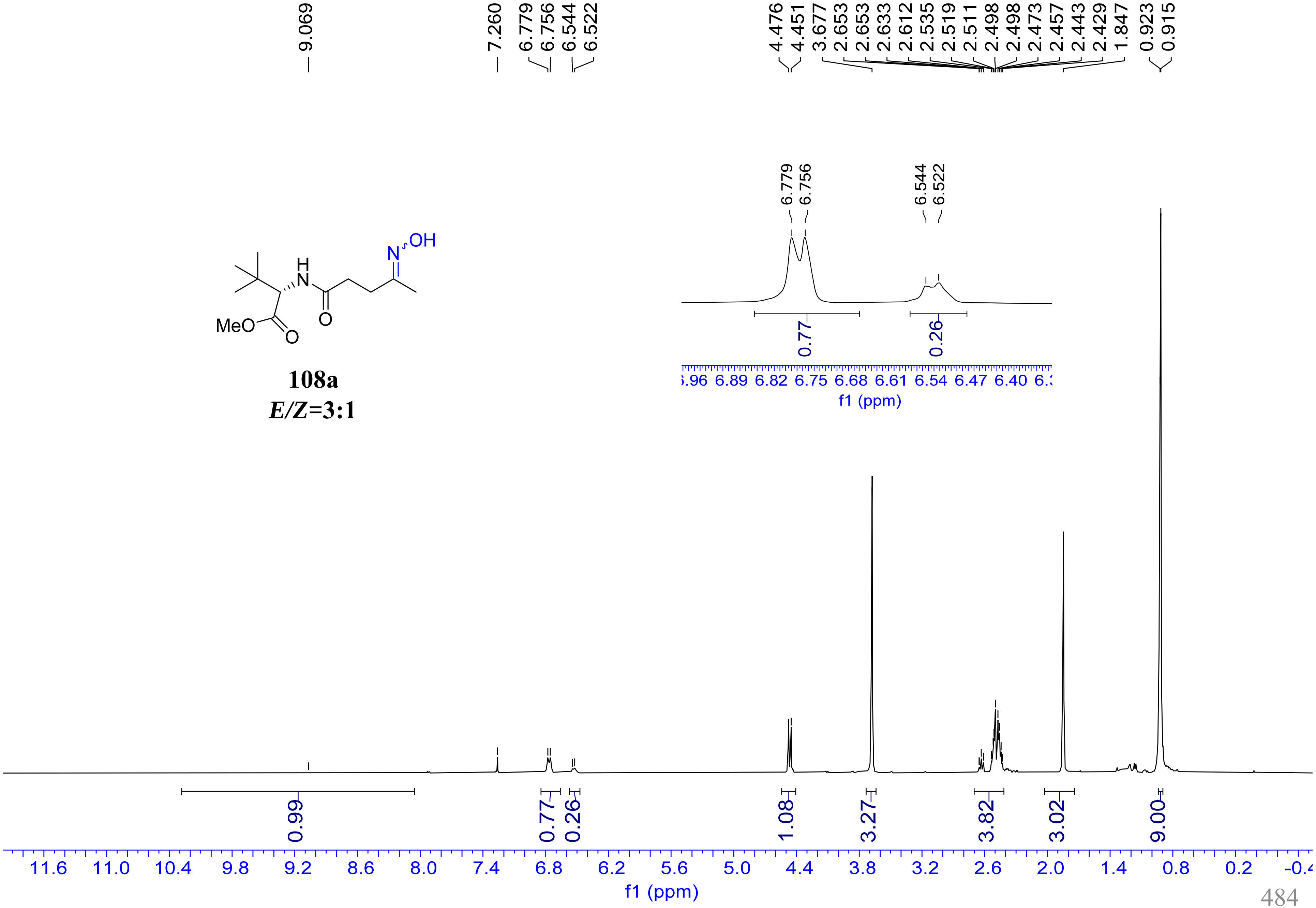

108a

***E/Z*=3:1**

^{13}C NMR 101 MHz, $CDCl_3$

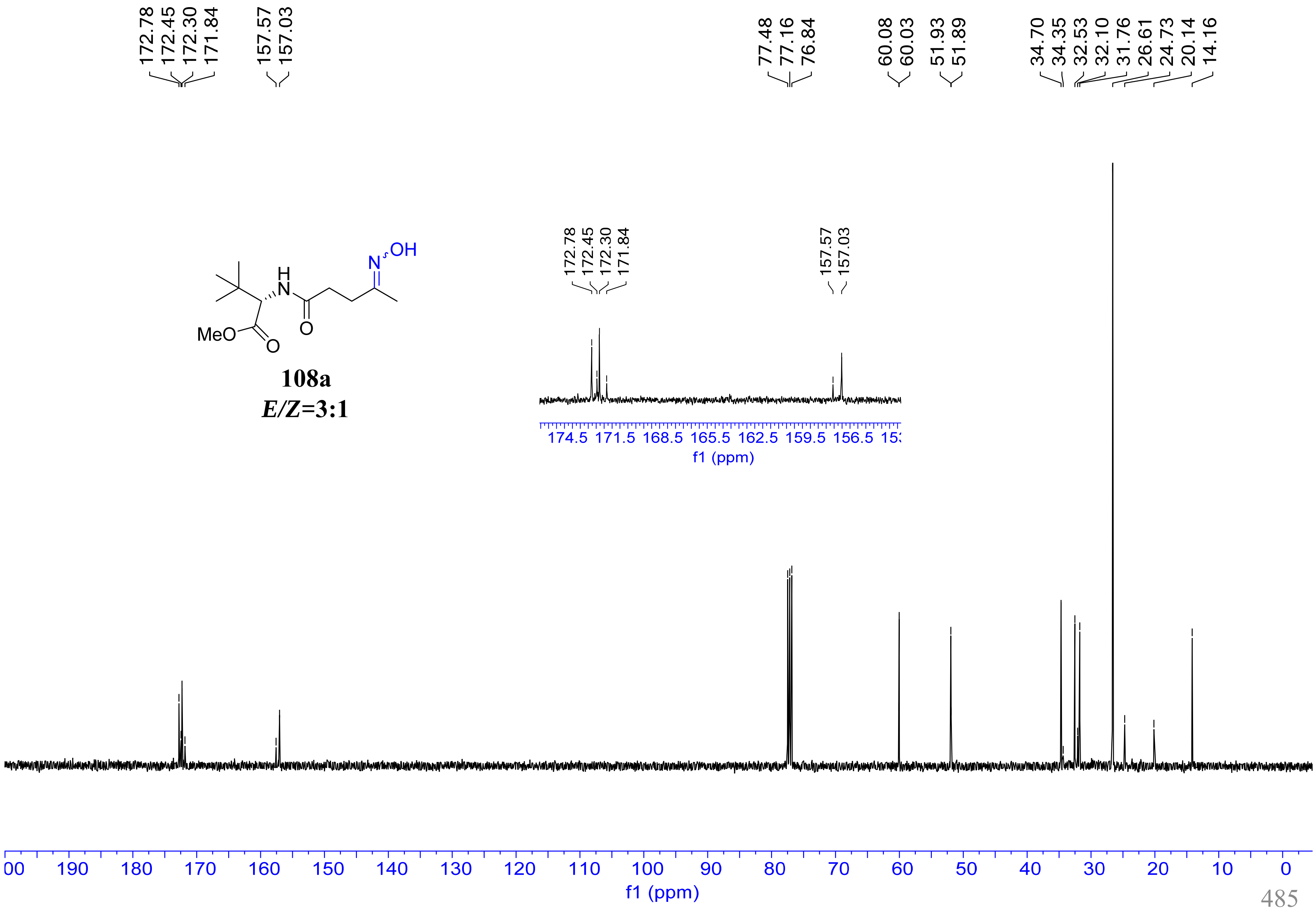

108a

***E/Z*=3:1**

^1H NMR, 400 MHz, $CDCl_3$

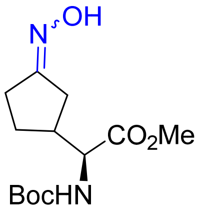

109a

***E/Z*=1.6:1**

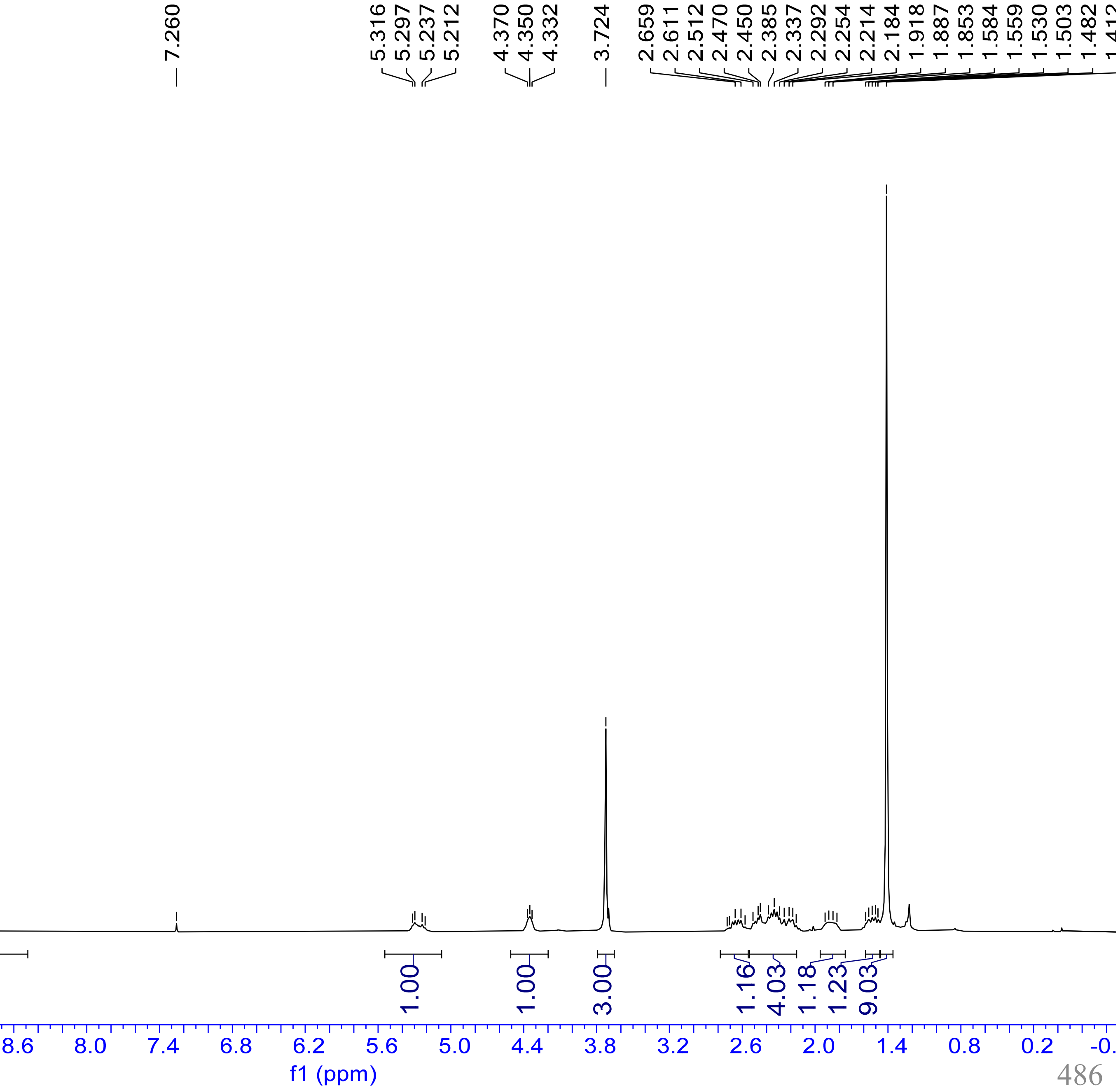

^{13}C NMR 101 MHz, $CDCl_3$

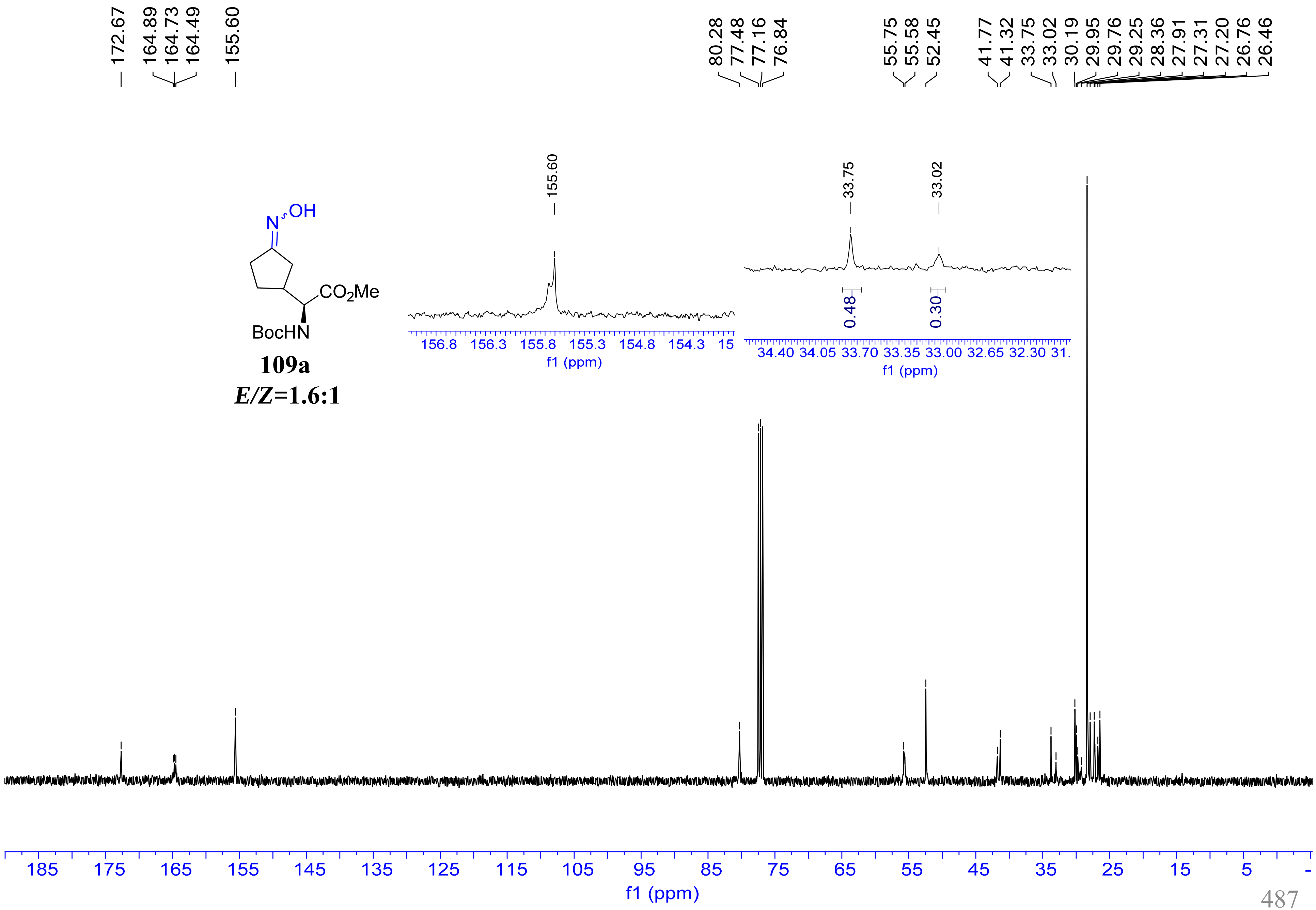

^{1}H NMR, 400 MHz, $CDCl_3$

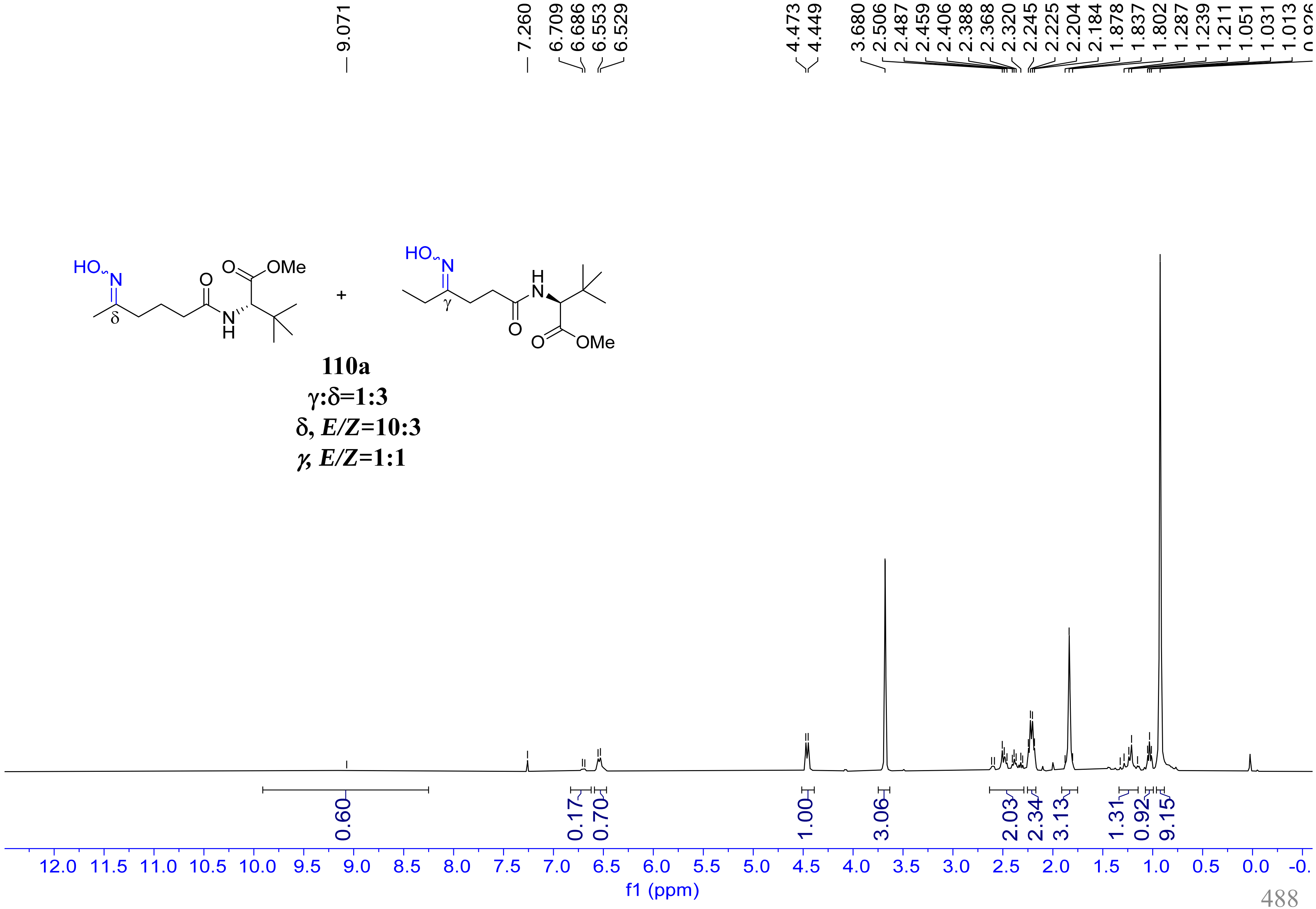

110a

γ:δ=1:3

δ, *E/Z*=10:3

γ, *E/Z*=1:1

^{13}C NMR 101 MHz, $CDCl_3$

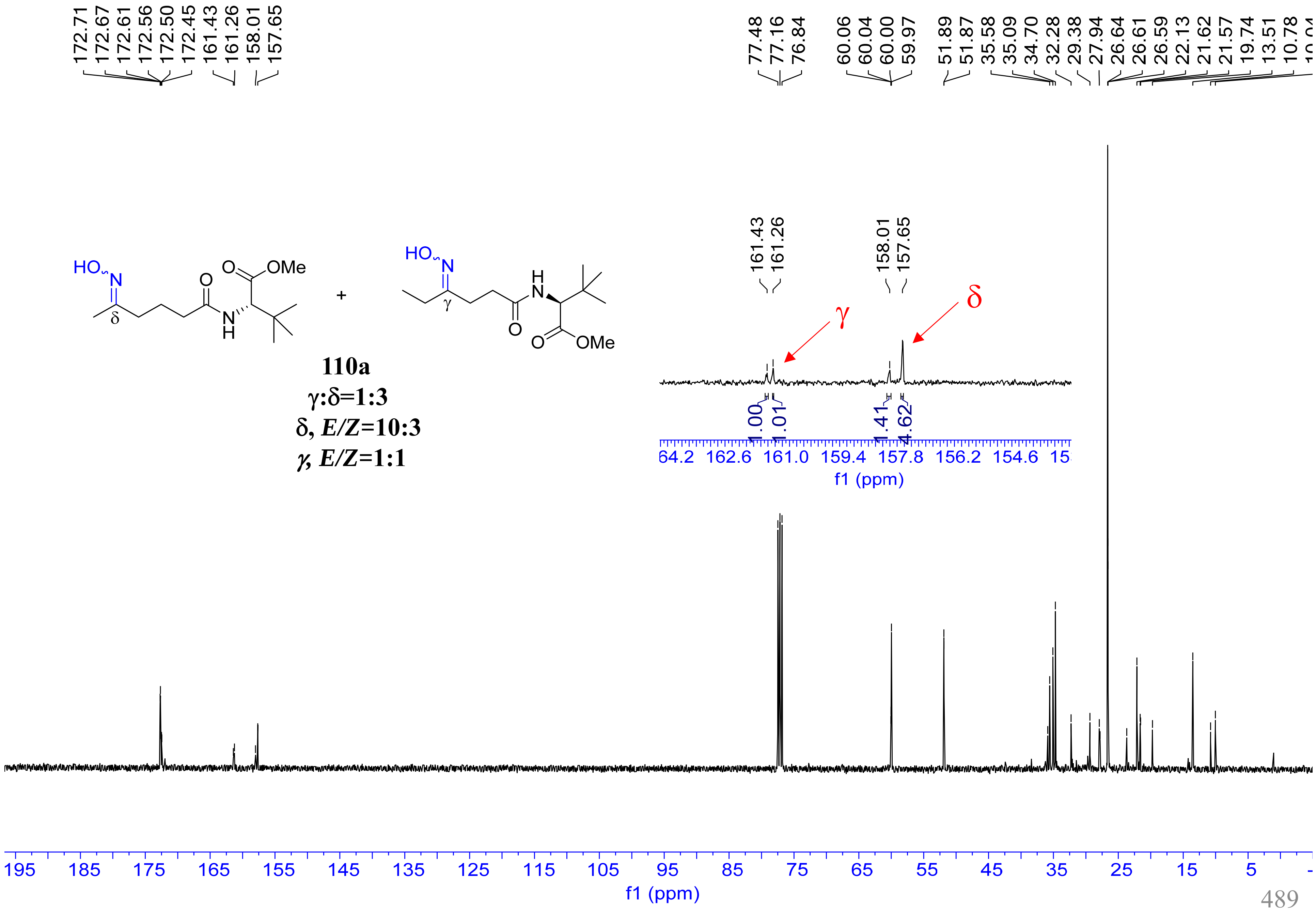

^{1}H NMR, 400 MHz, $CDCl_3$

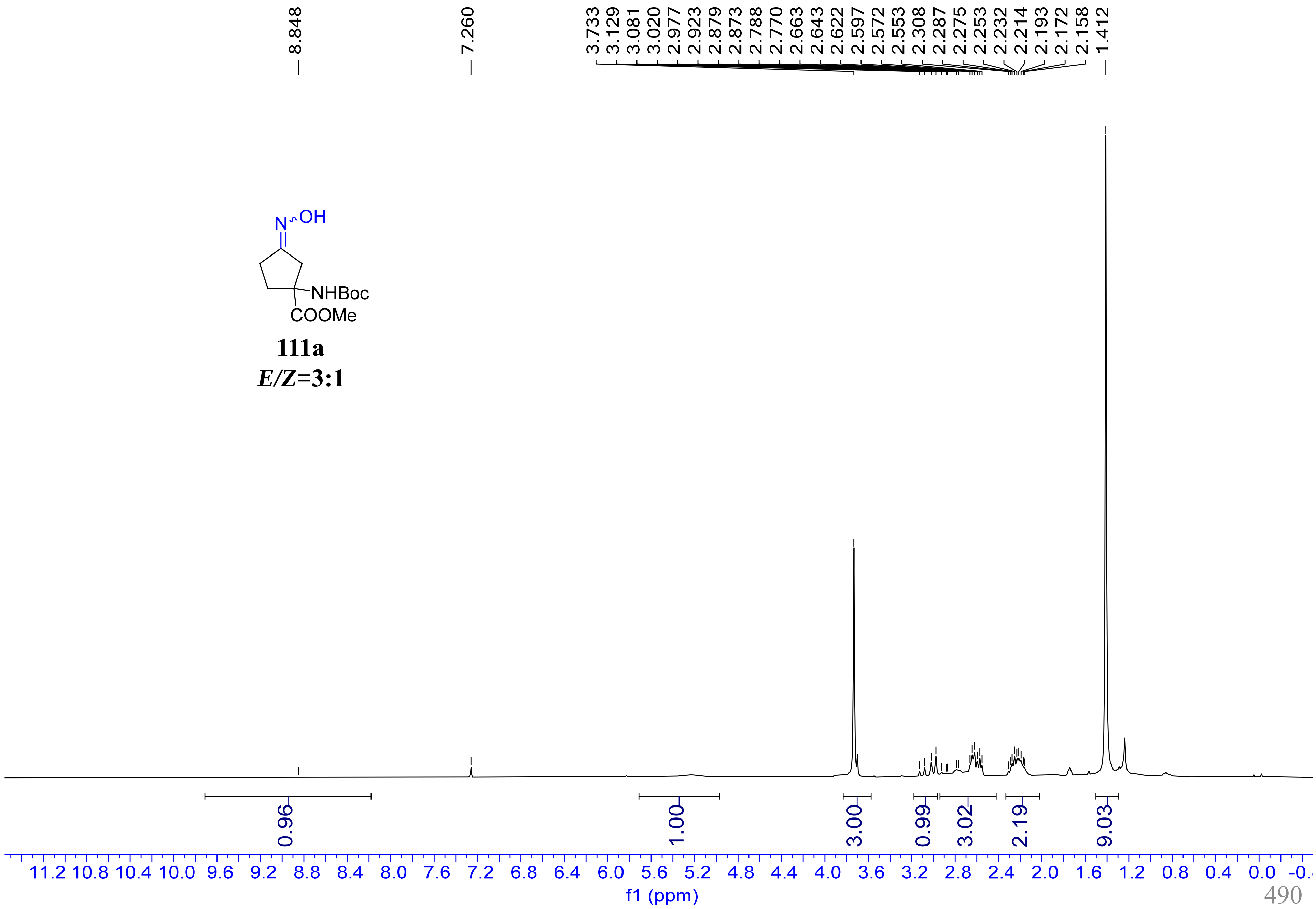

^{13}C NMR 101 MHz, $CDCl_3$

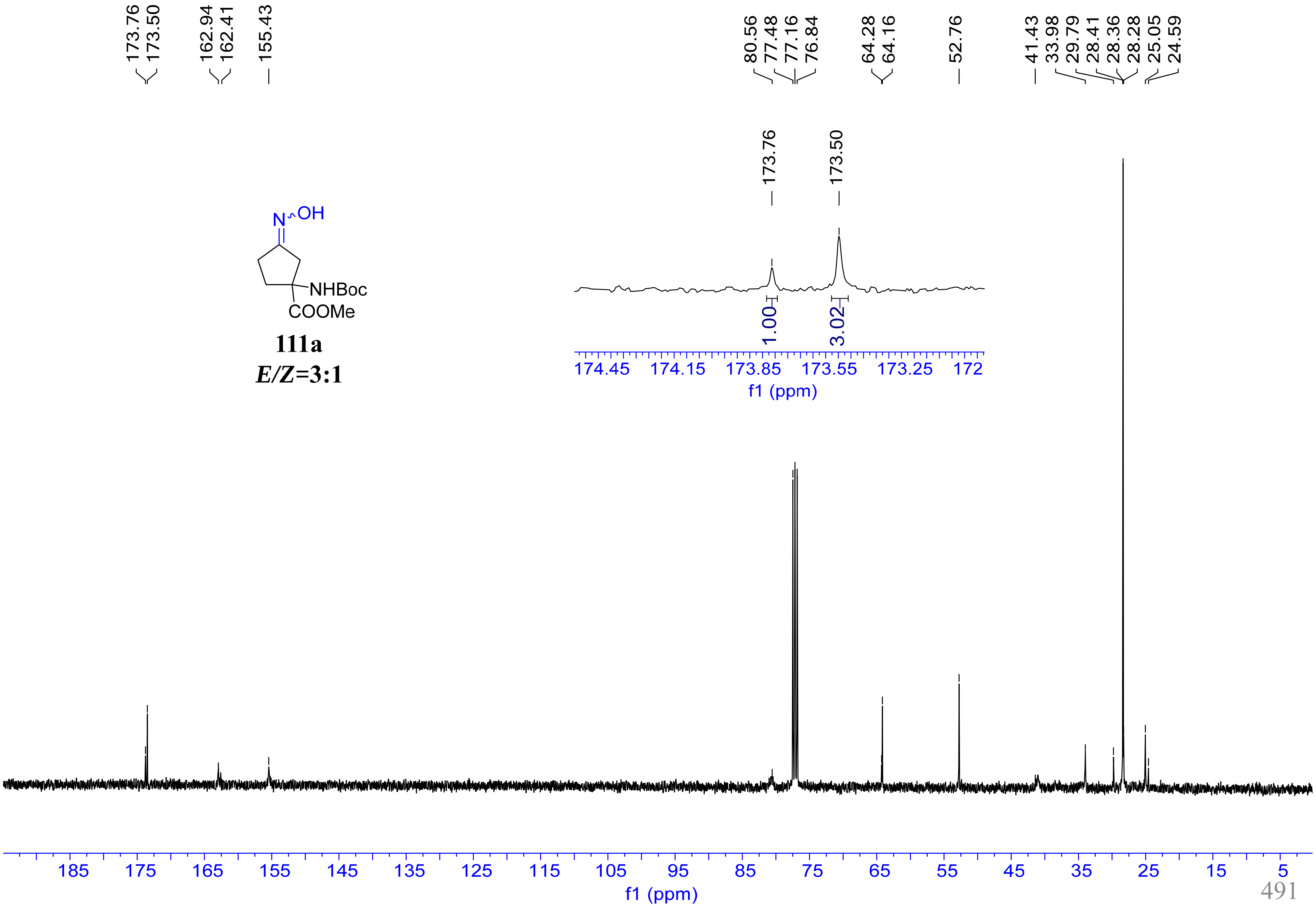

111a

***E/Z*=3:1**

^{1}H NMR, 400 MHz, $CDCl_3$

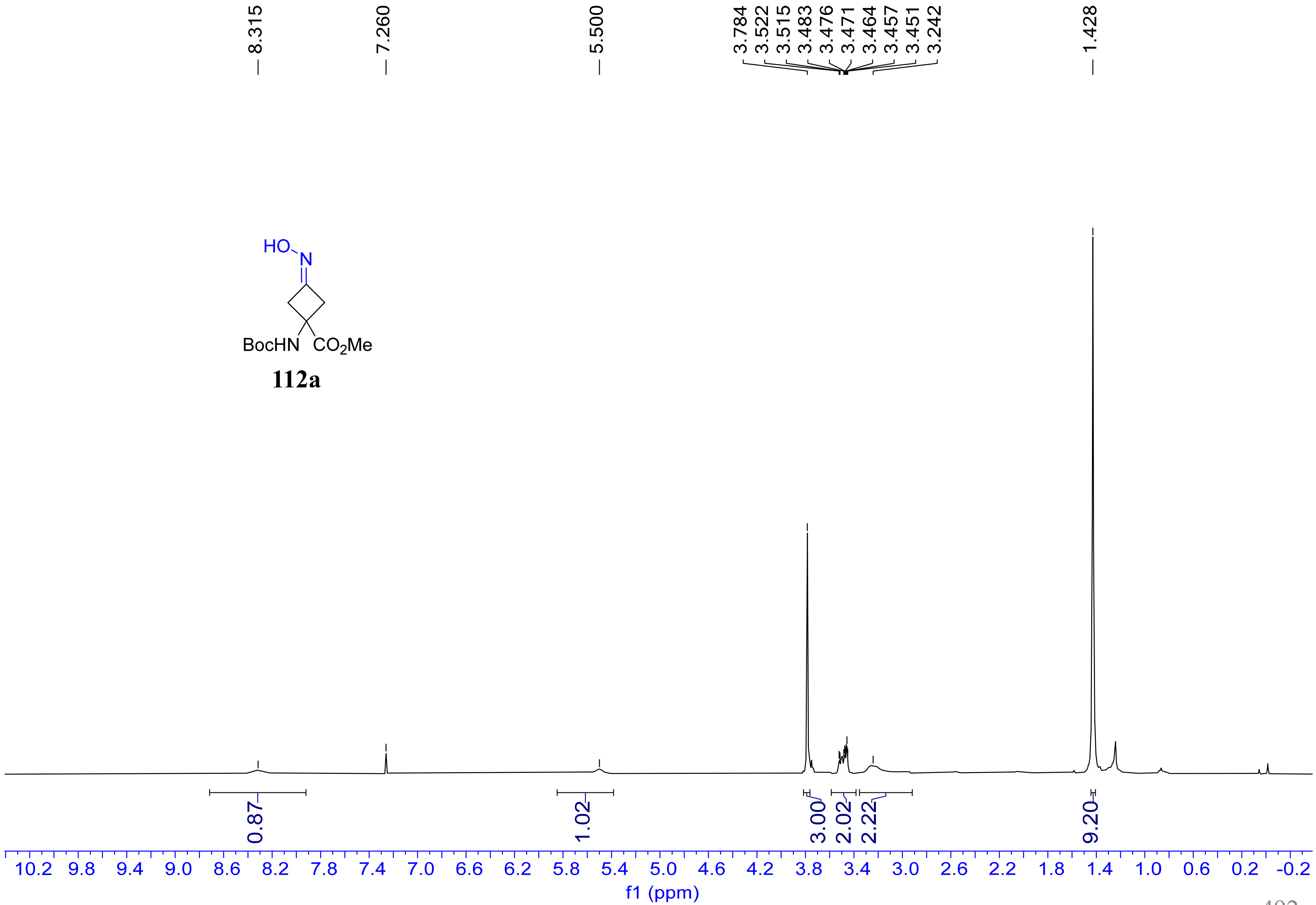

^{13}C NMR 101 MHz, $CDCl_3$

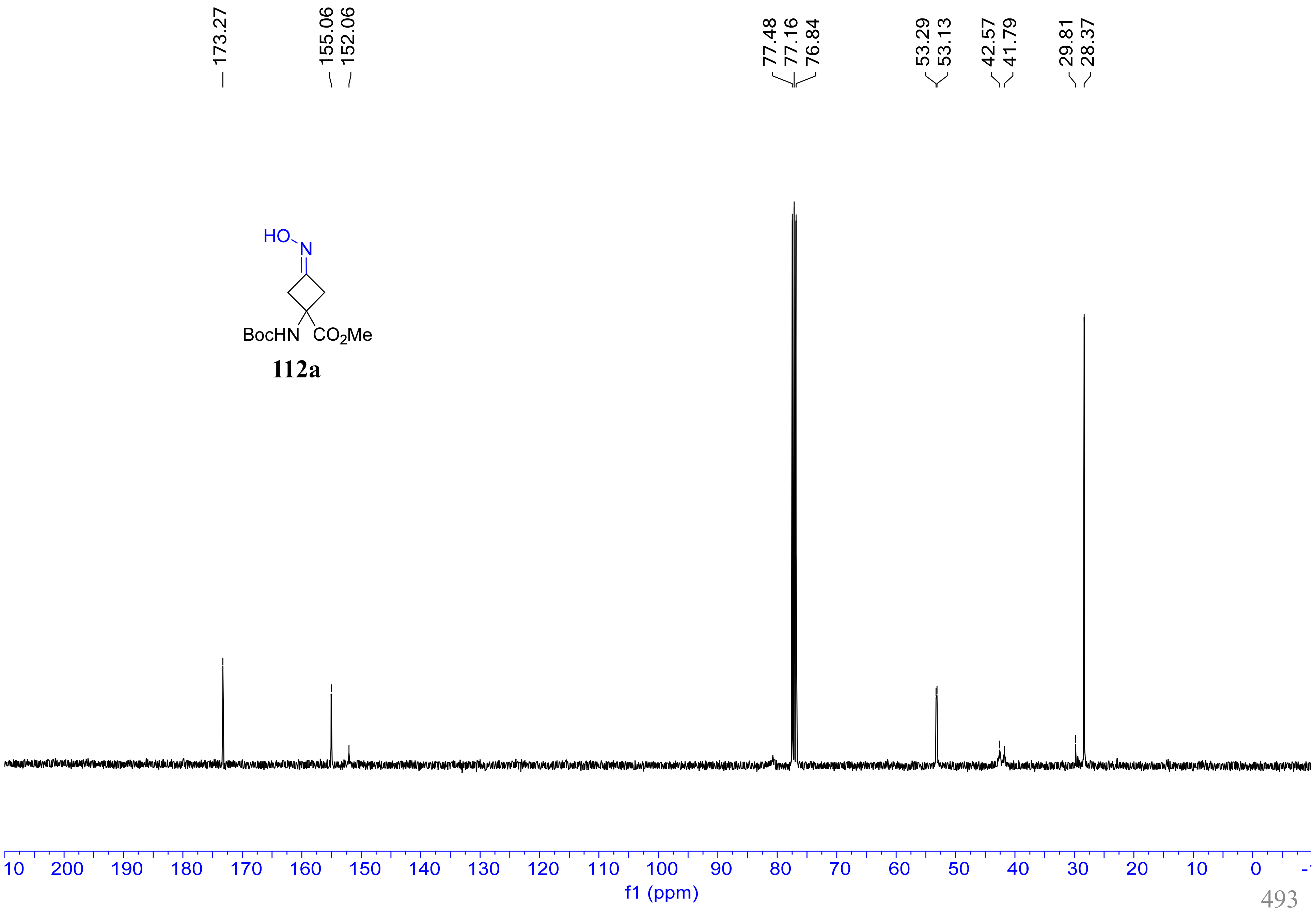

^{1}H NMR, 400 MHz, $CDCl_3$

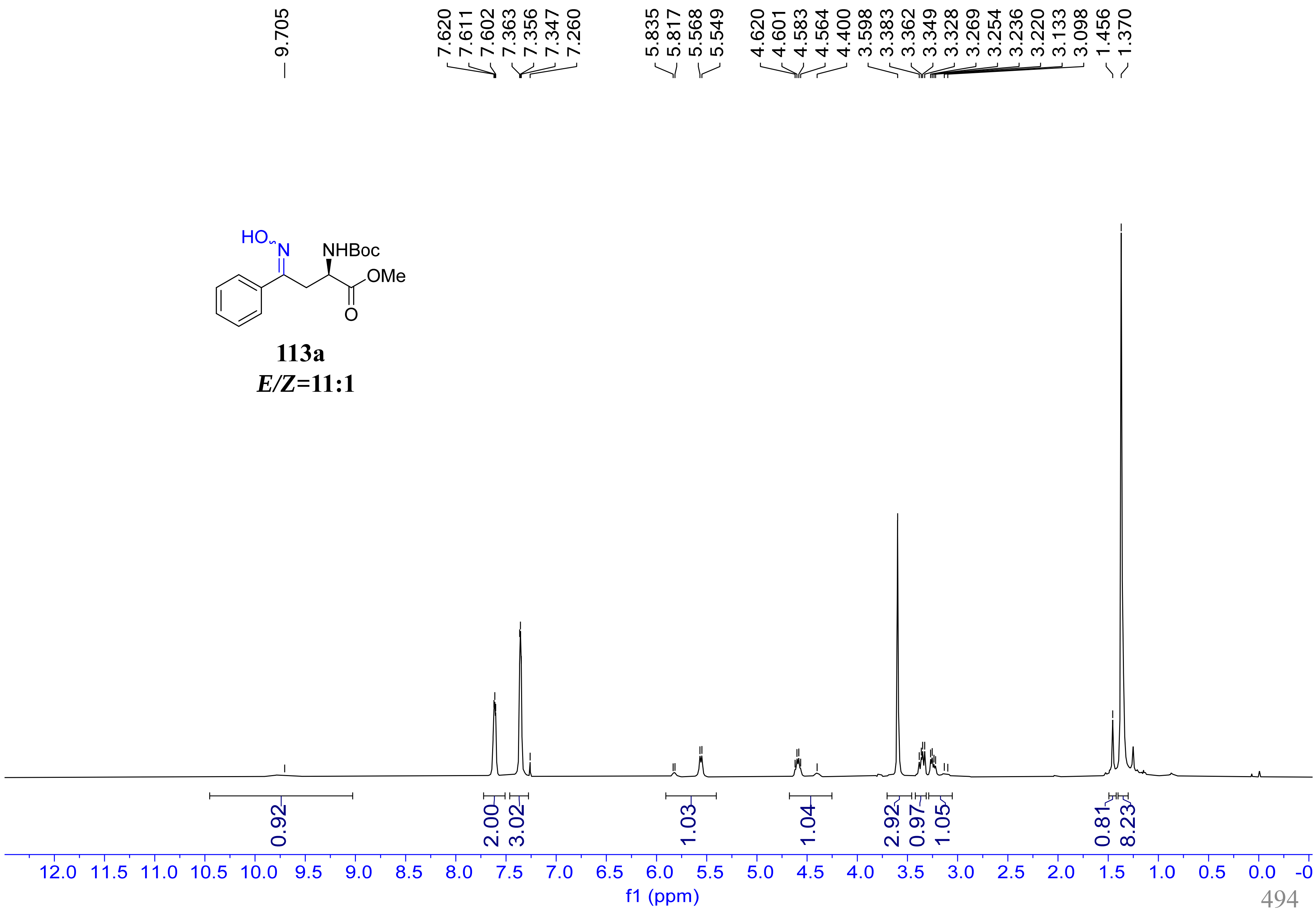

^{13}C NMR 101 MHz, $CDCl_3$

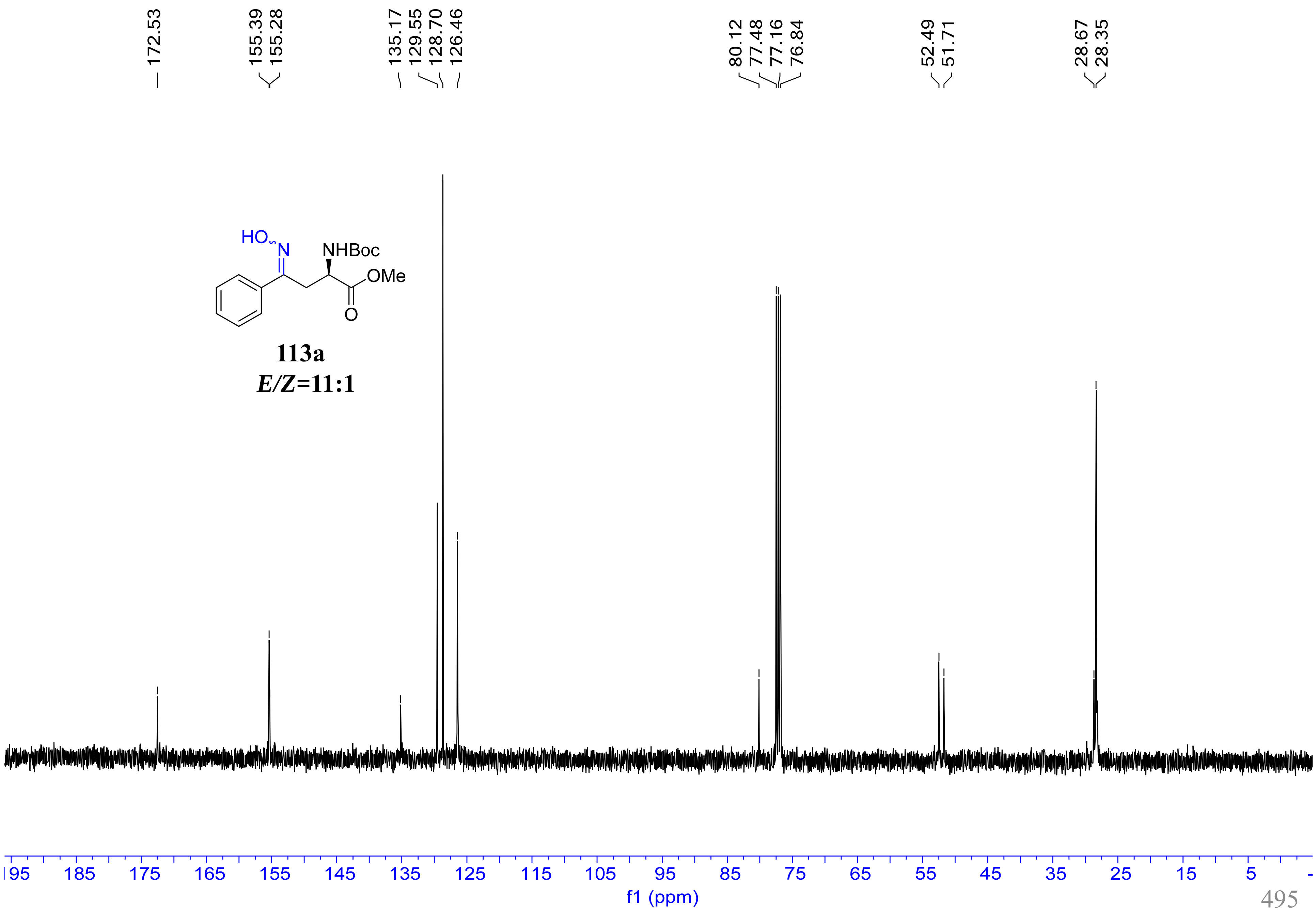

113a

***E/Z*=11:1**

^{1}H NMR, 400 MHz, CDCl$_3$

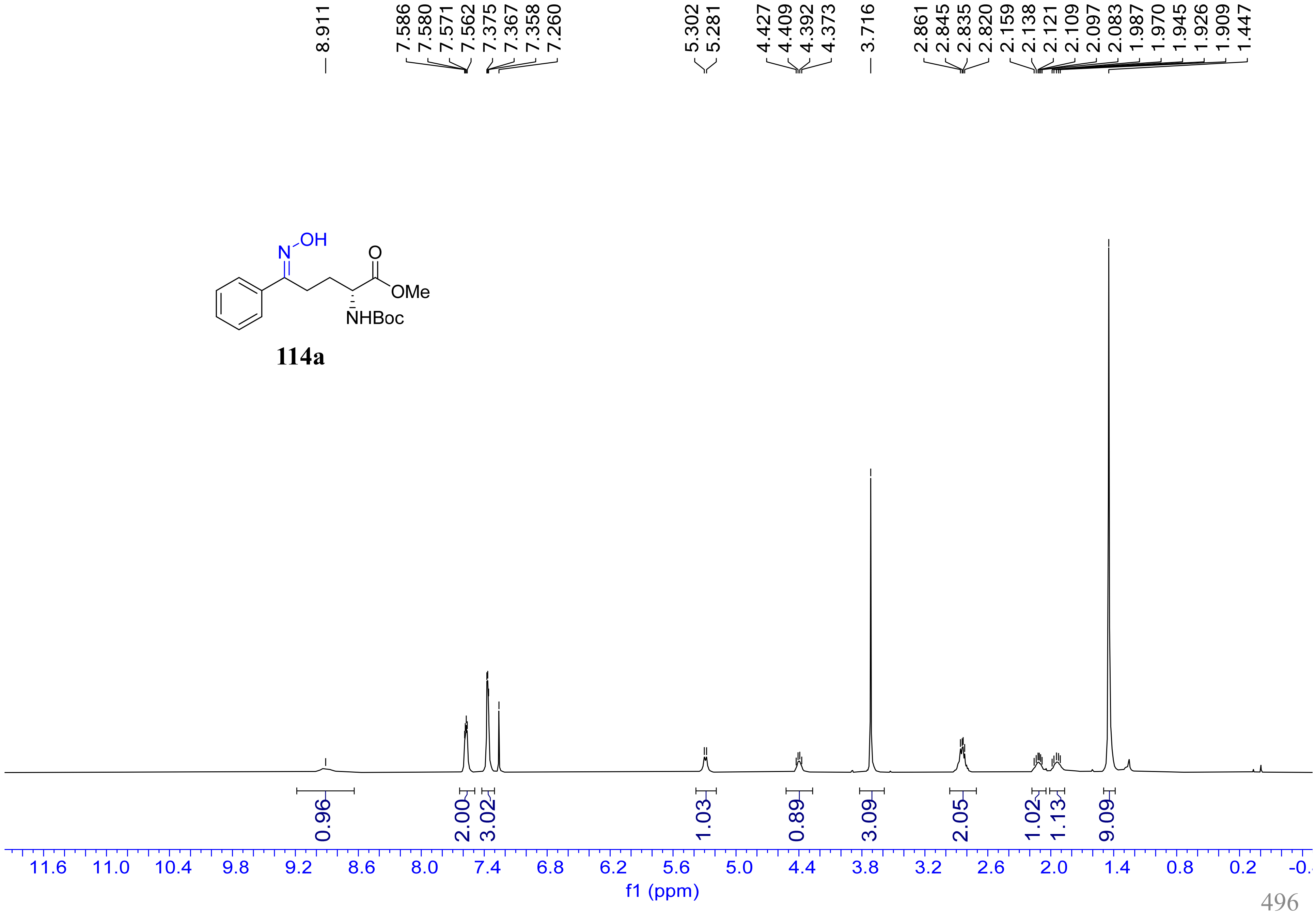

114a

^{13}C NMR 101 MHz, $CDCl_3$

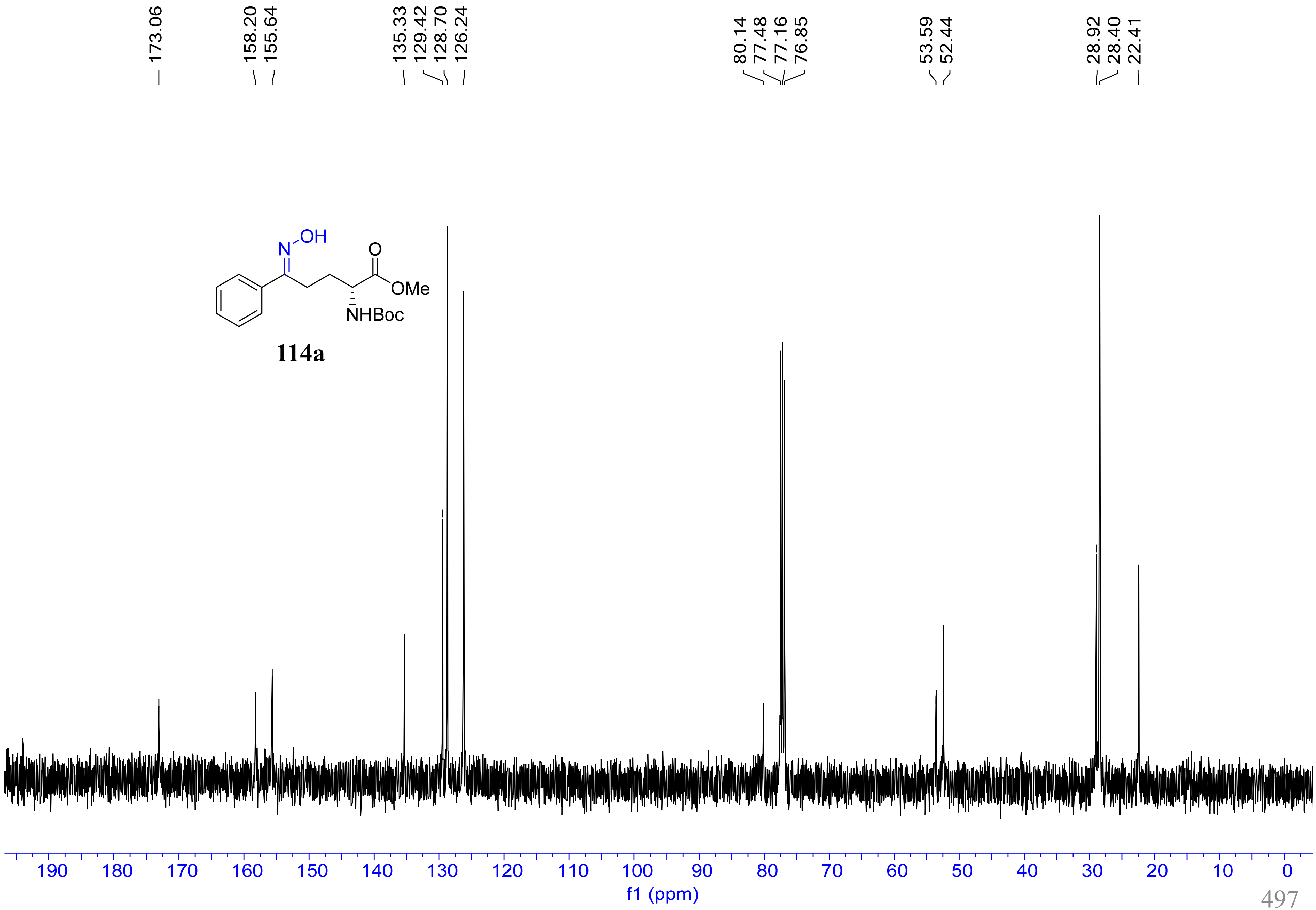

114a

^{1}H NMR, 400 MHz, $CDCl_3$

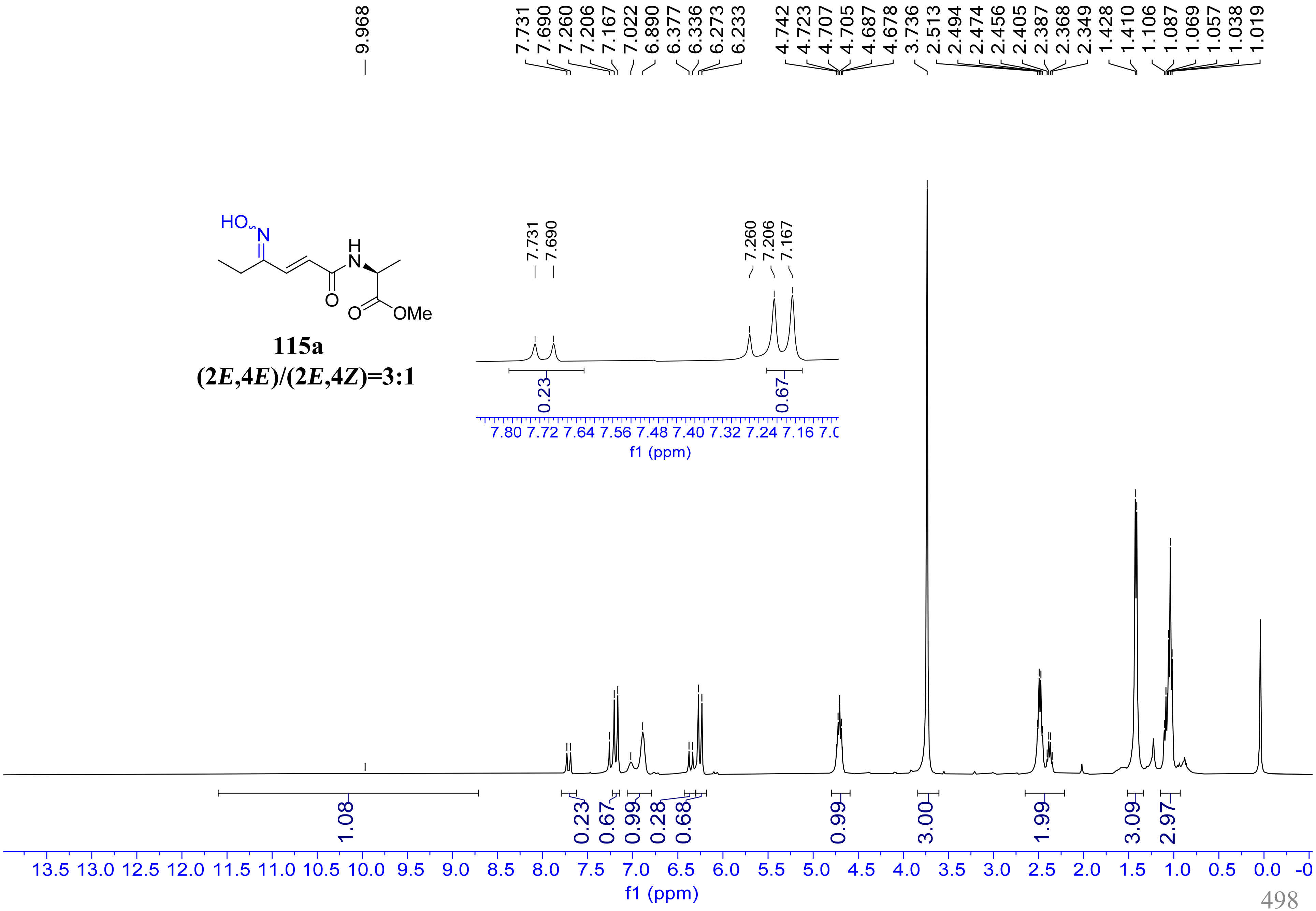

^{13}C NMR 101 MHz, $CDCl_3$

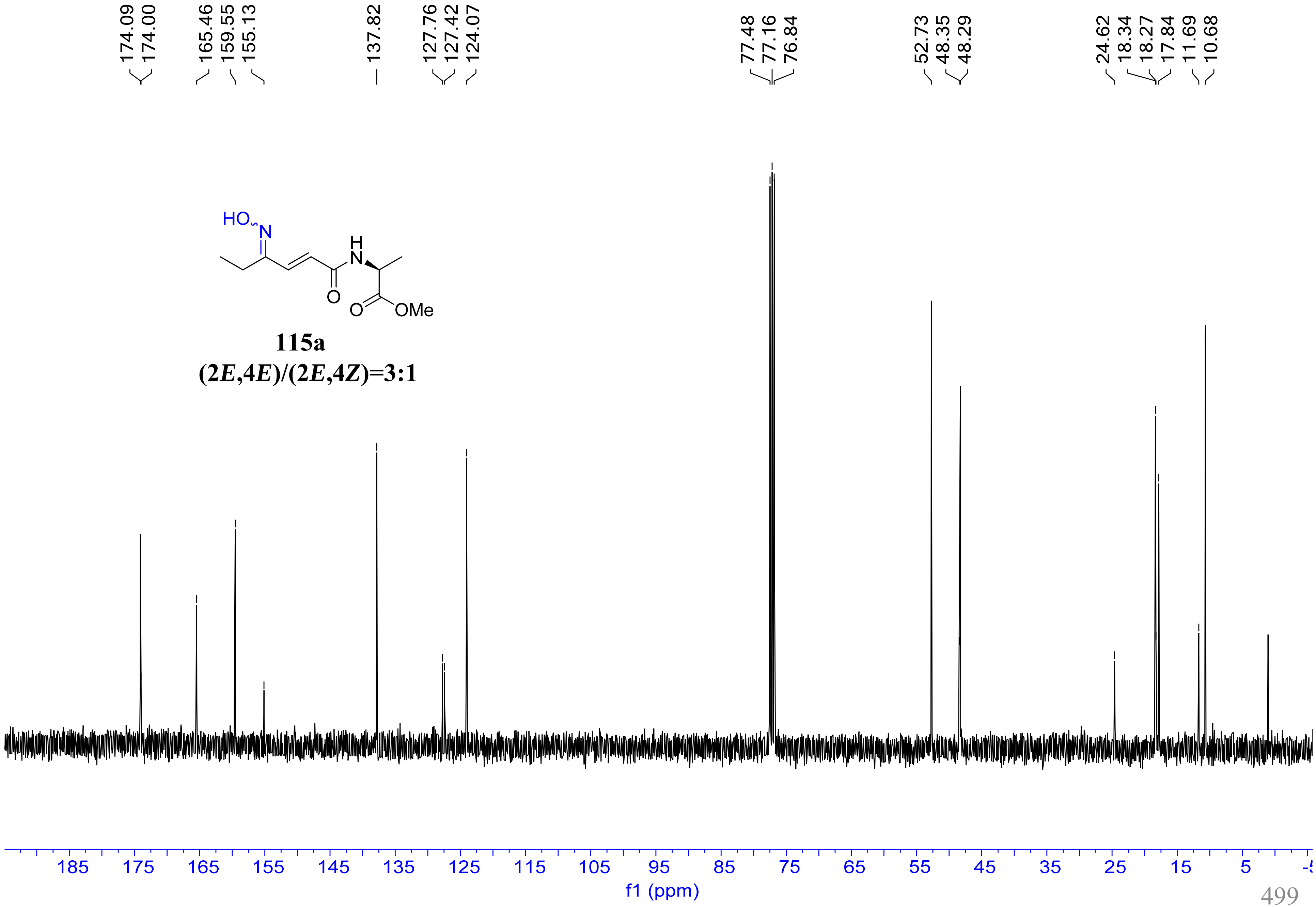

115a

(2*E*,4*E*)/(2*E*,4*Z*)=3:1

^{1}H NMR, 400 MHz, $CDCl_3$

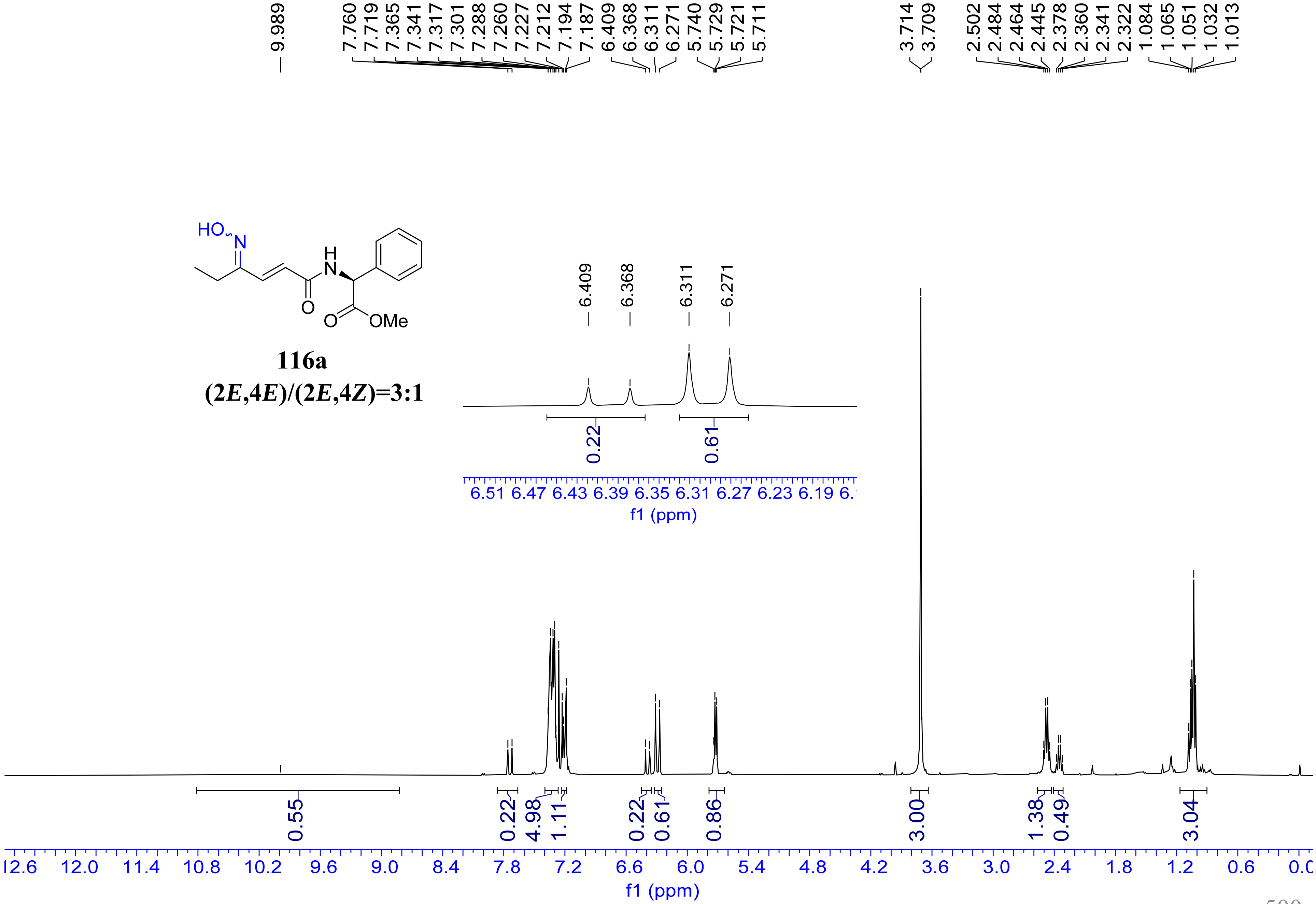

116a

(2*E*,4*E*)/(2*E*,4*Z*)=3:1

^{13}C NMR 101 MHz, $CDCl_3$

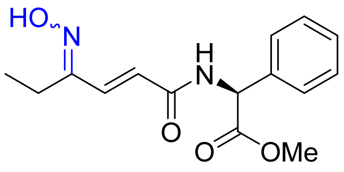

116a

(2*E*,4*E*)/(2*E*,4*Z*)=3:1

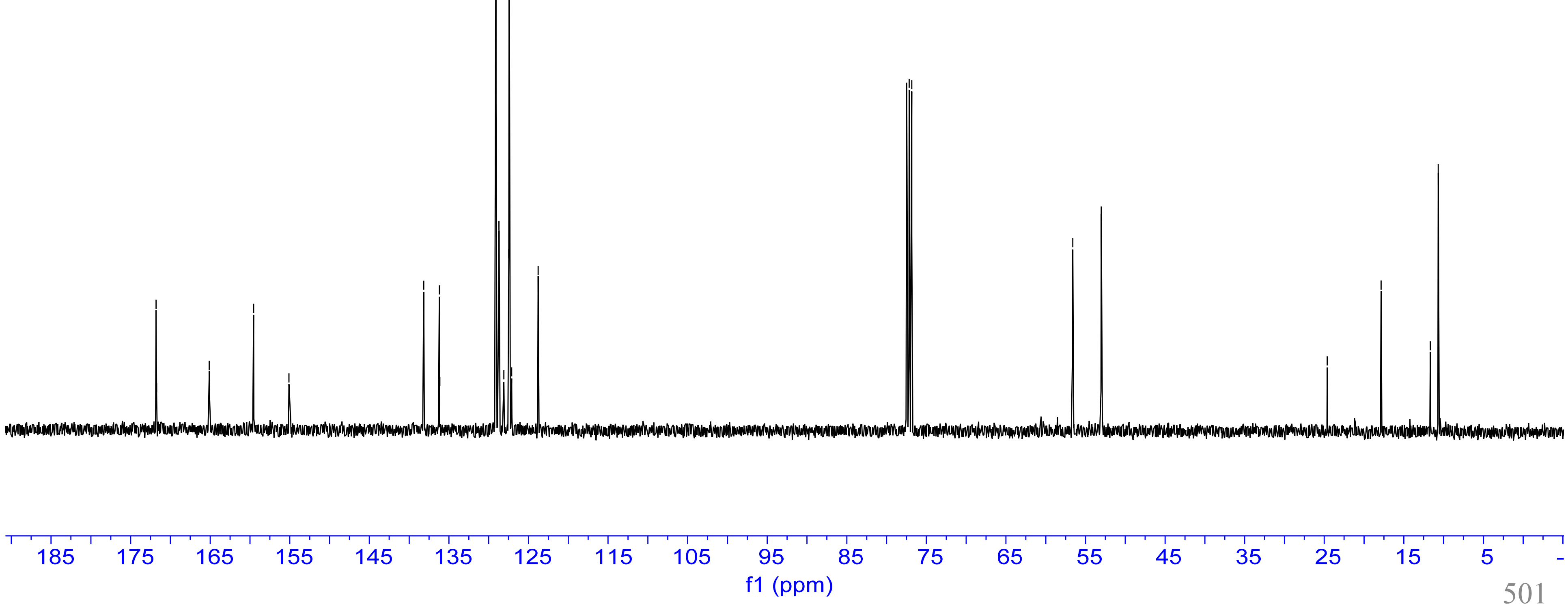

^{1}H NMR, 400 MHz, $CDCl_3$

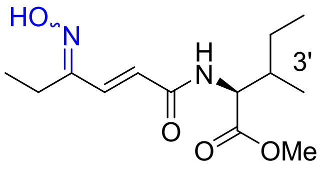

117a

(2*E*,4*E*)/(2*E*,4*Z*)=2.4:1

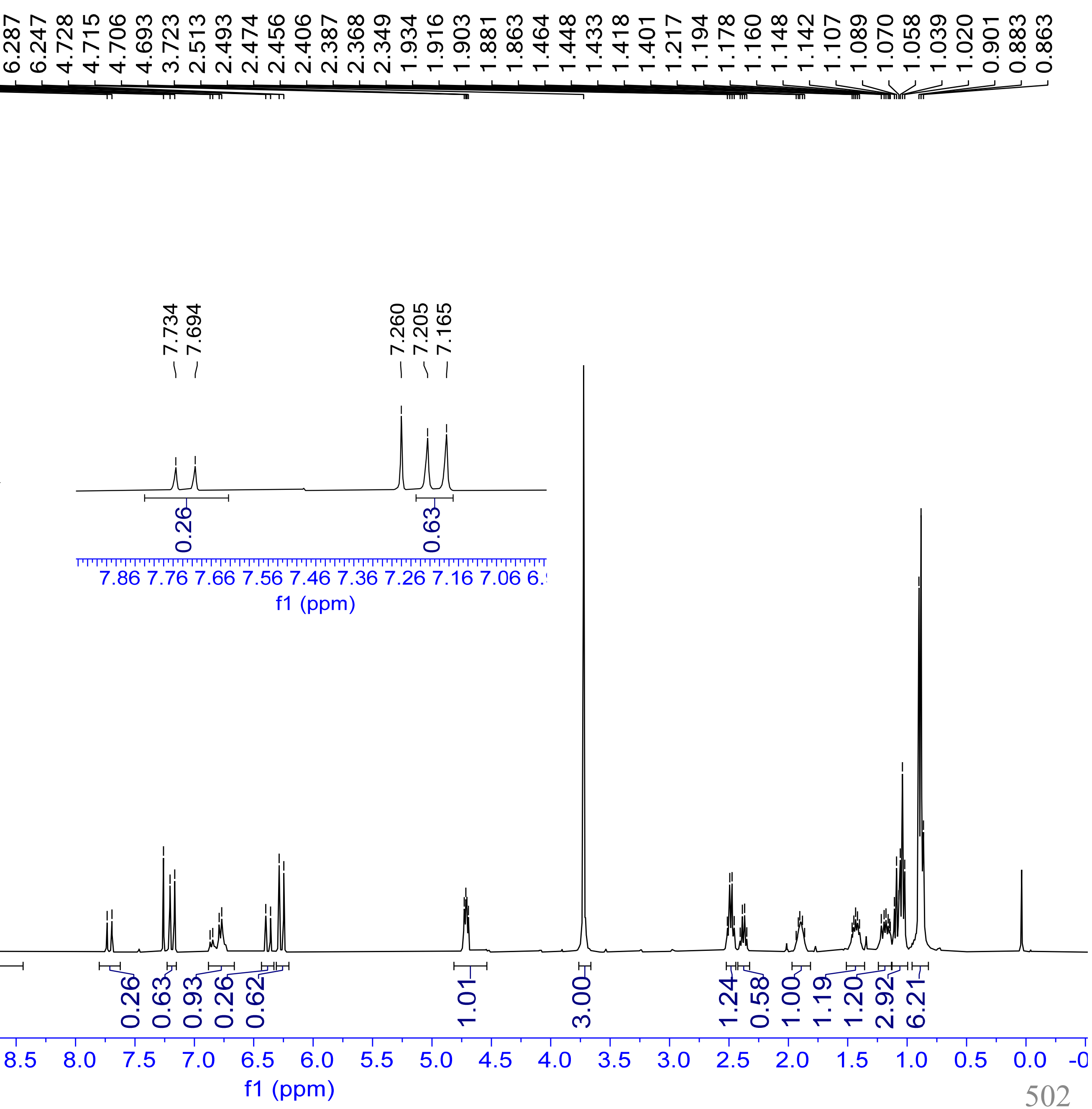

^{13}C NMR 101 MHz, $CDCl_3$

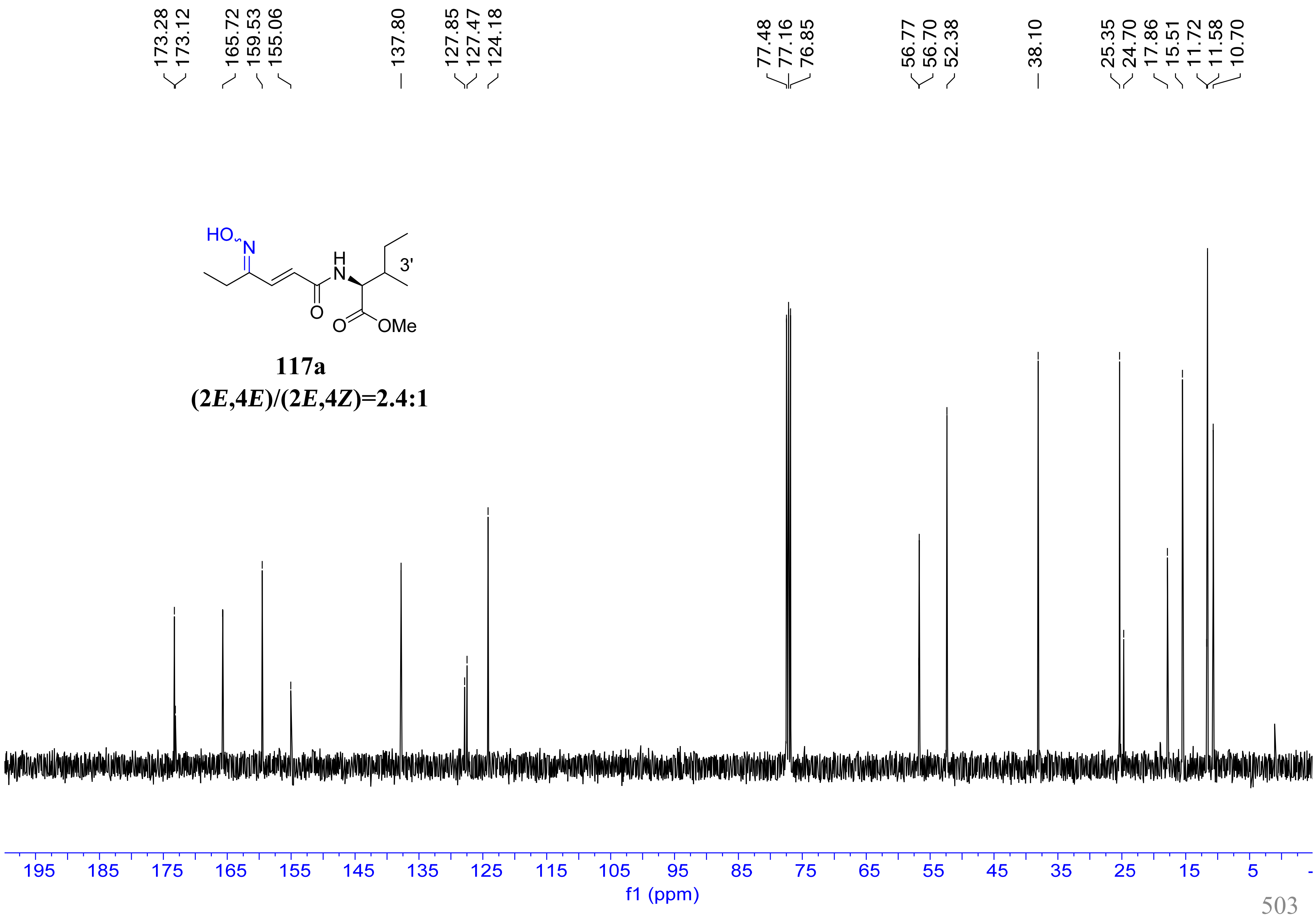

^{1}H NMR, 400 MHz, $CDCl_3$

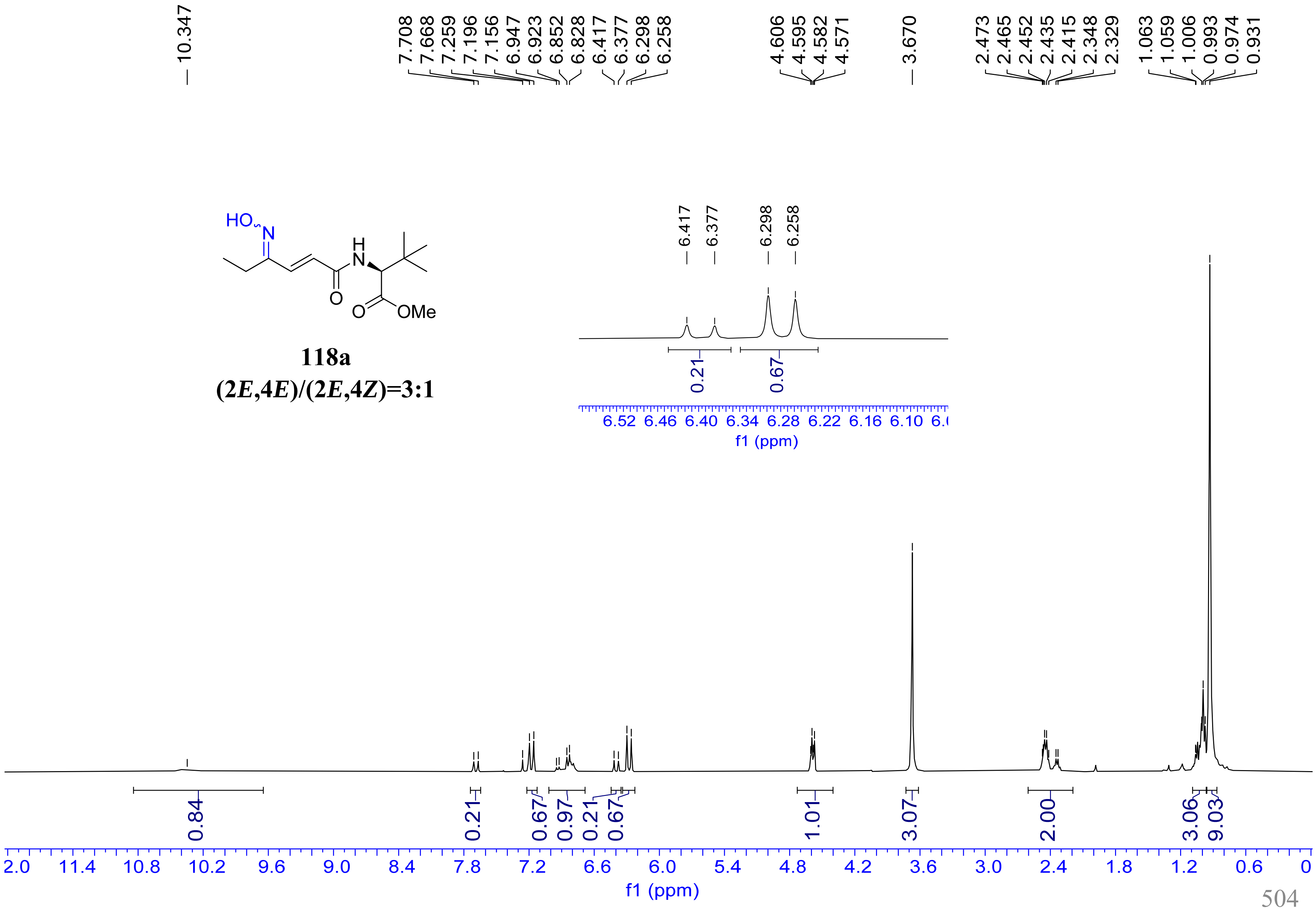

^{13}C NMR 101 MHz, $CDCl_3$

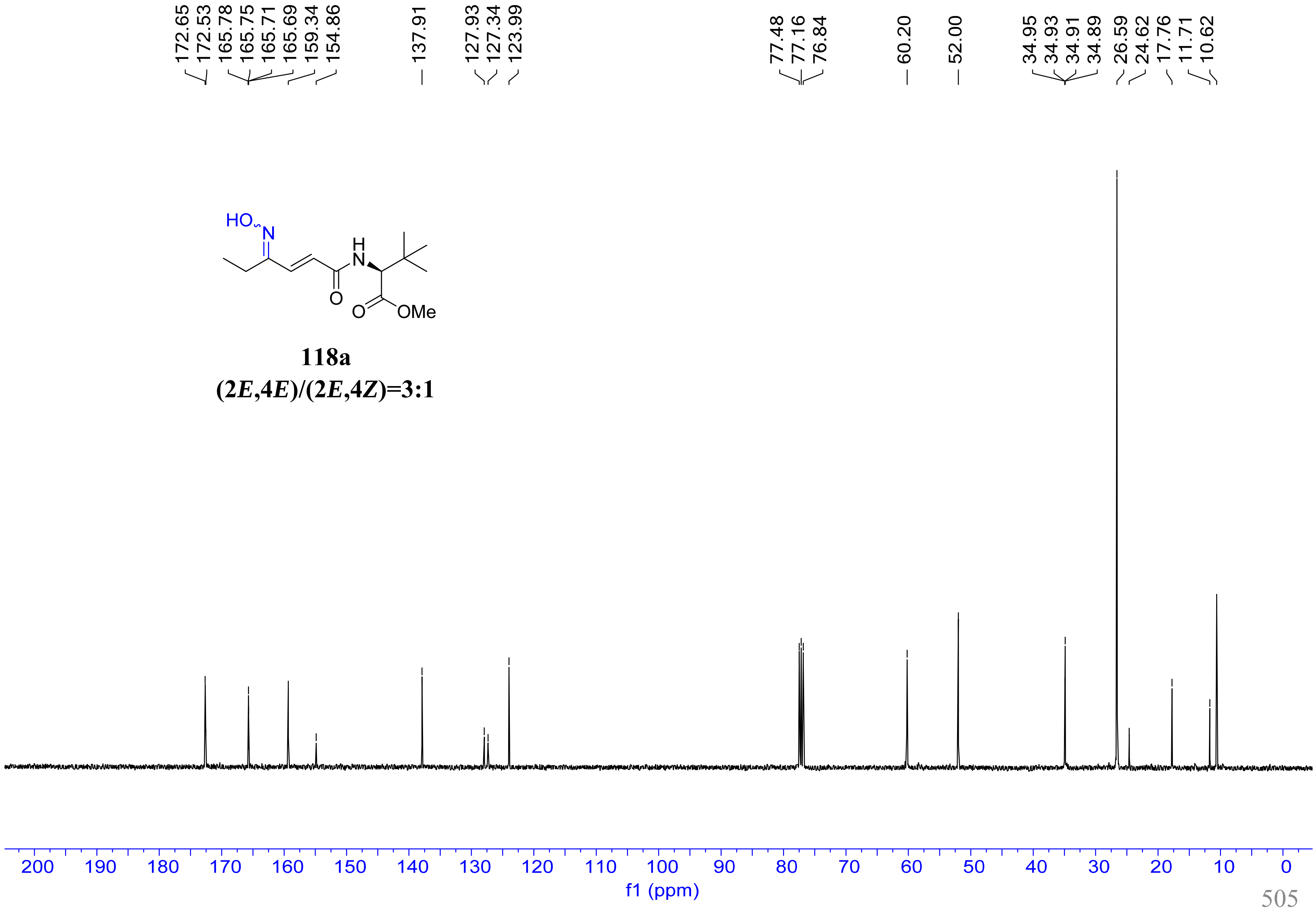

118a

(2*E*,4*E*)/(2*E*,4*Z*)=3:1

^{1}H NMR, 400 MHz, $CDCl_3$

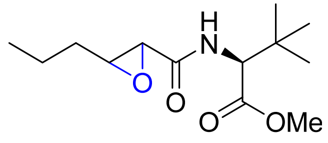

118b
dr = 4:1

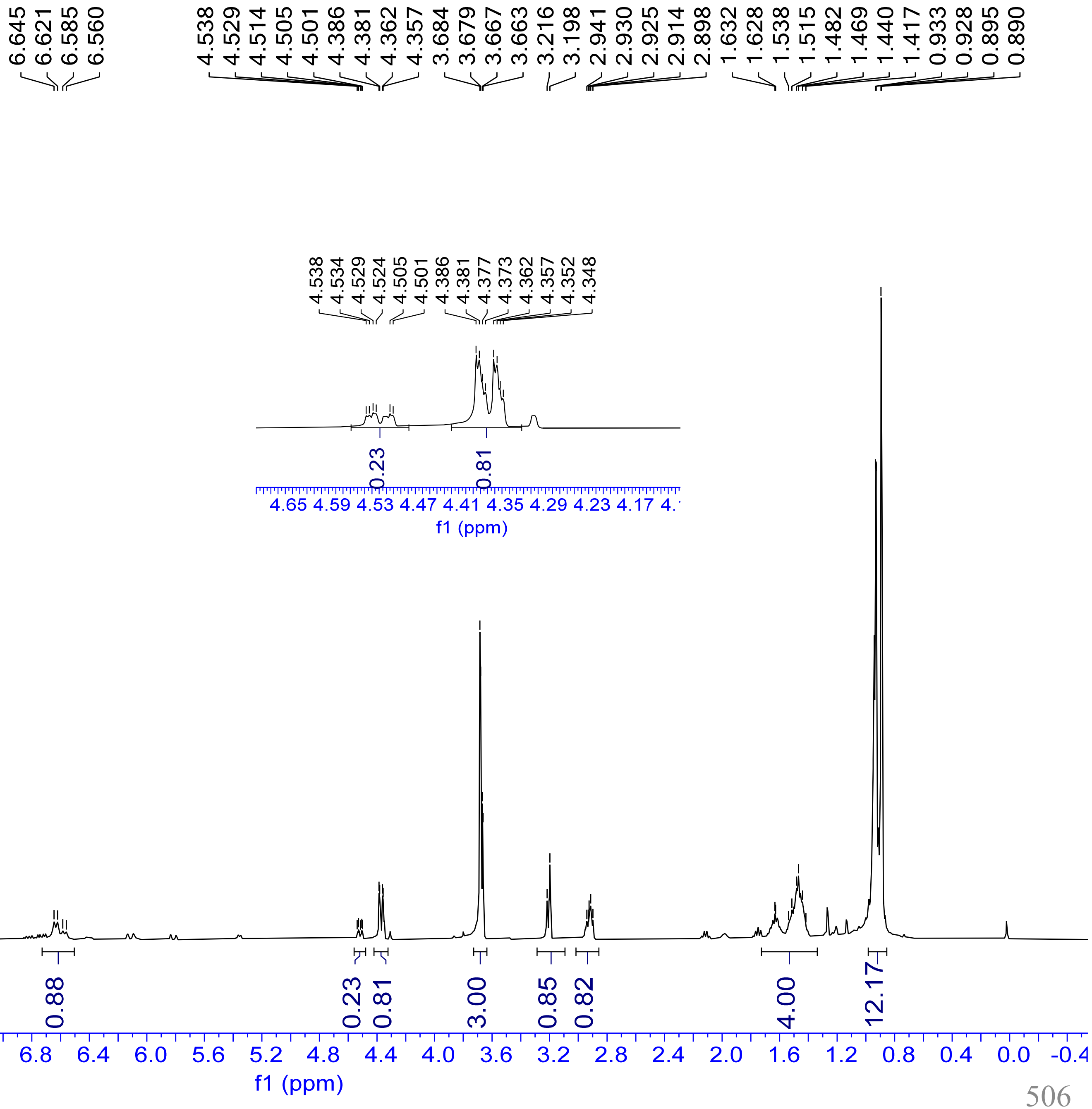

^{13}C NMR 101 MHz, $CDCl_3$

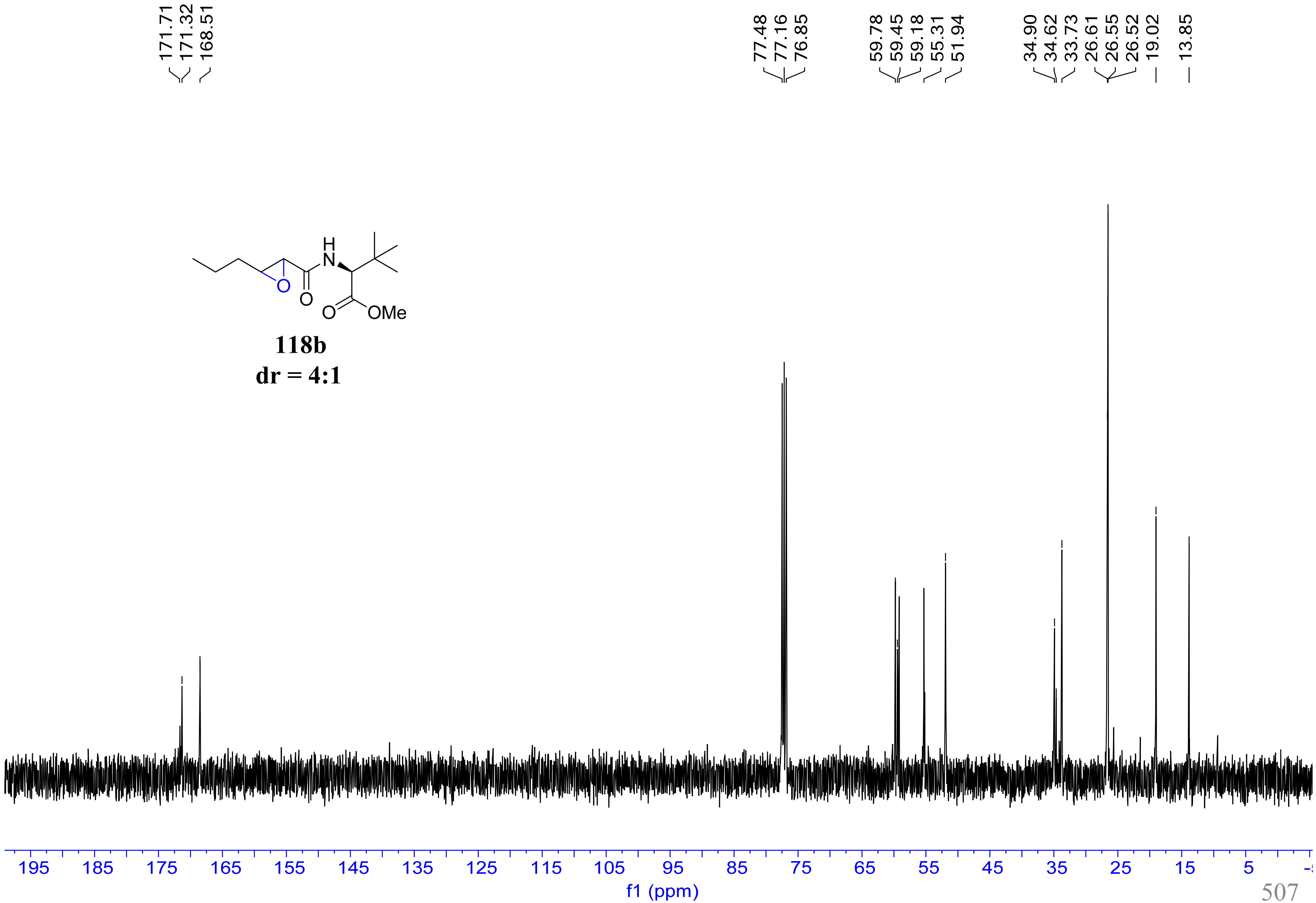

118b

dr = 4:1

^1H NMR, 400 MHz, $CDCl_3$

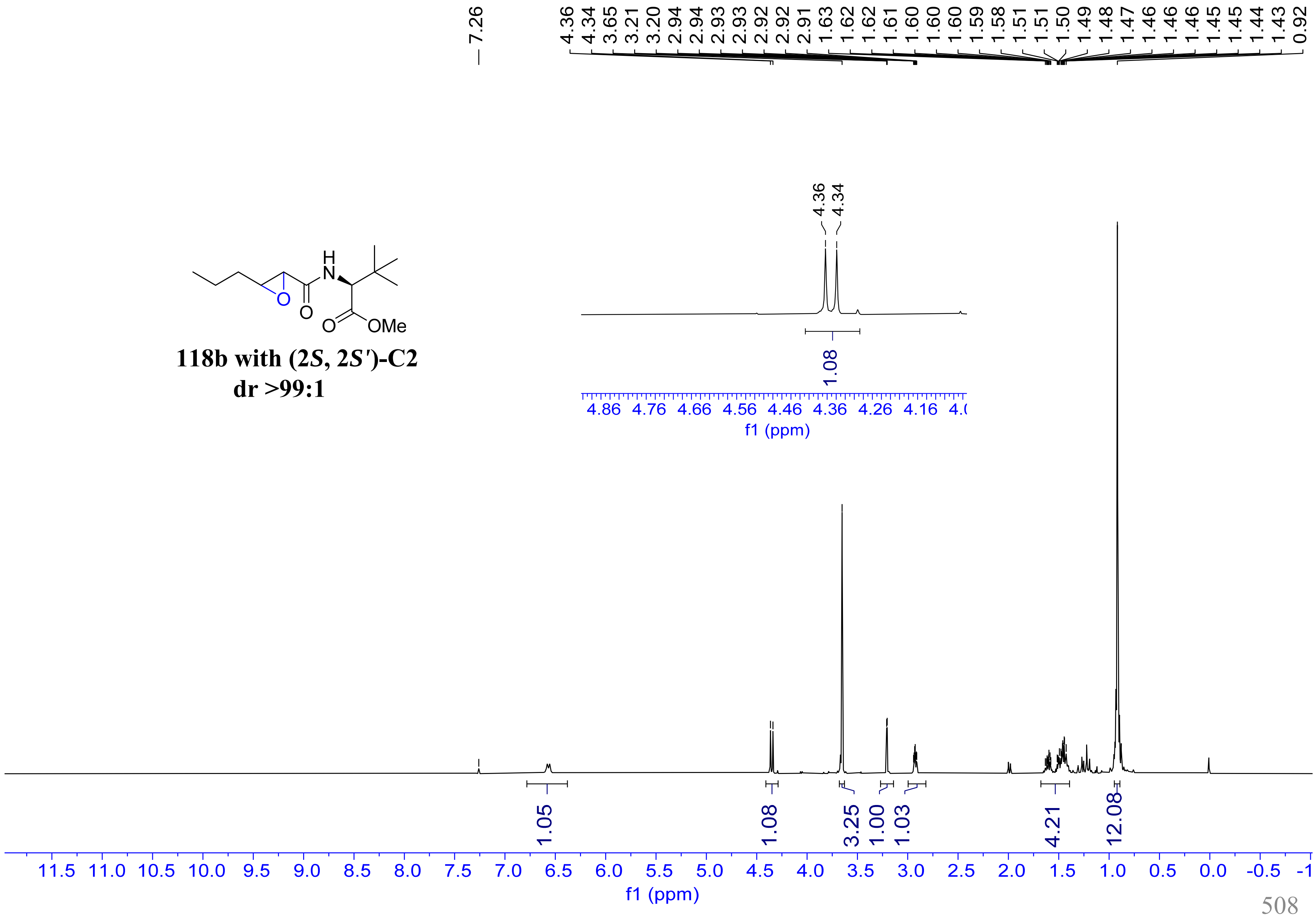

118b with (2*S*, 2*S′*)-C2
dr >99:1

^{13}C NMR 101 MHz, $CDCl_3$

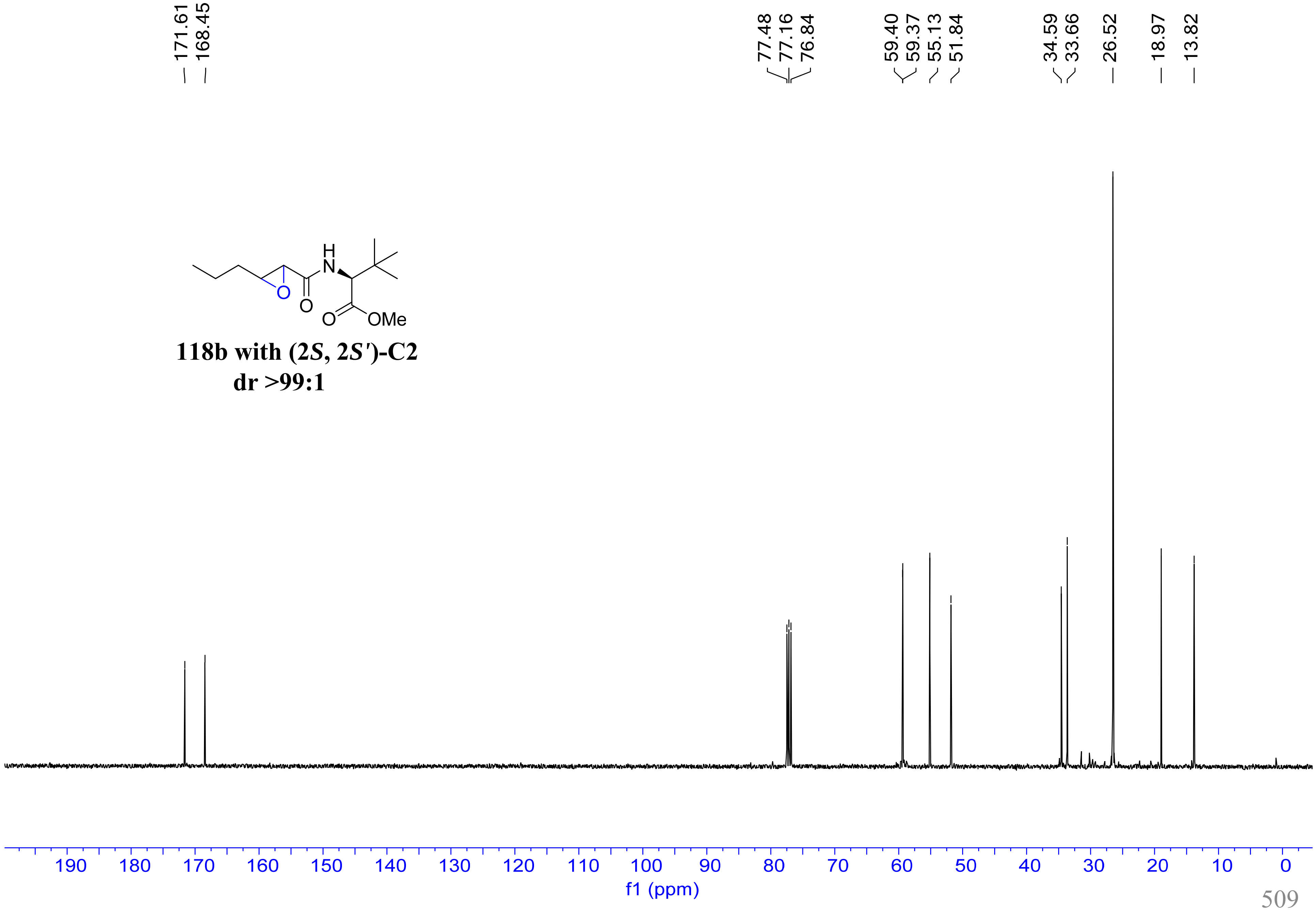

118b with (2*S*, 2*S*′)-C2
dr >99:1

^{1}H NMR, 400 MHz, CDCl$_3$

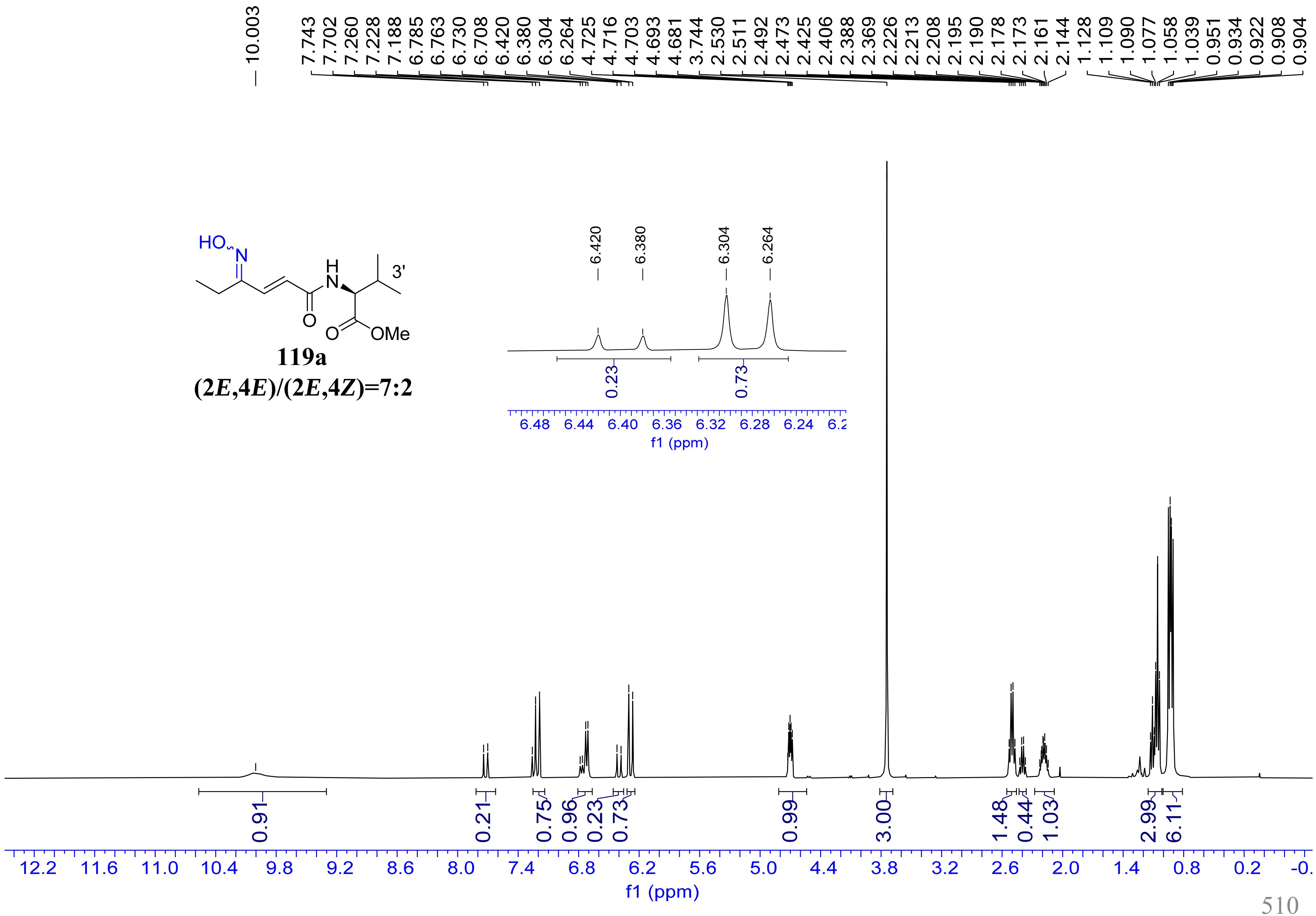

^{13}C NMR 101 MHz, $CDCl_3$

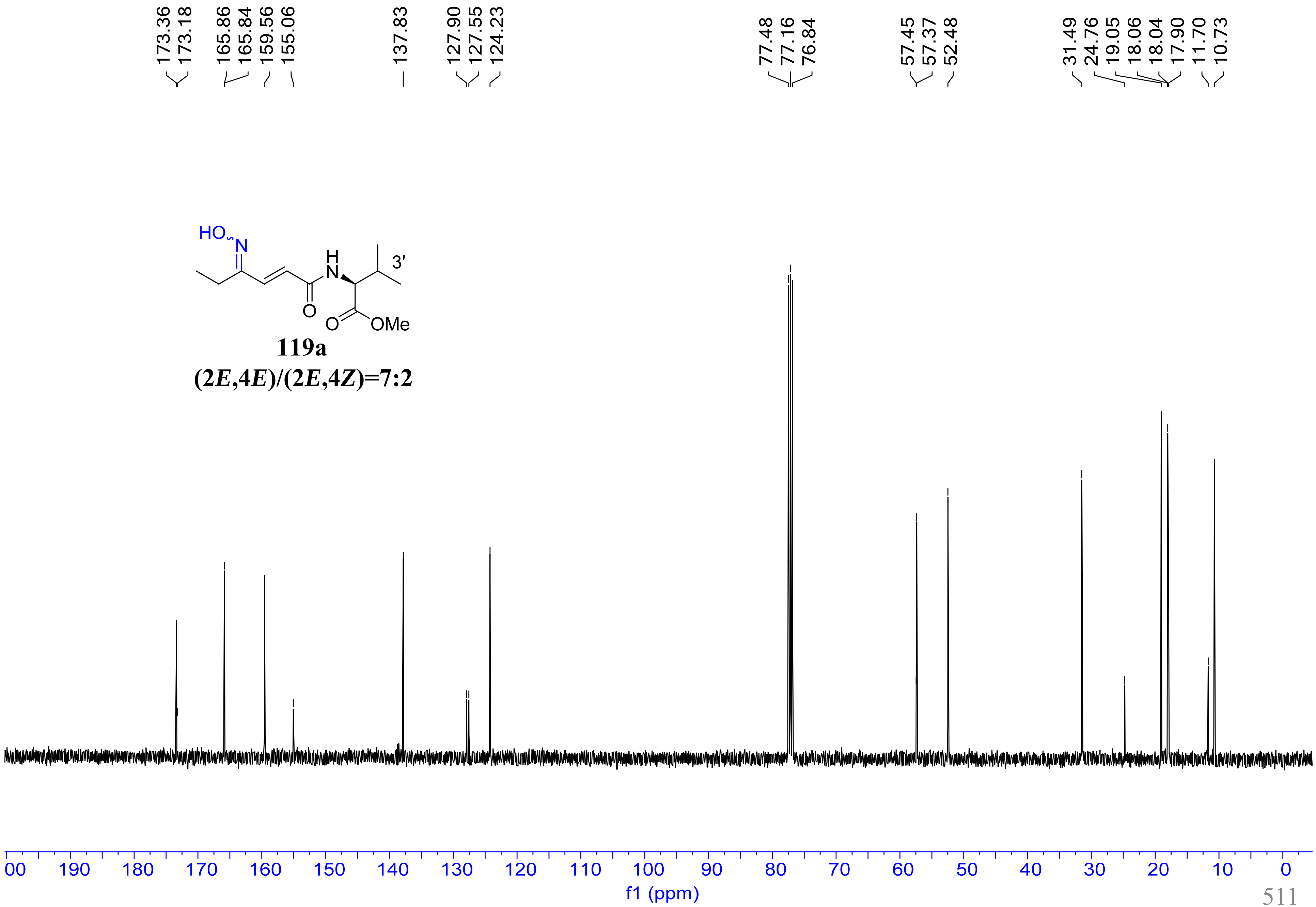

^{1}H NMR, 400 MHz, $CDCl_3$

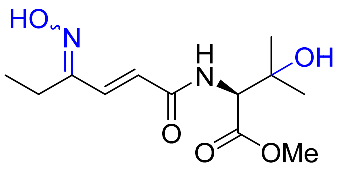

119a-3'-OH

(2*E*,4*E*)/(2*E*,4*Z*)=4:1

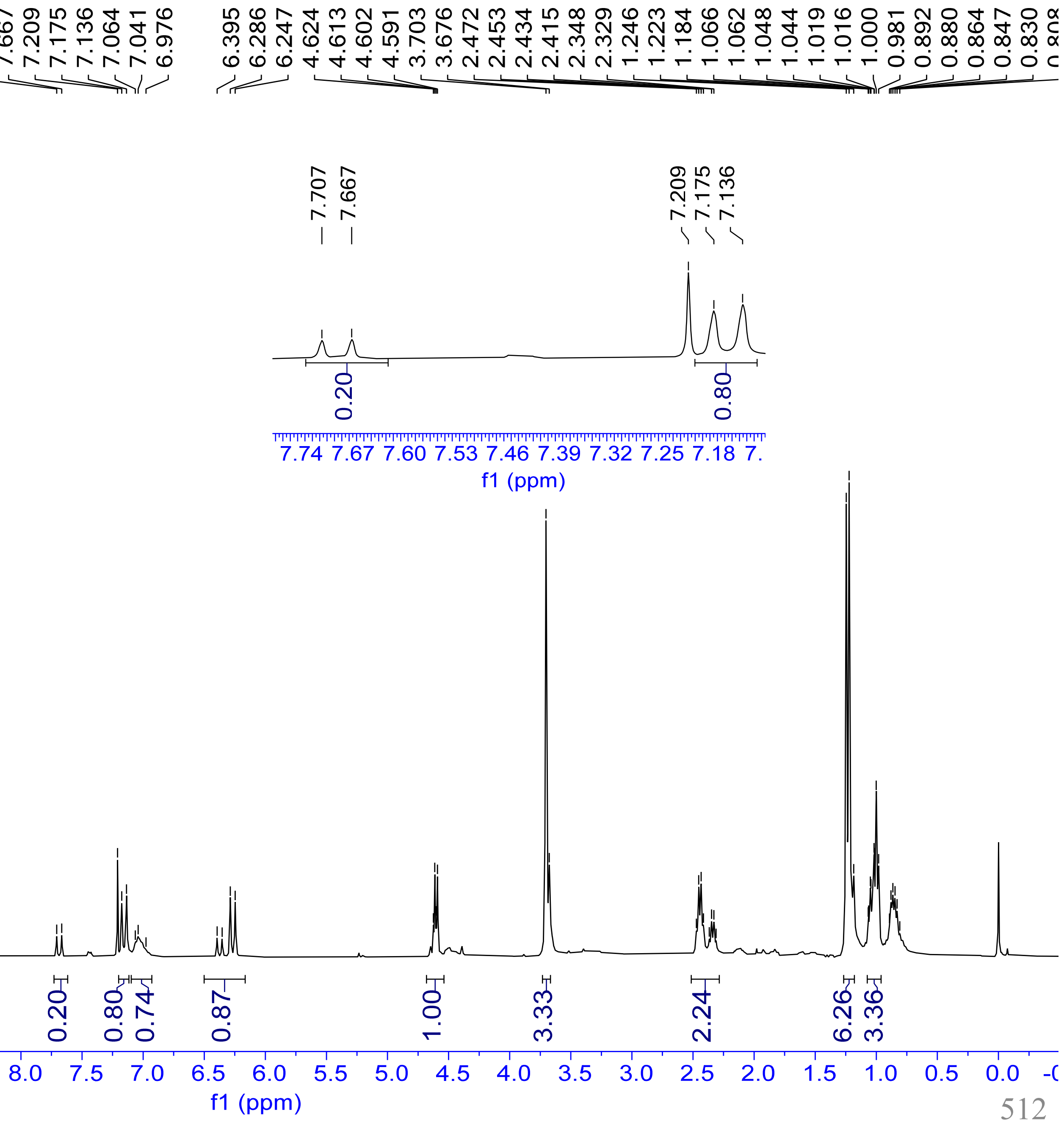

^{13}C NMR 101 MHz, $CDCl_3$

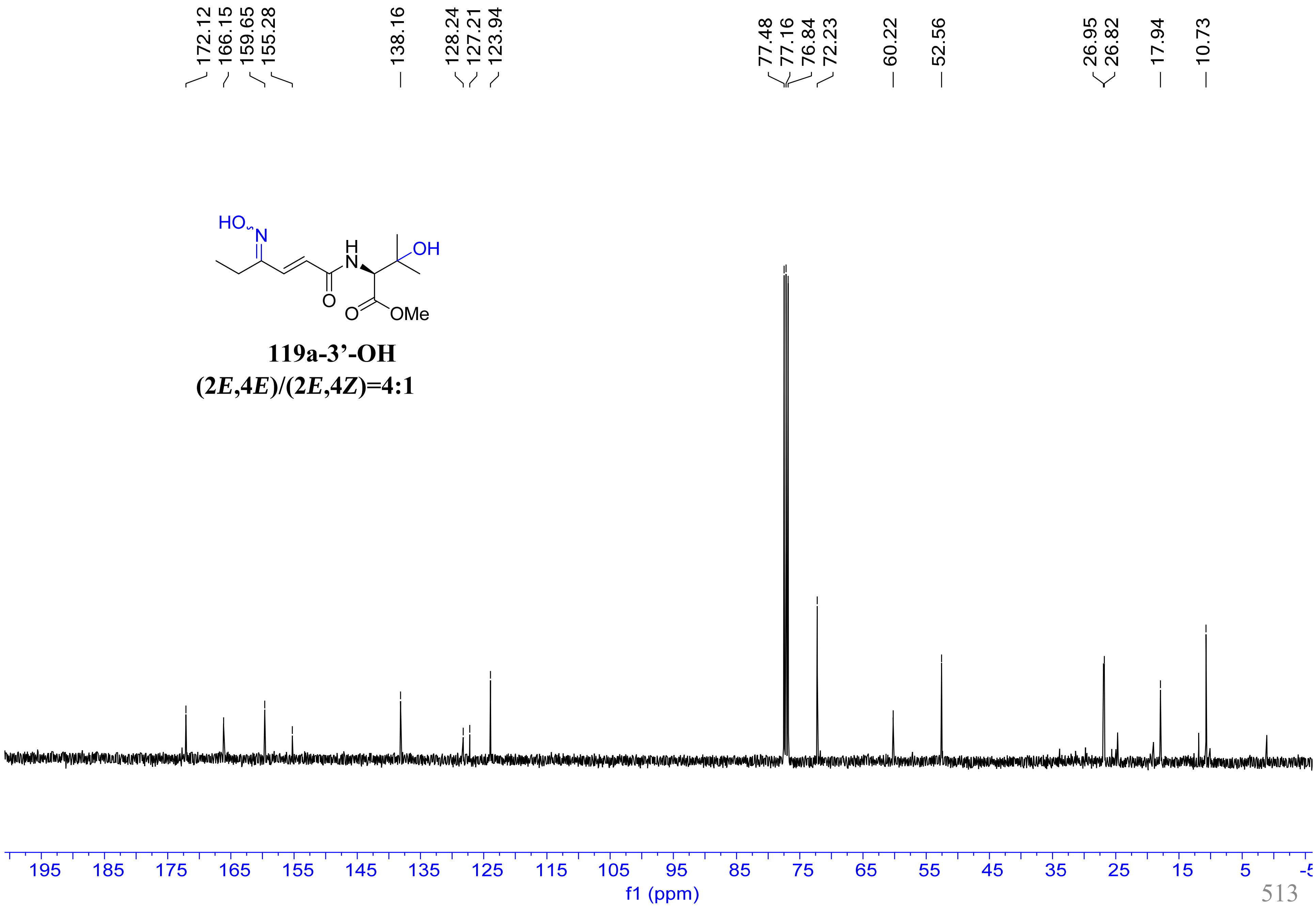

119a-3'-OH
(2*E*,4*E*)/(2*E*,4*Z*)=4:1

^{1}H NMR, 400 MHz, $CDCl_3$

120a, *E*-isomer

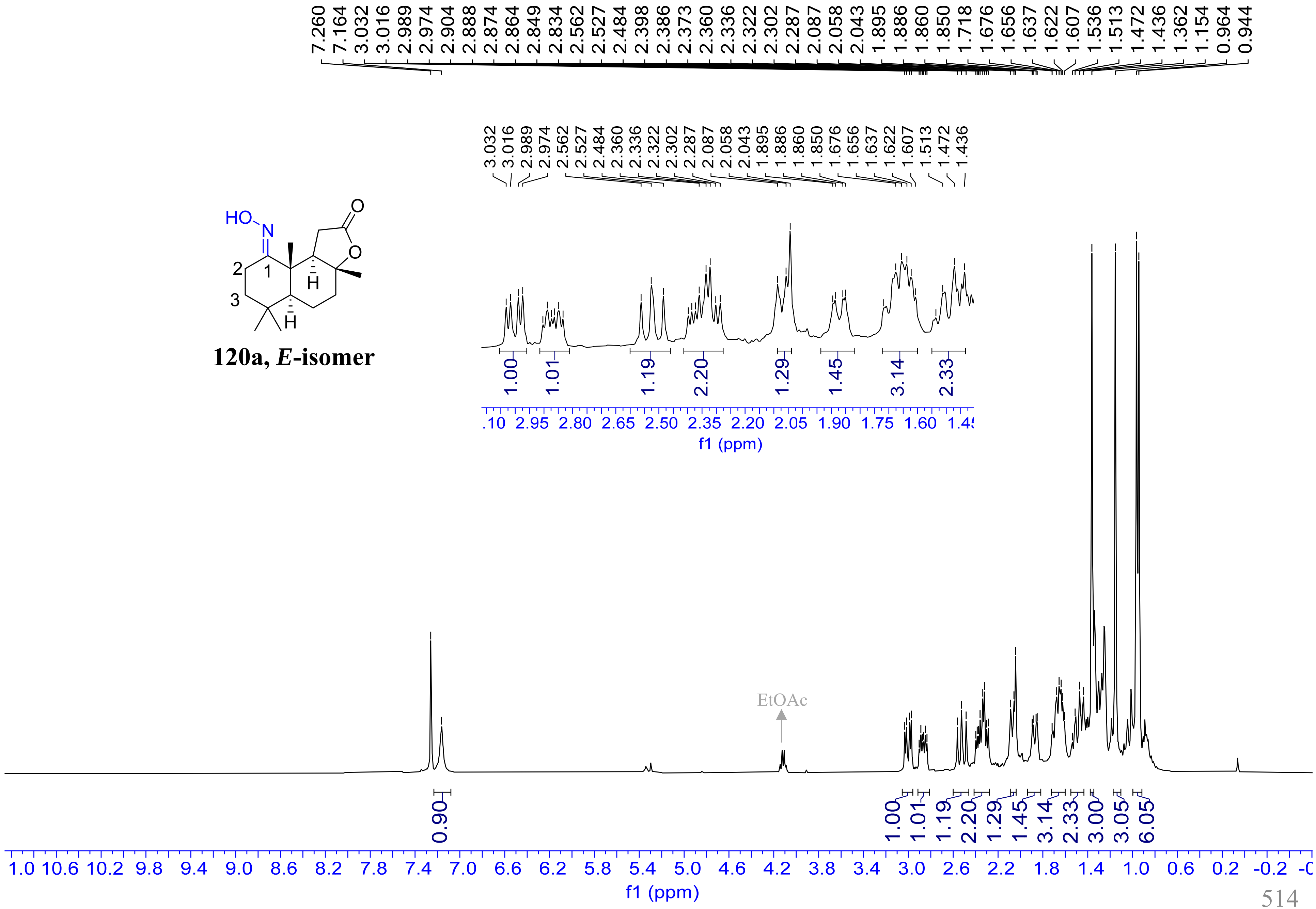

^{13}C NMR 101 MHz, $CDCl_3$

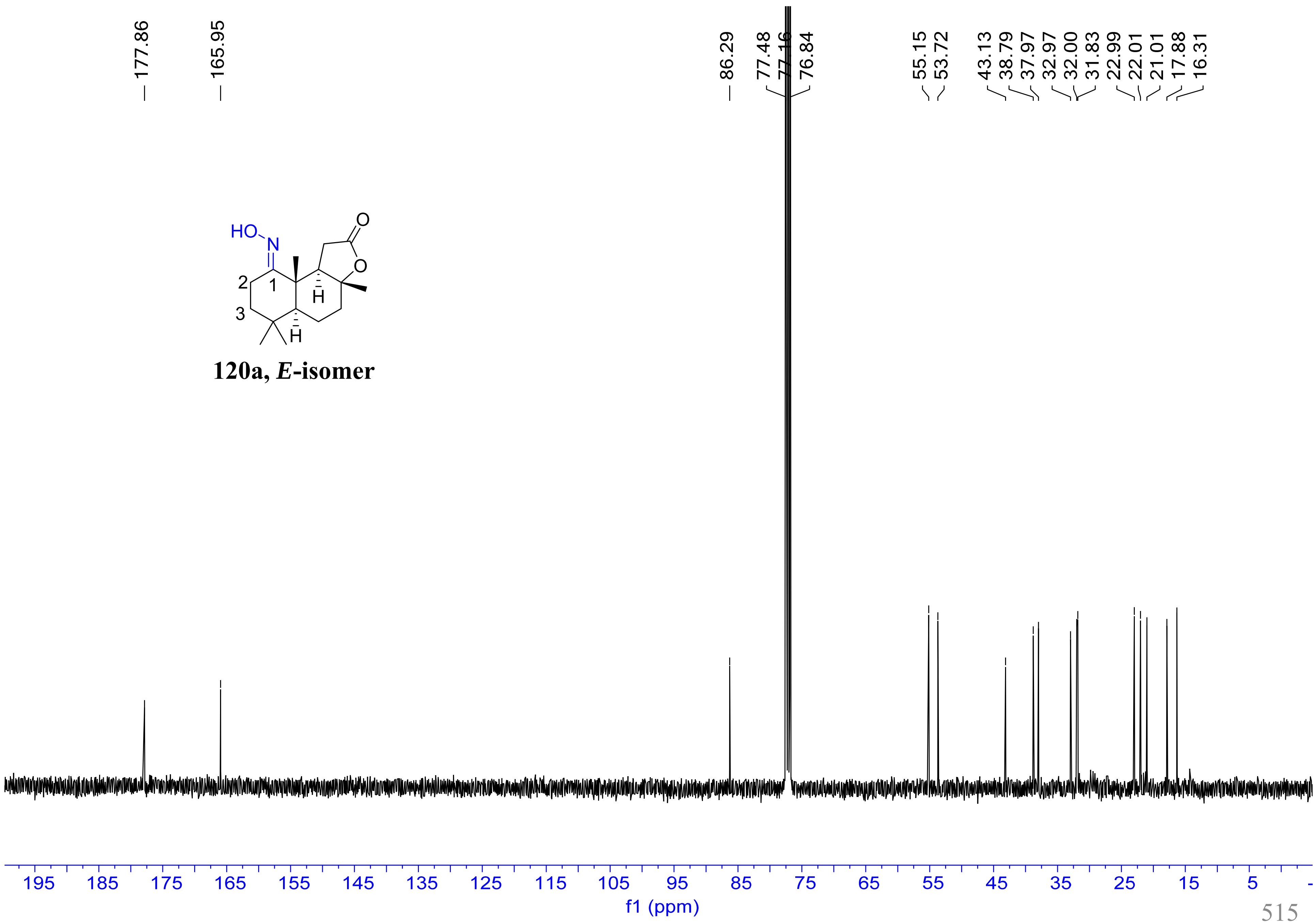

^{1}H NMR, 400 MHz, $CDCl_3$

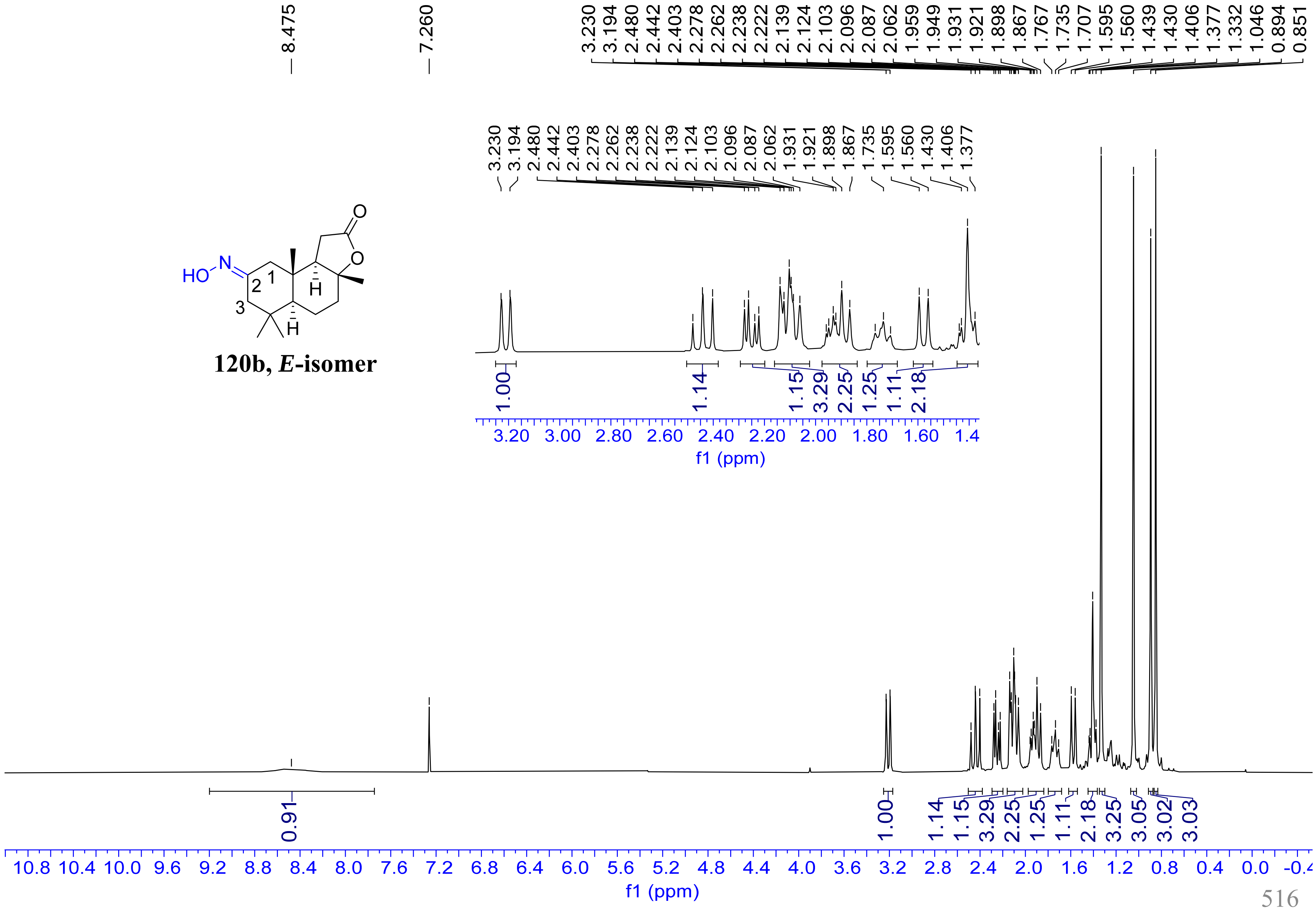

120b, *E*-isomer

^{13}C NMR 101 MHz, $CDCl_3$

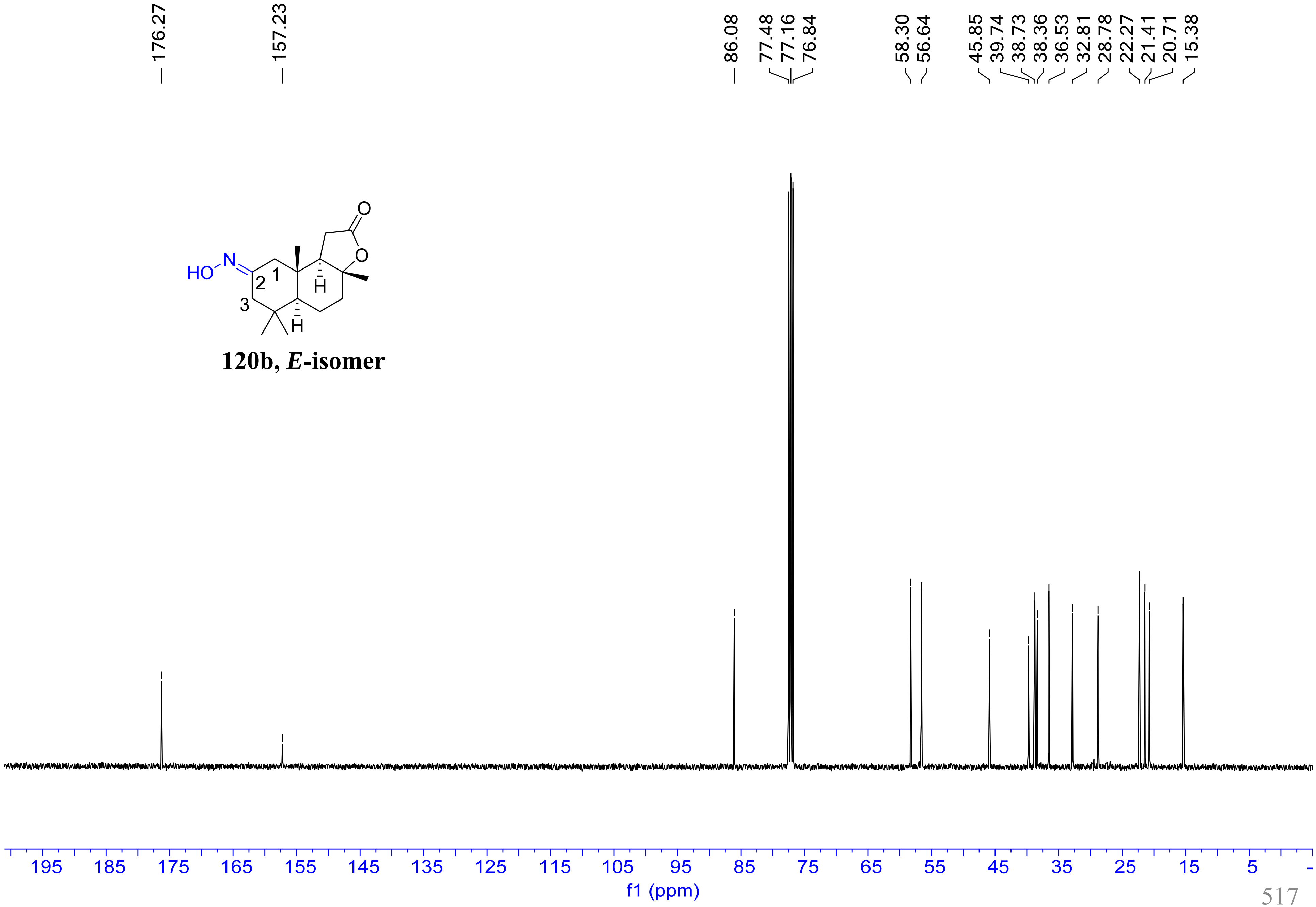

^{1}H NMR, 400 MHz, $CDCl_3$

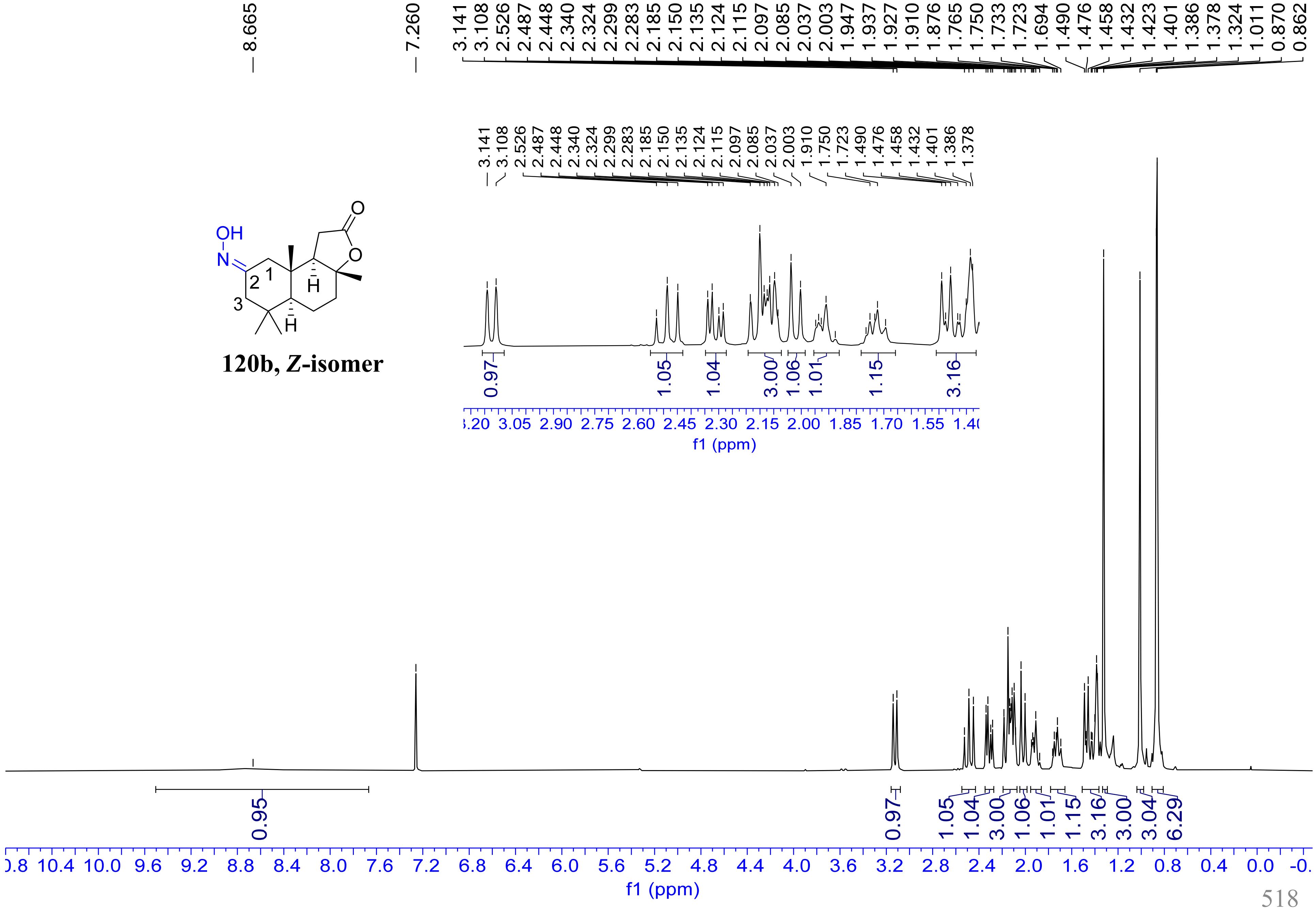

120b, *Z*-isomer

^{13}C NMR 101 MHz, $CDCl_3$

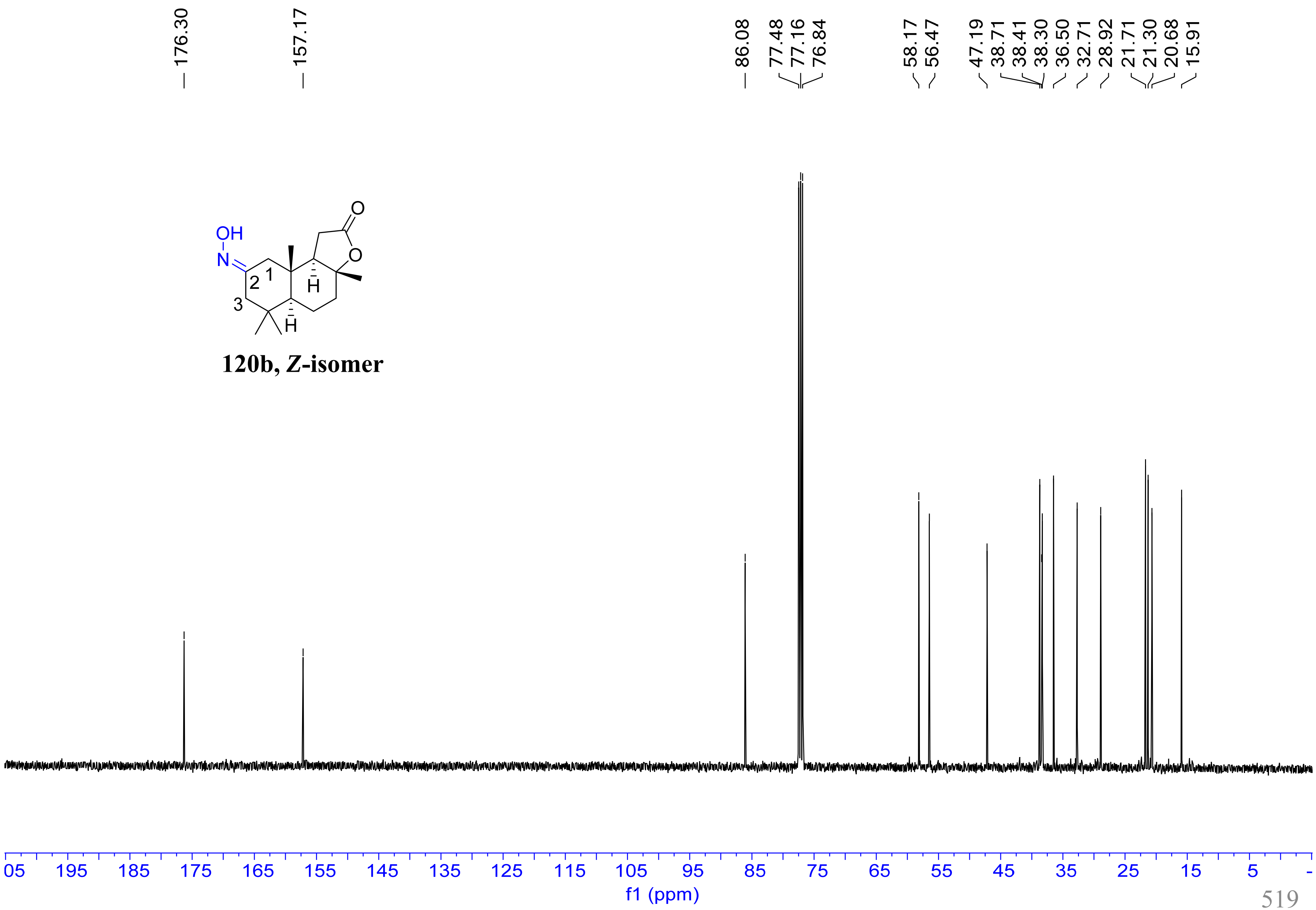

120b, *Z*-isomer

^{1}H NMR, 400 MHz, $CDCl_3$

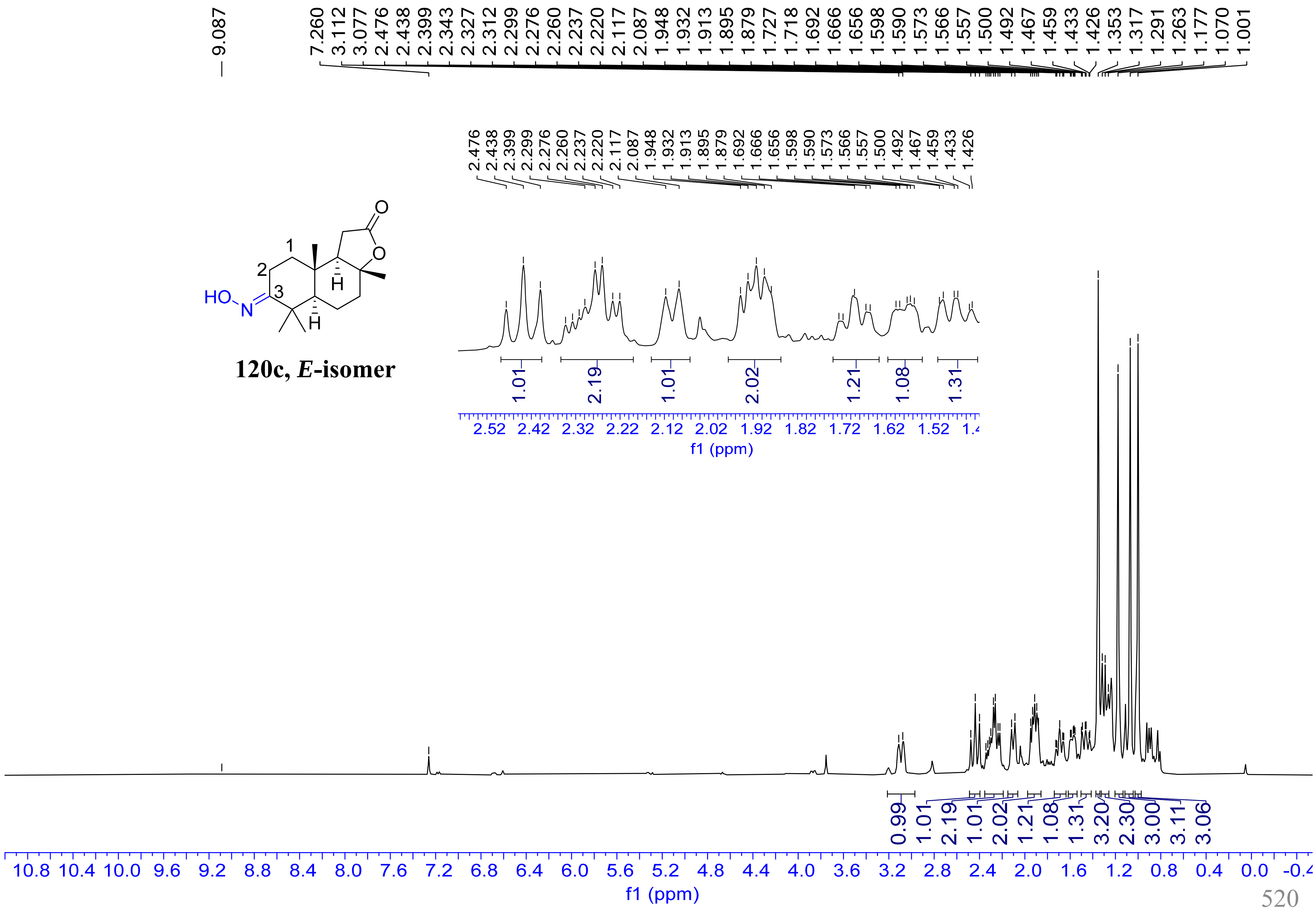

120c, *E*-isomer

^{13}C NMR 101 MHz, $CDCl_3$

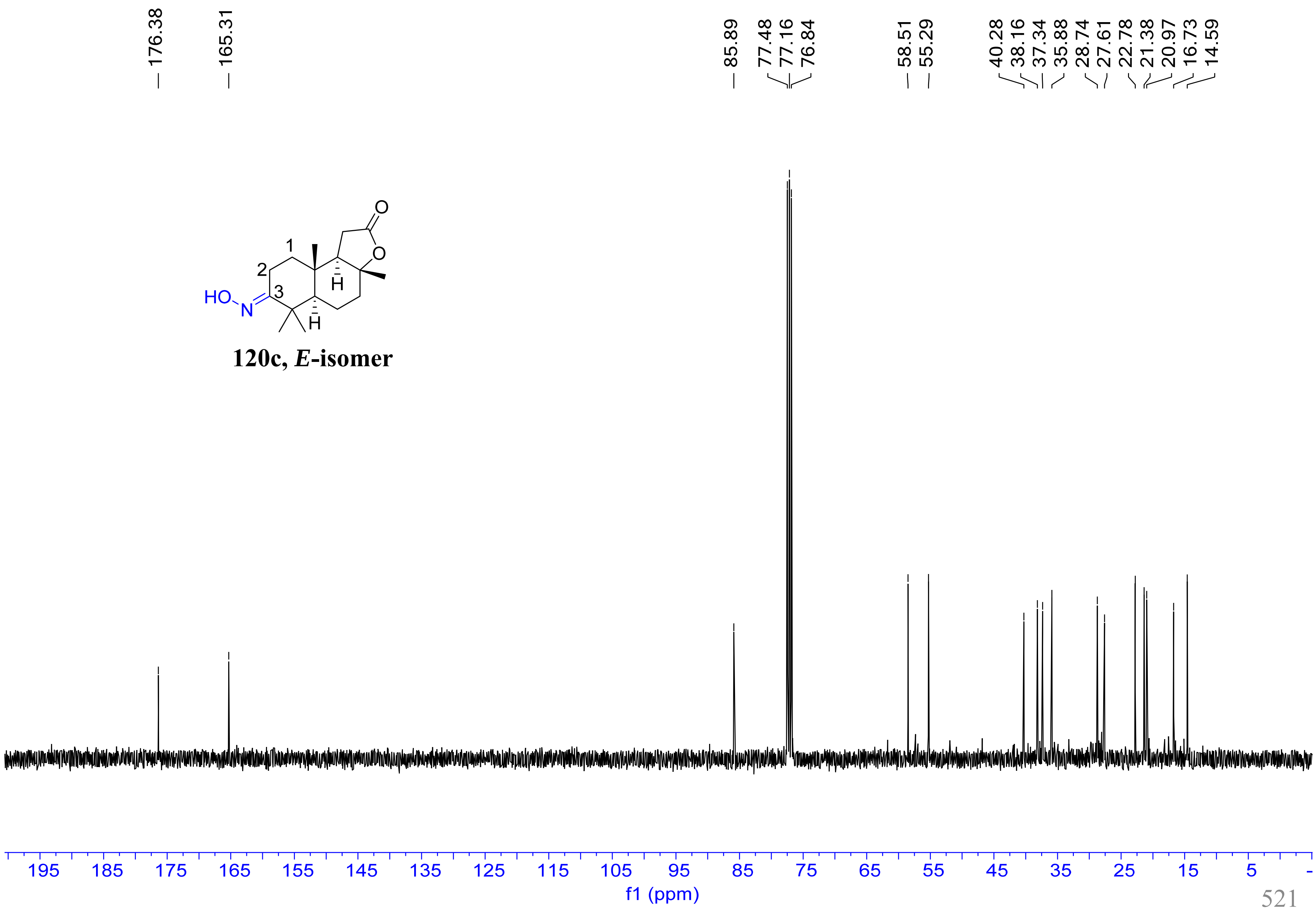

^{1}H NMR, 400 MHz, $CDCl_3$

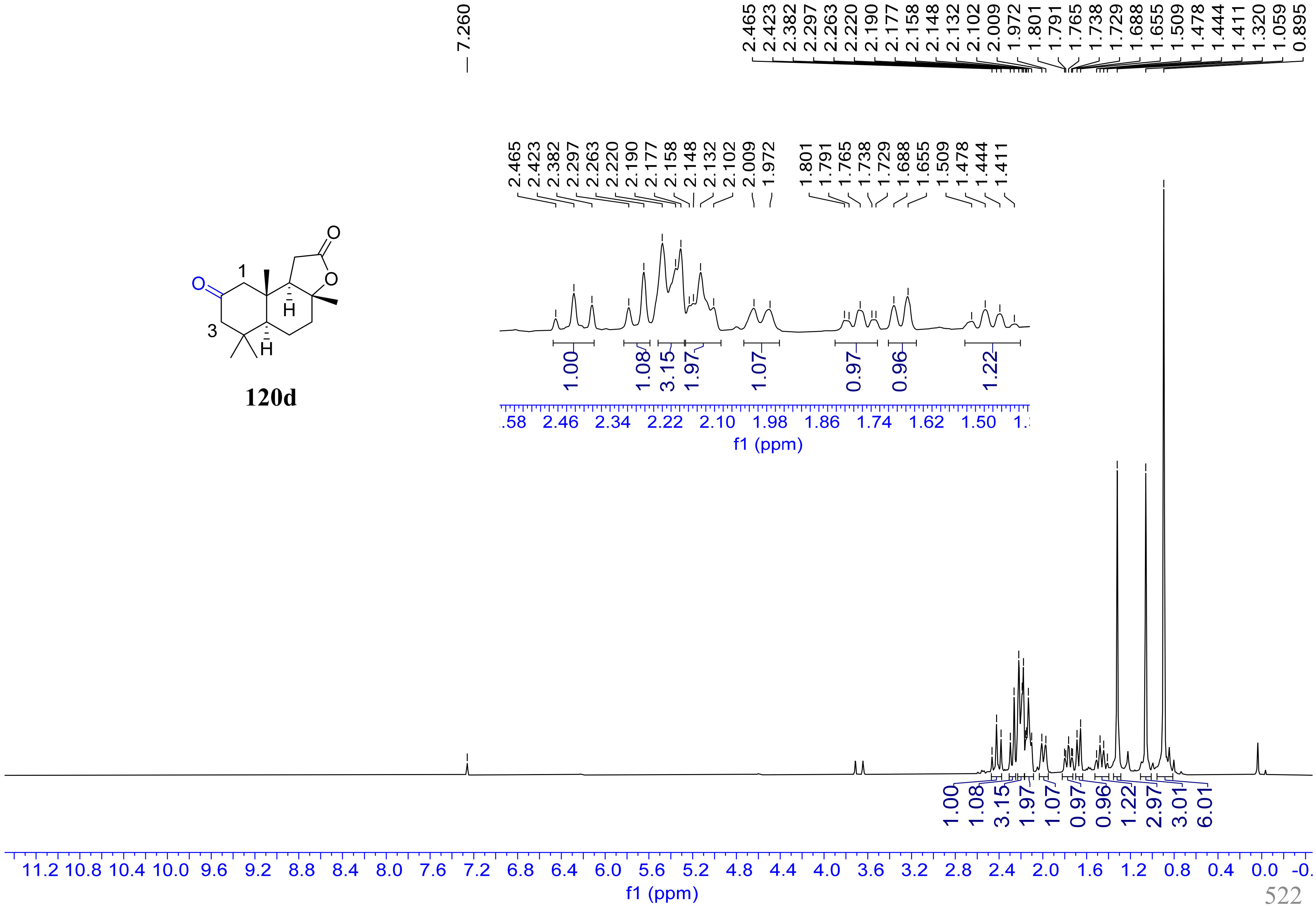

120d

^{13}C NMR 101 MHz, $CDCl_3$

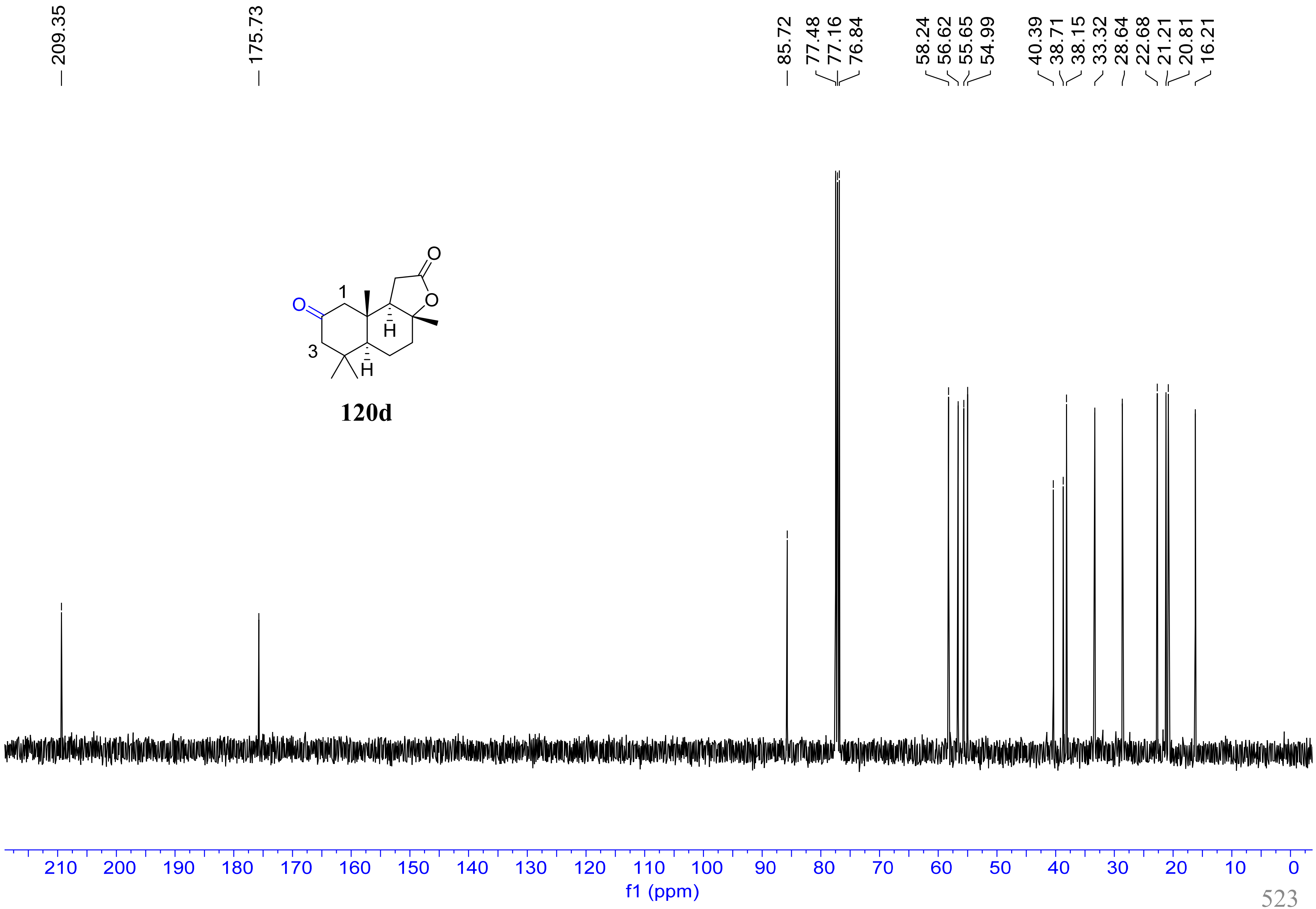

^{1}H NMR, 400 MHz, $CDCl_3$

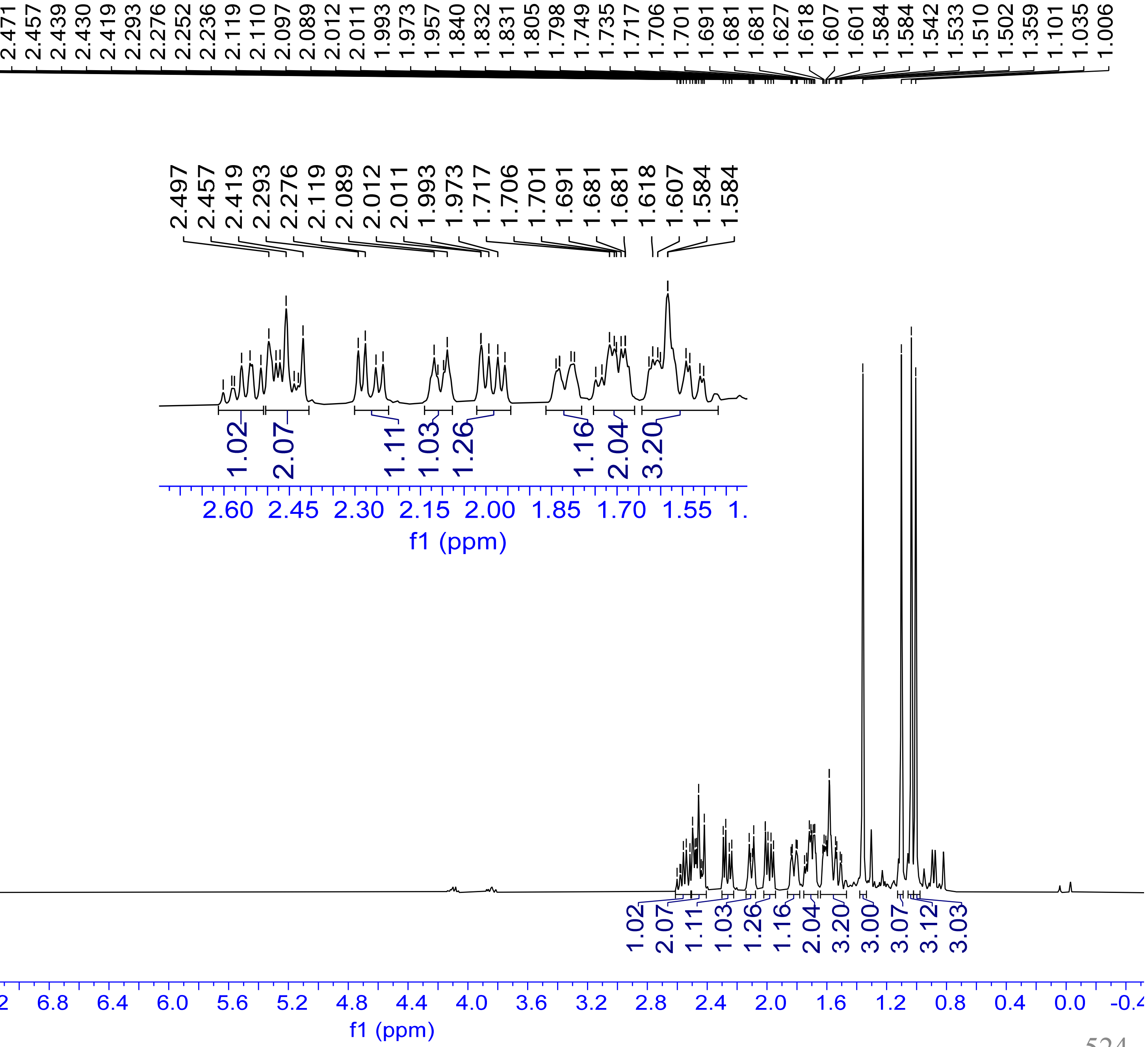

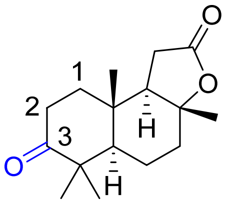

120e

^{13}C NMR 101 MHz, $CDCl_3$

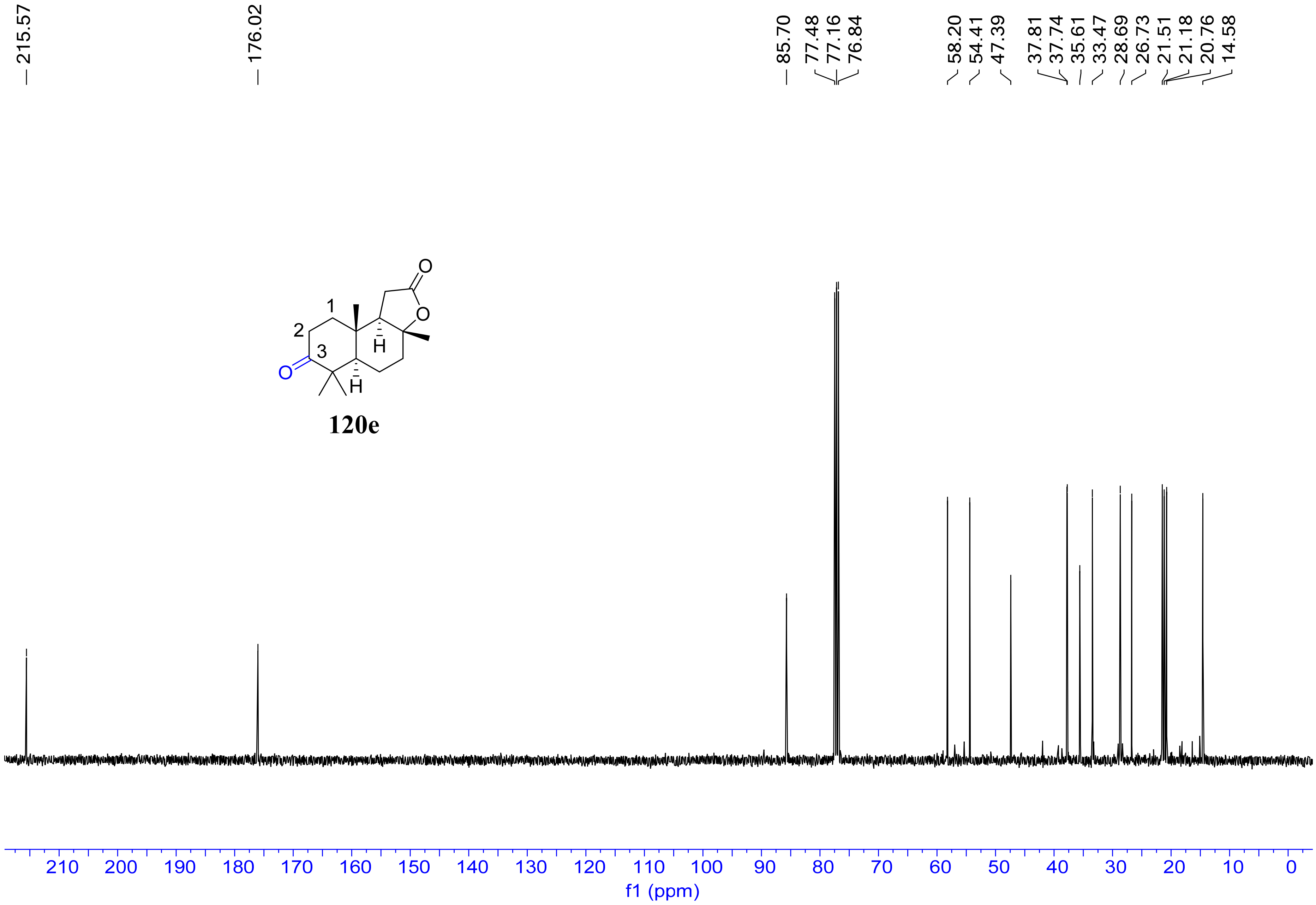

120e

^{1}H NMR, 400 MHz, $CDCl_3$

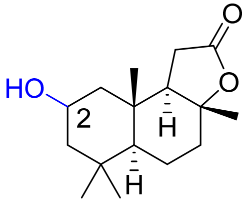

120f

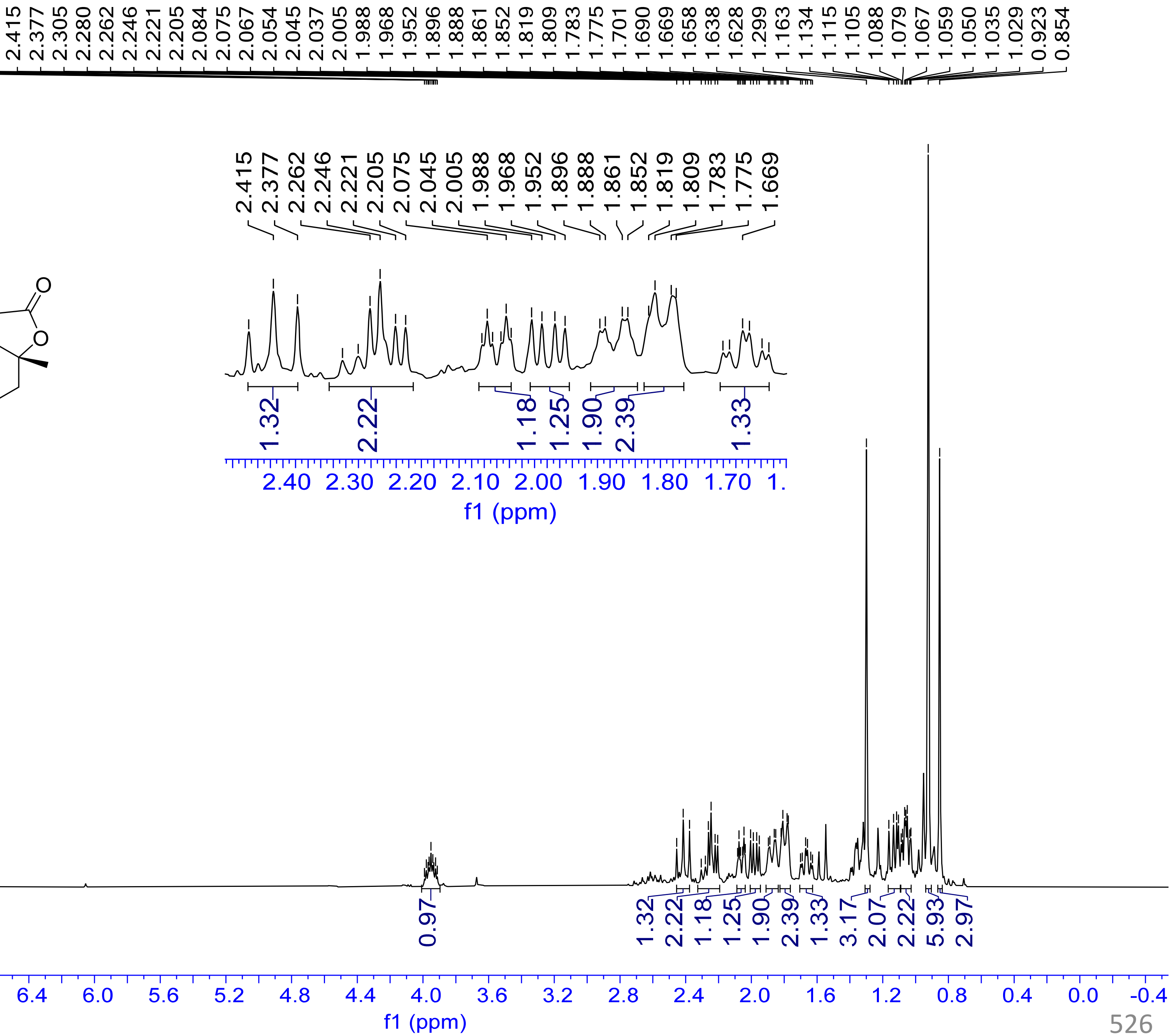

^{13}C NMR 101 MHz, $CDCl_3$

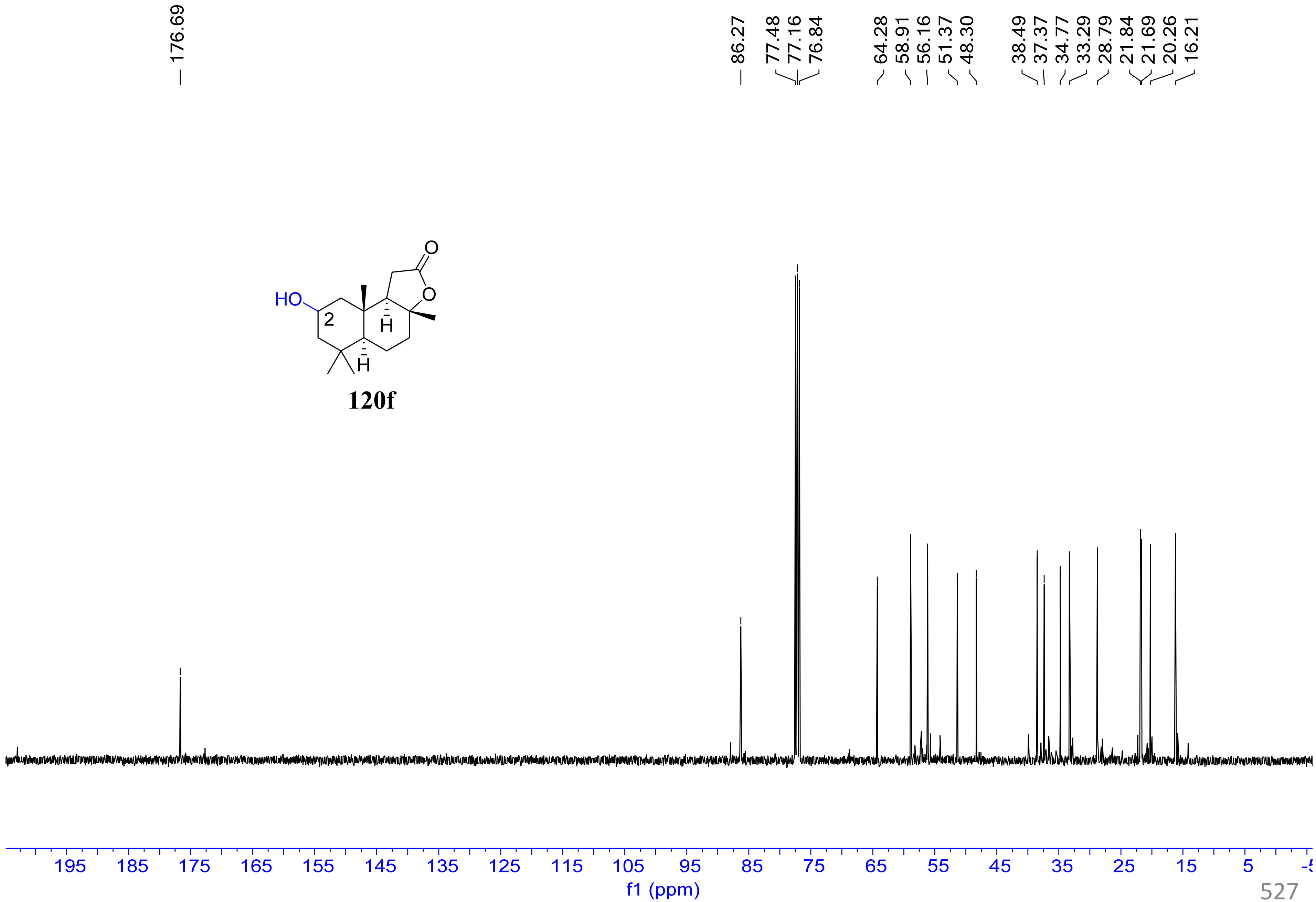

^{1}H NMR, 400 MHz, $CDCl_3$

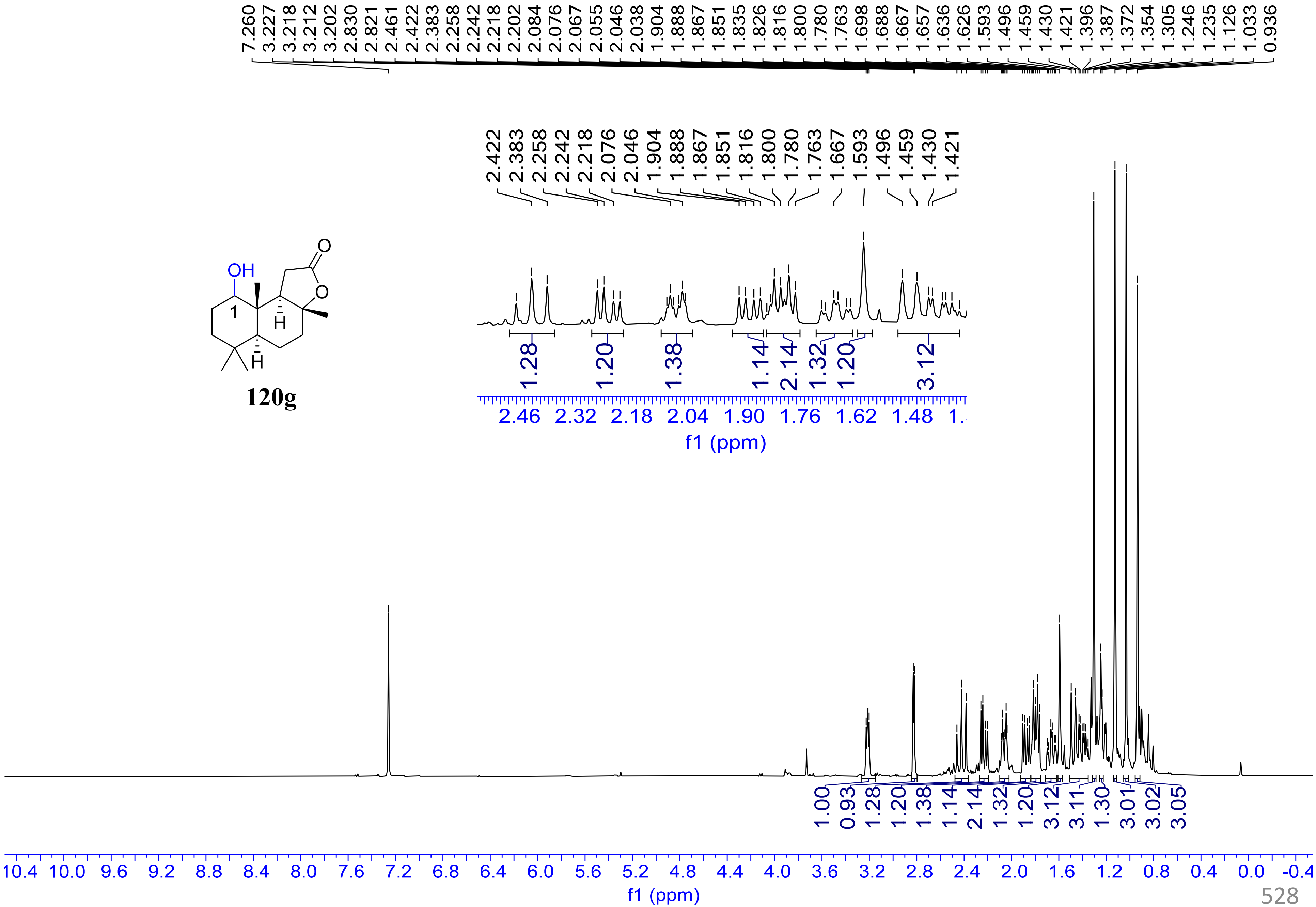

^{13}C NMR 101 MHz, $CDCl_3$

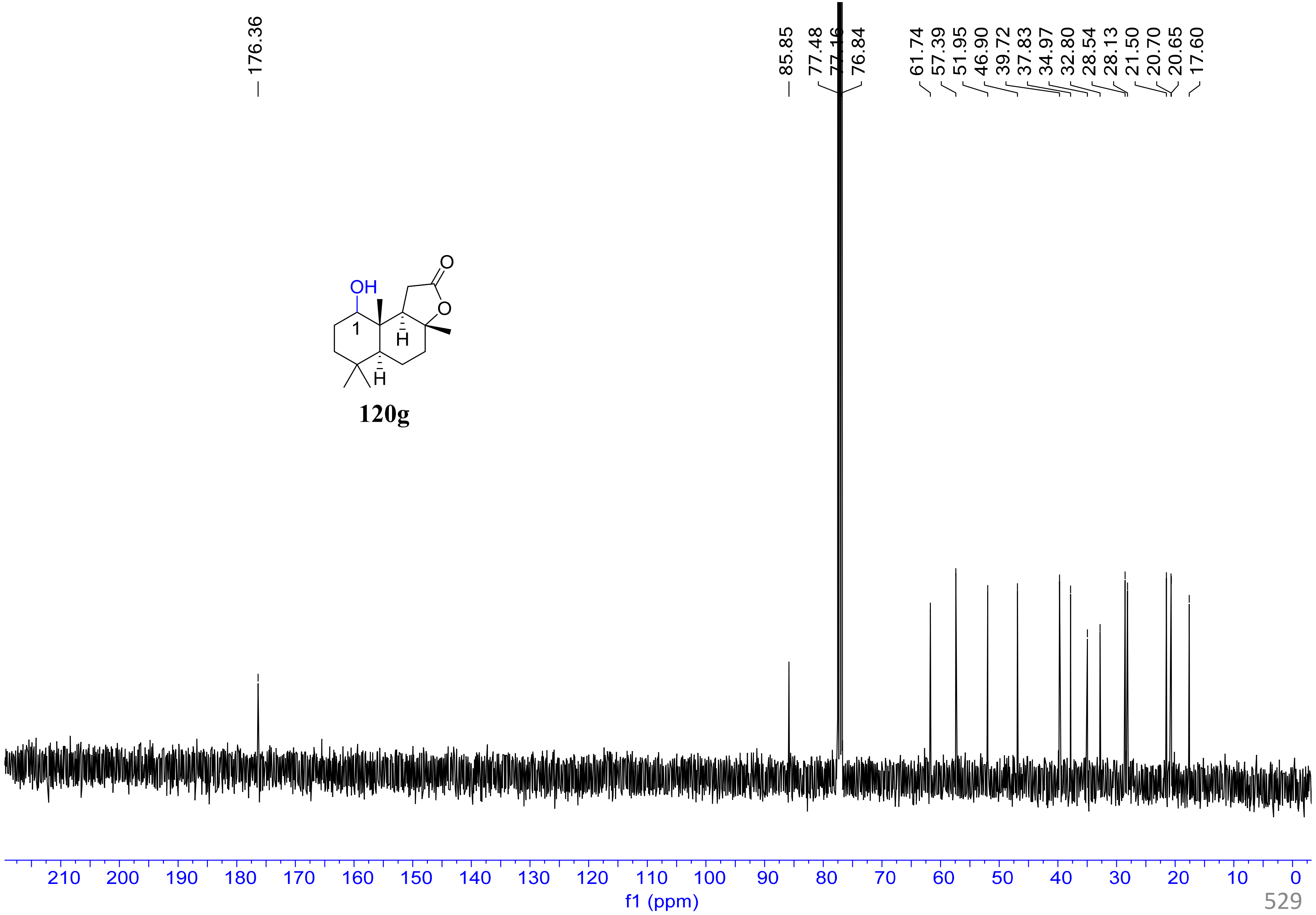

^{1}H NMR, 400 MHz, $CDCl_3$

121a

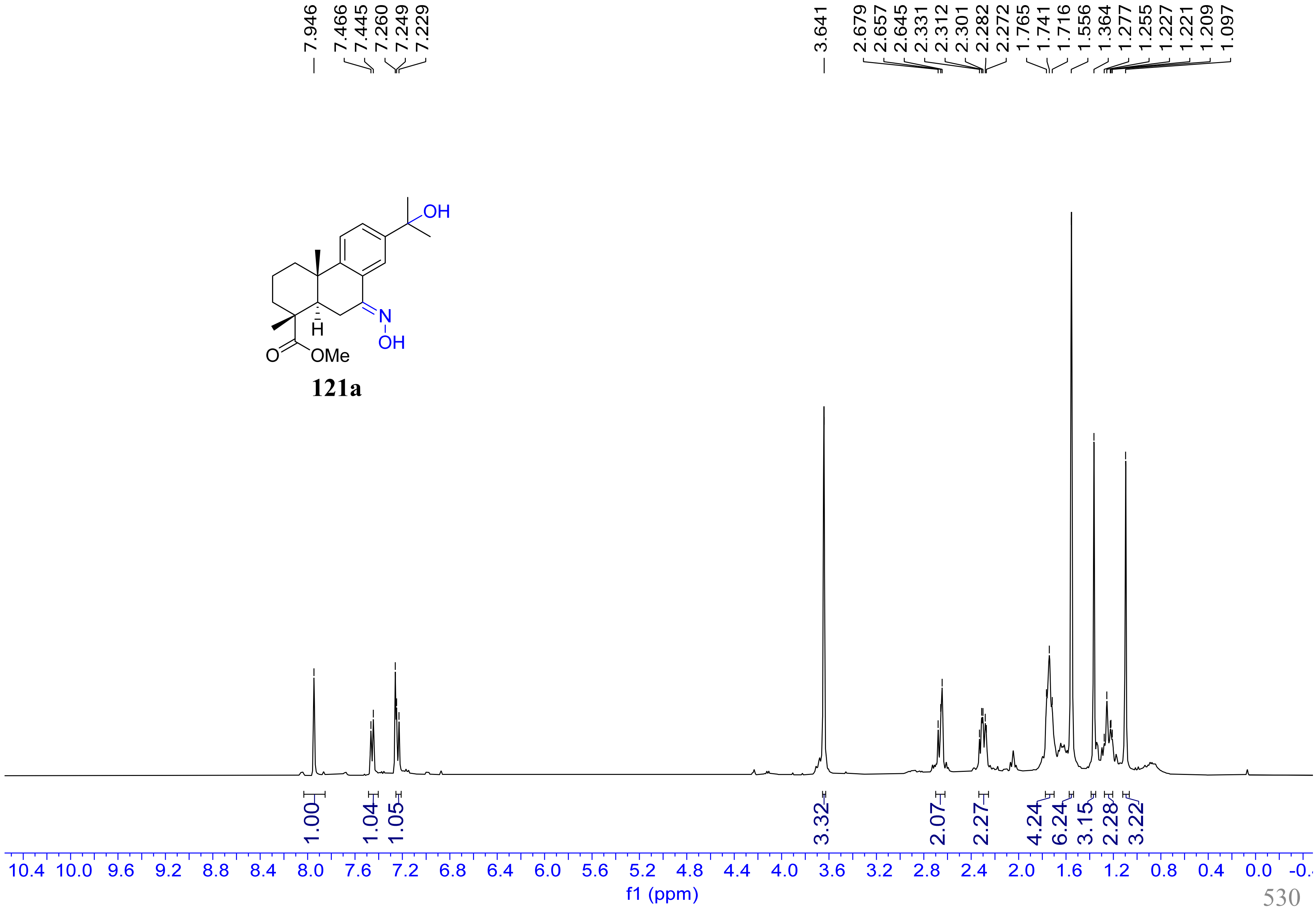

^{13}C NMR 101 MHz, $CDCl_3$

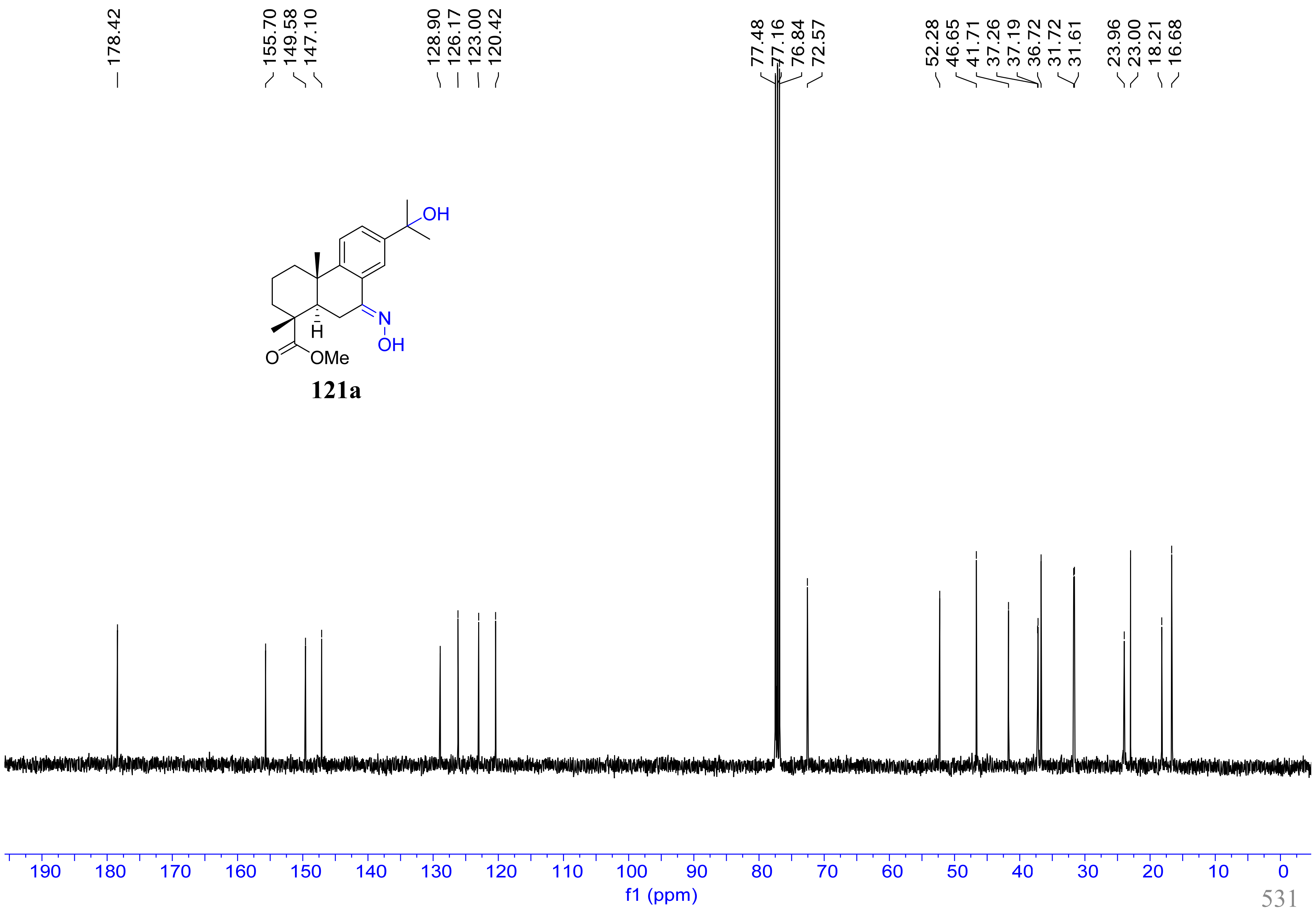

121a

^{1}H NMR, 400 MHz, $CDCl_3$

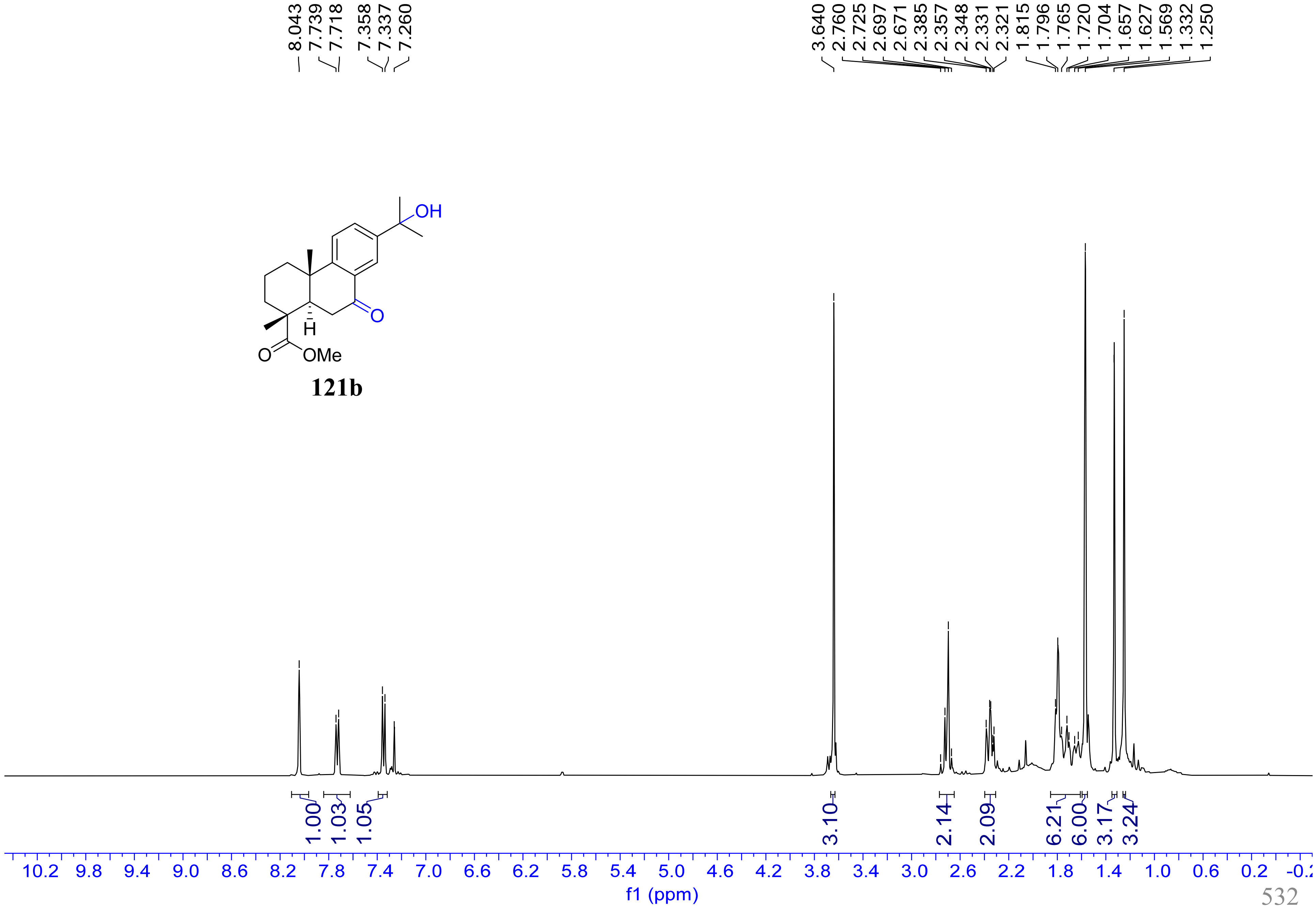

121b

^{13}C NMR 101 MHz, $CDCl_3$

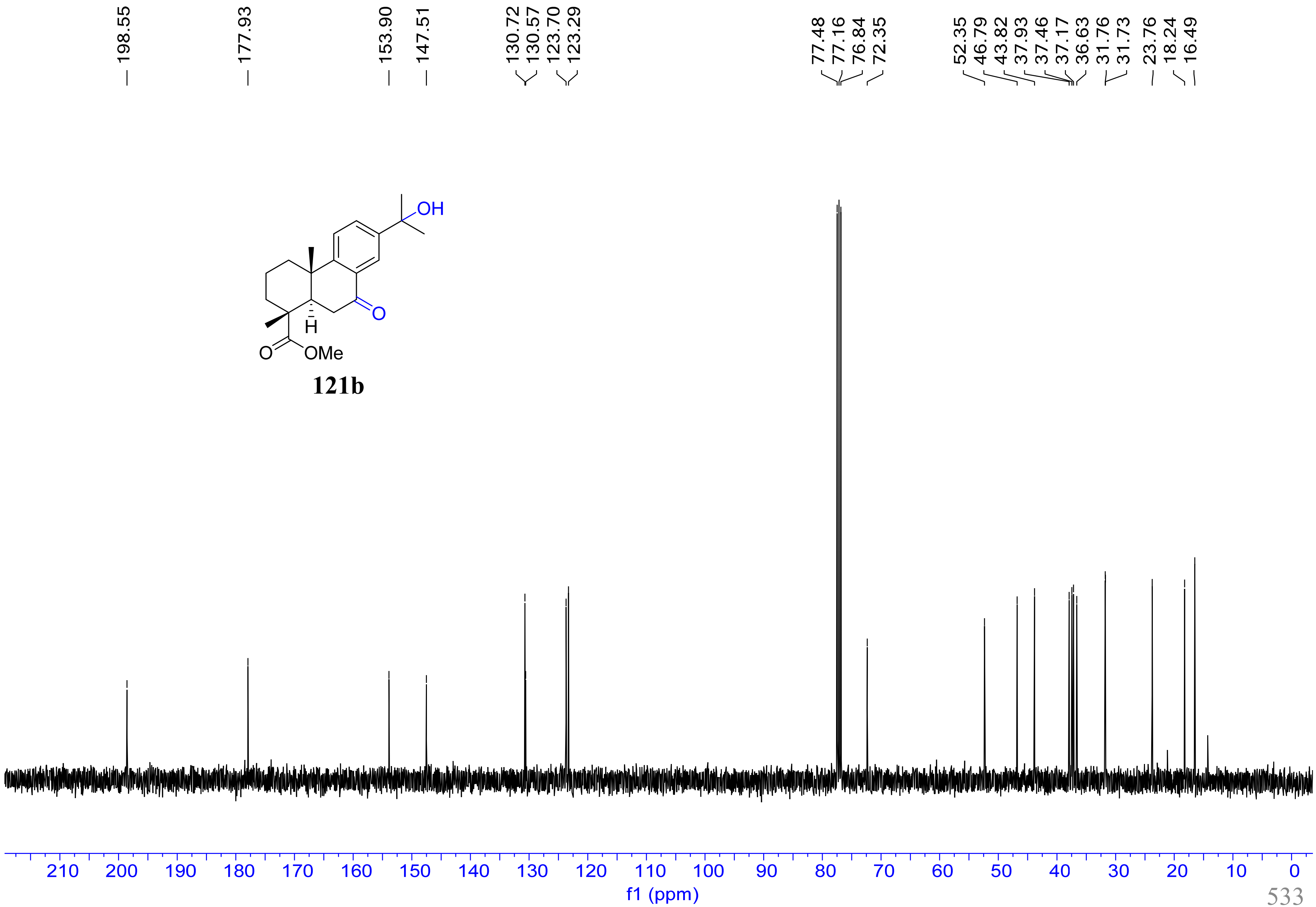

^{1}H NMR, 400 MHz, DMSO-d6

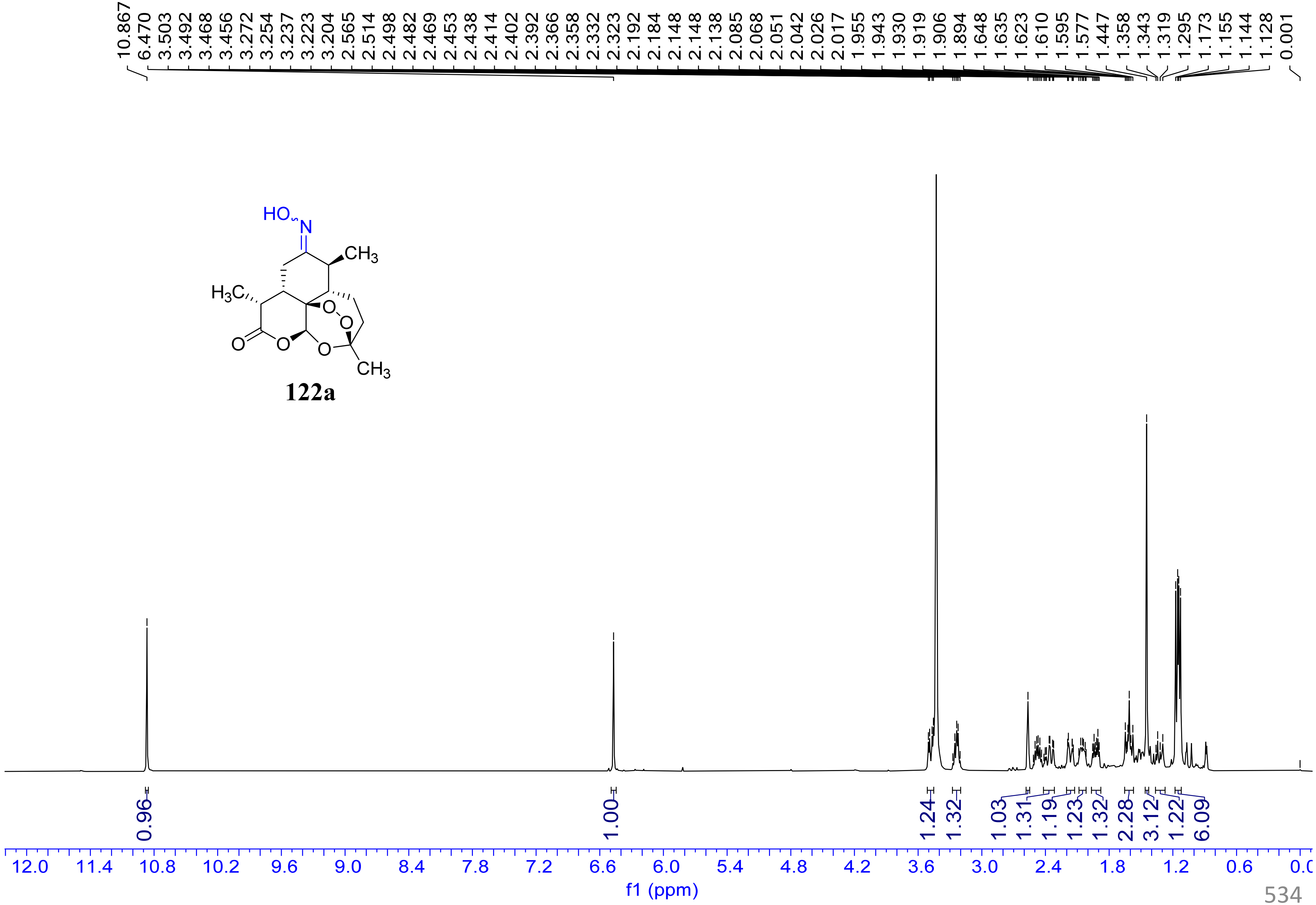

^{13}C NMR 101 MHz, DMSO-d6

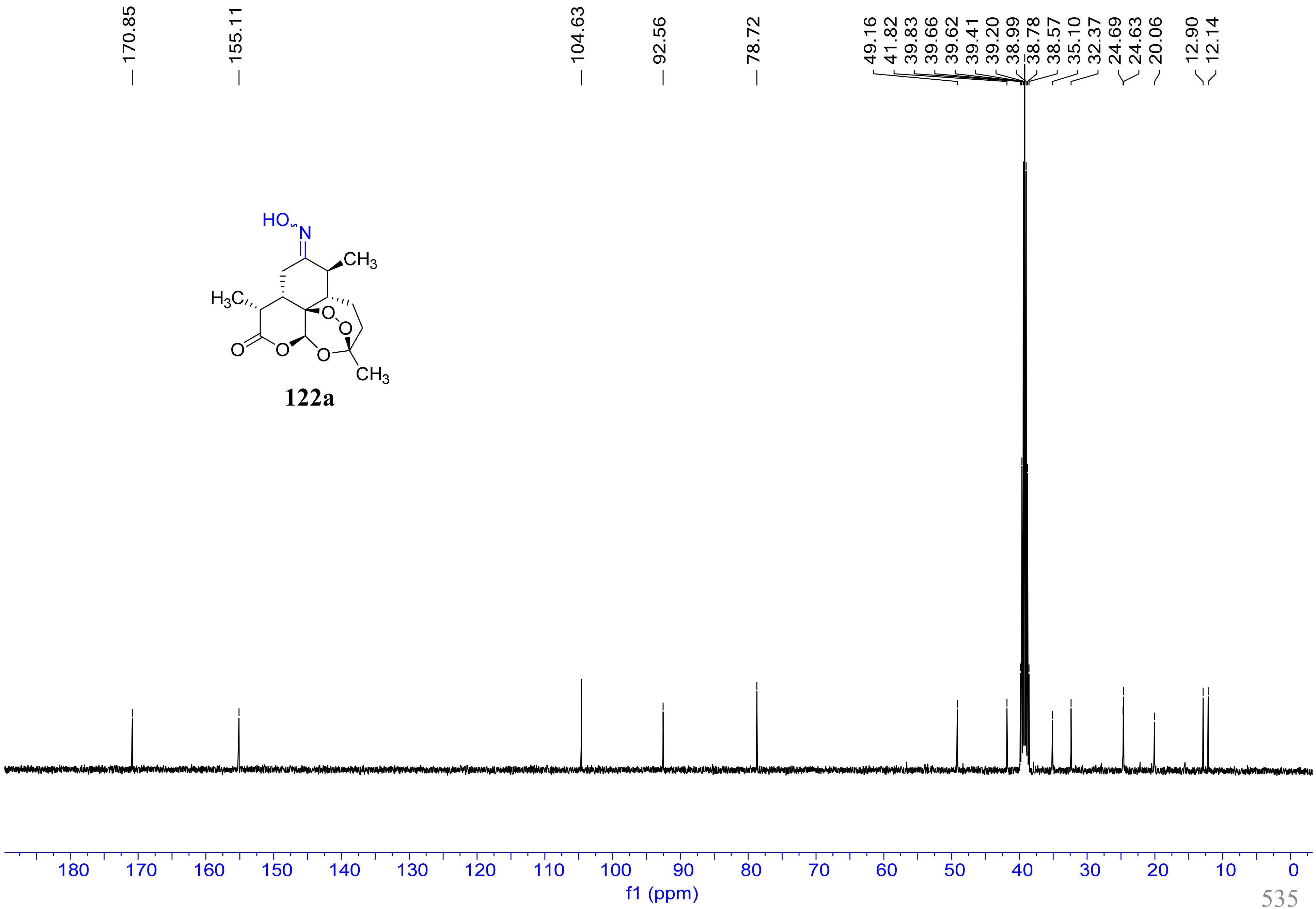

16. HPLC Traces

Chromatogram

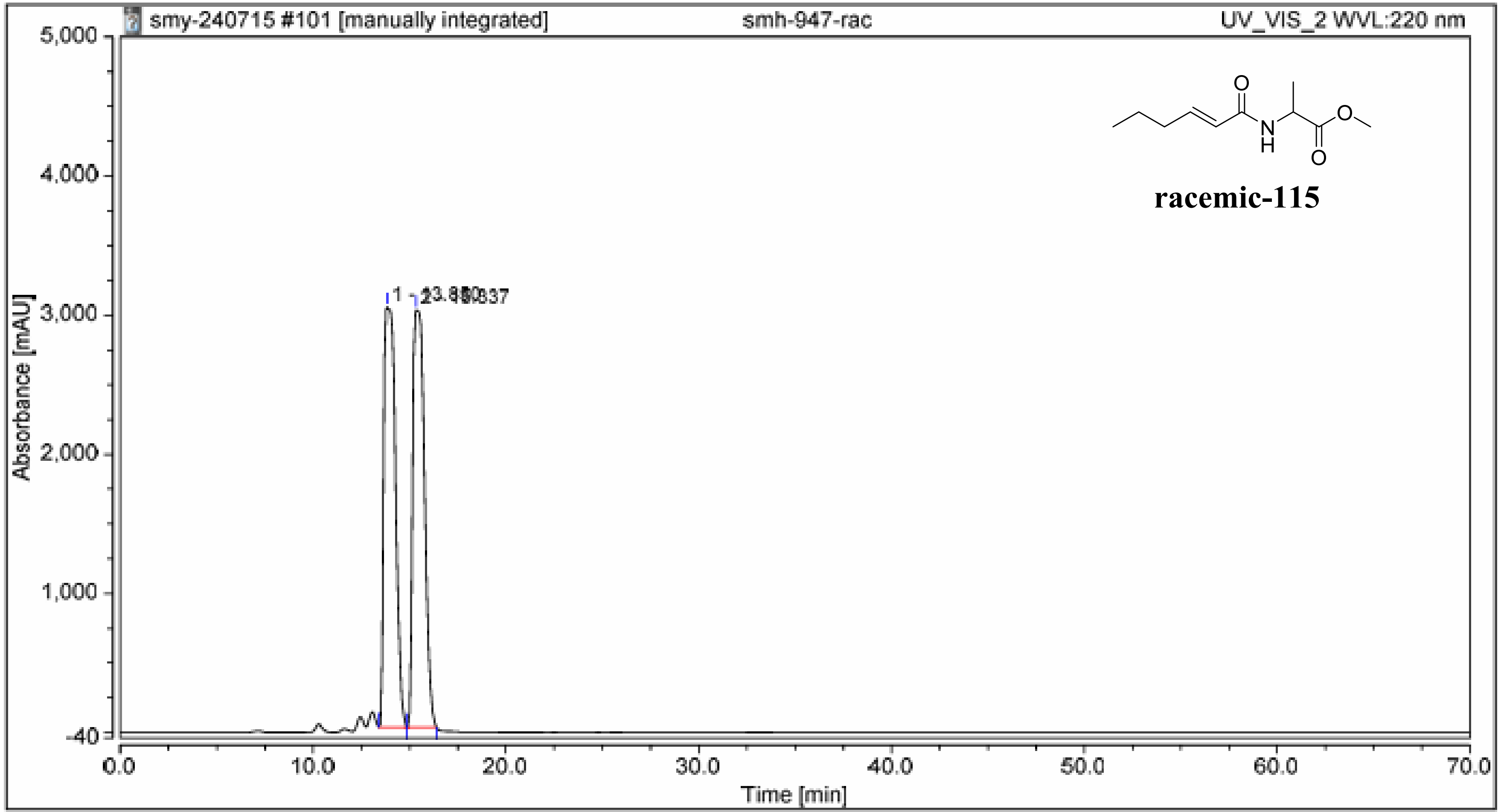

Integration Results

No.	Retention Time min	Area mAU*min	Relative Area %	Relative Height %	Amount n.a.
1	13.850	2047.724	48.98	50.17	n.a.
2	15.337	2132.701	51.02	49.83	n.a.
**Total:**		**4180.426**	**100.00**	**100.00**	

Chromatogram

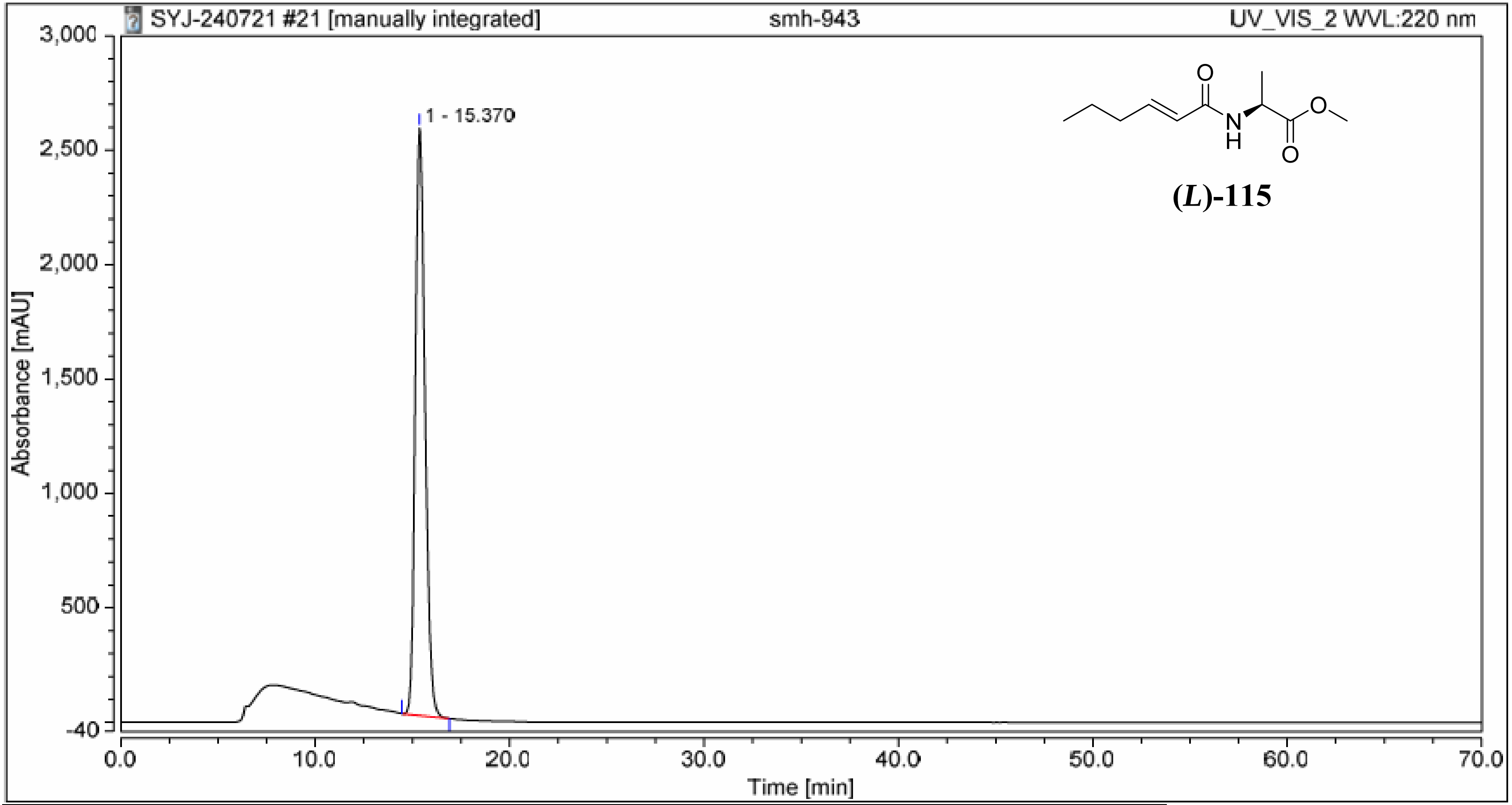

(*L*)-115

Integration Results

No.	Retention Time min	Area mAU*min	Relative Area %	Relative Height %	Amount n.a.
1	15.370	1507.230	100.00	100.00	n.a.
**Total:**		**1507.230**	**100.00**	**100.00**	

Chromatogram

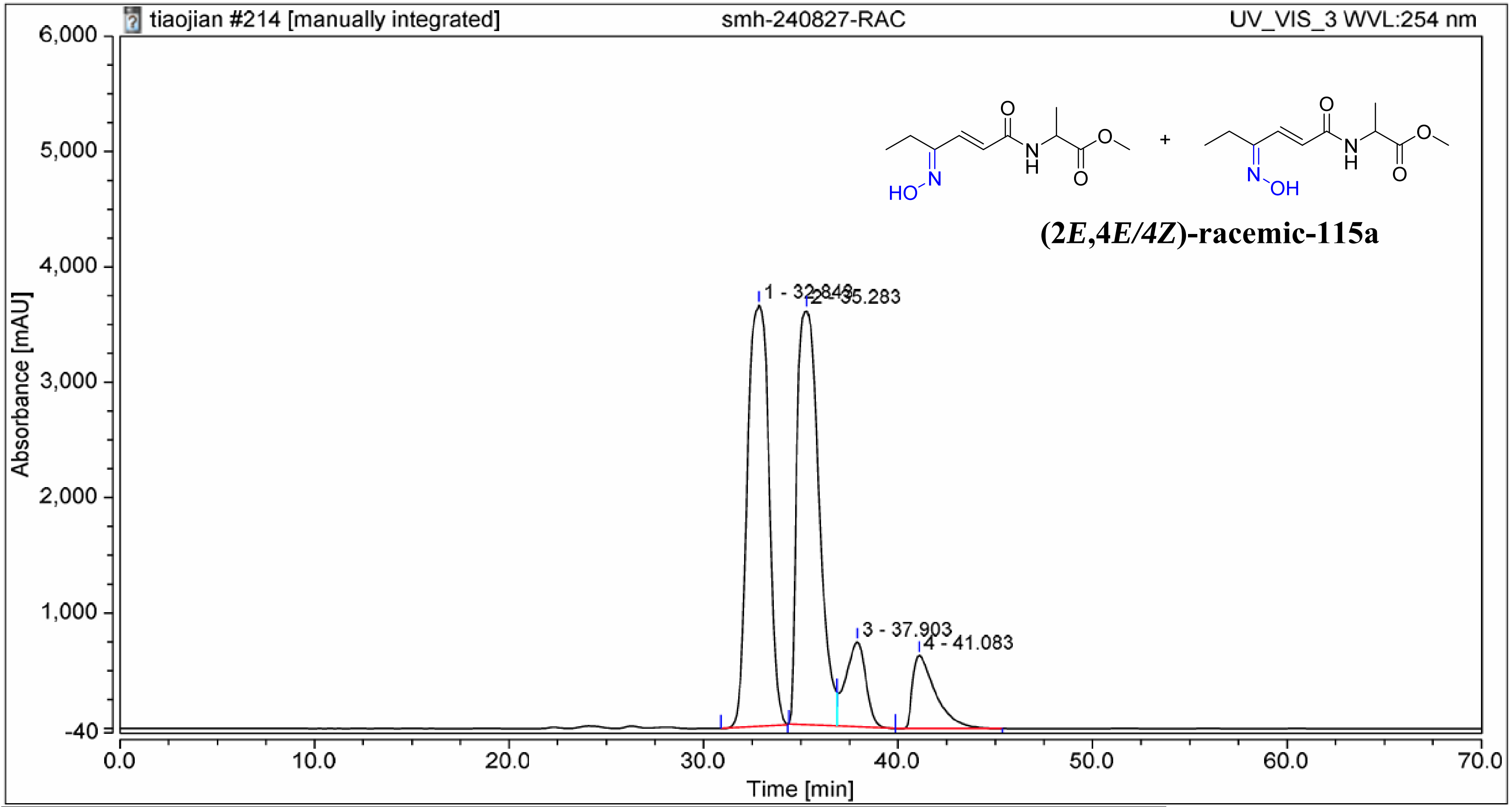

Integration Results

No.	Retention Time<br>min	Area<br>mAU*min	Relative Area<br>%	Relative Height<br>%	Amount<br>n.a.
1	32.843	4656.898	42.04	42.38	n.a.
2	35.283	4666.573	42.13	41.76	n.a.
3	37.903	894.617	8.08	8.54	n.a.
4	41.083	859.284	7.76	7.33	n.a.
**Total:**		**11077.372**	**100.00**	**100.00**	

Chromatogram

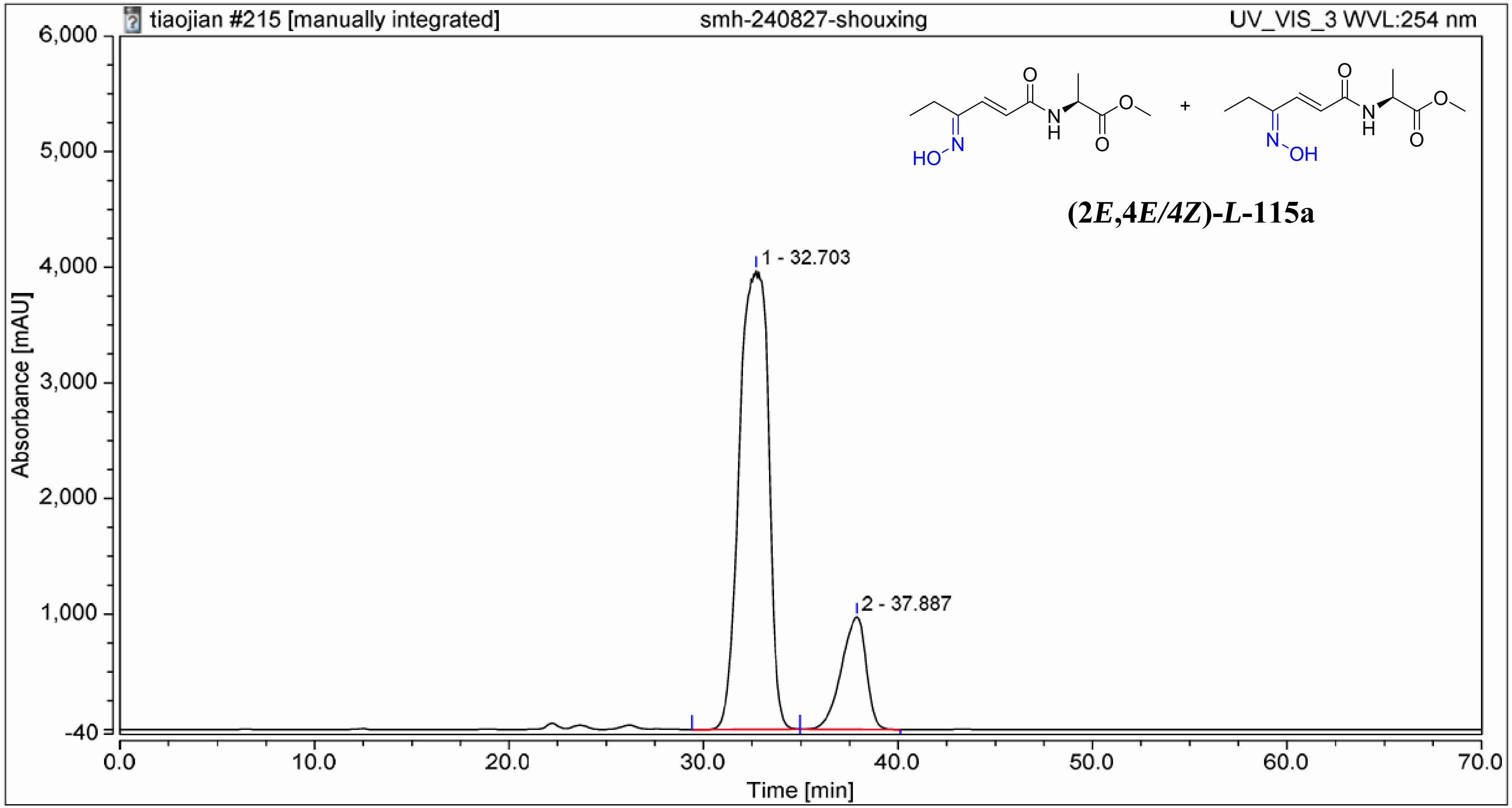

Integration Results

No.	Retention Time min	Area mAU*min	Relative Area %	Relative Height %	Amount n.a.
1	32.703	6872.067	82.78	80.32	n.a.
2	37.887	1429.925	17.22	19.68	n.a.
**Total:**		**8301.992**	**100.00**	**100.00**	

17. References

1. Richards, D. M. & Heel, R. C. *Drugs* **29**, 281–329 (1985).
2. Brogden, R. N., Heel, R. C., Speight, T. M. & Avery, G. S. *Drugs* **17**, 233–266 (1979).
3. Scott, L. J., Ormrod, D., K. & Goa, L. *Drugs* **61**, 143–266 (1979).
4. Brogden, R. N. & Campoli-Richards, D. M. *Drugs* **38**, 524–550 (1989).
5. Perry, C. & Scott, L. *Drugs* **64**, 1433–1464 (2004).
6. Tsuchiya, K., Kondo, M., Kida, M., Nakao, M., Iwahi, T., Nishi, T., Noji, Y., Takeuchi, M. & Nozaki, Y. *Antimicrob. Agents Chemother.* **19**, 56–65 (1981).
7. Bryson, H. M. & Brogden, R. N. *Drugs* **45**, 589–621 (1993).
8. Borin, M. T. *Drugs* **42**, 13−21(1991).
9. Chapman, T. M. & Perry, C. M. *Am. J. Respir. Med.* **2**, 75–107 (2003).
10. Kelly, P. J., Fisher, M., Lucas, H. & Krecek, R. C. *Vet. Parasitol.* **156**, 358–360 (2008).
11. McGuire, J. L., Phillips, A., Hahn, D. W., Tolman, E. L., Flor, S. & Kafrissen, M. E. *Am. J. Obstet. Gynecol.* **163**, 2127–2131 (1990).
12. Abrams, L. S., Skee, D. M., Wong, F. A., Anderson, N. J. & Leese, P. T. *J Clin Pharmacol* **41**, 1232−1237 (2001).
13. Gheorghiade, M., Blair, J. E., Filippatos, G. S., Macarie, C., Ruzyllo, W., Korewicki, J., Bubenek-Turconi, S. I., Ceracchi, M., Bianchetti, M., Carminati, P., et al. *J. Am. Coll. Cardiol.* **51**, 2276–2285 (2008).
14. Faustino, C., Serafim, C., Rijo, P. & Reis, C. P. *Expert Opin. Drug Deliv.* **13**, 1133–1148 (2016).
15. Perucca, E., Gatti, G. & Spina, E. *Clin. Pharmacokinet.* **27**, 175–190 (1994).
16. Green, M. D., Talbot, B. G. & Clark, C. R. *Life Sci.* **39**, 2263–2269 (1986).
17. Meijer, L., Skaltsounis, A. L., Magiatis, P., Polychronopoulos, P., Knockaert, M., Leost, M., Ryan, X. P., Vonica, C. A., Brivanlou, A., Dajani, R., Crovace, C., Tarricone, C., Musacchio, A., Roe, S. M., Pearl, L. & Greengard, P. *Chemistry & Biology* **10**, 1255–1266 (2003).
18. Chen, X., Yang, L., Oppenheim, J. J. & Howard, M. Z. *Phytother. Res.* **16**, 199–209 (2002).
19. Haack, T., Kurtkaya, S., Snyder, J. P. & Georg, G. I. *Org. Lett.* **5,** 5019–5022 (2003).
20. Shen, R., Lin, C. T., Bowman, E. J., Bowman, B. J. & Porco, J. A. *J. Am. Chem. Soc.* **125**, 7889–7901 (2003).

21. Dekker, K. A., Aiello, R. J., Hirai, H., Inagaki, T., Sakakibara, T., Suzuki, Y., Thompson, J. F., Yamauchi, Y. & Kojima, N. *J. Antibiot.* **51,** 14–20 (1998).

22. Katakawa, K., Kitajima, M., Aimi, N., Seki, H., Yamaguchi, K., Furihata, K., Harayama, T. & Takayama, H. *J. Org. Chem.* **70**, 658−663 (2005).

23. Paciorek, J., Höfler, D., Sokol, K. R., Wurst, K. & Magauer, T. *J. Am. Chem. Soc.* **144**, 19704−19708 (2022).

24. Fattorusso, E., Minale, L., Sodano, G., Moody, K. & Thomson, R. H. *J. Chem. Soc. D.* 752−753 (1970).

25. Anderson, Z. J. & Fox, D. J. *Org. Biomol. Chem.* **14**, 1450−1454 (2016).

26. Carney, J. R., Scheuer, P. J. & Kelly-Borges, M. *J. Nat. Prod.* **56**, 153–157 (1993).

27. Wu, H., Nakamura, H., Kobayashi, J., Ohizumi, Y. & Hirata, Y. *Experientia* **42**, 855−856 (1986).

28. Tabudravu, J. N., Eijsink, V. G., Gooday, G. W., Jaspars, M., Komander, D., Legg, M., Synstad, B. & van Aalten, D. M. *Bioorg. Med.Chem.* **10,** 1123−1128 (2002).

29. Nebbioso, A., Pereira, R., Khanwalkar, H., Matarese, F., García-Rodríguez, J., Miceli, M., Logie, C., Kedinger, V., Ferrara, F., Stunnenberg, H. G., et al. *Mol. Cancer Ther.* **10**, 2394–2404 (2011).

30. Dahlhoff, G., Niederer, J. P. M. & Hoelderich, W. F. *Catal. Rev.: Sci. Eng.* **43**, 381–441 (2001).

31. Thomas, J. M. & Raja, R. *Proc. Natl. Acad. Sci.* U. S. A. **102**, 13732–13736 (2005).

32. Kumar, R., Shah, S., Das, P. P., Bhagavanbhai, G. G. K., Al Fatesh, A. & Chowdhury, B. *Catal. Rev.* **61**, 516–594 (2019).

33. Wang, H., Qin, M., Wu, Q., Cheng, D., Meng, X., Wang, L. & Xiao, F. *Ind. Eng. Chem. Res.* **62,** 2217−2224 (2023).

34. Ritz, J., Fuchs, H., Kieczka, H. & Moran, W. C. *In Ullmann's Encyclopedia of Industrial Chemistry*; John Wiley & Sons Ltd.: Chichester, UK, (2011).

35. Bashkirov, A. N., et al. *Neftekhimiya* **1**, 527–534 (1961).

36. Griehl, W. & Ruestem, D. *Ind. Eng. Chem.* **62**, 16–22 (1970).

37. Barton, D. H. R. & Chavasiri, W. *Tetrahedron* **50**, 19–30 (1994).

38. Ishii, Y., Iwahama, T., Sakaguchi, S., Nakayama, K. & Nishiyama, Y. *J. Org. Chem.* **61**, 4520−4526 (1996).

39. Baucherel, X., Arends, I. W. C. E., Ellwood, S. & Sheldon, R. A. *Org. Process Res. Dev.* **7**,

426−428 (2003).

40. Feng, Q., Yuan, D. K., Wang, D. Q., Liang, X. M., Zhang, J. J., Wu, J. P. & Chen, F. H. *Green Sust. Chem.* **1**, 63-69 (2011).
41. Weissermel, A. H. J. *Industrial Organic Chemistry*, 4th ed.; Wiley-VCH Verlag GmbH & Co. KGaA: Weinheim, (2003).
42. Nikl, J., Hofman, K., Mossazghi, S., Möller, I. C., Mondeshki, D., Weinelt, F., Baumann, F. E. & Waldvogel, S. R. *Nat. Commun.* **14**, 4565 (2023).
43. Baucherel, X., Arends, I. W. C. E., Ellwood, S. & Sheldon, R. A. *Org. Process Res. Dev.* **7**, 426−428 (2003).
44. Han, J., Tan, L., Wan, Y., Li, G. & Anderson, S. N. *Dalton Trans.* **51**, 11620−11624 (2022).
45. Wang, W., Xu, D., Sun, Q. & Sun, W. *Chem. Asian J.* **13**, 2458−2464 (2018).
46. Chen, M. S. & White, M. C. *Science* **318**, 783−787 (2007).
47. Gómez, L., Bosch, I. G., Company, A., Benet-Buchholz, J., Polo, A., Sala, X., Ribas, X. & Costas, M. *Angew. Chem. Int. Ed.* **48**, 5720–5723 (2009).
48. Sisti, S., Galeotti, M., Scarchilli, F., Salamone, M., Costas, M. & Bietti, M. *J. Am. Chem. Soc.* **145**, 22086–22096 (2023).
49. Chen, M. S. & White, M. C. *Science* **327**, 566−571 (2010).
50. Milan, M., Bietti, M. & Costas, M. *ACS Cent. Sci.* **3**, 196−204 (2017).
51. Kawamata, Y., Yan, M., Liu, Z., Bao, D. H., Chen, J., Starr, J. T. & Baran, P. S. *J. Am. Chem. Soc.* **139**, 7448−7451 (2017).
52. Prat, I., Gmez, L., Canta, M., Ribas, X. & Costas, M. *Chem. Eur. J.* **19**, 1908–1913 (2013).
53. Canta, M., Font, D., Gmez, L., Ribas, X. & Costas, M. *Adv. Synth. Catal.* **356**, 818–830 (2014).
54. Catino, A. J., Forslund, R. E. & Doyle, M. P. *J. Am. Chem. Soc.* **126**, 13622−13623 (2004).
55. Vicens, L., Bietti, M. & Costas, M. *Angew. Chem., Int. Ed.* **60**, 4740−4746 (2021).
56. Zhou, J., Jia, X., Song, M., Huang, Z., Steiner, A., An, Q., Ma, J., Guo, Z., Zhang, Q., Sun, H., Robertson, C., Bacsa, J., Xiao, J. & Li, C. *Angew. Chem., Int. Ed.* **61**, No. e202205983 (2022).
57. Gormisky, P. E. & White, M. C. *J. Am. Chem. Soc.* **135**, 14052−14055 (2013).
58. Mikhalyova, E. A., Makhlynets, O. V., Palluccio, T. D., Filatov, A. S. & Rybak-Akimova, E. V. *Chem. Commun.* **48**, 687−689 (2012).
59. Cussó, O., Garcia-Bosch, I., Ribas, X., Lloret Fillol, J. & Costas, M. *J. Am. Chem. Soc.* **135**,

14871−14878 (2013).

60. Ottenbacher, R. V., Samsonenko, D. G., Talsi, E. P. & Bryliakov, K. P. *ACS Catal.* **4**, 1599−1606 (2014).

61. Shen, D., Miao, C., Wang, S., Xia, C. & Sun, W. *Org. Lett.* **16**, 1108−1111 (2014).

62. Call, A., Capocasa, G., Palone, A., Vicens, L., Aparicio, E., Afailal, N. C., Siakavaras, N., Saló, M. E. L., Bietti, M. & Costas, M. *J. Am. Chem. Soc.* **145**, 18094–18103 (2023).

63. Legters, J., Thijs, L. & Zwanenburg, B. *Red. Trav. Chim. Pays-Bas,* **111**, 001–015 (1992).

64. Ho, X. H., Jung, W. J., Shyama, P. K. & Jang, H. Y. *Catal. Sci. Technol.* **4**, 1914–1919 (2014).

65. Baffert, C., Collomb, M. N., Deronzier, A., Pécaut, J., Limburg, J., Crabtree, R. H. & Brudvig, G. W. *Inorg. Chem.* **41**, 1404−1411 (2002).

66. Hureau, C., Blondin, G., Charlot, M. F., Philouze, C., Nierlich, M., Césario, M. & Anxolabéhere-Mallart, E. *Inorg. Chem.* **44**, 3669−3683 (2005).

67. Yang, G., Mikhalyova, E. A., Filatov, A. S., Kryatov, S. V. & Rybak-Akimova, E. V. *Inorg. Chim. Acta* **546**, 121288 (2023).

68. Kasper, J. B., Vicens, L., de Roo, C. M., Hage, R., Costas, M. & Browne, W. R. *ACS Catal.* **13**, 6403−6415 (2023).

69. Du, J., Miao, C., Xia, C., Lee, Y.-M., Nam, W. & Sun, W. *ACS Catal*. **8**, 4528–4538 (2018).

70. Li, X.-X., Guo, M., Qiu, B., Cho, K.-B., Wei Sun, W. & Nam, W. *Inorg. Chem*. **58**, 14842–14852 (2019).

71. Lyakin, O. Y., Bryliakov, K. P. & Talsi, E. P, *Coord. Chem. Rev*. **384**, 126–139 (2019).

72. Kal, S., Xu, S. & Jr, L. Q. *Angew. Chem. Int. Ed*. **59**, 7332–7349 (2020).

73. Gong, Z., Wang, L., Xu, Y., Xie, D., Qi, X., Nam, W. & Guo, M., *Adv. Sci.* **11**, 2310333 (2024).

74. Rao, C. B., Rao, D. C., Babu, D. C. & Venkateswarlu, Y. *Eur. J. Org. Chem*. 2855–2859 (2010).

75. Basumatary, G. & Bez, G. *Tetrahedron Lett*. **58**, 4312–4315 (2017).

76. Ema, T., Ura, N., Yoshii, M., Korenaga, T. & Sakai, T. *Tetrahedron*, **65**, 9583–9591 (2009).

77. Päiviö, M., Mavrynsky, D., Leino, R. & Kanerva, L. T. *Eur. J. Org. Chem*. 1452–1457 (2011).

78. Lu, P., Hou, T., Gu, X. & Li, P. *Org. Lett.* **17**, 1954−1957 (2015).

79. Taylor; J., Whittall, N. & Hii, K. K. *Chem. Commun*. 5103–5105 (2005).

80. Li, J., Qu, S. & Zhao, W. *Angew. Chem., Int. Ed*. **59**, 2360−2364 (2020).

81. Zhang, X., Shen, C., Xia, C., Tian, X. & He, L. *Green Chem*. **20**, 5533–5539 (2018).

82. Furuta, A., Fukuyama, T. & Ryu, I. *Bull. Chem. Soc. Jpn*. **90**, 607–612 (2017).

83. O'Mahony, R. M., Lynch, D., Hayes, H. L. D., Thuama, E. N., Donnellan, P., Jones, R. C., Glennon, B., Collins, S. G. & Maguire, A. R., *Eur. J. Org. Chem*. 6533–6539 (2017).

84. Zhang, L., Si, X., Yang, Y., Witzel, S., Sekine, K., Rudolph, M., Rominger, F. & Hashmi, A. S. K. *ACS Catal*. **9**, 6118−6123 (2019).

85. Mosley, C. A., Myers, S. J., Murray, E. E., Santangelo, R., Tahirovic, Y. A., Kurtkaya, Mullasseril, P., Yuan, H., Lyuboslavsky, P., Le, P., Wilson, L. J., Yepes, M., Dingledine, R., Traynelis, S. F. & Liotta, D. C. *Bioorg. Med. Chem*, **17**, 6463–6480 (2009).

86. Shukla, P., Hsu, Y.-C. & Cheng, C. H. *J. Org. Chem*. **71**, 655-658 (2006).

87. Fu, L., Chen, Q., Wang, Z. & Nishihara, Y. *Org. Lett*. **22**, 2350−2353 (2020).

88. Johnston, C. P., smith, R. T., Allmendinger, S. & MacMillan, D. W. C. *Nature* **536**, 322–325 (2016).

89. Vicens, L., Bietti, M. & Costas, M. *Angew.Chem. Int. Ed*. **60**, 4740–4746 (2021).

90. Chen, Z., Cai, Q., Boni, Y. T. Liu, W., Fu, J. & Davies, H. M. L. *Org. Lett.* **25**, 3995–3999 (2023).

91. Lardy, S. W. & Schmidt, V. A. *J. Am. Chem. Soc*. **140**, 12318−12322 (2018).

92. Mbofana, C. T., Chong, E., Lawniczak, J. & Sanford, M. S. *Org. Lett*. **18**, 4258−4261 (2016).

93. Alcázar, J. *J. Comb. Chem.* **7**, 353–355 (2005).

94. Huang, G., Kouklovsky, C. & Torre, A. de la *J. Am. Chem. Soc*. **144**, 17803−17807 (2022).

95. Zhang, H., He, X., Wang, X., Yua, B., Zhao, S., Jiao, P., Jin, H., Liu, Z., Wang, K., Zhang, L. & Zhang, L. *Eur. J. Med. Chem*. **207**, 112774 (2020).

96. Zhang, X. & Rovis, T. *J. Am. Chem. Soc*. **143**, 21211−21217 (2021).

97. Rybak, A. & Meier, M. A. R. *Green Chem*. **9**, 1356–1361 (2007).

98. Tang, K. H. N., Uchida, K., Nishihara, K., Ito, M. & Shibata, T. *Org. Lett*. **24**, 1313−1317 (2022).

99. Wang, Z. X., Bai, X. Y., Yao, H. C. & Li, B. J. *J. Am. Chem. Soc*. **138**, 14872−14875 (2016).

100. Brewitz, L., Dumjahn, L., Zhao, Y., Owen, C. D., Laidlaw, S. M., Malla, T. R., Nguyen, D., Lukacik, P., Salah, E., Crawshaw, A. D., Warren, A. J., Trincao, J., Strain-Damerell, C., Carroll, M. W., Walsh, M. A. & Schofield, C. J. *J. Med. Chem*. **66**, 2663−2680 (2023).

101. Sheng, C., Ling, Z., Luo, Y. & Zhang, W. *Nat Commun*. **13**, 400 (2022).

102. Lee, G. S., Park, B. & Hong, S. H. *Nat Commun*. **13**, 5200 (2022).

103. Li, C. Q., Lang, K., Lu, H. J., Hu, Y., Cui, X., Wojtas, L. & Zhang, X. P. *Angew. Chem., Int. Ed*. **57**, 16837−16841 (2018).

104. Li, X., Che, X., Chen, G. H., Zhang, J., Yan, J. L., Zhang, Y. F., Zhang, L. S., Hsu, C. P., Gao, Y. Q. & Shi, Z. J. *Org. Lett*. **18**, 1234−1237 (2016).

105. Uraguchi, D., Kinoshita, N. & Ooi, T. *J. Am. Chem. Soc.* **132**, 12240–12242 (2010).

106. Chen, K., Hu, F., Zhang, S. Q. & Shi, B. F. *Chem. Sci*. **4**, 3906–3911 (2013).

107. Zhan, B. B., Li, Y., Xu, J. W., Nie, X. L., Fan, J., Jin, L. & Shi, B. F. *Angew. Chem. Int. Ed*. **57**, 5858–5862 (2018).

108. Wang, L., Wang, C. L., Lian, Z. H., Li, P. F., Kang, J. C., Zhou, J., Hao, Y., Liu, R. X., Bai, H. Y. & Zhang, S. Y. *Nat Commun.* **15**, 1483 (2024).

109. Luan, R., Lin, P., Li, K., Du, Y. & Su, W. *Nat Commun.* **15**, 1723 (2024).

110. Collier, P. N., Campbell, A. D., Patel, I., Raynham, T. M. & Taylor, R. J. K. *J. Org. Chem*. **67**, 1802−1815 (2002).

111. Collier, P. N., Campbell, A. D., Patel, I. & Taylor, R. J. K. *Tetrahedron* **58**, 6117−6125 (2002).

112. Vanos, C. M. & Lambert, T. H. *Chem. Sci*. **1**, 705–708 (2010).

113. Patil, V. V., Gayakwad, E. M. & Shankarling, G. S. *J. Org. Chem*. **81**, 781−786 (2016).

114. Suzuki, K., Watanabe, T. & Murahashi, S. I. *J. Org. Chem*. **78**, 2301−2310 (2013).

115. Hwu, J. R., Tseng, W. N., Patel, H. V., Wong, F. F., Horng, D. N., Liaw, B. R. & Lin, L. C. *J. Org. Chem*. **64**, 2211–2218 (1999).

116. Colin, B., Lavastre, O., Fouquay, S., Michaud, G., Simon, F. & Brusson, J. M. *ACS Comb. Sci*. **21**, 300−309 (2019).

117. Eshghi, H. & Hassankhan, A. *Org. Prep. Proced. Int.* **37**, 575–579 (2009).

118. Clive, D. L. J., Pham, M. P. & Subedi, R. *J. Am. Chem. Soc.* **129**, 2713–2717 (2007).

110. Torán, R., Vila, C., Sanz-Marco, A., Muñoz, M. C., Pedro, J. R. & Blay, G. *Eur. J. Org. Chem*. 627–630 (2020).

120. Li, B., Chen, J., Liu, D., Gridnev, I. D. & Zhang, W. *Nat. Chem.* **14**, 920–927 (2022).

121. Gao, Y., Liu, J., Li, Z., Guo, T., Xu, S., Zhu, H., Wei, F., Chen, S., Gebru, H. & Guo, K. *J. Org. Chem*. **83**, 2040−2049 (2018).

122. Zhu, C., Tang, P. & Yu, B. *J. Am. Chem. Soc.* **130**, 5872–5873 (2008).

123. Laudadio, G., Govaerts, S., Wang, Y., Ravelli, D., Koolman, H. F., Fagnoni, M., Djuric, S.

W. & Noël, T. *Angew.Chem. Int. Ed.* **57**, 4078–4082 (2018).

124. Biesinger, M. C., Payne, B. P., Grosvenor, A. P., Lau, L. W. M., Gerson, A. R. & Smart, R. St. C., *Appl. Surf. Sci.* **257***,* 2717−2730 (2011).

125. Ilton, E., S., Post, J. E., Heaney, P. J., Ling, F. T. & Kerisit, S. N., *Appl. Surf. Sci.* **366**, 475–485 (2016).

126. Naumkin, A. V., Kraut-Vass, A., Gaarenstroom, S. W. & Powell, C. J., NIST X-ray Photoelectron Spectroscopy Database (SRD 20), Version 5.0 (web version) (http://srdata.nist.gov/xps/) 2023.

127. Sheldrick, G. M., *Acta Crystallogr., Sect. A* **71**, 3-8 (2015).
